# End‐of‐life quality metrics among medicare decedents at minority‐serving cancer centers: A retrospective study

**DOI:** 10.1002/cam4.2752

**Published:** 2020-01-11

**Authors:** Garrett T. Wasp, Shama S. Alam, Gabriel A. Brooks, Inas S. Khayal, Nirav S. Kapadia, Donald Q. Carmichael, Andrea M. Austin, Amber E. Barnato

**Affiliations:** ^1^ Department of Medicine Geisel School of Medicine Hanover NH USA; ^2^ Norris Cotton Cancer Center at Dartmouth‐Hitchcock Medical Center Lebanon NH USA; ^3^ The Dartmouth Institute Geisel School of Medicine at Dartmouth Lebanon NH USA; ^4^ Thayer School of Engineering Dartmouth College Hanover NH USA

**Keywords:** cancer, end‐of‐life quality, minority, treatment intensity

## Abstract

**Background:**

We calculated the performance of National Cancer Institute (NCI)/National Comprehensive Cancer Network (NCCN) cancer centers’ end‐of‐life (EOL) quality metrics among minority and white decedents to explore center‐attributable sources of EOL disparities.

**Methods:**

We conducted a retrospective cohort study of Medicare beneficiaries with poor‐prognosis cancers who died between April 1, 2016 and December 31, 2016 and had any inpatient services in the last 6 months of life. We attributed patients’ EOL treatment to the center at which they received the preponderance of EOL inpatient services and calculated eight risk‐adjusted metrics of EOL quality (hospice admission ≤3 days before death; chemotherapy last 14 days of life; ≥2 emergency department (ED) visits; intensive care unit (ICU) admission; or life‐sustaining treatment last 30 days; hospice referral; palliative care; advance care planning last 6 months). We compared performance between patients across and within centers.

**Results:**

Among 126,434 patients, 10,119 received treatment at one of 54 NCI/NCCN centers. In aggregate, performance was worse among minorities for ED visits (10.3% vs 7.4%, *P* < .01), ICU admissions (32.9% vs 30.4%, *P* = .03), no hospice referral (39.5% vs 37.0%, *P* = .03), and life‐sustaining treatment (19.4% vs 16.2%, *P* < .01). Despite high within‐center correlation for minority and white metrics (0.61‐0.79; *P* < .01), five metrics demonstrated worse performance as the concentration of minorities increased: ED visits (*P* = .03), ICU admission (*P* < .01), no hospice referral (*P* < .01), and life‐sustaining treatments (*P* < .01).

**Conclusion:**

EOL quality metrics vary across NCI/NCCN centers. Within center, care was similar for minority and white patients. Minority‐serving centers had worse performance on many metrics.

## Graphical abstract

We evaluated end‐of‐life quality metrics by minority serving status at NCI/NCCN cancer centers. We found higher minority‐serving status associated with worse end‐of‐life quality on some but not all measures. However, at any given cancer center, care was similar for minority and white patients.

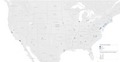

## 1 INTRODUCTION

The Institute of Medicine has identified increasingly aggressive, burdensome, and expensive end‐of‐life treatment as a major public health problem.^1^ The National Quality Forum (NQF) endorses five quality metrics relating to aggressive end‐of‐life treatment in patients with cancer, including receipt of chemotherapy in the last 14 days of life, more than one ED visit in the last 30 days of life, any ICU admission in the last 30 days of life, and late or no hospice.^2^ These end‐of‐life treatment intensity metrics vary by more than twofold across major academic centers.^3^, ^4^ Racial and ethnic minority groups are more likely to receive aggressive treatment,^5^, ^6^, ^7^ potentially in disproportion to their preferences.^8^, ^9^ Some of this is attributable to receipt of treatment at centers with systematically higher end‐of‐life intensity.^8^, ^10^

While these end‐of‐life treatment intensity metrics are widely recognized surrogates for end‐of‐life quality, other interventions, like palliative care, have been shown to improve end‐of‐life quality and promote less aggressive end‐of‐life care for patients with advanced cancer.^11^, ^12^ Additionally, advance care planning—the process by which people express their values and priorities for future medical care, including treatments they might wish to receive if they are near the end‐of‐life—has also been associated with improved end‐of‐life quality of care.^13^ As compared to the NQF metrics, relatively little is known about utilization of palliative care services and advance care planning for advanced cancer patients.

Our objective was to assess racial differences in performance on end‐of‐life quality metrics among NCI‐designated and/or NCCN‐affiliated cancer centers. We focused on NCI/NCCN centers because they set national standards for high quality care. Specifically, we sought to profile cancer centers’ minority and white‐specific end‐of‐life quality metrics and to test whether minority‐serving cancer centers had systematically lower end‐of‐life quality metrics.

## 2 METHODS

We conducted a retrospective study of decedents following previously developed methods,^4^, ^14^ enhanced using Part D chemotherapy prescriptions, palliative care codes, and advance care planning codes. We summarize these methods below.

### 2.1 Data

We used a 100% sample of Medicare fee‐for‐service beneficiaries drawn from 2015 to 2016 Centers for Medicare and Medicaid Services (CMS) files, including: (a) the Master Beneficiary Summary file, (b) the Medicare Provider Analysis and Review (MedPAR) file, (c) Physician/Supplier Carrier file, (d) the Outpatient file, (e) the Hospice file. We used a 40% subsample of these FFS beneficiaries drawn from: (f) Drug coverage/Part D files.

### 2.2 Cohort definition

We identified Medicare fee‐for‐service beneficiaries with poor prognosis cancers who died between April 1, 2016 and December 31, 2016 between the ages 66 and 99 for whom we had complete look‐back data between October 1, 2015 and March 31, 2016. We defined the study period as October 2015‐December 2016 to allow for uniform definitions of diagnosis and treatment using the International Classification of Diseases, Tenth Revision, Clinical Modification (ICD‐10‐CM). The lookback period was used to identify comorbidities for risk‐adjustment.^15^ The purpose of identifying patients with poor prognosis cancers was to specify a decedent cohort where deaths were likely to be attributable to cancer. We defined poor prognosis cancers using the method of Iezzoni and colleagues,^15^ as done in a prior study,^4^ which identifies conditions with a high risk of near‐term death. Briefly, this method identifies patients with metastatic cancers (as identified through diagnosis codes for secondary malignant neoplasms) as well as patients with primary cancers that are associated with high mortality risk regardless of cancer stage (eg pancreatic cancer and acute myeloid leukemia). Since the taxonomy for poor prognosis cancers was originally defined using the International Classification of Diseases, Ninth Revision, Clinical Modification (ICD‐9‐CM),^4^, ^15^ we used General Equivalence Mapping (GEM) to map them in the ICD‐10 system (See Appendix A).^16^ Additionally, we used a modification of the Agency for Healthcare Research and Quality (AHRQ) Clinical Classifications Software (CCS) approach to group cancers.^15^ We tested the validity of the ICD‐9 to ICD‐10 mapping by comparing case volumes in 2014‐2016.^17^ Lastly, in light of CMS suppression rules, we pooled heterogeneous race and ethnicity categories (Hispanic, black, Asian, and other) into a single minority group.

### 2.3 Hospital assignment

Beneficiaries with at least two outpatient oncologist visits or at least one hospital admission for cancer in the last 6 months of life were included in state and hospital referral region measure calculations (data not included in this report; see Dartmouthatlas.org for downloadable data). Only beneficiaries with at least one admission for cancer in the last 6 months of life were attributed to a particular hospital for hospital measure calculations.^15^, ^18^ We attributed the patient's medical care to the hospital providing the preponderance of cancer care hospitalizations in the last 6 months of life. We defined cancer care hospitalizations as those with a primary diagnosis of cancer or a secondary diagnosis of poor‐prognosis cancer.^15^

We obtained hospital characteristics from the 2015‐2016 Medicare Provider of Services File. We categorized hospitals into three, mutually exclusive groups based on their membership status as of December 31, 2017: NCI‐designated comprehensive cancer center and/or NCCN‐affiliated cancer centers (n = 54, hereafter referred to as “NCI/NCCN cancer centers”); other academic medical centers (n = 118); and community hospitals (n = 2,002).^19^, ^20^ For cancer centers with multiple satellite affiliates (eg Mayo Clinic, Dana‐Farber Cancer Institute, etc), we analyzed each geographically unique institution separately. We further stratified NCI/NCCN cancer centers into three groups based on the proportion of minority decedents served: low, defined as <15% minority‐serving (n = 20), medium, 15%‐30% minority‐serving (n = 18), and high, whose decedent population was more than 30% minorities (n = 15).

### 2.4 End‐of‐life care and quality metrics

We calculated NQF‐endorsed end‐of‐life cancer care quality metrics including: (a) receipt of chemotherapy in the last 14 days of life (NQF #0210), (b) more than one ED visit in the last 30 days of life (NQF #0211), (c) ICU admission in the last 30 days of life (NQF #0213), (d) nonreferral to hospice (NQF #0215), or (e) late (NQF #0216) referral to hospice, defined as within 3 days of death. In addition to these 5 metrics, we calculated: (f) life‐sustaining treatment rates in the last 30 days of life (eg, mechanical ventilation, hemodialysis, feeding tubes, or cardio‐pulmonary resuscitation); (g) palliative care claims in the last 6 months of life (ICD‐10 diagnosis codes Z51.5, V66.7); and (h) advance care planning claims in the last 6 months of life (billing codes G9054, S0257, 99 497, and 99 498). We identified chemotherapy administration using claims and event records from the carrier, outpatient, Part D (40% sample), and MEDPAR files. We used Healthcare Common Procedure Coding System (HCPCS) codes to identify use of chemotherapy drugs in the carrier, outpatient, and MEDPAR files, and we used NDC codes to identify chemotherapy prescription fills in the Part D event file (see HCPCS and NDC code lists in Appendix C,D respectively). In addition, we identified chemotherapy administration using the ICD10‐CM diagnosis codes Z51.11 and Z51.12 (carrier, outpatient, and MEDPAR files), the ICD10 procedure code 3E03305 (MEDPAR file), and revenue center codes 0331, 0332, and 0335 (MEDPAR file). We identified ED visits from the outpatient claims (not resulting in an admission), ICU admission from MedPAR claims, hospice use and length of stay from the hospice claims, life‐sustaining treatment use using ICD‐10 CM/PCS in MedPAR and the HCPCS in the Carrier claims, and palliative care and advance care planning visits using HCPCS in the Carrier claims (by any billing provider, not just specialty palliative care providers). A description of the codes used to classify end‐of‐life health care use is provided in Appendix E.

### 2.5 Statistical analysis

We summarized cohort characteristics and calculated age, gender, race, and comorbidity‐adjusted proportions and 95% confidence intervals (CIs) for the eight end‐of‐life quality metrics by hospital type (NCI/NCCN, academic medical center, community). We calculated quality metrics for all decedents, then separately for minority and white decedents. The magnitude of variation for each end‐of‐life quality measure among the NCI/NCCN centers was calculated by dividing the highest value by the lowest, nonzero, value. For race‐specific end‐of‐life quality metrics we did not include race as a risk‐adjuster. Following the Dartmouth Atlas algorithm,^3^ comorbidity adjustment included: CCS defined cancers of the lung, hematological and vague types^21^; hospital type, using NCI or NCCN designation as the reference; and Iezzoni chronic conditions,^15^ chronic pulmonary disease, coronary artery disease, congestive heart failure, peripheral vascular disease, severe chronic liver disease, diabetes w/end organ damage, chronic renal failure, and dementia.

For hospitals with at least 11 decedents (per CMS suppression rules), we calculated age, gender, race (when appropriate), and comorbidity‐adjusted proportions and 95% CIs for each measure among all decedents assigned to the hospital and then separately for minority and white decedents.

For NCI/NCCN centers, we then conducted within‐ and across‐center comparisons for each measure. Within centers we explored the correlation between minority and white‐specific rates for the end‐of‐life metrics using Pearson correlation coefficients and mean differences using Chi‐square tests. We restricted within‐center comparisons to metrics with sufficient sample size (ie, with at least 11 minority decedents) to calculate a minority‐specific rate in at least ten centers. Across centers we explored the relationship between the concentration of minorities and performance on end‐of‐life quality metrics. Specifically, we conducted a one‐sided Cochran‐Armitage test for trend across low, medium, and high minority serving centers. For 6 metrics (chemotherapy receipt, ED visits, ICU admission, late hospice, no hospice, and life‐sustaining treatments) we hypothesized an increasing trend from low to high minority serving; for palliative care and advance care planning we hypothesized a decreasing trend.

All statistical analyses were completed using SAS software version 9.4, and visualizations were completed using Tableau version 2018.3.2.

## 3 RESULTS

### 3.1 Cohort

**Figure 1:**
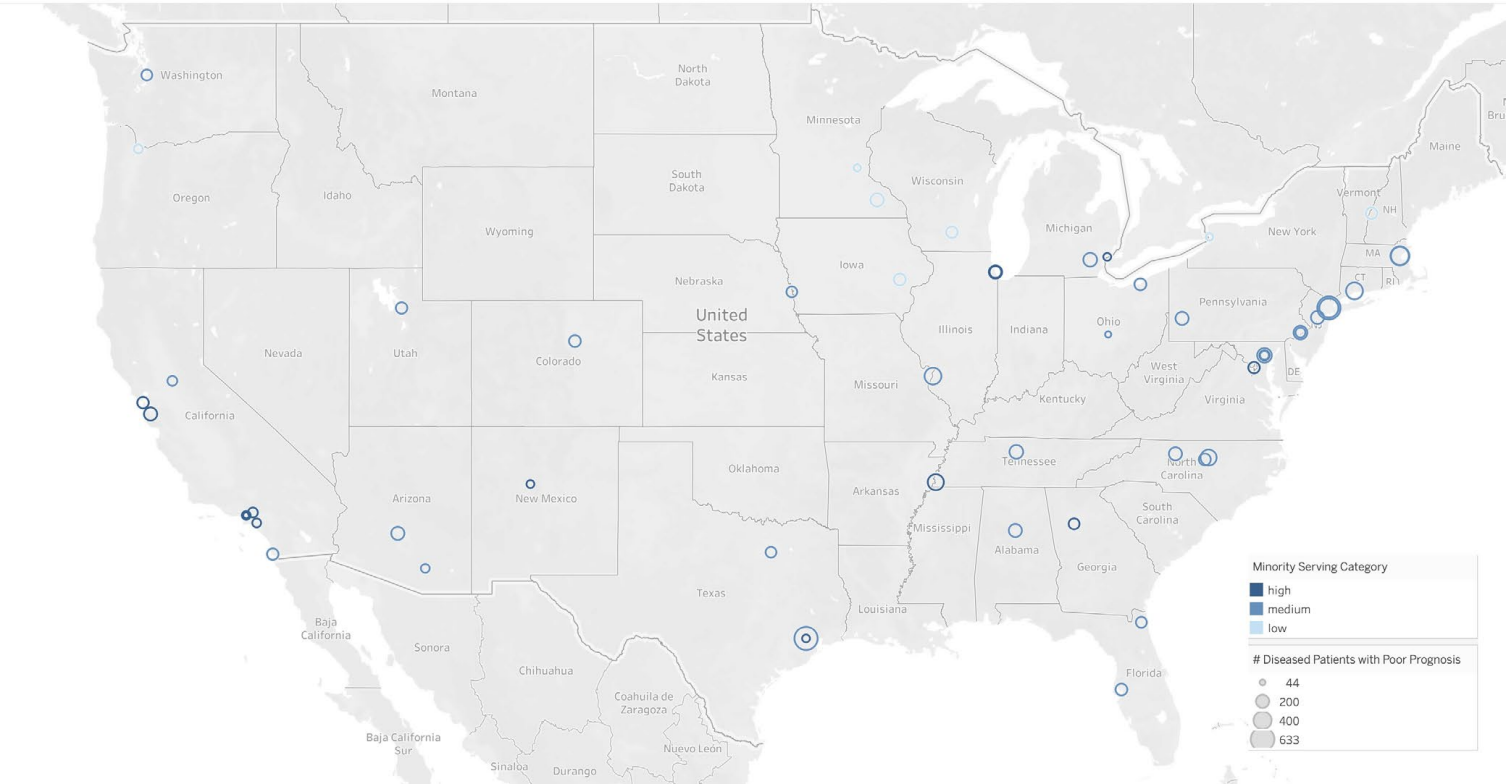
Geographic distribution, size, and minority concentration of NCI/NCCI cancer centers. Each bubble is a cancer center. The size of the bubble is proportionate to the total number of decedents assigned to the cancer center in 2016 and the shading of the bubble is proportionate to the concentration of minorities (low: <15%; medium: 15%‐30%; and high: >30%). For an interactive version of this figure, visit [Dartmouth Atlas URL TBD]. [NCI – National Cancer Institute; NCCN – National Comprehensive Cancer Network]

Among 126 434 Medicare patients with poor prognosis cancers who died, their cancer care was attributed to 2174 US hospitals in 2016, 22 020 (17.4%) were minorities. A total of 10 119 patients died at an NCI/NCCN cancer center, and 2 155 (21.3%) of these were minorities. On average, patients treated at NCI/NCCN cancer centers were younger and less likely to be enrolled in Medicaid than patients treated at academic medical centers or community hospitals (Table 1). Academic medical centers saw the highest proportion of minority patients (24.3%) and community hospitals saw the highest proportion of white patients (83.7%). Most NCI/NCCN cancer center hospitals are concentrated in major population centers (Figure 1). The 20 NCI/NCCN cancer centers classified as low minority‐serving centers cared for 17.5% of all minorities seen at NCI/NCCN centers, the 19 centers classified as medium minority‐serving cared for 47.8% of minorities and the 15 centers classified as high minority‐serving cared for 34.6% of minorities.

**Table 1:** Characteristics of Medicare fee‐for‐service beneficiaries 66 years of age or older who died with poor‐prognosis cancer^a^ in 2016, by hospital type

Variable	All hospitals (n = 2,174)	NCI or NCCN cancer centers (n = 54)	Academic medical centers (n = 118)	Community hospitals (n = 2002)
Number of decedents, n (% row)	126 434 (100)	10 119 (8.0)	11 706 (9.3)	104 609 (82.7)
Non‐Hispanic white n, (% column)	104 414 (82.6)	7964 (78.7)	8860 (75.7)	87 590 (83.7)
Minority^b^	22 020 (17.4)	2155 (21.3)	2846 (24.3)	17 019 (16.3)
Black	11 989 (9.5)	1124 (11.1)	1833 (15.7)	9032 (8.6)
Asian	2582 (2.0)	348 (3.4)	246 (2.1)	1988 (1.9)
Hispanic	5220 (4.1)	442 (4.4)	536 (4.6)	4242 (4.1)
Other	2229 (1.8)	241 (2.4)	231 (2.0)	1757 (1.7)
Age, mean (SD)	77.8 (7.7)	75.7 (7.1)	77.4 (7.8)	78.1 (7.7)
Female gender, n (% column)	60 190 (47.6)	4668 (46.1)	5697 (48.7)	49 825 (47.6)
Dual Full eligible any month in the last 6 months, n (% column)	17 598 (13.9)	1192 (11.8)	2160 (18.5)	14 246 (13.6)
Cancer type, n (% column)				
Bronchus, Lung	32 926 (26.0)	1671 (16.5)	2586 (22.1)	28 669 (27.4)
Hematologic malignancies	13 706 (10.8)	1416 (14.0)	1285 (11.0)	11 005 (10.5)
Vague, other unspecified	11 661 (9.2)	833 (8.2)	1047 (8.9)	9781 (9.4)
Pancreas	8746 (6.9)	788 (7.8)	922 (7.9)	7036 (6.7)
Colon/Rectum	8409 (6.7)	514 (5.1)	751 (6.4)	7144 (6.8)
Comorbidities^c^, mean (SD)	7.16 (2.7)	7.04 (2.6)	7.23 (2.8)	7.16 (2.7)

Abbreviation: SD, standard deviation.

a Appendix A.

b We pooled racial and ethnic groups to achieve sufficient sample size to address CMS suppression rules.

c Elixhauser comorbidity count.

### 3.2 End‐of‐life quality metrics

There was wide variation in end‐of‐life quality metrics across hospitals (Table 2). NCI/NCCN cancer centers varied more than two‐fold on all metrics. Receipt of chemotherapy in the last 14 days ranged from 0% to 9.7%; (2) more than one ED visit in the last 30 days ranged from 0%‐13.5%; (3) ICU admission in the last 30 days ranged from 13.7%‐59.1%; (4) late hospice enrollment ranged from 5.4%‐19.2%; (5) Nonreferral hospice ranged from 18.7%‐52.6%; (6) Life‐sustaining treatments in the last 30 days ranged from 6.3%‐36.1%; (7) palliative care claims ranged from 12.9%‐66.2%; and; (8) advance care planning claims ranged from 0% to 18.1%. Using benchmarks from Earle et al 2005,^22^ we found that for NCI/NCCN cancer centers with unsuppressed end‐of‐life measure values: 13 of 13 (100%) met chemotherapy in the last 14 days <10%; 2 of 33 (6.1%) met more than one ED visit in the last 30 days <4%; 0 of 54 (0%) met ICU admission in the last 30 days <4%; 8 of 38 (21.1%) met late hospice enrollment <8%; and 46 of 54 (85.2%) met Nonreferral hospice <45%. In aggregate, performance was worse among minorities for ED visits (10.3% vs 7.4%, *P* < .01), ICU admissions (32.9% vs 30.4%, *P* = .03), no hospice referral (39.5% vs 37.0%, *P* = .03), and life‐sustaining treatment (19.4% vs 16.2%, *P* < .01) (Table 2). Full details are reported in the Supplement; to download data, visit [https://www.dataverse.dartmouth.edu/dataset.xhtml?persistentId=doi:10.21989/D9/BWKLG5; See Supplement].

**Table 2:** Adjusted end of life treatment intensity among white and minority^a^ patients who died with poor‐prognosis cancers^b^ in 2016, by hospital type

Variable	All hospitals (n = 2,174)	Community hospitals (n = 2002)	Academic medical centers (n = 118)	NCI or NCCN cancer centers (n = 54)	NCI or NCCN minority vs white *P*‐value^c^
Receipt of infusion chemotherapy^d^ last 14 d, % (95% CI)					
All	3.9 (3.7 ‐ 4.2)	4.0 (3.8 ‐ 4.3)	3.0^e^	4.0^e^	.07
Minority	3.5 (3.0‐4.0)	3.6 (3.0‐4.3)	2.8^e^	3.3^e^	
White	4.0 (3.8‐4.3)	4.1 (3.8‐4.4)	3.0 (2.3‐3.9)	4.2 (3.3‐5.4)	
Receipt of infusion or oral chemotherapy^d^ last 14 d, % (95% CI)					
All	4.4 (4.1‐4.7)	4.5 (4.1‐4.8)	3.6 (2.8‐4.7)	4.5^e^	.75
Minority	4.1 (3.5‐4.8)	4.2 (3.5‐5.0)	3.5 (2.0‐6.1)	4.6^e^	
White	4.4 (4.1‐4.8)	4.5 (4.2‐4.9)	3.7 (2.8‐4.9)	4.4 (3.3‐5.9)	
Two or more ED visits last 30 d, % (95% CI)					
all	9.2 (8.9‐9.5)	9.3 (9.0‐9.7)	9.0 (8.0‐10.2)	8.0 (6.9‐9.2)	<.01
minority	11.1 (10.2‐12.0)	11.1 (10.1‐12.1)	11.9 (9.4‐15.0)	10.3 (7.8‐13.6)	
white	8.8 (8.5‐9.2)	9.0 (8.6‐9.4)	8.1 (7.0‐9.4)	7.4 (6.2‐8.7)	
ICU admission last 30 d, % (95% CI)					
All	34.5 (33.9‐35.2)	34.9 (34.2‐35.6)	33.8 (31.8‐35.9)	30.9 (28.8‐33.2)	.03
Minority	38.3 (36.7‐40.0)	39.3 (37.4‐41.2)	36.5 (31.7‐41.9)	32.9 (28.0‐38.5)	
White	33.7 (33.0‐34.4)	34.1 (33.3‐34.9)	32.9 (30.7‐35.3)	30.4 (28.0‐33.0)	
Late^f^ hospice referral, % (95% CI)					
All	12.8 (12.5‐13.2)	13.2 (12.8‐13.6)	12.4 (11.2‐13.7)	9.5 (8.3‐10.8)	.16
Minority	10.8 (9.9‐11.7)	11.0 (10.0‐12.1)	10.8 (8.4‐13.8)	8.7 (6.4‐11.7)	
White	13.3 (12.8‐13.7)	13.6 (13.1‐14.1)	12.9 (11.5‐14.4)	9.7 (8.4‐11.2)	
No hospice referral, % (95% CI)					
All	34.2 (33.5‐34.8)	33.4 (32.8‐34.1)	38.0 (35.9‐40.3)	37.5 (35.1‐40.1)	.03
Minority	38.9 (37.2‐40.6)	38.1 (36.2‐40.0)	43.4 (38.3‐49.2)	39.5 (34.2‐45.8)	
White	33.2 (32.5‐33.9)	32.5 (31.8‐33.3)	36.3 (33.9‐38.8)	37.0 (34.3‐39.9)	
Life‐sustaining treatment^g^ last 30 d, % (95% CI)					
All	15.7 (15.3‐16.2)	15.2 (14.7‐15.7)	19.8 (18.2‐21.4)	16.9 (15.3‐18.6)	<.01
Minority	20.9 (19.7‐22.2)	20.6 (19.2‐22.1)	24.1 (20.3‐28.6)	19.4 (15.7‐24.0)	
White	14.6 (14.2‐15.1)	14.1 (13.7‐14.7)	18.4 (16.7‐20.2)	16.2 (14.4‐18.1)	
Palliative care code^h^ last 6 mo, % (95% CI)					
All	26.3 (25.8‐26.9)	24.1 (23.5‐24.7)	38.9 (36.7‐41.2)	36.6 (34.2‐39.2)	.46
Minority	26.9 (25.4‐28.4)	24.0 (22.5‐25.6)	37.9 (33.1‐43.5)	36.0 (30.7‐42.1)	
White	26.2 (25.6‐26.9)	24.1 (23.5‐24.8)	39.2 (36.7‐41.8)	36.8 (34.1‐39.7)	
Advance care planning code^i^ last 6 mo, % (95% CI)					
All	5.8 (5.5‐6.1)	5.5 (5.2‐5.8)	10.2 (9.0‐11.4)	3.7 (3.0‐4.6)	.20
Minority	6.6 (5.9‐7.3)	6.3 (5.5‐7.1)	10.4 (8.1‐13.3)	4.2 (2.7‐6.5)	
White	5.6 (5.3‐5.9)	5.4 (5.0‐5.7)	10.1 (8.8‐11.6)	3.6 (2.8‐4.7)	

a We pooled racial and ethnic groups to achieve sufficient sample size to address CMS suppression rules.

b Appendix A.

c Represents an “N‐1” Chi‐squared test.

d Infusion chemotherapy from carrier claims calculated on a 100% sample; infusion or oral chemotherapy from carrier and Part D claims calculated on 40% sample.

e Due to large standard error estimates, the 95% confidence intervals extend beyond the range of possible probabilities, thus we have suppressed the confidence intervals.

f Admission onto Hospice three days or fewer before death.

g Mechanical ventilation, hemodialysis, enteral/parenteral feeding, cardio‐pulmonary resuscitation and intraaortic balloon pump placement.

h Includes nonhospice related palliative care content billed using ICD‐10 codes Z51.5 and V66.7.

i Includes only billed advance care planning conversations, which must be at least 16 minutes in length.

### 3.3 Within‐center comparisons

**Figure 2:**
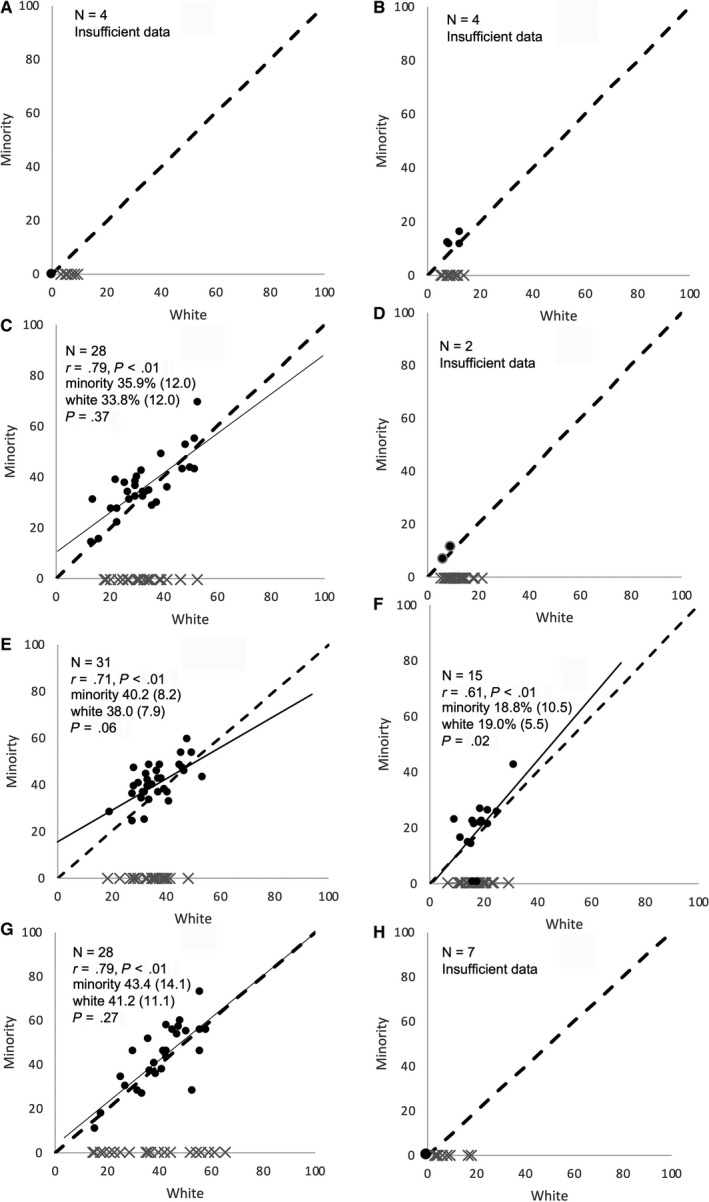
Panels A‐H. Correlation between white and minority‐specific end‐of‐life treatment intensity metrics, by NCI/NCCN cancer center. Panel A represents chemotherapy administration in the last 14 d of life, Panel B two or more emergency department visits in the last 30 d of life, Panel C ICU admissions in the last 30 d of life, Panel D late hospice referrals, Panel E no hospice referrals, Panel F life‐sustaining treatment in last 30 d, Panel G palliative care codes in last 6 mo of life, and Panel H advance care planning codes in the last 6 mo of life. Each bubble is a single cancer center; an “x” mark along the x‐axis represents cancer centers with insufficient sample size to calculate a minority‐specific measure. The y‐axis is the measure among minority decedents and the x‐axis is the measure among white decedents. Those centers with insufficient sample size to calculate either a white or minority‐specific measure are excluded from the panel. Listed in the upper left corner of each panel is N, the numbers of centers with sufficient sample size to calculate minority‐specific metrics. Below that is the correlation (rho) between cancer centers’ minority and white EOL measure and its *P*‐value. Next is the minority mean and standard deviation (SD) in parenthesis, followed by white mean and SD in parenthesis. The final *P*‐value is of the mean difference between minority and white EOL measure Reporting full information was restricted to metrics with white and minority‐specific metrics calculable for least 10 centers. [NCI – National Cancer Institute; NCCN – National Comprehensive Cancer Network]

Among the subset of NCI/NCCN centers with sufficient numbers of minority patients to calculate a minority‐specific value for 4 of 8 metrics (ranging from 15 for life‐sustaining treatment, 28 for ICU admission and palliative care codes, and 31 for nonhospice referral) there was strong correlation between end‐of‐life quality metrics among minority and white decedents (*r* = 0.61‐0.79; Figure 2). In this subset, within center minorities had slightly lower rates of life‐sustaining treatment in the last 30 days of life (18.8% vs 19.0%, *P* = .02); there were no significant differences for ICU use in the last 30 days of life (35.9% vs 33.8%, *P* = .37), no hospice referral (40.2% vs 38.0%, *P* = .06) and palliative care code use in the last 6 months of life (43.4% vs 41.2%, *P* = .27).

### 3.4 Across‐center comparisons

As the concentration of minorities served increased, end‐of‐life quality metrics decreased for 5 of 8 of the metrics (Table 3), including multiple ED visits (*P* = .03), any ICU admission (*P* < .01), no hospice referral (*P* < .01), life sustaining treatments (*P* < .01), and palliative care codes (*P* < .01).

**Table 3:** Patient characteristics and end of life treatment intensity metrics among decedents with poor prognosis cancer treated at NCI/NCCN cancer centers in 2016, by concentration of minorities served

Variable	Low^a^ (n = 20)	Medium^a^ (n = 19)	High^a^ (n = 15)	*P*‐value^b^
Non‐Hispanic white, n (% column)	3353 (89.9)	3447 (77.0)	1164 (60.9)	
Minority^c^, n (% column)	378 (10.1)	1031 (23.0)	746 (39.1)	
Black, n (% column)	174 (4.7)	542 (12.1)	408 (21.4)	
Asian, n (% column)	46 (1.2)	158 (3.5)	144 (7.5)	
Hispanic, n (% column)	73 (2.0)	233 (5.2)	136 (7.1)	
Other, n (% column)	85 (2.3)	98 (2.2)	58 (3.0)	
Age, mean (SD)	76 (7.3)	75 (7.0)	76 (7.2)	
Female gender, n (% column)	1658 (44.4)	2104 (47.0)	906 (47.4)	
Comorbidities^d^, mean (SD)	7.0 (2.6)	7.1 (2.6)	7.1 (2.6)	
Receipt of infusion chemotherapy^e^ last 14 days, %				
All	3.98	3.99	4.01	.48
Minority	4.22	3.25	3.02	.84
White	4.08	4.22	4.51	.27
Receipt of infusion or oral chemotherapy^e^ last 14 days, %				
All	4.22	4.65	4.58	.26
Minority	5.85	4.53	4.22	.83
White	4.07	4.70	4.84	.13
Two or more ED visits last 30 days,				
all	6.82	9.25	7.61	.03
minority	11.67	11.17	8.14	.98
white	6.01	8.55	7.60	<.01
ICU admission last 30 days, %				
All	29.33	29.78	36.53	<.01
Minority	29.23	30.73	37.64	<.01
White	29.09	29.43	36.02	<.01
Late^f^ hospice referral, %				
All	10.37	8.53	9.77	.91
Minority	9.24	8.96	8.03	.78
White	10.63	8.38	10.65	.87
No hospice referral, %				
All	34.99	39.36	38.26	<.01
Minority	38.56	39.79	39.60	.40
White	34.31	39.18	37.69	<.01
Life‐sustaining treatment^g^ last 30 days, %				
All	15.46	16.98	19.51	<.01
Minority	17.72	18.08	22.04	.02
White	14.88	16.59	18.34	<.01
Palliative care code^h^ last 6 mo, %				
All	39.85	34.22	36.08	<.01
Minority	36.34	35.13	36.76	.62
White	40.07	33.94	35.98	<.01
Advance care planning code^i^ last 6 mo, %				
All	2.41	4.48	4.38	1.00
Minority	Suppressed	4.97	4.01	.81
White	Suppressed	4.35	4.56	1.00

a Low: <15%; medium: 15%‐30%; and high: >30% minority.

b *P* values reflect across‐group test for trend for white and minority specific metrics of EOL treatment intensity, respectively, by using one‐sided Cochran‐Armitage method.

c We pooled racial and ethnic groups to achieve sufficient sample size to address CMS suppression rules

d Elixhauser comorbidity count.

e Infusion chemotherapy from carrier claims calculated on a 100% sample; infusion or oral chemotherapy from carrier and Part D claims calculated on 40% sample.

f Admission onto hospice three days or fewer before death.

g Mechanical ventilation, hemodialysis, feeding tubes, and cardio‐pulmonary resuscitation.

h Includes nonhospice related palliative care content billed using ICD‐10 codes Z51.5 and V66.7.

i Includes only billed advance care planning conversations, which must be at least 16 min in length.

## 4 DISCUSSION

In this retrospective cohort study of Medicare fee‐for‐service decedents with poor prognosis cancer whose cancer care could be attributed to either a NCI or NCCN cancer center, we demonstrate three major findings. First, our work redemonstrates findings that end‐of‐life quality metrics vary significantly, even across NCI/NCCN cancer centers. Second, within any given cancer center, rates of white and minority‐specific end‐of‐life quality metrics were highly correlated, suggesting that provider norms influence end‐of‐life treatment decision making, irrespective of patient race. Third, we found only partial support for our hypotheses that minorities systematically receive lower quality end‐of‐life care and that minority‐serving cancer centers perform worse on end‐of‐life quality metrics.

The wide range in end‐of‐life quality metrics, even among 54 elite NCI/NCCN cancer centers, reinforces the utility of these metrics, which were originally pioneered by Earle et al.^22^ We found that the range of chemotherapy receipt in the last 14 days at NCI/NCCN cancer centers in 2016 (0%‐9.1%) is lower than Earle's historical comparisons from 1999 when, on average, average of 11.6% of all Medicare cancer decedents received chemotherapy in the last 14 days of life.^23^ Based on benchmarks set at the 10th decile of highest performing hospitals in 1999 (<10% of patients),^22^ even when imputing the highest possible value for hospitals with suppressed counts, the majority of NCI/NCCN cancer centers achieved this benchmark in 2016. Similarly, a majority of centers met the benchmark for nonreferral to hospice (<45%). In contrast, no centers met the ICU benchmark (<4%), 2 centers met the benchmark for ED (<4%), and 8 met the late enrollment benchmark (<8%).

Our finding that end‐of‐life quality metrics varied widely across institutions, but that minority and white end‐of‐life treatment intensity were correlated within center, corresponds with previously published results showing hospital‐level correlation in end‐of‐life treatment intensity among black and white patients among cancer and noncancer related death.^6^ This provides support for qualitative research suggesting that provider norms—shared beliefs and behaviors among members of group or institution—influence end‐of‐life treatment decision making,^24^, ^25^ not patient preferences.^26^

We found partial support for our hypotheses that minorities receive systematically lower quality end‐of‐life cancer care than their white counterparts, and that this observation can be explained by minority‐serving centers having systematically lower end‐of‐life quality metrics. In aggregate, minorities had worse outcomes on 4 of 8 metrics. Five of 8 metrics showed a statistically significant trend toward worse performance on end‐of‐life quality metrics, measured among white patients and minority patients, as the center's concentration of minorities served increased. We speculate that this heterogeneity further evinces the presence of provider norms: both within and across institutions, various metrics are under the influence of various providers. For example, metrics such as ICU admission or life‐sustaining treatment use are more likely influenced by inpatient/ acute care providers, whereas chemotherapy use, ED use, hospice use, palliative care, and advance care planning are likely influenced by ambulatory oncology providers.

The strengths of this study include use of multiple end‐of‐life quality metrics, five of which are NQF‐endorsed. We used recent Medicare claims data, including Part D chemotherapy claims. Nevertheless, our analysis has many limitations. Those common to this class of retrospective research include biases introduced by the decedent follow‐back method^27^ and lack of generalizability to Medicare Advantage populations and commercially insured younger populations.^28^ Also, we restricted the cohort to the time period after ICD‐10 conversion to avoid problems with cohort comparability, resulting in small sample sizes—particularly for minority decedents—and therefore more suppression and less precision in center‐specific end‐of‐life quality metrics. Consequently, grouping black, Asian, and Hispanic patients into a single “minority” category limits understanding of potentially important differences between these distinct racial and ethnic groups. We restricted analyses to cohort members with at least one inpatient admission for purposes of hospital attribution. While the majority of patients with poor prognosis cancer had at least one hospitalization, centers with lower end‐of‐life hospitalization rates will have artificially elevated rates of inpatient service use (eg, ICU, life‐sustaining treatments) and potentially suppressed rates of hospice. Since we attributed patients’ EOL treatment to the center at which they received the preponderance of EOL inpatient services, patients who traveled to receive their cancer‐directed therapy at an NCI/NCCN cancer center but received the preponderance of their last 6 months of life inpatient care at their local hospital would be attributed to their local hospital rather than the NCI/NCCN center. This may be appropriate for hospital‐based services they received at the local hospital (eg, ICU use in the last 6 months of life) but not for outpatient‐based services they may have received at the NCI/NCCN center (eg, chemotherapy in the last 14 days of life). It is not possible to estimate the direction of this misspecification bias, unless we advanced some hypotheses regarding the relative EOL treatment intensity received by patient who did and did not travel for their cancer care. Additionally, we focused on 54 NCI/NCCN centers because they set national standards for high quality care; however, the substantial majority of cancer patients are treated in the other 2120 academic and community centers. Patients who seek care at NCI/NCCN cancer centers likely differ systematically from those treated in academic and community centers, including their preference for aggressiveness of treatment. While the NQF‐endorsed metrics have face validity,^29^, ^30^ there is no consensus regarding the “right” rate for each outcome; given prognostic uncertainty, it is not feasible to expect any of these metrics to be 0 or 100%.^23^

In summary, our findings reinforce the now well‐recognized phenomenon that hospital‐level practice patterns influence treatment for white and minority cancer patients. More work needs to be done to elucidate the mechanisms behind differences in end‐of‐life quality metrics—particularly norms of provider decision making and their influence on patient and family treatment expectations—as well as the ways in which concentration of minorities served may influence these phenomena.

## Data Availability

Currently reflects a draft version as the url is being created: The data that support the findings of this study are openly available in [Atlas Dataverse] at http://doi.org/%5B10.21989/D9/BWKLG5i], reference number [reference number].
