# Supplementary material for: End‐of‐life quality metrics among medicare decedents at minority‐serving cancer centers: A retrospective study
Source: Cancer Med. 2020 Jan 11;9(5):1911–21. doi: 10.1002/cam4.2752 (PMC7050066; doi:10.1002/cam4.2752)
Supplement: Supplementary file 1 [file CAM4-9-1911-s001.pdf]

ICD10 CCS Cancer Poor Prognosis

ICD10	CCS	ICD10_DESCRIPTION	CCS_DESCRIPTION
C153	12	Malignant neoplasm of upper third of esophagus	Cancer of esophagus
C154	12	Malignant neoplasm of middle third of esophagus	Cancer of esophagus
C155	12	Malignant neoplasm of lower third of esophagus	Cancer of esophagus
C158	12	Malignant neoplasm of overlapping sites of esophagus	Cancer of esophagus
C159	12	Malignant neoplasm of esophagus, unspecified	Cancer of esophagus
C160	13	Malignant neoplasm of cardia	Cancer of stomach
C161	13	Malignant neoplasm of fundus of stomach	Cancer of stomach
C162	13	Malignant neoplasm of body of stomach	Cancer of stomach
C163	13	Malignant neoplasm of pyloric antrum	Cancer of stomach
C164	13	Malignant neoplasm of pylorus	Cancer of stomach
C165	13	Malignant neoplasm of lesser curvature of stomach, unsp	Cancer of stomach
C166	13	Malignant neoplasm of greater curvature of stomach, unsp	Cancer of stomach
C168	13	Malignant neoplasm of overlapping sites of stomach	Cancer of stomach
C169	13	Malignant neoplasm of stomach, unspecified	Cancer of stomach
C220	16	Liver cell carcinoma	Cancer of liver and intrahepatic bile duct
C221	16	Intrahepatic bile duct carcinoma	Cancer of liver and intrahepatic bile duct
C227	16	Other specified carcinomas of liver	Cancer of liver and intrahepatic bile duct
C228	16	Malignant neoplasm of liver, primary, unspecified as to type	Cancer of liver and intrahepatic bile duct
C229	16	Malig neoplasm of liver, not specified as primary or sec	Cancer of liver and intrahepatic bile duct
C250	17	Malignant neoplasm of head of pancreas	Cancer of pancreas
C251	17	Malignant neoplasm of body of pancreas	Cancer of pancreas
C252	17	Malignant neoplasm of tail of pancreas	Cancer of pancreas
C253	17	Malignant neoplasm of pancreatic duct	Cancer of pancreas
C254	17	Malignant neoplasm of endocrine pancreas	Cancer of pancreas
C257	17	Malignant neoplasm of other parts of pancreas	Cancer of pancreas
C258	17	Malignant neoplasm of overlapping sites of pancreas	Cancer of pancreas
C259	17	Malignant neoplasm of pancreas, unspecified	Cancer of pancreas
C33	20	Malignant neoplasm of trachea	Cancer; other respiratory and intrathoracic
C3400	19	Malignant neoplasm of unspecified main bronchus	Cancer of bronchus; lung
C3401	19	Malignant neoplasm of right main bronchus	Cancer of bronchus; lung
C3402	19	Malignant neoplasm of left main bronchus	Cancer of bronchus; lung
C3410	19	Malignant neoplasm of upper lobe, unsp bronchus or lung	Cancer of bronchus; lung
C3411	19	Malignant neoplasm of upper lobe, right bronchus or lung	Cancer of bronchus; lung
C3412	19	Malignant neoplasm of upper lobe, left bronchus or lung	Cancer of bronchus; lung
C342	19	Malignant neoplasm of middle lobe, bronchus or lung	Cancer of bronchus; lung
C3430	19	Malignant neoplasm of lower lobe, unsp bronchus or lung	Cancer of bronchus; lung
C3431	19	Malignant neoplasm of lower lobe, right bronchus or lung	Cancer of bronchus; lung
C3432	19	Malignant neoplasm of lower lobe, left bronchus or lung	Cancer of bronchus; lung
C3480	19	Malignant neoplasm of ovrlp sites of unsp bronchus and lung	Cancer of bronchus; lung
C3481	19	Malignant neoplasm of ovrlp sites of right bronchus and lung	Cancer of bronchus; lung
C3482	19	Malignant neoplasm of ovrlp sites of left bronchus and lung	Cancer of bronchus; lung
C3490	19	Malignant neoplasm of unsp part of unsp bronchus or lung	Cancer of bronchus; lung
C3491	19	Malignant neoplasm of unsp part of right bronchus or lung	Cancer of bronchus; lung
C3492	19	Malignant neoplasm of unsp part of left bronchus or lung	Cancer of bronchus; lung
C384	20	Malignant neoplasm of pleura	Cancer; other respiratory and intrathoracic
C388	41	Malig neoplm of ovrlp sites of heart, mediastinum and pleura	Cancer; other and unspecified primary
C450	20	Mesothelioma of pleura	Cancer; other respiratory and intrathoracic
C480	18	Malignant neoplasm of retroperitoneum	Cancer of other GI organs; peritoneum

C481	18	Malignant neoplasm of specified parts of peritoneum	Cancer of other GI organs; peritoneum
C482	18	Malignant neoplasm of peritoneum, unspecified	Cancer of other GI organs; peritoneum
C488	18	Malig neoplasm of ovrlp sites of retroperiton and peritoneum	Cancer of other GI organs; peritoneum
C4A0	23	Merkel cell carcinoma of lip	Other non-epithelial cancer of skin
C4A10	23	Merkel cell carcinoma of unsp eyelid, including canthus	Other non-epithelial cancer of skin
C4A11	23	Merkel cell carcinoma of right eyelid, including canthus	Other non-epithelial cancer of skin
C4A12	23	Merkel cell carcinoma of left eyelid, including canthus	Other non-epithelial cancer of skin
C4A20	23	Merkel cell carcinoma of unsp ear and external auric canal	Other non-epithelial cancer of skin
C4A21	23	Merkel cell carcinoma of right ear and external auric canal	Other non-epithelial cancer of skin
C4A22	23	Merkel cell carcinoma of left ear and external auric canal	Other non-epithelial cancer of skin
C4A30	23	Merkel cell carcinoma of unspecified part of face	Other non-epithelial cancer of skin
C4A31	23	Merkel cell carcinoma of nose	Other non-epithelial cancer of skin
C4A39	23	Merkel cell carcinoma of other parts of face	Other non-epithelial cancer of skin
C4A4	23	Merkel cell carcinoma of scalp and neck	Other non-epithelial cancer of skin
C4A51	23	Merkel cell carcinoma of anal skin	Other non-epithelial cancer of skin
C4A52	23	Merkel cell carcinoma of skin of breast	Other non-epithelial cancer of skin
C4A59	23	Merkel cell carcinoma of other part of trunk	Other non-epithelial cancer of skin
C4A60	23	Merkel cell carcinoma of unsp upper limb, including shoulder	Other non-epithelial cancer of skin
C4A61	23	Merkel cell carcinoma of right upper limb, inc shoulder	Other non-epithelial cancer of skin
C4A62	23	Merkel cell carcinoma of left upper limb, including shoulder	Other non-epithelial cancer of skin
C4A70	23	Merkel cell carcinoma of unsp lower limb, including hip	Other non-epithelial cancer of skin
C4A71	23	Merkel cell carcinoma of right lower limb, including hip	Other non-epithelial cancer of skin
C4A72	23	Merkel cell carcinoma of left lower limb, including hip	Other non-epithelial cancer of skin
C4A8	23	Merkel cell carcinoma of overlapping sites	Other non-epithelial cancer of skin
C4A9	23	Merkel cell carcinoma, unspecified	Other non-epithelial cancer of skin
C561	27	Malignant neoplasm of right ovary	Cancer of ovary
C562	27	Malignant neoplasm of left ovary	Cancer of ovary
C569	27	Malignant neoplasm of unspecified ovary	Cancer of ovary
C5700	28	Malignant neoplasm of unspecified fallopian tube	Cancer of other female genital organs
C5701	28	Malignant neoplasm of right fallopian tube	Cancer of other female genital organs
C5702	28	Malignant neoplasm of left fallopian tube	Cancer of other female genital organs
C5710	28	Malignant neoplasm of unspecified broad ligament	Cancer of other female genital organs
C5711	28	Malignant neoplasm of right broad ligament	Cancer of other female genital organs
C5712	28	Malignant neoplasm of left broad ligament	Cancer of other female genital organs
C5720	28	Malignant neoplasm of unspecified round ligament	Cancer of other female genital organs
C5721	28	Malignant neoplasm of right round ligament	Cancer of other female genital organs
C5722	28	Malignant neoplasm of left round ligament	Cancer of other female genital organs
C573	28	Malignant neoplasm of parametrium	Cancer of other female genital organs
C574	28	Malignant neoplasm of uterine adnexa, unspecified	Cancer of other female genital organs
C710	35	Malignant neoplasm of cerebrum, except lobes and ventricles	Cancer of brain and nervous system
C711	35	Malignant neoplasm of frontal lobe	Cancer of brain and nervous system
C712	35	Malignant neoplasm of temporal lobe	Cancer of brain and nervous system
C713	35	Malignant neoplasm of parietal lobe	Cancer of brain and nervous system
C714	35	Malignant neoplasm of occipital lobe	Cancer of brain and nervous system
C715	35	Malignant neoplasm of cerebral ventricle	Cancer of brain and nervous system
C716	35	Malignant neoplasm of cerebellum	Cancer of brain and nervous system
C717	35	Malignant neoplasm of brain stem	Cancer of brain and nervous system
C718	35	Malignant neoplasm of overlapping sites of brain	Cancer of brain and nervous system
C719	35	Malignant neoplasm of brain, unspecified	Cancer of brain and nervous system
C770	42	Sec and unsp malig neoplasm of nodes of head, face and neck	Secondary malignancies
C771	42	Secondary and unsp malignant neoplasm of intrathorac nodes	Secondary malignancies

C772	42	Secondary and unsp malignant neoplasm of intra-abd nodes	Secondary malignancies
C773	42	Sec and unsp malig neoplasm of axilla and upper limb nodes	Secondary malignancies
C774	42	Sec and unsp malig neoplasm of inguinal and lower limb nodes	Secondary malignancies
C775	42	Secondary and unsp malignant neoplasm of intrapelv nodes	Secondary malignancies
C778	42	Sec and unsp malig neoplasm of nodes of multiple regions	Secondary malignancies
C779	42	Secondary and unsp malignant neoplasm of lymph node, unsp	Secondary malignancies
C7800	42	Secondary malignant neoplasm of unspecified lung	Secondary malignancies
C7801	42	Secondary malignant neoplasm of right lung	Secondary malignancies
C7802	42	Secondary malignant neoplasm of left lung	Secondary malignancies
C781	42	Secondary malignant neoplasm of mediastinum	Secondary malignancies
C782	42	Secondary malignant neoplasm of pleura	Secondary malignancies
C7830	42	Secondary malignant neoplasm of unsp respiratory organ	Secondary malignancies
C7839	42	Secondary malignant neoplasm of other respiratory organs	Secondary malignancies
C784	42	Secondary malignant neoplasm of small intestine	Secondary malignancies
C785	42	Secondary malignant neoplasm of large intestine and rectum	Secondary malignancies
C786	42	Secondary malignant neoplasm of retroperiton and peritoneum	Secondary malignancies
C787	42	Secondary malig neoplasm of liver and intrahepatic bile duct	Secondary malignancies
C7880	42	Secondary malignant neoplasm of unspecified digestive organ	Secondary malignancies
C7889	42	Secondary malignant neoplasm of other digestive organs	Secondary malignancies
C7900	42	[illegible] pelvis	Secondary malignancies
C7901	42	Secondary malignant neoplasm of r kidney and renal pelvis	Secondary malignancies
C7902	42	Secondary malignant neoplasm of left kidney and renal pelvis	Secondary malignancies
C7910	42	Secondary malignant neoplasm of unspecified urinary organs	Secondary malignancies
C7911	42	Secondary malignant neoplasm of bladder	Secondary malignancies
C7919	42	Secondary malignant neoplasm of other urinary organs	Secondary malignancies
C792	42	Secondary malignant neoplasm of skin	Secondary malignancies
C7931	42	Secondary malignant neoplasm of brain	Secondary malignancies
C7940	42	Secondary malignant neoplasm of unsp part of nervous system	Secondary malignancies
C7949	42	Secondary malignant neoplasm of oth parts of nervous system	Secondary malignancies
C7951	42	Secondary malignant neoplasm of bone	Secondary malignancies
C7952	42	Secondary malignant neoplasm of bone marrow	Secondary malignancies
C7960	42	Secondary malignant neoplasm of unspecified ovary	Secondary malignancies
C7961	42	Secondary malignant neoplasm of right ovary	Secondary malignancies
C7962	42	Secondary malignant neoplasm of left ovary	Secondary malignancies
C7970	42	Secondary malignant neoplasm of unspecified adrenal gland	Secondary malignancies
C7971	42	Secondary malignant neoplasm of right adrenal gland	Secondary malignancies
C7972	42	Secondary malignant neoplasm of left adrenal gland	Secondary malignancies
C7981	42	Secondary malignant neoplasm of breast	Secondary malignancies
C7982	42	Secondary malignant neoplasm of genital organs	Secondary malignancies
C7989	42	Secondary malignant neoplasm of other specified sites	Secondary malignancies
C799	42	Secondary malignant neoplasm of unspecified site	Secondary malignancies
C7A00	43	Malignant carcinoid tumor of unspecified site	[illegible] site
C7A010	18	Malignant carcinoid tumor of the duodenum	Cancer of other GI organs; peritoneum
C7A011	18	Malignant carcinoid tumor of the jejunum	Cancer of other GI organs; peritoneum
C7A012	18	Malignant carcinoid tumor of the ileum	Cancer of other GI organs; peritoneum
C7A019	18	Malignant carcinoid tumor of the sm int, unsp portion	Cancer of other GI organs; peritoneum
C7A020	14	Malignant carcinoid tumor of the appendix	Cancer of colon
C7A021	14	Malignant carcinoid tumor of the cecum	Cancer of colon
C7A022	14	Malignant carcinoid tumor of the ascending colon	Cancer of colon
C7A023	14	Malignant carcinoid tumor of the transverse colon	Cancer of colon
C7A024	14	Malignant carcinoid tumor of the descending colon	Cancer of colon

C7A025	14	Malignant carcinoid tumor of the sigmoid colon	Cancer of colon
C7A026	15	Malignant carcinoid tumor of the rectum	Cancer of rectum and anus
C7A029	14	Malignant carcinoid tumor of the lg int, unsp portion	Cancer of colon
C7A090	19	Malignant carcinoid tumor of the bronchus and lung	Cancer of bronchus; lung
C7A091	41	Malignant carcinoid tumor of the thymus	Cancer; other and unspecified primary
C7A092	13	Malignant carcinoid tumor of the stomach	Cancer of stomach
C7A093	33	Malignant carcinoid tumor of the kidney	Cancer of kidney and renal pelvis
C7A094	41	Malignant carcinoid tumor of the foregut NOS	Cancer; other and unspecified primary
C7A095	41	Malignant carcinoid tumor of the midgut NOS	Cancer; other and unspecified primary
C7A096	41	Malignant carcinoid tumor of the hindgut NOS	Cancer; other and unspecified primary
C7A098	43	Malignant carcinoid tumors of other sites	site
C7A1	43	Malignant poorly differentiated neuroendocrine tumors	site
C7A8	43	Other malignant neuroendocrine tumors	site
C7B00	43	Secondary carcinoid tumors, unspecified site	site
C7B01	42	Secondary carcinoid tumors of distant lymph nodes	Secondary malignancies
C7B02	42	Secondary carcinoid tumors of liver	Secondary malignancies
C7B03	42	Secondary carcinoid tumors of bone	Secondary malignancies
C7B04	42	Secondary carcinoid tumors of peritoneum	Secondary malignancies
C7B09	43	Secondary carcinoid tumors of other sites	site
C7B1	43	Secondary Merkel cell carcinoma	site
C7B8	43	Other secondary neuroendocrine tumors	site
C800	43	Disseminated malignant neoplasm, unspecified	site
C9100	39	Acute lymphoblastic leukemia not having achieved remission	Leukemias
C9101	39	Acute lymphoblastic leukemia, in remission	Leukemias
C9102	39	Acute lymphoblastic leukemia, in relapse	Leukemias
C9110	39	Chronic lymphocytic leuk of B-cell type not achieve remis	Leukemias
C9111	39	Chronic lymphocytic leukemia of B-cell type in remission	Leukemias
C9112	39	Chronic lymphocytic leukemia of B-cell type in relapse	Leukemias
C9130	39	Prolymphocytic leukemia of B-cell type not achieve remission	Leukemias
C9131	39	Prolymphocytic leukemia of B-cell type, in remission	Leukemias
C9132	39	Prolymphocytic leukemia of B-cell type, in relapse	Leukemias
C9140	39	Hairy cell leukemia not having achieved remission	Leukemias
C9141	39	Hairy cell leukemia, in remission	Leukemias
C9142	39	Hairy cell leukemia, in relapse	Leukemias
C9150	39	Adult T-cell lymph/leuk (HTLV-1-assoc) not achieve remission	Leukemias
C9151	39	Adult T-cell lymphoma/leukemia (HTLV-1-assoc), in remission	Leukemias
C9152	39	Adult T-cell lymphoma/leukemia (HTLV-1-assoc), in relapse	Leukemias
C9160	39	Prolymphocytic leukemia of T-cell type not achieve remission	Leukemias
C9161	39	Prolymphocytic leukemia of T-cell type, in remission	Leukemias
C9162	39	Prolymphocytic leukemia of T-cell type, in relapse	Leukemias
C9190	39	Lymphoid leukemia, unspecified not having achieved remission	Leukemias
C9191	39	Lymphoid leukemia, unspecified, in remission	Leukemias
C9192	39	Lymphoid leukemia, unspecified, in relapse	Leukemias
C91A0	39	Mature B-cell leukemia Burkitt-type not achieve remission	Leukemias
C91A1	39	Mature B-cell leukemia Burkitt-type, in remission	Leukemias
C91A2	39	Mature B-cell leukemia Burkitt-type, in relapse	Leukemias
C91Z0	39	Other lymphoid leukemia not having achieved remission	Leukemias
C91Z1	39	Other lymphoid leukemia, in remission	Leukemias
C91Z2	39	Other lymphoid leukemia, in relapse	Leukemias
C9200	39	Acute myeloblastic leukemia, not having achieved remission	Leukemias
C9201	39	Acute myeloblastic leukemia, in remission	Leukemias

C9202	39	Acute myeloblastic leukemia, in relapse	Leukemias
C9210	39	Chronic myeloid leuk, BCR/ABL-positive, not achieve remis	Leukemias
C9211	39	Chronic myeloid leukemia, BCR/ABL-positive, in remission	Leukemias
C9212	39	Chronic myeloid leukemia, BCR/ABL-positive, in relapse	Leukemias
C9220	39	Atyp chronic myeloid leuk, BCR/ABL-neg, not achieve remis	Leukemias
C9221	39	Atypical chronic myeloid leukemia, BCR/ABL-neg, in remission	Leukemias
C9222	39	Atypical chronic myeloid leukemia, BCR/ABL-neg, in relapse	Leukemias
C9230	39	Myeloid sarcoma, not having achieved remission	Leukemias
C9231	39	Myeloid sarcoma, in remission	Leukemias
C9232	39	Myeloid sarcoma, in relapse	Leukemias
C9240	39	Acute promyelocytic leukemia, not having achieved remission	Leukemias
C9241	39	Acute promyelocytic leukemia, in remission	Leukemias
C9242	39	Acute promyelocytic leukemia, in relapse	Leukemias
C9250	39	remission	Leukemias
C9251	39	Acute myelomonocytic leukemia, in remission	Leukemias
C9252	39	Acute myelomonocytic leukemia, in relapse	Leukemias
C9260	39	remis	Leukemias
C9261	39	Acute myeloid leukemia with 11q23-abnormality in remission	Leukemias
C9262	39	Acute myeloid leukemia with 11q23-abnormality in relapse	Leukemias
C9290	39	Myeloid leukemia, unspecified, not having achieved remission	Leukemias
C9291	39	Myeloid leukemia, unspecified in remission	Leukemias
C9292	39	Myeloid leukemia, unspecified in relapse	Leukemias
C92A0	39	Acute myeloid leuk w multilin dysplasia, not achieve remis	Leukemias
C92A1	39	Acute myeloid leukemia w multilin dysplasia, in remission	Leukemias
C92A2	39	Acute myeloid leukemia w multilineage dysplasia, in relapse	Leukemias
C92Z0	39	Other myeloid leukemia not having achieved remission	Leukemias
C92Z1	39	Other myeloid leukemia, in remission	Leukemias
C92Z2	39	Other myeloid leukemia, in relapse	Leukemias
C9300	39	Acute monoblastic/monocytic leukemia, not achieve remission	Leukemias
C9301	39	Acute monoblastic/monocytic leukemia, in remission	Leukemias
C9302	39	Acute monoblastic/monocytic leukemia, in relapse	Leukemias
C9310	39	Chronic myelomonocytic leukemia not achieve remission	Leukemias
C9311	39	Chronic myelomonocytic leukemia, in remission	Leukemias
C9312	39	Chronic myelomonocytic leukemia, in relapse	Leukemias
C9330	39	Juvenile myelomonocytic leukemia, not achieve remission	Leukemias
C9331	39	Juvenile myelomonocytic leukemia, in remission	Leukemias
C9332	39	Juvenile myelomonocytic leukemia, in relapse	Leukemias
C9390	39	Monocytic leukemia, unsp, not having achieved remission	Leukemias
C9391	39	Monocytic leukemia, unspecified in remission	Leukemias
C9392	39	Monocytic leukemia, unspecified in relapse	Leukemias
C93Z0	39	Other monocytic leukemia, not having achieved remission	Leukemias
C93Z1	39	Other monocytic leukemia, in remission	Leukemias
C93Z2	39	Other monocytic leukemia, in relapse	Leukemias
C9400	39	Acute erythroid leukemia, not having achieved remission	Leukemias
C9401	39	Acute erythroid leukemia, in remission	Leukemias
C9402	39	Acute erythroid leukemia, in relapse	Leukemias
C9420	39	Acute megakaryoblastic leukemia not achieve remission	Leukemias
C9421	39	Acute megakaryoblastic leukemia, in remission	Leukemias
C9422	39	Acute megakaryoblastic leukemia, in relapse	Leukemias
C9430	39	Mast cell leukemia not having achieved remission	Leukemias
C9431	39	Mast cell leukemia, in remission	Leukemias

C9432	39	Mast cell leukemia, in relapse	Leukemias
C9480	39	Other specified leukemias not having achieved remission	Leukemias
C9481	39	Other specified leukemias, in remission	Leukemias
C9482	39	Other specified leukemias, in relapse	Leukemias
C9500	39	Acute leukemia of unsp cell type not achieve remission	Leukemias
C9501	39	Acute leukemia of unspecified cell type, in remission	Leukemias
C9502	39	Acute leukemia of unspecified cell type, in relapse	Leukemias
C9510	39	Chronic leukemia of unsp cell type not achieve remission	Leukemias
C9511	39	Chronic leukemia of unspecified cell type, in remission	Leukemias
C9512	39	Chronic leukemia of unspecified cell type, in relapse	Leukemias
C9590	39	Leukemia, unspecified not having achieved remission	Leukemias
C9591	39	Leukemia, unspecified, in remission	Leukemias
C9592	39	Leukemia, unspecified, in relapse	Leukemias
J910	42	Malignant pleural effusion	Secondary malignancies

ICD10 to CCS

CCS	ICD10	CCS_DESCRIPTION
1	A150	Tuberculosis
1	A154	Tuberculosis
1	A155	Tuberculosis
1	A156	Tuberculosis
1	A157	Tuberculosis
1	A158	Tuberculosis
1	A159	Tuberculosis
1	A170	Tuberculosis
1	A171	Tuberculosis
1	A1781	Tuberculosis
1	A1782	Tuberculosis
1	A1783	Tuberculosis
1	A1789	Tuberculosis
1	A179	Tuberculosis
1	A1801	Tuberculosis
1	A1802	Tuberculosis
1	A1803	Tuberculosis
1	A1809	Tuberculosis
1	A1810	Tuberculosis
1	A1811	Tuberculosis
1	A1812	Tuberculosis
1	A1813	Tuberculosis
1	A1814	Tuberculosis
1	A1815	Tuberculosis
1	A1816	Tuberculosis
1	A1817	Tuberculosis
1	A1818	Tuberculosis
1	A182	Tuberculosis
1	A1831	Tuberculosis
1	A1832	Tuberculosis
1	A1839	Tuberculosis
1	A184	Tuberculosis
1	A1850	Tuberculosis
1	A1851	Tuberculosis
1	A1852	Tuberculosis
1	A1853	Tuberculosis
1	A1854	Tuberculosis
1	A1859	Tuberculosis
1	A186	Tuberculosis
1	A187	Tuberculosis
1	A1881	Tuberculosis
1	A1882	Tuberculosis
1	A1883	Tuberculosis
1	A1884	Tuberculosis
1	A1885	Tuberculosis
1	A1889	Tuberculosis
1	A190	Tuberculosis
1	A191	Tuberculosis

1	A192	Tuberculosis
1	A198	Tuberculosis
1	A199	Tuberculosis
1	B900	Tuberculosis
1	B901	Tuberculosis
1	B902	Tuberculosis
1	B908	Tuberculosis
1	B909	Tuberculosis
1	Z8611	Tuberculosis
2	A021	Septicemia (except in labor)
2	A207	Septicemia (except in labor)
2	A227	Septicemia (except in labor)
2	A267	Septicemia (except in labor)
2	A327	Septicemia (except in labor)
2	A392	Septicemia (except in labor)
2	A393	Septicemia (except in labor)
2	A394	Septicemia (except in labor)
2	A400	Septicemia (except in labor)
2	A401	Septicemia (except in labor)
2	A403	Septicemia (except in labor)
2	A408	Septicemia (except in labor)
2	A409	Septicemia (except in labor)
2	A4101	Septicemia (except in labor)
2	A4102	Septicemia (except in labor)
2	A411	Septicemia (except in labor)
2	A412	Septicemia (except in labor)
2	A413	Septicemia (except in labor)
2	A414	Septicemia (except in labor)
2	A4150	Septicemia (except in labor)
2	A4151	Septicemia (except in labor)
2	A4152	Septicemia (except in labor)
2	A4153	Septicemia (except in labor)
2	A4159	Septicemia (except in labor)
2	A4181	Septicemia (except in labor)
2	A4189	Septicemia (except in labor)
2	A419	Septicemia (except in labor)
2	A427	Septicemia (except in labor)
2	A5486	Septicemia (except in labor)
2	B007	Septicemia (except in labor)
2	B377	Septicemia (except in labor)
2	I76	Septicemia (except in labor)
2	P360	Septicemia (except in labor)
2	P3610	Septicemia (except in labor)
2	P3619	Septicemia (except in labor)
2	P362	Septicemia (except in labor)
2	P3630	Septicemia (except in labor)
2	P3639	Septicemia (except in labor)
2	P364	Septicemia (except in labor)
2	P365	Septicemia (except in labor)
2	P368	Septicemia (except in labor)
2	P369	Septicemia (except in labor)

2	R6520	Septicemia (except in labor)
3	A0102	Bacterial infection; unspecified site
3	A200	Bacterial infection; unspecified site
3	A208	Bacterial infection; unspecified site
3	A209	Bacterial infection; unspecified site
3	A217	Bacterial infection; unspecified site
3	A218	Bacterial infection; unspecified site
3	A219	Bacterial infection; unspecified site
3	A228	Bacterial infection; unspecified site
3	A229	Bacterial infection; unspecified site
3	A230	Bacterial infection; unspecified site
3	A231	Bacterial infection; unspecified site
3	A232	Bacterial infection; unspecified site
3	A233	Bacterial infection; unspecified site
3	A238	Bacterial infection; unspecified site
3	A239	Bacterial infection; unspecified site
3	A240	Bacterial infection; unspecified site
3	A241	Bacterial infection; unspecified site
3	A242	Bacterial infection; unspecified site
3	A243	Bacterial infection; unspecified site
3	A249	Bacterial infection; unspecified site
3	A250	Bacterial infection; unspecified site
3	A251	Bacterial infection; unspecified site
3	A259	Bacterial infection; unspecified site
3	A260	Bacterial infection; unspecified site
3	A268	Bacterial infection; unspecified site
3	A269	Bacterial infection; unspecified site
3	A2789	Bacterial infection; unspecified site
3	A280	Bacterial infection; unspecified site
3	A281	Bacterial infection; unspecified site
3	A282	Bacterial infection; unspecified site
3	A288	Bacterial infection; unspecified site
3	A289	Bacterial infection; unspecified site
3	A300	Bacterial infection; unspecified site
3	A301	Bacterial infection; unspecified site
3	A302	Bacterial infection; unspecified site
3	A303	Bacterial infection; unspecified site
3	A304	Bacterial infection; unspecified site
3	A305	Bacterial infection; unspecified site
3	A308	Bacterial infection; unspecified site
3	A309	Bacterial infection; unspecified site
3	A312	Bacterial infection; unspecified site
3	A318	Bacterial infection; unspecified site
3	A319	Bacterial infection; unspecified site
3	A320	Bacterial infection; unspecified site
3	A3281	Bacterial infection; unspecified site
3	A3282	Bacterial infection; unspecified site
3	A3289	Bacterial infection; unspecified site
3	A329	Bacterial infection; unspecified site
3	A35	Bacterial infection; unspecified site
3	A3682	Bacterial infection; unspecified site

3	A3683	Bacterial infection; unspecified site
3	A3684	Bacterial infection; unspecified site
3	A3689	Bacterial infection; unspecified site
3	A369	Bacterial infection; unspecified site
3	A3700	Bacterial infection; unspecified site
3	A3710	Bacterial infection; unspecified site
3	A3780	Bacterial infection; unspecified site
3	A3781	Bacterial infection; unspecified site
3	A3790	Bacterial infection; unspecified site
3	A3791	Bacterial infection; unspecified site
3	A380	Bacterial infection; unspecified site
3	A388	Bacterial infection; unspecified site
3	A389	Bacterial infection; unspecified site
3	A391	Bacterial infection; unspecified site
3	A3982	Bacterial infection; unspecified site
3	A3989	Bacterial infection; unspecified site
3	A399	Bacterial infection; unspecified site
3	A420	Bacterial infection; unspecified site
3	A421	Bacterial infection; unspecified site
3	A422	Bacterial infection; unspecified site
3	A4289	Bacterial infection; unspecified site
3	A429	Bacterial infection; unspecified site
3	A438	Bacterial infection; unspecified site
3	A439	Bacterial infection; unspecified site
3	A440	Bacterial infection; unspecified site
3	A441	Bacterial infection; unspecified site
3	A448	Bacterial infection; unspecified site
3	A449	Bacterial infection; unspecified site
3	A480	Bacterial infection; unspecified site
3	A482	Bacterial infection; unspecified site
3	A483	Bacterial infection; unspecified site
3	A484	Bacterial infection; unspecified site
3	A4852	Bacterial infection; unspecified site
3	A488	Bacterial infection; unspecified site
3	A4901	Bacterial infection; unspecified site
3	A4902	Bacterial infection; unspecified site
3	A491	Bacterial infection; unspecified site
3	A492	Bacterial infection; unspecified site
3	A493	Bacterial infection; unspecified site
3	A498	Bacterial infection; unspecified site
3	A499	Bacterial infection; unspecified site
3	A70	Bacterial infection; unspecified site
3	A7489	Bacterial infection; unspecified site
3	A749	Bacterial infection; unspecified site
3	A78	Bacterial infection; unspecified site
3	B471	Bacterial infection; unspecified site
3	B479	Bacterial infection; unspecified site
3	B950	Bacterial infection; unspecified site
3	B951	Bacterial infection; unspecified site
3	B952	Bacterial infection; unspecified site
3	B953	Bacterial infection; unspecified site

3	B954	Bacterial infection; unspecified site
3	B955	Bacterial infection; unspecified site
3	B9561	Bacterial infection; unspecified site
3	B9562	Bacterial infection; unspecified site
3	B957	Bacterial infection; unspecified site
3	B958	Bacterial infection; unspecified site
3	B960	Bacterial infection; unspecified site
3	B961	Bacterial infection; unspecified site
3	B9620	Bacterial infection; unspecified site
3	B9621	Bacterial infection; unspecified site
3	B9622	Bacterial infection; unspecified site
3	B9623	Bacterial infection; unspecified site
3	B9629	Bacterial infection; unspecified site
3	B963	Bacterial infection; unspecified site
3	B964	Bacterial infection; unspecified site
3	B965	Bacterial infection; unspecified site
3	B966	Bacterial infection; unspecified site
3	B967	Bacterial infection; unspecified site
3	B9681	Bacterial infection; unspecified site
3	B9682	Bacterial infection; unspecified site
3	B9689	Bacterial infection; unspecified site
3	I00	Bacterial infection; unspecified site
3	I029	Bacterial infection; unspecified site
3	K9081	Bacterial infection; unspecified site
3	R7881	Bacterial infection; unspecified site
3	Z1610	Bacterial infection; unspecified site
3	Z1611	Bacterial infection; unspecified site
3	Z1612	Bacterial infection; unspecified site
3	Z1619	Bacterial infection; unspecified site
3	Z1620	Bacterial infection; unspecified site
3	Z1621	Bacterial infection; unspecified site
3	Z1622	Bacterial infection; unspecified site
3	Z1623	Bacterial infection; unspecified site
3	Z1624	Bacterial infection; unspecified site
3	Z1629	Bacterial infection; unspecified site
3	Z1630	Bacterial infection; unspecified site
3	Z16341	Bacterial infection; unspecified site
3	Z16342	Bacterial infection; unspecified site
3	Z1635	Bacterial infection; unspecified site
3	Z1639	Bacterial infection; unspecified site
3	Z8614	Bacterial infection; unspecified site
4	B350	Mycoses
4	B351	Mycoses
4	B352	Mycoses
4	B353	Mycoses
4	B354	Mycoses
4	B355	Mycoses
4	B356	Mycoses
4	B358	Mycoses
4	B359	Mycoses
4	B360	Mycoses

4	B361	Mycoses
4	B362	Mycoses
4	B363	Mycoses
4	B368	Mycoses
4	B369	Mycoses
4	B370	Mycoses
4	B372	Mycoses
4	B373	Mycoses
4	B3741	Mycoses
4	B3742	Mycoses
4	B3749	Mycoses
4	B3781	Mycoses
4	B3782	Mycoses
4	B3783	Mycoses
4	B3784	Mycoses
4	B3789	Mycoses
4	B379	Mycoses
4	B383	Mycoses
4	B387	Mycoses
4	B3881	Mycoses
4	B3889	Mycoses
4	B389	Mycoses
4	B393	Mycoses
4	B394	Mycoses
4	B395	Mycoses
4	B399	Mycoses
4	B400	Mycoses
4	B401	Mycoses
4	B402	Mycoses
4	B403	Mycoses
4	B407	Mycoses
4	B4089	Mycoses
4	B409	Mycoses
4	B410	Mycoses
4	B417	Mycoses
4	B418	Mycoses
4	B419	Mycoses
4	B420	Mycoses
4	B421	Mycoses
4	B427	Mycoses
4	B4281	Mycoses
4	B4282	Mycoses
4	B4289	Mycoses
4	B429	Mycoses
4	B430	Mycoses
4	B431	Mycoses
4	B432	Mycoses
4	B438	Mycoses
4	B439	Mycoses
4	B440	Mycoses
4	B441	Mycoses

4	B442	Mycoses
4	B447	Mycoses
4	B4489	Mycoses
4	B449	Mycoses
4	B450	Mycoses
4	B452	Mycoses
4	B453	Mycoses
4	B457	Mycoses
4	B458	Mycoses
4	B459	Mycoses
4	B460	Mycoses
4	B461	Mycoses
4	B462	Mycoses
4	B463	Mycoses
4	B464	Mycoses
4	B465	Mycoses
4	B468	Mycoses
4	B469	Mycoses
4	B470	Mycoses
4	B480	Mycoses
4	B481	Mycoses
4	B482	Mycoses
4	B483	Mycoses
4	B484	Mycoses
4	B488	Mycoses
4	B49	Mycoses
5	B20	HIV infection
5	B9735	HIV infection
5	R75	HIV infection
5	Z21	HIV infection
6	B150	Hepatitis
6	B159	Hepatitis
6	B160	Hepatitis
6	B161	Hepatitis
6	B162	Hepatitis
6	B169	Hepatitis
6	B170	Hepatitis
6	B1710	Hepatitis
6	B1711	Hepatitis
6	B172	Hepatitis
6	B178	Hepatitis
6	B179	Hepatitis
6	B180	Hepatitis
6	B181	Hepatitis
6	B182	Hepatitis
6	B188	Hepatitis
6	B189	Hepatitis
6	B190	Hepatitis
6	B1910	Hepatitis
6	B1911	Hepatitis
6	B1920	Hepatitis

6	B1921	Hepatitis
6	B199	Hepatitis
6	B251	Hepatitis
6	B2681	Hepatitis
6	B581	Hepatitis
6	B942	Hepatitis
6	K710	Hepatitis
6	K712	Hepatitis
6	K713	Hepatitis
6	K714	Hepatitis
6	K7150	Hepatitis
6	K7151	Hepatitis
6	K716	Hepatitis
6	K717	Hepatitis
6	K730	Hepatitis
6	K731	Hepatitis
6	K732	Hepatitis
6	K738	Hepatitis
6	K739	Hepatitis
6	K752	Hepatitis
6	K753	Hepatitis
6	K754	Hepatitis
6	K7581	Hepatitis
6	K764	Hepatitis
7	A6000	Viral infection
7	A6001	Viral infection
7	A6002	Viral infection
7	A6003	Viral infection
7	A6004	Viral infection
7	A6009	Viral infection
7	A601	Viral infection
7	A609	Viral infection
7	A630	Viral infection
7	A820	Viral infection
7	A821	Viral infection
7	A829	Viral infection
7	A91	Viral infection
7	A920	Viral infection
7	A921	Viral infection
7	A9230	Viral infection
7	A9232	Viral infection
7	A9239	Viral infection
7	A924	Viral infection
7	A928	Viral infection
7	A929	Viral infection
7	A930	Viral infection
7	A931	Viral infection
7	A932	Viral infection
7	A938	Viral infection
7	A94	Viral infection
7	A950	Viral infection

7	A951	Viral infection
7	A959	Viral infection
7	A960	Viral infection
7	A961	Viral infection
7	A962	Viral infection
7	A968	Viral infection
7	A969	Viral infection
7	A980	Viral infection
7	A981	Viral infection
7	A982	Viral infection
7	A983	Viral infection
7	A984	Viral infection
7	A985	Viral infection
7	A988	Viral infection
7	A99	Viral infection
7	B000	Viral infection
7	B001	Viral infection
7	B002	Viral infection
7	B0081	Viral infection
7	B0082	Viral infection
7	B0089	Viral infection
7	B009	Viral infection
7	B0112	Viral infection
7	B0181	Viral infection
7	B0189	Viral infection
7	B019	Viral infection
7	B0221	Viral infection
7	B0222	Viral infection
7	B0223	Viral infection
7	B0224	Viral infection
7	B0229	Viral infection
7	B027	Viral infection
7	B028	Viral infection
7	B029	Viral infection
7	B03	Viral infection
7	B04	Viral infection
7	B054	Viral infection
7	B0589	Viral infection
7	B059	Viral infection
7	B0600	Viral infection
7	B0609	Viral infection
7	B0689	Viral infection
7	B069	Viral infection
7	B070	Viral infection
7	B078	Viral infection
7	B079	Viral infection
7	B08010	Viral infection
7	B08011	Viral infection
7	B0802	Viral infection
7	B0803	Viral infection
7	B0804	Viral infection

7	B0809	Viral infection
7	B081	Viral infection
7	B0820	Viral infection
7	B0821	Viral infection
7	B0822	Viral infection
7	B083	Viral infection
7	B084	Viral infection
7	B085	Viral infection
7	B0860	Viral infection
7	B0861	Viral infection
7	B0862	Viral infection
7	B0869	Viral infection
7	B0870	Viral infection
7	B0871	Viral infection
7	B0872	Viral infection
7	B0879	Viral infection
7	B088	Viral infection
7	B09	Viral infection
7	B1081	Viral infection
7	B1082	Viral infection
7	B1089	Viral infection
7	B250	Viral infection
7	B252	Viral infection
7	B258	Viral infection
7	B259	Viral infection
7	B260	Viral infection
7	B263	Viral infection
7	B2684	Viral infection
7	B2689	Viral infection
7	B269	Viral infection
7	B2700	Viral infection
7	B2709	Viral infection
7	B2710	Viral infection
7	B2719	Viral infection
7	B2780	Viral infection
7	B2789	Viral infection
7	B2790	Viral infection
7	B2799	Viral infection
7	B330	Viral infection
7	B331	Viral infection
7	B333	Viral infection
7	B334	Viral infection
7	B338	Viral infection
7	B340	Viral infection
7	B341	Viral infection
7	B342	Viral infection
7	B343	Viral infection
7	B344	Viral infection
7	B348	Viral infection
7	B349	Viral infection
7	B970	Viral infection

7	B9710	Viral infection
7	B9711	Viral infection
7	B9712	Viral infection
7	B9719	Viral infection
7	B9721	Viral infection
7	B9729	Viral infection
7	B9730	Viral infection
7	B9731	Viral infection
7	B9732	Viral infection
7	B9733	Viral infection
7	B9734	Viral infection
7	B9739	Viral infection
7	B974	Viral infection
7	B975	Viral infection
7	B976	Viral infection
7	B977	Viral infection
7	B9781	Viral infection
7	B9789	Viral infection
7	Z1633	Viral infection
8	A270	Other infections; including parasitic
8	A279	Other infections; including parasitic
8	A5900	Other infections; including parasitic
8	A5901	Other infections; including parasitic
8	A5902	Other infections; including parasitic
8	A5903	Other infections; including parasitic
8	A5909	Other infections; including parasitic
8	A598	Other infections; including parasitic
8	A599	Other infections; including parasitic
8	A65	Other infections; including parasitic
8	A660	Other infections; including parasitic
8	A661	Other infections; including parasitic
8	A662	Other infections; including parasitic
8	A663	Other infections; including parasitic
8	A664	Other infections; including parasitic
8	A665	Other infections; including parasitic
8	A666	Other infections; including parasitic
8	A667	Other infections; including parasitic
8	A668	Other infections; including parasitic
8	A669	Other infections; including parasitic
8	A670	Other infections; including parasitic
8	A671	Other infections; including parasitic
8	A672	Other infections; including parasitic
8	A673	Other infections; including parasitic
8	A679	Other infections; including parasitic
8	A680	Other infections; including parasitic
8	A681	Other infections; including parasitic
8	A689	Other infections; including parasitic
8	A690	Other infections; including parasitic
8	A691	Other infections; including parasitic
8	A6920	Other infections; including parasitic
8	A6922	Other infections; including parasitic

8	A6929	Other infections; including parasitic
8	A698	Other infections; including parasitic
8	A699	Other infections; including parasitic
8	A750	Other infections; including parasitic
8	A751	Other infections; including parasitic
8	A752	Other infections; including parasitic
8	A753	Other infections; including parasitic
8	A759	Other infections; including parasitic
8	A770	Other infections; including parasitic
8	A771	Other infections; including parasitic
8	A772	Other infections; including parasitic
8	A773	Other infections; including parasitic
8	A7740	Other infections; including parasitic
8	A7741	Other infections; including parasitic
8	A7749	Other infections; including parasitic
8	A778	Other infections; including parasitic
8	A779	Other infections; including parasitic
8	A790	Other infections; including parasitic
8	A791	Other infections; including parasitic
8	A7981	Other infections; including parasitic
8	A7989	Other infections; including parasitic
8	A799	Other infections; including parasitic
8	B500	Other infections; including parasitic
8	B508	Other infections; including parasitic
8	B509	Other infections; including parasitic
8	B510	Other infections; including parasitic
8	B518	Other infections; including parasitic
8	B519	Other infections; including parasitic
8	B520	Other infections; including parasitic
8	B528	Other infections; including parasitic
8	B529	Other infections; including parasitic
8	B530	Other infections; including parasitic
8	B531	Other infections; including parasitic
8	B538	Other infections; including parasitic
8	B54	Other infections; including parasitic
8	B550	Other infections; including parasitic
8	B551	Other infections; including parasitic
8	B552	Other infections; including parasitic
8	B559	Other infections; including parasitic
8	B560	Other infections; including parasitic
8	B561	Other infections; including parasitic
8	B569	Other infections; including parasitic
8	B570	Other infections; including parasitic
8	B571	Other infections; including parasitic
8	B572	Other infections; including parasitic
8	B5730	Other infections; including parasitic
8	B5739	Other infections; including parasitic
8	B5740	Other infections; including parasitic
8	B5749	Other infections; including parasitic
8	B575	Other infections; including parasitic
8	B5800	Other infections; including parasitic

8	B5882	Other infections; including parasitic
8	B5883	Other infections; including parasitic
8	B5889	Other infections; including parasitic
8	B589	Other infections; including parasitic
8	B600	Other infections; including parasitic
8	B6010	Other infections; including parasitic
8	B6012	Other infections; including parasitic
8	B6013	Other infections; including parasitic
8	B6019	Other infections; including parasitic
8	B602	Other infections; including parasitic
8	B608	Other infections; including parasitic
8	B64	Other infections; including parasitic
8	B650	Other infections; including parasitic
8	B651	Other infections; including parasitic
8	B652	Other infections; including parasitic
8	B658	Other infections; including parasitic
8	B659	Other infections; including parasitic
8	B660	Other infections; including parasitic
8	B661	Other infections; including parasitic
8	B662	Other infections; including parasitic
8	B663	Other infections; including parasitic
8	B664	Other infections; including parasitic
8	B665	Other infections; including parasitic
8	B668	Other infections; including parasitic
8	B669	Other infections; including parasitic
8	B670	Other infections; including parasitic
8	B671	Other infections; including parasitic
8	B672	Other infections; including parasitic
8	B6731	Other infections; including parasitic
8	B6732	Other infections; including parasitic
8	B6739	Other infections; including parasitic
8	B674	Other infections; including parasitic
8	B675	Other infections; including parasitic
8	B6761	Other infections; including parasitic
8	B6769	Other infections; including parasitic
8	B677	Other infections; including parasitic
8	B678	Other infections; including parasitic
8	B6790	Other infections; including parasitic
8	B6799	Other infections; including parasitic
8	B680	Other infections; including parasitic
8	B681	Other infections; including parasitic
8	B689	Other infections; including parasitic
8	B690	Other infections; including parasitic
8	B691	Other infections; including parasitic
8	B6981	Other infections; including parasitic
8	B6989	Other infections; including parasitic
8	B699	Other infections; including parasitic
8	B700	Other infections; including parasitic
8	B701	Other infections; including parasitic
8	B710	Other infections; including parasitic
8	B711	Other infections; including parasitic

8	B718	Other infections; including parasitic
8	B719	Other infections; including parasitic
8	B72	Other infections; including parasitic
8	B7300	Other infections; including parasitic
8	B7301	Other infections; including parasitic
8	B7302	Other infections; including parasitic
8	B7309	Other infections; including parasitic
8	B731	Other infections; including parasitic
8	B740	Other infections; including parasitic
8	B741	Other infections; including parasitic
8	B742	Other infections; including parasitic
8	B743	Other infections; including parasitic
8	B744	Other infections; including parasitic
8	B748	Other infections; including parasitic
8	B749	Other infections; including parasitic
8	B75	Other infections; including parasitic
8	B760	Other infections; including parasitic
8	B761	Other infections; including parasitic
8	B768	Other infections; including parasitic
8	B769	Other infections; including parasitic
8	B770	Other infections; including parasitic
8	B7789	Other infections; including parasitic
8	B779	Other infections; including parasitic
8	B780	Other infections; including parasitic
8	B781	Other infections; including parasitic
8	B787	Other infections; including parasitic
8	B789	Other infections; including parasitic
8	B79	Other infections; including parasitic
8	B80	Other infections; including parasitic
8	B810	Other infections; including parasitic
8	B811	Other infections; including parasitic
8	B812	Other infections; including parasitic
8	B813	Other infections; including parasitic
8	B814	Other infections; including parasitic
8	B818	Other infections; including parasitic
8	B820	Other infections; including parasitic
8	B829	Other infections; including parasitic
8	B830	Other infections; including parasitic
8	B831	Other infections; including parasitic
8	B832	Other infections; including parasitic
8	B833	Other infections; including parasitic
8	B834	Other infections; including parasitic
8	B838	Other infections; including parasitic
8	B839	Other infections; including parasitic
8	B850	Other infections; including parasitic
8	B851	Other infections; including parasitic
8	B852	Other infections; including parasitic
8	B853	Other infections; including parasitic
8	B854	Other infections; including parasitic
8	B86	Other infections; including parasitic
8	B870	Other infections; including parasitic

8	B871	Other infections; including parasitic
8	B872	Other infections; including parasitic
8	B873	Other infections; including parasitic
8	B874	Other infections; including parasitic
8	B8781	Other infections; including parasitic
8	B8782	Other infections; including parasitic
8	B8789	Other infections; including parasitic
8	B879	Other infections; including parasitic
8	B880	Other infections; including parasitic
8	B881	Other infections; including parasitic
8	B882	Other infections; including parasitic
8	B883	Other infections; including parasitic
8	B888	Other infections; including parasitic
8	B889	Other infections; including parasitic
8	B89	Other infections; including parasitic
8	B92	Other infections; including parasitic
8	B948	Other infections; including parasitic
8	B949	Other infections; including parasitic
8	B998	Other infections; including parasitic
8	B999	Other infections; including parasitic
8	L946	Other infections; including parasitic
8	Z1631	Other infections; including parasitic
8	Z1632	Other infections; including parasitic
8	Z8613	Other infections; including parasitic
8	Z8619	Other infections; including parasitic
9	A5001	Sexually transmitted infections (not HIV or hepatitis)
9	A5002	Sexually transmitted infections (not HIV or hepatitis)
9	A5003	Sexually transmitted infections (not HIV or hepatitis)
9	A5004	Sexually transmitted infections (not HIV or hepatitis)
9	A5005	Sexually transmitted infections (not HIV or hepatitis)
9	A5006	Sexually transmitted infections (not HIV or hepatitis)
9	A5007	Sexually transmitted infections (not HIV or hepatitis)
9	A5008	Sexually transmitted infections (not HIV or hepatitis)
9	A5009	Sexually transmitted infections (not HIV or hepatitis)
9	A501	Sexually transmitted infections (not HIV or hepatitis)
9	A502	Sexually transmitted infections (not HIV or hepatitis)
9	A5030	Sexually transmitted infections (not HIV or hepatitis)
9	A5031	Sexually transmitted infections (not HIV or hepatitis)
9	A5032	Sexually transmitted infections (not HIV or hepatitis)
9	A5039	Sexually transmitted infections (not HIV or hepatitis)
9	A5040	Sexually transmitted infections (not HIV or hepatitis)
9	A5041	Sexually transmitted infections (not HIV or hepatitis)
9	A5042	Sexually transmitted infections (not HIV or hepatitis)
9	A5043	Sexually transmitted infections (not HIV or hepatitis)
9	A5044	Sexually transmitted infections (not HIV or hepatitis)
9	A5045	Sexually transmitted infections (not HIV or hepatitis)
9	A5049	Sexually transmitted infections (not HIV or hepatitis)
9	A5051	Sexually transmitted infections (not HIV or hepatitis)
9	A5052	Sexually transmitted infections (not HIV or hepatitis)
9	A5053	Sexually transmitted infections (not HIV or hepatitis)
9	A5054	Sexually transmitted infections (not HIV or hepatitis)

9	A5055	Sexually transmitted infections (not HIV or hepatitis)
9	A5056	Sexually transmitted infections (not HIV or hepatitis)
9	A5057	Sexually transmitted infections (not HIV or hepatitis)
9	A5059	Sexually transmitted infections (not HIV or hepatitis)
9	A506	Sexually transmitted infections (not HIV or hepatitis)
9	A507	Sexually transmitted infections (not HIV or hepatitis)
9	A509	Sexually transmitted infections (not HIV or hepatitis)
9	A510	Sexually transmitted infections (not HIV or hepatitis)
9	A511	Sexually transmitted infections (not HIV or hepatitis)
9	A512	Sexually transmitted infections (not HIV or hepatitis)
9	A5131	Sexually transmitted infections (not HIV or hepatitis)
9	A5132	Sexually transmitted infections (not HIV or hepatitis)
9	A5139	Sexually transmitted infections (not HIV or hepatitis)
9	A5141	Sexually transmitted infections (not HIV or hepatitis)
9	A5142	Sexually transmitted infections (not HIV or hepatitis)
9	A5143	Sexually transmitted infections (not HIV or hepatitis)
9	A5144	Sexually transmitted infections (not HIV or hepatitis)
9	A5145	Sexually transmitted infections (not HIV or hepatitis)
9	A5146	Sexually transmitted infections (not HIV or hepatitis)
9	A5149	Sexually transmitted infections (not HIV or hepatitis)
9	A515	Sexually transmitted infections (not HIV or hepatitis)
9	A519	Sexually transmitted infections (not HIV or hepatitis)
9	A5200	Sexually transmitted infections (not HIV or hepatitis)
9	A5201	Sexually transmitted infections (not HIV or hepatitis)
9	A5202	Sexually transmitted infections (not HIV or hepatitis)
9	A5203	Sexually transmitted infections (not HIV or hepatitis)
9	A5204	Sexually transmitted infections (not HIV or hepatitis)
9	A5205	Sexually transmitted infections (not HIV or hepatitis)
9	A5206	Sexually transmitted infections (not HIV or hepatitis)
9	A5209	Sexually transmitted infections (not HIV or hepatitis)
9	A5210	Sexually transmitted infections (not HIV or hepatitis)
9	A5211	Sexually transmitted infections (not HIV or hepatitis)
9	A5212	Sexually transmitted infections (not HIV or hepatitis)
9	A5213	Sexually transmitted infections (not HIV or hepatitis)
9	A5214	Sexually transmitted infections (not HIV or hepatitis)
9	A5215	Sexually transmitted infections (not HIV or hepatitis)
9	A5216	Sexually transmitted infections (not HIV or hepatitis)
9	A5217	Sexually transmitted infections (not HIV or hepatitis)
9	A5219	Sexually transmitted infections (not HIV or hepatitis)
9	A522	Sexually transmitted infections (not HIV or hepatitis)
9	A523	Sexually transmitted infections (not HIV or hepatitis)
9	A5271	Sexually transmitted infections (not HIV or hepatitis)
9	A5272	Sexually transmitted infections (not HIV or hepatitis)
9	A5273	Sexually transmitted infections (not HIV or hepatitis)
9	A5274	Sexually transmitted infections (not HIV or hepatitis)
9	A5275	Sexually transmitted infections (not HIV or hepatitis)
9	A5276	Sexually transmitted infections (not HIV or hepatitis)
9	A5277	Sexually transmitted infections (not HIV or hepatitis)
9	A5278	Sexually transmitted infections (not HIV or hepatitis)
9	A5279	Sexually transmitted infections (not HIV or hepatitis)
9	A528	Sexually transmitted infections (not HIV or hepatitis)

9	A529	Sexually transmitted infections (not HIV or hepatitis)
9	A530	Sexually transmitted infections (not HIV or hepatitis)
9	A539	Sexually transmitted infections (not HIV or hepatitis)
9	A5400	Sexually transmitted infections (not HIV or hepatitis)
9	A5401	Sexually transmitted infections (not HIV or hepatitis)
9	A5402	Sexually transmitted infections (not HIV or hepatitis)
9	A5403	Sexually transmitted infections (not HIV or hepatitis)
9	A5409	Sexually transmitted infections (not HIV or hepatitis)
9	A541	Sexually transmitted infections (not HIV or hepatitis)
9	A5421	Sexually transmitted infections (not HIV or hepatitis)
9	A5422	Sexually transmitted infections (not HIV or hepatitis)
9	A5423	Sexually transmitted infections (not HIV or hepatitis)
9	A5424	Sexually transmitted infections (not HIV or hepatitis)
9	A5429	Sexually transmitted infections (not HIV or hepatitis)
9	A5430	Sexually transmitted infections (not HIV or hepatitis)
9	A5431	Sexually transmitted infections (not HIV or hepatitis)
9	A5432	Sexually transmitted infections (not HIV or hepatitis)
9	A5433	Sexually transmitted infections (not HIV or hepatitis)
9	A5439	Sexually transmitted infections (not HIV or hepatitis)
9	A5440	Sexually transmitted infections (not HIV or hepatitis)
9	A5441	Sexually transmitted infections (not HIV or hepatitis)
9	A5442	Sexually transmitted infections (not HIV or hepatitis)
9	A5443	Sexually transmitted infections (not HIV or hepatitis)
9	A5449	Sexually transmitted infections (not HIV or hepatitis)
9	A545	Sexually transmitted infections (not HIV or hepatitis)
9	A546	Sexually transmitted infections (not HIV or hepatitis)
9	A5481	Sexually transmitted infections (not HIV or hepatitis)
9	A5482	Sexually transmitted infections (not HIV or hepatitis)
9	A5483	Sexually transmitted infections (not HIV or hepatitis)
9	A5484	Sexually transmitted infections (not HIV or hepatitis)
9	A5485	Sexually transmitted infections (not HIV or hepatitis)
9	A5489	Sexually transmitted infections (not HIV or hepatitis)
9	A549	Sexually transmitted infections (not HIV or hepatitis)
9	A55	Sexually transmitted infections (not HIV or hepatitis)
9	A5600	Sexually transmitted infections (not HIV or hepatitis)
9	A5601	Sexually transmitted infections (not HIV or hepatitis)
9	A5602	Sexually transmitted infections (not HIV or hepatitis)
9	A5609	Sexually transmitted infections (not HIV or hepatitis)
9	A5611	Sexually transmitted infections (not HIV or hepatitis)
9	A5619	Sexually transmitted infections (not HIV or hepatitis)
9	A562	Sexually transmitted infections (not HIV or hepatitis)
9	A563	Sexually transmitted infections (not HIV or hepatitis)
9	A564	Sexually transmitted infections (not HIV or hepatitis)
9	A568	Sexually transmitted infections (not HIV or hepatitis)
9	A57	Sexually transmitted infections (not HIV or hepatitis)
9	A58	Sexually transmitted infections (not HIV or hepatitis)
9	A638	Sexually transmitted infections (not HIV or hepatitis)
9	A64	Sexually transmitted infections (not HIV or hepatitis)
9	R8581	Sexually transmitted infections (not HIV or hepatitis)
9	R8582	Sexually transmitted infections (not HIV or hepatitis)
9	R87810	Sexually transmitted infections (not HIV or hepatitis)

9	R87811	Sexually transmitted infections (not HIV or hepatitis)
9	R87820	Sexually transmitted infections (not HIV or hepatitis)
9	R87821	Sexually transmitted infections (not HIV or hepatitis)
10	R760	Immunizations and screening for infectious disease
10	R7611	Immunizations and screening for infectious disease
10	R7612	Immunizations and screening for infectious disease
10	R768	Immunizations and screening for infectious disease
10	R769	Immunizations and screening for infectious disease
10	Z03810	Immunizations and screening for infectious disease
10	Z03818	Immunizations and screening for infectious disease
10	Z110	Immunizations and screening for infectious disease
10	Z111	Immunizations and screening for infectious disease
10	Z112	Immunizations and screening for infectious disease
10	Z113	Immunizations and screening for infectious disease
10	Z114	Immunizations and screening for infectious disease
10	Z1151	Immunizations and screening for infectious disease
10	Z1159	Immunizations and screening for infectious disease
10	Z116	Immunizations and screening for infectious disease
10	Z118	Immunizations and screening for infectious disease
10	Z119	Immunizations and screening for infectious disease
10	Z2001	Immunizations and screening for infectious disease
10	Z2009	Immunizations and screening for infectious disease
10	Z201	Immunizations and screening for infectious disease
10	Z202	Immunizations and screening for infectious disease
10	Z203	Immunizations and screening for infectious disease
10	Z204	Immunizations and screening for infectious disease
10	Z205	Immunizations and screening for infectious disease
10	Z206	Immunizations and screening for infectious disease
10	Z207	Immunizations and screening for infectious disease
10	Z20810	Immunizations and screening for infectious disease
10	Z20811	Immunizations and screening for infectious disease
10	Z20818	Immunizations and screening for infectious disease
10	Z20820	Immunizations and screening for infectious disease
10	Z20828	Immunizations and screening for infectious disease
10	Z2089	Immunizations and screening for infectious disease
10	Z209	Immunizations and screening for infectious disease
10	Z220	Immunizations and screening for infectious disease
10	Z221	Immunizations and screening for infectious disease
10	Z222	Immunizations and screening for infectious disease
10	Z2231	Immunizations and screening for infectious disease
10	Z22321	Immunizations and screening for infectious disease
10	Z22322	Immunizations and screening for infectious disease
10	Z22330	Immunizations and screening for infectious disease
10	Z22338	Immunizations and screening for infectious disease
10	Z2239	Immunizations and screening for infectious disease
10	Z224	Immunizations and screening for infectious disease
10	Z2250	Immunizations and screening for infectious disease
10	Z2251	Immunizations and screening for infectious disease
10	Z2252	Immunizations and screening for infectious disease
10	Z2259	Immunizations and screening for infectious disease
10	Z226	Immunizations and screening for infectious disease

10	Z228	Immunizations and screening for infectious disease
10	Z229	Immunizations and screening for infectious disease
10	Z23	Immunizations and screening for infectious disease
11	C000	Cancer of head and neck
11	C001	Cancer of head and neck
11	C002	Cancer of head and neck
11	C003	Cancer of head and neck
11	C004	Cancer of head and neck
11	C005	Cancer of head and neck
11	C006	Cancer of head and neck
11	C008	Cancer of head and neck
11	C009	Cancer of head and neck
11	C01	Cancer of head and neck
11	C020	Cancer of head and neck
11	C021	Cancer of head and neck
11	C022	Cancer of head and neck
11	C023	Cancer of head and neck
11	C024	Cancer of head and neck
11	C028	Cancer of head and neck
11	C029	Cancer of head and neck
11	C030	Cancer of head and neck
11	C031	Cancer of head and neck
11	C039	Cancer of head and neck
11	C040	Cancer of head and neck
11	C041	Cancer of head and neck
11	C048	Cancer of head and neck
11	C049	Cancer of head and neck
11	C050	Cancer of head and neck
11	C051	Cancer of head and neck
11	C052	Cancer of head and neck
11	C058	Cancer of head and neck
11	C059	Cancer of head and neck
11	C060	Cancer of head and neck
11	C061	Cancer of head and neck
11	C062	Cancer of head and neck
11	C0680	Cancer of head and neck
11	C0689	Cancer of head and neck
11	C069	Cancer of head and neck
11	C07	Cancer of head and neck
11	C080	Cancer of head and neck
11	C081	Cancer of head and neck
11	C089	Cancer of head and neck
11	C090	Cancer of head and neck
11	C091	Cancer of head and neck
11	C098	Cancer of head and neck
11	C099	Cancer of head and neck
11	C100	Cancer of head and neck
11	C101	Cancer of head and neck
11	C102	Cancer of head and neck
11	C103	Cancer of head and neck
11	C104	Cancer of head and neck

11	C108	Cancer of head and neck
11	C109	Cancer of head and neck
11	C110	Cancer of head and neck
11	C111	Cancer of head and neck
11	C112	Cancer of head and neck
11	C113	Cancer of head and neck
11	C118	Cancer of head and neck
11	C119	Cancer of head and neck
11	C12	Cancer of head and neck
11	C130	Cancer of head and neck
11	C131	Cancer of head and neck
11	C132	Cancer of head and neck
11	C138	Cancer of head and neck
11	C139	Cancer of head and neck
11	C140	Cancer of head and neck
11	C142	Cancer of head and neck
11	C148	Cancer of head and neck
11	C300	Cancer of head and neck
11	C301	Cancer of head and neck
11	C310	Cancer of head and neck
11	C311	Cancer of head and neck
11	C312	Cancer of head and neck
11	C313	Cancer of head and neck
11	C318	Cancer of head and neck
11	C319	Cancer of head and neck
11	C320	Cancer of head and neck
11	C321	Cancer of head and neck
11	C322	Cancer of head and neck
11	C323	Cancer of head and neck
11	C328	Cancer of head and neck
11	C329	Cancer of head and neck
11	C760	Cancer of head and neck
11	D0000	Cancer of head and neck
11	D0001	Cancer of head and neck
11	D0002	Cancer of head and neck
11	D0003	Cancer of head and neck
11	D0004	Cancer of head and neck
11	D0005	Cancer of head and neck
11	D0006	Cancer of head and neck
11	D0007	Cancer of head and neck
11	D0008	Cancer of head and neck
11	D020	Cancer of head and neck
11	Z8521	Cancer of head and neck
11	Z85810	Cancer of head and neck
11	Z85818	Cancer of head and neck
11	Z85819	Cancer of head and neck
12	C153	Cancer of esophagus
12	C154	Cancer of esophagus
12	C155	Cancer of esophagus
12	C158	Cancer of esophagus
12	C159	Cancer of esophagus

12	D001	Cancer of esophagus
12	Z8501	Cancer of esophagus
13	C160	Cancer of stomach
13	C161	Cancer of stomach
13	C162	Cancer of stomach
13	C163	Cancer of stomach
13	C164	Cancer of stomach
13	C165	Cancer of stomach
13	C166	Cancer of stomach
13	C168	Cancer of stomach
13	C169	Cancer of stomach
13	C7A092	Cancer of stomach
13	D002	Cancer of stomach
13	Z85028	Cancer of stomach
14	C180	Cancer of colon
14	C181	Cancer of colon
14	C182	Cancer of colon
14	C183	Cancer of colon
14	C184	Cancer of colon
14	C185	Cancer of colon
14	C186	Cancer of colon
14	C187	Cancer of colon
14	C188	Cancer of colon
14	C189	Cancer of colon
14	C260	Cancer of colon
14	C7A020	Cancer of colon
14	C7A021	Cancer of colon
14	C7A022	Cancer of colon
14	C7A023	Cancer of colon
14	C7A024	Cancer of colon
14	C7A025	Cancer of colon
14	C7A029	Cancer of colon
14	D010	Cancer of colon
14	Z85038	Cancer of colon
15	C19	Cancer of rectum and anus
15	C20	Cancer of rectum and anus
15	C210	Cancer of rectum and anus
15	C211	Cancer of rectum and anus
15	C212	Cancer of rectum and anus
15	C218	Cancer of rectum and anus
15	C7A026	Cancer of rectum and anus
15	D011	Cancer of rectum and anus
15	D012	Cancer of rectum and anus
15	D013	Cancer of rectum and anus
15	R85610	Cancer of rectum and anus
15	R85611	Cancer of rectum and anus
15	R85612	Cancer of rectum and anus
15	R85613	Cancer of rectum and anus
15	R85614	Cancer of rectum and anus
15	Z85048	Cancer of rectum and anus
16	C220	Cancer of liver and intrahepatic bile duct

16	C221	Cancer of liver and intrahepatic bile duct
16	C222	Cancer of liver and intrahepatic bile duct
16	C223	Cancer of liver and intrahepatic bile duct
16	C224	Cancer of liver and intrahepatic bile duct
16	C227	Cancer of liver and intrahepatic bile duct
16	C228	Cancer of liver and intrahepatic bile duct
16	C229	Cancer of liver and intrahepatic bile duct
16	D015	Cancer of liver and intrahepatic bile duct
16	Z8505	Cancer of liver and intrahepatic bile duct
17	C250	Cancer of pancreas
17	C251	Cancer of pancreas
17	C252	Cancer of pancreas
17	C253	Cancer of pancreas
17	C254	Cancer of pancreas
17	C257	Cancer of pancreas
17	C258	Cancer of pancreas
17	C259	Cancer of pancreas
18	C170	Cancer of other GI organs; peritoneum
18	C171	Cancer of other GI organs; peritoneum
18	C172	Cancer of other GI organs; peritoneum
18	C173	Cancer of other GI organs; peritoneum
18	C178	Cancer of other GI organs; peritoneum
18	C179	Cancer of other GI organs; peritoneum
18	C23	Cancer of other GI organs; peritoneum
18	C240	Cancer of other GI organs; peritoneum
18	C241	Cancer of other GI organs; peritoneum
18	C248	Cancer of other GI organs; peritoneum
18	C249	Cancer of other GI organs; peritoneum
18	C261	Cancer of other GI organs; peritoneum
18	C269	Cancer of other GI organs; peritoneum
18	C451	Cancer of other GI organs; peritoneum
18	C480	Cancer of other GI organs; peritoneum
18	C481	Cancer of other GI organs; peritoneum
18	C482	Cancer of other GI organs; peritoneum
18	C488	Cancer of other GI organs; peritoneum
18	C7A010	Cancer of other GI organs; peritoneum
18	C7A011	Cancer of other GI organs; peritoneum
18	C7A012	Cancer of other GI organs; peritoneum
18	C7A019	Cancer of other GI organs; peritoneum
18	D0140	Cancer of other GI organs; peritoneum
18	D0149	Cancer of other GI organs; peritoneum
18	D017	Cancer of other GI organs; peritoneum
18	D019	Cancer of other GI organs; peritoneum
18	Z8500	Cancer of other GI organs; peritoneum
18	Z85068	Cancer of other GI organs; peritoneum
18	Z8507	Cancer of other GI organs; peritoneum
18	Z8509	Cancer of other GI organs; peritoneum
19	C3400	Cancer of bronchus; lung
19	C3401	Cancer of bronchus; lung
19	C3402	Cancer of bronchus; lung
19	C3410	Cancer of bronchus; lung

19	C3411	Cancer of bronchus; lung
19	C3412	Cancer of bronchus; lung
19	C342	Cancer of bronchus; lung
19	C3430	Cancer of bronchus; lung
19	C3431	Cancer of bronchus; lung
19	C3432	Cancer of bronchus; lung
19	C3480	Cancer of bronchus; lung
19	C3481	Cancer of bronchus; lung
19	C3482	Cancer of bronchus; lung
19	C3490	Cancer of bronchus; lung
19	C3491	Cancer of bronchus; lung
19	C3492	Cancer of bronchus; lung
19	C7A090	Cancer of bronchus; lung
19	D0220	Cancer of bronchus; lung
19	D0221	Cancer of bronchus; lung
19	D0222	Cancer of bronchus; lung
19	Z85118	Cancer of bronchus; lung
20	C33	Cancer; other respiratory and intrathoracic
20	C384	Cancer; other respiratory and intrathoracic
20	C390	Cancer; other respiratory and intrathoracic
20	C399	Cancer; other respiratory and intrathoracic
20	C450	Cancer; other respiratory and intrathoracic
20	D021	Cancer; other respiratory and intrathoracic
20	D023	Cancer; other respiratory and intrathoracic
20	D024	Cancer; other respiratory and intrathoracic
20	Z8512	Cancer; other respiratory and intrathoracic
20	Z8520	Cancer; other respiratory and intrathoracic
20	Z8522	Cancer; other respiratory and intrathoracic
21	C4000	Cancer of bone and connective tissue
21	C4001	Cancer of bone and connective tissue
21	C4002	Cancer of bone and connective tissue
21	C4010	Cancer of bone and connective tissue
21	C4011	Cancer of bone and connective tissue
21	C4012	Cancer of bone and connective tissue
21	C4020	Cancer of bone and connective tissue
21	C4021	Cancer of bone and connective tissue
21	C4022	Cancer of bone and connective tissue
21	C4030	Cancer of bone and connective tissue
21	C4031	Cancer of bone and connective tissue
21	C4032	Cancer of bone and connective tissue
21	C4080	Cancer of bone and connective tissue
21	C4081	Cancer of bone and connective tissue
21	C4082	Cancer of bone and connective tissue
21	C4090	Cancer of bone and connective tissue
21	C4091	Cancer of bone and connective tissue
21	C4092	Cancer of bone and connective tissue
21	C410	Cancer of bone and connective tissue
21	C411	Cancer of bone and connective tissue
21	C412	Cancer of bone and connective tissue
21	C413	Cancer of bone and connective tissue
21	C414	Cancer of bone and connective tissue

21	C419	Cancer of bone and connective tissue
21	C490	Cancer of bone and connective tissue
21	C4910	Cancer of bone and connective tissue
21	C4911	Cancer of bone and connective tissue
21	C4912	Cancer of bone and connective tissue
21	C4920	Cancer of bone and connective tissue
21	C4921	Cancer of bone and connective tissue
21	C4922	Cancer of bone and connective tissue
21	C493	Cancer of bone and connective tissue
21	C494	Cancer of bone and connective tissue
21	C495	Cancer of bone and connective tissue
21	C496	Cancer of bone and connective tissue
21	C498	Cancer of bone and connective tissue
21	C499	Cancer of bone and connective tissue
22	C430	Melanomas of skin
22	C4310	Melanomas of skin
22	C4311	Melanomas of skin
22	C4312	Melanomas of skin
22	C4320	Melanomas of skin
22	C4321	Melanomas of skin
22	C4322	Melanomas of skin
22	C4330	Melanomas of skin
22	C4331	Melanomas of skin
22	C4339	Melanomas of skin
22	C434	Melanomas of skin
22	C4351	Melanomas of skin
22	C4352	Melanomas of skin
22	C4359	Melanomas of skin
22	C4360	Melanomas of skin
22	C4361	Melanomas of skin
22	C4362	Melanomas of skin
22	C4370	Melanomas of skin
22	C4371	Melanomas of skin
22	C4372	Melanomas of skin
22	C438	Melanomas of skin
22	C439	Melanomas of skin
22	D030	Melanomas of skin
22	D0310	Melanomas of skin
22	D0311	Melanomas of skin
22	D0312	Melanomas of skin
22	D0320	Melanomas of skin
22	D0321	Melanomas of skin
22	D0322	Melanomas of skin
22	D0330	Melanomas of skin
22	D0339	Melanomas of skin
22	D034	Melanomas of skin
22	D0351	Melanomas of skin
22	D0352	Melanomas of skin
22	D0359	Melanomas of skin
22	D0360	Melanomas of skin
22	D0361	Melanomas of skin

22	D0362	Melanomas of skin
22	D0370	Melanomas of skin
22	D0371	Melanomas of skin
22	D0372	Melanomas of skin
22	D038	Melanomas of skin
22	D039	Melanomas of skin
22	Z85820	Melanomas of skin
23	C4400	Other non-epithelial cancer of skin
23	C4401	Other non-epithelial cancer of skin
23	C4402	Other non-epithelial cancer of skin
23	C4409	Other non-epithelial cancer of skin
23	C44101	Other non-epithelial cancer of skin
23	C44102	Other non-epithelial cancer of skin
23	C44109	Other non-epithelial cancer of skin
23	C44111	Other non-epithelial cancer of skin
23	C44112	Other non-epithelial cancer of skin
23	C44119	Other non-epithelial cancer of skin
23	C44121	Other non-epithelial cancer of skin
23	C44122	Other non-epithelial cancer of skin
23	C44129	Other non-epithelial cancer of skin
23	C44191	Other non-epithelial cancer of skin
23	C44192	Other non-epithelial cancer of skin
23	C44199	Other non-epithelial cancer of skin
23	C44201	Other non-epithelial cancer of skin
23	C44202	Other non-epithelial cancer of skin
23	C44209	Other non-epithelial cancer of skin
23	C44211	Other non-epithelial cancer of skin
23	C44212	Other non-epithelial cancer of skin
23	C44219	Other non-epithelial cancer of skin
23	C44221	Other non-epithelial cancer of skin
23	C44222	Other non-epithelial cancer of skin
23	C44229	Other non-epithelial cancer of skin
23	C44291	Other non-epithelial cancer of skin
23	C44292	Other non-epithelial cancer of skin
23	C44299	Other non-epithelial cancer of skin
23	C44300	Other non-epithelial cancer of skin
23	C44301	Other non-epithelial cancer of skin
23	C44309	Other non-epithelial cancer of skin
23	C44310	Other non-epithelial cancer of skin
23	C44311	Other non-epithelial cancer of skin
23	C44319	Other non-epithelial cancer of skin
23	C44320	Other non-epithelial cancer of skin
23	C44321	Other non-epithelial cancer of skin
23	C44329	Other non-epithelial cancer of skin
23	C44390	Other non-epithelial cancer of skin
23	C44391	Other non-epithelial cancer of skin
23	C44399	Other non-epithelial cancer of skin
23	C4440	Other non-epithelial cancer of skin
23	C4441	Other non-epithelial cancer of skin
23	C4442	Other non-epithelial cancer of skin
23	C4449	Other non-epithelial cancer of skin

23	C44500	Other non-epithelial cancer of skin
23	C44501	Other non-epithelial cancer of skin
23	C44509	Other non-epithelial cancer of skin
23	C44510	Other non-epithelial cancer of skin
23	C44511	Other non-epithelial cancer of skin
23	C44519	Other non-epithelial cancer of skin
23	C44520	Other non-epithelial cancer of skin
23	C44521	Other non-epithelial cancer of skin
23	C44529	Other non-epithelial cancer of skin
23	C44590	Other non-epithelial cancer of skin
23	C44591	Other non-epithelial cancer of skin
23	C44599	Other non-epithelial cancer of skin
23	C44601	Other non-epithelial cancer of skin
23	C44602	Other non-epithelial cancer of skin
23	C44609	Other non-epithelial cancer of skin
23	C44611	Other non-epithelial cancer of skin
23	C44612	Other non-epithelial cancer of skin
23	C44619	Other non-epithelial cancer of skin
23	C44621	Other non-epithelial cancer of skin
23	C44622	Other non-epithelial cancer of skin
23	C44629	Other non-epithelial cancer of skin
23	C44691	Other non-epithelial cancer of skin
23	C44692	Other non-epithelial cancer of skin
23	C44699	Other non-epithelial cancer of skin
23	C44701	Other non-epithelial cancer of skin
23	C44702	Other non-epithelial cancer of skin
23	C44709	Other non-epithelial cancer of skin
23	C44711	Other non-epithelial cancer of skin
23	C44712	Other non-epithelial cancer of skin
23	C44719	Other non-epithelial cancer of skin
23	C44721	Other non-epithelial cancer of skin
23	C44722	Other non-epithelial cancer of skin
23	C44729	Other non-epithelial cancer of skin
23	C44791	Other non-epithelial cancer of skin
23	C44792	Other non-epithelial cancer of skin
23	C44799	Other non-epithelial cancer of skin
23	C4480	Other non-epithelial cancer of skin
23	C4481	Other non-epithelial cancer of skin
23	C4482	Other non-epithelial cancer of skin
23	C4489	Other non-epithelial cancer of skin
23	C4490	Other non-epithelial cancer of skin
23	C4491	Other non-epithelial cancer of skin
23	C4492	Other non-epithelial cancer of skin
23	C4499	Other non-epithelial cancer of skin
23	C4A0	Other non-epithelial cancer of skin
23	C4A10	Other non-epithelial cancer of skin
23	C4A11	Other non-epithelial cancer of skin
23	C4A12	Other non-epithelial cancer of skin
23	C4A20	Other non-epithelial cancer of skin
23	C4A21	Other non-epithelial cancer of skin
23	C4A22	Other non-epithelial cancer of skin

23	C4A30	Other non-epithelial cancer of skin
23	C4A31	Other non-epithelial cancer of skin
23	C4A39	Other non-epithelial cancer of skin
23	C4A4	Other non-epithelial cancer of skin
23	C4A51	Other non-epithelial cancer of skin
23	C4A52	Other non-epithelial cancer of skin
23	C4A59	Other non-epithelial cancer of skin
23	C4A60	Other non-epithelial cancer of skin
23	C4A61	Other non-epithelial cancer of skin
23	C4A62	Other non-epithelial cancer of skin
23	C4A70	Other non-epithelial cancer of skin
23	C4A71	Other non-epithelial cancer of skin
23	C4A72	Other non-epithelial cancer of skin
23	C4A8	Other non-epithelial cancer of skin
23	C4A9	Other non-epithelial cancer of skin
23	D040	Other non-epithelial cancer of skin
23	D0410	Other non-epithelial cancer of skin
23	D0411	Other non-epithelial cancer of skin
23	D0412	Other non-epithelial cancer of skin
23	D0420	Other non-epithelial cancer of skin
23	D0421	Other non-epithelial cancer of skin
23	D0422	Other non-epithelial cancer of skin
23	D0430	Other non-epithelial cancer of skin
23	D0439	Other non-epithelial cancer of skin
23	D044	Other non-epithelial cancer of skin
23	D045	Other non-epithelial cancer of skin
23	D0460	Other non-epithelial cancer of skin
23	D0461	Other non-epithelial cancer of skin
23	D0462	Other non-epithelial cancer of skin
23	D0470	Other non-epithelial cancer of skin
23	D0471	Other non-epithelial cancer of skin
23	D0472	Other non-epithelial cancer of skin
23	D048	Other non-epithelial cancer of skin
23	D049	Other non-epithelial cancer of skin
23	Z85828	Other non-epithelial cancer of skin
24	C50011	Cancer of breast
24	C50012	Cancer of breast
24	C50019	Cancer of breast
24	C50021	Cancer of breast
24	C50022	Cancer of breast
24	C50029	Cancer of breast
24	C50111	Cancer of breast
24	C50112	Cancer of breast
24	C50119	Cancer of breast
24	C50121	Cancer of breast
24	C50122	Cancer of breast
24	C50129	Cancer of breast
24	C50211	Cancer of breast
24	C50212	Cancer of breast
24	C50219	Cancer of breast
24	C50221	Cancer of breast

24	C50222	Cancer of breast
24	C50229	Cancer of breast
24	C50311	Cancer of breast
24	C50312	Cancer of breast
24	C50319	Cancer of breast
24	C50321	Cancer of breast
24	C50322	Cancer of breast
24	C50329	Cancer of breast
24	C50411	Cancer of breast
24	C50412	Cancer of breast
24	C50419	Cancer of breast
24	C50421	Cancer of breast
24	C50422	Cancer of breast
24	C50429	Cancer of breast
24	C50511	Cancer of breast
24	C50512	Cancer of breast
24	C50519	Cancer of breast
24	C50521	Cancer of breast
24	C50522	Cancer of breast
24	C50529	Cancer of breast
24	C50611	Cancer of breast
24	C50612	Cancer of breast
24	C50619	Cancer of breast
24	C50621	Cancer of breast
24	C50622	Cancer of breast
24	C50629	Cancer of breast
24	C50811	Cancer of breast
24	C50812	Cancer of breast
24	C50819	Cancer of breast
24	C50821	Cancer of breast
24	C50822	Cancer of breast
24	C50829	Cancer of breast
24	C50911	Cancer of breast
24	C50912	Cancer of breast
24	C50919	Cancer of breast
24	C50921	Cancer of breast
24	C50922	Cancer of breast
24	C50929	Cancer of breast
24	D0500	Cancer of breast
24	D0501	Cancer of breast
24	D0502	Cancer of breast
24	D0510	Cancer of breast
24	D0511	Cancer of breast
24	D0512	Cancer of breast
24	D0580	Cancer of breast
24	D0581	Cancer of breast
24	D0582	Cancer of breast
24	D0590	Cancer of breast
24	D0591	Cancer of breast
24	D0592	Cancer of breast
24	Z853	Cancer of breast

24	Z86000	Cancer of breast
25	C540	Cancer of uterus
25	C541	Cancer of uterus
25	C542	Cancer of uterus
25	C543	Cancer of uterus
25	C548	Cancer of uterus
25	C549	Cancer of uterus
25	C55	Cancer of uterus
25	D070	Cancer of uterus
25	Z8542	Cancer of uterus
26	C530	Cancer of cervix
26	C531	Cancer of cervix
26	C538	Cancer of cervix
26	C539	Cancer of cervix
26	D060	Cancer of cervix
26	D061	Cancer of cervix
26	D067	Cancer of cervix
26	D069	Cancer of cervix
26	R87610	Cancer of cervix
26	R87611	Cancer of cervix
26	R87612	Cancer of cervix
26	R87613	Cancer of cervix
26	R87614	Cancer of cervix
26	Z8541	Cancer of cervix
26	Z86001	Cancer of cervix
27	C561	Cancer of ovary
27	C562	Cancer of ovary
27	C569	Cancer of ovary
27	Z8543	Cancer of ovary
28	C510	Cancer of other female genital organs
28	C511	Cancer of other female genital organs
28	C512	Cancer of other female genital organs
28	C518	Cancer of other female genital organs
28	C519	Cancer of other female genital organs
28	C52	Cancer of other female genital organs
28	C5700	Cancer of other female genital organs
28	C5701	Cancer of other female genital organs
28	C5702	Cancer of other female genital organs
28	C5710	Cancer of other female genital organs
28	C5711	Cancer of other female genital organs
28	C5712	Cancer of other female genital organs
28	C5720	Cancer of other female genital organs
28	C5721	Cancer of other female genital organs
28	C5722	Cancer of other female genital organs
28	C573	Cancer of other female genital organs
28	C574	Cancer of other female genital organs
28	C577	Cancer of other female genital organs
28	C578	Cancer of other female genital organs
28	C579	Cancer of other female genital organs
28	C58	Cancer of other female genital organs
28	D071	Cancer of other female genital organs

28	D072	Cancer of other female genital organs
28	D0730	Cancer of other female genital organs
28	D0739	Cancer of other female genital organs
28	R87620	Cancer of other female genital organs
28	R87621	Cancer of other female genital organs
28	R87622	Cancer of other female genital organs
28	R87623	Cancer of other female genital organs
28	R87624	Cancer of other female genital organs
28	Z8540	Cancer of other female genital organs
28	Z8544	Cancer of other female genital organs
29	C61	Cancer of prostate
29	D075	Cancer of prostate
29	Z8546	Cancer of prostate
30	C6200	Cancer of testis
30	C6201	Cancer of testis
30	C6202	Cancer of testis
30	C6210	Cancer of testis
30	C6211	Cancer of testis
30	C6212	Cancer of testis
30	C6290	Cancer of testis
30	C6291	Cancer of testis
30	C6292	Cancer of testis
30	Z8547	Cancer of testis
31	C600	Cancer of other male genital organs
31	C601	Cancer of other male genital organs
31	C602	Cancer of other male genital organs
31	C608	Cancer of other male genital organs
31	C609	Cancer of other male genital organs
31	C6300	Cancer of other male genital organs
31	C6301	Cancer of other male genital organs
31	C6302	Cancer of other male genital organs
31	C6310	Cancer of other male genital organs
31	C6311	Cancer of other male genital organs
31	C6312	Cancer of other male genital organs
31	C632	Cancer of other male genital organs
31	C637	Cancer of other male genital organs
31	C638	Cancer of other male genital organs
31	C639	Cancer of other male genital organs
31	D074	Cancer of other male genital organs
31	D0760	Cancer of other male genital organs
31	D0761	Cancer of other male genital organs
31	D0769	Cancer of other male genital organs
31	Z8545	Cancer of other male genital organs
31	Z8548	Cancer of other male genital organs
31	Z8549	Cancer of other male genital organs
32	C670	Cancer of bladder
32	C671	Cancer of bladder
32	C672	Cancer of bladder
32	C673	Cancer of bladder
32	C674	Cancer of bladder
32	C675	Cancer of bladder

32	C676	Cancer of bladder
32	C677	Cancer of bladder
32	C678	Cancer of bladder
32	C679	Cancer of bladder
32	D090	Cancer of bladder
32	Z8551	Cancer of bladder
33	C641	Cancer of kidney and renal pelvis
33	C642	Cancer of kidney and renal pelvis
33	C649	Cancer of kidney and renal pelvis
33	C651	Cancer of kidney and renal pelvis
33	C652	Cancer of kidney and renal pelvis
33	C659	Cancer of kidney and renal pelvis
33	C7A093	Cancer of kidney and renal pelvis
33	Z85528	Cancer of kidney and renal pelvis
33	Z8553	Cancer of kidney and renal pelvis
34	C661	Cancer of other urinary organs
34	C662	Cancer of other urinary organs
34	C669	Cancer of other urinary organs
34	C680	Cancer of other urinary organs
34	C681	Cancer of other urinary organs
34	C688	Cancer of other urinary organs
34	C689	Cancer of other urinary organs
34	D0910	Cancer of other urinary organs
34	D0919	Cancer of other urinary organs
34	Z8550	Cancer of other urinary organs
34	Z8554	Cancer of other urinary organs
34	Z8559	Cancer of other urinary organs
35	C470	Cancer of brain and nervous system
35	C4710	Cancer of brain and nervous system
35	C4711	Cancer of brain and nervous system
35	C4712	Cancer of brain and nervous system
35	C4720	Cancer of brain and nervous system
35	C4721	Cancer of brain and nervous system
35	C4722	Cancer of brain and nervous system
35	C473	Cancer of brain and nervous system
35	C474	Cancer of brain and nervous system
35	C475	Cancer of brain and nervous system
35	C476	Cancer of brain and nervous system
35	C478	Cancer of brain and nervous system
35	C479	Cancer of brain and nervous system
35	C700	Cancer of brain and nervous system
35	C701	Cancer of brain and nervous system
35	C709	Cancer of brain and nervous system
35	C710	Cancer of brain and nervous system
35	C711	Cancer of brain and nervous system
35	C712	Cancer of brain and nervous system
35	C713	Cancer of brain and nervous system
35	C714	Cancer of brain and nervous system
35	C715	Cancer of brain and nervous system
35	C716	Cancer of brain and nervous system
35	C717	Cancer of brain and nervous system

35	C718	Cancer of brain and nervous system
35	C719	Cancer of brain and nervous system
35	C720	Cancer of brain and nervous system
35	C721	Cancer of brain and nervous system
35	C7220	Cancer of brain and nervous system
35	C7221	Cancer of brain and nervous system
35	C7222	Cancer of brain and nervous system
35	C7230	Cancer of brain and nervous system
35	C7231	Cancer of brain and nervous system
35	C7232	Cancer of brain and nervous system
35	C7240	Cancer of brain and nervous system
35	C7241	Cancer of brain and nervous system
35	C7242	Cancer of brain and nervous system
35	C7250	Cancer of brain and nervous system
35	C7259	Cancer of brain and nervous system
35	C729	Cancer of brain and nervous system
35	Z85841	Cancer of brain and nervous system
35	Z85848	Cancer of brain and nervous system
36	C73	Cancer of thyroid
36	D093	Cancer of thyroid
36	E3122	Cancer of thyroid
36	E3123	Cancer of thyroid
36	Z85850	Cancer of thyroid
37	C8100	Hodgkin`s disease
37	C8101	Hodgkin`s disease
37	C8102	Hodgkin`s disease
37	C8103	Hodgkin`s disease
37	C8104	Hodgkin`s disease
37	C8105	Hodgkin`s disease
37	C8106	Hodgkin`s disease
37	C8107	Hodgkin`s disease
37	C8108	Hodgkin`s disease
37	C8109	Hodgkin`s disease
37	C8110	Hodgkin`s disease
37	C8111	Hodgkin`s disease
37	C8112	Hodgkin`s disease
37	C8113	Hodgkin`s disease
37	C8114	Hodgkin`s disease
37	C8115	Hodgkin`s disease
37	C8116	Hodgkin`s disease
37	C8117	Hodgkin`s disease
37	C8118	Hodgkin`s disease
37	C8119	Hodgkin`s disease
37	C8120	Hodgkin`s disease
37	C8121	Hodgkin`s disease
37	C8122	Hodgkin`s disease
37	C8123	Hodgkin`s disease
37	C8124	Hodgkin`s disease
37	C8125	Hodgkin`s disease
37	C8126	Hodgkin`s disease
37	C8127	Hodgkin`s disease

37	C8128	Hodgkin`s disease
37	C8129	Hodgkin`s disease
37	C8130	Hodgkin`s disease
37	C8131	Hodgkin`s disease
37	C8132	Hodgkin`s disease
37	C8133	Hodgkin`s disease
37	C8134	Hodgkin`s disease
37	C8135	Hodgkin`s disease
37	C8136	Hodgkin`s disease
37	C8137	Hodgkin`s disease
37	C8138	Hodgkin`s disease
37	C8139	Hodgkin`s disease
37	C8140	Hodgkin`s disease
37	C8141	Hodgkin`s disease
37	C8142	Hodgkin`s disease
37	C8143	Hodgkin`s disease
37	C8144	Hodgkin`s disease
37	C8145	Hodgkin`s disease
37	C8146	Hodgkin`s disease
37	C8147	Hodgkin`s disease
37	C8148	Hodgkin`s disease
37	C8149	Hodgkin`s disease
37	C8170	Hodgkin`s disease
37	C8171	Hodgkin`s disease
37	C8172	Hodgkin`s disease
37	C8173	Hodgkin`s disease
37	C8174	Hodgkin`s disease
37	C8175	Hodgkin`s disease
37	C8176	Hodgkin`s disease
37	C8177	Hodgkin`s disease
37	C8178	Hodgkin`s disease
37	C8179	Hodgkin`s disease
37	C8190	Hodgkin`s disease
37	C8191	Hodgkin`s disease
37	C8192	Hodgkin`s disease
37	C8193	Hodgkin`s disease
37	C8194	Hodgkin`s disease
37	C8195	Hodgkin`s disease
37	C8196	Hodgkin`s disease
37	C8197	Hodgkin`s disease
37	C8198	Hodgkin`s disease
37	C8199	Hodgkin`s disease
37	Z8571	Hodgkin`s disease
38	C8200	Non-Hodgkin`s lymphoma
38	C8201	Non-Hodgkin`s lymphoma
38	C8202	Non-Hodgkin`s lymphoma
38	C8203	Non-Hodgkin`s lymphoma
38	C8204	Non-Hodgkin`s lymphoma
38	C8205	Non-Hodgkin`s lymphoma
38	C8206	Non-Hodgkin`s lymphoma
38	C8207	Non-Hodgkin`s lymphoma

38	C8208	Non-Hodgkin`s lymphoma
38	C8209	Non-Hodgkin`s lymphoma
38	C8210	Non-Hodgkin`s lymphoma
38	C8211	Non-Hodgkin`s lymphoma
38	C8212	Non-Hodgkin`s lymphoma
38	C8213	Non-Hodgkin`s lymphoma
38	C8214	Non-Hodgkin`s lymphoma
38	C8215	Non-Hodgkin`s lymphoma
38	C8216	Non-Hodgkin`s lymphoma
38	C8217	Non-Hodgkin`s lymphoma
38	C8218	Non-Hodgkin`s lymphoma
38	C8219	Non-Hodgkin`s lymphoma
38	C8220	Non-Hodgkin`s lymphoma
38	C8221	Non-Hodgkin`s lymphoma
38	C8222	Non-Hodgkin`s lymphoma
38	C8223	Non-Hodgkin`s lymphoma
38	C8224	Non-Hodgkin`s lymphoma
38	C8225	Non-Hodgkin`s lymphoma
38	C8226	Non-Hodgkin`s lymphoma
38	C8227	Non-Hodgkin`s lymphoma
38	C8228	Non-Hodgkin`s lymphoma
38	C8229	Non-Hodgkin`s lymphoma
38	C8230	Non-Hodgkin`s lymphoma
38	C8231	Non-Hodgkin`s lymphoma
38	C8232	Non-Hodgkin`s lymphoma
38	C8233	Non-Hodgkin`s lymphoma
38	C8234	Non-Hodgkin`s lymphoma
38	C8235	Non-Hodgkin`s lymphoma
38	C8236	Non-Hodgkin`s lymphoma
38	C8237	Non-Hodgkin`s lymphoma
38	C8238	Non-Hodgkin`s lymphoma
38	C8239	Non-Hodgkin`s lymphoma
38	C8240	Non-Hodgkin`s lymphoma
38	C8241	Non-Hodgkin`s lymphoma
38	C8242	Non-Hodgkin`s lymphoma
38	C8243	Non-Hodgkin`s lymphoma
38	C8244	Non-Hodgkin`s lymphoma
38	C8245	Non-Hodgkin`s lymphoma
38	C8246	Non-Hodgkin`s lymphoma
38	C8247	Non-Hodgkin`s lymphoma
38	C8248	Non-Hodgkin`s lymphoma
38	C8249	Non-Hodgkin`s lymphoma
38	C8250	Non-Hodgkin`s lymphoma
38	C8251	Non-Hodgkin`s lymphoma
38	C8252	Non-Hodgkin`s lymphoma
38	C8253	Non-Hodgkin`s lymphoma
38	C8254	Non-Hodgkin`s lymphoma
38	C8255	Non-Hodgkin`s lymphoma
38	C8256	Non-Hodgkin`s lymphoma
38	C8257	Non-Hodgkin`s lymphoma
38	C8258	Non-Hodgkin`s lymphoma

38	C8259	Non-Hodgkin`s lymphoma
38	C8260	Non-Hodgkin`s lymphoma
38	C8261	Non-Hodgkin`s lymphoma
38	C8262	Non-Hodgkin`s lymphoma
38	C8263	Non-Hodgkin`s lymphoma
38	C8264	Non-Hodgkin`s lymphoma
38	C8265	Non-Hodgkin`s lymphoma
38	C8266	Non-Hodgkin`s lymphoma
38	C8267	Non-Hodgkin`s lymphoma
38	C8268	Non-Hodgkin`s lymphoma
38	C8269	Non-Hodgkin`s lymphoma
38	C8280	Non-Hodgkin`s lymphoma
38	C8281	Non-Hodgkin`s lymphoma
38	C8282	Non-Hodgkin`s lymphoma
38	C8283	Non-Hodgkin`s lymphoma
38	C8284	Non-Hodgkin`s lymphoma
38	C8285	Non-Hodgkin`s lymphoma
38	C8286	Non-Hodgkin`s lymphoma
38	C8287	Non-Hodgkin`s lymphoma
38	C8288	Non-Hodgkin`s lymphoma
38	C8289	Non-Hodgkin`s lymphoma
38	C8290	Non-Hodgkin`s lymphoma
38	C8291	Non-Hodgkin`s lymphoma
38	C8292	Non-Hodgkin`s lymphoma
38	C8293	Non-Hodgkin`s lymphoma
38	C8294	Non-Hodgkin`s lymphoma
38	C8295	Non-Hodgkin`s lymphoma
38	C8296	Non-Hodgkin`s lymphoma
38	C8297	Non-Hodgkin`s lymphoma
38	C8298	Non-Hodgkin`s lymphoma
38	C8299	Non-Hodgkin`s lymphoma
38	C8300	Non-Hodgkin`s lymphoma
38	C8301	Non-Hodgkin`s lymphoma
38	C8302	Non-Hodgkin`s lymphoma
38	C8303	Non-Hodgkin`s lymphoma
38	C8304	Non-Hodgkin`s lymphoma
38	C8305	Non-Hodgkin`s lymphoma
38	C8306	Non-Hodgkin`s lymphoma
38	C8307	Non-Hodgkin`s lymphoma
38	C8308	Non-Hodgkin`s lymphoma
38	C8309	Non-Hodgkin`s lymphoma
38	C8310	Non-Hodgkin`s lymphoma
38	C8311	Non-Hodgkin`s lymphoma
38	C8312	Non-Hodgkin`s lymphoma
38	C8313	Non-Hodgkin`s lymphoma
38	C8314	Non-Hodgkin`s lymphoma
38	C8315	Non-Hodgkin`s lymphoma
38	C8316	Non-Hodgkin`s lymphoma
38	C8317	Non-Hodgkin`s lymphoma
38	C8318	Non-Hodgkin`s lymphoma
38	C8319	Non-Hodgkin`s lymphoma

38	C8330	Non-Hodgkin`s lymphoma
38	C8331	Non-Hodgkin`s lymphoma
38	C8332	Non-Hodgkin`s lymphoma
38	C8333	Non-Hodgkin`s lymphoma
38	C8334	Non-Hodgkin`s lymphoma
38	C8335	Non-Hodgkin`s lymphoma
38	C8336	Non-Hodgkin`s lymphoma
38	C8337	Non-Hodgkin`s lymphoma
38	C8338	Non-Hodgkin`s lymphoma
38	C8339	Non-Hodgkin`s lymphoma
38	C8350	Non-Hodgkin`s lymphoma
38	C8351	Non-Hodgkin`s lymphoma
38	C8352	Non-Hodgkin`s lymphoma
38	C8353	Non-Hodgkin`s lymphoma
38	C8354	Non-Hodgkin`s lymphoma
38	C8355	Non-Hodgkin`s lymphoma
38	C8356	Non-Hodgkin`s lymphoma
38	C8357	Non-Hodgkin`s lymphoma
38	C8358	Non-Hodgkin`s lymphoma
38	C8359	Non-Hodgkin`s lymphoma
38	C8370	Non-Hodgkin`s lymphoma
38	C8371	Non-Hodgkin`s lymphoma
38	C8372	Non-Hodgkin`s lymphoma
38	C8373	Non-Hodgkin`s lymphoma
38	C8374	Non-Hodgkin`s lymphoma
38	C8375	Non-Hodgkin`s lymphoma
38	C8376	Non-Hodgkin`s lymphoma
38	C8377	Non-Hodgkin`s lymphoma
38	C8378	Non-Hodgkin`s lymphoma
38	C8379	Non-Hodgkin`s lymphoma
38	C8380	Non-Hodgkin`s lymphoma
38	C8381	Non-Hodgkin`s lymphoma
38	C8382	Non-Hodgkin`s lymphoma
38	C8383	Non-Hodgkin`s lymphoma
38	C8384	Non-Hodgkin`s lymphoma
38	C8385	Non-Hodgkin`s lymphoma
38	C8386	Non-Hodgkin`s lymphoma
38	C8387	Non-Hodgkin`s lymphoma
38	C8388	Non-Hodgkin`s lymphoma
38	C8389	Non-Hodgkin`s lymphoma
38	C8390	Non-Hodgkin`s lymphoma
38	C8391	Non-Hodgkin`s lymphoma
38	C8392	Non-Hodgkin`s lymphoma
38	C8393	Non-Hodgkin`s lymphoma
38	C8394	Non-Hodgkin`s lymphoma
38	C8395	Non-Hodgkin`s lymphoma
38	C8396	Non-Hodgkin`s lymphoma
38	C8397	Non-Hodgkin`s lymphoma
38	C8398	Non-Hodgkin`s lymphoma
38	C8399	Non-Hodgkin`s lymphoma
38	C8400	Non-Hodgkin`s lymphoma

38	C8401	Non-Hodgkin`s lymphoma
38	C8402	Non-Hodgkin`s lymphoma
38	C8403	Non-Hodgkin`s lymphoma
38	C8404	Non-Hodgkin`s lymphoma
38	C8405	Non-Hodgkin`s lymphoma
38	C8406	Non-Hodgkin`s lymphoma
38	C8407	Non-Hodgkin`s lymphoma
38	C8408	Non-Hodgkin`s lymphoma
38	C8409	Non-Hodgkin`s lymphoma
38	C8410	Non-Hodgkin`s lymphoma
38	C8411	Non-Hodgkin`s lymphoma
38	C8412	Non-Hodgkin`s lymphoma
38	C8413	Non-Hodgkin`s lymphoma
38	C8414	Non-Hodgkin`s lymphoma
38	C8415	Non-Hodgkin`s lymphoma
38	C8416	Non-Hodgkin`s lymphoma
38	C8417	Non-Hodgkin`s lymphoma
38	C8418	Non-Hodgkin`s lymphoma
38	C8419	Non-Hodgkin`s lymphoma
38	C8440	Non-Hodgkin`s lymphoma
38	C8441	Non-Hodgkin`s lymphoma
38	C8442	Non-Hodgkin`s lymphoma
38	C8443	Non-Hodgkin`s lymphoma
38	C8444	Non-Hodgkin`s lymphoma
38	C8445	Non-Hodgkin`s lymphoma
38	C8446	Non-Hodgkin`s lymphoma
38	C8447	Non-Hodgkin`s lymphoma
38	C8448	Non-Hodgkin`s lymphoma
38	C8449	Non-Hodgkin`s lymphoma
38	C8460	Non-Hodgkin`s lymphoma
38	C8461	Non-Hodgkin`s lymphoma
38	C8462	Non-Hodgkin`s lymphoma
38	C8463	Non-Hodgkin`s lymphoma
38	C8464	Non-Hodgkin`s lymphoma
38	C8465	Non-Hodgkin`s lymphoma
38	C8466	Non-Hodgkin`s lymphoma
38	C8467	Non-Hodgkin`s lymphoma
38	C8468	Non-Hodgkin`s lymphoma
38	C8469	Non-Hodgkin`s lymphoma
38	C8470	Non-Hodgkin`s lymphoma
38	C8471	Non-Hodgkin`s lymphoma
38	C8472	Non-Hodgkin`s lymphoma
38	C8473	Non-Hodgkin`s lymphoma
38	C8474	Non-Hodgkin`s lymphoma
38	C8475	Non-Hodgkin`s lymphoma
38	C8476	Non-Hodgkin`s lymphoma
38	C8477	Non-Hodgkin`s lymphoma
38	C8478	Non-Hodgkin`s lymphoma
38	C8479	Non-Hodgkin`s lymphoma
38	C8490	Non-Hodgkin`s lymphoma
38	C8491	Non-Hodgkin`s lymphoma

38	C8492	Non-Hodgkin`s lymphoma
38	C8493	Non-Hodgkin`s lymphoma
38	C8494	Non-Hodgkin`s lymphoma
38	C8495	Non-Hodgkin`s lymphoma
38	C8496	Non-Hodgkin`s lymphoma
38	C8497	Non-Hodgkin`s lymphoma
38	C8498	Non-Hodgkin`s lymphoma
38	C8499	Non-Hodgkin`s lymphoma
38	C84A0	Non-Hodgkin`s lymphoma
38	C84A1	Non-Hodgkin`s lymphoma
38	C84A2	Non-Hodgkin`s lymphoma
38	C84A3	Non-Hodgkin`s lymphoma
38	C84A4	Non-Hodgkin`s lymphoma
38	C84A5	Non-Hodgkin`s lymphoma
38	C84A6	Non-Hodgkin`s lymphoma
38	C84A7	Non-Hodgkin`s lymphoma
38	C84A8	Non-Hodgkin`s lymphoma
38	C84A9	Non-Hodgkin`s lymphoma
38	C84Z0	Non-Hodgkin`s lymphoma
38	C84Z1	Non-Hodgkin`s lymphoma
38	C84Z2	Non-Hodgkin`s lymphoma
38	C84Z3	Non-Hodgkin`s lymphoma
38	C84Z4	Non-Hodgkin`s lymphoma
38	C84Z5	Non-Hodgkin`s lymphoma
38	C84Z6	Non-Hodgkin`s lymphoma
38	C84Z7	Non-Hodgkin`s lymphoma
38	C84Z8	Non-Hodgkin`s lymphoma
38	C84Z9	Non-Hodgkin`s lymphoma
38	C8510	Non-Hodgkin`s lymphoma
38	C8511	Non-Hodgkin`s lymphoma
38	C8512	Non-Hodgkin`s lymphoma
38	C8513	Non-Hodgkin`s lymphoma
38	C8514	Non-Hodgkin`s lymphoma
38	C8515	Non-Hodgkin`s lymphoma
38	C8516	Non-Hodgkin`s lymphoma
38	C8517	Non-Hodgkin`s lymphoma
38	C8518	Non-Hodgkin`s lymphoma
38	C8519	Non-Hodgkin`s lymphoma
38	C8520	Non-Hodgkin`s lymphoma
38	C8521	Non-Hodgkin`s lymphoma
38	C8522	Non-Hodgkin`s lymphoma
38	C8523	Non-Hodgkin`s lymphoma
38	C8524	Non-Hodgkin`s lymphoma
38	C8525	Non-Hodgkin`s lymphoma
38	C8526	Non-Hodgkin`s lymphoma
38	C8527	Non-Hodgkin`s lymphoma
38	C8528	Non-Hodgkin`s lymphoma
38	C8529	Non-Hodgkin`s lymphoma
38	C8580	Non-Hodgkin`s lymphoma
38	C8581	Non-Hodgkin`s lymphoma
38	C8582	Non-Hodgkin`s lymphoma

38	C8583	Non-Hodgkin`s lymphoma
38	C8584	Non-Hodgkin`s lymphoma
38	C8585	Non-Hodgkin`s lymphoma
38	C8586	Non-Hodgkin`s lymphoma
38	C8587	Non-Hodgkin`s lymphoma
38	C8588	Non-Hodgkin`s lymphoma
38	C8589	Non-Hodgkin`s lymphoma
38	C8590	Non-Hodgkin`s lymphoma
38	C8591	Non-Hodgkin`s lymphoma
38	C8592	Non-Hodgkin`s lymphoma
38	C8593	Non-Hodgkin`s lymphoma
38	C8594	Non-Hodgkin`s lymphoma
38	C8595	Non-Hodgkin`s lymphoma
38	C8596	Non-Hodgkin`s lymphoma
38	C8597	Non-Hodgkin`s lymphoma
38	C8598	Non-Hodgkin`s lymphoma
38	C8599	Non-Hodgkin`s lymphoma
38	C860	Non-Hodgkin`s lymphoma
38	C861	Non-Hodgkin`s lymphoma
38	C862	Non-Hodgkin`s lymphoma
38	C863	Non-Hodgkin`s lymphoma
38	C864	Non-Hodgkin`s lymphoma
38	C865	Non-Hodgkin`s lymphoma
38	C866	Non-Hodgkin`s lymphoma
38	C880	Non-Hodgkin`s lymphoma
38	C882	Non-Hodgkin`s lymphoma
38	C883	Non-Hodgkin`s lymphoma
38	C884	Non-Hodgkin`s lymphoma
38	C888	Non-Hodgkin`s lymphoma
38	C889	Non-Hodgkin`s lymphoma
38	C964	Non-Hodgkin`s lymphoma
38	C969	Non-Hodgkin`s lymphoma
38	C96Z	Non-Hodgkin`s lymphoma
38	Z8572	Non-Hodgkin`s lymphoma
38	Z8579	Non-Hodgkin`s lymphoma
39	C9010	Leukemias
39	C9011	Leukemias
39	C9012	Leukemias
39	C9100	Leukemias
39	C9101	Leukemias
39	C9102	Leukemias
39	C9110	Leukemias
39	C9111	Leukemias
39	C9112	Leukemias
39	C9130	Leukemias
39	C9131	Leukemias
39	C9132	Leukemias
39	C9140	Leukemias
39	C9141	Leukemias
39	C9142	Leukemias
39	C9150	Leukemias

39	C9151	Leukemias
39	C9152	Leukemias
39	C9160	Leukemias
39	C9161	Leukemias
39	C9162	Leukemias
39	C9190	Leukemias
39	C9191	Leukemias
39	C9192	Leukemias
39	C91A0	Leukemias
39	C91A1	Leukemias
39	C91A2	Leukemias
39	C91Z0	Leukemias
39	C91Z1	Leukemias
39	C91Z2	Leukemias
39	C9200	Leukemias
39	C9201	Leukemias
39	C9202	Leukemias
39	C9210	Leukemias
39	C9211	Leukemias
39	C9212	Leukemias
39	C9220	Leukemias
39	C9221	Leukemias
39	C9222	Leukemias
39	C9230	Leukemias
39	C9231	Leukemias
39	C9232	Leukemias
39	C9240	Leukemias
39	C9241	Leukemias
39	C9242	Leukemias
39	C9250	Leukemias
39	C9251	Leukemias
39	C9252	Leukemias
39	C9260	Leukemias
39	C9261	Leukemias
39	C9262	Leukemias
39	C9290	Leukemias
39	C9291	Leukemias
39	C9292	Leukemias
39	C92A0	Leukemias
39	C92A1	Leukemias
39	C92A2	Leukemias
39	C92Z0	Leukemias
39	C92Z1	Leukemias
39	C92Z2	Leukemias
39	C9300	Leukemias
39	C9301	Leukemias
39	C9302	Leukemias
39	C9310	Leukemias
39	C9311	Leukemias
39	C9312	Leukemias
39	C9330	Leukemias

39	C9331	Leukemias
39	C9332	Leukemias
39	C9390	Leukemias
39	C9391	Leukemias
39	C9392	Leukemias
39	C93Z0	Leukemias
39	C93Z1	Leukemias
39	C93Z2	Leukemias
39	C9400	Leukemias
39	C9401	Leukemias
39	C9402	Leukemias
39	C9420	Leukemias
39	C9421	Leukemias
39	C9422	Leukemias
39	C9430	Leukemias
39	C9431	Leukemias
39	C9432	Leukemias
39	C9440	Leukemias
39	C9441	Leukemias
39	C9442	Leukemias
39	C946	Leukemias
39	C9480	Leukemias
39	C9481	Leukemias
39	C9482	Leukemias
39	C9500	Leukemias
39	C9501	Leukemias
39	C9502	Leukemias
39	C9510	Leukemias
39	C9511	Leukemias
39	C9512	Leukemias
39	C9590	Leukemias
39	C9591	Leukemias
39	C9592	Leukemias
39	Z856	Leukemias
40	C9000	Multiple myeloma
40	C9001	Multiple myeloma
40	C9002	Multiple myeloma
40	C9020	Multiple myeloma
40	C9021	Multiple myeloma
40	C9022	Multiple myeloma
40	C9030	Multiple myeloma
40	C9031	Multiple myeloma
40	C9032	Multiple myeloma
41	C37	Cancer; other and unspecified primary
41	C380	Cancer; other and unspecified primary
41	C381	Cancer; other and unspecified primary
41	C382	Cancer; other and unspecified primary
41	C383	Cancer; other and unspecified primary
41	C388	Cancer; other and unspecified primary
41	C452	Cancer; other and unspecified primary
41	C457	Cancer; other and unspecified primary

41	C460	Cancer; other and unspecified primary
41	C461	Cancer; other and unspecified primary
41	C462	Cancer; other and unspecified primary
41	C463	Cancer; other and unspecified primary
41	C464	Cancer; other and unspecified primary
41	C4650	Cancer; other and unspecified primary
41	C4651	Cancer; other and unspecified primary
41	C4652	Cancer; other and unspecified primary
41	C467	Cancer; other and unspecified primary
41	C469	Cancer; other and unspecified primary
41	C6900	Cancer; other and unspecified primary
41	C6901	Cancer; other and unspecified primary
41	C6902	Cancer; other and unspecified primary
41	C6910	Cancer; other and unspecified primary
41	C6911	Cancer; other and unspecified primary
41	C6912	Cancer; other and unspecified primary
41	C6920	Cancer; other and unspecified primary
41	C6921	Cancer; other and unspecified primary
41	C6922	Cancer; other and unspecified primary
41	C6930	Cancer; other and unspecified primary
41	C6931	Cancer; other and unspecified primary
41	C6932	Cancer; other and unspecified primary
41	C6940	Cancer; other and unspecified primary
41	C6941	Cancer; other and unspecified primary
41	C6942	Cancer; other and unspecified primary
41	C6950	Cancer; other and unspecified primary
41	C6951	Cancer; other and unspecified primary
41	C6952	Cancer; other and unspecified primary
41	C6960	Cancer; other and unspecified primary
41	C6961	Cancer; other and unspecified primary
41	C6962	Cancer; other and unspecified primary
41	C6980	Cancer; other and unspecified primary
41	C6981	Cancer; other and unspecified primary
41	C6982	Cancer; other and unspecified primary
41	C6990	Cancer; other and unspecified primary
41	C6991	Cancer; other and unspecified primary
41	C6992	Cancer; other and unspecified primary
41	C7400	Cancer; other and unspecified primary
41	C7401	Cancer; other and unspecified primary
41	C7402	Cancer; other and unspecified primary
41	C7410	Cancer; other and unspecified primary
41	C7411	Cancer; other and unspecified primary
41	C7412	Cancer; other and unspecified primary
41	C7490	Cancer; other and unspecified primary
41	C7491	Cancer; other and unspecified primary
41	C7492	Cancer; other and unspecified primary
41	C750	Cancer; other and unspecified primary
41	C751	Cancer; other and unspecified primary
41	C752	Cancer; other and unspecified primary
41	C753	Cancer; other and unspecified primary
41	C754	Cancer; other and unspecified primary

41	C755	Cancer; other and unspecified primary
41	C758	Cancer; other and unspecified primary
41	C759	Cancer; other and unspecified primary
41	C761	Cancer; other and unspecified primary
41	C762	Cancer; other and unspecified primary
41	C763	Cancer; other and unspecified primary
41	C7640	Cancer; other and unspecified primary
41	C7641	Cancer; other and unspecified primary
41	C7642	Cancer; other and unspecified primary
41	C7650	Cancer; other and unspecified primary
41	C7651	Cancer; other and unspecified primary
41	C7652	Cancer; other and unspecified primary
41	C768	Cancer; other and unspecified primary
41	C7A091	Cancer; other and unspecified primary
41	C7A094	Cancer; other and unspecified primary
41	C7A095	Cancer; other and unspecified primary
41	C7A096	Cancer; other and unspecified primary
41	C960	Cancer; other and unspecified primary
41	C962	Cancer; other and unspecified primary
41	C965	Cancer; other and unspecified primary
41	C966	Cancer; other and unspecified primary
41	C96A	Cancer; other and unspecified primary
41	D0920	Cancer; other and unspecified primary
41	D0921	Cancer; other and unspecified primary
41	D0922	Cancer; other and unspecified primary
41	D098	Cancer; other and unspecified primary
41	D099	Cancer; other and unspecified primary
41	Z85020	Cancer; other and unspecified primary
41	Z85030	Cancer; other and unspecified primary
41	Z85040	Cancer; other and unspecified primary
41	Z85060	Cancer; other and unspecified primary
41	Z85110	Cancer; other and unspecified primary
41	Z85230	Cancer; other and unspecified primary
41	Z85238	Cancer; other and unspecified primary
41	Z8529	Cancer; other and unspecified primary
41	Z85520	Cancer; other and unspecified primary
41	Z85821	Cancer; other and unspecified primary
41	Z85830	Cancer; other and unspecified primary
41	Z85831	Cancer; other and unspecified primary
41	Z85840	Cancer; other and unspecified primary
41	Z85858	Cancer; other and unspecified primary
41	Z8589	Cancer; other and unspecified primary
41	Z859	Cancer; other and unspecified primary
41	Z86008	Cancer; other and unspecified primary
41	Z86012	Cancer; other and unspecified primary
41	Z86018	Cancer; other and unspecified primary
41	Z8603	Cancer; other and unspecified primary
42	C770	Secondary malignancies
42	C771	Secondary malignancies
42	C772	Secondary malignancies
42	C773	Secondary malignancies

42	C774	Secondary malignancies
42	C775	Secondary malignancies
42	C778	Secondary malignancies
42	C779	Secondary malignancies
42	C7800	Secondary malignancies
42	C7801	Secondary malignancies
42	C7802	Secondary malignancies
42	C781	Secondary malignancies
42	C782	Secondary malignancies
42	C7830	Secondary malignancies
42	C7839	Secondary malignancies
42	C784	Secondary malignancies
42	C785	Secondary malignancies
42	C786	Secondary malignancies
42	C787	Secondary malignancies
42	C7880	Secondary malignancies
42	C7889	Secondary malignancies
42	C7900	Secondary malignancies
42	C7901	Secondary malignancies
42	C7902	Secondary malignancies
42	C7910	Secondary malignancies
42	C7911	Secondary malignancies
42	C7919	Secondary malignancies
42	C792	Secondary malignancies
42	C7931	Secondary malignancies
42	C7932	Secondary malignancies
42	C7940	Secondary malignancies
42	C7949	Secondary malignancies
42	C7951	Secondary malignancies
42	C7952	Secondary malignancies
42	C7960	Secondary malignancies
42	C7961	Secondary malignancies
42	C7962	Secondary malignancies
42	C7970	Secondary malignancies
42	C7971	Secondary malignancies
42	C7972	Secondary malignancies
42	C7981	Secondary malignancies
42	C7982	Secondary malignancies
42	C7989	Secondary malignancies
42	C799	Secondary malignancies
42	C7B01	Secondary malignancies
42	C7B02	Secondary malignancies
42	C7B03	Secondary malignancies
42	C7B04	Secondary malignancies
42	J910	Secondary malignancies
42	R180	Secondary malignancies
43	C459	Malignant neoplasm without specification of site
43	C7A00	Malignant neoplasm without specification of site
43	C7A098	Malignant neoplasm without specification of site
43	C7A1	Malignant neoplasm without specification of site
43	C7A8	Malignant neoplasm without specification of site

43	C7B00	Malignant neoplasm without specification of site
43	C7B09	Malignant neoplasm without specification of site
43	C7B1	Malignant neoplasm without specification of site
43	C7B8	Malignant neoplasm without specification of site
43	C800	Malignant neoplasm without specification of site
43	C801	Malignant neoplasm without specification of site
43	C802	Malignant neoplasm without specification of site
44	D3701	Neoplasms of unspecified nature or uncertain behavior
44	D3702	Neoplasms of unspecified nature or uncertain behavior
44	D37030	Neoplasms of unspecified nature or uncertain behavior
44	D37031	Neoplasms of unspecified nature or uncertain behavior
44	D37032	Neoplasms of unspecified nature or uncertain behavior
44	D37039	Neoplasms of unspecified nature or uncertain behavior
44	D3704	Neoplasms of unspecified nature or uncertain behavior
44	D3705	Neoplasms of unspecified nature or uncertain behavior
44	D3709	Neoplasms of unspecified nature or uncertain behavior
44	D371	Neoplasms of unspecified nature or uncertain behavior
44	D372	Neoplasms of unspecified nature or uncertain behavior
44	D373	Neoplasms of unspecified nature or uncertain behavior
44	D374	Neoplasms of unspecified nature or uncertain behavior
44	D375	Neoplasms of unspecified nature or uncertain behavior
44	D376	Neoplasms of unspecified nature or uncertain behavior
44	D378	Neoplasms of unspecified nature or uncertain behavior
44	D379	Neoplasms of unspecified nature or uncertain behavior
44	D380	Neoplasms of unspecified nature or uncertain behavior
44	D381	Neoplasms of unspecified nature or uncertain behavior
44	D382	Neoplasms of unspecified nature or uncertain behavior
44	D383	Neoplasms of unspecified nature or uncertain behavior
44	D384	Neoplasms of unspecified nature or uncertain behavior
44	D385	Neoplasms of unspecified nature or uncertain behavior
44	D386	Neoplasms of unspecified nature or uncertain behavior
44	D390	Neoplasms of unspecified nature or uncertain behavior
44	D3910	Neoplasms of unspecified nature or uncertain behavior
44	D3911	Neoplasms of unspecified nature or uncertain behavior
44	D3912	Neoplasms of unspecified nature or uncertain behavior
44	D392	Neoplasms of unspecified nature or uncertain behavior
44	D398	Neoplasms of unspecified nature or uncertain behavior
44	D399	Neoplasms of unspecified nature or uncertain behavior
44	D400	Neoplasms of unspecified nature or uncertain behavior
44	D4010	Neoplasms of unspecified nature or uncertain behavior
44	D4011	Neoplasms of unspecified nature or uncertain behavior
44	D4012	Neoplasms of unspecified nature or uncertain behavior
44	D408	Neoplasms of unspecified nature or uncertain behavior
44	D409	Neoplasms of unspecified nature or uncertain behavior
44	D4100	Neoplasms of unspecified nature or uncertain behavior
44	D4101	Neoplasms of unspecified nature or uncertain behavior
44	D4102	Neoplasms of unspecified nature or uncertain behavior
44	D4110	Neoplasms of unspecified nature or uncertain behavior
44	D4111	Neoplasms of unspecified nature or uncertain behavior
44	D4112	Neoplasms of unspecified nature or uncertain behavior
44	D4120	Neoplasms of unspecified nature or uncertain behavior

44	D4121	Neoplasms of unspecified nature or uncertain behavior
44	D4122	Neoplasms of unspecified nature or uncertain behavior
44	D413	Neoplasms of unspecified nature or uncertain behavior
44	D414	Neoplasms of unspecified nature or uncertain behavior
44	D418	Neoplasms of unspecified nature or uncertain behavior
44	D419	Neoplasms of unspecified nature or uncertain behavior
44	D420	Neoplasms of unspecified nature or uncertain behavior
44	D421	Neoplasms of unspecified nature or uncertain behavior
44	D429	Neoplasms of unspecified nature or uncertain behavior
44	D430	Neoplasms of unspecified nature or uncertain behavior
44	D431	Neoplasms of unspecified nature or uncertain behavior
44	D432	Neoplasms of unspecified nature or uncertain behavior
44	D433	Neoplasms of unspecified nature or uncertain behavior
44	D434	Neoplasms of unspecified nature or uncertain behavior
44	D438	Neoplasms of unspecified nature or uncertain behavior
44	D439	Neoplasms of unspecified nature or uncertain behavior
44	D440	Neoplasms of unspecified nature or uncertain behavior
44	D4410	Neoplasms of unspecified nature or uncertain behavior
44	D4411	Neoplasms of unspecified nature or uncertain behavior
44	D4412	Neoplasms of unspecified nature or uncertain behavior
44	D442	Neoplasms of unspecified nature or uncertain behavior
44	D443	Neoplasms of unspecified nature or uncertain behavior
44	D444	Neoplasms of unspecified nature or uncertain behavior
44	D445	Neoplasms of unspecified nature or uncertain behavior
44	D446	Neoplasms of unspecified nature or uncertain behavior
44	D447	Neoplasms of unspecified nature or uncertain behavior
44	D449	Neoplasms of unspecified nature or uncertain behavior
44	D45	Neoplasms of unspecified nature or uncertain behavior
44	D460	Neoplasms of unspecified nature or uncertain behavior
44	D461	Neoplasms of unspecified nature or uncertain behavior
44	D4620	Neoplasms of unspecified nature or uncertain behavior
44	D4621	Neoplasms of unspecified nature or uncertain behavior
44	D4622	Neoplasms of unspecified nature or uncertain behavior
44	D464	Neoplasms of unspecified nature or uncertain behavior
44	D469	Neoplasms of unspecified nature or uncertain behavior
44	D46A	Neoplasms of unspecified nature or uncertain behavior
44	D46B	Neoplasms of unspecified nature or uncertain behavior
44	D46C	Neoplasms of unspecified nature or uncertain behavior
44	D46Z	Neoplasms of unspecified nature or uncertain behavior
44	D470	Neoplasms of unspecified nature or uncertain behavior
44	D471	Neoplasms of unspecified nature or uncertain behavior
44	D472	Neoplasms of unspecified nature or uncertain behavior
44	D473	Neoplasms of unspecified nature or uncertain behavior
44	D474	Neoplasms of unspecified nature or uncertain behavior
44	D479	Neoplasms of unspecified nature or uncertain behavior
44	D47Z1	Neoplasms of unspecified nature or uncertain behavior
44	D47Z9	Neoplasms of unspecified nature or uncertain behavior
44	D480	Neoplasms of unspecified nature or uncertain behavior
44	D481	Neoplasms of unspecified nature or uncertain behavior
44	D482	Neoplasms of unspecified nature or uncertain behavior
44	D483	Neoplasms of unspecified nature or uncertain behavior

44	D484	Neoplasms of unspecified nature or uncertain behavior
44	D485	Neoplasms of unspecified nature or uncertain behavior
44	D4860	Neoplasms of unspecified nature or uncertain behavior
44	D4861	Neoplasms of unspecified nature or uncertain behavior
44	D4862	Neoplasms of unspecified nature or uncertain behavior
44	D487	Neoplasms of unspecified nature or uncertain behavior
44	D489	Neoplasms of unspecified nature or uncertain behavior
44	D490	Neoplasms of unspecified nature or uncertain behavior
44	D491	Neoplasms of unspecified nature or uncertain behavior
44	D492	Neoplasms of unspecified nature or uncertain behavior
44	D493	Neoplasms of unspecified nature or uncertain behavior
44	D494	Neoplasms of unspecified nature or uncertain behavior
44	D495	Neoplasms of unspecified nature or uncertain behavior
44	D496	Neoplasms of unspecified nature or uncertain behavior
44	D497	Neoplasms of unspecified nature or uncertain behavior
44	D4981	Neoplasms of unspecified nature or uncertain behavior
44	D4989	Neoplasms of unspecified nature or uncertain behavior
44	D499	Neoplasms of unspecified nature or uncertain behavior
45	Z510	Maintenance chemotherapy; radiotherapy
45	Z5111	Maintenance chemotherapy; radiotherapy
45	Z5112	Maintenance chemotherapy; radiotherapy
46	D250	Benign neoplasm of uterus
46	D251	Benign neoplasm of uterus
46	D252	Benign neoplasm of uterus
46	D259	Benign neoplasm of uterus
46	D260	Benign neoplasm of uterus
46	D261	Benign neoplasm of uterus
46	D267	Benign neoplasm of uterus
46	D269	Benign neoplasm of uterus
47	D100	Other and unspecified benign neoplasm
47	D101	Other and unspecified benign neoplasm
47	D102	Other and unspecified benign neoplasm
47	D1030	Other and unspecified benign neoplasm
47	D1039	Other and unspecified benign neoplasm
47	D104	Other and unspecified benign neoplasm
47	D105	Other and unspecified benign neoplasm
47	D106	Other and unspecified benign neoplasm
47	D107	Other and unspecified benign neoplasm
47	D109	Other and unspecified benign neoplasm
47	D110	Other and unspecified benign neoplasm
47	D117	Other and unspecified benign neoplasm
47	D119	Other and unspecified benign neoplasm
47	D120	Other and unspecified benign neoplasm
47	D121	Other and unspecified benign neoplasm
47	D122	Other and unspecified benign neoplasm
47	D123	Other and unspecified benign neoplasm
47	D124	Other and unspecified benign neoplasm
47	D125	Other and unspecified benign neoplasm
47	D126	Other and unspecified benign neoplasm
47	D127	Other and unspecified benign neoplasm
47	D128	Other and unspecified benign neoplasm

47	D129	Other and unspecified benign neoplasm
47	D130	Other and unspecified benign neoplasm
47	D131	Other and unspecified benign neoplasm
47	D132	Other and unspecified benign neoplasm
47	D1330	Other and unspecified benign neoplasm
47	D1339	Other and unspecified benign neoplasm
47	D134	Other and unspecified benign neoplasm
47	D135	Other and unspecified benign neoplasm
47	D136	Other and unspecified benign neoplasm
47	D137	Other and unspecified benign neoplasm
47	D139	Other and unspecified benign neoplasm
47	D140	Other and unspecified benign neoplasm
47	D141	Other and unspecified benign neoplasm
47	D142	Other and unspecified benign neoplasm
47	D1430	Other and unspecified benign neoplasm
47	D1431	Other and unspecified benign neoplasm
47	D1432	Other and unspecified benign neoplasm
47	D144	Other and unspecified benign neoplasm
47	D150	Other and unspecified benign neoplasm
47	D151	Other and unspecified benign neoplasm
47	D152	Other and unspecified benign neoplasm
47	D157	Other and unspecified benign neoplasm
47	D159	Other and unspecified benign neoplasm
47	D1600	Other and unspecified benign neoplasm
47	D1601	Other and unspecified benign neoplasm
47	D1602	Other and unspecified benign neoplasm
47	D1610	Other and unspecified benign neoplasm
47	D1611	Other and unspecified benign neoplasm
47	D1612	Other and unspecified benign neoplasm
47	D1620	Other and unspecified benign neoplasm
47	D1621	Other and unspecified benign neoplasm
47	D1622	Other and unspecified benign neoplasm
47	D1630	Other and unspecified benign neoplasm
47	D1631	Other and unspecified benign neoplasm
47	D1632	Other and unspecified benign neoplasm
47	D164	Other and unspecified benign neoplasm
47	D165	Other and unspecified benign neoplasm
47	D166	Other and unspecified benign neoplasm
47	D167	Other and unspecified benign neoplasm
47	D168	Other and unspecified benign neoplasm
47	D169	Other and unspecified benign neoplasm
47	D170	Other and unspecified benign neoplasm
47	D171	Other and unspecified benign neoplasm
47	D1720	Other and unspecified benign neoplasm
47	D1721	Other and unspecified benign neoplasm
47	D1722	Other and unspecified benign neoplasm
47	D1723	Other and unspecified benign neoplasm
47	D1724	Other and unspecified benign neoplasm
47	D1730	Other and unspecified benign neoplasm
47	D1739	Other and unspecified benign neoplasm
47	D174	Other and unspecified benign neoplasm

47	D175	Other and unspecified benign neoplasm
47	D176	Other and unspecified benign neoplasm
47	D1771	Other and unspecified benign neoplasm
47	D1772	Other and unspecified benign neoplasm
47	D1779	Other and unspecified benign neoplasm
47	D179	Other and unspecified benign neoplasm
47	D1800	Other and unspecified benign neoplasm
47	D1801	Other and unspecified benign neoplasm
47	D1802	Other and unspecified benign neoplasm
47	D1803	Other and unspecified benign neoplasm
47	D1809	Other and unspecified benign neoplasm
47	D181	Other and unspecified benign neoplasm
47	D190	Other and unspecified benign neoplasm
47	D191	Other and unspecified benign neoplasm
47	D197	Other and unspecified benign neoplasm
47	D199	Other and unspecified benign neoplasm
47	D200	Other and unspecified benign neoplasm
47	D201	Other and unspecified benign neoplasm
47	D210	Other and unspecified benign neoplasm
47	D2110	Other and unspecified benign neoplasm
47	D2111	Other and unspecified benign neoplasm
47	D2112	Other and unspecified benign neoplasm
47	D2120	Other and unspecified benign neoplasm
47	D2121	Other and unspecified benign neoplasm
47	D2122	Other and unspecified benign neoplasm
47	D213	Other and unspecified benign neoplasm
47	D214	Other and unspecified benign neoplasm
47	D215	Other and unspecified benign neoplasm
47	D216	Other and unspecified benign neoplasm
47	D219	Other and unspecified benign neoplasm
47	D220	Other and unspecified benign neoplasm
47	D2210	Other and unspecified benign neoplasm
47	D2211	Other and unspecified benign neoplasm
47	D2212	Other and unspecified benign neoplasm
47	D2220	Other and unspecified benign neoplasm
47	D2221	Other and unspecified benign neoplasm
47	D2222	Other and unspecified benign neoplasm
47	D2230	Other and unspecified benign neoplasm
47	D2239	Other and unspecified benign neoplasm
47	D224	Other and unspecified benign neoplasm
47	D225	Other and unspecified benign neoplasm
47	D2260	Other and unspecified benign neoplasm
47	D2261	Other and unspecified benign neoplasm
47	D2262	Other and unspecified benign neoplasm
47	D2270	Other and unspecified benign neoplasm
47	D2271	Other and unspecified benign neoplasm
47	D2272	Other and unspecified benign neoplasm
47	D229	Other and unspecified benign neoplasm
47	D230	Other and unspecified benign neoplasm
47	D2310	Other and unspecified benign neoplasm
47	D2311	Other and unspecified benign neoplasm

47	D2312	Other and unspecified benign neoplasm
47	D2320	Other and unspecified benign neoplasm
47	D2321	Other and unspecified benign neoplasm
47	D2322	Other and unspecified benign neoplasm
47	D2330	Other and unspecified benign neoplasm
47	D2339	Other and unspecified benign neoplasm
47	D234	Other and unspecified benign neoplasm
47	D235	Other and unspecified benign neoplasm
47	D2360	Other and unspecified benign neoplasm
47	D2361	Other and unspecified benign neoplasm
47	D2362	Other and unspecified benign neoplasm
47	D2370	Other and unspecified benign neoplasm
47	D2371	Other and unspecified benign neoplasm
47	D2372	Other and unspecified benign neoplasm
47	D239	Other and unspecified benign neoplasm
47	D241	Other and unspecified benign neoplasm
47	D242	Other and unspecified benign neoplasm
47	D249	Other and unspecified benign neoplasm
47	D270	Other and unspecified benign neoplasm
47	D271	Other and unspecified benign neoplasm
47	D279	Other and unspecified benign neoplasm
47	D280	Other and unspecified benign neoplasm
47	D281	Other and unspecified benign neoplasm
47	D282	Other and unspecified benign neoplasm
47	D287	Other and unspecified benign neoplasm
47	D289	Other and unspecified benign neoplasm
47	D290	Other and unspecified benign neoplasm
47	D291	Other and unspecified benign neoplasm
47	D2920	Other and unspecified benign neoplasm
47	D2921	Other and unspecified benign neoplasm
47	D2922	Other and unspecified benign neoplasm
47	D2930	Other and unspecified benign neoplasm
47	D2931	Other and unspecified benign neoplasm
47	D2932	Other and unspecified benign neoplasm
47	D294	Other and unspecified benign neoplasm
47	D298	Other and unspecified benign neoplasm
47	D299	Other and unspecified benign neoplasm
47	D3000	Other and unspecified benign neoplasm
47	D3001	Other and unspecified benign neoplasm
47	D3002	Other and unspecified benign neoplasm
47	D3010	Other and unspecified benign neoplasm
47	D3011	Other and unspecified benign neoplasm
47	D3012	Other and unspecified benign neoplasm
47	D3020	Other and unspecified benign neoplasm
47	D3021	Other and unspecified benign neoplasm
47	D3022	Other and unspecified benign neoplasm
47	D303	Other and unspecified benign neoplasm
47	D304	Other and unspecified benign neoplasm
47	D308	Other and unspecified benign neoplasm
47	D309	Other and unspecified benign neoplasm
47	D3100	Other and unspecified benign neoplasm

47	D3101	Other and unspecified benign neoplasm
47	D3102	Other and unspecified benign neoplasm
47	D3110	Other and unspecified benign neoplasm
47	D3111	Other and unspecified benign neoplasm
47	D3112	Other and unspecified benign neoplasm
47	D3120	Other and unspecified benign neoplasm
47	D3121	Other and unspecified benign neoplasm
47	D3122	Other and unspecified benign neoplasm
47	D3130	Other and unspecified benign neoplasm
47	D3131	Other and unspecified benign neoplasm
47	D3132	Other and unspecified benign neoplasm
47	D3140	Other and unspecified benign neoplasm
47	D3141	Other and unspecified benign neoplasm
47	D3142	Other and unspecified benign neoplasm
47	D3150	Other and unspecified benign neoplasm
47	D3151	Other and unspecified benign neoplasm
47	D3152	Other and unspecified benign neoplasm
47	D3160	Other and unspecified benign neoplasm
47	D3161	Other and unspecified benign neoplasm
47	D3162	Other and unspecified benign neoplasm
47	D3190	Other and unspecified benign neoplasm
47	D3191	Other and unspecified benign neoplasm
47	D3192	Other and unspecified benign neoplasm
47	D320	Other and unspecified benign neoplasm
47	D321	Other and unspecified benign neoplasm
47	D329	Other and unspecified benign neoplasm
47	D330	Other and unspecified benign neoplasm
47	D331	Other and unspecified benign neoplasm
47	D332	Other and unspecified benign neoplasm
47	D333	Other and unspecified benign neoplasm
47	D334	Other and unspecified benign neoplasm
47	D337	Other and unspecified benign neoplasm
47	D339	Other and unspecified benign neoplasm
47	D34	Other and unspecified benign neoplasm
47	D3500	Other and unspecified benign neoplasm
47	D3501	Other and unspecified benign neoplasm
47	D3502	Other and unspecified benign neoplasm
47	D351	Other and unspecified benign neoplasm
47	D352	Other and unspecified benign neoplasm
47	D353	Other and unspecified benign neoplasm
47	D354	Other and unspecified benign neoplasm
47	D355	Other and unspecified benign neoplasm
47	D356	Other and unspecified benign neoplasm
47	D357	Other and unspecified benign neoplasm
47	D359	Other and unspecified benign neoplasm
47	D360	Other and unspecified benign neoplasm
47	D3610	Other and unspecified benign neoplasm
47	D3611	Other and unspecified benign neoplasm
47	D3612	Other and unspecified benign neoplasm
47	D3613	Other and unspecified benign neoplasm
47	D3614	Other and unspecified benign neoplasm

47	D3615	Other and unspecified benign neoplasm
47	D3616	Other and unspecified benign neoplasm
47	D3617	Other and unspecified benign neoplasm
47	D367	Other and unspecified benign neoplasm
47	D369	Other and unspecified benign neoplasm
47	D3A00	Other and unspecified benign neoplasm
47	D3A010	Other and unspecified benign neoplasm
47	D3A011	Other and unspecified benign neoplasm
47	D3A012	Other and unspecified benign neoplasm
47	D3A019	Other and unspecified benign neoplasm
47	D3A020	Other and unspecified benign neoplasm
47	D3A021	Other and unspecified benign neoplasm
47	D3A022	Other and unspecified benign neoplasm
47	D3A023	Other and unspecified benign neoplasm
47	D3A024	Other and unspecified benign neoplasm
47	D3A025	Other and unspecified benign neoplasm
47	D3A026	Other and unspecified benign neoplasm
47	D3A029	Other and unspecified benign neoplasm
47	D3A090	Other and unspecified benign neoplasm
47	D3A091	Other and unspecified benign neoplasm
47	D3A092	Other and unspecified benign neoplasm
47	D3A093	Other and unspecified benign neoplasm
47	D3A094	Other and unspecified benign neoplasm
47	D3A095	Other and unspecified benign neoplasm
47	D3A096	Other and unspecified benign neoplasm
47	D3A098	Other and unspecified benign neoplasm
47	D3A8	Other and unspecified benign neoplasm
47	K317	Other and unspecified benign neoplasm
47	K635	Other and unspecified benign neoplasm
47	Z86010	Other and unspecified benign neoplasm
48	E000	Thyroid disorders
48	E001	Thyroid disorders
48	E002	Thyroid disorders
48	E009	Thyroid disorders
48	E010	Thyroid disorders
48	E011	Thyroid disorders
48	E012	Thyroid disorders
48	E018	Thyroid disorders
48	E02	Thyroid disorders
48	E030	Thyroid disorders
48	E031	Thyroid disorders
48	E032	Thyroid disorders
48	E033	Thyroid disorders
48	E034	Thyroid disorders
48	E035	Thyroid disorders
48	E038	Thyroid disorders
48	E039	Thyroid disorders
48	E040	Thyroid disorders
48	E041	Thyroid disorders
48	E042	Thyroid disorders
48	E048	Thyroid disorders

48	E049	Thyroid disorders
48	E0500	Thyroid disorders
48	E0501	Thyroid disorders
48	E0510	Thyroid disorders
48	E0511	Thyroid disorders
48	E0520	Thyroid disorders
48	E0521	Thyroid disorders
48	E0530	Thyroid disorders
48	E0531	Thyroid disorders
48	E0540	Thyroid disorders
48	E0541	Thyroid disorders
48	E0580	Thyroid disorders
48	E0581	Thyroid disorders
48	E0590	Thyroid disorders
48	E0591	Thyroid disorders
48	E060	Thyroid disorders
48	E061	Thyroid disorders
48	E062	Thyroid disorders
48	E063	Thyroid disorders
48	E064	Thyroid disorders
48	E065	Thyroid disorders
48	E069	Thyroid disorders
48	E070	Thyroid disorders
48	E071	Thyroid disorders
48	E0781	Thyroid disorders
48	E0789	Thyroid disorders
48	E079	Thyroid disorders
49	E089	Diabetes mellitus without complication
49	E099	Diabetes mellitus without complication
49	E109	Diabetes mellitus without complication
49	E119	Diabetes mellitus without complication
49	E139	Diabetes mellitus without complication
49	R7301	Diabetes mellitus without complication
49	R7302	Diabetes mellitus without complication
49	R7309	Diabetes mellitus without complication
49	R739	Diabetes mellitus without complication
49	R81	Diabetes mellitus without complication
49	R824	Diabetes mellitus without complication
49	Z4681	Diabetes mellitus without complication
49	Z9641	Diabetes mellitus without complication
50	E0800	Diabetes mellitus with complications
50	E0801	Diabetes mellitus with complications
50	E0810	Diabetes mellitus with complications
50	E0811	Diabetes mellitus with complications
50	E0821	Diabetes mellitus with complications
50	E0822	Diabetes mellitus with complications
50	E0829	Diabetes mellitus with complications
50	E08311	Diabetes mellitus with complications
50	E08319	Diabetes mellitus with complications
50	E08321	Diabetes mellitus with complications
50	E08329	Diabetes mellitus with complications

50	E08331	Diabetes mellitus with complications
50	E08339	Diabetes mellitus with complications
50	E08341	Diabetes mellitus with complications
50	E08349	Diabetes mellitus with complications
50	E08351	Diabetes mellitus with complications
50	E08359	Diabetes mellitus with complications
50	E0836	Diabetes mellitus with complications
50	E0839	Diabetes mellitus with complications
50	E0840	Diabetes mellitus with complications
50	E0841	Diabetes mellitus with complications
50	E0842	Diabetes mellitus with complications
50	E0843	Diabetes mellitus with complications
50	E0844	Diabetes mellitus with complications
50	E0849	Diabetes mellitus with complications
50	E0851	Diabetes mellitus with complications
50	E0852	Diabetes mellitus with complications
50	E0859	Diabetes mellitus with complications
50	E08610	Diabetes mellitus with complications
50	E08618	Diabetes mellitus with complications
50	E08620	Diabetes mellitus with complications
50	E08621	Diabetes mellitus with complications
50	E08622	Diabetes mellitus with complications
50	E08628	Diabetes mellitus with complications
50	E08630	Diabetes mellitus with complications
50	E08638	Diabetes mellitus with complications
50	E08641	Diabetes mellitus with complications
50	E08649	Diabetes mellitus with complications
50	E0865	Diabetes mellitus with complications
50	E0869	Diabetes mellitus with complications
50	E088	Diabetes mellitus with complications
50	E0900	Diabetes mellitus with complications
50	E0901	Diabetes mellitus with complications
50	E0910	Diabetes mellitus with complications
50	E0911	Diabetes mellitus with complications
50	E0921	Diabetes mellitus with complications
50	E0922	Diabetes mellitus with complications
50	E0929	Diabetes mellitus with complications
50	E09311	Diabetes mellitus with complications
50	E09319	Diabetes mellitus with complications
50	E09321	Diabetes mellitus with complications
50	E09329	Diabetes mellitus with complications
50	E09331	Diabetes mellitus with complications
50	E09339	Diabetes mellitus with complications
50	E09341	Diabetes mellitus with complications
50	E09349	Diabetes mellitus with complications
50	E09351	Diabetes mellitus with complications
50	E09359	Diabetes mellitus with complications
50	E0936	Diabetes mellitus with complications
50	E0939	Diabetes mellitus with complications
50	E0940	Diabetes mellitus with complications
50	E0941	Diabetes mellitus with complications

50	E0942	Diabetes mellitus with complications
50	E0943	Diabetes mellitus with complications
50	E0944	Diabetes mellitus with complications
50	E0949	Diabetes mellitus with complications
50	E0951	Diabetes mellitus with complications
50	E0952	Diabetes mellitus with complications
50	E0959	Diabetes mellitus with complications
50	E09610	Diabetes mellitus with complications
50	E09618	Diabetes mellitus with complications
50	E09620	Diabetes mellitus with complications
50	E09621	Diabetes mellitus with complications
50	E09622	Diabetes mellitus with complications
50	E09628	Diabetes mellitus with complications
50	E09630	Diabetes mellitus with complications
50	E09638	Diabetes mellitus with complications
50	E09641	Diabetes mellitus with complications
50	E09649	Diabetes mellitus with complications
50	E0965	Diabetes mellitus with complications
50	E0969	Diabetes mellitus with complications
50	E098	Diabetes mellitus with complications
50	E1010	Diabetes mellitus with complications
50	E1011	Diabetes mellitus with complications
50	E1021	Diabetes mellitus with complications
50	E1022	Diabetes mellitus with complications
50	E1029	Diabetes mellitus with complications
50	E10311	Diabetes mellitus with complications
50	E10319	Diabetes mellitus with complications
50	E10321	Diabetes mellitus with complications
50	E10329	Diabetes mellitus with complications
50	E10331	Diabetes mellitus with complications
50	E10339	Diabetes mellitus with complications
50	E10341	Diabetes mellitus with complications
50	E10349	Diabetes mellitus with complications
50	E10351	Diabetes mellitus with complications
50	E10359	Diabetes mellitus with complications
50	E1036	Diabetes mellitus with complications
50	E1039	Diabetes mellitus with complications
50	E1040	Diabetes mellitus with complications
50	E1041	Diabetes mellitus with complications
50	E1042	Diabetes mellitus with complications
50	E1043	Diabetes mellitus with complications
50	E1044	Diabetes mellitus with complications
50	E1049	Diabetes mellitus with complications
50	E1051	Diabetes mellitus with complications
50	E1052	Diabetes mellitus with complications
50	E1059	Diabetes mellitus with complications
50	E10610	Diabetes mellitus with complications
50	E10618	Diabetes mellitus with complications
50	E10620	Diabetes mellitus with complications
50	E10621	Diabetes mellitus with complications
50	E10622	Diabetes mellitus with complications

50	E10628	Diabetes mellitus with complications
50	E10630	Diabetes mellitus with complications
50	E10638	Diabetes mellitus with complications
50	E10641	Diabetes mellitus with complications
50	E10649	Diabetes mellitus with complications
50	E1065	Diabetes mellitus with complications
50	E1069	Diabetes mellitus with complications
50	E108	Diabetes mellitus with complications
50	E1100	Diabetes mellitus with complications
50	E1101	Diabetes mellitus with complications
50	E1121	Diabetes mellitus with complications
50	E1122	Diabetes mellitus with complications
50	E1129	Diabetes mellitus with complications
50	E11311	Diabetes mellitus with complications
50	E11319	Diabetes mellitus with complications
50	E11321	Diabetes mellitus with complications
50	E11329	Diabetes mellitus with complications
50	E11331	Diabetes mellitus with complications
50	E11339	Diabetes mellitus with complications
50	E11341	Diabetes mellitus with complications
50	E11349	Diabetes mellitus with complications
50	E11351	Diabetes mellitus with complications
50	E11359	Diabetes mellitus with complications
50	E1136	Diabetes mellitus with complications
50	E1139	Diabetes mellitus with complications
50	E1140	Diabetes mellitus with complications
50	E1141	Diabetes mellitus with complications
50	E1142	Diabetes mellitus with complications
50	E1143	Diabetes mellitus with complications
50	E1144	Diabetes mellitus with complications
50	E1149	Diabetes mellitus with complications
50	E1151	Diabetes mellitus with complications
50	E1152	Diabetes mellitus with complications
50	E1159	Diabetes mellitus with complications
50	E11610	Diabetes mellitus with complications
50	E11618	Diabetes mellitus with complications
50	E11620	Diabetes mellitus with complications
50	E11621	Diabetes mellitus with complications
50	E11622	Diabetes mellitus with complications
50	E11628	Diabetes mellitus with complications
50	E11630	Diabetes mellitus with complications
50	E11638	Diabetes mellitus with complications
50	E11641	Diabetes mellitus with complications
50	E11649	Diabetes mellitus with complications
50	E1165	Diabetes mellitus with complications
50	E1169	Diabetes mellitus with complications
50	E118	Diabetes mellitus with complications
50	E1300	Diabetes mellitus with complications
50	E1301	Diabetes mellitus with complications
50	E1310	Diabetes mellitus with complications
50	E1311	Diabetes mellitus with complications

50	E1321	Diabetes mellitus with complications
50	E1322	Diabetes mellitus with complications
50	E1329	Diabetes mellitus with complications
50	E13311	Diabetes mellitus with complications
50	E13319	Diabetes mellitus with complications
50	E13321	Diabetes mellitus with complications
50	E13329	Diabetes mellitus with complications
50	E13331	Diabetes mellitus with complications
50	E13339	Diabetes mellitus with complications
50	E13341	Diabetes mellitus with complications
50	E13349	Diabetes mellitus with complications
50	E13351	Diabetes mellitus with complications
50	E13359	Diabetes mellitus with complications
50	E1336	Diabetes mellitus with complications
50	E1339	Diabetes mellitus with complications
50	E1340	Diabetes mellitus with complications
50	E1341	Diabetes mellitus with complications
50	E1342	Diabetes mellitus with complications
50	E1343	Diabetes mellitus with complications
50	E1344	Diabetes mellitus with complications
50	E1349	Diabetes mellitus with complications
50	E1351	Diabetes mellitus with complications
50	E1352	Diabetes mellitus with complications
50	E1359	Diabetes mellitus with complications
50	E13610	Diabetes mellitus with complications
50	E13618	Diabetes mellitus with complications
50	E13620	Diabetes mellitus with complications
50	E13621	Diabetes mellitus with complications
50	E13622	Diabetes mellitus with complications
50	E13628	Diabetes mellitus with complications
50	E13630	Diabetes mellitus with complications
50	E13638	Diabetes mellitus with complications
50	E13641	Diabetes mellitus with complications
50	E13649	Diabetes mellitus with complications
50	E1365	Diabetes mellitus with complications
50	E1369	Diabetes mellitus with complications
50	E138	Diabetes mellitus with complications
50	G3289	Diabetes mellitus with complications
51	E15	Other endocrine disorders
51	E160	Other endocrine disorders
51	E161	Other endocrine disorders
51	E162	Other endocrine disorders
51	E163	Other endocrine disorders
51	E164	Other endocrine disorders
51	E168	Other endocrine disorders
51	E169	Other endocrine disorders
51	E200	Other endocrine disorders
51	E208	Other endocrine disorders
51	E209	Other endocrine disorders
51	E210	Other endocrine disorders
51	E211	Other endocrine disorders

51	E212	Other endocrine disorders
51	E213	Other endocrine disorders
51	E214	Other endocrine disorders
51	E215	Other endocrine disorders
51	E220	Other endocrine disorders
51	E221	Other endocrine disorders
51	E222	Other endocrine disorders
51	E228	Other endocrine disorders
51	E229	Other endocrine disorders
51	E230	Other endocrine disorders
51	E231	Other endocrine disorders
51	E232	Other endocrine disorders
51	E233	Other endocrine disorders
51	E236	Other endocrine disorders
51	E237	Other endocrine disorders
51	E240	Other endocrine disorders
51	E241	Other endocrine disorders
51	E242	Other endocrine disorders
51	E243	Other endocrine disorders
51	E244	Other endocrine disorders
51	E248	Other endocrine disorders
51	E249	Other endocrine disorders
51	E250	Other endocrine disorders
51	E258	Other endocrine disorders
51	E259	Other endocrine disorders
51	E2601	Other endocrine disorders
51	E2602	Other endocrine disorders
51	E2609	Other endocrine disorders
51	E261	Other endocrine disorders
51	E2681	Other endocrine disorders
51	E2689	Other endocrine disorders
51	E269	Other endocrine disorders
51	E270	Other endocrine disorders
51	E271	Other endocrine disorders
51	E272	Other endocrine disorders
51	E273	Other endocrine disorders
51	E2740	Other endocrine disorders
51	E2749	Other endocrine disorders
51	E275	Other endocrine disorders
51	E278	Other endocrine disorders
51	E279	Other endocrine disorders
51	E280	Other endocrine disorders
51	E281	Other endocrine disorders
51	E282	Other endocrine disorders
51	E288	Other endocrine disorders
51	E289	Other endocrine disorders
51	E290	Other endocrine disorders
51	E291	Other endocrine disorders
51	E298	Other endocrine disorders
51	E299	Other endocrine disorders
51	E300	Other endocrine disorders

51	E301	Other endocrine disorders
51	E308	Other endocrine disorders
51	E309	Other endocrine disorders
51	E310	Other endocrine disorders
51	E311	Other endocrine disorders
51	E3120	Other endocrine disorders
51	E3121	Other endocrine disorders
51	E318	Other endocrine disorders
51	E319	Other endocrine disorders
51	E320	Other endocrine disorders
51	E321	Other endocrine disorders
51	E328	Other endocrine disorders
51	E329	Other endocrine disorders
51	E340	Other endocrine disorders
51	E341	Other endocrine disorders
51	E342	Other endocrine disorders
51	E343	Other endocrine disorders
51	E344	Other endocrine disorders
51	E3450	Other endocrine disorders
51	E3451	Other endocrine disorders
51	E3452	Other endocrine disorders
51	E348	Other endocrine disorders
51	E349	Other endocrine disorders
51	E35	Other endocrine disorders
51	Z9483	Other endocrine disorders
51	Z9649	Other endocrine disorders
52	D81818	Nutritional deficiencies
52	D81819	Nutritional deficiencies
52	E40	Nutritional deficiencies
52	E41	Nutritional deficiencies
52	E42	Nutritional deficiencies
52	E43	Nutritional deficiencies
52	E440	Nutritional deficiencies
52	E441	Nutritional deficiencies
52	E45	Nutritional deficiencies
52	E46	Nutritional deficiencies
52	E500	Nutritional deficiencies
52	E501	Nutritional deficiencies
52	E502	Nutritional deficiencies
52	E503	Nutritional deficiencies
52	E504	Nutritional deficiencies
52	E505	Nutritional deficiencies
52	E506	Nutritional deficiencies
52	E507	Nutritional deficiencies
52	E508	Nutritional deficiencies
52	E509	Nutritional deficiencies
52	E5111	Nutritional deficiencies
52	E5112	Nutritional deficiencies
52	E512	Nutritional deficiencies
52	E518	Nutritional deficiencies
52	E519	Nutritional deficiencies

52	E52	Nutritional deficiencies
52	E530	Nutritional deficiencies
52	E531	Nutritional deficiencies
52	E538	Nutritional deficiencies
52	E539	Nutritional deficiencies
52	E54	Nutritional deficiencies
52	E550	Nutritional deficiencies
52	E559	Nutritional deficiencies
52	E560	Nutritional deficiencies
52	E561	Nutritional deficiencies
52	E568	Nutritional deficiencies
52	E569	Nutritional deficiencies
52	E58	Nutritional deficiencies
52	E59	Nutritional deficiencies
52	E60	Nutritional deficiencies
52	E610	Nutritional deficiencies
52	E611	Nutritional deficiencies
52	E612	Nutritional deficiencies
52	E613	Nutritional deficiencies
52	E614	Nutritional deficiencies
52	E615	Nutritional deficiencies
52	E616	Nutritional deficiencies
52	E617	Nutritional deficiencies
52	E618	Nutritional deficiencies
52	E619	Nutritional deficiencies
52	E630	Nutritional deficiencies
52	E631	Nutritional deficiencies
52	E638	Nutritional deficiencies
52	E639	Nutritional deficiencies
52	E640	Nutritional deficiencies
52	E641	Nutritional deficiencies
52	E642	Nutritional deficiencies
52	E643	Nutritional deficiencies
52	E648	Nutritional deficiencies
52	E649	Nutritional deficiencies
52	M830	Nutritional deficiencies
52	M831	Nutritional deficiencies
52	M832	Nutritional deficiencies
52	M833	Nutritional deficiencies
52	M834	Nutritional deficiencies
52	M835	Nutritional deficiencies
52	M838	Nutritional deficiencies
52	M839	Nutritional deficiencies
52	R64	Nutritional deficiencies
53	E780	Disorders of lipid metabolism
53	E781	Disorders of lipid metabolism
53	E782	Disorders of lipid metabolism
53	E783	Disorders of lipid metabolism
53	E784	Disorders of lipid metabolism
53	E785	Disorders of lipid metabolism
54	M1000	Gout and other crystal arthropathies

54	M10011	Gout and other crystal arthropathies
54	M10012	Gout and other crystal arthropathies
54	M10019	Gout and other crystal arthropathies
54	M10021	Gout and other crystal arthropathies
54	M10022	Gout and other crystal arthropathies
54	M10029	Gout and other crystal arthropathies
54	M10031	Gout and other crystal arthropathies
54	M10032	Gout and other crystal arthropathies
54	M10039	Gout and other crystal arthropathies
54	M10041	Gout and other crystal arthropathies
54	M10042	Gout and other crystal arthropathies
54	M10049	Gout and other crystal arthropathies
54	M10051	Gout and other crystal arthropathies
54	M10052	Gout and other crystal arthropathies
54	M10059	Gout and other crystal arthropathies
54	M10061	Gout and other crystal arthropathies
54	M10062	Gout and other crystal arthropathies
54	M10069	Gout and other crystal arthropathies
54	M10071	Gout and other crystal arthropathies
54	M10072	Gout and other crystal arthropathies
54	M10079	Gout and other crystal arthropathies
54	M1008	Gout and other crystal arthropathies
54	M1009	Gout and other crystal arthropathies
54	M1010	Gout and other crystal arthropathies
54	M10111	Gout and other crystal arthropathies
54	M10112	Gout and other crystal arthropathies
54	M10119	Gout and other crystal arthropathies
54	M10121	Gout and other crystal arthropathies
54	M10122	Gout and other crystal arthropathies
54	M10129	Gout and other crystal arthropathies
54	M10131	Gout and other crystal arthropathies
54	M10132	Gout and other crystal arthropathies
54	M10139	Gout and other crystal arthropathies
54	M10141	Gout and other crystal arthropathies
54	M10142	Gout and other crystal arthropathies
54	M10149	Gout and other crystal arthropathies
54	M10151	Gout and other crystal arthropathies
54	M10152	Gout and other crystal arthropathies
54	M10159	Gout and other crystal arthropathies
54	M10161	Gout and other crystal arthropathies
54	M10162	Gout and other crystal arthropathies
54	M10169	Gout and other crystal arthropathies
54	M10171	Gout and other crystal arthropathies
54	M10172	Gout and other crystal arthropathies
54	M10179	Gout and other crystal arthropathies
54	M1018	Gout and other crystal arthropathies
54	M1019	Gout and other crystal arthropathies
54	M1020	Gout and other crystal arthropathies
54	M10211	Gout and other crystal arthropathies
54	M10212	Gout and other crystal arthropathies
54	M10219	Gout and other crystal arthropathies

54	M10221	Gout and other crystal arthropathies
54	M10222	Gout and other crystal arthropathies
54	M10229	Gout and other crystal arthropathies
54	M10231	Gout and other crystal arthropathies
54	M10232	Gout and other crystal arthropathies
54	M10239	Gout and other crystal arthropathies
54	M10241	Gout and other crystal arthropathies
54	M10242	Gout and other crystal arthropathies
54	M10249	Gout and other crystal arthropathies
54	M10251	Gout and other crystal arthropathies
54	M10252	Gout and other crystal arthropathies
54	M10259	Gout and other crystal arthropathies
54	M10261	Gout and other crystal arthropathies
54	M10262	Gout and other crystal arthropathies
54	M10269	Gout and other crystal arthropathies
54	M10271	Gout and other crystal arthropathies
54	M10272	Gout and other crystal arthropathies
54	M10279	Gout and other crystal arthropathies
54	M1028	Gout and other crystal arthropathies
54	M1029	Gout and other crystal arthropathies
54	M1030	Gout and other crystal arthropathies
54	M10311	Gout and other crystal arthropathies
54	M10312	Gout and other crystal arthropathies
54	M10319	Gout and other crystal arthropathies
54	M10321	Gout and other crystal arthropathies
54	M10322	Gout and other crystal arthropathies
54	M10329	Gout and other crystal arthropathies
54	M10331	Gout and other crystal arthropathies
54	M10332	Gout and other crystal arthropathies
54	M10339	Gout and other crystal arthropathies
54	M10341	Gout and other crystal arthropathies
54	M10342	Gout and other crystal arthropathies
54	M10349	Gout and other crystal arthropathies
54	M10351	Gout and other crystal arthropathies
54	M10352	Gout and other crystal arthropathies
54	M10359	Gout and other crystal arthropathies
54	M10361	Gout and other crystal arthropathies
54	M10362	Gout and other crystal arthropathies
54	M10369	Gout and other crystal arthropathies
54	M10371	Gout and other crystal arthropathies
54	M10372	Gout and other crystal arthropathies
54	M10379	Gout and other crystal arthropathies
54	M1038	Gout and other crystal arthropathies
54	M1039	Gout and other crystal arthropathies
54	M1040	Gout and other crystal arthropathies
54	M10411	Gout and other crystal arthropathies
54	M10412	Gout and other crystal arthropathies
54	M10419	Gout and other crystal arthropathies
54	M10421	Gout and other crystal arthropathies
54	M10422	Gout and other crystal arthropathies
54	M10429	Gout and other crystal arthropathies

54	M10431	Gout and other crystal arthropathies
54	M10432	Gout and other crystal arthropathies
54	M10439	Gout and other crystal arthropathies
54	M10441	Gout and other crystal arthropathies
54	M10442	Gout and other crystal arthropathies
54	M10449	Gout and other crystal arthropathies
54	M10451	Gout and other crystal arthropathies
54	M10452	Gout and other crystal arthropathies
54	M10459	Gout and other crystal arthropathies
54	M10461	Gout and other crystal arthropathies
54	M10462	Gout and other crystal arthropathies
54	M10469	Gout and other crystal arthropathies
54	M10471	Gout and other crystal arthropathies
54	M10472	Gout and other crystal arthropathies
54	M10479	Gout and other crystal arthropathies
54	M1048	Gout and other crystal arthropathies
54	M1049	Gout and other crystal arthropathies
54	M109	Gout and other crystal arthropathies
54	M1100	Gout and other crystal arthropathies
54	M11011	Gout and other crystal arthropathies
54	M11012	Gout and other crystal arthropathies
54	M11019	Gout and other crystal arthropathies
54	M11021	Gout and other crystal arthropathies
54	M11022	Gout and other crystal arthropathies
54	M11029	Gout and other crystal arthropathies
54	M11031	Gout and other crystal arthropathies
54	M11032	Gout and other crystal arthropathies
54	M11039	Gout and other crystal arthropathies
54	M11041	Gout and other crystal arthropathies
54	M11042	Gout and other crystal arthropathies
54	M11049	Gout and other crystal arthropathies
54	M11051	Gout and other crystal arthropathies
54	M11052	Gout and other crystal arthropathies
54	M11059	Gout and other crystal arthropathies
54	M11061	Gout and other crystal arthropathies
54	M11062	Gout and other crystal arthropathies
54	M11069	Gout and other crystal arthropathies
54	M11071	Gout and other crystal arthropathies
54	M11072	Gout and other crystal arthropathies
54	M11079	Gout and other crystal arthropathies
54	M1108	Gout and other crystal arthropathies
54	M1109	Gout and other crystal arthropathies
54	M1110	Gout and other crystal arthropathies
54	M11111	Gout and other crystal arthropathies
54	M11112	Gout and other crystal arthropathies
54	M11119	Gout and other crystal arthropathies
54	M11121	Gout and other crystal arthropathies
54	M11122	Gout and other crystal arthropathies
54	M11129	Gout and other crystal arthropathies
54	M11131	Gout and other crystal arthropathies
54	M11132	Gout and other crystal arthropathies

54	M11139	Gout and other crystal arthropathies
54	M11141	Gout and other crystal arthropathies
54	M11142	Gout and other crystal arthropathies
54	M11149	Gout and other crystal arthropathies
54	M11151	Gout and other crystal arthropathies
54	M11152	Gout and other crystal arthropathies
54	M11159	Gout and other crystal arthropathies
54	M11161	Gout and other crystal arthropathies
54	M11162	Gout and other crystal arthropathies
54	M11169	Gout and other crystal arthropathies
54	M11171	Gout and other crystal arthropathies
54	M11172	Gout and other crystal arthropathies
54	M11179	Gout and other crystal arthropathies
54	M1118	Gout and other crystal arthropathies
54	M1119	Gout and other crystal arthropathies
54	M1120	Gout and other crystal arthropathies
54	M11211	Gout and other crystal arthropathies
54	M11212	Gout and other crystal arthropathies
54	M11219	Gout and other crystal arthropathies
54	M11221	Gout and other crystal arthropathies
54	M11222	Gout and other crystal arthropathies
54	M11229	Gout and other crystal arthropathies
54	M11231	Gout and other crystal arthropathies
54	M11232	Gout and other crystal arthropathies
54	M11239	Gout and other crystal arthropathies
54	M11241	Gout and other crystal arthropathies
54	M11242	Gout and other crystal arthropathies
54	M11249	Gout and other crystal arthropathies
54	M11251	Gout and other crystal arthropathies
54	M11252	Gout and other crystal arthropathies
54	M11259	Gout and other crystal arthropathies
54	M11261	Gout and other crystal arthropathies
54	M11262	Gout and other crystal arthropathies
54	M11269	Gout and other crystal arthropathies
54	M11271	Gout and other crystal arthropathies
54	M11272	Gout and other crystal arthropathies
54	M11279	Gout and other crystal arthropathies
54	M1128	Gout and other crystal arthropathies
54	M1129	Gout and other crystal arthropathies
54	M1180	Gout and other crystal arthropathies
54	M11811	Gout and other crystal arthropathies
54	M11812	Gout and other crystal arthropathies
54	M11819	Gout and other crystal arthropathies
54	M11821	Gout and other crystal arthropathies
54	M11822	Gout and other crystal arthropathies
54	M11829	Gout and other crystal arthropathies
54	M11831	Gout and other crystal arthropathies
54	M11832	Gout and other crystal arthropathies
54	M11839	Gout and other crystal arthropathies
54	M11841	Gout and other crystal arthropathies
54	M11842	Gout and other crystal arthropathies

54	M11849	Gout and other crystal arthropathies
54	M11851	Gout and other crystal arthropathies
54	M11852	Gout and other crystal arthropathies
54	M11859	Gout and other crystal arthropathies
54	M11861	Gout and other crystal arthropathies
54	M11862	Gout and other crystal arthropathies
54	M11869	Gout and other crystal arthropathies
54	M11871	Gout and other crystal arthropathies
54	M11872	Gout and other crystal arthropathies
54	M11879	Gout and other crystal arthropathies
54	M1188	Gout and other crystal arthropathies
54	M1189	Gout and other crystal arthropathies
54	M119	Gout and other crystal arthropathies
54	M1A00X0	Gout and other crystal arthropathies
54	M1A00X1	Gout and other crystal arthropathies
54	M1A0110	Gout and other crystal arthropathies
54	M1A0111	Gout and other crystal arthropathies
54	M1A0120	Gout and other crystal arthropathies
54	M1A0121	Gout and other crystal arthropathies
54	M1A0190	Gout and other crystal arthropathies
54	M1A0191	Gout and other crystal arthropathies
54	M1A0210	Gout and other crystal arthropathies
54	M1A0211	Gout and other crystal arthropathies
54	M1A0220	Gout and other crystal arthropathies
54	M1A0221	Gout and other crystal arthropathies
54	M1A0290	Gout and other crystal arthropathies
54	M1A0291	Gout and other crystal arthropathies
54	M1A0310	Gout and other crystal arthropathies
54	M1A0311	Gout and other crystal arthropathies
54	M1A0320	Gout and other crystal arthropathies
54	M1A0321	Gout and other crystal arthropathies
54	M1A0390	Gout and other crystal arthropathies
54	M1A0391	Gout and other crystal arthropathies
54	M1A0410	Gout and other crystal arthropathies
54	M1A0411	Gout and other crystal arthropathies
54	M1A0420	Gout and other crystal arthropathies
54	M1A0421	Gout and other crystal arthropathies
54	M1A0490	Gout and other crystal arthropathies
54	M1A0491	Gout and other crystal arthropathies
54	M1A0510	Gout and other crystal arthropathies
54	M1A0511	Gout and other crystal arthropathies
54	M1A0520	Gout and other crystal arthropathies
54	M1A0521	Gout and other crystal arthropathies
54	M1A0590	Gout and other crystal arthropathies
54	M1A0591	Gout and other crystal arthropathies
54	M1A0610	Gout and other crystal arthropathies
54	M1A0611	Gout and other crystal arthropathies
54	M1A0620	Gout and other crystal arthropathies
54	M1A0621	Gout and other crystal arthropathies
54	M1A0690	Gout and other crystal arthropathies
54	M1A0691	Gout and other crystal arthropathies

54	M1A0710	Gout and other crystal arthropathies
54	M1A0711	Gout and other crystal arthropathies
54	M1A0720	Gout and other crystal arthropathies
54	M1A0721	Gout and other crystal arthropathies
54	M1A0790	Gout and other crystal arthropathies
54	M1A0791	Gout and other crystal arthropathies
54	M1A08X0	Gout and other crystal arthropathies
54	M1A08X1	Gout and other crystal arthropathies
54	M1A09X0	Gout and other crystal arthropathies
54	M1A09X1	Gout and other crystal arthropathies
54	M1A10X0	Gout and other crystal arthropathies
54	M1A10X1	Gout and other crystal arthropathies
54	M1A1110	Gout and other crystal arthropathies
54	M1A1111	Gout and other crystal arthropathies
54	M1A1120	Gout and other crystal arthropathies
54	M1A1121	Gout and other crystal arthropathies
54	M1A1190	Gout and other crystal arthropathies
54	M1A1191	Gout and other crystal arthropathies
54	M1A1210	Gout and other crystal arthropathies
54	M1A1211	Gout and other crystal arthropathies
54	M1A1220	Gout and other crystal arthropathies
54	M1A1221	Gout and other crystal arthropathies
54	M1A1290	Gout and other crystal arthropathies
54	M1A1291	Gout and other crystal arthropathies
54	M1A1310	Gout and other crystal arthropathies
54	M1A1311	Gout and other crystal arthropathies
54	M1A1320	Gout and other crystal arthropathies
54	M1A1321	Gout and other crystal arthropathies
54	M1A1390	Gout and other crystal arthropathies
54	M1A1391	Gout and other crystal arthropathies
54	M1A1410	Gout and other crystal arthropathies
54	M1A1411	Gout and other crystal arthropathies
54	M1A1420	Gout and other crystal arthropathies
54	M1A1421	Gout and other crystal arthropathies
54	M1A1490	Gout and other crystal arthropathies
54	M1A1491	Gout and other crystal arthropathies
54	M1A1510	Gout and other crystal arthropathies
54	M1A1511	Gout and other crystal arthropathies
54	M1A1520	Gout and other crystal arthropathies
54	M1A1521	Gout and other crystal arthropathies
54	M1A1590	Gout and other crystal arthropathies
54	M1A1591	Gout and other crystal arthropathies
54	M1A1610	Gout and other crystal arthropathies
54	M1A1611	Gout and other crystal arthropathies
54	M1A1620	Gout and other crystal arthropathies
54	M1A1621	Gout and other crystal arthropathies
54	M1A1690	Gout and other crystal arthropathies
54	M1A1691	Gout and other crystal arthropathies
54	M1A1710	Gout and other crystal arthropathies
54	M1A1711	Gout and other crystal arthropathies
54	M1A1720	Gout and other crystal arthropathies

54	M1A1721	Gout and other crystal arthropathies
54	M1A1790	Gout and other crystal arthropathies
54	M1A1791	Gout and other crystal arthropathies
54	M1A18X0	Gout and other crystal arthropathies
54	M1A18X1	Gout and other crystal arthropathies
54	M1A19X0	Gout and other crystal arthropathies
54	M1A19X1	Gout and other crystal arthropathies
54	M1A20X0	Gout and other crystal arthropathies
54	M1A20X1	Gout and other crystal arthropathies
54	M1A2110	Gout and other crystal arthropathies
54	M1A2111	Gout and other crystal arthropathies
54	M1A2120	Gout and other crystal arthropathies
54	M1A2121	Gout and other crystal arthropathies
54	M1A2190	Gout and other crystal arthropathies
54	M1A2191	Gout and other crystal arthropathies
54	M1A2210	Gout and other crystal arthropathies
54	M1A2211	Gout and other crystal arthropathies
54	M1A2220	Gout and other crystal arthropathies
54	M1A2221	Gout and other crystal arthropathies
54	M1A2290	Gout and other crystal arthropathies
54	M1A2291	Gout and other crystal arthropathies
54	M1A2310	Gout and other crystal arthropathies
54	M1A2311	Gout and other crystal arthropathies
54	M1A2320	Gout and other crystal arthropathies
54	M1A2321	Gout and other crystal arthropathies
54	M1A2390	Gout and other crystal arthropathies
54	M1A2391	Gout and other crystal arthropathies
54	M1A2410	Gout and other crystal arthropathies
54	M1A2411	Gout and other crystal arthropathies
54	M1A2420	Gout and other crystal arthropathies
54	M1A2421	Gout and other crystal arthropathies
54	M1A2490	Gout and other crystal arthropathies
54	M1A2491	Gout and other crystal arthropathies
54	M1A2510	Gout and other crystal arthropathies
54	M1A2511	Gout and other crystal arthropathies
54	M1A2520	Gout and other crystal arthropathies
54	M1A2521	Gout and other crystal arthropathies
54	M1A2590	Gout and other crystal arthropathies
54	M1A2591	Gout and other crystal arthropathies
54	M1A2610	Gout and other crystal arthropathies
54	M1A2611	Gout and other crystal arthropathies
54	M1A2620	Gout and other crystal arthropathies
54	M1A2621	Gout and other crystal arthropathies
54	M1A2690	Gout and other crystal arthropathies
54	M1A2691	Gout and other crystal arthropathies
54	M1A2710	Gout and other crystal arthropathies
54	M1A2711	Gout and other crystal arthropathies
54	M1A2720	Gout and other crystal arthropathies
54	M1A2721	Gout and other crystal arthropathies
54	M1A2790	Gout and other crystal arthropathies
54	M1A2791	Gout and other crystal arthropathies

54	M1A28X0	Gout and other crystal arthropathies
54	M1A28X1	Gout and other crystal arthropathies
54	M1A29X0	Gout and other crystal arthropathies
54	M1A29X1	Gout and other crystal arthropathies
54	M1A30X0	Gout and other crystal arthropathies
54	M1A30X1	Gout and other crystal arthropathies
54	M1A3110	Gout and other crystal arthropathies
54	M1A3111	Gout and other crystal arthropathies
54	M1A3120	Gout and other crystal arthropathies
54	M1A3121	Gout and other crystal arthropathies
54	M1A3190	Gout and other crystal arthropathies
54	M1A3191	Gout and other crystal arthropathies
54	M1A3210	Gout and other crystal arthropathies
54	M1A3211	Gout and other crystal arthropathies
54	M1A3220	Gout and other crystal arthropathies
54	M1A3221	Gout and other crystal arthropathies
54	M1A3290	Gout and other crystal arthropathies
54	M1A3291	Gout and other crystal arthropathies
54	M1A3310	Gout and other crystal arthropathies
54	M1A3311	Gout and other crystal arthropathies
54	M1A3320	Gout and other crystal arthropathies
54	M1A3321	Gout and other crystal arthropathies
54	M1A3390	Gout and other crystal arthropathies
54	M1A3391	Gout and other crystal arthropathies
54	M1A3410	Gout and other crystal arthropathies
54	M1A3411	Gout and other crystal arthropathies
54	M1A3420	Gout and other crystal arthropathies
54	M1A3421	Gout and other crystal arthropathies
54	M1A3490	Gout and other crystal arthropathies
54	M1A3491	Gout and other crystal arthropathies
54	M1A3510	Gout and other crystal arthropathies
54	M1A3511	Gout and other crystal arthropathies
54	M1A3520	Gout and other crystal arthropathies
54	M1A3521	Gout and other crystal arthropathies
54	M1A3590	Gout and other crystal arthropathies
54	M1A3591	Gout and other crystal arthropathies
54	M1A3610	Gout and other crystal arthropathies
54	M1A3611	Gout and other crystal arthropathies
54	M1A3620	Gout and other crystal arthropathies
54	M1A3621	Gout and other crystal arthropathies
54	M1A3690	Gout and other crystal arthropathies
54	M1A3691	Gout and other crystal arthropathies
54	M1A3710	Gout and other crystal arthropathies
54	M1A3711	Gout and other crystal arthropathies
54	M1A3720	Gout and other crystal arthropathies
54	M1A3721	Gout and other crystal arthropathies
54	M1A3790	Gout and other crystal arthropathies
54	M1A3791	Gout and other crystal arthropathies
54	M1A38X0	Gout and other crystal arthropathies
54	M1A38X1	Gout and other crystal arthropathies
54	M1A39X0	Gout and other crystal arthropathies

54	M1A39X1	Gout and other crystal arthropathies
54	M1A40X0	Gout and other crystal arthropathies
54	M1A40X1	Gout and other crystal arthropathies
54	M1A4110	Gout and other crystal arthropathies
54	M1A4111	Gout and other crystal arthropathies
54	M1A4120	Gout and other crystal arthropathies
54	M1A4121	Gout and other crystal arthropathies
54	M1A4190	Gout and other crystal arthropathies
54	M1A4191	Gout and other crystal arthropathies
54	M1A4210	Gout and other crystal arthropathies
54	M1A4211	Gout and other crystal arthropathies
54	M1A4220	Gout and other crystal arthropathies
54	M1A4221	Gout and other crystal arthropathies
54	M1A4290	Gout and other crystal arthropathies
54	M1A4291	Gout and other crystal arthropathies
54	M1A4310	Gout and other crystal arthropathies
54	M1A4311	Gout and other crystal arthropathies
54	M1A4320	Gout and other crystal arthropathies
54	M1A4321	Gout and other crystal arthropathies
54	M1A4390	Gout and other crystal arthropathies
54	M1A4391	Gout and other crystal arthropathies
54	M1A4410	Gout and other crystal arthropathies
54	M1A4411	Gout and other crystal arthropathies
54	M1A4420	Gout and other crystal arthropathies
54	M1A4421	Gout and other crystal arthropathies
54	M1A4490	Gout and other crystal arthropathies
54	M1A4491	Gout and other crystal arthropathies
54	M1A4510	Gout and other crystal arthropathies
54	M1A4511	Gout and other crystal arthropathies
54	M1A4520	Gout and other crystal arthropathies
54	M1A4521	Gout and other crystal arthropathies
54	M1A4590	Gout and other crystal arthropathies
54	M1A4591	Gout and other crystal arthropathies
54	M1A4610	Gout and other crystal arthropathies
54	M1A4611	Gout and other crystal arthropathies
54	M1A4620	Gout and other crystal arthropathies
54	M1A4621	Gout and other crystal arthropathies
54	M1A4690	Gout and other crystal arthropathies
54	M1A4691	Gout and other crystal arthropathies
54	M1A4710	Gout and other crystal arthropathies
54	M1A4711	Gout and other crystal arthropathies
54	M1A4720	Gout and other crystal arthropathies
54	M1A4721	Gout and other crystal arthropathies
54	M1A4790	Gout and other crystal arthropathies
54	M1A4791	Gout and other crystal arthropathies
54	M1A48X0	Gout and other crystal arthropathies
54	M1A48X1	Gout and other crystal arthropathies
54	M1A49X0	Gout and other crystal arthropathies
54	M1A49X1	Gout and other crystal arthropathies
54	M1A9XX0	Gout and other crystal arthropathies
54	M1A9XX1	Gout and other crystal arthropathies

55	E860	Fluid and electrolyte disorders
55	E861	Fluid and electrolyte disorders
55	E869	Fluid and electrolyte disorders
55	E870	Fluid and electrolyte disorders
55	E871	Fluid and electrolyte disorders
55	E872	Fluid and electrolyte disorders
55	E873	Fluid and electrolyte disorders
55	E874	Fluid and electrolyte disorders
55	E875	Fluid and electrolyte disorders
55	E876	Fluid and electrolyte disorders
55	E8770	Fluid and electrolyte disorders
55	E8779	Fluid and electrolyte disorders
55	E878	Fluid and electrolyte disorders
56	E840	Cystic fibrosis
56	E8411	Cystic fibrosis
56	E8419	Cystic fibrosis
56	E848	Cystic fibrosis
56	E849	Cystic fibrosis
57	D800	Immunity disorders
57	D801	Immunity disorders
57	D802	Immunity disorders
57	D803	Immunity disorders
57	D804	Immunity disorders
57	D805	Immunity disorders
57	D806	Immunity disorders
57	D807	Immunity disorders
57	D808	Immunity disorders
57	D809	Immunity disorders
57	D810	Immunity disorders
57	D811	Immunity disorders
57	D812	Immunity disorders
57	D814	Immunity disorders
57	D816	Immunity disorders
57	D817	Immunity disorders
57	D8189	Immunity disorders
57	D819	Immunity disorders
57	D820	Immunity disorders
57	D821	Immunity disorders
57	D822	Immunity disorders
57	D823	Immunity disorders
57	D824	Immunity disorders
57	D828	Immunity disorders
57	D829	Immunity disorders
57	D830	Immunity disorders
57	D831	Immunity disorders
57	D832	Immunity disorders
57	D838	Immunity disorders
57	D839	Immunity disorders
57	D840	Immunity disorders
57	D841	Immunity disorders
57	D848	Immunity disorders

57	D849	Immunity disorders
57	D860	Immunity disorders
57	D861	Immunity disorders
57	D862	Immunity disorders
57	D863	Immunity disorders
57	D8682	Immunity disorders
57	D8683	Immunity disorders
57	D8684	Immunity disorders
57	D8685	Immunity disorders
57	D8686	Immunity disorders
57	D8687	Immunity disorders
57	D8689	Immunity disorders
57	D869	Immunity disorders
57	D890	Immunity disorders
57	D891	Immunity disorders
57	D892	Immunity disorders
57	D893	Immunity disorders
57	D8982	Immunity disorders
57	D8989	Immunity disorders
57	D899	Immunity disorders
58	D813	Other nutritional; endocrine; and metabolic disorders
58	D815	Other nutritional; endocrine; and metabolic disorders
58	D81810	Other nutritional; endocrine; and metabolic disorders
58	E201	Other nutritional; endocrine; and metabolic disorders
58	E65	Other nutritional; endocrine; and metabolic disorders
58	E6601	Other nutritional; endocrine; and metabolic disorders
58	E6609	Other nutritional; endocrine; and metabolic disorders
58	E661	Other nutritional; endocrine; and metabolic disorders
58	E662	Other nutritional; endocrine; and metabolic disorders
58	E663	Other nutritional; endocrine; and metabolic disorders
58	E668	Other nutritional; endocrine; and metabolic disorders
58	E669	Other nutritional; endocrine; and metabolic disorders
58	E670	Other nutritional; endocrine; and metabolic disorders
58	E671	Other nutritional; endocrine; and metabolic disorders
58	E672	Other nutritional; endocrine; and metabolic disorders
58	E673	Other nutritional; endocrine; and metabolic disorders
58	E678	Other nutritional; endocrine; and metabolic disorders
58	E68	Other nutritional; endocrine; and metabolic disorders
58	E700	Other nutritional; endocrine; and metabolic disorders
58	E701	Other nutritional; endocrine; and metabolic disorders
58	E7020	Other nutritional; endocrine; and metabolic disorders
58	E7021	Other nutritional; endocrine; and metabolic disorders
58	E7029	Other nutritional; endocrine; and metabolic disorders
58	E7030	Other nutritional; endocrine; and metabolic disorders
58	E70310	Other nutritional; endocrine; and metabolic disorders
58	E70311	Other nutritional; endocrine; and metabolic disorders
58	E70318	Other nutritional; endocrine; and metabolic disorders
58	E70319	Other nutritional; endocrine; and metabolic disorders
58	E70320	Other nutritional; endocrine; and metabolic disorders
58	E70321	Other nutritional; endocrine; and metabolic disorders
58	E70328	Other nutritional; endocrine; and metabolic disorders

58	E70329	Other nutritional; endocrine; and metabolic disorders
58	E70330	Other nutritional; endocrine; and metabolic disorders
58	E70331	Other nutritional; endocrine; and metabolic disorders
58	E70338	Other nutritional; endocrine; and metabolic disorders
58	E70339	Other nutritional; endocrine; and metabolic disorders
58	E7039	Other nutritional; endocrine; and metabolic disorders
58	E7040	Other nutritional; endocrine; and metabolic disorders
58	E7041	Other nutritional; endocrine; and metabolic disorders
58	E7049	Other nutritional; endocrine; and metabolic disorders
58	E705	Other nutritional; endocrine; and metabolic disorders
58	E708	Other nutritional; endocrine; and metabolic disorders
58	E709	Other nutritional; endocrine; and metabolic disorders
58	E710	Other nutritional; endocrine; and metabolic disorders
58	E71110	Other nutritional; endocrine; and metabolic disorders
58	E71111	Other nutritional; endocrine; and metabolic disorders
58	E71118	Other nutritional; endocrine; and metabolic disorders
58	E71120	Other nutritional; endocrine; and metabolic disorders
58	E71121	Other nutritional; endocrine; and metabolic disorders
58	E71128	Other nutritional; endocrine; and metabolic disorders
58	E7119	Other nutritional; endocrine; and metabolic disorders
58	E712	Other nutritional; endocrine; and metabolic disorders
58	E7130	Other nutritional; endocrine; and metabolic disorders
58	E71310	Other nutritional; endocrine; and metabolic disorders
58	E71311	Other nutritional; endocrine; and metabolic disorders
58	E71312	Other nutritional; endocrine; and metabolic disorders
58	E71313	Other nutritional; endocrine; and metabolic disorders
58	E71314	Other nutritional; endocrine; and metabolic disorders
58	E71318	Other nutritional; endocrine; and metabolic disorders
58	E7132	Other nutritional; endocrine; and metabolic disorders
58	E7139	Other nutritional; endocrine; and metabolic disorders
58	E7140	Other nutritional; endocrine; and metabolic disorders
58	E7141	Other nutritional; endocrine; and metabolic disorders
58	E7142	Other nutritional; endocrine; and metabolic disorders
58	E71440	Other nutritional; endocrine; and metabolic disorders
58	E71448	Other nutritional; endocrine; and metabolic disorders
58	E7150	Other nutritional; endocrine; and metabolic disorders
58	E71510	Other nutritional; endocrine; and metabolic disorders
58	E71511	Other nutritional; endocrine; and metabolic disorders
58	E71518	Other nutritional; endocrine; and metabolic disorders
58	E71520	Other nutritional; endocrine; and metabolic disorders
58	E71521	Other nutritional; endocrine; and metabolic disorders
58	E71522	Other nutritional; endocrine; and metabolic disorders
58	E71528	Other nutritional; endocrine; and metabolic disorders
58	E71529	Other nutritional; endocrine; and metabolic disorders
58	E7153	Other nutritional; endocrine; and metabolic disorders
58	E71540	Other nutritional; endocrine; and metabolic disorders
58	E71541	Other nutritional; endocrine; and metabolic disorders
58	E71542	Other nutritional; endocrine; and metabolic disorders
58	E71548	Other nutritional; endocrine; and metabolic disorders
58	E7200	Other nutritional; endocrine; and metabolic disorders
58	E7201	Other nutritional; endocrine; and metabolic disorders

58	E7202	Other nutritional; endocrine; and metabolic disorders
58	E7203	Other nutritional; endocrine; and metabolic disorders
58	E7204	Other nutritional; endocrine; and metabolic disorders
58	E7209	Other nutritional; endocrine; and metabolic disorders
58	E7210	Other nutritional; endocrine; and metabolic disorders
58	E7211	Other nutritional; endocrine; and metabolic disorders
58	E7212	Other nutritional; endocrine; and metabolic disorders
58	E7219	Other nutritional; endocrine; and metabolic disorders
58	E7220	Other nutritional; endocrine; and metabolic disorders
58	E7221	Other nutritional; endocrine; and metabolic disorders
58	E7222	Other nutritional; endocrine; and metabolic disorders
58	E7223	Other nutritional; endocrine; and metabolic disorders
58	E7229	Other nutritional; endocrine; and metabolic disorders
58	E723	Other nutritional; endocrine; and metabolic disorders
58	E724	Other nutritional; endocrine; and metabolic disorders
58	E7250	Other nutritional; endocrine; and metabolic disorders
58	E7251	Other nutritional; endocrine; and metabolic disorders
58	E7252	Other nutritional; endocrine; and metabolic disorders
58	E7253	Other nutritional; endocrine; and metabolic disorders
58	E7259	Other nutritional; endocrine; and metabolic disorders
58	E728	Other nutritional; endocrine; and metabolic disorders
58	E729	Other nutritional; endocrine; and metabolic disorders
58	E730	Other nutritional; endocrine; and metabolic disorders
58	E731	Other nutritional; endocrine; and metabolic disorders
58	E738	Other nutritional; endocrine; and metabolic disorders
58	E739	Other nutritional; endocrine; and metabolic disorders
58	E7400	Other nutritional; endocrine; and metabolic disorders
58	E7401	Other nutritional; endocrine; and metabolic disorders
58	E7402	Other nutritional; endocrine; and metabolic disorders
58	E7403	Other nutritional; endocrine; and metabolic disorders
58	E7404	Other nutritional; endocrine; and metabolic disorders
58	E7409	Other nutritional; endocrine; and metabolic disorders
58	E7410	Other nutritional; endocrine; and metabolic disorders
58	E7411	Other nutritional; endocrine; and metabolic disorders
58	E7412	Other nutritional; endocrine; and metabolic disorders
58	E7419	Other nutritional; endocrine; and metabolic disorders
58	E7420	Other nutritional; endocrine; and metabolic disorders
58	E7421	Other nutritional; endocrine; and metabolic disorders
58	E7429	Other nutritional; endocrine; and metabolic disorders
58	E7431	Other nutritional; endocrine; and metabolic disorders
58	E7439	Other nutritional; endocrine; and metabolic disorders
58	E744	Other nutritional; endocrine; and metabolic disorders
58	E748	Other nutritional; endocrine; and metabolic disorders
58	E749	Other nutritional; endocrine; and metabolic disorders
58	E7521	Other nutritional; endocrine; and metabolic disorders
58	E7522	Other nutritional; endocrine; and metabolic disorders
58	E75240	Other nutritional; endocrine; and metabolic disorders
58	E75241	Other nutritional; endocrine; and metabolic disorders
58	E75242	Other nutritional; endocrine; and metabolic disorders
58	E75243	Other nutritional; endocrine; and metabolic disorders
58	E75248	Other nutritional; endocrine; and metabolic disorders

58	E75249	Other nutritional; endocrine; and metabolic disorders
58	E753	Other nutritional; endocrine; and metabolic disorders
58	E755	Other nutritional; endocrine; and metabolic disorders
58	E756	Other nutritional; endocrine; and metabolic disorders
58	E7601	Other nutritional; endocrine; and metabolic disorders
58	E7602	Other nutritional; endocrine; and metabolic disorders
58	E7603	Other nutritional; endocrine; and metabolic disorders
58	E761	Other nutritional; endocrine; and metabolic disorders
58	E76210	Other nutritional; endocrine; and metabolic disorders
58	E76211	Other nutritional; endocrine; and metabolic disorders
58	E76219	Other nutritional; endocrine; and metabolic disorders
58	E7622	Other nutritional; endocrine; and metabolic disorders
58	E7629	Other nutritional; endocrine; and metabolic disorders
58	E763	Other nutritional; endocrine; and metabolic disorders
58	E768	Other nutritional; endocrine; and metabolic disorders
58	E769	Other nutritional; endocrine; and metabolic disorders
58	E770	Other nutritional; endocrine; and metabolic disorders
58	E771	Other nutritional; endocrine; and metabolic disorders
58	E778	Other nutritional; endocrine; and metabolic disorders
58	E779	Other nutritional; endocrine; and metabolic disorders
58	E786	Other nutritional; endocrine; and metabolic disorders
58	E7870	Other nutritional; endocrine; and metabolic disorders
58	E7879	Other nutritional; endocrine; and metabolic disorders
58	E7881	Other nutritional; endocrine; and metabolic disorders
58	E7889	Other nutritional; endocrine; and metabolic disorders
58	E789	Other nutritional; endocrine; and metabolic disorders
58	E790	Other nutritional; endocrine; and metabolic disorders
58	E791	Other nutritional; endocrine; and metabolic disorders
58	E792	Other nutritional; endocrine; and metabolic disorders
58	E798	Other nutritional; endocrine; and metabolic disorders
58	E799	Other nutritional; endocrine; and metabolic disorders
58	E800	Other nutritional; endocrine; and metabolic disorders
58	E801	Other nutritional; endocrine; and metabolic disorders
58	E8020	Other nutritional; endocrine; and metabolic disorders
58	E8021	Other nutritional; endocrine; and metabolic disorders
58	E8029	Other nutritional; endocrine; and metabolic disorders
58	E803	Other nutritional; endocrine; and metabolic disorders
58	E804	Other nutritional; endocrine; and metabolic disorders
58	E805	Other nutritional; endocrine; and metabolic disorders
58	E806	Other nutritional; endocrine; and metabolic disorders
58	E807	Other nutritional; endocrine; and metabolic disorders
58	E8300	Other nutritional; endocrine; and metabolic disorders
58	E8301	Other nutritional; endocrine; and metabolic disorders
58	E8309	Other nutritional; endocrine; and metabolic disorders
58	E8310	Other nutritional; endocrine; and metabolic disorders
58	E83110	Other nutritional; endocrine; and metabolic disorders
58	E83111	Other nutritional; endocrine; and metabolic disorders
58	E83118	Other nutritional; endocrine; and metabolic disorders
58	E83119	Other nutritional; endocrine; and metabolic disorders
58	E8319	Other nutritional; endocrine; and metabolic disorders
58	E832	Other nutritional; endocrine; and metabolic disorders

58	E8330	Other nutritional; endocrine; and metabolic disorders
58	E8331	Other nutritional; endocrine; and metabolic disorders
58	E8332	Other nutritional; endocrine; and metabolic disorders
58	E8339	Other nutritional; endocrine; and metabolic disorders
58	E8340	Other nutritional; endocrine; and metabolic disorders
58	E8341	Other nutritional; endocrine; and metabolic disorders
58	E8342	Other nutritional; endocrine; and metabolic disorders
58	E8349	Other nutritional; endocrine; and metabolic disorders
58	E8350	Other nutritional; endocrine; and metabolic disorders
58	E8351	Other nutritional; endocrine; and metabolic disorders
58	E8352	Other nutritional; endocrine; and metabolic disorders
58	E8359	Other nutritional; endocrine; and metabolic disorders
58	E8381	Other nutritional; endocrine; and metabolic disorders
58	E8389	Other nutritional; endocrine; and metabolic disorders
58	E839	Other nutritional; endocrine; and metabolic disorders
58	E850	Other nutritional; endocrine; and metabolic disorders
58	E851	Other nutritional; endocrine; and metabolic disorders
58	E852	Other nutritional; endocrine; and metabolic disorders
58	E853	Other nutritional; endocrine; and metabolic disorders
58	E854	Other nutritional; endocrine; and metabolic disorders
58	E858	Other nutritional; endocrine; and metabolic disorders
58	E859	Other nutritional; endocrine; and metabolic disorders
58	E8801	Other nutritional; endocrine; and metabolic disorders
58	E8809	Other nutritional; endocrine; and metabolic disorders
58	E881	Other nutritional; endocrine; and metabolic disorders
58	E882	Other nutritional; endocrine; and metabolic disorders
58	E8840	Other nutritional; endocrine; and metabolic disorders
58	E8841	Other nutritional; endocrine; and metabolic disorders
58	E8842	Other nutritional; endocrine; and metabolic disorders
58	E8849	Other nutritional; endocrine; and metabolic disorders
58	E8881	Other nutritional; endocrine; and metabolic disorders
58	E8889	Other nutritional; endocrine; and metabolic disorders
58	E889	Other nutritional; endocrine; and metabolic disorders
58	R620	Other nutritional; endocrine; and metabolic disorders
58	R6250	Other nutritional; endocrine; and metabolic disorders
58	R6251	Other nutritional; endocrine; and metabolic disorders
58	R6252	Other nutritional; endocrine; and metabolic disorders
58	R6259	Other nutritional; endocrine; and metabolic disorders
58	R627	Other nutritional; endocrine; and metabolic disorders
58	R631	Other nutritional; endocrine; and metabolic disorders
58	R633	Other nutritional; endocrine; and metabolic disorders
58	R634	Other nutritional; endocrine; and metabolic disorders
58	R635	Other nutritional; endocrine; and metabolic disorders
58	R636	Other nutritional; endocrine; and metabolic disorders
58	R638	Other nutritional; endocrine; and metabolic disorders
58	Z681	Other nutritional; endocrine; and metabolic disorders
58	Z6825	Other nutritional; endocrine; and metabolic disorders
58	Z6826	Other nutritional; endocrine; and metabolic disorders
58	Z6827	Other nutritional; endocrine; and metabolic disorders
58	Z6828	Other nutritional; endocrine; and metabolic disorders
58	Z6829	Other nutritional; endocrine; and metabolic disorders

58	Z6830	Other nutritional; endocrine; and metabolic disorders
58	Z6831	Other nutritional; endocrine; and metabolic disorders
58	Z6832	Other nutritional; endocrine; and metabolic disorders
58	Z6833	Other nutritional; endocrine; and metabolic disorders
58	Z6834	Other nutritional; endocrine; and metabolic disorders
58	Z6835	Other nutritional; endocrine; and metabolic disorders
58	Z6836	Other nutritional; endocrine; and metabolic disorders
58	Z6837	Other nutritional; endocrine; and metabolic disorders
58	Z6838	Other nutritional; endocrine; and metabolic disorders
58	Z6839	Other nutritional; endocrine; and metabolic disorders
58	Z6841	Other nutritional; endocrine; and metabolic disorders
58	Z6842	Other nutritional; endocrine; and metabolic disorders
58	Z6843	Other nutritional; endocrine; and metabolic disorders
58	Z6844	Other nutritional; endocrine; and metabolic disorders
58	Z6845	Other nutritional; endocrine; and metabolic disorders
58	Z6851	Other nutritional; endocrine; and metabolic disorders
58	Z6853	Other nutritional; endocrine; and metabolic disorders
58	Z6854	Other nutritional; endocrine; and metabolic disorders
58	Z8639	Other nutritional; endocrine; and metabolic disorders
59	D500	Deficiency and other anemia
59	D501	Deficiency and other anemia
59	D508	Deficiency and other anemia
59	D509	Deficiency and other anemia
59	D510	Deficiency and other anemia
59	D511	Deficiency and other anemia
59	D512	Deficiency and other anemia
59	D513	Deficiency and other anemia
59	D518	Deficiency and other anemia
59	D519	Deficiency and other anemia
59	D520	Deficiency and other anemia
59	D521	Deficiency and other anemia
59	D528	Deficiency and other anemia
59	D529	Deficiency and other anemia
59	D530	Deficiency and other anemia
59	D531	Deficiency and other anemia
59	D532	Deficiency and other anemia
59	D538	Deficiency and other anemia
59	D539	Deficiency and other anemia
59	D550	Deficiency and other anemia
59	D551	Deficiency and other anemia
59	D552	Deficiency and other anemia
59	D553	Deficiency and other anemia
59	D558	Deficiency and other anemia
59	D559	Deficiency and other anemia
59	D560	Deficiency and other anemia
59	D561	Deficiency and other anemia
59	D562	Deficiency and other anemia
59	D563	Deficiency and other anemia
59	D564	Deficiency and other anemia
59	D565	Deficiency and other anemia
59	D568	Deficiency and other anemia

59	D569	Deficiency and other anemia
59	D580	Deficiency and other anemia
59	D581	Deficiency and other anemia
59	D582	Deficiency and other anemia
59	D588	Deficiency and other anemia
59	D589	Deficiency and other anemia
59	D590	Deficiency and other anemia
59	D591	Deficiency and other anemia
59	D592	Deficiency and other anemia
59	D593	Deficiency and other anemia
59	D594	Deficiency and other anemia
59	D595	Deficiency and other anemia
59	D596	Deficiency and other anemia
59	D598	Deficiency and other anemia
59	D599	Deficiency and other anemia
59	D600	Deficiency and other anemia
59	D601	Deficiency and other anemia
59	D608	Deficiency and other anemia
59	D609	Deficiency and other anemia
59	D6101	Deficiency and other anemia
59	D6109	Deficiency and other anemia
59	D611	Deficiency and other anemia
59	D612	Deficiency and other anemia
59	D613	Deficiency and other anemia
59	D61810	Deficiency and other anemia
59	D61811	Deficiency and other anemia
59	D61818	Deficiency and other anemia
59	D6182	Deficiency and other anemia
59	D6189	Deficiency and other anemia
59	D619	Deficiency and other anemia
59	D630	Deficiency and other anemia
59	D631	Deficiency and other anemia
59	D638	Deficiency and other anemia
59	D640	Deficiency and other anemia
59	D641	Deficiency and other anemia
59	D642	Deficiency and other anemia
59	D643	Deficiency and other anemia
59	D644	Deficiency and other anemia
59	D6489	Deficiency and other anemia
59	D649	Deficiency and other anemia
59	Z8631	Deficiency and other anemia
60	D62	Acute posthemorrhagic anemia
61	D5700	Sickle cell anemia
61	D5701	Sickle cell anemia
61	D5702	Sickle cell anemia
61	D571	Sickle cell anemia
61	D5720	Sickle cell anemia
61	D57211	Sickle cell anemia
61	D57212	Sickle cell anemia
61	D57219	Sickle cell anemia
61	D573	Sickle cell anemia

61	D5740	Sickle cell anemia
61	D57411	Sickle cell anemia
61	D57412	Sickle cell anemia
61	D57419	Sickle cell anemia
61	D5780	Sickle cell anemia
61	D57811	Sickle cell anemia
61	D57812	Sickle cell anemia
61	D57819	Sickle cell anemia
62	D65	Coagulation and hemorrhagic disorders
62	D66	Coagulation and hemorrhagic disorders
62	D67	Coagulation and hemorrhagic disorders
62	D680	Coagulation and hemorrhagic disorders
62	D681	Coagulation and hemorrhagic disorders
62	D682	Coagulation and hemorrhagic disorders
62	D68311	Coagulation and hemorrhagic disorders
62	D68312	Coagulation and hemorrhagic disorders
62	D68318	Coagulation and hemorrhagic disorders
62	D6832	Coagulation and hemorrhagic disorders
62	D684	Coagulation and hemorrhagic disorders
62	D6851	Coagulation and hemorrhagic disorders
62	D6852	Coagulation and hemorrhagic disorders
62	D6859	Coagulation and hemorrhagic disorders
62	D6861	Coagulation and hemorrhagic disorders
62	D6862	Coagulation and hemorrhagic disorders
62	D6869	Coagulation and hemorrhagic disorders
62	D688	Coagulation and hemorrhagic disorders
62	D689	Coagulation and hemorrhagic disorders
62	D690	Coagulation and hemorrhagic disorders
62	D691	Coagulation and hemorrhagic disorders
62	D692	Coagulation and hemorrhagic disorders
62	D693	Coagulation and hemorrhagic disorders
62	D6941	Coagulation and hemorrhagic disorders
62	D6942	Coagulation and hemorrhagic disorders
62	D6949	Coagulation and hemorrhagic disorders
62	D6959	Coagulation and hemorrhagic disorders
62	D696	Coagulation and hemorrhagic disorders
62	D698	Coagulation and hemorrhagic disorders
62	D699	Coagulation and hemorrhagic disorders
62	D7582	Coagulation and hemorrhagic disorders
62	R233	Coagulation and hemorrhagic disorders
63	D700	Diseases of white blood cells
63	D701	Diseases of white blood cells
63	D702	Diseases of white blood cells
63	D703	Diseases of white blood cells
63	D704	Diseases of white blood cells
63	D708	Diseases of white blood cells
63	D709	Diseases of white blood cells
63	D71	Diseases of white blood cells
63	D720	Diseases of white blood cells
63	D721	Diseases of white blood cells
63	D72810	Diseases of white blood cells

63	D72818	Diseases of white blood cells
63	D72819	Diseases of white blood cells
63	D72820	Diseases of white blood cells
63	D72821	Diseases of white blood cells
63	D72822	Diseases of white blood cells
63	D72823	Diseases of white blood cells
63	D72824	Diseases of white blood cells
63	D72825	Diseases of white blood cells
63	D72828	Diseases of white blood cells
63	D72829	Diseases of white blood cells
63	D7289	Diseases of white blood cells
63	D729	Diseases of white blood cells
63	D7381	Diseases of white blood cells
63	D761	Diseases of white blood cells
63	D762	Diseases of white blood cells
63	D763	Diseases of white blood cells
64	D730	Other hematologic conditions
64	D731	Other hematologic conditions
64	D732	Other hematologic conditions
64	D733	Other hematologic conditions
64	D734	Other hematologic conditions
64	D735	Other hematologic conditions
64	D7389	Other hematologic conditions
64	D739	Other hematologic conditions
64	D740	Other hematologic conditions
64	D748	Other hematologic conditions
64	D749	Other hematologic conditions
64	D750	Other hematologic conditions
64	D751	Other hematologic conditions
64	D7581	Other hematologic conditions
64	D7589	Other hematologic conditions
64	D759	Other hematologic conditions
64	D77	Other hematologic conditions
64	R700	Other hematologic conditions
64	R701	Other hematologic conditions
64	R710	Other hematologic conditions
64	R718	Other hematologic conditions
64	R770	Other hematologic conditions
64	R771	Other hematologic conditions
64	R772	Other hematologic conditions
64	R778	Other hematologic conditions
64	R779	Other hematologic conditions
64	Z862	Other hematologic conditions
76	A0101	Meningitis (except that caused by tuberculosis or sexually transmitted disease)
76	A0221	Meningitis (except that caused by tuberculosis or sexually transmitted disease)
76	A203	Meningitis (except that caused by tuberculosis or sexually transmitted disease)
76	A2781	Meningitis (except that caused by tuberculosis or sexually transmitted disease)
76	A3211	Meningitis (except that caused by tuberculosis or sexually transmitted disease)
76	A390	Meningitis (except that caused by tuberculosis or sexually transmitted disease)
76	A4281	Meningitis (except that caused by tuberculosis or sexually transmitted disease)
76	A6921	Meningitis (except that caused by tuberculosis or sexually transmitted disease)

76	A870	Meningitis (except that caused by tuberculosis or sexually transmitted disease)
76	A871	Meningitis (except that caused by tuberculosis or sexually transmitted disease)
76	A872	Meningitis (except that caused by tuberculosis or sexually transmitted disease)
76	A878	Meningitis (except that caused by tuberculosis or sexually transmitted disease)
76	A879	Meningitis (except that caused by tuberculosis or sexually transmitted disease)
76	B003	Meningitis (except that caused by tuberculosis or sexually transmitted disease)
76	B010	Meningitis (except that caused by tuberculosis or sexually transmitted disease)
76	B021	Meningitis (except that caused by tuberculosis or sexually transmitted disease)
76	B051	Meningitis (except that caused by tuberculosis or sexually transmitted disease)
76	B0602	Meningitis (except that caused by tuberculosis or sexually transmitted disease)
76	B261	Meningitis (except that caused by tuberculosis or sexually transmitted disease)
76	B2702	Meningitis (except that caused by tuberculosis or sexually transmitted disease)
76	B2712	Meningitis (except that caused by tuberculosis or sexually transmitted disease)
76	B2782	Meningitis (except that caused by tuberculosis or sexually transmitted disease)
76	B2792	Meningitis (except that caused by tuberculosis or sexually transmitted disease)
76	B375	Meningitis (except that caused by tuberculosis or sexually transmitted disease)
76	B384	Meningitis (except that caused by tuberculosis or sexually transmitted disease)
76	B451	Meningitis (except that caused by tuberculosis or sexually transmitted disease)
76	B5741	Meningitis (except that caused by tuberculosis or sexually transmitted disease)
76	D8681	Meningitis (except that caused by tuberculosis or sexually transmitted disease)
76	G000	Meningitis (except that caused by tuberculosis or sexually transmitted disease)
76	G001	Meningitis (except that caused by tuberculosis or sexually transmitted disease)
76	G002	Meningitis (except that caused by tuberculosis or sexually transmitted disease)
76	G003	Meningitis (except that caused by tuberculosis or sexually transmitted disease)
76	G008	Meningitis (except that caused by tuberculosis or sexually transmitted disease)
76	G009	Meningitis (except that caused by tuberculosis or sexually transmitted disease)
76	G01	Meningitis (except that caused by tuberculosis or sexually transmitted disease)
76	G02	Meningitis (except that caused by tuberculosis or sexually transmitted disease)
76	G030	Meningitis (except that caused by tuberculosis or sexually transmitted disease)
76	G031	Meningitis (except that caused by tuberculosis or sexually transmitted disease)
76	G032	Meningitis (except that caused by tuberculosis or sexually transmitted disease)
76	G038	Meningitis (except that caused by tuberculosis or sexually transmitted disease)
76	G039	Meningitis (except that caused by tuberculosis or sexually transmitted disease)
77	A3212	Encephalitis (except that caused by tuberculosis or sexually transmitted disease)
77	A3981	Encephalitis (except that caused by tuberculosis or sexually transmitted disease)
77	A4282	Encephalitis (except that caused by tuberculosis or sexually transmitted disease)
77	A811	Encephalitis (except that caused by tuberculosis or sexually transmitted disease)
77	A830	Encephalitis (except that caused by tuberculosis or sexually transmitted disease)
77	A831	Encephalitis (except that caused by tuberculosis or sexually transmitted disease)
77	A832	Encephalitis (except that caused by tuberculosis or sexually transmitted disease)
77	A833	Encephalitis (except that caused by tuberculosis or sexually transmitted disease)
77	A834	Encephalitis (except that caused by tuberculosis or sexually transmitted disease)
77	A835	Encephalitis (except that caused by tuberculosis or sexually transmitted disease)
77	A836	Encephalitis (except that caused by tuberculosis or sexually transmitted disease)
77	A838	Encephalitis (except that caused by tuberculosis or sexually transmitted disease)
77	A839	Encephalitis (except that caused by tuberculosis or sexually transmitted disease)
77	A840	Encephalitis (except that caused by tuberculosis or sexually transmitted disease)
77	A841	Encephalitis (except that caused by tuberculosis or sexually transmitted disease)
77	A848	Encephalitis (except that caused by tuberculosis or sexually transmitted disease)
77	A849	Encephalitis (except that caused by tuberculosis or sexually transmitted disease)
77	A850	Encephalitis (except that caused by tuberculosis or sexually transmitted disease)

77	A851	Encephalitis (except that caused by tuberculosis or sexually transmitted disease)
77	A852	Encephalitis (except that caused by tuberculosis or sexually transmitted disease)
77	A858	Encephalitis (except that caused by tuberculosis or sexually transmitted disease)
77	A86	Encephalitis (except that caused by tuberculosis or sexually transmitted disease)
77	A89	Encephalitis (except that caused by tuberculosis or sexually transmitted disease)
77	A90	Encephalitis (except that caused by tuberculosis or sexually transmitted disease)
77	A922	Encephalitis (except that caused by tuberculosis or sexually transmitted disease)
77	A9231	Encephalitis (except that caused by tuberculosis or sexually transmitted disease)
77	B004	Encephalitis (except that caused by tuberculosis or sexually transmitted disease)
77	B0111	Encephalitis (except that caused by tuberculosis or sexually transmitted disease)
77	B020	Encephalitis (except that caused by tuberculosis or sexually transmitted disease)
77	B050	Encephalitis (except that caused by tuberculosis or sexually transmitted disease)
77	B0601	Encephalitis (except that caused by tuberculosis or sexually transmitted disease)
77	B1001	Encephalitis (except that caused by tuberculosis or sexually transmitted disease)
77	B1009	Encephalitis (except that caused by tuberculosis or sexually transmitted disease)
77	B262	Encephalitis (except that caused by tuberculosis or sexually transmitted disease)
77	B4081	Encephalitis (except that caused by tuberculosis or sexually transmitted disease)
77	B5742	Encephalitis (except that caused by tuberculosis or sexually transmitted disease)
77	B582	Encephalitis (except that caused by tuberculosis or sexually transmitted disease)
77	B6011	Encephalitis (except that caused by tuberculosis or sexually transmitted disease)
77	B941	Encephalitis (except that caused by tuberculosis or sexually transmitted disease)
77	G0400	Encephalitis (except that caused by tuberculosis or sexually transmitted disease)
77	G0401	Encephalitis (except that caused by tuberculosis or sexually transmitted disease)
77	G0402	Encephalitis (except that caused by tuberculosis or sexually transmitted disease)
77	G042	Encephalitis (except that caused by tuberculosis or sexually transmitted disease)
77	G0430	Encephalitis (except that caused by tuberculosis or sexually transmitted disease)
77	G0431	Encephalitis (except that caused by tuberculosis or sexually transmitted disease)
77	G0432	Encephalitis (except that caused by tuberculosis or sexually transmitted disease)
77	G0439	Encephalitis (except that caused by tuberculosis or sexually transmitted disease)
77	G0481	Encephalitis (except that caused by tuberculosis or sexually transmitted disease)
77	G0489	Encephalitis (except that caused by tuberculosis or sexually transmitted disease)
77	G0490	Encephalitis (except that caused by tuberculosis or sexually transmitted disease)
77	G0491	Encephalitis (except that caused by tuberculosis or sexually transmitted disease)
77	G053	Encephalitis (except that caused by tuberculosis or sexually transmitted disease)
77	G054	Encephalitis (except that caused by tuberculosis or sexually transmitted disease)
78	A800	Other CNS infection and poliomyelitis
78	A801	Other CNS infection and poliomyelitis
78	A802	Other CNS infection and poliomyelitis
78	A8030	Other CNS infection and poliomyelitis
78	A8039	Other CNS infection and poliomyelitis
78	A804	Other CNS infection and poliomyelitis
78	A809	Other CNS infection and poliomyelitis
78	A8100	Other CNS infection and poliomyelitis
78	A8101	Other CNS infection and poliomyelitis
78	A8109	Other CNS infection and poliomyelitis
78	A812	Other CNS infection and poliomyelitis
78	A8181	Other CNS infection and poliomyelitis
78	A8182	Other CNS infection and poliomyelitis
78	A8183	Other CNS infection and poliomyelitis
78	A8189	Other CNS infection and poliomyelitis
78	A819	Other CNS infection and poliomyelitis

78	A880	Other CNS infection and poliomyelitis
78	A881	Other CNS infection and poliomyelitis
78	A888	Other CNS infection and poliomyelitis
78	B91	Other CNS infection and poliomyelitis
78	G060	Other CNS infection and poliomyelitis
78	G061	Other CNS infection and poliomyelitis
78	G062	Other CNS infection and poliomyelitis
78	G07	Other CNS infection and poliomyelitis
78	G09	Other CNS infection and poliomyelitis
78	G14	Other CNS infection and poliomyelitis
78	I673	Other CNS infection and poliomyelitis
78	Z8612	Other CNS infection and poliomyelitis
79	G20	Parkinson`s disease
80	G35	Multiple sclerosis
81	E7500	Other hereditary and degenerative nervous system conditions
81	E7501	Other hereditary and degenerative nervous system conditions
81	E7502	Other hereditary and degenerative nervous system conditions
81	E7509	Other hereditary and degenerative nervous system conditions
81	E7510	Other hereditary and degenerative nervous system conditions
81	E7511	Other hereditary and degenerative nervous system conditions
81	E7519	Other hereditary and degenerative nervous system conditions
81	E7523	Other hereditary and degenerative nervous system conditions
81	E7525	Other hereditary and degenerative nervous system conditions
81	E7529	Other hereditary and degenerative nervous system conditions
81	E754	Other hereditary and degenerative nervous system conditions
81	G10	Other hereditary and degenerative nervous system conditions
81	G110	Other hereditary and degenerative nervous system conditions
81	G111	Other hereditary and degenerative nervous system conditions
81	G112	Other hereditary and degenerative nervous system conditions
81	G113	Other hereditary and degenerative nervous system conditions
81	G114	Other hereditary and degenerative nervous system conditions
81	G118	Other hereditary and degenerative nervous system conditions
81	G119	Other hereditary and degenerative nervous system conditions
81	G120	Other hereditary and degenerative nervous system conditions
81	G121	Other hereditary and degenerative nervous system conditions
81	G1220	Other hereditary and degenerative nervous system conditions
81	G1221	Other hereditary and degenerative nervous system conditions
81	G1222	Other hereditary and degenerative nervous system conditions
81	G1229	Other hereditary and degenerative nervous system conditions
81	G128	Other hereditary and degenerative nervous system conditions
81	G129	Other hereditary and degenerative nervous system conditions
81	G230	Other hereditary and degenerative nervous system conditions
81	G231	Other hereditary and degenerative nervous system conditions
81	G232	Other hereditary and degenerative nervous system conditions
81	G238	Other hereditary and degenerative nervous system conditions
81	G239	Other hereditary and degenerative nervous system conditions
81	G2401	Other hereditary and degenerative nervous system conditions
81	G2402	Other hereditary and degenerative nervous system conditions
81	G2409	Other hereditary and degenerative nervous system conditions
81	G241	Other hereditary and degenerative nervous system conditions
81	G242	Other hereditary and degenerative nervous system conditions

81	G243	Other hereditary and degenerative nervous system conditions
81	G244	Other hereditary and degenerative nervous system conditions
81	G245	Other hereditary and degenerative nervous system conditions
81	G248	Other hereditary and degenerative nervous system conditions
81	G249	Other hereditary and degenerative nervous system conditions
81	G250	Other hereditary and degenerative nervous system conditions
81	G251	Other hereditary and degenerative nervous system conditions
81	G252	Other hereditary and degenerative nervous system conditions
81	G253	Other hereditary and degenerative nervous system conditions
81	G254	Other hereditary and degenerative nervous system conditions
81	G255	Other hereditary and degenerative nervous system conditions
81	G2561	Other hereditary and degenerative nervous system conditions
81	G2569	Other hereditary and degenerative nervous system conditions
81	G2570	Other hereditary and degenerative nervous system conditions
81	G2571	Other hereditary and degenerative nervous system conditions
81	G2579	Other hereditary and degenerative nervous system conditions
81	G2581	Other hereditary and degenerative nervous system conditions
81	G2582	Other hereditary and degenerative nervous system conditions
81	G2583	Other hereditary and degenerative nervous system conditions
81	G2589	Other hereditary and degenerative nervous system conditions
81	G259	Other hereditary and degenerative nervous system conditions
81	G26	Other hereditary and degenerative nervous system conditions
81	G312	Other hereditary and degenerative nervous system conditions
81	G3181	Other hereditary and degenerative nervous system conditions
81	G3182	Other hereditary and degenerative nervous system conditions
81	G3184	Other hereditary and degenerative nervous system conditions
81	G3185	Other hereditary and degenerative nervous system conditions
81	G3189	Other hereditary and degenerative nervous system conditions
81	G319	Other hereditary and degenerative nervous system conditions
81	G320	Other hereditary and degenerative nervous system conditions
81	G3281	Other hereditary and degenerative nervous system conditions
81	G803	Other hereditary and degenerative nervous system conditions
81	G9001	Other hereditary and degenerative nervous system conditions
81	G9009	Other hereditary and degenerative nervous system conditions
81	G903	Other hereditary and degenerative nervous system conditions
81	G904	Other hereditary and degenerative nervous system conditions
81	G990	Other hereditary and degenerative nervous system conditions
81	G992	Other hereditary and degenerative nervous system conditions
82	G041	Paralysis
82	G800	Paralysis
82	G801	Paralysis
82	G802	Paralysis
82	G804	Paralysis
82	G808	Paralysis
82	G809	Paralysis
82	G8100	Paralysis
82	G8101	Paralysis
82	G8102	Paralysis
82	G8103	Paralysis
82	G8104	Paralysis
82	G8110	Paralysis

82	G8111	Paralysis
82	G8112	Paralysis
82	G8113	Paralysis
82	G8114	Paralysis
82	G8190	Paralysis
82	G8191	Paralysis
82	G8192	Paralysis
82	G8193	Paralysis
82	G8194	Paralysis
82	G8220	Paralysis
82	G8221	Paralysis
82	G8222	Paralysis
82	G8250	Paralysis
82	G8251	Paralysis
82	G8252	Paralysis
82	G8253	Paralysis
82	G8254	Paralysis
82	G830	Paralysis
82	G8310	Paralysis
82	G8311	Paralysis
82	G8312	Paralysis
82	G8313	Paralysis
82	G8314	Paralysis
82	G8320	Paralysis
82	G8321	Paralysis
82	G8322	Paralysis
82	G8323	Paralysis
82	G8324	Paralysis
82	G8330	Paralysis
82	G8331	Paralysis
82	G8332	Paralysis
82	G8333	Paralysis
82	G8334	Paralysis
82	G834	Paralysis
82	G835	Paralysis
82	G8381	Paralysis
82	G8382	Paralysis
82	G8383	Paralysis
82	G8384	Paralysis
82	G8389	Paralysis
82	G839	Paralysis
82	R295	Paralysis
82	R532	Paralysis
83	G40001	Epilepsy; convulsions
83	G40009	Epilepsy; convulsions
83	G40011	Epilepsy; convulsions
83	G40019	Epilepsy; convulsions
83	G40101	Epilepsy; convulsions
83	G40109	Epilepsy; convulsions
83	G40111	Epilepsy; convulsions
83	G40119	Epilepsy; convulsions

83	G40201	Epilepsy; convulsions
83	G40209	Epilepsy; convulsions
83	G40211	Epilepsy; convulsions
83	G40219	Epilepsy; convulsions
83	G40301	Epilepsy; convulsions
83	G40309	Epilepsy; convulsions
83	G40311	Epilepsy; convulsions
83	G40319	Epilepsy; convulsions
83	G40401	Epilepsy; convulsions
83	G40409	Epilepsy; convulsions
83	G40411	Epilepsy; convulsions
83	G40419	Epilepsy; convulsions
83	G40501	Epilepsy; convulsions
83	G40509	Epilepsy; convulsions
83	G40801	Epilepsy; convulsions
83	G40802	Epilepsy; convulsions
83	G40803	Epilepsy; convulsions
83	G40804	Epilepsy; convulsions
83	G40811	Epilepsy; convulsions
83	G40812	Epilepsy; convulsions
83	G40813	Epilepsy; convulsions
83	G40814	Epilepsy; convulsions
83	G40821	Epilepsy; convulsions
83	G40822	Epilepsy; convulsions
83	G40823	Epilepsy; convulsions
83	G40824	Epilepsy; convulsions
83	G4089	Epilepsy; convulsions
83	G40901	Epilepsy; convulsions
83	G40909	Epilepsy; convulsions
83	G40911	Epilepsy; convulsions
83	G40919	Epilepsy; convulsions
83	G40A01	Epilepsy; convulsions
83	G40A09	Epilepsy; convulsions
83	G40A11	Epilepsy; convulsions
83	G40A19	Epilepsy; convulsions
83	G40B01	Epilepsy; convulsions
83	G40B09	Epilepsy; convulsions
83	G40B11	Epilepsy; convulsions
83	G40B19	Epilepsy; convulsions
83	R5600	Epilepsy; convulsions
83	R5601	Epilepsy; convulsions
83	R561	Epilepsy; convulsions
83	R569	Epilepsy; convulsions
84	G43001	Headache; including migraine
84	G43009	Headache; including migraine
84	G43011	Headache; including migraine
84	G43019	Headache; including migraine
84	G43101	Headache; including migraine
84	G43109	Headache; including migraine
84	G43111	Headache; including migraine
84	G43119	Headache; including migraine

84	G43401	Headache; including migraine
84	G43409	Headache; including migraine
84	G43411	Headache; including migraine
84	G43419	Headache; including migraine
84	G43501	Headache; including migraine
84	G43509	Headache; including migraine
84	G43511	Headache; including migraine
84	G43519	Headache; including migraine
84	G43701	Headache; including migraine
84	G43709	Headache; including migraine
84	G43711	Headache; including migraine
84	G43719	Headache; including migraine
84	G43801	Headache; including migraine
84	G43809	Headache; including migraine
84	G43811	Headache; including migraine
84	G43819	Headache; including migraine
84	G43821	Headache; including migraine
84	G43829	Headache; including migraine
84	G43831	Headache; including migraine
84	G43839	Headache; including migraine
84	G43901	Headache; including migraine
84	G43909	Headache; including migraine
84	G43911	Headache; including migraine
84	G43919	Headache; including migraine
84	G43A0	Headache; including migraine
84	G43A1	Headache; including migraine
84	G43B0	Headache; including migraine
84	G43B1	Headache; including migraine
84	G43C0	Headache; including migraine
84	G43C1	Headache; including migraine
84	G43D0	Headache; including migraine
84	G43D1	Headache; including migraine
84	G44001	Headache; including migraine
84	G44009	Headache; including migraine
84	G44011	Headache; including migraine
84	G44019	Headache; including migraine
84	G44021	Headache; including migraine
84	G44029	Headache; including migraine
84	G44031	Headache; including migraine
84	G44039	Headache; including migraine
84	G44041	Headache; including migraine
84	G44049	Headache; including migraine
84	G44051	Headache; including migraine
84	G44059	Headache; including migraine
84	G44091	Headache; including migraine
84	G44099	Headache; including migraine
84	G441	Headache; including migraine
84	G44201	Headache; including migraine
84	G44209	Headache; including migraine
84	G44211	Headache; including migraine
84	G44219	Headache; including migraine

84	G44221	Headache; including migraine
84	G44229	Headache; including migraine
84	G44301	Headache; including migraine
84	G44309	Headache; including migraine
84	G44311	Headache; including migraine
84	G44319	Headache; including migraine
84	G44321	Headache; including migraine
84	G44329	Headache; including migraine
84	G4440	Headache; including migraine
84	G4441	Headache; including migraine
84	G4451	Headache; including migraine
84	G4452	Headache; including migraine
84	G4453	Headache; including migraine
84	G4459	Headache; including migraine
84	G4481	Headache; including migraine
84	G4482	Headache; including migraine
84	G4483	Headache; including migraine
84	G4484	Headache; including migraine
84	G4485	Headache; including migraine
84	G4489	Headache; including migraine
84	R51	Headache; including migraine
85	G931	Coma; stupor; and brain damage
85	R400	Coma; stupor; and brain damage
85	R401	Coma; stupor; and brain damage
85	R4020	Coma; stupor; and brain damage
85	R402110	Coma; stupor; and brain damage
85	R402111	Coma; stupor; and brain damage
85	R402112	Coma; stupor; and brain damage
85	R402113	Coma; stupor; and brain damage
85	R402114	Coma; stupor; and brain damage
85	R402120	Coma; stupor; and brain damage
85	R402121	Coma; stupor; and brain damage
85	R402122	Coma; stupor; and brain damage
85	R402123	Coma; stupor; and brain damage
85	R402124	Coma; stupor; and brain damage
85	R402130	Coma; stupor; and brain damage
85	R402131	Coma; stupor; and brain damage
85	R402132	Coma; stupor; and brain damage
85	R402133	Coma; stupor; and brain damage
85	R402134	Coma; stupor; and brain damage
85	R402140	Coma; stupor; and brain damage
85	R402141	Coma; stupor; and brain damage
85	R402142	Coma; stupor; and brain damage
85	R402143	Coma; stupor; and brain damage
85	R402144	Coma; stupor; and brain damage
85	R402210	Coma; stupor; and brain damage
85	R402211	Coma; stupor; and brain damage
85	R402212	Coma; stupor; and brain damage
85	R402213	Coma; stupor; and brain damage
85	R402214	Coma; stupor; and brain damage
85	R402220	Coma; stupor; and brain damage

85	R402221	Coma; stupor; and brain damage
85	R402222	Coma; stupor; and brain damage
85	R402223	Coma; stupor; and brain damage
85	R402224	Coma; stupor; and brain damage
85	R402230	Coma; stupor; and brain damage
85	R402231	Coma; stupor; and brain damage
85	R402232	Coma; stupor; and brain damage
85	R402233	Coma; stupor; and brain damage
85	R402234	Coma; stupor; and brain damage
85	R402240	Coma; stupor; and brain damage
85	R402241	Coma; stupor; and brain damage
85	R402242	Coma; stupor; and brain damage
85	R402243	Coma; stupor; and brain damage
85	R402244	Coma; stupor; and brain damage
85	R402250	Coma; stupor; and brain damage
85	R402251	Coma; stupor; and brain damage
85	R402252	Coma; stupor; and brain damage
85	R402253	Coma; stupor; and brain damage
85	R402254	Coma; stupor; and brain damage
85	R402310	Coma; stupor; and brain damage
85	R402311	Coma; stupor; and brain damage
85	R402312	Coma; stupor; and brain damage
85	R402313	Coma; stupor; and brain damage
85	R402314	Coma; stupor; and brain damage
85	R402320	Coma; stupor; and brain damage
85	R402321	Coma; stupor; and brain damage
85	R402322	Coma; stupor; and brain damage
85	R402323	Coma; stupor; and brain damage
85	R402324	Coma; stupor; and brain damage
85	R402330	Coma; stupor; and brain damage
85	R402331	Coma; stupor; and brain damage
85	R402332	Coma; stupor; and brain damage
85	R402333	Coma; stupor; and brain damage
85	R402334	Coma; stupor; and brain damage
85	R402340	Coma; stupor; and brain damage
85	R402341	Coma; stupor; and brain damage
85	R402342	Coma; stupor; and brain damage
85	R402343	Coma; stupor; and brain damage
85	R402344	Coma; stupor; and brain damage
85	R402350	Coma; stupor; and brain damage
85	R402351	Coma; stupor; and brain damage
85	R402352	Coma; stupor; and brain damage
85	R402353	Coma; stupor; and brain damage
85	R402354	Coma; stupor; and brain damage
85	R402360	Coma; stupor; and brain damage
85	R402361	Coma; stupor; and brain damage
85	R402362	Coma; stupor; and brain damage
85	R402363	Coma; stupor; and brain damage
85	R402364	Coma; stupor; and brain damage
85	R40241	Coma; stupor; and brain damage
85	R40242	Coma; stupor; and brain damage

85	R40243	Coma; stupor; and brain damage
85	R40244	Coma; stupor; and brain damage
85	R403	Coma; stupor; and brain damage
86	H25011	Cataract
86	H25012	Cataract
86	H25013	Cataract
86	H25019	Cataract
86	H25031	Cataract
86	H25032	Cataract
86	H25033	Cataract
86	H25039	Cataract
86	H25041	Cataract
86	H25042	Cataract
86	H25043	Cataract
86	H25049	Cataract
86	H25091	Cataract
86	H25092	Cataract
86	H25093	Cataract
86	H25099	Cataract
86	H2510	Cataract
86	H2511	Cataract
86	H2512	Cataract
86	H2513	Cataract
86	H2520	Cataract
86	H2521	Cataract
86	H2522	Cataract
86	H2523	Cataract
86	H25811	Cataract
86	H25812	Cataract
86	H25813	Cataract
86	H25819	Cataract
86	H2589	Cataract
86	H259	Cataract
86	H26001	Cataract
86	H26002	Cataract
86	H26003	Cataract
86	H26009	Cataract
86	H26011	Cataract
86	H26012	Cataract
86	H26013	Cataract
86	H26019	Cataract
86	H26031	Cataract
86	H26032	Cataract
86	H26033	Cataract
86	H26039	Cataract
86	H26041	Cataract
86	H26042	Cataract
86	H26043	Cataract
86	H26049	Cataract
86	H26051	Cataract
86	H26052	Cataract

86	H26053	Cataract
86	H26059	Cataract
86	H26061	Cataract
86	H26062	Cataract
86	H26063	Cataract
86	H26069	Cataract
86	H2609	Cataract
86	H26101	Cataract
86	H26102	Cataract
86	H26103	Cataract
86	H26109	Cataract
86	H26111	Cataract
86	H26112	Cataract
86	H26113	Cataract
86	H26119	Cataract
86	H26121	Cataract
86	H26122	Cataract
86	H26123	Cataract
86	H26129	Cataract
86	H26131	Cataract
86	H26132	Cataract
86	H26133	Cataract
86	H26139	Cataract
86	H2620	Cataract
86	H26211	Cataract
86	H26212	Cataract
86	H26213	Cataract
86	H26219	Cataract
86	H26221	Cataract
86	H26222	Cataract
86	H26223	Cataract
86	H26229	Cataract
86	H26231	Cataract
86	H26232	Cataract
86	H26233	Cataract
86	H26239	Cataract
86	H2630	Cataract
86	H2631	Cataract
86	H2632	Cataract
86	H2633	Cataract
86	H2640	Cataract
86	H26411	Cataract
86	H26412	Cataract
86	H26413	Cataract
86	H26419	Cataract
86	H26491	Cataract
86	H26492	Cataract
86	H26493	Cataract
86	H26499	Cataract
86	H268	Cataract
86	H269	Cataract

86	H28	Cataract
86	Z961	Cataract
87	H33001	Retinal detachments; defects; vascular occlusion; and retinopathy
87	H33002	Retinal detachments; defects; vascular occlusion; and retinopathy
87	H33003	Retinal detachments; defects; vascular occlusion; and retinopathy
87	H33009	Retinal detachments; defects; vascular occlusion; and retinopathy
87	H33011	Retinal detachments; defects; vascular occlusion; and retinopathy
87	H33012	Retinal detachments; defects; vascular occlusion; and retinopathy
87	H33013	Retinal detachments; defects; vascular occlusion; and retinopathy
87	H33019	Retinal detachments; defects; vascular occlusion; and retinopathy
87	H33021	Retinal detachments; defects; vascular occlusion; and retinopathy
87	H33022	Retinal detachments; defects; vascular occlusion; and retinopathy
87	H33023	Retinal detachments; defects; vascular occlusion; and retinopathy
87	H33029	Retinal detachments; defects; vascular occlusion; and retinopathy
87	H33031	Retinal detachments; defects; vascular occlusion; and retinopathy
87	H33032	Retinal detachments; defects; vascular occlusion; and retinopathy
87	H33033	Retinal detachments; defects; vascular occlusion; and retinopathy
87	H33039	Retinal detachments; defects; vascular occlusion; and retinopathy
87	H33041	Retinal detachments; defects; vascular occlusion; and retinopathy
87	H33042	Retinal detachments; defects; vascular occlusion; and retinopathy
87	H33043	Retinal detachments; defects; vascular occlusion; and retinopathy
87	H33049	Retinal detachments; defects; vascular occlusion; and retinopathy
87	H33051	Retinal detachments; defects; vascular occlusion; and retinopathy
87	H33052	Retinal detachments; defects; vascular occlusion; and retinopathy
87	H33053	Retinal detachments; defects; vascular occlusion; and retinopathy
87	H33059	Retinal detachments; defects; vascular occlusion; and retinopathy
87	H33101	Retinal detachments; defects; vascular occlusion; and retinopathy
87	H33102	Retinal detachments; defects; vascular occlusion; and retinopathy
87	H33103	Retinal detachments; defects; vascular occlusion; and retinopathy
87	H33109	Retinal detachments; defects; vascular occlusion; and retinopathy
87	H33111	Retinal detachments; defects; vascular occlusion; and retinopathy
87	H33112	Retinal detachments; defects; vascular occlusion; and retinopathy
87	H33113	Retinal detachments; defects; vascular occlusion; and retinopathy
87	H33119	Retinal detachments; defects; vascular occlusion; and retinopathy
87	H33191	Retinal detachments; defects; vascular occlusion; and retinopathy
87	H33192	Retinal detachments; defects; vascular occlusion; and retinopathy
87	H33193	Retinal detachments; defects; vascular occlusion; and retinopathy
87	H33199	Retinal detachments; defects; vascular occlusion; and retinopathy
87	H3320	Retinal detachments; defects; vascular occlusion; and retinopathy
87	H3321	Retinal detachments; defects; vascular occlusion; and retinopathy
87	H3322	Retinal detachments; defects; vascular occlusion; and retinopathy
87	H3323	Retinal detachments; defects; vascular occlusion; and retinopathy
87	H33301	Retinal detachments; defects; vascular occlusion; and retinopathy
87	H33302	Retinal detachments; defects; vascular occlusion; and retinopathy
87	H33303	Retinal detachments; defects; vascular occlusion; and retinopathy
87	H33309	Retinal detachments; defects; vascular occlusion; and retinopathy
87	H33311	Retinal detachments; defects; vascular occlusion; and retinopathy
87	H33312	Retinal detachments; defects; vascular occlusion; and retinopathy
87	H33313	Retinal detachments; defects; vascular occlusion; and retinopathy
87	H33319	Retinal detachments; defects; vascular occlusion; and retinopathy
87	H33321	Retinal detachments; defects; vascular occlusion; and retinopathy

87	H33322	Retinal detachments; defects; vascular occlusion; and retinopathy
87	H33323	Retinal detachments; defects; vascular occlusion; and retinopathy
87	H33329	Retinal detachments; defects; vascular occlusion; and retinopathy
87	H33331	Retinal detachments; defects; vascular occlusion; and retinopathy
87	H33332	Retinal detachments; defects; vascular occlusion; and retinopathy
87	H33333	Retinal detachments; defects; vascular occlusion; and retinopathy
87	H33339	Retinal detachments; defects; vascular occlusion; and retinopathy
87	H3340	Retinal detachments; defects; vascular occlusion; and retinopathy
87	H3341	Retinal detachments; defects; vascular occlusion; and retinopathy
87	H3342	Retinal detachments; defects; vascular occlusion; and retinopathy
87	H3343	Retinal detachments; defects; vascular occlusion; and retinopathy
87	H338	Retinal detachments; defects; vascular occlusion; and retinopathy
87	H3400	Retinal detachments; defects; vascular occlusion; and retinopathy
87	H3401	Retinal detachments; defects; vascular occlusion; and retinopathy
87	H3402	Retinal detachments; defects; vascular occlusion; and retinopathy
87	H3403	Retinal detachments; defects; vascular occlusion; and retinopathy
87	H3410	Retinal detachments; defects; vascular occlusion; and retinopathy
87	H3411	Retinal detachments; defects; vascular occlusion; and retinopathy
87	H3412	Retinal detachments; defects; vascular occlusion; and retinopathy
87	H3413	Retinal detachments; defects; vascular occlusion; and retinopathy
87	H34211	Retinal detachments; defects; vascular occlusion; and retinopathy
87	H34212	Retinal detachments; defects; vascular occlusion; and retinopathy
87	H34213	Retinal detachments; defects; vascular occlusion; and retinopathy
87	H34219	Retinal detachments; defects; vascular occlusion; and retinopathy
87	H34231	Retinal detachments; defects; vascular occlusion; and retinopathy
87	H34232	Retinal detachments; defects; vascular occlusion; and retinopathy
87	H34233	Retinal detachments; defects; vascular occlusion; and retinopathy
87	H34239	Retinal detachments; defects; vascular occlusion; and retinopathy
87	H34811	Retinal detachments; defects; vascular occlusion; and retinopathy
87	H34812	Retinal detachments; defects; vascular occlusion; and retinopathy
87	H34813	Retinal detachments; defects; vascular occlusion; and retinopathy
87	H34819	Retinal detachments; defects; vascular occlusion; and retinopathy
87	H34821	Retinal detachments; defects; vascular occlusion; and retinopathy
87	H34822	Retinal detachments; defects; vascular occlusion; and retinopathy
87	H34823	Retinal detachments; defects; vascular occlusion; and retinopathy
87	H34829	Retinal detachments; defects; vascular occlusion; and retinopathy
87	H34831	Retinal detachments; defects; vascular occlusion; and retinopathy
87	H34832	Retinal detachments; defects; vascular occlusion; and retinopathy
87	H34833	Retinal detachments; defects; vascular occlusion; and retinopathy
87	H34839	Retinal detachments; defects; vascular occlusion; and retinopathy
87	H349	Retinal detachments; defects; vascular occlusion; and retinopathy
87	H3500	Retinal detachments; defects; vascular occlusion; and retinopathy
87	H35011	Retinal detachments; defects; vascular occlusion; and retinopathy
87	H35012	Retinal detachments; defects; vascular occlusion; and retinopathy
87	H35013	Retinal detachments; defects; vascular occlusion; and retinopathy
87	H35019	Retinal detachments; defects; vascular occlusion; and retinopathy
87	H35021	Retinal detachments; defects; vascular occlusion; and retinopathy
87	H35022	Retinal detachments; defects; vascular occlusion; and retinopathy
87	H35023	Retinal detachments; defects; vascular occlusion; and retinopathy
87	H35029	Retinal detachments; defects; vascular occlusion; and retinopathy
87	H35031	Retinal detachments; defects; vascular occlusion; and retinopathy

87	H35032	Retinal detachments; defects; vascular occlusion; and retinopathy
87	H35033	Retinal detachments; defects; vascular occlusion; and retinopathy
87	H35039	Retinal detachments; defects; vascular occlusion; and retinopathy
87	H35041	Retinal detachments; defects; vascular occlusion; and retinopathy
87	H35042	Retinal detachments; defects; vascular occlusion; and retinopathy
87	H35043	Retinal detachments; defects; vascular occlusion; and retinopathy
87	H35049	Retinal detachments; defects; vascular occlusion; and retinopathy
87	H35051	Retinal detachments; defects; vascular occlusion; and retinopathy
87	H35052	Retinal detachments; defects; vascular occlusion; and retinopathy
87	H35053	Retinal detachments; defects; vascular occlusion; and retinopathy
87	H35059	Retinal detachments; defects; vascular occlusion; and retinopathy
87	H35061	Retinal detachments; defects; vascular occlusion; and retinopathy
87	H35062	Retinal detachments; defects; vascular occlusion; and retinopathy
87	H35063	Retinal detachments; defects; vascular occlusion; and retinopathy
87	H35069	Retinal detachments; defects; vascular occlusion; and retinopathy
87	H35071	Retinal detachments; defects; vascular occlusion; and retinopathy
87	H35072	Retinal detachments; defects; vascular occlusion; and retinopathy
87	H35073	Retinal detachments; defects; vascular occlusion; and retinopathy
87	H35079	Retinal detachments; defects; vascular occlusion; and retinopathy
87	H3509	Retinal detachments; defects; vascular occlusion; and retinopathy
87	H35101	Retinal detachments; defects; vascular occlusion; and retinopathy
87	H35102	Retinal detachments; defects; vascular occlusion; and retinopathy
87	H35103	Retinal detachments; defects; vascular occlusion; and retinopathy
87	H35109	Retinal detachments; defects; vascular occlusion; and retinopathy
87	H35111	Retinal detachments; defects; vascular occlusion; and retinopathy
87	H35112	Retinal detachments; defects; vascular occlusion; and retinopathy
87	H35113	Retinal detachments; defects; vascular occlusion; and retinopathy
87	H35119	Retinal detachments; defects; vascular occlusion; and retinopathy
87	H35121	Retinal detachments; defects; vascular occlusion; and retinopathy
87	H35122	Retinal detachments; defects; vascular occlusion; and retinopathy
87	H35123	Retinal detachments; defects; vascular occlusion; and retinopathy
87	H35129	Retinal detachments; defects; vascular occlusion; and retinopathy
87	H35131	Retinal detachments; defects; vascular occlusion; and retinopathy
87	H35132	Retinal detachments; defects; vascular occlusion; and retinopathy
87	H35133	Retinal detachments; defects; vascular occlusion; and retinopathy
87	H35139	Retinal detachments; defects; vascular occlusion; and retinopathy
87	H35141	Retinal detachments; defects; vascular occlusion; and retinopathy
87	H35142	Retinal detachments; defects; vascular occlusion; and retinopathy
87	H35143	Retinal detachments; defects; vascular occlusion; and retinopathy
87	H35149	Retinal detachments; defects; vascular occlusion; and retinopathy
87	H35151	Retinal detachments; defects; vascular occlusion; and retinopathy
87	H35152	Retinal detachments; defects; vascular occlusion; and retinopathy
87	H35153	Retinal detachments; defects; vascular occlusion; and retinopathy
87	H35159	Retinal detachments; defects; vascular occlusion; and retinopathy
87	H35161	Retinal detachments; defects; vascular occlusion; and retinopathy
87	H35162	Retinal detachments; defects; vascular occlusion; and retinopathy
87	H35163	Retinal detachments; defects; vascular occlusion; and retinopathy
87	H35169	Retinal detachments; defects; vascular occlusion; and retinopathy
87	H35171	Retinal detachments; defects; vascular occlusion; and retinopathy
87	H35172	Retinal detachments; defects; vascular occlusion; and retinopathy
87	H35173	Retinal detachments; defects; vascular occlusion; and retinopathy

87	H35179	Retinal detachments; defects; vascular occlusion; and retinopathy
87	H3520	Retinal detachments; defects; vascular occlusion; and retinopathy
87	H3521	Retinal detachments; defects; vascular occlusion; and retinopathy
87	H3522	Retinal detachments; defects; vascular occlusion; and retinopathy
87	H3523	Retinal detachments; defects; vascular occlusion; and retinopathy
87	H3530	Retinal detachments; defects; vascular occlusion; and retinopathy
87	H3531	Retinal detachments; defects; vascular occlusion; and retinopathy
87	H3532	Retinal detachments; defects; vascular occlusion; and retinopathy
87	H35341	Retinal detachments; defects; vascular occlusion; and retinopathy
87	H35342	Retinal detachments; defects; vascular occlusion; and retinopathy
87	H35343	Retinal detachments; defects; vascular occlusion; and retinopathy
87	H35349	Retinal detachments; defects; vascular occlusion; and retinopathy
87	H35351	Retinal detachments; defects; vascular occlusion; and retinopathy
87	H35352	Retinal detachments; defects; vascular occlusion; and retinopathy
87	H35353	Retinal detachments; defects; vascular occlusion; and retinopathy
87	H35359	Retinal detachments; defects; vascular occlusion; and retinopathy
87	H35361	Retinal detachments; defects; vascular occlusion; and retinopathy
87	H35362	Retinal detachments; defects; vascular occlusion; and retinopathy
87	H35363	Retinal detachments; defects; vascular occlusion; and retinopathy
87	H35369	Retinal detachments; defects; vascular occlusion; and retinopathy
87	H35371	Retinal detachments; defects; vascular occlusion; and retinopathy
87	H35372	Retinal detachments; defects; vascular occlusion; and retinopathy
87	H35373	Retinal detachments; defects; vascular occlusion; and retinopathy
87	H35379	Retinal detachments; defects; vascular occlusion; and retinopathy
87	H35381	Retinal detachments; defects; vascular occlusion; and retinopathy
87	H35382	Retinal detachments; defects; vascular occlusion; and retinopathy
87	H35383	Retinal detachments; defects; vascular occlusion; and retinopathy
87	H35389	Retinal detachments; defects; vascular occlusion; and retinopathy
87	H3540	Retinal detachments; defects; vascular occlusion; and retinopathy
87	H35411	Retinal detachments; defects; vascular occlusion; and retinopathy
87	H35412	Retinal detachments; defects; vascular occlusion; and retinopathy
87	H35413	Retinal detachments; defects; vascular occlusion; and retinopathy
87	H35419	Retinal detachments; defects; vascular occlusion; and retinopathy
87	H35421	Retinal detachments; defects; vascular occlusion; and retinopathy
87	H35422	Retinal detachments; defects; vascular occlusion; and retinopathy
87	H35423	Retinal detachments; defects; vascular occlusion; and retinopathy
87	H35429	Retinal detachments; defects; vascular occlusion; and retinopathy
87	H35431	Retinal detachments; defects; vascular occlusion; and retinopathy
87	H35432	Retinal detachments; defects; vascular occlusion; and retinopathy
87	H35433	Retinal detachments; defects; vascular occlusion; and retinopathy
87	H35439	Retinal detachments; defects; vascular occlusion; and retinopathy
87	H35441	Retinal detachments; defects; vascular occlusion; and retinopathy
87	H35442	Retinal detachments; defects; vascular occlusion; and retinopathy
87	H35443	Retinal detachments; defects; vascular occlusion; and retinopathy
87	H35449	Retinal detachments; defects; vascular occlusion; and retinopathy
87	H35451	Retinal detachments; defects; vascular occlusion; and retinopathy
87	H35452	Retinal detachments; defects; vascular occlusion; and retinopathy
87	H35453	Retinal detachments; defects; vascular occlusion; and retinopathy
87	H35459	Retinal detachments; defects; vascular occlusion; and retinopathy
87	H35461	Retinal detachments; defects; vascular occlusion; and retinopathy
87	H35462	Retinal detachments; defects; vascular occlusion; and retinopathy

87	H35463	Retinal detachments; defects; vascular occlusion; and retinopathy
87	H35469	Retinal detachments; defects; vascular occlusion; and retinopathy
87	H3550	Retinal detachments; defects; vascular occlusion; and retinopathy
87	H3551	Retinal detachments; defects; vascular occlusion; and retinopathy
87	H3552	Retinal detachments; defects; vascular occlusion; and retinopathy
87	H3553	Retinal detachments; defects; vascular occlusion; and retinopathy
87	H3554	Retinal detachments; defects; vascular occlusion; and retinopathy
87	H3560	Retinal detachments; defects; vascular occlusion; and retinopathy
87	H3561	Retinal detachments; defects; vascular occlusion; and retinopathy
87	H3562	Retinal detachments; defects; vascular occlusion; and retinopathy
87	H3563	Retinal detachments; defects; vascular occlusion; and retinopathy
87	H3570	Retinal detachments; defects; vascular occlusion; and retinopathy
87	H35711	Retinal detachments; defects; vascular occlusion; and retinopathy
87	H35712	Retinal detachments; defects; vascular occlusion; and retinopathy
87	H35713	Retinal detachments; defects; vascular occlusion; and retinopathy
87	H35719	Retinal detachments; defects; vascular occlusion; and retinopathy
87	H35721	Retinal detachments; defects; vascular occlusion; and retinopathy
87	H35722	Retinal detachments; defects; vascular occlusion; and retinopathy
87	H35723	Retinal detachments; defects; vascular occlusion; and retinopathy
87	H35729	Retinal detachments; defects; vascular occlusion; and retinopathy
87	H35731	Retinal detachments; defects; vascular occlusion; and retinopathy
87	H35732	Retinal detachments; defects; vascular occlusion; and retinopathy
87	H35733	Retinal detachments; defects; vascular occlusion; and retinopathy
87	H35739	Retinal detachments; defects; vascular occlusion; and retinopathy
87	H3581	Retinal detachments; defects; vascular occlusion; and retinopathy
87	H3582	Retinal detachments; defects; vascular occlusion; and retinopathy
87	H3589	Retinal detachments; defects; vascular occlusion; and retinopathy
87	H359	Retinal detachments; defects; vascular occlusion; and retinopathy
87	H36	Retinal detachments; defects; vascular occlusion; and retinopathy
88	H40001	Glaucoma
88	H40002	Glaucoma
88	H40003	Glaucoma
88	H40009	Glaucoma
88	H40011	Glaucoma
88	H40012	Glaucoma
88	H40013	Glaucoma
88	H40019	Glaucoma
88	H40021	Glaucoma
88	H40022	Glaucoma
88	H40023	Glaucoma
88	H40029	Glaucoma
88	H40031	Glaucoma
88	H40032	Glaucoma
88	H40033	Glaucoma
88	H40039	Glaucoma
88	H40041	Glaucoma
88	H40042	Glaucoma
88	H40043	Glaucoma
88	H40049	Glaucoma
88	H40051	Glaucoma
88	H40052	Glaucoma

88	H40053	Glaucoma
88	H40059	Glaucoma
88	H40061	Glaucoma
88	H40062	Glaucoma
88	H40063	Glaucoma
88	H40069	Glaucoma
88	H4010X0	Glaucoma
88	H4010X1	Glaucoma
88	H4010X2	Glaucoma
88	H4010X3	Glaucoma
88	H4010X4	Glaucoma
88	H4011X0	Glaucoma
88	H4011X1	Glaucoma
88	H4011X2	Glaucoma
88	H4011X3	Glaucoma
88	H4011X4	Glaucoma
88	H401210	Glaucoma
88	H401211	Glaucoma
88	H401212	Glaucoma
88	H401213	Glaucoma
88	H401214	Glaucoma
88	H401220	Glaucoma
88	H401221	Glaucoma
88	H401222	Glaucoma
88	H401223	Glaucoma
88	H401224	Glaucoma
88	H401230	Glaucoma
88	H401231	Glaucoma
88	H401232	Glaucoma
88	H401233	Glaucoma
88	H401234	Glaucoma
88	H401290	Glaucoma
88	H401291	Glaucoma
88	H401292	Glaucoma
88	H401293	Glaucoma
88	H401294	Glaucoma
88	H401310	Glaucoma
88	H401311	Glaucoma
88	H401312	Glaucoma
88	H401313	Glaucoma
88	H401314	Glaucoma
88	H401320	Glaucoma
88	H401321	Glaucoma
88	H401322	Glaucoma
88	H401323	Glaucoma
88	H401324	Glaucoma
88	H401330	Glaucoma
88	H401331	Glaucoma
88	H401332	Glaucoma
88	H401333	Glaucoma
88	H401334	Glaucoma

88	H401390	Glaucoma
88	H401391	Glaucoma
88	H401392	Glaucoma
88	H401393	Glaucoma
88	H401394	Glaucoma
88	H401410	Glaucoma
88	H401411	Glaucoma
88	H401412	Glaucoma
88	H401413	Glaucoma
88	H401414	Glaucoma
88	H401420	Glaucoma
88	H401421	Glaucoma
88	H401422	Glaucoma
88	H401423	Glaucoma
88	H401424	Glaucoma
88	H401430	Glaucoma
88	H401431	Glaucoma
88	H401432	Glaucoma
88	H401433	Glaucoma
88	H401434	Glaucoma
88	H401490	Glaucoma
88	H401491	Glaucoma
88	H401492	Glaucoma
88	H401493	Glaucoma
88	H401494	Glaucoma
88	H40151	Glaucoma
88	H40152	Glaucoma
88	H40153	Glaucoma
88	H40159	Glaucoma
88	H4020X0	Glaucoma
88	H4020X1	Glaucoma
88	H4020X2	Glaucoma
88	H4020X3	Glaucoma
88	H4020X4	Glaucoma
88	H40211	Glaucoma
88	H40212	Glaucoma
88	H40213	Glaucoma
88	H40219	Glaucoma
88	H402210	Glaucoma
88	H402211	Glaucoma
88	H402212	Glaucoma
88	H402213	Glaucoma
88	H402214	Glaucoma
88	H402220	Glaucoma
88	H402221	Glaucoma
88	H402222	Glaucoma
88	H402223	Glaucoma
88	H402224	Glaucoma
88	H402230	Glaucoma
88	H402231	Glaucoma
88	H402232	Glaucoma

88	H402233	Glaucoma
88	H402234	Glaucoma
88	H402290	Glaucoma
88	H402291	Glaucoma
88	H402292	Glaucoma
88	H402293	Glaucoma
88	H402294	Glaucoma
88	H40231	Glaucoma
88	H40232	Glaucoma
88	H40233	Glaucoma
88	H40239	Glaucoma
88	H40241	Glaucoma
88	H40242	Glaucoma
88	H40243	Glaucoma
88	H40249	Glaucoma
88	H4030X0	Glaucoma
88	H4030X1	Glaucoma
88	H4030X2	Glaucoma
88	H4030X3	Glaucoma
88	H4030X4	Glaucoma
88	H4031X0	Glaucoma
88	H4031X1	Glaucoma
88	H4031X2	Glaucoma
88	H4031X3	Glaucoma
88	H4031X4	Glaucoma
88	H4032X0	Glaucoma
88	H4032X1	Glaucoma
88	H4032X2	Glaucoma
88	H4032X3	Glaucoma
88	H4032X4	Glaucoma
88	H4033X0	Glaucoma
88	H4033X1	Glaucoma
88	H4033X2	Glaucoma
88	H4033X3	Glaucoma
88	H4033X4	Glaucoma
88	H4040X0	Glaucoma
88	H4040X1	Glaucoma
88	H4040X2	Glaucoma
88	H4040X3	Glaucoma
88	H4040X4	Glaucoma
88	H4041X0	Glaucoma
88	H4041X1	Glaucoma
88	H4041X2	Glaucoma
88	H4041X3	Glaucoma
88	H4041X4	Glaucoma
88	H4042X0	Glaucoma
88	H4042X1	Glaucoma
88	H4042X2	Glaucoma
88	H4042X3	Glaucoma
88	H4042X4	Glaucoma
88	H4043X0	Glaucoma

88	H4043X1	Glaucoma
88	H4043X2	Glaucoma
88	H4043X3	Glaucoma
88	H4043X4	Glaucoma
88	H4050X0	Glaucoma
88	H4050X1	Glaucoma
88	H4050X2	Glaucoma
88	H4050X3	Glaucoma
88	H4050X4	Glaucoma
88	H4051X0	Glaucoma
88	H4051X1	Glaucoma
88	H4051X2	Glaucoma
88	H4051X3	Glaucoma
88	H4051X4	Glaucoma
88	H4052X0	Glaucoma
88	H4052X1	Glaucoma
88	H4052X2	Glaucoma
88	H4052X3	Glaucoma
88	H4052X4	Glaucoma
88	H4053X0	Glaucoma
88	H4053X1	Glaucoma
88	H4053X2	Glaucoma
88	H4053X3	Glaucoma
88	H4053X4	Glaucoma
88	H4060X0	Glaucoma
88	H4060X1	Glaucoma
88	H4060X2	Glaucoma
88	H4060X3	Glaucoma
88	H4060X4	Glaucoma
88	H4061X0	Glaucoma
88	H4061X1	Glaucoma
88	H4061X2	Glaucoma
88	H4061X3	Glaucoma
88	H4061X4	Glaucoma
88	H4062X0	Glaucoma
88	H4062X1	Glaucoma
88	H4062X2	Glaucoma
88	H4062X3	Glaucoma
88	H4062X4	Glaucoma
88	H4063X0	Glaucoma
88	H4063X1	Glaucoma
88	H4063X2	Glaucoma
88	H4063X3	Glaucoma
88	H4063X4	Glaucoma
88	H40811	Glaucoma
88	H40812	Glaucoma
88	H40813	Glaucoma
88	H40819	Glaucoma
88	H40821	Glaucoma
88	H40822	Glaucoma
88	H40823	Glaucoma

88	H40829	Glaucoma
88	H40831	Glaucoma
88	H40832	Glaucoma
88	H40833	Glaucoma
88	H40839	Glaucoma
88	H4089	Glaucoma
88	H409	Glaucoma
88	H42	Glaucoma
89	H5200	Blindness and vision defects
89	H5201	Blindness and vision defects
89	H5202	Blindness and vision defects
89	H5203	Blindness and vision defects
89	H5210	Blindness and vision defects
89	H5211	Blindness and vision defects
89	H5212	Blindness and vision defects
89	H5213	Blindness and vision defects
89	H52201	Blindness and vision defects
89	H52202	Blindness and vision defects
89	H52203	Blindness and vision defects
89	H52209	Blindness and vision defects
89	H52211	Blindness and vision defects
89	H52212	Blindness and vision defects
89	H52213	Blindness and vision defects
89	H52219	Blindness and vision defects
89	H52221	Blindness and vision defects
89	H52222	Blindness and vision defects
89	H52223	Blindness and vision defects
89	H52229	Blindness and vision defects
89	H5231	Blindness and vision defects
89	H5232	Blindness and vision defects
89	H524	Blindness and vision defects
89	H52511	Blindness and vision defects
89	H52512	Blindness and vision defects
89	H52513	Blindness and vision defects
89	H52519	Blindness and vision defects
89	H52521	Blindness and vision defects
89	H52522	Blindness and vision defects
89	H52523	Blindness and vision defects
89	H52529	Blindness and vision defects
89	H52531	Blindness and vision defects
89	H52532	Blindness and vision defects
89	H52533	Blindness and vision defects
89	H52539	Blindness and vision defects
89	H526	Blindness and vision defects
89	H527	Blindness and vision defects
89	H53001	Blindness and vision defects
89	H53002	Blindness and vision defects
89	H53003	Blindness and vision defects
89	H53009	Blindness and vision defects
89	H53011	Blindness and vision defects
89	H53012	Blindness and vision defects

89	H53013	Blindness and vision defects
89	H53019	Blindness and vision defects
89	H53021	Blindness and vision defects
89	H53022	Blindness and vision defects
89	H53023	Blindness and vision defects
89	H53029	Blindness and vision defects
89	H53031	Blindness and vision defects
89	H53032	Blindness and vision defects
89	H53033	Blindness and vision defects
89	H53039	Blindness and vision defects
89	H5310	Blindness and vision defects
89	H5311	Blindness and vision defects
89	H53121	Blindness and vision defects
89	H53122	Blindness and vision defects
89	H53123	Blindness and vision defects
89	H53129	Blindness and vision defects
89	H53131	Blindness and vision defects
89	H53132	Blindness and vision defects
89	H53133	Blindness and vision defects
89	H53139	Blindness and vision defects
89	H53141	Blindness and vision defects
89	H53142	Blindness and vision defects
89	H53143	Blindness and vision defects
89	H53149	Blindness and vision defects
89	H5315	Blindness and vision defects
89	H5316	Blindness and vision defects
89	H5319	Blindness and vision defects
89	H532	Blindness and vision defects
89	H5330	Blindness and vision defects
89	H5331	Blindness and vision defects
89	H5332	Blindness and vision defects
89	H5333	Blindness and vision defects
89	H5334	Blindness and vision defects
89	H5340	Blindness and vision defects
89	H53411	Blindness and vision defects
89	H53412	Blindness and vision defects
89	H53413	Blindness and vision defects
89	H53419	Blindness and vision defects
89	H53421	Blindness and vision defects
89	H53422	Blindness and vision defects
89	H53423	Blindness and vision defects
89	H53429	Blindness and vision defects
89	H53431	Blindness and vision defects
89	H53432	Blindness and vision defects
89	H53433	Blindness and vision defects
89	H53439	Blindness and vision defects
89	H53451	Blindness and vision defects
89	H53452	Blindness and vision defects
89	H53453	Blindness and vision defects
89	H53459	Blindness and vision defects
89	H53461	Blindness and vision defects

89	H53462	Blindness and vision defects
89	H53469	Blindness and vision defects
89	H5347	Blindness and vision defects
89	H53481	Blindness and vision defects
89	H53482	Blindness and vision defects
89	H53483	Blindness and vision defects
89	H53489	Blindness and vision defects
89	H5350	Blindness and vision defects
89	H5351	Blindness and vision defects
89	H5352	Blindness and vision defects
89	H5353	Blindness and vision defects
89	H5354	Blindness and vision defects
89	H5355	Blindness and vision defects
89	H5359	Blindness and vision defects
89	H5360	Blindness and vision defects
89	H5361	Blindness and vision defects
89	H5362	Blindness and vision defects
89	H5363	Blindness and vision defects
89	H5369	Blindness and vision defects
89	H5371	Blindness and vision defects
89	H5372	Blindness and vision defects
89	H538	Blindness and vision defects
89	H539	Blindness and vision defects
89	H540	Blindness and vision defects
89	H5410	Blindness and vision defects
89	H5411	Blindness and vision defects
89	H5412	Blindness and vision defects
89	H542	Blindness and vision defects
89	H543	Blindness and vision defects
89	H5440	Blindness and vision defects
89	H5441	Blindness and vision defects
89	H5442	Blindness and vision defects
89	H5450	Blindness and vision defects
89	H5451	Blindness and vision defects
89	H5452	Blindness and vision defects
89	H5460	Blindness and vision defects
89	H5461	Blindness and vision defects
89	H5462	Blindness and vision defects
89	H547	Blindness and vision defects
89	H548	Blindness and vision defects
89	R441	Blindness and vision defects
89	R483	Blindness and vision defects
89	Z973	Blindness and vision defects
90	A211	Inflammation; infection of eye (except that caused by tuberculosis or sexually transmitteddisease)
90	A3686	Inflammation; infection of eye (except that caused by tuberculosis or sexually transmitteddisease)
90	A710	Inflammation; infection of eye (except that caused by tuberculosis or sexually transmitteddisease)
90	A711	Inflammation; infection of eye (except that caused by tuberculosis or sexually transmitteddisease)
90	A719	Inflammation; infection of eye (except that caused by tuberculosis or sexually transmitteddisease)
90	A740	Inflammation; infection of eye (except that caused by tuberculosis or sexually transmitteddisease)
90	B0050	Inflammation; infection of eye (except that caused by tuberculosis or sexually transmitteddisease)
90	B0051	Inflammation; infection of eye (except that caused by tuberculosis or sexually transmitteddisease)

90	B0052	Inflammation; infection of eye (except that caused by tuberculosis or sexually transmitteddisease)
90	B0053	Inflammation; infection of eye (except that caused by tuberculosis or sexually transmitteddisease)
90	B0059	Inflammation; infection of eye (except that caused by tuberculosis or sexually transmitteddisease)
90	B0230	Inflammation; infection of eye (except that caused by tuberculosis or sexually transmitteddisease)
90	B0231	Inflammation; infection of eye (except that caused by tuberculosis or sexually transmitteddisease)
90	B0232	Inflammation; infection of eye (except that caused by tuberculosis or sexually transmitteddisease)
90	B0233	Inflammation; infection of eye (except that caused by tuberculosis or sexually transmitteddisease)
90	B0234	Inflammation; infection of eye (except that caused by tuberculosis or sexually transmitteddisease)
90	B0239	Inflammation; infection of eye (except that caused by tuberculosis or sexually transmitteddisease)
90	B0581	Inflammation; infection of eye (except that caused by tuberculosis or sexually transmitteddisease)
90	B300	Inflammation; infection of eye (except that caused by tuberculosis or sexually transmitteddisease)
90	B301	Inflammation; infection of eye (except that caused by tuberculosis or sexually transmitteddisease)
90	B302	Inflammation; infection of eye (except that caused by tuberculosis or sexually transmitteddisease)
90	B303	Inflammation; infection of eye (except that caused by tuberculosis or sexually transmitteddisease)
90	B308	Inflammation; infection of eye (except that caused by tuberculosis or sexually transmitteddisease)
90	B309	Inflammation; infection of eye (except that caused by tuberculosis or sexually transmitteddisease)
90	B5801	Inflammation; infection of eye (except that caused by tuberculosis or sexually transmitteddisease)
90	B5809	Inflammation; infection of eye (except that caused by tuberculosis or sexually transmitteddisease)
90	B940	Inflammation; infection of eye (except that caused by tuberculosis or sexually transmitteddisease)
90	H00011	Inflammation; infection of eye (except that caused by tuberculosis or sexually transmitteddisease)
90	H00012	Inflammation; infection of eye (except that caused by tuberculosis or sexually transmitteddisease)
90	H00013	Inflammation; infection of eye (except that caused by tuberculosis or sexually transmitteddisease)
90	H00014	Inflammation; infection of eye (except that caused by tuberculosis or sexually transmitteddisease)
90	H00015	Inflammation; infection of eye (except that caused by tuberculosis or sexually transmitteddisease)
90	H00016	Inflammation; infection of eye (except that caused by tuberculosis or sexually transmitteddisease)
90	H00019	Inflammation; infection of eye (except that caused by tuberculosis or sexually transmitteddisease)
90	H00021	Inflammation; infection of eye (except that caused by tuberculosis or sexually transmitteddisease)
90	H00022	Inflammation; infection of eye (except that caused by tuberculosis or sexually transmitteddisease)
90	H00023	Inflammation; infection of eye (except that caused by tuberculosis or sexually transmitteddisease)
90	H00024	Inflammation; infection of eye (except that caused by tuberculosis or sexually transmitteddisease)
90	H00025	Inflammation; infection of eye (except that caused by tuberculosis or sexually transmitteddisease)
90	H00026	Inflammation; infection of eye (except that caused by tuberculosis or sexually transmitteddisease)
90	H00029	Inflammation; infection of eye (except that caused by tuberculosis or sexually transmitteddisease)
90	H00031	Inflammation; infection of eye (except that caused by tuberculosis or sexually transmitteddisease)
90	H00032	Inflammation; infection of eye (except that caused by tuberculosis or sexually transmitteddisease)
90	H00033	Inflammation; infection of eye (except that caused by tuberculosis or sexually transmitteddisease)
90	H00034	Inflammation; infection of eye (except that caused by tuberculosis or sexually transmitteddisease)
90	H00035	Inflammation; infection of eye (except that caused by tuberculosis or sexually transmitteddisease)
90	H00036	Inflammation; infection of eye (except that caused by tuberculosis or sexually transmitteddisease)
90	H00039	Inflammation; infection of eye (except that caused by tuberculosis or sexually transmitteddisease)
90	H01001	Inflammation; infection of eye (except that caused by tuberculosis or sexually transmitteddisease)
90	H01002	Inflammation; infection of eye (except that caused by tuberculosis or sexually transmitteddisease)
90	H01003	Inflammation; infection of eye (except that caused by tuberculosis or sexually transmitteddisease)
90	H01004	Inflammation; infection of eye (except that caused by tuberculosis or sexually transmitteddisease)
90	H01005	Inflammation; infection of eye (except that caused by tuberculosis or sexually transmitteddisease)
90	H01006	Inflammation; infection of eye (except that caused by tuberculosis or sexually transmitteddisease)
90	H01009	Inflammation; infection of eye (except that caused by tuberculosis or sexually transmitteddisease)
90	H01011	Inflammation; infection of eye (except that caused by tuberculosis or sexually transmitteddisease)
90	H01012	Inflammation; infection of eye (except that caused by tuberculosis or sexually transmitteddisease)
90	H01013	Inflammation; infection of eye (except that caused by tuberculosis or sexually transmitteddisease)
90	H01014	Inflammation; infection of eye (except that caused by tuberculosis or sexually transmitteddisease)

90	H01015	Inflammation; infection of eye (except that caused by tuberculosis or sexually transmitteddisease)
90	H01016	Inflammation; infection of eye (except that caused by tuberculosis or sexually transmitteddisease)
90	H01019	Inflammation; infection of eye (except that caused by tuberculosis or sexually transmitteddisease)
90	H01021	Inflammation; infection of eye (except that caused by tuberculosis or sexually transmitteddisease)
90	H01022	Inflammation; infection of eye (except that caused by tuberculosis or sexually transmitteddisease)
90	H01023	Inflammation; infection of eye (except that caused by tuberculosis or sexually transmitteddisease)
90	H01024	Inflammation; infection of eye (except that caused by tuberculosis or sexually transmitteddisease)
90	H01025	Inflammation; infection of eye (except that caused by tuberculosis or sexually transmitteddisease)
90	H01026	Inflammation; infection of eye (except that caused by tuberculosis or sexually transmitteddisease)
90	H01029	Inflammation; infection of eye (except that caused by tuberculosis or sexually transmitteddisease)
90	H01111	Inflammation; infection of eye (except that caused by tuberculosis or sexually transmitteddisease)
90	H01112	Inflammation; infection of eye (except that caused by tuberculosis or sexually transmitteddisease)
90	H01113	Inflammation; infection of eye (except that caused by tuberculosis or sexually transmitteddisease)
90	H01114	Inflammation; infection of eye (except that caused by tuberculosis or sexually transmitteddisease)
90	H01115	Inflammation; infection of eye (except that caused by tuberculosis or sexually transmitteddisease)
90	H01116	Inflammation; infection of eye (except that caused by tuberculosis or sexually transmitteddisease)
90	H01119	Inflammation; infection of eye (except that caused by tuberculosis or sexually transmitteddisease)
90	H01121	Inflammation; infection of eye (except that caused by tuberculosis or sexually transmitteddisease)
90	H01122	Inflammation; infection of eye (except that caused by tuberculosis or sexually transmitteddisease)
90	H01123	Inflammation; infection of eye (except that caused by tuberculosis or sexually transmitteddisease)
90	H01124	Inflammation; infection of eye (except that caused by tuberculosis or sexually transmitteddisease)
90	H01125	Inflammation; infection of eye (except that caused by tuberculosis or sexually transmitteddisease)
90	H01126	Inflammation; infection of eye (except that caused by tuberculosis or sexually transmitteddisease)
90	H01129	Inflammation; infection of eye (except that caused by tuberculosis or sexually transmitteddisease)
90	H01131	Inflammation; infection of eye (except that caused by tuberculosis or sexually transmitteddisease)
90	H01132	Inflammation; infection of eye (except that caused by tuberculosis or sexually transmitteddisease)
90	H01133	Inflammation; infection of eye (except that caused by tuberculosis or sexually transmitteddisease)
90	H01134	Inflammation; infection of eye (except that caused by tuberculosis or sexually transmitteddisease)
90	H01135	Inflammation; infection of eye (except that caused by tuberculosis or sexually transmitteddisease)
90	H01136	Inflammation; infection of eye (except that caused by tuberculosis or sexually transmitteddisease)
90	H01139	Inflammation; infection of eye (except that caused by tuberculosis or sexually transmitteddisease)
90	H01141	Inflammation; infection of eye (except that caused by tuberculosis or sexually transmitteddisease)
90	H01142	Inflammation; infection of eye (except that caused by tuberculosis or sexually transmitteddisease)
90	H01143	Inflammation; infection of eye (except that caused by tuberculosis or sexually transmitteddisease)
90	H01144	Inflammation; infection of eye (except that caused by tuberculosis or sexually transmitteddisease)
90	H01145	Inflammation; infection of eye (except that caused by tuberculosis or sexually transmitteddisease)
90	H01146	Inflammation; infection of eye (except that caused by tuberculosis or sexually transmitteddisease)
90	H01149	Inflammation; infection of eye (except that caused by tuberculosis or sexually transmitteddisease)
90	H018	Inflammation; infection of eye (except that caused by tuberculosis or sexually transmitteddisease)
90	H019	Inflammation; infection of eye (except that caused by tuberculosis or sexually transmitteddisease)
90	H04001	Inflammation; infection of eye (except that caused by tuberculosis or sexually transmitteddisease)
90	H04002	Inflammation; infection of eye (except that caused by tuberculosis or sexually transmitteddisease)
90	H04003	Inflammation; infection of eye (except that caused by tuberculosis or sexually transmitteddisease)
90	H04009	Inflammation; infection of eye (except that caused by tuberculosis or sexually transmitteddisease)
90	H04011	Inflammation; infection of eye (except that caused by tuberculosis or sexually transmitteddisease)
90	H04012	Inflammation; infection of eye (except that caused by tuberculosis or sexually transmitteddisease)
90	H04013	Inflammation; infection of eye (except that caused by tuberculosis or sexually transmitteddisease)
90	H04019	Inflammation; infection of eye (except that caused by tuberculosis or sexually transmitteddisease)
90	H04021	Inflammation; infection of eye (except that caused by tuberculosis or sexually transmitteddisease)
90	H04022	Inflammation; infection of eye (except that caused by tuberculosis or sexually transmitteddisease)
90	H04023	Inflammation; infection of eye (except that caused by tuberculosis or sexually transmitteddisease)

90	H04029	Inflammation; infection of eye (except that caused by tuberculosis or sexually transmitteddisease)
90	H04031	Inflammation; infection of eye (except that caused by tuberculosis or sexually transmitteddisease)
90	H04032	Inflammation; infection of eye (except that caused by tuberculosis or sexually transmitteddisease)
90	H04033	Inflammation; infection of eye (except that caused by tuberculosis or sexually transmitteddisease)
90	H04039	Inflammation; infection of eye (except that caused by tuberculosis or sexually transmitteddisease)
90	H04301	Inflammation; infection of eye (except that caused by tuberculosis or sexually transmitteddisease)
90	H04302	Inflammation; infection of eye (except that caused by tuberculosis or sexually transmitteddisease)
90	H04303	Inflammation; infection of eye (except that caused by tuberculosis or sexually transmitteddisease)
90	H04309	Inflammation; infection of eye (except that caused by tuberculosis or sexually transmitteddisease)
90	H04311	Inflammation; infection of eye (except that caused by tuberculosis or sexually transmitteddisease)
90	H04312	Inflammation; infection of eye (except that caused by tuberculosis or sexually transmitteddisease)
90	H04313	Inflammation; infection of eye (except that caused by tuberculosis or sexually transmitteddisease)
90	H04319	Inflammation; infection of eye (except that caused by tuberculosis or sexually transmitteddisease)
90	H04321	Inflammation; infection of eye (except that caused by tuberculosis or sexually transmitteddisease)
90	H04322	Inflammation; infection of eye (except that caused by tuberculosis or sexually transmitteddisease)
90	H04323	Inflammation; infection of eye (except that caused by tuberculosis or sexually transmitteddisease)
90	H04329	Inflammation; infection of eye (except that caused by tuberculosis or sexually transmitteddisease)
90	H04331	Inflammation; infection of eye (except that caused by tuberculosis or sexually transmitteddisease)
90	H04332	Inflammation; infection of eye (except that caused by tuberculosis or sexually transmitteddisease)
90	H04333	Inflammation; infection of eye (except that caused by tuberculosis or sexually transmitteddisease)
90	H04339	Inflammation; infection of eye (except that caused by tuberculosis or sexually transmitteddisease)
90	H04411	Inflammation; infection of eye (except that caused by tuberculosis or sexually transmitteddisease)
90	H04412	Inflammation; infection of eye (except that caused by tuberculosis or sexually transmitteddisease)
90	H04413	Inflammation; infection of eye (except that caused by tuberculosis or sexually transmitteddisease)
90	H04419	Inflammation; infection of eye (except that caused by tuberculosis or sexually transmitteddisease)
90	H04421	Inflammation; infection of eye (except that caused by tuberculosis or sexually transmitteddisease)
90	H04422	Inflammation; infection of eye (except that caused by tuberculosis or sexually transmitteddisease)
90	H04423	Inflammation; infection of eye (except that caused by tuberculosis or sexually transmitteddisease)
90	H04429	Inflammation; infection of eye (except that caused by tuberculosis or sexually transmitteddisease)
90	H04431	Inflammation; infection of eye (except that caused by tuberculosis or sexually transmitteddisease)
90	H04432	Inflammation; infection of eye (except that caused by tuberculosis or sexually transmitteddisease)
90	H04433	Inflammation; infection of eye (except that caused by tuberculosis or sexually transmitteddisease)
90	H04439	Inflammation; infection of eye (except that caused by tuberculosis or sexually transmitteddisease)
90	H0500	Inflammation; infection of eye (except that caused by tuberculosis or sexually transmitteddisease)
90	H05011	Inflammation; infection of eye (except that caused by tuberculosis or sexually transmitteddisease)
90	H05012	Inflammation; infection of eye (except that caused by tuberculosis or sexually transmitteddisease)
90	H05013	Inflammation; infection of eye (except that caused by tuberculosis or sexually transmitteddisease)
90	H05019	Inflammation; infection of eye (except that caused by tuberculosis or sexually transmitteddisease)
90	H05021	Inflammation; infection of eye (except that caused by tuberculosis or sexually transmitteddisease)
90	H05022	Inflammation; infection of eye (except that caused by tuberculosis or sexually transmitteddisease)
90	H05023	Inflammation; infection of eye (except that caused by tuberculosis or sexually transmitteddisease)
90	H05029	Inflammation; infection of eye (except that caused by tuberculosis or sexually transmitteddisease)
90	H05031	Inflammation; infection of eye (except that caused by tuberculosis or sexually transmitteddisease)
90	H05032	Inflammation; infection of eye (except that caused by tuberculosis or sexually transmitteddisease)
90	H05033	Inflammation; infection of eye (except that caused by tuberculosis or sexually transmitteddisease)
90	H05039	Inflammation; infection of eye (except that caused by tuberculosis or sexually transmitteddisease)
90	H05041	Inflammation; infection of eye (except that caused by tuberculosis or sexually transmitteddisease)
90	H05042	Inflammation; infection of eye (except that caused by tuberculosis or sexually transmitteddisease)
90	H05043	Inflammation; infection of eye (except that caused by tuberculosis or sexually transmitteddisease)
90	H05049	Inflammation; infection of eye (except that caused by tuberculosis or sexually transmitteddisease)
90	H0510	Inflammation; infection of eye (except that caused by tuberculosis or sexually transmitteddisease)

90	H05111	Inflammation; infection of eye (except that caused by tuberculosis or sexually transmitteddisease)
90	H05112	Inflammation; infection of eye (except that caused by tuberculosis or sexually transmitteddisease)
90	H05113	Inflammation; infection of eye (except that caused by tuberculosis or sexually transmitteddisease)
90	H05119	Inflammation; infection of eye (except that caused by tuberculosis or sexually transmitteddisease)
90	H05121	Inflammation; infection of eye (except that caused by tuberculosis or sexually transmitteddisease)
90	H05122	Inflammation; infection of eye (except that caused by tuberculosis or sexually transmitteddisease)
90	H05123	Inflammation; infection of eye (except that caused by tuberculosis or sexually transmitteddisease)
90	H05129	Inflammation; infection of eye (except that caused by tuberculosis or sexually transmitteddisease)
90	H10011	Inflammation; infection of eye (except that caused by tuberculosis or sexually transmitteddisease)
90	H10012	Inflammation; infection of eye (except that caused by tuberculosis or sexually transmitteddisease)
90	H10013	Inflammation; infection of eye (except that caused by tuberculosis or sexually transmitteddisease)
90	H10019	Inflammation; infection of eye (except that caused by tuberculosis or sexually transmitteddisease)
90	H10021	Inflammation; infection of eye (except that caused by tuberculosis or sexually transmitteddisease)
90	H10022	Inflammation; infection of eye (except that caused by tuberculosis or sexually transmitteddisease)
90	H10023	Inflammation; infection of eye (except that caused by tuberculosis or sexually transmitteddisease)
90	H10029	Inflammation; infection of eye (except that caused by tuberculosis or sexually transmitteddisease)
90	H1010	Inflammation; infection of eye (except that caused by tuberculosis or sexually transmitteddisease)
90	H1011	Inflammation; infection of eye (except that caused by tuberculosis or sexually transmitteddisease)
90	H1012	Inflammation; infection of eye (except that caused by tuberculosis or sexually transmitteddisease)
90	H1013	Inflammation; infection of eye (except that caused by tuberculosis or sexually transmitteddisease)
90	H10211	Inflammation; infection of eye (except that caused by tuberculosis or sexually transmitteddisease)
90	H10212	Inflammation; infection of eye (except that caused by tuberculosis or sexually transmitteddisease)
90	H10213	Inflammation; infection of eye (except that caused by tuberculosis or sexually transmitteddisease)
90	H10219	Inflammation; infection of eye (except that caused by tuberculosis or sexually transmitteddisease)
90	H10221	Inflammation; infection of eye (except that caused by tuberculosis or sexually transmitteddisease)
90	H10222	Inflammation; infection of eye (except that caused by tuberculosis or sexually transmitteddisease)
90	H10223	Inflammation; infection of eye (except that caused by tuberculosis or sexually transmitteddisease)
90	H10229	Inflammation; infection of eye (except that caused by tuberculosis or sexually transmitteddisease)
90	H10231	Inflammation; infection of eye (except that caused by tuberculosis or sexually transmitteddisease)
90	H10232	Inflammation; infection of eye (except that caused by tuberculosis or sexually transmitteddisease)
90	H10233	Inflammation; infection of eye (except that caused by tuberculosis or sexually transmitteddisease)
90	H10239	Inflammation; infection of eye (except that caused by tuberculosis or sexually transmitteddisease)
90	H1030	Inflammation; infection of eye (except that caused by tuberculosis or sexually transmitteddisease)
90	H1031	Inflammation; infection of eye (except that caused by tuberculosis or sexually transmitteddisease)
90	H1032	Inflammation; infection of eye (except that caused by tuberculosis or sexually transmitteddisease)
90	H1033	Inflammation; infection of eye (except that caused by tuberculosis or sexually transmitteddisease)
90	H10401	Inflammation; infection of eye (except that caused by tuberculosis or sexually transmitteddisease)
90	H10402	Inflammation; infection of eye (except that caused by tuberculosis or sexually transmitteddisease)
90	H10403	Inflammation; infection of eye (except that caused by tuberculosis or sexually transmitteddisease)
90	H10409	Inflammation; infection of eye (except that caused by tuberculosis or sexually transmitteddisease)
90	H10411	Inflammation; infection of eye (except that caused by tuberculosis or sexually transmitteddisease)
90	H10412	Inflammation; infection of eye (except that caused by tuberculosis or sexually transmitteddisease)
90	H10413	Inflammation; infection of eye (except that caused by tuberculosis or sexually transmitteddisease)
90	H10419	Inflammation; infection of eye (except that caused by tuberculosis or sexually transmitteddisease)
90	H10421	Inflammation; infection of eye (except that caused by tuberculosis or sexually transmitteddisease)
90	H10422	Inflammation; infection of eye (except that caused by tuberculosis or sexually transmitteddisease)
90	H10423	Inflammation; infection of eye (except that caused by tuberculosis or sexually transmitteddisease)
90	H10429	Inflammation; infection of eye (except that caused by tuberculosis or sexually transmitteddisease)
90	H10431	Inflammation; infection of eye (except that caused by tuberculosis or sexually transmitteddisease)
90	H10432	Inflammation; infection of eye (except that caused by tuberculosis or sexually transmitteddisease)
90	H10433	Inflammation; infection of eye (except that caused by tuberculosis or sexually transmitteddisease)

90	H10439	Inflammation; infection of eye (except that caused by tuberculosis or sexually transmitteddisease)
90	H1044	Inflammation; infection of eye (except that caused by tuberculosis or sexually transmitteddisease)
90	H1045	Inflammation; infection of eye (except that caused by tuberculosis or sexually transmitteddisease)
90	H10501	Inflammation; infection of eye (except that caused by tuberculosis or sexually transmitteddisease)
90	H10502	Inflammation; infection of eye (except that caused by tuberculosis or sexually transmitteddisease)
90	H10503	Inflammation; infection of eye (except that caused by tuberculosis or sexually transmitteddisease)
90	H10509	Inflammation; infection of eye (except that caused by tuberculosis or sexually transmitteddisease)
90	H10511	Inflammation; infection of eye (except that caused by tuberculosis or sexually transmitteddisease)
90	H10512	Inflammation; infection of eye (except that caused by tuberculosis or sexually transmitteddisease)
90	H10513	Inflammation; infection of eye (except that caused by tuberculosis or sexually transmitteddisease)
90	H10519	Inflammation; infection of eye (except that caused by tuberculosis or sexually transmitteddisease)
90	H10521	Inflammation; infection of eye (except that caused by tuberculosis or sexually transmitteddisease)
90	H10522	Inflammation; infection of eye (except that caused by tuberculosis or sexually transmitteddisease)
90	H10523	Inflammation; infection of eye (except that caused by tuberculosis or sexually transmitteddisease)
90	H10529	Inflammation; infection of eye (except that caused by tuberculosis or sexually transmitteddisease)
90	H10531	Inflammation; infection of eye (except that caused by tuberculosis or sexually transmitteddisease)
90	H10532	Inflammation; infection of eye (except that caused by tuberculosis or sexually transmitteddisease)
90	H10533	Inflammation; infection of eye (except that caused by tuberculosis or sexually transmitteddisease)
90	H10539	Inflammation; infection of eye (except that caused by tuberculosis or sexually transmitteddisease)
90	H1089	Inflammation; infection of eye (except that caused by tuberculosis or sexually transmitteddisease)
90	H109	Inflammation; infection of eye (except that caused by tuberculosis or sexually transmitteddisease)
90	H15001	Inflammation; infection of eye (except that caused by tuberculosis or sexually transmitteddisease)
90	H15002	Inflammation; infection of eye (except that caused by tuberculosis or sexually transmitteddisease)
90	H15003	Inflammation; infection of eye (except that caused by tuberculosis or sexually transmitteddisease)
90	H15009	Inflammation; infection of eye (except that caused by tuberculosis or sexually transmitteddisease)
90	H15011	Inflammation; infection of eye (except that caused by tuberculosis or sexually transmitteddisease)
90	H15012	Inflammation; infection of eye (except that caused by tuberculosis or sexually transmitteddisease)
90	H15013	Inflammation; infection of eye (except that caused by tuberculosis or sexually transmitteddisease)
90	H15019	Inflammation; infection of eye (except that caused by tuberculosis or sexually transmitteddisease)
90	H15021	Inflammation; infection of eye (except that caused by tuberculosis or sexually transmitteddisease)
90	H15022	Inflammation; infection of eye (except that caused by tuberculosis or sexually transmitteddisease)
90	H15023	Inflammation; infection of eye (except that caused by tuberculosis or sexually transmitteddisease)
90	H15029	Inflammation; infection of eye (except that caused by tuberculosis or sexually transmitteddisease)
90	H15031	Inflammation; infection of eye (except that caused by tuberculosis or sexually transmitteddisease)
90	H15032	Inflammation; infection of eye (except that caused by tuberculosis or sexually transmitteddisease)
90	H15033	Inflammation; infection of eye (except that caused by tuberculosis or sexually transmitteddisease)
90	H15039	Inflammation; infection of eye (except that caused by tuberculosis or sexually transmitteddisease)
90	H15041	Inflammation; infection of eye (except that caused by tuberculosis or sexually transmitteddisease)
90	H15042	Inflammation; infection of eye (except that caused by tuberculosis or sexually transmitteddisease)
90	H15043	Inflammation; infection of eye (except that caused by tuberculosis or sexually transmitteddisease)
90	H15049	Inflammation; infection of eye (except that caused by tuberculosis or sexually transmitteddisease)
90	H15051	Inflammation; infection of eye (except that caused by tuberculosis or sexually transmitteddisease)
90	H15052	Inflammation; infection of eye (except that caused by tuberculosis or sexually transmitteddisease)
90	H15053	Inflammation; infection of eye (except that caused by tuberculosis or sexually transmitteddisease)
90	H15059	Inflammation; infection of eye (except that caused by tuberculosis or sexually transmitteddisease)
90	H15091	Inflammation; infection of eye (except that caused by tuberculosis or sexually transmitteddisease)
90	H15092	Inflammation; infection of eye (except that caused by tuberculosis or sexually transmitteddisease)
90	H15093	Inflammation; infection of eye (except that caused by tuberculosis or sexually transmitteddisease)
90	H15099	Inflammation; infection of eye (except that caused by tuberculosis or sexually transmitteddisease)
90	H15101	Inflammation; infection of eye (except that caused by tuberculosis or sexually transmitteddisease)
90	H15102	Inflammation; infection of eye (except that caused by tuberculosis or sexually transmitteddisease)

90	H15103	Inflammation; infection of eye (except that caused by tuberculosis or sexually transmitteddisease)
90	H15109	Inflammation; infection of eye (except that caused by tuberculosis or sexually transmitteddisease)
90	H15111	Inflammation; infection of eye (except that caused by tuberculosis or sexually transmitteddisease)
90	H15112	Inflammation; infection of eye (except that caused by tuberculosis or sexually transmitteddisease)
90	H15113	Inflammation; infection of eye (except that caused by tuberculosis or sexually transmitteddisease)
90	H15119	Inflammation; infection of eye (except that caused by tuberculosis or sexually transmitteddisease)
90	H15121	Inflammation; infection of eye (except that caused by tuberculosis or sexually transmitteddisease)
90	H15122	Inflammation; infection of eye (except that caused by tuberculosis or sexually transmitteddisease)
90	H15123	Inflammation; infection of eye (except that caused by tuberculosis or sexually transmitteddisease)
90	H15129	Inflammation; infection of eye (except that caused by tuberculosis or sexually transmitteddisease)
90	H16101	Inflammation; infection of eye (except that caused by tuberculosis or sexually transmitteddisease)
90	H16102	Inflammation; infection of eye (except that caused by tuberculosis or sexually transmitteddisease)
90	H16103	Inflammation; infection of eye (except that caused by tuberculosis or sexually transmitteddisease)
90	H16109	Inflammation; infection of eye (except that caused by tuberculosis or sexually transmitteddisease)
90	H16111	Inflammation; infection of eye (except that caused by tuberculosis or sexually transmitteddisease)
90	H16112	Inflammation; infection of eye (except that caused by tuberculosis or sexually transmitteddisease)
90	H16113	Inflammation; infection of eye (except that caused by tuberculosis or sexually transmitteddisease)
90	H16119	Inflammation; infection of eye (except that caused by tuberculosis or sexually transmitteddisease)
90	H16121	Inflammation; infection of eye (except that caused by tuberculosis or sexually transmitteddisease)
90	H16122	Inflammation; infection of eye (except that caused by tuberculosis or sexually transmitteddisease)
90	H16123	Inflammation; infection of eye (except that caused by tuberculosis or sexually transmitteddisease)
90	H16129	Inflammation; infection of eye (except that caused by tuberculosis or sexually transmitteddisease)
90	H16131	Inflammation; infection of eye (except that caused by tuberculosis or sexually transmitteddisease)
90	H16132	Inflammation; infection of eye (except that caused by tuberculosis or sexually transmitteddisease)
90	H16133	Inflammation; infection of eye (except that caused by tuberculosis or sexually transmitteddisease)
90	H16139	Inflammation; infection of eye (except that caused by tuberculosis or sexually transmitteddisease)
90	H16141	Inflammation; infection of eye (except that caused by tuberculosis or sexually transmitteddisease)
90	H16142	Inflammation; infection of eye (except that caused by tuberculosis or sexually transmitteddisease)
90	H16143	Inflammation; infection of eye (except that caused by tuberculosis or sexually transmitteddisease)
90	H16149	Inflammation; infection of eye (except that caused by tuberculosis or sexually transmitteddisease)
90	H16201	Inflammation; infection of eye (except that caused by tuberculosis or sexually transmitteddisease)
90	H16202	Inflammation; infection of eye (except that caused by tuberculosis or sexually transmitteddisease)
90	H16203	Inflammation; infection of eye (except that caused by tuberculosis or sexually transmitteddisease)
90	H16209	Inflammation; infection of eye (except that caused by tuberculosis or sexually transmitteddisease)
90	H16211	Inflammation; infection of eye (except that caused by tuberculosis or sexually transmitteddisease)
90	H16212	Inflammation; infection of eye (except that caused by tuberculosis or sexually transmitteddisease)
90	H16213	Inflammation; infection of eye (except that caused by tuberculosis or sexually transmitteddisease)
90	H16219	Inflammation; infection of eye (except that caused by tuberculosis or sexually transmitteddisease)
90	H16221	Inflammation; infection of eye (except that caused by tuberculosis or sexually transmitteddisease)
90	H16222	Inflammation; infection of eye (except that caused by tuberculosis or sexually transmitteddisease)
90	H16223	Inflammation; infection of eye (except that caused by tuberculosis or sexually transmitteddisease)
90	H16229	Inflammation; infection of eye (except that caused by tuberculosis or sexually transmitteddisease)
90	H16231	Inflammation; infection of eye (except that caused by tuberculosis or sexually transmitteddisease)
90	H16232	Inflammation; infection of eye (except that caused by tuberculosis or sexually transmitteddisease)
90	H16233	Inflammation; infection of eye (except that caused by tuberculosis or sexually transmitteddisease)
90	H16239	Inflammation; infection of eye (except that caused by tuberculosis or sexually transmitteddisease)
90	H16241	Inflammation; infection of eye (except that caused by tuberculosis or sexually transmitteddisease)
90	H16242	Inflammation; infection of eye (except that caused by tuberculosis or sexually transmitteddisease)
90	H16243	Inflammation; infection of eye (except that caused by tuberculosis or sexually transmitteddisease)
90	H16249	Inflammation; infection of eye (except that caused by tuberculosis or sexually transmitteddisease)
90	H16251	Inflammation; infection of eye (except that caused by tuberculosis or sexually transmitteddisease)

90	H16252	Inflammation; infection of eye (except that caused by tuberculosis or sexually transmitteddisease)
90	H16253	Inflammation; infection of eye (except that caused by tuberculosis or sexually transmitteddisease)
90	H16259	Inflammation; infection of eye (except that caused by tuberculosis or sexually transmitteddisease)
90	H16261	Inflammation; infection of eye (except that caused by tuberculosis or sexually transmitteddisease)
90	H16262	Inflammation; infection of eye (except that caused by tuberculosis or sexually transmitteddisease)
90	H16263	Inflammation; infection of eye (except that caused by tuberculosis or sexually transmitteddisease)
90	H16269	Inflammation; infection of eye (except that caused by tuberculosis or sexually transmitteddisease)
90	H16291	Inflammation; infection of eye (except that caused by tuberculosis or sexually transmitteddisease)
90	H16292	Inflammation; infection of eye (except that caused by tuberculosis or sexually transmitteddisease)
90	H16293	Inflammation; infection of eye (except that caused by tuberculosis or sexually transmitteddisease)
90	H16299	Inflammation; infection of eye (except that caused by tuberculosis or sexually transmitteddisease)
90	H16301	Inflammation; infection of eye (except that caused by tuberculosis or sexually transmitteddisease)
90	H16302	Inflammation; infection of eye (except that caused by tuberculosis or sexually transmitteddisease)
90	H16303	Inflammation; infection of eye (except that caused by tuberculosis or sexually transmitteddisease)
90	H16309	Inflammation; infection of eye (except that caused by tuberculosis or sexually transmitteddisease)
90	H16311	Inflammation; infection of eye (except that caused by tuberculosis or sexually transmitteddisease)
90	H16312	Inflammation; infection of eye (except that caused by tuberculosis or sexually transmitteddisease)
90	H16313	Inflammation; infection of eye (except that caused by tuberculosis or sexually transmitteddisease)
90	H16319	Inflammation; infection of eye (except that caused by tuberculosis or sexually transmitteddisease)
90	H16321	Inflammation; infection of eye (except that caused by tuberculosis or sexually transmitteddisease)
90	H16322	Inflammation; infection of eye (except that caused by tuberculosis or sexually transmitteddisease)
90	H16323	Inflammation; infection of eye (except that caused by tuberculosis or sexually transmitteddisease)
90	H16329	Inflammation; infection of eye (except that caused by tuberculosis or sexually transmitteddisease)
90	H16331	Inflammation; infection of eye (except that caused by tuberculosis or sexually transmitteddisease)
90	H16332	Inflammation; infection of eye (except that caused by tuberculosis or sexually transmitteddisease)
90	H16333	Inflammation; infection of eye (except that caused by tuberculosis or sexually transmitteddisease)
90	H16339	Inflammation; infection of eye (except that caused by tuberculosis or sexually transmitteddisease)
90	H16391	Inflammation; infection of eye (except that caused by tuberculosis or sexually transmitteddisease)
90	H16392	Inflammation; infection of eye (except that caused by tuberculosis or sexually transmitteddisease)
90	H16393	Inflammation; infection of eye (except that caused by tuberculosis or sexually transmitteddisease)
90	H16399	Inflammation; infection of eye (except that caused by tuberculosis or sexually transmitteddisease)
90	H168	Inflammation; infection of eye (except that caused by tuberculosis or sexually transmitteddisease)
90	H169	Inflammation; infection of eye (except that caused by tuberculosis or sexually transmitteddisease)
90	H2000	Inflammation; infection of eye (except that caused by tuberculosis or sexually transmitteddisease)
90	H20011	Inflammation; infection of eye (except that caused by tuberculosis or sexually transmitteddisease)
90	H20012	Inflammation; infection of eye (except that caused by tuberculosis or sexually transmitteddisease)
90	H20013	Inflammation; infection of eye (except that caused by tuberculosis or sexually transmitteddisease)
90	H20019	Inflammation; infection of eye (except that caused by tuberculosis or sexually transmitteddisease)
90	H20021	Inflammation; infection of eye (except that caused by tuberculosis or sexually transmitteddisease)
90	H20022	Inflammation; infection of eye (except that caused by tuberculosis or sexually transmitteddisease)
90	H20023	Inflammation; infection of eye (except that caused by tuberculosis or sexually transmitteddisease)
90	H20029	Inflammation; infection of eye (except that caused by tuberculosis or sexually transmitteddisease)
90	H20031	Inflammation; infection of eye (except that caused by tuberculosis or sexually transmitteddisease)
90	H20032	Inflammation; infection of eye (except that caused by tuberculosis or sexually transmitteddisease)
90	H20033	Inflammation; infection of eye (except that caused by tuberculosis or sexually transmitteddisease)
90	H20039	Inflammation; infection of eye (except that caused by tuberculosis or sexually transmitteddisease)
90	H20041	Inflammation; infection of eye (except that caused by tuberculosis or sexually transmitteddisease)
90	H20042	Inflammation; infection of eye (except that caused by tuberculosis or sexually transmitteddisease)
90	H20043	Inflammation; infection of eye (except that caused by tuberculosis or sexually transmitteddisease)
90	H20049	Inflammation; infection of eye (except that caused by tuberculosis or sexually transmitteddisease)
90	H20051	Inflammation; infection of eye (except that caused by tuberculosis or sexually transmitteddisease)

90	H20052	Inflammation; infection of eye (except that caused by tuberculosis or sexually transmitteddisease)
90	H20053	Inflammation; infection of eye (except that caused by tuberculosis or sexually transmitteddisease)
90	H20059	Inflammation; infection of eye (except that caused by tuberculosis or sexually transmitteddisease)
90	H2010	Inflammation; infection of eye (except that caused by tuberculosis or sexually transmitteddisease)
90	H2011	Inflammation; infection of eye (except that caused by tuberculosis or sexually transmitteddisease)
90	H2012	Inflammation; infection of eye (except that caused by tuberculosis or sexually transmitteddisease)
90	H2013	Inflammation; infection of eye (except that caused by tuberculosis or sexually transmitteddisease)
90	H2020	Inflammation; infection of eye (except that caused by tuberculosis or sexually transmitteddisease)
90	H2021	Inflammation; infection of eye (except that caused by tuberculosis or sexually transmitteddisease)
90	H2022	Inflammation; infection of eye (except that caused by tuberculosis or sexually transmitteddisease)
90	H2023	Inflammation; infection of eye (except that caused by tuberculosis or sexually transmitteddisease)
90	H20811	Inflammation; infection of eye (except that caused by tuberculosis or sexually transmitteddisease)
90	H20812	Inflammation; infection of eye (except that caused by tuberculosis or sexually transmitteddisease)
90	H20813	Inflammation; infection of eye (except that caused by tuberculosis or sexually transmitteddisease)
90	H20819	Inflammation; infection of eye (except that caused by tuberculosis or sexually transmitteddisease)
90	H20821	Inflammation; infection of eye (except that caused by tuberculosis or sexually transmitteddisease)
90	H20822	Inflammation; infection of eye (except that caused by tuberculosis or sexually transmitteddisease)
90	H20823	Inflammation; infection of eye (except that caused by tuberculosis or sexually transmitteddisease)
90	H20829	Inflammation; infection of eye (except that caused by tuberculosis or sexually transmitteddisease)
90	H209	Inflammation; infection of eye (except that caused by tuberculosis or sexually transmitteddisease)
90	H21331	Inflammation; infection of eye (except that caused by tuberculosis or sexually transmitteddisease)
90	H21332	Inflammation; infection of eye (except that caused by tuberculosis or sexually transmitteddisease)
90	H21333	Inflammation; infection of eye (except that caused by tuberculosis or sexually transmitteddisease)
90	H21339	Inflammation; infection of eye (except that caused by tuberculosis or sexually transmitteddisease)
90	H30001	Inflammation; infection of eye (except that caused by tuberculosis or sexually transmitteddisease)
90	H30002	Inflammation; infection of eye (except that caused by tuberculosis or sexually transmitteddisease)
90	H30003	Inflammation; infection of eye (except that caused by tuberculosis or sexually transmitteddisease)
90	H30009	Inflammation; infection of eye (except that caused by tuberculosis or sexually transmitteddisease)
90	H30011	Inflammation; infection of eye (except that caused by tuberculosis or sexually transmitteddisease)
90	H30012	Inflammation; infection of eye (except that caused by tuberculosis or sexually transmitteddisease)
90	H30013	Inflammation; infection of eye (except that caused by tuberculosis or sexually transmitteddisease)
90	H30019	Inflammation; infection of eye (except that caused by tuberculosis or sexually transmitteddisease)
90	H30021	Inflammation; infection of eye (except that caused by tuberculosis or sexually transmitteddisease)
90	H30022	Inflammation; infection of eye (except that caused by tuberculosis or sexually transmitteddisease)
90	H30023	Inflammation; infection of eye (except that caused by tuberculosis or sexually transmitteddisease)
90	H30029	Inflammation; infection of eye (except that caused by tuberculosis or sexually transmitteddisease)
90	H30031	Inflammation; infection of eye (except that caused by tuberculosis or sexually transmitteddisease)
90	H30032	Inflammation; infection of eye (except that caused by tuberculosis or sexually transmitteddisease)
90	H30033	Inflammation; infection of eye (except that caused by tuberculosis or sexually transmitteddisease)
90	H30039	Inflammation; infection of eye (except that caused by tuberculosis or sexually transmitteddisease)
90	H30041	Inflammation; infection of eye (except that caused by tuberculosis or sexually transmitteddisease)
90	H30042	Inflammation; infection of eye (except that caused by tuberculosis or sexually transmitteddisease)
90	H30043	Inflammation; infection of eye (except that caused by tuberculosis or sexually transmitteddisease)
90	H30049	Inflammation; infection of eye (except that caused by tuberculosis or sexually transmitteddisease)
90	H30101	Inflammation; infection of eye (except that caused by tuberculosis or sexually transmitteddisease)
90	H30102	Inflammation; infection of eye (except that caused by tuberculosis or sexually transmitteddisease)
90	H30103	Inflammation; infection of eye (except that caused by tuberculosis or sexually transmitteddisease)
90	H30109	Inflammation; infection of eye (except that caused by tuberculosis or sexually transmitteddisease)
90	H30111	Inflammation; infection of eye (except that caused by tuberculosis or sexually transmitteddisease)
90	H30112	Inflammation; infection of eye (except that caused by tuberculosis or sexually transmitteddisease)
90	H30113	Inflammation; infection of eye (except that caused by tuberculosis or sexually transmitteddisease)

90	H30119	Inflammation; infection of eye (except that caused by tuberculosis or sexually transmitteddisease)
90	H30121	Inflammation; infection of eye (except that caused by tuberculosis or sexually transmitteddisease)
90	H30122	Inflammation; infection of eye (except that caused by tuberculosis or sexually transmitteddisease)
90	H30123	Inflammation; infection of eye (except that caused by tuberculosis or sexually transmitteddisease)
90	H30129	Inflammation; infection of eye (except that caused by tuberculosis or sexually transmitteddisease)
90	H30131	Inflammation; infection of eye (except that caused by tuberculosis or sexually transmitteddisease)
90	H30132	Inflammation; infection of eye (except that caused by tuberculosis or sexually transmitteddisease)
90	H30133	Inflammation; infection of eye (except that caused by tuberculosis or sexually transmitteddisease)
90	H30139	Inflammation; infection of eye (except that caused by tuberculosis or sexually transmitteddisease)
90	H30141	Inflammation; infection of eye (except that caused by tuberculosis or sexually transmitteddisease)
90	H30142	Inflammation; infection of eye (except that caused by tuberculosis or sexually transmitteddisease)
90	H30143	Inflammation; infection of eye (except that caused by tuberculosis or sexually transmitteddisease)
90	H30149	Inflammation; infection of eye (except that caused by tuberculosis or sexually transmitteddisease)
90	H3020	Inflammation; infection of eye (except that caused by tuberculosis or sexually transmitteddisease)
90	H3021	Inflammation; infection of eye (except that caused by tuberculosis or sexually transmitteddisease)
90	H3022	Inflammation; infection of eye (except that caused by tuberculosis or sexually transmitteddisease)
90	H3023	Inflammation; infection of eye (except that caused by tuberculosis or sexually transmitteddisease)
90	H30811	Inflammation; infection of eye (except that caused by tuberculosis or sexually transmitteddisease)
90	H30812	Inflammation; infection of eye (except that caused by tuberculosis or sexually transmitteddisease)
90	H30813	Inflammation; infection of eye (except that caused by tuberculosis or sexually transmitteddisease)
90	H30819	Inflammation; infection of eye (except that caused by tuberculosis or sexually transmitteddisease)
90	H30891	Inflammation; infection of eye (except that caused by tuberculosis or sexually transmitteddisease)
90	H30892	Inflammation; infection of eye (except that caused by tuberculosis or sexually transmitteddisease)
90	H30893	Inflammation; infection of eye (except that caused by tuberculosis or sexually transmitteddisease)
90	H30899	Inflammation; infection of eye (except that caused by tuberculosis or sexually transmitteddisease)
90	H3090	Inflammation; infection of eye (except that caused by tuberculosis or sexually transmitteddisease)
90	H3091	Inflammation; infection of eye (except that caused by tuberculosis or sexually transmitteddisease)
90	H3092	Inflammation; infection of eye (except that caused by tuberculosis or sexually transmitteddisease)
90	H3093	Inflammation; infection of eye (except that caused by tuberculosis or sexually transmitteddisease)
90	H33121	Inflammation; infection of eye (except that caused by tuberculosis or sexually transmitteddisease)
90	H33122	Inflammation; infection of eye (except that caused by tuberculosis or sexually transmitteddisease)
90	H33123	Inflammation; infection of eye (except that caused by tuberculosis or sexually transmitteddisease)
90	H33129	Inflammation; infection of eye (except that caused by tuberculosis or sexually transmitteddisease)
90	H44001	Inflammation; infection of eye (except that caused by tuberculosis or sexually transmitteddisease)
90	H44002	Inflammation; infection of eye (except that caused by tuberculosis or sexually transmitteddisease)
90	H44003	Inflammation; infection of eye (except that caused by tuberculosis or sexually transmitteddisease)
90	H44009	Inflammation; infection of eye (except that caused by tuberculosis or sexually transmitteddisease)
90	H44011	Inflammation; infection of eye (except that caused by tuberculosis or sexually transmitteddisease)
90	H44012	Inflammation; infection of eye (except that caused by tuberculosis or sexually transmitteddisease)
90	H44013	Inflammation; infection of eye (except that caused by tuberculosis or sexually transmitteddisease)
90	H44019	Inflammation; infection of eye (except that caused by tuberculosis or sexually transmitteddisease)
90	H44021	Inflammation; infection of eye (except that caused by tuberculosis or sexually transmitteddisease)
90	H44022	Inflammation; infection of eye (except that caused by tuberculosis or sexually transmitteddisease)
90	H44023	Inflammation; infection of eye (except that caused by tuberculosis or sexually transmitteddisease)
90	H44029	Inflammation; infection of eye (except that caused by tuberculosis or sexually transmitteddisease)
90	H44111	Inflammation; infection of eye (except that caused by tuberculosis or sexually transmitteddisease)
90	H44112	Inflammation; infection of eye (except that caused by tuberculosis or sexually transmitteddisease)
90	H44113	Inflammation; infection of eye (except that caused by tuberculosis or sexually transmitteddisease)
90	H44119	Inflammation; infection of eye (except that caused by tuberculosis or sexually transmitteddisease)
90	H44121	Inflammation; infection of eye (except that caused by tuberculosis or sexually transmitteddisease)
90	H44122	Inflammation; infection of eye (except that caused by tuberculosis or sexually transmitteddisease)

90	H44123	Inflammation; infection of eye (except that caused by tuberculosis or sexually transmitteddisease)
90	H44129	Inflammation; infection of eye (except that caused by tuberculosis or sexually transmitteddisease)
90	H44131	Inflammation; infection of eye (except that caused by tuberculosis or sexually transmitteddisease)
90	H44132	Inflammation; infection of eye (except that caused by tuberculosis or sexually transmitteddisease)
90	H44133	Inflammation; infection of eye (except that caused by tuberculosis or sexually transmitteddisease)
90	H44139	Inflammation; infection of eye (except that caused by tuberculosis or sexually transmitteddisease)
90	H4419	Inflammation; infection of eye (except that caused by tuberculosis or sexually transmitteddisease)
90	H4600	Inflammation; infection of eye (except that caused by tuberculosis or sexually transmitteddisease)
90	H4601	Inflammation; infection of eye (except that caused by tuberculosis or sexually transmitteddisease)
90	H4602	Inflammation; infection of eye (except that caused by tuberculosis or sexually transmitteddisease)
90	H4603	Inflammation; infection of eye (except that caused by tuberculosis or sexually transmitteddisease)
90	H4610	Inflammation; infection of eye (except that caused by tuberculosis or sexually transmitteddisease)
90	H4611	Inflammation; infection of eye (except that caused by tuberculosis or sexually transmitteddisease)
90	H4612	Inflammation; infection of eye (except that caused by tuberculosis or sexually transmitteddisease)
90	H4613	Inflammation; infection of eye (except that caused by tuberculosis or sexually transmitteddisease)
90	H462	Inflammation; infection of eye (except that caused by tuberculosis or sexually transmitteddisease)
90	H463	Inflammation; infection of eye (except that caused by tuberculosis or sexually transmitteddisease)
90	H468	Inflammation; infection of eye (except that caused by tuberculosis or sexually transmitteddisease)
90	H469	Inflammation; infection of eye (except that caused by tuberculosis or sexually transmitteddisease)
91	H0011	Other eye disorders
91	H0012	Other eye disorders
91	H0013	Other eye disorders
91	H0014	Other eye disorders
91	H0015	Other eye disorders
91	H0016	Other eye disorders
91	H0019	Other eye disorders
91	H02001	Other eye disorders
91	H02002	Other eye disorders
91	H02003	Other eye disorders
91	H02004	Other eye disorders
91	H02005	Other eye disorders
91	H02006	Other eye disorders
91	H02009	Other eye disorders
91	H02011	Other eye disorders
91	H02012	Other eye disorders
91	H02013	Other eye disorders
91	H02014	Other eye disorders
91	H02015	Other eye disorders
91	H02016	Other eye disorders
91	H02019	Other eye disorders
91	H02021	Other eye disorders
91	H02022	Other eye disorders
91	H02023	Other eye disorders
91	H02024	Other eye disorders
91	H02025	Other eye disorders
91	H02026	Other eye disorders
91	H02029	Other eye disorders
91	H02031	Other eye disorders
91	H02032	Other eye disorders
91	H02033	Other eye disorders
91	H02034	Other eye disorders

91	H02035	Other eye disorders
91	H02036	Other eye disorders
91	H02039	Other eye disorders
91	H02041	Other eye disorders
91	H02042	Other eye disorders
91	H02043	Other eye disorders
91	H02044	Other eye disorders
91	H02045	Other eye disorders
91	H02046	Other eye disorders
91	H02049	Other eye disorders
91	H02051	Other eye disorders
91	H02052	Other eye disorders
91	H02053	Other eye disorders
91	H02054	Other eye disorders
91	H02055	Other eye disorders
91	H02056	Other eye disorders
91	H02059	Other eye disorders
91	H02101	Other eye disorders
91	H02102	Other eye disorders
91	H02103	Other eye disorders
91	H02104	Other eye disorders
91	H02105	Other eye disorders
91	H02106	Other eye disorders
91	H02109	Other eye disorders
91	H02111	Other eye disorders
91	H02112	Other eye disorders
91	H02113	Other eye disorders
91	H02114	Other eye disorders
91	H02115	Other eye disorders
91	H02116	Other eye disorders
91	H02119	Other eye disorders
91	H02121	Other eye disorders
91	H02122	Other eye disorders
91	H02123	Other eye disorders
91	H02124	Other eye disorders
91	H02125	Other eye disorders
91	H02126	Other eye disorders
91	H02129	Other eye disorders
91	H02131	Other eye disorders
91	H02132	Other eye disorders
91	H02133	Other eye disorders
91	H02134	Other eye disorders
91	H02135	Other eye disorders
91	H02136	Other eye disorders
91	H02139	Other eye disorders
91	H02141	Other eye disorders
91	H02142	Other eye disorders
91	H02143	Other eye disorders
91	H02144	Other eye disorders
91	H02145	Other eye disorders
91	H02146	Other eye disorders

91	H02149	Other eye disorders
91	H02201	Other eye disorders
91	H02202	Other eye disorders
91	H02203	Other eye disorders
91	H02204	Other eye disorders
91	H02205	Other eye disorders
91	H02206	Other eye disorders
91	H02209	Other eye disorders
91	H02211	Other eye disorders
91	H02212	Other eye disorders
91	H02213	Other eye disorders
91	H02214	Other eye disorders
91	H02215	Other eye disorders
91	H02216	Other eye disorders
91	H02219	Other eye disorders
91	H02221	Other eye disorders
91	H02222	Other eye disorders
91	H02223	Other eye disorders
91	H02224	Other eye disorders
91	H02225	Other eye disorders
91	H02226	Other eye disorders
91	H02229	Other eye disorders
91	H02231	Other eye disorders
91	H02232	Other eye disorders
91	H02233	Other eye disorders
91	H02234	Other eye disorders
91	H02235	Other eye disorders
91	H02236	Other eye disorders
91	H02239	Other eye disorders
91	H0230	Other eye disorders
91	H0231	Other eye disorders
91	H0232	Other eye disorders
91	H0233	Other eye disorders
91	H0234	Other eye disorders
91	H0235	Other eye disorders
91	H0236	Other eye disorders
91	H02401	Other eye disorders
91	H02402	Other eye disorders
91	H02403	Other eye disorders
91	H02409	Other eye disorders
91	H02411	Other eye disorders
91	H02412	Other eye disorders
91	H02413	Other eye disorders
91	H02419	Other eye disorders
91	H02421	Other eye disorders
91	H02422	Other eye disorders
91	H02423	Other eye disorders
91	H02429	Other eye disorders
91	H02431	Other eye disorders
91	H02432	Other eye disorders
91	H02433	Other eye disorders

91	H02439	Other eye disorders
91	H02511	Other eye disorders
91	H02512	Other eye disorders
91	H02513	Other eye disorders
91	H02514	Other eye disorders
91	H02515	Other eye disorders
91	H02516	Other eye disorders
91	H02519	Other eye disorders
91	H02521	Other eye disorders
91	H02522	Other eye disorders
91	H02523	Other eye disorders
91	H02524	Other eye disorders
91	H02525	Other eye disorders
91	H02526	Other eye disorders
91	H02529	Other eye disorders
91	H02531	Other eye disorders
91	H02532	Other eye disorders
91	H02533	Other eye disorders
91	H02534	Other eye disorders
91	H02535	Other eye disorders
91	H02536	Other eye disorders
91	H02539	Other eye disorders
91	H0259	Other eye disorders
91	H0260	Other eye disorders
91	H0261	Other eye disorders
91	H0262	Other eye disorders
91	H0263	Other eye disorders
91	H0264	Other eye disorders
91	H0265	Other eye disorders
91	H0266	Other eye disorders
91	H0270	Other eye disorders
91	H02711	Other eye disorders
91	H02712	Other eye disorders
91	H02713	Other eye disorders
91	H02714	Other eye disorders
91	H02715	Other eye disorders
91	H02716	Other eye disorders
91	H02719	Other eye disorders
91	H02721	Other eye disorders
91	H02722	Other eye disorders
91	H02723	Other eye disorders
91	H02724	Other eye disorders
91	H02725	Other eye disorders
91	H02726	Other eye disorders
91	H02729	Other eye disorders
91	H02731	Other eye disorders
91	H02732	Other eye disorders
91	H02733	Other eye disorders
91	H02734	Other eye disorders
91	H02735	Other eye disorders
91	H02736	Other eye disorders

91	H02739	Other eye disorders
91	H0279	Other eye disorders
91	H02811	Other eye disorders
91	H02812	Other eye disorders
91	H02813	Other eye disorders
91	H02814	Other eye disorders
91	H02815	Other eye disorders
91	H02816	Other eye disorders
91	H02819	Other eye disorders
91	H02821	Other eye disorders
91	H02822	Other eye disorders
91	H02823	Other eye disorders
91	H02824	Other eye disorders
91	H02825	Other eye disorders
91	H02826	Other eye disorders
91	H02829	Other eye disorders
91	H02831	Other eye disorders
91	H02832	Other eye disorders
91	H02833	Other eye disorders
91	H02834	Other eye disorders
91	H02835	Other eye disorders
91	H02836	Other eye disorders
91	H02839	Other eye disorders
91	H02841	Other eye disorders
91	H02842	Other eye disorders
91	H02843	Other eye disorders
91	H02844	Other eye disorders
91	H02845	Other eye disorders
91	H02846	Other eye disorders
91	H02849	Other eye disorders
91	H02851	Other eye disorders
91	H02852	Other eye disorders
91	H02853	Other eye disorders
91	H02854	Other eye disorders
91	H02855	Other eye disorders
91	H02856	Other eye disorders
91	H02859	Other eye disorders
91	H02861	Other eye disorders
91	H02862	Other eye disorders
91	H02863	Other eye disorders
91	H02864	Other eye disorders
91	H02865	Other eye disorders
91	H02866	Other eye disorders
91	H02869	Other eye disorders
91	H02871	Other eye disorders
91	H02872	Other eye disorders
91	H02873	Other eye disorders
91	H02874	Other eye disorders
91	H02875	Other eye disorders
91	H02876	Other eye disorders
91	H02879	Other eye disorders

91	H0289	Other eye disorders
91	H029	Other eye disorders
91	H04111	Other eye disorders
91	H04112	Other eye disorders
91	H04113	Other eye disorders
91	H04119	Other eye disorders
91	H04121	Other eye disorders
91	H04122	Other eye disorders
91	H04123	Other eye disorders
91	H04129	Other eye disorders
91	H04131	Other eye disorders
91	H04132	Other eye disorders
91	H04133	Other eye disorders
91	H04139	Other eye disorders
91	H04141	Other eye disorders
91	H04142	Other eye disorders
91	H04143	Other eye disorders
91	H04149	Other eye disorders
91	H04151	Other eye disorders
91	H04152	Other eye disorders
91	H04153	Other eye disorders
91	H04159	Other eye disorders
91	H04161	Other eye disorders
91	H04162	Other eye disorders
91	H04163	Other eye disorders
91	H04169	Other eye disorders
91	H0419	Other eye disorders
91	H04201	Other eye disorders
91	H04202	Other eye disorders
91	H04203	Other eye disorders
91	H04209	Other eye disorders
91	H04211	Other eye disorders
91	H04212	Other eye disorders
91	H04213	Other eye disorders
91	H04219	Other eye disorders
91	H04221	Other eye disorders
91	H04222	Other eye disorders
91	H04223	Other eye disorders
91	H04229	Other eye disorders
91	H04511	Other eye disorders
91	H04512	Other eye disorders
91	H04513	Other eye disorders
91	H04519	Other eye disorders
91	H04521	Other eye disorders
91	H04522	Other eye disorders
91	H04523	Other eye disorders
91	H04529	Other eye disorders
91	H04531	Other eye disorders
91	H04532	Other eye disorders
91	H04533	Other eye disorders
91	H04539	Other eye disorders

91	H04541	Other eye disorders
91	H04542	Other eye disorders
91	H04543	Other eye disorders
91	H04549	Other eye disorders
91	H04551	Other eye disorders
91	H04552	Other eye disorders
91	H04553	Other eye disorders
91	H04559	Other eye disorders
91	H04561	Other eye disorders
91	H04562	Other eye disorders
91	H04563	Other eye disorders
91	H04569	Other eye disorders
91	H04571	Other eye disorders
91	H04572	Other eye disorders
91	H04573	Other eye disorders
91	H04579	Other eye disorders
91	H04611	Other eye disorders
91	H04612	Other eye disorders
91	H04613	Other eye disorders
91	H04619	Other eye disorders
91	H0469	Other eye disorders
91	H04811	Other eye disorders
91	H04812	Other eye disorders
91	H04813	Other eye disorders
91	H04819	Other eye disorders
91	H0489	Other eye disorders
91	H049	Other eye disorders
91	H0520	Other eye disorders
91	H05211	Other eye disorders
91	H05212	Other eye disorders
91	H05213	Other eye disorders
91	H05219	Other eye disorders
91	H05221	Other eye disorders
91	H05222	Other eye disorders
91	H05223	Other eye disorders
91	H05229	Other eye disorders
91	H05231	Other eye disorders
91	H05232	Other eye disorders
91	H05233	Other eye disorders
91	H05239	Other eye disorders
91	H05241	Other eye disorders
91	H05242	Other eye disorders
91	H05243	Other eye disorders
91	H05249	Other eye disorders
91	H05251	Other eye disorders
91	H05252	Other eye disorders
91	H05253	Other eye disorders
91	H05259	Other eye disorders
91	H05261	Other eye disorders
91	H05262	Other eye disorders
91	H05263	Other eye disorders

91	H05269	Other eye disorders
91	H0530	Other eye disorders
91	H05311	Other eye disorders
91	H05312	Other eye disorders
91	H05313	Other eye disorders
91	H05319	Other eye disorders
91	H05321	Other eye disorders
91	H05322	Other eye disorders
91	H05323	Other eye disorders
91	H05329	Other eye disorders
91	H05331	Other eye disorders
91	H05332	Other eye disorders
91	H05333	Other eye disorders
91	H05339	Other eye disorders
91	H05341	Other eye disorders
91	H05342	Other eye disorders
91	H05343	Other eye disorders
91	H05349	Other eye disorders
91	H05351	Other eye disorders
91	H05352	Other eye disorders
91	H05353	Other eye disorders
91	H05359	Other eye disorders
91	H05401	Other eye disorders
91	H05402	Other eye disorders
91	H05403	Other eye disorders
91	H05409	Other eye disorders
91	H05411	Other eye disorders
91	H05412	Other eye disorders
91	H05413	Other eye disorders
91	H05419	Other eye disorders
91	H05421	Other eye disorders
91	H05422	Other eye disorders
91	H05423	Other eye disorders
91	H05429	Other eye disorders
91	H0550	Other eye disorders
91	H0551	Other eye disorders
91	H0552	Other eye disorders
91	H0553	Other eye disorders
91	H05811	Other eye disorders
91	H05812	Other eye disorders
91	H05813	Other eye disorders
91	H05819	Other eye disorders
91	H05821	Other eye disorders
91	H05822	Other eye disorders
91	H05823	Other eye disorders
91	H05829	Other eye disorders
91	H0589	Other eye disorders
91	H059	Other eye disorders
91	H10811	Other eye disorders
91	H10812	Other eye disorders
91	H10813	Other eye disorders

91	H10819	Other eye disorders
91	H11001	Other eye disorders
91	H11002	Other eye disorders
91	H11003	Other eye disorders
91	H11009	Other eye disorders
91	H11011	Other eye disorders
91	H11012	Other eye disorders
91	H11013	Other eye disorders
91	H11019	Other eye disorders
91	H11021	Other eye disorders
91	H11022	Other eye disorders
91	H11023	Other eye disorders
91	H11029	Other eye disorders
91	H11031	Other eye disorders
91	H11032	Other eye disorders
91	H11033	Other eye disorders
91	H11039	Other eye disorders
91	H11041	Other eye disorders
91	H11042	Other eye disorders
91	H11043	Other eye disorders
91	H11049	Other eye disorders
91	H11051	Other eye disorders
91	H11052	Other eye disorders
91	H11053	Other eye disorders
91	H11059	Other eye disorders
91	H11061	Other eye disorders
91	H11062	Other eye disorders
91	H11063	Other eye disorders
91	H11069	Other eye disorders
91	H1110	Other eye disorders
91	H11111	Other eye disorders
91	H11112	Other eye disorders
91	H11113	Other eye disorders
91	H11119	Other eye disorders
91	H11121	Other eye disorders
91	H11122	Other eye disorders
91	H11123	Other eye disorders
91	H11129	Other eye disorders
91	H11131	Other eye disorders
91	H11132	Other eye disorders
91	H11133	Other eye disorders
91	H11139	Other eye disorders
91	H11141	Other eye disorders
91	H11142	Other eye disorders
91	H11143	Other eye disorders
91	H11149	Other eye disorders
91	H11151	Other eye disorders
91	H11152	Other eye disorders
91	H11153	Other eye disorders
91	H11159	Other eye disorders
91	H11211	Other eye disorders

91	H11212	Other eye disorders
91	H11213	Other eye disorders
91	H11219	Other eye disorders
91	H11221	Other eye disorders
91	H11222	Other eye disorders
91	H11223	Other eye disorders
91	H11229	Other eye disorders
91	H11231	Other eye disorders
91	H11232	Other eye disorders
91	H11233	Other eye disorders
91	H11239	Other eye disorders
91	H11241	Other eye disorders
91	H11242	Other eye disorders
91	H11243	Other eye disorders
91	H11249	Other eye disorders
91	H1130	Other eye disorders
91	H1131	Other eye disorders
91	H1132	Other eye disorders
91	H1133	Other eye disorders
91	H11411	Other eye disorders
91	H11412	Other eye disorders
91	H11413	Other eye disorders
91	H11419	Other eye disorders
91	H11421	Other eye disorders
91	H11422	Other eye disorders
91	H11423	Other eye disorders
91	H11429	Other eye disorders
91	H11431	Other eye disorders
91	H11432	Other eye disorders
91	H11433	Other eye disorders
91	H11439	Other eye disorders
91	H11441	Other eye disorders
91	H11442	Other eye disorders
91	H11443	Other eye disorders
91	H11449	Other eye disorders
91	H11811	Other eye disorders
91	H11812	Other eye disorders
91	H11813	Other eye disorders
91	H11819	Other eye disorders
91	H11821	Other eye disorders
91	H11822	Other eye disorders
91	H11823	Other eye disorders
91	H11829	Other eye disorders
91	H1189	Other eye disorders
91	H119	Other eye disorders
91	H15811	Other eye disorders
91	H15812	Other eye disorders
91	H15813	Other eye disorders
91	H15819	Other eye disorders
91	H15821	Other eye disorders
91	H15822	Other eye disorders

91	H15823	Other eye disorders
91	H15829	Other eye disorders
91	H15831	Other eye disorders
91	H15832	Other eye disorders
91	H15833	Other eye disorders
91	H15839	Other eye disorders
91	H15841	Other eye disorders
91	H15842	Other eye disorders
91	H15843	Other eye disorders
91	H15849	Other eye disorders
91	H15851	Other eye disorders
91	H15852	Other eye disorders
91	H15853	Other eye disorders
91	H15859	Other eye disorders
91	H1589	Other eye disorders
91	H159	Other eye disorders
91	H16001	Other eye disorders
91	H16002	Other eye disorders
91	H16003	Other eye disorders
91	H16009	Other eye disorders
91	H16011	Other eye disorders
91	H16012	Other eye disorders
91	H16013	Other eye disorders
91	H16019	Other eye disorders
91	H16021	Other eye disorders
91	H16022	Other eye disorders
91	H16023	Other eye disorders
91	H16029	Other eye disorders
91	H16031	Other eye disorders
91	H16032	Other eye disorders
91	H16033	Other eye disorders
91	H16039	Other eye disorders
91	H16041	Other eye disorders
91	H16042	Other eye disorders
91	H16043	Other eye disorders
91	H16049	Other eye disorders
91	H16051	Other eye disorders
91	H16052	Other eye disorders
91	H16053	Other eye disorders
91	H16059	Other eye disorders
91	H16061	Other eye disorders
91	H16062	Other eye disorders
91	H16063	Other eye disorders
91	H16069	Other eye disorders
91	H16071	Other eye disorders
91	H16072	Other eye disorders
91	H16073	Other eye disorders
91	H16079	Other eye disorders
91	H16401	Other eye disorders
91	H16402	Other eye disorders
91	H16403	Other eye disorders

91	H16409	Other eye disorders
91	H16411	Other eye disorders
91	H16412	Other eye disorders
91	H16413	Other eye disorders
91	H16419	Other eye disorders
91	H16421	Other eye disorders
91	H16422	Other eye disorders
91	H16423	Other eye disorders
91	H16429	Other eye disorders
91	H16431	Other eye disorders
91	H16432	Other eye disorders
91	H16433	Other eye disorders
91	H16439	Other eye disorders
91	H16441	Other eye disorders
91	H16442	Other eye disorders
91	H16443	Other eye disorders
91	H16449	Other eye disorders
91	H1700	Other eye disorders
91	H1701	Other eye disorders
91	H1702	Other eye disorders
91	H1703	Other eye disorders
91	H1710	Other eye disorders
91	H1711	Other eye disorders
91	H1712	Other eye disorders
91	H1713	Other eye disorders
91	H17811	Other eye disorders
91	H17812	Other eye disorders
91	H17813	Other eye disorders
91	H17819	Other eye disorders
91	H17821	Other eye disorders
91	H17822	Other eye disorders
91	H17823	Other eye disorders
91	H17829	Other eye disorders
91	H1789	Other eye disorders
91	H179	Other eye disorders
91	H18001	Other eye disorders
91	H18002	Other eye disorders
91	H18003	Other eye disorders
91	H18009	Other eye disorders
91	H18011	Other eye disorders
91	H18012	Other eye disorders
91	H18013	Other eye disorders
91	H18019	Other eye disorders
91	H18021	Other eye disorders
91	H18022	Other eye disorders
91	H18023	Other eye disorders
91	H18029	Other eye disorders
91	H18031	Other eye disorders
91	H18032	Other eye disorders
91	H18033	Other eye disorders
91	H18039	Other eye disorders

91	H18041	Other eye disorders
91	H18042	Other eye disorders
91	H18043	Other eye disorders
91	H18049	Other eye disorders
91	H18051	Other eye disorders
91	H18052	Other eye disorders
91	H18053	Other eye disorders
91	H18059	Other eye disorders
91	H18061	Other eye disorders
91	H18062	Other eye disorders
91	H18063	Other eye disorders
91	H18069	Other eye disorders
91	H1810	Other eye disorders
91	H1811	Other eye disorders
91	H1812	Other eye disorders
91	H1813	Other eye disorders
91	H1820	Other eye disorders
91	H18211	Other eye disorders
91	H18212	Other eye disorders
91	H18213	Other eye disorders
91	H18219	Other eye disorders
91	H18221	Other eye disorders
91	H18222	Other eye disorders
91	H18223	Other eye disorders
91	H18229	Other eye disorders
91	H18231	Other eye disorders
91	H18232	Other eye disorders
91	H18233	Other eye disorders
91	H18239	Other eye disorders
91	H1830	Other eye disorders
91	H18311	Other eye disorders
91	H18312	Other eye disorders
91	H18313	Other eye disorders
91	H18319	Other eye disorders
91	H18321	Other eye disorders
91	H18322	Other eye disorders
91	H18323	Other eye disorders
91	H18329	Other eye disorders
91	H18331	Other eye disorders
91	H18332	Other eye disorders
91	H18333	Other eye disorders
91	H18339	Other eye disorders
91	H1840	Other eye disorders
91	H18411	Other eye disorders
91	H18412	Other eye disorders
91	H18413	Other eye disorders
91	H18419	Other eye disorders
91	H18421	Other eye disorders
91	H18422	Other eye disorders
91	H18423	Other eye disorders
91	H18429	Other eye disorders

91	H1843	Other eye disorders
91	H18441	Other eye disorders
91	H18442	Other eye disorders
91	H18443	Other eye disorders
91	H18449	Other eye disorders
91	H18451	Other eye disorders
91	H18452	Other eye disorders
91	H18453	Other eye disorders
91	H18459	Other eye disorders
91	H18461	Other eye disorders
91	H18462	Other eye disorders
91	H18463	Other eye disorders
91	H18469	Other eye disorders
91	H1849	Other eye disorders
91	H1850	Other eye disorders
91	H1851	Other eye disorders
91	H1852	Other eye disorders
91	H1853	Other eye disorders
91	H1854	Other eye disorders
91	H1855	Other eye disorders
91	H1859	Other eye disorders
91	H18601	Other eye disorders
91	H18602	Other eye disorders
91	H18603	Other eye disorders
91	H18609	Other eye disorders
91	H18611	Other eye disorders
91	H18612	Other eye disorders
91	H18613	Other eye disorders
91	H18619	Other eye disorders
91	H18621	Other eye disorders
91	H18622	Other eye disorders
91	H18623	Other eye disorders
91	H18629	Other eye disorders
91	H1870	Other eye disorders
91	H18711	Other eye disorders
91	H18712	Other eye disorders
91	H18713	Other eye disorders
91	H18719	Other eye disorders
91	H18721	Other eye disorders
91	H18722	Other eye disorders
91	H18723	Other eye disorders
91	H18729	Other eye disorders
91	H18731	Other eye disorders
91	H18732	Other eye disorders
91	H18733	Other eye disorders
91	H18739	Other eye disorders
91	H18791	Other eye disorders
91	H18792	Other eye disorders
91	H18793	Other eye disorders
91	H18799	Other eye disorders
91	H18811	Other eye disorders

91	H18812	Other eye disorders
91	H18813	Other eye disorders
91	H18819	Other eye disorders
91	H18821	Other eye disorders
91	H18822	Other eye disorders
91	H18823	Other eye disorders
91	H18829	Other eye disorders
91	H18831	Other eye disorders
91	H18832	Other eye disorders
91	H18833	Other eye disorders
91	H18839	Other eye disorders
91	H18891	Other eye disorders
91	H18892	Other eye disorders
91	H18893	Other eye disorders
91	H18899	Other eye disorders
91	H189	Other eye disorders
91	H2100	Other eye disorders
91	H2101	Other eye disorders
91	H2102	Other eye disorders
91	H2103	Other eye disorders
91	H211X1	Other eye disorders
91	H211X2	Other eye disorders
91	H211X3	Other eye disorders
91	H211X9	Other eye disorders
91	H21211	Other eye disorders
91	H21212	Other eye disorders
91	H21213	Other eye disorders
91	H21219	Other eye disorders
91	H21221	Other eye disorders
91	H21222	Other eye disorders
91	H21223	Other eye disorders
91	H21229	Other eye disorders
91	H21231	Other eye disorders
91	H21232	Other eye disorders
91	H21233	Other eye disorders
91	H21239	Other eye disorders
91	H21241	Other eye disorders
91	H21242	Other eye disorders
91	H21243	Other eye disorders
91	H21249	Other eye disorders
91	H21251	Other eye disorders
91	H21252	Other eye disorders
91	H21253	Other eye disorders
91	H21259	Other eye disorders
91	H21261	Other eye disorders
91	H21262	Other eye disorders
91	H21263	Other eye disorders
91	H21269	Other eye disorders
91	H21271	Other eye disorders
91	H21272	Other eye disorders
91	H21273	Other eye disorders

91	H21279	Other eye disorders
91	H2129	Other eye disorders
91	H21301	Other eye disorders
91	H21302	Other eye disorders
91	H21303	Other eye disorders
91	H21309	Other eye disorders
91	H21311	Other eye disorders
91	H21312	Other eye disorders
91	H21313	Other eye disorders
91	H21319	Other eye disorders
91	H21321	Other eye disorders
91	H21322	Other eye disorders
91	H21323	Other eye disorders
91	H21329	Other eye disorders
91	H21341	Other eye disorders
91	H21342	Other eye disorders
91	H21343	Other eye disorders
91	H21349	Other eye disorders
91	H21351	Other eye disorders
91	H21352	Other eye disorders
91	H21353	Other eye disorders
91	H21359	Other eye disorders
91	H2140	Other eye disorders
91	H2141	Other eye disorders
91	H2142	Other eye disorders
91	H2143	Other eye disorders
91	H21501	Other eye disorders
91	H21502	Other eye disorders
91	H21503	Other eye disorders
91	H21509	Other eye disorders
91	H21511	Other eye disorders
91	H21512	Other eye disorders
91	H21513	Other eye disorders
91	H21519	Other eye disorders
91	H21521	Other eye disorders
91	H21522	Other eye disorders
91	H21523	Other eye disorders
91	H21529	Other eye disorders
91	H21531	Other eye disorders
91	H21532	Other eye disorders
91	H21533	Other eye disorders
91	H21539	Other eye disorders
91	H21541	Other eye disorders
91	H21542	Other eye disorders
91	H21543	Other eye disorders
91	H21549	Other eye disorders
91	H21551	Other eye disorders
91	H21552	Other eye disorders
91	H21553	Other eye disorders
91	H21559	Other eye disorders
91	H21561	Other eye disorders

91	H21562	Other eye disorders
91	H21563	Other eye disorders
91	H21569	Other eye disorders
91	H2181	Other eye disorders
91	H2182	Other eye disorders
91	H2189	Other eye disorders
91	H219	Other eye disorders
91	H22	Other eye disorders
91	H2700	Other eye disorders
91	H2701	Other eye disorders
91	H2702	Other eye disorders
91	H2703	Other eye disorders
91	H2710	Other eye disorders
91	H27111	Other eye disorders
91	H27112	Other eye disorders
91	H27113	Other eye disorders
91	H27119	Other eye disorders
91	H27121	Other eye disorders
91	H27122	Other eye disorders
91	H27123	Other eye disorders
91	H27129	Other eye disorders
91	H27131	Other eye disorders
91	H27132	Other eye disorders
91	H27133	Other eye disorders
91	H27139	Other eye disorders
91	H278	Other eye disorders
91	H279	Other eye disorders
91	H31001	Other eye disorders
91	H31002	Other eye disorders
91	H31003	Other eye disorders
91	H31009	Other eye disorders
91	H31011	Other eye disorders
91	H31012	Other eye disorders
91	H31013	Other eye disorders
91	H31019	Other eye disorders
91	H31021	Other eye disorders
91	H31022	Other eye disorders
91	H31023	Other eye disorders
91	H31029	Other eye disorders
91	H31091	Other eye disorders
91	H31092	Other eye disorders
91	H31093	Other eye disorders
91	H31099	Other eye disorders
91	H31101	Other eye disorders
91	H31102	Other eye disorders
91	H31103	Other eye disorders
91	H31109	Other eye disorders
91	H31111	Other eye disorders
91	H31112	Other eye disorders
91	H31113	Other eye disorders
91	H31119	Other eye disorders

91	H31121	Other eye disorders
91	H31122	Other eye disorders
91	H31123	Other eye disorders
91	H31129	Other eye disorders
91	H3120	Other eye disorders
91	H3121	Other eye disorders
91	H3122	Other eye disorders
91	H3123	Other eye disorders
91	H3129	Other eye disorders
91	H31301	Other eye disorders
91	H31302	Other eye disorders
91	H31303	Other eye disorders
91	H31309	Other eye disorders
91	H31311	Other eye disorders
91	H31312	Other eye disorders
91	H31313	Other eye disorders
91	H31319	Other eye disorders
91	H31321	Other eye disorders
91	H31322	Other eye disorders
91	H31323	Other eye disorders
91	H31329	Other eye disorders
91	H31401	Other eye disorders
91	H31402	Other eye disorders
91	H31403	Other eye disorders
91	H31409	Other eye disorders
91	H31411	Other eye disorders
91	H31412	Other eye disorders
91	H31413	Other eye disorders
91	H31419	Other eye disorders
91	H31421	Other eye disorders
91	H31422	Other eye disorders
91	H31423	Other eye disorders
91	H31429	Other eye disorders
91	H318	Other eye disorders
91	H319	Other eye disorders
91	H32	Other eye disorders
91	H3533	Other eye disorders
91	H4300	Other eye disorders
91	H4301	Other eye disorders
91	H4302	Other eye disorders
91	H4303	Other eye disorders
91	H4310	Other eye disorders
91	H4311	Other eye disorders
91	H4312	Other eye disorders
91	H4313	Other eye disorders
91	H4320	Other eye disorders
91	H4321	Other eye disorders
91	H4322	Other eye disorders
91	H4323	Other eye disorders
91	H43311	Other eye disorders
91	H43312	Other eye disorders

91	H43313	Other eye disorders
91	H43319	Other eye disorders
91	H43391	Other eye disorders
91	H43392	Other eye disorders
91	H43393	Other eye disorders
91	H43399	Other eye disorders
91	H43811	Other eye disorders
91	H43812	Other eye disorders
91	H43813	Other eye disorders
91	H43819	Other eye disorders
91	H43821	Other eye disorders
91	H43822	Other eye disorders
91	H43823	Other eye disorders
91	H43829	Other eye disorders
91	H4389	Other eye disorders
91	H439	Other eye disorders
91	H4420	Other eye disorders
91	H4421	Other eye disorders
91	H4422	Other eye disorders
91	H4423	Other eye disorders
91	H4430	Other eye disorders
91	H44311	Other eye disorders
91	H44312	Other eye disorders
91	H44313	Other eye disorders
91	H44319	Other eye disorders
91	H44321	Other eye disorders
91	H44322	Other eye disorders
91	H44323	Other eye disorders
91	H44329	Other eye disorders
91	H44391	Other eye disorders
91	H44392	Other eye disorders
91	H44393	Other eye disorders
91	H44399	Other eye disorders
91	H4440	Other eye disorders
91	H44411	Other eye disorders
91	H44412	Other eye disorders
91	H44413	Other eye disorders
91	H44419	Other eye disorders
91	H44421	Other eye disorders
91	H44422	Other eye disorders
91	H44423	Other eye disorders
91	H44429	Other eye disorders
91	H44431	Other eye disorders
91	H44432	Other eye disorders
91	H44433	Other eye disorders
91	H44439	Other eye disorders
91	H44441	Other eye disorders
91	H44442	Other eye disorders
91	H44443	Other eye disorders
91	H44449	Other eye disorders
91	H4450	Other eye disorders

91	H44511	Other eye disorders
91	H44512	Other eye disorders
91	H44513	Other eye disorders
91	H44519	Other eye disorders
91	H44521	Other eye disorders
91	H44522	Other eye disorders
91	H44523	Other eye disorders
91	H44529	Other eye disorders
91	H44531	Other eye disorders
91	H44532	Other eye disorders
91	H44533	Other eye disorders
91	H44539	Other eye disorders
91	H44601	Other eye disorders
91	H44602	Other eye disorders
91	H44603	Other eye disorders
91	H44609	Other eye disorders
91	H44611	Other eye disorders
91	H44612	Other eye disorders
91	H44613	Other eye disorders
91	H44619	Other eye disorders
91	H44621	Other eye disorders
91	H44622	Other eye disorders
91	H44623	Other eye disorders
91	H44629	Other eye disorders
91	H44631	Other eye disorders
91	H44632	Other eye disorders
91	H44633	Other eye disorders
91	H44639	Other eye disorders
91	H44641	Other eye disorders
91	H44642	Other eye disorders
91	H44643	Other eye disorders
91	H44649	Other eye disorders
91	H44651	Other eye disorders
91	H44652	Other eye disorders
91	H44653	Other eye disorders
91	H44659	Other eye disorders
91	H44691	Other eye disorders
91	H44692	Other eye disorders
91	H44693	Other eye disorders
91	H44699	Other eye disorders
91	H44701	Other eye disorders
91	H44702	Other eye disorders
91	H44703	Other eye disorders
91	H44709	Other eye disorders
91	H44711	Other eye disorders
91	H44712	Other eye disorders
91	H44713	Other eye disorders
91	H44719	Other eye disorders
91	H44721	Other eye disorders
91	H44722	Other eye disorders
91	H44723	Other eye disorders

91	H44729	Other eye disorders
91	H44731	Other eye disorders
91	H44732	Other eye disorders
91	H44733	Other eye disorders
91	H44739	Other eye disorders
91	H44741	Other eye disorders
91	H44742	Other eye disorders
91	H44743	Other eye disorders
91	H44749	Other eye disorders
91	H44751	Other eye disorders
91	H44752	Other eye disorders
91	H44753	Other eye disorders
91	H44759	Other eye disorders
91	H44791	Other eye disorders
91	H44792	Other eye disorders
91	H44793	Other eye disorders
91	H44799	Other eye disorders
91	H44811	Other eye disorders
91	H44812	Other eye disorders
91	H44813	Other eye disorders
91	H44819	Other eye disorders
91	H44821	Other eye disorders
91	H44822	Other eye disorders
91	H44823	Other eye disorders
91	H44829	Other eye disorders
91	H4489	Other eye disorders
91	H449	Other eye disorders
91	H47011	Other eye disorders
91	H47012	Other eye disorders
91	H47013	Other eye disorders
91	H47019	Other eye disorders
91	H47021	Other eye disorders
91	H47022	Other eye disorders
91	H47023	Other eye disorders
91	H47029	Other eye disorders
91	H47031	Other eye disorders
91	H47032	Other eye disorders
91	H47033	Other eye disorders
91	H47039	Other eye disorders
91	H47091	Other eye disorders
91	H47092	Other eye disorders
91	H47093	Other eye disorders
91	H47099	Other eye disorders
91	H4710	Other eye disorders
91	H4711	Other eye disorders
91	H4712	Other eye disorders
91	H4713	Other eye disorders
91	H47141	Other eye disorders
91	H47142	Other eye disorders
91	H47143	Other eye disorders
91	H47149	Other eye disorders

91	H4720	Other eye disorders
91	H47211	Other eye disorders
91	H47212	Other eye disorders
91	H47213	Other eye disorders
91	H47219	Other eye disorders
91	H4722	Other eye disorders
91	H47231	Other eye disorders
91	H47232	Other eye disorders
91	H47233	Other eye disorders
91	H47239	Other eye disorders
91	H47291	Other eye disorders
91	H47292	Other eye disorders
91	H47293	Other eye disorders
91	H47299	Other eye disorders
91	H47311	Other eye disorders
91	H47312	Other eye disorders
91	H47313	Other eye disorders
91	H47319	Other eye disorders
91	H47321	Other eye disorders
91	H47322	Other eye disorders
91	H47323	Other eye disorders
91	H47329	Other eye disorders
91	H47331	Other eye disorders
91	H47332	Other eye disorders
91	H47333	Other eye disorders
91	H47339	Other eye disorders
91	H47391	Other eye disorders
91	H47392	Other eye disorders
91	H47393	Other eye disorders
91	H47399	Other eye disorders
91	H4741	Other eye disorders
91	H4742	Other eye disorders
91	H4743	Other eye disorders
91	H4749	Other eye disorders
91	H47511	Other eye disorders
91	H47512	Other eye disorders
91	H47519	Other eye disorders
91	H47521	Other eye disorders
91	H47522	Other eye disorders
91	H47529	Other eye disorders
91	H47531	Other eye disorders
91	H47532	Other eye disorders
91	H47539	Other eye disorders
91	H47611	Other eye disorders
91	H47612	Other eye disorders
91	H47619	Other eye disorders
91	H47621	Other eye disorders
91	H47622	Other eye disorders
91	H47629	Other eye disorders
91	H47631	Other eye disorders
91	H47632	Other eye disorders

91	H47639	Other eye disorders
91	H47641	Other eye disorders
91	H47642	Other eye disorders
91	H47649	Other eye disorders
91	H479	Other eye disorders
91	H4900	Other eye disorders
91	H4901	Other eye disorders
91	H4902	Other eye disorders
91	H4903	Other eye disorders
91	H4910	Other eye disorders
91	H4911	Other eye disorders
91	H4912	Other eye disorders
91	H4913	Other eye disorders
91	H4920	Other eye disorders
91	H4921	Other eye disorders
91	H4922	Other eye disorders
91	H4923	Other eye disorders
91	H4930	Other eye disorders
91	H4931	Other eye disorders
91	H4932	Other eye disorders
91	H4933	Other eye disorders
91	H4940	Other eye disorders
91	H4941	Other eye disorders
91	H4942	Other eye disorders
91	H4943	Other eye disorders
91	H49811	Other eye disorders
91	H49812	Other eye disorders
91	H49813	Other eye disorders
91	H49819	Other eye disorders
91	H49881	Other eye disorders
91	H49882	Other eye disorders
91	H49883	Other eye disorders
91	H49889	Other eye disorders
91	H499	Other eye disorders
91	H5000	Other eye disorders
91	H50011	Other eye disorders
91	H50012	Other eye disorders
91	H50021	Other eye disorders
91	H50022	Other eye disorders
91	H50031	Other eye disorders
91	H50032	Other eye disorders
91	H50041	Other eye disorders
91	H50042	Other eye disorders
91	H5005	Other eye disorders
91	H5006	Other eye disorders
91	H5007	Other eye disorders
91	H5008	Other eye disorders
91	H5010	Other eye disorders
91	H50111	Other eye disorders
91	H50112	Other eye disorders
91	H50121	Other eye disorders

91	H50122	Other eye disorders
91	H50131	Other eye disorders
91	H50132	Other eye disorders
91	H50141	Other eye disorders
91	H50142	Other eye disorders
91	H5015	Other eye disorders
91	H5016	Other eye disorders
91	H5017	Other eye disorders
91	H5018	Other eye disorders
91	H5021	Other eye disorders
91	H5022	Other eye disorders
91	H5030	Other eye disorders
91	H50311	Other eye disorders
91	H50312	Other eye disorders
91	H5032	Other eye disorders
91	H50331	Other eye disorders
91	H50332	Other eye disorders
91	H5034	Other eye disorders
91	H5040	Other eye disorders
91	H50411	Other eye disorders
91	H50412	Other eye disorders
91	H5042	Other eye disorders
91	H5043	Other eye disorders
91	H5050	Other eye disorders
91	H5051	Other eye disorders
91	H5052	Other eye disorders
91	H5053	Other eye disorders
91	H5054	Other eye disorders
91	H5055	Other eye disorders
91	H5060	Other eye disorders
91	H50611	Other eye disorders
91	H50612	Other eye disorders
91	H5069	Other eye disorders
91	H50811	Other eye disorders
91	H50812	Other eye disorders
91	H5089	Other eye disorders
91	H509	Other eye disorders
91	H510	Other eye disorders
91	H5111	Other eye disorders
91	H5112	Other eye disorders
91	H5120	Other eye disorders
91	H5121	Other eye disorders
91	H5122	Other eye disorders
91	H5123	Other eye disorders
91	H518	Other eye disorders
91	H519	Other eye disorders
91	H5500	Other eye disorders
91	H5501	Other eye disorders
91	H5502	Other eye disorders
91	H5503	Other eye disorders
91	H5504	Other eye disorders

91	H5509	Other eye disorders
91	H5581	Other eye disorders
91	H5589	Other eye disorders
91	H5700	Other eye disorders
91	H5701	Other eye disorders
91	H5702	Other eye disorders
91	H5703	Other eye disorders
91	H5704	Other eye disorders
91	H57051	Other eye disorders
91	H57052	Other eye disorders
91	H57053	Other eye disorders
91	H57059	Other eye disorders
91	H5709	Other eye disorders
91	H5710	Other eye disorders
91	H5711	Other eye disorders
91	H5712	Other eye disorders
91	H5713	Other eye disorders
91	H578	Other eye disorders
91	H579	Other eye disorders
91	R29891	Other eye disorders
91	Z4420	Other eye disorders
91	Z4421	Other eye disorders
91	Z4422	Other eye disorders
91	Z460	Other eye disorders
91	Z947	Other eye disorders
91	Z970	Other eye disorders
91	Z9841	Other eye disorders
91	Z9842	Other eye disorders
91	Z9849	Other eye disorders
91	Z9883	Other eye disorders
92	B053	Otitis media and related conditions
92	H6500	Otitis media and related conditions
92	H6501	Otitis media and related conditions
92	H6502	Otitis media and related conditions
92	H6503	Otitis media and related conditions
92	H6504	Otitis media and related conditions
92	H6505	Otitis media and related conditions
92	H6506	Otitis media and related conditions
92	H6507	Otitis media and related conditions
92	H65111	Otitis media and related conditions
92	H65112	Otitis media and related conditions
92	H65113	Otitis media and related conditions
92	H65114	Otitis media and related conditions
92	H65115	Otitis media and related conditions
92	H65116	Otitis media and related conditions
92	H65117	Otitis media and related conditions
92	H65119	Otitis media and related conditions
92	H65191	Otitis media and related conditions
92	H65192	Otitis media and related conditions
92	H65193	Otitis media and related conditions
92	H65194	Otitis media and related conditions

92	H65195	Otitis media and related conditions
92	H65196	Otitis media and related conditions
92	H65197	Otitis media and related conditions
92	H65199	Otitis media and related conditions
92	H6520	Otitis media and related conditions
92	H6521	Otitis media and related conditions
92	H6522	Otitis media and related conditions
92	H6523	Otitis media and related conditions
92	H6530	Otitis media and related conditions
92	H6531	Otitis media and related conditions
92	H6532	Otitis media and related conditions
92	H6533	Otitis media and related conditions
92	H65411	Otitis media and related conditions
92	H65412	Otitis media and related conditions
92	H65413	Otitis media and related conditions
92	H65419	Otitis media and related conditions
92	H65491	Otitis media and related conditions
92	H65492	Otitis media and related conditions
92	H65493	Otitis media and related conditions
92	H65499	Otitis media and related conditions
92	H6590	Otitis media and related conditions
92	H6591	Otitis media and related conditions
92	H6592	Otitis media and related conditions
92	H6593	Otitis media and related conditions
92	H66001	Otitis media and related conditions
92	H66002	Otitis media and related conditions
92	H66003	Otitis media and related conditions
92	H66004	Otitis media and related conditions
92	H66005	Otitis media and related conditions
92	H66006	Otitis media and related conditions
92	H66007	Otitis media and related conditions
92	H66009	Otitis media and related conditions
92	H66011	Otitis media and related conditions
92	H66012	Otitis media and related conditions
92	H66013	Otitis media and related conditions
92	H66014	Otitis media and related conditions
92	H66015	Otitis media and related conditions
92	H66016	Otitis media and related conditions
92	H66017	Otitis media and related conditions
92	H66019	Otitis media and related conditions
92	H6610	Otitis media and related conditions
92	H6611	Otitis media and related conditions
92	H6612	Otitis media and related conditions
92	H6613	Otitis media and related conditions
92	H6620	Otitis media and related conditions
92	H6621	Otitis media and related conditions
92	H6622	Otitis media and related conditions
92	H6623	Otitis media and related conditions
92	H663X1	Otitis media and related conditions
92	H663X2	Otitis media and related conditions
92	H663X3	Otitis media and related conditions

92	H663X9	Otitis media and related conditions
92	H6640	Otitis media and related conditions
92	H6641	Otitis media and related conditions
92	H6642	Otitis media and related conditions
92	H6643	Otitis media and related conditions
92	H6690	Otitis media and related conditions
92	H6691	Otitis media and related conditions
92	H6692	Otitis media and related conditions
92	H6693	Otitis media and related conditions
92	H671	Otitis media and related conditions
92	H672	Otitis media and related conditions
92	H673	Otitis media and related conditions
92	H679	Otitis media and related conditions
92	H68001	Otitis media and related conditions
92	H68002	Otitis media and related conditions
92	H68003	Otitis media and related conditions
92	H68009	Otitis media and related conditions
92	H68011	Otitis media and related conditions
92	H68012	Otitis media and related conditions
92	H68013	Otitis media and related conditions
92	H68019	Otitis media and related conditions
92	H68021	Otitis media and related conditions
92	H68022	Otitis media and related conditions
92	H68023	Otitis media and related conditions
92	H68029	Otitis media and related conditions
92	H68101	Otitis media and related conditions
92	H68102	Otitis media and related conditions
92	H68103	Otitis media and related conditions
92	H68109	Otitis media and related conditions
92	H68111	Otitis media and related conditions
92	H68112	Otitis media and related conditions
92	H68113	Otitis media and related conditions
92	H68119	Otitis media and related conditions
92	H68121	Otitis media and related conditions
92	H68122	Otitis media and related conditions
92	H68123	Otitis media and related conditions
92	H68129	Otitis media and related conditions
92	H68131	Otitis media and related conditions
92	H68132	Otitis media and related conditions
92	H68133	Otitis media and related conditions
92	H68139	Otitis media and related conditions
92	H6900	Otitis media and related conditions
92	H6901	Otitis media and related conditions
92	H6902	Otitis media and related conditions
92	H6903	Otitis media and related conditions
92	H6980	Otitis media and related conditions
92	H6981	Otitis media and related conditions
92	H6982	Otitis media and related conditions
92	H6983	Otitis media and related conditions
92	H6990	Otitis media and related conditions
92	H6991	Otitis media and related conditions

92	H6992	Otitis media and related conditions
92	H6993	Otitis media and related conditions
92	H70001	Otitis media and related conditions
92	H70002	Otitis media and related conditions
92	H70003	Otitis media and related conditions
92	H70009	Otitis media and related conditions
92	H70011	Otitis media and related conditions
92	H70012	Otitis media and related conditions
92	H70013	Otitis media and related conditions
92	H70019	Otitis media and related conditions
92	H70091	Otitis media and related conditions
92	H70092	Otitis media and related conditions
92	H70093	Otitis media and related conditions
92	H70099	Otitis media and related conditions
92	H7010	Otitis media and related conditions
92	H7011	Otitis media and related conditions
92	H7012	Otitis media and related conditions
92	H7013	Otitis media and related conditions
92	H70201	Otitis media and related conditions
92	H70202	Otitis media and related conditions
92	H70203	Otitis media and related conditions
92	H70209	Otitis media and related conditions
92	H70211	Otitis media and related conditions
92	H70212	Otitis media and related conditions
92	H70213	Otitis media and related conditions
92	H70219	Otitis media and related conditions
92	H70221	Otitis media and related conditions
92	H70222	Otitis media and related conditions
92	H70223	Otitis media and related conditions
92	H70229	Otitis media and related conditions
92	H70811	Otitis media and related conditions
92	H70812	Otitis media and related conditions
92	H70813	Otitis media and related conditions
92	H70819	Otitis media and related conditions
92	H70891	Otitis media and related conditions
92	H70892	Otitis media and related conditions
92	H70893	Otitis media and related conditions
92	H70899	Otitis media and related conditions
92	H7090	Otitis media and related conditions
92	H7091	Otitis media and related conditions
92	H7092	Otitis media and related conditions
92	H7093	Otitis media and related conditions
92	H7200	Otitis media and related conditions
92	H7201	Otitis media and related conditions
92	H7202	Otitis media and related conditions
92	H7203	Otitis media and related conditions
92	H7210	Otitis media and related conditions
92	H7211	Otitis media and related conditions
92	H7212	Otitis media and related conditions
92	H7213	Otitis media and related conditions
92	H722X1	Otitis media and related conditions

92	H722X2	Otitis media and related conditions
92	H722X3	Otitis media and related conditions
92	H722X9	Otitis media and related conditions
92	H72811	Otitis media and related conditions
92	H72812	Otitis media and related conditions
92	H72813	Otitis media and related conditions
92	H72819	Otitis media and related conditions
92	H72821	Otitis media and related conditions
92	H72822	Otitis media and related conditions
92	H72823	Otitis media and related conditions
92	H72829	Otitis media and related conditions
92	H7290	Otitis media and related conditions
92	H7291	Otitis media and related conditions
92	H7292	Otitis media and related conditions
92	H7293	Otitis media and related conditions
92	H73811	Otitis media and related conditions
92	H73812	Otitis media and related conditions
92	H73813	Otitis media and related conditions
92	H73819	Otitis media and related conditions
92	H73821	Otitis media and related conditions
92	H73822	Otitis media and related conditions
92	H73823	Otitis media and related conditions
92	H73829	Otitis media and related conditions
92	H73891	Otitis media and related conditions
92	H73892	Otitis media and related conditions
92	H73893	Otitis media and related conditions
92	H73899	Otitis media and related conditions
92	H7390	Otitis media and related conditions
92	H7391	Otitis media and related conditions
92	H7392	Otitis media and related conditions
92	H7393	Otitis media and related conditions
92	H7401	Otitis media and related conditions
92	H7402	Otitis media and related conditions
92	H7403	Otitis media and related conditions
92	H7409	Otitis media and related conditions
92	H7411	Otitis media and related conditions
92	H7412	Otitis media and related conditions
92	H7413	Otitis media and related conditions
92	H7419	Otitis media and related conditions
92	H7420	Otitis media and related conditions
92	H7421	Otitis media and related conditions
92	H7422	Otitis media and related conditions
92	H7423	Otitis media and related conditions
92	H74311	Otitis media and related conditions
92	H74312	Otitis media and related conditions
92	H74313	Otitis media and related conditions
92	H74319	Otitis media and related conditions
92	H74321	Otitis media and related conditions
92	H74322	Otitis media and related conditions
92	H74323	Otitis media and related conditions
92	H74329	Otitis media and related conditions

92	H74391	Otitis media and related conditions
92	H74392	Otitis media and related conditions
92	H74393	Otitis media and related conditions
92	H74399	Otitis media and related conditions
92	H7500	Otitis media and related conditions
92	H7501	Otitis media and related conditions
92	H7502	Otitis media and related conditions
92	H7503	Otitis media and related conditions
92	H7580	Otitis media and related conditions
92	H7581	Otitis media and related conditions
92	H7582	Otitis media and related conditions
92	H7583	Otitis media and related conditions
92	H8000	Otitis media and related conditions
92	H8001	Otitis media and related conditions
92	H8002	Otitis media and related conditions
92	H8003	Otitis media and related conditions
92	H8010	Otitis media and related conditions
92	H8011	Otitis media and related conditions
92	H8012	Otitis media and related conditions
92	H8013	Otitis media and related conditions
92	H8020	Otitis media and related conditions
92	H8021	Otitis media and related conditions
92	H8022	Otitis media and related conditions
92	H8023	Otitis media and related conditions
92	H8080	Otitis media and related conditions
92	H8081	Otitis media and related conditions
92	H8082	Otitis media and related conditions
92	H8083	Otitis media and related conditions
92	H8090	Otitis media and related conditions
92	H8091	Otitis media and related conditions
92	H8092	Otitis media and related conditions
92	H8093	Otitis media and related conditions
92	H9500	Otitis media and related conditions
92	H9501	Otitis media and related conditions
92	H9502	Otitis media and related conditions
92	H9503	Otitis media and related conditions
92	H95111	Otitis media and related conditions
92	H95112	Otitis media and related conditions
92	H95113	Otitis media and related conditions
92	H95119	Otitis media and related conditions
92	H95121	Otitis media and related conditions
92	H95122	Otitis media and related conditions
92	H95123	Otitis media and related conditions
92	H95129	Otitis media and related conditions
92	H95131	Otitis media and related conditions
92	H95132	Otitis media and related conditions
92	H95133	Otitis media and related conditions
92	H95139	Otitis media and related conditions
92	H95191	Otitis media and related conditions
92	H95192	Otitis media and related conditions
92	H95193	Otitis media and related conditions

92	H95199	Otitis media and related conditions
92	J1083	Otitis media and related conditions
93	H8101	Conditions associated with dizziness or vertigo
93	H8102	Conditions associated with dizziness or vertigo
93	H8103	Conditions associated with dizziness or vertigo
93	H8109	Conditions associated with dizziness or vertigo
93	H8110	Conditions associated with dizziness or vertigo
93	H8111	Conditions associated with dizziness or vertigo
93	H8112	Conditions associated with dizziness or vertigo
93	H8113	Conditions associated with dizziness or vertigo
93	H8120	Conditions associated with dizziness or vertigo
93	H8121	Conditions associated with dizziness or vertigo
93	H8122	Conditions associated with dizziness or vertigo
93	H8123	Conditions associated with dizziness or vertigo
93	H81311	Conditions associated with dizziness or vertigo
93	H81312	Conditions associated with dizziness or vertigo
93	H81313	Conditions associated with dizziness or vertigo
93	H81319	Conditions associated with dizziness or vertigo
93	H81391	Conditions associated with dizziness or vertigo
93	H81392	Conditions associated with dizziness or vertigo
93	H81393	Conditions associated with dizziness or vertigo
93	H81399	Conditions associated with dizziness or vertigo
93	H8141	Conditions associated with dizziness or vertigo
93	H8142	Conditions associated with dizziness or vertigo
93	H8143	Conditions associated with dizziness or vertigo
93	H8149	Conditions associated with dizziness or vertigo
93	H818X1	Conditions associated with dizziness or vertigo
93	H818X2	Conditions associated with dizziness or vertigo
93	H818X3	Conditions associated with dizziness or vertigo
93	H818X9	Conditions associated with dizziness or vertigo
93	H8190	Conditions associated with dizziness or vertigo
93	H8191	Conditions associated with dizziness or vertigo
93	H8192	Conditions associated with dizziness or vertigo
93	H8193	Conditions associated with dizziness or vertigo
93	H821	Conditions associated with dizziness or vertigo
93	H822	Conditions associated with dizziness or vertigo
93	H823	Conditions associated with dizziness or vertigo
93	H829	Conditions associated with dizziness or vertigo
93	H8301	Conditions associated with dizziness or vertigo
93	H8302	Conditions associated with dizziness or vertigo
93	H8303	Conditions associated with dizziness or vertigo
93	H8309	Conditions associated with dizziness or vertigo
93	H8311	Conditions associated with dizziness or vertigo
93	H8312	Conditions associated with dizziness or vertigo
93	H8313	Conditions associated with dizziness or vertigo
93	H8319	Conditions associated with dizziness or vertigo
93	H832X1	Conditions associated with dizziness or vertigo
93	H832X2	Conditions associated with dizziness or vertigo
93	H832X3	Conditions associated with dizziness or vertigo
93	H832X9	Conditions associated with dizziness or vertigo
93	H838X1	Conditions associated with dizziness or vertigo

93	H838X2	Conditions associated with dizziness or vertigo
93	H838X3	Conditions associated with dizziness or vertigo
93	H838X9	Conditions associated with dizziness or vertigo
93	H8390	Conditions associated with dizziness or vertigo
93	H8391	Conditions associated with dizziness or vertigo
93	H8392	Conditions associated with dizziness or vertigo
93	H8393	Conditions associated with dizziness or vertigo
93	R42	Conditions associated with dizziness or vertigo
94	H6000	Other ear and sense organ disorders
94	H6001	Other ear and sense organ disorders
94	H6002	Other ear and sense organ disorders
94	H6003	Other ear and sense organ disorders
94	H6010	Other ear and sense organ disorders
94	H6011	Other ear and sense organ disorders
94	H6012	Other ear and sense organ disorders
94	H6013	Other ear and sense organ disorders
94	H6020	Other ear and sense organ disorders
94	H6021	Other ear and sense organ disorders
94	H6022	Other ear and sense organ disorders
94	H6023	Other ear and sense organ disorders
94	H60311	Other ear and sense organ disorders
94	H60312	Other ear and sense organ disorders
94	H60313	Other ear and sense organ disorders
94	H60319	Other ear and sense organ disorders
94	H60321	Other ear and sense organ disorders
94	H60322	Other ear and sense organ disorders
94	H60323	Other ear and sense organ disorders
94	H60329	Other ear and sense organ disorders
94	H60331	Other ear and sense organ disorders
94	H60332	Other ear and sense organ disorders
94	H60333	Other ear and sense organ disorders
94	H60339	Other ear and sense organ disorders
94	H60391	Other ear and sense organ disorders
94	H60392	Other ear and sense organ disorders
94	H60393	Other ear and sense organ disorders
94	H60399	Other ear and sense organ disorders
94	H6040	Other ear and sense organ disorders
94	H6041	Other ear and sense organ disorders
94	H6042	Other ear and sense organ disorders
94	H6043	Other ear and sense organ disorders
94	H60501	Other ear and sense organ disorders
94	H60502	Other ear and sense organ disorders
94	H60503	Other ear and sense organ disorders
94	H60509	Other ear and sense organ disorders
94	H60511	Other ear and sense organ disorders
94	H60512	Other ear and sense organ disorders
94	H60513	Other ear and sense organ disorders
94	H60519	Other ear and sense organ disorders
94	H60521	Other ear and sense organ disorders
94	H60522	Other ear and sense organ disorders
94	H60523	Other ear and sense organ disorders

94	H60529	Other ear and sense organ disorders
94	H60531	Other ear and sense organ disorders
94	H60532	Other ear and sense organ disorders
94	H60533	Other ear and sense organ disorders
94	H60539	Other ear and sense organ disorders
94	H60541	Other ear and sense organ disorders
94	H60542	Other ear and sense organ disorders
94	H60543	Other ear and sense organ disorders
94	H60549	Other ear and sense organ disorders
94	H60551	Other ear and sense organ disorders
94	H60552	Other ear and sense organ disorders
94	H60553	Other ear and sense organ disorders
94	H60559	Other ear and sense organ disorders
94	H60591	Other ear and sense organ disorders
94	H60592	Other ear and sense organ disorders
94	H60593	Other ear and sense organ disorders
94	H60599	Other ear and sense organ disorders
94	H6060	Other ear and sense organ disorders
94	H6061	Other ear and sense organ disorders
94	H6062	Other ear and sense organ disorders
94	H6063	Other ear and sense organ disorders
94	H608X1	Other ear and sense organ disorders
94	H608X2	Other ear and sense organ disorders
94	H608X3	Other ear and sense organ disorders
94	H608X9	Other ear and sense organ disorders
94	H6090	Other ear and sense organ disorders
94	H6091	Other ear and sense organ disorders
94	H6092	Other ear and sense organ disorders
94	H6093	Other ear and sense organ disorders
94	H61001	Other ear and sense organ disorders
94	H61002	Other ear and sense organ disorders
94	H61003	Other ear and sense organ disorders
94	H61009	Other ear and sense organ disorders
94	H61011	Other ear and sense organ disorders
94	H61012	Other ear and sense organ disorders
94	H61013	Other ear and sense organ disorders
94	H61019	Other ear and sense organ disorders
94	H61021	Other ear and sense organ disorders
94	H61022	Other ear and sense organ disorders
94	H61023	Other ear and sense organ disorders
94	H61029	Other ear and sense organ disorders
94	H61031	Other ear and sense organ disorders
94	H61032	Other ear and sense organ disorders
94	H61033	Other ear and sense organ disorders
94	H61039	Other ear and sense organ disorders
94	H61101	Other ear and sense organ disorders
94	H61102	Other ear and sense organ disorders
94	H61103	Other ear and sense organ disorders
94	H61109	Other ear and sense organ disorders
94	H61111	Other ear and sense organ disorders
94	H61112	Other ear and sense organ disorders

94	H61113	Other ear and sense organ disorders
94	H61119	Other ear and sense organ disorders
94	H61121	Other ear and sense organ disorders
94	H61122	Other ear and sense organ disorders
94	H61123	Other ear and sense organ disorders
94	H61129	Other ear and sense organ disorders
94	H61191	Other ear and sense organ disorders
94	H61192	Other ear and sense organ disorders
94	H61193	Other ear and sense organ disorders
94	H61199	Other ear and sense organ disorders
94	H6120	Other ear and sense organ disorders
94	H6121	Other ear and sense organ disorders
94	H6122	Other ear and sense organ disorders
94	H6123	Other ear and sense organ disorders
94	H61301	Other ear and sense organ disorders
94	H61302	Other ear and sense organ disorders
94	H61303	Other ear and sense organ disorders
94	H61309	Other ear and sense organ disorders
94	H61311	Other ear and sense organ disorders
94	H61312	Other ear and sense organ disorders
94	H61313	Other ear and sense organ disorders
94	H61319	Other ear and sense organ disorders
94	H61321	Other ear and sense organ disorders
94	H61322	Other ear and sense organ disorders
94	H61323	Other ear and sense organ disorders
94	H61329	Other ear and sense organ disorders
94	H61391	Other ear and sense organ disorders
94	H61392	Other ear and sense organ disorders
94	H61393	Other ear and sense organ disorders
94	H61399	Other ear and sense organ disorders
94	H61811	Other ear and sense organ disorders
94	H61812	Other ear and sense organ disorders
94	H61813	Other ear and sense organ disorders
94	H61819	Other ear and sense organ disorders
94	H61891	Other ear and sense organ disorders
94	H61892	Other ear and sense organ disorders
94	H61893	Other ear and sense organ disorders
94	H61899	Other ear and sense organ disorders
94	H6190	Other ear and sense organ disorders
94	H6191	Other ear and sense organ disorders
94	H6192	Other ear and sense organ disorders
94	H6193	Other ear and sense organ disorders
94	H6240	Other ear and sense organ disorders
94	H6241	Other ear and sense organ disorders
94	H6242	Other ear and sense organ disorders
94	H6243	Other ear and sense organ disorders
94	H628X1	Other ear and sense organ disorders
94	H628X2	Other ear and sense organ disorders
94	H628X3	Other ear and sense organ disorders
94	H628X9	Other ear and sense organ disorders
94	H7100	Other ear and sense organ disorders

94	H7101	Other ear and sense organ disorders
94	H7102	Other ear and sense organ disorders
94	H7103	Other ear and sense organ disorders
94	H7110	Other ear and sense organ disorders
94	H7111	Other ear and sense organ disorders
94	H7112	Other ear and sense organ disorders
94	H7113	Other ear and sense organ disorders
94	H7120	Other ear and sense organ disorders
94	H7121	Other ear and sense organ disorders
94	H7122	Other ear and sense organ disorders
94	H7123	Other ear and sense organ disorders
94	H7130	Other ear and sense organ disorders
94	H7131	Other ear and sense organ disorders
94	H7132	Other ear and sense organ disorders
94	H7133	Other ear and sense organ disorders
94	H7190	Other ear and sense organ disorders
94	H7191	Other ear and sense organ disorders
94	H7192	Other ear and sense organ disorders
94	H7193	Other ear and sense organ disorders
94	H73001	Other ear and sense organ disorders
94	H73002	Other ear and sense organ disorders
94	H73003	Other ear and sense organ disorders
94	H73009	Other ear and sense organ disorders
94	H73011	Other ear and sense organ disorders
94	H73012	Other ear and sense organ disorders
94	H73013	Other ear and sense organ disorders
94	H73019	Other ear and sense organ disorders
94	H73091	Other ear and sense organ disorders
94	H73092	Other ear and sense organ disorders
94	H73093	Other ear and sense organ disorders
94	H73099	Other ear and sense organ disorders
94	H7310	Other ear and sense organ disorders
94	H7311	Other ear and sense organ disorders
94	H7312	Other ear and sense organ disorders
94	H7313	Other ear and sense organ disorders
94	H7320	Other ear and sense organ disorders
94	H7321	Other ear and sense organ disorders
94	H7322	Other ear and sense organ disorders
94	H7323	Other ear and sense organ disorders
94	H7440	Other ear and sense organ disorders
94	H7441	Other ear and sense organ disorders
94	H7442	Other ear and sense organ disorders
94	H7443	Other ear and sense organ disorders
94	H748X1	Other ear and sense organ disorders
94	H748X2	Other ear and sense organ disorders
94	H748X3	Other ear and sense organ disorders
94	H748X9	Other ear and sense organ disorders
94	H7490	Other ear and sense organ disorders
94	H7491	Other ear and sense organ disorders
94	H7492	Other ear and sense organ disorders
94	H7493	Other ear and sense organ disorders

94	H833X1	Other ear and sense organ disorders
94	H833X2	Other ear and sense organ disorders
94	H833X3	Other ear and sense organ disorders
94	H833X9	Other ear and sense organ disorders
94	H900	Other ear and sense organ disorders
94	H9011	Other ear and sense organ disorders
94	H9012	Other ear and sense organ disorders
94	H902	Other ear and sense organ disorders
94	H903	Other ear and sense organ disorders
94	H9041	Other ear and sense organ disorders
94	H9042	Other ear and sense organ disorders
94	H905	Other ear and sense organ disorders
94	H906	Other ear and sense organ disorders
94	H9071	Other ear and sense organ disorders
94	H9072	Other ear and sense organ disorders
94	H908	Other ear and sense organ disorders
94	H9101	Other ear and sense organ disorders
94	H9102	Other ear and sense organ disorders
94	H9103	Other ear and sense organ disorders
94	H9109	Other ear and sense organ disorders
94	H9110	Other ear and sense organ disorders
94	H9111	Other ear and sense organ disorders
94	H9112	Other ear and sense organ disorders
94	H9113	Other ear and sense organ disorders
94	H9120	Other ear and sense organ disorders
94	H9121	Other ear and sense organ disorders
94	H9122	Other ear and sense organ disorders
94	H9123	Other ear and sense organ disorders
94	H913	Other ear and sense organ disorders
94	H918X1	Other ear and sense organ disorders
94	H918X2	Other ear and sense organ disorders
94	H918X3	Other ear and sense organ disorders
94	H918X9	Other ear and sense organ disorders
94	H9190	Other ear and sense organ disorders
94	H9191	Other ear and sense organ disorders
94	H9192	Other ear and sense organ disorders
94	H9193	Other ear and sense organ disorders
94	H9201	Other ear and sense organ disorders
94	H9202	Other ear and sense organ disorders
94	H9203	Other ear and sense organ disorders
94	H9209	Other ear and sense organ disorders
94	H9210	Other ear and sense organ disorders
94	H9211	Other ear and sense organ disorders
94	H9212	Other ear and sense organ disorders
94	H9213	Other ear and sense organ disorders
94	H9220	Other ear and sense organ disorders
94	H9221	Other ear and sense organ disorders
94	H9222	Other ear and sense organ disorders
94	H9223	Other ear and sense organ disorders
94	H93011	Other ear and sense organ disorders
94	H93012	Other ear and sense organ disorders

94	H93013	Other ear and sense organ disorders
94	H93019	Other ear and sense organ disorders
94	H93091	Other ear and sense organ disorders
94	H93092	Other ear and sense organ disorders
94	H93093	Other ear and sense organ disorders
94	H93099	Other ear and sense organ disorders
94	H9311	Other ear and sense organ disorders
94	H9312	Other ear and sense organ disorders
94	H9313	Other ear and sense organ disorders
94	H9319	Other ear and sense organ disorders
94	H93211	Other ear and sense organ disorders
94	H93212	Other ear and sense organ disorders
94	H93213	Other ear and sense organ disorders
94	H93219	Other ear and sense organ disorders
94	H93221	Other ear and sense organ disorders
94	H93222	Other ear and sense organ disorders
94	H93223	Other ear and sense organ disorders
94	H93229	Other ear and sense organ disorders
94	H93231	Other ear and sense organ disorders
94	H93232	Other ear and sense organ disorders
94	H93233	Other ear and sense organ disorders
94	H93239	Other ear and sense organ disorders
94	H93241	Other ear and sense organ disorders
94	H93242	Other ear and sense organ disorders
94	H93243	Other ear and sense organ disorders
94	H93249	Other ear and sense organ disorders
94	H9325	Other ear and sense organ disorders
94	H93291	Other ear and sense organ disorders
94	H93292	Other ear and sense organ disorders
94	H93293	Other ear and sense organ disorders
94	H93299	Other ear and sense organ disorders
94	H933X1	Other ear and sense organ disorders
94	H933X2	Other ear and sense organ disorders
94	H933X3	Other ear and sense organ disorders
94	H933X9	Other ear and sense organ disorders
94	H938X1	Other ear and sense organ disorders
94	H938X2	Other ear and sense organ disorders
94	H938X3	Other ear and sense organ disorders
94	H938X9	Other ear and sense organ disorders
94	H9390	Other ear and sense organ disorders
94	H9391	Other ear and sense organ disorders
94	H9392	Other ear and sense organ disorders
94	H9393	Other ear and sense organ disorders
94	H9400	Other ear and sense organ disorders
94	H9401	Other ear and sense organ disorders
94	H9402	Other ear and sense organ disorders
94	H9403	Other ear and sense organ disorders
94	H9480	Other ear and sense organ disorders
94	H9481	Other ear and sense organ disorders
94	H9482	Other ear and sense organ disorders
94	H9483	Other ear and sense organ disorders

94	Z461	Other ear and sense organ disorders
94	Z7382	Other ear and sense organ disorders
94	Z9620	Other ear and sense organ disorders
94	Z9621	Other ear and sense organ disorders
94	Z9622	Other ear and sense organ disorders
94	Z9629	Other ear and sense organ disorders
94	Z974	Other ear and sense organ disorders
95	B2701	Other nervous system disorders
95	B2711	Other nervous system disorders
95	B2781	Other nervous system disorders
95	B2791	Other nervous system disorders
95	G08	Other nervous system disorders
95	G130	Other nervous system disorders
95	G131	Other nervous system disorders
95	G132	Other nervous system disorders
95	G138	Other nervous system disorders
95	G210	Other nervous system disorders
95	G2111	Other nervous system disorders
95	G2119	Other nervous system disorders
95	G212	Other nervous system disorders
95	G213	Other nervous system disorders
95	G214	Other nervous system disorders
95	G218	Other nervous system disorders
95	G219	Other nervous system disorders
95	G360	Other nervous system disorders
95	G361	Other nervous system disorders
95	G368	Other nervous system disorders
95	G369	Other nervous system disorders
95	G370	Other nervous system disorders
95	G371	Other nervous system disorders
95	G372	Other nervous system disorders
95	G373	Other nervous system disorders
95	G374	Other nervous system disorders
95	G375	Other nervous system disorders
95	G378	Other nervous system disorders
95	G379	Other nervous system disorders
95	G4720	Other nervous system disorders
95	G4721	Other nervous system disorders
95	G4722	Other nervous system disorders
95	G4723	Other nervous system disorders
95	G4724	Other nervous system disorders
95	G4725	Other nervous system disorders
95	G4726	Other nervous system disorders
95	G4727	Other nervous system disorders
95	G4729	Other nervous system disorders
95	G47411	Other nervous system disorders
95	G47419	Other nervous system disorders
95	G47421	Other nervous system disorders
95	G47429	Other nervous system disorders
95	G4763	Other nervous system disorders
95	G500	Other nervous system disorders

95	G501	Other nervous system disorders
95	G508	Other nervous system disorders
95	G509	Other nervous system disorders
95	G510	Other nervous system disorders
95	G511	Other nervous system disorders
95	G512	Other nervous system disorders
95	G513	Other nervous system disorders
95	G514	Other nervous system disorders
95	G518	Other nervous system disorders
95	G519	Other nervous system disorders
95	G520	Other nervous system disorders
95	G521	Other nervous system disorders
95	G522	Other nervous system disorders
95	G523	Other nervous system disorders
95	G527	Other nervous system disorders
95	G528	Other nervous system disorders
95	G529	Other nervous system disorders
95	G53	Other nervous system disorders
95	G540	Other nervous system disorders
95	G541	Other nervous system disorders
95	G542	Other nervous system disorders
95	G543	Other nervous system disorders
95	G544	Other nervous system disorders
95	G545	Other nervous system disorders
95	G546	Other nervous system disorders
95	G547	Other nervous system disorders
95	G548	Other nervous system disorders
95	G549	Other nervous system disorders
95	G55	Other nervous system disorders
95	G5600	Other nervous system disorders
95	G5601	Other nervous system disorders
95	G5602	Other nervous system disorders
95	G5610	Other nervous system disorders
95	G5611	Other nervous system disorders
95	G5612	Other nervous system disorders
95	G5620	Other nervous system disorders
95	G5621	Other nervous system disorders
95	G5622	Other nervous system disorders
95	G5630	Other nervous system disorders
95	G5631	Other nervous system disorders
95	G5632	Other nervous system disorders
95	G5640	Other nervous system disorders
95	G5641	Other nervous system disorders
95	G5642	Other nervous system disorders
95	G5680	Other nervous system disorders
95	G5681	Other nervous system disorders
95	G5682	Other nervous system disorders
95	G5690	Other nervous system disorders
95	G5691	Other nervous system disorders
95	G5692	Other nervous system disorders
95	G5700	Other nervous system disorders

95	G5701	Other nervous system disorders
95	G5702	Other nervous system disorders
95	G5710	Other nervous system disorders
95	G5711	Other nervous system disorders
95	G5712	Other nervous system disorders
95	G5720	Other nervous system disorders
95	G5721	Other nervous system disorders
95	G5722	Other nervous system disorders
95	G5730	Other nervous system disorders
95	G5731	Other nervous system disorders
95	G5732	Other nervous system disorders
95	G5740	Other nervous system disorders
95	G5741	Other nervous system disorders
95	G5742	Other nervous system disorders
95	G5750	Other nervous system disorders
95	G5751	Other nervous system disorders
95	G5752	Other nervous system disorders
95	G5760	Other nervous system disorders
95	G5761	Other nervous system disorders
95	G5762	Other nervous system disorders
95	G5770	Other nervous system disorders
95	G5771	Other nervous system disorders
95	G5772	Other nervous system disorders
95	G5780	Other nervous system disorders
95	G5781	Other nervous system disorders
95	G5782	Other nervous system disorders
95	G5790	Other nervous system disorders
95	G5791	Other nervous system disorders
95	G5792	Other nervous system disorders
95	G580	Other nervous system disorders
95	G587	Other nervous system disorders
95	G588	Other nervous system disorders
95	G589	Other nervous system disorders
95	G59	Other nervous system disorders
95	G600	Other nervous system disorders
95	G601	Other nervous system disorders
95	G602	Other nervous system disorders
95	G603	Other nervous system disorders
95	G608	Other nervous system disorders
95	G609	Other nervous system disorders
95	G610	Other nervous system disorders
95	G611	Other nervous system disorders
95	G6181	Other nervous system disorders
95	G6189	Other nervous system disorders
95	G619	Other nervous system disorders
95	G620	Other nervous system disorders
95	G622	Other nervous system disorders
95	G6281	Other nervous system disorders
95	G6282	Other nervous system disorders
95	G6289	Other nervous system disorders
95	G629	Other nervous system disorders

95	G63	Other nervous system disorders
95	G64	Other nervous system disorders
95	G650	Other nervous system disorders
95	G651	Other nervous system disorders
95	G652	Other nervous system disorders
95	G7000	Other nervous system disorders
95	G7001	Other nervous system disorders
95	G701	Other nervous system disorders
95	G702	Other nervous system disorders
95	G7080	Other nervous system disorders
95	G7081	Other nervous system disorders
95	G7089	Other nervous system disorders
95	G709	Other nervous system disorders
95	G710	Other nervous system disorders
95	G7111	Other nervous system disorders
95	G7112	Other nervous system disorders
95	G7113	Other nervous system disorders
95	G7114	Other nervous system disorders
95	G7119	Other nervous system disorders
95	G712	Other nervous system disorders
95	G713	Other nervous system disorders
95	G718	Other nervous system disorders
95	G719	Other nervous system disorders
95	G720	Other nervous system disorders
95	G721	Other nervous system disorders
95	G722	Other nervous system disorders
95	G723	Other nervous system disorders
95	G7241	Other nervous system disorders
95	G7249	Other nervous system disorders
95	G7281	Other nervous system disorders
95	G7289	Other nervous system disorders
95	G729	Other nervous system disorders
95	G731	Other nervous system disorders
95	G733	Other nervous system disorders
95	G737	Other nervous system disorders
95	G890	Other nervous system disorders
95	G8911	Other nervous system disorders
95	G8912	Other nervous system disorders
95	G8918	Other nervous system disorders
95	G8921	Other nervous system disorders
95	G8922	Other nervous system disorders
95	G8928	Other nervous system disorders
95	G8929	Other nervous system disorders
95	G893	Other nervous system disorders
95	G894	Other nervous system disorders
95	G902	Other nervous system disorders
95	G9050	Other nervous system disorders
95	G90511	Other nervous system disorders
95	G90512	Other nervous system disorders
95	G90513	Other nervous system disorders
95	G90519	Other nervous system disorders

95	G90521	Other nervous system disorders
95	G90522	Other nervous system disorders
95	G90523	Other nervous system disorders
95	G90529	Other nervous system disorders
95	G9059	Other nervous system disorders
95	G908	Other nervous system disorders
95	G909	Other nervous system disorders
95	G910	Other nervous system disorders
95	G911	Other nervous system disorders
95	G912	Other nervous system disorders
95	G913	Other nervous system disorders
95	G914	Other nervous system disorders
95	G918	Other nervous system disorders
95	G919	Other nervous system disorders
95	G92	Other nervous system disorders
95	G930	Other nervous system disorders
95	G932	Other nervous system disorders
95	G9340	Other nervous system disorders
95	G9341	Other nervous system disorders
95	G9349	Other nervous system disorders
95	G935	Other nervous system disorders
95	G936	Other nervous system disorders
95	G937	Other nervous system disorders
95	G9381	Other nervous system disorders
95	G9382	Other nervous system disorders
95	G9389	Other nervous system disorders
95	G939	Other nervous system disorders
95	G94	Other nervous system disorders
95	G950	Other nervous system disorders
95	G9511	Other nervous system disorders
95	G9519	Other nervous system disorders
95	G9520	Other nervous system disorders
95	G9529	Other nervous system disorders
95	G9581	Other nervous system disorders
95	G9589	Other nervous system disorders
95	G959	Other nervous system disorders
95	G960	Other nervous system disorders
95	G9612	Other nervous system disorders
95	G9619	Other nervous system disorders
95	G968	Other nervous system disorders
95	G969	Other nervous system disorders
95	G980	Other nervous system disorders
95	G988	Other nervous system disorders
95	G998	Other nervous system disorders
95	J1081	Other nervous system disorders
95	R200	Other nervous system disorders
95	R201	Other nervous system disorders
95	R202	Other nervous system disorders
95	R203	Other nervous system disorders
95	R208	Other nervous system disorders
95	R209	Other nervous system disorders

95	R250	Other nervous system disorders
95	R251	Other nervous system disorders
95	R253	Other nervous system disorders
95	R258	Other nervous system disorders
95	R259	Other nervous system disorders
95	R260	Other nervous system disorders
95	R261	Other nervous system disorders
95	R262	Other nervous system disorders
95	R2681	Other nervous system disorders
95	R2689	Other nervous system disorders
95	R269	Other nervous system disorders
95	R270	Other nervous system disorders
95	R278	Other nervous system disorders
95	R279	Other nervous system disorders
95	R290	Other nervous system disorders
95	R292	Other nervous system disorders
95	R414	Other nervous system disorders
95	R41840	Other nervous system disorders
95	R41841	Other nervous system disorders
95	R41842	Other nervous system disorders
95	R41843	Other nervous system disorders
95	R41844	Other nervous system disorders
95	R4189	Other nervous system disorders
95	R430	Other nervous system disorders
95	R431	Other nervous system disorders
95	R432	Other nervous system disorders
95	R438	Other nervous system disorders
95	R439	Other nervous system disorders
95	R4701	Other nervous system disorders
95	R4702	Other nervous system disorders
95	R471	Other nervous system disorders
95	R4781	Other nervous system disorders
95	R4782	Other nervous system disorders
95	R4789	Other nervous system disorders
95	R479	Other nervous system disorders
95	R481	Other nervous system disorders
95	R482	Other nervous system disorders
95	R488	Other nervous system disorders
95	R489	Other nervous system disorders
95	R900	Other nervous system disorders
95	R9082	Other nervous system disorders
95	Z462	Other nervous system disorders
95	Z86011	Other nervous system disorders
95	Z8661	Other nervous system disorders
95	Z8669	Other nervous system disorders
95	Z982	Other nervous system disorders
96	I050	Heart valve disorders
96	I051	Heart valve disorders
96	I052	Heart valve disorders
96	I058	Heart valve disorders
96	I059	Heart valve disorders

96	I060	Heart valve disorders
96	I061	Heart valve disorders
96	I062	Heart valve disorders
96	I068	Heart valve disorders
96	I069	Heart valve disorders
96	I070	Heart valve disorders
96	I071	Heart valve disorders
96	I072	Heart valve disorders
96	I078	Heart valve disorders
96	I079	Heart valve disorders
96	I080	Heart valve disorders
96	I081	Heart valve disorders
96	I082	Heart valve disorders
96	I083	Heart valve disorders
96	I088	Heart valve disorders
96	I089	Heart valve disorders
96	I091	Heart valve disorders
96	I0989	Heart valve disorders
96	I340	Heart valve disorders
96	I341	Heart valve disorders
96	I342	Heart valve disorders
96	I348	Heart valve disorders
96	I349	Heart valve disorders
96	I350	Heart valve disorders
96	I351	Heart valve disorders
96	I352	Heart valve disorders
96	I358	Heart valve disorders
96	I359	Heart valve disorders
96	I360	Heart valve disorders
96	I361	Heart valve disorders
96	I362	Heart valve disorders
96	I368	Heart valve disorders
96	I369	Heart valve disorders
96	I370	Heart valve disorders
96	I371	Heart valve disorders
96	I372	Heart valve disorders
96	I378	Heart valve disorders
96	I379	Heart valve disorders
96	R008	Heart valve disorders
96	R009	Heart valve disorders
96	R010	Heart valve disorders
96	R011	Heart valve disorders
96	R012	Heart valve disorders
96	Z952	Heart valve disorders
96	Z953	Heart valve disorders
96	Z954	Heart valve disorders
97	A3681	Peri-; endo-; and myocarditis; cardiomyopathy (except that caused by tuberculosis or sexually transmitted disease
97	A381	Peri-; endo-; and myocarditis; cardiomyopathy (except that caused by tuberculosis or sexually transmitted disease
97	A3950	Peri-; endo-; and myocarditis; cardiomyopathy (except that caused by tuberculosis or sexually transmitted disease
97	A3951	Peri-; endo-; and myocarditis; cardiomyopathy (except that caused by tuberculosis or sexually transmitted disease
97	A3952	Peri-; endo-; and myocarditis; cardiomyopathy (except that caused by tuberculosis or sexually transmitted disease

97	A3953	Peri-; endo-; and myocarditis; cardiomyopathy (except that caused by tuberculosis or sexually transmitted disease
97	B2682	Peri-; endo-; and myocarditis; cardiomyopathy (except that caused by tuberculosis or sexually transmitted disease
97	B3320	Peri-; endo-; and myocarditis; cardiomyopathy (except that caused by tuberculosis or sexually transmitted disease
97	B3321	Peri-; endo-; and myocarditis; cardiomyopathy (except that caused by tuberculosis or sexually transmitted disease
97	B3322	Peri-; endo-; and myocarditis; cardiomyopathy (except that caused by tuberculosis or sexually transmitted disease
97	B3323	Peri-; endo-; and myocarditis; cardiomyopathy (except that caused by tuberculosis or sexually transmitted disease
97	B3324	Peri-; endo-; and myocarditis; cardiomyopathy (except that caused by tuberculosis or sexually transmitted disease
97	B376	Peri-; endo-; and myocarditis; cardiomyopathy (except that caused by tuberculosis or sexually transmitted disease
97	B5881	Peri-; endo-; and myocarditis; cardiomyopathy (except that caused by tuberculosis or sexually transmitted disease
97	I010	Peri-; endo-; and myocarditis; cardiomyopathy (except that caused by tuberculosis or sexually transmitted disease
97	I011	Peri-; endo-; and myocarditis; cardiomyopathy (except that caused by tuberculosis or sexually transmitted disease
97	I012	Peri-; endo-; and myocarditis; cardiomyopathy (except that caused by tuberculosis or sexually transmitted disease
97	I018	Peri-; endo-; and myocarditis; cardiomyopathy (except that caused by tuberculosis or sexually transmitted disease
97	I019	Peri-; endo-; and myocarditis; cardiomyopathy (except that caused by tuberculosis or sexually transmitted disease
97	I020	Peri-; endo-; and myocarditis; cardiomyopathy (except that caused by tuberculosis or sexually transmitted disease
97	I090	Peri-; endo-; and myocarditis; cardiomyopathy (except that caused by tuberculosis or sexually transmitted disease
97	I092	Peri-; endo-; and myocarditis; cardiomyopathy (except that caused by tuberculosis or sexually transmitted disease
97	I099	Peri-; endo-; and myocarditis; cardiomyopathy (except that caused by tuberculosis or sexually transmitted disease
97	I230	Peri-; endo-; and myocarditis; cardiomyopathy (except that caused by tuberculosis or sexually transmitted disease
97	I300	Peri-; endo-; and myocarditis; cardiomyopathy (except that caused by tuberculosis or sexually transmitted disease
97	I301	Peri-; endo-; and myocarditis; cardiomyopathy (except that caused by tuberculosis or sexually transmitted disease
97	I308	Peri-; endo-; and myocarditis; cardiomyopathy (except that caused by tuberculosis or sexually transmitted disease
97	I309	Peri-; endo-; and myocarditis; cardiomyopathy (except that caused by tuberculosis or sexually transmitted disease
97	I310	Peri-; endo-; and myocarditis; cardiomyopathy (except that caused by tuberculosis or sexually transmitted disease
97	I311	Peri-; endo-; and myocarditis; cardiomyopathy (except that caused by tuberculosis or sexually transmitted disease
97	I312	Peri-; endo-; and myocarditis; cardiomyopathy (except that caused by tuberculosis or sexually transmitted disease
97	I313	Peri-; endo-; and myocarditis; cardiomyopathy (except that caused by tuberculosis or sexually transmitted disease
97	I314	Peri-; endo-; and myocarditis; cardiomyopathy (except that caused by tuberculosis or sexually transmitted disease
97	I318	Peri-; endo-; and myocarditis; cardiomyopathy (except that caused by tuberculosis or sexually transmitted disease
97	I319	Peri-; endo-; and myocarditis; cardiomyopathy (except that caused by tuberculosis or sexually transmitted disease
97	I32	Peri-; endo-; and myocarditis; cardiomyopathy (except that caused by tuberculosis or sexually transmitted disease
97	I330	Peri-; endo-; and myocarditis; cardiomyopathy (except that caused by tuberculosis or sexually transmitted disease
97	I339	Peri-; endo-; and myocarditis; cardiomyopathy (except that caused by tuberculosis or sexually transmitted disease
97	I38	Peri-; endo-; and myocarditis; cardiomyopathy (except that caused by tuberculosis or sexually transmitted disease
97	I39	Peri-; endo-; and myocarditis; cardiomyopathy (except that caused by tuberculosis or sexually transmitted disease
97	I400	Peri-; endo-; and myocarditis; cardiomyopathy (except that caused by tuberculosis or sexually transmitted disease
97	I401	Peri-; endo-; and myocarditis; cardiomyopathy (except that caused by tuberculosis or sexually transmitted disease
97	I408	Peri-; endo-; and myocarditis; cardiomyopathy (except that caused by tuberculosis or sexually transmitted disease
97	I409	Peri-; endo-; and myocarditis; cardiomyopathy (except that caused by tuberculosis or sexually transmitted disease
97	I41	Peri-; endo-; and myocarditis; cardiomyopathy (except that caused by tuberculosis or sexually transmitted disease
97	I420	Peri-; endo-; and myocarditis; cardiomyopathy (except that caused by tuberculosis or sexually transmitted disease
97	I421	Peri-; endo-; and myocarditis; cardiomyopathy (except that caused by tuberculosis or sexually transmitted disease
97	I422	Peri-; endo-; and myocarditis; cardiomyopathy (except that caused by tuberculosis or sexually transmitted disease
97	I423	Peri-; endo-; and myocarditis; cardiomyopathy (except that caused by tuberculosis or sexually transmitted disease
97	I424	Peri-; endo-; and myocarditis; cardiomyopathy (except that caused by tuberculosis or sexually transmitted disease
97	I425	Peri-; endo-; and myocarditis; cardiomyopathy (except that caused by tuberculosis or sexually transmitted disease
97	I427	Peri-; endo-; and myocarditis; cardiomyopathy (except that caused by tuberculosis or sexually transmitted disease
97	I428	Peri-; endo-; and myocarditis; cardiomyopathy (except that caused by tuberculosis or sexually transmitted disease
97	I429	Peri-; endo-; and myocarditis; cardiomyopathy (except that caused by tuberculosis or sexually transmitted disease
97	I43	Peri-; endo-; and myocarditis; cardiomyopathy (except that caused by tuberculosis or sexually transmitted disease
97	I514	Peri-; endo-; and myocarditis; cardiomyopathy (except that caused by tuberculosis or sexually transmitted disease

97	J1082	Peri-; endo-; and myocarditis; cardiomyopathy (except that caused by tuberculosis or sexually transmitted disease
98	I10	Essential hypertension
99	I110	Hypertension with complications and secondary hypertension
99	I119	Hypertension with complications and secondary hypertension
99	I120	Hypertension with complications and secondary hypertension
99	I129	Hypertension with complications and secondary hypertension
99	I130	Hypertension with complications and secondary hypertension
99	I1310	Hypertension with complications and secondary hypertension
99	I1311	Hypertension with complications and secondary hypertension
99	I132	Hypertension with complications and secondary hypertension
99	I150	Hypertension with complications and secondary hypertension
99	I151	Hypertension with complications and secondary hypertension
99	I152	Hypertension with complications and secondary hypertension
99	I158	Hypertension with complications and secondary hypertension
99	I159	Hypertension with complications and secondary hypertension
99	I674	Hypertension with complications and secondary hypertension
99	N262	Hypertension with complications and secondary hypertension
100	I2101	Acute myocardial infarction
100	I2102	Acute myocardial infarction
100	I2109	Acute myocardial infarction
100	I2111	Acute myocardial infarction
100	I2119	Acute myocardial infarction
100	I2121	Acute myocardial infarction
100	I2129	Acute myocardial infarction
100	I213	Acute myocardial infarction
100	I214	Acute myocardial infarction
100	I220	Acute myocardial infarction
100	I221	Acute myocardial infarction
100	I222	Acute myocardial infarction
100	I228	Acute myocardial infarction
100	I229	Acute myocardial infarction
101	I200	Coronary atherosclerosis and other heart disease
101	I201	Coronary atherosclerosis and other heart disease
101	I208	Coronary atherosclerosis and other heart disease
101	I209	Coronary atherosclerosis and other heart disease
101	I237	Coronary atherosclerosis and other heart disease
101	I240	Coronary atherosclerosis and other heart disease
101	I241	Coronary atherosclerosis and other heart disease
101	I248	Coronary atherosclerosis and other heart disease
101	I249	Coronary atherosclerosis and other heart disease
101	I2510	Coronary atherosclerosis and other heart disease
101	I25110	Coronary atherosclerosis and other heart disease
101	I25111	Coronary atherosclerosis and other heart disease
101	I25118	Coronary atherosclerosis and other heart disease
101	I25119	Coronary atherosclerosis and other heart disease
101	I252	Coronary atherosclerosis and other heart disease
101	I255	Coronary atherosclerosis and other heart disease
101	I256	Coronary atherosclerosis and other heart disease
101	I25750	Coronary atherosclerosis and other heart disease
101	I25751	Coronary atherosclerosis and other heart disease
101	I25758	Coronary atherosclerosis and other heart disease

101	I25759	Coronary atherosclerosis and other heart disease
101	I25811	Coronary atherosclerosis and other heart disease
101	I2582	Coronary atherosclerosis and other heart disease
101	I2583	Coronary atherosclerosis and other heart disease
101	I2584	Coronary atherosclerosis and other heart disease
101	I2589	Coronary atherosclerosis and other heart disease
101	I259	Coronary atherosclerosis and other heart disease
101	Z951	Coronary atherosclerosis and other heart disease
101	Z955	Coronary atherosclerosis and other heart disease
101	Z9861	Coronary atherosclerosis and other heart disease
102	R072	Nonspecific chest pain
102	R0782	Nonspecific chest pain
102	R0789	Nonspecific chest pain
102	R079	Nonspecific chest pain
103	I2601	Pulmonary heart disease
103	I2602	Pulmonary heart disease
103	I2609	Pulmonary heart disease
103	I2690	Pulmonary heart disease
103	I2692	Pulmonary heart disease
103	I2699	Pulmonary heart disease
103	I270	Pulmonary heart disease
103	I271	Pulmonary heart disease
103	I272	Pulmonary heart disease
103	I2781	Pulmonary heart disease
103	I2782	Pulmonary heart disease
103	I2789	Pulmonary heart disease
103	I279	Pulmonary heart disease
103	I280	Pulmonary heart disease
103	I281	Pulmonary heart disease
103	I288	Pulmonary heart disease
103	I289	Pulmonary heart disease
103	Z86711	Pulmonary heart disease
104	I231	Other and ill-defined heart disease
104	I232	Other and ill-defined heart disease
104	I233	Other and ill-defined heart disease
104	I234	Other and ill-defined heart disease
104	I235	Other and ill-defined heart disease
104	I236	Other and ill-defined heart disease
104	I238	Other and ill-defined heart disease
104	I253	Other and ill-defined heart disease
104	I2541	Other and ill-defined heart disease
104	I2542	Other and ill-defined heart disease
104	I510	Other and ill-defined heart disease
104	I511	Other and ill-defined heart disease
104	I512	Other and ill-defined heart disease
104	I513	Other and ill-defined heart disease
104	I515	Other and ill-defined heart disease
104	I517	Other and ill-defined heart disease
104	I5181	Other and ill-defined heart disease
104	I5189	Other and ill-defined heart disease
104	I519	Other and ill-defined heart disease

104	I52	Other and ill-defined heart disease
105	I440	Conduction disorders
105	I441	Conduction disorders
105	I442	Conduction disorders
105	I4430	Conduction disorders
105	I4439	Conduction disorders
105	I444	Conduction disorders
105	I445	Conduction disorders
105	I4460	Conduction disorders
105	I4469	Conduction disorders
105	I447	Conduction disorders
105	I450	Conduction disorders
105	I4510	Conduction disorders
105	I4519	Conduction disorders
105	I452	Conduction disorders
105	I453	Conduction disorders
105	I454	Conduction disorders
105	I455	Conduction disorders
105	I456	Conduction disorders
105	I4581	Conduction disorders
105	I4589	Conduction disorders
105	I459	Conduction disorders
105	Z45010	Conduction disorders
105	Z45018	Conduction disorders
105	Z4502	Conduction disorders
105	Z4509	Conduction disorders
105	Z950	Conduction disorders
105	Z95810	Conduction disorders
106	I470	Cardiac dysrhythmias
106	I471	Cardiac dysrhythmias
106	I472	Cardiac dysrhythmias
106	I479	Cardiac dysrhythmias
106	I480	Cardiac dysrhythmias
106	I481	Cardiac dysrhythmias
106	I482	Cardiac dysrhythmias
106	I483	Cardiac dysrhythmias
106	I484	Cardiac dysrhythmias
106	I4891	Cardiac dysrhythmias
106	I4892	Cardiac dysrhythmias
106	I491	Cardiac dysrhythmias
106	I492	Cardiac dysrhythmias
106	I493	Cardiac dysrhythmias
106	I4940	Cardiac dysrhythmias
106	I4949	Cardiac dysrhythmias
106	I495	Cardiac dysrhythmias
106	I498	Cardiac dysrhythmias
106	I499	Cardiac dysrhythmias
106	R000	Cardiac dysrhythmias
106	R001	Cardiac dysrhythmias
106	R002	Cardiac dysrhythmias
107	I462	Cardiac arrest and ventricular fibrillation

107	I468	Cardiac arrest and ventricular fibrillation
107	I469	Cardiac arrest and ventricular fibrillation
107	I4901	Cardiac arrest and ventricular fibrillation
107	I4902	Cardiac arrest and ventricular fibrillation
108	I0981	Congestive heart failure; nonhypertensive
108	I501	Congestive heart failure; nonhypertensive
108	I5020	Congestive heart failure; nonhypertensive
108	I5021	Congestive heart failure; nonhypertensive
108	I5022	Congestive heart failure; nonhypertensive
108	I5023	Congestive heart failure; nonhypertensive
108	I5030	Congestive heart failure; nonhypertensive
108	I5031	Congestive heart failure; nonhypertensive
108	I5032	Congestive heart failure; nonhypertensive
108	I5033	Congestive heart failure; nonhypertensive
108	I5040	Congestive heart failure; nonhypertensive
108	I5041	Congestive heart failure; nonhypertensive
108	I5042	Congestive heart failure; nonhypertensive
108	I5043	Congestive heart failure; nonhypertensive
108	I509	Congestive heart failure; nonhypertensive
109	G43601	Acute cerebrovascular disease
109	G43609	Acute cerebrovascular disease
109	G43611	Acute cerebrovascular disease
109	G43619	Acute cerebrovascular disease
109	I6000	Acute cerebrovascular disease
109	I6001	Acute cerebrovascular disease
109	I6002	Acute cerebrovascular disease
109	I6010	Acute cerebrovascular disease
109	I6011	Acute cerebrovascular disease
109	I6012	Acute cerebrovascular disease
109	I6020	Acute cerebrovascular disease
109	I6021	Acute cerebrovascular disease
109	I6022	Acute cerebrovascular disease
109	I6030	Acute cerebrovascular disease
109	I6031	Acute cerebrovascular disease
109	I6032	Acute cerebrovascular disease
109	I604	Acute cerebrovascular disease
109	I6050	Acute cerebrovascular disease
109	I6051	Acute cerebrovascular disease
109	I6052	Acute cerebrovascular disease
109	I606	Acute cerebrovascular disease
109	I607	Acute cerebrovascular disease
109	I608	Acute cerebrovascular disease
109	I609	Acute cerebrovascular disease
109	I610	Acute cerebrovascular disease
109	I611	Acute cerebrovascular disease
109	I612	Acute cerebrovascular disease
109	I613	Acute cerebrovascular disease
109	I614	Acute cerebrovascular disease
109	I615	Acute cerebrovascular disease
109	I616	Acute cerebrovascular disease
109	I618	Acute cerebrovascular disease

109	I619	Acute cerebrovascular disease
109	I6200	Acute cerebrovascular disease
109	I6201	Acute cerebrovascular disease
109	I6202	Acute cerebrovascular disease
109	I6203	Acute cerebrovascular disease
109	I621	Acute cerebrovascular disease
109	I629	Acute cerebrovascular disease
109	I6300	Acute cerebrovascular disease
109	I63011	Acute cerebrovascular disease
109	I63012	Acute cerebrovascular disease
109	I63019	Acute cerebrovascular disease
109	I6302	Acute cerebrovascular disease
109	I63031	Acute cerebrovascular disease
109	I63032	Acute cerebrovascular disease
109	I63039	Acute cerebrovascular disease
109	I6309	Acute cerebrovascular disease
109	I6310	Acute cerebrovascular disease
109	I63111	Acute cerebrovascular disease
109	I63112	Acute cerebrovascular disease
109	I63119	Acute cerebrovascular disease
109	I6312	Acute cerebrovascular disease
109	I63131	Acute cerebrovascular disease
109	I63132	Acute cerebrovascular disease
109	I63139	Acute cerebrovascular disease
109	I6319	Acute cerebrovascular disease
109	I6320	Acute cerebrovascular disease
109	I63211	Acute cerebrovascular disease
109	I63212	Acute cerebrovascular disease
109	I63219	Acute cerebrovascular disease
109	I6322	Acute cerebrovascular disease
109	I63231	Acute cerebrovascular disease
109	I63232	Acute cerebrovascular disease
109	I63239	Acute cerebrovascular disease
109	I6329	Acute cerebrovascular disease
109	I6330	Acute cerebrovascular disease
109	I63311	Acute cerebrovascular disease
109	I63312	Acute cerebrovascular disease
109	I63319	Acute cerebrovascular disease
109	I63321	Acute cerebrovascular disease
109	I63322	Acute cerebrovascular disease
109	I63329	Acute cerebrovascular disease
109	I63331	Acute cerebrovascular disease
109	I63332	Acute cerebrovascular disease
109	I63339	Acute cerebrovascular disease
109	I63341	Acute cerebrovascular disease
109	I63342	Acute cerebrovascular disease
109	I63349	Acute cerebrovascular disease
109	I6339	Acute cerebrovascular disease
109	I6340	Acute cerebrovascular disease
109	I63411	Acute cerebrovascular disease
109	I63412	Acute cerebrovascular disease

109	I63419	Acute cerebrovascular disease
109	I63421	Acute cerebrovascular disease
109	I63422	Acute cerebrovascular disease
109	I63429	Acute cerebrovascular disease
109	I63431	Acute cerebrovascular disease
109	I63432	Acute cerebrovascular disease
109	I63439	Acute cerebrovascular disease
109	I63441	Acute cerebrovascular disease
109	I63442	Acute cerebrovascular disease
109	I63449	Acute cerebrovascular disease
109	I6349	Acute cerebrovascular disease
109	I6350	Acute cerebrovascular disease
109	I63511	Acute cerebrovascular disease
109	I63512	Acute cerebrovascular disease
109	I63519	Acute cerebrovascular disease
109	I63521	Acute cerebrovascular disease
109	I63522	Acute cerebrovascular disease
109	I63529	Acute cerebrovascular disease
109	I63531	Acute cerebrovascular disease
109	I63532	Acute cerebrovascular disease
109	I63539	Acute cerebrovascular disease
109	I63541	Acute cerebrovascular disease
109	I63542	Acute cerebrovascular disease
109	I63549	Acute cerebrovascular disease
109	I6359	Acute cerebrovascular disease
109	I636	Acute cerebrovascular disease
109	I638	Acute cerebrovascular disease
109	I639	Acute cerebrovascular disease
109	I6601	Acute cerebrovascular disease
109	I6602	Acute cerebrovascular disease
109	I6603	Acute cerebrovascular disease
109	I6609	Acute cerebrovascular disease
109	I6611	Acute cerebrovascular disease
109	I6612	Acute cerebrovascular disease
109	I6613	Acute cerebrovascular disease
109	I6619	Acute cerebrovascular disease
109	I6621	Acute cerebrovascular disease
109	I6622	Acute cerebrovascular disease
109	I6623	Acute cerebrovascular disease
109	I6629	Acute cerebrovascular disease
109	I663	Acute cerebrovascular disease
109	I668	Acute cerebrovascular disease
109	I669	Acute cerebrovascular disease
110	I6501	Occlusion or stenosis of precerebral arteries
110	I6502	Occlusion or stenosis of precerebral arteries
110	I6503	Occlusion or stenosis of precerebral arteries
110	I6509	Occlusion or stenosis of precerebral arteries
110	I651	Occlusion or stenosis of precerebral arteries
110	I6521	Occlusion or stenosis of precerebral arteries
110	I6522	Occlusion or stenosis of precerebral arteries
110	I6523	Occlusion or stenosis of precerebral arteries

110	I6529	Occlusion or stenosis of precerebral arteries
110	I658	Occlusion or stenosis of precerebral arteries
110	I659	Occlusion or stenosis of precerebral arteries
111	G460	Other and ill-defined cerebrovascular disease
111	G461	Other and ill-defined cerebrovascular disease
111	G462	Other and ill-defined cerebrovascular disease
111	G463	Other and ill-defined cerebrovascular disease
111	G464	Other and ill-defined cerebrovascular disease
111	G465	Other and ill-defined cerebrovascular disease
111	G466	Other and ill-defined cerebrovascular disease
111	G467	Other and ill-defined cerebrovascular disease
111	G468	Other and ill-defined cerebrovascular disease
111	I671	Other and ill-defined cerebrovascular disease
111	I672	Other and ill-defined cerebrovascular disease
111	I675	Other and ill-defined cerebrovascular disease
111	I676	Other and ill-defined cerebrovascular disease
111	I677	Other and ill-defined cerebrovascular disease
111	I6781	Other and ill-defined cerebrovascular disease
111	I6782	Other and ill-defined cerebrovascular disease
111	I6783	Other and ill-defined cerebrovascular disease
111	I67841	Other and ill-defined cerebrovascular disease
111	I67848	Other and ill-defined cerebrovascular disease
111	I6789	Other and ill-defined cerebrovascular disease
111	I679	Other and ill-defined cerebrovascular disease
111	I680	Other and ill-defined cerebrovascular disease
111	I682	Other and ill-defined cerebrovascular disease
111	I688	Other and ill-defined cerebrovascular disease
112	B0681	Transient cerebral ischemia
112	G450	Transient cerebral ischemia
112	G451	Transient cerebral ischemia
112	G452	Transient cerebral ischemia
112	G453	Transient cerebral ischemia
112	G454	Transient cerebral ischemia
112	G458	Transient cerebral ischemia
112	G459	Transient cerebral ischemia
113	I6900	Late effects of cerebrovascular disease
113	I6901	Late effects of cerebrovascular disease
113	I69020	Late effects of cerebrovascular disease
113	I69021	Late effects of cerebrovascular disease
113	I69022	Late effects of cerebrovascular disease
113	I69023	Late effects of cerebrovascular disease
113	I69028	Late effects of cerebrovascular disease
113	I69031	Late effects of cerebrovascular disease
113	I69032	Late effects of cerebrovascular disease
113	I69033	Late effects of cerebrovascular disease
113	I69034	Late effects of cerebrovascular disease
113	I69039	Late effects of cerebrovascular disease
113	I69041	Late effects of cerebrovascular disease
113	I69042	Late effects of cerebrovascular disease
113	I69043	Late effects of cerebrovascular disease
113	I69044	Late effects of cerebrovascular disease

113	I69049	Late effects of cerebrovascular disease
113	I69051	Late effects of cerebrovascular disease
113	I69052	Late effects of cerebrovascular disease
113	I69053	Late effects of cerebrovascular disease
113	I69054	Late effects of cerebrovascular disease
113	I69059	Late effects of cerebrovascular disease
113	I69061	Late effects of cerebrovascular disease
113	I69062	Late effects of cerebrovascular disease
113	I69063	Late effects of cerebrovascular disease
113	I69064	Late effects of cerebrovascular disease
113	I69065	Late effects of cerebrovascular disease
113	I69069	Late effects of cerebrovascular disease
113	I69090	Late effects of cerebrovascular disease
113	I69091	Late effects of cerebrovascular disease
113	I69092	Late effects of cerebrovascular disease
113	I69093	Late effects of cerebrovascular disease
113	I69098	Late effects of cerebrovascular disease
113	I6910	Late effects of cerebrovascular disease
113	I6911	Late effects of cerebrovascular disease
113	I69120	Late effects of cerebrovascular disease
113	I69121	Late effects of cerebrovascular disease
113	I69122	Late effects of cerebrovascular disease
113	I69123	Late effects of cerebrovascular disease
113	I69128	Late effects of cerebrovascular disease
113	I69131	Late effects of cerebrovascular disease
113	I69132	Late effects of cerebrovascular disease
113	I69133	Late effects of cerebrovascular disease
113	I69134	Late effects of cerebrovascular disease
113	I69139	Late effects of cerebrovascular disease
113	I69141	Late effects of cerebrovascular disease
113	I69142	Late effects of cerebrovascular disease
113	I69143	Late effects of cerebrovascular disease
113	I69144	Late effects of cerebrovascular disease
113	I69149	Late effects of cerebrovascular disease
113	I69151	Late effects of cerebrovascular disease
113	I69152	Late effects of cerebrovascular disease
113	I69153	Late effects of cerebrovascular disease
113	I69154	Late effects of cerebrovascular disease
113	I69159	Late effects of cerebrovascular disease
113	I69161	Late effects of cerebrovascular disease
113	I69162	Late effects of cerebrovascular disease
113	I69163	Late effects of cerebrovascular disease
113	I69164	Late effects of cerebrovascular disease
113	I69165	Late effects of cerebrovascular disease
113	I69169	Late effects of cerebrovascular disease
113	I69190	Late effects of cerebrovascular disease
113	I69191	Late effects of cerebrovascular disease
113	I69192	Late effects of cerebrovascular disease
113	I69193	Late effects of cerebrovascular disease
113	I69198	Late effects of cerebrovascular disease
113	I6920	Late effects of cerebrovascular disease

113	I6921	Late effects of cerebrovascular disease
113	I69220	Late effects of cerebrovascular disease
113	I69221	Late effects of cerebrovascular disease
113	I69222	Late effects of cerebrovascular disease
113	I69223	Late effects of cerebrovascular disease
113	I69228	Late effects of cerebrovascular disease
113	I69231	Late effects of cerebrovascular disease
113	I69232	Late effects of cerebrovascular disease
113	I69233	Late effects of cerebrovascular disease
113	I69234	Late effects of cerebrovascular disease
113	I69239	Late effects of cerebrovascular disease
113	I69241	Late effects of cerebrovascular disease
113	I69242	Late effects of cerebrovascular disease
113	I69243	Late effects of cerebrovascular disease
113	I69244	Late effects of cerebrovascular disease
113	I69249	Late effects of cerebrovascular disease
113	I69251	Late effects of cerebrovascular disease
113	I69252	Late effects of cerebrovascular disease
113	I69253	Late effects of cerebrovascular disease
113	I69254	Late effects of cerebrovascular disease
113	I69259	Late effects of cerebrovascular disease
113	I69261	Late effects of cerebrovascular disease
113	I69262	Late effects of cerebrovascular disease
113	I69263	Late effects of cerebrovascular disease
113	I69264	Late effects of cerebrovascular disease
113	I69265	Late effects of cerebrovascular disease
113	I69269	Late effects of cerebrovascular disease
113	I69290	Late effects of cerebrovascular disease
113	I69291	Late effects of cerebrovascular disease
113	I69292	Late effects of cerebrovascular disease
113	I69293	Late effects of cerebrovascular disease
113	I69298	Late effects of cerebrovascular disease
113	I6930	Late effects of cerebrovascular disease
113	I6931	Late effects of cerebrovascular disease
113	I69320	Late effects of cerebrovascular disease
113	I69321	Late effects of cerebrovascular disease
113	I69322	Late effects of cerebrovascular disease
113	I69323	Late effects of cerebrovascular disease
113	I69328	Late effects of cerebrovascular disease
113	I69331	Late effects of cerebrovascular disease
113	I69332	Late effects of cerebrovascular disease
113	I69333	Late effects of cerebrovascular disease
113	I69334	Late effects of cerebrovascular disease
113	I69339	Late effects of cerebrovascular disease
113	I69341	Late effects of cerebrovascular disease
113	I69342	Late effects of cerebrovascular disease
113	I69343	Late effects of cerebrovascular disease
113	I69344	Late effects of cerebrovascular disease
113	I69349	Late effects of cerebrovascular disease
113	I69351	Late effects of cerebrovascular disease
113	I69352	Late effects of cerebrovascular disease

113	I69353	Late effects of cerebrovascular disease
113	I69354	Late effects of cerebrovascular disease
113	I69359	Late effects of cerebrovascular disease
113	I69361	Late effects of cerebrovascular disease
113	I69362	Late effects of cerebrovascular disease
113	I69363	Late effects of cerebrovascular disease
113	I69364	Late effects of cerebrovascular disease
113	I69365	Late effects of cerebrovascular disease
113	I69369	Late effects of cerebrovascular disease
113	I69390	Late effects of cerebrovascular disease
113	I69391	Late effects of cerebrovascular disease
113	I69392	Late effects of cerebrovascular disease
113	I69393	Late effects of cerebrovascular disease
113	I69398	Late effects of cerebrovascular disease
113	I6980	Late effects of cerebrovascular disease
113	I6981	Late effects of cerebrovascular disease
113	I69820	Late effects of cerebrovascular disease
113	I69821	Late effects of cerebrovascular disease
113	I69822	Late effects of cerebrovascular disease
113	I69823	Late effects of cerebrovascular disease
113	I69828	Late effects of cerebrovascular disease
113	I69831	Late effects of cerebrovascular disease
113	I69832	Late effects of cerebrovascular disease
113	I69833	Late effects of cerebrovascular disease
113	I69834	Late effects of cerebrovascular disease
113	I69839	Late effects of cerebrovascular disease
113	I69841	Late effects of cerebrovascular disease
113	I69842	Late effects of cerebrovascular disease
113	I69843	Late effects of cerebrovascular disease
113	I69844	Late effects of cerebrovascular disease
113	I69849	Late effects of cerebrovascular disease
113	I69851	Late effects of cerebrovascular disease
113	I69852	Late effects of cerebrovascular disease
113	I69853	Late effects of cerebrovascular disease
113	I69854	Late effects of cerebrovascular disease
113	I69859	Late effects of cerebrovascular disease
113	I69861	Late effects of cerebrovascular disease
113	I69862	Late effects of cerebrovascular disease
113	I69863	Late effects of cerebrovascular disease
113	I69864	Late effects of cerebrovascular disease
113	I69865	Late effects of cerebrovascular disease
113	I69869	Late effects of cerebrovascular disease
113	I69890	Late effects of cerebrovascular disease
113	I69891	Late effects of cerebrovascular disease
113	I69892	Late effects of cerebrovascular disease
113	I69893	Late effects of cerebrovascular disease
113	I69898	Late effects of cerebrovascular disease
113	I6990	Late effects of cerebrovascular disease
113	I6991	Late effects of cerebrovascular disease
113	I69920	Late effects of cerebrovascular disease
113	I69921	Late effects of cerebrovascular disease

113	I69922	Late effects of cerebrovascular disease
113	I69923	Late effects of cerebrovascular disease
113	I69928	Late effects of cerebrovascular disease
113	I69931	Late effects of cerebrovascular disease
113	I69932	Late effects of cerebrovascular disease
113	I69933	Late effects of cerebrovascular disease
113	I69934	Late effects of cerebrovascular disease
113	I69939	Late effects of cerebrovascular disease
113	I69941	Late effects of cerebrovascular disease
113	I69942	Late effects of cerebrovascular disease
113	I69943	Late effects of cerebrovascular disease
113	I69944	Late effects of cerebrovascular disease
113	I69949	Late effects of cerebrovascular disease
113	I69951	Late effects of cerebrovascular disease
113	I69952	Late effects of cerebrovascular disease
113	I69953	Late effects of cerebrovascular disease
113	I69954	Late effects of cerebrovascular disease
113	I69959	Late effects of cerebrovascular disease
113	I69961	Late effects of cerebrovascular disease
113	I69962	Late effects of cerebrovascular disease
113	I69963	Late effects of cerebrovascular disease
113	I69964	Late effects of cerebrovascular disease
113	I69965	Late effects of cerebrovascular disease
113	I69969	Late effects of cerebrovascular disease
113	I69990	Late effects of cerebrovascular disease
113	I69991	Late effects of cerebrovascular disease
113	I69992	Late effects of cerebrovascular disease
113	I69993	Late effects of cerebrovascular disease
113	I69998	Late effects of cerebrovascular disease
114	I700	Peripheral and visceral atherosclerosis
114	I701	Peripheral and visceral atherosclerosis
114	I70201	Peripheral and visceral atherosclerosis
114	I70202	Peripheral and visceral atherosclerosis
114	I70203	Peripheral and visceral atherosclerosis
114	I70208	Peripheral and visceral atherosclerosis
114	I70209	Peripheral and visceral atherosclerosis
114	I70211	Peripheral and visceral atherosclerosis
114	I70212	Peripheral and visceral atherosclerosis
114	I70213	Peripheral and visceral atherosclerosis
114	I70218	Peripheral and visceral atherosclerosis
114	I70219	Peripheral and visceral atherosclerosis
114	I70221	Peripheral and visceral atherosclerosis
114	I70222	Peripheral and visceral atherosclerosis
114	I70223	Peripheral and visceral atherosclerosis
114	I70228	Peripheral and visceral atherosclerosis
114	I70229	Peripheral and visceral atherosclerosis
114	I70231	Peripheral and visceral atherosclerosis
114	I70232	Peripheral and visceral atherosclerosis
114	I70233	Peripheral and visceral atherosclerosis
114	I70234	Peripheral and visceral atherosclerosis
114	I70235	Peripheral and visceral atherosclerosis

114	I70238	Peripheral and visceral atherosclerosis
114	I70239	Peripheral and visceral atherosclerosis
114	I70241	Peripheral and visceral atherosclerosis
114	I70242	Peripheral and visceral atherosclerosis
114	I70243	Peripheral and visceral atherosclerosis
114	I70244	Peripheral and visceral atherosclerosis
114	I70245	Peripheral and visceral atherosclerosis
114	I70248	Peripheral and visceral atherosclerosis
114	I70249	Peripheral and visceral atherosclerosis
114	I7025	Peripheral and visceral atherosclerosis
114	I70291	Peripheral and visceral atherosclerosis
114	I70292	Peripheral and visceral atherosclerosis
114	I70293	Peripheral and visceral atherosclerosis
114	I70298	Peripheral and visceral atherosclerosis
114	I70299	Peripheral and visceral atherosclerosis
114	I708	Peripheral and visceral atherosclerosis
114	I7090	Peripheral and visceral atherosclerosis
114	I7091	Peripheral and visceral atherosclerosis
114	I7092	Peripheral and visceral atherosclerosis
114	I739	Peripheral and visceral atherosclerosis
114	K550	Peripheral and visceral atherosclerosis
114	K551	Peripheral and visceral atherosclerosis
114	K558	Peripheral and visceral atherosclerosis
114	K559	Peripheral and visceral atherosclerosis
115	I670	Aortic; peripheral; and visceral artery aneurysms
115	I7100	Aortic; peripheral; and visceral artery aneurysms
115	I7101	Aortic; peripheral; and visceral artery aneurysms
115	I7102	Aortic; peripheral; and visceral artery aneurysms
115	I7103	Aortic; peripheral; and visceral artery aneurysms
115	I711	Aortic; peripheral; and visceral artery aneurysms
115	I712	Aortic; peripheral; and visceral artery aneurysms
115	I713	Aortic; peripheral; and visceral artery aneurysms
115	I714	Aortic; peripheral; and visceral artery aneurysms
115	I715	Aortic; peripheral; and visceral artery aneurysms
115	I716	Aortic; peripheral; and visceral artery aneurysms
115	I718	Aortic; peripheral; and visceral artery aneurysms
115	I719	Aortic; peripheral; and visceral artery aneurysms
115	I720	Aortic; peripheral; and visceral artery aneurysms
115	I721	Aortic; peripheral; and visceral artery aneurysms
115	I722	Aortic; peripheral; and visceral artery aneurysms
115	I723	Aortic; peripheral; and visceral artery aneurysms
115	I724	Aortic; peripheral; and visceral artery aneurysms
115	I728	Aortic; peripheral; and visceral artery aneurysms
115	I729	Aortic; peripheral; and visceral artery aneurysms
115	I7771	Aortic; peripheral; and visceral artery aneurysms
115	I7772	Aortic; peripheral; and visceral artery aneurysms
115	I7773	Aortic; peripheral; and visceral artery aneurysms
115	I7774	Aortic; peripheral; and visceral artery aneurysms
115	I7779	Aortic; peripheral; and visceral artery aneurysms
115	I77810	Aortic; peripheral; and visceral artery aneurysms
115	I77811	Aortic; peripheral; and visceral artery aneurysms

115	I77812	Aortic; peripheral; and visceral artery aneurysms
115	I77819	Aortic; peripheral; and visceral artery aneurysms
115	I790	Aortic; peripheral; and visceral artery aneurysms
116	I7401	Aortic and peripheral arterial embolism or thrombosis
116	I7409	Aortic and peripheral arterial embolism or thrombosis
116	I7410	Aortic and peripheral arterial embolism or thrombosis
116	I7411	Aortic and peripheral arterial embolism or thrombosis
116	I7419	Aortic and peripheral arterial embolism or thrombosis
116	I742	Aortic and peripheral arterial embolism or thrombosis
116	I743	Aortic and peripheral arterial embolism or thrombosis
116	I744	Aortic and peripheral arterial embolism or thrombosis
116	I745	Aortic and peripheral arterial embolism or thrombosis
116	I748	Aortic and peripheral arterial embolism or thrombosis
116	I749	Aortic and peripheral arterial embolism or thrombosis
116	I75011	Aortic and peripheral arterial embolism or thrombosis
116	I75012	Aortic and peripheral arterial embolism or thrombosis
116	I75013	Aortic and peripheral arterial embolism or thrombosis
116	I75019	Aortic and peripheral arterial embolism or thrombosis
116	I75021	Aortic and peripheral arterial embolism or thrombosis
116	I75022	Aortic and peripheral arterial embolism or thrombosis
116	I75023	Aortic and peripheral arterial embolism or thrombosis
116	I75029	Aortic and peripheral arterial embolism or thrombosis
116	I7581	Aortic and peripheral arterial embolism or thrombosis
116	I7589	Aortic and peripheral arterial embolism or thrombosis
117	I7300	Other circulatory disease
117	I731	Other circulatory disease
117	I7381	Other circulatory disease
117	I7389	Other circulatory disease
117	I770	Other circulatory disease
117	I771	Other circulatory disease
117	I772	Other circulatory disease
117	I773	Other circulatory disease
117	I774	Other circulatory disease
117	I775	Other circulatory disease
117	I776	Other circulatory disease
117	I7789	Other circulatory disease
117	I779	Other circulatory disease
117	I780	Other circulatory disease
117	I781	Other circulatory disease
117	I788	Other circulatory disease
117	I789	Other circulatory disease
117	I791	Other circulatory disease
117	I798	Other circulatory disease
117	I950	Other circulatory disease
117	I951	Other circulatory disease
117	I9589	Other circulatory disease
117	I959	Other circulatory disease
117	I998	Other circulatory disease
117	I999	Other circulatory disease
117	M310	Other circulatory disease
117	R030	Other circulatory disease

117	R031	Other circulatory disease
117	R0989	Other circulatory disease
117	R58	Other circulatory disease
117	Z8673	Other circulatory disease
117	Z8674	Other circulatory disease
117	Z8679	Other circulatory disease
117	Z941	Other circulatory disease
117	Z943	Other circulatory disease
117	Z95811	Other circulatory disease
117	Z95812	Other circulatory disease
117	Z95818	Other circulatory disease
117	Z95820	Other circulatory disease
117	Z95828	Other circulatory disease
117	Z959	Other circulatory disease
117	Z9862	Other circulatory disease
118	I8000	Phlebitis; thrombophlebitis and thromboembolism
118	I8001	Phlebitis; thrombophlebitis and thromboembolism
118	I8002	Phlebitis; thrombophlebitis and thromboembolism
118	I8003	Phlebitis; thrombophlebitis and thromboembolism
118	I8010	Phlebitis; thrombophlebitis and thromboembolism
118	I8011	Phlebitis; thrombophlebitis and thromboembolism
118	I8012	Phlebitis; thrombophlebitis and thromboembolism
118	I8013	Phlebitis; thrombophlebitis and thromboembolism
118	I80201	Phlebitis; thrombophlebitis and thromboembolism
118	I80202	Phlebitis; thrombophlebitis and thromboembolism
118	I80203	Phlebitis; thrombophlebitis and thromboembolism
118	I80209	Phlebitis; thrombophlebitis and thromboembolism
118	I80211	Phlebitis; thrombophlebitis and thromboembolism
118	I80212	Phlebitis; thrombophlebitis and thromboembolism
118	I80213	Phlebitis; thrombophlebitis and thromboembolism
118	I80219	Phlebitis; thrombophlebitis and thromboembolism
118	I80221	Phlebitis; thrombophlebitis and thromboembolism
118	I80222	Phlebitis; thrombophlebitis and thromboembolism
118	I80223	Phlebitis; thrombophlebitis and thromboembolism
118	I80229	Phlebitis; thrombophlebitis and thromboembolism
118	I80231	Phlebitis; thrombophlebitis and thromboembolism
118	I80232	Phlebitis; thrombophlebitis and thromboembolism
118	I80233	Phlebitis; thrombophlebitis and thromboembolism
118	I80239	Phlebitis; thrombophlebitis and thromboembolism
118	I80291	Phlebitis; thrombophlebitis and thromboembolism
118	I80292	Phlebitis; thrombophlebitis and thromboembolism
118	I80293	Phlebitis; thrombophlebitis and thromboembolism
118	I80299	Phlebitis; thrombophlebitis and thromboembolism
118	I803	Phlebitis; thrombophlebitis and thromboembolism
118	I808	Phlebitis; thrombophlebitis and thromboembolism
118	I809	Phlebitis; thrombophlebitis and thromboembolism
118	I81	Phlebitis; thrombophlebitis and thromboembolism
118	I820	Phlebitis; thrombophlebitis and thromboembolism
118	I821	Phlebitis; thrombophlebitis and thromboembolism
118	I82210	Phlebitis; thrombophlebitis and thromboembolism
118	I82211	Phlebitis; thrombophlebitis and thromboembolism

118	I82220	Phlebitis; thrombophlebitis and thromboembolism
118	I82221	Phlebitis; thrombophlebitis and thromboembolism
118	I82290	Phlebitis; thrombophlebitis and thromboembolism
118	I82291	Phlebitis; thrombophlebitis and thromboembolism
118	I823	Phlebitis; thrombophlebitis and thromboembolism
118	I82401	Phlebitis; thrombophlebitis and thromboembolism
118	I82402	Phlebitis; thrombophlebitis and thromboembolism
118	I82403	Phlebitis; thrombophlebitis and thromboembolism
118	I82409	Phlebitis; thrombophlebitis and thromboembolism
118	I82411	Phlebitis; thrombophlebitis and thromboembolism
118	I82412	Phlebitis; thrombophlebitis and thromboembolism
118	I82413	Phlebitis; thrombophlebitis and thromboembolism
118	I82419	Phlebitis; thrombophlebitis and thromboembolism
118	I82421	Phlebitis; thrombophlebitis and thromboembolism
118	I82422	Phlebitis; thrombophlebitis and thromboembolism
118	I82423	Phlebitis; thrombophlebitis and thromboembolism
118	I82429	Phlebitis; thrombophlebitis and thromboembolism
118	I82431	Phlebitis; thrombophlebitis and thromboembolism
118	I82432	Phlebitis; thrombophlebitis and thromboembolism
118	I82433	Phlebitis; thrombophlebitis and thromboembolism
118	I82439	Phlebitis; thrombophlebitis and thromboembolism
118	I82441	Phlebitis; thrombophlebitis and thromboembolism
118	I82442	Phlebitis; thrombophlebitis and thromboembolism
118	I82443	Phlebitis; thrombophlebitis and thromboembolism
118	I82449	Phlebitis; thrombophlebitis and thromboembolism
118	I82491	Phlebitis; thrombophlebitis and thromboembolism
118	I82492	Phlebitis; thrombophlebitis and thromboembolism
118	I82493	Phlebitis; thrombophlebitis and thromboembolism
118	I82499	Phlebitis; thrombophlebitis and thromboembolism
118	I824Y1	Phlebitis; thrombophlebitis and thromboembolism
118	I824Y2	Phlebitis; thrombophlebitis and thromboembolism
118	I824Y3	Phlebitis; thrombophlebitis and thromboembolism
118	I824Y9	Phlebitis; thrombophlebitis and thromboembolism
118	I824Z1	Phlebitis; thrombophlebitis and thromboembolism
118	I824Z2	Phlebitis; thrombophlebitis and thromboembolism
118	I824Z3	Phlebitis; thrombophlebitis and thromboembolism
118	I824Z9	Phlebitis; thrombophlebitis and thromboembolism
118	I82501	Phlebitis; thrombophlebitis and thromboembolism
118	I82502	Phlebitis; thrombophlebitis and thromboembolism
118	I82503	Phlebitis; thrombophlebitis and thromboembolism
118	I82509	Phlebitis; thrombophlebitis and thromboembolism
118	I82511	Phlebitis; thrombophlebitis and thromboembolism
118	I82512	Phlebitis; thrombophlebitis and thromboembolism
118	I82513	Phlebitis; thrombophlebitis and thromboembolism
118	I82519	Phlebitis; thrombophlebitis and thromboembolism
118	I82521	Phlebitis; thrombophlebitis and thromboembolism
118	I82522	Phlebitis; thrombophlebitis and thromboembolism
118	I82523	Phlebitis; thrombophlebitis and thromboembolism
118	I82529	Phlebitis; thrombophlebitis and thromboembolism
118	I82531	Phlebitis; thrombophlebitis and thromboembolism
118	I82532	Phlebitis; thrombophlebitis and thromboembolism

118	I82533	Phlebitis; thrombophlebitis and thromboembolism
118	I82539	Phlebitis; thrombophlebitis and thromboembolism
118	I82541	Phlebitis; thrombophlebitis and thromboembolism
118	I82542	Phlebitis; thrombophlebitis and thromboembolism
118	I82543	Phlebitis; thrombophlebitis and thromboembolism
118	I82549	Phlebitis; thrombophlebitis and thromboembolism
118	I82591	Phlebitis; thrombophlebitis and thromboembolism
118	I82592	Phlebitis; thrombophlebitis and thromboembolism
118	I82593	Phlebitis; thrombophlebitis and thromboembolism
118	I82599	Phlebitis; thrombophlebitis and thromboembolism
118	I825Y1	Phlebitis; thrombophlebitis and thromboembolism
118	I825Y2	Phlebitis; thrombophlebitis and thromboembolism
118	I825Y3	Phlebitis; thrombophlebitis and thromboembolism
118	I825Y9	Phlebitis; thrombophlebitis and thromboembolism
118	I825Z1	Phlebitis; thrombophlebitis and thromboembolism
118	I825Z2	Phlebitis; thrombophlebitis and thromboembolism
118	I825Z3	Phlebitis; thrombophlebitis and thromboembolism
118	I825Z9	Phlebitis; thrombophlebitis and thromboembolism
118	I82601	Phlebitis; thrombophlebitis and thromboembolism
118	I82602	Phlebitis; thrombophlebitis and thromboembolism
118	I82603	Phlebitis; thrombophlebitis and thromboembolism
118	I82609	Phlebitis; thrombophlebitis and thromboembolism
118	I82611	Phlebitis; thrombophlebitis and thromboembolism
118	I82612	Phlebitis; thrombophlebitis and thromboembolism
118	I82613	Phlebitis; thrombophlebitis and thromboembolism
118	I82619	Phlebitis; thrombophlebitis and thromboembolism
118	I82621	Phlebitis; thrombophlebitis and thromboembolism
118	I82622	Phlebitis; thrombophlebitis and thromboembolism
118	I82623	Phlebitis; thrombophlebitis and thromboembolism
118	I82629	Phlebitis; thrombophlebitis and thromboembolism
118	I82701	Phlebitis; thrombophlebitis and thromboembolism
118	I82702	Phlebitis; thrombophlebitis and thromboembolism
118	I82703	Phlebitis; thrombophlebitis and thromboembolism
118	I82709	Phlebitis; thrombophlebitis and thromboembolism
118	I82711	Phlebitis; thrombophlebitis and thromboembolism
118	I82712	Phlebitis; thrombophlebitis and thromboembolism
118	I82713	Phlebitis; thrombophlebitis and thromboembolism
118	I82719	Phlebitis; thrombophlebitis and thromboembolism
118	I82721	Phlebitis; thrombophlebitis and thromboembolism
118	I82722	Phlebitis; thrombophlebitis and thromboembolism
118	I82723	Phlebitis; thrombophlebitis and thromboembolism
118	I82729	Phlebitis; thrombophlebitis and thromboembolism
118	I82811	Phlebitis; thrombophlebitis and thromboembolism
118	I82812	Phlebitis; thrombophlebitis and thromboembolism
118	I82813	Phlebitis; thrombophlebitis and thromboembolism
118	I82819	Phlebitis; thrombophlebitis and thromboembolism
118	I82890	Phlebitis; thrombophlebitis and thromboembolism
118	I82891	Phlebitis; thrombophlebitis and thromboembolism
118	I8290	Phlebitis; thrombophlebitis and thromboembolism
118	I8291	Phlebitis; thrombophlebitis and thromboembolism
118	I82A11	Phlebitis; thrombophlebitis and thromboembolism

118	I82A12	Phlebitis; thrombophlebitis and thromboembolism
118	I82A13	Phlebitis; thrombophlebitis and thromboembolism
118	I82A19	Phlebitis; thrombophlebitis and thromboembolism
118	I82A21	Phlebitis; thrombophlebitis and thromboembolism
118	I82A22	Phlebitis; thrombophlebitis and thromboembolism
118	I82A23	Phlebitis; thrombophlebitis and thromboembolism
118	I82A29	Phlebitis; thrombophlebitis and thromboembolism
118	I82B11	Phlebitis; thrombophlebitis and thromboembolism
118	I82B12	Phlebitis; thrombophlebitis and thromboembolism
118	I82B13	Phlebitis; thrombophlebitis and thromboembolism
118	I82B19	Phlebitis; thrombophlebitis and thromboembolism
118	I82B21	Phlebitis; thrombophlebitis and thromboembolism
118	I82B22	Phlebitis; thrombophlebitis and thromboembolism
118	I82B23	Phlebitis; thrombophlebitis and thromboembolism
118	I82B29	Phlebitis; thrombophlebitis and thromboembolism
118	I82C11	Phlebitis; thrombophlebitis and thromboembolism
118	I82C12	Phlebitis; thrombophlebitis and thromboembolism
118	I82C13	Phlebitis; thrombophlebitis and thromboembolism
118	I82C19	Phlebitis; thrombophlebitis and thromboembolism
118	I82C21	Phlebitis; thrombophlebitis and thromboembolism
118	I82C22	Phlebitis; thrombophlebitis and thromboembolism
118	I82C23	Phlebitis; thrombophlebitis and thromboembolism
118	I82C29	Phlebitis; thrombophlebitis and thromboembolism
118	Z86718	Phlebitis; thrombophlebitis and thromboembolism
118	Z8672	Phlebitis; thrombophlebitis and thromboembolism
119	I83001	Varicose veins of lower extremity
119	I83002	Varicose veins of lower extremity
119	I83003	Varicose veins of lower extremity
119	I83004	Varicose veins of lower extremity
119	I83005	Varicose veins of lower extremity
119	I83008	Varicose veins of lower extremity
119	I83009	Varicose veins of lower extremity
119	I83011	Varicose veins of lower extremity
119	I83012	Varicose veins of lower extremity
119	I83013	Varicose veins of lower extremity
119	I83014	Varicose veins of lower extremity
119	I83015	Varicose veins of lower extremity
119	I83018	Varicose veins of lower extremity
119	I83019	Varicose veins of lower extremity
119	I83021	Varicose veins of lower extremity
119	I83022	Varicose veins of lower extremity
119	I83023	Varicose veins of lower extremity
119	I83024	Varicose veins of lower extremity
119	I83025	Varicose veins of lower extremity
119	I83028	Varicose veins of lower extremity
119	I83029	Varicose veins of lower extremity
119	I8310	Varicose veins of lower extremity
119	I8311	Varicose veins of lower extremity
119	I8312	Varicose veins of lower extremity
119	I83201	Varicose veins of lower extremity
119	I83202	Varicose veins of lower extremity

119	I83203	Varicose veins of lower extremity
119	I83204	Varicose veins of lower extremity
119	I83205	Varicose veins of lower extremity
119	I83208	Varicose veins of lower extremity
119	I83209	Varicose veins of lower extremity
119	I83211	Varicose veins of lower extremity
119	I83212	Varicose veins of lower extremity
119	I83213	Varicose veins of lower extremity
119	I83214	Varicose veins of lower extremity
119	I83215	Varicose veins of lower extremity
119	I83218	Varicose veins of lower extremity
119	I83219	Varicose veins of lower extremity
119	I83221	Varicose veins of lower extremity
119	I83222	Varicose veins of lower extremity
119	I83223	Varicose veins of lower extremity
119	I83224	Varicose veins of lower extremity
119	I83225	Varicose veins of lower extremity
119	I83228	Varicose veins of lower extremity
119	I83229	Varicose veins of lower extremity
119	I83811	Varicose veins of lower extremity
119	I83812	Varicose veins of lower extremity
119	I83813	Varicose veins of lower extremity
119	I83819	Varicose veins of lower extremity
119	I83891	Varicose veins of lower extremity
119	I83892	Varicose veins of lower extremity
119	I83893	Varicose veins of lower extremity
119	I83899	Varicose veins of lower extremity
119	I8390	Varicose veins of lower extremity
119	I8391	Varicose veins of lower extremity
119	I8392	Varicose veins of lower extremity
119	I8393	Varicose veins of lower extremity
120	K640	Hemorrhoids
120	K641	Hemorrhoids
120	K642	Hemorrhoids
120	K643	Hemorrhoids
120	K644	Hemorrhoids
120	K645	Hemorrhoids
120	K648	Hemorrhoids
120	K649	Hemorrhoids
121	I860	Other diseases of veins and lymphatics
121	I861	Other diseases of veins and lymphatics
121	I862	Other diseases of veins and lymphatics
121	I863	Other diseases of veins and lymphatics
121	I864	Other diseases of veins and lymphatics
121	I868	Other diseases of veins and lymphatics
121	I87001	Other diseases of veins and lymphatics
121	I87002	Other diseases of veins and lymphatics
121	I87003	Other diseases of veins and lymphatics
121	I87009	Other diseases of veins and lymphatics
121	I87011	Other diseases of veins and lymphatics
121	I87012	Other diseases of veins and lymphatics

121	I87013	Other diseases of veins and lymphatics
121	I87019	Other diseases of veins and lymphatics
121	I87021	Other diseases of veins and lymphatics
121	I87022	Other diseases of veins and lymphatics
121	I87023	Other diseases of veins and lymphatics
121	I87029	Other diseases of veins and lymphatics
121	I87031	Other diseases of veins and lymphatics
121	I87032	Other diseases of veins and lymphatics
121	I87033	Other diseases of veins and lymphatics
121	I87039	Other diseases of veins and lymphatics
121	I87091	Other diseases of veins and lymphatics
121	I87092	Other diseases of veins and lymphatics
121	I87093	Other diseases of veins and lymphatics
121	I87099	Other diseases of veins and lymphatics
121	I871	Other diseases of veins and lymphatics
121	I872	Other diseases of veins and lymphatics
121	I87301	Other diseases of veins and lymphatics
121	I87302	Other diseases of veins and lymphatics
121	I87303	Other diseases of veins and lymphatics
121	I87309	Other diseases of veins and lymphatics
121	I87311	Other diseases of veins and lymphatics
121	I87312	Other diseases of veins and lymphatics
121	I87313	Other diseases of veins and lymphatics
121	I87319	Other diseases of veins and lymphatics
121	I87321	Other diseases of veins and lymphatics
121	I87322	Other diseases of veins and lymphatics
121	I87323	Other diseases of veins and lymphatics
121	I87329	Other diseases of veins and lymphatics
121	I87331	Other diseases of veins and lymphatics
121	I87332	Other diseases of veins and lymphatics
121	I87333	Other diseases of veins and lymphatics
121	I87339	Other diseases of veins and lymphatics
121	I87391	Other diseases of veins and lymphatics
121	I87392	Other diseases of veins and lymphatics
121	I87393	Other diseases of veins and lymphatics
121	I87399	Other diseases of veins and lymphatics
121	I878	Other diseases of veins and lymphatics
121	I879	Other diseases of veins and lymphatics
121	I890	Other diseases of veins and lymphatics
121	I891	Other diseases of veins and lymphatics
121	I898	Other diseases of veins and lymphatics
121	I899	Other diseases of veins and lymphatics
122	A0103	Pneumonia (except that caused by tuberculosis or sexually transmitted disease)
122	A0222	Pneumonia (except that caused by tuberculosis or sexually transmitted disease)
122	A202	Pneumonia (except that caused by tuberculosis or sexually transmitted disease)
122	A212	Pneumonia (except that caused by tuberculosis or sexually transmitted disease)
122	A221	Pneumonia (except that caused by tuberculosis or sexually transmitted disease)
122	A310	Pneumonia (except that caused by tuberculosis or sexually transmitted disease)
122	A3701	Pneumonia (except that caused by tuberculosis or sexually transmitted disease)
122	A3711	Pneumonia (except that caused by tuberculosis or sexually transmitted disease)
122	A430	Pneumonia (except that caused by tuberculosis or sexually transmitted disease)

122	A481	Pneumonia (except that caused by tuberculosis or sexually transmitted disease)
122	B012	Pneumonia (except that caused by tuberculosis or sexually transmitted disease)
122	B052	Pneumonia (except that caused by tuberculosis or sexually transmitted disease)
122	B371	Pneumonia (except that caused by tuberculosis or sexually transmitted disease)
122	B380	Pneumonia (except that caused by tuberculosis or sexually transmitted disease)
122	B381	Pneumonia (except that caused by tuberculosis or sexually transmitted disease)
122	B382	Pneumonia (except that caused by tuberculosis or sexually transmitted disease)
122	B390	Pneumonia (except that caused by tuberculosis or sexually transmitted disease)
122	B391	Pneumonia (except that caused by tuberculosis or sexually transmitted disease)
122	B392	Pneumonia (except that caused by tuberculosis or sexually transmitted disease)
122	B583	Pneumonia (except that caused by tuberculosis or sexually transmitted disease)
122	B59	Pneumonia (except that caused by tuberculosis or sexually transmitted disease)
122	B7781	Pneumonia (except that caused by tuberculosis or sexually transmitted disease)
122	J120	Pneumonia (except that caused by tuberculosis or sexually transmitted disease)
122	J121	Pneumonia (except that caused by tuberculosis or sexually transmitted disease)
122	J122	Pneumonia (except that caused by tuberculosis or sexually transmitted disease)
122	J123	Pneumonia (except that caused by tuberculosis or sexually transmitted disease)
122	J1281	Pneumonia (except that caused by tuberculosis or sexually transmitted disease)
122	J1289	Pneumonia (except that caused by tuberculosis or sexually transmitted disease)
122	J129	Pneumonia (except that caused by tuberculosis or sexually transmitted disease)
122	J13	Pneumonia (except that caused by tuberculosis or sexually transmitted disease)
122	J14	Pneumonia (except that caused by tuberculosis or sexually transmitted disease)
122	J150	Pneumonia (except that caused by tuberculosis or sexually transmitted disease)
122	J151	Pneumonia (except that caused by tuberculosis or sexually transmitted disease)
122	J1520	Pneumonia (except that caused by tuberculosis or sexually transmitted disease)
122	J15211	Pneumonia (except that caused by tuberculosis or sexually transmitted disease)
122	J15212	Pneumonia (except that caused by tuberculosis or sexually transmitted disease)
122	J1529	Pneumonia (except that caused by tuberculosis or sexually transmitted disease)
122	J153	Pneumonia (except that caused by tuberculosis or sexually transmitted disease)
122	J154	Pneumonia (except that caused by tuberculosis or sexually transmitted disease)
122	J155	Pneumonia (except that caused by tuberculosis or sexually transmitted disease)
122	J156	Pneumonia (except that caused by tuberculosis or sexually transmitted disease)
122	J157	Pneumonia (except that caused by tuberculosis or sexually transmitted disease)
122	J158	Pneumonia (except that caused by tuberculosis or sexually transmitted disease)
122	J159	Pneumonia (except that caused by tuberculosis or sexually transmitted disease)
122	J160	Pneumonia (except that caused by tuberculosis or sexually transmitted disease)
122	J168	Pneumonia (except that caused by tuberculosis or sexually transmitted disease)
122	J17	Pneumonia (except that caused by tuberculosis or sexually transmitted disease)
122	J180	Pneumonia (except that caused by tuberculosis or sexually transmitted disease)
122	J181	Pneumonia (except that caused by tuberculosis or sexually transmitted disease)
122	J188	Pneumonia (except that caused by tuberculosis or sexually transmitted disease)
122	J189	Pneumonia (except that caused by tuberculosis or sexually transmitted disease)
122	J851	Pneumonia (except that caused by tuberculosis or sexually transmitted disease)
122	J852	Pneumonia (except that caused by tuberculosis or sexually transmitted disease)
123	J09X1	Influenza
123	J09X2	Influenza
123	J09X3	Influenza
123	J09X9	Influenza
123	J1000	Influenza
123	J1001	Influenza
123	J1008	Influenza

123	J101	Influenza
123	J102	Influenza
123	J1089	Influenza
123	J1100	Influenza
123	J1108	Influenza
123	J111	Influenza
123	J112	Influenza
123	J1181	Influenza
123	J1182	Influenza
123	J1183	Influenza
123	J1189	Influenza
124	J0380	Acute and chronic tonsillitis
124	J0381	Acute and chronic tonsillitis
124	J0390	Acute and chronic tonsillitis
124	J0391	Acute and chronic tonsillitis
124	J3501	Acute and chronic tonsillitis
124	J3502	Acute and chronic tonsillitis
124	J3503	Acute and chronic tonsillitis
124	J351	Acute and chronic tonsillitis
124	J352	Acute and chronic tonsillitis
124	J353	Acute and chronic tonsillitis
124	J358	Acute and chronic tonsillitis
124	J359	Acute and chronic tonsillitis
124	J36	Acute and chronic tonsillitis
125	J200	Acute bronchitis
125	J201	Acute bronchitis
125	J202	Acute bronchitis
125	J203	Acute bronchitis
125	J204	Acute bronchitis
125	J205	Acute bronchitis
125	J206	Acute bronchitis
125	J207	Acute bronchitis
125	J208	Acute bronchitis
125	J209	Acute bronchitis
125	J210	Acute bronchitis
125	J211	Acute bronchitis
125	J218	Acute bronchitis
125	J219	Acute bronchitis
126	A360	Other upper respiratory infections
126	A361	Other upper respiratory infections
126	A362	Other upper respiratory infections
126	J00	Other upper respiratory infections
126	J0100	Other upper respiratory infections
126	J0101	Other upper respiratory infections
126	J0110	Other upper respiratory infections
126	J0111	Other upper respiratory infections
126	J0120	Other upper respiratory infections
126	J0121	Other upper respiratory infections
126	J0130	Other upper respiratory infections
126	J0131	Other upper respiratory infections
126	J0140	Other upper respiratory infections

126	J0141	Other upper respiratory infections
126	J0180	Other upper respiratory infections
126	J0181	Other upper respiratory infections
126	J0190	Other upper respiratory infections
126	J0191	Other upper respiratory infections
126	J020	Other upper respiratory infections
126	J028	Other upper respiratory infections
126	J029	Other upper respiratory infections
126	J0300	Other upper respiratory infections
126	J0301	Other upper respiratory infections
126	J040	Other upper respiratory infections
126	J0410	Other upper respiratory infections
126	J0411	Other upper respiratory infections
126	J042	Other upper respiratory infections
126	J0430	Other upper respiratory infections
126	J0431	Other upper respiratory infections
126	J050	Other upper respiratory infections
126	J0510	Other upper respiratory infections
126	J0511	Other upper respiratory infections
126	J060	Other upper respiratory infections
126	J069	Other upper respiratory infections
126	J320	Other upper respiratory infections
126	J321	Other upper respiratory infections
126	J322	Other upper respiratory infections
126	J323	Other upper respiratory infections
126	J324	Other upper respiratory infections
126	J328	Other upper respiratory infections
126	J329	Other upper respiratory infections
126	R0982	Other upper respiratory infections
127	J40	Chronic obstructive pulmonary disease and bronchiectasis
127	J410	Chronic obstructive pulmonary disease and bronchiectasis
127	J411	Chronic obstructive pulmonary disease and bronchiectasis
127	J418	Chronic obstructive pulmonary disease and bronchiectasis
127	J42	Chronic obstructive pulmonary disease and bronchiectasis
127	J430	Chronic obstructive pulmonary disease and bronchiectasis
127	J431	Chronic obstructive pulmonary disease and bronchiectasis
127	J432	Chronic obstructive pulmonary disease and bronchiectasis
127	J438	Chronic obstructive pulmonary disease and bronchiectasis
127	J439	Chronic obstructive pulmonary disease and bronchiectasis
127	J440	Chronic obstructive pulmonary disease and bronchiectasis
127	J441	Chronic obstructive pulmonary disease and bronchiectasis
127	J449	Chronic obstructive pulmonary disease and bronchiectasis
127	J470	Chronic obstructive pulmonary disease and bronchiectasis
127	J471	Chronic obstructive pulmonary disease and bronchiectasis
127	J479	Chronic obstructive pulmonary disease and bronchiectasis
128	J4520	Asthma
128	J4521	Asthma
128	J4522	Asthma
128	J4530	Asthma
128	J4531	Asthma
128	J4532	Asthma

128	J4540	Asthma
128	J4541	Asthma
128	J4542	Asthma
128	J4550	Asthma
128	J4551	Asthma
128	J4552	Asthma
128	J45901	Asthma
128	J45902	Asthma
128	J45909	Asthma
128	J45990	Asthma
128	J45991	Asthma
128	J45998	Asthma
129	J690	Aspiration pneumonitis; food/vomitus
130	J860	Pleurisy; pneumothorax; pulmonary collapse
130	J869	Pleurisy; pneumothorax; pulmonary collapse
130	J90	Pleurisy; pneumothorax; pulmonary collapse
130	J918	Pleurisy; pneumothorax; pulmonary collapse
130	J920	Pleurisy; pneumothorax; pulmonary collapse
130	J929	Pleurisy; pneumothorax; pulmonary collapse
130	J930	Pleurisy; pneumothorax; pulmonary collapse
130	J9311	Pleurisy; pneumothorax; pulmonary collapse
130	J9312	Pleurisy; pneumothorax; pulmonary collapse
130	J9381	Pleurisy; pneumothorax; pulmonary collapse
130	J9382	Pleurisy; pneumothorax; pulmonary collapse
130	J9383	Pleurisy; pneumothorax; pulmonary collapse
130	J939	Pleurisy; pneumothorax; pulmonary collapse
130	J940	Pleurisy; pneumothorax; pulmonary collapse
130	J941	Pleurisy; pneumothorax; pulmonary collapse
130	J942	Pleurisy; pneumothorax; pulmonary collapse
130	J948	Pleurisy; pneumothorax; pulmonary collapse
130	J949	Pleurisy; pneumothorax; pulmonary collapse
130	J9811	Pleurisy; pneumothorax; pulmonary collapse
130	J9819	Pleurisy; pneumothorax; pulmonary collapse
130	J982	Pleurisy; pneumothorax; pulmonary collapse
130	J983	Pleurisy; pneumothorax; pulmonary collapse
130	R091	Pleurisy; pneumothorax; pulmonary collapse
131	J80	Respiratory failure; insufficiency; arrest (adult)
131	J9600	Respiratory failure; insufficiency; arrest (adult)
131	J9601	Respiratory failure; insufficiency; arrest (adult)
131	J9602	Respiratory failure; insufficiency; arrest (adult)
131	J9610	Respiratory failure; insufficiency; arrest (adult)
131	J9611	Respiratory failure; insufficiency; arrest (adult)
131	J9612	Respiratory failure; insufficiency; arrest (adult)
131	J9620	Respiratory failure; insufficiency; arrest (adult)
131	J9621	Respiratory failure; insufficiency; arrest (adult)
131	J9622	Respiratory failure; insufficiency; arrest (adult)
131	J9690	Respiratory failure; insufficiency; arrest (adult)
131	J9691	Respiratory failure; insufficiency; arrest (adult)
131	J9692	Respiratory failure; insufficiency; arrest (adult)
131	R0901	Respiratory failure; insufficiency; arrest (adult)
131	R092	Respiratory failure; insufficiency; arrest (adult)

131	Z9911	Respiratory failure; insufficiency; arrest (adult)
131	Z9912	Respiratory failure; insufficiency; arrest (adult)
131	Z9981	Respiratory failure; insufficiency; arrest (adult)
132	J60	Lung disease due to external agents
132	J61	Lung disease due to external agents
132	J620	Lung disease due to external agents
132	J628	Lung disease due to external agents
132	J630	Lung disease due to external agents
132	J631	Lung disease due to external agents
132	J632	Lung disease due to external agents
132	J633	Lung disease due to external agents
132	J634	Lung disease due to external agents
132	J635	Lung disease due to external agents
132	J636	Lung disease due to external agents
132	J64	Lung disease due to external agents
132	J65	Lung disease due to external agents
132	J660	Lung disease due to external agents
132	J661	Lung disease due to external agents
132	J662	Lung disease due to external agents
132	J668	Lung disease due to external agents
132	J670	Lung disease due to external agents
132	J671	Lung disease due to external agents
132	J672	Lung disease due to external agents
132	J673	Lung disease due to external agents
132	J674	Lung disease due to external agents
132	J675	Lung disease due to external agents
132	J676	Lung disease due to external agents
132	J677	Lung disease due to external agents
132	J678	Lung disease due to external agents
132	J679	Lung disease due to external agents
132	J680	Lung disease due to external agents
132	J681	Lung disease due to external agents
132	J682	Lung disease due to external agents
132	J683	Lung disease due to external agents
132	J684	Lung disease due to external agents
132	J688	Lung disease due to external agents
132	J689	Lung disease due to external agents
132	J691	Lung disease due to external agents
132	J698	Lung disease due to external agents
132	J700	Lung disease due to external agents
132	J701	Lung disease due to external agents
132	J702	Lung disease due to external agents
132	J703	Lung disease due to external agents
132	J704	Lung disease due to external agents
132	J705	Lung disease due to external agents
132	J708	Lung disease due to external agents
132	J709	Lung disease due to external agents
133	J182	Other lower respiratory disease
133	J22	Other lower respiratory disease
133	J810	Other lower respiratory disease
133	J811	Other lower respiratory disease

133	J82	Other lower respiratory disease
133	J8401	Other lower respiratory disease
133	J8402	Other lower respiratory disease
133	J8403	Other lower respiratory disease
133	J8409	Other lower respiratory disease
133	J8410	Other lower respiratory disease
133	J84111	Other lower respiratory disease
133	J84112	Other lower respiratory disease
133	J84113	Other lower respiratory disease
133	J84114	Other lower respiratory disease
133	J84115	Other lower respiratory disease
133	J84116	Other lower respiratory disease
133	J84117	Other lower respiratory disease
133	J8417	Other lower respiratory disease
133	J842	Other lower respiratory disease
133	J8481	Other lower respiratory disease
133	J8482	Other lower respiratory disease
133	J8483	Other lower respiratory disease
133	J84841	Other lower respiratory disease
133	J84842	Other lower respiratory disease
133	J84843	Other lower respiratory disease
133	J84848	Other lower respiratory disease
133	J8489	Other lower respiratory disease
133	J849	Other lower respiratory disease
133	J853	Other lower respiratory disease
133	J984	Other lower respiratory disease
133	J986	Other lower respiratory disease
133	J988	Other lower respiratory disease
133	J989	Other lower respiratory disease
133	J99	Other lower respiratory disease
133	R042	Other lower respiratory disease
133	R0481	Other lower respiratory disease
133	R0489	Other lower respiratory disease
133	R049	Other lower respiratory disease
133	R05	Other lower respiratory disease
133	R0600	Other lower respiratory disease
133	R0601	Other lower respiratory disease
133	R0602	Other lower respiratory disease
133	R0609	Other lower respiratory disease
133	R062	Other lower respiratory disease
133	R063	Other lower respiratory disease
133	R064	Other lower respiratory disease
133	R066	Other lower respiratory disease
133	R0681	Other lower respiratory disease
133	R0682	Other lower respiratory disease
133	R0683	Other lower respiratory disease
133	R0689	Other lower respiratory disease
133	R069	Other lower respiratory disease
133	R071	Other lower respiratory disease
133	R0781	Other lower respiratory disease
133	R0902	Other lower respiratory disease

133	R093	Other lower respiratory disease
133	R230	Other lower respiratory disease
133	R911	Other lower respiratory disease
133	R918	Other lower respiratory disease
133	Z8701	Other lower respiratory disease
133	Z8709	Other lower respiratory disease
133	Z942	Other lower respiratory disease
134	J300	Other upper respiratory disease
134	J301	Other upper respiratory disease
134	J302	Other upper respiratory disease
134	J3081	Other upper respiratory disease
134	J3089	Other upper respiratory disease
134	J309	Other upper respiratory disease
134	J310	Other upper respiratory disease
134	J311	Other upper respiratory disease
134	J312	Other upper respiratory disease
134	J330	Other upper respiratory disease
134	J331	Other upper respiratory disease
134	J338	Other upper respiratory disease
134	J339	Other upper respiratory disease
134	J340	Other upper respiratory disease
134	J341	Other upper respiratory disease
134	J342	Other upper respiratory disease
134	J343	Other upper respiratory disease
134	J3481	Other upper respiratory disease
134	J3489	Other upper respiratory disease
134	J349	Other upper respiratory disease
134	J370	Other upper respiratory disease
134	J371	Other upper respiratory disease
134	J3800	Other upper respiratory disease
134	J3801	Other upper respiratory disease
134	J3802	Other upper respiratory disease
134	J381	Other upper respiratory disease
134	J382	Other upper respiratory disease
134	J383	Other upper respiratory disease
134	J384	Other upper respiratory disease
134	J385	Other upper respiratory disease
134	J386	Other upper respiratory disease
134	J387	Other upper respiratory disease
134	J390	Other upper respiratory disease
134	J391	Other upper respiratory disease
134	J392	Other upper respiratory disease
134	J393	Other upper respiratory disease
134	J398	Other upper respiratory disease
134	J399	Other upper respiratory disease
134	J9801	Other upper respiratory disease
134	J9809	Other upper respiratory disease
134	J985	Other upper respiratory disease
134	R040	Other upper respiratory disease
134	R041	Other upper respiratory disease
134	R061	Other upper respiratory disease

134	R065	Other upper respiratory disease
134	R067	Other upper respiratory disease
134	R070	Other upper respiratory disease
134	R0981	Other upper respiratory disease
134	R196	Other upper respiratory disease
134	R490	Other upper respiratory disease
134	R491	Other upper respiratory disease
134	R4921	Other upper respiratory disease
134	R4922	Other upper respiratory disease
134	R498	Other upper respiratory disease
134	R499	Other upper respiratory disease
134	Z430	Other upper respiratory disease
134	Z930	Other upper respiratory disease
134	Z963	Other upper respiratory disease
135	A000	Intestinal infection
135	A001	Intestinal infection
135	A009	Intestinal infection
135	A0100	Intestinal infection
135	A0109	Intestinal infection
135	A011	Intestinal infection
135	A012	Intestinal infection
135	A013	Intestinal infection
135	A014	Intestinal infection
135	A020	Intestinal infection
135	A0220	Intestinal infection
135	A0225	Intestinal infection
135	A0229	Intestinal infection
135	A028	Intestinal infection
135	A029	Intestinal infection
135	A030	Intestinal infection
135	A031	Intestinal infection
135	A032	Intestinal infection
135	A033	Intestinal infection
135	A038	Intestinal infection
135	A039	Intestinal infection
135	A040	Intestinal infection
135	A041	Intestinal infection
135	A042	Intestinal infection
135	A043	Intestinal infection
135	A044	Intestinal infection
135	A045	Intestinal infection
135	A046	Intestinal infection
135	A047	Intestinal infection
135	A048	Intestinal infection
135	A049	Intestinal infection
135	A050	Intestinal infection
135	A051	Intestinal infection
135	A052	Intestinal infection
135	A053	Intestinal infection
135	A054	Intestinal infection
135	A055	Intestinal infection

135	A058	Intestinal infection
135	A059	Intestinal infection
135	A060	Intestinal infection
135	A061	Intestinal infection
135	A062	Intestinal infection
135	A063	Intestinal infection
135	A064	Intestinal infection
135	A065	Intestinal infection
135	A066	Intestinal infection
135	A067	Intestinal infection
135	A0681	Intestinal infection
135	A0682	Intestinal infection
135	A0689	Intestinal infection
135	A069	Intestinal infection
135	A070	Intestinal infection
135	A071	Intestinal infection
135	A072	Intestinal infection
135	A073	Intestinal infection
135	A074	Intestinal infection
135	A078	Intestinal infection
135	A079	Intestinal infection
135	A080	Intestinal infection
135	A0811	Intestinal infection
135	A0819	Intestinal infection
135	A082	Intestinal infection
135	A0831	Intestinal infection
135	A0832	Intestinal infection
135	A0839	Intestinal infection
135	A084	Intestinal infection
135	A088	Intestinal infection
135	A09	Intestinal infection
135	A213	Intestinal infection
135	A222	Intestinal infection
136	K000	Disorders of teeth and jaw
136	K001	Disorders of teeth and jaw
136	K002	Disorders of teeth and jaw
136	K003	Disorders of teeth and jaw
136	K004	Disorders of teeth and jaw
136	K005	Disorders of teeth and jaw
136	K006	Disorders of teeth and jaw
136	K007	Disorders of teeth and jaw
136	K008	Disorders of teeth and jaw
136	K009	Disorders of teeth and jaw
136	K010	Disorders of teeth and jaw
136	K011	Disorders of teeth and jaw
136	K023	Disorders of teeth and jaw
136	K0251	Disorders of teeth and jaw
136	K0252	Disorders of teeth and jaw
136	K0253	Disorders of teeth and jaw
136	K0261	Disorders of teeth and jaw
136	K0262	Disorders of teeth and jaw

136	K0263	Disorders of teeth and jaw
136	K027	Disorders of teeth and jaw
136	K029	Disorders of teeth and jaw
136	K030	Disorders of teeth and jaw
136	K031	Disorders of teeth and jaw
136	K032	Disorders of teeth and jaw
136	K033	Disorders of teeth and jaw
136	K034	Disorders of teeth and jaw
136	K035	Disorders of teeth and jaw
136	K036	Disorders of teeth and jaw
136	K037	Disorders of teeth and jaw
136	K0381	Disorders of teeth and jaw
136	K0389	Disorders of teeth and jaw
136	K039	Disorders of teeth and jaw
136	K040	Disorders of teeth and jaw
136	K041	Disorders of teeth and jaw
136	K042	Disorders of teeth and jaw
136	K043	Disorders of teeth and jaw
136	K044	Disorders of teeth and jaw
136	K045	Disorders of teeth and jaw
136	K046	Disorders of teeth and jaw
136	K047	Disorders of teeth and jaw
136	K048	Disorders of teeth and jaw
136	K0490	Disorders of teeth and jaw
136	K0499	Disorders of teeth and jaw
136	K0500	Disorders of teeth and jaw
136	K0501	Disorders of teeth and jaw
136	K0510	Disorders of teeth and jaw
136	K0511	Disorders of teeth and jaw
136	K0520	Disorders of teeth and jaw
136	K0521	Disorders of teeth and jaw
136	K0522	Disorders of teeth and jaw
136	K0530	Disorders of teeth and jaw
136	K0531	Disorders of teeth and jaw
136	K0532	Disorders of teeth and jaw
136	K054	Disorders of teeth and jaw
136	K055	Disorders of teeth and jaw
136	K056	Disorders of teeth and jaw
136	K060	Disorders of teeth and jaw
136	K061	Disorders of teeth and jaw
136	K062	Disorders of teeth and jaw
136	K068	Disorders of teeth and jaw
136	K069	Disorders of teeth and jaw
136	K080	Disorders of teeth and jaw
136	K08101	Disorders of teeth and jaw
136	K08102	Disorders of teeth and jaw
136	K08103	Disorders of teeth and jaw
136	K08104	Disorders of teeth and jaw
136	K08109	Disorders of teeth and jaw
136	K08111	Disorders of teeth and jaw
136	K08112	Disorders of teeth and jaw

136	K08113	Disorders of teeth and jaw
136	K08114	Disorders of teeth and jaw
136	K08119	Disorders of teeth and jaw
136	K08121	Disorders of teeth and jaw
136	K08122	Disorders of teeth and jaw
136	K08123	Disorders of teeth and jaw
136	K08124	Disorders of teeth and jaw
136	K08129	Disorders of teeth and jaw
136	K08131	Disorders of teeth and jaw
136	K08132	Disorders of teeth and jaw
136	K08133	Disorders of teeth and jaw
136	K08134	Disorders of teeth and jaw
136	K08139	Disorders of teeth and jaw
136	K08191	Disorders of teeth and jaw
136	K08192	Disorders of teeth and jaw
136	K08193	Disorders of teeth and jaw
136	K08194	Disorders of teeth and jaw
136	K08199	Disorders of teeth and jaw
136	K0820	Disorders of teeth and jaw
136	K0821	Disorders of teeth and jaw
136	K0822	Disorders of teeth and jaw
136	K0823	Disorders of teeth and jaw
136	K0824	Disorders of teeth and jaw
136	K0825	Disorders of teeth and jaw
136	K0826	Disorders of teeth and jaw
136	K083	Disorders of teeth and jaw
136	K08401	Disorders of teeth and jaw
136	K08402	Disorders of teeth and jaw
136	K08403	Disorders of teeth and jaw
136	K08404	Disorders of teeth and jaw
136	K08409	Disorders of teeth and jaw
136	K08411	Disorders of teeth and jaw
136	K08412	Disorders of teeth and jaw
136	K08413	Disorders of teeth and jaw
136	K08414	Disorders of teeth and jaw
136	K08419	Disorders of teeth and jaw
136	K08421	Disorders of teeth and jaw
136	K08422	Disorders of teeth and jaw
136	K08423	Disorders of teeth and jaw
136	K08424	Disorders of teeth and jaw
136	K08429	Disorders of teeth and jaw
136	K08431	Disorders of teeth and jaw
136	K08432	Disorders of teeth and jaw
136	K08433	Disorders of teeth and jaw
136	K08434	Disorders of teeth and jaw
136	K08439	Disorders of teeth and jaw
136	K08491	Disorders of teeth and jaw
136	K08492	Disorders of teeth and jaw
136	K08493	Disorders of teeth and jaw
136	K08494	Disorders of teeth and jaw
136	K08499	Disorders of teeth and jaw

136	K0850	Disorders of teeth and jaw
136	K0851	Disorders of teeth and jaw
136	K0852	Disorders of teeth and jaw
136	K08530	Disorders of teeth and jaw
136	K08531	Disorders of teeth and jaw
136	K08539	Disorders of teeth and jaw
136	K0854	Disorders of teeth and jaw
136	K0855	Disorders of teeth and jaw
136	K0856	Disorders of teeth and jaw
136	K0859	Disorders of teeth and jaw
136	K088	Disorders of teeth and jaw
136	K089	Disorders of teeth and jaw
136	K090	Disorders of teeth and jaw
136	K091	Disorders of teeth and jaw
136	M2600	Disorders of teeth and jaw
136	M2601	Disorders of teeth and jaw
136	M2602	Disorders of teeth and jaw
136	M2603	Disorders of teeth and jaw
136	M2604	Disorders of teeth and jaw
136	M2605	Disorders of teeth and jaw
136	M2606	Disorders of teeth and jaw
136	M2607	Disorders of teeth and jaw
136	M2609	Disorders of teeth and jaw
136	M2610	Disorders of teeth and jaw
136	M2611	Disorders of teeth and jaw
136	M2612	Disorders of teeth and jaw
136	M2619	Disorders of teeth and jaw
136	M2620	Disorders of teeth and jaw
136	M26211	Disorders of teeth and jaw
136	M26212	Disorders of teeth and jaw
136	M26213	Disorders of teeth and jaw
136	M26219	Disorders of teeth and jaw
136	M26220	Disorders of teeth and jaw
136	M26221	Disorders of teeth and jaw
136	M2623	Disorders of teeth and jaw
136	M2624	Disorders of teeth and jaw
136	M2625	Disorders of teeth and jaw
136	M2629	Disorders of teeth and jaw
136	M2630	Disorders of teeth and jaw
136	M2631	Disorders of teeth and jaw
136	M2632	Disorders of teeth and jaw
136	M2633	Disorders of teeth and jaw
136	M2634	Disorders of teeth and jaw
136	M2635	Disorders of teeth and jaw
136	M2636	Disorders of teeth and jaw
136	M2637	Disorders of teeth and jaw
136	M2639	Disorders of teeth and jaw
136	M264	Disorders of teeth and jaw
136	M2650	Disorders of teeth and jaw
136	M2651	Disorders of teeth and jaw
136	M2652	Disorders of teeth and jaw

136	M2653	Disorders of teeth and jaw
136	M2654	Disorders of teeth and jaw
136	M2655	Disorders of teeth and jaw
136	M2656	Disorders of teeth and jaw
136	M2657	Disorders of teeth and jaw
136	M2659	Disorders of teeth and jaw
136	M2660	Disorders of teeth and jaw
136	M2661	Disorders of teeth and jaw
136	M2662	Disorders of teeth and jaw
136	M2663	Disorders of teeth and jaw
136	M2669	Disorders of teeth and jaw
136	M2670	Disorders of teeth and jaw
136	M2671	Disorders of teeth and jaw
136	M2672	Disorders of teeth and jaw
136	M2673	Disorders of teeth and jaw
136	M2674	Disorders of teeth and jaw
136	M2679	Disorders of teeth and jaw
136	M2681	Disorders of teeth and jaw
136	M2682	Disorders of teeth and jaw
136	M2689	Disorders of teeth and jaw
136	M269	Disorders of teeth and jaw
136	M270	Disorders of teeth and jaw
136	M271	Disorders of teeth and jaw
136	M272	Disorders of teeth and jaw
136	M273	Disorders of teeth and jaw
136	M2740	Disorders of teeth and jaw
136	M2749	Disorders of teeth and jaw
136	M2751	Disorders of teeth and jaw
136	M2752	Disorders of teeth and jaw
136	M2753	Disorders of teeth and jaw
136	M2759	Disorders of teeth and jaw
136	M2761	Disorders of teeth and jaw
136	M2762	Disorders of teeth and jaw
136	M2763	Disorders of teeth and jaw
136	M2769	Disorders of teeth and jaw
136	M278	Disorders of teeth and jaw
136	M279	Disorders of teeth and jaw
136	R6884	Disorders of teeth and jaw
136	S032XXA	Disorders of teeth and jaw
136	S032XXD	Disorders of teeth and jaw
136	S032XXS	Disorders of teeth and jaw
136	Z463	Disorders of teeth and jaw
136	Z464	Disorders of teeth and jaw
136	Z965	Disorders of teeth and jaw
136	Z98810	Disorders of teeth and jaw
136	Z98811	Disorders of teeth and jaw
136	Z98818	Disorders of teeth and jaw
137	K098	Diseases of mouth; excluding dental
137	K099	Diseases of mouth; excluding dental
137	K110	Diseases of mouth; excluding dental
137	K111	Diseases of mouth; excluding dental

137	K1120	Diseases of mouth; excluding dental
137	K1121	Diseases of mouth; excluding dental
137	K1122	Diseases of mouth; excluding dental
137	K1123	Diseases of mouth; excluding dental
137	K113	Diseases of mouth; excluding dental
137	K114	Diseases of mouth; excluding dental
137	K115	Diseases of mouth; excluding dental
137	K116	Diseases of mouth; excluding dental
137	K117	Diseases of mouth; excluding dental
137	K118	Diseases of mouth; excluding dental
137	K119	Diseases of mouth; excluding dental
137	K120	Diseases of mouth; excluding dental
137	K121	Diseases of mouth; excluding dental
137	K122	Diseases of mouth; excluding dental
137	K1230	Diseases of mouth; excluding dental
137	K1239	Diseases of mouth; excluding dental
137	K130	Diseases of mouth; excluding dental
137	K131	Diseases of mouth; excluding dental
137	K1321	Diseases of mouth; excluding dental
137	K1322	Diseases of mouth; excluding dental
137	K1323	Diseases of mouth; excluding dental
137	K1324	Diseases of mouth; excluding dental
137	K1329	Diseases of mouth; excluding dental
137	K133	Diseases of mouth; excluding dental
137	K134	Diseases of mouth; excluding dental
137	K135	Diseases of mouth; excluding dental
137	K136	Diseases of mouth; excluding dental
137	K1370	Diseases of mouth; excluding dental
137	K1379	Diseases of mouth; excluding dental
137	K140	Diseases of mouth; excluding dental
137	K141	Diseases of mouth; excluding dental
137	K142	Diseases of mouth; excluding dental
137	K143	Diseases of mouth; excluding dental
137	K144	Diseases of mouth; excluding dental
137	K145	Diseases of mouth; excluding dental
137	K146	Diseases of mouth; excluding dental
137	K148	Diseases of mouth; excluding dental
137	K149	Diseases of mouth; excluding dental
137	R682	Diseases of mouth; excluding dental
138	B5731	Esophageal disorders
138	I8500	Esophageal disorders
138	I8510	Esophageal disorders
138	K200	Esophageal disorders
138	K208	Esophageal disorders
138	K209	Esophageal disorders
138	K210	Esophageal disorders
138	K219	Esophageal disorders
138	K220	Esophageal disorders
138	K2210	Esophageal disorders
138	K2211	Esophageal disorders
138	K222	Esophageal disorders

138	K223	Esophageal disorders
138	K224	Esophageal disorders
138	K225	Esophageal disorders
138	K226	Esophageal disorders
138	K2270	Esophageal disorders
138	K22710	Esophageal disorders
138	K22711	Esophageal disorders
138	K22719	Esophageal disorders
138	K228	Esophageal disorders
138	K229	Esophageal disorders
138	K23	Esophageal disorders
139	K251	Gastroduodenal ulcer (except hemorrhage)
139	K253	Gastroduodenal ulcer (except hemorrhage)
139	K255	Gastroduodenal ulcer (except hemorrhage)
139	K257	Gastroduodenal ulcer (except hemorrhage)
139	K259	Gastroduodenal ulcer (except hemorrhage)
139	K261	Gastroduodenal ulcer (except hemorrhage)
139	K263	Gastroduodenal ulcer (except hemorrhage)
139	K265	Gastroduodenal ulcer (except hemorrhage)
139	K267	Gastroduodenal ulcer (except hemorrhage)
139	K269	Gastroduodenal ulcer (except hemorrhage)
139	K271	Gastroduodenal ulcer (except hemorrhage)
139	K273	Gastroduodenal ulcer (except hemorrhage)
139	K275	Gastroduodenal ulcer (except hemorrhage)
139	K277	Gastroduodenal ulcer (except hemorrhage)
139	K279	Gastroduodenal ulcer (except hemorrhage)
139	K281	Gastroduodenal ulcer (except hemorrhage)
139	K283	Gastroduodenal ulcer (except hemorrhage)
139	K285	Gastroduodenal ulcer (except hemorrhage)
139	K287	Gastroduodenal ulcer (except hemorrhage)
139	K289	Gastroduodenal ulcer (except hemorrhage)
139	Z8711	Gastroduodenal ulcer (except hemorrhage)
140	K2900	Gastritis and duodenitis
140	K2901	Gastritis and duodenitis
140	K2930	Gastritis and duodenitis
140	K2931	Gastritis and duodenitis
140	K2940	Gastritis and duodenitis
140	K2941	Gastritis and duodenitis
140	K2950	Gastritis and duodenitis
140	K2951	Gastritis and duodenitis
140	K2960	Gastritis and duodenitis
140	K2961	Gastritis and duodenitis
140	K2970	Gastritis and duodenitis
140	K2971	Gastritis and duodenitis
140	K2980	Gastritis and duodenitis
140	K2981	Gastritis and duodenitis
140	K2990	Gastritis and duodenitis
140	K2991	Gastritis and duodenitis
141	K30	Other disorders of stomach and duodenum
141	K310	Other disorders of stomach and duodenum
141	K311	Other disorders of stomach and duodenum

141	K312	Other disorders of stomach and duodenum
141	K313	Other disorders of stomach and duodenum
141	K314	Other disorders of stomach and duodenum
141	K315	Other disorders of stomach and duodenum
141	K316	Other disorders of stomach and duodenum
141	K31811	Other disorders of stomach and duodenum
141	K31819	Other disorders of stomach and duodenum
141	K3182	Other disorders of stomach and duodenum
141	K3183	Other disorders of stomach and duodenum
141	K3184	Other disorders of stomach and duodenum
141	K3189	Other disorders of stomach and duodenum
141	K319	Other disorders of stomach and duodenum
142	K352	Appendicitis and other appendiceal conditions
142	K353	Appendicitis and other appendiceal conditions
142	K3580	Appendicitis and other appendiceal conditions
142	K3589	Appendicitis and other appendiceal conditions
142	K36	Appendicitis and other appendiceal conditions
142	K37	Appendicitis and other appendiceal conditions
142	K380	Appendicitis and other appendiceal conditions
142	K381	Appendicitis and other appendiceal conditions
142	K382	Appendicitis and other appendiceal conditions
142	K383	Appendicitis and other appendiceal conditions
142	K388	Appendicitis and other appendiceal conditions
142	K389	Appendicitis and other appendiceal conditions
143	K4000	Abdominal hernia
143	K4001	Abdominal hernia
143	K4010	Abdominal hernia
143	K4011	Abdominal hernia
143	K4020	Abdominal hernia
143	K4021	Abdominal hernia
143	K4030	Abdominal hernia
143	K4031	Abdominal hernia
143	K4040	Abdominal hernia
143	K4041	Abdominal hernia
143	K4090	Abdominal hernia
143	K4091	Abdominal hernia
143	K4100	Abdominal hernia
143	K4101	Abdominal hernia
143	K4110	Abdominal hernia
143	K4111	Abdominal hernia
143	K4120	Abdominal hernia
143	K4121	Abdominal hernia
143	K4130	Abdominal hernia
143	K4131	Abdominal hernia
143	K4140	Abdominal hernia
143	K4141	Abdominal hernia
143	K4190	Abdominal hernia
143	K4191	Abdominal hernia
143	K420	Abdominal hernia
143	K421	Abdominal hernia
143	K429	Abdominal hernia

143	K430	Abdominal hernia
143	K431	Abdominal hernia
143	K432	Abdominal hernia
143	K433	Abdominal hernia
143	K434	Abdominal hernia
143	K435	Abdominal hernia
143	K436	Abdominal hernia
143	K437	Abdominal hernia
143	K439	Abdominal hernia
143	K440	Abdominal hernia
143	K441	Abdominal hernia
143	K449	Abdominal hernia
143	K450	Abdominal hernia
143	K451	Abdominal hernia
143	K458	Abdominal hernia
143	K460	Abdominal hernia
143	K461	Abdominal hernia
143	K469	Abdominal hernia
144	K5000	Regional enteritis and ulcerative colitis
144	K50011	Regional enteritis and ulcerative colitis
144	K50012	Regional enteritis and ulcerative colitis
144	K50013	Regional enteritis and ulcerative colitis
144	K50014	Regional enteritis and ulcerative colitis
144	K50018	Regional enteritis and ulcerative colitis
144	K50019	Regional enteritis and ulcerative colitis
144	K5010	Regional enteritis and ulcerative colitis
144	K50111	Regional enteritis and ulcerative colitis
144	K50112	Regional enteritis and ulcerative colitis
144	K50113	Regional enteritis and ulcerative colitis
144	K50114	Regional enteritis and ulcerative colitis
144	K50118	Regional enteritis and ulcerative colitis
144	K50119	Regional enteritis and ulcerative colitis
144	K5080	Regional enteritis and ulcerative colitis
144	K50811	Regional enteritis and ulcerative colitis
144	K50812	Regional enteritis and ulcerative colitis
144	K50813	Regional enteritis and ulcerative colitis
144	K50814	Regional enteritis and ulcerative colitis
144	K50818	Regional enteritis and ulcerative colitis
144	K50819	Regional enteritis and ulcerative colitis
144	K5090	Regional enteritis and ulcerative colitis
144	K50911	Regional enteritis and ulcerative colitis
144	K50912	Regional enteritis and ulcerative colitis
144	K50913	Regional enteritis and ulcerative colitis
144	K50914	Regional enteritis and ulcerative colitis
144	K50918	Regional enteritis and ulcerative colitis
144	K50919	Regional enteritis and ulcerative colitis
144	K5100	Regional enteritis and ulcerative colitis
144	K51011	Regional enteritis and ulcerative colitis
144	K51012	Regional enteritis and ulcerative colitis
144	K51013	Regional enteritis and ulcerative colitis
144	K51014	Regional enteritis and ulcerative colitis

144	K51018	Regional enteritis and ulcerative colitis
144	K51019	Regional enteritis and ulcerative colitis
144	K5120	Regional enteritis and ulcerative colitis
144	K51211	Regional enteritis and ulcerative colitis
144	K51212	Regional enteritis and ulcerative colitis
144	K51213	Regional enteritis and ulcerative colitis
144	K51214	Regional enteritis and ulcerative colitis
144	K51218	Regional enteritis and ulcerative colitis
144	K51219	Regional enteritis and ulcerative colitis
144	K5130	Regional enteritis and ulcerative colitis
144	K51311	Regional enteritis and ulcerative colitis
144	K51312	Regional enteritis and ulcerative colitis
144	K51313	Regional enteritis and ulcerative colitis
144	K51314	Regional enteritis and ulcerative colitis
144	K51318	Regional enteritis and ulcerative colitis
144	K51319	Regional enteritis and ulcerative colitis
144	K5140	Regional enteritis and ulcerative colitis
144	K51411	Regional enteritis and ulcerative colitis
144	K51412	Regional enteritis and ulcerative colitis
144	K51413	Regional enteritis and ulcerative colitis
144	K51414	Regional enteritis and ulcerative colitis
144	K51418	Regional enteritis and ulcerative colitis
144	K51419	Regional enteritis and ulcerative colitis
144	K5150	Regional enteritis and ulcerative colitis
144	K51511	Regional enteritis and ulcerative colitis
144	K51512	Regional enteritis and ulcerative colitis
144	K51513	Regional enteritis and ulcerative colitis
144	K51514	Regional enteritis and ulcerative colitis
144	K51518	Regional enteritis and ulcerative colitis
144	K51519	Regional enteritis and ulcerative colitis
144	K5180	Regional enteritis and ulcerative colitis
144	K51811	Regional enteritis and ulcerative colitis
144	K51812	Regional enteritis and ulcerative colitis
144	K51813	Regional enteritis and ulcerative colitis
144	K51814	Regional enteritis and ulcerative colitis
144	K51818	Regional enteritis and ulcerative colitis
144	K51819	Regional enteritis and ulcerative colitis
144	K5190	Regional enteritis and ulcerative colitis
144	K51911	Regional enteritis and ulcerative colitis
144	K51912	Regional enteritis and ulcerative colitis
144	K51913	Regional enteritis and ulcerative colitis
144	K51914	Regional enteritis and ulcerative colitis
144	K51918	Regional enteritis and ulcerative colitis
144	K51919	Regional enteritis and ulcerative colitis
145	K560	Intestinal obstruction without hernia
145	K561	Intestinal obstruction without hernia
145	K562	Intestinal obstruction without hernia
145	K563	Intestinal obstruction without hernia
145	K5641	Intestinal obstruction without hernia
145	K5649	Intestinal obstruction without hernia
145	K565	Intestinal obstruction without hernia

145	K5660	Intestinal obstruction without hernia
145	K5669	Intestinal obstruction without hernia
145	K567	Intestinal obstruction without hernia
146	K5700	Diverticulosis and diverticulitis
146	K5701	Diverticulosis and diverticulitis
146	K5710	Diverticulosis and diverticulitis
146	K5711	Diverticulosis and diverticulitis
146	K5712	Diverticulosis and diverticulitis
146	K5713	Diverticulosis and diverticulitis
146	K5720	Diverticulosis and diverticulitis
146	K5721	Diverticulosis and diverticulitis
146	K5730	Diverticulosis and diverticulitis
146	K5731	Diverticulosis and diverticulitis
146	K5732	Diverticulosis and diverticulitis
146	K5733	Diverticulosis and diverticulitis
146	K5740	Diverticulosis and diverticulitis
146	K5741	Diverticulosis and diverticulitis
146	K5750	Diverticulosis and diverticulitis
146	K5751	Diverticulosis and diverticulitis
146	K5752	Diverticulosis and diverticulitis
146	K5753	Diverticulosis and diverticulitis
146	K5780	Diverticulosis and diverticulitis
146	K5781	Diverticulosis and diverticulitis
146	K5790	Diverticulosis and diverticulitis
146	K5791	Diverticulosis and diverticulitis
146	K5792	Diverticulosis and diverticulitis
146	K5793	Diverticulosis and diverticulitis
147	K594	Anal and rectal conditions
147	K600	Anal and rectal conditions
147	K601	Anal and rectal conditions
147	K602	Anal and rectal conditions
147	K603	Anal and rectal conditions
147	K604	Anal and rectal conditions
147	K605	Anal and rectal conditions
147	K610	Anal and rectal conditions
147	K611	Anal and rectal conditions
147	K612	Anal and rectal conditions
147	K613	Anal and rectal conditions
147	K614	Anal and rectal conditions
147	K620	Anal and rectal conditions
147	K621	Anal and rectal conditions
147	K622	Anal and rectal conditions
147	K623	Anal and rectal conditions
147	K624	Anal and rectal conditions
147	K626	Anal and rectal conditions
147	K627	Anal and rectal conditions
147	K6281	Anal and rectal conditions
147	K6282	Anal and rectal conditions
147	K6289	Anal and rectal conditions
147	K629	Anal and rectal conditions
148	A7481	Peritonitis and intestinal abscess

148	K630	Peritonitis and intestinal abscess
148	K650	Peritonitis and intestinal abscess
148	K651	Peritonitis and intestinal abscess
148	K652	Peritonitis and intestinal abscess
148	K653	Peritonitis and intestinal abscess
148	K654	Peritonitis and intestinal abscess
148	K658	Peritonitis and intestinal abscess
148	K659	Peritonitis and intestinal abscess
148	K67	Peritonitis and intestinal abscess
148	K6819	Peritonitis and intestinal abscess
149	K8000	Biliary tract disease
149	K8001	Biliary tract disease
149	K8010	Biliary tract disease
149	K8011	Biliary tract disease
149	K8012	Biliary tract disease
149	K8013	Biliary tract disease
149	K8018	Biliary tract disease
149	K8019	Biliary tract disease
149	K8020	Biliary tract disease
149	K8021	Biliary tract disease
149	K8030	Biliary tract disease
149	K8031	Biliary tract disease
149	K8032	Biliary tract disease
149	K8033	Biliary tract disease
149	K8034	Biliary tract disease
149	K8035	Biliary tract disease
149	K8036	Biliary tract disease
149	K8037	Biliary tract disease
149	K8040	Biliary tract disease
149	K8041	Biliary tract disease
149	K8042	Biliary tract disease
149	K8043	Biliary tract disease
149	K8044	Biliary tract disease
149	K8045	Biliary tract disease
149	K8046	Biliary tract disease
149	K8047	Biliary tract disease
149	K8050	Biliary tract disease
149	K8051	Biliary tract disease
149	K8060	Biliary tract disease
149	K8061	Biliary tract disease
149	K8062	Biliary tract disease
149	K8063	Biliary tract disease
149	K8064	Biliary tract disease
149	K8065	Biliary tract disease
149	K8066	Biliary tract disease
149	K8067	Biliary tract disease
149	K8070	Biliary tract disease
149	K8071	Biliary tract disease
149	K8080	Biliary tract disease
149	K8081	Biliary tract disease
149	K810	Biliary tract disease

149	K811	Biliary tract disease
149	K812	Biliary tract disease
149	K819	Biliary tract disease
149	K820	Biliary tract disease
149	K821	Biliary tract disease
149	K822	Biliary tract disease
149	K823	Biliary tract disease
149	K824	Biliary tract disease
149	K828	Biliary tract disease
149	K829	Biliary tract disease
149	K830	Biliary tract disease
149	K831	Biliary tract disease
149	K832	Biliary tract disease
149	K833	Biliary tract disease
149	K834	Biliary tract disease
149	K835	Biliary tract disease
149	K838	Biliary tract disease
149	K839	Biliary tract disease
149	K87	Biliary tract disease
149	K915	Biliary tract disease
151	K7041	Other liver diseases
151	K7110	Other liver diseases
151	K7111	Other liver diseases
151	K718	Other liver diseases
151	K719	Other liver diseases
151	K7200	Other liver diseases
151	K7201	Other liver diseases
151	K7210	Other liver diseases
151	K7211	Other liver diseases
151	K7290	Other liver diseases
151	K7291	Other liver diseases
151	K740	Other liver diseases
151	K741	Other liver diseases
151	K742	Other liver diseases
151	K743	Other liver diseases
151	K744	Other liver diseases
151	K745	Other liver diseases
151	K7460	Other liver diseases
151	K7469	Other liver diseases
151	K750	Other liver diseases
151	K751	Other liver diseases
151	K7589	Other liver diseases
151	K759	Other liver diseases
151	K760	Other liver diseases
151	K761	Other liver diseases
151	K762	Other liver diseases
151	K763	Other liver diseases
151	K765	Other liver diseases
151	K766	Other liver diseases
151	K767	Other liver diseases
151	K7681	Other liver diseases

151	K7689	Other liver diseases
151	K769	Other liver diseases
151	K77	Other liver diseases
151	R160	Other liver diseases
151	R162	Other liver diseases
151	R17	Other liver diseases
151	R740	Other liver diseases
151	R748	Other liver diseases
151	R749	Other liver diseases
151	Z944	Other liver diseases
152	K850	Pancreatic disorders (not diabetes)
152	K851	Pancreatic disorders (not diabetes)
152	K852	Pancreatic disorders (not diabetes)
152	K853	Pancreatic disorders (not diabetes)
152	K858	Pancreatic disorders (not diabetes)
152	K859	Pancreatic disorders (not diabetes)
152	K860	Pancreatic disorders (not diabetes)
152	K861	Pancreatic disorders (not diabetes)
152	K862	Pancreatic disorders (not diabetes)
152	K863	Pancreatic disorders (not diabetes)
152	K868	Pancreatic disorders (not diabetes)
152	K869	Pancreatic disorders (not diabetes)
152	K903	Pancreatic disorders (not diabetes)
153	I8501	Gastrointestinal hemorrhage
153	I8511	Gastrointestinal hemorrhage
153	K250	Gastrointestinal hemorrhage
153	K252	Gastrointestinal hemorrhage
153	K254	Gastrointestinal hemorrhage
153	K256	Gastrointestinal hemorrhage
153	K260	Gastrointestinal hemorrhage
153	K262	Gastrointestinal hemorrhage
153	K264	Gastrointestinal hemorrhage
153	K266	Gastrointestinal hemorrhage
153	K270	Gastrointestinal hemorrhage
153	K272	Gastrointestinal hemorrhage
153	K274	Gastrointestinal hemorrhage
153	K276	Gastrointestinal hemorrhage
153	K280	Gastrointestinal hemorrhage
153	K282	Gastrointestinal hemorrhage
153	K284	Gastrointestinal hemorrhage
153	K286	Gastrointestinal hemorrhage
153	K625	Gastrointestinal hemorrhage
153	K920	Gastrointestinal hemorrhage
153	K921	Gastrointestinal hemorrhage
153	K922	Gastrointestinal hemorrhage
154	K5281	Noninfectious gastroenteritis
154	K5282	Noninfectious gastroenteritis
154	K5289	Noninfectious gastroenteritis
154	K529	Noninfectious gastroenteritis
155	B5732	Other gastrointestinal disorders
155	K520	Other gastrointestinal disorders

155	K521	Other gastrointestinal disorders
155	K5520	Other gastrointestinal disorders
155	K5521	Other gastrointestinal disorders
155	K580	Other gastrointestinal disorders
155	K589	Other gastrointestinal disorders
155	K5900	Other gastrointestinal disorders
155	K5901	Other gastrointestinal disorders
155	K5902	Other gastrointestinal disorders
155	K5909	Other gastrointestinal disorders
155	K591	Other gastrointestinal disorders
155	K592	Other gastrointestinal disorders
155	K593	Other gastrointestinal disorders
155	K598	Other gastrointestinal disorders
155	K599	Other gastrointestinal disorders
155	K631	Other gastrointestinal disorders
155	K632	Other gastrointestinal disorders
155	K633	Other gastrointestinal disorders
155	K634	Other gastrointestinal disorders
155	K6381	Other gastrointestinal disorders
155	K6389	Other gastrointestinal disorders
155	K639	Other gastrointestinal disorders
155	K660	Other gastrointestinal disorders
155	K661	Other gastrointestinal disorders
155	K668	Other gastrointestinal disorders
155	K669	Other gastrointestinal disorders
155	K689	Other gastrointestinal disorders
155	K900	Other gastrointestinal disorders
155	K901	Other gastrointestinal disorders
155	K902	Other gastrointestinal disorders
155	K904	Other gastrointestinal disorders
155	K9089	Other gastrointestinal disorders
155	K909	Other gastrointestinal disorders
155	K9281	Other gastrointestinal disorders
155	K9289	Other gastrointestinal disorders
155	K929	Other gastrointestinal disorders
155	R1113	Other gastrointestinal disorders
155	R12	Other gastrointestinal disorders
155	R130	Other gastrointestinal disorders
155	R1310	Other gastrointestinal disorders
155	R1311	Other gastrointestinal disorders
155	R1312	Other gastrointestinal disorders
155	R1313	Other gastrointestinal disorders
155	R1314	Other gastrointestinal disorders
155	R1319	Other gastrointestinal disorders
155	R140	Other gastrointestinal disorders
155	R141	Other gastrointestinal disorders
155	R142	Other gastrointestinal disorders
155	R143	Other gastrointestinal disorders
155	R150	Other gastrointestinal disorders
155	R151	Other gastrointestinal disorders
155	R152	Other gastrointestinal disorders

155	R159	Other gastrointestinal disorders
155	R161	Other gastrointestinal disorders
155	R188	Other gastrointestinal disorders
155	R1900	Other gastrointestinal disorders
155	R1901	Other gastrointestinal disorders
155	R1902	Other gastrointestinal disorders
155	R1903	Other gastrointestinal disorders
155	R1904	Other gastrointestinal disorders
155	R1905	Other gastrointestinal disorders
155	R1906	Other gastrointestinal disorders
155	R1907	Other gastrointestinal disorders
155	R1909	Other gastrointestinal disorders
155	R1911	Other gastrointestinal disorders
155	R1912	Other gastrointestinal disorders
155	R1915	Other gastrointestinal disorders
155	R192	Other gastrointestinal disorders
155	R1930	Other gastrointestinal disorders
155	R1931	Other gastrointestinal disorders
155	R1932	Other gastrointestinal disorders
155	R1933	Other gastrointestinal disorders
155	R1934	Other gastrointestinal disorders
155	R1935	Other gastrointestinal disorders
155	R1936	Other gastrointestinal disorders
155	R1937	Other gastrointestinal disorders
155	R194	Other gastrointestinal disorders
155	R195	Other gastrointestinal disorders
155	R197	Other gastrointestinal disorders
155	R198	Other gastrointestinal disorders
155	Z431	Other gastrointestinal disorders
155	Z432	Other gastrointestinal disorders
155	Z433	Other gastrointestinal disorders
155	Z434	Other gastrointestinal disorders
155	Z4651	Other gastrointestinal disorders
155	Z4659	Other gastrointestinal disorders
155	Z8719	Other gastrointestinal disorders
155	Z931	Other gastrointestinal disorders
155	Z932	Other gastrointestinal disorders
155	Z933	Other gastrointestinal disorders
155	Z934	Other gastrointestinal disorders
155	Z9482	Other gastrointestinal disorders
155	Z980	Other gastrointestinal disorders
155	Z9884	Other gastrointestinal disorders
156	B2683	Nephritis; nephrosis; renal sclerosis
156	N000	Nephritis; nephrosis; renal sclerosis
156	N001	Nephritis; nephrosis; renal sclerosis
156	N002	Nephritis; nephrosis; renal sclerosis
156	N003	Nephritis; nephrosis; renal sclerosis
156	N004	Nephritis; nephrosis; renal sclerosis
156	N005	Nephritis; nephrosis; renal sclerosis
156	N006	Nephritis; nephrosis; renal sclerosis
156	N007	Nephritis; nephrosis; renal sclerosis

156	N008	Nephritis; nephrosis; renal sclerosis
156	N009	Nephritis; nephrosis; renal sclerosis
156	N010	Nephritis; nephrosis; renal sclerosis
156	N011	Nephritis; nephrosis; renal sclerosis
156	N012	Nephritis; nephrosis; renal sclerosis
156	N013	Nephritis; nephrosis; renal sclerosis
156	N014	Nephritis; nephrosis; renal sclerosis
156	N015	Nephritis; nephrosis; renal sclerosis
156	N016	Nephritis; nephrosis; renal sclerosis
156	N017	Nephritis; nephrosis; renal sclerosis
156	N018	Nephritis; nephrosis; renal sclerosis
156	N019	Nephritis; nephrosis; renal sclerosis
156	N020	Nephritis; nephrosis; renal sclerosis
156	N021	Nephritis; nephrosis; renal sclerosis
156	N022	Nephritis; nephrosis; renal sclerosis
156	N023	Nephritis; nephrosis; renal sclerosis
156	N024	Nephritis; nephrosis; renal sclerosis
156	N025	Nephritis; nephrosis; renal sclerosis
156	N026	Nephritis; nephrosis; renal sclerosis
156	N027	Nephritis; nephrosis; renal sclerosis
156	N028	Nephritis; nephrosis; renal sclerosis
156	N029	Nephritis; nephrosis; renal sclerosis
156	N030	Nephritis; nephrosis; renal sclerosis
156	N031	Nephritis; nephrosis; renal sclerosis
156	N032	Nephritis; nephrosis; renal sclerosis
156	N033	Nephritis; nephrosis; renal sclerosis
156	N034	Nephritis; nephrosis; renal sclerosis
156	N035	Nephritis; nephrosis; renal sclerosis
156	N036	Nephritis; nephrosis; renal sclerosis
156	N037	Nephritis; nephrosis; renal sclerosis
156	N038	Nephritis; nephrosis; renal sclerosis
156	N039	Nephritis; nephrosis; renal sclerosis
156	N040	Nephritis; nephrosis; renal sclerosis
156	N041	Nephritis; nephrosis; renal sclerosis
156	N042	Nephritis; nephrosis; renal sclerosis
156	N043	Nephritis; nephrosis; renal sclerosis
156	N044	Nephritis; nephrosis; renal sclerosis
156	N045	Nephritis; nephrosis; renal sclerosis
156	N046	Nephritis; nephrosis; renal sclerosis
156	N047	Nephritis; nephrosis; renal sclerosis
156	N048	Nephritis; nephrosis; renal sclerosis
156	N049	Nephritis; nephrosis; renal sclerosis
156	N050	Nephritis; nephrosis; renal sclerosis
156	N051	Nephritis; nephrosis; renal sclerosis
156	N052	Nephritis; nephrosis; renal sclerosis
156	N053	Nephritis; nephrosis; renal sclerosis
156	N054	Nephritis; nephrosis; renal sclerosis
156	N055	Nephritis; nephrosis; renal sclerosis
156	N056	Nephritis; nephrosis; renal sclerosis
156	N057	Nephritis; nephrosis; renal sclerosis
156	N058	Nephritis; nephrosis; renal sclerosis

156	N059	Nephritis; nephrosis; renal sclerosis
156	N060	Nephritis; nephrosis; renal sclerosis
156	N061	Nephritis; nephrosis; renal sclerosis
156	N062	Nephritis; nephrosis; renal sclerosis
156	N063	Nephritis; nephrosis; renal sclerosis
156	N064	Nephritis; nephrosis; renal sclerosis
156	N065	Nephritis; nephrosis; renal sclerosis
156	N066	Nephritis; nephrosis; renal sclerosis
156	N067	Nephritis; nephrosis; renal sclerosis
156	N068	Nephritis; nephrosis; renal sclerosis
156	N069	Nephritis; nephrosis; renal sclerosis
156	N070	Nephritis; nephrosis; renal sclerosis
156	N071	Nephritis; nephrosis; renal sclerosis
156	N072	Nephritis; nephrosis; renal sclerosis
156	N073	Nephritis; nephrosis; renal sclerosis
156	N074	Nephritis; nephrosis; renal sclerosis
156	N075	Nephritis; nephrosis; renal sclerosis
156	N076	Nephritis; nephrosis; renal sclerosis
156	N077	Nephritis; nephrosis; renal sclerosis
156	N078	Nephritis; nephrosis; renal sclerosis
156	N079	Nephritis; nephrosis; renal sclerosis
156	N08	Nephritis; nephrosis; renal sclerosis
156	N140	Nephritis; nephrosis; renal sclerosis
156	N141	Nephritis; nephrosis; renal sclerosis
156	N142	Nephritis; nephrosis; renal sclerosis
156	N143	Nephritis; nephrosis; renal sclerosis
156	N144	Nephritis; nephrosis; renal sclerosis
156	N150	Nephritis; nephrosis; renal sclerosis
156	N158	Nephritis; nephrosis; renal sclerosis
156	N261	Nephritis; nephrosis; renal sclerosis
156	N269	Nephritis; nephrosis; renal sclerosis
157	N170	Acute and unspecified renal failure
157	N171	Acute and unspecified renal failure
157	N172	Acute and unspecified renal failure
157	N178	Acute and unspecified renal failure
157	N179	Acute and unspecified renal failure
157	N19	Acute and unspecified renal failure
158	N181	Chronic kidney disease
158	N182	Chronic kidney disease
158	N183	Chronic kidney disease
158	N184	Chronic kidney disease
158	N185	Chronic kidney disease
158	N186	Chronic kidney disease
158	N189	Chronic kidney disease
158	R880	Chronic kidney disease
158	Z4901	Chronic kidney disease
158	Z4902	Chronic kidney disease
158	Z4931	Chronic kidney disease
158	Z4932	Chronic kidney disease
158	Z9115	Chronic kidney disease
158	Z940	Chronic kidney disease

158	Z992	Chronic kidney disease
159	A3685	Urinary tract infections
159	N10	Urinary tract infections
159	N110	Urinary tract infections
159	N111	Urinary tract infections
159	N118	Urinary tract infections
159	N119	Urinary tract infections
159	N12	Urinary tract infections
159	N136	Urinary tract infections
159	N151	Urinary tract infections
159	N3000	Urinary tract infections
159	N3001	Urinary tract infections
159	N3010	Urinary tract infections
159	N3011	Urinary tract infections
159	N3020	Urinary tract infections
159	N3021	Urinary tract infections
159	N3030	Urinary tract infections
159	N3031	Urinary tract infections
159	N3040	Urinary tract infections
159	N3041	Urinary tract infections
159	N3080	Urinary tract infections
159	N3081	Urinary tract infections
159	N3090	Urinary tract infections
159	N3091	Urinary tract infections
159	N340	Urinary tract infections
159	N341	Urinary tract infections
159	N342	Urinary tract infections
159	N343	Urinary tract infections
159	N35111	Urinary tract infections
159	N35112	Urinary tract infections
159	N35113	Urinary tract infections
159	N35114	Urinary tract infections
159	N35119	Urinary tract infections
159	N3512	Urinary tract infections
159	N37	Urinary tract infections
159	N390	Urinary tract infections
160	N200	Calculus of urinary tract
160	N201	Calculus of urinary tract
160	N202	Calculus of urinary tract
160	N209	Calculus of urinary tract
160	N210	Calculus of urinary tract
160	N211	Calculus of urinary tract
160	N218	Calculus of urinary tract
160	N219	Calculus of urinary tract
160	N22	Calculus of urinary tract
160	N23	Calculus of urinary tract
160	Z87442	Calculus of urinary tract
161	N131	Other diseases of kidney and ureters
161	N132	Other diseases of kidney and ureters
161	N1330	Other diseases of kidney and ureters
161	N1339	Other diseases of kidney and ureters

161	N134	Other diseases of kidney and ureters
161	N135	Other diseases of kidney and ureters
161	N1370	Other diseases of kidney and ureters
161	N1371	Other diseases of kidney and ureters
161	N13721	Other diseases of kidney and ureters
161	N13722	Other diseases of kidney and ureters
161	N13729	Other diseases of kidney and ureters
161	N13731	Other diseases of kidney and ureters
161	N13732	Other diseases of kidney and ureters
161	N13739	Other diseases of kidney and ureters
161	N138	Other diseases of kidney and ureters
161	N159	Other diseases of kidney and ureters
161	N16	Other diseases of kidney and ureters
161	N250	Other diseases of kidney and ureters
161	N251	Other diseases of kidney and ureters
161	N2581	Other diseases of kidney and ureters
161	N2589	Other diseases of kidney and ureters
161	N259	Other diseases of kidney and ureters
161	N270	Other diseases of kidney and ureters
161	N271	Other diseases of kidney and ureters
161	N279	Other diseases of kidney and ureters
161	N280	Other diseases of kidney and ureters
161	N281	Other diseases of kidney and ureters
161	N2881	Other diseases of kidney and ureters
161	N2882	Other diseases of kidney and ureters
161	N2883	Other diseases of kidney and ureters
161	N2884	Other diseases of kidney and ureters
161	N2885	Other diseases of kidney and ureters
161	N2886	Other diseases of kidney and ureters
161	N2889	Other diseases of kidney and ureters
161	N289	Other diseases of kidney and ureters
161	N29	Other diseases of kidney and ureters
161	R802	Other diseases of kidney and ureters
161	Z87441	Other diseases of kidney and ureters
162	N310	Other diseases of bladder and urethra
162	N311	Other diseases of bladder and urethra
162	N312	Other diseases of bladder and urethra
162	N318	Other diseases of bladder and urethra
162	N319	Other diseases of bladder and urethra
162	N320	Other diseases of bladder and urethra
162	N321	Other diseases of bladder and urethra
162	N322	Other diseases of bladder and urethra
162	N323	Other diseases of bladder and urethra
162	N3281	Other diseases of bladder and urethra
162	N3289	Other diseases of bladder and urethra
162	N329	Other diseases of bladder and urethra
162	N33	Other diseases of bladder and urethra
162	N35010	Other diseases of bladder and urethra
162	N35011	Other diseases of bladder and urethra
162	N35012	Other diseases of bladder and urethra
162	N35013	Other diseases of bladder and urethra

162	N35014	Other diseases of bladder and urethra
162	N35021	Other diseases of bladder and urethra
162	N35028	Other diseases of bladder and urethra
162	N358	Other diseases of bladder and urethra
162	N359	Other diseases of bladder and urethra
162	N360	Other diseases of bladder and urethra
162	N361	Other diseases of bladder and urethra
162	N362	Other diseases of bladder and urethra
162	N3641	Other diseases of bladder and urethra
162	N3642	Other diseases of bladder and urethra
162	N3643	Other diseases of bladder and urethra
162	N3644	Other diseases of bladder and urethra
162	N365	Other diseases of bladder and urethra
162	N368	Other diseases of bladder and urethra
163	N139	Genitourinary symptoms and ill-defined conditions
163	N369	Genitourinary symptoms and ill-defined conditions
163	N393	Genitourinary symptoms and ill-defined conditions
163	N3941	Genitourinary symptoms and ill-defined conditions
163	N3942	Genitourinary symptoms and ill-defined conditions
163	N3943	Genitourinary symptoms and ill-defined conditions
163	N3944	Genitourinary symptoms and ill-defined conditions
163	N3945	Genitourinary symptoms and ill-defined conditions
163	N3946	Genitourinary symptoms and ill-defined conditions
163	N39490	Genitourinary symptoms and ill-defined conditions
163	N39498	Genitourinary symptoms and ill-defined conditions
163	N398	Genitourinary symptoms and ill-defined conditions
163	N399	Genitourinary symptoms and ill-defined conditions
163	R300	Genitourinary symptoms and ill-defined conditions
163	R301	Genitourinary symptoms and ill-defined conditions
163	R309	Genitourinary symptoms and ill-defined conditions
163	R310	Genitourinary symptoms and ill-defined conditions
163	R311	Genitourinary symptoms and ill-defined conditions
163	R312	Genitourinary symptoms and ill-defined conditions
163	R319	Genitourinary symptoms and ill-defined conditions
163	R32	Genitourinary symptoms and ill-defined conditions
163	R330	Genitourinary symptoms and ill-defined conditions
163	R338	Genitourinary symptoms and ill-defined conditions
163	R339	Genitourinary symptoms and ill-defined conditions
163	R34	Genitourinary symptoms and ill-defined conditions
163	R350	Genitourinary symptoms and ill-defined conditions
163	R351	Genitourinary symptoms and ill-defined conditions
163	R358	Genitourinary symptoms and ill-defined conditions
163	R360	Genitourinary symptoms and ill-defined conditions
163	R369	Genitourinary symptoms and ill-defined conditions
163	R390	Genitourinary symptoms and ill-defined conditions
163	R3911	Genitourinary symptoms and ill-defined conditions
163	R3912	Genitourinary symptoms and ill-defined conditions
163	R3913	Genitourinary symptoms and ill-defined conditions
163	R3914	Genitourinary symptoms and ill-defined conditions
163	R3915	Genitourinary symptoms and ill-defined conditions
163	R3916	Genitourinary symptoms and ill-defined conditions

163	R3919	Genitourinary symptoms and ill-defined conditions
163	R392	Genitourinary symptoms and ill-defined conditions
163	R3981	Genitourinary symptoms and ill-defined conditions
163	R3989	Genitourinary symptoms and ill-defined conditions
163	R399	Genitourinary symptoms and ill-defined conditions
163	R800	Genitourinary symptoms and ill-defined conditions
163	R801	Genitourinary symptoms and ill-defined conditions
163	R803	Genitourinary symptoms and ill-defined conditions
163	R808	Genitourinary symptoms and ill-defined conditions
163	R809	Genitourinary symptoms and ill-defined conditions
163	R820	Genitourinary symptoms and ill-defined conditions
163	R821	Genitourinary symptoms and ill-defined conditions
163	R822	Genitourinary symptoms and ill-defined conditions
163	R823	Genitourinary symptoms and ill-defined conditions
163	R827	Genitourinary symptoms and ill-defined conditions
163	R828	Genitourinary symptoms and ill-defined conditions
163	R8290	Genitourinary symptoms and ill-defined conditions
163	R8291	Genitourinary symptoms and ill-defined conditions
163	R8299	Genitourinary symptoms and ill-defined conditions
163	Z435	Genitourinary symptoms and ill-defined conditions
163	Z436	Genitourinary symptoms and ill-defined conditions
163	Z466	Genitourinary symptoms and ill-defined conditions
163	Z87440	Genitourinary symptoms and ill-defined conditions
163	Z87448	Genitourinary symptoms and ill-defined conditions
163	Z9350	Genitourinary symptoms and ill-defined conditions
163	Z9351	Genitourinary symptoms and ill-defined conditions
163	Z9352	Genitourinary symptoms and ill-defined conditions
163	Z9359	Genitourinary symptoms and ill-defined conditions
163	Z936	Genitourinary symptoms and ill-defined conditions
163	Z960	Genitourinary symptoms and ill-defined conditions
164	N400	Hyperplasia of prostate
164	N401	Hyperplasia of prostate
164	N402	Hyperplasia of prostate
164	N403	Hyperplasia of prostate
165	N410	Inflammatory conditions of male genital organs
165	N411	Inflammatory conditions of male genital organs
165	N412	Inflammatory conditions of male genital organs
165	N413	Inflammatory conditions of male genital organs
165	N414	Inflammatory conditions of male genital organs
165	N418	Inflammatory conditions of male genital organs
165	N419	Inflammatory conditions of male genital organs
165	N431	Inflammatory conditions of male genital organs
165	N451	Inflammatory conditions of male genital organs
165	N452	Inflammatory conditions of male genital organs
165	N453	Inflammatory conditions of male genital organs
165	N454	Inflammatory conditions of male genital organs
165	N476	Inflammatory conditions of male genital organs
165	N481	Inflammatory conditions of male genital organs
165	N4821	Inflammatory conditions of male genital organs
165	N4822	Inflammatory conditions of male genital organs
165	N4829	Inflammatory conditions of male genital organs

165	N490	Inflammatory conditions of male genital organs
165	N491	Inflammatory conditions of male genital organs
165	N492	Inflammatory conditions of male genital organs
165	N493	Inflammatory conditions of male genital organs
165	N498	Inflammatory conditions of male genital organs
165	N499	Inflammatory conditions of male genital organs
166	N420	Other male genital disorders
166	N421	Other male genital disorders
166	N423	Other male genital disorders
166	N4281	Other male genital disorders
166	N4282	Other male genital disorders
166	N4283	Other male genital disorders
166	N4289	Other male genital disorders
166	N429	Other male genital disorders
166	N430	Other male genital disorders
166	N432	Other male genital disorders
166	N433	Other male genital disorders
166	N4340	Other male genital disorders
166	N4341	Other male genital disorders
166	N4342	Other male genital disorders
166	N4400	Other male genital disorders
166	N4401	Other male genital disorders
166	N4402	Other male genital disorders
166	N4403	Other male genital disorders
166	N4404	Other male genital disorders
166	N441	Other male genital disorders
166	N442	Other male genital disorders
166	N448	Other male genital disorders
166	N4601	Other male genital disorders
166	N46021	Other male genital disorders
166	N46022	Other male genital disorders
166	N46023	Other male genital disorders
166	N46024	Other male genital disorders
166	N46025	Other male genital disorders
166	N46029	Other male genital disorders
166	N4611	Other male genital disorders
166	N46121	Other male genital disorders
166	N46122	Other male genital disorders
166	N46123	Other male genital disorders
166	N46124	Other male genital disorders
166	N46125	Other male genital disorders
166	N46129	Other male genital disorders
166	N468	Other male genital disorders
166	N469	Other male genital disorders
166	N470	Other male genital disorders
166	N471	Other male genital disorders
166	N472	Other male genital disorders
166	N473	Other male genital disorders
166	N474	Other male genital disorders
166	N475	Other male genital disorders
166	N477	Other male genital disorders

166	N478	Other male genital disorders
166	N480	Other male genital disorders
166	N4830	Other male genital disorders
166	N4831	Other male genital disorders
166	N4832	Other male genital disorders
166	N4833	Other male genital disorders
166	N4839	Other male genital disorders
166	N485	Other male genital disorders
166	N486	Other male genital disorders
166	N4881	Other male genital disorders
166	N4882	Other male genital disorders
166	N4883	Other male genital disorders
166	N4889	Other male genital disorders
166	N489	Other male genital disorders
166	N500	Other male genital disorders
166	N501	Other male genital disorders
166	N503	Other male genital disorders
166	N508	Other male genital disorders
166	N509	Other male genital disorders
166	N51	Other male genital disorders
166	N5201	Other male genital disorders
166	N5202	Other male genital disorders
166	N5203	Other male genital disorders
166	N521	Other male genital disorders
166	N522	Other male genital disorders
166	N5231	Other male genital disorders
166	N5232	Other male genital disorders
166	N5233	Other male genital disorders
166	N5234	Other male genital disorders
166	N5239	Other male genital disorders
166	N528	Other male genital disorders
166	N529	Other male genital disorders
166	N5311	Other male genital disorders
166	N5312	Other male genital disorders
166	N5313	Other male genital disorders
166	N5314	Other male genital disorders
166	N5319	Other male genital disorders
166	N538	Other male genital disorders
166	N539	Other male genital disorders
166	R361	Other male genital disorders
166	Z87430	Other male genital disorders
166	Z87438	Other male genital disorders
167	N6001	Nonmalignant breast conditions
167	N6002	Nonmalignant breast conditions
167	N6009	Nonmalignant breast conditions
167	N6011	Nonmalignant breast conditions
167	N6012	Nonmalignant breast conditions
167	N6019	Nonmalignant breast conditions
167	N6021	Nonmalignant breast conditions
167	N6022	Nonmalignant breast conditions
167	N6029	Nonmalignant breast conditions

167	N6031	Nonmalignant breast conditions
167	N6032	Nonmalignant breast conditions
167	N6039	Nonmalignant breast conditions
167	N6041	Nonmalignant breast conditions
167	N6042	Nonmalignant breast conditions
167	N6049	Nonmalignant breast conditions
167	N6081	Nonmalignant breast conditions
167	N6082	Nonmalignant breast conditions
167	N6089	Nonmalignant breast conditions
167	N6091	Nonmalignant breast conditions
167	N6092	Nonmalignant breast conditions
167	N6099	Nonmalignant breast conditions
167	N61	Nonmalignant breast conditions
167	N62	Nonmalignant breast conditions
167	N63	Nonmalignant breast conditions
167	N640	Nonmalignant breast conditions
167	N641	Nonmalignant breast conditions
167	N642	Nonmalignant breast conditions
167	N643	Nonmalignant breast conditions
167	N644	Nonmalignant breast conditions
167	N6451	Nonmalignant breast conditions
167	N6452	Nonmalignant breast conditions
167	N6453	Nonmalignant breast conditions
167	N6459	Nonmalignant breast conditions
167	N6481	Nonmalignant breast conditions
167	N6482	Nonmalignant breast conditions
167	N6489	Nonmalignant breast conditions
167	N649	Nonmalignant breast conditions
167	N650	Nonmalignant breast conditions
167	N651	Nonmalignant breast conditions
167	R920	Nonmalignant breast conditions
167	R921	Nonmalignant breast conditions
168	N7001	Inflammatory diseases of female pelvic organs
168	N7002	Inflammatory diseases of female pelvic organs
168	N7003	Inflammatory diseases of female pelvic organs
168	N7011	Inflammatory diseases of female pelvic organs
168	N7012	Inflammatory diseases of female pelvic organs
168	N7013	Inflammatory diseases of female pelvic organs
168	N7091	Inflammatory diseases of female pelvic organs
168	N7092	Inflammatory diseases of female pelvic organs
168	N7093	Inflammatory diseases of female pelvic organs
168	N710	Inflammatory diseases of female pelvic organs
168	N711	Inflammatory diseases of female pelvic organs
168	N719	Inflammatory diseases of female pelvic organs
168	N72	Inflammatory diseases of female pelvic organs
168	N730	Inflammatory diseases of female pelvic organs
168	N731	Inflammatory diseases of female pelvic organs
168	N732	Inflammatory diseases of female pelvic organs
168	N733	Inflammatory diseases of female pelvic organs
168	N734	Inflammatory diseases of female pelvic organs
168	N735	Inflammatory diseases of female pelvic organs

168	N736	Inflammatory diseases of female pelvic organs
168	N738	Inflammatory diseases of female pelvic organs
168	N739	Inflammatory diseases of female pelvic organs
168	N74	Inflammatory diseases of female pelvic organs
168	N750	Inflammatory diseases of female pelvic organs
168	N751	Inflammatory diseases of female pelvic organs
168	N758	Inflammatory diseases of female pelvic organs
168	N759	Inflammatory diseases of female pelvic organs
168	N760	Inflammatory diseases of female pelvic organs
168	N761	Inflammatory diseases of female pelvic organs
168	N762	Inflammatory diseases of female pelvic organs
168	N763	Inflammatory diseases of female pelvic organs
168	N764	Inflammatory diseases of female pelvic organs
168	N765	Inflammatory diseases of female pelvic organs
168	N766	Inflammatory diseases of female pelvic organs
168	N7681	Inflammatory diseases of female pelvic organs
168	N7689	Inflammatory diseases of female pelvic organs
168	N770	Inflammatory diseases of female pelvic organs
168	N771	Inflammatory diseases of female pelvic organs
169	N800	Endometriosis
169	N801	Endometriosis
169	N802	Endometriosis
169	N803	Endometriosis
169	N804	Endometriosis
169	N805	Endometriosis
169	N806	Endometriosis
169	N808	Endometriosis
169	N809	Endometriosis
170	N810	Prolapse of female genital organs
170	N8110	Prolapse of female genital organs
170	N8111	Prolapse of female genital organs
170	N8112	Prolapse of female genital organs
170	N812	Prolapse of female genital organs
170	N813	Prolapse of female genital organs
170	N814	Prolapse of female genital organs
170	N815	Prolapse of female genital organs
170	N816	Prolapse of female genital organs
170	N8181	Prolapse of female genital organs
170	N8182	Prolapse of female genital organs
170	N8183	Prolapse of female genital organs
170	N8184	Prolapse of female genital organs
170	N8185	Prolapse of female genital organs
170	N8189	Prolapse of female genital organs
170	N819	Prolapse of female genital organs
171	N897	Menstrual disorders
171	N910	Menstrual disorders
171	N911	Menstrual disorders
171	N912	Menstrual disorders
171	N913	Menstrual disorders
171	N914	Menstrual disorders
171	N915	Menstrual disorders

171	N920	Menstrual disorders
171	N921	Menstrual disorders
171	N922	Menstrual disorders
171	N923	Menstrual disorders
171	N925	Menstrual disorders
171	N926	Menstrual disorders
171	N944	Menstrual disorders
171	N945	Menstrual disorders
171	N946	Menstrual disorders
172	N830	Ovarian cyst
172	N831	Ovarian cyst
172	N8320	Ovarian cyst
172	N8329	Ovarian cyst
173	E28310	Menopausal disorders
173	E28319	Menopausal disorders
173	E2839	Menopausal disorders
173	N924	Menopausal disorders
173	N950	Menopausal disorders
173	N951	Menopausal disorders
173	N952	Menopausal disorders
173	N958	Menopausal disorders
173	N959	Menopausal disorders
173	Z79890	Menopausal disorders
174	N970	Female infertility
174	N971	Female infertility
174	N972	Female infertility
174	N978	Female infertility
174	N979	Female infertility
175	N820	Other female genital disorders
175	N821	Other female genital disorders
175	N822	Other female genital disorders
175	N823	Other female genital disorders
175	N824	Other female genital disorders
175	N825	Other female genital disorders
175	N828	Other female genital disorders
175	N829	Other female genital disorders
175	N8331	Other female genital disorders
175	N8332	Other female genital disorders
175	N8333	Other female genital disorders
175	N834	Other female genital disorders
175	N8351	Other female genital disorders
175	N8352	Other female genital disorders
175	N8353	Other female genital disorders
175	N836	Other female genital disorders
175	N837	Other female genital disorders
175	N838	Other female genital disorders
175	N839	Other female genital disorders
175	N840	Other female genital disorders
175	N841	Other female genital disorders
175	N842	Other female genital disorders
175	N843	Other female genital disorders

175	N848	Other female genital disorders
175	N849	Other female genital disorders
175	N8500	Other female genital disorders
175	N8501	Other female genital disorders
175	N8502	Other female genital disorders
175	N852	Other female genital disorders
175	N853	Other female genital disorders
175	N854	Other female genital disorders
175	N855	Other female genital disorders
175	N856	Other female genital disorders
175	N857	Other female genital disorders
175	N858	Other female genital disorders
175	N859	Other female genital disorders
175	N86	Other female genital disorders
175	N870	Other female genital disorders
175	N871	Other female genital disorders
175	N879	Other female genital disorders
175	N880	Other female genital disorders
175	N881	Other female genital disorders
175	N882	Other female genital disorders
175	N883	Other female genital disorders
175	N884	Other female genital disorders
175	N888	Other female genital disorders
175	N889	Other female genital disorders
175	N890	Other female genital disorders
175	N891	Other female genital disorders
175	N893	Other female genital disorders
175	N894	Other female genital disorders
175	N895	Other female genital disorders
175	N896	Other female genital disorders
175	N898	Other female genital disorders
175	N899	Other female genital disorders
175	N900	Other female genital disorders
175	N901	Other female genital disorders
175	N903	Other female genital disorders
175	N904	Other female genital disorders
175	N905	Other female genital disorders
175	N906	Other female genital disorders
175	N907	Other female genital disorders
175	N90810	Other female genital disorders
175	N90811	Other female genital disorders
175	N90812	Other female genital disorders
175	N90813	Other female genital disorders
175	N90818	Other female genital disorders
175	N9089	Other female genital disorders
175	N909	Other female genital disorders
175	N930	Other female genital disorders
175	N938	Other female genital disorders
175	N939	Other female genital disorders
175	N940	Other female genital disorders
175	N941	Other female genital disorders

175	N942	Other female genital disorders
175	N943	Other female genital disorders
175	N94810	Other female genital disorders
175	N94818	Other female genital disorders
175	N94819	Other female genital disorders
175	N9489	Other female genital disorders
175	N949	Other female genital disorders
175	N96	Other female genital disorders
175	Z437	Other female genital disorders
175	Z87410	Other female genital disorders
175	Z87411	Other female genital disorders
175	Z87412	Other female genital disorders
175	Z8742	Other female genital disorders
175	Z8751	Other female genital disorders
176	Z30011	Contraceptive and procreative management
176	Z30012	Contraceptive and procreative management
176	Z30013	Contraceptive and procreative management
176	Z30014	Contraceptive and procreative management
176	Z30018	Contraceptive and procreative management
176	Z30019	Contraceptive and procreative management
176	Z3002	Contraceptive and procreative management
176	Z3009	Contraceptive and procreative management
176	Z302	Contraceptive and procreative management
176	Z3040	Contraceptive and procreative management
176	Z3041	Contraceptive and procreative management
176	Z3042	Contraceptive and procreative management
176	Z30430	Contraceptive and procreative management
176	Z30431	Contraceptive and procreative management
176	Z30432	Contraceptive and procreative management
176	Z30433	Contraceptive and procreative management
176	Z3049	Contraceptive and procreative management
176	Z308	Contraceptive and procreative management
176	Z309	Contraceptive and procreative management
176	Z310	Contraceptive and procreative management
176	Z3141	Contraceptive and procreative management
176	Z3142	Contraceptive and procreative management
176	Z31430	Contraceptive and procreative management
176	Z31438	Contraceptive and procreative management
176	Z31440	Contraceptive and procreative management
176	Z31441	Contraceptive and procreative management
176	Z31448	Contraceptive and procreative management
176	Z3149	Contraceptive and procreative management
176	Z315	Contraceptive and procreative management
176	Z3161	Contraceptive and procreative management
176	Z3162	Contraceptive and procreative management
176	Z3169	Contraceptive and procreative management
176	Z3181	Contraceptive and procreative management
176	Z3182	Contraceptive and procreative management
176	Z3183	Contraceptive and procreative management
176	Z3184	Contraceptive and procreative management
176	Z3189	Contraceptive and procreative management

176	Z319	Contraceptive and procreative management
176	Z920	Contraceptive and procreative management
176	Z975	Contraceptive and procreative management
176	Z9851	Contraceptive and procreative management
176	Z9852	Contraceptive and procreative management
177	O030	Spontaneous abortion
177	O031	Spontaneous abortion
177	O032	Spontaneous abortion
177	O0330	Spontaneous abortion
177	O0331	Spontaneous abortion
177	O0332	Spontaneous abortion
177	O0333	Spontaneous abortion
177	O0334	Spontaneous abortion
177	O0335	Spontaneous abortion
177	O0336	Spontaneous abortion
177	O0337	Spontaneous abortion
177	O0338	Spontaneous abortion
177	O0339	Spontaneous abortion
177	O034	Spontaneous abortion
177	O035	Spontaneous abortion
177	O036	Spontaneous abortion
177	O037	Spontaneous abortion
177	O0380	Spontaneous abortion
177	O0381	Spontaneous abortion
177	O0382	Spontaneous abortion
177	O0383	Spontaneous abortion
177	O0384	Spontaneous abortion
177	O0385	Spontaneous abortion
177	O0386	Spontaneous abortion
177	O0387	Spontaneous abortion
177	O0388	Spontaneous abortion
177	O0389	Spontaneous abortion
177	O039	Spontaneous abortion
178	O045	Induced abortion
178	O046	Induced abortion
178	O047	Induced abortion
178	O0480	Induced abortion
178	O0481	Induced abortion
178	O0482	Induced abortion
178	O0483	Induced abortion
178	O0484	Induced abortion
178	O0485	Induced abortion
178	O0486	Induced abortion
178	O0487	Induced abortion
178	O0488	Induced abortion
178	O0489	Induced abortion
178	O070	Induced abortion
178	O071	Induced abortion
178	O072	Induced abortion
178	O0730	Induced abortion
178	O0731	Induced abortion

178	O0732	Induced abortion
178	O0733	Induced abortion
178	O0734	Induced abortion
178	O0735	Induced abortion
178	O0736	Induced abortion
178	O0737	Induced abortion
178	O0738	Induced abortion
178	O0739	Induced abortion
178	O074	Induced abortion
178	Z332	Induced abortion
179	O080	Postabortion complications
179	O081	Postabortion complications
179	O082	Postabortion complications
179	O083	Postabortion complications
179	O084	Postabortion complications
179	O085	Postabortion complications
179	O086	Postabortion complications
179	O087	Postabortion complications
179	O0881	Postabortion complications
179	O0882	Postabortion complications
179	O0883	Postabortion complications
179	O0889	Postabortion complications
179	O089	Postabortion complications
180	O000	Ectopic pregnancy
180	O001	Ectopic pregnancy
180	O002	Ectopic pregnancy
180	O008	Ectopic pregnancy
180	O009	Ectopic pregnancy
181	O010	Other complications of pregnancy
181	O011	Other complications of pregnancy
181	O019	Other complications of pregnancy
181	O020	Other complications of pregnancy
181	O021	Other complications of pregnancy
181	O0281	Other complications of pregnancy
181	O0289	Other complications of pregnancy
181	O029	Other complications of pregnancy
181	O0900	Other complications of pregnancy
181	O0901	Other complications of pregnancy
181	O0902	Other complications of pregnancy
181	O0903	Other complications of pregnancy
181	O0910	Other complications of pregnancy
181	O0911	Other complications of pregnancy
181	O0912	Other complications of pregnancy
181	O0913	Other complications of pregnancy
181	O09211	Other complications of pregnancy
181	O09212	Other complications of pregnancy
181	O09213	Other complications of pregnancy
181	O09219	Other complications of pregnancy
181	O09291	Other complications of pregnancy
181	O09292	Other complications of pregnancy
181	O09293	Other complications of pregnancy

181	O09299	Other complications of pregnancy
181	O0930	Other complications of pregnancy
181	O0931	Other complications of pregnancy
181	O0932	Other complications of pregnancy
181	O0933	Other complications of pregnancy
181	O0940	Other complications of pregnancy
181	O0941	Other complications of pregnancy
181	O0942	Other complications of pregnancy
181	O0943	Other complications of pregnancy
181	O09511	Other complications of pregnancy
181	O09512	Other complications of pregnancy
181	O09513	Other complications of pregnancy
181	O09519	Other complications of pregnancy
181	O09521	Other complications of pregnancy
181	O09522	Other complications of pregnancy
181	O09523	Other complications of pregnancy
181	O09529	Other complications of pregnancy
181	O09611	Other complications of pregnancy
181	O09612	Other complications of pregnancy
181	O09613	Other complications of pregnancy
181	O09619	Other complications of pregnancy
181	O09621	Other complications of pregnancy
181	O09622	Other complications of pregnancy
181	O09623	Other complications of pregnancy
181	O09629	Other complications of pregnancy
181	O0970	Other complications of pregnancy
181	O0971	Other complications of pregnancy
181	O0972	Other complications of pregnancy
181	O0973	Other complications of pregnancy
181	O09811	Other complications of pregnancy
181	O09812	Other complications of pregnancy
181	O09813	Other complications of pregnancy
181	O09819	Other complications of pregnancy
181	O09821	Other complications of pregnancy
181	O09822	Other complications of pregnancy
181	O09823	Other complications of pregnancy
181	O09829	Other complications of pregnancy
181	O09891	Other complications of pregnancy
181	O09892	Other complications of pregnancy
181	O09893	Other complications of pregnancy
181	O09899	Other complications of pregnancy
181	O0990	Other complications of pregnancy
181	O0991	Other complications of pregnancy
181	O0992	Other complications of pregnancy
181	O0993	Other complications of pregnancy
181	O1200	Other complications of pregnancy
181	O1201	Other complications of pregnancy
181	O1202	Other complications of pregnancy
181	O1203	Other complications of pregnancy
181	O1210	Other complications of pregnancy
181	O1211	Other complications of pregnancy

181	O1212	Other complications of pregnancy
181	O1213	Other complications of pregnancy
181	O1220	Other complications of pregnancy
181	O1221	Other complications of pregnancy
181	O1222	Other complications of pregnancy
181	O1223	Other complications of pregnancy
181	O210	Other complications of pregnancy
181	O211	Other complications of pregnancy
181	O212	Other complications of pregnancy
181	O218	Other complications of pregnancy
181	O219	Other complications of pregnancy
181	O2200	Other complications of pregnancy
181	O2201	Other complications of pregnancy
181	O2202	Other complications of pregnancy
181	O2203	Other complications of pregnancy
181	O2210	Other complications of pregnancy
181	O2211	Other complications of pregnancy
181	O2212	Other complications of pregnancy
181	O2213	Other complications of pregnancy
181	O2220	Other complications of pregnancy
181	O2221	Other complications of pregnancy
181	O2222	Other complications of pregnancy
181	O2223	Other complications of pregnancy
181	O2230	Other complications of pregnancy
181	O2231	Other complications of pregnancy
181	O2232	Other complications of pregnancy
181	O2233	Other complications of pregnancy
181	O2240	Other complications of pregnancy
181	O2241	Other complications of pregnancy
181	O2242	Other complications of pregnancy
181	O2243	Other complications of pregnancy
181	O2250	Other complications of pregnancy
181	O2251	Other complications of pregnancy
181	O2252	Other complications of pregnancy
181	O2253	Other complications of pregnancy
181	O228X1	Other complications of pregnancy
181	O228X2	Other complications of pregnancy
181	O228X3	Other complications of pregnancy
181	O228X9	Other complications of pregnancy
181	O2290	Other complications of pregnancy
181	O2291	Other complications of pregnancy
181	O2292	Other complications of pregnancy
181	O2293	Other complications of pregnancy
181	O2300	Other complications of pregnancy
181	O2301	Other complications of pregnancy
181	O2302	Other complications of pregnancy
181	O2303	Other complications of pregnancy
181	O2310	Other complications of pregnancy
181	O2311	Other complications of pregnancy
181	O2312	Other complications of pregnancy
181	O2313	Other complications of pregnancy

181	O2320	Other complications of pregnancy
181	O2321	Other complications of pregnancy
181	O2322	Other complications of pregnancy
181	O2323	Other complications of pregnancy
181	O2330	Other complications of pregnancy
181	O2331	Other complications of pregnancy
181	O2332	Other complications of pregnancy
181	O2333	Other complications of pregnancy
181	O2340	Other complications of pregnancy
181	O2341	Other complications of pregnancy
181	O2342	Other complications of pregnancy
181	O2343	Other complications of pregnancy
181	O23511	Other complications of pregnancy
181	O23512	Other complications of pregnancy
181	O23513	Other complications of pregnancy
181	O23519	Other complications of pregnancy
181	O23521	Other complications of pregnancy
181	O23522	Other complications of pregnancy
181	O23523	Other complications of pregnancy
181	O23529	Other complications of pregnancy
181	O23591	Other complications of pregnancy
181	O23592	Other complications of pregnancy
181	O23593	Other complications of pregnancy
181	O23599	Other complications of pregnancy
181	O2390	Other complications of pregnancy
181	O2391	Other complications of pregnancy
181	O2392	Other complications of pregnancy
181	O2393	Other complications of pregnancy
181	O2510	Other complications of pregnancy
181	O2511	Other complications of pregnancy
181	O2512	Other complications of pregnancy
181	O2513	Other complications of pregnancy
181	O2600	Other complications of pregnancy
181	O2601	Other complications of pregnancy
181	O2602	Other complications of pregnancy
181	O2603	Other complications of pregnancy
181	O2610	Other complications of pregnancy
181	O2611	Other complications of pregnancy
181	O2612	Other complications of pregnancy
181	O2613	Other complications of pregnancy
181	O2620	Other complications of pregnancy
181	O2621	Other complications of pregnancy
181	O2622	Other complications of pregnancy
181	O2623	Other complications of pregnancy
181	O2630	Other complications of pregnancy
181	O2631	Other complications of pregnancy
181	O2632	Other complications of pregnancy
181	O2633	Other complications of pregnancy
181	O2640	Other complications of pregnancy
181	O2641	Other complications of pregnancy
181	O2642	Other complications of pregnancy

181	O2643	Other complications of pregnancy
181	O2650	Other complications of pregnancy
181	O2651	Other complications of pregnancy
181	O2652	Other complications of pregnancy
181	O2653	Other complications of pregnancy
181	O26611	Other complications of pregnancy
181	O26612	Other complications of pregnancy
181	O26613	Other complications of pregnancy
181	O26619	Other complications of pregnancy
181	O26711	Other complications of pregnancy
181	O26712	Other complications of pregnancy
181	O26713	Other complications of pregnancy
181	O26719	Other complications of pregnancy
181	O26811	Other complications of pregnancy
181	O26812	Other complications of pregnancy
181	O26813	Other complications of pregnancy
181	O26819	Other complications of pregnancy
181	O26821	Other complications of pregnancy
181	O26822	Other complications of pregnancy
181	O26823	Other complications of pregnancy
181	O26829	Other complications of pregnancy
181	O26831	Other complications of pregnancy
181	O26832	Other complications of pregnancy
181	O26833	Other complications of pregnancy
181	O26839	Other complications of pregnancy
181	O26841	Other complications of pregnancy
181	O26842	Other complications of pregnancy
181	O26843	Other complications of pregnancy
181	O26849	Other complications of pregnancy
181	O26851	Other complications of pregnancy
181	O26852	Other complications of pregnancy
181	O26853	Other complications of pregnancy
181	O26859	Other complications of pregnancy
181	O2686	Other complications of pregnancy
181	O26872	Other complications of pregnancy
181	O26873	Other complications of pregnancy
181	O26879	Other complications of pregnancy
181	O26891	Other complications of pregnancy
181	O26892	Other complications of pregnancy
181	O26893	Other complications of pregnancy
181	O26899	Other complications of pregnancy
181	O2690	Other complications of pregnancy
181	O2691	Other complications of pregnancy
181	O2692	Other complications of pregnancy
181	O2693	Other complications of pregnancy
181	O280	Other complications of pregnancy
181	O281	Other complications of pregnancy
181	O282	Other complications of pregnancy
181	O283	Other complications of pregnancy
181	O284	Other complications of pregnancy
181	O285	Other complications of pregnancy

181	O288	Other complications of pregnancy
181	O289	Other complications of pregnancy
181	O29011	Other complications of pregnancy
181	O29012	Other complications of pregnancy
181	O29013	Other complications of pregnancy
181	O29019	Other complications of pregnancy
181	O29021	Other complications of pregnancy
181	O29022	Other complications of pregnancy
181	O29023	Other complications of pregnancy
181	O29029	Other complications of pregnancy
181	O29091	Other complications of pregnancy
181	O29092	Other complications of pregnancy
181	O29093	Other complications of pregnancy
181	O29099	Other complications of pregnancy
181	O29111	Other complications of pregnancy
181	O29112	Other complications of pregnancy
181	O29113	Other complications of pregnancy
181	O29119	Other complications of pregnancy
181	O29121	Other complications of pregnancy
181	O29122	Other complications of pregnancy
181	O29123	Other complications of pregnancy
181	O29129	Other complications of pregnancy
181	O29191	Other complications of pregnancy
181	O29192	Other complications of pregnancy
181	O29193	Other complications of pregnancy
181	O29199	Other complications of pregnancy
181	O29211	Other complications of pregnancy
181	O29212	Other complications of pregnancy
181	O29213	Other complications of pregnancy
181	O29219	Other complications of pregnancy
181	O29291	Other complications of pregnancy
181	O29292	Other complications of pregnancy
181	O29293	Other complications of pregnancy
181	O29299	Other complications of pregnancy
181	O293X1	Other complications of pregnancy
181	O293X2	Other complications of pregnancy
181	O293X3	Other complications of pregnancy
181	O293X9	Other complications of pregnancy
181	O2940	Other complications of pregnancy
181	O2941	Other complications of pregnancy
181	O2942	Other complications of pregnancy
181	O2943	Other complications of pregnancy
181	O295X1	Other complications of pregnancy
181	O295X2	Other complications of pregnancy
181	O295X3	Other complications of pregnancy
181	O295X9	Other complications of pregnancy
181	O2960	Other complications of pregnancy
181	O2961	Other complications of pregnancy
181	O2962	Other complications of pregnancy
181	O2963	Other complications of pregnancy
181	O298X1	Other complications of pregnancy

181	O298X2	Other complications of pregnancy
181	O298X3	Other complications of pregnancy
181	O298X9	Other complications of pregnancy
181	O2990	Other complications of pregnancy
181	O2991	Other complications of pregnancy
181	O2992	Other complications of pregnancy
181	O2993	Other complications of pregnancy
181	O3100X0	Other complications of pregnancy
181	O3100X1	Other complications of pregnancy
181	O3100X2	Other complications of pregnancy
181	O3100X3	Other complications of pregnancy
181	O3100X4	Other complications of pregnancy
181	O3100X5	Other complications of pregnancy
181	O3100X9	Other complications of pregnancy
181	O3101X0	Other complications of pregnancy
181	O3101X1	Other complications of pregnancy
181	O3101X2	Other complications of pregnancy
181	O3101X3	Other complications of pregnancy
181	O3101X4	Other complications of pregnancy
181	O3101X5	Other complications of pregnancy
181	O3101X9	Other complications of pregnancy
181	O3102X0	Other complications of pregnancy
181	O3102X1	Other complications of pregnancy
181	O3102X2	Other complications of pregnancy
181	O3102X3	Other complications of pregnancy
181	O3102X4	Other complications of pregnancy
181	O3102X5	Other complications of pregnancy
181	O3102X9	Other complications of pregnancy
181	O3103X0	Other complications of pregnancy
181	O3103X1	Other complications of pregnancy
181	O3103X2	Other complications of pregnancy
181	O3103X3	Other complications of pregnancy
181	O3103X4	Other complications of pregnancy
181	O3103X5	Other complications of pregnancy
181	O3103X9	Other complications of pregnancy
181	O3110X0	Other complications of pregnancy
181	O3110X1	Other complications of pregnancy
181	O3110X2	Other complications of pregnancy
181	O3110X3	Other complications of pregnancy
181	O3110X4	Other complications of pregnancy
181	O3110X5	Other complications of pregnancy
181	O3110X9	Other complications of pregnancy
181	O3111X0	Other complications of pregnancy
181	O3111X1	Other complications of pregnancy
181	O3111X2	Other complications of pregnancy
181	O3111X3	Other complications of pregnancy
181	O3111X4	Other complications of pregnancy
181	O3111X5	Other complications of pregnancy
181	O3111X9	Other complications of pregnancy
181	O3112X0	Other complications of pregnancy
181	O3112X1	Other complications of pregnancy

181	O3112X2	Other complications of pregnancy
181	O3112X3	Other complications of pregnancy
181	O3112X4	Other complications of pregnancy
181	O3112X5	Other complications of pregnancy
181	O3112X9	Other complications of pregnancy
181	O3113X0	Other complications of pregnancy
181	O3113X1	Other complications of pregnancy
181	O3113X2	Other complications of pregnancy
181	O3113X3	Other complications of pregnancy
181	O3113X4	Other complications of pregnancy
181	O3113X5	Other complications of pregnancy
181	O3113X9	Other complications of pregnancy
181	O3120X0	Other complications of pregnancy
181	O3120X1	Other complications of pregnancy
181	O3120X2	Other complications of pregnancy
181	O3120X3	Other complications of pregnancy
181	O3120X4	Other complications of pregnancy
181	O3120X5	Other complications of pregnancy
181	O3120X9	Other complications of pregnancy
181	O3121X0	Other complications of pregnancy
181	O3121X1	Other complications of pregnancy
181	O3121X2	Other complications of pregnancy
181	O3121X3	Other complications of pregnancy
181	O3121X4	Other complications of pregnancy
181	O3121X5	Other complications of pregnancy
181	O3121X9	Other complications of pregnancy
181	O3122X0	Other complications of pregnancy
181	O3122X1	Other complications of pregnancy
181	O3122X2	Other complications of pregnancy
181	O3122X3	Other complications of pregnancy
181	O3122X4	Other complications of pregnancy
181	O3122X5	Other complications of pregnancy
181	O3122X9	Other complications of pregnancy
181	O3123X0	Other complications of pregnancy
181	O3123X1	Other complications of pregnancy
181	O3123X2	Other complications of pregnancy
181	O3123X3	Other complications of pregnancy
181	O3123X4	Other complications of pregnancy
181	O3123X5	Other complications of pregnancy
181	O3123X9	Other complications of pregnancy
181	O318X10	Other complications of pregnancy
181	O318X11	Other complications of pregnancy
181	O318X12	Other complications of pregnancy
181	O318X13	Other complications of pregnancy
181	O318X14	Other complications of pregnancy
181	O318X15	Other complications of pregnancy
181	O318X19	Other complications of pregnancy
181	O318X20	Other complications of pregnancy
181	O318X21	Other complications of pregnancy
181	O318X22	Other complications of pregnancy
181	O318X23	Other complications of pregnancy

181	O318X24	Other complications of pregnancy
181	O318X25	Other complications of pregnancy
181	O318X29	Other complications of pregnancy
181	O318X30	Other complications of pregnancy
181	O318X31	Other complications of pregnancy
181	O318X32	Other complications of pregnancy
181	O318X33	Other complications of pregnancy
181	O318X34	Other complications of pregnancy
181	O318X35	Other complications of pregnancy
181	O318X39	Other complications of pregnancy
181	O318X90	Other complications of pregnancy
181	O318X91	Other complications of pregnancy
181	O318X92	Other complications of pregnancy
181	O318X93	Other complications of pregnancy
181	O318X94	Other complications of pregnancy
181	O318X95	Other complications of pregnancy
181	O318X99	Other complications of pregnancy
181	O3400	Other complications of pregnancy
181	O3401	Other complications of pregnancy
181	O3402	Other complications of pregnancy
181	O3403	Other complications of pregnancy
181	O3410	Other complications of pregnancy
181	O3411	Other complications of pregnancy
181	O3412	Other complications of pregnancy
181	O3413	Other complications of pregnancy
181	O3429	Other complications of pregnancy
181	O3430	Other complications of pregnancy
181	O3431	Other complications of pregnancy
181	O3432	Other complications of pregnancy
181	O3433	Other complications of pregnancy
181	O3440	Other complications of pregnancy
181	O3441	Other complications of pregnancy
181	O3442	Other complications of pregnancy
181	O3443	Other complications of pregnancy
181	O34511	Other complications of pregnancy
181	O34512	Other complications of pregnancy
181	O34513	Other complications of pregnancy
181	O34519	Other complications of pregnancy
181	O34521	Other complications of pregnancy
181	O34522	Other complications of pregnancy
181	O34523	Other complications of pregnancy
181	O34529	Other complications of pregnancy
181	O34531	Other complications of pregnancy
181	O34532	Other complications of pregnancy
181	O34533	Other complications of pregnancy
181	O34539	Other complications of pregnancy
181	O34591	Other complications of pregnancy
181	O34592	Other complications of pregnancy
181	O34593	Other complications of pregnancy
181	O34599	Other complications of pregnancy
181	O3460	Other complications of pregnancy

181	O3461	Other complications of pregnancy
181	O3462	Other complications of pregnancy
181	O3463	Other complications of pregnancy
181	O3470	Other complications of pregnancy
181	O3471	Other complications of pregnancy
181	O3472	Other complications of pregnancy
181	O3473	Other complications of pregnancy
181	O3480	Other complications of pregnancy
181	O3481	Other complications of pregnancy
181	O3482	Other complications of pregnancy
181	O3483	Other complications of pregnancy
181	O3490	Other complications of pregnancy
181	O3491	Other complications of pregnancy
181	O3492	Other complications of pregnancy
181	O3493	Other complications of pregnancy
181	O350XX0	Other complications of pregnancy
181	O350XX1	Other complications of pregnancy
181	O350XX2	Other complications of pregnancy
181	O350XX3	Other complications of pregnancy
181	O350XX4	Other complications of pregnancy
181	O350XX5	Other complications of pregnancy
181	O350XX9	Other complications of pregnancy
181	O351XX0	Other complications of pregnancy
181	O351XX1	Other complications of pregnancy
181	O351XX2	Other complications of pregnancy
181	O351XX3	Other complications of pregnancy
181	O351XX4	Other complications of pregnancy
181	O351XX5	Other complications of pregnancy
181	O351XX9	Other complications of pregnancy
181	O352XX0	Other complications of pregnancy
181	O352XX1	Other complications of pregnancy
181	O352XX2	Other complications of pregnancy
181	O352XX3	Other complications of pregnancy
181	O352XX4	Other complications of pregnancy
181	O352XX5	Other complications of pregnancy
181	O352XX9	Other complications of pregnancy
181	O353XX0	Other complications of pregnancy
181	O353XX1	Other complications of pregnancy
181	O353XX2	Other complications of pregnancy
181	O353XX3	Other complications of pregnancy
181	O353XX4	Other complications of pregnancy
181	O353XX5	Other complications of pregnancy
181	O353XX9	Other complications of pregnancy
181	O360110	Other complications of pregnancy
181	O360111	Other complications of pregnancy
181	O360112	Other complications of pregnancy
181	O360113	Other complications of pregnancy
181	O360114	Other complications of pregnancy
181	O360115	Other complications of pregnancy
181	O360119	Other complications of pregnancy
181	O360120	Other complications of pregnancy

181	O360121	Other complications of pregnancy
181	O360122	Other complications of pregnancy
181	O360123	Other complications of pregnancy
181	O360124	Other complications of pregnancy
181	O360125	Other complications of pregnancy
181	O360129	Other complications of pregnancy
181	O360130	Other complications of pregnancy
181	O360131	Other complications of pregnancy
181	O360132	Other complications of pregnancy
181	O360133	Other complications of pregnancy
181	O360134	Other complications of pregnancy
181	O360135	Other complications of pregnancy
181	O360139	Other complications of pregnancy
181	O360190	Other complications of pregnancy
181	O360191	Other complications of pregnancy
181	O360192	Other complications of pregnancy
181	O360193	Other complications of pregnancy
181	O360194	Other complications of pregnancy
181	O360195	Other complications of pregnancy
181	O360199	Other complications of pregnancy
181	O360910	Other complications of pregnancy
181	O360911	Other complications of pregnancy
181	O360912	Other complications of pregnancy
181	O360913	Other complications of pregnancy
181	O360914	Other complications of pregnancy
181	O360915	Other complications of pregnancy
181	O360919	Other complications of pregnancy
181	O360920	Other complications of pregnancy
181	O360921	Other complications of pregnancy
181	O360922	Other complications of pregnancy
181	O360923	Other complications of pregnancy
181	O360924	Other complications of pregnancy
181	O360925	Other complications of pregnancy
181	O360929	Other complications of pregnancy
181	O360930	Other complications of pregnancy
181	O360931	Other complications of pregnancy
181	O360932	Other complications of pregnancy
181	O360933	Other complications of pregnancy
181	O360934	Other complications of pregnancy
181	O360935	Other complications of pregnancy
181	O360939	Other complications of pregnancy
181	O360990	Other complications of pregnancy
181	O360991	Other complications of pregnancy
181	O360992	Other complications of pregnancy
181	O360993	Other complications of pregnancy
181	O360994	Other complications of pregnancy
181	O360995	Other complications of pregnancy
181	O360999	Other complications of pregnancy
181	O361110	Other complications of pregnancy
181	O361111	Other complications of pregnancy
181	O361112	Other complications of pregnancy

181	O361113	Other complications of pregnancy
181	O361114	Other complications of pregnancy
181	O361115	Other complications of pregnancy
181	O361119	Other complications of pregnancy
181	O361120	Other complications of pregnancy
181	O361121	Other complications of pregnancy
181	O361122	Other complications of pregnancy
181	O361123	Other complications of pregnancy
181	O361124	Other complications of pregnancy
181	O361125	Other complications of pregnancy
181	O361129	Other complications of pregnancy
181	O361130	Other complications of pregnancy
181	O361131	Other complications of pregnancy
181	O361132	Other complications of pregnancy
181	O361133	Other complications of pregnancy
181	O361134	Other complications of pregnancy
181	O361135	Other complications of pregnancy
181	O361139	Other complications of pregnancy
181	O361190	Other complications of pregnancy
181	O361191	Other complications of pregnancy
181	O361192	Other complications of pregnancy
181	O361193	Other complications of pregnancy
181	O361194	Other complications of pregnancy
181	O361195	Other complications of pregnancy
181	O361199	Other complications of pregnancy
181	O361910	Other complications of pregnancy
181	O361911	Other complications of pregnancy
181	O361912	Other complications of pregnancy
181	O361913	Other complications of pregnancy
181	O361914	Other complications of pregnancy
181	O361915	Other complications of pregnancy
181	O361919	Other complications of pregnancy
181	O361920	Other complications of pregnancy
181	O361921	Other complications of pregnancy
181	O361922	Other complications of pregnancy
181	O361923	Other complications of pregnancy
181	O361924	Other complications of pregnancy
181	O361925	Other complications of pregnancy
181	O361929	Other complications of pregnancy
181	O361930	Other complications of pregnancy
181	O361931	Other complications of pregnancy
181	O361932	Other complications of pregnancy
181	O361933	Other complications of pregnancy
181	O361934	Other complications of pregnancy
181	O361935	Other complications of pregnancy
181	O361939	Other complications of pregnancy
181	O361990	Other complications of pregnancy
181	O361991	Other complications of pregnancy
181	O361992	Other complications of pregnancy
181	O361993	Other complications of pregnancy
181	O361994	Other complications of pregnancy

181	O361995	Other complications of pregnancy
181	O361999	Other complications of pregnancy
181	O3620X0	Other complications of pregnancy
181	O3620X1	Other complications of pregnancy
181	O3620X2	Other complications of pregnancy
181	O3620X3	Other complications of pregnancy
181	O3620X4	Other complications of pregnancy
181	O3620X5	Other complications of pregnancy
181	O3620X9	Other complications of pregnancy
181	O3621X0	Other complications of pregnancy
181	O3621X1	Other complications of pregnancy
181	O3621X2	Other complications of pregnancy
181	O3621X3	Other complications of pregnancy
181	O3621X4	Other complications of pregnancy
181	O3621X5	Other complications of pregnancy
181	O3621X9	Other complications of pregnancy
181	O3622X0	Other complications of pregnancy
181	O3622X1	Other complications of pregnancy
181	O3622X2	Other complications of pregnancy
181	O3622X3	Other complications of pregnancy
181	O3622X4	Other complications of pregnancy
181	O3622X5	Other complications of pregnancy
181	O3622X9	Other complications of pregnancy
181	O3623X0	Other complications of pregnancy
181	O3623X1	Other complications of pregnancy
181	O3623X2	Other complications of pregnancy
181	O3623X3	Other complications of pregnancy
181	O3623X4	Other complications of pregnancy
181	O3623X5	Other complications of pregnancy
181	O3623X9	Other complications of pregnancy
181	O365110	Other complications of pregnancy
181	O365111	Other complications of pregnancy
181	O365112	Other complications of pregnancy
181	O365113	Other complications of pregnancy
181	O365114	Other complications of pregnancy
181	O365115	Other complications of pregnancy
181	O365119	Other complications of pregnancy
181	O365120	Other complications of pregnancy
181	O365121	Other complications of pregnancy
181	O365122	Other complications of pregnancy
181	O365123	Other complications of pregnancy
181	O365124	Other complications of pregnancy
181	O365125	Other complications of pregnancy
181	O365129	Other complications of pregnancy
181	O365130	Other complications of pregnancy
181	O365131	Other complications of pregnancy
181	O365132	Other complications of pregnancy
181	O365133	Other complications of pregnancy
181	O365134	Other complications of pregnancy
181	O365135	Other complications of pregnancy
181	O365139	Other complications of pregnancy

181	O365190	Other complications of pregnancy
181	O365191	Other complications of pregnancy
181	O365192	Other complications of pregnancy
181	O365193	Other complications of pregnancy
181	O365194	Other complications of pregnancy
181	O365195	Other complications of pregnancy
181	O365199	Other complications of pregnancy
181	O365910	Other complications of pregnancy
181	O365911	Other complications of pregnancy
181	O365912	Other complications of pregnancy
181	O365913	Other complications of pregnancy
181	O365914	Other complications of pregnancy
181	O365915	Other complications of pregnancy
181	O365919	Other complications of pregnancy
181	O365920	Other complications of pregnancy
181	O365921	Other complications of pregnancy
181	O365922	Other complications of pregnancy
181	O365923	Other complications of pregnancy
181	O365924	Other complications of pregnancy
181	O365925	Other complications of pregnancy
181	O365929	Other complications of pregnancy
181	O365930	Other complications of pregnancy
181	O365931	Other complications of pregnancy
181	O365932	Other complications of pregnancy
181	O365933	Other complications of pregnancy
181	O365934	Other complications of pregnancy
181	O365935	Other complications of pregnancy
181	O365939	Other complications of pregnancy
181	O365990	Other complications of pregnancy
181	O365991	Other complications of pregnancy
181	O365992	Other complications of pregnancy
181	O365993	Other complications of pregnancy
181	O365994	Other complications of pregnancy
181	O365995	Other complications of pregnancy
181	O365999	Other complications of pregnancy
181	O3660X0	Other complications of pregnancy
181	O3660X1	Other complications of pregnancy
181	O3660X2	Other complications of pregnancy
181	O3660X3	Other complications of pregnancy
181	O3660X4	Other complications of pregnancy
181	O3660X5	Other complications of pregnancy
181	O3660X9	Other complications of pregnancy
181	O3661X0	Other complications of pregnancy
181	O3661X1	Other complications of pregnancy
181	O3661X2	Other complications of pregnancy
181	O3661X3	Other complications of pregnancy
181	O3661X4	Other complications of pregnancy
181	O3661X5	Other complications of pregnancy
181	O3661X9	Other complications of pregnancy
181	O3662X0	Other complications of pregnancy
181	O3662X1	Other complications of pregnancy

181	O3662X2	Other complications of pregnancy
181	O3662X3	Other complications of pregnancy
181	O3662X4	Other complications of pregnancy
181	O3662X5	Other complications of pregnancy
181	O3662X9	Other complications of pregnancy
181	O3663X0	Other complications of pregnancy
181	O3663X1	Other complications of pregnancy
181	O3663X2	Other complications of pregnancy
181	O3663X3	Other complications of pregnancy
181	O3663X4	Other complications of pregnancy
181	O3663X5	Other complications of pregnancy
181	O3663X9	Other complications of pregnancy
181	O3670X0	Other complications of pregnancy
181	O3670X1	Other complications of pregnancy
181	O3670X2	Other complications of pregnancy
181	O3670X3	Other complications of pregnancy
181	O3670X4	Other complications of pregnancy
181	O3670X5	Other complications of pregnancy
181	O3670X9	Other complications of pregnancy
181	O3671X0	Other complications of pregnancy
181	O3671X1	Other complications of pregnancy
181	O3671X2	Other complications of pregnancy
181	O3671X3	Other complications of pregnancy
181	O3671X4	Other complications of pregnancy
181	O3671X5	Other complications of pregnancy
181	O3671X9	Other complications of pregnancy
181	O3672X0	Other complications of pregnancy
181	O3672X1	Other complications of pregnancy
181	O3672X2	Other complications of pregnancy
181	O3672X3	Other complications of pregnancy
181	O3672X4	Other complications of pregnancy
181	O3672X5	Other complications of pregnancy
181	O3672X9	Other complications of pregnancy
181	O3673X0	Other complications of pregnancy
181	O3673X1	Other complications of pregnancy
181	O3673X2	Other complications of pregnancy
181	O3673X3	Other complications of pregnancy
181	O3673X4	Other complications of pregnancy
181	O3673X5	Other complications of pregnancy
181	O3673X9	Other complications of pregnancy
181	O3680X0	Other complications of pregnancy
181	O3680X1	Other complications of pregnancy
181	O3680X2	Other complications of pregnancy
181	O3680X3	Other complications of pregnancy
181	O3680X4	Other complications of pregnancy
181	O3680X5	Other complications of pregnancy
181	O3680X9	Other complications of pregnancy
181	O368120	Other complications of pregnancy
181	O368121	Other complications of pregnancy
181	O368122	Other complications of pregnancy
181	O368123	Other complications of pregnancy

181	O368124	Other complications of pregnancy
181	O368125	Other complications of pregnancy
181	O368129	Other complications of pregnancy
181	O368130	Other complications of pregnancy
181	O368131	Other complications of pregnancy
181	O368132	Other complications of pregnancy
181	O368133	Other complications of pregnancy
181	O368134	Other complications of pregnancy
181	O368135	Other complications of pregnancy
181	O368139	Other complications of pregnancy
181	O368190	Other complications of pregnancy
181	O368191	Other complications of pregnancy
181	O368192	Other complications of pregnancy
181	O368193	Other complications of pregnancy
181	O368194	Other complications of pregnancy
181	O368195	Other complications of pregnancy
181	O368199	Other complications of pregnancy
181	O368210	Other complications of pregnancy
181	O368211	Other complications of pregnancy
181	O368212	Other complications of pregnancy
181	O368213	Other complications of pregnancy
181	O368214	Other complications of pregnancy
181	O368215	Other complications of pregnancy
181	O368219	Other complications of pregnancy
181	O368220	Other complications of pregnancy
181	O368221	Other complications of pregnancy
181	O368222	Other complications of pregnancy
181	O368223	Other complications of pregnancy
181	O368224	Other complications of pregnancy
181	O368225	Other complications of pregnancy
181	O368229	Other complications of pregnancy
181	O368230	Other complications of pregnancy
181	O368231	Other complications of pregnancy
181	O368232	Other complications of pregnancy
181	O368233	Other complications of pregnancy
181	O368234	Other complications of pregnancy
181	O368235	Other complications of pregnancy
181	O368239	Other complications of pregnancy
181	O368290	Other complications of pregnancy
181	O368291	Other complications of pregnancy
181	O368292	Other complications of pregnancy
181	O368293	Other complications of pregnancy
181	O368294	Other complications of pregnancy
181	O368295	Other complications of pregnancy
181	O368299	Other complications of pregnancy
181	O368910	Other complications of pregnancy
181	O368911	Other complications of pregnancy
181	O368912	Other complications of pregnancy
181	O368913	Other complications of pregnancy
181	O368914	Other complications of pregnancy
181	O368915	Other complications of pregnancy

181	O368919	Other complications of pregnancy
181	O368920	Other complications of pregnancy
181	O368921	Other complications of pregnancy
181	O368922	Other complications of pregnancy
181	O368923	Other complications of pregnancy
181	O368924	Other complications of pregnancy
181	O368925	Other complications of pregnancy
181	O368929	Other complications of pregnancy
181	O368930	Other complications of pregnancy
181	O368931	Other complications of pregnancy
181	O368932	Other complications of pregnancy
181	O368933	Other complications of pregnancy
181	O368934	Other complications of pregnancy
181	O368935	Other complications of pregnancy
181	O368939	Other complications of pregnancy
181	O368990	Other complications of pregnancy
181	O368991	Other complications of pregnancy
181	O368992	Other complications of pregnancy
181	O368993	Other complications of pregnancy
181	O368994	Other complications of pregnancy
181	O368995	Other complications of pregnancy
181	O368999	Other complications of pregnancy
181	O3690X0	Other complications of pregnancy
181	O3690X1	Other complications of pregnancy
181	O3690X2	Other complications of pregnancy
181	O3690X3	Other complications of pregnancy
181	O3690X4	Other complications of pregnancy
181	O3690X5	Other complications of pregnancy
181	O3690X9	Other complications of pregnancy
181	O3691X0	Other complications of pregnancy
181	O3691X1	Other complications of pregnancy
181	O3691X2	Other complications of pregnancy
181	O3691X3	Other complications of pregnancy
181	O3691X4	Other complications of pregnancy
181	O3691X5	Other complications of pregnancy
181	O3691X9	Other complications of pregnancy
181	O3692X0	Other complications of pregnancy
181	O3692X1	Other complications of pregnancy
181	O3692X2	Other complications of pregnancy
181	O3692X3	Other complications of pregnancy
181	O3692X4	Other complications of pregnancy
181	O3692X5	Other complications of pregnancy
181	O3692X9	Other complications of pregnancy
181	O3693X0	Other complications of pregnancy
181	O3693X1	Other complications of pregnancy
181	O3693X2	Other complications of pregnancy
181	O3693X3	Other complications of pregnancy
181	O3693X4	Other complications of pregnancy
181	O3693X5	Other complications of pregnancy
181	O3693X9	Other complications of pregnancy
181	O43011	Other complications of pregnancy

181	O43012	Other complications of pregnancy
181	O43013	Other complications of pregnancy
181	O43019	Other complications of pregnancy
181	O43021	Other complications of pregnancy
181	O43022	Other complications of pregnancy
181	O43023	Other complications of pregnancy
181	O43029	Other complications of pregnancy
181	O43101	Other complications of pregnancy
181	O43102	Other complications of pregnancy
181	O43103	Other complications of pregnancy
181	O43109	Other complications of pregnancy
181	O43111	Other complications of pregnancy
181	O43112	Other complications of pregnancy
181	O43113	Other complications of pregnancy
181	O43119	Other complications of pregnancy
181	O43191	Other complications of pregnancy
181	O43192	Other complications of pregnancy
181	O43193	Other complications of pregnancy
181	O43199	Other complications of pregnancy
181	O43211	Other complications of pregnancy
181	O43212	Other complications of pregnancy
181	O43213	Other complications of pregnancy
181	O43219	Other complications of pregnancy
181	O43221	Other complications of pregnancy
181	O43222	Other complications of pregnancy
181	O43223	Other complications of pregnancy
181	O43229	Other complications of pregnancy
181	O43231	Other complications of pregnancy
181	O43232	Other complications of pregnancy
181	O43233	Other complications of pregnancy
181	O43239	Other complications of pregnancy
181	O43811	Other complications of pregnancy
181	O43812	Other complications of pregnancy
181	O43813	Other complications of pregnancy
181	O43819	Other complications of pregnancy
181	O43891	Other complications of pregnancy
181	O43892	Other complications of pregnancy
181	O43893	Other complications of pregnancy
181	O43899	Other complications of pregnancy
181	O4390	Other complications of pregnancy
181	O4391	Other complications of pregnancy
181	O4392	Other complications of pregnancy
181	O4393	Other complications of pregnancy
181	O88011	Other complications of pregnancy
181	O88012	Other complications of pregnancy
181	O88013	Other complications of pregnancy
181	O88019	Other complications of pregnancy
181	O88111	Other complications of pregnancy
181	O88112	Other complications of pregnancy
181	O88113	Other complications of pregnancy
181	O88119	Other complications of pregnancy

181	O88211	Other complications of pregnancy
181	O88212	Other complications of pregnancy
181	O88213	Other complications of pregnancy
181	O88219	Other complications of pregnancy
181	O88311	Other complications of pregnancy
181	O88312	Other complications of pregnancy
181	O88313	Other complications of pregnancy
181	O88319	Other complications of pregnancy
181	O88811	Other complications of pregnancy
181	O88812	Other complications of pregnancy
181	O88813	Other complications of pregnancy
181	O88819	Other complications of pregnancy
181	O91011	Other complications of pregnancy
181	O91012	Other complications of pregnancy
181	O91013	Other complications of pregnancy
181	O91019	Other complications of pregnancy
181	O91111	Other complications of pregnancy
181	O91112	Other complications of pregnancy
181	O91113	Other complications of pregnancy
181	O91119	Other complications of pregnancy
181	O91211	Other complications of pregnancy
181	O91212	Other complications of pregnancy
181	O91213	Other complications of pregnancy
181	O91219	Other complications of pregnancy
181	O92011	Other complications of pregnancy
181	O92012	Other complications of pregnancy
181	O92013	Other complications of pregnancy
181	O92019	Other complications of pregnancy
181	O92111	Other complications of pregnancy
181	O92112	Other complications of pregnancy
181	O92113	Other complications of pregnancy
181	O92119	Other complications of pregnancy
181	O98011	Other complications of pregnancy
181	O98012	Other complications of pregnancy
181	O98013	Other complications of pregnancy
181	O98019	Other complications of pregnancy
181	O98111	Other complications of pregnancy
181	O98112	Other complications of pregnancy
181	O98113	Other complications of pregnancy
181	O98119	Other complications of pregnancy
181	O98211	Other complications of pregnancy
181	O98212	Other complications of pregnancy
181	O98213	Other complications of pregnancy
181	O98219	Other complications of pregnancy
181	O98311	Other complications of pregnancy
181	O98312	Other complications of pregnancy
181	O98313	Other complications of pregnancy
181	O98319	Other complications of pregnancy
181	O98411	Other complications of pregnancy
181	O98412	Other complications of pregnancy
181	O98413	Other complications of pregnancy

181	O98419	Other complications of pregnancy
181	O98511	Other complications of pregnancy
181	O98512	Other complications of pregnancy
181	O98513	Other complications of pregnancy
181	O98519	Other complications of pregnancy
181	O98611	Other complications of pregnancy
181	O98612	Other complications of pregnancy
181	O98613	Other complications of pregnancy
181	O98619	Other complications of pregnancy
181	O98711	Other complications of pregnancy
181	O98712	Other complications of pregnancy
181	O98713	Other complications of pregnancy
181	O98719	Other complications of pregnancy
181	O98811	Other complications of pregnancy
181	O98812	Other complications of pregnancy
181	O98813	Other complications of pregnancy
181	O98819	Other complications of pregnancy
181	O98911	Other complications of pregnancy
181	O98912	Other complications of pregnancy
181	O98913	Other complications of pregnancy
181	O98919	Other complications of pregnancy
181	O99011	Other complications of pregnancy
181	O99012	Other complications of pregnancy
181	O99013	Other complications of pregnancy
181	O99019	Other complications of pregnancy
181	O99111	Other complications of pregnancy
181	O99112	Other complications of pregnancy
181	O99113	Other complications of pregnancy
181	O99119	Other complications of pregnancy
181	O99210	Other complications of pregnancy
181	O99211	Other complications of pregnancy
181	O99212	Other complications of pregnancy
181	O99213	Other complications of pregnancy
181	O99280	Other complications of pregnancy
181	O99281	Other complications of pregnancy
181	O99282	Other complications of pregnancy
181	O99283	Other complications of pregnancy
181	O99330	Other complications of pregnancy
181	O99331	Other complications of pregnancy
181	O99332	Other complications of pregnancy
181	O99333	Other complications of pregnancy
181	O99340	Other complications of pregnancy
181	O99341	Other complications of pregnancy
181	O99342	Other complications of pregnancy
181	O99343	Other complications of pregnancy
181	O99345	Other complications of pregnancy
181	O99350	Other complications of pregnancy
181	O99351	Other complications of pregnancy
181	O99352	Other complications of pregnancy
181	O99353	Other complications of pregnancy
181	O99411	Other complications of pregnancy

181	O99412	Other complications of pregnancy
181	O99413	Other complications of pregnancy
181	O99419	Other complications of pregnancy
181	O99511	Other complications of pregnancy
181	O99512	Other complications of pregnancy
181	O99513	Other complications of pregnancy
181	O99519	Other complications of pregnancy
181	O99611	Other complications of pregnancy
181	O99612	Other complications of pregnancy
181	O99613	Other complications of pregnancy
181	O99619	Other complications of pregnancy
181	O99711	Other complications of pregnancy
181	O99712	Other complications of pregnancy
181	O99713	Other complications of pregnancy
181	O99719	Other complications of pregnancy
181	O99820	Other complications of pregnancy
181	O99830	Other complications of pregnancy
181	O99840	Other complications of pregnancy
181	O99841	Other complications of pregnancy
181	O99842	Other complications of pregnancy
181	O99843	Other complications of pregnancy
181	O9989	Other complications of pregnancy
181	O9A111	Other complications of pregnancy
181	O9A112	Other complications of pregnancy
181	O9A113	Other complications of pregnancy
181	O9A119	Other complications of pregnancy
181	O9A211	Other complications of pregnancy
181	O9A212	Other complications of pregnancy
181	O9A213	Other complications of pregnancy
181	O9A219	Other complications of pregnancy
181	O9A311	Other complications of pregnancy
181	O9A312	Other complications of pregnancy
181	O9A313	Other complications of pregnancy
181	O9A319	Other complications of pregnancy
181	O9A411	Other complications of pregnancy
181	O9A412	Other complications of pregnancy
181	O9A413	Other complications of pregnancy
181	O9A419	Other complications of pregnancy
181	O9A511	Other complications of pregnancy
181	O9A512	Other complications of pregnancy
181	O9A513	Other complications of pregnancy
181	O9A519	Other complications of pregnancy
181	Z371	Other complications of pregnancy
181	Z372	Other complications of pregnancy
181	Z373	Other complications of pregnancy
181	Z374	Other complications of pregnancy
181	Z3750	Other complications of pregnancy
181	Z3751	Other complications of pregnancy
181	Z3752	Other complications of pregnancy
181	Z3753	Other complications of pregnancy
181	Z3754	Other complications of pregnancy

181	Z3759	Other complications of pregnancy
181	Z3760	Other complications of pregnancy
181	Z3761	Other complications of pregnancy
181	Z3762	Other complications of pregnancy
181	Z3763	Other complications of pregnancy
181	Z3764	Other complications of pregnancy
181	Z3769	Other complications of pregnancy
181	Z377	Other complications of pregnancy
181	Z379	Other complications of pregnancy
181	Z3A49	Other complications of pregnancy
182	O200	Hemorrhage during pregnancy; abruptio placenta; placenta previa
182	O208	Hemorrhage during pregnancy; abruptio placenta; placenta previa
182	O209	Hemorrhage during pregnancy; abruptio placenta; placenta previa
182	O4400	Hemorrhage during pregnancy; abruptio placenta; placenta previa
182	O4401	Hemorrhage during pregnancy; abruptio placenta; placenta previa
182	O4402	Hemorrhage during pregnancy; abruptio placenta; placenta previa
182	O4403	Hemorrhage during pregnancy; abruptio placenta; placenta previa
182	O4410	Hemorrhage during pregnancy; abruptio placenta; placenta previa
182	O4411	Hemorrhage during pregnancy; abruptio placenta; placenta previa
182	O4412	Hemorrhage during pregnancy; abruptio placenta; placenta previa
182	O4413	Hemorrhage during pregnancy; abruptio placenta; placenta previa
182	O45001	Hemorrhage during pregnancy; abruptio placenta; placenta previa
182	O45002	Hemorrhage during pregnancy; abruptio placenta; placenta previa
182	O45003	Hemorrhage during pregnancy; abruptio placenta; placenta previa
182	O45009	Hemorrhage during pregnancy; abruptio placenta; placenta previa
182	O45011	Hemorrhage during pregnancy; abruptio placenta; placenta previa
182	O45012	Hemorrhage during pregnancy; abruptio placenta; placenta previa
182	O45013	Hemorrhage during pregnancy; abruptio placenta; placenta previa
182	O45019	Hemorrhage during pregnancy; abruptio placenta; placenta previa
182	O45021	Hemorrhage during pregnancy; abruptio placenta; placenta previa
182	O45022	Hemorrhage during pregnancy; abruptio placenta; placenta previa
182	O45023	Hemorrhage during pregnancy; abruptio placenta; placenta previa
182	O45029	Hemorrhage during pregnancy; abruptio placenta; placenta previa
182	O45091	Hemorrhage during pregnancy; abruptio placenta; placenta previa
182	O45092	Hemorrhage during pregnancy; abruptio placenta; placenta previa
182	O45093	Hemorrhage during pregnancy; abruptio placenta; placenta previa
182	O45099	Hemorrhage during pregnancy; abruptio placenta; placenta previa
182	O458X1	Hemorrhage during pregnancy; abruptio placenta; placenta previa
182	O458X2	Hemorrhage during pregnancy; abruptio placenta; placenta previa
182	O458X3	Hemorrhage during pregnancy; abruptio placenta; placenta previa
182	O458X9	Hemorrhage during pregnancy; abruptio placenta; placenta previa
182	O4590	Hemorrhage during pregnancy; abruptio placenta; placenta previa
182	O4591	Hemorrhage during pregnancy; abruptio placenta; placenta previa
182	O4592	Hemorrhage during pregnancy; abruptio placenta; placenta previa
182	O4593	Hemorrhage during pregnancy; abruptio placenta; placenta previa
182	O46001	Hemorrhage during pregnancy; abruptio placenta; placenta previa
182	O46002	Hemorrhage during pregnancy; abruptio placenta; placenta previa
182	O46003	Hemorrhage during pregnancy; abruptio placenta; placenta previa
182	O46009	Hemorrhage during pregnancy; abruptio placenta; placenta previa
182	O46011	Hemorrhage during pregnancy; abruptio placenta; placenta previa
182	O46012	Hemorrhage during pregnancy; abruptio placenta; placenta previa

182	O46013	Hemorrhage during pregnancy; abruptio placenta; placenta previa
182	O46019	Hemorrhage during pregnancy; abruptio placenta; placenta previa
182	O46021	Hemorrhage during pregnancy; abruptio placenta; placenta previa
182	O46022	Hemorrhage during pregnancy; abruptio placenta; placenta previa
182	O46023	Hemorrhage during pregnancy; abruptio placenta; placenta previa
182	O46029	Hemorrhage during pregnancy; abruptio placenta; placenta previa
182	O46091	Hemorrhage during pregnancy; abruptio placenta; placenta previa
182	O46092	Hemorrhage during pregnancy; abruptio placenta; placenta previa
182	O46093	Hemorrhage during pregnancy; abruptio placenta; placenta previa
182	O46099	Hemorrhage during pregnancy; abruptio placenta; placenta previa
182	O468X1	Hemorrhage during pregnancy; abruptio placenta; placenta previa
182	O468X2	Hemorrhage during pregnancy; abruptio placenta; placenta previa
182	O468X3	Hemorrhage during pregnancy; abruptio placenta; placenta previa
182	O468X9	Hemorrhage during pregnancy; abruptio placenta; placenta previa
182	O4690	Hemorrhage during pregnancy; abruptio placenta; placenta previa
182	O4691	Hemorrhage during pregnancy; abruptio placenta; placenta previa
182	O4692	Hemorrhage during pregnancy; abruptio placenta; placenta previa
182	O4693	Hemorrhage during pregnancy; abruptio placenta; placenta previa
182	O670	Hemorrhage during pregnancy; abruptio placenta; placenta previa
182	O678	Hemorrhage during pregnancy; abruptio placenta; placenta previa
182	O679	Hemorrhage during pregnancy; abruptio placenta; placenta previa
183	O10011	Hypertension complicating pregnancy; childbirth and the puerperium
183	O10012	Hypertension complicating pregnancy; childbirth and the puerperium
183	O10013	Hypertension complicating pregnancy; childbirth and the puerperium
183	O10019	Hypertension complicating pregnancy; childbirth and the puerperium
183	O1002	Hypertension complicating pregnancy; childbirth and the puerperium
183	O1003	Hypertension complicating pregnancy; childbirth and the puerperium
183	O10111	Hypertension complicating pregnancy; childbirth and the puerperium
183	O10112	Hypertension complicating pregnancy; childbirth and the puerperium
183	O10113	Hypertension complicating pregnancy; childbirth and the puerperium
183	O10119	Hypertension complicating pregnancy; childbirth and the puerperium
183	O1012	Hypertension complicating pregnancy; childbirth and the puerperium
183	O1013	Hypertension complicating pregnancy; childbirth and the puerperium
183	O10211	Hypertension complicating pregnancy; childbirth and the puerperium
183	O10212	Hypertension complicating pregnancy; childbirth and the puerperium
183	O10213	Hypertension complicating pregnancy; childbirth and the puerperium
183	O10219	Hypertension complicating pregnancy; childbirth and the puerperium
183	O1022	Hypertension complicating pregnancy; childbirth and the puerperium
183	O1023	Hypertension complicating pregnancy; childbirth and the puerperium
183	O10311	Hypertension complicating pregnancy; childbirth and the puerperium
183	O10312	Hypertension complicating pregnancy; childbirth and the puerperium
183	O10313	Hypertension complicating pregnancy; childbirth and the puerperium
183	O10319	Hypertension complicating pregnancy; childbirth and the puerperium
183	O1032	Hypertension complicating pregnancy; childbirth and the puerperium
183	O1033	Hypertension complicating pregnancy; childbirth and the puerperium
183	O10411	Hypertension complicating pregnancy; childbirth and the puerperium
183	O10412	Hypertension complicating pregnancy; childbirth and the puerperium
183	O10413	Hypertension complicating pregnancy; childbirth and the puerperium
183	O10419	Hypertension complicating pregnancy; childbirth and the puerperium
183	O1042	Hypertension complicating pregnancy; childbirth and the puerperium
183	O1043	Hypertension complicating pregnancy; childbirth and the puerperium

183	O10911	Hypertension complicating pregnancy; childbirth and the puerperium
183	O10912	Hypertension complicating pregnancy; childbirth and the puerperium
183	O10913	Hypertension complicating pregnancy; childbirth and the puerperium
183	O10919	Hypertension complicating pregnancy; childbirth and the puerperium
183	O1092	Hypertension complicating pregnancy; childbirth and the puerperium
183	O1093	Hypertension complicating pregnancy; childbirth and the puerperium
183	O111	Hypertension complicating pregnancy; childbirth and the puerperium
183	O112	Hypertension complicating pregnancy; childbirth and the puerperium
183	O113	Hypertension complicating pregnancy; childbirth and the puerperium
183	O119	Hypertension complicating pregnancy; childbirth and the puerperium
183	O131	Hypertension complicating pregnancy; childbirth and the puerperium
183	O132	Hypertension complicating pregnancy; childbirth and the puerperium
183	O133	Hypertension complicating pregnancy; childbirth and the puerperium
183	O139	Hypertension complicating pregnancy; childbirth and the puerperium
183	O1400	Hypertension complicating pregnancy; childbirth and the puerperium
183	O1402	Hypertension complicating pregnancy; childbirth and the puerperium
183	O1403	Hypertension complicating pregnancy; childbirth and the puerperium
183	O1410	Hypertension complicating pregnancy; childbirth and the puerperium
183	O1412	Hypertension complicating pregnancy; childbirth and the puerperium
183	O1413	Hypertension complicating pregnancy; childbirth and the puerperium
183	O1420	Hypertension complicating pregnancy; childbirth and the puerperium
183	O1422	Hypertension complicating pregnancy; childbirth and the puerperium
183	O1423	Hypertension complicating pregnancy; childbirth and the puerperium
183	O1490	Hypertension complicating pregnancy; childbirth and the puerperium
183	O1492	Hypertension complicating pregnancy; childbirth and the puerperium
183	O1493	Hypertension complicating pregnancy; childbirth and the puerperium
183	O1500	Hypertension complicating pregnancy; childbirth and the puerperium
183	O1502	Hypertension complicating pregnancy; childbirth and the puerperium
183	O1503	Hypertension complicating pregnancy; childbirth and the puerperium
183	O151	Hypertension complicating pregnancy; childbirth and the puerperium
183	O152	Hypertension complicating pregnancy; childbirth and the puerperium
183	O159	Hypertension complicating pregnancy; childbirth and the puerperium
183	O161	Hypertension complicating pregnancy; childbirth and the puerperium
183	O162	Hypertension complicating pregnancy; childbirth and the puerperium
183	O163	Hypertension complicating pregnancy; childbirth and the puerperium
183	O169	Hypertension complicating pregnancy; childbirth and the puerperium
184	O4700	Early or threatened labor
184	O4702	Early or threatened labor
184	O4703	Early or threatened labor
184	O471	Early or threatened labor
184	O479	Early or threatened labor
184	O6000	Early or threatened labor
184	O6002	Early or threatened labor
184	O6003	Early or threatened labor
184	O6010X0	Early or threatened labor
184	O6010X1	Early or threatened labor
184	O6010X2	Early or threatened labor
184	O6010X3	Early or threatened labor
184	O6010X4	Early or threatened labor
184	O6010X5	Early or threatened labor
184	O6010X9	Early or threatened labor

184	O6012X0	Early or threatened labor
184	O6012X1	Early or threatened labor
184	O6012X2	Early or threatened labor
184	O6012X3	Early or threatened labor
184	O6012X4	Early or threatened labor
184	O6012X5	Early or threatened labor
184	O6012X9	Early or threatened labor
184	O6013X0	Early or threatened labor
184	O6013X1	Early or threatened labor
184	O6013X2	Early or threatened labor
184	O6013X3	Early or threatened labor
184	O6013X4	Early or threatened labor
184	O6013X5	Early or threatened labor
184	O6013X9	Early or threatened labor
184	O6014X0	Early or threatened labor
184	O6014X1	Early or threatened labor
184	O6014X2	Early or threatened labor
184	O6014X3	Early or threatened labor
184	O6014X4	Early or threatened labor
184	O6014X5	Early or threatened labor
184	O6014X9	Early or threatened labor
184	O6020X0	Early or threatened labor
184	O6020X1	Early or threatened labor
184	O6020X2	Early or threatened labor
184	O6020X3	Early or threatened labor
184	O6020X4	Early or threatened labor
184	O6020X5	Early or threatened labor
184	O6020X9	Early or threatened labor
184	O6022X0	Early or threatened labor
184	O6022X1	Early or threatened labor
184	O6022X2	Early or threatened labor
184	O6022X3	Early or threatened labor
184	O6022X4	Early or threatened labor
184	O6022X5	Early or threatened labor
184	O6022X9	Early or threatened labor
184	O6023X0	Early or threatened labor
184	O6023X1	Early or threatened labor
184	O6023X2	Early or threatened labor
184	O6023X3	Early or threatened labor
184	O6023X4	Early or threatened labor
184	O6023X5	Early or threatened labor
184	O6023X9	Early or threatened labor
185	O480	Prolonged pregnancy
185	O481	Prolonged pregnancy
186	O24011	Diabetes or abnormal glucose tolerance complicating pregnancy; childbirth; or the puerperium
186	O24012	Diabetes or abnormal glucose tolerance complicating pregnancy; childbirth; or the puerperium
186	O24013	Diabetes or abnormal glucose tolerance complicating pregnancy; childbirth; or the puerperium
186	O24019	Diabetes or abnormal glucose tolerance complicating pregnancy; childbirth; or the puerperium
186	O2402	Diabetes or abnormal glucose tolerance complicating pregnancy; childbirth; or the puerperium
186	O2403	Diabetes or abnormal glucose tolerance complicating pregnancy; childbirth; or the puerperium
186	O24111	Diabetes or abnormal glucose tolerance complicating pregnancy; childbirth; or the puerperium

186	O24112	Diabetes or abnormal glucose tolerance complicating pregnancy; childbirth; or the puerperium
186	O24113	Diabetes or abnormal glucose tolerance complicating pregnancy; childbirth; or the puerperium
186	O24119	Diabetes or abnormal glucose tolerance complicating pregnancy; childbirth; or the puerperium
186	O2412	Diabetes or abnormal glucose tolerance complicating pregnancy; childbirth; or the puerperium
186	O2413	Diabetes or abnormal glucose tolerance complicating pregnancy; childbirth; or the puerperium
186	O24311	Diabetes or abnormal glucose tolerance complicating pregnancy; childbirth; or the puerperium
186	O24312	Diabetes or abnormal glucose tolerance complicating pregnancy; childbirth; or the puerperium
186	O24313	Diabetes or abnormal glucose tolerance complicating pregnancy; childbirth; or the puerperium
186	O24319	Diabetes or abnormal glucose tolerance complicating pregnancy; childbirth; or the puerperium
186	O2432	Diabetes or abnormal glucose tolerance complicating pregnancy; childbirth; or the puerperium
186	O2433	Diabetes or abnormal glucose tolerance complicating pregnancy; childbirth; or the puerperium
186	O24410	Diabetes or abnormal glucose tolerance complicating pregnancy; childbirth; or the puerperium
186	O24414	Diabetes or abnormal glucose tolerance complicating pregnancy; childbirth; or the puerperium
186	O24419	Diabetes or abnormal glucose tolerance complicating pregnancy; childbirth; or the puerperium
186	O24420	Diabetes or abnormal glucose tolerance complicating pregnancy; childbirth; or the puerperium
186	O24424	Diabetes or abnormal glucose tolerance complicating pregnancy; childbirth; or the puerperium
186	O24429	Diabetes or abnormal glucose tolerance complicating pregnancy; childbirth; or the puerperium
186	O24430	Diabetes or abnormal glucose tolerance complicating pregnancy; childbirth; or the puerperium
186	O24434	Diabetes or abnormal glucose tolerance complicating pregnancy; childbirth; or the puerperium
186	O24439	Diabetes or abnormal glucose tolerance complicating pregnancy; childbirth; or the puerperium
186	O24811	Diabetes or abnormal glucose tolerance complicating pregnancy; childbirth; or the puerperium
186	O24812	Diabetes or abnormal glucose tolerance complicating pregnancy; childbirth; or the puerperium
186	O24813	Diabetes or abnormal glucose tolerance complicating pregnancy; childbirth; or the puerperium
186	O24819	Diabetes or abnormal glucose tolerance complicating pregnancy; childbirth; or the puerperium
186	O2482	Diabetes or abnormal glucose tolerance complicating pregnancy; childbirth; or the puerperium
186	O2483	Diabetes or abnormal glucose tolerance complicating pregnancy; childbirth; or the puerperium
186	O24911	Diabetes or abnormal glucose tolerance complicating pregnancy; childbirth; or the puerperium
186	O24912	Diabetes or abnormal glucose tolerance complicating pregnancy; childbirth; or the puerperium
186	O24913	Diabetes or abnormal glucose tolerance complicating pregnancy; childbirth; or the puerperium
186	O24919	Diabetes or abnormal glucose tolerance complicating pregnancy; childbirth; or the puerperium
186	O2492	Diabetes or abnormal glucose tolerance complicating pregnancy; childbirth; or the puerperium
186	O2493	Diabetes or abnormal glucose tolerance complicating pregnancy; childbirth; or the puerperium
186	O99810	Diabetes or abnormal glucose tolerance complicating pregnancy; childbirth; or the puerperium
186	O99814	Diabetes or abnormal glucose tolerance complicating pregnancy; childbirth; or the puerperium
186	O99815	Diabetes or abnormal glucose tolerance complicating pregnancy; childbirth; or the puerperium
186	Z8632	Diabetes or abnormal glucose tolerance complicating pregnancy; childbirth; or the puerperium
187	O320XX0	Malposition; malpresentation
187	O320XX1	Malposition; malpresentation
187	O320XX2	Malposition; malpresentation
187	O320XX3	Malposition; malpresentation
187	O320XX4	Malposition; malpresentation
187	O320XX5	Malposition; malpresentation
187	O320XX9	Malposition; malpresentation
187	O321XX0	Malposition; malpresentation
187	O321XX1	Malposition; malpresentation
187	O321XX2	Malposition; malpresentation
187	O321XX3	Malposition; malpresentation
187	O321XX4	Malposition; malpresentation
187	O321XX5	Malposition; malpresentation
187	O321XX9	Malposition; malpresentation
187	O322XX0	Malposition; malpresentation

187	O322XX1	Malposition; malpresentation
187	O322XX2	Malposition; malpresentation
187	O322XX3	Malposition; malpresentation
187	O322XX4	Malposition; malpresentation
187	O322XX5	Malposition; malpresentation
187	O322XX9	Malposition; malpresentation
187	O323XX0	Malposition; malpresentation
187	O323XX1	Malposition; malpresentation
187	O323XX2	Malposition; malpresentation
187	O323XX3	Malposition; malpresentation
187	O323XX4	Malposition; malpresentation
187	O323XX5	Malposition; malpresentation
187	O323XX9	Malposition; malpresentation
187	O324XX0	Malposition; malpresentation
187	O324XX1	Malposition; malpresentation
187	O324XX2	Malposition; malpresentation
187	O324XX3	Malposition; malpresentation
187	O324XX4	Malposition; malpresentation
187	O324XX5	Malposition; malpresentation
187	O324XX9	Malposition; malpresentation
187	O326XX0	Malposition; malpresentation
187	O326XX1	Malposition; malpresentation
187	O326XX2	Malposition; malpresentation
187	O326XX3	Malposition; malpresentation
187	O326XX4	Malposition; malpresentation
187	O326XX5	Malposition; malpresentation
187	O326XX9	Malposition; malpresentation
187	O328XX0	Malposition; malpresentation
187	O328XX1	Malposition; malpresentation
187	O328XX2	Malposition; malpresentation
187	O328XX3	Malposition; malpresentation
187	O328XX4	Malposition; malpresentation
187	O328XX5	Malposition; malpresentation
187	O328XX9	Malposition; malpresentation
187	O329XX0	Malposition; malpresentation
187	O329XX1	Malposition; malpresentation
187	O329XX2	Malposition; malpresentation
187	O329XX3	Malposition; malpresentation
187	O329XX4	Malposition; malpresentation
187	O329XX5	Malposition; malpresentation
187	O329XX9	Malposition; malpresentation
187	O641XX0	Malposition; malpresentation
187	O641XX1	Malposition; malpresentation
187	O641XX2	Malposition; malpresentation
187	O641XX3	Malposition; malpresentation
187	O641XX4	Malposition; malpresentation
187	O641XX5	Malposition; malpresentation
187	O641XX9	Malposition; malpresentation
187	O642XX0	Malposition; malpresentation
187	O642XX1	Malposition; malpresentation
187	O642XX2	Malposition; malpresentation

187	O642XX3	Malposition; malpresentation
187	O642XX4	Malposition; malpresentation
187	O642XX5	Malposition; malpresentation
187	O642XX9	Malposition; malpresentation
187	O643XX0	Malposition; malpresentation
187	O643XX1	Malposition; malpresentation
187	O643XX2	Malposition; malpresentation
187	O643XX3	Malposition; malpresentation
187	O643XX4	Malposition; malpresentation
187	O643XX5	Malposition; malpresentation
187	O643XX9	Malposition; malpresentation
187	O644XX0	Malposition; malpresentation
187	O644XX1	Malposition; malpresentation
187	O644XX2	Malposition; malpresentation
187	O644XX3	Malposition; malpresentation
187	O644XX4	Malposition; malpresentation
187	O644XX5	Malposition; malpresentation
187	O644XX9	Malposition; malpresentation
187	O645XX0	Malposition; malpresentation
187	O645XX1	Malposition; malpresentation
187	O645XX2	Malposition; malpresentation
187	O645XX3	Malposition; malpresentation
187	O645XX4	Malposition; malpresentation
187	O645XX5	Malposition; malpresentation
187	O645XX9	Malposition; malpresentation
187	O648XX0	Malposition; malpresentation
187	O648XX1	Malposition; malpresentation
187	O648XX2	Malposition; malpresentation
187	O648XX3	Malposition; malpresentation
187	O648XX4	Malposition; malpresentation
187	O648XX5	Malposition; malpresentation
187	O648XX9	Malposition; malpresentation
187	O649XX0	Malposition; malpresentation
187	O649XX1	Malposition; malpresentation
187	O649XX2	Malposition; malpresentation
187	O649XX3	Malposition; malpresentation
187	O649XX4	Malposition; malpresentation
187	O649XX5	Malposition; malpresentation
187	O649XX9	Malposition; malpresentation
188	O30021	Fetopelvic disproportion; obstruction
188	O30022	Fetopelvic disproportion; obstruction
188	O30023	Fetopelvic disproportion; obstruction
188	O30029	Fetopelvic disproportion; obstruction
188	O330	Fetopelvic disproportion; obstruction
188	O331	Fetopelvic disproportion; obstruction
188	O332	Fetopelvic disproportion; obstruction
188	O333XX0	Fetopelvic disproportion; obstruction
188	O333XX1	Fetopelvic disproportion; obstruction
188	O333XX2	Fetopelvic disproportion; obstruction
188	O333XX3	Fetopelvic disproportion; obstruction
188	O333XX4	Fetopelvic disproportion; obstruction

188	O333XX5	Fetopelvic disproportion; obstruction
188	O333XX9	Fetopelvic disproportion; obstruction
188	O334XX0	Fetopelvic disproportion; obstruction
188	O334XX1	Fetopelvic disproportion; obstruction
188	O334XX2	Fetopelvic disproportion; obstruction
188	O334XX3	Fetopelvic disproportion; obstruction
188	O334XX4	Fetopelvic disproportion; obstruction
188	O334XX5	Fetopelvic disproportion; obstruction
188	O334XX9	Fetopelvic disproportion; obstruction
188	O335XX0	Fetopelvic disproportion; obstruction
188	O335XX1	Fetopelvic disproportion; obstruction
188	O335XX2	Fetopelvic disproportion; obstruction
188	O335XX3	Fetopelvic disproportion; obstruction
188	O335XX4	Fetopelvic disproportion; obstruction
188	O335XX5	Fetopelvic disproportion; obstruction
188	O335XX9	Fetopelvic disproportion; obstruction
188	O336XX0	Fetopelvic disproportion; obstruction
188	O336XX1	Fetopelvic disproportion; obstruction
188	O336XX2	Fetopelvic disproportion; obstruction
188	O336XX3	Fetopelvic disproportion; obstruction
188	O336XX4	Fetopelvic disproportion; obstruction
188	O336XX5	Fetopelvic disproportion; obstruction
188	O336XX9	Fetopelvic disproportion; obstruction
188	O337	Fetopelvic disproportion; obstruction
188	O338	Fetopelvic disproportion; obstruction
188	O339	Fetopelvic disproportion; obstruction
188	O640XX0	Fetopelvic disproportion; obstruction
188	O640XX1	Fetopelvic disproportion; obstruction
188	O640XX2	Fetopelvic disproportion; obstruction
188	O640XX3	Fetopelvic disproportion; obstruction
188	O640XX4	Fetopelvic disproportion; obstruction
188	O640XX5	Fetopelvic disproportion; obstruction
188	O640XX9	Fetopelvic disproportion; obstruction
188	O650	Fetopelvic disproportion; obstruction
188	O651	Fetopelvic disproportion; obstruction
188	O652	Fetopelvic disproportion; obstruction
188	O653	Fetopelvic disproportion; obstruction
188	O654	Fetopelvic disproportion; obstruction
188	O655	Fetopelvic disproportion; obstruction
188	O658	Fetopelvic disproportion; obstruction
188	O659	Fetopelvic disproportion; obstruction
188	O660	Fetopelvic disproportion; obstruction
188	O661	Fetopelvic disproportion; obstruction
188	O662	Fetopelvic disproportion; obstruction
188	O663	Fetopelvic disproportion; obstruction
188	O6640	Fetopelvic disproportion; obstruction
188	O6641	Fetopelvic disproportion; obstruction
188	O666	Fetopelvic disproportion; obstruction
188	O668	Fetopelvic disproportion; obstruction
188	O669	Fetopelvic disproportion; obstruction
189	O3421	Previous C-section

190	O620	Fetal distress and abnormal forces of labor
190	O621	Fetal distress and abnormal forces of labor
190	O622	Fetal distress and abnormal forces of labor
190	O623	Fetal distress and abnormal forces of labor
190	O624	Fetal distress and abnormal forces of labor
190	O628	Fetal distress and abnormal forces of labor
190	O629	Fetal distress and abnormal forces of labor
190	O630	Fetal distress and abnormal forces of labor
190	O631	Fetal distress and abnormal forces of labor
190	O632	Fetal distress and abnormal forces of labor
190	O639	Fetal distress and abnormal forces of labor
190	O68	Fetal distress and abnormal forces of labor
190	O771	Fetal distress and abnormal forces of labor
190	O778	Fetal distress and abnormal forces of labor
190	O779	Fetal distress and abnormal forces of labor
191	O401XX0	Polyhydramnios and other problems of amniotic cavity
191	O401XX1	Polyhydramnios and other problems of amniotic cavity
191	O401XX2	Polyhydramnios and other problems of amniotic cavity
191	O401XX3	Polyhydramnios and other problems of amniotic cavity
191	O401XX4	Polyhydramnios and other problems of amniotic cavity
191	O401XX5	Polyhydramnios and other problems of amniotic cavity
191	O401XX9	Polyhydramnios and other problems of amniotic cavity
191	O402XX0	Polyhydramnios and other problems of amniotic cavity
191	O402XX1	Polyhydramnios and other problems of amniotic cavity
191	O402XX2	Polyhydramnios and other problems of amniotic cavity
191	O402XX3	Polyhydramnios and other problems of amniotic cavity
191	O402XX4	Polyhydramnios and other problems of amniotic cavity
191	O402XX5	Polyhydramnios and other problems of amniotic cavity
191	O402XX9	Polyhydramnios and other problems of amniotic cavity
191	O403XX0	Polyhydramnios and other problems of amniotic cavity
191	O403XX1	Polyhydramnios and other problems of amniotic cavity
191	O403XX2	Polyhydramnios and other problems of amniotic cavity
191	O403XX3	Polyhydramnios and other problems of amniotic cavity
191	O403XX4	Polyhydramnios and other problems of amniotic cavity
191	O403XX5	Polyhydramnios and other problems of amniotic cavity
191	O403XX9	Polyhydramnios and other problems of amniotic cavity
191	O409XX0	Polyhydramnios and other problems of amniotic cavity
191	O409XX1	Polyhydramnios and other problems of amniotic cavity
191	O409XX2	Polyhydramnios and other problems of amniotic cavity
191	O409XX3	Polyhydramnios and other problems of amniotic cavity
191	O409XX4	Polyhydramnios and other problems of amniotic cavity
191	O409XX5	Polyhydramnios and other problems of amniotic cavity
191	O409XX9	Polyhydramnios and other problems of amniotic cavity
191	O4100X0	Polyhydramnios and other problems of amniotic cavity
191	O4100X1	Polyhydramnios and other problems of amniotic cavity
191	O4100X2	Polyhydramnios and other problems of amniotic cavity
191	O4100X3	Polyhydramnios and other problems of amniotic cavity
191	O4100X4	Polyhydramnios and other problems of amniotic cavity
191	O4100X5	Polyhydramnios and other problems of amniotic cavity
191	O4100X9	Polyhydramnios and other problems of amniotic cavity
191	O4101X0	Polyhydramnios and other problems of amniotic cavity

191	O4101X1	Polyhydramnios and other problems of amniotic cavity
191	O4101X2	Polyhydramnios and other problems of amniotic cavity
191	O4101X3	Polyhydramnios and other problems of amniotic cavity
191	O4101X4	Polyhydramnios and other problems of amniotic cavity
191	O4101X5	Polyhydramnios and other problems of amniotic cavity
191	O4101X9	Polyhydramnios and other problems of amniotic cavity
191	O4102X0	Polyhydramnios and other problems of amniotic cavity
191	O4102X1	Polyhydramnios and other problems of amniotic cavity
191	O4102X2	Polyhydramnios and other problems of amniotic cavity
191	O4102X3	Polyhydramnios and other problems of amniotic cavity
191	O4102X4	Polyhydramnios and other problems of amniotic cavity
191	O4102X5	Polyhydramnios and other problems of amniotic cavity
191	O4102X9	Polyhydramnios and other problems of amniotic cavity
191	O4103X0	Polyhydramnios and other problems of amniotic cavity
191	O4103X1	Polyhydramnios and other problems of amniotic cavity
191	O4103X2	Polyhydramnios and other problems of amniotic cavity
191	O4103X3	Polyhydramnios and other problems of amniotic cavity
191	O4103X4	Polyhydramnios and other problems of amniotic cavity
191	O4103X5	Polyhydramnios and other problems of amniotic cavity
191	O4103X9	Polyhydramnios and other problems of amniotic cavity
191	O411010	Polyhydramnios and other problems of amniotic cavity
191	O411011	Polyhydramnios and other problems of amniotic cavity
191	O411012	Polyhydramnios and other problems of amniotic cavity
191	O411013	Polyhydramnios and other problems of amniotic cavity
191	O411014	Polyhydramnios and other problems of amniotic cavity
191	O411015	Polyhydramnios and other problems of amniotic cavity
191	O411019	Polyhydramnios and other problems of amniotic cavity
191	O411020	Polyhydramnios and other problems of amniotic cavity
191	O411021	Polyhydramnios and other problems of amniotic cavity
191	O411022	Polyhydramnios and other problems of amniotic cavity
191	O411023	Polyhydramnios and other problems of amniotic cavity
191	O411024	Polyhydramnios and other problems of amniotic cavity
191	O411025	Polyhydramnios and other problems of amniotic cavity
191	O411029	Polyhydramnios and other problems of amniotic cavity
191	O411030	Polyhydramnios and other problems of amniotic cavity
191	O411031	Polyhydramnios and other problems of amniotic cavity
191	O411032	Polyhydramnios and other problems of amniotic cavity
191	O411033	Polyhydramnios and other problems of amniotic cavity
191	O411034	Polyhydramnios and other problems of amniotic cavity
191	O411035	Polyhydramnios and other problems of amniotic cavity
191	O411039	Polyhydramnios and other problems of amniotic cavity
191	O411090	Polyhydramnios and other problems of amniotic cavity
191	O411091	Polyhydramnios and other problems of amniotic cavity
191	O411092	Polyhydramnios and other problems of amniotic cavity
191	O411093	Polyhydramnios and other problems of amniotic cavity
191	O411094	Polyhydramnios and other problems of amniotic cavity
191	O411095	Polyhydramnios and other problems of amniotic cavity
191	O411099	Polyhydramnios and other problems of amniotic cavity
191	O411210	Polyhydramnios and other problems of amniotic cavity
191	O411211	Polyhydramnios and other problems of amniotic cavity
191	O411212	Polyhydramnios and other problems of amniotic cavity

191	O411213	Polyhydramnios and other problems of amniotic cavity
191	O411214	Polyhydramnios and other problems of amniotic cavity
191	O411215	Polyhydramnios and other problems of amniotic cavity
191	O411219	Polyhydramnios and other problems of amniotic cavity
191	O411220	Polyhydramnios and other problems of amniotic cavity
191	O411221	Polyhydramnios and other problems of amniotic cavity
191	O411222	Polyhydramnios and other problems of amniotic cavity
191	O411223	Polyhydramnios and other problems of amniotic cavity
191	O411224	Polyhydramnios and other problems of amniotic cavity
191	O411225	Polyhydramnios and other problems of amniotic cavity
191	O411229	Polyhydramnios and other problems of amniotic cavity
191	O411230	Polyhydramnios and other problems of amniotic cavity
191	O411231	Polyhydramnios and other problems of amniotic cavity
191	O411232	Polyhydramnios and other problems of amniotic cavity
191	O411233	Polyhydramnios and other problems of amniotic cavity
191	O411234	Polyhydramnios and other problems of amniotic cavity
191	O411235	Polyhydramnios and other problems of amniotic cavity
191	O411239	Polyhydramnios and other problems of amniotic cavity
191	O411290	Polyhydramnios and other problems of amniotic cavity
191	O411291	Polyhydramnios and other problems of amniotic cavity
191	O411292	Polyhydramnios and other problems of amniotic cavity
191	O411293	Polyhydramnios and other problems of amniotic cavity
191	O411294	Polyhydramnios and other problems of amniotic cavity
191	O411295	Polyhydramnios and other problems of amniotic cavity
191	O411299	Polyhydramnios and other problems of amniotic cavity
191	O411410	Polyhydramnios and other problems of amniotic cavity
191	O411411	Polyhydramnios and other problems of amniotic cavity
191	O411412	Polyhydramnios and other problems of amniotic cavity
191	O411413	Polyhydramnios and other problems of amniotic cavity
191	O411414	Polyhydramnios and other problems of amniotic cavity
191	O411415	Polyhydramnios and other problems of amniotic cavity
191	O411419	Polyhydramnios and other problems of amniotic cavity
191	O411420	Polyhydramnios and other problems of amniotic cavity
191	O411421	Polyhydramnios and other problems of amniotic cavity
191	O411422	Polyhydramnios and other problems of amniotic cavity
191	O411423	Polyhydramnios and other problems of amniotic cavity
191	O411424	Polyhydramnios and other problems of amniotic cavity
191	O411425	Polyhydramnios and other problems of amniotic cavity
191	O411429	Polyhydramnios and other problems of amniotic cavity
191	O411430	Polyhydramnios and other problems of amniotic cavity
191	O411431	Polyhydramnios and other problems of amniotic cavity
191	O411432	Polyhydramnios and other problems of amniotic cavity
191	O411433	Polyhydramnios and other problems of amniotic cavity
191	O411434	Polyhydramnios and other problems of amniotic cavity
191	O411435	Polyhydramnios and other problems of amniotic cavity
191	O411439	Polyhydramnios and other problems of amniotic cavity
191	O411490	Polyhydramnios and other problems of amniotic cavity
191	O411491	Polyhydramnios and other problems of amniotic cavity
191	O411492	Polyhydramnios and other problems of amniotic cavity
191	O411493	Polyhydramnios and other problems of amniotic cavity
191	O411494	Polyhydramnios and other problems of amniotic cavity

191	O411495	Polyhydramnios and other problems of amniotic cavity
191	O411499	Polyhydramnios and other problems of amniotic cavity
191	O418X10	Polyhydramnios and other problems of amniotic cavity
191	O418X11	Polyhydramnios and other problems of amniotic cavity
191	O418X12	Polyhydramnios and other problems of amniotic cavity
191	O418X13	Polyhydramnios and other problems of amniotic cavity
191	O418X14	Polyhydramnios and other problems of amniotic cavity
191	O418X15	Polyhydramnios and other problems of amniotic cavity
191	O418X19	Polyhydramnios and other problems of amniotic cavity
191	O418X20	Polyhydramnios and other problems of amniotic cavity
191	O418X21	Polyhydramnios and other problems of amniotic cavity
191	O418X22	Polyhydramnios and other problems of amniotic cavity
191	O418X23	Polyhydramnios and other problems of amniotic cavity
191	O418X24	Polyhydramnios and other problems of amniotic cavity
191	O418X25	Polyhydramnios and other problems of amniotic cavity
191	O418X29	Polyhydramnios and other problems of amniotic cavity
191	O418X30	Polyhydramnios and other problems of amniotic cavity
191	O418X31	Polyhydramnios and other problems of amniotic cavity
191	O418X32	Polyhydramnios and other problems of amniotic cavity
191	O418X33	Polyhydramnios and other problems of amniotic cavity
191	O418X34	Polyhydramnios and other problems of amniotic cavity
191	O418X35	Polyhydramnios and other problems of amniotic cavity
191	O418X39	Polyhydramnios and other problems of amniotic cavity
191	O418X90	Polyhydramnios and other problems of amniotic cavity
191	O418X91	Polyhydramnios and other problems of amniotic cavity
191	O418X92	Polyhydramnios and other problems of amniotic cavity
191	O418X93	Polyhydramnios and other problems of amniotic cavity
191	O418X94	Polyhydramnios and other problems of amniotic cavity
191	O418X95	Polyhydramnios and other problems of amniotic cavity
191	O418X99	Polyhydramnios and other problems of amniotic cavity
191	O4190X0	Polyhydramnios and other problems of amniotic cavity
191	O4190X1	Polyhydramnios and other problems of amniotic cavity
191	O4190X2	Polyhydramnios and other problems of amniotic cavity
191	O4190X3	Polyhydramnios and other problems of amniotic cavity
191	O4190X4	Polyhydramnios and other problems of amniotic cavity
191	O4190X5	Polyhydramnios and other problems of amniotic cavity
191	O4190X9	Polyhydramnios and other problems of amniotic cavity
191	O4191X0	Polyhydramnios and other problems of amniotic cavity
191	O4191X1	Polyhydramnios and other problems of amniotic cavity
191	O4191X2	Polyhydramnios and other problems of amniotic cavity
191	O4191X3	Polyhydramnios and other problems of amniotic cavity
191	O4191X4	Polyhydramnios and other problems of amniotic cavity
191	O4191X5	Polyhydramnios and other problems of amniotic cavity
191	O4191X9	Polyhydramnios and other problems of amniotic cavity
191	O4192X0	Polyhydramnios and other problems of amniotic cavity
191	O4192X1	Polyhydramnios and other problems of amniotic cavity
191	O4192X2	Polyhydramnios and other problems of amniotic cavity
191	O4192X3	Polyhydramnios and other problems of amniotic cavity
191	O4192X4	Polyhydramnios and other problems of amniotic cavity
191	O4192X5	Polyhydramnios and other problems of amniotic cavity
191	O4192X9	Polyhydramnios and other problems of amniotic cavity

191	O4193X0	Polyhydramnios and other problems of amniotic cavity
191	O4193X1	Polyhydramnios and other problems of amniotic cavity
191	O4193X2	Polyhydramnios and other problems of amniotic cavity
191	O4193X3	Polyhydramnios and other problems of amniotic cavity
191	O4193X4	Polyhydramnios and other problems of amniotic cavity
191	O4193X5	Polyhydramnios and other problems of amniotic cavity
191	O4193X9	Polyhydramnios and other problems of amniotic cavity
191	O4200	Polyhydramnios and other problems of amniotic cavity
191	O42011	Polyhydramnios and other problems of amniotic cavity
191	O42012	Polyhydramnios and other problems of amniotic cavity
191	O42013	Polyhydramnios and other problems of amniotic cavity
191	O42019	Polyhydramnios and other problems of amniotic cavity
191	O4202	Polyhydramnios and other problems of amniotic cavity
191	O4210	Polyhydramnios and other problems of amniotic cavity
191	O42111	Polyhydramnios and other problems of amniotic cavity
191	O42112	Polyhydramnios and other problems of amniotic cavity
191	O42113	Polyhydramnios and other problems of amniotic cavity
191	O42119	Polyhydramnios and other problems of amniotic cavity
191	O4212	Polyhydramnios and other problems of amniotic cavity
191	O4290	Polyhydramnios and other problems of amniotic cavity
191	O42911	Polyhydramnios and other problems of amniotic cavity
191	O42912	Polyhydramnios and other problems of amniotic cavity
191	O42913	Polyhydramnios and other problems of amniotic cavity
191	O42919	Polyhydramnios and other problems of amniotic cavity
191	O4292	Polyhydramnios and other problems of amniotic cavity
192	O43121	Umbilical cord complication
192	O43122	Umbilical cord complication
192	O43123	Umbilical cord complication
192	O43129	Umbilical cord complication
192	O690XX0	Umbilical cord complication
192	O690XX1	Umbilical cord complication
192	O690XX2	Umbilical cord complication
192	O690XX3	Umbilical cord complication
192	O690XX4	Umbilical cord complication
192	O690XX5	Umbilical cord complication
192	O690XX9	Umbilical cord complication
192	O691XX0	Umbilical cord complication
192	O691XX1	Umbilical cord complication
192	O691XX2	Umbilical cord complication
192	O691XX3	Umbilical cord complication
192	O691XX4	Umbilical cord complication
192	O691XX5	Umbilical cord complication
192	O691XX9	Umbilical cord complication
192	O692XX0	Umbilical cord complication
192	O692XX1	Umbilical cord complication
192	O692XX2	Umbilical cord complication
192	O692XX3	Umbilical cord complication
192	O692XX4	Umbilical cord complication
192	O692XX5	Umbilical cord complication
192	O692XX9	Umbilical cord complication
192	O693XX0	Umbilical cord complication

192	O693XX1	Umbilical cord complication
192	O693XX2	Umbilical cord complication
192	O693XX3	Umbilical cord complication
192	O693XX4	Umbilical cord complication
192	O693XX5	Umbilical cord complication
192	O693XX9	Umbilical cord complication
192	O694XX0	Umbilical cord complication
192	O694XX1	Umbilical cord complication
192	O694XX2	Umbilical cord complication
192	O694XX3	Umbilical cord complication
192	O694XX4	Umbilical cord complication
192	O694XX5	Umbilical cord complication
192	O694XX9	Umbilical cord complication
192	O695XX0	Umbilical cord complication
192	O695XX1	Umbilical cord complication
192	O695XX2	Umbilical cord complication
192	O695XX3	Umbilical cord complication
192	O695XX4	Umbilical cord complication
192	O695XX5	Umbilical cord complication
192	O695XX9	Umbilical cord complication
192	O6981X0	Umbilical cord complication
192	O6981X1	Umbilical cord complication
192	O6981X2	Umbilical cord complication
192	O6981X3	Umbilical cord complication
192	O6981X4	Umbilical cord complication
192	O6981X5	Umbilical cord complication
192	O6981X9	Umbilical cord complication
192	O6982X0	Umbilical cord complication
192	O6982X1	Umbilical cord complication
192	O6982X2	Umbilical cord complication
192	O6982X3	Umbilical cord complication
192	O6982X4	Umbilical cord complication
192	O6982X5	Umbilical cord complication
192	O6982X9	Umbilical cord complication
192	O6989X0	Umbilical cord complication
192	O6989X1	Umbilical cord complication
192	O6989X2	Umbilical cord complication
192	O6989X3	Umbilical cord complication
192	O6989X4	Umbilical cord complication
192	O6989X5	Umbilical cord complication
192	O6989X9	Umbilical cord complication
192	O699XX0	Umbilical cord complication
192	O699XX1	Umbilical cord complication
192	O699XX2	Umbilical cord complication
192	O699XX3	Umbilical cord complication
192	O699XX4	Umbilical cord complication
192	O699XX5	Umbilical cord complication
192	O699XX9	Umbilical cord complication
193	O700	OB-related trauma to perineum and vulva
193	O701	OB-related trauma to perineum and vulva
193	O702	OB-related trauma to perineum and vulva

193	O703	OB-related trauma to perineum and vulva
193	O704	OB-related trauma to perineum and vulva
193	O709	OB-related trauma to perineum and vulva
193	O7182	OB-related trauma to perineum and vulva
194	O665	Forceps delivery
195	A34	Other complications of birth; puerperium affecting management of mother
195	O252	Other complications of birth; puerperium affecting management of mother
195	O253	Other complications of birth; puerperium affecting management of mother
195	O2662	Other complications of birth; puerperium affecting management of mother
195	O2663	Other complications of birth; puerperium affecting management of mother
195	O2672	Other complications of birth; puerperium affecting management of mother
195	O2673	Other complications of birth; puerperium affecting management of mother
195	O354XX0	Other complications of birth; puerperium affecting management of mother
195	O354XX1	Other complications of birth; puerperium affecting management of mother
195	O354XX2	Other complications of birth; puerperium affecting management of mother
195	O354XX3	Other complications of birth; puerperium affecting management of mother
195	O354XX4	Other complications of birth; puerperium affecting management of mother
195	O354XX5	Other complications of birth; puerperium affecting management of mother
195	O354XX9	Other complications of birth; puerperium affecting management of mother
195	O356XX0	Other complications of birth; puerperium affecting management of mother
195	O356XX1	Other complications of birth; puerperium affecting management of mother
195	O356XX2	Other complications of birth; puerperium affecting management of mother
195	O356XX3	Other complications of birth; puerperium affecting management of mother
195	O356XX4	Other complications of birth; puerperium affecting management of mother
195	O356XX5	Other complications of birth; puerperium affecting management of mother
195	O356XX9	Other complications of birth; puerperium affecting management of mother
195	O357XX0	Other complications of birth; puerperium affecting management of mother
195	O357XX1	Other complications of birth; puerperium affecting management of mother
195	O357XX2	Other complications of birth; puerperium affecting management of mother
195	O357XX3	Other complications of birth; puerperium affecting management of mother
195	O357XX4	Other complications of birth; puerperium affecting management of mother
195	O357XX5	Other complications of birth; puerperium affecting management of mother
195	O357XX9	Other complications of birth; puerperium affecting management of mother
195	O358XX0	Other complications of birth; puerperium affecting management of mother
195	O358XX1	Other complications of birth; puerperium affecting management of mother
195	O358XX2	Other complications of birth; puerperium affecting management of mother
195	O358XX3	Other complications of birth; puerperium affecting management of mother
195	O358XX4	Other complications of birth; puerperium affecting management of mother
195	O358XX5	Other complications of birth; puerperium affecting management of mother
195	O358XX9	Other complications of birth; puerperium affecting management of mother
195	O359XX0	Other complications of birth; puerperium affecting management of mother
195	O359XX1	Other complications of birth; puerperium affecting management of mother
195	O359XX2	Other complications of birth; puerperium affecting management of mother
195	O359XX3	Other complications of birth; puerperium affecting management of mother
195	O359XX4	Other complications of birth; puerperium affecting management of mother
195	O359XX5	Other complications of birth; puerperium affecting management of mother
195	O359XX9	Other complications of birth; puerperium affecting management of mother
195	O364XX0	Other complications of birth; puerperium affecting management of mother
195	O364XX1	Other complications of birth; puerperium affecting management of mother
195	O364XX2	Other complications of birth; puerperium affecting management of mother
195	O364XX3	Other complications of birth; puerperium affecting management of mother

195	O364XX4	Other complications of birth; puerperium affecting management of mother
195	O364XX5	Other complications of birth; puerperium affecting management of mother
195	O364XX9	Other complications of birth; puerperium affecting management of mother
195	O610	Other complications of birth; puerperium affecting management of mother
195	O611	Other complications of birth; puerperium affecting management of mother
195	O618	Other complications of birth; puerperium affecting management of mother
195	O619	Other complications of birth; puerperium affecting management of mother
195	O7100	Other complications of birth; puerperium affecting management of mother
195	O7102	Other complications of birth; puerperium affecting management of mother
195	O7103	Other complications of birth; puerperium affecting management of mother
195	O711	Other complications of birth; puerperium affecting management of mother
195	O712	Other complications of birth; puerperium affecting management of mother
195	O713	Other complications of birth; puerperium affecting management of mother
195	O714	Other complications of birth; puerperium affecting management of mother
195	O715	Other complications of birth; puerperium affecting management of mother
195	O716	Other complications of birth; puerperium affecting management of mother
195	O717	Other complications of birth; puerperium affecting management of mother
195	O7181	Other complications of birth; puerperium affecting management of mother
195	O7189	Other complications of birth; puerperium affecting management of mother
195	O719	Other complications of birth; puerperium affecting management of mother
195	O720	Other complications of birth; puerperium affecting management of mother
195	O721	Other complications of birth; puerperium affecting management of mother
195	O722	Other complications of birth; puerperium affecting management of mother
195	O723	Other complications of birth; puerperium affecting management of mother
195	O730	Other complications of birth; puerperium affecting management of mother
195	O731	Other complications of birth; puerperium affecting management of mother
195	O740	Other complications of birth; puerperium affecting management of mother
195	O741	Other complications of birth; puerperium affecting management of mother
195	O742	Other complications of birth; puerperium affecting management of mother
195	O743	Other complications of birth; puerperium affecting management of mother
195	O744	Other complications of birth; puerperium affecting management of mother
195	O745	Other complications of birth; puerperium affecting management of mother
195	O746	Other complications of birth; puerperium affecting management of mother
195	O747	Other complications of birth; puerperium affecting management of mother
195	O748	Other complications of birth; puerperium affecting management of mother
195	O749	Other complications of birth; puerperium affecting management of mother
195	O750	Other complications of birth; puerperium affecting management of mother
195	O751	Other complications of birth; puerperium affecting management of mother
195	O752	Other complications of birth; puerperium affecting management of mother
195	O753	Other complications of birth; puerperium affecting management of mother
195	O754	Other complications of birth; puerperium affecting management of mother
195	O755	Other complications of birth; puerperium affecting management of mother
195	O7581	Other complications of birth; puerperium affecting management of mother
195	O7582	Other complications of birth; puerperium affecting management of mother
195	O7589	Other complications of birth; puerperium affecting management of mother
195	O759	Other complications of birth; puerperium affecting management of mother
195	O76	Other complications of birth; puerperium affecting management of mother
195	O770	Other complications of birth; puerperium affecting management of mother
195	O82	Other complications of birth; puerperium affecting management of mother
195	O85	Other complications of birth; puerperium affecting management of mother
195	O860	Other complications of birth; puerperium affecting management of mother

195	O8611	Other complications of birth; puerperium affecting management of mother
195	O8612	Other complications of birth; puerperium affecting management of mother
195	O8613	Other complications of birth; puerperium affecting management of mother
195	O8619	Other complications of birth; puerperium affecting management of mother
195	O8620	Other complications of birth; puerperium affecting management of mother
195	O8621	Other complications of birth; puerperium affecting management of mother
195	O8622	Other complications of birth; puerperium affecting management of mother
195	O8629	Other complications of birth; puerperium affecting management of mother
195	O864	Other complications of birth; puerperium affecting management of mother
195	O8681	Other complications of birth; puerperium affecting management of mother
195	O8689	Other complications of birth; puerperium affecting management of mother
195	O870	Other complications of birth; puerperium affecting management of mother
195	O871	Other complications of birth; puerperium affecting management of mother
195	O872	Other complications of birth; puerperium affecting management of mother
195	O873	Other complications of birth; puerperium affecting management of mother
195	O874	Other complications of birth; puerperium affecting management of mother
195	O878	Other complications of birth; puerperium affecting management of mother
195	O879	Other complications of birth; puerperium affecting management of mother
195	O8802	Other complications of birth; puerperium affecting management of mother
195	O8803	Other complications of birth; puerperium affecting management of mother
195	O8812	Other complications of birth; puerperium affecting management of mother
195	O8813	Other complications of birth; puerperium affecting management of mother
195	O8822	Other complications of birth; puerperium affecting management of mother
195	O8823	Other complications of birth; puerperium affecting management of mother
195	O8832	Other complications of birth; puerperium affecting management of mother
195	O8833	Other complications of birth; puerperium affecting management of mother
195	O8882	Other complications of birth; puerperium affecting management of mother
195	O8883	Other complications of birth; puerperium affecting management of mother
195	O8901	Other complications of birth; puerperium affecting management of mother
195	O8909	Other complications of birth; puerperium affecting management of mother
195	O891	Other complications of birth; puerperium affecting management of mother
195	O892	Other complications of birth; puerperium affecting management of mother
195	O893	Other complications of birth; puerperium affecting management of mother
195	O894	Other complications of birth; puerperium affecting management of mother
195	O895	Other complications of birth; puerperium affecting management of mother
195	O896	Other complications of birth; puerperium affecting management of mother
195	O898	Other complications of birth; puerperium affecting management of mother
195	O899	Other complications of birth; puerperium affecting management of mother
195	O900	Other complications of birth; puerperium affecting management of mother
195	O901	Other complications of birth; puerperium affecting management of mother
195	O902	Other complications of birth; puerperium affecting management of mother
195	O903	Other complications of birth; puerperium affecting management of mother
195	O904	Other complications of birth; puerperium affecting management of mother
195	O905	Other complications of birth; puerperium affecting management of mother
195	O9081	Other complications of birth; puerperium affecting management of mother
195	O9089	Other complications of birth; puerperium affecting management of mother
195	O909	Other complications of birth; puerperium affecting management of mother
195	O9102	Other complications of birth; puerperium affecting management of mother
195	O9103	Other complications of birth; puerperium affecting management of mother
195	O9112	Other complications of birth; puerperium affecting management of mother
195	O9113	Other complications of birth; puerperium affecting management of mother

195	O9122	Other complications of birth; puerperium affecting management of mother
195	O9123	Other complications of birth; puerperium affecting management of mother
195	O9202	Other complications of birth; puerperium affecting management of mother
195	O9203	Other complications of birth; puerperium affecting management of mother
195	O9212	Other complications of birth; puerperium affecting management of mother
195	O9213	Other complications of birth; puerperium affecting management of mother
195	O9220	Other complications of birth; puerperium affecting management of mother
195	O9229	Other complications of birth; puerperium affecting management of mother
195	O923	Other complications of birth; puerperium affecting management of mother
195	O924	Other complications of birth; puerperium affecting management of mother
195	O925	Other complications of birth; puerperium affecting management of mother
195	O926	Other complications of birth; puerperium affecting management of mother
195	O9270	Other complications of birth; puerperium affecting management of mother
195	O9279	Other complications of birth; puerperium affecting management of mother
195	O94	Other complications of birth; puerperium affecting management of mother
195	O9802	Other complications of birth; puerperium affecting management of mother
195	O9803	Other complications of birth; puerperium affecting management of mother
195	O9812	Other complications of birth; puerperium affecting management of mother
195	O9813	Other complications of birth; puerperium affecting management of mother
195	O9822	Other complications of birth; puerperium affecting management of mother
195	O9823	Other complications of birth; puerperium affecting management of mother
195	O9832	Other complications of birth; puerperium affecting management of mother
195	O9833	Other complications of birth; puerperium affecting management of mother
195	O9842	Other complications of birth; puerperium affecting management of mother
195	O9843	Other complications of birth; puerperium affecting management of mother
195	O9852	Other complications of birth; puerperium affecting management of mother
195	O9853	Other complications of birth; puerperium affecting management of mother
195	O9862	Other complications of birth; puerperium affecting management of mother
195	O9863	Other complications of birth; puerperium affecting management of mother
195	O9872	Other complications of birth; puerperium affecting management of mother
195	O9873	Other complications of birth; puerperium affecting management of mother
195	O9882	Other complications of birth; puerperium affecting management of mother
195	O9883	Other complications of birth; puerperium affecting management of mother
195	O9892	Other complications of birth; puerperium affecting management of mother
195	O9893	Other complications of birth; puerperium affecting management of mother
195	O9902	Other complications of birth; puerperium affecting management of mother
195	O9903	Other complications of birth; puerperium affecting management of mother
195	O9912	Other complications of birth; puerperium affecting management of mother
195	O9913	Other complications of birth; puerperium affecting management of mother
195	O99214	Other complications of birth; puerperium affecting management of mother
195	O99215	Other complications of birth; puerperium affecting management of mother
195	O99284	Other complications of birth; puerperium affecting management of mother
195	O99285	Other complications of birth; puerperium affecting management of mother
195	O99334	Other complications of birth; puerperium affecting management of mother
195	O99335	Other complications of birth; puerperium affecting management of mother
195	O99344	Other complications of birth; puerperium affecting management of mother
195	O99354	Other complications of birth; puerperium affecting management of mother
195	O99355	Other complications of birth; puerperium affecting management of mother
195	O9942	Other complications of birth; puerperium affecting management of mother
195	O9943	Other complications of birth; puerperium affecting management of mother
195	O9952	Other complications of birth; puerperium affecting management of mother

195	O9953	Other complications of birth; puerperium affecting management of mother
195	O9962	Other complications of birth; puerperium affecting management of mother
195	O9963	Other complications of birth; puerperium affecting management of mother
195	O9972	Other complications of birth; puerperium affecting management of mother
195	O9973	Other complications of birth; puerperium affecting management of mother
195	O99824	Other complications of birth; puerperium affecting management of mother
195	O99825	Other complications of birth; puerperium affecting management of mother
195	O99834	Other complications of birth; puerperium affecting management of mother
195	O99835	Other complications of birth; puerperium affecting management of mother
195	O99844	Other complications of birth; puerperium affecting management of mother
195	O99845	Other complications of birth; puerperium affecting management of mother
195	O9A12	Other complications of birth; puerperium affecting management of mother
195	O9A13	Other complications of birth; puerperium affecting management of mother
195	O9A22	Other complications of birth; puerperium affecting management of mother
195	O9A23	Other complications of birth; puerperium affecting management of mother
195	O9A32	Other complications of birth; puerperium affecting management of mother
195	O9A33	Other complications of birth; puerperium affecting management of mother
195	O9A42	Other complications of birth; puerperium affecting management of mother
195	O9A43	Other complications of birth; puerperium affecting management of mother
195	O9A52	Other complications of birth; puerperium affecting management of mother
195	O9A53	Other complications of birth; puerperium affecting management of mother
196	O30001	Normal pregnancy and/or delivery
196	O30002	Normal pregnancy and/or delivery
196	O30003	Normal pregnancy and/or delivery
196	O30009	Normal pregnancy and/or delivery
196	O30011	Normal pregnancy and/or delivery
196	O30012	Normal pregnancy and/or delivery
196	O30013	Normal pregnancy and/or delivery
196	O30019	Normal pregnancy and/or delivery
196	O30031	Normal pregnancy and/or delivery
196	O30032	Normal pregnancy and/or delivery
196	O30033	Normal pregnancy and/or delivery
196	O30039	Normal pregnancy and/or delivery
196	O30041	Normal pregnancy and/or delivery
196	O30042	Normal pregnancy and/or delivery
196	O30043	Normal pregnancy and/or delivery
196	O30049	Normal pregnancy and/or delivery
196	O30091	Normal pregnancy and/or delivery
196	O30092	Normal pregnancy and/or delivery
196	O30093	Normal pregnancy and/or delivery
196	O30099	Normal pregnancy and/or delivery
196	O30101	Normal pregnancy and/or delivery
196	O30102	Normal pregnancy and/or delivery
196	O30103	Normal pregnancy and/or delivery
196	O30109	Normal pregnancy and/or delivery
196	O30111	Normal pregnancy and/or delivery
196	O30112	Normal pregnancy and/or delivery
196	O30113	Normal pregnancy and/or delivery
196	O30119	Normal pregnancy and/or delivery
196	O30121	Normal pregnancy and/or delivery
196	O30122	Normal pregnancy and/or delivery

196	O30123	Normal pregnancy and/or delivery
196	O30129	Normal pregnancy and/or delivery
196	O30191	Normal pregnancy and/or delivery
196	O30192	Normal pregnancy and/or delivery
196	O30193	Normal pregnancy and/or delivery
196	O30199	Normal pregnancy and/or delivery
196	O30201	Normal pregnancy and/or delivery
196	O30202	Normal pregnancy and/or delivery
196	O30203	Normal pregnancy and/or delivery
196	O30209	Normal pregnancy and/or delivery
196	O30211	Normal pregnancy and/or delivery
196	O30212	Normal pregnancy and/or delivery
196	O30213	Normal pregnancy and/or delivery
196	O30219	Normal pregnancy and/or delivery
196	O30221	Normal pregnancy and/or delivery
196	O30222	Normal pregnancy and/or delivery
196	O30223	Normal pregnancy and/or delivery
196	O30229	Normal pregnancy and/or delivery
196	O30291	Normal pregnancy and/or delivery
196	O30292	Normal pregnancy and/or delivery
196	O30293	Normal pregnancy and/or delivery
196	O30299	Normal pregnancy and/or delivery
196	O30801	Normal pregnancy and/or delivery
196	O30802	Normal pregnancy and/or delivery
196	O30803	Normal pregnancy and/or delivery
196	O30809	Normal pregnancy and/or delivery
196	O30811	Normal pregnancy and/or delivery
196	O30812	Normal pregnancy and/or delivery
196	O30813	Normal pregnancy and/or delivery
196	O30819	Normal pregnancy and/or delivery
196	O30821	Normal pregnancy and/or delivery
196	O30822	Normal pregnancy and/or delivery
196	O30823	Normal pregnancy and/or delivery
196	O30829	Normal pregnancy and/or delivery
196	O30891	Normal pregnancy and/or delivery
196	O30892	Normal pregnancy and/or delivery
196	O30893	Normal pregnancy and/or delivery
196	O30899	Normal pregnancy and/or delivery
196	O3090	Normal pregnancy and/or delivery
196	O3091	Normal pregnancy and/or delivery
196	O3092	Normal pregnancy and/or delivery
196	O3093	Normal pregnancy and/or delivery
196	O3130X0	Normal pregnancy and/or delivery
196	O3130X1	Normal pregnancy and/or delivery
196	O3130X2	Normal pregnancy and/or delivery
196	O3130X3	Normal pregnancy and/or delivery
196	O3130X4	Normal pregnancy and/or delivery
196	O3130X5	Normal pregnancy and/or delivery
196	O3130X9	Normal pregnancy and/or delivery
196	O3131X0	Normal pregnancy and/or delivery
196	O3131X1	Normal pregnancy and/or delivery

196	O3131X2	Normal pregnancy and/or delivery
196	O3131X3	Normal pregnancy and/or delivery
196	O3131X4	Normal pregnancy and/or delivery
196	O3131X5	Normal pregnancy and/or delivery
196	O3131X9	Normal pregnancy and/or delivery
196	O3132X0	Normal pregnancy and/or delivery
196	O3132X1	Normal pregnancy and/or delivery
196	O3132X2	Normal pregnancy and/or delivery
196	O3132X3	Normal pregnancy and/or delivery
196	O3132X4	Normal pregnancy and/or delivery
196	O3132X5	Normal pregnancy and/or delivery
196	O3132X9	Normal pregnancy and/or delivery
196	O3133X0	Normal pregnancy and/or delivery
196	O3133X1	Normal pregnancy and/or delivery
196	O3133X2	Normal pregnancy and/or delivery
196	O3133X3	Normal pregnancy and/or delivery
196	O3133X4	Normal pregnancy and/or delivery
196	O3133X5	Normal pregnancy and/or delivery
196	O3133X9	Normal pregnancy and/or delivery
196	O80	Normal pregnancy and/or delivery
196	Z3201	Normal pregnancy and/or delivery
196	Z331	Normal pregnancy and/or delivery
196	Z3400	Normal pregnancy and/or delivery
196	Z3401	Normal pregnancy and/or delivery
196	Z3402	Normal pregnancy and/or delivery
196	Z3403	Normal pregnancy and/or delivery
196	Z3480	Normal pregnancy and/or delivery
196	Z3481	Normal pregnancy and/or delivery
196	Z3482	Normal pregnancy and/or delivery
196	Z3483	Normal pregnancy and/or delivery
196	Z3490	Normal pregnancy and/or delivery
196	Z3491	Normal pregnancy and/or delivery
196	Z3492	Normal pregnancy and/or delivery
196	Z3493	Normal pregnancy and/or delivery
196	Z370	Normal pregnancy and/or delivery
196	Z390	Normal pregnancy and/or delivery
196	Z391	Normal pregnancy and/or delivery
196	Z392	Normal pregnancy and/or delivery
197	A201	Skin and subcutaneous tissue infections
197	A210	Skin and subcutaneous tissue infections
197	A220	Skin and subcutaneous tissue infections
197	A311	Skin and subcutaneous tissue infections
197	A363	Skin and subcutaneous tissue infections
197	A431	Skin and subcutaneous tissue infections
197	A46	Skin and subcutaneous tissue infections
197	L0100	Skin and subcutaneous tissue infections
197	L0101	Skin and subcutaneous tissue infections
197	L0102	Skin and subcutaneous tissue infections
197	L0103	Skin and subcutaneous tissue infections
197	L0109	Skin and subcutaneous tissue infections
197	L011	Skin and subcutaneous tissue infections

197	L0201	Skin and subcutaneous tissue infections
197	L0202	Skin and subcutaneous tissue infections
197	L0203	Skin and subcutaneous tissue infections
197	L0211	Skin and subcutaneous tissue infections
197	L0212	Skin and subcutaneous tissue infections
197	L0213	Skin and subcutaneous tissue infections
197	L02211	Skin and subcutaneous tissue infections
197	L02212	Skin and subcutaneous tissue infections
197	L02213	Skin and subcutaneous tissue infections
197	L02214	Skin and subcutaneous tissue infections
197	L02215	Skin and subcutaneous tissue infections
197	L02216	Skin and subcutaneous tissue infections
197	L02219	Skin and subcutaneous tissue infections
197	L02221	Skin and subcutaneous tissue infections
197	L02222	Skin and subcutaneous tissue infections
197	L02223	Skin and subcutaneous tissue infections
197	L02224	Skin and subcutaneous tissue infections
197	L02225	Skin and subcutaneous tissue infections
197	L02226	Skin and subcutaneous tissue infections
197	L02229	Skin and subcutaneous tissue infections
197	L02231	Skin and subcutaneous tissue infections
197	L02232	Skin and subcutaneous tissue infections
197	L02233	Skin and subcutaneous tissue infections
197	L02234	Skin and subcutaneous tissue infections
197	L02235	Skin and subcutaneous tissue infections
197	L02236	Skin and subcutaneous tissue infections
197	L02239	Skin and subcutaneous tissue infections
197	L0231	Skin and subcutaneous tissue infections
197	L0232	Skin and subcutaneous tissue infections
197	L0233	Skin and subcutaneous tissue infections
197	L02411	Skin and subcutaneous tissue infections
197	L02412	Skin and subcutaneous tissue infections
197	L02413	Skin and subcutaneous tissue infections
197	L02414	Skin and subcutaneous tissue infections
197	L02415	Skin and subcutaneous tissue infections
197	L02416	Skin and subcutaneous tissue infections
197	L02419	Skin and subcutaneous tissue infections
197	L02421	Skin and subcutaneous tissue infections
197	L02422	Skin and subcutaneous tissue infections
197	L02423	Skin and subcutaneous tissue infections
197	L02424	Skin and subcutaneous tissue infections
197	L02425	Skin and subcutaneous tissue infections
197	L02426	Skin and subcutaneous tissue infections
197	L02429	Skin and subcutaneous tissue infections
197	L02431	Skin and subcutaneous tissue infections
197	L02432	Skin and subcutaneous tissue infections
197	L02433	Skin and subcutaneous tissue infections
197	L02434	Skin and subcutaneous tissue infections
197	L02435	Skin and subcutaneous tissue infections
197	L02436	Skin and subcutaneous tissue infections
197	L02439	Skin and subcutaneous tissue infections

197	L02511	Skin and subcutaneous tissue infections
197	L02512	Skin and subcutaneous tissue infections
197	L02519	Skin and subcutaneous tissue infections
197	L02521	Skin and subcutaneous tissue infections
197	L02522	Skin and subcutaneous tissue infections
197	L02529	Skin and subcutaneous tissue infections
197	L02531	Skin and subcutaneous tissue infections
197	L02532	Skin and subcutaneous tissue infections
197	L02539	Skin and subcutaneous tissue infections
197	L02611	Skin and subcutaneous tissue infections
197	L02612	Skin and subcutaneous tissue infections
197	L02619	Skin and subcutaneous tissue infections
197	L02621	Skin and subcutaneous tissue infections
197	L02622	Skin and subcutaneous tissue infections
197	L02629	Skin and subcutaneous tissue infections
197	L02631	Skin and subcutaneous tissue infections
197	L02632	Skin and subcutaneous tissue infections
197	L02639	Skin and subcutaneous tissue infections
197	L02811	Skin and subcutaneous tissue infections
197	L02818	Skin and subcutaneous tissue infections
197	L02821	Skin and subcutaneous tissue infections
197	L02828	Skin and subcutaneous tissue infections
197	L02831	Skin and subcutaneous tissue infections
197	L02838	Skin and subcutaneous tissue infections
197	L0291	Skin and subcutaneous tissue infections
197	L0292	Skin and subcutaneous tissue infections
197	L0293	Skin and subcutaneous tissue infections
197	L03011	Skin and subcutaneous tissue infections
197	L03012	Skin and subcutaneous tissue infections
197	L03019	Skin and subcutaneous tissue infections
197	L03021	Skin and subcutaneous tissue infections
197	L03022	Skin and subcutaneous tissue infections
197	L03029	Skin and subcutaneous tissue infections
197	L03031	Skin and subcutaneous tissue infections
197	L03032	Skin and subcutaneous tissue infections
197	L03039	Skin and subcutaneous tissue infections
197	L03041	Skin and subcutaneous tissue infections
197	L03042	Skin and subcutaneous tissue infections
197	L03049	Skin and subcutaneous tissue infections
197	L03111	Skin and subcutaneous tissue infections
197	L03112	Skin and subcutaneous tissue infections
197	L03113	Skin and subcutaneous tissue infections
197	L03114	Skin and subcutaneous tissue infections
197	L03115	Skin and subcutaneous tissue infections
197	L03116	Skin and subcutaneous tissue infections
197	L03119	Skin and subcutaneous tissue infections
197	L03121	Skin and subcutaneous tissue infections
197	L03122	Skin and subcutaneous tissue infections
197	L03123	Skin and subcutaneous tissue infections
197	L03124	Skin and subcutaneous tissue infections
197	L03125	Skin and subcutaneous tissue infections

197	L03126	Skin and subcutaneous tissue infections
197	L03129	Skin and subcutaneous tissue infections
197	L03211	Skin and subcutaneous tissue infections
197	L03212	Skin and subcutaneous tissue infections
197	L03221	Skin and subcutaneous tissue infections
197	L03222	Skin and subcutaneous tissue infections
197	L03311	Skin and subcutaneous tissue infections
197	L03312	Skin and subcutaneous tissue infections
197	L03313	Skin and subcutaneous tissue infections
197	L03314	Skin and subcutaneous tissue infections
197	L03315	Skin and subcutaneous tissue infections
197	L03316	Skin and subcutaneous tissue infections
197	L03317	Skin and subcutaneous tissue infections
197	L03319	Skin and subcutaneous tissue infections
197	L03321	Skin and subcutaneous tissue infections
197	L03322	Skin and subcutaneous tissue infections
197	L03323	Skin and subcutaneous tissue infections
197	L03324	Skin and subcutaneous tissue infections
197	L03325	Skin and subcutaneous tissue infections
197	L03326	Skin and subcutaneous tissue infections
197	L03327	Skin and subcutaneous tissue infections
197	L03329	Skin and subcutaneous tissue infections
197	L03811	Skin and subcutaneous tissue infections
197	L03818	Skin and subcutaneous tissue infections
197	L03891	Skin and subcutaneous tissue infections
197	L03898	Skin and subcutaneous tissue infections
197	L0390	Skin and subcutaneous tissue infections
197	L0391	Skin and subcutaneous tissue infections
197	L0501	Skin and subcutaneous tissue infections
197	L0502	Skin and subcutaneous tissue infections
197	L0591	Skin and subcutaneous tissue infections
197	L0592	Skin and subcutaneous tissue infections
197	L080	Skin and subcutaneous tissue infections
197	L081	Skin and subcutaneous tissue infections
197	L0881	Skin and subcutaneous tissue infections
197	L0882	Skin and subcutaneous tissue infections
197	L0889	Skin and subcutaneous tissue infections
197	L089	Skin and subcutaneous tissue infections
197	L88	Skin and subcutaneous tissue infections
197	L983	Skin and subcutaneous tissue infections
198	B653	Other inflammatory condition of skin
198	L00	Other inflammatory condition of skin
198	L100	Other inflammatory condition of skin
198	L101	Other inflammatory condition of skin
198	L102	Other inflammatory condition of skin
198	L103	Other inflammatory condition of skin
198	L104	Other inflammatory condition of skin
198	L105	Other inflammatory condition of skin
198	L1081	Other inflammatory condition of skin
198	L1089	Other inflammatory condition of skin
198	L109	Other inflammatory condition of skin

198	L110	Other inflammatory condition of skin
198	L111	Other inflammatory condition of skin
198	L118	Other inflammatory condition of skin
198	L119	Other inflammatory condition of skin
198	L120	Other inflammatory condition of skin
198	L121	Other inflammatory condition of skin
198	L122	Other inflammatory condition of skin
198	L1230	Other inflammatory condition of skin
198	L1231	Other inflammatory condition of skin
198	L1235	Other inflammatory condition of skin
198	L128	Other inflammatory condition of skin
198	L129	Other inflammatory condition of skin
198	L130	Other inflammatory condition of skin
198	L131	Other inflammatory condition of skin
198	L138	Other inflammatory condition of skin
198	L139	Other inflammatory condition of skin
198	L14	Other inflammatory condition of skin
198	L210	Other inflammatory condition of skin
198	L211	Other inflammatory condition of skin
198	L218	Other inflammatory condition of skin
198	L219	Other inflammatory condition of skin
198	L26	Other inflammatory condition of skin
198	L280	Other inflammatory condition of skin
198	L281	Other inflammatory condition of skin
198	L282	Other inflammatory condition of skin
198	L290	Other inflammatory condition of skin
198	L291	Other inflammatory condition of skin
198	L292	Other inflammatory condition of skin
198	L293	Other inflammatory condition of skin
198	L298	Other inflammatory condition of skin
198	L299	Other inflammatory condition of skin
198	L303	Other inflammatory condition of skin
198	L304	Other inflammatory condition of skin
198	L305	Other inflammatory condition of skin
198	L400	Other inflammatory condition of skin
198	L401	Other inflammatory condition of skin
198	L402	Other inflammatory condition of skin
198	L403	Other inflammatory condition of skin
198	L404	Other inflammatory condition of skin
198	L4050	Other inflammatory condition of skin
198	L4051	Other inflammatory condition of skin
198	L4052	Other inflammatory condition of skin
198	L4053	Other inflammatory condition of skin
198	L4054	Other inflammatory condition of skin
198	L4059	Other inflammatory condition of skin
198	L408	Other inflammatory condition of skin
198	L409	Other inflammatory condition of skin
198	L410	Other inflammatory condition of skin
198	L411	Other inflammatory condition of skin
198	L413	Other inflammatory condition of skin
198	L414	Other inflammatory condition of skin

198	L415	Other inflammatory condition of skin
198	L418	Other inflammatory condition of skin
198	L419	Other inflammatory condition of skin
198	L42	Other inflammatory condition of skin
198	L430	Other inflammatory condition of skin
198	L431	Other inflammatory condition of skin
198	L432	Other inflammatory condition of skin
198	L433	Other inflammatory condition of skin
198	L438	Other inflammatory condition of skin
198	L439	Other inflammatory condition of skin
198	L440	Other inflammatory condition of skin
198	L441	Other inflammatory condition of skin
198	L442	Other inflammatory condition of skin
198	L443	Other inflammatory condition of skin
198	L444	Other inflammatory condition of skin
198	L448	Other inflammatory condition of skin
198	L449	Other inflammatory condition of skin
198	L45	Other inflammatory condition of skin
198	L490	Other inflammatory condition of skin
198	L491	Other inflammatory condition of skin
198	L492	Other inflammatory condition of skin
198	L493	Other inflammatory condition of skin
198	L494	Other inflammatory condition of skin
198	L495	Other inflammatory condition of skin
198	L496	Other inflammatory condition of skin
198	L497	Other inflammatory condition of skin
198	L498	Other inflammatory condition of skin
198	L499	Other inflammatory condition of skin
198	L510	Other inflammatory condition of skin
198	L511	Other inflammatory condition of skin
198	L512	Other inflammatory condition of skin
198	L513	Other inflammatory condition of skin
198	L518	Other inflammatory condition of skin
198	L519	Other inflammatory condition of skin
198	L52	Other inflammatory condition of skin
198	L530	Other inflammatory condition of skin
198	L531	Other inflammatory condition of skin
198	L532	Other inflammatory condition of skin
198	L533	Other inflammatory condition of skin
198	L538	Other inflammatory condition of skin
198	L539	Other inflammatory condition of skin
198	L54	Other inflammatory condition of skin
198	L550	Other inflammatory condition of skin
198	L551	Other inflammatory condition of skin
198	L552	Other inflammatory condition of skin
198	L559	Other inflammatory condition of skin
198	L661	Other inflammatory condition of skin
198	L710	Other inflammatory condition of skin
198	L711	Other inflammatory condition of skin
198	L718	Other inflammatory condition of skin
198	L719	Other inflammatory condition of skin

198	L920	Other inflammatory condition of skin
198	L930	Other inflammatory condition of skin
198	L931	Other inflammatory condition of skin
198	L932	Other inflammatory condition of skin
198	L950	Other inflammatory condition of skin
198	L951	Other inflammatory condition of skin
198	L958	Other inflammatory condition of skin
198	L959	Other inflammatory condition of skin
198	L981	Other inflammatory condition of skin
198	L982	Other inflammatory condition of skin
199	L89000	Chronic ulcer of skin
199	L89001	Chronic ulcer of skin
199	L89002	Chronic ulcer of skin
199	L89003	Chronic ulcer of skin
199	L89004	Chronic ulcer of skin
199	L89009	Chronic ulcer of skin
199	L89010	Chronic ulcer of skin
199	L89011	Chronic ulcer of skin
199	L89012	Chronic ulcer of skin
199	L89013	Chronic ulcer of skin
199	L89014	Chronic ulcer of skin
199	L89019	Chronic ulcer of skin
199	L89020	Chronic ulcer of skin
199	L89021	Chronic ulcer of skin
199	L89022	Chronic ulcer of skin
199	L89023	Chronic ulcer of skin
199	L89024	Chronic ulcer of skin
199	L89029	Chronic ulcer of skin
199	L89100	Chronic ulcer of skin
199	L89101	Chronic ulcer of skin
199	L89102	Chronic ulcer of skin
199	L89103	Chronic ulcer of skin
199	L89104	Chronic ulcer of skin
199	L89109	Chronic ulcer of skin
199	L89110	Chronic ulcer of skin
199	L89111	Chronic ulcer of skin
199	L89112	Chronic ulcer of skin
199	L89113	Chronic ulcer of skin
199	L89114	Chronic ulcer of skin
199	L89119	Chronic ulcer of skin
199	L89120	Chronic ulcer of skin
199	L89121	Chronic ulcer of skin
199	L89122	Chronic ulcer of skin
199	L89123	Chronic ulcer of skin
199	L89124	Chronic ulcer of skin
199	L89129	Chronic ulcer of skin
199	L89130	Chronic ulcer of skin
199	L89131	Chronic ulcer of skin
199	L89132	Chronic ulcer of skin
199	L89133	Chronic ulcer of skin
199	L89134	Chronic ulcer of skin

199	L89139	Chronic ulcer of skin
199	L89140	Chronic ulcer of skin
199	L89141	Chronic ulcer of skin
199	L89142	Chronic ulcer of skin
199	L89143	Chronic ulcer of skin
199	L89144	Chronic ulcer of skin
199	L89149	Chronic ulcer of skin
199	L89150	Chronic ulcer of skin
199	L89151	Chronic ulcer of skin
199	L89152	Chronic ulcer of skin
199	L89153	Chronic ulcer of skin
199	L89154	Chronic ulcer of skin
199	L89159	Chronic ulcer of skin
199	L89200	Chronic ulcer of skin
199	L89201	Chronic ulcer of skin
199	L89202	Chronic ulcer of skin
199	L89203	Chronic ulcer of skin
199	L89204	Chronic ulcer of skin
199	L89209	Chronic ulcer of skin
199	L89210	Chronic ulcer of skin
199	L89211	Chronic ulcer of skin
199	L89212	Chronic ulcer of skin
199	L89213	Chronic ulcer of skin
199	L89214	Chronic ulcer of skin
199	L89219	Chronic ulcer of skin
199	L89220	Chronic ulcer of skin
199	L89221	Chronic ulcer of skin
199	L89222	Chronic ulcer of skin
199	L89223	Chronic ulcer of skin
199	L89224	Chronic ulcer of skin
199	L89229	Chronic ulcer of skin
199	L89300	Chronic ulcer of skin
199	L89301	Chronic ulcer of skin
199	L89302	Chronic ulcer of skin
199	L89303	Chronic ulcer of skin
199	L89304	Chronic ulcer of skin
199	L89309	Chronic ulcer of skin
199	L89310	Chronic ulcer of skin
199	L89311	Chronic ulcer of skin
199	L89312	Chronic ulcer of skin
199	L89313	Chronic ulcer of skin
199	L89314	Chronic ulcer of skin
199	L89319	Chronic ulcer of skin
199	L89320	Chronic ulcer of skin
199	L89321	Chronic ulcer of skin
199	L89322	Chronic ulcer of skin
199	L89323	Chronic ulcer of skin
199	L89324	Chronic ulcer of skin
199	L89329	Chronic ulcer of skin
199	L8940	Chronic ulcer of skin
199	L8941	Chronic ulcer of skin

199	L8942	Chronic ulcer of skin
199	L8943	Chronic ulcer of skin
199	L8944	Chronic ulcer of skin
199	L8945	Chronic ulcer of skin
199	L89500	Chronic ulcer of skin
199	L89501	Chronic ulcer of skin
199	L89502	Chronic ulcer of skin
199	L89503	Chronic ulcer of skin
199	L89504	Chronic ulcer of skin
199	L89509	Chronic ulcer of skin
199	L89510	Chronic ulcer of skin
199	L89511	Chronic ulcer of skin
199	L89512	Chronic ulcer of skin
199	L89513	Chronic ulcer of skin
199	L89514	Chronic ulcer of skin
199	L89519	Chronic ulcer of skin
199	L89520	Chronic ulcer of skin
199	L89521	Chronic ulcer of skin
199	L89522	Chronic ulcer of skin
199	L89523	Chronic ulcer of skin
199	L89524	Chronic ulcer of skin
199	L89529	Chronic ulcer of skin
199	L89600	Chronic ulcer of skin
199	L89601	Chronic ulcer of skin
199	L89602	Chronic ulcer of skin
199	L89603	Chronic ulcer of skin
199	L89604	Chronic ulcer of skin
199	L89609	Chronic ulcer of skin
199	L89610	Chronic ulcer of skin
199	L89611	Chronic ulcer of skin
199	L89612	Chronic ulcer of skin
199	L89613	Chronic ulcer of skin
199	L89614	Chronic ulcer of skin
199	L89619	Chronic ulcer of skin
199	L89620	Chronic ulcer of skin
199	L89621	Chronic ulcer of skin
199	L89622	Chronic ulcer of skin
199	L89623	Chronic ulcer of skin
199	L89624	Chronic ulcer of skin
199	L89629	Chronic ulcer of skin
199	L89810	Chronic ulcer of skin
199	L89811	Chronic ulcer of skin
199	L89812	Chronic ulcer of skin
199	L89813	Chronic ulcer of skin
199	L89814	Chronic ulcer of skin
199	L89819	Chronic ulcer of skin
199	L89890	Chronic ulcer of skin
199	L89891	Chronic ulcer of skin
199	L89892	Chronic ulcer of skin
199	L89893	Chronic ulcer of skin
199	L89894	Chronic ulcer of skin

199	L89899	Chronic ulcer of skin
199	L8990	Chronic ulcer of skin
199	L8991	Chronic ulcer of skin
199	L8992	Chronic ulcer of skin
199	L8993	Chronic ulcer of skin
199	L8994	Chronic ulcer of skin
199	L8995	Chronic ulcer of skin
199	L97101	Chronic ulcer of skin
199	L97102	Chronic ulcer of skin
199	L97103	Chronic ulcer of skin
199	L97104	Chronic ulcer of skin
199	L97109	Chronic ulcer of skin
199	L97111	Chronic ulcer of skin
199	L97112	Chronic ulcer of skin
199	L97113	Chronic ulcer of skin
199	L97114	Chronic ulcer of skin
199	L97119	Chronic ulcer of skin
199	L97121	Chronic ulcer of skin
199	L97122	Chronic ulcer of skin
199	L97123	Chronic ulcer of skin
199	L97124	Chronic ulcer of skin
199	L97129	Chronic ulcer of skin
199	L97201	Chronic ulcer of skin
199	L97202	Chronic ulcer of skin
199	L97203	Chronic ulcer of skin
199	L97204	Chronic ulcer of skin
199	L97209	Chronic ulcer of skin
199	L97211	Chronic ulcer of skin
199	L97212	Chronic ulcer of skin
199	L97213	Chronic ulcer of skin
199	L97214	Chronic ulcer of skin
199	L97219	Chronic ulcer of skin
199	L97221	Chronic ulcer of skin
199	L97222	Chronic ulcer of skin
199	L97223	Chronic ulcer of skin
199	L97224	Chronic ulcer of skin
199	L97229	Chronic ulcer of skin
199	L97301	Chronic ulcer of skin
199	L97302	Chronic ulcer of skin
199	L97303	Chronic ulcer of skin
199	L97304	Chronic ulcer of skin
199	L97309	Chronic ulcer of skin
199	L97311	Chronic ulcer of skin
199	L97312	Chronic ulcer of skin
199	L97313	Chronic ulcer of skin
199	L97314	Chronic ulcer of skin
199	L97319	Chronic ulcer of skin
199	L97321	Chronic ulcer of skin
199	L97322	Chronic ulcer of skin
199	L97323	Chronic ulcer of skin
199	L97324	Chronic ulcer of skin

199	L97329	Chronic ulcer of skin
199	L97401	Chronic ulcer of skin
199	L97402	Chronic ulcer of skin
199	L97403	Chronic ulcer of skin
199	L97404	Chronic ulcer of skin
199	L97409	Chronic ulcer of skin
199	L97411	Chronic ulcer of skin
199	L97412	Chronic ulcer of skin
199	L97413	Chronic ulcer of skin
199	L97414	Chronic ulcer of skin
199	L97419	Chronic ulcer of skin
199	L97421	Chronic ulcer of skin
199	L97422	Chronic ulcer of skin
199	L97423	Chronic ulcer of skin
199	L97424	Chronic ulcer of skin
199	L97429	Chronic ulcer of skin
199	L97501	Chronic ulcer of skin
199	L97502	Chronic ulcer of skin
199	L97503	Chronic ulcer of skin
199	L97504	Chronic ulcer of skin
199	L97509	Chronic ulcer of skin
199	L97511	Chronic ulcer of skin
199	L97512	Chronic ulcer of skin
199	L97513	Chronic ulcer of skin
199	L97514	Chronic ulcer of skin
199	L97519	Chronic ulcer of skin
199	L97521	Chronic ulcer of skin
199	L97522	Chronic ulcer of skin
199	L97523	Chronic ulcer of skin
199	L97524	Chronic ulcer of skin
199	L97529	Chronic ulcer of skin
199	L97801	Chronic ulcer of skin
199	L97802	Chronic ulcer of skin
199	L97803	Chronic ulcer of skin
199	L97804	Chronic ulcer of skin
199	L97809	Chronic ulcer of skin
199	L97811	Chronic ulcer of skin
199	L97812	Chronic ulcer of skin
199	L97813	Chronic ulcer of skin
199	L97814	Chronic ulcer of skin
199	L97819	Chronic ulcer of skin
199	L97821	Chronic ulcer of skin
199	L97822	Chronic ulcer of skin
199	L97823	Chronic ulcer of skin
199	L97824	Chronic ulcer of skin
199	L97829	Chronic ulcer of skin
199	L97901	Chronic ulcer of skin
199	L97902	Chronic ulcer of skin
199	L97903	Chronic ulcer of skin
199	L97904	Chronic ulcer of skin
199	L97909	Chronic ulcer of skin

199	L97911	Chronic ulcer of skin
199	L97912	Chronic ulcer of skin
199	L97913	Chronic ulcer of skin
199	L97914	Chronic ulcer of skin
199	L97919	Chronic ulcer of skin
199	L97921	Chronic ulcer of skin
199	L97922	Chronic ulcer of skin
199	L97923	Chronic ulcer of skin
199	L97924	Chronic ulcer of skin
199	L97929	Chronic ulcer of skin
199	L98411	Chronic ulcer of skin
199	L98412	Chronic ulcer of skin
199	L98413	Chronic ulcer of skin
199	L98414	Chronic ulcer of skin
199	L98419	Chronic ulcer of skin
199	L98421	Chronic ulcer of skin
199	L98422	Chronic ulcer of skin
199	L98423	Chronic ulcer of skin
199	L98424	Chronic ulcer of skin
199	L98429	Chronic ulcer of skin
199	L98491	Chronic ulcer of skin
199	L98492	Chronic ulcer of skin
199	L98493	Chronic ulcer of skin
199	L98494	Chronic ulcer of skin
199	L98499	Chronic ulcer of skin
200	L301	Other skin disorders
200	L565	Other skin disorders
200	L570	Other skin disorders
200	L571	Other skin disorders
200	L572	Other skin disorders
200	L573	Other skin disorders
200	L574	Other skin disorders
200	L575	Other skin disorders
200	L600	Other skin disorders
200	L601	Other skin disorders
200	L602	Other skin disorders
200	L603	Other skin disorders
200	L604	Other skin disorders
200	L605	Other skin disorders
200	L608	Other skin disorders
200	L609	Other skin disorders
200	L62	Other skin disorders
200	L630	Other skin disorders
200	L631	Other skin disorders
200	L632	Other skin disorders
200	L638	Other skin disorders
200	L639	Other skin disorders
200	L640	Other skin disorders
200	L648	Other skin disorders
200	L649	Other skin disorders
200	L650	Other skin disorders

200	L651	Other skin disorders
200	L652	Other skin disorders
200	L658	Other skin disorders
200	L659	Other skin disorders
200	L660	Other skin disorders
200	L662	Other skin disorders
200	L663	Other skin disorders
200	L664	Other skin disorders
200	L668	Other skin disorders
200	L669	Other skin disorders
200	L670	Other skin disorders
200	L671	Other skin disorders
200	L678	Other skin disorders
200	L679	Other skin disorders
200	L680	Other skin disorders
200	L681	Other skin disorders
200	L682	Other skin disorders
200	L683	Other skin disorders
200	L688	Other skin disorders
200	L689	Other skin disorders
200	L700	Other skin disorders
200	L701	Other skin disorders
200	L702	Other skin disorders
200	L703	Other skin disorders
200	L704	Other skin disorders
200	L705	Other skin disorders
200	L708	Other skin disorders
200	L709	Other skin disorders
200	L720	Other skin disorders
200	L7211	Other skin disorders
200	L7212	Other skin disorders
200	L722	Other skin disorders
200	L723	Other skin disorders
200	L728	Other skin disorders
200	L729	Other skin disorders
200	L730	Other skin disorders
200	L731	Other skin disorders
200	L732	Other skin disorders
200	L738	Other skin disorders
200	L739	Other skin disorders
200	L740	Other skin disorders
200	L741	Other skin disorders
200	L742	Other skin disorders
200	L743	Other skin disorders
200	L744	Other skin disorders
200	L74510	Other skin disorders
200	L74511	Other skin disorders
200	L74512	Other skin disorders
200	L74513	Other skin disorders
200	L74519	Other skin disorders
200	L7452	Other skin disorders

200	L748	Other skin disorders
200	L749	Other skin disorders
200	L750	Other skin disorders
200	L751	Other skin disorders
200	L752	Other skin disorders
200	L758	Other skin disorders
200	L759	Other skin disorders
200	L80	Other skin disorders
200	L810	Other skin disorders
200	L811	Other skin disorders
200	L812	Other skin disorders
200	L813	Other skin disorders
200	L814	Other skin disorders
200	L815	Other skin disorders
200	L816	Other skin disorders
200	L817	Other skin disorders
200	L818	Other skin disorders
200	L819	Other skin disorders
200	L820	Other skin disorders
200	L821	Other skin disorders
200	L83	Other skin disorders
200	L84	Other skin disorders
200	L850	Other skin disorders
200	L851	Other skin disorders
200	L852	Other skin disorders
200	L853	Other skin disorders
200	L858	Other skin disorders
200	L859	Other skin disorders
200	L86	Other skin disorders
200	L870	Other skin disorders
200	L871	Other skin disorders
200	L872	Other skin disorders
200	L878	Other skin disorders
200	L879	Other skin disorders
200	L900	Other skin disorders
200	L901	Other skin disorders
200	L902	Other skin disorders
200	L903	Other skin disorders
200	L904	Other skin disorders
200	L905	Other skin disorders
200	L906	Other skin disorders
200	L908	Other skin disorders
200	L909	Other skin disorders
200	L910	Other skin disorders
200	L918	Other skin disorders
200	L919	Other skin disorders
200	L921	Other skin disorders
200	L922	Other skin disorders
200	L923	Other skin disorders
200	L928	Other skin disorders
200	L929	Other skin disorders

200	L940	Other skin disorders
200	L941	Other skin disorders
200	L942	Other skin disorders
200	L943	Other skin disorders
200	L944	Other skin disorders
200	L945	Other skin disorders
200	L948	Other skin disorders
200	L949	Other skin disorders
200	L980	Other skin disorders
200	L985	Other skin disorders
200	L986	Other skin disorders
200	L988	Other skin disorders
200	L989	Other skin disorders
200	L99	Other skin disorders
200	R21	Other skin disorders
200	R220	Other skin disorders
200	R221	Other skin disorders
200	R222	Other skin disorders
200	R2230	Other skin disorders
200	R2231	Other skin disorders
200	R2232	Other skin disorders
200	R2233	Other skin disorders
200	R2240	Other skin disorders
200	R2241	Other skin disorders
200	R2242	Other skin disorders
200	R2243	Other skin disorders
200	R229	Other skin disorders
200	R234	Other skin disorders
200	R238	Other skin disorders
200	R239	Other skin disorders
200	R61	Other skin disorders
200	Z872	Other skin disorders
200	Z945	Other skin disorders
200	Z9681	Other skin disorders
201	A0104	Infective arthritis and osteomyelitis (except that caused by tuberculosis or sexually transmitted disease)
201	A0105	Infective arthritis and osteomyelitis (except that caused by tuberculosis or sexually transmitted disease)
201	A0223	Infective arthritis and osteomyelitis (except that caused by tuberculosis or sexually transmitted disease)
201	A0224	Infective arthritis and osteomyelitis (except that caused by tuberculosis or sexually transmitted disease)
201	A3983	Infective arthritis and osteomyelitis (except that caused by tuberculosis or sexually transmitted disease)
201	A3984	Infective arthritis and osteomyelitis (except that caused by tuberculosis or sexually transmitted disease)
201	A6923	Infective arthritis and osteomyelitis (except that caused by tuberculosis or sexually transmitted disease)
201	B0682	Infective arthritis and osteomyelitis (except that caused by tuberculosis or sexually transmitted disease)
201	B2685	Infective arthritis and osteomyelitis (except that caused by tuberculosis or sexually transmitted disease)
201	M0000	Infective arthritis and osteomyelitis (except that caused by tuberculosis or sexually transmitted disease)
201	M00011	Infective arthritis and osteomyelitis (except that caused by tuberculosis or sexually transmitted disease)
201	M00012	Infective arthritis and osteomyelitis (except that caused by tuberculosis or sexually transmitted disease)
201	M00019	Infective arthritis and osteomyelitis (except that caused by tuberculosis or sexually transmitted disease)
201	M00021	Infective arthritis and osteomyelitis (except that caused by tuberculosis or sexually transmitted disease)
201	M00022	Infective arthritis and osteomyelitis (except that caused by tuberculosis or sexually transmitted disease)
201	M00029	Infective arthritis and osteomyelitis (except that caused by tuberculosis or sexually transmitted disease)
201	M00031	Infective arthritis and osteomyelitis (except that caused by tuberculosis or sexually transmitted disease)

201	M00032	Infective arthritis and osteomyelitis (except that caused by tuberculosis or sexually transmitted disease)
201	M00039	Infective arthritis and osteomyelitis (except that caused by tuberculosis or sexually transmitted disease)
201	M00041	Infective arthritis and osteomyelitis (except that caused by tuberculosis or sexually transmitted disease)
201	M00042	Infective arthritis and osteomyelitis (except that caused by tuberculosis or sexually transmitted disease)
201	M00049	Infective arthritis and osteomyelitis (except that caused by tuberculosis or sexually transmitted disease)
201	M00051	Infective arthritis and osteomyelitis (except that caused by tuberculosis or sexually transmitted disease)
201	M00052	Infective arthritis and osteomyelitis (except that caused by tuberculosis or sexually transmitted disease)
201	M00059	Infective arthritis and osteomyelitis (except that caused by tuberculosis or sexually transmitted disease)
201	M00061	Infective arthritis and osteomyelitis (except that caused by tuberculosis or sexually transmitted disease)
201	M00062	Infective arthritis and osteomyelitis (except that caused by tuberculosis or sexually transmitted disease)
201	M00069	Infective arthritis and osteomyelitis (except that caused by tuberculosis or sexually transmitted disease)
201	M00071	Infective arthritis and osteomyelitis (except that caused by tuberculosis or sexually transmitted disease)
201	M00072	Infective arthritis and osteomyelitis (except that caused by tuberculosis or sexually transmitted disease)
201	M00079	Infective arthritis and osteomyelitis (except that caused by tuberculosis or sexually transmitted disease)
201	M0008	Infective arthritis and osteomyelitis (except that caused by tuberculosis or sexually transmitted disease)
201	M0009	Infective arthritis and osteomyelitis (except that caused by tuberculosis or sexually transmitted disease)
201	M0010	Infective arthritis and osteomyelitis (except that caused by tuberculosis or sexually transmitted disease)
201	M00111	Infective arthritis and osteomyelitis (except that caused by tuberculosis or sexually transmitted disease)
201	M00112	Infective arthritis and osteomyelitis (except that caused by tuberculosis or sexually transmitted disease)
201	M00119	Infective arthritis and osteomyelitis (except that caused by tuberculosis or sexually transmitted disease)
201	M00121	Infective arthritis and osteomyelitis (except that caused by tuberculosis or sexually transmitted disease)
201	M00122	Infective arthritis and osteomyelitis (except that caused by tuberculosis or sexually transmitted disease)
201	M00129	Infective arthritis and osteomyelitis (except that caused by tuberculosis or sexually transmitted disease)
201	M00131	Infective arthritis and osteomyelitis (except that caused by tuberculosis or sexually transmitted disease)
201	M00132	Infective arthritis and osteomyelitis (except that caused by tuberculosis or sexually transmitted disease)
201	M00139	Infective arthritis and osteomyelitis (except that caused by tuberculosis or sexually transmitted disease)
201	M00141	Infective arthritis and osteomyelitis (except that caused by tuberculosis or sexually transmitted disease)
201	M00142	Infective arthritis and osteomyelitis (except that caused by tuberculosis or sexually transmitted disease)
201	M00149	Infective arthritis and osteomyelitis (except that caused by tuberculosis or sexually transmitted disease)
201	M00151	Infective arthritis and osteomyelitis (except that caused by tuberculosis or sexually transmitted disease)
201	M00152	Infective arthritis and osteomyelitis (except that caused by tuberculosis or sexually transmitted disease)
201	M00159	Infective arthritis and osteomyelitis (except that caused by tuberculosis or sexually transmitted disease)
201	M00161	Infective arthritis and osteomyelitis (except that caused by tuberculosis or sexually transmitted disease)
201	M00162	Infective arthritis and osteomyelitis (except that caused by tuberculosis or sexually transmitted disease)
201	M00169	Infective arthritis and osteomyelitis (except that caused by tuberculosis or sexually transmitted disease)
201	M00171	Infective arthritis and osteomyelitis (except that caused by tuberculosis or sexually transmitted disease)
201	M00172	Infective arthritis and osteomyelitis (except that caused by tuberculosis or sexually transmitted disease)
201	M00179	Infective arthritis and osteomyelitis (except that caused by tuberculosis or sexually transmitted disease)
201	M0018	Infective arthritis and osteomyelitis (except that caused by tuberculosis or sexually transmitted disease)
201	M0019	Infective arthritis and osteomyelitis (except that caused by tuberculosis or sexually transmitted disease)
201	M0020	Infective arthritis and osteomyelitis (except that caused by tuberculosis or sexually transmitted disease)
201	M00211	Infective arthritis and osteomyelitis (except that caused by tuberculosis or sexually transmitted disease)
201	M00212	Infective arthritis and osteomyelitis (except that caused by tuberculosis or sexually transmitted disease)
201	M00219	Infective arthritis and osteomyelitis (except that caused by tuberculosis or sexually transmitted disease)
201	M00221	Infective arthritis and osteomyelitis (except that caused by tuberculosis or sexually transmitted disease)
201	M00222	Infective arthritis and osteomyelitis (except that caused by tuberculosis or sexually transmitted disease)
201	M00229	Infective arthritis and osteomyelitis (except that caused by tuberculosis or sexually transmitted disease)
201	M00231	Infective arthritis and osteomyelitis (except that caused by tuberculosis or sexually transmitted disease)
201	M00232	Infective arthritis and osteomyelitis (except that caused by tuberculosis or sexually transmitted disease)
201	M00239	Infective arthritis and osteomyelitis (except that caused by tuberculosis or sexually transmitted disease)
201	M00241	Infective arthritis and osteomyelitis (except that caused by tuberculosis or sexually transmitted disease)

201	M00242	Infective arthritis and osteomyelitis (except that caused by tuberculosis or sexually transmitted disease)
201	M00249	Infective arthritis and osteomyelitis (except that caused by tuberculosis or sexually transmitted disease)
201	M00251	Infective arthritis and osteomyelitis (except that caused by tuberculosis or sexually transmitted disease)
201	M00252	Infective arthritis and osteomyelitis (except that caused by tuberculosis or sexually transmitted disease)
201	M00259	Infective arthritis and osteomyelitis (except that caused by tuberculosis or sexually transmitted disease)
201	M00261	Infective arthritis and osteomyelitis (except that caused by tuberculosis or sexually transmitted disease)
201	M00262	Infective arthritis and osteomyelitis (except that caused by tuberculosis or sexually transmitted disease)
201	M00269	Infective arthritis and osteomyelitis (except that caused by tuberculosis or sexually transmitted disease)
201	M00271	Infective arthritis and osteomyelitis (except that caused by tuberculosis or sexually transmitted disease)
201	M00272	Infective arthritis and osteomyelitis (except that caused by tuberculosis or sexually transmitted disease)
201	M00279	Infective arthritis and osteomyelitis (except that caused by tuberculosis or sexually transmitted disease)
201	M0028	Infective arthritis and osteomyelitis (except that caused by tuberculosis or sexually transmitted disease)
201	M0029	Infective arthritis and osteomyelitis (except that caused by tuberculosis or sexually transmitted disease)
201	M0080	Infective arthritis and osteomyelitis (except that caused by tuberculosis or sexually transmitted disease)
201	M00811	Infective arthritis and osteomyelitis (except that caused by tuberculosis or sexually transmitted disease)
201	M00812	Infective arthritis and osteomyelitis (except that caused by tuberculosis or sexually transmitted disease)
201	M00819	Infective arthritis and osteomyelitis (except that caused by tuberculosis or sexually transmitted disease)
201	M00821	Infective arthritis and osteomyelitis (except that caused by tuberculosis or sexually transmitted disease)
201	M00822	Infective arthritis and osteomyelitis (except that caused by tuberculosis or sexually transmitted disease)
201	M00829	Infective arthritis and osteomyelitis (except that caused by tuberculosis or sexually transmitted disease)
201	M00831	Infective arthritis and osteomyelitis (except that caused by tuberculosis or sexually transmitted disease)
201	M00832	Infective arthritis and osteomyelitis (except that caused by tuberculosis or sexually transmitted disease)
201	M00839	Infective arthritis and osteomyelitis (except that caused by tuberculosis or sexually transmitted disease)
201	M00841	Infective arthritis and osteomyelitis (except that caused by tuberculosis or sexually transmitted disease)
201	M00842	Infective arthritis and osteomyelitis (except that caused by tuberculosis or sexually transmitted disease)
201	M00849	Infective arthritis and osteomyelitis (except that caused by tuberculosis or sexually transmitted disease)
201	M00851	Infective arthritis and osteomyelitis (except that caused by tuberculosis or sexually transmitted disease)
201	M00852	Infective arthritis and osteomyelitis (except that caused by tuberculosis or sexually transmitted disease)
201	M00859	Infective arthritis and osteomyelitis (except that caused by tuberculosis or sexually transmitted disease)
201	M00861	Infective arthritis and osteomyelitis (except that caused by tuberculosis or sexually transmitted disease)
201	M00862	Infective arthritis and osteomyelitis (except that caused by tuberculosis or sexually transmitted disease)
201	M00869	Infective arthritis and osteomyelitis (except that caused by tuberculosis or sexually transmitted disease)
201	M00871	Infective arthritis and osteomyelitis (except that caused by tuberculosis or sexually transmitted disease)
201	M00872	Infective arthritis and osteomyelitis (except that caused by tuberculosis or sexually transmitted disease)
201	M00879	Infective arthritis and osteomyelitis (except that caused by tuberculosis or sexually transmitted disease)
201	M0088	Infective arthritis and osteomyelitis (except that caused by tuberculosis or sexually transmitted disease)
201	M0089	Infective arthritis and osteomyelitis (except that caused by tuberculosis or sexually transmitted disease)
201	M009	Infective arthritis and osteomyelitis (except that caused by tuberculosis or sexually transmitted disease)
201	M01X0	Infective arthritis and osteomyelitis (except that caused by tuberculosis or sexually transmitted disease)
201	M01X11	Infective arthritis and osteomyelitis (except that caused by tuberculosis or sexually transmitted disease)
201	M01X12	Infective arthritis and osteomyelitis (except that caused by tuberculosis or sexually transmitted disease)
201	M01X19	Infective arthritis and osteomyelitis (except that caused by tuberculosis or sexually transmitted disease)
201	M01X21	Infective arthritis and osteomyelitis (except that caused by tuberculosis or sexually transmitted disease)
201	M01X22	Infective arthritis and osteomyelitis (except that caused by tuberculosis or sexually transmitted disease)
201	M01X29	Infective arthritis and osteomyelitis (except that caused by tuberculosis or sexually transmitted disease)
201	M01X31	Infective arthritis and osteomyelitis (except that caused by tuberculosis or sexually transmitted disease)
201	M01X32	Infective arthritis and osteomyelitis (except that caused by tuberculosis or sexually transmitted disease)
201	M01X39	Infective arthritis and osteomyelitis (except that caused by tuberculosis or sexually transmitted disease)
201	M01X41	Infective arthritis and osteomyelitis (except that caused by tuberculosis or sexually transmitted disease)
201	M01X42	Infective arthritis and osteomyelitis (except that caused by tuberculosis or sexually transmitted disease)
201	M01X49	Infective arthritis and osteomyelitis (except that caused by tuberculosis or sexually transmitted disease)

201	M01X51	Infective arthritis and osteomyelitis (except that caused by tuberculosis or sexually transmitted disease)
201	M01X52	Infective arthritis and osteomyelitis (except that caused by tuberculosis or sexually transmitted disease)
201	M01X59	Infective arthritis and osteomyelitis (except that caused by tuberculosis or sexually transmitted disease)
201	M01X61	Infective arthritis and osteomyelitis (except that caused by tuberculosis or sexually transmitted disease)
201	M01X62	Infective arthritis and osteomyelitis (except that caused by tuberculosis or sexually transmitted disease)
201	M01X69	Infective arthritis and osteomyelitis (except that caused by tuberculosis or sexually transmitted disease)
201	M01X71	Infective arthritis and osteomyelitis (except that caused by tuberculosis or sexually transmitted disease)
201	M01X72	Infective arthritis and osteomyelitis (except that caused by tuberculosis or sexually transmitted disease)
201	M01X79	Infective arthritis and osteomyelitis (except that caused by tuberculosis or sexually transmitted disease)
201	M01X8	Infective arthritis and osteomyelitis (except that caused by tuberculosis or sexually transmitted disease)
201	M01X9	Infective arthritis and osteomyelitis (except that caused by tuberculosis or sexually transmitted disease)
201	M0210	Infective arthritis and osteomyelitis (except that caused by tuberculosis or sexually transmitted disease)
201	M02111	Infective arthritis and osteomyelitis (except that caused by tuberculosis or sexually transmitted disease)
201	M02112	Infective arthritis and osteomyelitis (except that caused by tuberculosis or sexually transmitted disease)
201	M02119	Infective arthritis and osteomyelitis (except that caused by tuberculosis or sexually transmitted disease)
201	M02121	Infective arthritis and osteomyelitis (except that caused by tuberculosis or sexually transmitted disease)
201	M02122	Infective arthritis and osteomyelitis (except that caused by tuberculosis or sexually transmitted disease)
201	M02129	Infective arthritis and osteomyelitis (except that caused by tuberculosis or sexually transmitted disease)
201	M02131	Infective arthritis and osteomyelitis (except that caused by tuberculosis or sexually transmitted disease)
201	M02132	Infective arthritis and osteomyelitis (except that caused by tuberculosis or sexually transmitted disease)
201	M02139	Infective arthritis and osteomyelitis (except that caused by tuberculosis or sexually transmitted disease)
201	M02141	Infective arthritis and osteomyelitis (except that caused by tuberculosis or sexually transmitted disease)
201	M02142	Infective arthritis and osteomyelitis (except that caused by tuberculosis or sexually transmitted disease)
201	M02149	Infective arthritis and osteomyelitis (except that caused by tuberculosis or sexually transmitted disease)
201	M02151	Infective arthritis and osteomyelitis (except that caused by tuberculosis or sexually transmitted disease)
201	M02152	Infective arthritis and osteomyelitis (except that caused by tuberculosis or sexually transmitted disease)
201	M02159	Infective arthritis and osteomyelitis (except that caused by tuberculosis or sexually transmitted disease)
201	M02161	Infective arthritis and osteomyelitis (except that caused by tuberculosis or sexually transmitted disease)
201	M02162	Infective arthritis and osteomyelitis (except that caused by tuberculosis or sexually transmitted disease)
201	M02169	Infective arthritis and osteomyelitis (except that caused by tuberculosis or sexually transmitted disease)
201	M02171	Infective arthritis and osteomyelitis (except that caused by tuberculosis or sexually transmitted disease)
201	M02172	Infective arthritis and osteomyelitis (except that caused by tuberculosis or sexually transmitted disease)
201	M02179	Infective arthritis and osteomyelitis (except that caused by tuberculosis or sexually transmitted disease)
201	M0218	Infective arthritis and osteomyelitis (except that caused by tuberculosis or sexually transmitted disease)
201	M0219	Infective arthritis and osteomyelitis (except that caused by tuberculosis or sexually transmitted disease)
201	M0230	Infective arthritis and osteomyelitis (except that caused by tuberculosis or sexually transmitted disease)
201	M02311	Infective arthritis and osteomyelitis (except that caused by tuberculosis or sexually transmitted disease)
201	M02312	Infective arthritis and osteomyelitis (except that caused by tuberculosis or sexually transmitted disease)
201	M02319	Infective arthritis and osteomyelitis (except that caused by tuberculosis or sexually transmitted disease)
201	M02321	Infective arthritis and osteomyelitis (except that caused by tuberculosis or sexually transmitted disease)
201	M02322	Infective arthritis and osteomyelitis (except that caused by tuberculosis or sexually transmitted disease)
201	M02329	Infective arthritis and osteomyelitis (except that caused by tuberculosis or sexually transmitted disease)
201	M02331	Infective arthritis and osteomyelitis (except that caused by tuberculosis or sexually transmitted disease)
201	M02332	Infective arthritis and osteomyelitis (except that caused by tuberculosis or sexually transmitted disease)
201	M02339	Infective arthritis and osteomyelitis (except that caused by tuberculosis or sexually transmitted disease)
201	M02341	Infective arthritis and osteomyelitis (except that caused by tuberculosis or sexually transmitted disease)
201	M02342	Infective arthritis and osteomyelitis (except that caused by tuberculosis or sexually transmitted disease)
201	M02349	Infective arthritis and osteomyelitis (except that caused by tuberculosis or sexually transmitted disease)
201	M02351	Infective arthritis and osteomyelitis (except that caused by tuberculosis or sexually transmitted disease)
201	M02352	Infective arthritis and osteomyelitis (except that caused by tuberculosis or sexually transmitted disease)
201	M02359	Infective arthritis and osteomyelitis (except that caused by tuberculosis or sexually transmitted disease)

201	M02361	Infective arthritis and osteomyelitis (except that caused by tuberculosis or sexually transmitted disease)
201	M02362	Infective arthritis and osteomyelitis (except that caused by tuberculosis or sexually transmitted disease)
201	M02369	Infective arthritis and osteomyelitis (except that caused by tuberculosis or sexually transmitted disease)
201	M02371	Infective arthritis and osteomyelitis (except that caused by tuberculosis or sexually transmitted disease)
201	M02372	Infective arthritis and osteomyelitis (except that caused by tuberculosis or sexually transmitted disease)
201	M02379	Infective arthritis and osteomyelitis (except that caused by tuberculosis or sexually transmitted disease)
201	M0238	Infective arthritis and osteomyelitis (except that caused by tuberculosis or sexually transmitted disease)
201	M0239	Infective arthritis and osteomyelitis (except that caused by tuberculosis or sexually transmitted disease)
201	M0280	Infective arthritis and osteomyelitis (except that caused by tuberculosis or sexually transmitted disease)
201	M02811	Infective arthritis and osteomyelitis (except that caused by tuberculosis or sexually transmitted disease)
201	M02812	Infective arthritis and osteomyelitis (except that caused by tuberculosis or sexually transmitted disease)
201	M02819	Infective arthritis and osteomyelitis (except that caused by tuberculosis or sexually transmitted disease)
201	M02821	Infective arthritis and osteomyelitis (except that caused by tuberculosis or sexually transmitted disease)
201	M02822	Infective arthritis and osteomyelitis (except that caused by tuberculosis or sexually transmitted disease)
201	M02829	Infective arthritis and osteomyelitis (except that caused by tuberculosis or sexually transmitted disease)
201	M02831	Infective arthritis and osteomyelitis (except that caused by tuberculosis or sexually transmitted disease)
201	M02832	Infective arthritis and osteomyelitis (except that caused by tuberculosis or sexually transmitted disease)
201	M02839	Infective arthritis and osteomyelitis (except that caused by tuberculosis or sexually transmitted disease)
201	M02841	Infective arthritis and osteomyelitis (except that caused by tuberculosis or sexually transmitted disease)
201	M02842	Infective arthritis and osteomyelitis (except that caused by tuberculosis or sexually transmitted disease)
201	M02849	Infective arthritis and osteomyelitis (except that caused by tuberculosis or sexually transmitted disease)
201	M02851	Infective arthritis and osteomyelitis (except that caused by tuberculosis or sexually transmitted disease)
201	M02852	Infective arthritis and osteomyelitis (except that caused by tuberculosis or sexually transmitted disease)
201	M02859	Infective arthritis and osteomyelitis (except that caused by tuberculosis or sexually transmitted disease)
201	M02861	Infective arthritis and osteomyelitis (except that caused by tuberculosis or sexually transmitted disease)
201	M02862	Infective arthritis and osteomyelitis (except that caused by tuberculosis or sexually transmitted disease)
201	M02869	Infective arthritis and osteomyelitis (except that caused by tuberculosis or sexually transmitted disease)
201	M02871	Infective arthritis and osteomyelitis (except that caused by tuberculosis or sexually transmitted disease)
201	M02872	Infective arthritis and osteomyelitis (except that caused by tuberculosis or sexually transmitted disease)
201	M02879	Infective arthritis and osteomyelitis (except that caused by tuberculosis or sexually transmitted disease)
201	M0288	Infective arthritis and osteomyelitis (except that caused by tuberculosis or sexually transmitted disease)
201	M0289	Infective arthritis and osteomyelitis (except that caused by tuberculosis or sexually transmitted disease)
201	M029	Infective arthritis and osteomyelitis (except that caused by tuberculosis or sexually transmitted disease)
201	M4620	Infective arthritis and osteomyelitis (except that caused by tuberculosis or sexually transmitted disease)
201	M4621	Infective arthritis and osteomyelitis (except that caused by tuberculosis or sexually transmitted disease)
201	M4622	Infective arthritis and osteomyelitis (except that caused by tuberculosis or sexually transmitted disease)
201	M4623	Infective arthritis and osteomyelitis (except that caused by tuberculosis or sexually transmitted disease)
201	M4624	Infective arthritis and osteomyelitis (except that caused by tuberculosis or sexually transmitted disease)
201	M4625	Infective arthritis and osteomyelitis (except that caused by tuberculosis or sexually transmitted disease)
201	M4626	Infective arthritis and osteomyelitis (except that caused by tuberculosis or sexually transmitted disease)
201	M4627	Infective arthritis and osteomyelitis (except that caused by tuberculosis or sexually transmitted disease)
201	M4628	Infective arthritis and osteomyelitis (except that caused by tuberculosis or sexually transmitted disease)
201	M8600	Infective arthritis and osteomyelitis (except that caused by tuberculosis or sexually transmitted disease)
201	M86011	Infective arthritis and osteomyelitis (except that caused by tuberculosis or sexually transmitted disease)
201	M86012	Infective arthritis and osteomyelitis (except that caused by tuberculosis or sexually transmitted disease)
201	M86019	Infective arthritis and osteomyelitis (except that caused by tuberculosis or sexually transmitted disease)
201	M86021	Infective arthritis and osteomyelitis (except that caused by tuberculosis or sexually transmitted disease)
201	M86022	Infective arthritis and osteomyelitis (except that caused by tuberculosis or sexually transmitted disease)
201	M86029	Infective arthritis and osteomyelitis (except that caused by tuberculosis or sexually transmitted disease)
201	M86031	Infective arthritis and osteomyelitis (except that caused by tuberculosis or sexually transmitted disease)
201	M86032	Infective arthritis and osteomyelitis (except that caused by tuberculosis or sexually transmitted disease)

201	M86039	Infective arthritis and osteomyelitis (except that caused by tuberculosis or sexually transmitted disease)
201	M86041	Infective arthritis and osteomyelitis (except that caused by tuberculosis or sexually transmitted disease)
201	M86042	Infective arthritis and osteomyelitis (except that caused by tuberculosis or sexually transmitted disease)
201	M86049	Infective arthritis and osteomyelitis (except that caused by tuberculosis or sexually transmitted disease)
201	M86051	Infective arthritis and osteomyelitis (except that caused by tuberculosis or sexually transmitted disease)
201	M86052	Infective arthritis and osteomyelitis (except that caused by tuberculosis or sexually transmitted disease)
201	M86059	Infective arthritis and osteomyelitis (except that caused by tuberculosis or sexually transmitted disease)
201	M86061	Infective arthritis and osteomyelitis (except that caused by tuberculosis or sexually transmitted disease)
201	M86062	Infective arthritis and osteomyelitis (except that caused by tuberculosis or sexually transmitted disease)
201	M86069	Infective arthritis and osteomyelitis (except that caused by tuberculosis or sexually transmitted disease)
201	M86071	Infective arthritis and osteomyelitis (except that caused by tuberculosis or sexually transmitted disease)
201	M86072	Infective arthritis and osteomyelitis (except that caused by tuberculosis or sexually transmitted disease)
201	M86079	Infective arthritis and osteomyelitis (except that caused by tuberculosis or sexually transmitted disease)
201	M8608	Infective arthritis and osteomyelitis (except that caused by tuberculosis or sexually transmitted disease)
201	M8609	Infective arthritis and osteomyelitis (except that caused by tuberculosis or sexually transmitted disease)
201	M8610	Infective arthritis and osteomyelitis (except that caused by tuberculosis or sexually transmitted disease)
201	M86111	Infective arthritis and osteomyelitis (except that caused by tuberculosis or sexually transmitted disease)
201	M86112	Infective arthritis and osteomyelitis (except that caused by tuberculosis or sexually transmitted disease)
201	M86119	Infective arthritis and osteomyelitis (except that caused by tuberculosis or sexually transmitted disease)
201	M86121	Infective arthritis and osteomyelitis (except that caused by tuberculosis or sexually transmitted disease)
201	M86122	Infective arthritis and osteomyelitis (except that caused by tuberculosis or sexually transmitted disease)
201	M86129	Infective arthritis and osteomyelitis (except that caused by tuberculosis or sexually transmitted disease)
201	M86131	Infective arthritis and osteomyelitis (except that caused by tuberculosis or sexually transmitted disease)
201	M86132	Infective arthritis and osteomyelitis (except that caused by tuberculosis or sexually transmitted disease)
201	M86139	Infective arthritis and osteomyelitis (except that caused by tuberculosis or sexually transmitted disease)
201	M86141	Infective arthritis and osteomyelitis (except that caused by tuberculosis or sexually transmitted disease)
201	M86142	Infective arthritis and osteomyelitis (except that caused by tuberculosis or sexually transmitted disease)
201	M86149	Infective arthritis and osteomyelitis (except that caused by tuberculosis or sexually transmitted disease)
201	M86151	Infective arthritis and osteomyelitis (except that caused by tuberculosis or sexually transmitted disease)
201	M86152	Infective arthritis and osteomyelitis (except that caused by tuberculosis or sexually transmitted disease)
201	M86159	Infective arthritis and osteomyelitis (except that caused by tuberculosis or sexually transmitted disease)
201	M86161	Infective arthritis and osteomyelitis (except that caused by tuberculosis or sexually transmitted disease)
201	M86162	Infective arthritis and osteomyelitis (except that caused by tuberculosis or sexually transmitted disease)
201	M86169	Infective arthritis and osteomyelitis (except that caused by tuberculosis or sexually transmitted disease)
201	M86171	Infective arthritis and osteomyelitis (except that caused by tuberculosis or sexually transmitted disease)
201	M86172	Infective arthritis and osteomyelitis (except that caused by tuberculosis or sexually transmitted disease)
201	M86179	Infective arthritis and osteomyelitis (except that caused by tuberculosis or sexually transmitted disease)
201	M8618	Infective arthritis and osteomyelitis (except that caused by tuberculosis or sexually transmitted disease)
201	M8619	Infective arthritis and osteomyelitis (except that caused by tuberculosis or sexually transmitted disease)
201	M8620	Infective arthritis and osteomyelitis (except that caused by tuberculosis or sexually transmitted disease)
201	M86211	Infective arthritis and osteomyelitis (except that caused by tuberculosis or sexually transmitted disease)
201	M86212	Infective arthritis and osteomyelitis (except that caused by tuberculosis or sexually transmitted disease)
201	M86219	Infective arthritis and osteomyelitis (except that caused by tuberculosis or sexually transmitted disease)
201	M86221	Infective arthritis and osteomyelitis (except that caused by tuberculosis or sexually transmitted disease)
201	M86222	Infective arthritis and osteomyelitis (except that caused by tuberculosis or sexually transmitted disease)
201	M86229	Infective arthritis and osteomyelitis (except that caused by tuberculosis or sexually transmitted disease)
201	M86231	Infective arthritis and osteomyelitis (except that caused by tuberculosis or sexually transmitted disease)
201	M86232	Infective arthritis and osteomyelitis (except that caused by tuberculosis or sexually transmitted disease)
201	M86239	Infective arthritis and osteomyelitis (except that caused by tuberculosis or sexually transmitted disease)
201	M86241	Infective arthritis and osteomyelitis (except that caused by tuberculosis or sexually transmitted disease)
201	M86242	Infective arthritis and osteomyelitis (except that caused by tuberculosis or sexually transmitted disease)

201	M86249	Infective arthritis and osteomyelitis (except that caused by tuberculosis or sexually transmitted disease)
201	M86251	Infective arthritis and osteomyelitis (except that caused by tuberculosis or sexually transmitted disease)
201	M86252	Infective arthritis and osteomyelitis (except that caused by tuberculosis or sexually transmitted disease)
201	M86259	Infective arthritis and osteomyelitis (except that caused by tuberculosis or sexually transmitted disease)
201	M86261	Infective arthritis and osteomyelitis (except that caused by tuberculosis or sexually transmitted disease)
201	M86262	Infective arthritis and osteomyelitis (except that caused by tuberculosis or sexually transmitted disease)
201	M86269	Infective arthritis and osteomyelitis (except that caused by tuberculosis or sexually transmitted disease)
201	M86271	Infective arthritis and osteomyelitis (except that caused by tuberculosis or sexually transmitted disease)
201	M86272	Infective arthritis and osteomyelitis (except that caused by tuberculosis or sexually transmitted disease)
201	M86279	Infective arthritis and osteomyelitis (except that caused by tuberculosis or sexually transmitted disease)
201	M8628	Infective arthritis and osteomyelitis (except that caused by tuberculosis or sexually transmitted disease)
201	M8629	Infective arthritis and osteomyelitis (except that caused by tuberculosis or sexually transmitted disease)
201	M8630	Infective arthritis and osteomyelitis (except that caused by tuberculosis or sexually transmitted disease)
201	M86311	Infective arthritis and osteomyelitis (except that caused by tuberculosis or sexually transmitted disease)
201	M86312	Infective arthritis and osteomyelitis (except that caused by tuberculosis or sexually transmitted disease)
201	M86319	Infective arthritis and osteomyelitis (except that caused by tuberculosis or sexually transmitted disease)
201	M86321	Infective arthritis and osteomyelitis (except that caused by tuberculosis or sexually transmitted disease)
201	M86322	Infective arthritis and osteomyelitis (except that caused by tuberculosis or sexually transmitted disease)
201	M86329	Infective arthritis and osteomyelitis (except that caused by tuberculosis or sexually transmitted disease)
201	M86331	Infective arthritis and osteomyelitis (except that caused by tuberculosis or sexually transmitted disease)
201	M86332	Infective arthritis and osteomyelitis (except that caused by tuberculosis or sexually transmitted disease)
201	M86339	Infective arthritis and osteomyelitis (except that caused by tuberculosis or sexually transmitted disease)
201	M86341	Infective arthritis and osteomyelitis (except that caused by tuberculosis or sexually transmitted disease)
201	M86342	Infective arthritis and osteomyelitis (except that caused by tuberculosis or sexually transmitted disease)
201	M86349	Infective arthritis and osteomyelitis (except that caused by tuberculosis or sexually transmitted disease)
201	M86351	Infective arthritis and osteomyelitis (except that caused by tuberculosis or sexually transmitted disease)
201	M86352	Infective arthritis and osteomyelitis (except that caused by tuberculosis or sexually transmitted disease)
201	M86359	Infective arthritis and osteomyelitis (except that caused by tuberculosis or sexually transmitted disease)
201	M86361	Infective arthritis and osteomyelitis (except that caused by tuberculosis or sexually transmitted disease)
201	M86362	Infective arthritis and osteomyelitis (except that caused by tuberculosis or sexually transmitted disease)
201	M86369	Infective arthritis and osteomyelitis (except that caused by tuberculosis or sexually transmitted disease)
201	M86371	Infective arthritis and osteomyelitis (except that caused by tuberculosis or sexually transmitted disease)
201	M86372	Infective arthritis and osteomyelitis (except that caused by tuberculosis or sexually transmitted disease)
201	M86379	Infective arthritis and osteomyelitis (except that caused by tuberculosis or sexually transmitted disease)
201	M8638	Infective arthritis and osteomyelitis (except that caused by tuberculosis or sexually transmitted disease)
201	M8639	Infective arthritis and osteomyelitis (except that caused by tuberculosis or sexually transmitted disease)
201	M8640	Infective arthritis and osteomyelitis (except that caused by tuberculosis or sexually transmitted disease)
201	M86411	Infective arthritis and osteomyelitis (except that caused by tuberculosis or sexually transmitted disease)
201	M86412	Infective arthritis and osteomyelitis (except that caused by tuberculosis or sexually transmitted disease)
201	M86419	Infective arthritis and osteomyelitis (except that caused by tuberculosis or sexually transmitted disease)
201	M86421	Infective arthritis and osteomyelitis (except that caused by tuberculosis or sexually transmitted disease)
201	M86422	Infective arthritis and osteomyelitis (except that caused by tuberculosis or sexually transmitted disease)
201	M86429	Infective arthritis and osteomyelitis (except that caused by tuberculosis or sexually transmitted disease)
201	M86431	Infective arthritis and osteomyelitis (except that caused by tuberculosis or sexually transmitted disease)
201	M86432	Infective arthritis and osteomyelitis (except that caused by tuberculosis or sexually transmitted disease)
201	M86439	Infective arthritis and osteomyelitis (except that caused by tuberculosis or sexually transmitted disease)
201	M86441	Infective arthritis and osteomyelitis (except that caused by tuberculosis or sexually transmitted disease)
201	M86442	Infective arthritis and osteomyelitis (except that caused by tuberculosis or sexually transmitted disease)
201	M86449	Infective arthritis and osteomyelitis (except that caused by tuberculosis or sexually transmitted disease)
201	M86451	Infective arthritis and osteomyelitis (except that caused by tuberculosis or sexually transmitted disease)
201	M86452	Infective arthritis and osteomyelitis (except that caused by tuberculosis or sexually transmitted disease)

201	M86459	Infective arthritis and osteomyelitis (except that caused by tuberculosis or sexually transmitted disease)
201	M86461	Infective arthritis and osteomyelitis (except that caused by tuberculosis or sexually transmitted disease)
201	M86462	Infective arthritis and osteomyelitis (except that caused by tuberculosis or sexually transmitted disease)
201	M86469	Infective arthritis and osteomyelitis (except that caused by tuberculosis or sexually transmitted disease)
201	M86471	Infective arthritis and osteomyelitis (except that caused by tuberculosis or sexually transmitted disease)
201	M86472	Infective arthritis and osteomyelitis (except that caused by tuberculosis or sexually transmitted disease)
201	M86479	Infective arthritis and osteomyelitis (except that caused by tuberculosis or sexually transmitted disease)
201	M8648	Infective arthritis and osteomyelitis (except that caused by tuberculosis or sexually transmitted disease)
201	M8649	Infective arthritis and osteomyelitis (except that caused by tuberculosis or sexually transmitted disease)
201	M8650	Infective arthritis and osteomyelitis (except that caused by tuberculosis or sexually transmitted disease)
201	M86511	Infective arthritis and osteomyelitis (except that caused by tuberculosis or sexually transmitted disease)
201	M86512	Infective arthritis and osteomyelitis (except that caused by tuberculosis or sexually transmitted disease)
201	M86519	Infective arthritis and osteomyelitis (except that caused by tuberculosis or sexually transmitted disease)
201	M86521	Infective arthritis and osteomyelitis (except that caused by tuberculosis or sexually transmitted disease)
201	M86522	Infective arthritis and osteomyelitis (except that caused by tuberculosis or sexually transmitted disease)
201	M86529	Infective arthritis and osteomyelitis (except that caused by tuberculosis or sexually transmitted disease)
201	M86531	Infective arthritis and osteomyelitis (except that caused by tuberculosis or sexually transmitted disease)
201	M86532	Infective arthritis and osteomyelitis (except that caused by tuberculosis or sexually transmitted disease)
201	M86539	Infective arthritis and osteomyelitis (except that caused by tuberculosis or sexually transmitted disease)
201	M86541	Infective arthritis and osteomyelitis (except that caused by tuberculosis or sexually transmitted disease)
201	M86542	Infective arthritis and osteomyelitis (except that caused by tuberculosis or sexually transmitted disease)
201	M86549	Infective arthritis and osteomyelitis (except that caused by tuberculosis or sexually transmitted disease)
201	M86551	Infective arthritis and osteomyelitis (except that caused by tuberculosis or sexually transmitted disease)
201	M86552	Infective arthritis and osteomyelitis (except that caused by tuberculosis or sexually transmitted disease)
201	M86559	Infective arthritis and osteomyelitis (except that caused by tuberculosis or sexually transmitted disease)
201	M86561	Infective arthritis and osteomyelitis (except that caused by tuberculosis or sexually transmitted disease)
201	M86562	Infective arthritis and osteomyelitis (except that caused by tuberculosis or sexually transmitted disease)
201	M86569	Infective arthritis and osteomyelitis (except that caused by tuberculosis or sexually transmitted disease)
201	M86571	Infective arthritis and osteomyelitis (except that caused by tuberculosis or sexually transmitted disease)
201	M86572	Infective arthritis and osteomyelitis (except that caused by tuberculosis or sexually transmitted disease)
201	M86579	Infective arthritis and osteomyelitis (except that caused by tuberculosis or sexually transmitted disease)
201	M8658	Infective arthritis and osteomyelitis (except that caused by tuberculosis or sexually transmitted disease)
201	M8659	Infective arthritis and osteomyelitis (except that caused by tuberculosis or sexually transmitted disease)
201	M8660	Infective arthritis and osteomyelitis (except that caused by tuberculosis or sexually transmitted disease)
201	M86611	Infective arthritis and osteomyelitis (except that caused by tuberculosis or sexually transmitted disease)
201	M86612	Infective arthritis and osteomyelitis (except that caused by tuberculosis or sexually transmitted disease)
201	M86619	Infective arthritis and osteomyelitis (except that caused by tuberculosis or sexually transmitted disease)
201	M86621	Infective arthritis and osteomyelitis (except that caused by tuberculosis or sexually transmitted disease)
201	M86622	Infective arthritis and osteomyelitis (except that caused by tuberculosis or sexually transmitted disease)
201	M86629	Infective arthritis and osteomyelitis (except that caused by tuberculosis or sexually transmitted disease)
201	M86631	Infective arthritis and osteomyelitis (except that caused by tuberculosis or sexually transmitted disease)
201	M86632	Infective arthritis and osteomyelitis (except that caused by tuberculosis or sexually transmitted disease)
201	M86639	Infective arthritis and osteomyelitis (except that caused by tuberculosis or sexually transmitted disease)
201	M86641	Infective arthritis and osteomyelitis (except that caused by tuberculosis or sexually transmitted disease)
201	M86642	Infective arthritis and osteomyelitis (except that caused by tuberculosis or sexually transmitted disease)
201	M86649	Infective arthritis and osteomyelitis (except that caused by tuberculosis or sexually transmitted disease)
201	M86651	Infective arthritis and osteomyelitis (except that caused by tuberculosis or sexually transmitted disease)
201	M86652	Infective arthritis and osteomyelitis (except that caused by tuberculosis or sexually transmitted disease)
201	M86659	Infective arthritis and osteomyelitis (except that caused by tuberculosis or sexually transmitted disease)
201	M86661	Infective arthritis and osteomyelitis (except that caused by tuberculosis or sexually transmitted disease)
201	M86662	Infective arthritis and osteomyelitis (except that caused by tuberculosis or sexually transmitted disease)

201	M86669	Infective arthritis and osteomyelitis (except that caused by tuberculosis or sexually transmitted disease)
201	M86671	Infective arthritis and osteomyelitis (except that caused by tuberculosis or sexually transmitted disease)
201	M86672	Infective arthritis and osteomyelitis (except that caused by tuberculosis or sexually transmitted disease)
201	M86679	Infective arthritis and osteomyelitis (except that caused by tuberculosis or sexually transmitted disease)
201	M8668	Infective arthritis and osteomyelitis (except that caused by tuberculosis or sexually transmitted disease)
201	M8669	Infective arthritis and osteomyelitis (except that caused by tuberculosis or sexually transmitted disease)
201	M868X0	Infective arthritis and osteomyelitis (except that caused by tuberculosis or sexually transmitted disease)
201	M868X1	Infective arthritis and osteomyelitis (except that caused by tuberculosis or sexually transmitted disease)
201	M868X2	Infective arthritis and osteomyelitis (except that caused by tuberculosis or sexually transmitted disease)
201	M868X3	Infective arthritis and osteomyelitis (except that caused by tuberculosis or sexually transmitted disease)
201	M868X4	Infective arthritis and osteomyelitis (except that caused by tuberculosis or sexually transmitted disease)
201	M868X5	Infective arthritis and osteomyelitis (except that caused by tuberculosis or sexually transmitted disease)
201	M868X6	Infective arthritis and osteomyelitis (except that caused by tuberculosis or sexually transmitted disease)
201	M868X7	Infective arthritis and osteomyelitis (except that caused by tuberculosis or sexually transmitted disease)
201	M868X8	Infective arthritis and osteomyelitis (except that caused by tuberculosis or sexually transmitted disease)
201	M868X9	Infective arthritis and osteomyelitis (except that caused by tuberculosis or sexually transmitted disease)
201	M869	Infective arthritis and osteomyelitis (except that caused by tuberculosis or sexually transmitted disease)
201	M8960	Infective arthritis and osteomyelitis (except that caused by tuberculosis or sexually transmitted disease)
201	M89611	Infective arthritis and osteomyelitis (except that caused by tuberculosis or sexually transmitted disease)
201	M89612	Infective arthritis and osteomyelitis (except that caused by tuberculosis or sexually transmitted disease)
201	M89619	Infective arthritis and osteomyelitis (except that caused by tuberculosis or sexually transmitted disease)
201	M89621	Infective arthritis and osteomyelitis (except that caused by tuberculosis or sexually transmitted disease)
201	M89622	Infective arthritis and osteomyelitis (except that caused by tuberculosis or sexually transmitted disease)
201	M89629	Infective arthritis and osteomyelitis (except that caused by tuberculosis or sexually transmitted disease)
201	M89631	Infective arthritis and osteomyelitis (except that caused by tuberculosis or sexually transmitted disease)
201	M89632	Infective arthritis and osteomyelitis (except that caused by tuberculosis or sexually transmitted disease)
201	M89639	Infective arthritis and osteomyelitis (except that caused by tuberculosis or sexually transmitted disease)
201	M89641	Infective arthritis and osteomyelitis (except that caused by tuberculosis or sexually transmitted disease)
201	M89642	Infective arthritis and osteomyelitis (except that caused by tuberculosis or sexually transmitted disease)
201	M89649	Infective arthritis and osteomyelitis (except that caused by tuberculosis or sexually transmitted disease)
201	M89651	Infective arthritis and osteomyelitis (except that caused by tuberculosis or sexually transmitted disease)
201	M89652	Infective arthritis and osteomyelitis (except that caused by tuberculosis or sexually transmitted disease)
201	M89659	Infective arthritis and osteomyelitis (except that caused by tuberculosis or sexually transmitted disease)
201	M89661	Infective arthritis and osteomyelitis (except that caused by tuberculosis or sexually transmitted disease)
201	M89662	Infective arthritis and osteomyelitis (except that caused by tuberculosis or sexually transmitted disease)
201	M89669	Infective arthritis and osteomyelitis (except that caused by tuberculosis or sexually transmitted disease)
201	M89671	Infective arthritis and osteomyelitis (except that caused by tuberculosis or sexually transmitted disease)
201	M89672	Infective arthritis and osteomyelitis (except that caused by tuberculosis or sexually transmitted disease)
201	M89679	Infective arthritis and osteomyelitis (except that caused by tuberculosis or sexually transmitted disease)
201	M8968	Infective arthritis and osteomyelitis (except that caused by tuberculosis or sexually transmitted disease)
201	M8969	Infective arthritis and osteomyelitis (except that caused by tuberculosis or sexually transmitted disease)
202	M0500	Rheumatoid arthritis and related disease
202	M05011	Rheumatoid arthritis and related disease
202	M05012	Rheumatoid arthritis and related disease
202	M05019	Rheumatoid arthritis and related disease
202	M05021	Rheumatoid arthritis and related disease
202	M05022	Rheumatoid arthritis and related disease
202	M05029	Rheumatoid arthritis and related disease
202	M05031	Rheumatoid arthritis and related disease
202	M05032	Rheumatoid arthritis and related disease
202	M05039	Rheumatoid arthritis and related disease

202	M05041	Rheumatoid arthritis and related disease
202	M05042	Rheumatoid arthritis and related disease
202	M05049	Rheumatoid arthritis and related disease
202	M05051	Rheumatoid arthritis and related disease
202	M05052	Rheumatoid arthritis and related disease
202	M05059	Rheumatoid arthritis and related disease
202	M05061	Rheumatoid arthritis and related disease
202	M05062	Rheumatoid arthritis and related disease
202	M05069	Rheumatoid arthritis and related disease
202	M05071	Rheumatoid arthritis and related disease
202	M05072	Rheumatoid arthritis and related disease
202	M05079	Rheumatoid arthritis and related disease
202	M0509	Rheumatoid arthritis and related disease
202	M0510	Rheumatoid arthritis and related disease
202	M05111	Rheumatoid arthritis and related disease
202	M05112	Rheumatoid arthritis and related disease
202	M05119	Rheumatoid arthritis and related disease
202	M05121	Rheumatoid arthritis and related disease
202	M05122	Rheumatoid arthritis and related disease
202	M05129	Rheumatoid arthritis and related disease
202	M05131	Rheumatoid arthritis and related disease
202	M05132	Rheumatoid arthritis and related disease
202	M05139	Rheumatoid arthritis and related disease
202	M05141	Rheumatoid arthritis and related disease
202	M05142	Rheumatoid arthritis and related disease
202	M05149	Rheumatoid arthritis and related disease
202	M05151	Rheumatoid arthritis and related disease
202	M05152	Rheumatoid arthritis and related disease
202	M05159	Rheumatoid arthritis and related disease
202	M05161	Rheumatoid arthritis and related disease
202	M05162	Rheumatoid arthritis and related disease
202	M05169	Rheumatoid arthritis and related disease
202	M05171	Rheumatoid arthritis and related disease
202	M05172	Rheumatoid arthritis and related disease
202	M05179	Rheumatoid arthritis and related disease
202	M0519	Rheumatoid arthritis and related disease
202	M0520	Rheumatoid arthritis and related disease
202	M05211	Rheumatoid arthritis and related disease
202	M05212	Rheumatoid arthritis and related disease
202	M05219	Rheumatoid arthritis and related disease
202	M05221	Rheumatoid arthritis and related disease
202	M05222	Rheumatoid arthritis and related disease
202	M05229	Rheumatoid arthritis and related disease
202	M05231	Rheumatoid arthritis and related disease
202	M05232	Rheumatoid arthritis and related disease
202	M05239	Rheumatoid arthritis and related disease
202	M05241	Rheumatoid arthritis and related disease
202	M05242	Rheumatoid arthritis and related disease
202	M05249	Rheumatoid arthritis and related disease
202	M05251	Rheumatoid arthritis and related disease
202	M05252	Rheumatoid arthritis and related disease

202	M05259	Rheumatoid arthritis and related disease
202	M05261	Rheumatoid arthritis and related disease
202	M05262	Rheumatoid arthritis and related disease
202	M05269	Rheumatoid arthritis and related disease
202	M05271	Rheumatoid arthritis and related disease
202	M05272	Rheumatoid arthritis and related disease
202	M05279	Rheumatoid arthritis and related disease
202	M0529	Rheumatoid arthritis and related disease
202	M0530	Rheumatoid arthritis and related disease
202	M05311	Rheumatoid arthritis and related disease
202	M05312	Rheumatoid arthritis and related disease
202	M05319	Rheumatoid arthritis and related disease
202	M05321	Rheumatoid arthritis and related disease
202	M05322	Rheumatoid arthritis and related disease
202	M05329	Rheumatoid arthritis and related disease
202	M05331	Rheumatoid arthritis and related disease
202	M05332	Rheumatoid arthritis and related disease
202	M05339	Rheumatoid arthritis and related disease
202	M05341	Rheumatoid arthritis and related disease
202	M05342	Rheumatoid arthritis and related disease
202	M05349	Rheumatoid arthritis and related disease
202	M05351	Rheumatoid arthritis and related disease
202	M05352	Rheumatoid arthritis and related disease
202	M05359	Rheumatoid arthritis and related disease
202	M05361	Rheumatoid arthritis and related disease
202	M05362	Rheumatoid arthritis and related disease
202	M05369	Rheumatoid arthritis and related disease
202	M05371	Rheumatoid arthritis and related disease
202	M05372	Rheumatoid arthritis and related disease
202	M05379	Rheumatoid arthritis and related disease
202	M0539	Rheumatoid arthritis and related disease
202	M0540	Rheumatoid arthritis and related disease
202	M05411	Rheumatoid arthritis and related disease
202	M05412	Rheumatoid arthritis and related disease
202	M05419	Rheumatoid arthritis and related disease
202	M05421	Rheumatoid arthritis and related disease
202	M05422	Rheumatoid arthritis and related disease
202	M05429	Rheumatoid arthritis and related disease
202	M05431	Rheumatoid arthritis and related disease
202	M05432	Rheumatoid arthritis and related disease
202	M05439	Rheumatoid arthritis and related disease
202	M05441	Rheumatoid arthritis and related disease
202	M05442	Rheumatoid arthritis and related disease
202	M05449	Rheumatoid arthritis and related disease
202	M05451	Rheumatoid arthritis and related disease
202	M05452	Rheumatoid arthritis and related disease
202	M05459	Rheumatoid arthritis and related disease
202	M05461	Rheumatoid arthritis and related disease
202	M05462	Rheumatoid arthritis and related disease
202	M05469	Rheumatoid arthritis and related disease
202	M05471	Rheumatoid arthritis and related disease

202	M05472	Rheumatoid arthritis and related disease
202	M05479	Rheumatoid arthritis and related disease
202	M0549	Rheumatoid arthritis and related disease
202	M0550	Rheumatoid arthritis and related disease
202	M05511	Rheumatoid arthritis and related disease
202	M05512	Rheumatoid arthritis and related disease
202	M05519	Rheumatoid arthritis and related disease
202	M05521	Rheumatoid arthritis and related disease
202	M05522	Rheumatoid arthritis and related disease
202	M05529	Rheumatoid arthritis and related disease
202	M05531	Rheumatoid arthritis and related disease
202	M05532	Rheumatoid arthritis and related disease
202	M05539	Rheumatoid arthritis and related disease
202	M05541	Rheumatoid arthritis and related disease
202	M05542	Rheumatoid arthritis and related disease
202	M05549	Rheumatoid arthritis and related disease
202	M05551	Rheumatoid arthritis and related disease
202	M05552	Rheumatoid arthritis and related disease
202	M05559	Rheumatoid arthritis and related disease
202	M05561	Rheumatoid arthritis and related disease
202	M05562	Rheumatoid arthritis and related disease
202	M05569	Rheumatoid arthritis and related disease
202	M05571	Rheumatoid arthritis and related disease
202	M05572	Rheumatoid arthritis and related disease
202	M05579	Rheumatoid arthritis and related disease
202	M0559	Rheumatoid arthritis and related disease
202	M0560	Rheumatoid arthritis and related disease
202	M05611	Rheumatoid arthritis and related disease
202	M05612	Rheumatoid arthritis and related disease
202	M05619	Rheumatoid arthritis and related disease
202	M05621	Rheumatoid arthritis and related disease
202	M05622	Rheumatoid arthritis and related disease
202	M05629	Rheumatoid arthritis and related disease
202	M05631	Rheumatoid arthritis and related disease
202	M05632	Rheumatoid arthritis and related disease
202	M05639	Rheumatoid arthritis and related disease
202	M05641	Rheumatoid arthritis and related disease
202	M05642	Rheumatoid arthritis and related disease
202	M05649	Rheumatoid arthritis and related disease
202	M05651	Rheumatoid arthritis and related disease
202	M05652	Rheumatoid arthritis and related disease
202	M05659	Rheumatoid arthritis and related disease
202	M05661	Rheumatoid arthritis and related disease
202	M05662	Rheumatoid arthritis and related disease
202	M05669	Rheumatoid arthritis and related disease
202	M05671	Rheumatoid arthritis and related disease
202	M05672	Rheumatoid arthritis and related disease
202	M05679	Rheumatoid arthritis and related disease
202	M0569	Rheumatoid arthritis and related disease
202	M0570	Rheumatoid arthritis and related disease
202	M05711	Rheumatoid arthritis and related disease

202	M05712	Rheumatoid arthritis and related disease
202	M05719	Rheumatoid arthritis and related disease
202	M05721	Rheumatoid arthritis and related disease
202	M05722	Rheumatoid arthritis and related disease
202	M05729	Rheumatoid arthritis and related disease
202	M05731	Rheumatoid arthritis and related disease
202	M05732	Rheumatoid arthritis and related disease
202	M05739	Rheumatoid arthritis and related disease
202	M05741	Rheumatoid arthritis and related disease
202	M05742	Rheumatoid arthritis and related disease
202	M05749	Rheumatoid arthritis and related disease
202	M05751	Rheumatoid arthritis and related disease
202	M05752	Rheumatoid arthritis and related disease
202	M05759	Rheumatoid arthritis and related disease
202	M05761	Rheumatoid arthritis and related disease
202	M05762	Rheumatoid arthritis and related disease
202	M05769	Rheumatoid arthritis and related disease
202	M05771	Rheumatoid arthritis and related disease
202	M05772	Rheumatoid arthritis and related disease
202	M05779	Rheumatoid arthritis and related disease
202	M0579	Rheumatoid arthritis and related disease
202	M0580	Rheumatoid arthritis and related disease
202	M05811	Rheumatoid arthritis and related disease
202	M05812	Rheumatoid arthritis and related disease
202	M05819	Rheumatoid arthritis and related disease
202	M05821	Rheumatoid arthritis and related disease
202	M05822	Rheumatoid arthritis and related disease
202	M05829	Rheumatoid arthritis and related disease
202	M05831	Rheumatoid arthritis and related disease
202	M05832	Rheumatoid arthritis and related disease
202	M05839	Rheumatoid arthritis and related disease
202	M05841	Rheumatoid arthritis and related disease
202	M05842	Rheumatoid arthritis and related disease
202	M05849	Rheumatoid arthritis and related disease
202	M05851	Rheumatoid arthritis and related disease
202	M05852	Rheumatoid arthritis and related disease
202	M05859	Rheumatoid arthritis and related disease
202	M05861	Rheumatoid arthritis and related disease
202	M05862	Rheumatoid arthritis and related disease
202	M05869	Rheumatoid arthritis and related disease
202	M05871	Rheumatoid arthritis and related disease
202	M05872	Rheumatoid arthritis and related disease
202	M05879	Rheumatoid arthritis and related disease
202	M0589	Rheumatoid arthritis and related disease
202	M059	Rheumatoid arthritis and related disease
202	M0600	Rheumatoid arthritis and related disease
202	M06011	Rheumatoid arthritis and related disease
202	M06012	Rheumatoid arthritis and related disease
202	M06019	Rheumatoid arthritis and related disease
202	M06021	Rheumatoid arthritis and related disease
202	M06022	Rheumatoid arthritis and related disease

202	M06029	Rheumatoid arthritis and related disease
202	M06031	Rheumatoid arthritis and related disease
202	M06032	Rheumatoid arthritis and related disease
202	M06039	Rheumatoid arthritis and related disease
202	M06041	Rheumatoid arthritis and related disease
202	M06042	Rheumatoid arthritis and related disease
202	M06049	Rheumatoid arthritis and related disease
202	M06051	Rheumatoid arthritis and related disease
202	M06052	Rheumatoid arthritis and related disease
202	M06059	Rheumatoid arthritis and related disease
202	M06061	Rheumatoid arthritis and related disease
202	M06062	Rheumatoid arthritis and related disease
202	M06069	Rheumatoid arthritis and related disease
202	M06071	Rheumatoid arthritis and related disease
202	M06072	Rheumatoid arthritis and related disease
202	M06079	Rheumatoid arthritis and related disease
202	M0608	Rheumatoid arthritis and related disease
202	M0609	Rheumatoid arthritis and related disease
202	M061	Rheumatoid arthritis and related disease
202	M0620	Rheumatoid arthritis and related disease
202	M06211	Rheumatoid arthritis and related disease
202	M06212	Rheumatoid arthritis and related disease
202	M06219	Rheumatoid arthritis and related disease
202	M06221	Rheumatoid arthritis and related disease
202	M06222	Rheumatoid arthritis and related disease
202	M06229	Rheumatoid arthritis and related disease
202	M06231	Rheumatoid arthritis and related disease
202	M06232	Rheumatoid arthritis and related disease
202	M06239	Rheumatoid arthritis and related disease
202	M06241	Rheumatoid arthritis and related disease
202	M06242	Rheumatoid arthritis and related disease
202	M06249	Rheumatoid arthritis and related disease
202	M06251	Rheumatoid arthritis and related disease
202	M06252	Rheumatoid arthritis and related disease
202	M06259	Rheumatoid arthritis and related disease
202	M06261	Rheumatoid arthritis and related disease
202	M06262	Rheumatoid arthritis and related disease
202	M06269	Rheumatoid arthritis and related disease
202	M06271	Rheumatoid arthritis and related disease
202	M06272	Rheumatoid arthritis and related disease
202	M06279	Rheumatoid arthritis and related disease
202	M0628	Rheumatoid arthritis and related disease
202	M0629	Rheumatoid arthritis and related disease
202	M0630	Rheumatoid arthritis and related disease
202	M06311	Rheumatoid arthritis and related disease
202	M06312	Rheumatoid arthritis and related disease
202	M06319	Rheumatoid arthritis and related disease
202	M06321	Rheumatoid arthritis and related disease
202	M06322	Rheumatoid arthritis and related disease
202	M06329	Rheumatoid arthritis and related disease
202	M06331	Rheumatoid arthritis and related disease

202	M06332	Rheumatoid arthritis and related disease
202	M06339	Rheumatoid arthritis and related disease
202	M06341	Rheumatoid arthritis and related disease
202	M06342	Rheumatoid arthritis and related disease
202	M06349	Rheumatoid arthritis and related disease
202	M06351	Rheumatoid arthritis and related disease
202	M06352	Rheumatoid arthritis and related disease
202	M06359	Rheumatoid arthritis and related disease
202	M06361	Rheumatoid arthritis and related disease
202	M06362	Rheumatoid arthritis and related disease
202	M06369	Rheumatoid arthritis and related disease
202	M06371	Rheumatoid arthritis and related disease
202	M06372	Rheumatoid arthritis and related disease
202	M06379	Rheumatoid arthritis and related disease
202	M0638	Rheumatoid arthritis and related disease
202	M0639	Rheumatoid arthritis and related disease
202	M064	Rheumatoid arthritis and related disease
202	M0680	Rheumatoid arthritis and related disease
202	M06811	Rheumatoid arthritis and related disease
202	M06812	Rheumatoid arthritis and related disease
202	M06819	Rheumatoid arthritis and related disease
202	M06821	Rheumatoid arthritis and related disease
202	M06822	Rheumatoid arthritis and related disease
202	M06829	Rheumatoid arthritis and related disease
202	M06831	Rheumatoid arthritis and related disease
202	M06832	Rheumatoid arthritis and related disease
202	M06839	Rheumatoid arthritis and related disease
202	M06841	Rheumatoid arthritis and related disease
202	M06842	Rheumatoid arthritis and related disease
202	M06849	Rheumatoid arthritis and related disease
202	M06851	Rheumatoid arthritis and related disease
202	M06852	Rheumatoid arthritis and related disease
202	M06859	Rheumatoid arthritis and related disease
202	M06861	Rheumatoid arthritis and related disease
202	M06862	Rheumatoid arthritis and related disease
202	M06869	Rheumatoid arthritis and related disease
202	M06871	Rheumatoid arthritis and related disease
202	M06872	Rheumatoid arthritis and related disease
202	M06879	Rheumatoid arthritis and related disease
202	M0688	Rheumatoid arthritis and related disease
202	M0689	Rheumatoid arthritis and related disease
202	M069	Rheumatoid arthritis and related disease
202	M0800	Rheumatoid arthritis and related disease
202	M08011	Rheumatoid arthritis and related disease
202	M08012	Rheumatoid arthritis and related disease
202	M08019	Rheumatoid arthritis and related disease
202	M08021	Rheumatoid arthritis and related disease
202	M08022	Rheumatoid arthritis and related disease
202	M08029	Rheumatoid arthritis and related disease
202	M08031	Rheumatoid arthritis and related disease
202	M08032	Rheumatoid arthritis and related disease

202	M08039	Rheumatoid arthritis and related disease
202	M08041	Rheumatoid arthritis and related disease
202	M08042	Rheumatoid arthritis and related disease
202	M08049	Rheumatoid arthritis and related disease
202	M08051	Rheumatoid arthritis and related disease
202	M08052	Rheumatoid arthritis and related disease
202	M08059	Rheumatoid arthritis and related disease
202	M08061	Rheumatoid arthritis and related disease
202	M08062	Rheumatoid arthritis and related disease
202	M08069	Rheumatoid arthritis and related disease
202	M08071	Rheumatoid arthritis and related disease
202	M08072	Rheumatoid arthritis and related disease
202	M08079	Rheumatoid arthritis and related disease
202	M0808	Rheumatoid arthritis and related disease
202	M0809	Rheumatoid arthritis and related disease
202	M081	Rheumatoid arthritis and related disease
202	M0820	Rheumatoid arthritis and related disease
202	M08211	Rheumatoid arthritis and related disease
202	M08212	Rheumatoid arthritis and related disease
202	M08219	Rheumatoid arthritis and related disease
202	M08221	Rheumatoid arthritis and related disease
202	M08222	Rheumatoid arthritis and related disease
202	M08229	Rheumatoid arthritis and related disease
202	M08231	Rheumatoid arthritis and related disease
202	M08232	Rheumatoid arthritis and related disease
202	M08239	Rheumatoid arthritis and related disease
202	M08241	Rheumatoid arthritis and related disease
202	M08242	Rheumatoid arthritis and related disease
202	M08249	Rheumatoid arthritis and related disease
202	M08251	Rheumatoid arthritis and related disease
202	M08252	Rheumatoid arthritis and related disease
202	M08259	Rheumatoid arthritis and related disease
202	M08261	Rheumatoid arthritis and related disease
202	M08262	Rheumatoid arthritis and related disease
202	M08269	Rheumatoid arthritis and related disease
202	M08271	Rheumatoid arthritis and related disease
202	M08272	Rheumatoid arthritis and related disease
202	M08279	Rheumatoid arthritis and related disease
202	M0828	Rheumatoid arthritis and related disease
202	M0829	Rheumatoid arthritis and related disease
202	M083	Rheumatoid arthritis and related disease
202	M0840	Rheumatoid arthritis and related disease
202	M08411	Rheumatoid arthritis and related disease
202	M08412	Rheumatoid arthritis and related disease
202	M08419	Rheumatoid arthritis and related disease
202	M08421	Rheumatoid arthritis and related disease
202	M08422	Rheumatoid arthritis and related disease
202	M08429	Rheumatoid arthritis and related disease
202	M08431	Rheumatoid arthritis and related disease
202	M08432	Rheumatoid arthritis and related disease
202	M08439	Rheumatoid arthritis and related disease

202	M08441	Rheumatoid arthritis and related disease
202	M08442	Rheumatoid arthritis and related disease
202	M08449	Rheumatoid arthritis and related disease
202	M08451	Rheumatoid arthritis and related disease
202	M08452	Rheumatoid arthritis and related disease
202	M08459	Rheumatoid arthritis and related disease
202	M08461	Rheumatoid arthritis and related disease
202	M08462	Rheumatoid arthritis and related disease
202	M08469	Rheumatoid arthritis and related disease
202	M08471	Rheumatoid arthritis and related disease
202	M08472	Rheumatoid arthritis and related disease
202	M08479	Rheumatoid arthritis and related disease
202	M0848	Rheumatoid arthritis and related disease
202	M0880	Rheumatoid arthritis and related disease
202	M08811	Rheumatoid arthritis and related disease
202	M08812	Rheumatoid arthritis and related disease
202	M08819	Rheumatoid arthritis and related disease
202	M08821	Rheumatoid arthritis and related disease
202	M08822	Rheumatoid arthritis and related disease
202	M08829	Rheumatoid arthritis and related disease
202	M08831	Rheumatoid arthritis and related disease
202	M08832	Rheumatoid arthritis and related disease
202	M08839	Rheumatoid arthritis and related disease
202	M08841	Rheumatoid arthritis and related disease
202	M08842	Rheumatoid arthritis and related disease
202	M08849	Rheumatoid arthritis and related disease
202	M08851	Rheumatoid arthritis and related disease
202	M08852	Rheumatoid arthritis and related disease
202	M08859	Rheumatoid arthritis and related disease
202	M08861	Rheumatoid arthritis and related disease
202	M08862	Rheumatoid arthritis and related disease
202	M08869	Rheumatoid arthritis and related disease
202	M08871	Rheumatoid arthritis and related disease
202	M08872	Rheumatoid arthritis and related disease
202	M08879	Rheumatoid arthritis and related disease
202	M0888	Rheumatoid arthritis and related disease
202	M0889	Rheumatoid arthritis and related disease
202	M0890	Rheumatoid arthritis and related disease
202	M08911	Rheumatoid arthritis and related disease
202	M08912	Rheumatoid arthritis and related disease
202	M08919	Rheumatoid arthritis and related disease
202	M08921	Rheumatoid arthritis and related disease
202	M08922	Rheumatoid arthritis and related disease
202	M08929	Rheumatoid arthritis and related disease
202	M08931	Rheumatoid arthritis and related disease
202	M08932	Rheumatoid arthritis and related disease
202	M08939	Rheumatoid arthritis and related disease
202	M08941	Rheumatoid arthritis and related disease
202	M08942	Rheumatoid arthritis and related disease
202	M08949	Rheumatoid arthritis and related disease
202	M08951	Rheumatoid arthritis and related disease

202	M08952	Rheumatoid arthritis and related disease
202	M08959	Rheumatoid arthritis and related disease
202	M08961	Rheumatoid arthritis and related disease
202	M08962	Rheumatoid arthritis and related disease
202	M08969	Rheumatoid arthritis and related disease
202	M08971	Rheumatoid arthritis and related disease
202	M08972	Rheumatoid arthritis and related disease
202	M08979	Rheumatoid arthritis and related disease
202	M0898	Rheumatoid arthritis and related disease
202	M0899	Rheumatoid arthritis and related disease
202	M1200	Rheumatoid arthritis and related disease
202	M12011	Rheumatoid arthritis and related disease
202	M12012	Rheumatoid arthritis and related disease
202	M12019	Rheumatoid arthritis and related disease
202	M12021	Rheumatoid arthritis and related disease
202	M12022	Rheumatoid arthritis and related disease
202	M12029	Rheumatoid arthritis and related disease
202	M12031	Rheumatoid arthritis and related disease
202	M12032	Rheumatoid arthritis and related disease
202	M12039	Rheumatoid arthritis and related disease
202	M12041	Rheumatoid arthritis and related disease
202	M12042	Rheumatoid arthritis and related disease
202	M12049	Rheumatoid arthritis and related disease
202	M12051	Rheumatoid arthritis and related disease
202	M12052	Rheumatoid arthritis and related disease
202	M12059	Rheumatoid arthritis and related disease
202	M12061	Rheumatoid arthritis and related disease
202	M12062	Rheumatoid arthritis and related disease
202	M12069	Rheumatoid arthritis and related disease
202	M12071	Rheumatoid arthritis and related disease
202	M12072	Rheumatoid arthritis and related disease
202	M12079	Rheumatoid arthritis and related disease
202	M1208	Rheumatoid arthritis and related disease
202	M1209	Rheumatoid arthritis and related disease
202	M450	Rheumatoid arthritis and related disease
202	M451	Rheumatoid arthritis and related disease
202	M452	Rheumatoid arthritis and related disease
202	M453	Rheumatoid arthritis and related disease
202	M454	Rheumatoid arthritis and related disease
202	M455	Rheumatoid arthritis and related disease
202	M456	Rheumatoid arthritis and related disease
202	M457	Rheumatoid arthritis and related disease
202	M458	Rheumatoid arthritis and related disease
202	M459	Rheumatoid arthritis and related disease
202	M488X1	Rheumatoid arthritis and related disease
202	M488X2	Rheumatoid arthritis and related disease
202	M488X3	Rheumatoid arthritis and related disease
202	M488X4	Rheumatoid arthritis and related disease
202	M488X5	Rheumatoid arthritis and related disease
202	M488X6	Rheumatoid arthritis and related disease
202	M488X7	Rheumatoid arthritis and related disease

202	M488X8	Rheumatoid arthritis and related disease
202	M488X9	Rheumatoid arthritis and related disease
203	M150	Osteoarthritis
203	M151	Osteoarthritis
203	M152	Osteoarthritis
203	M153	Osteoarthritis
203	M154	Osteoarthritis
203	M158	Osteoarthritis
203	M159	Osteoarthritis
203	M160	Osteoarthritis
203	M1610	Osteoarthritis
203	M1611	Osteoarthritis
203	M1612	Osteoarthritis
203	M162	Osteoarthritis
203	M1630	Osteoarthritis
203	M1631	Osteoarthritis
203	M1632	Osteoarthritis
203	M164	Osteoarthritis
203	M1650	Osteoarthritis
203	M1651	Osteoarthritis
203	M1652	Osteoarthritis
203	M166	Osteoarthritis
203	M167	Osteoarthritis
203	M169	Osteoarthritis
203	M170	Osteoarthritis
203	M1710	Osteoarthritis
203	M1711	Osteoarthritis
203	M1712	Osteoarthritis
203	M172	Osteoarthritis
203	M1730	Osteoarthritis
203	M1731	Osteoarthritis
203	M1732	Osteoarthritis
203	M174	Osteoarthritis
203	M175	Osteoarthritis
203	M179	Osteoarthritis
203	M180	Osteoarthritis
203	M1810	Osteoarthritis
203	M1811	Osteoarthritis
203	M1812	Osteoarthritis
203	M182	Osteoarthritis
203	M1830	Osteoarthritis
203	M1831	Osteoarthritis
203	M1832	Osteoarthritis
203	M184	Osteoarthritis
203	M1850	Osteoarthritis
203	M1851	Osteoarthritis
203	M1852	Osteoarthritis
203	M189	Osteoarthritis
203	M19011	Osteoarthritis
203	M19012	Osteoarthritis
203	M19019	Osteoarthritis

203	M19021	Osteoarthritis
203	M19022	Osteoarthritis
203	M19029	Osteoarthritis
203	M19031	Osteoarthritis
203	M19032	Osteoarthritis
203	M19039	Osteoarthritis
203	M19041	Osteoarthritis
203	M19042	Osteoarthritis
203	M19049	Osteoarthritis
203	M19071	Osteoarthritis
203	M19072	Osteoarthritis
203	M19079	Osteoarthritis
203	M19111	Osteoarthritis
203	M19112	Osteoarthritis
203	M19119	Osteoarthritis
203	M19121	Osteoarthritis
203	M19122	Osteoarthritis
203	M19129	Osteoarthritis
203	M19131	Osteoarthritis
203	M19132	Osteoarthritis
203	M19139	Osteoarthritis
203	M19141	Osteoarthritis
203	M19142	Osteoarthritis
203	M19149	Osteoarthritis
203	M19171	Osteoarthritis
203	M19172	Osteoarthritis
203	M19179	Osteoarthritis
203	M19211	Osteoarthritis
203	M19212	Osteoarthritis
203	M19219	Osteoarthritis
203	M19221	Osteoarthritis
203	M19222	Osteoarthritis
203	M19229	Osteoarthritis
203	M19231	Osteoarthritis
203	M19232	Osteoarthritis
203	M19239	Osteoarthritis
203	M19241	Osteoarthritis
203	M19242	Osteoarthritis
203	M19249	Osteoarthritis
203	M19271	Osteoarthritis
203	M19272	Osteoarthritis
203	M19279	Osteoarthritis
203	M1990	Osteoarthritis
203	M1991	Osteoarthritis
203	M1992	Osteoarthritis
203	M1993	Osteoarthritis
204	M0200	Other non-traumatic joint disorders
204	M02011	Other non-traumatic joint disorders
204	M02012	Other non-traumatic joint disorders
204	M02019	Other non-traumatic joint disorders
204	M02021	Other non-traumatic joint disorders

204	M02022	Other non-traumatic joint disorders
204	M02029	Other non-traumatic joint disorders
204	M02031	Other non-traumatic joint disorders
204	M02032	Other non-traumatic joint disorders
204	M02039	Other non-traumatic joint disorders
204	M02041	Other non-traumatic joint disorders
204	M02042	Other non-traumatic joint disorders
204	M02049	Other non-traumatic joint disorders
204	M02051	Other non-traumatic joint disorders
204	M02052	Other non-traumatic joint disorders
204	M02059	Other non-traumatic joint disorders
204	M02061	Other non-traumatic joint disorders
204	M02062	Other non-traumatic joint disorders
204	M02069	Other non-traumatic joint disorders
204	M02071	Other non-traumatic joint disorders
204	M02072	Other non-traumatic joint disorders
204	M02079	Other non-traumatic joint disorders
204	M0208	Other non-traumatic joint disorders
204	M0209	Other non-traumatic joint disorders
204	M0220	Other non-traumatic joint disorders
204	M02211	Other non-traumatic joint disorders
204	M02212	Other non-traumatic joint disorders
204	M02219	Other non-traumatic joint disorders
204	M02221	Other non-traumatic joint disorders
204	M02222	Other non-traumatic joint disorders
204	M02229	Other non-traumatic joint disorders
204	M02231	Other non-traumatic joint disorders
204	M02232	Other non-traumatic joint disorders
204	M02239	Other non-traumatic joint disorders
204	M02241	Other non-traumatic joint disorders
204	M02242	Other non-traumatic joint disorders
204	M02249	Other non-traumatic joint disorders
204	M02251	Other non-traumatic joint disorders
204	M02252	Other non-traumatic joint disorders
204	M02259	Other non-traumatic joint disorders
204	M02261	Other non-traumatic joint disorders
204	M02262	Other non-traumatic joint disorders
204	M02269	Other non-traumatic joint disorders
204	M02271	Other non-traumatic joint disorders
204	M02272	Other non-traumatic joint disorders
204	M02279	Other non-traumatic joint disorders
204	M0228	Other non-traumatic joint disorders
204	M0229	Other non-traumatic joint disorders
204	M0760	Other non-traumatic joint disorders
204	M07611	Other non-traumatic joint disorders
204	M07612	Other non-traumatic joint disorders
204	M07619	Other non-traumatic joint disorders
204	M07621	Other non-traumatic joint disorders
204	M07622	Other non-traumatic joint disorders
204	M07629	Other non-traumatic joint disorders
204	M07631	Other non-traumatic joint disorders

204	M07632	Other non-traumatic joint disorders
204	M07639	Other non-traumatic joint disorders
204	M07641	Other non-traumatic joint disorders
204	M07642	Other non-traumatic joint disorders
204	M07649	Other non-traumatic joint disorders
204	M07651	Other non-traumatic joint disorders
204	M07652	Other non-traumatic joint disorders
204	M07659	Other non-traumatic joint disorders
204	M07661	Other non-traumatic joint disorders
204	M07662	Other non-traumatic joint disorders
204	M07669	Other non-traumatic joint disorders
204	M07671	Other non-traumatic joint disorders
204	M07672	Other non-traumatic joint disorders
204	M07679	Other non-traumatic joint disorders
204	M0768	Other non-traumatic joint disorders
204	M0769	Other non-traumatic joint disorders
204	M1210	Other non-traumatic joint disorders
204	M12111	Other non-traumatic joint disorders
204	M12112	Other non-traumatic joint disorders
204	M12119	Other non-traumatic joint disorders
204	M12121	Other non-traumatic joint disorders
204	M12122	Other non-traumatic joint disorders
204	M12129	Other non-traumatic joint disorders
204	M12131	Other non-traumatic joint disorders
204	M12132	Other non-traumatic joint disorders
204	M12139	Other non-traumatic joint disorders
204	M12141	Other non-traumatic joint disorders
204	M12142	Other non-traumatic joint disorders
204	M12149	Other non-traumatic joint disorders
204	M12151	Other non-traumatic joint disorders
204	M12152	Other non-traumatic joint disorders
204	M12159	Other non-traumatic joint disorders
204	M12161	Other non-traumatic joint disorders
204	M12162	Other non-traumatic joint disorders
204	M12169	Other non-traumatic joint disorders
204	M12171	Other non-traumatic joint disorders
204	M12172	Other non-traumatic joint disorders
204	M12179	Other non-traumatic joint disorders
204	M1218	Other non-traumatic joint disorders
204	M1219	Other non-traumatic joint disorders
204	M1220	Other non-traumatic joint disorders
204	M12211	Other non-traumatic joint disorders
204	M12212	Other non-traumatic joint disorders
204	M12219	Other non-traumatic joint disorders
204	M12221	Other non-traumatic joint disorders
204	M12222	Other non-traumatic joint disorders
204	M12229	Other non-traumatic joint disorders
204	M12231	Other non-traumatic joint disorders
204	M12232	Other non-traumatic joint disorders
204	M12239	Other non-traumatic joint disorders
204	M12241	Other non-traumatic joint disorders

204	M12242	Other non-traumatic joint disorders
204	M12249	Other non-traumatic joint disorders
204	M12251	Other non-traumatic joint disorders
204	M12252	Other non-traumatic joint disorders
204	M12259	Other non-traumatic joint disorders
204	M12261	Other non-traumatic joint disorders
204	M12262	Other non-traumatic joint disorders
204	M12269	Other non-traumatic joint disorders
204	M12271	Other non-traumatic joint disorders
204	M12272	Other non-traumatic joint disorders
204	M12279	Other non-traumatic joint disorders
204	M1228	Other non-traumatic joint disorders
204	M1229	Other non-traumatic joint disorders
204	M1230	Other non-traumatic joint disorders
204	M12311	Other non-traumatic joint disorders
204	M12312	Other non-traumatic joint disorders
204	M12319	Other non-traumatic joint disorders
204	M12321	Other non-traumatic joint disorders
204	M12322	Other non-traumatic joint disorders
204	M12329	Other non-traumatic joint disorders
204	M12331	Other non-traumatic joint disorders
204	M12332	Other non-traumatic joint disorders
204	M12339	Other non-traumatic joint disorders
204	M12341	Other non-traumatic joint disorders
204	M12342	Other non-traumatic joint disorders
204	M12349	Other non-traumatic joint disorders
204	M12351	Other non-traumatic joint disorders
204	M12352	Other non-traumatic joint disorders
204	M12359	Other non-traumatic joint disorders
204	M12361	Other non-traumatic joint disorders
204	M12362	Other non-traumatic joint disorders
204	M12369	Other non-traumatic joint disorders
204	M12371	Other non-traumatic joint disorders
204	M12372	Other non-traumatic joint disorders
204	M12379	Other non-traumatic joint disorders
204	M1238	Other non-traumatic joint disorders
204	M1239	Other non-traumatic joint disorders
204	M1240	Other non-traumatic joint disorders
204	M12411	Other non-traumatic joint disorders
204	M12412	Other non-traumatic joint disorders
204	M12419	Other non-traumatic joint disorders
204	M12421	Other non-traumatic joint disorders
204	M12422	Other non-traumatic joint disorders
204	M12429	Other non-traumatic joint disorders
204	M12431	Other non-traumatic joint disorders
204	M12432	Other non-traumatic joint disorders
204	M12439	Other non-traumatic joint disorders
204	M12441	Other non-traumatic joint disorders
204	M12442	Other non-traumatic joint disorders
204	M12449	Other non-traumatic joint disorders
204	M12451	Other non-traumatic joint disorders

204	M12452	Other non-traumatic joint disorders
204	M12459	Other non-traumatic joint disorders
204	M12461	Other non-traumatic joint disorders
204	M12462	Other non-traumatic joint disorders
204	M12469	Other non-traumatic joint disorders
204	M12471	Other non-traumatic joint disorders
204	M12472	Other non-traumatic joint disorders
204	M12479	Other non-traumatic joint disorders
204	M1248	Other non-traumatic joint disorders
204	M1249	Other non-traumatic joint disorders
204	M1280	Other non-traumatic joint disorders
204	M12811	Other non-traumatic joint disorders
204	M12812	Other non-traumatic joint disorders
204	M12819	Other non-traumatic joint disorders
204	M12821	Other non-traumatic joint disorders
204	M12822	Other non-traumatic joint disorders
204	M12829	Other non-traumatic joint disorders
204	M12831	Other non-traumatic joint disorders
204	M12832	Other non-traumatic joint disorders
204	M12839	Other non-traumatic joint disorders
204	M12841	Other non-traumatic joint disorders
204	M12842	Other non-traumatic joint disorders
204	M12849	Other non-traumatic joint disorders
204	M12851	Other non-traumatic joint disorders
204	M12852	Other non-traumatic joint disorders
204	M12859	Other non-traumatic joint disorders
204	M12861	Other non-traumatic joint disorders
204	M12862	Other non-traumatic joint disorders
204	M12869	Other non-traumatic joint disorders
204	M12871	Other non-traumatic joint disorders
204	M12872	Other non-traumatic joint disorders
204	M12879	Other non-traumatic joint disorders
204	M1288	Other non-traumatic joint disorders
204	M1289	Other non-traumatic joint disorders
204	M129	Other non-traumatic joint disorders
204	M130	Other non-traumatic joint disorders
204	M1310	Other non-traumatic joint disorders
204	M13111	Other non-traumatic joint disorders
204	M13112	Other non-traumatic joint disorders
204	M13119	Other non-traumatic joint disorders
204	M13121	Other non-traumatic joint disorders
204	M13122	Other non-traumatic joint disorders
204	M13129	Other non-traumatic joint disorders
204	M13131	Other non-traumatic joint disorders
204	M13132	Other non-traumatic joint disorders
204	M13139	Other non-traumatic joint disorders
204	M13141	Other non-traumatic joint disorders
204	M13142	Other non-traumatic joint disorders
204	M13149	Other non-traumatic joint disorders
204	M13151	Other non-traumatic joint disorders
204	M13152	Other non-traumatic joint disorders

204	M13159	Other non-traumatic joint disorders
204	M13161	Other non-traumatic joint disorders
204	M13162	Other non-traumatic joint disorders
204	M13169	Other non-traumatic joint disorders
204	M13171	Other non-traumatic joint disorders
204	M13172	Other non-traumatic joint disorders
204	M13179	Other non-traumatic joint disorders
204	M1380	Other non-traumatic joint disorders
204	M13811	Other non-traumatic joint disorders
204	M13812	Other non-traumatic joint disorders
204	M13819	Other non-traumatic joint disorders
204	M13821	Other non-traumatic joint disorders
204	M13822	Other non-traumatic joint disorders
204	M13829	Other non-traumatic joint disorders
204	M13831	Other non-traumatic joint disorders
204	M13832	Other non-traumatic joint disorders
204	M13839	Other non-traumatic joint disorders
204	M13841	Other non-traumatic joint disorders
204	M13842	Other non-traumatic joint disorders
204	M13849	Other non-traumatic joint disorders
204	M13851	Other non-traumatic joint disorders
204	M13852	Other non-traumatic joint disorders
204	M13859	Other non-traumatic joint disorders
204	M13861	Other non-traumatic joint disorders
204	M13862	Other non-traumatic joint disorders
204	M13869	Other non-traumatic joint disorders
204	M13871	Other non-traumatic joint disorders
204	M13872	Other non-traumatic joint disorders
204	M13879	Other non-traumatic joint disorders
204	M1388	Other non-traumatic joint disorders
204	M1389	Other non-traumatic joint disorders
204	M1460	Other non-traumatic joint disorders
204	M14611	Other non-traumatic joint disorders
204	M14612	Other non-traumatic joint disorders
204	M14619	Other non-traumatic joint disorders
204	M14621	Other non-traumatic joint disorders
204	M14622	Other non-traumatic joint disorders
204	M14629	Other non-traumatic joint disorders
204	M14631	Other non-traumatic joint disorders
204	M14632	Other non-traumatic joint disorders
204	M14639	Other non-traumatic joint disorders
204	M14641	Other non-traumatic joint disorders
204	M14642	Other non-traumatic joint disorders
204	M14649	Other non-traumatic joint disorders
204	M14651	Other non-traumatic joint disorders
204	M14652	Other non-traumatic joint disorders
204	M14659	Other non-traumatic joint disorders
204	M14661	Other non-traumatic joint disorders
204	M14662	Other non-traumatic joint disorders
204	M14669	Other non-traumatic joint disorders
204	M14671	Other non-traumatic joint disorders

204	M14672	Other non-traumatic joint disorders
204	M14679	Other non-traumatic joint disorders
204	M1468	Other non-traumatic joint disorders
204	M1469	Other non-traumatic joint disorders
204	M1480	Other non-traumatic joint disorders
204	M14811	Other non-traumatic joint disorders
204	M14812	Other non-traumatic joint disorders
204	M14819	Other non-traumatic joint disorders
204	M14821	Other non-traumatic joint disorders
204	M14822	Other non-traumatic joint disorders
204	M14829	Other non-traumatic joint disorders
204	M14831	Other non-traumatic joint disorders
204	M14832	Other non-traumatic joint disorders
204	M14839	Other non-traumatic joint disorders
204	M14841	Other non-traumatic joint disorders
204	M14842	Other non-traumatic joint disorders
204	M14849	Other non-traumatic joint disorders
204	M14851	Other non-traumatic joint disorders
204	M14852	Other non-traumatic joint disorders
204	M14859	Other non-traumatic joint disorders
204	M14861	Other non-traumatic joint disorders
204	M14862	Other non-traumatic joint disorders
204	M14869	Other non-traumatic joint disorders
204	M14871	Other non-traumatic joint disorders
204	M14872	Other non-traumatic joint disorders
204	M14879	Other non-traumatic joint disorders
204	M1488	Other non-traumatic joint disorders
204	M1489	Other non-traumatic joint disorders
204	M2400	Other non-traumatic joint disorders
204	M24011	Other non-traumatic joint disorders
204	M24012	Other non-traumatic joint disorders
204	M24019	Other non-traumatic joint disorders
204	M24021	Other non-traumatic joint disorders
204	M24022	Other non-traumatic joint disorders
204	M24029	Other non-traumatic joint disorders
204	M24031	Other non-traumatic joint disorders
204	M24032	Other non-traumatic joint disorders
204	M24039	Other non-traumatic joint disorders
204	M24041	Other non-traumatic joint disorders
204	M24042	Other non-traumatic joint disorders
204	M24049	Other non-traumatic joint disorders
204	M24051	Other non-traumatic joint disorders
204	M24052	Other non-traumatic joint disorders
204	M24059	Other non-traumatic joint disorders
204	M24071	Other non-traumatic joint disorders
204	M24072	Other non-traumatic joint disorders
204	M24073	Other non-traumatic joint disorders
204	M24074	Other non-traumatic joint disorders
204	M24075	Other non-traumatic joint disorders
204	M24076	Other non-traumatic joint disorders
204	M2408	Other non-traumatic joint disorders

204	M2430	Other non-traumatic joint disorders
204	M24311	Other non-traumatic joint disorders
204	M24312	Other non-traumatic joint disorders
204	M24319	Other non-traumatic joint disorders
204	M24321	Other non-traumatic joint disorders
204	M24322	Other non-traumatic joint disorders
204	M24329	Other non-traumatic joint disorders
204	M24331	Other non-traumatic joint disorders
204	M24332	Other non-traumatic joint disorders
204	M24339	Other non-traumatic joint disorders
204	M24341	Other non-traumatic joint disorders
204	M24342	Other non-traumatic joint disorders
204	M24349	Other non-traumatic joint disorders
204	M24351	Other non-traumatic joint disorders
204	M24352	Other non-traumatic joint disorders
204	M24359	Other non-traumatic joint disorders
204	M24361	Other non-traumatic joint disorders
204	M24362	Other non-traumatic joint disorders
204	M24369	Other non-traumatic joint disorders
204	M24371	Other non-traumatic joint disorders
204	M24372	Other non-traumatic joint disorders
204	M24373	Other non-traumatic joint disorders
204	M24374	Other non-traumatic joint disorders
204	M24375	Other non-traumatic joint disorders
204	M24376	Other non-traumatic joint disorders
204	M2460	Other non-traumatic joint disorders
204	M24611	Other non-traumatic joint disorders
204	M24612	Other non-traumatic joint disorders
204	M24619	Other non-traumatic joint disorders
204	M24621	Other non-traumatic joint disorders
204	M24622	Other non-traumatic joint disorders
204	M24629	Other non-traumatic joint disorders
204	M24631	Other non-traumatic joint disorders
204	M24632	Other non-traumatic joint disorders
204	M24639	Other non-traumatic joint disorders
204	M24641	Other non-traumatic joint disorders
204	M24642	Other non-traumatic joint disorders
204	M24649	Other non-traumatic joint disorders
204	M24651	Other non-traumatic joint disorders
204	M24652	Other non-traumatic joint disorders
204	M24659	Other non-traumatic joint disorders
204	M24661	Other non-traumatic joint disorders
204	M24662	Other non-traumatic joint disorders
204	M24669	Other non-traumatic joint disorders
204	M24671	Other non-traumatic joint disorders
204	M24672	Other non-traumatic joint disorders
204	M24673	Other non-traumatic joint disorders
204	M24674	Other non-traumatic joint disorders
204	M24675	Other non-traumatic joint disorders
204	M24676	Other non-traumatic joint disorders
204	M247	Other non-traumatic joint disorders

204	M2480	Other non-traumatic joint disorders
204	M24811	Other non-traumatic joint disorders
204	M24812	Other non-traumatic joint disorders
204	M24819	Other non-traumatic joint disorders
204	M24821	Other non-traumatic joint disorders
204	M24822	Other non-traumatic joint disorders
204	M24829	Other non-traumatic joint disorders
204	M24831	Other non-traumatic joint disorders
204	M24832	Other non-traumatic joint disorders
204	M24839	Other non-traumatic joint disorders
204	M24841	Other non-traumatic joint disorders
204	M24842	Other non-traumatic joint disorders
204	M24849	Other non-traumatic joint disorders
204	M24851	Other non-traumatic joint disorders
204	M24852	Other non-traumatic joint disorders
204	M24859	Other non-traumatic joint disorders
204	M24871	Other non-traumatic joint disorders
204	M24872	Other non-traumatic joint disorders
204	M24873	Other non-traumatic joint disorders
204	M24874	Other non-traumatic joint disorders
204	M24875	Other non-traumatic joint disorders
204	M24876	Other non-traumatic joint disorders
204	M249	Other non-traumatic joint disorders
204	M2500	Other non-traumatic joint disorders
204	M25011	Other non-traumatic joint disorders
204	M25012	Other non-traumatic joint disorders
204	M25019	Other non-traumatic joint disorders
204	M25021	Other non-traumatic joint disorders
204	M25022	Other non-traumatic joint disorders
204	M25029	Other non-traumatic joint disorders
204	M25031	Other non-traumatic joint disorders
204	M25032	Other non-traumatic joint disorders
204	M25039	Other non-traumatic joint disorders
204	M25041	Other non-traumatic joint disorders
204	M25042	Other non-traumatic joint disorders
204	M25049	Other non-traumatic joint disorders
204	M25051	Other non-traumatic joint disorders
204	M25052	Other non-traumatic joint disorders
204	M25059	Other non-traumatic joint disorders
204	M25061	Other non-traumatic joint disorders
204	M25062	Other non-traumatic joint disorders
204	M25069	Other non-traumatic joint disorders
204	M25071	Other non-traumatic joint disorders
204	M25072	Other non-traumatic joint disorders
204	M25073	Other non-traumatic joint disorders
204	M25074	Other non-traumatic joint disorders
204	M25075	Other non-traumatic joint disorders
204	M25076	Other non-traumatic joint disorders
204	M2508	Other non-traumatic joint disorders
204	M2510	Other non-traumatic joint disorders
204	M25111	Other non-traumatic joint disorders

204	M25112	Other non-traumatic joint disorders
204	M25119	Other non-traumatic joint disorders
204	M25121	Other non-traumatic joint disorders
204	M25122	Other non-traumatic joint disorders
204	M25129	Other non-traumatic joint disorders
204	M25131	Other non-traumatic joint disorders
204	M25132	Other non-traumatic joint disorders
204	M25139	Other non-traumatic joint disorders
204	M25141	Other non-traumatic joint disorders
204	M25142	Other non-traumatic joint disorders
204	M25149	Other non-traumatic joint disorders
204	M25151	Other non-traumatic joint disorders
204	M25152	Other non-traumatic joint disorders
204	M25159	Other non-traumatic joint disorders
204	M25161	Other non-traumatic joint disorders
204	M25162	Other non-traumatic joint disorders
204	M25169	Other non-traumatic joint disorders
204	M25171	Other non-traumatic joint disorders
204	M25172	Other non-traumatic joint disorders
204	M25173	Other non-traumatic joint disorders
204	M25174	Other non-traumatic joint disorders
204	M25175	Other non-traumatic joint disorders
204	M25176	Other non-traumatic joint disorders
204	M2518	Other non-traumatic joint disorders
204	M2520	Other non-traumatic joint disorders
204	M25211	Other non-traumatic joint disorders
204	M25212	Other non-traumatic joint disorders
204	M25219	Other non-traumatic joint disorders
204	M25221	Other non-traumatic joint disorders
204	M25222	Other non-traumatic joint disorders
204	M25229	Other non-traumatic joint disorders
204	M25231	Other non-traumatic joint disorders
204	M25232	Other non-traumatic joint disorders
204	M25239	Other non-traumatic joint disorders
204	M25241	Other non-traumatic joint disorders
204	M25242	Other non-traumatic joint disorders
204	M25249	Other non-traumatic joint disorders
204	M25251	Other non-traumatic joint disorders
204	M25252	Other non-traumatic joint disorders
204	M25259	Other non-traumatic joint disorders
204	M25261	Other non-traumatic joint disorders
204	M25262	Other non-traumatic joint disorders
204	M25269	Other non-traumatic joint disorders
204	M25271	Other non-traumatic joint disorders
204	M25272	Other non-traumatic joint disorders
204	M25279	Other non-traumatic joint disorders
204	M2528	Other non-traumatic joint disorders
204	M2530	Other non-traumatic joint disorders
204	M25311	Other non-traumatic joint disorders
204	M25312	Other non-traumatic joint disorders
204	M25319	Other non-traumatic joint disorders

204	M25321	Other non-traumatic joint disorders
204	M25322	Other non-traumatic joint disorders
204	M25329	Other non-traumatic joint disorders
204	M25331	Other non-traumatic joint disorders
204	M25332	Other non-traumatic joint disorders
204	M25339	Other non-traumatic joint disorders
204	M25341	Other non-traumatic joint disorders
204	M25342	Other non-traumatic joint disorders
204	M25349	Other non-traumatic joint disorders
204	M25351	Other non-traumatic joint disorders
204	M25352	Other non-traumatic joint disorders
204	M25359	Other non-traumatic joint disorders
204	M25361	Other non-traumatic joint disorders
204	M25362	Other non-traumatic joint disorders
204	M25369	Other non-traumatic joint disorders
204	M25371	Other non-traumatic joint disorders
204	M25372	Other non-traumatic joint disorders
204	M25373	Other non-traumatic joint disorders
204	M25374	Other non-traumatic joint disorders
204	M25375	Other non-traumatic joint disorders
204	M25376	Other non-traumatic joint disorders
204	M2540	Other non-traumatic joint disorders
204	M25411	Other non-traumatic joint disorders
204	M25412	Other non-traumatic joint disorders
204	M25419	Other non-traumatic joint disorders
204	M25421	Other non-traumatic joint disorders
204	M25422	Other non-traumatic joint disorders
204	M25429	Other non-traumatic joint disorders
204	M25431	Other non-traumatic joint disorders
204	M25432	Other non-traumatic joint disorders
204	M25439	Other non-traumatic joint disorders
204	M25441	Other non-traumatic joint disorders
204	M25442	Other non-traumatic joint disorders
204	M25449	Other non-traumatic joint disorders
204	M25451	Other non-traumatic joint disorders
204	M25452	Other non-traumatic joint disorders
204	M25459	Other non-traumatic joint disorders
204	M25461	Other non-traumatic joint disorders
204	M25462	Other non-traumatic joint disorders
204	M25469	Other non-traumatic joint disorders
204	M25471	Other non-traumatic joint disorders
204	M25472	Other non-traumatic joint disorders
204	M25473	Other non-traumatic joint disorders
204	M25474	Other non-traumatic joint disorders
204	M25475	Other non-traumatic joint disorders
204	M25476	Other non-traumatic joint disorders
204	M2548	Other non-traumatic joint disorders
204	M2550	Other non-traumatic joint disorders
204	M25511	Other non-traumatic joint disorders
204	M25512	Other non-traumatic joint disorders
204	M25519	Other non-traumatic joint disorders

204	M25521	Other non-traumatic joint disorders
204	M25522	Other non-traumatic joint disorders
204	M25529	Other non-traumatic joint disorders
204	M25531	Other non-traumatic joint disorders
204	M25532	Other non-traumatic joint disorders
204	M25539	Other non-traumatic joint disorders
204	M25551	Other non-traumatic joint disorders
204	M25552	Other non-traumatic joint disorders
204	M25559	Other non-traumatic joint disorders
204	M25561	Other non-traumatic joint disorders
204	M25562	Other non-traumatic joint disorders
204	M25569	Other non-traumatic joint disorders
204	M25571	Other non-traumatic joint disorders
204	M25572	Other non-traumatic joint disorders
204	M25579	Other non-traumatic joint disorders
204	M2560	Other non-traumatic joint disorders
204	M25611	Other non-traumatic joint disorders
204	M25612	Other non-traumatic joint disorders
204	M25619	Other non-traumatic joint disorders
204	M25621	Other non-traumatic joint disorders
204	M25622	Other non-traumatic joint disorders
204	M25629	Other non-traumatic joint disorders
204	M25631	Other non-traumatic joint disorders
204	M25632	Other non-traumatic joint disorders
204	M25639	Other non-traumatic joint disorders
204	M25641	Other non-traumatic joint disorders
204	M25642	Other non-traumatic joint disorders
204	M25649	Other non-traumatic joint disorders
204	M25651	Other non-traumatic joint disorders
204	M25652	Other non-traumatic joint disorders
204	M25659	Other non-traumatic joint disorders
204	M25661	Other non-traumatic joint disorders
204	M25662	Other non-traumatic joint disorders
204	M25669	Other non-traumatic joint disorders
204	M25671	Other non-traumatic joint disorders
204	M25672	Other non-traumatic joint disorders
204	M25673	Other non-traumatic joint disorders
204	M25674	Other non-traumatic joint disorders
204	M25675	Other non-traumatic joint disorders
204	M25676	Other non-traumatic joint disorders
204	M2570	Other non-traumatic joint disorders
204	M25711	Other non-traumatic joint disorders
204	M25712	Other non-traumatic joint disorders
204	M25719	Other non-traumatic joint disorders
204	M25721	Other non-traumatic joint disorders
204	M25722	Other non-traumatic joint disorders
204	M25729	Other non-traumatic joint disorders
204	M25731	Other non-traumatic joint disorders
204	M25732	Other non-traumatic joint disorders
204	M25739	Other non-traumatic joint disorders
204	M25741	Other non-traumatic joint disorders

204	M25742	Other non-traumatic joint disorders
204	M25749	Other non-traumatic joint disorders
204	M25751	Other non-traumatic joint disorders
204	M25752	Other non-traumatic joint disorders
204	M25759	Other non-traumatic joint disorders
204	M25761	Other non-traumatic joint disorders
204	M25762	Other non-traumatic joint disorders
204	M25769	Other non-traumatic joint disorders
204	M25771	Other non-traumatic joint disorders
204	M25772	Other non-traumatic joint disorders
204	M25773	Other non-traumatic joint disorders
204	M25774	Other non-traumatic joint disorders
204	M25775	Other non-traumatic joint disorders
204	M25776	Other non-traumatic joint disorders
204	M2578	Other non-traumatic joint disorders
204	M2580	Other non-traumatic joint disorders
204	M25811	Other non-traumatic joint disorders
204	M25812	Other non-traumatic joint disorders
204	M25819	Other non-traumatic joint disorders
204	M25821	Other non-traumatic joint disorders
204	M25822	Other non-traumatic joint disorders
204	M25829	Other non-traumatic joint disorders
204	M25831	Other non-traumatic joint disorders
204	M25832	Other non-traumatic joint disorders
204	M25839	Other non-traumatic joint disorders
204	M25841	Other non-traumatic joint disorders
204	M25842	Other non-traumatic joint disorders
204	M25849	Other non-traumatic joint disorders
204	M25851	Other non-traumatic joint disorders
204	M25852	Other non-traumatic joint disorders
204	M25859	Other non-traumatic joint disorders
204	M25861	Other non-traumatic joint disorders
204	M25862	Other non-traumatic joint disorders
204	M25869	Other non-traumatic joint disorders
204	M25871	Other non-traumatic joint disorders
204	M25872	Other non-traumatic joint disorders
204	M25879	Other non-traumatic joint disorders
204	M259	Other non-traumatic joint disorders
204	M361	Other non-traumatic joint disorders
204	M362	Other non-traumatic joint disorders
204	M363	Other non-traumatic joint disorders
204	M364	Other non-traumatic joint disorders
204	R294	Other non-traumatic joint disorders
205	M4320	Spondylosis; intervertebral disc disorders; other back problems
205	M4321	Spondylosis; intervertebral disc disorders; other back problems
205	M4322	Spondylosis; intervertebral disc disorders; other back problems
205	M4323	Spondylosis; intervertebral disc disorders; other back problems
205	M4324	Spondylosis; intervertebral disc disorders; other back problems
205	M4325	Spondylosis; intervertebral disc disorders; other back problems
205	M4326	Spondylosis; intervertebral disc disorders; other back problems
205	M4327	Spondylosis; intervertebral disc disorders; other back problems

205	M4328	Spondylosis; intervertebral disc disorders; other back problems
205	M436	Spondylosis; intervertebral disc disorders; other back problems
205	M4600	Spondylosis; intervertebral disc disorders; other back problems
205	M4601	Spondylosis; intervertebral disc disorders; other back problems
205	M4602	Spondylosis; intervertebral disc disorders; other back problems
205	M4603	Spondylosis; intervertebral disc disorders; other back problems
205	M4604	Spondylosis; intervertebral disc disorders; other back problems
205	M4605	Spondylosis; intervertebral disc disorders; other back problems
205	M4606	Spondylosis; intervertebral disc disorders; other back problems
205	M4607	Spondylosis; intervertebral disc disorders; other back problems
205	M4608	Spondylosis; intervertebral disc disorders; other back problems
205	M4609	Spondylosis; intervertebral disc disorders; other back problems
205	M461	Spondylosis; intervertebral disc disorders; other back problems
205	M4630	Spondylosis; intervertebral disc disorders; other back problems
205	M4631	Spondylosis; intervertebral disc disorders; other back problems
205	M4632	Spondylosis; intervertebral disc disorders; other back problems
205	M4633	Spondylosis; intervertebral disc disorders; other back problems
205	M4634	Spondylosis; intervertebral disc disorders; other back problems
205	M4635	Spondylosis; intervertebral disc disorders; other back problems
205	M4636	Spondylosis; intervertebral disc disorders; other back problems
205	M4637	Spondylosis; intervertebral disc disorders; other back problems
205	M4638	Spondylosis; intervertebral disc disorders; other back problems
205	M4639	Spondylosis; intervertebral disc disorders; other back problems
205	M4640	Spondylosis; intervertebral disc disorders; other back problems
205	M4641	Spondylosis; intervertebral disc disorders; other back problems
205	M4642	Spondylosis; intervertebral disc disorders; other back problems
205	M4643	Spondylosis; intervertebral disc disorders; other back problems
205	M4644	Spondylosis; intervertebral disc disorders; other back problems
205	M4645	Spondylosis; intervertebral disc disorders; other back problems
205	M4646	Spondylosis; intervertebral disc disorders; other back problems
205	M4647	Spondylosis; intervertebral disc disorders; other back problems
205	M4648	Spondylosis; intervertebral disc disorders; other back problems
205	M4649	Spondylosis; intervertebral disc disorders; other back problems
205	M4650	Spondylosis; intervertebral disc disorders; other back problems
205	M4651	Spondylosis; intervertebral disc disorders; other back problems
205	M4652	Spondylosis; intervertebral disc disorders; other back problems
205	M4653	Spondylosis; intervertebral disc disorders; other back problems
205	M4654	Spondylosis; intervertebral disc disorders; other back problems
205	M4655	Spondylosis; intervertebral disc disorders; other back problems
205	M4656	Spondylosis; intervertebral disc disorders; other back problems
205	M4657	Spondylosis; intervertebral disc disorders; other back problems
205	M4658	Spondylosis; intervertebral disc disorders; other back problems
205	M4659	Spondylosis; intervertebral disc disorders; other back problems
205	M4680	Spondylosis; intervertebral disc disorders; other back problems
205	M4681	Spondylosis; intervertebral disc disorders; other back problems
205	M4682	Spondylosis; intervertebral disc disorders; other back problems
205	M4683	Spondylosis; intervertebral disc disorders; other back problems
205	M4684	Spondylosis; intervertebral disc disorders; other back problems
205	M4685	Spondylosis; intervertebral disc disorders; other back problems
205	M4686	Spondylosis; intervertebral disc disorders; other back problems
205	M4687	Spondylosis; intervertebral disc disorders; other back problems

205	M4688	Spondylosis; intervertebral disc disorders; other back problems
205	M4689	Spondylosis; intervertebral disc disorders; other back problems
205	M4690	Spondylosis; intervertebral disc disorders; other back problems
205	M4691	Spondylosis; intervertebral disc disorders; other back problems
205	M4692	Spondylosis; intervertebral disc disorders; other back problems
205	M4693	Spondylosis; intervertebral disc disorders; other back problems
205	M4694	Spondylosis; intervertebral disc disorders; other back problems
205	M4695	Spondylosis; intervertebral disc disorders; other back problems
205	M4696	Spondylosis; intervertebral disc disorders; other back problems
205	M4697	Spondylosis; intervertebral disc disorders; other back problems
205	M4698	Spondylosis; intervertebral disc disorders; other back problems
205	M4699	Spondylosis; intervertebral disc disorders; other back problems
205	M47011	Spondylosis; intervertebral disc disorders; other back problems
205	M47012	Spondylosis; intervertebral disc disorders; other back problems
205	M47013	Spondylosis; intervertebral disc disorders; other back problems
205	M47014	Spondylosis; intervertebral disc disorders; other back problems
205	M47015	Spondylosis; intervertebral disc disorders; other back problems
205	M47016	Spondylosis; intervertebral disc disorders; other back problems
205	M47019	Spondylosis; intervertebral disc disorders; other back problems
205	M47021	Spondylosis; intervertebral disc disorders; other back problems
205	M47022	Spondylosis; intervertebral disc disorders; other back problems
205	M47029	Spondylosis; intervertebral disc disorders; other back problems
205	M4710	Spondylosis; intervertebral disc disorders; other back problems
205	M4711	Spondylosis; intervertebral disc disorders; other back problems
205	M4712	Spondylosis; intervertebral disc disorders; other back problems
205	M4713	Spondylosis; intervertebral disc disorders; other back problems
205	M4714	Spondylosis; intervertebral disc disorders; other back problems
205	M4715	Spondylosis; intervertebral disc disorders; other back problems
205	M4716	Spondylosis; intervertebral disc disorders; other back problems
205	M4717	Spondylosis; intervertebral disc disorders; other back problems
205	M4718	Spondylosis; intervertebral disc disorders; other back problems
205	M4720	Spondylosis; intervertebral disc disorders; other back problems
205	M4721	Spondylosis; intervertebral disc disorders; other back problems
205	M4722	Spondylosis; intervertebral disc disorders; other back problems
205	M4723	Spondylosis; intervertebral disc disorders; other back problems
205	M4724	Spondylosis; intervertebral disc disorders; other back problems
205	M4725	Spondylosis; intervertebral disc disorders; other back problems
205	M4726	Spondylosis; intervertebral disc disorders; other back problems
205	M4727	Spondylosis; intervertebral disc disorders; other back problems
205	M4728	Spondylosis; intervertebral disc disorders; other back problems
205	M47811	Spondylosis; intervertebral disc disorders; other back problems
205	M47812	Spondylosis; intervertebral disc disorders; other back problems
205	M47813	Spondylosis; intervertebral disc disorders; other back problems
205	M47814	Spondylosis; intervertebral disc disorders; other back problems
205	M47815	Spondylosis; intervertebral disc disorders; other back problems
205	M47816	Spondylosis; intervertebral disc disorders; other back problems
205	M47817	Spondylosis; intervertebral disc disorders; other back problems
205	M47818	Spondylosis; intervertebral disc disorders; other back problems
205	M47819	Spondylosis; intervertebral disc disorders; other back problems
205	M47891	Spondylosis; intervertebral disc disorders; other back problems
205	M47892	Spondylosis; intervertebral disc disorders; other back problems

205	M47893	Spondylosis; intervertebral disc disorders; other back problems
205	M47894	Spondylosis; intervertebral disc disorders; other back problems
205	M47895	Spondylosis; intervertebral disc disorders; other back problems
205	M47896	Spondylosis; intervertebral disc disorders; other back problems
205	M47897	Spondylosis; intervertebral disc disorders; other back problems
205	M47898	Spondylosis; intervertebral disc disorders; other back problems
205	M47899	Spondylosis; intervertebral disc disorders; other back problems
205	M479	Spondylosis; intervertebral disc disorders; other back problems
205	M4800	Spondylosis; intervertebral disc disorders; other back problems
205	M4801	Spondylosis; intervertebral disc disorders; other back problems
205	M4802	Spondylosis; intervertebral disc disorders; other back problems
205	M4803	Spondylosis; intervertebral disc disorders; other back problems
205	M4804	Spondylosis; intervertebral disc disorders; other back problems
205	M4805	Spondylosis; intervertebral disc disorders; other back problems
205	M4806	Spondylosis; intervertebral disc disorders; other back problems
205	M4807	Spondylosis; intervertebral disc disorders; other back problems
205	M4808	Spondylosis; intervertebral disc disorders; other back problems
205	M4810	Spondylosis; intervertebral disc disorders; other back problems
205	M4811	Spondylosis; intervertebral disc disorders; other back problems
205	M4812	Spondylosis; intervertebral disc disorders; other back problems
205	M4813	Spondylosis; intervertebral disc disorders; other back problems
205	M4814	Spondylosis; intervertebral disc disorders; other back problems
205	M4815	Spondylosis; intervertebral disc disorders; other back problems
205	M4816	Spondylosis; intervertebral disc disorders; other back problems
205	M4817	Spondylosis; intervertebral disc disorders; other back problems
205	M4818	Spondylosis; intervertebral disc disorders; other back problems
205	M4819	Spondylosis; intervertebral disc disorders; other back problems
205	M4820	Spondylosis; intervertebral disc disorders; other back problems
205	M4821	Spondylosis; intervertebral disc disorders; other back problems
205	M4822	Spondylosis; intervertebral disc disorders; other back problems
205	M4823	Spondylosis; intervertebral disc disorders; other back problems
205	M4824	Spondylosis; intervertebral disc disorders; other back problems
205	M4825	Spondylosis; intervertebral disc disorders; other back problems
205	M4826	Spondylosis; intervertebral disc disorders; other back problems
205	M4827	Spondylosis; intervertebral disc disorders; other back problems
205	M4830	Spondylosis; intervertebral disc disorders; other back problems
205	M4831	Spondylosis; intervertebral disc disorders; other back problems
205	M4832	Spondylosis; intervertebral disc disorders; other back problems
205	M4833	Spondylosis; intervertebral disc disorders; other back problems
205	M4834	Spondylosis; intervertebral disc disorders; other back problems
205	M4835	Spondylosis; intervertebral disc disorders; other back problems
205	M4836	Spondylosis; intervertebral disc disorders; other back problems
205	M4837	Spondylosis; intervertebral disc disorders; other back problems
205	M4838	Spondylosis; intervertebral disc disorders; other back problems
205	M489	Spondylosis; intervertebral disc disorders; other back problems
205	M4980	Spondylosis; intervertebral disc disorders; other back problems
205	M4981	Spondylosis; intervertebral disc disorders; other back problems
205	M4982	Spondylosis; intervertebral disc disorders; other back problems
205	M4983	Spondylosis; intervertebral disc disorders; other back problems
205	M4984	Spondylosis; intervertebral disc disorders; other back problems
205	M4985	Spondylosis; intervertebral disc disorders; other back problems

205	M4986	Spondylosis; intervertebral disc disorders; other back problems
205	M4987	Spondylosis; intervertebral disc disorders; other back problems
205	M4988	Spondylosis; intervertebral disc disorders; other back problems
205	M4989	Spondylosis; intervertebral disc disorders; other back problems
205	M5000	Spondylosis; intervertebral disc disorders; other back problems
205	M5001	Spondylosis; intervertebral disc disorders; other back problems
205	M5002	Spondylosis; intervertebral disc disorders; other back problems
205	M5003	Spondylosis; intervertebral disc disorders; other back problems
205	M5010	Spondylosis; intervertebral disc disorders; other back problems
205	M5011	Spondylosis; intervertebral disc disorders; other back problems
205	M5012	Spondylosis; intervertebral disc disorders; other back problems
205	M5013	Spondylosis; intervertebral disc disorders; other back problems
205	M5020	Spondylosis; intervertebral disc disorders; other back problems
205	M5021	Spondylosis; intervertebral disc disorders; other back problems
205	M5022	Spondylosis; intervertebral disc disorders; other back problems
205	M5023	Spondylosis; intervertebral disc disorders; other back problems
205	M5030	Spondylosis; intervertebral disc disorders; other back problems
205	M5031	Spondylosis; intervertebral disc disorders; other back problems
205	M5032	Spondylosis; intervertebral disc disorders; other back problems
205	M5033	Spondylosis; intervertebral disc disorders; other back problems
205	M5080	Spondylosis; intervertebral disc disorders; other back problems
205	M5081	Spondylosis; intervertebral disc disorders; other back problems
205	M5082	Spondylosis; intervertebral disc disorders; other back problems
205	M5083	Spondylosis; intervertebral disc disorders; other back problems
205	M5090	Spondylosis; intervertebral disc disorders; other back problems
205	M5091	Spondylosis; intervertebral disc disorders; other back problems
205	M5092	Spondylosis; intervertebral disc disorders; other back problems
205	M5093	Spondylosis; intervertebral disc disorders; other back problems
205	M5104	Spondylosis; intervertebral disc disorders; other back problems
205	M5105	Spondylosis; intervertebral disc disorders; other back problems
205	M5106	Spondylosis; intervertebral disc disorders; other back problems
205	M5107	Spondylosis; intervertebral disc disorders; other back problems
205	M5114	Spondylosis; intervertebral disc disorders; other back problems
205	M5115	Spondylosis; intervertebral disc disorders; other back problems
205	M5116	Spondylosis; intervertebral disc disorders; other back problems
205	M5117	Spondylosis; intervertebral disc disorders; other back problems
205	M5124	Spondylosis; intervertebral disc disorders; other back problems
205	M5125	Spondylosis; intervertebral disc disorders; other back problems
205	M5126	Spondylosis; intervertebral disc disorders; other back problems
205	M5127	Spondylosis; intervertebral disc disorders; other back problems
205	M5134	Spondylosis; intervertebral disc disorders; other back problems
205	M5135	Spondylosis; intervertebral disc disorders; other back problems
205	M5136	Spondylosis; intervertebral disc disorders; other back problems
205	M5137	Spondylosis; intervertebral disc disorders; other back problems
205	M5144	Spondylosis; intervertebral disc disorders; other back problems
205	M5145	Spondylosis; intervertebral disc disorders; other back problems
205	M5146	Spondylosis; intervertebral disc disorders; other back problems
205	M5147	Spondylosis; intervertebral disc disorders; other back problems
205	M5184	Spondylosis; intervertebral disc disorders; other back problems
205	M5185	Spondylosis; intervertebral disc disorders; other back problems
205	M5186	Spondylosis; intervertebral disc disorders; other back problems

205	M5187	Spondylosis; intervertebral disc disorders; other back problems
205	M519	Spondylosis; intervertebral disc disorders; other back problems
205	M530	Spondylosis; intervertebral disc disorders; other back problems
205	M531	Spondylosis; intervertebral disc disorders; other back problems
205	M532X1	Spondylosis; intervertebral disc disorders; other back problems
205	M532X2	Spondylosis; intervertebral disc disorders; other back problems
205	M532X3	Spondylosis; intervertebral disc disorders; other back problems
205	M532X4	Spondylosis; intervertebral disc disorders; other back problems
205	M532X5	Spondylosis; intervertebral disc disorders; other back problems
205	M532X6	Spondylosis; intervertebral disc disorders; other back problems
205	M532X7	Spondylosis; intervertebral disc disorders; other back problems
205	M532X8	Spondylosis; intervertebral disc disorders; other back problems
205	M532X9	Spondylosis; intervertebral disc disorders; other back problems
205	M533	Spondylosis; intervertebral disc disorders; other back problems
205	M5380	Spondylosis; intervertebral disc disorders; other back problems
205	M5381	Spondylosis; intervertebral disc disorders; other back problems
205	M5382	Spondylosis; intervertebral disc disorders; other back problems
205	M5383	Spondylosis; intervertebral disc disorders; other back problems
205	M5384	Spondylosis; intervertebral disc disorders; other back problems
205	M5385	Spondylosis; intervertebral disc disorders; other back problems
205	M5386	Spondylosis; intervertebral disc disorders; other back problems
205	M5387	Spondylosis; intervertebral disc disorders; other back problems
205	M5388	Spondylosis; intervertebral disc disorders; other back problems
205	M539	Spondylosis; intervertebral disc disorders; other back problems
205	M5400	Spondylosis; intervertebral disc disorders; other back problems
205	M5401	Spondylosis; intervertebral disc disorders; other back problems
205	M5402	Spondylosis; intervertebral disc disorders; other back problems
205	M5403	Spondylosis; intervertebral disc disorders; other back problems
205	M5404	Spondylosis; intervertebral disc disorders; other back problems
205	M5405	Spondylosis; intervertebral disc disorders; other back problems
205	M5406	Spondylosis; intervertebral disc disorders; other back problems
205	M5407	Spondylosis; intervertebral disc disorders; other back problems
205	M5408	Spondylosis; intervertebral disc disorders; other back problems
205	M5409	Spondylosis; intervertebral disc disorders; other back problems
205	M5410	Spondylosis; intervertebral disc disorders; other back problems
205	M5411	Spondylosis; intervertebral disc disorders; other back problems
205	M5412	Spondylosis; intervertebral disc disorders; other back problems
205	M5413	Spondylosis; intervertebral disc disorders; other back problems
205	M5414	Spondylosis; intervertebral disc disorders; other back problems
205	M5415	Spondylosis; intervertebral disc disorders; other back problems
205	M5416	Spondylosis; intervertebral disc disorders; other back problems
205	M5417	Spondylosis; intervertebral disc disorders; other back problems
205	M5418	Spondylosis; intervertebral disc disorders; other back problems
205	M542	Spondylosis; intervertebral disc disorders; other back problems
205	M5430	Spondylosis; intervertebral disc disorders; other back problems
205	M5431	Spondylosis; intervertebral disc disorders; other back problems
205	M5432	Spondylosis; intervertebral disc disorders; other back problems
205	M5440	Spondylosis; intervertebral disc disorders; other back problems
205	M5441	Spondylosis; intervertebral disc disorders; other back problems
205	M5442	Spondylosis; intervertebral disc disorders; other back problems
205	M545	Spondylosis; intervertebral disc disorders; other back problems

205	M546	Spondylosis; intervertebral disc disorders; other back problems
205	M5481	Spondylosis; intervertebral disc disorders; other back problems
205	M5489	Spondylosis; intervertebral disc disorders; other back problems
205	M549	Spondylosis; intervertebral disc disorders; other back problems
205	M62830	Spondylosis; intervertebral disc disorders; other back problems
205	M961	Spondylosis; intervertebral disc disorders; other back problems
205	M9920	Spondylosis; intervertebral disc disorders; other back problems
205	M9921	Spondylosis; intervertebral disc disorders; other back problems
205	M9922	Spondylosis; intervertebral disc disorders; other back problems
205	M9923	Spondylosis; intervertebral disc disorders; other back problems
205	M9924	Spondylosis; intervertebral disc disorders; other back problems
205	M9925	Spondylosis; intervertebral disc disorders; other back problems
205	M9926	Spondylosis; intervertebral disc disorders; other back problems
205	M9927	Spondylosis; intervertebral disc disorders; other back problems
205	M9928	Spondylosis; intervertebral disc disorders; other back problems
205	M9929	Spondylosis; intervertebral disc disorders; other back problems
205	M9930	Spondylosis; intervertebral disc disorders; other back problems
205	M9931	Spondylosis; intervertebral disc disorders; other back problems
205	M9932	Spondylosis; intervertebral disc disorders; other back problems
205	M9933	Spondylosis; intervertebral disc disorders; other back problems
205	M9934	Spondylosis; intervertebral disc disorders; other back problems
205	M9935	Spondylosis; intervertebral disc disorders; other back problems
205	M9936	Spondylosis; intervertebral disc disorders; other back problems
205	M9937	Spondylosis; intervertebral disc disorders; other back problems
205	M9938	Spondylosis; intervertebral disc disorders; other back problems
205	M9939	Spondylosis; intervertebral disc disorders; other back problems
205	M9940	Spondylosis; intervertebral disc disorders; other back problems
205	M9941	Spondylosis; intervertebral disc disorders; other back problems
205	M9942	Spondylosis; intervertebral disc disorders; other back problems
205	M9943	Spondylosis; intervertebral disc disorders; other back problems
205	M9944	Spondylosis; intervertebral disc disorders; other back problems
205	M9945	Spondylosis; intervertebral disc disorders; other back problems
205	M9946	Spondylosis; intervertebral disc disorders; other back problems
205	M9947	Spondylosis; intervertebral disc disorders; other back problems
205	M9948	Spondylosis; intervertebral disc disorders; other back problems
205	M9949	Spondylosis; intervertebral disc disorders; other back problems
205	M9950	Spondylosis; intervertebral disc disorders; other back problems
205	M9951	Spondylosis; intervertebral disc disorders; other back problems
205	M9952	Spondylosis; intervertebral disc disorders; other back problems
205	M9953	Spondylosis; intervertebral disc disorders; other back problems
205	M9954	Spondylosis; intervertebral disc disorders; other back problems
205	M9955	Spondylosis; intervertebral disc disorders; other back problems
205	M9956	Spondylosis; intervertebral disc disorders; other back problems
205	M9957	Spondylosis; intervertebral disc disorders; other back problems
205	M9958	Spondylosis; intervertebral disc disorders; other back problems
205	M9959	Spondylosis; intervertebral disc disorders; other back problems
205	M9960	Spondylosis; intervertebral disc disorders; other back problems
205	M9961	Spondylosis; intervertebral disc disorders; other back problems
205	M9962	Spondylosis; intervertebral disc disorders; other back problems
205	M9963	Spondylosis; intervertebral disc disorders; other back problems
205	M9964	Spondylosis; intervertebral disc disorders; other back problems

205	M9965	Spondylosis; intervertebral disc disorders; other back problems
205	M9966	Spondylosis; intervertebral disc disorders; other back problems
205	M9967	Spondylosis; intervertebral disc disorders; other back problems
205	M9968	Spondylosis; intervertebral disc disorders; other back problems
205	M9969	Spondylosis; intervertebral disc disorders; other back problems
205	M9970	Spondylosis; intervertebral disc disorders; other back problems
205	M9971	Spondylosis; intervertebral disc disorders; other back problems
205	M9972	Spondylosis; intervertebral disc disorders; other back problems
205	M9973	Spondylosis; intervertebral disc disorders; other back problems
205	M9974	Spondylosis; intervertebral disc disorders; other back problems
205	M9975	Spondylosis; intervertebral disc disorders; other back problems
205	M9976	Spondylosis; intervertebral disc disorders; other back problems
205	M9977	Spondylosis; intervertebral disc disorders; other back problems
205	M9978	Spondylosis; intervertebral disc disorders; other back problems
205	M9979	Spondylosis; intervertebral disc disorders; other back problems
206	M810	Osteoporosis
206	M816	Osteoporosis
206	M818	Osteoporosis
207	M8000XA	Pathological fracture
207	M8000XD	Pathological fracture
207	M8000XG	Pathological fracture
207	M8000XK	Pathological fracture
207	M8000XP	Pathological fracture
207	M8000XS	Pathological fracture
207	M80011A	Pathological fracture
207	M80011D	Pathological fracture
207	M80011G	Pathological fracture
207	M80011K	Pathological fracture
207	M80011P	Pathological fracture
207	M80011S	Pathological fracture
207	M80012A	Pathological fracture
207	M80012D	Pathological fracture
207	M80012G	Pathological fracture
207	M80012K	Pathological fracture
207	M80012P	Pathological fracture
207	M80012S	Pathological fracture
207	M80019A	Pathological fracture
207	M80019D	Pathological fracture
207	M80019G	Pathological fracture
207	M80019K	Pathological fracture
207	M80019P	Pathological fracture
207	M80019S	Pathological fracture
207	M80021A	Pathological fracture
207	M80021D	Pathological fracture
207	M80021G	Pathological fracture
207	M80021K	Pathological fracture
207	M80021P	Pathological fracture
207	M80021S	Pathological fracture
207	M80022A	Pathological fracture
207	M80022D	Pathological fracture
207	M80022G	Pathological fracture

207	M80022K	Pathological fracture
207	M80022P	Pathological fracture
207	M80022S	Pathological fracture
207	M80029A	Pathological fracture
207	M80029D	Pathological fracture
207	M80029G	Pathological fracture
207	M80029K	Pathological fracture
207	M80029P	Pathological fracture
207	M80029S	Pathological fracture
207	M80031A	Pathological fracture
207	M80031D	Pathological fracture
207	M80031G	Pathological fracture
207	M80031K	Pathological fracture
207	M80031P	Pathological fracture
207	M80031S	Pathological fracture
207	M80032A	Pathological fracture
207	M80032D	Pathological fracture
207	M80032G	Pathological fracture
207	M80032K	Pathological fracture
207	M80032P	Pathological fracture
207	M80032S	Pathological fracture
207	M80039A	Pathological fracture
207	M80039D	Pathological fracture
207	M80039G	Pathological fracture
207	M80039K	Pathological fracture
207	M80039P	Pathological fracture
207	M80039S	Pathological fracture
207	M80041A	Pathological fracture
207	M80041D	Pathological fracture
207	M80041G	Pathological fracture
207	M80041K	Pathological fracture
207	M80041P	Pathological fracture
207	M80041S	Pathological fracture
207	M80042A	Pathological fracture
207	M80042D	Pathological fracture
207	M80042G	Pathological fracture
207	M80042K	Pathological fracture
207	M80042P	Pathological fracture
207	M80042S	Pathological fracture
207	M80049A	Pathological fracture
207	M80049D	Pathological fracture
207	M80049G	Pathological fracture
207	M80049K	Pathological fracture
207	M80049P	Pathological fracture
207	M80049S	Pathological fracture
207	M80051A	Pathological fracture
207	M80051D	Pathological fracture
207	M80051G	Pathological fracture
207	M80051K	Pathological fracture
207	M80051P	Pathological fracture
207	M80051S	Pathological fracture

207	M80052A	Pathological fracture
207	M80052D	Pathological fracture
207	M80052G	Pathological fracture
207	M80052K	Pathological fracture
207	M80052P	Pathological fracture
207	M80052S	Pathological fracture
207	M80059A	Pathological fracture
207	M80059D	Pathological fracture
207	M80059G	Pathological fracture
207	M80059K	Pathological fracture
207	M80059P	Pathological fracture
207	M80059S	Pathological fracture
207	M80061A	Pathological fracture
207	M80061D	Pathological fracture
207	M80061G	Pathological fracture
207	M80061K	Pathological fracture
207	M80061P	Pathological fracture
207	M80061S	Pathological fracture
207	M80062A	Pathological fracture
207	M80062D	Pathological fracture
207	M80062G	Pathological fracture
207	M80062K	Pathological fracture
207	M80062P	Pathological fracture
207	M80062S	Pathological fracture
207	M80069A	Pathological fracture
207	M80069D	Pathological fracture
207	M80069G	Pathological fracture
207	M80069K	Pathological fracture
207	M80069P	Pathological fracture
207	M80069S	Pathological fracture
207	M80071A	Pathological fracture
207	M80071D	Pathological fracture
207	M80071G	Pathological fracture
207	M80071K	Pathological fracture
207	M80071P	Pathological fracture
207	M80071S	Pathological fracture
207	M80072A	Pathological fracture
207	M80072D	Pathological fracture
207	M80072G	Pathological fracture
207	M80072K	Pathological fracture
207	M80072P	Pathological fracture
207	M80072S	Pathological fracture
207	M80079A	Pathological fracture
207	M80079D	Pathological fracture
207	M80079G	Pathological fracture
207	M80079K	Pathological fracture
207	M80079P	Pathological fracture
207	M80079S	Pathological fracture
207	M8008XA	Pathological fracture
207	M8008XD	Pathological fracture
207	M8008XG	Pathological fracture

207	M8008XK	Pathological fracture
207	M8008XP	Pathological fracture
207	M8008XS	Pathological fracture
207	M8080XA	Pathological fracture
207	M8080XD	Pathological fracture
207	M8080XG	Pathological fracture
207	M8080XK	Pathological fracture
207	M8080XP	Pathological fracture
207	M8080XS	Pathological fracture
207	M80811A	Pathological fracture
207	M80811D	Pathological fracture
207	M80811G	Pathological fracture
207	M80811K	Pathological fracture
207	M80811P	Pathological fracture
207	M80811S	Pathological fracture
207	M80812A	Pathological fracture
207	M80812D	Pathological fracture
207	M80812G	Pathological fracture
207	M80812K	Pathological fracture
207	M80812P	Pathological fracture
207	M80812S	Pathological fracture
207	M80819A	Pathological fracture
207	M80819D	Pathological fracture
207	M80819G	Pathological fracture
207	M80819K	Pathological fracture
207	M80819P	Pathological fracture
207	M80819S	Pathological fracture
207	M80821A	Pathological fracture
207	M80821D	Pathological fracture
207	M80821G	Pathological fracture
207	M80821K	Pathological fracture
207	M80821P	Pathological fracture
207	M80821S	Pathological fracture
207	M80822A	Pathological fracture
207	M80822D	Pathological fracture
207	M80822G	Pathological fracture
207	M80822K	Pathological fracture
207	M80822P	Pathological fracture
207	M80822S	Pathological fracture
207	M80829A	Pathological fracture
207	M80829D	Pathological fracture
207	M80829G	Pathological fracture
207	M80829K	Pathological fracture
207	M80829P	Pathological fracture
207	M80829S	Pathological fracture
207	M80831A	Pathological fracture
207	M80831D	Pathological fracture
207	M80831G	Pathological fracture
207	M80831K	Pathological fracture
207	M80831P	Pathological fracture
207	M80831S	Pathological fracture

207	M80832A	Pathological fracture
207	M80832D	Pathological fracture
207	M80832G	Pathological fracture
207	M80832K	Pathological fracture
207	M80832P	Pathological fracture
207	M80832S	Pathological fracture
207	M80839A	Pathological fracture
207	M80839D	Pathological fracture
207	M80839G	Pathological fracture
207	M80839K	Pathological fracture
207	M80839P	Pathological fracture
207	M80839S	Pathological fracture
207	M80841A	Pathological fracture
207	M80841D	Pathological fracture
207	M80841G	Pathological fracture
207	M80841K	Pathological fracture
207	M80841P	Pathological fracture
207	M80841S	Pathological fracture
207	M80842A	Pathological fracture
207	M80842D	Pathological fracture
207	M80842G	Pathological fracture
207	M80842K	Pathological fracture
207	M80842P	Pathological fracture
207	M80842S	Pathological fracture
207	M80849A	Pathological fracture
207	M80849D	Pathological fracture
207	M80849G	Pathological fracture
207	M80849K	Pathological fracture
207	M80849P	Pathological fracture
207	M80849S	Pathological fracture
207	M80851A	Pathological fracture
207	M80851D	Pathological fracture
207	M80851G	Pathological fracture
207	M80851K	Pathological fracture
207	M80851P	Pathological fracture
207	M80851S	Pathological fracture
207	M80852A	Pathological fracture
207	M80852D	Pathological fracture
207	M80852G	Pathological fracture
207	M80852K	Pathological fracture
207	M80852P	Pathological fracture
207	M80852S	Pathological fracture
207	M80859A	Pathological fracture
207	M80859D	Pathological fracture
207	M80859G	Pathological fracture
207	M80859K	Pathological fracture
207	M80859P	Pathological fracture
207	M80859S	Pathological fracture
207	M80861A	Pathological fracture
207	M80861D	Pathological fracture
207	M80861G	Pathological fracture

207	M80861K	Pathological fracture
207	M80861P	Pathological fracture
207	M80861S	Pathological fracture
207	M80862A	Pathological fracture
207	M80862D	Pathological fracture
207	M80862G	Pathological fracture
207	M80862K	Pathological fracture
207	M80862P	Pathological fracture
207	M80862S	Pathological fracture
207	M80869A	Pathological fracture
207	M80869D	Pathological fracture
207	M80869G	Pathological fracture
207	M80869K	Pathological fracture
207	M80869P	Pathological fracture
207	M80869S	Pathological fracture
207	M80871A	Pathological fracture
207	M80871D	Pathological fracture
207	M80871G	Pathological fracture
207	M80871K	Pathological fracture
207	M80871P	Pathological fracture
207	M80871S	Pathological fracture
207	M80872A	Pathological fracture
207	M80872D	Pathological fracture
207	M80872G	Pathological fracture
207	M80872K	Pathological fracture
207	M80872P	Pathological fracture
207	M80872S	Pathological fracture
207	M80879A	Pathological fracture
207	M80879D	Pathological fracture
207	M80879G	Pathological fracture
207	M80879K	Pathological fracture
207	M80879P	Pathological fracture
207	M80879S	Pathological fracture
207	M8088XA	Pathological fracture
207	M8088XD	Pathological fracture
207	M8088XG	Pathological fracture
207	M8088XK	Pathological fracture
207	M8088XP	Pathological fracture
207	M8088XS	Pathological fracture
207	M8440XA	Pathological fracture
207	M8440XD	Pathological fracture
207	M8440XG	Pathological fracture
207	M8440XK	Pathological fracture
207	M8440XP	Pathological fracture
207	M8440XS	Pathological fracture
207	M84411A	Pathological fracture
207	M84411D	Pathological fracture
207	M84411G	Pathological fracture
207	M84411K	Pathological fracture
207	M84411P	Pathological fracture
207	M84411S	Pathological fracture

207	M84412A	Pathological fracture
207	M84412D	Pathological fracture
207	M84412G	Pathological fracture
207	M84412K	Pathological fracture
207	M84412P	Pathological fracture
207	M84412S	Pathological fracture
207	M84419A	Pathological fracture
207	M84419D	Pathological fracture
207	M84419G	Pathological fracture
207	M84419K	Pathological fracture
207	M84419P	Pathological fracture
207	M84419S	Pathological fracture
207	M84421A	Pathological fracture
207	M84421D	Pathological fracture
207	M84421G	Pathological fracture
207	M84421K	Pathological fracture
207	M84421P	Pathological fracture
207	M84421S	Pathological fracture
207	M84422A	Pathological fracture
207	M84422D	Pathological fracture
207	M84422G	Pathological fracture
207	M84422K	Pathological fracture
207	M84422P	Pathological fracture
207	M84422S	Pathological fracture
207	M84429A	Pathological fracture
207	M84429D	Pathological fracture
207	M84429G	Pathological fracture
207	M84429K	Pathological fracture
207	M84429P	Pathological fracture
207	M84429S	Pathological fracture
207	M84431A	Pathological fracture
207	M84431D	Pathological fracture
207	M84431G	Pathological fracture
207	M84431K	Pathological fracture
207	M84431P	Pathological fracture
207	M84431S	Pathological fracture
207	M84432A	Pathological fracture
207	M84432D	Pathological fracture
207	M84432G	Pathological fracture
207	M84432K	Pathological fracture
207	M84432P	Pathological fracture
207	M84432S	Pathological fracture
207	M84433A	Pathological fracture
207	M84433D	Pathological fracture
207	M84433G	Pathological fracture
207	M84433K	Pathological fracture
207	M84433P	Pathological fracture
207	M84433S	Pathological fracture
207	M84434A	Pathological fracture
207	M84434D	Pathological fracture
207	M84434G	Pathological fracture

207	M84434K	Pathological fracture
207	M84434P	Pathological fracture
207	M84434S	Pathological fracture
207	M84439A	Pathological fracture
207	M84439D	Pathological fracture
207	M84439G	Pathological fracture
207	M84439K	Pathological fracture
207	M84439P	Pathological fracture
207	M84439S	Pathological fracture
207	M84441A	Pathological fracture
207	M84441D	Pathological fracture
207	M84441G	Pathological fracture
207	M84441K	Pathological fracture
207	M84441P	Pathological fracture
207	M84441S	Pathological fracture
207	M84442A	Pathological fracture
207	M84442D	Pathological fracture
207	M84442G	Pathological fracture
207	M84442K	Pathological fracture
207	M84442P	Pathological fracture
207	M84442S	Pathological fracture
207	M84443A	Pathological fracture
207	M84443D	Pathological fracture
207	M84443G	Pathological fracture
207	M84443K	Pathological fracture
207	M84443P	Pathological fracture
207	M84443S	Pathological fracture
207	M84444A	Pathological fracture
207	M84444D	Pathological fracture
207	M84444G	Pathological fracture
207	M84444K	Pathological fracture
207	M84444P	Pathological fracture
207	M84444S	Pathological fracture
207	M84445A	Pathological fracture
207	M84445D	Pathological fracture
207	M84445G	Pathological fracture
207	M84445K	Pathological fracture
207	M84445P	Pathological fracture
207	M84445S	Pathological fracture
207	M84446A	Pathological fracture
207	M84446D	Pathological fracture
207	M84446G	Pathological fracture
207	M84446K	Pathological fracture
207	M84446P	Pathological fracture
207	M84446S	Pathological fracture
207	M84451A	Pathological fracture
207	M84451D	Pathological fracture
207	M84451G	Pathological fracture
207	M84451K	Pathological fracture
207	M84451P	Pathological fracture
207	M84451S	Pathological fracture

207	M84452A	Pathological fracture
207	M84452D	Pathological fracture
207	M84452G	Pathological fracture
207	M84452K	Pathological fracture
207	M84452P	Pathological fracture
207	M84452S	Pathological fracture
207	M84453A	Pathological fracture
207	M84453D	Pathological fracture
207	M84453G	Pathological fracture
207	M84453K	Pathological fracture
207	M84453P	Pathological fracture
207	M84453S	Pathological fracture
207	M84454A	Pathological fracture
207	M84454D	Pathological fracture
207	M84454G	Pathological fracture
207	M84454K	Pathological fracture
207	M84454P	Pathological fracture
207	M84454S	Pathological fracture
207	M84459A	Pathological fracture
207	M84459D	Pathological fracture
207	M84459G	Pathological fracture
207	M84459K	Pathological fracture
207	M84459P	Pathological fracture
207	M84459S	Pathological fracture
207	M84461A	Pathological fracture
207	M84461D	Pathological fracture
207	M84461G	Pathological fracture
207	M84461K	Pathological fracture
207	M84461P	Pathological fracture
207	M84461S	Pathological fracture
207	M84462A	Pathological fracture
207	M84462D	Pathological fracture
207	M84462G	Pathological fracture
207	M84462K	Pathological fracture
207	M84462P	Pathological fracture
207	M84462S	Pathological fracture
207	M84463A	Pathological fracture
207	M84463D	Pathological fracture
207	M84463G	Pathological fracture
207	M84463K	Pathological fracture
207	M84463P	Pathological fracture
207	M84463S	Pathological fracture
207	M84464A	Pathological fracture
207	M84464D	Pathological fracture
207	M84464G	Pathological fracture
207	M84464K	Pathological fracture
207	M84464P	Pathological fracture
207	M84464S	Pathological fracture
207	M84469A	Pathological fracture
207	M84469D	Pathological fracture
207	M84469G	Pathological fracture

207	M84469K	Pathological fracture
207	M84469P	Pathological fracture
207	M84469S	Pathological fracture
207	M84471A	Pathological fracture
207	M84471D	Pathological fracture
207	M84471G	Pathological fracture
207	M84471K	Pathological fracture
207	M84471P	Pathological fracture
207	M84471S	Pathological fracture
207	M84472A	Pathological fracture
207	M84472D	Pathological fracture
207	M84472G	Pathological fracture
207	M84472K	Pathological fracture
207	M84472P	Pathological fracture
207	M84472S	Pathological fracture
207	M84473A	Pathological fracture
207	M84473D	Pathological fracture
207	M84473G	Pathological fracture
207	M84473K	Pathological fracture
207	M84473P	Pathological fracture
207	M84473S	Pathological fracture
207	M84474A	Pathological fracture
207	M84474D	Pathological fracture
207	M84474G	Pathological fracture
207	M84474K	Pathological fracture
207	M84474P	Pathological fracture
207	M84474S	Pathological fracture
207	M84475A	Pathological fracture
207	M84475D	Pathological fracture
207	M84475G	Pathological fracture
207	M84475K	Pathological fracture
207	M84475P	Pathological fracture
207	M84475S	Pathological fracture
207	M84476A	Pathological fracture
207	M84476D	Pathological fracture
207	M84476G	Pathological fracture
207	M84476K	Pathological fracture
207	M84476P	Pathological fracture
207	M84476S	Pathological fracture
207	M84477A	Pathological fracture
207	M84477D	Pathological fracture
207	M84477G	Pathological fracture
207	M84477K	Pathological fracture
207	M84477P	Pathological fracture
207	M84477S	Pathological fracture
207	M84478A	Pathological fracture
207	M84478D	Pathological fracture
207	M84478G	Pathological fracture
207	M84478K	Pathological fracture
207	M84478P	Pathological fracture
207	M84478S	Pathological fracture

207	M84479A	Pathological fracture
207	M84479D	Pathological fracture
207	M84479G	Pathological fracture
207	M84479K	Pathological fracture
207	M84479P	Pathological fracture
207	M84479S	Pathological fracture
207	M8448XA	Pathological fracture
207	M8448XD	Pathological fracture
207	M8448XG	Pathological fracture
207	M8448XK	Pathological fracture
207	M8448XP	Pathological fracture
207	M8448XS	Pathological fracture
207	M8450XA	Pathological fracture
207	M8450XD	Pathological fracture
207	M8450XG	Pathological fracture
207	M8450XK	Pathological fracture
207	M8450XP	Pathological fracture
207	M8450XS	Pathological fracture
207	M84511A	Pathological fracture
207	M84511D	Pathological fracture
207	M84511G	Pathological fracture
207	M84511K	Pathological fracture
207	M84511P	Pathological fracture
207	M84511S	Pathological fracture
207	M84512A	Pathological fracture
207	M84512D	Pathological fracture
207	M84512G	Pathological fracture
207	M84512K	Pathological fracture
207	M84512P	Pathological fracture
207	M84512S	Pathological fracture
207	M84519A	Pathological fracture
207	M84519D	Pathological fracture
207	M84519G	Pathological fracture
207	M84519K	Pathological fracture
207	M84519P	Pathological fracture
207	M84519S	Pathological fracture
207	M84521A	Pathological fracture
207	M84521D	Pathological fracture
207	M84521G	Pathological fracture
207	M84521K	Pathological fracture
207	M84521P	Pathological fracture
207	M84521S	Pathological fracture
207	M84522A	Pathological fracture
207	M84522D	Pathological fracture
207	M84522G	Pathological fracture
207	M84522K	Pathological fracture
207	M84522P	Pathological fracture
207	M84522S	Pathological fracture
207	M84529A	Pathological fracture
207	M84529D	Pathological fracture
207	M84529G	Pathological fracture

207	M84529K	Pathological fracture
207	M84529P	Pathological fracture
207	M84529S	Pathological fracture
207	M84531A	Pathological fracture
207	M84531D	Pathological fracture
207	M84531G	Pathological fracture
207	M84531K	Pathological fracture
207	M84531P	Pathological fracture
207	M84531S	Pathological fracture
207	M84532A	Pathological fracture
207	M84532D	Pathological fracture
207	M84532G	Pathological fracture
207	M84532K	Pathological fracture
207	M84532P	Pathological fracture
207	M84532S	Pathological fracture
207	M84533A	Pathological fracture
207	M84533D	Pathological fracture
207	M84533G	Pathological fracture
207	M84533K	Pathological fracture
207	M84533P	Pathological fracture
207	M84533S	Pathological fracture
207	M84534A	Pathological fracture
207	M84534D	Pathological fracture
207	M84534G	Pathological fracture
207	M84534K	Pathological fracture
207	M84534P	Pathological fracture
207	M84534S	Pathological fracture
207	M84539A	Pathological fracture
207	M84539D	Pathological fracture
207	M84539G	Pathological fracture
207	M84539K	Pathological fracture
207	M84539P	Pathological fracture
207	M84539S	Pathological fracture
207	M84541A	Pathological fracture
207	M84541D	Pathological fracture
207	M84541G	Pathological fracture
207	M84541K	Pathological fracture
207	M84541P	Pathological fracture
207	M84541S	Pathological fracture
207	M84542A	Pathological fracture
207	M84542D	Pathological fracture
207	M84542G	Pathological fracture
207	M84542K	Pathological fracture
207	M84542P	Pathological fracture
207	M84542S	Pathological fracture
207	M84549A	Pathological fracture
207	M84549D	Pathological fracture
207	M84549G	Pathological fracture
207	M84549K	Pathological fracture
207	M84549P	Pathological fracture
207	M84549S	Pathological fracture

207	M84550A	Pathological fracture
207	M84550D	Pathological fracture
207	M84550G	Pathological fracture
207	M84550K	Pathological fracture
207	M84550P	Pathological fracture
207	M84550S	Pathological fracture
207	M84551A	Pathological fracture
207	M84551D	Pathological fracture
207	M84551G	Pathological fracture
207	M84551K	Pathological fracture
207	M84551P	Pathological fracture
207	M84551S	Pathological fracture
207	M84552A	Pathological fracture
207	M84552D	Pathological fracture
207	M84552G	Pathological fracture
207	M84552K	Pathological fracture
207	M84552P	Pathological fracture
207	M84552S	Pathological fracture
207	M84553A	Pathological fracture
207	M84553D	Pathological fracture
207	M84553G	Pathological fracture
207	M84553K	Pathological fracture
207	M84553P	Pathological fracture
207	M84553S	Pathological fracture
207	M84559A	Pathological fracture
207	M84559D	Pathological fracture
207	M84559G	Pathological fracture
207	M84559K	Pathological fracture
207	M84559P	Pathological fracture
207	M84559S	Pathological fracture
207	M84561A	Pathological fracture
207	M84561D	Pathological fracture
207	M84561G	Pathological fracture
207	M84561K	Pathological fracture
207	M84561P	Pathological fracture
207	M84561S	Pathological fracture
207	M84562A	Pathological fracture
207	M84562D	Pathological fracture
207	M84562G	Pathological fracture
207	M84562K	Pathological fracture
207	M84562P	Pathological fracture
207	M84562S	Pathological fracture
207	M84563A	Pathological fracture
207	M84563D	Pathological fracture
207	M84563G	Pathological fracture
207	M84563K	Pathological fracture
207	M84563P	Pathological fracture
207	M84563S	Pathological fracture
207	M84564A	Pathological fracture
207	M84564D	Pathological fracture
207	M84564G	Pathological fracture

207	M84564K	Pathological fracture
207	M84564P	Pathological fracture
207	M84564S	Pathological fracture
207	M84569A	Pathological fracture
207	M84569D	Pathological fracture
207	M84569G	Pathological fracture
207	M84569K	Pathological fracture
207	M84569P	Pathological fracture
207	M84569S	Pathological fracture
207	M84571A	Pathological fracture
207	M84571D	Pathological fracture
207	M84571G	Pathological fracture
207	M84571K	Pathological fracture
207	M84571P	Pathological fracture
207	M84571S	Pathological fracture
207	M84572A	Pathological fracture
207	M84572D	Pathological fracture
207	M84572G	Pathological fracture
207	M84572K	Pathological fracture
207	M84572P	Pathological fracture
207	M84572S	Pathological fracture
207	M84573A	Pathological fracture
207	M84573D	Pathological fracture
207	M84573G	Pathological fracture
207	M84573K	Pathological fracture
207	M84573P	Pathological fracture
207	M84573S	Pathological fracture
207	M84574A	Pathological fracture
207	M84574D	Pathological fracture
207	M84574G	Pathological fracture
207	M84574K	Pathological fracture
207	M84574P	Pathological fracture
207	M84574S	Pathological fracture
207	M84575A	Pathological fracture
207	M84575D	Pathological fracture
207	M84575G	Pathological fracture
207	M84575K	Pathological fracture
207	M84575P	Pathological fracture
207	M84575S	Pathological fracture
207	M84576A	Pathological fracture
207	M84576D	Pathological fracture
207	M84576G	Pathological fracture
207	M84576K	Pathological fracture
207	M84576P	Pathological fracture
207	M84576S	Pathological fracture
207	M8458XA	Pathological fracture
207	M8458XD	Pathological fracture
207	M8458XG	Pathological fracture
207	M8458XK	Pathological fracture
207	M8458XP	Pathological fracture
207	M8458XS	Pathological fracture

207	M8460XA	Pathological fracture
207	M8460XD	Pathological fracture
207	M8460XG	Pathological fracture
207	M8460XK	Pathological fracture
207	M8460XP	Pathological fracture
207	M8460XS	Pathological fracture
207	M84611A	Pathological fracture
207	M84611D	Pathological fracture
207	M84611G	Pathological fracture
207	M84611K	Pathological fracture
207	M84611P	Pathological fracture
207	M84611S	Pathological fracture
207	M84612A	Pathological fracture
207	M84612D	Pathological fracture
207	M84612G	Pathological fracture
207	M84612K	Pathological fracture
207	M84612P	Pathological fracture
207	M84612S	Pathological fracture
207	M84619A	Pathological fracture
207	M84619D	Pathological fracture
207	M84619G	Pathological fracture
207	M84619K	Pathological fracture
207	M84619P	Pathological fracture
207	M84619S	Pathological fracture
207	M84621A	Pathological fracture
207	M84621D	Pathological fracture
207	M84621G	Pathological fracture
207	M84621K	Pathological fracture
207	M84621P	Pathological fracture
207	M84621S	Pathological fracture
207	M84622A	Pathological fracture
207	M84622D	Pathological fracture
207	M84622G	Pathological fracture
207	M84622K	Pathological fracture
207	M84622P	Pathological fracture
207	M84622S	Pathological fracture
207	M84629A	Pathological fracture
207	M84629D	Pathological fracture
207	M84629G	Pathological fracture
207	M84629K	Pathological fracture
207	M84629P	Pathological fracture
207	M84629S	Pathological fracture
207	M84631A	Pathological fracture
207	M84631D	Pathological fracture
207	M84631G	Pathological fracture
207	M84631K	Pathological fracture
207	M84631P	Pathological fracture
207	M84631S	Pathological fracture
207	M84632A	Pathological fracture
207	M84632D	Pathological fracture
207	M84632G	Pathological fracture

207	M84632K	Pathological fracture
207	M84632P	Pathological fracture
207	M84632S	Pathological fracture
207	M84633A	Pathological fracture
207	M84633D	Pathological fracture
207	M84633G	Pathological fracture
207	M84633K	Pathological fracture
207	M84633P	Pathological fracture
207	M84633S	Pathological fracture
207	M84634A	Pathological fracture
207	M84634D	Pathological fracture
207	M84634G	Pathological fracture
207	M84634K	Pathological fracture
207	M84634P	Pathological fracture
207	M84634S	Pathological fracture
207	M84639A	Pathological fracture
207	M84639D	Pathological fracture
207	M84639G	Pathological fracture
207	M84639K	Pathological fracture
207	M84639P	Pathological fracture
207	M84639S	Pathological fracture
207	M84641A	Pathological fracture
207	M84641D	Pathological fracture
207	M84641G	Pathological fracture
207	M84641K	Pathological fracture
207	M84641P	Pathological fracture
207	M84641S	Pathological fracture
207	M84642A	Pathological fracture
207	M84642D	Pathological fracture
207	M84642G	Pathological fracture
207	M84642K	Pathological fracture
207	M84642P	Pathological fracture
207	M84642S	Pathological fracture
207	M84649A	Pathological fracture
207	M84649D	Pathological fracture
207	M84649G	Pathological fracture
207	M84649K	Pathological fracture
207	M84649P	Pathological fracture
207	M84649S	Pathological fracture
207	M84650A	Pathological fracture
207	M84650D	Pathological fracture
207	M84650G	Pathological fracture
207	M84650K	Pathological fracture
207	M84650P	Pathological fracture
207	M84650S	Pathological fracture
207	M84651A	Pathological fracture
207	M84651D	Pathological fracture
207	M84651G	Pathological fracture
207	M84651K	Pathological fracture
207	M84651P	Pathological fracture
207	M84651S	Pathological fracture

207	M84652A	Pathological fracture
207	M84652D	Pathological fracture
207	M84652G	Pathological fracture
207	M84652K	Pathological fracture
207	M84652P	Pathological fracture
207	M84652S	Pathological fracture
207	M84653A	Pathological fracture
207	M84653D	Pathological fracture
207	M84653G	Pathological fracture
207	M84653K	Pathological fracture
207	M84653P	Pathological fracture
207	M84653S	Pathological fracture
207	M84659A	Pathological fracture
207	M84659D	Pathological fracture
207	M84659G	Pathological fracture
207	M84659K	Pathological fracture
207	M84659P	Pathological fracture
207	M84659S	Pathological fracture
207	M84661A	Pathological fracture
207	M84661D	Pathological fracture
207	M84661G	Pathological fracture
207	M84661K	Pathological fracture
207	M84661P	Pathological fracture
207	M84661S	Pathological fracture
207	M84662A	Pathological fracture
207	M84662D	Pathological fracture
207	M84662G	Pathological fracture
207	M84662K	Pathological fracture
207	M84662P	Pathological fracture
207	M84662S	Pathological fracture
207	M84663A	Pathological fracture
207	M84663D	Pathological fracture
207	M84663G	Pathological fracture
207	M84663K	Pathological fracture
207	M84663P	Pathological fracture
207	M84663S	Pathological fracture
207	M84664A	Pathological fracture
207	M84664D	Pathological fracture
207	M84664G	Pathological fracture
207	M84664K	Pathological fracture
207	M84664P	Pathological fracture
207	M84664S	Pathological fracture
207	M84669A	Pathological fracture
207	M84669D	Pathological fracture
207	M84669G	Pathological fracture
207	M84669K	Pathological fracture
207	M84669P	Pathological fracture
207	M84669S	Pathological fracture
207	M84671A	Pathological fracture
207	M84671D	Pathological fracture
207	M84671G	Pathological fracture

207	M84671K	Pathological fracture
207	M84671P	Pathological fracture
207	M84671S	Pathological fracture
207	M84672A	Pathological fracture
207	M84672D	Pathological fracture
207	M84672G	Pathological fracture
207	M84672K	Pathological fracture
207	M84672P	Pathological fracture
207	M84672S	Pathological fracture
207	M84673A	Pathological fracture
207	M84673D	Pathological fracture
207	M84673G	Pathological fracture
207	M84673K	Pathological fracture
207	M84673P	Pathological fracture
207	M84673S	Pathological fracture
207	M84674A	Pathological fracture
207	M84674D	Pathological fracture
207	M84674G	Pathological fracture
207	M84674K	Pathological fracture
207	M84674P	Pathological fracture
207	M84674S	Pathological fracture
207	M84675A	Pathological fracture
207	M84675D	Pathological fracture
207	M84675G	Pathological fracture
207	M84675K	Pathological fracture
207	M84675P	Pathological fracture
207	M84675S	Pathological fracture
207	M84676A	Pathological fracture
207	M84676D	Pathological fracture
207	M84676G	Pathological fracture
207	M84676K	Pathological fracture
207	M84676P	Pathological fracture
207	M84676S	Pathological fracture
207	M8468XA	Pathological fracture
207	M8468XD	Pathological fracture
207	M8468XG	Pathological fracture
207	M8468XK	Pathological fracture
207	M8468XP	Pathological fracture
207	M8468XS	Pathological fracture
207	Z87310	Pathological fracture
207	Z87311	Pathological fracture
208	M2010	Acquired foot deformities
208	M2011	Acquired foot deformities
208	M2012	Acquired foot deformities
208	M2020	Acquired foot deformities
208	M2021	Acquired foot deformities
208	M2022	Acquired foot deformities
208	M2030	Acquired foot deformities
208	M2031	Acquired foot deformities
208	M2032	Acquired foot deformities
208	M2040	Acquired foot deformities

208	M2041	Acquired foot deformities
208	M2042	Acquired foot deformities
208	M205X1	Acquired foot deformities
208	M205X2	Acquired foot deformities
208	M205X9	Acquired foot deformities
208	M2060	Acquired foot deformities
208	M2061	Acquired foot deformities
208	M2062	Acquired foot deformities
208	M21071	Acquired foot deformities
208	M21072	Acquired foot deformities
208	M21079	Acquired foot deformities
208	M21171	Acquired foot deformities
208	M21172	Acquired foot deformities
208	M21179	Acquired foot deformities
208	M21371	Acquired foot deformities
208	M21372	Acquired foot deformities
208	M21379	Acquired foot deformities
208	M2140	Acquired foot deformities
208	M2141	Acquired foot deformities
208	M2142	Acquired foot deformities
208	M21531	Acquired foot deformities
208	M21532	Acquired foot deformities
208	M21539	Acquired foot deformities
208	M21541	Acquired foot deformities
208	M21542	Acquired foot deformities
208	M21549	Acquired foot deformities
208	M216X1	Acquired foot deformities
208	M216X2	Acquired foot deformities
208	M216X9	Acquired foot deformities
209	M20001	Other acquired deformities
209	M20002	Other acquired deformities
209	M20009	Other acquired deformities
209	M20011	Other acquired deformities
209	M20012	Other acquired deformities
209	M20019	Other acquired deformities
209	M20021	Other acquired deformities
209	M20022	Other acquired deformities
209	M20029	Other acquired deformities
209	M20031	Other acquired deformities
209	M20032	Other acquired deformities
209	M20039	Other acquired deformities
209	M20091	Other acquired deformities
209	M20092	Other acquired deformities
209	M20099	Other acquired deformities
209	M2100	Other acquired deformities
209	M21021	Other acquired deformities
209	M21022	Other acquired deformities
209	M21029	Other acquired deformities
209	M21051	Other acquired deformities
209	M21052	Other acquired deformities
209	M21059	Other acquired deformities

209	M21061	Other acquired deformities
209	M21062	Other acquired deformities
209	M21069	Other acquired deformities
209	M2110	Other acquired deformities
209	M21121	Other acquired deformities
209	M21122	Other acquired deformities
209	M21129	Other acquired deformities
209	M21151	Other acquired deformities
209	M21152	Other acquired deformities
209	M21159	Other acquired deformities
209	M21161	Other acquired deformities
209	M21162	Other acquired deformities
209	M21169	Other acquired deformities
209	M2120	Other acquired deformities
209	M21211	Other acquired deformities
209	M21212	Other acquired deformities
209	M21219	Other acquired deformities
209	M21221	Other acquired deformities
209	M21222	Other acquired deformities
209	M21229	Other acquired deformities
209	M21231	Other acquired deformities
209	M21232	Other acquired deformities
209	M21239	Other acquired deformities
209	M21241	Other acquired deformities
209	M21242	Other acquired deformities
209	M21249	Other acquired deformities
209	M21251	Other acquired deformities
209	M21252	Other acquired deformities
209	M21259	Other acquired deformities
209	M21261	Other acquired deformities
209	M21262	Other acquired deformities
209	M21269	Other acquired deformities
209	M21271	Other acquired deformities
209	M21272	Other acquired deformities
209	M21279	Other acquired deformities
209	M21331	Other acquired deformities
209	M21332	Other acquired deformities
209	M21339	Other acquired deformities
209	M21511	Other acquired deformities
209	M21512	Other acquired deformities
209	M21519	Other acquired deformities
209	M21521	Other acquired deformities
209	M21522	Other acquired deformities
209	M21529	Other acquired deformities
209	M2170	Other acquired deformities
209	M21721	Other acquired deformities
209	M21722	Other acquired deformities
209	M21729	Other acquired deformities
209	M21731	Other acquired deformities
209	M21732	Other acquired deformities
209	M21733	Other acquired deformities

209	M21734	Other acquired deformities
209	M21739	Other acquired deformities
209	M21751	Other acquired deformities
209	M21752	Other acquired deformities
209	M21759	Other acquired deformities
209	M21761	Other acquired deformities
209	M21762	Other acquired deformities
209	M21763	Other acquired deformities
209	M21764	Other acquired deformities
209	M21769	Other acquired deformities
209	M2180	Other acquired deformities
209	M21821	Other acquired deformities
209	M21822	Other acquired deformities
209	M21829	Other acquired deformities
209	M21831	Other acquired deformities
209	M21832	Other acquired deformities
209	M21839	Other acquired deformities
209	M21851	Other acquired deformities
209	M21852	Other acquired deformities
209	M21859	Other acquired deformities
209	M21861	Other acquired deformities
209	M21862	Other acquired deformities
209	M21869	Other acquired deformities
209	M2190	Other acquired deformities
209	M21921	Other acquired deformities
209	M21922	Other acquired deformities
209	M21929	Other acquired deformities
209	M21931	Other acquired deformities
209	M21932	Other acquired deformities
209	M21939	Other acquired deformities
209	M21941	Other acquired deformities
209	M21942	Other acquired deformities
209	M21949	Other acquired deformities
209	M21951	Other acquired deformities
209	M21952	Other acquired deformities
209	M21959	Other acquired deformities
209	M21961	Other acquired deformities
209	M21962	Other acquired deformities
209	M21969	Other acquired deformities
209	M2450	Other acquired deformities
209	M24511	Other acquired deformities
209	M24512	Other acquired deformities
209	M24519	Other acquired deformities
209	M24521	Other acquired deformities
209	M24522	Other acquired deformities
209	M24529	Other acquired deformities
209	M24531	Other acquired deformities
209	M24532	Other acquired deformities
209	M24539	Other acquired deformities
209	M24541	Other acquired deformities
209	M24542	Other acquired deformities

209	M24549	Other acquired deformities
209	M24551	Other acquired deformities
209	M24552	Other acquired deformities
209	M24559	Other acquired deformities
209	M24561	Other acquired deformities
209	M24562	Other acquired deformities
209	M24569	Other acquired deformities
209	M24571	Other acquired deformities
209	M24572	Other acquired deformities
209	M24573	Other acquired deformities
209	M24574	Other acquired deformities
209	M24575	Other acquired deformities
209	M24576	Other acquired deformities
209	M4000	Other acquired deformities
209	M4003	Other acquired deformities
209	M4004	Other acquired deformities
209	M4005	Other acquired deformities
209	M4010	Other acquired deformities
209	M4012	Other acquired deformities
209	M4013	Other acquired deformities
209	M4014	Other acquired deformities
209	M4015	Other acquired deformities
209	M40202	Other acquired deformities
209	M40203	Other acquired deformities
209	M40204	Other acquired deformities
209	M40205	Other acquired deformities
209	M40209	Other acquired deformities
209	M40292	Other acquired deformities
209	M40293	Other acquired deformities
209	M40294	Other acquired deformities
209	M40295	Other acquired deformities
209	M40299	Other acquired deformities
209	M4030	Other acquired deformities
209	M4035	Other acquired deformities
209	M4036	Other acquired deformities
209	M4037	Other acquired deformities
209	M4040	Other acquired deformities
209	M4045	Other acquired deformities
209	M4046	Other acquired deformities
209	M4047	Other acquired deformities
209	M4050	Other acquired deformities
209	M4055	Other acquired deformities
209	M4056	Other acquired deformities
209	M4057	Other acquired deformities
209	M4130	Other acquired deformities
209	M4134	Other acquired deformities
209	M4135	Other acquired deformities
209	M4140	Other acquired deformities
209	M4141	Other acquired deformities
209	M4142	Other acquired deformities
209	M4143	Other acquired deformities

209	M4144	Other acquired deformities
209	M4145	Other acquired deformities
209	M4146	Other acquired deformities
209	M4147	Other acquired deformities
209	M4150	Other acquired deformities
209	M4152	Other acquired deformities
209	M4153	Other acquired deformities
209	M4154	Other acquired deformities
209	M4155	Other acquired deformities
209	M4156	Other acquired deformities
209	M4157	Other acquired deformities
209	M4180	Other acquired deformities
209	M4182	Other acquired deformities
209	M4183	Other acquired deformities
209	M4184	Other acquired deformities
209	M4185	Other acquired deformities
209	M4186	Other acquired deformities
209	M4187	Other acquired deformities
209	M419	Other acquired deformities
209	M4300	Other acquired deformities
209	M4301	Other acquired deformities
209	M4302	Other acquired deformities
209	M4303	Other acquired deformities
209	M4304	Other acquired deformities
209	M4305	Other acquired deformities
209	M4306	Other acquired deformities
209	M4307	Other acquired deformities
209	M4308	Other acquired deformities
209	M4309	Other acquired deformities
209	M4310	Other acquired deformities
209	M4311	Other acquired deformities
209	M4312	Other acquired deformities
209	M4313	Other acquired deformities
209	M4314	Other acquired deformities
209	M4315	Other acquired deformities
209	M4316	Other acquired deformities
209	M4317	Other acquired deformities
209	M4318	Other acquired deformities
209	M4319	Other acquired deformities
209	M438X1	Other acquired deformities
209	M438X2	Other acquired deformities
209	M438X3	Other acquired deformities
209	M438X4	Other acquired deformities
209	M438X5	Other acquired deformities
209	M438X6	Other acquired deformities
209	M438X7	Other acquired deformities
209	M438X8	Other acquired deformities
209	M438X9	Other acquired deformities
209	M439	Other acquired deformities
209	M950	Other acquired deformities
209	M9510	Other acquired deformities

209	M9511	Other acquired deformities
209	M9512	Other acquired deformities
209	M952	Other acquired deformities
209	M953	Other acquired deformities
209	M954	Other acquired deformities
209	M955	Other acquired deformities
209	M958	Other acquired deformities
209	M959	Other acquired deformities
209	M962	Other acquired deformities
209	M963	Other acquired deformities
209	M964	Other acquired deformities
209	M965	Other acquired deformities
209	M9980	Other acquired deformities
209	M9981	Other acquired deformities
209	M9982	Other acquired deformities
209	M9983	Other acquired deformities
209	M9984	Other acquired deformities
209	M9985	Other acquired deformities
209	M9986	Other acquired deformities
209	M9987	Other acquired deformities
209	M9988	Other acquired deformities
209	M9989	Other acquired deformities
209	M999	Other acquired deformities
210	M300	Systemic lupus erythematosus and connective tissue disorders
210	M301	Systemic lupus erythematosus and connective tissue disorders
210	M302	Systemic lupus erythematosus and connective tissue disorders
210	M303	Systemic lupus erythematosus and connective tissue disorders
210	M308	Systemic lupus erythematosus and connective tissue disorders
210	M311	Systemic lupus erythematosus and connective tissue disorders
210	M312	Systemic lupus erythematosus and connective tissue disorders
210	M3130	Systemic lupus erythematosus and connective tissue disorders
210	M3131	Systemic lupus erythematosus and connective tissue disorders
210	M314	Systemic lupus erythematosus and connective tissue disorders
210	M315	Systemic lupus erythematosus and connective tissue disorders
210	M316	Systemic lupus erythematosus and connective tissue disorders
210	M317	Systemic lupus erythematosus and connective tissue disorders
210	M318	Systemic lupus erythematosus and connective tissue disorders
210	M319	Systemic lupus erythematosus and connective tissue disorders
210	M320	Systemic lupus erythematosus and connective tissue disorders
210	M3210	Systemic lupus erythematosus and connective tissue disorders
210	M3211	Systemic lupus erythematosus and connective tissue disorders
210	M3212	Systemic lupus erythematosus and connective tissue disorders
210	M3213	Systemic lupus erythematosus and connective tissue disorders
210	M3214	Systemic lupus erythematosus and connective tissue disorders
210	M3215	Systemic lupus erythematosus and connective tissue disorders
210	M3219	Systemic lupus erythematosus and connective tissue disorders
210	M328	Systemic lupus erythematosus and connective tissue disorders
210	M329	Systemic lupus erythematosus and connective tissue disorders
210	M3300	Systemic lupus erythematosus and connective tissue disorders
210	M3301	Systemic lupus erythematosus and connective tissue disorders
210	M3302	Systemic lupus erythematosus and connective tissue disorders

210	M3309	Systemic lupus erythematosus and connective tissue disorders
210	M3310	Systemic lupus erythematosus and connective tissue disorders
210	M3311	Systemic lupus erythematosus and connective tissue disorders
210	M3312	Systemic lupus erythematosus and connective tissue disorders
210	M3319	Systemic lupus erythematosus and connective tissue disorders
210	M3320	Systemic lupus erythematosus and connective tissue disorders
210	M3321	Systemic lupus erythematosus and connective tissue disorders
210	M3322	Systemic lupus erythematosus and connective tissue disorders
210	M3329	Systemic lupus erythematosus and connective tissue disorders
210	M3390	Systemic lupus erythematosus and connective tissue disorders
210	M3391	Systemic lupus erythematosus and connective tissue disorders
210	M3392	Systemic lupus erythematosus and connective tissue disorders
210	M3399	Systemic lupus erythematosus and connective tissue disorders
210	M340	Systemic lupus erythematosus and connective tissue disorders
210	M341	Systemic lupus erythematosus and connective tissue disorders
210	M342	Systemic lupus erythematosus and connective tissue disorders
210	M3481	Systemic lupus erythematosus and connective tissue disorders
210	M3482	Systemic lupus erythematosus and connective tissue disorders
210	M3483	Systemic lupus erythematosus and connective tissue disorders
210	M3489	Systemic lupus erythematosus and connective tissue disorders
210	M349	Systemic lupus erythematosus and connective tissue disorders
210	M3500	Systemic lupus erythematosus and connective tissue disorders
210	M3501	Systemic lupus erythematosus and connective tissue disorders
210	M3502	Systemic lupus erythematosus and connective tissue disorders
210	M3503	Systemic lupus erythematosus and connective tissue disorders
210	M3504	Systemic lupus erythematosus and connective tissue disorders
210	M3509	Systemic lupus erythematosus and connective tissue disorders
210	M351	Systemic lupus erythematosus and connective tissue disorders
210	M355	Systemic lupus erythematosus and connective tissue disorders
210	M359	Systemic lupus erythematosus and connective tissue disorders
210	M360	Systemic lupus erythematosus and connective tissue disorders
210	M368	Systemic lupus erythematosus and connective tissue disorders
211	G4762	Other connective tissue disease
211	K6812	Other connective tissue disease
211	M2420	Other connective tissue disease
211	M24211	Other connective tissue disease
211	M24212	Other connective tissue disease
211	M24219	Other connective tissue disease
211	M24221	Other connective tissue disease
211	M24222	Other connective tissue disease
211	M24229	Other connective tissue disease
211	M24231	Other connective tissue disease
211	M24232	Other connective tissue disease
211	M24239	Other connective tissue disease
211	M24241	Other connective tissue disease
211	M24242	Other connective tissue disease
211	M24249	Other connective tissue disease
211	M24251	Other connective tissue disease
211	M24252	Other connective tissue disease
211	M24259	Other connective tissue disease
211	M24271	Other connective tissue disease

211	M24272	Other connective tissue disease
211	M24273	Other connective tissue disease
211	M24274	Other connective tissue disease
211	M24275	Other connective tissue disease
211	M24276	Other connective tissue disease
211	M2428	Other connective tissue disease
211	M352	Other connective tissue disease
211	M353	Other connective tissue disease
211	M354	Other connective tissue disease
211	M356	Other connective tissue disease
211	M357	Other connective tissue disease
211	M358	Other connective tissue disease
211	M60000	Other connective tissue disease
211	M60001	Other connective tissue disease
211	M60002	Other connective tissue disease
211	M60003	Other connective tissue disease
211	M60004	Other connective tissue disease
211	M60005	Other connective tissue disease
211	M60009	Other connective tissue disease
211	M60011	Other connective tissue disease
211	M60012	Other connective tissue disease
211	M60019	Other connective tissue disease
211	M60021	Other connective tissue disease
211	M60022	Other connective tissue disease
211	M60029	Other connective tissue disease
211	M60031	Other connective tissue disease
211	M60032	Other connective tissue disease
211	M60039	Other connective tissue disease
211	M60041	Other connective tissue disease
211	M60042	Other connective tissue disease
211	M60043	Other connective tissue disease
211	M60044	Other connective tissue disease
211	M60045	Other connective tissue disease
211	M60046	Other connective tissue disease
211	M60051	Other connective tissue disease
211	M60052	Other connective tissue disease
211	M60059	Other connective tissue disease
211	M60061	Other connective tissue disease
211	M60062	Other connective tissue disease
211	M60069	Other connective tissue disease
211	M60070	Other connective tissue disease
211	M60071	Other connective tissue disease
211	M60072	Other connective tissue disease
211	M60073	Other connective tissue disease
211	M60074	Other connective tissue disease
211	M60075	Other connective tissue disease
211	M60076	Other connective tissue disease
211	M60077	Other connective tissue disease
211	M60078	Other connective tissue disease
211	M6008	Other connective tissue disease
211	M6009	Other connective tissue disease

211	M6010	Other connective tissue disease
211	M60111	Other connective tissue disease
211	M60112	Other connective tissue disease
211	M60119	Other connective tissue disease
211	M60121	Other connective tissue disease
211	M60122	Other connective tissue disease
211	M60129	Other connective tissue disease
211	M60131	Other connective tissue disease
211	M60132	Other connective tissue disease
211	M60139	Other connective tissue disease
211	M60141	Other connective tissue disease
211	M60142	Other connective tissue disease
211	M60149	Other connective tissue disease
211	M60151	Other connective tissue disease
211	M60152	Other connective tissue disease
211	M60159	Other connective tissue disease
211	M60161	Other connective tissue disease
211	M60162	Other connective tissue disease
211	M60169	Other connective tissue disease
211	M60171	Other connective tissue disease
211	M60172	Other connective tissue disease
211	M60179	Other connective tissue disease
211	M6018	Other connective tissue disease
211	M6019	Other connective tissue disease
211	M6020	Other connective tissue disease
211	M60211	Other connective tissue disease
211	M60212	Other connective tissue disease
211	M60219	Other connective tissue disease
211	M60221	Other connective tissue disease
211	M60222	Other connective tissue disease
211	M60229	Other connective tissue disease
211	M60231	Other connective tissue disease
211	M60232	Other connective tissue disease
211	M60239	Other connective tissue disease
211	M60241	Other connective tissue disease
211	M60242	Other connective tissue disease
211	M60249	Other connective tissue disease
211	M60251	Other connective tissue disease
211	M60252	Other connective tissue disease
211	M60259	Other connective tissue disease
211	M60261	Other connective tissue disease
211	M60262	Other connective tissue disease
211	M60269	Other connective tissue disease
211	M60271	Other connective tissue disease
211	M60272	Other connective tissue disease
211	M60279	Other connective tissue disease
211	M6028	Other connective tissue disease
211	M6080	Other connective tissue disease
211	M60811	Other connective tissue disease
211	M60812	Other connective tissue disease
211	M60819	Other connective tissue disease

211	M60821	Other connective tissue disease
211	M60822	Other connective tissue disease
211	M60829	Other connective tissue disease
211	M60831	Other connective tissue disease
211	M60832	Other connective tissue disease
211	M60839	Other connective tissue disease
211	M60841	Other connective tissue disease
211	M60842	Other connective tissue disease
211	M60849	Other connective tissue disease
211	M60851	Other connective tissue disease
211	M60852	Other connective tissue disease
211	M60859	Other connective tissue disease
211	M60861	Other connective tissue disease
211	M60862	Other connective tissue disease
211	M60869	Other connective tissue disease
211	M60871	Other connective tissue disease
211	M60872	Other connective tissue disease
211	M60879	Other connective tissue disease
211	M6088	Other connective tissue disease
211	M6089	Other connective tissue disease
211	M609	Other connective tissue disease
211	M6100	Other connective tissue disease
211	M61011	Other connective tissue disease
211	M61012	Other connective tissue disease
211	M61019	Other connective tissue disease
211	M61021	Other connective tissue disease
211	M61022	Other connective tissue disease
211	M61029	Other connective tissue disease
211	M61031	Other connective tissue disease
211	M61032	Other connective tissue disease
211	M61039	Other connective tissue disease
211	M61041	Other connective tissue disease
211	M61042	Other connective tissue disease
211	M61049	Other connective tissue disease
211	M61051	Other connective tissue disease
211	M61052	Other connective tissue disease
211	M61059	Other connective tissue disease
211	M61061	Other connective tissue disease
211	M61062	Other connective tissue disease
211	M61069	Other connective tissue disease
211	M61071	Other connective tissue disease
211	M61072	Other connective tissue disease
211	M61079	Other connective tissue disease
211	M6108	Other connective tissue disease
211	M6109	Other connective tissue disease
211	M6110	Other connective tissue disease
211	M61111	Other connective tissue disease
211	M61112	Other connective tissue disease
211	M61119	Other connective tissue disease
211	M61121	Other connective tissue disease
211	M61122	Other connective tissue disease

211	M61129	Other connective tissue disease
211	M61131	Other connective tissue disease
211	M61132	Other connective tissue disease
211	M61139	Other connective tissue disease
211	M61141	Other connective tissue disease
211	M61142	Other connective tissue disease
211	M61143	Other connective tissue disease
211	M61144	Other connective tissue disease
211	M61145	Other connective tissue disease
211	M61146	Other connective tissue disease
211	M61151	Other connective tissue disease
211	M61152	Other connective tissue disease
211	M61159	Other connective tissue disease
211	M61161	Other connective tissue disease
211	M61162	Other connective tissue disease
211	M61169	Other connective tissue disease
211	M61171	Other connective tissue disease
211	M61172	Other connective tissue disease
211	M61173	Other connective tissue disease
211	M61174	Other connective tissue disease
211	M61175	Other connective tissue disease
211	M61176	Other connective tissue disease
211	M61177	Other connective tissue disease
211	M61178	Other connective tissue disease
211	M61179	Other connective tissue disease
211	M6118	Other connective tissue disease
211	M6119	Other connective tissue disease
211	M6120	Other connective tissue disease
211	M61211	Other connective tissue disease
211	M61212	Other connective tissue disease
211	M61219	Other connective tissue disease
211	M61221	Other connective tissue disease
211	M61222	Other connective tissue disease
211	M61229	Other connective tissue disease
211	M61231	Other connective tissue disease
211	M61232	Other connective tissue disease
211	M61239	Other connective tissue disease
211	M61241	Other connective tissue disease
211	M61242	Other connective tissue disease
211	M61249	Other connective tissue disease
211	M61251	Other connective tissue disease
211	M61252	Other connective tissue disease
211	M61259	Other connective tissue disease
211	M61261	Other connective tissue disease
211	M61262	Other connective tissue disease
211	M61269	Other connective tissue disease
211	M61271	Other connective tissue disease
211	M61272	Other connective tissue disease
211	M61279	Other connective tissue disease
211	M6128	Other connective tissue disease
211	M6129	Other connective tissue disease

211	M6130	Other connective tissue disease
211	M61311	Other connective tissue disease
211	M61312	Other connective tissue disease
211	M61319	Other connective tissue disease
211	M61321	Other connective tissue disease
211	M61322	Other connective tissue disease
211	M61329	Other connective tissue disease
211	M61331	Other connective tissue disease
211	M61332	Other connective tissue disease
211	M61339	Other connective tissue disease
211	M61341	Other connective tissue disease
211	M61342	Other connective tissue disease
211	M61349	Other connective tissue disease
211	M61351	Other connective tissue disease
211	M61352	Other connective tissue disease
211	M61359	Other connective tissue disease
211	M61361	Other connective tissue disease
211	M61362	Other connective tissue disease
211	M61369	Other connective tissue disease
211	M61371	Other connective tissue disease
211	M61372	Other connective tissue disease
211	M61379	Other connective tissue disease
211	M6138	Other connective tissue disease
211	M6139	Other connective tissue disease
211	M6140	Other connective tissue disease
211	M61411	Other connective tissue disease
211	M61412	Other connective tissue disease
211	M61419	Other connective tissue disease
211	M61421	Other connective tissue disease
211	M61422	Other connective tissue disease
211	M61429	Other connective tissue disease
211	M61431	Other connective tissue disease
211	M61432	Other connective tissue disease
211	M61439	Other connective tissue disease
211	M61441	Other connective tissue disease
211	M61442	Other connective tissue disease
211	M61449	Other connective tissue disease
211	M61451	Other connective tissue disease
211	M61452	Other connective tissue disease
211	M61459	Other connective tissue disease
211	M61461	Other connective tissue disease
211	M61462	Other connective tissue disease
211	M61469	Other connective tissue disease
211	M61471	Other connective tissue disease
211	M61472	Other connective tissue disease
211	M61479	Other connective tissue disease
211	M6148	Other connective tissue disease
211	M6149	Other connective tissue disease
211	M6150	Other connective tissue disease
211	M61511	Other connective tissue disease
211	M61512	Other connective tissue disease

211	M61519	Other connective tissue disease
211	M61521	Other connective tissue disease
211	M61522	Other connective tissue disease
211	M61529	Other connective tissue disease
211	M61531	Other connective tissue disease
211	M61532	Other connective tissue disease
211	M61539	Other connective tissue disease
211	M61541	Other connective tissue disease
211	M61542	Other connective tissue disease
211	M61549	Other connective tissue disease
211	M61551	Other connective tissue disease
211	M61552	Other connective tissue disease
211	M61559	Other connective tissue disease
211	M61561	Other connective tissue disease
211	M61562	Other connective tissue disease
211	M61569	Other connective tissue disease
211	M61571	Other connective tissue disease
211	M61572	Other connective tissue disease
211	M61579	Other connective tissue disease
211	M6158	Other connective tissue disease
211	M6159	Other connective tissue disease
211	M619	Other connective tissue disease
211	M6200	Other connective tissue disease
211	M62011	Other connective tissue disease
211	M62012	Other connective tissue disease
211	M62019	Other connective tissue disease
211	M62021	Other connective tissue disease
211	M62022	Other connective tissue disease
211	M62029	Other connective tissue disease
211	M62031	Other connective tissue disease
211	M62032	Other connective tissue disease
211	M62039	Other connective tissue disease
211	M62041	Other connective tissue disease
211	M62042	Other connective tissue disease
211	M62049	Other connective tissue disease
211	M62051	Other connective tissue disease
211	M62052	Other connective tissue disease
211	M62059	Other connective tissue disease
211	M62061	Other connective tissue disease
211	M62062	Other connective tissue disease
211	M62069	Other connective tissue disease
211	M62071	Other connective tissue disease
211	M62072	Other connective tissue disease
211	M62079	Other connective tissue disease
211	M6208	Other connective tissue disease
211	M6210	Other connective tissue disease
211	M62111	Other connective tissue disease
211	M62112	Other connective tissue disease
211	M62119	Other connective tissue disease
211	M62121	Other connective tissue disease
211	M62122	Other connective tissue disease

211	M62129	Other connective tissue disease
211	M62131	Other connective tissue disease
211	M62132	Other connective tissue disease
211	M62139	Other connective tissue disease
211	M62141	Other connective tissue disease
211	M62142	Other connective tissue disease
211	M62149	Other connective tissue disease
211	M62151	Other connective tissue disease
211	M62152	Other connective tissue disease
211	M62159	Other connective tissue disease
211	M62161	Other connective tissue disease
211	M62162	Other connective tissue disease
211	M62169	Other connective tissue disease
211	M62171	Other connective tissue disease
211	M62172	Other connective tissue disease
211	M62179	Other connective tissue disease
211	M6218	Other connective tissue disease
211	M6220	Other connective tissue disease
211	M62211	Other connective tissue disease
211	M62212	Other connective tissue disease
211	M62219	Other connective tissue disease
211	M62221	Other connective tissue disease
211	M62222	Other connective tissue disease
211	M62229	Other connective tissue disease
211	M62231	Other connective tissue disease
211	M62232	Other connective tissue disease
211	M62239	Other connective tissue disease
211	M62241	Other connective tissue disease
211	M62242	Other connective tissue disease
211	M62249	Other connective tissue disease
211	M62251	Other connective tissue disease
211	M62252	Other connective tissue disease
211	M62259	Other connective tissue disease
211	M62261	Other connective tissue disease
211	M62262	Other connective tissue disease
211	M62269	Other connective tissue disease
211	M62271	Other connective tissue disease
211	M62272	Other connective tissue disease
211	M62279	Other connective tissue disease
211	M6228	Other connective tissue disease
211	M623	Other connective tissue disease
211	M6240	Other connective tissue disease
211	M62411	Other connective tissue disease
211	M62412	Other connective tissue disease
211	M62419	Other connective tissue disease
211	M62421	Other connective tissue disease
211	M62422	Other connective tissue disease
211	M62429	Other connective tissue disease
211	M62431	Other connective tissue disease
211	M62432	Other connective tissue disease
211	M62439	Other connective tissue disease

211	M62441	Other connective tissue disease
211	M62442	Other connective tissue disease
211	M62449	Other connective tissue disease
211	M62451	Other connective tissue disease
211	M62452	Other connective tissue disease
211	M62459	Other connective tissue disease
211	M62461	Other connective tissue disease
211	M62462	Other connective tissue disease
211	M62469	Other connective tissue disease
211	M62471	Other connective tissue disease
211	M62472	Other connective tissue disease
211	M62479	Other connective tissue disease
211	M6248	Other connective tissue disease
211	M6249	Other connective tissue disease
211	M6250	Other connective tissue disease
211	M62511	Other connective tissue disease
211	M62512	Other connective tissue disease
211	M62519	Other connective tissue disease
211	M62521	Other connective tissue disease
211	M62522	Other connective tissue disease
211	M62529	Other connective tissue disease
211	M62531	Other connective tissue disease
211	M62532	Other connective tissue disease
211	M62539	Other connective tissue disease
211	M62541	Other connective tissue disease
211	M62542	Other connective tissue disease
211	M62549	Other connective tissue disease
211	M62551	Other connective tissue disease
211	M62552	Other connective tissue disease
211	M62559	Other connective tissue disease
211	M62561	Other connective tissue disease
211	M62562	Other connective tissue disease
211	M62569	Other connective tissue disease
211	M62571	Other connective tissue disease
211	M62572	Other connective tissue disease
211	M62579	Other connective tissue disease
211	M6258	Other connective tissue disease
211	M6259	Other connective tissue disease
211	M6281	Other connective tissue disease
211	M6282	Other connective tissue disease
211	M62831	Other connective tissue disease
211	M62838	Other connective tissue disease
211	M6289	Other connective tissue disease
211	M629	Other connective tissue disease
211	M6380	Other connective tissue disease
211	M63811	Other connective tissue disease
211	M63812	Other connective tissue disease
211	M63819	Other connective tissue disease
211	M63821	Other connective tissue disease
211	M63822	Other connective tissue disease
211	M63829	Other connective tissue disease

211	M63831	Other connective tissue disease
211	M63832	Other connective tissue disease
211	M63839	Other connective tissue disease
211	M63841	Other connective tissue disease
211	M63842	Other connective tissue disease
211	M63849	Other connective tissue disease
211	M63851	Other connective tissue disease
211	M63852	Other connective tissue disease
211	M63859	Other connective tissue disease
211	M63861	Other connective tissue disease
211	M63862	Other connective tissue disease
211	M63869	Other connective tissue disease
211	M63871	Other connective tissue disease
211	M63872	Other connective tissue disease
211	M63879	Other connective tissue disease
211	M6388	Other connective tissue disease
211	M6389	Other connective tissue disease
211	M6500	Other connective tissue disease
211	M65011	Other connective tissue disease
211	M65012	Other connective tissue disease
211	M65019	Other connective tissue disease
211	M65021	Other connective tissue disease
211	M65022	Other connective tissue disease
211	M65029	Other connective tissue disease
211	M65031	Other connective tissue disease
211	M65032	Other connective tissue disease
211	M65039	Other connective tissue disease
211	M65041	Other connective tissue disease
211	M65042	Other connective tissue disease
211	M65049	Other connective tissue disease
211	M65051	Other connective tissue disease
211	M65052	Other connective tissue disease
211	M65059	Other connective tissue disease
211	M65061	Other connective tissue disease
211	M65062	Other connective tissue disease
211	M65069	Other connective tissue disease
211	M65071	Other connective tissue disease
211	M65072	Other connective tissue disease
211	M65079	Other connective tissue disease
211	M6508	Other connective tissue disease
211	M6510	Other connective tissue disease
211	M65111	Other connective tissue disease
211	M65112	Other connective tissue disease
211	M65119	Other connective tissue disease
211	M65121	Other connective tissue disease
211	M65122	Other connective tissue disease
211	M65129	Other connective tissue disease
211	M65131	Other connective tissue disease
211	M65132	Other connective tissue disease
211	M65139	Other connective tissue disease
211	M65141	Other connective tissue disease

211	M65142	Other connective tissue disease
211	M65149	Other connective tissue disease
211	M65151	Other connective tissue disease
211	M65152	Other connective tissue disease
211	M65159	Other connective tissue disease
211	M65161	Other connective tissue disease
211	M65162	Other connective tissue disease
211	M65169	Other connective tissue disease
211	M65171	Other connective tissue disease
211	M65172	Other connective tissue disease
211	M65179	Other connective tissue disease
211	M6518	Other connective tissue disease
211	M6519	Other connective tissue disease
211	M6520	Other connective tissue disease
211	M65221	Other connective tissue disease
211	M65222	Other connective tissue disease
211	M65229	Other connective tissue disease
211	M65231	Other connective tissue disease
211	M65232	Other connective tissue disease
211	M65239	Other connective tissue disease
211	M65241	Other connective tissue disease
211	M65242	Other connective tissue disease
211	M65249	Other connective tissue disease
211	M65251	Other connective tissue disease
211	M65252	Other connective tissue disease
211	M65259	Other connective tissue disease
211	M65261	Other connective tissue disease
211	M65262	Other connective tissue disease
211	M65269	Other connective tissue disease
211	M65271	Other connective tissue disease
211	M65272	Other connective tissue disease
211	M65279	Other connective tissue disease
211	M6528	Other connective tissue disease
211	M6529	Other connective tissue disease
211	M6530	Other connective tissue disease
211	M65311	Other connective tissue disease
211	M65312	Other connective tissue disease
211	M65319	Other connective tissue disease
211	M65321	Other connective tissue disease
211	M65322	Other connective tissue disease
211	M65329	Other connective tissue disease
211	M65331	Other connective tissue disease
211	M65332	Other connective tissue disease
211	M65339	Other connective tissue disease
211	M65341	Other connective tissue disease
211	M65342	Other connective tissue disease
211	M65349	Other connective tissue disease
211	M65351	Other connective tissue disease
211	M65352	Other connective tissue disease
211	M65359	Other connective tissue disease
211	M654	Other connective tissue disease

211	M6580	Other connective tissue disease
211	M65811	Other connective tissue disease
211	M65812	Other connective tissue disease
211	M65819	Other connective tissue disease
211	M65821	Other connective tissue disease
211	M65822	Other connective tissue disease
211	M65829	Other connective tissue disease
211	M65831	Other connective tissue disease
211	M65832	Other connective tissue disease
211	M65839	Other connective tissue disease
211	M65841	Other connective tissue disease
211	M65842	Other connective tissue disease
211	M65849	Other connective tissue disease
211	M65851	Other connective tissue disease
211	M65852	Other connective tissue disease
211	M65859	Other connective tissue disease
211	M65861	Other connective tissue disease
211	M65862	Other connective tissue disease
211	M65869	Other connective tissue disease
211	M65871	Other connective tissue disease
211	M65872	Other connective tissue disease
211	M65879	Other connective tissue disease
211	M6588	Other connective tissue disease
211	M6589	Other connective tissue disease
211	M659	Other connective tissue disease
211	M660	Other connective tissue disease
211	M6610	Other connective tissue disease
211	M66111	Other connective tissue disease
211	M66112	Other connective tissue disease
211	M66119	Other connective tissue disease
211	M66121	Other connective tissue disease
211	M66122	Other connective tissue disease
211	M66129	Other connective tissue disease
211	M66131	Other connective tissue disease
211	M66132	Other connective tissue disease
211	M66139	Other connective tissue disease
211	M66141	Other connective tissue disease
211	M66142	Other connective tissue disease
211	M66143	Other connective tissue disease
211	M66144	Other connective tissue disease
211	M66145	Other connective tissue disease
211	M66146	Other connective tissue disease
211	M66151	Other connective tissue disease
211	M66152	Other connective tissue disease
211	M66159	Other connective tissue disease
211	M66171	Other connective tissue disease
211	M66172	Other connective tissue disease
211	M66173	Other connective tissue disease
211	M66174	Other connective tissue disease
211	M66175	Other connective tissue disease
211	M66176	Other connective tissue disease

211	M66177	Other connective tissue disease
211	M66178	Other connective tissue disease
211	M66179	Other connective tissue disease
211	M6618	Other connective tissue disease
211	M6620	Other connective tissue disease
211	M66211	Other connective tissue disease
211	M66212	Other connective tissue disease
211	M66219	Other connective tissue disease
211	M66221	Other connective tissue disease
211	M66222	Other connective tissue disease
211	M66229	Other connective tissue disease
211	M66231	Other connective tissue disease
211	M66232	Other connective tissue disease
211	M66239	Other connective tissue disease
211	M66241	Other connective tissue disease
211	M66242	Other connective tissue disease
211	M66249	Other connective tissue disease
211	M66251	Other connective tissue disease
211	M66252	Other connective tissue disease
211	M66259	Other connective tissue disease
211	M66261	Other connective tissue disease
211	M66262	Other connective tissue disease
211	M66269	Other connective tissue disease
211	M66271	Other connective tissue disease
211	M66272	Other connective tissue disease
211	M66279	Other connective tissue disease
211	M6628	Other connective tissue disease
211	M6629	Other connective tissue disease
211	M6630	Other connective tissue disease
211	M66311	Other connective tissue disease
211	M66312	Other connective tissue disease
211	M66319	Other connective tissue disease
211	M66321	Other connective tissue disease
211	M66322	Other connective tissue disease
211	M66329	Other connective tissue disease
211	M66331	Other connective tissue disease
211	M66332	Other connective tissue disease
211	M66339	Other connective tissue disease
211	M66341	Other connective tissue disease
211	M66342	Other connective tissue disease
211	M66349	Other connective tissue disease
211	M66351	Other connective tissue disease
211	M66352	Other connective tissue disease
211	M66359	Other connective tissue disease
211	M66361	Other connective tissue disease
211	M66362	Other connective tissue disease
211	M66369	Other connective tissue disease
211	M66371	Other connective tissue disease
211	M66372	Other connective tissue disease
211	M66379	Other connective tissue disease
211	M6638	Other connective tissue disease

211	M6639	Other connective tissue disease
211	M6680	Other connective tissue disease
211	M66811	Other connective tissue disease
211	M66812	Other connective tissue disease
211	M66819	Other connective tissue disease
211	M66821	Other connective tissue disease
211	M66822	Other connective tissue disease
211	M66829	Other connective tissue disease
211	M66831	Other connective tissue disease
211	M66832	Other connective tissue disease
211	M66839	Other connective tissue disease
211	M66841	Other connective tissue disease
211	M66842	Other connective tissue disease
211	M66849	Other connective tissue disease
211	M66851	Other connective tissue disease
211	M66852	Other connective tissue disease
211	M66859	Other connective tissue disease
211	M66861	Other connective tissue disease
211	M66862	Other connective tissue disease
211	M66869	Other connective tissue disease
211	M66871	Other connective tissue disease
211	M66872	Other connective tissue disease
211	M66879	Other connective tissue disease
211	M6688	Other connective tissue disease
211	M6689	Other connective tissue disease
211	M669	Other connective tissue disease
211	M6700	Other connective tissue disease
211	M6701	Other connective tissue disease
211	M6702	Other connective tissue disease
211	M6720	Other connective tissue disease
211	M67211	Other connective tissue disease
211	M67212	Other connective tissue disease
211	M67219	Other connective tissue disease
211	M67221	Other connective tissue disease
211	M67222	Other connective tissue disease
211	M67229	Other connective tissue disease
211	M67231	Other connective tissue disease
211	M67232	Other connective tissue disease
211	M67239	Other connective tissue disease
211	M67241	Other connective tissue disease
211	M67242	Other connective tissue disease
211	M67249	Other connective tissue disease
211	M67251	Other connective tissue disease
211	M67252	Other connective tissue disease
211	M67259	Other connective tissue disease
211	M67261	Other connective tissue disease
211	M67262	Other connective tissue disease
211	M67269	Other connective tissue disease
211	M67271	Other connective tissue disease
211	M67272	Other connective tissue disease
211	M67279	Other connective tissue disease

211	M6728	Other connective tissue disease
211	M6729	Other connective tissue disease
211	M6730	Other connective tissue disease
211	M67311	Other connective tissue disease
211	M67312	Other connective tissue disease
211	M67319	Other connective tissue disease
211	M67321	Other connective tissue disease
211	M67322	Other connective tissue disease
211	M67329	Other connective tissue disease
211	M67331	Other connective tissue disease
211	M67332	Other connective tissue disease
211	M67339	Other connective tissue disease
211	M67341	Other connective tissue disease
211	M67342	Other connective tissue disease
211	M67349	Other connective tissue disease
211	M67351	Other connective tissue disease
211	M67352	Other connective tissue disease
211	M67359	Other connective tissue disease
211	M67361	Other connective tissue disease
211	M67362	Other connective tissue disease
211	M67369	Other connective tissue disease
211	M67371	Other connective tissue disease
211	M67372	Other connective tissue disease
211	M67379	Other connective tissue disease
211	M6738	Other connective tissue disease
211	M6739	Other connective tissue disease
211	M6740	Other connective tissue disease
211	M67411	Other connective tissue disease
211	M67412	Other connective tissue disease
211	M67419	Other connective tissue disease
211	M67421	Other connective tissue disease
211	M67422	Other connective tissue disease
211	M67429	Other connective tissue disease
211	M67431	Other connective tissue disease
211	M67432	Other connective tissue disease
211	M67439	Other connective tissue disease
211	M67441	Other connective tissue disease
211	M67442	Other connective tissue disease
211	M67449	Other connective tissue disease
211	M67451	Other connective tissue disease
211	M67452	Other connective tissue disease
211	M67459	Other connective tissue disease
211	M67461	Other connective tissue disease
211	M67462	Other connective tissue disease
211	M67469	Other connective tissue disease
211	M67471	Other connective tissue disease
211	M67472	Other connective tissue disease
211	M67479	Other connective tissue disease
211	M6748	Other connective tissue disease
211	M6749	Other connective tissue disease
211	M6750	Other connective tissue disease

211	M6751	Other connective tissue disease
211	M6752	Other connective tissue disease
211	M6780	Other connective tissue disease
211	M67811	Other connective tissue disease
211	M67812	Other connective tissue disease
211	M67813	Other connective tissue disease
211	M67814	Other connective tissue disease
211	M67819	Other connective tissue disease
211	M67821	Other connective tissue disease
211	M67822	Other connective tissue disease
211	M67823	Other connective tissue disease
211	M67824	Other connective tissue disease
211	M67829	Other connective tissue disease
211	M67831	Other connective tissue disease
211	M67832	Other connective tissue disease
211	M67833	Other connective tissue disease
211	M67834	Other connective tissue disease
211	M67839	Other connective tissue disease
211	M67841	Other connective tissue disease
211	M67842	Other connective tissue disease
211	M67843	Other connective tissue disease
211	M67844	Other connective tissue disease
211	M67849	Other connective tissue disease
211	M67851	Other connective tissue disease
211	M67852	Other connective tissue disease
211	M67853	Other connective tissue disease
211	M67854	Other connective tissue disease
211	M67859	Other connective tissue disease
211	M67861	Other connective tissue disease
211	M67862	Other connective tissue disease
211	M67863	Other connective tissue disease
211	M67864	Other connective tissue disease
211	M67869	Other connective tissue disease
211	M67871	Other connective tissue disease
211	M67872	Other connective tissue disease
211	M67873	Other connective tissue disease
211	M67874	Other connective tissue disease
211	M67879	Other connective tissue disease
211	M6788	Other connective tissue disease
211	M6789	Other connective tissue disease
211	M6790	Other connective tissue disease
211	M67911	Other connective tissue disease
211	M67912	Other connective tissue disease
211	M67919	Other connective tissue disease
211	M67921	Other connective tissue disease
211	M67922	Other connective tissue disease
211	M67929	Other connective tissue disease
211	M67931	Other connective tissue disease
211	M67932	Other connective tissue disease
211	M67939	Other connective tissue disease
211	M67941	Other connective tissue disease

211	M67942	Other connective tissue disease
211	M67949	Other connective tissue disease
211	M67951	Other connective tissue disease
211	M67952	Other connective tissue disease
211	M67959	Other connective tissue disease
211	M67961	Other connective tissue disease
211	M67962	Other connective tissue disease
211	M67969	Other connective tissue disease
211	M67971	Other connective tissue disease
211	M67972	Other connective tissue disease
211	M67979	Other connective tissue disease
211	M6798	Other connective tissue disease
211	M6799	Other connective tissue disease
211	M70031	Other connective tissue disease
211	M70032	Other connective tissue disease
211	M70039	Other connective tissue disease
211	M70041	Other connective tissue disease
211	M70042	Other connective tissue disease
211	M70049	Other connective tissue disease
211	M7010	Other connective tissue disease
211	M7011	Other connective tissue disease
211	M7012	Other connective tissue disease
211	M7020	Other connective tissue disease
211	M7021	Other connective tissue disease
211	M7022	Other connective tissue disease
211	M7030	Other connective tissue disease
211	M7031	Other connective tissue disease
211	M7032	Other connective tissue disease
211	M7040	Other connective tissue disease
211	M7041	Other connective tissue disease
211	M7042	Other connective tissue disease
211	M7050	Other connective tissue disease
211	M7051	Other connective tissue disease
211	M7052	Other connective tissue disease
211	M7060	Other connective tissue disease
211	M7061	Other connective tissue disease
211	M7062	Other connective tissue disease
211	M7070	Other connective tissue disease
211	M7071	Other connective tissue disease
211	M7072	Other connective tissue disease
211	M7080	Other connective tissue disease
211	M70811	Other connective tissue disease
211	M70812	Other connective tissue disease
211	M70819	Other connective tissue disease
211	M70821	Other connective tissue disease
211	M70822	Other connective tissue disease
211	M70829	Other connective tissue disease
211	M70831	Other connective tissue disease
211	M70832	Other connective tissue disease
211	M70839	Other connective tissue disease
211	M70841	Other connective tissue disease

211	M70842	Other connective tissue disease
211	M70849	Other connective tissue disease
211	M70851	Other connective tissue disease
211	M70852	Other connective tissue disease
211	M70859	Other connective tissue disease
211	M70861	Other connective tissue disease
211	M70862	Other connective tissue disease
211	M70869	Other connective tissue disease
211	M70871	Other connective tissue disease
211	M70872	Other connective tissue disease
211	M70879	Other connective tissue disease
211	M7088	Other connective tissue disease
211	M7089	Other connective tissue disease
211	M7090	Other connective tissue disease
211	M70911	Other connective tissue disease
211	M70912	Other connective tissue disease
211	M70919	Other connective tissue disease
211	M70921	Other connective tissue disease
211	M70922	Other connective tissue disease
211	M70929	Other connective tissue disease
211	M70931	Other connective tissue disease
211	M70932	Other connective tissue disease
211	M70939	Other connective tissue disease
211	M70941	Other connective tissue disease
211	M70942	Other connective tissue disease
211	M70949	Other connective tissue disease
211	M70951	Other connective tissue disease
211	M70952	Other connective tissue disease
211	M70959	Other connective tissue disease
211	M70961	Other connective tissue disease
211	M70962	Other connective tissue disease
211	M70969	Other connective tissue disease
211	M70971	Other connective tissue disease
211	M70972	Other connective tissue disease
211	M70979	Other connective tissue disease
211	M7098	Other connective tissue disease
211	M7099	Other connective tissue disease
211	M7100	Other connective tissue disease
211	M71011	Other connective tissue disease
211	M71012	Other connective tissue disease
211	M71019	Other connective tissue disease
211	M71021	Other connective tissue disease
211	M71022	Other connective tissue disease
211	M71029	Other connective tissue disease
211	M71031	Other connective tissue disease
211	M71032	Other connective tissue disease
211	M71039	Other connective tissue disease
211	M71041	Other connective tissue disease
211	M71042	Other connective tissue disease
211	M71049	Other connective tissue disease
211	M71051	Other connective tissue disease

211	M71052	Other connective tissue disease
211	M71059	Other connective tissue disease
211	M71061	Other connective tissue disease
211	M71062	Other connective tissue disease
211	M71069	Other connective tissue disease
211	M71071	Other connective tissue disease
211	M71072	Other connective tissue disease
211	M71079	Other connective tissue disease
211	M7108	Other connective tissue disease
211	M7109	Other connective tissue disease
211	M7110	Other connective tissue disease
211	M71111	Other connective tissue disease
211	M71112	Other connective tissue disease
211	M71119	Other connective tissue disease
211	M71121	Other connective tissue disease
211	M71122	Other connective tissue disease
211	M71129	Other connective tissue disease
211	M71131	Other connective tissue disease
211	M71132	Other connective tissue disease
211	M71139	Other connective tissue disease
211	M71141	Other connective tissue disease
211	M71142	Other connective tissue disease
211	M71149	Other connective tissue disease
211	M71151	Other connective tissue disease
211	M71152	Other connective tissue disease
211	M71159	Other connective tissue disease
211	M71161	Other connective tissue disease
211	M71162	Other connective tissue disease
211	M71169	Other connective tissue disease
211	M71171	Other connective tissue disease
211	M71172	Other connective tissue disease
211	M71179	Other connective tissue disease
211	M7118	Other connective tissue disease
211	M7119	Other connective tissue disease
211	M7120	Other connective tissue disease
211	M7121	Other connective tissue disease
211	M7122	Other connective tissue disease
211	M7130	Other connective tissue disease
211	M71311	Other connective tissue disease
211	M71312	Other connective tissue disease
211	M71319	Other connective tissue disease
211	M71321	Other connective tissue disease
211	M71322	Other connective tissue disease
211	M71329	Other connective tissue disease
211	M71331	Other connective tissue disease
211	M71332	Other connective tissue disease
211	M71339	Other connective tissue disease
211	M71341	Other connective tissue disease
211	M71342	Other connective tissue disease
211	M71349	Other connective tissue disease
211	M71351	Other connective tissue disease

211	M71352	Other connective tissue disease
211	M71359	Other connective tissue disease
211	M71371	Other connective tissue disease
211	M71372	Other connective tissue disease
211	M71379	Other connective tissue disease
211	M7138	Other connective tissue disease
211	M7139	Other connective tissue disease
211	M7140	Other connective tissue disease
211	M71421	Other connective tissue disease
211	M71422	Other connective tissue disease
211	M71429	Other connective tissue disease
211	M71431	Other connective tissue disease
211	M71432	Other connective tissue disease
211	M71439	Other connective tissue disease
211	M71441	Other connective tissue disease
211	M71442	Other connective tissue disease
211	M71449	Other connective tissue disease
211	M71451	Other connective tissue disease
211	M71452	Other connective tissue disease
211	M71459	Other connective tissue disease
211	M71461	Other connective tissue disease
211	M71462	Other connective tissue disease
211	M71469	Other connective tissue disease
211	M71471	Other connective tissue disease
211	M71472	Other connective tissue disease
211	M71479	Other connective tissue disease
211	M7148	Other connective tissue disease
211	M7149	Other connective tissue disease
211	M7150	Other connective tissue disease
211	M71521	Other connective tissue disease
211	M71522	Other connective tissue disease
211	M71529	Other connective tissue disease
211	M71531	Other connective tissue disease
211	M71532	Other connective tissue disease
211	M71539	Other connective tissue disease
211	M71541	Other connective tissue disease
211	M71542	Other connective tissue disease
211	M71549	Other connective tissue disease
211	M71551	Other connective tissue disease
211	M71552	Other connective tissue disease
211	M71559	Other connective tissue disease
211	M71561	Other connective tissue disease
211	M71562	Other connective tissue disease
211	M71569	Other connective tissue disease
211	M71571	Other connective tissue disease
211	M71572	Other connective tissue disease
211	M71579	Other connective tissue disease
211	M7158	Other connective tissue disease
211	M7180	Other connective tissue disease
211	M71811	Other connective tissue disease
211	M71812	Other connective tissue disease

211	M71819	Other connective tissue disease
211	M71821	Other connective tissue disease
211	M71822	Other connective tissue disease
211	M71829	Other connective tissue disease
211	M71831	Other connective tissue disease
211	M71832	Other connective tissue disease
211	M71839	Other connective tissue disease
211	M71841	Other connective tissue disease
211	M71842	Other connective tissue disease
211	M71849	Other connective tissue disease
211	M71851	Other connective tissue disease
211	M71852	Other connective tissue disease
211	M71859	Other connective tissue disease
211	M71861	Other connective tissue disease
211	M71862	Other connective tissue disease
211	M71869	Other connective tissue disease
211	M71871	Other connective tissue disease
211	M71872	Other connective tissue disease
211	M71879	Other connective tissue disease
211	M7188	Other connective tissue disease
211	M7189	Other connective tissue disease
211	M719	Other connective tissue disease
211	M720	Other connective tissue disease
211	M721	Other connective tissue disease
211	M722	Other connective tissue disease
211	M724	Other connective tissue disease
211	M726	Other connective tissue disease
211	M728	Other connective tissue disease
211	M729	Other connective tissue disease
211	M7500	Other connective tissue disease
211	M7501	Other connective tissue disease
211	M7502	Other connective tissue disease
211	M75100	Other connective tissue disease
211	M75101	Other connective tissue disease
211	M75102	Other connective tissue disease
211	M75110	Other connective tissue disease
211	M75111	Other connective tissue disease
211	M75112	Other connective tissue disease
211	M75120	Other connective tissue disease
211	M75121	Other connective tissue disease
211	M75122	Other connective tissue disease
211	M7520	Other connective tissue disease
211	M7521	Other connective tissue disease
211	M7522	Other connective tissue disease
211	M7530	Other connective tissue disease
211	M7531	Other connective tissue disease
211	M7532	Other connective tissue disease
211	M7540	Other connective tissue disease
211	M7541	Other connective tissue disease
211	M7542	Other connective tissue disease
211	M7550	Other connective tissue disease

211	M7551	Other connective tissue disease
211	M7552	Other connective tissue disease
211	M7580	Other connective tissue disease
211	M7581	Other connective tissue disease
211	M7582	Other connective tissue disease
211	M7590	Other connective tissue disease
211	M7591	Other connective tissue disease
211	M7592	Other connective tissue disease
211	M7600	Other connective tissue disease
211	M7601	Other connective tissue disease
211	M7602	Other connective tissue disease
211	M7610	Other connective tissue disease
211	M7611	Other connective tissue disease
211	M7612	Other connective tissue disease
211	M7620	Other connective tissue disease
211	M7621	Other connective tissue disease
211	M7622	Other connective tissue disease
211	M7630	Other connective tissue disease
211	M7631	Other connective tissue disease
211	M7632	Other connective tissue disease
211	M7640	Other connective tissue disease
211	M7641	Other connective tissue disease
211	M7642	Other connective tissue disease
211	M7650	Other connective tissue disease
211	M7651	Other connective tissue disease
211	M7652	Other connective tissue disease
211	M7660	Other connective tissue disease
211	M7661	Other connective tissue disease
211	M7662	Other connective tissue disease
211	M7670	Other connective tissue disease
211	M7671	Other connective tissue disease
211	M7672	Other connective tissue disease
211	M76811	Other connective tissue disease
211	M76812	Other connective tissue disease
211	M76819	Other connective tissue disease
211	M76821	Other connective tissue disease
211	M76822	Other connective tissue disease
211	M76829	Other connective tissue disease
211	M76891	Other connective tissue disease
211	M76892	Other connective tissue disease
211	M76899	Other connective tissue disease
211	M769	Other connective tissue disease
211	M7700	Other connective tissue disease
211	M7701	Other connective tissue disease
211	M7702	Other connective tissue disease
211	M7710	Other connective tissue disease
211	M7711	Other connective tissue disease
211	M7712	Other connective tissue disease
211	M7720	Other connective tissue disease
211	M7721	Other connective tissue disease
211	M7722	Other connective tissue disease

211	M7730	Other connective tissue disease
211	M7731	Other connective tissue disease
211	M7732	Other connective tissue disease
211	M7740	Other connective tissue disease
211	M7741	Other connective tissue disease
211	M7742	Other connective tissue disease
211	M7750	Other connective tissue disease
211	M7751	Other connective tissue disease
211	M7752	Other connective tissue disease
211	M778	Other connective tissue disease
211	M779	Other connective tissue disease
211	M790	Other connective tissue disease
211	M791	Other connective tissue disease
211	M792	Other connective tissue disease
211	M793	Other connective tissue disease
211	M794	Other connective tissue disease
211	M795	Other connective tissue disease
211	M79601	Other connective tissue disease
211	M79602	Other connective tissue disease
211	M79603	Other connective tissue disease
211	M79604	Other connective tissue disease
211	M79605	Other connective tissue disease
211	M79606	Other connective tissue disease
211	M79609	Other connective tissue disease
211	M79621	Other connective tissue disease
211	M79622	Other connective tissue disease
211	M79629	Other connective tissue disease
211	M79631	Other connective tissue disease
211	M79632	Other connective tissue disease
211	M79639	Other connective tissue disease
211	M79641	Other connective tissue disease
211	M79642	Other connective tissue disease
211	M79643	Other connective tissue disease
211	M79644	Other connective tissue disease
211	M79645	Other connective tissue disease
211	M79646	Other connective tissue disease
211	M79651	Other connective tissue disease
211	M79652	Other connective tissue disease
211	M79659	Other connective tissue disease
211	M79661	Other connective tissue disease
211	M79662	Other connective tissue disease
211	M79669	Other connective tissue disease
211	M79671	Other connective tissue disease
211	M79672	Other connective tissue disease
211	M79673	Other connective tissue disease
211	M79674	Other connective tissue disease
211	M79675	Other connective tissue disease
211	M79676	Other connective tissue disease
211	M797	Other connective tissue disease
211	M7981	Other connective tissue disease
211	M7989	Other connective tissue disease

211	M799	Other connective tissue disease
211	M79A11	Other connective tissue disease
211	M79A12	Other connective tissue disease
211	M79A19	Other connective tissue disease
211	M79A21	Other connective tissue disease
211	M79A22	Other connective tissue disease
211	M79A29	Other connective tissue disease
211	M79A3	Other connective tissue disease
211	M79A9	Other connective tissue disease
211	R252	Other connective tissue disease
211	R293	Other connective tissue disease
211	R296	Other connective tissue disease
211	R29810	Other connective tissue disease
211	R29818	Other connective tissue disease
211	R29890	Other connective tissue disease
211	R29898	Other connective tissue disease
211	R2990	Other connective tissue disease
211	R2991	Other connective tissue disease
211	Z8739	Other connective tissue disease
211	Z9660	Other connective tissue disease
211	Z96611	Other connective tissue disease
211	Z96612	Other connective tissue disease
211	Z96619	Other connective tissue disease
211	Z96621	Other connective tissue disease
211	Z96622	Other connective tissue disease
211	Z96629	Other connective tissue disease
211	Z96631	Other connective tissue disease
211	Z96632	Other connective tissue disease
211	Z96639	Other connective tissue disease
211	Z96641	Other connective tissue disease
211	Z96642	Other connective tissue disease
211	Z96643	Other connective tissue disease
211	Z96649	Other connective tissue disease
211	Z96651	Other connective tissue disease
211	Z96652	Other connective tissue disease
211	Z96653	Other connective tissue disease
211	Z96659	Other connective tissue disease
211	Z96661	Other connective tissue disease
211	Z96662	Other connective tissue disease
211	Z96669	Other connective tissue disease
211	Z96691	Other connective tissue disease
211	Z96692	Other connective tissue disease
211	Z96693	Other connective tissue disease
211	Z96698	Other connective tissue disease
211	Z967	Other connective tissue disease
211	Z9710	Other connective tissue disease
211	Z9711	Other connective tissue disease
211	Z9712	Other connective tissue disease
211	Z9713	Other connective tissue disease
211	Z9714	Other connective tissue disease
211	Z9715	Other connective tissue disease

211	Z9716	Other connective tissue disease
211	Z981	Other connective tissue disease
212	M4100	Other bone disease and musculoskeletal deformities
212	M4102	Other bone disease and musculoskeletal deformities
212	M4103	Other bone disease and musculoskeletal deformities
212	M4104	Other bone disease and musculoskeletal deformities
212	M4105	Other bone disease and musculoskeletal deformities
212	M4106	Other bone disease and musculoskeletal deformities
212	M4107	Other bone disease and musculoskeletal deformities
212	M4108	Other bone disease and musculoskeletal deformities
212	M41112	Other bone disease and musculoskeletal deformities
212	M41113	Other bone disease and musculoskeletal deformities
212	M41114	Other bone disease and musculoskeletal deformities
212	M41115	Other bone disease and musculoskeletal deformities
212	M41116	Other bone disease and musculoskeletal deformities
212	M41117	Other bone disease and musculoskeletal deformities
212	M41119	Other bone disease and musculoskeletal deformities
212	M41122	Other bone disease and musculoskeletal deformities
212	M41123	Other bone disease and musculoskeletal deformities
212	M41124	Other bone disease and musculoskeletal deformities
212	M41125	Other bone disease and musculoskeletal deformities
212	M41126	Other bone disease and musculoskeletal deformities
212	M41127	Other bone disease and musculoskeletal deformities
212	M41129	Other bone disease and musculoskeletal deformities
212	M4120	Other bone disease and musculoskeletal deformities
212	M4122	Other bone disease and musculoskeletal deformities
212	M4123	Other bone disease and musculoskeletal deformities
212	M4124	Other bone disease and musculoskeletal deformities
212	M4125	Other bone disease and musculoskeletal deformities
212	M4126	Other bone disease and musculoskeletal deformities
212	M4127	Other bone disease and musculoskeletal deformities
212	M4200	Other bone disease and musculoskeletal deformities
212	M4201	Other bone disease and musculoskeletal deformities
212	M4202	Other bone disease and musculoskeletal deformities
212	M4203	Other bone disease and musculoskeletal deformities
212	M4204	Other bone disease and musculoskeletal deformities
212	M4205	Other bone disease and musculoskeletal deformities
212	M4206	Other bone disease and musculoskeletal deformities
212	M4207	Other bone disease and musculoskeletal deformities
212	M4208	Other bone disease and musculoskeletal deformities
212	M4209	Other bone disease and musculoskeletal deformities
212	M4210	Other bone disease and musculoskeletal deformities
212	M4211	Other bone disease and musculoskeletal deformities
212	M4212	Other bone disease and musculoskeletal deformities
212	M4213	Other bone disease and musculoskeletal deformities
212	M4214	Other bone disease and musculoskeletal deformities
212	M4215	Other bone disease and musculoskeletal deformities
212	M4216	Other bone disease and musculoskeletal deformities
212	M4217	Other bone disease and musculoskeletal deformities
212	M4218	Other bone disease and musculoskeletal deformities
212	M4219	Other bone disease and musculoskeletal deformities

212	M429	Other bone disease and musculoskeletal deformities
212	M8480	Other bone disease and musculoskeletal deformities
212	M84811	Other bone disease and musculoskeletal deformities
212	M84812	Other bone disease and musculoskeletal deformities
212	M84819	Other bone disease and musculoskeletal deformities
212	M84821	Other bone disease and musculoskeletal deformities
212	M84822	Other bone disease and musculoskeletal deformities
212	M84829	Other bone disease and musculoskeletal deformities
212	M84831	Other bone disease and musculoskeletal deformities
212	M84832	Other bone disease and musculoskeletal deformities
212	M84833	Other bone disease and musculoskeletal deformities
212	M84834	Other bone disease and musculoskeletal deformities
212	M84839	Other bone disease and musculoskeletal deformities
212	M84841	Other bone disease and musculoskeletal deformities
212	M84842	Other bone disease and musculoskeletal deformities
212	M84849	Other bone disease and musculoskeletal deformities
212	M84851	Other bone disease and musculoskeletal deformities
212	M84852	Other bone disease and musculoskeletal deformities
212	M84859	Other bone disease and musculoskeletal deformities
212	M84861	Other bone disease and musculoskeletal deformities
212	M84862	Other bone disease and musculoskeletal deformities
212	M84863	Other bone disease and musculoskeletal deformities
212	M84864	Other bone disease and musculoskeletal deformities
212	M84869	Other bone disease and musculoskeletal deformities
212	M84871	Other bone disease and musculoskeletal deformities
212	M84872	Other bone disease and musculoskeletal deformities
212	M84879	Other bone disease and musculoskeletal deformities
212	M8488	Other bone disease and musculoskeletal deformities
212	M849	Other bone disease and musculoskeletal deformities
212	M8500	Other bone disease and musculoskeletal deformities
212	M85011	Other bone disease and musculoskeletal deformities
212	M85012	Other bone disease and musculoskeletal deformities
212	M85019	Other bone disease and musculoskeletal deformities
212	M85021	Other bone disease and musculoskeletal deformities
212	M85022	Other bone disease and musculoskeletal deformities
212	M85029	Other bone disease and musculoskeletal deformities
212	M85031	Other bone disease and musculoskeletal deformities
212	M85032	Other bone disease and musculoskeletal deformities
212	M85039	Other bone disease and musculoskeletal deformities
212	M85041	Other bone disease and musculoskeletal deformities
212	M85042	Other bone disease and musculoskeletal deformities
212	M85049	Other bone disease and musculoskeletal deformities
212	M85051	Other bone disease and musculoskeletal deformities
212	M85052	Other bone disease and musculoskeletal deformities
212	M85059	Other bone disease and musculoskeletal deformities
212	M85061	Other bone disease and musculoskeletal deformities
212	M85062	Other bone disease and musculoskeletal deformities
212	M85069	Other bone disease and musculoskeletal deformities
212	M85071	Other bone disease and musculoskeletal deformities
212	M85072	Other bone disease and musculoskeletal deformities
212	M85079	Other bone disease and musculoskeletal deformities

212	M8508	Other bone disease and musculoskeletal deformities
212	M8509	Other bone disease and musculoskeletal deformities
212	M8510	Other bone disease and musculoskeletal deformities
212	M85111	Other bone disease and musculoskeletal deformities
212	M85112	Other bone disease and musculoskeletal deformities
212	M85119	Other bone disease and musculoskeletal deformities
212	M85121	Other bone disease and musculoskeletal deformities
212	M85122	Other bone disease and musculoskeletal deformities
212	M85129	Other bone disease and musculoskeletal deformities
212	M85131	Other bone disease and musculoskeletal deformities
212	M85132	Other bone disease and musculoskeletal deformities
212	M85139	Other bone disease and musculoskeletal deformities
212	M85141	Other bone disease and musculoskeletal deformities
212	M85142	Other bone disease and musculoskeletal deformities
212	M85149	Other bone disease and musculoskeletal deformities
212	M85151	Other bone disease and musculoskeletal deformities
212	M85152	Other bone disease and musculoskeletal deformities
212	M85159	Other bone disease and musculoskeletal deformities
212	M85161	Other bone disease and musculoskeletal deformities
212	M85162	Other bone disease and musculoskeletal deformities
212	M85169	Other bone disease and musculoskeletal deformities
212	M85171	Other bone disease and musculoskeletal deformities
212	M85172	Other bone disease and musculoskeletal deformities
212	M85179	Other bone disease and musculoskeletal deformities
212	M8518	Other bone disease and musculoskeletal deformities
212	M8519	Other bone disease and musculoskeletal deformities
212	M852	Other bone disease and musculoskeletal deformities
212	M8530	Other bone disease and musculoskeletal deformities
212	M85311	Other bone disease and musculoskeletal deformities
212	M85312	Other bone disease and musculoskeletal deformities
212	M85319	Other bone disease and musculoskeletal deformities
212	M85321	Other bone disease and musculoskeletal deformities
212	M85322	Other bone disease and musculoskeletal deformities
212	M85329	Other bone disease and musculoskeletal deformities
212	M85331	Other bone disease and musculoskeletal deformities
212	M85332	Other bone disease and musculoskeletal deformities
212	M85339	Other bone disease and musculoskeletal deformities
212	M85341	Other bone disease and musculoskeletal deformities
212	M85342	Other bone disease and musculoskeletal deformities
212	M85349	Other bone disease and musculoskeletal deformities
212	M85351	Other bone disease and musculoskeletal deformities
212	M85352	Other bone disease and musculoskeletal deformities
212	M85359	Other bone disease and musculoskeletal deformities
212	M85361	Other bone disease and musculoskeletal deformities
212	M85362	Other bone disease and musculoskeletal deformities
212	M85369	Other bone disease and musculoskeletal deformities
212	M85371	Other bone disease and musculoskeletal deformities
212	M85372	Other bone disease and musculoskeletal deformities
212	M85379	Other bone disease and musculoskeletal deformities
212	M8538	Other bone disease and musculoskeletal deformities
212	M8539	Other bone disease and musculoskeletal deformities

212	M8540	Other bone disease and musculoskeletal deformities
212	M85411	Other bone disease and musculoskeletal deformities
212	M85412	Other bone disease and musculoskeletal deformities
212	M85419	Other bone disease and musculoskeletal deformities
212	M85421	Other bone disease and musculoskeletal deformities
212	M85422	Other bone disease and musculoskeletal deformities
212	M85429	Other bone disease and musculoskeletal deformities
212	M85431	Other bone disease and musculoskeletal deformities
212	M85432	Other bone disease and musculoskeletal deformities
212	M85439	Other bone disease and musculoskeletal deformities
212	M85441	Other bone disease and musculoskeletal deformities
212	M85442	Other bone disease and musculoskeletal deformities
212	M85449	Other bone disease and musculoskeletal deformities
212	M85451	Other bone disease and musculoskeletal deformities
212	M85452	Other bone disease and musculoskeletal deformities
212	M85459	Other bone disease and musculoskeletal deformities
212	M85461	Other bone disease and musculoskeletal deformities
212	M85462	Other bone disease and musculoskeletal deformities
212	M85469	Other bone disease and musculoskeletal deformities
212	M85471	Other bone disease and musculoskeletal deformities
212	M85472	Other bone disease and musculoskeletal deformities
212	M85479	Other bone disease and musculoskeletal deformities
212	M8548	Other bone disease and musculoskeletal deformities
212	M8550	Other bone disease and musculoskeletal deformities
212	M85511	Other bone disease and musculoskeletal deformities
212	M85512	Other bone disease and musculoskeletal deformities
212	M85519	Other bone disease and musculoskeletal deformities
212	M85521	Other bone disease and musculoskeletal deformities
212	M85522	Other bone disease and musculoskeletal deformities
212	M85529	Other bone disease and musculoskeletal deformities
212	M85531	Other bone disease and musculoskeletal deformities
212	M85532	Other bone disease and musculoskeletal deformities
212	M85539	Other bone disease and musculoskeletal deformities
212	M85541	Other bone disease and musculoskeletal deformities
212	M85542	Other bone disease and musculoskeletal deformities
212	M85549	Other bone disease and musculoskeletal deformities
212	M85551	Other bone disease and musculoskeletal deformities
212	M85552	Other bone disease and musculoskeletal deformities
212	M85559	Other bone disease and musculoskeletal deformities
212	M85561	Other bone disease and musculoskeletal deformities
212	M85562	Other bone disease and musculoskeletal deformities
212	M85569	Other bone disease and musculoskeletal deformities
212	M85571	Other bone disease and musculoskeletal deformities
212	M85572	Other bone disease and musculoskeletal deformities
212	M85579	Other bone disease and musculoskeletal deformities
212	M8558	Other bone disease and musculoskeletal deformities
212	M8559	Other bone disease and musculoskeletal deformities
212	M8560	Other bone disease and musculoskeletal deformities
212	M85611	Other bone disease and musculoskeletal deformities
212	M85612	Other bone disease and musculoskeletal deformities
212	M85619	Other bone disease and musculoskeletal deformities

212	M85621	Other bone disease and musculoskeletal deformities
212	M85622	Other bone disease and musculoskeletal deformities
212	M85629	Other bone disease and musculoskeletal deformities
212	M85631	Other bone disease and musculoskeletal deformities
212	M85632	Other bone disease and musculoskeletal deformities
212	M85639	Other bone disease and musculoskeletal deformities
212	M85641	Other bone disease and musculoskeletal deformities
212	M85642	Other bone disease and musculoskeletal deformities
212	M85649	Other bone disease and musculoskeletal deformities
212	M85651	Other bone disease and musculoskeletal deformities
212	M85652	Other bone disease and musculoskeletal deformities
212	M85659	Other bone disease and musculoskeletal deformities
212	M85661	Other bone disease and musculoskeletal deformities
212	M85662	Other bone disease and musculoskeletal deformities
212	M85669	Other bone disease and musculoskeletal deformities
212	M85671	Other bone disease and musculoskeletal deformities
212	M85672	Other bone disease and musculoskeletal deformities
212	M85679	Other bone disease and musculoskeletal deformities
212	M8568	Other bone disease and musculoskeletal deformities
212	M8569	Other bone disease and musculoskeletal deformities
212	M8580	Other bone disease and musculoskeletal deformities
212	M85811	Other bone disease and musculoskeletal deformities
212	M85812	Other bone disease and musculoskeletal deformities
212	M85819	Other bone disease and musculoskeletal deformities
212	M85821	Other bone disease and musculoskeletal deformities
212	M85822	Other bone disease and musculoskeletal deformities
212	M85829	Other bone disease and musculoskeletal deformities
212	M85831	Other bone disease and musculoskeletal deformities
212	M85832	Other bone disease and musculoskeletal deformities
212	M85839	Other bone disease and musculoskeletal deformities
212	M85841	Other bone disease and musculoskeletal deformities
212	M85842	Other bone disease and musculoskeletal deformities
212	M85849	Other bone disease and musculoskeletal deformities
212	M85851	Other bone disease and musculoskeletal deformities
212	M85852	Other bone disease and musculoskeletal deformities
212	M85859	Other bone disease and musculoskeletal deformities
212	M85861	Other bone disease and musculoskeletal deformities
212	M85862	Other bone disease and musculoskeletal deformities
212	M85869	Other bone disease and musculoskeletal deformities
212	M85871	Other bone disease and musculoskeletal deformities
212	M85872	Other bone disease and musculoskeletal deformities
212	M85879	Other bone disease and musculoskeletal deformities
212	M8588	Other bone disease and musculoskeletal deformities
212	M8589	Other bone disease and musculoskeletal deformities
212	M859	Other bone disease and musculoskeletal deformities
212	M8700	Other bone disease and musculoskeletal deformities
212	M87011	Other bone disease and musculoskeletal deformities
212	M87012	Other bone disease and musculoskeletal deformities
212	M87019	Other bone disease and musculoskeletal deformities
212	M87021	Other bone disease and musculoskeletal deformities
212	M87022	Other bone disease and musculoskeletal deformities

212	M87029	Other bone disease and musculoskeletal deformities
212	M87031	Other bone disease and musculoskeletal deformities
212	M87032	Other bone disease and musculoskeletal deformities
212	M87033	Other bone disease and musculoskeletal deformities
212	M87034	Other bone disease and musculoskeletal deformities
212	M87035	Other bone disease and musculoskeletal deformities
212	M87036	Other bone disease and musculoskeletal deformities
212	M87037	Other bone disease and musculoskeletal deformities
212	M87038	Other bone disease and musculoskeletal deformities
212	M87039	Other bone disease and musculoskeletal deformities
212	M87041	Other bone disease and musculoskeletal deformities
212	M87042	Other bone disease and musculoskeletal deformities
212	M87043	Other bone disease and musculoskeletal deformities
212	M87044	Other bone disease and musculoskeletal deformities
212	M87045	Other bone disease and musculoskeletal deformities
212	M87046	Other bone disease and musculoskeletal deformities
212	M87050	Other bone disease and musculoskeletal deformities
212	M87051	Other bone disease and musculoskeletal deformities
212	M87052	Other bone disease and musculoskeletal deformities
212	M87059	Other bone disease and musculoskeletal deformities
212	M87061	Other bone disease and musculoskeletal deformities
212	M87062	Other bone disease and musculoskeletal deformities
212	M87063	Other bone disease and musculoskeletal deformities
212	M87064	Other bone disease and musculoskeletal deformities
212	M87065	Other bone disease and musculoskeletal deformities
212	M87066	Other bone disease and musculoskeletal deformities
212	M87071	Other bone disease and musculoskeletal deformities
212	M87072	Other bone disease and musculoskeletal deformities
212	M87073	Other bone disease and musculoskeletal deformities
212	M87074	Other bone disease and musculoskeletal deformities
212	M87075	Other bone disease and musculoskeletal deformities
212	M87076	Other bone disease and musculoskeletal deformities
212	M87077	Other bone disease and musculoskeletal deformities
212	M87078	Other bone disease and musculoskeletal deformities
212	M87079	Other bone disease and musculoskeletal deformities
212	M8708	Other bone disease and musculoskeletal deformities
212	M8709	Other bone disease and musculoskeletal deformities
212	M8710	Other bone disease and musculoskeletal deformities
212	M87111	Other bone disease and musculoskeletal deformities
212	M87112	Other bone disease and musculoskeletal deformities
212	M87119	Other bone disease and musculoskeletal deformities
212	M87121	Other bone disease and musculoskeletal deformities
212	M87122	Other bone disease and musculoskeletal deformities
212	M87129	Other bone disease and musculoskeletal deformities
212	M87131	Other bone disease and musculoskeletal deformities
212	M87132	Other bone disease and musculoskeletal deformities
212	M87133	Other bone disease and musculoskeletal deformities
212	M87134	Other bone disease and musculoskeletal deformities
212	M87135	Other bone disease and musculoskeletal deformities
212	M87136	Other bone disease and musculoskeletal deformities
212	M87137	Other bone disease and musculoskeletal deformities

212	M87138	Other bone disease and musculoskeletal deformities
212	M87139	Other bone disease and musculoskeletal deformities
212	M87141	Other bone disease and musculoskeletal deformities
212	M87142	Other bone disease and musculoskeletal deformities
212	M87143	Other bone disease and musculoskeletal deformities
212	M87144	Other bone disease and musculoskeletal deformities
212	M87145	Other bone disease and musculoskeletal deformities
212	M87146	Other bone disease and musculoskeletal deformities
212	M87150	Other bone disease and musculoskeletal deformities
212	M87151	Other bone disease and musculoskeletal deformities
212	M87152	Other bone disease and musculoskeletal deformities
212	M87159	Other bone disease and musculoskeletal deformities
212	M87161	Other bone disease and musculoskeletal deformities
212	M87162	Other bone disease and musculoskeletal deformities
212	M87163	Other bone disease and musculoskeletal deformities
212	M87164	Other bone disease and musculoskeletal deformities
212	M87165	Other bone disease and musculoskeletal deformities
212	M87166	Other bone disease and musculoskeletal deformities
212	M87171	Other bone disease and musculoskeletal deformities
212	M87172	Other bone disease and musculoskeletal deformities
212	M87173	Other bone disease and musculoskeletal deformities
212	M87174	Other bone disease and musculoskeletal deformities
212	M87175	Other bone disease and musculoskeletal deformities
212	M87176	Other bone disease and musculoskeletal deformities
212	M87177	Other bone disease and musculoskeletal deformities
212	M87178	Other bone disease and musculoskeletal deformities
212	M87179	Other bone disease and musculoskeletal deformities
212	M87180	Other bone disease and musculoskeletal deformities
212	M87188	Other bone disease and musculoskeletal deformities
212	M8719	Other bone disease and musculoskeletal deformities
212	M8720	Other bone disease and musculoskeletal deformities
212	M87211	Other bone disease and musculoskeletal deformities
212	M87212	Other bone disease and musculoskeletal deformities
212	M87219	Other bone disease and musculoskeletal deformities
212	M87221	Other bone disease and musculoskeletal deformities
212	M87222	Other bone disease and musculoskeletal deformities
212	M87229	Other bone disease and musculoskeletal deformities
212	M87231	Other bone disease and musculoskeletal deformities
212	M87232	Other bone disease and musculoskeletal deformities
212	M87233	Other bone disease and musculoskeletal deformities
212	M87234	Other bone disease and musculoskeletal deformities
212	M87235	Other bone disease and musculoskeletal deformities
212	M87236	Other bone disease and musculoskeletal deformities
212	M87237	Other bone disease and musculoskeletal deformities
212	M87238	Other bone disease and musculoskeletal deformities
212	M87239	Other bone disease and musculoskeletal deformities
212	M87241	Other bone disease and musculoskeletal deformities
212	M87242	Other bone disease and musculoskeletal deformities
212	M87243	Other bone disease and musculoskeletal deformities
212	M87244	Other bone disease and musculoskeletal deformities
212	M87245	Other bone disease and musculoskeletal deformities

212	M87246	Other bone disease and musculoskeletal deformities
212	M87250	Other bone disease and musculoskeletal deformities
212	M87251	Other bone disease and musculoskeletal deformities
212	M87252	Other bone disease and musculoskeletal deformities
212	M87256	Other bone disease and musculoskeletal deformities
212	M87261	Other bone disease and musculoskeletal deformities
212	M87262	Other bone disease and musculoskeletal deformities
212	M87263	Other bone disease and musculoskeletal deformities
212	M87264	Other bone disease and musculoskeletal deformities
212	M87265	Other bone disease and musculoskeletal deformities
212	M87266	Other bone disease and musculoskeletal deformities
212	M87271	Other bone disease and musculoskeletal deformities
212	M87272	Other bone disease and musculoskeletal deformities
212	M87273	Other bone disease and musculoskeletal deformities
212	M87274	Other bone disease and musculoskeletal deformities
212	M87275	Other bone disease and musculoskeletal deformities
212	M87276	Other bone disease and musculoskeletal deformities
212	M87277	Other bone disease and musculoskeletal deformities
212	M87278	Other bone disease and musculoskeletal deformities
212	M87279	Other bone disease and musculoskeletal deformities
212	M8728	Other bone disease and musculoskeletal deformities
212	M8729	Other bone disease and musculoskeletal deformities
212	M8730	Other bone disease and musculoskeletal deformities
212	M87311	Other bone disease and musculoskeletal deformities
212	M87312	Other bone disease and musculoskeletal deformities
212	M87319	Other bone disease and musculoskeletal deformities
212	M87321	Other bone disease and musculoskeletal deformities
212	M87322	Other bone disease and musculoskeletal deformities
212	M87329	Other bone disease and musculoskeletal deformities
212	M87331	Other bone disease and musculoskeletal deformities
212	M87332	Other bone disease and musculoskeletal deformities
212	M87333	Other bone disease and musculoskeletal deformities
212	M87334	Other bone disease and musculoskeletal deformities
212	M87335	Other bone disease and musculoskeletal deformities
212	M87336	Other bone disease and musculoskeletal deformities
212	M87337	Other bone disease and musculoskeletal deformities
212	M87338	Other bone disease and musculoskeletal deformities
212	M87339	Other bone disease and musculoskeletal deformities
212	M87341	Other bone disease and musculoskeletal deformities
212	M87342	Other bone disease and musculoskeletal deformities
212	M87343	Other bone disease and musculoskeletal deformities
212	M87344	Other bone disease and musculoskeletal deformities
212	M87345	Other bone disease and musculoskeletal deformities
212	M87346	Other bone disease and musculoskeletal deformities
212	M87350	Other bone disease and musculoskeletal deformities
212	M87351	Other bone disease and musculoskeletal deformities
212	M87352	Other bone disease and musculoskeletal deformities
212	M87353	Other bone disease and musculoskeletal deformities
212	M87361	Other bone disease and musculoskeletal deformities
212	M87362	Other bone disease and musculoskeletal deformities
212	M87363	Other bone disease and musculoskeletal deformities

212	M87364	Other bone disease and musculoskeletal deformities
212	M87365	Other bone disease and musculoskeletal deformities
212	M87366	Other bone disease and musculoskeletal deformities
212	M87371	Other bone disease and musculoskeletal deformities
212	M87372	Other bone disease and musculoskeletal deformities
212	M87373	Other bone disease and musculoskeletal deformities
212	M87374	Other bone disease and musculoskeletal deformities
212	M87375	Other bone disease and musculoskeletal deformities
212	M87376	Other bone disease and musculoskeletal deformities
212	M87377	Other bone disease and musculoskeletal deformities
212	M87378	Other bone disease and musculoskeletal deformities
212	M87379	Other bone disease and musculoskeletal deformities
212	M8738	Other bone disease and musculoskeletal deformities
212	M8739	Other bone disease and musculoskeletal deformities
212	M8780	Other bone disease and musculoskeletal deformities
212	M87811	Other bone disease and musculoskeletal deformities
212	M87812	Other bone disease and musculoskeletal deformities
212	M87819	Other bone disease and musculoskeletal deformities
212	M87821	Other bone disease and musculoskeletal deformities
212	M87822	Other bone disease and musculoskeletal deformities
212	M87829	Other bone disease and musculoskeletal deformities
212	M87831	Other bone disease and musculoskeletal deformities
212	M87832	Other bone disease and musculoskeletal deformities
212	M87833	Other bone disease and musculoskeletal deformities
212	M87834	Other bone disease and musculoskeletal deformities
212	M87835	Other bone disease and musculoskeletal deformities
212	M87836	Other bone disease and musculoskeletal deformities
212	M87837	Other bone disease and musculoskeletal deformities
212	M87838	Other bone disease and musculoskeletal deformities
212	M87839	Other bone disease and musculoskeletal deformities
212	M87841	Other bone disease and musculoskeletal deformities
212	M87842	Other bone disease and musculoskeletal deformities
212	M87843	Other bone disease and musculoskeletal deformities
212	M87844	Other bone disease and musculoskeletal deformities
212	M87845	Other bone disease and musculoskeletal deformities
212	M87849	Other bone disease and musculoskeletal deformities
212	M87850	Other bone disease and musculoskeletal deformities
212	M87851	Other bone disease and musculoskeletal deformities
212	M87852	Other bone disease and musculoskeletal deformities
212	M87859	Other bone disease and musculoskeletal deformities
212	M87861	Other bone disease and musculoskeletal deformities
212	M87862	Other bone disease and musculoskeletal deformities
212	M87863	Other bone disease and musculoskeletal deformities
212	M87864	Other bone disease and musculoskeletal deformities
212	M87865	Other bone disease and musculoskeletal deformities
212	M87869	Other bone disease and musculoskeletal deformities
212	M87871	Other bone disease and musculoskeletal deformities
212	M87872	Other bone disease and musculoskeletal deformities
212	M87873	Other bone disease and musculoskeletal deformities
212	M87874	Other bone disease and musculoskeletal deformities
212	M87875	Other bone disease and musculoskeletal deformities

212	M87876	Other bone disease and musculoskeletal deformities
212	M87877	Other bone disease and musculoskeletal deformities
212	M87878	Other bone disease and musculoskeletal deformities
212	M87879	Other bone disease and musculoskeletal deformities
212	M8788	Other bone disease and musculoskeletal deformities
212	M8789	Other bone disease and musculoskeletal deformities
212	M879	Other bone disease and musculoskeletal deformities
212	M880	Other bone disease and musculoskeletal deformities
212	M881	Other bone disease and musculoskeletal deformities
212	M88811	Other bone disease and musculoskeletal deformities
212	M88812	Other bone disease and musculoskeletal deformities
212	M88819	Other bone disease and musculoskeletal deformities
212	M88821	Other bone disease and musculoskeletal deformities
212	M88822	Other bone disease and musculoskeletal deformities
212	M88829	Other bone disease and musculoskeletal deformities
212	M88831	Other bone disease and musculoskeletal deformities
212	M88832	Other bone disease and musculoskeletal deformities
212	M88839	Other bone disease and musculoskeletal deformities
212	M88841	Other bone disease and musculoskeletal deformities
212	M88842	Other bone disease and musculoskeletal deformities
212	M88849	Other bone disease and musculoskeletal deformities
212	M88851	Other bone disease and musculoskeletal deformities
212	M88852	Other bone disease and musculoskeletal deformities
212	M88859	Other bone disease and musculoskeletal deformities
212	M88861	Other bone disease and musculoskeletal deformities
212	M88862	Other bone disease and musculoskeletal deformities
212	M88869	Other bone disease and musculoskeletal deformities
212	M88871	Other bone disease and musculoskeletal deformities
212	M88872	Other bone disease and musculoskeletal deformities
212	M88879	Other bone disease and musculoskeletal deformities
212	M8888	Other bone disease and musculoskeletal deformities
212	M8889	Other bone disease and musculoskeletal deformities
212	M889	Other bone disease and musculoskeletal deformities
212	M8900	Other bone disease and musculoskeletal deformities
212	M89011	Other bone disease and musculoskeletal deformities
212	M89012	Other bone disease and musculoskeletal deformities
212	M89019	Other bone disease and musculoskeletal deformities
212	M89021	Other bone disease and musculoskeletal deformities
212	M89022	Other bone disease and musculoskeletal deformities
212	M89029	Other bone disease and musculoskeletal deformities
212	M89031	Other bone disease and musculoskeletal deformities
212	M89032	Other bone disease and musculoskeletal deformities
212	M89039	Other bone disease and musculoskeletal deformities
212	M89041	Other bone disease and musculoskeletal deformities
212	M89042	Other bone disease and musculoskeletal deformities
212	M89049	Other bone disease and musculoskeletal deformities
212	M89051	Other bone disease and musculoskeletal deformities
212	M89052	Other bone disease and musculoskeletal deformities
212	M89059	Other bone disease and musculoskeletal deformities
212	M89061	Other bone disease and musculoskeletal deformities
212	M89062	Other bone disease and musculoskeletal deformities

212	M89069	Other bone disease and musculoskeletal deformities
212	M89071	Other bone disease and musculoskeletal deformities
212	M89072	Other bone disease and musculoskeletal deformities
212	M89079	Other bone disease and musculoskeletal deformities
212	M8908	Other bone disease and musculoskeletal deformities
212	M8909	Other bone disease and musculoskeletal deformities
212	M89121	Other bone disease and musculoskeletal deformities
212	M89122	Other bone disease and musculoskeletal deformities
212	M89123	Other bone disease and musculoskeletal deformities
212	M89124	Other bone disease and musculoskeletal deformities
212	M89125	Other bone disease and musculoskeletal deformities
212	M89126	Other bone disease and musculoskeletal deformities
212	M89127	Other bone disease and musculoskeletal deformities
212	M89128	Other bone disease and musculoskeletal deformities
212	M89129	Other bone disease and musculoskeletal deformities
212	M89131	Other bone disease and musculoskeletal deformities
212	M89132	Other bone disease and musculoskeletal deformities
212	M89133	Other bone disease and musculoskeletal deformities
212	M89134	Other bone disease and musculoskeletal deformities
212	M89138	Other bone disease and musculoskeletal deformities
212	M89139	Other bone disease and musculoskeletal deformities
212	M89151	Other bone disease and musculoskeletal deformities
212	M89152	Other bone disease and musculoskeletal deformities
212	M89153	Other bone disease and musculoskeletal deformities
212	M89154	Other bone disease and musculoskeletal deformities
212	M89155	Other bone disease and musculoskeletal deformities
212	M89156	Other bone disease and musculoskeletal deformities
212	M89157	Other bone disease and musculoskeletal deformities
212	M89158	Other bone disease and musculoskeletal deformities
212	M89159	Other bone disease and musculoskeletal deformities
212	M89160	Other bone disease and musculoskeletal deformities
212	M89161	Other bone disease and musculoskeletal deformities
212	M89162	Other bone disease and musculoskeletal deformities
212	M89163	Other bone disease and musculoskeletal deformities
212	M89164	Other bone disease and musculoskeletal deformities
212	M89165	Other bone disease and musculoskeletal deformities
212	M89166	Other bone disease and musculoskeletal deformities
212	M89167	Other bone disease and musculoskeletal deformities
212	M89168	Other bone disease and musculoskeletal deformities
212	M89169	Other bone disease and musculoskeletal deformities
212	M8918	Other bone disease and musculoskeletal deformities
212	M8920	Other bone disease and musculoskeletal deformities
212	M89211	Other bone disease and musculoskeletal deformities
212	M89212	Other bone disease and musculoskeletal deformities
212	M89219	Other bone disease and musculoskeletal deformities
212	M89221	Other bone disease and musculoskeletal deformities
212	M89222	Other bone disease and musculoskeletal deformities
212	M89229	Other bone disease and musculoskeletal deformities
212	M89231	Other bone disease and musculoskeletal deformities
212	M89232	Other bone disease and musculoskeletal deformities
212	M89233	Other bone disease and musculoskeletal deformities

212	M89234	Other bone disease and musculoskeletal deformities
212	M89239	Other bone disease and musculoskeletal deformities
212	M89241	Other bone disease and musculoskeletal deformities
212	M89242	Other bone disease and musculoskeletal deformities
212	M89249	Other bone disease and musculoskeletal deformities
212	M89251	Other bone disease and musculoskeletal deformities
212	M89252	Other bone disease and musculoskeletal deformities
212	M89259	Other bone disease and musculoskeletal deformities
212	M89261	Other bone disease and musculoskeletal deformities
212	M89262	Other bone disease and musculoskeletal deformities
212	M89263	Other bone disease and musculoskeletal deformities
212	M89264	Other bone disease and musculoskeletal deformities
212	M89269	Other bone disease and musculoskeletal deformities
212	M89271	Other bone disease and musculoskeletal deformities
212	M89272	Other bone disease and musculoskeletal deformities
212	M89279	Other bone disease and musculoskeletal deformities
212	M8928	Other bone disease and musculoskeletal deformities
212	M8929	Other bone disease and musculoskeletal deformities
212	M8930	Other bone disease and musculoskeletal deformities
212	M89311	Other bone disease and musculoskeletal deformities
212	M89312	Other bone disease and musculoskeletal deformities
212	M89319	Other bone disease and musculoskeletal deformities
212	M89321	Other bone disease and musculoskeletal deformities
212	M89322	Other bone disease and musculoskeletal deformities
212	M89329	Other bone disease and musculoskeletal deformities
212	M89331	Other bone disease and musculoskeletal deformities
212	M89332	Other bone disease and musculoskeletal deformities
212	M89333	Other bone disease and musculoskeletal deformities
212	M89334	Other bone disease and musculoskeletal deformities
212	M89339	Other bone disease and musculoskeletal deformities
212	M89341	Other bone disease and musculoskeletal deformities
212	M89342	Other bone disease and musculoskeletal deformities
212	M89349	Other bone disease and musculoskeletal deformities
212	M89351	Other bone disease and musculoskeletal deformities
212	M89352	Other bone disease and musculoskeletal deformities
212	M89359	Other bone disease and musculoskeletal deformities
212	M89361	Other bone disease and musculoskeletal deformities
212	M89362	Other bone disease and musculoskeletal deformities
212	M89363	Other bone disease and musculoskeletal deformities
212	M89364	Other bone disease and musculoskeletal deformities
212	M89369	Other bone disease and musculoskeletal deformities
212	M89371	Other bone disease and musculoskeletal deformities
212	M89372	Other bone disease and musculoskeletal deformities
212	M89379	Other bone disease and musculoskeletal deformities
212	M8938	Other bone disease and musculoskeletal deformities
212	M8939	Other bone disease and musculoskeletal deformities
212	M8940	Other bone disease and musculoskeletal deformities
212	M89411	Other bone disease and musculoskeletal deformities
212	M89412	Other bone disease and musculoskeletal deformities
212	M89419	Other bone disease and musculoskeletal deformities
212	M89421	Other bone disease and musculoskeletal deformities

212	M89422	Other bone disease and musculoskeletal deformities
212	M89429	Other bone disease and musculoskeletal deformities
212	M89431	Other bone disease and musculoskeletal deformities
212	M89432	Other bone disease and musculoskeletal deformities
212	M89439	Other bone disease and musculoskeletal deformities
212	M89441	Other bone disease and musculoskeletal deformities
212	M89442	Other bone disease and musculoskeletal deformities
212	M89449	Other bone disease and musculoskeletal deformities
212	M89451	Other bone disease and musculoskeletal deformities
212	M89452	Other bone disease and musculoskeletal deformities
212	M89459	Other bone disease and musculoskeletal deformities
212	M89461	Other bone disease and musculoskeletal deformities
212	M89462	Other bone disease and musculoskeletal deformities
212	M89469	Other bone disease and musculoskeletal deformities
212	M89471	Other bone disease and musculoskeletal deformities
212	M89472	Other bone disease and musculoskeletal deformities
212	M89479	Other bone disease and musculoskeletal deformities
212	M8948	Other bone disease and musculoskeletal deformities
212	M8949	Other bone disease and musculoskeletal deformities
212	M8950	Other bone disease and musculoskeletal deformities
212	M89511	Other bone disease and musculoskeletal deformities
212	M89512	Other bone disease and musculoskeletal deformities
212	M89519	Other bone disease and musculoskeletal deformities
212	M89521	Other bone disease and musculoskeletal deformities
212	M89522	Other bone disease and musculoskeletal deformities
212	M89529	Other bone disease and musculoskeletal deformities
212	M89531	Other bone disease and musculoskeletal deformities
212	M89532	Other bone disease and musculoskeletal deformities
212	M89539	Other bone disease and musculoskeletal deformities
212	M89541	Other bone disease and musculoskeletal deformities
212	M89542	Other bone disease and musculoskeletal deformities
212	M89549	Other bone disease and musculoskeletal deformities
212	M89551	Other bone disease and musculoskeletal deformities
212	M89552	Other bone disease and musculoskeletal deformities
212	M89559	Other bone disease and musculoskeletal deformities
212	M89561	Other bone disease and musculoskeletal deformities
212	M89562	Other bone disease and musculoskeletal deformities
212	M89569	Other bone disease and musculoskeletal deformities
212	M89571	Other bone disease and musculoskeletal deformities
212	M89572	Other bone disease and musculoskeletal deformities
212	M89579	Other bone disease and musculoskeletal deformities
212	M8958	Other bone disease and musculoskeletal deformities
212	M8959	Other bone disease and musculoskeletal deformities
212	M8970	Other bone disease and musculoskeletal deformities
212	M89711	Other bone disease and musculoskeletal deformities
212	M89712	Other bone disease and musculoskeletal deformities
212	M89719	Other bone disease and musculoskeletal deformities
212	M89721	Other bone disease and musculoskeletal deformities
212	M89722	Other bone disease and musculoskeletal deformities
212	M89729	Other bone disease and musculoskeletal deformities
212	M89731	Other bone disease and musculoskeletal deformities

212	M89732	Other bone disease and musculoskeletal deformities
212	M89739	Other bone disease and musculoskeletal deformities
212	M89741	Other bone disease and musculoskeletal deformities
212	M89742	Other bone disease and musculoskeletal deformities
212	M89749	Other bone disease and musculoskeletal deformities
212	M89751	Other bone disease and musculoskeletal deformities
212	M89752	Other bone disease and musculoskeletal deformities
212	M89759	Other bone disease and musculoskeletal deformities
212	M89761	Other bone disease and musculoskeletal deformities
212	M89762	Other bone disease and musculoskeletal deformities
212	M89769	Other bone disease and musculoskeletal deformities
212	M89771	Other bone disease and musculoskeletal deformities
212	M89772	Other bone disease and musculoskeletal deformities
212	M89779	Other bone disease and musculoskeletal deformities
212	M8978	Other bone disease and musculoskeletal deformities
212	M8979	Other bone disease and musculoskeletal deformities
212	M898X0	Other bone disease and musculoskeletal deformities
212	M898X1	Other bone disease and musculoskeletal deformities
212	M898X2	Other bone disease and musculoskeletal deformities
212	M898X3	Other bone disease and musculoskeletal deformities
212	M898X4	Other bone disease and musculoskeletal deformities
212	M898X5	Other bone disease and musculoskeletal deformities
212	M898X6	Other bone disease and musculoskeletal deformities
212	M898X7	Other bone disease and musculoskeletal deformities
212	M898X8	Other bone disease and musculoskeletal deformities
212	M898X9	Other bone disease and musculoskeletal deformities
212	M899	Other bone disease and musculoskeletal deformities
212	M9050	Other bone disease and musculoskeletal deformities
212	M90511	Other bone disease and musculoskeletal deformities
212	M90512	Other bone disease and musculoskeletal deformities
212	M90519	Other bone disease and musculoskeletal deformities
212	M90521	Other bone disease and musculoskeletal deformities
212	M90522	Other bone disease and musculoskeletal deformities
212	M90529	Other bone disease and musculoskeletal deformities
212	M90531	Other bone disease and musculoskeletal deformities
212	M90532	Other bone disease and musculoskeletal deformities
212	M90539	Other bone disease and musculoskeletal deformities
212	M90541	Other bone disease and musculoskeletal deformities
212	M90542	Other bone disease and musculoskeletal deformities
212	M90549	Other bone disease and musculoskeletal deformities
212	M90551	Other bone disease and musculoskeletal deformities
212	M90552	Other bone disease and musculoskeletal deformities
212	M90559	Other bone disease and musculoskeletal deformities
212	M90561	Other bone disease and musculoskeletal deformities
212	M90562	Other bone disease and musculoskeletal deformities
212	M90569	Other bone disease and musculoskeletal deformities
212	M90571	Other bone disease and musculoskeletal deformities
212	M90572	Other bone disease and musculoskeletal deformities
212	M90579	Other bone disease and musculoskeletal deformities
212	M9058	Other bone disease and musculoskeletal deformities
212	M9059	Other bone disease and musculoskeletal deformities

212	M9060	Other bone disease and musculoskeletal deformities
212	M90611	Other bone disease and musculoskeletal deformities
212	M90612	Other bone disease and musculoskeletal deformities
212	M90619	Other bone disease and musculoskeletal deformities
212	M90621	Other bone disease and musculoskeletal deformities
212	M90622	Other bone disease and musculoskeletal deformities
212	M90629	Other bone disease and musculoskeletal deformities
212	M90631	Other bone disease and musculoskeletal deformities
212	M90632	Other bone disease and musculoskeletal deformities
212	M90639	Other bone disease and musculoskeletal deformities
212	M90641	Other bone disease and musculoskeletal deformities
212	M90642	Other bone disease and musculoskeletal deformities
212	M90649	Other bone disease and musculoskeletal deformities
212	M90651	Other bone disease and musculoskeletal deformities
212	M90652	Other bone disease and musculoskeletal deformities
212	M90659	Other bone disease and musculoskeletal deformities
212	M90661	Other bone disease and musculoskeletal deformities
212	M90662	Other bone disease and musculoskeletal deformities
212	M90669	Other bone disease and musculoskeletal deformities
212	M90671	Other bone disease and musculoskeletal deformities
212	M90672	Other bone disease and musculoskeletal deformities
212	M90679	Other bone disease and musculoskeletal deformities
212	M9068	Other bone disease and musculoskeletal deformities
212	M9069	Other bone disease and musculoskeletal deformities
212	M9080	Other bone disease and musculoskeletal deformities
212	M90811	Other bone disease and musculoskeletal deformities
212	M90812	Other bone disease and musculoskeletal deformities
212	M90819	Other bone disease and musculoskeletal deformities
212	M90821	Other bone disease and musculoskeletal deformities
212	M90822	Other bone disease and musculoskeletal deformities
212	M90829	Other bone disease and musculoskeletal deformities
212	M90831	Other bone disease and musculoskeletal deformities
212	M90832	Other bone disease and musculoskeletal deformities
212	M90839	Other bone disease and musculoskeletal deformities
212	M90841	Other bone disease and musculoskeletal deformities
212	M90842	Other bone disease and musculoskeletal deformities
212	M90849	Other bone disease and musculoskeletal deformities
212	M90851	Other bone disease and musculoskeletal deformities
212	M90852	Other bone disease and musculoskeletal deformities
212	M90859	Other bone disease and musculoskeletal deformities
212	M90861	Other bone disease and musculoskeletal deformities
212	M90862	Other bone disease and musculoskeletal deformities
212	M90869	Other bone disease and musculoskeletal deformities
212	M90871	Other bone disease and musculoskeletal deformities
212	M90872	Other bone disease and musculoskeletal deformities
212	M90879	Other bone disease and musculoskeletal deformities
212	M9088	Other bone disease and musculoskeletal deformities
212	M9089	Other bone disease and musculoskeletal deformities
212	M910	Other bone disease and musculoskeletal deformities
212	M9110	Other bone disease and musculoskeletal deformities
212	M9111	Other bone disease and musculoskeletal deformities

212	M9112	Other bone disease and musculoskeletal deformities
212	M9120	Other bone disease and musculoskeletal deformities
212	M9121	Other bone disease and musculoskeletal deformities
212	M9122	Other bone disease and musculoskeletal deformities
212	M9130	Other bone disease and musculoskeletal deformities
212	M9131	Other bone disease and musculoskeletal deformities
212	M9132	Other bone disease and musculoskeletal deformities
212	M9140	Other bone disease and musculoskeletal deformities
212	M9141	Other bone disease and musculoskeletal deformities
212	M9142	Other bone disease and musculoskeletal deformities
212	M9180	Other bone disease and musculoskeletal deformities
212	M9181	Other bone disease and musculoskeletal deformities
212	M9182	Other bone disease and musculoskeletal deformities
212	M9190	Other bone disease and musculoskeletal deformities
212	M9191	Other bone disease and musculoskeletal deformities
212	M9192	Other bone disease and musculoskeletal deformities
212	M9200	Other bone disease and musculoskeletal deformities
212	M9201	Other bone disease and musculoskeletal deformities
212	M9202	Other bone disease and musculoskeletal deformities
212	M9210	Other bone disease and musculoskeletal deformities
212	M9211	Other bone disease and musculoskeletal deformities
212	M9212	Other bone disease and musculoskeletal deformities
212	M92201	Other bone disease and musculoskeletal deformities
212	M92202	Other bone disease and musculoskeletal deformities
212	M92209	Other bone disease and musculoskeletal deformities
212	M92211	Other bone disease and musculoskeletal deformities
212	M92212	Other bone disease and musculoskeletal deformities
212	M92219	Other bone disease and musculoskeletal deformities
212	M92221	Other bone disease and musculoskeletal deformities
212	M92222	Other bone disease and musculoskeletal deformities
212	M92229	Other bone disease and musculoskeletal deformities
212	M92291	Other bone disease and musculoskeletal deformities
212	M92292	Other bone disease and musculoskeletal deformities
212	M92299	Other bone disease and musculoskeletal deformities
212	M9230	Other bone disease and musculoskeletal deformities
212	M9231	Other bone disease and musculoskeletal deformities
212	M9232	Other bone disease and musculoskeletal deformities
212	M9240	Other bone disease and musculoskeletal deformities
212	M9241	Other bone disease and musculoskeletal deformities
212	M9242	Other bone disease and musculoskeletal deformities
212	M9250	Other bone disease and musculoskeletal deformities
212	M9251	Other bone disease and musculoskeletal deformities
212	M9252	Other bone disease and musculoskeletal deformities
212	M9260	Other bone disease and musculoskeletal deformities
212	M9261	Other bone disease and musculoskeletal deformities
212	M9262	Other bone disease and musculoskeletal deformities
212	M9270	Other bone disease and musculoskeletal deformities
212	M9271	Other bone disease and musculoskeletal deformities
212	M9272	Other bone disease and musculoskeletal deformities
212	M928	Other bone disease and musculoskeletal deformities
212	M929	Other bone disease and musculoskeletal deformities

212	M93001	Other bone disease and musculoskeletal deformities
212	M93002	Other bone disease and musculoskeletal deformities
212	M93003	Other bone disease and musculoskeletal deformities
212	M93011	Other bone disease and musculoskeletal deformities
212	M93012	Other bone disease and musculoskeletal deformities
212	M93013	Other bone disease and musculoskeletal deformities
212	M93021	Other bone disease and musculoskeletal deformities
212	M93022	Other bone disease and musculoskeletal deformities
212	M93023	Other bone disease and musculoskeletal deformities
212	M93031	Other bone disease and musculoskeletal deformities
212	M93032	Other bone disease and musculoskeletal deformities
212	M93033	Other bone disease and musculoskeletal deformities
212	M931	Other bone disease and musculoskeletal deformities
212	M9320	Other bone disease and musculoskeletal deformities
212	M93211	Other bone disease and musculoskeletal deformities
212	M93212	Other bone disease and musculoskeletal deformities
212	M93219	Other bone disease and musculoskeletal deformities
212	M93221	Other bone disease and musculoskeletal deformities
212	M93222	Other bone disease and musculoskeletal deformities
212	M93229	Other bone disease and musculoskeletal deformities
212	M93231	Other bone disease and musculoskeletal deformities
212	M93232	Other bone disease and musculoskeletal deformities
212	M93239	Other bone disease and musculoskeletal deformities
212	M93241	Other bone disease and musculoskeletal deformities
212	M93242	Other bone disease and musculoskeletal deformities
212	M93249	Other bone disease and musculoskeletal deformities
212	M93251	Other bone disease and musculoskeletal deformities
212	M93252	Other bone disease and musculoskeletal deformities
212	M93259	Other bone disease and musculoskeletal deformities
212	M93261	Other bone disease and musculoskeletal deformities
212	M93262	Other bone disease and musculoskeletal deformities
212	M93269	Other bone disease and musculoskeletal deformities
212	M93271	Other bone disease and musculoskeletal deformities
212	M93272	Other bone disease and musculoskeletal deformities
212	M93279	Other bone disease and musculoskeletal deformities
212	M9328	Other bone disease and musculoskeletal deformities
212	M9329	Other bone disease and musculoskeletal deformities
212	M9380	Other bone disease and musculoskeletal deformities
212	M93811	Other bone disease and musculoskeletal deformities
212	M93812	Other bone disease and musculoskeletal deformities
212	M93819	Other bone disease and musculoskeletal deformities
212	M93821	Other bone disease and musculoskeletal deformities
212	M93822	Other bone disease and musculoskeletal deformities
212	M93829	Other bone disease and musculoskeletal deformities
212	M93831	Other bone disease and musculoskeletal deformities
212	M93832	Other bone disease and musculoskeletal deformities
212	M93839	Other bone disease and musculoskeletal deformities
212	M93841	Other bone disease and musculoskeletal deformities
212	M93842	Other bone disease and musculoskeletal deformities
212	M93849	Other bone disease and musculoskeletal deformities
212	M93851	Other bone disease and musculoskeletal deformities

212	M93852	Other bone disease and musculoskeletal deformities
212	M93859	Other bone disease and musculoskeletal deformities
212	M93861	Other bone disease and musculoskeletal deformities
212	M93862	Other bone disease and musculoskeletal deformities
212	M93869	Other bone disease and musculoskeletal deformities
212	M93871	Other bone disease and musculoskeletal deformities
212	M93872	Other bone disease and musculoskeletal deformities
212	M93879	Other bone disease and musculoskeletal deformities
212	M9388	Other bone disease and musculoskeletal deformities
212	M9389	Other bone disease and musculoskeletal deformities
212	M9390	Other bone disease and musculoskeletal deformities
212	M93911	Other bone disease and musculoskeletal deformities
212	M93912	Other bone disease and musculoskeletal deformities
212	M93919	Other bone disease and musculoskeletal deformities
212	M93921	Other bone disease and musculoskeletal deformities
212	M93922	Other bone disease and musculoskeletal deformities
212	M93929	Other bone disease and musculoskeletal deformities
212	M93931	Other bone disease and musculoskeletal deformities
212	M93932	Other bone disease and musculoskeletal deformities
212	M93939	Other bone disease and musculoskeletal deformities
212	M93941	Other bone disease and musculoskeletal deformities
212	M93942	Other bone disease and musculoskeletal deformities
212	M93949	Other bone disease and musculoskeletal deformities
212	M93951	Other bone disease and musculoskeletal deformities
212	M93952	Other bone disease and musculoskeletal deformities
212	M93959	Other bone disease and musculoskeletal deformities
212	M93961	Other bone disease and musculoskeletal deformities
212	M93962	Other bone disease and musculoskeletal deformities
212	M93969	Other bone disease and musculoskeletal deformities
212	M93971	Other bone disease and musculoskeletal deformities
212	M93972	Other bone disease and musculoskeletal deformities
212	M93979	Other bone disease and musculoskeletal deformities
212	M9398	Other bone disease and musculoskeletal deformities
212	M9399	Other bone disease and musculoskeletal deformities
212	M940	Other bone disease and musculoskeletal deformities
212	M941	Other bone disease and musculoskeletal deformities
212	M9420	Other bone disease and musculoskeletal deformities
212	M94211	Other bone disease and musculoskeletal deformities
212	M94212	Other bone disease and musculoskeletal deformities
212	M94219	Other bone disease and musculoskeletal deformities
212	M94221	Other bone disease and musculoskeletal deformities
212	M94222	Other bone disease and musculoskeletal deformities
212	M94229	Other bone disease and musculoskeletal deformities
212	M94231	Other bone disease and musculoskeletal deformities
212	M94232	Other bone disease and musculoskeletal deformities
212	M94239	Other bone disease and musculoskeletal deformities
212	M94241	Other bone disease and musculoskeletal deformities
212	M94242	Other bone disease and musculoskeletal deformities
212	M94249	Other bone disease and musculoskeletal deformities
212	M94251	Other bone disease and musculoskeletal deformities
212	M94252	Other bone disease and musculoskeletal deformities

212	M94259	Other bone disease and musculoskeletal deformities
212	M94261	Other bone disease and musculoskeletal deformities
212	M94262	Other bone disease and musculoskeletal deformities
212	M94269	Other bone disease and musculoskeletal deformities
212	M94271	Other bone disease and musculoskeletal deformities
212	M94272	Other bone disease and musculoskeletal deformities
212	M94279	Other bone disease and musculoskeletal deformities
212	M9428	Other bone disease and musculoskeletal deformities
212	M9429	Other bone disease and musculoskeletal deformities
212	M94351	Other bone disease and musculoskeletal deformities
212	M94352	Other bone disease and musculoskeletal deformities
212	M94359	Other bone disease and musculoskeletal deformities
212	M948X0	Other bone disease and musculoskeletal deformities
212	M948X1	Other bone disease and musculoskeletal deformities
212	M948X2	Other bone disease and musculoskeletal deformities
212	M948X3	Other bone disease and musculoskeletal deformities
212	M948X4	Other bone disease and musculoskeletal deformities
212	M948X5	Other bone disease and musculoskeletal deformities
212	M948X6	Other bone disease and musculoskeletal deformities
212	M948X7	Other bone disease and musculoskeletal deformities
212	M948X8	Other bone disease and musculoskeletal deformities
212	M948X9	Other bone disease and musculoskeletal deformities
212	M949	Other bone disease and musculoskeletal deformities
212	M9900	Other bone disease and musculoskeletal deformities
212	M9901	Other bone disease and musculoskeletal deformities
212	M9902	Other bone disease and musculoskeletal deformities
212	M9903	Other bone disease and musculoskeletal deformities
212	M9904	Other bone disease and musculoskeletal deformities
212	M9905	Other bone disease and musculoskeletal deformities
212	M9906	Other bone disease and musculoskeletal deformities
212	M9907	Other bone disease and musculoskeletal deformities
212	M9908	Other bone disease and musculoskeletal deformities
212	M9909	Other bone disease and musculoskeletal deformities
212	Z89011	Other bone disease and musculoskeletal deformities
212	Z89012	Other bone disease and musculoskeletal deformities
212	Z89019	Other bone disease and musculoskeletal deformities
212	Z89021	Other bone disease and musculoskeletal deformities
212	Z89022	Other bone disease and musculoskeletal deformities
212	Z89029	Other bone disease and musculoskeletal deformities
212	Z89111	Other bone disease and musculoskeletal deformities
212	Z89112	Other bone disease and musculoskeletal deformities
212	Z89119	Other bone disease and musculoskeletal deformities
212	Z89121	Other bone disease and musculoskeletal deformities
212	Z89122	Other bone disease and musculoskeletal deformities
212	Z89129	Other bone disease and musculoskeletal deformities
212	Z89201	Other bone disease and musculoskeletal deformities
212	Z89202	Other bone disease and musculoskeletal deformities
212	Z89209	Other bone disease and musculoskeletal deformities
212	Z89211	Other bone disease and musculoskeletal deformities
212	Z89212	Other bone disease and musculoskeletal deformities
212	Z89219	Other bone disease and musculoskeletal deformities

212	Z89221	Other bone disease and musculoskeletal deformities
212	Z89222	Other bone disease and musculoskeletal deformities
212	Z89229	Other bone disease and musculoskeletal deformities
212	Z89231	Other bone disease and musculoskeletal deformities
212	Z89232	Other bone disease and musculoskeletal deformities
212	Z89239	Other bone disease and musculoskeletal deformities
212	Z89411	Other bone disease and musculoskeletal deformities
212	Z89412	Other bone disease and musculoskeletal deformities
212	Z89419	Other bone disease and musculoskeletal deformities
212	Z89421	Other bone disease and musculoskeletal deformities
212	Z89422	Other bone disease and musculoskeletal deformities
212	Z89429	Other bone disease and musculoskeletal deformities
212	Z89431	Other bone disease and musculoskeletal deformities
212	Z89432	Other bone disease and musculoskeletal deformities
212	Z89439	Other bone disease and musculoskeletal deformities
212	Z89441	Other bone disease and musculoskeletal deformities
212	Z89442	Other bone disease and musculoskeletal deformities
212	Z89449	Other bone disease and musculoskeletal deformities
212	Z89511	Other bone disease and musculoskeletal deformities
212	Z89512	Other bone disease and musculoskeletal deformities
212	Z89519	Other bone disease and musculoskeletal deformities
212	Z89521	Other bone disease and musculoskeletal deformities
212	Z89522	Other bone disease and musculoskeletal deformities
212	Z89529	Other bone disease and musculoskeletal deformities
212	Z89611	Other bone disease and musculoskeletal deformities
212	Z89612	Other bone disease and musculoskeletal deformities
212	Z89619	Other bone disease and musculoskeletal deformities
212	Z89621	Other bone disease and musculoskeletal deformities
212	Z89622	Other bone disease and musculoskeletal deformities
212	Z89629	Other bone disease and musculoskeletal deformities
212	Z899	Other bone disease and musculoskeletal deformities
212	Z946	Other bone disease and musculoskeletal deformities
213	P293	Cardiac and circulatory congenital anomalies
213	Q200	Cardiac and circulatory congenital anomalies
213	Q201	Cardiac and circulatory congenital anomalies
213	Q202	Cardiac and circulatory congenital anomalies
213	Q203	Cardiac and circulatory congenital anomalies
213	Q204	Cardiac and circulatory congenital anomalies
213	Q205	Cardiac and circulatory congenital anomalies
213	Q206	Cardiac and circulatory congenital anomalies
213	Q208	Cardiac and circulatory congenital anomalies
213	Q209	Cardiac and circulatory congenital anomalies
213	Q210	Cardiac and circulatory congenital anomalies
213	Q211	Cardiac and circulatory congenital anomalies
213	Q212	Cardiac and circulatory congenital anomalies
213	Q213	Cardiac and circulatory congenital anomalies
213	Q214	Cardiac and circulatory congenital anomalies
213	Q218	Cardiac and circulatory congenital anomalies
213	Q219	Cardiac and circulatory congenital anomalies
213	Q220	Cardiac and circulatory congenital anomalies
213	Q221	Cardiac and circulatory congenital anomalies

213	Q222	Cardiac and circulatory congenital anomalies
213	Q223	Cardiac and circulatory congenital anomalies
213	Q224	Cardiac and circulatory congenital anomalies
213	Q225	Cardiac and circulatory congenital anomalies
213	Q226	Cardiac and circulatory congenital anomalies
213	Q228	Cardiac and circulatory congenital anomalies
213	Q229	Cardiac and circulatory congenital anomalies
213	Q230	Cardiac and circulatory congenital anomalies
213	Q231	Cardiac and circulatory congenital anomalies
213	Q232	Cardiac and circulatory congenital anomalies
213	Q233	Cardiac and circulatory congenital anomalies
213	Q234	Cardiac and circulatory congenital anomalies
213	Q238	Cardiac and circulatory congenital anomalies
213	Q239	Cardiac and circulatory congenital anomalies
213	Q240	Cardiac and circulatory congenital anomalies
213	Q241	Cardiac and circulatory congenital anomalies
213	Q242	Cardiac and circulatory congenital anomalies
213	Q243	Cardiac and circulatory congenital anomalies
213	Q244	Cardiac and circulatory congenital anomalies
213	Q245	Cardiac and circulatory congenital anomalies
213	Q246	Cardiac and circulatory congenital anomalies
213	Q248	Cardiac and circulatory congenital anomalies
213	Q249	Cardiac and circulatory congenital anomalies
213	Q250	Cardiac and circulatory congenital anomalies
213	Q251	Cardiac and circulatory congenital anomalies
213	Q252	Cardiac and circulatory congenital anomalies
213	Q253	Cardiac and circulatory congenital anomalies
213	Q254	Cardiac and circulatory congenital anomalies
213	Q255	Cardiac and circulatory congenital anomalies
213	Q256	Cardiac and circulatory congenital anomalies
213	Q2571	Cardiac and circulatory congenital anomalies
213	Q2572	Cardiac and circulatory congenital anomalies
213	Q2579	Cardiac and circulatory congenital anomalies
213	Q258	Cardiac and circulatory congenital anomalies
213	Q259	Cardiac and circulatory congenital anomalies
213	Q260	Cardiac and circulatory congenital anomalies
213	Q261	Cardiac and circulatory congenital anomalies
213	Q262	Cardiac and circulatory congenital anomalies
213	Q263	Cardiac and circulatory congenital anomalies
213	Q264	Cardiac and circulatory congenital anomalies
213	Q265	Cardiac and circulatory congenital anomalies
213	Q266	Cardiac and circulatory congenital anomalies
213	Q268	Cardiac and circulatory congenital anomalies
213	Q269	Cardiac and circulatory congenital anomalies
213	Q270	Cardiac and circulatory congenital anomalies
213	Q271	Cardiac and circulatory congenital anomalies
213	Q272	Cardiac and circulatory congenital anomalies
213	Q2730	Cardiac and circulatory congenital anomalies
213	Q2731	Cardiac and circulatory congenital anomalies
213	Q2732	Cardiac and circulatory congenital anomalies
213	Q2733	Cardiac and circulatory congenital anomalies

213	Q2734	Cardiac and circulatory congenital anomalies
213	Q2739	Cardiac and circulatory congenital anomalies
213	Q274	Cardiac and circulatory congenital anomalies
213	Q278	Cardiac and circulatory congenital anomalies
213	Q279	Cardiac and circulatory congenital anomalies
213	Q280	Cardiac and circulatory congenital anomalies
213	Q281	Cardiac and circulatory congenital anomalies
213	Q282	Cardiac and circulatory congenital anomalies
213	Q283	Cardiac and circulatory congenital anomalies
213	Q288	Cardiac and circulatory congenital anomalies
213	Q289	Cardiac and circulatory congenital anomalies
213	Z8774	Cardiac and circulatory congenital anomalies
214	Q380	Digestive congenital anomalies
214	Q381	Digestive congenital anomalies
214	Q382	Digestive congenital anomalies
214	Q383	Digestive congenital anomalies
214	Q384	Digestive congenital anomalies
214	Q385	Digestive congenital anomalies
214	Q386	Digestive congenital anomalies
214	Q387	Digestive congenital anomalies
214	Q388	Digestive congenital anomalies
214	Q390	Digestive congenital anomalies
214	Q391	Digestive congenital anomalies
214	Q392	Digestive congenital anomalies
214	Q393	Digestive congenital anomalies
214	Q394	Digestive congenital anomalies
214	Q395	Digestive congenital anomalies
214	Q396	Digestive congenital anomalies
214	Q398	Digestive congenital anomalies
214	Q399	Digestive congenital anomalies
214	Q400	Digestive congenital anomalies
214	Q401	Digestive congenital anomalies
214	Q402	Digestive congenital anomalies
214	Q403	Digestive congenital anomalies
214	Q408	Digestive congenital anomalies
214	Q409	Digestive congenital anomalies
214	Q410	Digestive congenital anomalies
214	Q411	Digestive congenital anomalies
214	Q412	Digestive congenital anomalies
214	Q418	Digestive congenital anomalies
214	Q419	Digestive congenital anomalies
214	Q420	Digestive congenital anomalies
214	Q421	Digestive congenital anomalies
214	Q422	Digestive congenital anomalies
214	Q423	Digestive congenital anomalies
214	Q428	Digestive congenital anomalies
214	Q429	Digestive congenital anomalies
214	Q430	Digestive congenital anomalies
214	Q431	Digestive congenital anomalies
214	Q432	Digestive congenital anomalies
214	Q433	Digestive congenital anomalies

214	Q434	Digestive congenital anomalies
214	Q435	Digestive congenital anomalies
214	Q436	Digestive congenital anomalies
214	Q437	Digestive congenital anomalies
214	Q438	Digestive congenital anomalies
214	Q439	Digestive congenital anomalies
214	Q440	Digestive congenital anomalies
214	Q441	Digestive congenital anomalies
214	Q442	Digestive congenital anomalies
214	Q443	Digestive congenital anomalies
214	Q444	Digestive congenital anomalies
214	Q445	Digestive congenital anomalies
214	Q446	Digestive congenital anomalies
214	Q447	Digestive congenital anomalies
214	Q450	Digestive congenital anomalies
214	Q451	Digestive congenital anomalies
214	Q452	Digestive congenital anomalies
214	Q453	Digestive congenital anomalies
214	Q458	Digestive congenital anomalies
214	Q459	Digestive congenital anomalies
214	Z87738	Digestive congenital anomalies
215	Q5001	Genitourinary congenital anomalies
215	Q5002	Genitourinary congenital anomalies
215	Q501	Genitourinary congenital anomalies
215	Q502	Genitourinary congenital anomalies
215	Q5031	Genitourinary congenital anomalies
215	Q5032	Genitourinary congenital anomalies
215	Q5039	Genitourinary congenital anomalies
215	Q504	Genitourinary congenital anomalies
215	Q505	Genitourinary congenital anomalies
215	Q506	Genitourinary congenital anomalies
215	Q510	Genitourinary congenital anomalies
215	Q5110	Genitourinary congenital anomalies
215	Q5111	Genitourinary congenital anomalies
215	Q512	Genitourinary congenital anomalies
215	Q513	Genitourinary congenital anomalies
215	Q514	Genitourinary congenital anomalies
215	Q515	Genitourinary congenital anomalies
215	Q516	Genitourinary congenital anomalies
215	Q517	Genitourinary congenital anomalies
215	Q51810	Genitourinary congenital anomalies
215	Q51811	Genitourinary congenital anomalies
215	Q51818	Genitourinary congenital anomalies
215	Q51820	Genitourinary congenital anomalies
215	Q51821	Genitourinary congenital anomalies
215	Q51828	Genitourinary congenital anomalies
215	Q519	Genitourinary congenital anomalies
215	Q520	Genitourinary congenital anomalies
215	Q5210	Genitourinary congenital anomalies
215	Q5211	Genitourinary congenital anomalies
215	Q5212	Genitourinary congenital anomalies

215	Q522	Genitourinary congenital anomalies
215	Q523	Genitourinary congenital anomalies
215	Q524	Genitourinary congenital anomalies
215	Q525	Genitourinary congenital anomalies
215	Q526	Genitourinary congenital anomalies
215	Q5270	Genitourinary congenital anomalies
215	Q5271	Genitourinary congenital anomalies
215	Q5279	Genitourinary congenital anomalies
215	Q528	Genitourinary congenital anomalies
215	Q529	Genitourinary congenital anomalies
215	Q5300	Genitourinary congenital anomalies
215	Q5301	Genitourinary congenital anomalies
215	Q5302	Genitourinary congenital anomalies
215	Q5310	Genitourinary congenital anomalies
215	Q5311	Genitourinary congenital anomalies
215	Q5312	Genitourinary congenital anomalies
215	Q5320	Genitourinary congenital anomalies
215	Q5321	Genitourinary congenital anomalies
215	Q5322	Genitourinary congenital anomalies
215	Q539	Genitourinary congenital anomalies
215	Q540	Genitourinary congenital anomalies
215	Q541	Genitourinary congenital anomalies
215	Q542	Genitourinary congenital anomalies
215	Q543	Genitourinary congenital anomalies
215	Q544	Genitourinary congenital anomalies
215	Q548	Genitourinary congenital anomalies
215	Q549	Genitourinary congenital anomalies
215	Q550	Genitourinary congenital anomalies
215	Q551	Genitourinary congenital anomalies
215	Q5520	Genitourinary congenital anomalies
215	Q5521	Genitourinary congenital anomalies
215	Q5522	Genitourinary congenital anomalies
215	Q5523	Genitourinary congenital anomalies
215	Q5529	Genitourinary congenital anomalies
215	Q553	Genitourinary congenital anomalies
215	Q554	Genitourinary congenital anomalies
215	Q555	Genitourinary congenital anomalies
215	Q5561	Genitourinary congenital anomalies
215	Q5562	Genitourinary congenital anomalies
215	Q5563	Genitourinary congenital anomalies
215	Q5564	Genitourinary congenital anomalies
215	Q5569	Genitourinary congenital anomalies
215	Q557	Genitourinary congenital anomalies
215	Q558	Genitourinary congenital anomalies
215	Q559	Genitourinary congenital anomalies
215	Q560	Genitourinary congenital anomalies
215	Q561	Genitourinary congenital anomalies
215	Q562	Genitourinary congenital anomalies
215	Q563	Genitourinary congenital anomalies
215	Q564	Genitourinary congenital anomalies
215	Q600	Genitourinary congenital anomalies

215	Q601	Genitourinary congenital anomalies
215	Q602	Genitourinary congenital anomalies
215	Q603	Genitourinary congenital anomalies
215	Q604	Genitourinary congenital anomalies
215	Q605	Genitourinary congenital anomalies
215	Q606	Genitourinary congenital anomalies
215	Q6100	Genitourinary congenital anomalies
215	Q6101	Genitourinary congenital anomalies
215	Q6102	Genitourinary congenital anomalies
215	Q6111	Genitourinary congenital anomalies
215	Q6119	Genitourinary congenital anomalies
215	Q612	Genitourinary congenital anomalies
215	Q613	Genitourinary congenital anomalies
215	Q614	Genitourinary congenital anomalies
215	Q615	Genitourinary congenital anomalies
215	Q618	Genitourinary congenital anomalies
215	Q619	Genitourinary congenital anomalies
215	Q620	Genitourinary congenital anomalies
215	Q6210	Genitourinary congenital anomalies
215	Q6211	Genitourinary congenital anomalies
215	Q6212	Genitourinary congenital anomalies
215	Q622	Genitourinary congenital anomalies
215	Q6231	Genitourinary congenital anomalies
215	Q6232	Genitourinary congenital anomalies
215	Q6239	Genitourinary congenital anomalies
215	Q624	Genitourinary congenital anomalies
215	Q625	Genitourinary congenital anomalies
215	Q6260	Genitourinary congenital anomalies
215	Q6261	Genitourinary congenital anomalies
215	Q6262	Genitourinary congenital anomalies
215	Q6263	Genitourinary congenital anomalies
215	Q6269	Genitourinary congenital anomalies
215	Q627	Genitourinary congenital anomalies
215	Q628	Genitourinary congenital anomalies
215	Q630	Genitourinary congenital anomalies
215	Q631	Genitourinary congenital anomalies
215	Q632	Genitourinary congenital anomalies
215	Q633	Genitourinary congenital anomalies
215	Q638	Genitourinary congenital anomalies
215	Q639	Genitourinary congenital anomalies
215	Q640	Genitourinary congenital anomalies
215	Q6410	Genitourinary congenital anomalies
215	Q6411	Genitourinary congenital anomalies
215	Q6412	Genitourinary congenital anomalies
215	Q6419	Genitourinary congenital anomalies
215	Q642	Genitourinary congenital anomalies
215	Q6431	Genitourinary congenital anomalies
215	Q6432	Genitourinary congenital anomalies
215	Q6433	Genitourinary congenital anomalies
215	Q6439	Genitourinary congenital anomalies
215	Q644	Genitourinary congenital anomalies

215	Q645	Genitourinary congenital anomalies
215	Q646	Genitourinary congenital anomalies
215	Q6470	Genitourinary congenital anomalies
215	Q6471	Genitourinary congenital anomalies
215	Q6472	Genitourinary congenital anomalies
215	Q6473	Genitourinary congenital anomalies
215	Q6474	Genitourinary congenital anomalies
215	Q6475	Genitourinary congenital anomalies
215	Q6479	Genitourinary congenital anomalies
215	Q648	Genitourinary congenital anomalies
215	Q649	Genitourinary congenital anomalies
215	Z87710	Genitourinary congenital anomalies
215	Z87718	Genitourinary congenital anomalies
216	G901	Nervous system congenital anomalies
216	Q000	Nervous system congenital anomalies
216	Q001	Nervous system congenital anomalies
216	Q002	Nervous system congenital anomalies
216	Q010	Nervous system congenital anomalies
216	Q011	Nervous system congenital anomalies
216	Q012	Nervous system congenital anomalies
216	Q018	Nervous system congenital anomalies
216	Q019	Nervous system congenital anomalies
216	Q02	Nervous system congenital anomalies
216	Q030	Nervous system congenital anomalies
216	Q031	Nervous system congenital anomalies
216	Q038	Nervous system congenital anomalies
216	Q039	Nervous system congenital anomalies
216	Q040	Nervous system congenital anomalies
216	Q041	Nervous system congenital anomalies
216	Q042	Nervous system congenital anomalies
216	Q043	Nervous system congenital anomalies
216	Q044	Nervous system congenital anomalies
216	Q045	Nervous system congenital anomalies
216	Q046	Nervous system congenital anomalies
216	Q048	Nervous system congenital anomalies
216	Q049	Nervous system congenital anomalies
216	Q050	Nervous system congenital anomalies
216	Q051	Nervous system congenital anomalies
216	Q052	Nervous system congenital anomalies
216	Q053	Nervous system congenital anomalies
216	Q054	Nervous system congenital anomalies
216	Q055	Nervous system congenital anomalies
216	Q056	Nervous system congenital anomalies
216	Q057	Nervous system congenital anomalies
216	Q058	Nervous system congenital anomalies
216	Q059	Nervous system congenital anomalies
216	Q060	Nervous system congenital anomalies
216	Q061	Nervous system congenital anomalies
216	Q062	Nervous system congenital anomalies
216	Q063	Nervous system congenital anomalies
216	Q064	Nervous system congenital anomalies

216	Q068	Nervous system congenital anomalies
216	Q069	Nervous system congenital anomalies
216	Q0700	Nervous system congenital anomalies
216	Q0701	Nervous system congenital anomalies
216	Q0702	Nervous system congenital anomalies
216	Q0703	Nervous system congenital anomalies
216	Q078	Nervous system congenital anomalies
216	Q079	Nervous system congenital anomalies
216	Q8500	Nervous system congenital anomalies
216	Q8501	Nervous system congenital anomalies
216	Q8502	Nervous system congenital anomalies
216	Q8503	Nervous system congenital anomalies
216	Q8509	Nervous system congenital anomalies
216	Z87728	Nervous system congenital anomalies
217	E7871	Other congenital anomalies
217	E7872	Other congenital anomalies
217	Q100	Other congenital anomalies
217	Q101	Other congenital anomalies
217	Q102	Other congenital anomalies
217	Q103	Other congenital anomalies
217	Q104	Other congenital anomalies
217	Q105	Other congenital anomalies
217	Q106	Other congenital anomalies
217	Q107	Other congenital anomalies
217	Q110	Other congenital anomalies
217	Q111	Other congenital anomalies
217	Q112	Other congenital anomalies
217	Q113	Other congenital anomalies
217	Q120	Other congenital anomalies
217	Q121	Other congenital anomalies
217	Q122	Other congenital anomalies
217	Q123	Other congenital anomalies
217	Q124	Other congenital anomalies
217	Q128	Other congenital anomalies
217	Q129	Other congenital anomalies
217	Q130	Other congenital anomalies
217	Q131	Other congenital anomalies
217	Q132	Other congenital anomalies
217	Q133	Other congenital anomalies
217	Q134	Other congenital anomalies
217	Q135	Other congenital anomalies
217	Q1381	Other congenital anomalies
217	Q1389	Other congenital anomalies
217	Q139	Other congenital anomalies
217	Q140	Other congenital anomalies
217	Q141	Other congenital anomalies
217	Q142	Other congenital anomalies
217	Q143	Other congenital anomalies
217	Q148	Other congenital anomalies
217	Q149	Other congenital anomalies
217	Q150	Other congenital anomalies

217	Q158	Other congenital anomalies
217	Q159	Other congenital anomalies
217	Q160	Other congenital anomalies
217	Q161	Other congenital anomalies
217	Q162	Other congenital anomalies
217	Q163	Other congenital anomalies
217	Q164	Other congenital anomalies
217	Q165	Other congenital anomalies
217	Q169	Other congenital anomalies
217	Q170	Other congenital anomalies
217	Q171	Other congenital anomalies
217	Q172	Other congenital anomalies
217	Q173	Other congenital anomalies
217	Q174	Other congenital anomalies
217	Q175	Other congenital anomalies
217	Q178	Other congenital anomalies
217	Q179	Other congenital anomalies
217	Q180	Other congenital anomalies
217	Q181	Other congenital anomalies
217	Q182	Other congenital anomalies
217	Q183	Other congenital anomalies
217	Q184	Other congenital anomalies
217	Q185	Other congenital anomalies
217	Q186	Other congenital anomalies
217	Q187	Other congenital anomalies
217	Q188	Other congenital anomalies
217	Q189	Other congenital anomalies
217	Q300	Other congenital anomalies
217	Q301	Other congenital anomalies
217	Q302	Other congenital anomalies
217	Q303	Other congenital anomalies
217	Q308	Other congenital anomalies
217	Q309	Other congenital anomalies
217	Q310	Other congenital anomalies
217	Q311	Other congenital anomalies
217	Q312	Other congenital anomalies
217	Q313	Other congenital anomalies
217	Q315	Other congenital anomalies
217	Q318	Other congenital anomalies
217	Q319	Other congenital anomalies
217	Q320	Other congenital anomalies
217	Q321	Other congenital anomalies
217	Q322	Other congenital anomalies
217	Q323	Other congenital anomalies
217	Q324	Other congenital anomalies
217	Q330	Other congenital anomalies
217	Q331	Other congenital anomalies
217	Q332	Other congenital anomalies
217	Q333	Other congenital anomalies
217	Q334	Other congenital anomalies
217	Q335	Other congenital anomalies

217	Q336	Other congenital anomalies
217	Q338	Other congenital anomalies
217	Q339	Other congenital anomalies
217	Q340	Other congenital anomalies
217	Q341	Other congenital anomalies
217	Q348	Other congenital anomalies
217	Q349	Other congenital anomalies
217	Q351	Other congenital anomalies
217	Q353	Other congenital anomalies
217	Q355	Other congenital anomalies
217	Q357	Other congenital anomalies
217	Q359	Other congenital anomalies
217	Q360	Other congenital anomalies
217	Q361	Other congenital anomalies
217	Q369	Other congenital anomalies
217	Q370	Other congenital anomalies
217	Q371	Other congenital anomalies
217	Q372	Other congenital anomalies
217	Q373	Other congenital anomalies
217	Q374	Other congenital anomalies
217	Q375	Other congenital anomalies
217	Q378	Other congenital anomalies
217	Q379	Other congenital anomalies
217	Q6500	Other congenital anomalies
217	Q6501	Other congenital anomalies
217	Q6502	Other congenital anomalies
217	Q651	Other congenital anomalies
217	Q652	Other congenital anomalies
217	Q6530	Other congenital anomalies
217	Q6531	Other congenital anomalies
217	Q6532	Other congenital anomalies
217	Q654	Other congenital anomalies
217	Q655	Other congenital anomalies
217	Q656	Other congenital anomalies
217	Q6581	Other congenital anomalies
217	Q6582	Other congenital anomalies
217	Q6589	Other congenital anomalies
217	Q659	Other congenital anomalies
217	Q660	Other congenital anomalies
217	Q661	Other congenital anomalies
217	Q662	Other congenital anomalies
217	Q663	Other congenital anomalies
217	Q664	Other congenital anomalies
217	Q6650	Other congenital anomalies
217	Q6651	Other congenital anomalies
217	Q6652	Other congenital anomalies
217	Q666	Other congenital anomalies
217	Q667	Other congenital anomalies
217	Q6680	Other congenital anomalies
217	Q6681	Other congenital anomalies
217	Q6682	Other congenital anomalies

217	Q6689	Other congenital anomalies
217	Q669	Other congenital anomalies
217	Q670	Other congenital anomalies
217	Q671	Other congenital anomalies
217	Q672	Other congenital anomalies
217	Q673	Other congenital anomalies
217	Q674	Other congenital anomalies
217	Q675	Other congenital anomalies
217	Q676	Other congenital anomalies
217	Q677	Other congenital anomalies
217	Q678	Other congenital anomalies
217	Q680	Other congenital anomalies
217	Q681	Other congenital anomalies
217	Q682	Other congenital anomalies
217	Q683	Other congenital anomalies
217	Q684	Other congenital anomalies
217	Q685	Other congenital anomalies
217	Q686	Other congenital anomalies
217	Q688	Other congenital anomalies
217	Q690	Other congenital anomalies
217	Q691	Other congenital anomalies
217	Q692	Other congenital anomalies
217	Q699	Other congenital anomalies
217	Q7000	Other congenital anomalies
217	Q7001	Other congenital anomalies
217	Q7002	Other congenital anomalies
217	Q7003	Other congenital anomalies
217	Q7010	Other congenital anomalies
217	Q7011	Other congenital anomalies
217	Q7012	Other congenital anomalies
217	Q7013	Other congenital anomalies
217	Q7020	Other congenital anomalies
217	Q7021	Other congenital anomalies
217	Q7022	Other congenital anomalies
217	Q7023	Other congenital anomalies
217	Q7030	Other congenital anomalies
217	Q7031	Other congenital anomalies
217	Q7032	Other congenital anomalies
217	Q7033	Other congenital anomalies
217	Q704	Other congenital anomalies
217	Q709	Other congenital anomalies
217	Q7100	Other congenital anomalies
217	Q7101	Other congenital anomalies
217	Q7102	Other congenital anomalies
217	Q7103	Other congenital anomalies
217	Q7110	Other congenital anomalies
217	Q7111	Other congenital anomalies
217	Q7112	Other congenital anomalies
217	Q7113	Other congenital anomalies
217	Q7120	Other congenital anomalies
217	Q7121	Other congenital anomalies

217	Q7122	Other congenital anomalies
217	Q7123	Other congenital anomalies
217	Q7130	Other congenital anomalies
217	Q7131	Other congenital anomalies
217	Q7132	Other congenital anomalies
217	Q7133	Other congenital anomalies
217	Q7140	Other congenital anomalies
217	Q7141	Other congenital anomalies
217	Q7142	Other congenital anomalies
217	Q7143	Other congenital anomalies
217	Q7150	Other congenital anomalies
217	Q7151	Other congenital anomalies
217	Q7152	Other congenital anomalies
217	Q7153	Other congenital anomalies
217	Q7160	Other congenital anomalies
217	Q7161	Other congenital anomalies
217	Q7162	Other congenital anomalies
217	Q7163	Other congenital anomalies
217	Q71811	Other congenital anomalies
217	Q71812	Other congenital anomalies
217	Q71813	Other congenital anomalies
217	Q71819	Other congenital anomalies
217	Q71891	Other congenital anomalies
217	Q71892	Other congenital anomalies
217	Q71893	Other congenital anomalies
217	Q71899	Other congenital anomalies
217	Q7190	Other congenital anomalies
217	Q7191	Other congenital anomalies
217	Q7192	Other congenital anomalies
217	Q7193	Other congenital anomalies
217	Q7200	Other congenital anomalies
217	Q7201	Other congenital anomalies
217	Q7202	Other congenital anomalies
217	Q7203	Other congenital anomalies
217	Q7210	Other congenital anomalies
217	Q7211	Other congenital anomalies
217	Q7212	Other congenital anomalies
217	Q7213	Other congenital anomalies
217	Q7220	Other congenital anomalies
217	Q7221	Other congenital anomalies
217	Q7222	Other congenital anomalies
217	Q7223	Other congenital anomalies
217	Q7230	Other congenital anomalies
217	Q7231	Other congenital anomalies
217	Q7232	Other congenital anomalies
217	Q7233	Other congenital anomalies
217	Q7240	Other congenital anomalies
217	Q7241	Other congenital anomalies
217	Q7242	Other congenital anomalies
217	Q7243	Other congenital anomalies
217	Q7250	Other congenital anomalies

217	Q7251	Other congenital anomalies
217	Q7252	Other congenital anomalies
217	Q7253	Other congenital anomalies
217	Q7260	Other congenital anomalies
217	Q7261	Other congenital anomalies
217	Q7262	Other congenital anomalies
217	Q7263	Other congenital anomalies
217	Q7270	Other congenital anomalies
217	Q7271	Other congenital anomalies
217	Q7272	Other congenital anomalies
217	Q7273	Other congenital anomalies
217	Q72811	Other congenital anomalies
217	Q72812	Other congenital anomalies
217	Q72813	Other congenital anomalies
217	Q72819	Other congenital anomalies
217	Q72891	Other congenital anomalies
217	Q72892	Other congenital anomalies
217	Q72893	Other congenital anomalies
217	Q72899	Other congenital anomalies
217	Q7290	Other congenital anomalies
217	Q7291	Other congenital anomalies
217	Q7292	Other congenital anomalies
217	Q7293	Other congenital anomalies
217	Q730	Other congenital anomalies
217	Q731	Other congenital anomalies
217	Q738	Other congenital anomalies
217	Q740	Other congenital anomalies
217	Q741	Other congenital anomalies
217	Q742	Other congenital anomalies
217	Q743	Other congenital anomalies
217	Q748	Other congenital anomalies
217	Q749	Other congenital anomalies
217	Q750	Other congenital anomalies
217	Q751	Other congenital anomalies
217	Q752	Other congenital anomalies
217	Q753	Other congenital anomalies
217	Q754	Other congenital anomalies
217	Q755	Other congenital anomalies
217	Q758	Other congenital anomalies
217	Q759	Other congenital anomalies
217	Q760	Other congenital anomalies
217	Q761	Other congenital anomalies
217	Q762	Other congenital anomalies
217	Q763	Other congenital anomalies
217	Q76411	Other congenital anomalies
217	Q76412	Other congenital anomalies
217	Q76413	Other congenital anomalies
217	Q76414	Other congenital anomalies
217	Q76415	Other congenital anomalies
217	Q76419	Other congenital anomalies
217	Q76425	Other congenital anomalies

217	Q76426	Other congenital anomalies
217	Q76427	Other congenital anomalies
217	Q76428	Other congenital anomalies
217	Q76429	Other congenital anomalies
217	Q7649	Other congenital anomalies
217	Q765	Other congenital anomalies
217	Q766	Other congenital anomalies
217	Q767	Other congenital anomalies
217	Q768	Other congenital anomalies
217	Q769	Other congenital anomalies
217	Q770	Other congenital anomalies
217	Q771	Other congenital anomalies
217	Q772	Other congenital anomalies
217	Q773	Other congenital anomalies
217	Q774	Other congenital anomalies
217	Q775	Other congenital anomalies
217	Q776	Other congenital anomalies
217	Q777	Other congenital anomalies
217	Q778	Other congenital anomalies
217	Q779	Other congenital anomalies
217	Q780	Other congenital anomalies
217	Q781	Other congenital anomalies
217	Q782	Other congenital anomalies
217	Q783	Other congenital anomalies
217	Q784	Other congenital anomalies
217	Q785	Other congenital anomalies
217	Q786	Other congenital anomalies
217	Q788	Other congenital anomalies
217	Q789	Other congenital anomalies
217	Q790	Other congenital anomalies
217	Q791	Other congenital anomalies
217	Q792	Other congenital anomalies
217	Q793	Other congenital anomalies
217	Q794	Other congenital anomalies
217	Q7951	Other congenital anomalies
217	Q7959	Other congenital anomalies
217	Q796	Other congenital anomalies
217	Q798	Other congenital anomalies
217	Q799	Other congenital anomalies
217	Q800	Other congenital anomalies
217	Q801	Other congenital anomalies
217	Q802	Other congenital anomalies
217	Q803	Other congenital anomalies
217	Q804	Other congenital anomalies
217	Q808	Other congenital anomalies
217	Q809	Other congenital anomalies
217	Q810	Other congenital anomalies
217	Q811	Other congenital anomalies
217	Q812	Other congenital anomalies
217	Q818	Other congenital anomalies
217	Q819	Other congenital anomalies

217	Q820	Other congenital anomalies
217	Q821	Other congenital anomalies
217	Q822	Other congenital anomalies
217	Q823	Other congenital anomalies
217	Q824	Other congenital anomalies
217	Q825	Other congenital anomalies
217	Q828	Other congenital anomalies
217	Q829	Other congenital anomalies
217	Q830	Other congenital anomalies
217	Q831	Other congenital anomalies
217	Q832	Other congenital anomalies
217	Q833	Other congenital anomalies
217	Q838	Other congenital anomalies
217	Q839	Other congenital anomalies
217	Q840	Other congenital anomalies
217	Q841	Other congenital anomalies
217	Q842	Other congenital anomalies
217	Q843	Other congenital anomalies
217	Q844	Other congenital anomalies
217	Q845	Other congenital anomalies
217	Q846	Other congenital anomalies
217	Q848	Other congenital anomalies
217	Q849	Other congenital anomalies
217	Q851	Other congenital anomalies
217	Q858	Other congenital anomalies
217	Q859	Other congenital anomalies
217	Q870	Other congenital anomalies
217	Q871	Other congenital anomalies
217	Q872	Other congenital anomalies
217	Q873	Other congenital anomalies
217	Q8740	Other congenital anomalies
217	Q87410	Other congenital anomalies
217	Q87418	Other congenital anomalies
217	Q8742	Other congenital anomalies
217	Q8743	Other congenital anomalies
217	Q875	Other congenital anomalies
217	Q8781	Other congenital anomalies
217	Q8789	Other congenital anomalies
217	Q8901	Other congenital anomalies
217	Q8909	Other congenital anomalies
217	Q891	Other congenital anomalies
217	Q892	Other congenital anomalies
217	Q893	Other congenital anomalies
217	Q894	Other congenital anomalies
217	Q897	Other congenital anomalies
217	Q898	Other congenital anomalies
217	Q899	Other congenital anomalies
217	Q900	Other congenital anomalies
217	Q901	Other congenital anomalies
217	Q902	Other congenital anomalies
217	Q909	Other congenital anomalies

217	Q910	Other congenital anomalies
217	Q911	Other congenital anomalies
217	Q912	Other congenital anomalies
217	Q913	Other congenital anomalies
217	Q914	Other congenital anomalies
217	Q915	Other congenital anomalies
217	Q916	Other congenital anomalies
217	Q917	Other congenital anomalies
217	Q920	Other congenital anomalies
217	Q921	Other congenital anomalies
217	Q922	Other congenital anomalies
217	Q925	Other congenital anomalies
217	Q9261	Other congenital anomalies
217	Q9262	Other congenital anomalies
217	Q927	Other congenital anomalies
217	Q928	Other congenital anomalies
217	Q929	Other congenital anomalies
217	Q930	Other congenital anomalies
217	Q931	Other congenital anomalies
217	Q932	Other congenital anomalies
217	Q933	Other congenital anomalies
217	Q934	Other congenital anomalies
217	Q935	Other congenital anomalies
217	Q937	Other congenital anomalies
217	Q9381	Other congenital anomalies
217	Q9388	Other congenital anomalies
217	Q9389	Other congenital anomalies
217	Q939	Other congenital anomalies
217	Q950	Other congenital anomalies
217	Q951	Other congenital anomalies
217	Q952	Other congenital anomalies
217	Q953	Other congenital anomalies
217	Q955	Other congenital anomalies
217	Q958	Other congenital anomalies
217	Q959	Other congenital anomalies
217	Q960	Other congenital anomalies
217	Q961	Other congenital anomalies
217	Q962	Other congenital anomalies
217	Q963	Other congenital anomalies
217	Q964	Other congenital anomalies
217	Q968	Other congenital anomalies
217	Q969	Other congenital anomalies
217	Q970	Other congenital anomalies
217	Q971	Other congenital anomalies
217	Q972	Other congenital anomalies
217	Q973	Other congenital anomalies
217	Q978	Other congenital anomalies
217	Q979	Other congenital anomalies
217	Q980	Other congenital anomalies
217	Q981	Other congenital anomalies
217	Q983	Other congenital anomalies

217	Q984	Other congenital anomalies
217	Q985	Other congenital anomalies
217	Q986	Other congenital anomalies
217	Q987	Other congenital anomalies
217	Q988	Other congenital anomalies
217	Q989	Other congenital anomalies
217	Q990	Other congenital anomalies
217	Q991	Other congenital anomalies
217	Q992	Other congenital anomalies
217	Q998	Other congenital anomalies
217	Q999	Other congenital anomalies
217	Z87720	Other congenital anomalies
217	Z87721	Other congenital anomalies
217	Z87730	Other congenital anomalies
217	Z8775	Other congenital anomalies
217	Z8776	Other congenital anomalies
217	Z87790	Other congenital anomalies
217	Z87798	Other congenital anomalies
218	Z3800	Liveborn
218	Z3801	Liveborn
218	Z381	Liveborn
218	Z382	Liveborn
218	Z3830	Liveborn
218	Z3831	Liveborn
218	Z384	Liveborn
218	Z385	Liveborn
218	Z3861	Liveborn
218	Z3862	Liveborn
218	Z3863	Liveborn
218	Z3864	Liveborn
218	Z3865	Liveborn
218	Z3866	Liveborn
218	Z3868	Liveborn
218	Z3869	Liveborn
218	Z387	Liveborn
218	Z388	Liveborn
219	P0500	Short gestation; low birth weight; and fetal growth retardation
219	P0501	Short gestation; low birth weight; and fetal growth retardation
219	P0502	Short gestation; low birth weight; and fetal growth retardation
219	P0503	Short gestation; low birth weight; and fetal growth retardation
219	P0504	Short gestation; low birth weight; and fetal growth retardation
219	P0505	Short gestation; low birth weight; and fetal growth retardation
219	P0506	Short gestation; low birth weight; and fetal growth retardation
219	P0507	Short gestation; low birth weight; and fetal growth retardation
219	P0508	Short gestation; low birth weight; and fetal growth retardation
219	P0510	Short gestation; low birth weight; and fetal growth retardation
219	P0511	Short gestation; low birth weight; and fetal growth retardation
219	P0512	Short gestation; low birth weight; and fetal growth retardation
219	P0513	Short gestation; low birth weight; and fetal growth retardation
219	P0514	Short gestation; low birth weight; and fetal growth retardation
219	P0515	Short gestation; low birth weight; and fetal growth retardation

219	P0516	Short gestation; low birth weight; and fetal growth retardation
219	P0517	Short gestation; low birth weight; and fetal growth retardation
219	P0518	Short gestation; low birth weight; and fetal growth retardation
219	P052	Short gestation; low birth weight; and fetal growth retardation
219	P059	Short gestation; low birth weight; and fetal growth retardation
219	P0700	Short gestation; low birth weight; and fetal growth retardation
219	P0701	Short gestation; low birth weight; and fetal growth retardation
219	P0702	Short gestation; low birth weight; and fetal growth retardation
219	P0703	Short gestation; low birth weight; and fetal growth retardation
219	P0710	Short gestation; low birth weight; and fetal growth retardation
219	P0714	Short gestation; low birth weight; and fetal growth retardation
219	P0715	Short gestation; low birth weight; and fetal growth retardation
219	P0716	Short gestation; low birth weight; and fetal growth retardation
219	P0717	Short gestation; low birth weight; and fetal growth retardation
219	P0718	Short gestation; low birth weight; and fetal growth retardation
219	P0720	Short gestation; low birth weight; and fetal growth retardation
219	P0721	Short gestation; low birth weight; and fetal growth retardation
219	P0722	Short gestation; low birth weight; and fetal growth retardation
219	P0723	Short gestation; low birth weight; and fetal growth retardation
219	P0724	Short gestation; low birth weight; and fetal growth retardation
219	P0725	Short gestation; low birth weight; and fetal growth retardation
219	P0726	Short gestation; low birth weight; and fetal growth retardation
219	P0730	Short gestation; low birth weight; and fetal growth retardation
219	P0731	Short gestation; low birth weight; and fetal growth retardation
219	P0732	Short gestation; low birth weight; and fetal growth retardation
219	P0733	Short gestation; low birth weight; and fetal growth retardation
219	P0734	Short gestation; low birth weight; and fetal growth retardation
219	P0735	Short gestation; low birth weight; and fetal growth retardation
219	P0736	Short gestation; low birth weight; and fetal growth retardation
219	P0737	Short gestation; low birth weight; and fetal growth retardation
219	P0738	Short gestation; low birth weight; and fetal growth retardation
219	P0739	Short gestation; low birth weight; and fetal growth retardation
220	P190	Intrauterine hypoxia and birth asphyxia
220	P191	Intrauterine hypoxia and birth asphyxia
220	P192	Intrauterine hypoxia and birth asphyxia
220	P199	Intrauterine hypoxia and birth asphyxia
220	P9160	Intrauterine hypoxia and birth asphyxia
220	P9161	Intrauterine hypoxia and birth asphyxia
220	P9162	Intrauterine hypoxia and birth asphyxia
220	P9163	Intrauterine hypoxia and birth asphyxia
221	P220	Respiratory distress syndrome
222	P550	Hemolytic jaundice and perinatal jaundice
222	P551	Hemolytic jaundice and perinatal jaundice
222	P558	Hemolytic jaundice and perinatal jaundice
222	P559	Hemolytic jaundice and perinatal jaundice
222	P560	Hemolytic jaundice and perinatal jaundice
222	P5690	Hemolytic jaundice and perinatal jaundice
222	P5699	Hemolytic jaundice and perinatal jaundice
222	P570	Hemolytic jaundice and perinatal jaundice
222	P578	Hemolytic jaundice and perinatal jaundice
222	P579	Hemolytic jaundice and perinatal jaundice

222	P580	Hemolytic jaundice and perinatal jaundice
222	P581	Hemolytic jaundice and perinatal jaundice
222	P582	Hemolytic jaundice and perinatal jaundice
222	P583	Hemolytic jaundice and perinatal jaundice
222	P5841	Hemolytic jaundice and perinatal jaundice
222	P5842	Hemolytic jaundice and perinatal jaundice
222	P585	Hemolytic jaundice and perinatal jaundice
222	P588	Hemolytic jaundice and perinatal jaundice
222	P589	Hemolytic jaundice and perinatal jaundice
222	P590	Hemolytic jaundice and perinatal jaundice
222	P591	Hemolytic jaundice and perinatal jaundice
222	P5920	Hemolytic jaundice and perinatal jaundice
222	P5929	Hemolytic jaundice and perinatal jaundice
222	P593	Hemolytic jaundice and perinatal jaundice
222	P598	Hemolytic jaundice and perinatal jaundice
222	P599	Hemolytic jaundice and perinatal jaundice
223	P100	Birth trauma
223	P101	Birth trauma
223	P102	Birth trauma
223	P103	Birth trauma
223	P104	Birth trauma
223	P108	Birth trauma
223	P109	Birth trauma
223	P110	Birth trauma
223	P111	Birth trauma
223	P112	Birth trauma
223	P113	Birth trauma
223	P114	Birth trauma
223	P115	Birth trauma
223	P119	Birth trauma
223	P120	Birth trauma
223	P121	Birth trauma
223	P122	Birth trauma
223	P123	Birth trauma
223	P124	Birth trauma
223	P1281	Birth trauma
223	P1289	Birth trauma
223	P129	Birth trauma
223	P130	Birth trauma
223	P131	Birth trauma
223	P132	Birth trauma
223	P133	Birth trauma
223	P134	Birth trauma
223	P138	Birth trauma
223	P139	Birth trauma
223	P140	Birth trauma
223	P141	Birth trauma
223	P142	Birth trauma
223	P143	Birth trauma
223	P148	Birth trauma
223	P149	Birth trauma

223	P150	Birth trauma
223	P151	Birth trauma
223	P152	Birth trauma
223	P153	Birth trauma
223	P154	Birth trauma
223	P155	Birth trauma
223	P156	Birth trauma
223	P158	Birth trauma
223	P159	Birth trauma
224	A33	Other perinatal conditions
224	A4851	Other perinatal conditions
224	P000	Other perinatal conditions
224	P001	Other perinatal conditions
224	P002	Other perinatal conditions
224	P003	Other perinatal conditions
224	P004	Other perinatal conditions
224	P005	Other perinatal conditions
224	P006	Other perinatal conditions
224	P007	Other perinatal conditions
224	P0081	Other perinatal conditions
224	P0089	Other perinatal conditions
224	P009	Other perinatal conditions
224	P010	Other perinatal conditions
224	P011	Other perinatal conditions
224	P012	Other perinatal conditions
224	P013	Other perinatal conditions
224	P014	Other perinatal conditions
224	P015	Other perinatal conditions
224	P016	Other perinatal conditions
224	P017	Other perinatal conditions
224	P018	Other perinatal conditions
224	P019	Other perinatal conditions
224	P020	Other perinatal conditions
224	P021	Other perinatal conditions
224	P0220	Other perinatal conditions
224	P0229	Other perinatal conditions
224	P023	Other perinatal conditions
224	P024	Other perinatal conditions
224	P025	Other perinatal conditions
224	P0260	Other perinatal conditions
224	P0269	Other perinatal conditions
224	P027	Other perinatal conditions
224	P028	Other perinatal conditions
224	P029	Other perinatal conditions
224	P030	Other perinatal conditions
224	P031	Other perinatal conditions
224	P032	Other perinatal conditions
224	P033	Other perinatal conditions
224	P034	Other perinatal conditions
224	P035	Other perinatal conditions
224	P036	Other perinatal conditions

224	P03810	Other perinatal conditions
224	P03811	Other perinatal conditions
224	P03819	Other perinatal conditions
224	P0382	Other perinatal conditions
224	P0389	Other perinatal conditions
224	P039	Other perinatal conditions
224	P040	Other perinatal conditions
224	P041	Other perinatal conditions
224	P042	Other perinatal conditions
224	P045	Other perinatal conditions
224	P046	Other perinatal conditions
224	P048	Other perinatal conditions
224	P049	Other perinatal conditions
224	P080	Other perinatal conditions
224	P081	Other perinatal conditions
224	P0821	Other perinatal conditions
224	P0822	Other perinatal conditions
224	P09	Other perinatal conditions
224	P221	Other perinatal conditions
224	P228	Other perinatal conditions
224	P229	Other perinatal conditions
224	P230	Other perinatal conditions
224	P231	Other perinatal conditions
224	P232	Other perinatal conditions
224	P233	Other perinatal conditions
224	P234	Other perinatal conditions
224	P235	Other perinatal conditions
224	P236	Other perinatal conditions
224	P238	Other perinatal conditions
224	P239	Other perinatal conditions
224	P2400	Other perinatal conditions
224	P2401	Other perinatal conditions
224	P2410	Other perinatal conditions
224	P2411	Other perinatal conditions
224	P2420	Other perinatal conditions
224	P2421	Other perinatal conditions
224	P2430	Other perinatal conditions
224	P2431	Other perinatal conditions
224	P2480	Other perinatal conditions
224	P2481	Other perinatal conditions
224	P249	Other perinatal conditions
224	P250	Other perinatal conditions
224	P251	Other perinatal conditions
224	P252	Other perinatal conditions
224	P253	Other perinatal conditions
224	P258	Other perinatal conditions
224	P260	Other perinatal conditions
224	P261	Other perinatal conditions
224	P268	Other perinatal conditions
224	P269	Other perinatal conditions
224	P270	Other perinatal conditions

224	P271	Other perinatal conditions
224	P278	Other perinatal conditions
224	P279	Other perinatal conditions
224	P280	Other perinatal conditions
224	P2810	Other perinatal conditions
224	P2811	Other perinatal conditions
224	P2819	Other perinatal conditions
224	P282	Other perinatal conditions
224	P283	Other perinatal conditions
224	P284	Other perinatal conditions
224	P285	Other perinatal conditions
224	P2881	Other perinatal conditions
224	P2889	Other perinatal conditions
224	P289	Other perinatal conditions
224	P290	Other perinatal conditions
224	P2911	Other perinatal conditions
224	P2912	Other perinatal conditions
224	P292	Other perinatal conditions
224	P294	Other perinatal conditions
224	P2981	Other perinatal conditions
224	P2989	Other perinatal conditions
224	P299	Other perinatal conditions
224	P350	Other perinatal conditions
224	P351	Other perinatal conditions
224	P352	Other perinatal conditions
224	P353	Other perinatal conditions
224	P358	Other perinatal conditions
224	P359	Other perinatal conditions
224	P370	Other perinatal conditions
224	P371	Other perinatal conditions
224	P372	Other perinatal conditions
224	P373	Other perinatal conditions
224	P374	Other perinatal conditions
224	P375	Other perinatal conditions
224	P378	Other perinatal conditions
224	P379	Other perinatal conditions
224	P381	Other perinatal conditions
224	P389	Other perinatal conditions
224	P390	Other perinatal conditions
224	P391	Other perinatal conditions
224	P392	Other perinatal conditions
224	P393	Other perinatal conditions
224	P394	Other perinatal conditions
224	P398	Other perinatal conditions
224	P399	Other perinatal conditions
224	P500	Other perinatal conditions
224	P501	Other perinatal conditions
224	P502	Other perinatal conditions
224	P503	Other perinatal conditions
224	P504	Other perinatal conditions
224	P505	Other perinatal conditions

224	P508	Other perinatal conditions
224	P509	Other perinatal conditions
224	P510	Other perinatal conditions
224	P518	Other perinatal conditions
224	P519	Other perinatal conditions
224	P520	Other perinatal conditions
224	P521	Other perinatal conditions
224	P5221	Other perinatal conditions
224	P5222	Other perinatal conditions
224	P523	Other perinatal conditions
224	P524	Other perinatal conditions
224	P525	Other perinatal conditions
224	P526	Other perinatal conditions
224	P528	Other perinatal conditions
224	P529	Other perinatal conditions
224	P53	Other perinatal conditions
224	P540	Other perinatal conditions
224	P541	Other perinatal conditions
224	P542	Other perinatal conditions
224	P543	Other perinatal conditions
224	P544	Other perinatal conditions
224	P545	Other perinatal conditions
224	P546	Other perinatal conditions
224	P548	Other perinatal conditions
224	P549	Other perinatal conditions
224	P60	Other perinatal conditions
224	P610	Other perinatal conditions
224	P611	Other perinatal conditions
224	P612	Other perinatal conditions
224	P613	Other perinatal conditions
224	P614	Other perinatal conditions
224	P615	Other perinatal conditions
224	P616	Other perinatal conditions
224	P618	Other perinatal conditions
224	P619	Other perinatal conditions
224	P700	Other perinatal conditions
224	P701	Other perinatal conditions
224	P702	Other perinatal conditions
224	P703	Other perinatal conditions
224	P704	Other perinatal conditions
224	P708	Other perinatal conditions
224	P709	Other perinatal conditions
224	P710	Other perinatal conditions
224	P711	Other perinatal conditions
224	P712	Other perinatal conditions
224	P713	Other perinatal conditions
224	P714	Other perinatal conditions
224	P718	Other perinatal conditions
224	P719	Other perinatal conditions
224	P720	Other perinatal conditions
224	P721	Other perinatal conditions

224	P722	Other perinatal conditions
224	P728	Other perinatal conditions
224	P729	Other perinatal conditions
224	P740	Other perinatal conditions
224	P741	Other perinatal conditions
224	P742	Other perinatal conditions
224	P743	Other perinatal conditions
224	P744	Other perinatal conditions
224	P745	Other perinatal conditions
224	P746	Other perinatal conditions
224	P748	Other perinatal conditions
224	P749	Other perinatal conditions
224	P760	Other perinatal conditions
224	P761	Other perinatal conditions
224	P762	Other perinatal conditions
224	P768	Other perinatal conditions
224	P769	Other perinatal conditions
224	P771	Other perinatal conditions
224	P772	Other perinatal conditions
224	P773	Other perinatal conditions
224	P779	Other perinatal conditions
224	P780	Other perinatal conditions
224	P781	Other perinatal conditions
224	P782	Other perinatal conditions
224	P783	Other perinatal conditions
224	P7881	Other perinatal conditions
224	P7882	Other perinatal conditions
224	P7883	Other perinatal conditions
224	P7889	Other perinatal conditions
224	P789	Other perinatal conditions
224	P800	Other perinatal conditions
224	P808	Other perinatal conditions
224	P809	Other perinatal conditions
224	P810	Other perinatal conditions
224	P818	Other perinatal conditions
224	P819	Other perinatal conditions
224	P830	Other perinatal conditions
224	P831	Other perinatal conditions
224	P832	Other perinatal conditions
224	P8330	Other perinatal conditions
224	P8339	Other perinatal conditions
224	P834	Other perinatal conditions
224	P835	Other perinatal conditions
224	P836	Other perinatal conditions
224	P838	Other perinatal conditions
224	P839	Other perinatal conditions
224	P84	Other perinatal conditions
224	P90	Other perinatal conditions
224	P910	Other perinatal conditions
224	P911	Other perinatal conditions
224	P912	Other perinatal conditions

224	P913	Other perinatal conditions
224	P914	Other perinatal conditions
224	P915	Other perinatal conditions
224	P918	Other perinatal conditions
224	P919	Other perinatal conditions
224	P9201	Other perinatal conditions
224	P9209	Other perinatal conditions
224	P921	Other perinatal conditions
224	P922	Other perinatal conditions
224	P923	Other perinatal conditions
224	P924	Other perinatal conditions
224	P925	Other perinatal conditions
224	P926	Other perinatal conditions
224	P928	Other perinatal conditions
224	P929	Other perinatal conditions
224	P930	Other perinatal conditions
224	P938	Other perinatal conditions
224	P940	Other perinatal conditions
224	P941	Other perinatal conditions
224	P942	Other perinatal conditions
224	P948	Other perinatal conditions
224	P949	Other perinatal conditions
224	P95	Other perinatal conditions
224	P960	Other perinatal conditions
224	P963	Other perinatal conditions
224	P965	Other perinatal conditions
224	P9681	Other perinatal conditions
224	P9682	Other perinatal conditions
224	P9683	Other perinatal conditions
224	P9689	Other perinatal conditions
224	P969	Other perinatal conditions
224	Q861	Other perinatal conditions
224	Q862	Other perinatal conditions
224	Q868	Other perinatal conditions
224	R1083	Other perinatal conditions
224	R6811	Other perinatal conditions
224	R6812	Other perinatal conditions
224	R6813	Other perinatal conditions
224	R6819	Other perinatal conditions
224	Z412	Other perinatal conditions
225	M1250	Joint disorders and dislocations; trauma-related
225	M12511	Joint disorders and dislocations; trauma-related
225	M12512	Joint disorders and dislocations; trauma-related
225	M12519	Joint disorders and dislocations; trauma-related
225	M12521	Joint disorders and dislocations; trauma-related
225	M12522	Joint disorders and dislocations; trauma-related
225	M12529	Joint disorders and dislocations; trauma-related
225	M12531	Joint disorders and dislocations; trauma-related
225	M12532	Joint disorders and dislocations; trauma-related
225	M12539	Joint disorders and dislocations; trauma-related
225	M12541	Joint disorders and dislocations; trauma-related

225	M12542	Joint disorders and dislocations; trauma-related
225	M12549	Joint disorders and dislocations; trauma-related
225	M12551	Joint disorders and dislocations; trauma-related
225	M12552	Joint disorders and dislocations; trauma-related
225	M12559	Joint disorders and dislocations; trauma-related
225	M12561	Joint disorders and dislocations; trauma-related
225	M12562	Joint disorders and dislocations; trauma-related
225	M12569	Joint disorders and dislocations; trauma-related
225	M12571	Joint disorders and dislocations; trauma-related
225	M12572	Joint disorders and dislocations; trauma-related
225	M12579	Joint disorders and dislocations; trauma-related
225	M1258	Joint disorders and dislocations; trauma-related
225	M1259	Joint disorders and dislocations; trauma-related
225	M2200	Joint disorders and dislocations; trauma-related
225	M2201	Joint disorders and dislocations; trauma-related
225	M2202	Joint disorders and dislocations; trauma-related
225	M2210	Joint disorders and dislocations; trauma-related
225	M2211	Joint disorders and dislocations; trauma-related
225	M2212	Joint disorders and dislocations; trauma-related
225	M222X1	Joint disorders and dislocations; trauma-related
225	M222X2	Joint disorders and dislocations; trauma-related
225	M222X9	Joint disorders and dislocations; trauma-related
225	M223X1	Joint disorders and dislocations; trauma-related
225	M223X2	Joint disorders and dislocations; trauma-related
225	M223X9	Joint disorders and dislocations; trauma-related
225	M2240	Joint disorders and dislocations; trauma-related
225	M2241	Joint disorders and dislocations; trauma-related
225	M2242	Joint disorders and dislocations; trauma-related
225	M228X1	Joint disorders and dislocations; trauma-related
225	M228X2	Joint disorders and dislocations; trauma-related
225	M228X9	Joint disorders and dislocations; trauma-related
225	M2290	Joint disorders and dislocations; trauma-related
225	M2291	Joint disorders and dislocations; trauma-related
225	M2292	Joint disorders and dislocations; trauma-related
225	M23000	Joint disorders and dislocations; trauma-related
225	M23001	Joint disorders and dislocations; trauma-related
225	M23002	Joint disorders and dislocations; trauma-related
225	M23003	Joint disorders and dislocations; trauma-related
225	M23004	Joint disorders and dislocations; trauma-related
225	M23005	Joint disorders and dislocations; trauma-related
225	M23006	Joint disorders and dislocations; trauma-related
225	M23007	Joint disorders and dislocations; trauma-related
225	M23009	Joint disorders and dislocations; trauma-related
225	M23011	Joint disorders and dislocations; trauma-related
225	M23012	Joint disorders and dislocations; trauma-related
225	M23019	Joint disorders and dislocations; trauma-related
225	M23021	Joint disorders and dislocations; trauma-related
225	M23022	Joint disorders and dislocations; trauma-related
225	M23029	Joint disorders and dislocations; trauma-related
225	M23031	Joint disorders and dislocations; trauma-related
225	M23032	Joint disorders and dislocations; trauma-related

225	M23039	Joint disorders and dislocations; trauma-related
225	M23041	Joint disorders and dislocations; trauma-related
225	M23042	Joint disorders and dislocations; trauma-related
225	M23049	Joint disorders and dislocations; trauma-related
225	M23051	Joint disorders and dislocations; trauma-related
225	M23052	Joint disorders and dislocations; trauma-related
225	M23059	Joint disorders and dislocations; trauma-related
225	M23061	Joint disorders and dislocations; trauma-related
225	M23062	Joint disorders and dislocations; trauma-related
225	M23069	Joint disorders and dislocations; trauma-related
225	M23200	Joint disorders and dislocations; trauma-related
225	M23201	Joint disorders and dislocations; trauma-related
225	M23202	Joint disorders and dislocations; trauma-related
225	M23203	Joint disorders and dislocations; trauma-related
225	M23204	Joint disorders and dislocations; trauma-related
225	M23205	Joint disorders and dislocations; trauma-related
225	M23206	Joint disorders and dislocations; trauma-related
225	M23207	Joint disorders and dislocations; trauma-related
225	M23209	Joint disorders and dislocations; trauma-related
225	M23211	Joint disorders and dislocations; trauma-related
225	M23212	Joint disorders and dislocations; trauma-related
225	M23219	Joint disorders and dislocations; trauma-related
225	M23221	Joint disorders and dislocations; trauma-related
225	M23222	Joint disorders and dislocations; trauma-related
225	M23229	Joint disorders and dislocations; trauma-related
225	M23231	Joint disorders and dislocations; trauma-related
225	M23232	Joint disorders and dislocations; trauma-related
225	M23239	Joint disorders and dislocations; trauma-related
225	M23241	Joint disorders and dislocations; trauma-related
225	M23242	Joint disorders and dislocations; trauma-related
225	M23249	Joint disorders and dislocations; trauma-related
225	M23251	Joint disorders and dislocations; trauma-related
225	M23252	Joint disorders and dislocations; trauma-related
225	M23259	Joint disorders and dislocations; trauma-related
225	M23261	Joint disorders and dislocations; trauma-related
225	M23262	Joint disorders and dislocations; trauma-related
225	M23269	Joint disorders and dislocations; trauma-related
225	M23300	Joint disorders and dislocations; trauma-related
225	M23301	Joint disorders and dislocations; trauma-related
225	M23302	Joint disorders and dislocations; trauma-related
225	M23303	Joint disorders and dislocations; trauma-related
225	M23304	Joint disorders and dislocations; trauma-related
225	M23305	Joint disorders and dislocations; trauma-related
225	M23306	Joint disorders and dislocations; trauma-related
225	M23307	Joint disorders and dislocations; trauma-related
225	M23309	Joint disorders and dislocations; trauma-related
225	M23311	Joint disorders and dislocations; trauma-related
225	M23312	Joint disorders and dislocations; trauma-related
225	M23319	Joint disorders and dislocations; trauma-related
225	M23321	Joint disorders and dislocations; trauma-related
225	M23322	Joint disorders and dislocations; trauma-related

225	M23329	Joint disorders and dislocations; trauma-related
225	M23331	Joint disorders and dislocations; trauma-related
225	M23332	Joint disorders and dislocations; trauma-related
225	M23339	Joint disorders and dislocations; trauma-related
225	M23341	Joint disorders and dislocations; trauma-related
225	M23342	Joint disorders and dislocations; trauma-related
225	M23349	Joint disorders and dislocations; trauma-related
225	M23351	Joint disorders and dislocations; trauma-related
225	M23352	Joint disorders and dislocations; trauma-related
225	M23359	Joint disorders and dislocations; trauma-related
225	M23361	Joint disorders and dislocations; trauma-related
225	M23362	Joint disorders and dislocations; trauma-related
225	M23369	Joint disorders and dislocations; trauma-related
225	M2340	Joint disorders and dislocations; trauma-related
225	M2341	Joint disorders and dislocations; trauma-related
225	M2342	Joint disorders and dislocations; trauma-related
225	M2350	Joint disorders and dislocations; trauma-related
225	M2351	Joint disorders and dislocations; trauma-related
225	M2352	Joint disorders and dislocations; trauma-related
225	M23601	Joint disorders and dislocations; trauma-related
225	M23602	Joint disorders and dislocations; trauma-related
225	M23609	Joint disorders and dislocations; trauma-related
225	M23611	Joint disorders and dislocations; trauma-related
225	M23612	Joint disorders and dislocations; trauma-related
225	M23619	Joint disorders and dislocations; trauma-related
225	M23621	Joint disorders and dislocations; trauma-related
225	M23622	Joint disorders and dislocations; trauma-related
225	M23629	Joint disorders and dislocations; trauma-related
225	M23631	Joint disorders and dislocations; trauma-related
225	M23632	Joint disorders and dislocations; trauma-related
225	M23639	Joint disorders and dislocations; trauma-related
225	M23641	Joint disorders and dislocations; trauma-related
225	M23642	Joint disorders and dislocations; trauma-related
225	M23649	Joint disorders and dislocations; trauma-related
225	M23671	Joint disorders and dislocations; trauma-related
225	M23672	Joint disorders and dislocations; trauma-related
225	M23679	Joint disorders and dislocations; trauma-related
225	M238X1	Joint disorders and dislocations; trauma-related
225	M238X2	Joint disorders and dislocations; trauma-related
225	M238X9	Joint disorders and dislocations; trauma-related
225	M2390	Joint disorders and dislocations; trauma-related
225	M2391	Joint disorders and dislocations; trauma-related
225	M2392	Joint disorders and dislocations; trauma-related
225	M2410	Joint disorders and dislocations; trauma-related
225	M24111	Joint disorders and dislocations; trauma-related
225	M24112	Joint disorders and dislocations; trauma-related
225	M24119	Joint disorders and dislocations; trauma-related
225	M24121	Joint disorders and dislocations; trauma-related
225	M24122	Joint disorders and dislocations; trauma-related
225	M24129	Joint disorders and dislocations; trauma-related
225	M24131	Joint disorders and dislocations; trauma-related

225	M24132	Joint disorders and dislocations; trauma-related
225	M24139	Joint disorders and dislocations; trauma-related
225	M24141	Joint disorders and dislocations; trauma-related
225	M24142	Joint disorders and dislocations; trauma-related
225	M24149	Joint disorders and dislocations; trauma-related
225	M24151	Joint disorders and dislocations; trauma-related
225	M24152	Joint disorders and dislocations; trauma-related
225	M24159	Joint disorders and dislocations; trauma-related
225	M24171	Joint disorders and dislocations; trauma-related
225	M24172	Joint disorders and dislocations; trauma-related
225	M24173	Joint disorders and dislocations; trauma-related
225	M24174	Joint disorders and dislocations; trauma-related
225	M24175	Joint disorders and dislocations; trauma-related
225	M24176	Joint disorders and dislocations; trauma-related
225	M2440	Joint disorders and dislocations; trauma-related
225	M24411	Joint disorders and dislocations; trauma-related
225	M24412	Joint disorders and dislocations; trauma-related
225	M24419	Joint disorders and dislocations; trauma-related
225	M24421	Joint disorders and dislocations; trauma-related
225	M24422	Joint disorders and dislocations; trauma-related
225	M24429	Joint disorders and dislocations; trauma-related
225	M24431	Joint disorders and dislocations; trauma-related
225	M24432	Joint disorders and dislocations; trauma-related
225	M24439	Joint disorders and dislocations; trauma-related
225	M24441	Joint disorders and dislocations; trauma-related
225	M24442	Joint disorders and dislocations; trauma-related
225	M24443	Joint disorders and dislocations; trauma-related
225	M24444	Joint disorders and dislocations; trauma-related
225	M24445	Joint disorders and dislocations; trauma-related
225	M24446	Joint disorders and dislocations; trauma-related
225	M24451	Joint disorders and dislocations; trauma-related
225	M24452	Joint disorders and dislocations; trauma-related
225	M24459	Joint disorders and dislocations; trauma-related
225	M24461	Joint disorders and dislocations; trauma-related
225	M24462	Joint disorders and dislocations; trauma-related
225	M24469	Joint disorders and dislocations; trauma-related
225	M24471	Joint disorders and dislocations; trauma-related
225	M24472	Joint disorders and dislocations; trauma-related
225	M24473	Joint disorders and dislocations; trauma-related
225	M24474	Joint disorders and dislocations; trauma-related
225	M24475	Joint disorders and dislocations; trauma-related
225	M24476	Joint disorders and dislocations; trauma-related
225	M24477	Joint disorders and dislocations; trauma-related
225	M24478	Joint disorders and dislocations; trauma-related
225	M24479	Joint disorders and dislocations; trauma-related
225	M433	Joint disorders and dislocations; trauma-related
225	M434	Joint disorders and dislocations; trauma-related
225	M435X2	Joint disorders and dislocations; trauma-related
225	M435X3	Joint disorders and dislocations; trauma-related
225	M435X4	Joint disorders and dislocations; trauma-related
225	M435X5	Joint disorders and dislocations; trauma-related

225	M435X6	Joint disorders and dislocations; trauma-related
225	M435X7	Joint disorders and dislocations; trauma-related
225	M435X8	Joint disorders and dislocations; trauma-related
225	M435X9	Joint disorders and dislocations; trauma-related
225	M9910	Joint disorders and dislocations; trauma-related
225	M9911	Joint disorders and dislocations; trauma-related
225	M9912	Joint disorders and dislocations; trauma-related
225	M9913	Joint disorders and dislocations; trauma-related
225	M9914	Joint disorders and dislocations; trauma-related
225	M9915	Joint disorders and dislocations; trauma-related
225	M9916	Joint disorders and dislocations; trauma-related
225	M9917	Joint disorders and dislocations; trauma-related
225	M9918	Joint disorders and dislocations; trauma-related
225	M9919	Joint disorders and dislocations; trauma-related
225	S030XXA	Joint disorders and dislocations; trauma-related
225	S030XXD	Joint disorders and dislocations; trauma-related
225	S030XXS	Joint disorders and dislocations; trauma-related
225	S031XXS	Joint disorders and dislocations; trauma-related
225	S130XXA	Joint disorders and dislocations; trauma-related
225	S130XXD	Joint disorders and dislocations; trauma-related
225	S130XXS	Joint disorders and dislocations; trauma-related
225	S13100A	Joint disorders and dislocations; trauma-related
225	S13100D	Joint disorders and dislocations; trauma-related
225	S13100S	Joint disorders and dislocations; trauma-related
225	S13101A	Joint disorders and dislocations; trauma-related
225	S13101D	Joint disorders and dislocations; trauma-related
225	S13101S	Joint disorders and dislocations; trauma-related
225	S13110A	Joint disorders and dislocations; trauma-related
225	S13110D	Joint disorders and dislocations; trauma-related
225	S13110S	Joint disorders and dislocations; trauma-related
225	S13111A	Joint disorders and dislocations; trauma-related
225	S13111D	Joint disorders and dislocations; trauma-related
225	S13111S	Joint disorders and dislocations; trauma-related
225	S13120A	Joint disorders and dislocations; trauma-related
225	S13120D	Joint disorders and dislocations; trauma-related
225	S13120S	Joint disorders and dislocations; trauma-related
225	S13121A	Joint disorders and dislocations; trauma-related
225	S13121D	Joint disorders and dislocations; trauma-related
225	S13121S	Joint disorders and dislocations; trauma-related
225	S13130A	Joint disorders and dislocations; trauma-related
225	S13130D	Joint disorders and dislocations; trauma-related
225	S13130S	Joint disorders and dislocations; trauma-related
225	S13131A	Joint disorders and dislocations; trauma-related
225	S13131D	Joint disorders and dislocations; trauma-related
225	S13131S	Joint disorders and dislocations; trauma-related
225	S13140A	Joint disorders and dislocations; trauma-related
225	S13140D	Joint disorders and dislocations; trauma-related
225	S13140S	Joint disorders and dislocations; trauma-related
225	S13141A	Joint disorders and dislocations; trauma-related
225	S13141D	Joint disorders and dislocations; trauma-related
225	S13141S	Joint disorders and dislocations; trauma-related

225	S13150A	Joint disorders and dislocations; trauma-related
225	S13150D	Joint disorders and dislocations; trauma-related
225	S13150S	Joint disorders and dislocations; trauma-related
225	S13151A	Joint disorders and dislocations; trauma-related
225	S13151D	Joint disorders and dislocations; trauma-related
225	S13151S	Joint disorders and dislocations; trauma-related
225	S13160A	Joint disorders and dislocations; trauma-related
225	S13160D	Joint disorders and dislocations; trauma-related
225	S13160S	Joint disorders and dislocations; trauma-related
225	S13161A	Joint disorders and dislocations; trauma-related
225	S13161D	Joint disorders and dislocations; trauma-related
225	S13161S	Joint disorders and dislocations; trauma-related
225	S13170A	Joint disorders and dislocations; trauma-related
225	S13170D	Joint disorders and dislocations; trauma-related
225	S13170S	Joint disorders and dislocations; trauma-related
225	S13171A	Joint disorders and dislocations; trauma-related
225	S13171D	Joint disorders and dislocations; trauma-related
225	S13171S	Joint disorders and dislocations; trauma-related
225	S13180A	Joint disorders and dislocations; trauma-related
225	S13180D	Joint disorders and dislocations; trauma-related
225	S13180S	Joint disorders and dislocations; trauma-related
225	S13181A	Joint disorders and dislocations; trauma-related
225	S13181D	Joint disorders and dislocations; trauma-related
225	S13181S	Joint disorders and dislocations; trauma-related
225	S1320XA	Joint disorders and dislocations; trauma-related
225	S1320XD	Joint disorders and dislocations; trauma-related
225	S1320XS	Joint disorders and dislocations; trauma-related
225	S1329XA	Joint disorders and dislocations; trauma-related
225	S1329XD	Joint disorders and dislocations; trauma-related
225	S1329XS	Joint disorders and dislocations; trauma-related
225	S230XXA	Joint disorders and dislocations; trauma-related
225	S230XXD	Joint disorders and dislocations; trauma-related
225	S230XXS	Joint disorders and dislocations; trauma-related
225	S23100A	Joint disorders and dislocations; trauma-related
225	S23100D	Joint disorders and dislocations; trauma-related
225	S23100S	Joint disorders and dislocations; trauma-related
225	S23101A	Joint disorders and dislocations; trauma-related
225	S23101D	Joint disorders and dislocations; trauma-related
225	S23101S	Joint disorders and dislocations; trauma-related
225	S23110A	Joint disorders and dislocations; trauma-related
225	S23110D	Joint disorders and dislocations; trauma-related
225	S23110S	Joint disorders and dislocations; trauma-related
225	S23111A	Joint disorders and dislocations; trauma-related
225	S23111D	Joint disorders and dislocations; trauma-related
225	S23111S	Joint disorders and dislocations; trauma-related
225	S23120A	Joint disorders and dislocations; trauma-related
225	S23120D	Joint disorders and dislocations; trauma-related
225	S23120S	Joint disorders and dislocations; trauma-related
225	S23121A	Joint disorders and dislocations; trauma-related
225	S23121D	Joint disorders and dislocations; trauma-related
225	S23121S	Joint disorders and dislocations; trauma-related

225	S23122A	Joint disorders and dislocations; trauma-related
225	S23122D	Joint disorders and dislocations; trauma-related
225	S23122S	Joint disorders and dislocations; trauma-related
225	S23123A	Joint disorders and dislocations; trauma-related
225	S23123D	Joint disorders and dislocations; trauma-related
225	S23123S	Joint disorders and dislocations; trauma-related
225	S23130A	Joint disorders and dislocations; trauma-related
225	S23130D	Joint disorders and dislocations; trauma-related
225	S23130S	Joint disorders and dislocations; trauma-related
225	S23131A	Joint disorders and dislocations; trauma-related
225	S23131D	Joint disorders and dislocations; trauma-related
225	S23131S	Joint disorders and dislocations; trauma-related
225	S23132A	Joint disorders and dislocations; trauma-related
225	S23132D	Joint disorders and dislocations; trauma-related
225	S23132S	Joint disorders and dislocations; trauma-related
225	S23133A	Joint disorders and dislocations; trauma-related
225	S23133D	Joint disorders and dislocations; trauma-related
225	S23133S	Joint disorders and dislocations; trauma-related
225	S23140A	Joint disorders and dislocations; trauma-related
225	S23140D	Joint disorders and dislocations; trauma-related
225	S23140S	Joint disorders and dislocations; trauma-related
225	S23141A	Joint disorders and dislocations; trauma-related
225	S23141D	Joint disorders and dislocations; trauma-related
225	S23141S	Joint disorders and dislocations; trauma-related
225	S23142A	Joint disorders and dislocations; trauma-related
225	S23142D	Joint disorders and dislocations; trauma-related
225	S23142S	Joint disorders and dislocations; trauma-related
225	S23143A	Joint disorders and dislocations; trauma-related
225	S23143D	Joint disorders and dislocations; trauma-related
225	S23143S	Joint disorders and dislocations; trauma-related
225	S23150A	Joint disorders and dislocations; trauma-related
225	S23150D	Joint disorders and dislocations; trauma-related
225	S23150S	Joint disorders and dislocations; trauma-related
225	S23151A	Joint disorders and dislocations; trauma-related
225	S23151D	Joint disorders and dislocations; trauma-related
225	S23151S	Joint disorders and dislocations; trauma-related
225	S23152A	Joint disorders and dislocations; trauma-related
225	S23152D	Joint disorders and dislocations; trauma-related
225	S23152S	Joint disorders and dislocations; trauma-related
225	S23153A	Joint disorders and dislocations; trauma-related
225	S23153D	Joint disorders and dislocations; trauma-related
225	S23153S	Joint disorders and dislocations; trauma-related
225	S23160A	Joint disorders and dislocations; trauma-related
225	S23160D	Joint disorders and dislocations; trauma-related
225	S23160S	Joint disorders and dislocations; trauma-related
225	S23161A	Joint disorders and dislocations; trauma-related
225	S23161D	Joint disorders and dislocations; trauma-related
225	S23161S	Joint disorders and dislocations; trauma-related
225	S23162A	Joint disorders and dislocations; trauma-related
225	S23162D	Joint disorders and dislocations; trauma-related
225	S23162S	Joint disorders and dislocations; trauma-related

225	S23163A	Joint disorders and dislocations; trauma-related
225	S23163D	Joint disorders and dislocations; trauma-related
225	S23163S	Joint disorders and dislocations; trauma-related
225	S23170A	Joint disorders and dislocations; trauma-related
225	S23170D	Joint disorders and dislocations; trauma-related
225	S23170S	Joint disorders and dislocations; trauma-related
225	S23171A	Joint disorders and dislocations; trauma-related
225	S23171D	Joint disorders and dislocations; trauma-related
225	S23171S	Joint disorders and dislocations; trauma-related
225	S2320XA	Joint disorders and dislocations; trauma-related
225	S2320XD	Joint disorders and dislocations; trauma-related
225	S2320XS	Joint disorders and dislocations; trauma-related
225	S2329XA	Joint disorders and dislocations; trauma-related
225	S2329XD	Joint disorders and dislocations; trauma-related
225	S2329XS	Joint disorders and dislocations; trauma-related
225	S330XXA	Joint disorders and dislocations; trauma-related
225	S330XXD	Joint disorders and dislocations; trauma-related
225	S330XXS	Joint disorders and dislocations; trauma-related
225	S33100A	Joint disorders and dislocations; trauma-related
225	S33100D	Joint disorders and dislocations; trauma-related
225	S33100S	Joint disorders and dislocations; trauma-related
225	S33101A	Joint disorders and dislocations; trauma-related
225	S33101D	Joint disorders and dislocations; trauma-related
225	S33101S	Joint disorders and dislocations; trauma-related
225	S33110A	Joint disorders and dislocations; trauma-related
225	S33110D	Joint disorders and dislocations; trauma-related
225	S33110S	Joint disorders and dislocations; trauma-related
225	S33111A	Joint disorders and dislocations; trauma-related
225	S33111D	Joint disorders and dislocations; trauma-related
225	S33111S	Joint disorders and dislocations; trauma-related
225	S33120A	Joint disorders and dislocations; trauma-related
225	S33120D	Joint disorders and dislocations; trauma-related
225	S33120S	Joint disorders and dislocations; trauma-related
225	S33121A	Joint disorders and dislocations; trauma-related
225	S33121D	Joint disorders and dislocations; trauma-related
225	S33121S	Joint disorders and dislocations; trauma-related
225	S33130A	Joint disorders and dislocations; trauma-related
225	S33130D	Joint disorders and dislocations; trauma-related
225	S33130S	Joint disorders and dislocations; trauma-related
225	S33131A	Joint disorders and dislocations; trauma-related
225	S33131D	Joint disorders and dislocations; trauma-related
225	S33131S	Joint disorders and dislocations; trauma-related
225	S33140A	Joint disorders and dislocations; trauma-related
225	S33140D	Joint disorders and dislocations; trauma-related
225	S33140S	Joint disorders and dislocations; trauma-related
225	S33141A	Joint disorders and dislocations; trauma-related
225	S33141D	Joint disorders and dislocations; trauma-related
225	S33141S	Joint disorders and dislocations; trauma-related
225	S332XXA	Joint disorders and dislocations; trauma-related
225	S332XXD	Joint disorders and dislocations; trauma-related
225	S332XXS	Joint disorders and dislocations; trauma-related

225	S3330XA	Joint disorders and dislocations; trauma-related
225	S3330XD	Joint disorders and dislocations; trauma-related
225	S3330XS	Joint disorders and dislocations; trauma-related
225	S3339XA	Joint disorders and dislocations; trauma-related
225	S3339XD	Joint disorders and dislocations; trauma-related
225	S3339XS	Joint disorders and dislocations; trauma-related
225	S334XXA	Joint disorders and dislocations; trauma-related
225	S334XXD	Joint disorders and dislocations; trauma-related
225	S334XXS	Joint disorders and dislocations; trauma-related
225	S43001A	Joint disorders and dislocations; trauma-related
225	S43001D	Joint disorders and dislocations; trauma-related
225	S43001S	Joint disorders and dislocations; trauma-related
225	S43002A	Joint disorders and dislocations; trauma-related
225	S43002D	Joint disorders and dislocations; trauma-related
225	S43002S	Joint disorders and dislocations; trauma-related
225	S43003A	Joint disorders and dislocations; trauma-related
225	S43003D	Joint disorders and dislocations; trauma-related
225	S43003S	Joint disorders and dislocations; trauma-related
225	S43004A	Joint disorders and dislocations; trauma-related
225	S43004D	Joint disorders and dislocations; trauma-related
225	S43004S	Joint disorders and dislocations; trauma-related
225	S43005A	Joint disorders and dislocations; trauma-related
225	S43005D	Joint disorders and dislocations; trauma-related
225	S43005S	Joint disorders and dislocations; trauma-related
225	S43006A	Joint disorders and dislocations; trauma-related
225	S43006D	Joint disorders and dislocations; trauma-related
225	S43006S	Joint disorders and dislocations; trauma-related
225	S43011A	Joint disorders and dislocations; trauma-related
225	S43011D	Joint disorders and dislocations; trauma-related
225	S43011S	Joint disorders and dislocations; trauma-related
225	S43012A	Joint disorders and dislocations; trauma-related
225	S43012D	Joint disorders and dislocations; trauma-related
225	S43012S	Joint disorders and dislocations; trauma-related
225	S43013A	Joint disorders and dislocations; trauma-related
225	S43013D	Joint disorders and dislocations; trauma-related
225	S43013S	Joint disorders and dislocations; trauma-related
225	S43014A	Joint disorders and dislocations; trauma-related
225	S43014D	Joint disorders and dislocations; trauma-related
225	S43014S	Joint disorders and dislocations; trauma-related
225	S43015A	Joint disorders and dislocations; trauma-related
225	S43015D	Joint disorders and dislocations; trauma-related
225	S43015S	Joint disorders and dislocations; trauma-related
225	S43016A	Joint disorders and dislocations; trauma-related
225	S43016D	Joint disorders and dislocations; trauma-related
225	S43016S	Joint disorders and dislocations; trauma-related
225	S43021A	Joint disorders and dislocations; trauma-related
225	S43021D	Joint disorders and dislocations; trauma-related
225	S43021S	Joint disorders and dislocations; trauma-related
225	S43022A	Joint disorders and dislocations; trauma-related
225	S43022D	Joint disorders and dislocations; trauma-related
225	S43022S	Joint disorders and dislocations; trauma-related

225	S43023A	Joint disorders and dislocations; trauma-related
225	S43023D	Joint disorders and dislocations; trauma-related
225	S43023S	Joint disorders and dislocations; trauma-related
225	S43024A	Joint disorders and dislocations; trauma-related
225	S43024D	Joint disorders and dislocations; trauma-related
225	S43024S	Joint disorders and dislocations; trauma-related
225	S43025A	Joint disorders and dislocations; trauma-related
225	S43025D	Joint disorders and dislocations; trauma-related
225	S43025S	Joint disorders and dislocations; trauma-related
225	S43026A	Joint disorders and dislocations; trauma-related
225	S43026D	Joint disorders and dislocations; trauma-related
225	S43026S	Joint disorders and dislocations; trauma-related
225	S43031A	Joint disorders and dislocations; trauma-related
225	S43031D	Joint disorders and dislocations; trauma-related
225	S43031S	Joint disorders and dislocations; trauma-related
225	S43032A	Joint disorders and dislocations; trauma-related
225	S43032D	Joint disorders and dislocations; trauma-related
225	S43032S	Joint disorders and dislocations; trauma-related
225	S43033A	Joint disorders and dislocations; trauma-related
225	S43033D	Joint disorders and dislocations; trauma-related
225	S43033S	Joint disorders and dislocations; trauma-related
225	S43034A	Joint disorders and dislocations; trauma-related
225	S43034D	Joint disorders and dislocations; trauma-related
225	S43034S	Joint disorders and dislocations; trauma-related
225	S43035A	Joint disorders and dislocations; trauma-related
225	S43035D	Joint disorders and dislocations; trauma-related
225	S43035S	Joint disorders and dislocations; trauma-related
225	S43036A	Joint disorders and dislocations; trauma-related
225	S43036D	Joint disorders and dislocations; trauma-related
225	S43036S	Joint disorders and dislocations; trauma-related
225	S43081A	Joint disorders and dislocations; trauma-related
225	S43081D	Joint disorders and dislocations; trauma-related
225	S43081S	Joint disorders and dislocations; trauma-related
225	S43082A	Joint disorders and dislocations; trauma-related
225	S43082D	Joint disorders and dislocations; trauma-related
225	S43082S	Joint disorders and dislocations; trauma-related
225	S43083A	Joint disorders and dislocations; trauma-related
225	S43083D	Joint disorders and dislocations; trauma-related
225	S43083S	Joint disorders and dislocations; trauma-related
225	S43084A	Joint disorders and dislocations; trauma-related
225	S43084D	Joint disorders and dislocations; trauma-related
225	S43084S	Joint disorders and dislocations; trauma-related
225	S43085A	Joint disorders and dislocations; trauma-related
225	S43085D	Joint disorders and dislocations; trauma-related
225	S43085S	Joint disorders and dislocations; trauma-related
225	S43086A	Joint disorders and dislocations; trauma-related
225	S43086D	Joint disorders and dislocations; trauma-related
225	S43086S	Joint disorders and dislocations; trauma-related
225	S43101A	Joint disorders and dislocations; trauma-related
225	S43101D	Joint disorders and dislocations; trauma-related
225	S43101S	Joint disorders and dislocations; trauma-related

225	S43102A	Joint disorders and dislocations; trauma-related
225	S43102D	Joint disorders and dislocations; trauma-related
225	S43102S	Joint disorders and dislocations; trauma-related
225	S43109A	Joint disorders and dislocations; trauma-related
225	S43109D	Joint disorders and dislocations; trauma-related
225	S43109S	Joint disorders and dislocations; trauma-related
225	S43111A	Joint disorders and dislocations; trauma-related
225	S43111D	Joint disorders and dislocations; trauma-related
225	S43111S	Joint disorders and dislocations; trauma-related
225	S43112A	Joint disorders and dislocations; trauma-related
225	S43112D	Joint disorders and dislocations; trauma-related
225	S43112S	Joint disorders and dislocations; trauma-related
225	S43119A	Joint disorders and dislocations; trauma-related
225	S43119D	Joint disorders and dislocations; trauma-related
225	S43119S	Joint disorders and dislocations; trauma-related
225	S43121A	Joint disorders and dislocations; trauma-related
225	S43121D	Joint disorders and dislocations; trauma-related
225	S43121S	Joint disorders and dislocations; trauma-related
225	S43122A	Joint disorders and dislocations; trauma-related
225	S43122D	Joint disorders and dislocations; trauma-related
225	S43122S	Joint disorders and dislocations; trauma-related
225	S43129A	Joint disorders and dislocations; trauma-related
225	S43129D	Joint disorders and dislocations; trauma-related
225	S43129S	Joint disorders and dislocations; trauma-related
225	S43131A	Joint disorders and dislocations; trauma-related
225	S43131D	Joint disorders and dislocations; trauma-related
225	S43131S	Joint disorders and dislocations; trauma-related
225	S43132A	Joint disorders and dislocations; trauma-related
225	S43132D	Joint disorders and dislocations; trauma-related
225	S43132S	Joint disorders and dislocations; trauma-related
225	S43139A	Joint disorders and dislocations; trauma-related
225	S43139D	Joint disorders and dislocations; trauma-related
225	S43139S	Joint disorders and dislocations; trauma-related
225	S43141A	Joint disorders and dislocations; trauma-related
225	S43141D	Joint disorders and dislocations; trauma-related
225	S43141S	Joint disorders and dislocations; trauma-related
225	S43142A	Joint disorders and dislocations; trauma-related
225	S43142D	Joint disorders and dislocations; trauma-related
225	S43142S	Joint disorders and dislocations; trauma-related
225	S43149A	Joint disorders and dislocations; trauma-related
225	S43149D	Joint disorders and dislocations; trauma-related
225	S43149S	Joint disorders and dislocations; trauma-related
225	S43151A	Joint disorders and dislocations; trauma-related
225	S43151D	Joint disorders and dislocations; trauma-related
225	S43151S	Joint disorders and dislocations; trauma-related
225	S43152A	Joint disorders and dislocations; trauma-related
225	S43152D	Joint disorders and dislocations; trauma-related
225	S43152S	Joint disorders and dislocations; trauma-related
225	S43159A	Joint disorders and dislocations; trauma-related
225	S43159D	Joint disorders and dislocations; trauma-related
225	S43159S	Joint disorders and dislocations; trauma-related

225	S43201A	Joint disorders and dislocations; trauma-related
225	S43201D	Joint disorders and dislocations; trauma-related
225	S43201S	Joint disorders and dislocations; trauma-related
225	S43202A	Joint disorders and dislocations; trauma-related
225	S43202D	Joint disorders and dislocations; trauma-related
225	S43202S	Joint disorders and dislocations; trauma-related
225	S43203A	Joint disorders and dislocations; trauma-related
225	S43203D	Joint disorders and dislocations; trauma-related
225	S43203S	Joint disorders and dislocations; trauma-related
225	S43204A	Joint disorders and dislocations; trauma-related
225	S43204D	Joint disorders and dislocations; trauma-related
225	S43204S	Joint disorders and dislocations; trauma-related
225	S43205A	Joint disorders and dislocations; trauma-related
225	S43205D	Joint disorders and dislocations; trauma-related
225	S43205S	Joint disorders and dislocations; trauma-related
225	S43206A	Joint disorders and dislocations; trauma-related
225	S43206D	Joint disorders and dislocations; trauma-related
225	S43206S	Joint disorders and dislocations; trauma-related
225	S43211A	Joint disorders and dislocations; trauma-related
225	S43211D	Joint disorders and dislocations; trauma-related
225	S43211S	Joint disorders and dislocations; trauma-related
225	S43212A	Joint disorders and dislocations; trauma-related
225	S43212D	Joint disorders and dislocations; trauma-related
225	S43212S	Joint disorders and dislocations; trauma-related
225	S43213A	Joint disorders and dislocations; trauma-related
225	S43213D	Joint disorders and dislocations; trauma-related
225	S43213S	Joint disorders and dislocations; trauma-related
225	S43214A	Joint disorders and dislocations; trauma-related
225	S43214D	Joint disorders and dislocations; trauma-related
225	S43214S	Joint disorders and dislocations; trauma-related
225	S43215A	Joint disorders and dislocations; trauma-related
225	S43215D	Joint disorders and dislocations; trauma-related
225	S43215S	Joint disorders and dislocations; trauma-related
225	S43216A	Joint disorders and dislocations; trauma-related
225	S43216D	Joint disorders and dislocations; trauma-related
225	S43216S	Joint disorders and dislocations; trauma-related
225	S43221A	Joint disorders and dislocations; trauma-related
225	S43221D	Joint disorders and dislocations; trauma-related
225	S43221S	Joint disorders and dislocations; trauma-related
225	S43222A	Joint disorders and dislocations; trauma-related
225	S43222D	Joint disorders and dislocations; trauma-related
225	S43222S	Joint disorders and dislocations; trauma-related
225	S43223A	Joint disorders and dislocations; trauma-related
225	S43223D	Joint disorders and dislocations; trauma-related
225	S43223S	Joint disorders and dislocations; trauma-related
225	S43224A	Joint disorders and dislocations; trauma-related
225	S43224D	Joint disorders and dislocations; trauma-related
225	S43224S	Joint disorders and dislocations; trauma-related
225	S43225A	Joint disorders and dislocations; trauma-related
225	S43225D	Joint disorders and dislocations; trauma-related
225	S43225S	Joint disorders and dislocations; trauma-related

225	S43226A	Joint disorders and dislocations; trauma-related
225	S43226D	Joint disorders and dislocations; trauma-related
225	S43226S	Joint disorders and dislocations; trauma-related
225	S43301A	Joint disorders and dislocations; trauma-related
225	S43301D	Joint disorders and dislocations; trauma-related
225	S43301S	Joint disorders and dislocations; trauma-related
225	S43302A	Joint disorders and dislocations; trauma-related
225	S43302D	Joint disorders and dislocations; trauma-related
225	S43302S	Joint disorders and dislocations; trauma-related
225	S43303A	Joint disorders and dislocations; trauma-related
225	S43303D	Joint disorders and dislocations; trauma-related
225	S43303S	Joint disorders and dislocations; trauma-related
225	S43304A	Joint disorders and dislocations; trauma-related
225	S43304D	Joint disorders and dislocations; trauma-related
225	S43304S	Joint disorders and dislocations; trauma-related
225	S43305A	Joint disorders and dislocations; trauma-related
225	S43305D	Joint disorders and dislocations; trauma-related
225	S43305S	Joint disorders and dislocations; trauma-related
225	S43306A	Joint disorders and dislocations; trauma-related
225	S43306D	Joint disorders and dislocations; trauma-related
225	S43306S	Joint disorders and dislocations; trauma-related
225	S43311A	Joint disorders and dislocations; trauma-related
225	S43311D	Joint disorders and dislocations; trauma-related
225	S43311S	Joint disorders and dislocations; trauma-related
225	S43312A	Joint disorders and dislocations; trauma-related
225	S43312D	Joint disorders and dislocations; trauma-related
225	S43312S	Joint disorders and dislocations; trauma-related
225	S43313A	Joint disorders and dislocations; trauma-related
225	S43313D	Joint disorders and dislocations; trauma-related
225	S43313S	Joint disorders and dislocations; trauma-related
225	S43314A	Joint disorders and dislocations; trauma-related
225	S43314D	Joint disorders and dislocations; trauma-related
225	S43314S	Joint disorders and dislocations; trauma-related
225	S43315A	Joint disorders and dislocations; trauma-related
225	S43315D	Joint disorders and dislocations; trauma-related
225	S43315S	Joint disorders and dislocations; trauma-related
225	S43316A	Joint disorders and dislocations; trauma-related
225	S43316D	Joint disorders and dislocations; trauma-related
225	S43316S	Joint disorders and dislocations; trauma-related
225	S43391A	Joint disorders and dislocations; trauma-related
225	S43391D	Joint disorders and dislocations; trauma-related
225	S43391S	Joint disorders and dislocations; trauma-related
225	S43392A	Joint disorders and dislocations; trauma-related
225	S43392D	Joint disorders and dislocations; trauma-related
225	S43392S	Joint disorders and dislocations; trauma-related
225	S43393A	Joint disorders and dislocations; trauma-related
225	S43393D	Joint disorders and dislocations; trauma-related
225	S43393S	Joint disorders and dislocations; trauma-related
225	S43394A	Joint disorders and dislocations; trauma-related
225	S43394D	Joint disorders and dislocations; trauma-related
225	S43394S	Joint disorders and dislocations; trauma-related

225	S43395A	Joint disorders and dislocations; trauma-related
225	S43395D	Joint disorders and dislocations; trauma-related
225	S43395S	Joint disorders and dislocations; trauma-related
225	S43396A	Joint disorders and dislocations; trauma-related
225	S43396D	Joint disorders and dislocations; trauma-related
225	S43396S	Joint disorders and dislocations; trauma-related
225	S53001A	Joint disorders and dislocations; trauma-related
225	S53001D	Joint disorders and dislocations; trauma-related
225	S53001S	Joint disorders and dislocations; trauma-related
225	S53002A	Joint disorders and dislocations; trauma-related
225	S53002D	Joint disorders and dislocations; trauma-related
225	S53002S	Joint disorders and dislocations; trauma-related
225	S53003A	Joint disorders and dislocations; trauma-related
225	S53003D	Joint disorders and dislocations; trauma-related
225	S53003S	Joint disorders and dislocations; trauma-related
225	S53004A	Joint disorders and dislocations; trauma-related
225	S53004D	Joint disorders and dislocations; trauma-related
225	S53004S	Joint disorders and dislocations; trauma-related
225	S53005A	Joint disorders and dislocations; trauma-related
225	S53005D	Joint disorders and dislocations; trauma-related
225	S53005S	Joint disorders and dislocations; trauma-related
225	S53006A	Joint disorders and dislocations; trauma-related
225	S53006D	Joint disorders and dislocations; trauma-related
225	S53006S	Joint disorders and dislocations; trauma-related
225	S53011A	Joint disorders and dislocations; trauma-related
225	S53011D	Joint disorders and dislocations; trauma-related
225	S53011S	Joint disorders and dislocations; trauma-related
225	S53012A	Joint disorders and dislocations; trauma-related
225	S53012D	Joint disorders and dislocations; trauma-related
225	S53012S	Joint disorders and dislocations; trauma-related
225	S53013A	Joint disorders and dislocations; trauma-related
225	S53013D	Joint disorders and dislocations; trauma-related
225	S53013S	Joint disorders and dislocations; trauma-related
225	S53014A	Joint disorders and dislocations; trauma-related
225	S53014D	Joint disorders and dislocations; trauma-related
225	S53014S	Joint disorders and dislocations; trauma-related
225	S53015A	Joint disorders and dislocations; trauma-related
225	S53015D	Joint disorders and dislocations; trauma-related
225	S53015S	Joint disorders and dislocations; trauma-related
225	S53016A	Joint disorders and dislocations; trauma-related
225	S53016D	Joint disorders and dislocations; trauma-related
225	S53016S	Joint disorders and dislocations; trauma-related
225	S53021A	Joint disorders and dislocations; trauma-related
225	S53021D	Joint disorders and dislocations; trauma-related
225	S53021S	Joint disorders and dislocations; trauma-related
225	S53022A	Joint disorders and dislocations; trauma-related
225	S53022D	Joint disorders and dislocations; trauma-related
225	S53022S	Joint disorders and dislocations; trauma-related
225	S53023A	Joint disorders and dislocations; trauma-related
225	S53023D	Joint disorders and dislocations; trauma-related
225	S53023S	Joint disorders and dislocations; trauma-related

225	S53024A	Joint disorders and dislocations; trauma-related
225	S53024D	Joint disorders and dislocations; trauma-related
225	S53024S	Joint disorders and dislocations; trauma-related
225	S53025A	Joint disorders and dislocations; trauma-related
225	S53025D	Joint disorders and dislocations; trauma-related
225	S53025S	Joint disorders and dislocations; trauma-related
225	S53026A	Joint disorders and dislocations; trauma-related
225	S53026D	Joint disorders and dislocations; trauma-related
225	S53026S	Joint disorders and dislocations; trauma-related
225	S53031A	Joint disorders and dislocations; trauma-related
225	S53031D	Joint disorders and dislocations; trauma-related
225	S53031S	Joint disorders and dislocations; trauma-related
225	S53032A	Joint disorders and dislocations; trauma-related
225	S53032D	Joint disorders and dislocations; trauma-related
225	S53032S	Joint disorders and dislocations; trauma-related
225	S53033A	Joint disorders and dislocations; trauma-related
225	S53033D	Joint disorders and dislocations; trauma-related
225	S53033S	Joint disorders and dislocations; trauma-related
225	S53091A	Joint disorders and dislocations; trauma-related
225	S53091D	Joint disorders and dislocations; trauma-related
225	S53091S	Joint disorders and dislocations; trauma-related
225	S53092A	Joint disorders and dislocations; trauma-related
225	S53092D	Joint disorders and dislocations; trauma-related
225	S53092S	Joint disorders and dislocations; trauma-related
225	S53093A	Joint disorders and dislocations; trauma-related
225	S53093D	Joint disorders and dislocations; trauma-related
225	S53093S	Joint disorders and dislocations; trauma-related
225	S53094A	Joint disorders and dislocations; trauma-related
225	S53094D	Joint disorders and dislocations; trauma-related
225	S53094S	Joint disorders and dislocations; trauma-related
225	S53095A	Joint disorders and dislocations; trauma-related
225	S53095D	Joint disorders and dislocations; trauma-related
225	S53095S	Joint disorders and dislocations; trauma-related
225	S53096A	Joint disorders and dislocations; trauma-related
225	S53096D	Joint disorders and dislocations; trauma-related
225	S53096S	Joint disorders and dislocations; trauma-related
225	S53101A	Joint disorders and dislocations; trauma-related
225	S53101D	Joint disorders and dislocations; trauma-related
225	S53101S	Joint disorders and dislocations; trauma-related
225	S53102A	Joint disorders and dislocations; trauma-related
225	S53102D	Joint disorders and dislocations; trauma-related
225	S53102S	Joint disorders and dislocations; trauma-related
225	S53103A	Joint disorders and dislocations; trauma-related
225	S53103D	Joint disorders and dislocations; trauma-related
225	S53103S	Joint disorders and dislocations; trauma-related
225	S53104A	Joint disorders and dislocations; trauma-related
225	S53104D	Joint disorders and dislocations; trauma-related
225	S53104S	Joint disorders and dislocations; trauma-related
225	S53105A	Joint disorders and dislocations; trauma-related
225	S53105D	Joint disorders and dislocations; trauma-related
225	S53105S	Joint disorders and dislocations; trauma-related

225	S53106A	Joint disorders and dislocations; trauma-related
225	S53106D	Joint disorders and dislocations; trauma-related
225	S53106S	Joint disorders and dislocations; trauma-related
225	S53111A	Joint disorders and dislocations; trauma-related
225	S53111D	Joint disorders and dislocations; trauma-related
225	S53111S	Joint disorders and dislocations; trauma-related
225	S53112A	Joint disorders and dislocations; trauma-related
225	S53112D	Joint disorders and dislocations; trauma-related
225	S53112S	Joint disorders and dislocations; trauma-related
225	S53113A	Joint disorders and dislocations; trauma-related
225	S53113D	Joint disorders and dislocations; trauma-related
225	S53113S	Joint disorders and dislocations; trauma-related
225	S53114A	Joint disorders and dislocations; trauma-related
225	S53114D	Joint disorders and dislocations; trauma-related
225	S53114S	Joint disorders and dislocations; trauma-related
225	S53115A	Joint disorders and dislocations; trauma-related
225	S53115D	Joint disorders and dislocations; trauma-related
225	S53115S	Joint disorders and dislocations; trauma-related
225	S53116A	Joint disorders and dislocations; trauma-related
225	S53116D	Joint disorders and dislocations; trauma-related
225	S53116S	Joint disorders and dislocations; trauma-related
225	S53121A	Joint disorders and dislocations; trauma-related
225	S53121D	Joint disorders and dislocations; trauma-related
225	S53121S	Joint disorders and dislocations; trauma-related
225	S53122A	Joint disorders and dislocations; trauma-related
225	S53122D	Joint disorders and dislocations; trauma-related
225	S53122S	Joint disorders and dislocations; trauma-related
225	S53123A	Joint disorders and dislocations; trauma-related
225	S53123D	Joint disorders and dislocations; trauma-related
225	S53123S	Joint disorders and dislocations; trauma-related
225	S53124A	Joint disorders and dislocations; trauma-related
225	S53124D	Joint disorders and dislocations; trauma-related
225	S53124S	Joint disorders and dislocations; trauma-related
225	S53125A	Joint disorders and dislocations; trauma-related
225	S53125D	Joint disorders and dislocations; trauma-related
225	S53125S	Joint disorders and dislocations; trauma-related
225	S53126A	Joint disorders and dislocations; trauma-related
225	S53126D	Joint disorders and dislocations; trauma-related
225	S53126S	Joint disorders and dislocations; trauma-related
225	S53131A	Joint disorders and dislocations; trauma-related
225	S53131D	Joint disorders and dislocations; trauma-related
225	S53131S	Joint disorders and dislocations; trauma-related
225	S53132A	Joint disorders and dislocations; trauma-related
225	S53132D	Joint disorders and dislocations; trauma-related
225	S53132S	Joint disorders and dislocations; trauma-related
225	S53133A	Joint disorders and dislocations; trauma-related
225	S53133D	Joint disorders and dislocations; trauma-related
225	S53133S	Joint disorders and dislocations; trauma-related
225	S53134A	Joint disorders and dislocations; trauma-related
225	S53134D	Joint disorders and dislocations; trauma-related
225	S53134S	Joint disorders and dislocations; trauma-related

225	S53135A	Joint disorders and dislocations; trauma-related
225	S53135D	Joint disorders and dislocations; trauma-related
225	S53135S	Joint disorders and dislocations; trauma-related
225	S53136A	Joint disorders and dislocations; trauma-related
225	S53136D	Joint disorders and dislocations; trauma-related
225	S53136S	Joint disorders and dislocations; trauma-related
225	S53141A	Joint disorders and dislocations; trauma-related
225	S53141D	Joint disorders and dislocations; trauma-related
225	S53141S	Joint disorders and dislocations; trauma-related
225	S53142A	Joint disorders and dislocations; trauma-related
225	S53142D	Joint disorders and dislocations; trauma-related
225	S53142S	Joint disorders and dislocations; trauma-related
225	S53143A	Joint disorders and dislocations; trauma-related
225	S53143D	Joint disorders and dislocations; trauma-related
225	S53143S	Joint disorders and dislocations; trauma-related
225	S53144A	Joint disorders and dislocations; trauma-related
225	S53144D	Joint disorders and dislocations; trauma-related
225	S53144S	Joint disorders and dislocations; trauma-related
225	S53145A	Joint disorders and dislocations; trauma-related
225	S53145D	Joint disorders and dislocations; trauma-related
225	S53145S	Joint disorders and dislocations; trauma-related
225	S53146A	Joint disorders and dislocations; trauma-related
225	S53146D	Joint disorders and dislocations; trauma-related
225	S53146S	Joint disorders and dislocations; trauma-related
225	S53191A	Joint disorders and dislocations; trauma-related
225	S53191D	Joint disorders and dislocations; trauma-related
225	S53191S	Joint disorders and dislocations; trauma-related
225	S53192A	Joint disorders and dislocations; trauma-related
225	S53192D	Joint disorders and dislocations; trauma-related
225	S53192S	Joint disorders and dislocations; trauma-related
225	S53193A	Joint disorders and dislocations; trauma-related
225	S53193D	Joint disorders and dislocations; trauma-related
225	S53193S	Joint disorders and dislocations; trauma-related
225	S53194A	Joint disorders and dislocations; trauma-related
225	S53194D	Joint disorders and dislocations; trauma-related
225	S53194S	Joint disorders and dislocations; trauma-related
225	S53195A	Joint disorders and dislocations; trauma-related
225	S53195D	Joint disorders and dislocations; trauma-related
225	S53195S	Joint disorders and dislocations; trauma-related
225	S53196A	Joint disorders and dislocations; trauma-related
225	S53196D	Joint disorders and dislocations; trauma-related
225	S53196S	Joint disorders and dislocations; trauma-related
225	S63001A	Joint disorders and dislocations; trauma-related
225	S63001D	Joint disorders and dislocations; trauma-related
225	S63001S	Joint disorders and dislocations; trauma-related
225	S63002A	Joint disorders and dislocations; trauma-related
225	S63002D	Joint disorders and dislocations; trauma-related
225	S63002S	Joint disorders and dislocations; trauma-related
225	S63003A	Joint disorders and dislocations; trauma-related
225	S63003D	Joint disorders and dislocations; trauma-related
225	S63003S	Joint disorders and dislocations; trauma-related

225	S63004A	Joint disorders and dislocations; trauma-related
225	S63004D	Joint disorders and dislocations; trauma-related
225	S63004S	Joint disorders and dislocations; trauma-related
225	S63005A	Joint disorders and dislocations; trauma-related
225	S63005D	Joint disorders and dislocations; trauma-related
225	S63005S	Joint disorders and dislocations; trauma-related
225	S63006A	Joint disorders and dislocations; trauma-related
225	S63006D	Joint disorders and dislocations; trauma-related
225	S63006S	Joint disorders and dislocations; trauma-related
225	S63011A	Joint disorders and dislocations; trauma-related
225	S63011D	Joint disorders and dislocations; trauma-related
225	S63011S	Joint disorders and dislocations; trauma-related
225	S63012A	Joint disorders and dislocations; trauma-related
225	S63012D	Joint disorders and dislocations; trauma-related
225	S63012S	Joint disorders and dislocations; trauma-related
225	S63013A	Joint disorders and dislocations; trauma-related
225	S63013D	Joint disorders and dislocations; trauma-related
225	S63013S	Joint disorders and dislocations; trauma-related
225	S63014A	Joint disorders and dislocations; trauma-related
225	S63014D	Joint disorders and dislocations; trauma-related
225	S63014S	Joint disorders and dislocations; trauma-related
225	S63015A	Joint disorders and dislocations; trauma-related
225	S63015D	Joint disorders and dislocations; trauma-related
225	S63015S	Joint disorders and dislocations; trauma-related
225	S63016A	Joint disorders and dislocations; trauma-related
225	S63016D	Joint disorders and dislocations; trauma-related
225	S63016S	Joint disorders and dislocations; trauma-related
225	S63021A	Joint disorders and dislocations; trauma-related
225	S63021D	Joint disorders and dislocations; trauma-related
225	S63021S	Joint disorders and dislocations; trauma-related
225	S63022A	Joint disorders and dislocations; trauma-related
225	S63022D	Joint disorders and dislocations; trauma-related
225	S63022S	Joint disorders and dislocations; trauma-related
225	S63023A	Joint disorders and dislocations; trauma-related
225	S63023D	Joint disorders and dislocations; trauma-related
225	S63023S	Joint disorders and dislocations; trauma-related
225	S63024A	Joint disorders and dislocations; trauma-related
225	S63024D	Joint disorders and dislocations; trauma-related
225	S63024S	Joint disorders and dislocations; trauma-related
225	S63025A	Joint disorders and dislocations; trauma-related
225	S63025D	Joint disorders and dislocations; trauma-related
225	S63025S	Joint disorders and dislocations; trauma-related
225	S63026A	Joint disorders and dislocations; trauma-related
225	S63026D	Joint disorders and dislocations; trauma-related
225	S63026S	Joint disorders and dislocations; trauma-related
225	S63031A	Joint disorders and dislocations; trauma-related
225	S63031D	Joint disorders and dislocations; trauma-related
225	S63031S	Joint disorders and dislocations; trauma-related
225	S63032A	Joint disorders and dislocations; trauma-related
225	S63032D	Joint disorders and dislocations; trauma-related
225	S63032S	Joint disorders and dislocations; trauma-related

225	S63033A	Joint disorders and dislocations; trauma-related
225	S63033D	Joint disorders and dislocations; trauma-related
225	S63033S	Joint disorders and dislocations; trauma-related
225	S63034A	Joint disorders and dislocations; trauma-related
225	S63034D	Joint disorders and dislocations; trauma-related
225	S63034S	Joint disorders and dislocations; trauma-related
225	S63035A	Joint disorders and dislocations; trauma-related
225	S63035D	Joint disorders and dislocations; trauma-related
225	S63035S	Joint disorders and dislocations; trauma-related
225	S63036A	Joint disorders and dislocations; trauma-related
225	S63036D	Joint disorders and dislocations; trauma-related
225	S63036S	Joint disorders and dislocations; trauma-related
225	S63041A	Joint disorders and dislocations; trauma-related
225	S63041D	Joint disorders and dislocations; trauma-related
225	S63041S	Joint disorders and dislocations; trauma-related
225	S63042A	Joint disorders and dislocations; trauma-related
225	S63042D	Joint disorders and dislocations; trauma-related
225	S63042S	Joint disorders and dislocations; trauma-related
225	S63043A	Joint disorders and dislocations; trauma-related
225	S63043D	Joint disorders and dislocations; trauma-related
225	S63043S	Joint disorders and dislocations; trauma-related
225	S63044A	Joint disorders and dislocations; trauma-related
225	S63044D	Joint disorders and dislocations; trauma-related
225	S63044S	Joint disorders and dislocations; trauma-related
225	S63045A	Joint disorders and dislocations; trauma-related
225	S63045D	Joint disorders and dislocations; trauma-related
225	S63045S	Joint disorders and dislocations; trauma-related
225	S63046A	Joint disorders and dislocations; trauma-related
225	S63046D	Joint disorders and dislocations; trauma-related
225	S63046S	Joint disorders and dislocations; trauma-related
225	S63051A	Joint disorders and dislocations; trauma-related
225	S63051D	Joint disorders and dislocations; trauma-related
225	S63051S	Joint disorders and dislocations; trauma-related
225	S63052A	Joint disorders and dislocations; trauma-related
225	S63052D	Joint disorders and dislocations; trauma-related
225	S63052S	Joint disorders and dislocations; trauma-related
225	S63053A	Joint disorders and dislocations; trauma-related
225	S63053D	Joint disorders and dislocations; trauma-related
225	S63053S	Joint disorders and dislocations; trauma-related
225	S63054A	Joint disorders and dislocations; trauma-related
225	S63054D	Joint disorders and dislocations; trauma-related
225	S63054S	Joint disorders and dislocations; trauma-related
225	S63055A	Joint disorders and dislocations; trauma-related
225	S63055D	Joint disorders and dislocations; trauma-related
225	S63055S	Joint disorders and dislocations; trauma-related
225	S63056A	Joint disorders and dislocations; trauma-related
225	S63056D	Joint disorders and dislocations; trauma-related
225	S63056S	Joint disorders and dislocations; trauma-related
225	S63061A	Joint disorders and dislocations; trauma-related
225	S63061D	Joint disorders and dislocations; trauma-related
225	S63061S	Joint disorders and dislocations; trauma-related

225	S63062A	Joint disorders and dislocations; trauma-related
225	S63062D	Joint disorders and dislocations; trauma-related
225	S63062S	Joint disorders and dislocations; trauma-related
225	S63063A	Joint disorders and dislocations; trauma-related
225	S63063D	Joint disorders and dislocations; trauma-related
225	S63063S	Joint disorders and dislocations; trauma-related
225	S63064A	Joint disorders and dislocations; trauma-related
225	S63064D	Joint disorders and dislocations; trauma-related
225	S63064S	Joint disorders and dislocations; trauma-related
225	S63065A	Joint disorders and dislocations; trauma-related
225	S63065D	Joint disorders and dislocations; trauma-related
225	S63065S	Joint disorders and dislocations; trauma-related
225	S63066A	Joint disorders and dislocations; trauma-related
225	S63066D	Joint disorders and dislocations; trauma-related
225	S63066S	Joint disorders and dislocations; trauma-related
225	S63071A	Joint disorders and dislocations; trauma-related
225	S63071D	Joint disorders and dislocations; trauma-related
225	S63071S	Joint disorders and dislocations; trauma-related
225	S63072A	Joint disorders and dislocations; trauma-related
225	S63072D	Joint disorders and dislocations; trauma-related
225	S63072S	Joint disorders and dislocations; trauma-related
225	S63073A	Joint disorders and dislocations; trauma-related
225	S63073D	Joint disorders and dislocations; trauma-related
225	S63073S	Joint disorders and dislocations; trauma-related
225	S63074A	Joint disorders and dislocations; trauma-related
225	S63074D	Joint disorders and dislocations; trauma-related
225	S63074S	Joint disorders and dislocations; trauma-related
225	S63075A	Joint disorders and dislocations; trauma-related
225	S63075D	Joint disorders and dislocations; trauma-related
225	S63075S	Joint disorders and dislocations; trauma-related
225	S63076A	Joint disorders and dislocations; trauma-related
225	S63076D	Joint disorders and dislocations; trauma-related
225	S63076S	Joint disorders and dislocations; trauma-related
225	S63091A	Joint disorders and dislocations; trauma-related
225	S63091D	Joint disorders and dislocations; trauma-related
225	S63091S	Joint disorders and dislocations; trauma-related
225	S63092A	Joint disorders and dislocations; trauma-related
225	S63092D	Joint disorders and dislocations; trauma-related
225	S63092S	Joint disorders and dislocations; trauma-related
225	S63093A	Joint disorders and dislocations; trauma-related
225	S63093D	Joint disorders and dislocations; trauma-related
225	S63093S	Joint disorders and dislocations; trauma-related
225	S63094A	Joint disorders and dislocations; trauma-related
225	S63094D	Joint disorders and dislocations; trauma-related
225	S63094S	Joint disorders and dislocations; trauma-related
225	S63095A	Joint disorders and dislocations; trauma-related
225	S63095D	Joint disorders and dislocations; trauma-related
225	S63095S	Joint disorders and dislocations; trauma-related
225	S63096A	Joint disorders and dislocations; trauma-related
225	S63096D	Joint disorders and dislocations; trauma-related
225	S63096S	Joint disorders and dislocations; trauma-related

225	S63101A	Joint disorders and dislocations; trauma-related
225	S63101D	Joint disorders and dislocations; trauma-related
225	S63101S	Joint disorders and dislocations; trauma-related
225	S63102A	Joint disorders and dislocations; trauma-related
225	S63102D	Joint disorders and dislocations; trauma-related
225	S63102S	Joint disorders and dislocations; trauma-related
225	S63103A	Joint disorders and dislocations; trauma-related
225	S63103D	Joint disorders and dislocations; trauma-related
225	S63103S	Joint disorders and dislocations; trauma-related
225	S63104A	Joint disorders and dislocations; trauma-related
225	S63104D	Joint disorders and dislocations; trauma-related
225	S63104S	Joint disorders and dislocations; trauma-related
225	S63105A	Joint disorders and dislocations; trauma-related
225	S63105D	Joint disorders and dislocations; trauma-related
225	S63105S	Joint disorders and dislocations; trauma-related
225	S63106A	Joint disorders and dislocations; trauma-related
225	S63106D	Joint disorders and dislocations; trauma-related
225	S63106S	Joint disorders and dislocations; trauma-related
225	S63111A	Joint disorders and dislocations; trauma-related
225	S63111D	Joint disorders and dislocations; trauma-related
225	S63111S	Joint disorders and dislocations; trauma-related
225	S63112A	Joint disorders and dislocations; trauma-related
225	S63112D	Joint disorders and dislocations; trauma-related
225	S63112S	Joint disorders and dislocations; trauma-related
225	S63113A	Joint disorders and dislocations; trauma-related
225	S63113D	Joint disorders and dislocations; trauma-related
225	S63113S	Joint disorders and dislocations; trauma-related
225	S63114A	Joint disorders and dislocations; trauma-related
225	S63114D	Joint disorders and dislocations; trauma-related
225	S63114S	Joint disorders and dislocations; trauma-related
225	S63115A	Joint disorders and dislocations; trauma-related
225	S63115D	Joint disorders and dislocations; trauma-related
225	S63115S	Joint disorders and dislocations; trauma-related
225	S63116A	Joint disorders and dislocations; trauma-related
225	S63116D	Joint disorders and dislocations; trauma-related
225	S63116S	Joint disorders and dislocations; trauma-related
225	S63121A	Joint disorders and dislocations; trauma-related
225	S63121D	Joint disorders and dislocations; trauma-related
225	S63121S	Joint disorders and dislocations; trauma-related
225	S63122A	Joint disorders and dislocations; trauma-related
225	S63122D	Joint disorders and dislocations; trauma-related
225	S63122S	Joint disorders and dislocations; trauma-related
225	S63123A	Joint disorders and dislocations; trauma-related
225	S63123D	Joint disorders and dislocations; trauma-related
225	S63123S	Joint disorders and dislocations; trauma-related
225	S63124A	Joint disorders and dislocations; trauma-related
225	S63124D	Joint disorders and dislocations; trauma-related
225	S63124S	Joint disorders and dislocations; trauma-related
225	S63125A	Joint disorders and dislocations; trauma-related
225	S63125D	Joint disorders and dislocations; trauma-related
225	S63125S	Joint disorders and dislocations; trauma-related

225	S63126A	Joint disorders and dislocations; trauma-related
225	S63126D	Joint disorders and dislocations; trauma-related
225	S63126S	Joint disorders and dislocations; trauma-related
225	S63131A	Joint disorders and dislocations; trauma-related
225	S63131D	Joint disorders and dislocations; trauma-related
225	S63131S	Joint disorders and dislocations; trauma-related
225	S63132A	Joint disorders and dislocations; trauma-related
225	S63132D	Joint disorders and dislocations; trauma-related
225	S63132S	Joint disorders and dislocations; trauma-related
225	S63133A	Joint disorders and dislocations; trauma-related
225	S63133D	Joint disorders and dislocations; trauma-related
225	S63133S	Joint disorders and dislocations; trauma-related
225	S63134A	Joint disorders and dislocations; trauma-related
225	S63134D	Joint disorders and dislocations; trauma-related
225	S63134S	Joint disorders and dislocations; trauma-related
225	S63135A	Joint disorders and dislocations; trauma-related
225	S63135D	Joint disorders and dislocations; trauma-related
225	S63135S	Joint disorders and dislocations; trauma-related
225	S63136A	Joint disorders and dislocations; trauma-related
225	S63136D	Joint disorders and dislocations; trauma-related
225	S63136S	Joint disorders and dislocations; trauma-related
225	S63141A	Joint disorders and dislocations; trauma-related
225	S63141D	Joint disorders and dislocations; trauma-related
225	S63141S	Joint disorders and dislocations; trauma-related
225	S63142A	Joint disorders and dislocations; trauma-related
225	S63142D	Joint disorders and dislocations; trauma-related
225	S63142S	Joint disorders and dislocations; trauma-related
225	S63143A	Joint disorders and dislocations; trauma-related
225	S63143D	Joint disorders and dislocations; trauma-related
225	S63143S	Joint disorders and dislocations; trauma-related
225	S63144A	Joint disorders and dislocations; trauma-related
225	S63144D	Joint disorders and dislocations; trauma-related
225	S63144S	Joint disorders and dislocations; trauma-related
225	S63145A	Joint disorders and dislocations; trauma-related
225	S63145D	Joint disorders and dislocations; trauma-related
225	S63145S	Joint disorders and dislocations; trauma-related
225	S63146A	Joint disorders and dislocations; trauma-related
225	S63146D	Joint disorders and dislocations; trauma-related
225	S63146S	Joint disorders and dislocations; trauma-related
225	S63200A	Joint disorders and dislocations; trauma-related
225	S63200D	Joint disorders and dislocations; trauma-related
225	S63200S	Joint disorders and dislocations; trauma-related
225	S63201A	Joint disorders and dislocations; trauma-related
225	S63201D	Joint disorders and dislocations; trauma-related
225	S63201S	Joint disorders and dislocations; trauma-related
225	S63202A	Joint disorders and dislocations; trauma-related
225	S63202D	Joint disorders and dislocations; trauma-related
225	S63202S	Joint disorders and dislocations; trauma-related
225	S63203A	Joint disorders and dislocations; trauma-related
225	S63203D	Joint disorders and dislocations; trauma-related
225	S63203S	Joint disorders and dislocations; trauma-related

225	S63204A	Joint disorders and dislocations; trauma-related
225	S63204D	Joint disorders and dislocations; trauma-related
225	S63204S	Joint disorders and dislocations; trauma-related
225	S63205A	Joint disorders and dislocations; trauma-related
225	S63205D	Joint disorders and dislocations; trauma-related
225	S63205S	Joint disorders and dislocations; trauma-related
225	S63206A	Joint disorders and dislocations; trauma-related
225	S63206D	Joint disorders and dislocations; trauma-related
225	S63206S	Joint disorders and dislocations; trauma-related
225	S63207A	Joint disorders and dislocations; trauma-related
225	S63207D	Joint disorders and dislocations; trauma-related
225	S63207S	Joint disorders and dislocations; trauma-related
225	S63208A	Joint disorders and dislocations; trauma-related
225	S63208D	Joint disorders and dislocations; trauma-related
225	S63208S	Joint disorders and dislocations; trauma-related
225	S63209A	Joint disorders and dislocations; trauma-related
225	S63209D	Joint disorders and dislocations; trauma-related
225	S63209S	Joint disorders and dislocations; trauma-related
225	S63210A	Joint disorders and dislocations; trauma-related
225	S63210D	Joint disorders and dislocations; trauma-related
225	S63210S	Joint disorders and dislocations; trauma-related
225	S63211A	Joint disorders and dislocations; trauma-related
225	S63211D	Joint disorders and dislocations; trauma-related
225	S63211S	Joint disorders and dislocations; trauma-related
225	S63212A	Joint disorders and dislocations; trauma-related
225	S63212D	Joint disorders and dislocations; trauma-related
225	S63212S	Joint disorders and dislocations; trauma-related
225	S63213A	Joint disorders and dislocations; trauma-related
225	S63213D	Joint disorders and dislocations; trauma-related
225	S63213S	Joint disorders and dislocations; trauma-related
225	S63214A	Joint disorders and dislocations; trauma-related
225	S63214D	Joint disorders and dislocations; trauma-related
225	S63214S	Joint disorders and dislocations; trauma-related
225	S63215A	Joint disorders and dislocations; trauma-related
225	S63215D	Joint disorders and dislocations; trauma-related
225	S63215S	Joint disorders and dislocations; trauma-related
225	S63216A	Joint disorders and dislocations; trauma-related
225	S63216D	Joint disorders and dislocations; trauma-related
225	S63216S	Joint disorders and dislocations; trauma-related
225	S63217A	Joint disorders and dislocations; trauma-related
225	S63217D	Joint disorders and dislocations; trauma-related
225	S63217S	Joint disorders and dislocations; trauma-related
225	S63218A	Joint disorders and dislocations; trauma-related
225	S63218D	Joint disorders and dislocations; trauma-related
225	S63218S	Joint disorders and dislocations; trauma-related
225	S63219A	Joint disorders and dislocations; trauma-related
225	S63219D	Joint disorders and dislocations; trauma-related
225	S63219S	Joint disorders and dislocations; trauma-related
225	S63220A	Joint disorders and dislocations; trauma-related
225	S63220D	Joint disorders and dislocations; trauma-related
225	S63220S	Joint disorders and dislocations; trauma-related

225	S63221A	Joint disorders and dislocations; trauma-related
225	S63221D	Joint disorders and dislocations; trauma-related
225	S63221S	Joint disorders and dislocations; trauma-related
225	S63222A	Joint disorders and dislocations; trauma-related
225	S63222D	Joint disorders and dislocations; trauma-related
225	S63222S	Joint disorders and dislocations; trauma-related
225	S63223A	Joint disorders and dislocations; trauma-related
225	S63223D	Joint disorders and dislocations; trauma-related
225	S63223S	Joint disorders and dislocations; trauma-related
225	S63224A	Joint disorders and dislocations; trauma-related
225	S63224D	Joint disorders and dislocations; trauma-related
225	S63224S	Joint disorders and dislocations; trauma-related
225	S63225A	Joint disorders and dislocations; trauma-related
225	S63225D	Joint disorders and dislocations; trauma-related
225	S63225S	Joint disorders and dislocations; trauma-related
225	S63226A	Joint disorders and dislocations; trauma-related
225	S63226D	Joint disorders and dislocations; trauma-related
225	S63226S	Joint disorders and dislocations; trauma-related
225	S63227A	Joint disorders and dislocations; trauma-related
225	S63227D	Joint disorders and dislocations; trauma-related
225	S63227S	Joint disorders and dislocations; trauma-related
225	S63228A	Joint disorders and dislocations; trauma-related
225	S63228D	Joint disorders and dislocations; trauma-related
225	S63228S	Joint disorders and dislocations; trauma-related
225	S63229A	Joint disorders and dislocations; trauma-related
225	S63229D	Joint disorders and dislocations; trauma-related
225	S63229S	Joint disorders and dislocations; trauma-related
225	S63230A	Joint disorders and dislocations; trauma-related
225	S63230D	Joint disorders and dislocations; trauma-related
225	S63230S	Joint disorders and dislocations; trauma-related
225	S63231A	Joint disorders and dislocations; trauma-related
225	S63231D	Joint disorders and dislocations; trauma-related
225	S63231S	Joint disorders and dislocations; trauma-related
225	S63232A	Joint disorders and dislocations; trauma-related
225	S63232D	Joint disorders and dislocations; trauma-related
225	S63232S	Joint disorders and dislocations; trauma-related
225	S63233A	Joint disorders and dislocations; trauma-related
225	S63233D	Joint disorders and dislocations; trauma-related
225	S63233S	Joint disorders and dislocations; trauma-related
225	S63234A	Joint disorders and dislocations; trauma-related
225	S63234D	Joint disorders and dislocations; trauma-related
225	S63234S	Joint disorders and dislocations; trauma-related
225	S63235A	Joint disorders and dislocations; trauma-related
225	S63235D	Joint disorders and dislocations; trauma-related
225	S63235S	Joint disorders and dislocations; trauma-related
225	S63236A	Joint disorders and dislocations; trauma-related
225	S63236D	Joint disorders and dislocations; trauma-related
225	S63236S	Joint disorders and dislocations; trauma-related
225	S63237A	Joint disorders and dislocations; trauma-related
225	S63237D	Joint disorders and dislocations; trauma-related
225	S63237S	Joint disorders and dislocations; trauma-related

225	S63238A	Joint disorders and dislocations; trauma-related
225	S63238D	Joint disorders and dislocations; trauma-related
225	S63238S	Joint disorders and dislocations; trauma-related
225	S63239A	Joint disorders and dislocations; trauma-related
225	S63239D	Joint disorders and dislocations; trauma-related
225	S63239S	Joint disorders and dislocations; trauma-related
225	S63240A	Joint disorders and dislocations; trauma-related
225	S63240D	Joint disorders and dislocations; trauma-related
225	S63240S	Joint disorders and dislocations; trauma-related
225	S63241A	Joint disorders and dislocations; trauma-related
225	S63241D	Joint disorders and dislocations; trauma-related
225	S63241S	Joint disorders and dislocations; trauma-related
225	S63242A	Joint disorders and dislocations; trauma-related
225	S63242D	Joint disorders and dislocations; trauma-related
225	S63242S	Joint disorders and dislocations; trauma-related
225	S63243A	Joint disorders and dislocations; trauma-related
225	S63243D	Joint disorders and dislocations; trauma-related
225	S63243S	Joint disorders and dislocations; trauma-related
225	S63244A	Joint disorders and dislocations; trauma-related
225	S63244D	Joint disorders and dislocations; trauma-related
225	S63244S	Joint disorders and dislocations; trauma-related
225	S63245A	Joint disorders and dislocations; trauma-related
225	S63245D	Joint disorders and dislocations; trauma-related
225	S63245S	Joint disorders and dislocations; trauma-related
225	S63246A	Joint disorders and dislocations; trauma-related
225	S63246D	Joint disorders and dislocations; trauma-related
225	S63246S	Joint disorders and dislocations; trauma-related
225	S63247A	Joint disorders and dislocations; trauma-related
225	S63247D	Joint disorders and dislocations; trauma-related
225	S63247S	Joint disorders and dislocations; trauma-related
225	S63248A	Joint disorders and dislocations; trauma-related
225	S63248D	Joint disorders and dislocations; trauma-related
225	S63248S	Joint disorders and dislocations; trauma-related
225	S63249A	Joint disorders and dislocations; trauma-related
225	S63249D	Joint disorders and dislocations; trauma-related
225	S63249S	Joint disorders and dislocations; trauma-related
225	S63250A	Joint disorders and dislocations; trauma-related
225	S63250D	Joint disorders and dislocations; trauma-related
225	S63250S	Joint disorders and dislocations; trauma-related
225	S63251A	Joint disorders and dislocations; trauma-related
225	S63251D	Joint disorders and dislocations; trauma-related
225	S63251S	Joint disorders and dislocations; trauma-related
225	S63252A	Joint disorders and dislocations; trauma-related
225	S63252D	Joint disorders and dislocations; trauma-related
225	S63252S	Joint disorders and dislocations; trauma-related
225	S63253A	Joint disorders and dislocations; trauma-related
225	S63253D	Joint disorders and dislocations; trauma-related
225	S63253S	Joint disorders and dislocations; trauma-related
225	S63254A	Joint disorders and dislocations; trauma-related
225	S63254D	Joint disorders and dislocations; trauma-related
225	S63254S	Joint disorders and dislocations; trauma-related

225	S63255A	Joint disorders and dislocations; trauma-related
225	S63255D	Joint disorders and dislocations; trauma-related
225	S63255S	Joint disorders and dislocations; trauma-related
225	S63256A	Joint disorders and dislocations; trauma-related
225	S63256D	Joint disorders and dislocations; trauma-related
225	S63256S	Joint disorders and dislocations; trauma-related
225	S63257A	Joint disorders and dislocations; trauma-related
225	S63257D	Joint disorders and dislocations; trauma-related
225	S63257S	Joint disorders and dislocations; trauma-related
225	S63258A	Joint disorders and dislocations; trauma-related
225	S63258D	Joint disorders and dislocations; trauma-related
225	S63258S	Joint disorders and dislocations; trauma-related
225	S63259A	Joint disorders and dislocations; trauma-related
225	S63259D	Joint disorders and dislocations; trauma-related
225	S63259S	Joint disorders and dislocations; trauma-related
225	S63260A	Joint disorders and dislocations; trauma-related
225	S63260D	Joint disorders and dislocations; trauma-related
225	S63260S	Joint disorders and dislocations; trauma-related
225	S63261A	Joint disorders and dislocations; trauma-related
225	S63261D	Joint disorders and dislocations; trauma-related
225	S63261S	Joint disorders and dislocations; trauma-related
225	S63262A	Joint disorders and dislocations; trauma-related
225	S63262D	Joint disorders and dislocations; trauma-related
225	S63262S	Joint disorders and dislocations; trauma-related
225	S63263A	Joint disorders and dislocations; trauma-related
225	S63263D	Joint disorders and dislocations; trauma-related
225	S63263S	Joint disorders and dislocations; trauma-related
225	S63264A	Joint disorders and dislocations; trauma-related
225	S63264D	Joint disorders and dislocations; trauma-related
225	S63264S	Joint disorders and dislocations; trauma-related
225	S63265A	Joint disorders and dislocations; trauma-related
225	S63265D	Joint disorders and dislocations; trauma-related
225	S63265S	Joint disorders and dislocations; trauma-related
225	S63266A	Joint disorders and dislocations; trauma-related
225	S63266D	Joint disorders and dislocations; trauma-related
225	S63266S	Joint disorders and dislocations; trauma-related
225	S63267A	Joint disorders and dislocations; trauma-related
225	S63267D	Joint disorders and dislocations; trauma-related
225	S63267S	Joint disorders and dislocations; trauma-related
225	S63268A	Joint disorders and dislocations; trauma-related
225	S63268D	Joint disorders and dislocations; trauma-related
225	S63268S	Joint disorders and dislocations; trauma-related
225	S63269A	Joint disorders and dislocations; trauma-related
225	S63269D	Joint disorders and dislocations; trauma-related
225	S63269S	Joint disorders and dislocations; trauma-related
225	S63270A	Joint disorders and dislocations; trauma-related
225	S63270D	Joint disorders and dislocations; trauma-related
225	S63270S	Joint disorders and dislocations; trauma-related
225	S63271A	Joint disorders and dislocations; trauma-related
225	S63271D	Joint disorders and dislocations; trauma-related
225	S63271S	Joint disorders and dislocations; trauma-related

225	S63272A	Joint disorders and dislocations; trauma-related
225	S63272D	Joint disorders and dislocations; trauma-related
225	S63272S	Joint disorders and dislocations; trauma-related
225	S63273A	Joint disorders and dislocations; trauma-related
225	S63273D	Joint disorders and dislocations; trauma-related
225	S63273S	Joint disorders and dislocations; trauma-related
225	S63274A	Joint disorders and dislocations; trauma-related
225	S63274D	Joint disorders and dislocations; trauma-related
225	S63274S	Joint disorders and dislocations; trauma-related
225	S63275A	Joint disorders and dislocations; trauma-related
225	S63275D	Joint disorders and dislocations; trauma-related
225	S63275S	Joint disorders and dislocations; trauma-related
225	S63276A	Joint disorders and dislocations; trauma-related
225	S63276D	Joint disorders and dislocations; trauma-related
225	S63276S	Joint disorders and dislocations; trauma-related
225	S63277A	Joint disorders and dislocations; trauma-related
225	S63277D	Joint disorders and dislocations; trauma-related
225	S63277S	Joint disorders and dislocations; trauma-related
225	S63278A	Joint disorders and dislocations; trauma-related
225	S63278D	Joint disorders and dislocations; trauma-related
225	S63278S	Joint disorders and dislocations; trauma-related
225	S63279A	Joint disorders and dislocations; trauma-related
225	S63279D	Joint disorders and dislocations; trauma-related
225	S63279S	Joint disorders and dislocations; trauma-related
225	S63280A	Joint disorders and dislocations; trauma-related
225	S63280D	Joint disorders and dislocations; trauma-related
225	S63280S	Joint disorders and dislocations; trauma-related
225	S63281A	Joint disorders and dislocations; trauma-related
225	S63281D	Joint disorders and dislocations; trauma-related
225	S63281S	Joint disorders and dislocations; trauma-related
225	S63282A	Joint disorders and dislocations; trauma-related
225	S63282D	Joint disorders and dislocations; trauma-related
225	S63282S	Joint disorders and dislocations; trauma-related
225	S63283A	Joint disorders and dislocations; trauma-related
225	S63283D	Joint disorders and dislocations; trauma-related
225	S63283S	Joint disorders and dislocations; trauma-related
225	S63284A	Joint disorders and dislocations; trauma-related
225	S63284D	Joint disorders and dislocations; trauma-related
225	S63284S	Joint disorders and dislocations; trauma-related
225	S63285A	Joint disorders and dislocations; trauma-related
225	S63285D	Joint disorders and dislocations; trauma-related
225	S63285S	Joint disorders and dislocations; trauma-related
225	S63286A	Joint disorders and dislocations; trauma-related
225	S63286D	Joint disorders and dislocations; trauma-related
225	S63286S	Joint disorders and dislocations; trauma-related
225	S63287A	Joint disorders and dislocations; trauma-related
225	S63287D	Joint disorders and dislocations; trauma-related
225	S63287S	Joint disorders and dislocations; trauma-related
225	S63288A	Joint disorders and dislocations; trauma-related
225	S63288D	Joint disorders and dislocations; trauma-related
225	S63288S	Joint disorders and dislocations; trauma-related

225	S63289A	Joint disorders and dislocations; trauma-related
225	S63289D	Joint disorders and dislocations; trauma-related
225	S63289S	Joint disorders and dislocations; trauma-related
225	S63290A	Joint disorders and dislocations; trauma-related
225	S63290D	Joint disorders and dislocations; trauma-related
225	S63290S	Joint disorders and dislocations; trauma-related
225	S63291A	Joint disorders and dislocations; trauma-related
225	S63291D	Joint disorders and dislocations; trauma-related
225	S63291S	Joint disorders and dislocations; trauma-related
225	S63292A	Joint disorders and dislocations; trauma-related
225	S63292D	Joint disorders and dislocations; trauma-related
225	S63292S	Joint disorders and dislocations; trauma-related
225	S63293A	Joint disorders and dislocations; trauma-related
225	S63293D	Joint disorders and dislocations; trauma-related
225	S63293S	Joint disorders and dislocations; trauma-related
225	S63294A	Joint disorders and dislocations; trauma-related
225	S63294D	Joint disorders and dislocations; trauma-related
225	S63294S	Joint disorders and dislocations; trauma-related
225	S63295A	Joint disorders and dislocations; trauma-related
225	S63295D	Joint disorders and dislocations; trauma-related
225	S63295S	Joint disorders and dislocations; trauma-related
225	S63296A	Joint disorders and dislocations; trauma-related
225	S63296D	Joint disorders and dislocations; trauma-related
225	S63296S	Joint disorders and dislocations; trauma-related
225	S63297A	Joint disorders and dislocations; trauma-related
225	S63297D	Joint disorders and dislocations; trauma-related
225	S63297S	Joint disorders and dislocations; trauma-related
225	S63298A	Joint disorders and dislocations; trauma-related
225	S63298D	Joint disorders and dislocations; trauma-related
225	S63298S	Joint disorders and dislocations; trauma-related
225	S63299A	Joint disorders and dislocations; trauma-related
225	S63299D	Joint disorders and dislocations; trauma-related
225	S63299S	Joint disorders and dislocations; trauma-related
225	S73001A	Joint disorders and dislocations; trauma-related
225	S73001D	Joint disorders and dislocations; trauma-related
225	S73001S	Joint disorders and dislocations; trauma-related
225	S73002A	Joint disorders and dislocations; trauma-related
225	S73002D	Joint disorders and dislocations; trauma-related
225	S73002S	Joint disorders and dislocations; trauma-related
225	S73003A	Joint disorders and dislocations; trauma-related
225	S73003D	Joint disorders and dislocations; trauma-related
225	S73003S	Joint disorders and dislocations; trauma-related
225	S73004A	Joint disorders and dislocations; trauma-related
225	S73004D	Joint disorders and dislocations; trauma-related
225	S73004S	Joint disorders and dislocations; trauma-related
225	S73005A	Joint disorders and dislocations; trauma-related
225	S73005D	Joint disorders and dislocations; trauma-related
225	S73005S	Joint disorders and dislocations; trauma-related
225	S73006A	Joint disorders and dislocations; trauma-related
225	S73006D	Joint disorders and dislocations; trauma-related
225	S73006S	Joint disorders and dislocations; trauma-related

225	S73011A	Joint disorders and dislocations; trauma-related
225	S73011D	Joint disorders and dislocations; trauma-related
225	S73011S	Joint disorders and dislocations; trauma-related
225	S73012A	Joint disorders and dislocations; trauma-related
225	S73012D	Joint disorders and dislocations; trauma-related
225	S73012S	Joint disorders and dislocations; trauma-related
225	S73013A	Joint disorders and dislocations; trauma-related
225	S73013D	Joint disorders and dislocations; trauma-related
225	S73013S	Joint disorders and dislocations; trauma-related
225	S73014A	Joint disorders and dislocations; trauma-related
225	S73014D	Joint disorders and dislocations; trauma-related
225	S73014S	Joint disorders and dislocations; trauma-related
225	S73015A	Joint disorders and dislocations; trauma-related
225	S73015D	Joint disorders and dislocations; trauma-related
225	S73015S	Joint disorders and dislocations; trauma-related
225	S73016A	Joint disorders and dislocations; trauma-related
225	S73016D	Joint disorders and dislocations; trauma-related
225	S73016S	Joint disorders and dislocations; trauma-related
225	S73021A	Joint disorders and dislocations; trauma-related
225	S73021D	Joint disorders and dislocations; trauma-related
225	S73021S	Joint disorders and dislocations; trauma-related
225	S73022A	Joint disorders and dislocations; trauma-related
225	S73022D	Joint disorders and dislocations; trauma-related
225	S73022S	Joint disorders and dislocations; trauma-related
225	S73023A	Joint disorders and dislocations; trauma-related
225	S73023D	Joint disorders and dislocations; trauma-related
225	S73023S	Joint disorders and dislocations; trauma-related
225	S73024A	Joint disorders and dislocations; trauma-related
225	S73024D	Joint disorders and dislocations; trauma-related
225	S73024S	Joint disorders and dislocations; trauma-related
225	S73025A	Joint disorders and dislocations; trauma-related
225	S73025D	Joint disorders and dislocations; trauma-related
225	S73025S	Joint disorders and dislocations; trauma-related
225	S73026A	Joint disorders and dislocations; trauma-related
225	S73026D	Joint disorders and dislocations; trauma-related
225	S73026S	Joint disorders and dislocations; trauma-related
225	S73031A	Joint disorders and dislocations; trauma-related
225	S73031D	Joint disorders and dislocations; trauma-related
225	S73031S	Joint disorders and dislocations; trauma-related
225	S73032A	Joint disorders and dislocations; trauma-related
225	S73032D	Joint disorders and dislocations; trauma-related
225	S73032S	Joint disorders and dislocations; trauma-related
225	S73033A	Joint disorders and dislocations; trauma-related
225	S73033D	Joint disorders and dislocations; trauma-related
225	S73033S	Joint disorders and dislocations; trauma-related
225	S73034A	Joint disorders and dislocations; trauma-related
225	S73034D	Joint disorders and dislocations; trauma-related
225	S73034S	Joint disorders and dislocations; trauma-related
225	S73035A	Joint disorders and dislocations; trauma-related
225	S73035D	Joint disorders and dislocations; trauma-related
225	S73035S	Joint disorders and dislocations; trauma-related

225	S73036A	Joint disorders and dislocations; trauma-related
225	S73036D	Joint disorders and dislocations; trauma-related
225	S73036S	Joint disorders and dislocations; trauma-related
225	S73041A	Joint disorders and dislocations; trauma-related
225	S73041D	Joint disorders and dislocations; trauma-related
225	S73041S	Joint disorders and dislocations; trauma-related
225	S73042A	Joint disorders and dislocations; trauma-related
225	S73042D	Joint disorders and dislocations; trauma-related
225	S73042S	Joint disorders and dislocations; trauma-related
225	S73043A	Joint disorders and dislocations; trauma-related
225	S73043D	Joint disorders and dislocations; trauma-related
225	S73043S	Joint disorders and dislocations; trauma-related
225	S73044A	Joint disorders and dislocations; trauma-related
225	S73044D	Joint disorders and dislocations; trauma-related
225	S73044S	Joint disorders and dislocations; trauma-related
225	S73045A	Joint disorders and dislocations; trauma-related
225	S73045D	Joint disorders and dislocations; trauma-related
225	S73045S	Joint disorders and dislocations; trauma-related
225	S73046A	Joint disorders and dislocations; trauma-related
225	S73046D	Joint disorders and dislocations; trauma-related
225	S73046S	Joint disorders and dislocations; trauma-related
225	S83001A	Joint disorders and dislocations; trauma-related
225	S83001D	Joint disorders and dislocations; trauma-related
225	S83001S	Joint disorders and dislocations; trauma-related
225	S83002A	Joint disorders and dislocations; trauma-related
225	S83002D	Joint disorders and dislocations; trauma-related
225	S83002S	Joint disorders and dislocations; trauma-related
225	S83003A	Joint disorders and dislocations; trauma-related
225	S83003D	Joint disorders and dislocations; trauma-related
225	S83003S	Joint disorders and dislocations; trauma-related
225	S83004A	Joint disorders and dislocations; trauma-related
225	S83004D	Joint disorders and dislocations; trauma-related
225	S83004S	Joint disorders and dislocations; trauma-related
225	S83005A	Joint disorders and dislocations; trauma-related
225	S83005D	Joint disorders and dislocations; trauma-related
225	S83005S	Joint disorders and dislocations; trauma-related
225	S83006A	Joint disorders and dislocations; trauma-related
225	S83006D	Joint disorders and dislocations; trauma-related
225	S83006S	Joint disorders and dislocations; trauma-related
225	S83011A	Joint disorders and dislocations; trauma-related
225	S83011D	Joint disorders and dislocations; trauma-related
225	S83011S	Joint disorders and dislocations; trauma-related
225	S83012A	Joint disorders and dislocations; trauma-related
225	S83012D	Joint disorders and dislocations; trauma-related
225	S83012S	Joint disorders and dislocations; trauma-related
225	S83013A	Joint disorders and dislocations; trauma-related
225	S83013D	Joint disorders and dislocations; trauma-related
225	S83013S	Joint disorders and dislocations; trauma-related
225	S83014A	Joint disorders and dislocations; trauma-related
225	S83014D	Joint disorders and dislocations; trauma-related
225	S83014S	Joint disorders and dislocations; trauma-related

225	S83015A	Joint disorders and dislocations; trauma-related
225	S83015D	Joint disorders and dislocations; trauma-related
225	S83015S	Joint disorders and dislocations; trauma-related
225	S83016A	Joint disorders and dislocations; trauma-related
225	S83016D	Joint disorders and dislocations; trauma-related
225	S83016S	Joint disorders and dislocations; trauma-related
225	S83091A	Joint disorders and dislocations; trauma-related
225	S83091D	Joint disorders and dislocations; trauma-related
225	S83091S	Joint disorders and dislocations; trauma-related
225	S83092A	Joint disorders and dislocations; trauma-related
225	S83092D	Joint disorders and dislocations; trauma-related
225	S83092S	Joint disorders and dislocations; trauma-related
225	S83093A	Joint disorders and dislocations; trauma-related
225	S83093D	Joint disorders and dislocations; trauma-related
225	S83093S	Joint disorders and dislocations; trauma-related
225	S83094A	Joint disorders and dislocations; trauma-related
225	S83094D	Joint disorders and dislocations; trauma-related
225	S83094S	Joint disorders and dislocations; trauma-related
225	S83095A	Joint disorders and dislocations; trauma-related
225	S83095D	Joint disorders and dislocations; trauma-related
225	S83095S	Joint disorders and dislocations; trauma-related
225	S83096A	Joint disorders and dislocations; trauma-related
225	S83096D	Joint disorders and dislocations; trauma-related
225	S83096S	Joint disorders and dislocations; trauma-related
225	S83101A	Joint disorders and dislocations; trauma-related
225	S83101D	Joint disorders and dislocations; trauma-related
225	S83101S	Joint disorders and dislocations; trauma-related
225	S83102A	Joint disorders and dislocations; trauma-related
225	S83102D	Joint disorders and dislocations; trauma-related
225	S83102S	Joint disorders and dislocations; trauma-related
225	S83103A	Joint disorders and dislocations; trauma-related
225	S83103D	Joint disorders and dislocations; trauma-related
225	S83103S	Joint disorders and dislocations; trauma-related
225	S83104A	Joint disorders and dislocations; trauma-related
225	S83104D	Joint disorders and dislocations; trauma-related
225	S83104S	Joint disorders and dislocations; trauma-related
225	S83105A	Joint disorders and dislocations; trauma-related
225	S83105D	Joint disorders and dislocations; trauma-related
225	S83105S	Joint disorders and dislocations; trauma-related
225	S83106A	Joint disorders and dislocations; trauma-related
225	S83106D	Joint disorders and dislocations; trauma-related
225	S83106S	Joint disorders and dislocations; trauma-related
225	S83111A	Joint disorders and dislocations; trauma-related
225	S83111D	Joint disorders and dislocations; trauma-related
225	S83111S	Joint disorders and dislocations; trauma-related
225	S83112A	Joint disorders and dislocations; trauma-related
225	S83112D	Joint disorders and dislocations; trauma-related
225	S83112S	Joint disorders and dislocations; trauma-related
225	S83113A	Joint disorders and dislocations; trauma-related
225	S83113D	Joint disorders and dislocations; trauma-related
225	S83113S	Joint disorders and dislocations; trauma-related

225	S83114A	Joint disorders and dislocations; trauma-related
225	S83114D	Joint disorders and dislocations; trauma-related
225	S83114S	Joint disorders and dislocations; trauma-related
225	S83115A	Joint disorders and dislocations; trauma-related
225	S83115D	Joint disorders and dislocations; trauma-related
225	S83115S	Joint disorders and dislocations; trauma-related
225	S83116A	Joint disorders and dislocations; trauma-related
225	S83116D	Joint disorders and dislocations; trauma-related
225	S83116S	Joint disorders and dislocations; trauma-related
225	S83121A	Joint disorders and dislocations; trauma-related
225	S83121D	Joint disorders and dislocations; trauma-related
225	S83121S	Joint disorders and dislocations; trauma-related
225	S83122A	Joint disorders and dislocations; trauma-related
225	S83122D	Joint disorders and dislocations; trauma-related
225	S83122S	Joint disorders and dislocations; trauma-related
225	S83123A	Joint disorders and dislocations; trauma-related
225	S83123D	Joint disorders and dislocations; trauma-related
225	S83123S	Joint disorders and dislocations; trauma-related
225	S83124A	Joint disorders and dislocations; trauma-related
225	S83124D	Joint disorders and dislocations; trauma-related
225	S83124S	Joint disorders and dislocations; trauma-related
225	S83125A	Joint disorders and dislocations; trauma-related
225	S83125D	Joint disorders and dislocations; trauma-related
225	S83125S	Joint disorders and dislocations; trauma-related
225	S83126A	Joint disorders and dislocations; trauma-related
225	S83126D	Joint disorders and dislocations; trauma-related
225	S83126S	Joint disorders and dislocations; trauma-related
225	S83131A	Joint disorders and dislocations; trauma-related
225	S83131D	Joint disorders and dislocations; trauma-related
225	S83131S	Joint disorders and dislocations; trauma-related
225	S83132A	Joint disorders and dislocations; trauma-related
225	S83132D	Joint disorders and dislocations; trauma-related
225	S83132S	Joint disorders and dislocations; trauma-related
225	S83133A	Joint disorders and dislocations; trauma-related
225	S83133D	Joint disorders and dislocations; trauma-related
225	S83133S	Joint disorders and dislocations; trauma-related
225	S83134A	Joint disorders and dislocations; trauma-related
225	S83134D	Joint disorders and dislocations; trauma-related
225	S83134S	Joint disorders and dislocations; trauma-related
225	S83135A	Joint disorders and dislocations; trauma-related
225	S83135D	Joint disorders and dislocations; trauma-related
225	S83135S	Joint disorders and dislocations; trauma-related
225	S83136A	Joint disorders and dislocations; trauma-related
225	S83136D	Joint disorders and dislocations; trauma-related
225	S83136S	Joint disorders and dislocations; trauma-related
225	S83141A	Joint disorders and dislocations; trauma-related
225	S83141D	Joint disorders and dislocations; trauma-related
225	S83141S	Joint disorders and dislocations; trauma-related
225	S83142A	Joint disorders and dislocations; trauma-related
225	S83142D	Joint disorders and dislocations; trauma-related
225	S83142S	Joint disorders and dislocations; trauma-related

225	S83143A	Joint disorders and dislocations; trauma-related
225	S83143D	Joint disorders and dislocations; trauma-related
225	S83143S	Joint disorders and dislocations; trauma-related
225	S83144A	Joint disorders and dislocations; trauma-related
225	S83144D	Joint disorders and dislocations; trauma-related
225	S83144S	Joint disorders and dislocations; trauma-related
225	S83145A	Joint disorders and dislocations; trauma-related
225	S83145D	Joint disorders and dislocations; trauma-related
225	S83145S	Joint disorders and dislocations; trauma-related
225	S83146A	Joint disorders and dislocations; trauma-related
225	S83146D	Joint disorders and dislocations; trauma-related
225	S83146S	Joint disorders and dislocations; trauma-related
225	S83191A	Joint disorders and dislocations; trauma-related
225	S83191D	Joint disorders and dislocations; trauma-related
225	S83191S	Joint disorders and dislocations; trauma-related
225	S83192A	Joint disorders and dislocations; trauma-related
225	S83192D	Joint disorders and dislocations; trauma-related
225	S83192S	Joint disorders and dislocations; trauma-related
225	S83193A	Joint disorders and dislocations; trauma-related
225	S83193D	Joint disorders and dislocations; trauma-related
225	S83193S	Joint disorders and dislocations; trauma-related
225	S83194A	Joint disorders and dislocations; trauma-related
225	S83194D	Joint disorders and dislocations; trauma-related
225	S83194S	Joint disorders and dislocations; trauma-related
225	S83195A	Joint disorders and dislocations; trauma-related
225	S83195D	Joint disorders and dislocations; trauma-related
225	S83195S	Joint disorders and dislocations; trauma-related
225	S83196A	Joint disorders and dislocations; trauma-related
225	S83196D	Joint disorders and dislocations; trauma-related
225	S83196S	Joint disorders and dislocations; trauma-related
225	S83200A	Joint disorders and dislocations; trauma-related
225	S83200D	Joint disorders and dislocations; trauma-related
225	S83200S	Joint disorders and dislocations; trauma-related
225	S83201A	Joint disorders and dislocations; trauma-related
225	S83201D	Joint disorders and dislocations; trauma-related
225	S83201S	Joint disorders and dislocations; trauma-related
225	S83202A	Joint disorders and dislocations; trauma-related
225	S83202D	Joint disorders and dislocations; trauma-related
225	S83202S	Joint disorders and dislocations; trauma-related
225	S83203A	Joint disorders and dislocations; trauma-related
225	S83203D	Joint disorders and dislocations; trauma-related
225	S83203S	Joint disorders and dislocations; trauma-related
225	S83204A	Joint disorders and dislocations; trauma-related
225	S83204D	Joint disorders and dislocations; trauma-related
225	S83204S	Joint disorders and dislocations; trauma-related
225	S83205A	Joint disorders and dislocations; trauma-related
225	S83205D	Joint disorders and dislocations; trauma-related
225	S83205S	Joint disorders and dislocations; trauma-related
225	S83206A	Joint disorders and dislocations; trauma-related
225	S83206D	Joint disorders and dislocations; trauma-related
225	S83206S	Joint disorders and dislocations; trauma-related

225	S83207A	Joint disorders and dislocations; trauma-related
225	S83207D	Joint disorders and dislocations; trauma-related
225	S83207S	Joint disorders and dislocations; trauma-related
225	S83209A	Joint disorders and dislocations; trauma-related
225	S83209D	Joint disorders and dislocations; trauma-related
225	S83209S	Joint disorders and dislocations; trauma-related
225	S83211A	Joint disorders and dislocations; trauma-related
225	S83211D	Joint disorders and dislocations; trauma-related
225	S83211S	Joint disorders and dislocations; trauma-related
225	S83212A	Joint disorders and dislocations; trauma-related
225	S83212D	Joint disorders and dislocations; trauma-related
225	S83212S	Joint disorders and dislocations; trauma-related
225	S83219A	Joint disorders and dislocations; trauma-related
225	S83219D	Joint disorders and dislocations; trauma-related
225	S83219S	Joint disorders and dislocations; trauma-related
225	S83221A	Joint disorders and dislocations; trauma-related
225	S83221D	Joint disorders and dislocations; trauma-related
225	S83221S	Joint disorders and dislocations; trauma-related
225	S83222A	Joint disorders and dislocations; trauma-related
225	S83222D	Joint disorders and dislocations; trauma-related
225	S83222S	Joint disorders and dislocations; trauma-related
225	S83229A	Joint disorders and dislocations; trauma-related
225	S83229D	Joint disorders and dislocations; trauma-related
225	S83229S	Joint disorders and dislocations; trauma-related
225	S83231A	Joint disorders and dislocations; trauma-related
225	S83231D	Joint disorders and dislocations; trauma-related
225	S83231S	Joint disorders and dislocations; trauma-related
225	S83232A	Joint disorders and dislocations; trauma-related
225	S83232D	Joint disorders and dislocations; trauma-related
225	S83232S	Joint disorders and dislocations; trauma-related
225	S83239A	Joint disorders and dislocations; trauma-related
225	S83239D	Joint disorders and dislocations; trauma-related
225	S83239S	Joint disorders and dislocations; trauma-related
225	S83241A	Joint disorders and dislocations; trauma-related
225	S83241D	Joint disorders and dislocations; trauma-related
225	S83241S	Joint disorders and dislocations; trauma-related
225	S83242A	Joint disorders and dislocations; trauma-related
225	S83242D	Joint disorders and dislocations; trauma-related
225	S83242S	Joint disorders and dislocations; trauma-related
225	S83249A	Joint disorders and dislocations; trauma-related
225	S83249D	Joint disorders and dislocations; trauma-related
225	S83249S	Joint disorders and dislocations; trauma-related
225	S83251A	Joint disorders and dislocations; trauma-related
225	S83251D	Joint disorders and dislocations; trauma-related
225	S83251S	Joint disorders and dislocations; trauma-related
225	S83252A	Joint disorders and dislocations; trauma-related
225	S83252D	Joint disorders and dislocations; trauma-related
225	S83252S	Joint disorders and dislocations; trauma-related
225	S83259A	Joint disorders and dislocations; trauma-related
225	S83259D	Joint disorders and dislocations; trauma-related
225	S83259S	Joint disorders and dislocations; trauma-related

225	S83261A	Joint disorders and dislocations; trauma-related
225	S83261D	Joint disorders and dislocations; trauma-related
225	S83261S	Joint disorders and dislocations; trauma-related
225	S83262A	Joint disorders and dislocations; trauma-related
225	S83262D	Joint disorders and dislocations; trauma-related
225	S83262S	Joint disorders and dislocations; trauma-related
225	S83269A	Joint disorders and dislocations; trauma-related
225	S83269D	Joint disorders and dislocations; trauma-related
225	S83269S	Joint disorders and dislocations; trauma-related
225	S83271A	Joint disorders and dislocations; trauma-related
225	S83271D	Joint disorders and dislocations; trauma-related
225	S83271S	Joint disorders and dislocations; trauma-related
225	S83272A	Joint disorders and dislocations; trauma-related
225	S83272D	Joint disorders and dislocations; trauma-related
225	S83272S	Joint disorders and dislocations; trauma-related
225	S83279A	Joint disorders and dislocations; trauma-related
225	S83279D	Joint disorders and dislocations; trauma-related
225	S83279S	Joint disorders and dislocations; trauma-related
225	S83281A	Joint disorders and dislocations; trauma-related
225	S83281D	Joint disorders and dislocations; trauma-related
225	S83281S	Joint disorders and dislocations; trauma-related
225	S83282A	Joint disorders and dislocations; trauma-related
225	S83282D	Joint disorders and dislocations; trauma-related
225	S83282S	Joint disorders and dislocations; trauma-related
225	S83289A	Joint disorders and dislocations; trauma-related
225	S83289D	Joint disorders and dislocations; trauma-related
225	S83289S	Joint disorders and dislocations; trauma-related
225	S8330XA	Joint disorders and dislocations; trauma-related
225	S8330XD	Joint disorders and dislocations; trauma-related
225	S8330XS	Joint disorders and dislocations; trauma-related
225	S8331XA	Joint disorders and dislocations; trauma-related
225	S8331XD	Joint disorders and dislocations; trauma-related
225	S8331XS	Joint disorders and dislocations; trauma-related
225	S8332XA	Joint disorders and dislocations; trauma-related
225	S8332XD	Joint disorders and dislocations; trauma-related
225	S8332XS	Joint disorders and dislocations; trauma-related
225	S9301XA	Joint disorders and dislocations; trauma-related
225	S9301XD	Joint disorders and dislocations; trauma-related
225	S9301XS	Joint disorders and dislocations; trauma-related
225	S9302XA	Joint disorders and dislocations; trauma-related
225	S9302XD	Joint disorders and dislocations; trauma-related
225	S9302XS	Joint disorders and dislocations; trauma-related
225	S9303XA	Joint disorders and dislocations; trauma-related
225	S9303XD	Joint disorders and dislocations; trauma-related
225	S9303XS	Joint disorders and dislocations; trauma-related
225	S9304XA	Joint disorders and dislocations; trauma-related
225	S9304XD	Joint disorders and dislocations; trauma-related
225	S9304XS	Joint disorders and dislocations; trauma-related
225	S9305XA	Joint disorders and dislocations; trauma-related
225	S9305XD	Joint disorders and dislocations; trauma-related
225	S9305XS	Joint disorders and dislocations; trauma-related

225	S9306XA	Joint disorders and dislocations; trauma-related
225	S9306XD	Joint disorders and dislocations; trauma-related
225	S9306XS	Joint disorders and dislocations; trauma-related
225	S93101A	Joint disorders and dislocations; trauma-related
225	S93101D	Joint disorders and dislocations; trauma-related
225	S93101S	Joint disorders and dislocations; trauma-related
225	S93102A	Joint disorders and dislocations; trauma-related
225	S93102D	Joint disorders and dislocations; trauma-related
225	S93102S	Joint disorders and dislocations; trauma-related
225	S93103A	Joint disorders and dislocations; trauma-related
225	S93103D	Joint disorders and dislocations; trauma-related
225	S93103S	Joint disorders and dislocations; trauma-related
225	S93104A	Joint disorders and dislocations; trauma-related
225	S93104D	Joint disorders and dislocations; trauma-related
225	S93104S	Joint disorders and dislocations; trauma-related
225	S93105A	Joint disorders and dislocations; trauma-related
225	S93105D	Joint disorders and dislocations; trauma-related
225	S93105S	Joint disorders and dislocations; trauma-related
225	S93106A	Joint disorders and dislocations; trauma-related
225	S93106D	Joint disorders and dislocations; trauma-related
225	S93106S	Joint disorders and dislocations; trauma-related
225	S93111A	Joint disorders and dislocations; trauma-related
225	S93111D	Joint disorders and dislocations; trauma-related
225	S93111S	Joint disorders and dislocations; trauma-related
225	S93112A	Joint disorders and dislocations; trauma-related
225	S93112D	Joint disorders and dislocations; trauma-related
225	S93112S	Joint disorders and dislocations; trauma-related
225	S93113A	Joint disorders and dislocations; trauma-related
225	S93113D	Joint disorders and dislocations; trauma-related
225	S93113S	Joint disorders and dislocations; trauma-related
225	S93114A	Joint disorders and dislocations; trauma-related
225	S93114D	Joint disorders and dislocations; trauma-related
225	S93114S	Joint disorders and dislocations; trauma-related
225	S93115A	Joint disorders and dislocations; trauma-related
225	S93115D	Joint disorders and dislocations; trauma-related
225	S93115S	Joint disorders and dislocations; trauma-related
225	S93116A	Joint disorders and dislocations; trauma-related
225	S93116D	Joint disorders and dislocations; trauma-related
225	S93116S	Joint disorders and dislocations; trauma-related
225	S93119A	Joint disorders and dislocations; trauma-related
225	S93119D	Joint disorders and dislocations; trauma-related
225	S93119S	Joint disorders and dislocations; trauma-related
225	S93121A	Joint disorders and dislocations; trauma-related
225	S93121D	Joint disorders and dislocations; trauma-related
225	S93121S	Joint disorders and dislocations; trauma-related
225	S93122A	Joint disorders and dislocations; trauma-related
225	S93122D	Joint disorders and dislocations; trauma-related
225	S93122S	Joint disorders and dislocations; trauma-related
225	S93123A	Joint disorders and dislocations; trauma-related
225	S93123D	Joint disorders and dislocations; trauma-related
225	S93123S	Joint disorders and dislocations; trauma-related

225	S93124A	Joint disorders and dislocations; trauma-related
225	S93124D	Joint disorders and dislocations; trauma-related
225	S93124S	Joint disorders and dislocations; trauma-related
225	S93125A	Joint disorders and dislocations; trauma-related
225	S93125D	Joint disorders and dislocations; trauma-related
225	S93125S	Joint disorders and dislocations; trauma-related
225	S93126A	Joint disorders and dislocations; trauma-related
225	S93126D	Joint disorders and dislocations; trauma-related
225	S93126S	Joint disorders and dislocations; trauma-related
225	S93129A	Joint disorders and dislocations; trauma-related
225	S93129D	Joint disorders and dislocations; trauma-related
225	S93129S	Joint disorders and dislocations; trauma-related
225	S93131A	Joint disorders and dislocations; trauma-related
225	S93131D	Joint disorders and dislocations; trauma-related
225	S93131S	Joint disorders and dislocations; trauma-related
225	S93132A	Joint disorders and dislocations; trauma-related
225	S93132D	Joint disorders and dislocations; trauma-related
225	S93132S	Joint disorders and dislocations; trauma-related
225	S93133A	Joint disorders and dislocations; trauma-related
225	S93133D	Joint disorders and dislocations; trauma-related
225	S93133S	Joint disorders and dislocations; trauma-related
225	S93134A	Joint disorders and dislocations; trauma-related
225	S93134D	Joint disorders and dislocations; trauma-related
225	S93134S	Joint disorders and dislocations; trauma-related
225	S93135A	Joint disorders and dislocations; trauma-related
225	S93135D	Joint disorders and dislocations; trauma-related
225	S93135S	Joint disorders and dislocations; trauma-related
225	S93136A	Joint disorders and dislocations; trauma-related
225	S93136D	Joint disorders and dislocations; trauma-related
225	S93136S	Joint disorders and dislocations; trauma-related
225	S93139A	Joint disorders and dislocations; trauma-related
225	S93139D	Joint disorders and dislocations; trauma-related
225	S93139S	Joint disorders and dislocations; trauma-related
225	S93141A	Joint disorders and dislocations; trauma-related
225	S93141D	Joint disorders and dislocations; trauma-related
225	S93141S	Joint disorders and dislocations; trauma-related
225	S93142A	Joint disorders and dislocations; trauma-related
225	S93142D	Joint disorders and dislocations; trauma-related
225	S93142S	Joint disorders and dislocations; trauma-related
225	S93143A	Joint disorders and dislocations; trauma-related
225	S93143D	Joint disorders and dislocations; trauma-related
225	S93143S	Joint disorders and dislocations; trauma-related
225	S93144A	Joint disorders and dislocations; trauma-related
225	S93144D	Joint disorders and dislocations; trauma-related
225	S93144S	Joint disorders and dislocations; trauma-related
225	S93145A	Joint disorders and dislocations; trauma-related
225	S93145D	Joint disorders and dislocations; trauma-related
225	S93145S	Joint disorders and dislocations; trauma-related
225	S93146A	Joint disorders and dislocations; trauma-related
225	S93146D	Joint disorders and dislocations; trauma-related
225	S93146S	Joint disorders and dislocations; trauma-related

225	S93149A	Joint disorders and dislocations; trauma-related
225	S93149D	Joint disorders and dislocations; trauma-related
225	S93149S	Joint disorders and dislocations; trauma-related
225	S93301A	Joint disorders and dislocations; trauma-related
225	S93301D	Joint disorders and dislocations; trauma-related
225	S93301S	Joint disorders and dislocations; trauma-related
225	S93302A	Joint disorders and dislocations; trauma-related
225	S93302D	Joint disorders and dislocations; trauma-related
225	S93302S	Joint disorders and dislocations; trauma-related
225	S93303A	Joint disorders and dislocations; trauma-related
225	S93303D	Joint disorders and dislocations; trauma-related
225	S93303S	Joint disorders and dislocations; trauma-related
225	S93304A	Joint disorders and dislocations; trauma-related
225	S93304D	Joint disorders and dislocations; trauma-related
225	S93304S	Joint disorders and dislocations; trauma-related
225	S93305A	Joint disorders and dislocations; trauma-related
225	S93305D	Joint disorders and dislocations; trauma-related
225	S93305S	Joint disorders and dislocations; trauma-related
225	S93306A	Joint disorders and dislocations; trauma-related
225	S93306D	Joint disorders and dislocations; trauma-related
225	S93306S	Joint disorders and dislocations; trauma-related
225	S93311A	Joint disorders and dislocations; trauma-related
225	S93311D	Joint disorders and dislocations; trauma-related
225	S93311S	Joint disorders and dislocations; trauma-related
225	S93312A	Joint disorders and dislocations; trauma-related
225	S93312D	Joint disorders and dislocations; trauma-related
225	S93312S	Joint disorders and dislocations; trauma-related
225	S93313A	Joint disorders and dislocations; trauma-related
225	S93313D	Joint disorders and dislocations; trauma-related
225	S93313S	Joint disorders and dislocations; trauma-related
225	S93314A	Joint disorders and dislocations; trauma-related
225	S93314D	Joint disorders and dislocations; trauma-related
225	S93314S	Joint disorders and dislocations; trauma-related
225	S93315A	Joint disorders and dislocations; trauma-related
225	S93315D	Joint disorders and dislocations; trauma-related
225	S93315S	Joint disorders and dislocations; trauma-related
225	S93316A	Joint disorders and dislocations; trauma-related
225	S93316D	Joint disorders and dislocations; trauma-related
225	S93316S	Joint disorders and dislocations; trauma-related
225	S93321A	Joint disorders and dislocations; trauma-related
225	S93321D	Joint disorders and dislocations; trauma-related
225	S93321S	Joint disorders and dislocations; trauma-related
225	S93322A	Joint disorders and dislocations; trauma-related
225	S93322D	Joint disorders and dislocations; trauma-related
225	S93322S	Joint disorders and dislocations; trauma-related
225	S93323A	Joint disorders and dislocations; trauma-related
225	S93323D	Joint disorders and dislocations; trauma-related
225	S93323S	Joint disorders and dislocations; trauma-related
225	S93324A	Joint disorders and dislocations; trauma-related
225	S93324D	Joint disorders and dislocations; trauma-related
225	S93324S	Joint disorders and dislocations; trauma-related

225	S93325A	Joint disorders and dislocations; trauma-related
225	S93325D	Joint disorders and dislocations; trauma-related
225	S93325S	Joint disorders and dislocations; trauma-related
225	S93326A	Joint disorders and dislocations; trauma-related
225	S93326D	Joint disorders and dislocations; trauma-related
225	S93326S	Joint disorders and dislocations; trauma-related
225	S93331A	Joint disorders and dislocations; trauma-related
225	S93331D	Joint disorders and dislocations; trauma-related
225	S93331S	Joint disorders and dislocations; trauma-related
225	S93332A	Joint disorders and dislocations; trauma-related
225	S93332D	Joint disorders and dislocations; trauma-related
225	S93332S	Joint disorders and dislocations; trauma-related
225	S93333A	Joint disorders and dislocations; trauma-related
225	S93333D	Joint disorders and dislocations; trauma-related
225	S93333S	Joint disorders and dislocations; trauma-related
225	S93334A	Joint disorders and dislocations; trauma-related
225	S93334D	Joint disorders and dislocations; trauma-related
225	S93334S	Joint disorders and dislocations; trauma-related
225	S93335A	Joint disorders and dislocations; trauma-related
225	S93335D	Joint disorders and dislocations; trauma-related
225	S93335S	Joint disorders and dislocations; trauma-related
225	S93336A	Joint disorders and dislocations; trauma-related
225	S93336D	Joint disorders and dislocations; trauma-related
225	S93336S	Joint disorders and dislocations; trauma-related
226	M84350K	Fracture of neck of femur (hip)
226	M84350P	Fracture of neck of femur (hip)
226	M84351A	Fracture of neck of femur (hip)
226	M84351D	Fracture of neck of femur (hip)
226	M84351G	Fracture of neck of femur (hip)
226	M84351K	Fracture of neck of femur (hip)
226	M84351P	Fracture of neck of femur (hip)
226	M84351S	Fracture of neck of femur (hip)
226	M84352A	Fracture of neck of femur (hip)
226	M84352D	Fracture of neck of femur (hip)
226	M84352G	Fracture of neck of femur (hip)
226	M84352K	Fracture of neck of femur (hip)
226	M84352P	Fracture of neck of femur (hip)
226	M84352S	Fracture of neck of femur (hip)
226	M84353A	Fracture of neck of femur (hip)
226	M84353D	Fracture of neck of femur (hip)
226	M84353G	Fracture of neck of femur (hip)
226	M84353K	Fracture of neck of femur (hip)
226	M84353P	Fracture of neck of femur (hip)
226	M84353S	Fracture of neck of femur (hip)
226	M84359A	Fracture of neck of femur (hip)
226	M84359D	Fracture of neck of femur (hip)
226	M84359G	Fracture of neck of femur (hip)
226	M84359K	Fracture of neck of femur (hip)
226	M84359P	Fracture of neck of femur (hip)
226	M84359S	Fracture of neck of femur (hip)
226	S72001A	Fracture of neck of femur (hip)

226	S72001B	Fracture of neck of femur (hip)
226	S72001C	Fracture of neck of femur (hip)
226	S72001D	Fracture of neck of femur (hip)
226	S72001E	Fracture of neck of femur (hip)
226	S72001F	Fracture of neck of femur (hip)
226	S72001G	Fracture of neck of femur (hip)
226	S72001H	Fracture of neck of femur (hip)
226	S72001J	Fracture of neck of femur (hip)
226	S72001K	Fracture of neck of femur (hip)
226	S72001M	Fracture of neck of femur (hip)
226	S72001N	Fracture of neck of femur (hip)
226	S72001P	Fracture of neck of femur (hip)
226	S72001Q	Fracture of neck of femur (hip)
226	S72001R	Fracture of neck of femur (hip)
226	S72001S	Fracture of neck of femur (hip)
226	S72002A	Fracture of neck of femur (hip)
226	S72002B	Fracture of neck of femur (hip)
226	S72002C	Fracture of neck of femur (hip)
226	S72002D	Fracture of neck of femur (hip)
226	S72002E	Fracture of neck of femur (hip)
226	S72002F	Fracture of neck of femur (hip)
226	S72002G	Fracture of neck of femur (hip)
226	S72002H	Fracture of neck of femur (hip)
226	S72002J	Fracture of neck of femur (hip)
226	S72002K	Fracture of neck of femur (hip)
226	S72002M	Fracture of neck of femur (hip)
226	S72002N	Fracture of neck of femur (hip)
226	S72002P	Fracture of neck of femur (hip)
226	S72002Q	Fracture of neck of femur (hip)
226	S72002R	Fracture of neck of femur (hip)
226	S72002S	Fracture of neck of femur (hip)
226	S72009A	Fracture of neck of femur (hip)
226	S72009B	Fracture of neck of femur (hip)
226	S72009C	Fracture of neck of femur (hip)
226	S72009D	Fracture of neck of femur (hip)
226	S72009E	Fracture of neck of femur (hip)
226	S72009F	Fracture of neck of femur (hip)
226	S72009G	Fracture of neck of femur (hip)
226	S72009H	Fracture of neck of femur (hip)
226	S72009J	Fracture of neck of femur (hip)
226	S72009K	Fracture of neck of femur (hip)
226	S72009M	Fracture of neck of femur (hip)
226	S72009N	Fracture of neck of femur (hip)
226	S72009P	Fracture of neck of femur (hip)
226	S72009Q	Fracture of neck of femur (hip)
226	S72009R	Fracture of neck of femur (hip)
226	S72009S	Fracture of neck of femur (hip)
226	S72011A	Fracture of neck of femur (hip)
226	S72011B	Fracture of neck of femur (hip)
226	S72011C	Fracture of neck of femur (hip)
226	S72011D	Fracture of neck of femur (hip)

226	S72011E	Fracture of neck of femur (hip)
226	S72011F	Fracture of neck of femur (hip)
226	S72011G	Fracture of neck of femur (hip)
226	S72011H	Fracture of neck of femur (hip)
226	S72011J	Fracture of neck of femur (hip)
226	S72011K	Fracture of neck of femur (hip)
226	S72011M	Fracture of neck of femur (hip)
226	S72011N	Fracture of neck of femur (hip)
226	S72011P	Fracture of neck of femur (hip)
226	S72011Q	Fracture of neck of femur (hip)
226	S72011R	Fracture of neck of femur (hip)
226	S72011S	Fracture of neck of femur (hip)
226	S72012A	Fracture of neck of femur (hip)
226	S72012B	Fracture of neck of femur (hip)
226	S72012C	Fracture of neck of femur (hip)
226	S72012D	Fracture of neck of femur (hip)
226	S72012E	Fracture of neck of femur (hip)
226	S72012F	Fracture of neck of femur (hip)
226	S72012G	Fracture of neck of femur (hip)
226	S72012H	Fracture of neck of femur (hip)
226	S72012J	Fracture of neck of femur (hip)
226	S72012K	Fracture of neck of femur (hip)
226	S72012M	Fracture of neck of femur (hip)
226	S72012N	Fracture of neck of femur (hip)
226	S72012P	Fracture of neck of femur (hip)
226	S72012Q	Fracture of neck of femur (hip)
226	S72012R	Fracture of neck of femur (hip)
226	S72012S	Fracture of neck of femur (hip)
226	S72019A	Fracture of neck of femur (hip)
226	S72019B	Fracture of neck of femur (hip)
226	S72019C	Fracture of neck of femur (hip)
226	S72019D	Fracture of neck of femur (hip)
226	S72019E	Fracture of neck of femur (hip)
226	S72019F	Fracture of neck of femur (hip)
226	S72019G	Fracture of neck of femur (hip)
226	S72019H	Fracture of neck of femur (hip)
226	S72019J	Fracture of neck of femur (hip)
226	S72019K	Fracture of neck of femur (hip)
226	S72019M	Fracture of neck of femur (hip)
226	S72019N	Fracture of neck of femur (hip)
226	S72019P	Fracture of neck of femur (hip)
226	S72019Q	Fracture of neck of femur (hip)
226	S72019R	Fracture of neck of femur (hip)
226	S72019S	Fracture of neck of femur (hip)
226	S72021A	Fracture of neck of femur (hip)
226	S72021B	Fracture of neck of femur (hip)
226	S72021C	Fracture of neck of femur (hip)
226	S72021D	Fracture of neck of femur (hip)
226	S72021E	Fracture of neck of femur (hip)
226	S72021F	Fracture of neck of femur (hip)
226	S72021G	Fracture of neck of femur (hip)

226	S72021H	Fracture of neck of femur (hip)
226	S72021J	Fracture of neck of femur (hip)
226	S72021K	Fracture of neck of femur (hip)
226	S72021M	Fracture of neck of femur (hip)
226	S72021N	Fracture of neck of femur (hip)
226	S72021P	Fracture of neck of femur (hip)
226	S72021Q	Fracture of neck of femur (hip)
226	S72021R	Fracture of neck of femur (hip)
226	S72021S	Fracture of neck of femur (hip)
226	S72022A	Fracture of neck of femur (hip)
226	S72022B	Fracture of neck of femur (hip)
226	S72022C	Fracture of neck of femur (hip)
226	S72022D	Fracture of neck of femur (hip)
226	S72022E	Fracture of neck of femur (hip)
226	S72022F	Fracture of neck of femur (hip)
226	S72022G	Fracture of neck of femur (hip)
226	S72022H	Fracture of neck of femur (hip)
226	S72022J	Fracture of neck of femur (hip)
226	S72022K	Fracture of neck of femur (hip)
226	S72022M	Fracture of neck of femur (hip)
226	S72022N	Fracture of neck of femur (hip)
226	S72022P	Fracture of neck of femur (hip)
226	S72022Q	Fracture of neck of femur (hip)
226	S72022R	Fracture of neck of femur (hip)
226	S72022S	Fracture of neck of femur (hip)
226	S72023A	Fracture of neck of femur (hip)
226	S72023B	Fracture of neck of femur (hip)
226	S72023C	Fracture of neck of femur (hip)
226	S72023D	Fracture of neck of femur (hip)
226	S72023E	Fracture of neck of femur (hip)
226	S72023F	Fracture of neck of femur (hip)
226	S72023G	Fracture of neck of femur (hip)
226	S72023H	Fracture of neck of femur (hip)
226	S72023J	Fracture of neck of femur (hip)
226	S72023K	Fracture of neck of femur (hip)
226	S72023M	Fracture of neck of femur (hip)
226	S72023N	Fracture of neck of femur (hip)
226	S72023P	Fracture of neck of femur (hip)
226	S72023Q	Fracture of neck of femur (hip)
226	S72023R	Fracture of neck of femur (hip)
226	S72023S	Fracture of neck of femur (hip)
226	S72024A	Fracture of neck of femur (hip)
226	S72024B	Fracture of neck of femur (hip)
226	S72024C	Fracture of neck of femur (hip)
226	S72024D	Fracture of neck of femur (hip)
226	S72024E	Fracture of neck of femur (hip)
226	S72024F	Fracture of neck of femur (hip)
226	S72024G	Fracture of neck of femur (hip)
226	S72024H	Fracture of neck of femur (hip)
226	S72024J	Fracture of neck of femur (hip)
226	S72024K	Fracture of neck of femur (hip)

226	S72024M	Fracture of neck of femur (hip)
226	S72024N	Fracture of neck of femur (hip)
226	S72024P	Fracture of neck of femur (hip)
226	S72024Q	Fracture of neck of femur (hip)
226	S72024R	Fracture of neck of femur (hip)
226	S72024S	Fracture of neck of femur (hip)
226	S72025A	Fracture of neck of femur (hip)
226	S72025B	Fracture of neck of femur (hip)
226	S72025C	Fracture of neck of femur (hip)
226	S72025D	Fracture of neck of femur (hip)
226	S72025E	Fracture of neck of femur (hip)
226	S72025F	Fracture of neck of femur (hip)
226	S72025G	Fracture of neck of femur (hip)
226	S72025H	Fracture of neck of femur (hip)
226	S72025J	Fracture of neck of femur (hip)
226	S72025K	Fracture of neck of femur (hip)
226	S72025M	Fracture of neck of femur (hip)
226	S72025N	Fracture of neck of femur (hip)
226	S72025P	Fracture of neck of femur (hip)
226	S72025Q	Fracture of neck of femur (hip)
226	S72025R	Fracture of neck of femur (hip)
226	S72025S	Fracture of neck of femur (hip)
226	S72026A	Fracture of neck of femur (hip)
226	S72026B	Fracture of neck of femur (hip)
226	S72026C	Fracture of neck of femur (hip)
226	S72026D	Fracture of neck of femur (hip)
226	S72026E	Fracture of neck of femur (hip)
226	S72026F	Fracture of neck of femur (hip)
226	S72026G	Fracture of neck of femur (hip)
226	S72026H	Fracture of neck of femur (hip)
226	S72026J	Fracture of neck of femur (hip)
226	S72026K	Fracture of neck of femur (hip)
226	S72026M	Fracture of neck of femur (hip)
226	S72026N	Fracture of neck of femur (hip)
226	S72026P	Fracture of neck of femur (hip)
226	S72026Q	Fracture of neck of femur (hip)
226	S72026R	Fracture of neck of femur (hip)
226	S72026S	Fracture of neck of femur (hip)
226	S72031A	Fracture of neck of femur (hip)
226	S72031B	Fracture of neck of femur (hip)
226	S72031C	Fracture of neck of femur (hip)
226	S72031D	Fracture of neck of femur (hip)
226	S72031E	Fracture of neck of femur (hip)
226	S72031F	Fracture of neck of femur (hip)
226	S72031G	Fracture of neck of femur (hip)
226	S72031H	Fracture of neck of femur (hip)
226	S72031J	Fracture of neck of femur (hip)
226	S72031K	Fracture of neck of femur (hip)
226	S72031M	Fracture of neck of femur (hip)
226	S72031N	Fracture of neck of femur (hip)
226	S72031P	Fracture of neck of femur (hip)

226	S72031Q	Fracture of neck of femur (hip)
226	S72031R	Fracture of neck of femur (hip)
226	S72031S	Fracture of neck of femur (hip)
226	S72032A	Fracture of neck of femur (hip)
226	S72032B	Fracture of neck of femur (hip)
226	S72032C	Fracture of neck of femur (hip)
226	S72032D	Fracture of neck of femur (hip)
226	S72032E	Fracture of neck of femur (hip)
226	S72032F	Fracture of neck of femur (hip)
226	S72032G	Fracture of neck of femur (hip)
226	S72032H	Fracture of neck of femur (hip)
226	S72032J	Fracture of neck of femur (hip)
226	S72032K	Fracture of neck of femur (hip)
226	S72032M	Fracture of neck of femur (hip)
226	S72032N	Fracture of neck of femur (hip)
226	S72032P	Fracture of neck of femur (hip)
226	S72032Q	Fracture of neck of femur (hip)
226	S72032R	Fracture of neck of femur (hip)
226	S72032S	Fracture of neck of femur (hip)
226	S72033A	Fracture of neck of femur (hip)
226	S72033B	Fracture of neck of femur (hip)
226	S72033C	Fracture of neck of femur (hip)
226	S72033D	Fracture of neck of femur (hip)
226	S72033E	Fracture of neck of femur (hip)
226	S72033F	Fracture of neck of femur (hip)
226	S72033G	Fracture of neck of femur (hip)
226	S72033H	Fracture of neck of femur (hip)
226	S72033J	Fracture of neck of femur (hip)
226	S72033K	Fracture of neck of femur (hip)
226	S72033M	Fracture of neck of femur (hip)
226	S72033N	Fracture of neck of femur (hip)
226	S72033P	Fracture of neck of femur (hip)
226	S72033Q	Fracture of neck of femur (hip)
226	S72033R	Fracture of neck of femur (hip)
226	S72033S	Fracture of neck of femur (hip)
226	S72034A	Fracture of neck of femur (hip)
226	S72034B	Fracture of neck of femur (hip)
226	S72034C	Fracture of neck of femur (hip)
226	S72034D	Fracture of neck of femur (hip)
226	S72034E	Fracture of neck of femur (hip)
226	S72034F	Fracture of neck of femur (hip)
226	S72034G	Fracture of neck of femur (hip)
226	S72034H	Fracture of neck of femur (hip)
226	S72034J	Fracture of neck of femur (hip)
226	S72034K	Fracture of neck of femur (hip)
226	S72034M	Fracture of neck of femur (hip)
226	S72034N	Fracture of neck of femur (hip)
226	S72034P	Fracture of neck of femur (hip)
226	S72034Q	Fracture of neck of femur (hip)
226	S72034R	Fracture of neck of femur (hip)
226	S72034S	Fracture of neck of femur (hip)

226	S72035A	Fracture of neck of femur (hip)
226	S72035B	Fracture of neck of femur (hip)
226	S72035C	Fracture of neck of femur (hip)
226	S72035D	Fracture of neck of femur (hip)
226	S72035E	Fracture of neck of femur (hip)
226	S72035F	Fracture of neck of femur (hip)
226	S72035G	Fracture of neck of femur (hip)
226	S72035H	Fracture of neck of femur (hip)
226	S72035J	Fracture of neck of femur (hip)
226	S72035K	Fracture of neck of femur (hip)
226	S72035M	Fracture of neck of femur (hip)
226	S72035N	Fracture of neck of femur (hip)
226	S72035P	Fracture of neck of femur (hip)
226	S72035Q	Fracture of neck of femur (hip)
226	S72035R	Fracture of neck of femur (hip)
226	S72035S	Fracture of neck of femur (hip)
226	S72036A	Fracture of neck of femur (hip)
226	S72036B	Fracture of neck of femur (hip)
226	S72036C	Fracture of neck of femur (hip)
226	S72036D	Fracture of neck of femur (hip)
226	S72036E	Fracture of neck of femur (hip)
226	S72036F	Fracture of neck of femur (hip)
226	S72036G	Fracture of neck of femur (hip)
226	S72036H	Fracture of neck of femur (hip)
226	S72036J	Fracture of neck of femur (hip)
226	S72036K	Fracture of neck of femur (hip)
226	S72036M	Fracture of neck of femur (hip)
226	S72036N	Fracture of neck of femur (hip)
226	S72036P	Fracture of neck of femur (hip)
226	S72036Q	Fracture of neck of femur (hip)
226	S72036R	Fracture of neck of femur (hip)
226	S72036S	Fracture of neck of femur (hip)
226	S72041A	Fracture of neck of femur (hip)
226	S72041B	Fracture of neck of femur (hip)
226	S72041C	Fracture of neck of femur (hip)
226	S72041D	Fracture of neck of femur (hip)
226	S72041E	Fracture of neck of femur (hip)
226	S72041F	Fracture of neck of femur (hip)
226	S72041G	Fracture of neck of femur (hip)
226	S72041H	Fracture of neck of femur (hip)
226	S72041J	Fracture of neck of femur (hip)
226	S72041K	Fracture of neck of femur (hip)
226	S72041M	Fracture of neck of femur (hip)
226	S72041N	Fracture of neck of femur (hip)
226	S72041P	Fracture of neck of femur (hip)
226	S72041Q	Fracture of neck of femur (hip)
226	S72041R	Fracture of neck of femur (hip)
226	S72041S	Fracture of neck of femur (hip)
226	S72042A	Fracture of neck of femur (hip)
226	S72042B	Fracture of neck of femur (hip)
226	S72042C	Fracture of neck of femur (hip)

226	S72042D	Fracture of neck of femur (hip)
226	S72042E	Fracture of neck of femur (hip)
226	S72042F	Fracture of neck of femur (hip)
226	S72042G	Fracture of neck of femur (hip)
226	S72042H	Fracture of neck of femur (hip)
226	S72042J	Fracture of neck of femur (hip)
226	S72042K	Fracture of neck of femur (hip)
226	S72042M	Fracture of neck of femur (hip)
226	S72042N	Fracture of neck of femur (hip)
226	S72042P	Fracture of neck of femur (hip)
226	S72042Q	Fracture of neck of femur (hip)
226	S72042R	Fracture of neck of femur (hip)
226	S72042S	Fracture of neck of femur (hip)
226	S72043A	Fracture of neck of femur (hip)
226	S72043B	Fracture of neck of femur (hip)
226	S72043C	Fracture of neck of femur (hip)
226	S72043D	Fracture of neck of femur (hip)
226	S72043E	Fracture of neck of femur (hip)
226	S72043F	Fracture of neck of femur (hip)
226	S72043G	Fracture of neck of femur (hip)
226	S72043H	Fracture of neck of femur (hip)
226	S72043J	Fracture of neck of femur (hip)
226	S72043K	Fracture of neck of femur (hip)
226	S72043M	Fracture of neck of femur (hip)
226	S72043N	Fracture of neck of femur (hip)
226	S72043P	Fracture of neck of femur (hip)
226	S72043Q	Fracture of neck of femur (hip)
226	S72043R	Fracture of neck of femur (hip)
226	S72043S	Fracture of neck of femur (hip)
226	S72044A	Fracture of neck of femur (hip)
226	S72044B	Fracture of neck of femur (hip)
226	S72044C	Fracture of neck of femur (hip)
226	S72044D	Fracture of neck of femur (hip)
226	S72044E	Fracture of neck of femur (hip)
226	S72044F	Fracture of neck of femur (hip)
226	S72044G	Fracture of neck of femur (hip)
226	S72044H	Fracture of neck of femur (hip)
226	S72044J	Fracture of neck of femur (hip)
226	S72044K	Fracture of neck of femur (hip)
226	S72044M	Fracture of neck of femur (hip)
226	S72044N	Fracture of neck of femur (hip)
226	S72044P	Fracture of neck of femur (hip)
226	S72044Q	Fracture of neck of femur (hip)
226	S72044R	Fracture of neck of femur (hip)
226	S72044S	Fracture of neck of femur (hip)
226	S72045A	Fracture of neck of femur (hip)
226	S72045B	Fracture of neck of femur (hip)
226	S72045C	Fracture of neck of femur (hip)
226	S72045D	Fracture of neck of femur (hip)
226	S72045E	Fracture of neck of femur (hip)
226	S72045F	Fracture of neck of femur (hip)

226	S72045G	Fracture of neck of femur (hip)
226	S72045H	Fracture of neck of femur (hip)
226	S72045J	Fracture of neck of femur (hip)
226	S72045K	Fracture of neck of femur (hip)
226	S72045M	Fracture of neck of femur (hip)
226	S72045N	Fracture of neck of femur (hip)
226	S72045P	Fracture of neck of femur (hip)
226	S72045Q	Fracture of neck of femur (hip)
226	S72045R	Fracture of neck of femur (hip)
226	S72045S	Fracture of neck of femur (hip)
226	S72046A	Fracture of neck of femur (hip)
226	S72046B	Fracture of neck of femur (hip)
226	S72046C	Fracture of neck of femur (hip)
226	S72046D	Fracture of neck of femur (hip)
226	S72046E	Fracture of neck of femur (hip)
226	S72046F	Fracture of neck of femur (hip)
226	S72046G	Fracture of neck of femur (hip)
226	S72046H	Fracture of neck of femur (hip)
226	S72046J	Fracture of neck of femur (hip)
226	S72046K	Fracture of neck of femur (hip)
226	S72046M	Fracture of neck of femur (hip)
226	S72046N	Fracture of neck of femur (hip)
226	S72046P	Fracture of neck of femur (hip)
226	S72046Q	Fracture of neck of femur (hip)
226	S72046R	Fracture of neck of femur (hip)
226	S72046S	Fracture of neck of femur (hip)
226	S72051A	Fracture of neck of femur (hip)
226	S72051B	Fracture of neck of femur (hip)
226	S72051C	Fracture of neck of femur (hip)
226	S72051D	Fracture of neck of femur (hip)
226	S72051E	Fracture of neck of femur (hip)
226	S72051F	Fracture of neck of femur (hip)
226	S72051G	Fracture of neck of femur (hip)
226	S72051H	Fracture of neck of femur (hip)
226	S72051J	Fracture of neck of femur (hip)
226	S72051K	Fracture of neck of femur (hip)
226	S72051M	Fracture of neck of femur (hip)
226	S72051N	Fracture of neck of femur (hip)
226	S72051P	Fracture of neck of femur (hip)
226	S72051Q	Fracture of neck of femur (hip)
226	S72051R	Fracture of neck of femur (hip)
226	S72051S	Fracture of neck of femur (hip)
226	S72052A	Fracture of neck of femur (hip)
226	S72052B	Fracture of neck of femur (hip)
226	S72052C	Fracture of neck of femur (hip)
226	S72052D	Fracture of neck of femur (hip)
226	S72052E	Fracture of neck of femur (hip)
226	S72052F	Fracture of neck of femur (hip)
226	S72052G	Fracture of neck of femur (hip)
226	S72052H	Fracture of neck of femur (hip)
226	S72052J	Fracture of neck of femur (hip)

226	S72052K	Fracture of neck of femur (hip)
226	S72052M	Fracture of neck of femur (hip)
226	S72052N	Fracture of neck of femur (hip)
226	S72052P	Fracture of neck of femur (hip)
226	S72052Q	Fracture of neck of femur (hip)
226	S72052R	Fracture of neck of femur (hip)
226	S72052S	Fracture of neck of femur (hip)
226	S72059A	Fracture of neck of femur (hip)
226	S72059B	Fracture of neck of femur (hip)
226	S72059C	Fracture of neck of femur (hip)
226	S72059D	Fracture of neck of femur (hip)
226	S72059E	Fracture of neck of femur (hip)
226	S72059F	Fracture of neck of femur (hip)
226	S72059G	Fracture of neck of femur (hip)
226	S72059H	Fracture of neck of femur (hip)
226	S72059J	Fracture of neck of femur (hip)
226	S72059K	Fracture of neck of femur (hip)
226	S72059M	Fracture of neck of femur (hip)
226	S72059N	Fracture of neck of femur (hip)
226	S72059P	Fracture of neck of femur (hip)
226	S72059Q	Fracture of neck of femur (hip)
226	S72059R	Fracture of neck of femur (hip)
226	S72059S	Fracture of neck of femur (hip)
226	S72061A	Fracture of neck of femur (hip)
226	S72061B	Fracture of neck of femur (hip)
226	S72061C	Fracture of neck of femur (hip)
226	S72061D	Fracture of neck of femur (hip)
226	S72061E	Fracture of neck of femur (hip)
226	S72061F	Fracture of neck of femur (hip)
226	S72061G	Fracture of neck of femur (hip)
226	S72061H	Fracture of neck of femur (hip)
226	S72061J	Fracture of neck of femur (hip)
226	S72061K	Fracture of neck of femur (hip)
226	S72061M	Fracture of neck of femur (hip)
226	S72061N	Fracture of neck of femur (hip)
226	S72061P	Fracture of neck of femur (hip)
226	S72061Q	Fracture of neck of femur (hip)
226	S72061R	Fracture of neck of femur (hip)
226	S72061S	Fracture of neck of femur (hip)
226	S72062A	Fracture of neck of femur (hip)
226	S72062B	Fracture of neck of femur (hip)
226	S72062C	Fracture of neck of femur (hip)
226	S72062D	Fracture of neck of femur (hip)
226	S72062E	Fracture of neck of femur (hip)
226	S72062F	Fracture of neck of femur (hip)
226	S72062G	Fracture of neck of femur (hip)
226	S72062H	Fracture of neck of femur (hip)
226	S72062J	Fracture of neck of femur (hip)
226	S72062K	Fracture of neck of femur (hip)
226	S72062M	Fracture of neck of femur (hip)
226	S72062N	Fracture of neck of femur (hip)

226	S72062P	Fracture of neck of femur (hip)
226	S72062Q	Fracture of neck of femur (hip)
226	S72062R	Fracture of neck of femur (hip)
226	S72062S	Fracture of neck of femur (hip)
226	S72063A	Fracture of neck of femur (hip)
226	S72063B	Fracture of neck of femur (hip)
226	S72063C	Fracture of neck of femur (hip)
226	S72063D	Fracture of neck of femur (hip)
226	S72063E	Fracture of neck of femur (hip)
226	S72063F	Fracture of neck of femur (hip)
226	S72063G	Fracture of neck of femur (hip)
226	S72063H	Fracture of neck of femur (hip)
226	S72063J	Fracture of neck of femur (hip)
226	S72063K	Fracture of neck of femur (hip)
226	S72063M	Fracture of neck of femur (hip)
226	S72063N	Fracture of neck of femur (hip)
226	S72063P	Fracture of neck of femur (hip)
226	S72063Q	Fracture of neck of femur (hip)
226	S72063R	Fracture of neck of femur (hip)
226	S72063S	Fracture of neck of femur (hip)
226	S72064A	Fracture of neck of femur (hip)
226	S72064B	Fracture of neck of femur (hip)
226	S72064C	Fracture of neck of femur (hip)
226	S72064D	Fracture of neck of femur (hip)
226	S72064E	Fracture of neck of femur (hip)
226	S72064F	Fracture of neck of femur (hip)
226	S72064G	Fracture of neck of femur (hip)
226	S72064H	Fracture of neck of femur (hip)
226	S72064J	Fracture of neck of femur (hip)
226	S72064K	Fracture of neck of femur (hip)
226	S72064M	Fracture of neck of femur (hip)
226	S72064N	Fracture of neck of femur (hip)
226	S72064P	Fracture of neck of femur (hip)
226	S72064Q	Fracture of neck of femur (hip)
226	S72064R	Fracture of neck of femur (hip)
226	S72064S	Fracture of neck of femur (hip)
226	S72065A	Fracture of neck of femur (hip)
226	S72065B	Fracture of neck of femur (hip)
226	S72065C	Fracture of neck of femur (hip)
226	S72065D	Fracture of neck of femur (hip)
226	S72065E	Fracture of neck of femur (hip)
226	S72065F	Fracture of neck of femur (hip)
226	S72065G	Fracture of neck of femur (hip)
226	S72065H	Fracture of neck of femur (hip)
226	S72065J	Fracture of neck of femur (hip)
226	S72065K	Fracture of neck of femur (hip)
226	S72065M	Fracture of neck of femur (hip)
226	S72065N	Fracture of neck of femur (hip)
226	S72065P	Fracture of neck of femur (hip)
226	S72065Q	Fracture of neck of femur (hip)
226	S72065R	Fracture of neck of femur (hip)

226	S72065S	Fracture of neck of femur (hip)
226	S72066A	Fracture of neck of femur (hip)
226	S72066B	Fracture of neck of femur (hip)
226	S72066C	Fracture of neck of femur (hip)
226	S72066D	Fracture of neck of femur (hip)
226	S72066E	Fracture of neck of femur (hip)
226	S72066F	Fracture of neck of femur (hip)
226	S72066G	Fracture of neck of femur (hip)
226	S72066H	Fracture of neck of femur (hip)
226	S72066J	Fracture of neck of femur (hip)
226	S72066K	Fracture of neck of femur (hip)
226	S72066M	Fracture of neck of femur (hip)
226	S72066N	Fracture of neck of femur (hip)
226	S72066P	Fracture of neck of femur (hip)
226	S72066Q	Fracture of neck of femur (hip)
226	S72066R	Fracture of neck of femur (hip)
226	S72066S	Fracture of neck of femur (hip)
226	S72091A	Fracture of neck of femur (hip)
226	S72091B	Fracture of neck of femur (hip)
226	S72091C	Fracture of neck of femur (hip)
226	S72091D	Fracture of neck of femur (hip)
226	S72091E	Fracture of neck of femur (hip)
226	S72091F	Fracture of neck of femur (hip)
226	S72091G	Fracture of neck of femur (hip)
226	S72091H	Fracture of neck of femur (hip)
226	S72091J	Fracture of neck of femur (hip)
226	S72091K	Fracture of neck of femur (hip)
226	S72091M	Fracture of neck of femur (hip)
226	S72091N	Fracture of neck of femur (hip)
226	S72091P	Fracture of neck of femur (hip)
226	S72091Q	Fracture of neck of femur (hip)
226	S72091R	Fracture of neck of femur (hip)
226	S72091S	Fracture of neck of femur (hip)
226	S72092A	Fracture of neck of femur (hip)
226	S72092B	Fracture of neck of femur (hip)
226	S72092C	Fracture of neck of femur (hip)
226	S72092D	Fracture of neck of femur (hip)
226	S72092E	Fracture of neck of femur (hip)
226	S72092F	Fracture of neck of femur (hip)
226	S72092G	Fracture of neck of femur (hip)
226	S72092H	Fracture of neck of femur (hip)
226	S72092J	Fracture of neck of femur (hip)
226	S72092K	Fracture of neck of femur (hip)
226	S72092M	Fracture of neck of femur (hip)
226	S72092N	Fracture of neck of femur (hip)
226	S72092P	Fracture of neck of femur (hip)
226	S72092Q	Fracture of neck of femur (hip)
226	S72092R	Fracture of neck of femur (hip)
226	S72092S	Fracture of neck of femur (hip)
226	S72099A	Fracture of neck of femur (hip)
226	S72099B	Fracture of neck of femur (hip)

226	S72099C	Fracture of neck of femur (hip)
226	S72099D	Fracture of neck of femur (hip)
226	S72099E	Fracture of neck of femur (hip)
226	S72099F	Fracture of neck of femur (hip)
226	S72099G	Fracture of neck of femur (hip)
226	S72099H	Fracture of neck of femur (hip)
226	S72099J	Fracture of neck of femur (hip)
226	S72099K	Fracture of neck of femur (hip)
226	S72099M	Fracture of neck of femur (hip)
226	S72099N	Fracture of neck of femur (hip)
226	S72099P	Fracture of neck of femur (hip)
226	S72099Q	Fracture of neck of femur (hip)
226	S72099R	Fracture of neck of femur (hip)
226	S72099S	Fracture of neck of femur (hip)
226	S72101A	Fracture of neck of femur (hip)
226	S72101B	Fracture of neck of femur (hip)
226	S72101C	Fracture of neck of femur (hip)
226	S72101D	Fracture of neck of femur (hip)
226	S72101E	Fracture of neck of femur (hip)
226	S72101F	Fracture of neck of femur (hip)
226	S72101G	Fracture of neck of femur (hip)
226	S72101H	Fracture of neck of femur (hip)
226	S72101J	Fracture of neck of femur (hip)
226	S72101K	Fracture of neck of femur (hip)
226	S72101M	Fracture of neck of femur (hip)
226	S72101N	Fracture of neck of femur (hip)
226	S72101P	Fracture of neck of femur (hip)
226	S72101Q	Fracture of neck of femur (hip)
226	S72101R	Fracture of neck of femur (hip)
226	S72101S	Fracture of neck of femur (hip)
226	S72102A	Fracture of neck of femur (hip)
226	S72102B	Fracture of neck of femur (hip)
226	S72102C	Fracture of neck of femur (hip)
226	S72102D	Fracture of neck of femur (hip)
226	S72102E	Fracture of neck of femur (hip)
226	S72102F	Fracture of neck of femur (hip)
226	S72102G	Fracture of neck of femur (hip)
226	S72102H	Fracture of neck of femur (hip)
226	S72102J	Fracture of neck of femur (hip)
226	S72102K	Fracture of neck of femur (hip)
226	S72102M	Fracture of neck of femur (hip)
226	S72102N	Fracture of neck of femur (hip)
226	S72102P	Fracture of neck of femur (hip)
226	S72102Q	Fracture of neck of femur (hip)
226	S72102R	Fracture of neck of femur (hip)
226	S72102S	Fracture of neck of femur (hip)
226	S72109A	Fracture of neck of femur (hip)
226	S72109B	Fracture of neck of femur (hip)
226	S72109C	Fracture of neck of femur (hip)
226	S72109D	Fracture of neck of femur (hip)
226	S72109E	Fracture of neck of femur (hip)

226	S72109F	Fracture of neck of femur (hip)
226	S72109G	Fracture of neck of femur (hip)
226	S72109H	Fracture of neck of femur (hip)
226	S72109J	Fracture of neck of femur (hip)
226	S72109K	Fracture of neck of femur (hip)
226	S72109M	Fracture of neck of femur (hip)
226	S72109N	Fracture of neck of femur (hip)
226	S72109P	Fracture of neck of femur (hip)
226	S72109Q	Fracture of neck of femur (hip)
226	S72109R	Fracture of neck of femur (hip)
226	S72109S	Fracture of neck of femur (hip)
226	S72111A	Fracture of neck of femur (hip)
226	S72111B	Fracture of neck of femur (hip)
226	S72111C	Fracture of neck of femur (hip)
226	S72111D	Fracture of neck of femur (hip)
226	S72111E	Fracture of neck of femur (hip)
226	S72111F	Fracture of neck of femur (hip)
226	S72111G	Fracture of neck of femur (hip)
226	S72111H	Fracture of neck of femur (hip)
226	S72111J	Fracture of neck of femur (hip)
226	S72111K	Fracture of neck of femur (hip)
226	S72111M	Fracture of neck of femur (hip)
226	S72111N	Fracture of neck of femur (hip)
226	S72111P	Fracture of neck of femur (hip)
226	S72111Q	Fracture of neck of femur (hip)
226	S72111R	Fracture of neck of femur (hip)
226	S72111S	Fracture of neck of femur (hip)
226	S72112A	Fracture of neck of femur (hip)
226	S72112B	Fracture of neck of femur (hip)
226	S72112C	Fracture of neck of femur (hip)
226	S72112D	Fracture of neck of femur (hip)
226	S72112E	Fracture of neck of femur (hip)
226	S72112F	Fracture of neck of femur (hip)
226	S72112G	Fracture of neck of femur (hip)
226	S72112H	Fracture of neck of femur (hip)
226	S72112J	Fracture of neck of femur (hip)
226	S72112K	Fracture of neck of femur (hip)
226	S72112M	Fracture of neck of femur (hip)
226	S72112N	Fracture of neck of femur (hip)
226	S72112P	Fracture of neck of femur (hip)
226	S72112Q	Fracture of neck of femur (hip)
226	S72112R	Fracture of neck of femur (hip)
226	S72112S	Fracture of neck of femur (hip)
226	S72113A	Fracture of neck of femur (hip)
226	S72113B	Fracture of neck of femur (hip)
226	S72113C	Fracture of neck of femur (hip)
226	S72113D	Fracture of neck of femur (hip)
226	S72113E	Fracture of neck of femur (hip)
226	S72113F	Fracture of neck of femur (hip)
226	S72113G	Fracture of neck of femur (hip)
226	S72113H	Fracture of neck of femur (hip)

226	S72113J	Fracture of neck of femur (hip)
226	S72113K	Fracture of neck of femur (hip)
226	S72113M	Fracture of neck of femur (hip)
226	S72113N	Fracture of neck of femur (hip)
226	S72113P	Fracture of neck of femur (hip)
226	S72113Q	Fracture of neck of femur (hip)
226	S72113R	Fracture of neck of femur (hip)
226	S72113S	Fracture of neck of femur (hip)
226	S72114A	Fracture of neck of femur (hip)
226	S72114B	Fracture of neck of femur (hip)
226	S72114C	Fracture of neck of femur (hip)
226	S72114D	Fracture of neck of femur (hip)
226	S72114E	Fracture of neck of femur (hip)
226	S72114F	Fracture of neck of femur (hip)
226	S72114G	Fracture of neck of femur (hip)
226	S72114H	Fracture of neck of femur (hip)
226	S72114J	Fracture of neck of femur (hip)
226	S72114K	Fracture of neck of femur (hip)
226	S72114M	Fracture of neck of femur (hip)
226	S72114N	Fracture of neck of femur (hip)
226	S72114P	Fracture of neck of femur (hip)
226	S72114Q	Fracture of neck of femur (hip)
226	S72114R	Fracture of neck of femur (hip)
226	S72114S	Fracture of neck of femur (hip)
226	S72115A	Fracture of neck of femur (hip)
226	S72115B	Fracture of neck of femur (hip)
226	S72115C	Fracture of neck of femur (hip)
226	S72115D	Fracture of neck of femur (hip)
226	S72115E	Fracture of neck of femur (hip)
226	S72115F	Fracture of neck of femur (hip)
226	S72115G	Fracture of neck of femur (hip)
226	S72115H	Fracture of neck of femur (hip)
226	S72115J	Fracture of neck of femur (hip)
226	S72115K	Fracture of neck of femur (hip)
226	S72115M	Fracture of neck of femur (hip)
226	S72115N	Fracture of neck of femur (hip)
226	S72115P	Fracture of neck of femur (hip)
226	S72115Q	Fracture of neck of femur (hip)
226	S72115R	Fracture of neck of femur (hip)
226	S72115S	Fracture of neck of femur (hip)
226	S72116A	Fracture of neck of femur (hip)
226	S72116B	Fracture of neck of femur (hip)
226	S72116C	Fracture of neck of femur (hip)
226	S72116D	Fracture of neck of femur (hip)
226	S72116E	Fracture of neck of femur (hip)
226	S72116F	Fracture of neck of femur (hip)
226	S72116G	Fracture of neck of femur (hip)
226	S72116H	Fracture of neck of femur (hip)
226	S72116J	Fracture of neck of femur (hip)
226	S72116K	Fracture of neck of femur (hip)
226	S72116M	Fracture of neck of femur (hip)

226	S72116N	Fracture of neck of femur (hip)
226	S72116P	Fracture of neck of femur (hip)
226	S72116Q	Fracture of neck of femur (hip)
226	S72116R	Fracture of neck of femur (hip)
226	S72116S	Fracture of neck of femur (hip)
226	S72121A	Fracture of neck of femur (hip)
226	S72121B	Fracture of neck of femur (hip)
226	S72121C	Fracture of neck of femur (hip)
226	S72121D	Fracture of neck of femur (hip)
226	S72121E	Fracture of neck of femur (hip)
226	S72121F	Fracture of neck of femur (hip)
226	S72121G	Fracture of neck of femur (hip)
226	S72121H	Fracture of neck of femur (hip)
226	S72121J	Fracture of neck of femur (hip)
226	S72121K	Fracture of neck of femur (hip)
226	S72121M	Fracture of neck of femur (hip)
226	S72121N	Fracture of neck of femur (hip)
226	S72121P	Fracture of neck of femur (hip)
226	S72121Q	Fracture of neck of femur (hip)
226	S72121R	Fracture of neck of femur (hip)
226	S72121S	Fracture of neck of femur (hip)
226	S72122A	Fracture of neck of femur (hip)
226	S72122B	Fracture of neck of femur (hip)
226	S72122C	Fracture of neck of femur (hip)
226	S72122D	Fracture of neck of femur (hip)
226	S72122E	Fracture of neck of femur (hip)
226	S72122F	Fracture of neck of femur (hip)
226	S72122G	Fracture of neck of femur (hip)
226	S72122H	Fracture of neck of femur (hip)
226	S72122J	Fracture of neck of femur (hip)
226	S72122K	Fracture of neck of femur (hip)
226	S72122M	Fracture of neck of femur (hip)
226	S72122N	Fracture of neck of femur (hip)
226	S72122P	Fracture of neck of femur (hip)
226	S72122Q	Fracture of neck of femur (hip)
226	S72122R	Fracture of neck of femur (hip)
226	S72122S	Fracture of neck of femur (hip)
226	S72123A	Fracture of neck of femur (hip)
226	S72123B	Fracture of neck of femur (hip)
226	S72123C	Fracture of neck of femur (hip)
226	S72123D	Fracture of neck of femur (hip)
226	S72123E	Fracture of neck of femur (hip)
226	S72123F	Fracture of neck of femur (hip)
226	S72123G	Fracture of neck of femur (hip)
226	S72123H	Fracture of neck of femur (hip)
226	S72123J	Fracture of neck of femur (hip)
226	S72123K	Fracture of neck of femur (hip)
226	S72123M	Fracture of neck of femur (hip)
226	S72123N	Fracture of neck of femur (hip)
226	S72123P	Fracture of neck of femur (hip)
226	S72123Q	Fracture of neck of femur (hip)

226	S72123R	Fracture of neck of femur (hip)
226	S72123S	Fracture of neck of femur (hip)
226	S72124A	Fracture of neck of femur (hip)
226	S72124B	Fracture of neck of femur (hip)
226	S72124C	Fracture of neck of femur (hip)
226	S72124D	Fracture of neck of femur (hip)
226	S72124E	Fracture of neck of femur (hip)
226	S72124F	Fracture of neck of femur (hip)
226	S72124G	Fracture of neck of femur (hip)
226	S72124H	Fracture of neck of femur (hip)
226	S72124J	Fracture of neck of femur (hip)
226	S72124K	Fracture of neck of femur (hip)
226	S72124M	Fracture of neck of femur (hip)
226	S72124N	Fracture of neck of femur (hip)
226	S72124P	Fracture of neck of femur (hip)
226	S72124Q	Fracture of neck of femur (hip)
226	S72124R	Fracture of neck of femur (hip)
226	S72124S	Fracture of neck of femur (hip)
226	S72125A	Fracture of neck of femur (hip)
226	S72125B	Fracture of neck of femur (hip)
226	S72125C	Fracture of neck of femur (hip)
226	S72125D	Fracture of neck of femur (hip)
226	S72125E	Fracture of neck of femur (hip)
226	S72125F	Fracture of neck of femur (hip)
226	S72125G	Fracture of neck of femur (hip)
226	S72125H	Fracture of neck of femur (hip)
226	S72125J	Fracture of neck of femur (hip)
226	S72125K	Fracture of neck of femur (hip)
226	S72125M	Fracture of neck of femur (hip)
226	S72125N	Fracture of neck of femur (hip)
226	S72125P	Fracture of neck of femur (hip)
226	S72125Q	Fracture of neck of femur (hip)
226	S72125R	Fracture of neck of femur (hip)
226	S72125S	Fracture of neck of femur (hip)
226	S72126A	Fracture of neck of femur (hip)
226	S72126B	Fracture of neck of femur (hip)
226	S72126C	Fracture of neck of femur (hip)
226	S72126D	Fracture of neck of femur (hip)
226	S72126E	Fracture of neck of femur (hip)
226	S72126F	Fracture of neck of femur (hip)
226	S72126G	Fracture of neck of femur (hip)
226	S72126H	Fracture of neck of femur (hip)
226	S72126J	Fracture of neck of femur (hip)
226	S72126K	Fracture of neck of femur (hip)
226	S72126M	Fracture of neck of femur (hip)
226	S72126N	Fracture of neck of femur (hip)
226	S72126P	Fracture of neck of femur (hip)
226	S72126Q	Fracture of neck of femur (hip)
226	S72126R	Fracture of neck of femur (hip)
226	S72126S	Fracture of neck of femur (hip)
226	S72131A	Fracture of neck of femur (hip)

226	S72131B	Fracture of neck of femur (hip)
226	S72131C	Fracture of neck of femur (hip)
226	S72131D	Fracture of neck of femur (hip)
226	S72131E	Fracture of neck of femur (hip)
226	S72131F	Fracture of neck of femur (hip)
226	S72131G	Fracture of neck of femur (hip)
226	S72131H	Fracture of neck of femur (hip)
226	S72131J	Fracture of neck of femur (hip)
226	S72131K	Fracture of neck of femur (hip)
226	S72131M	Fracture of neck of femur (hip)
226	S72131N	Fracture of neck of femur (hip)
226	S72131P	Fracture of neck of femur (hip)
226	S72131Q	Fracture of neck of femur (hip)
226	S72131R	Fracture of neck of femur (hip)
226	S72131S	Fracture of neck of femur (hip)
226	S72132A	Fracture of neck of femur (hip)
226	S72132B	Fracture of neck of femur (hip)
226	S72132C	Fracture of neck of femur (hip)
226	S72132D	Fracture of neck of femur (hip)
226	S72132E	Fracture of neck of femur (hip)
226	S72132F	Fracture of neck of femur (hip)
226	S72132G	Fracture of neck of femur (hip)
226	S72132H	Fracture of neck of femur (hip)
226	S72132J	Fracture of neck of femur (hip)
226	S72132K	Fracture of neck of femur (hip)
226	S72132M	Fracture of neck of femur (hip)
226	S72132N	Fracture of neck of femur (hip)
226	S72132P	Fracture of neck of femur (hip)
226	S72132Q	Fracture of neck of femur (hip)
226	S72132R	Fracture of neck of femur (hip)
226	S72132S	Fracture of neck of femur (hip)
226	S72133A	Fracture of neck of femur (hip)
226	S72133B	Fracture of neck of femur (hip)
226	S72133C	Fracture of neck of femur (hip)
226	S72133D	Fracture of neck of femur (hip)
226	S72133E	Fracture of neck of femur (hip)
226	S72133F	Fracture of neck of femur (hip)
226	S72133G	Fracture of neck of femur (hip)
226	S72133H	Fracture of neck of femur (hip)
226	S72133J	Fracture of neck of femur (hip)
226	S72133K	Fracture of neck of femur (hip)
226	S72133M	Fracture of neck of femur (hip)
226	S72133N	Fracture of neck of femur (hip)
226	S72133P	Fracture of neck of femur (hip)
226	S72133Q	Fracture of neck of femur (hip)
226	S72133R	Fracture of neck of femur (hip)
226	S72133S	Fracture of neck of femur (hip)
226	S72134A	Fracture of neck of femur (hip)
226	S72134B	Fracture of neck of femur (hip)
226	S72134C	Fracture of neck of femur (hip)
226	S72134D	Fracture of neck of femur (hip)

226	S72134E	Fracture of neck of femur (hip)
226	S72134F	Fracture of neck of femur (hip)
226	S72134G	Fracture of neck of femur (hip)
226	S72134H	Fracture of neck of femur (hip)
226	S72134J	Fracture of neck of femur (hip)
226	S72134K	Fracture of neck of femur (hip)
226	S72134M	Fracture of neck of femur (hip)
226	S72134N	Fracture of neck of femur (hip)
226	S72134P	Fracture of neck of femur (hip)
226	S72134Q	Fracture of neck of femur (hip)
226	S72134R	Fracture of neck of femur (hip)
226	S72134S	Fracture of neck of femur (hip)
226	S72135A	Fracture of neck of femur (hip)
226	S72135B	Fracture of neck of femur (hip)
226	S72135C	Fracture of neck of femur (hip)
226	S72135D	Fracture of neck of femur (hip)
226	S72135E	Fracture of neck of femur (hip)
226	S72135F	Fracture of neck of femur (hip)
226	S72135G	Fracture of neck of femur (hip)
226	S72135H	Fracture of neck of femur (hip)
226	S72135J	Fracture of neck of femur (hip)
226	S72135K	Fracture of neck of femur (hip)
226	S72135M	Fracture of neck of femur (hip)
226	S72135N	Fracture of neck of femur (hip)
226	S72135P	Fracture of neck of femur (hip)
226	S72135Q	Fracture of neck of femur (hip)
226	S72135R	Fracture of neck of femur (hip)
226	S72135S	Fracture of neck of femur (hip)
226	S72136A	Fracture of neck of femur (hip)
226	S72136B	Fracture of neck of femur (hip)
226	S72136C	Fracture of neck of femur (hip)
226	S72136D	Fracture of neck of femur (hip)
226	S72136E	Fracture of neck of femur (hip)
226	S72136F	Fracture of neck of femur (hip)
226	S72136G	Fracture of neck of femur (hip)
226	S72136H	Fracture of neck of femur (hip)
226	S72136J	Fracture of neck of femur (hip)
226	S72136K	Fracture of neck of femur (hip)
226	S72136M	Fracture of neck of femur (hip)
226	S72136N	Fracture of neck of femur (hip)
226	S72136P	Fracture of neck of femur (hip)
226	S72136Q	Fracture of neck of femur (hip)
226	S72136R	Fracture of neck of femur (hip)
226	S72136S	Fracture of neck of femur (hip)
226	S72141A	Fracture of neck of femur (hip)
226	S72141B	Fracture of neck of femur (hip)
226	S72141C	Fracture of neck of femur (hip)
226	S72141D	Fracture of neck of femur (hip)
226	S72141E	Fracture of neck of femur (hip)
226	S72141F	Fracture of neck of femur (hip)
226	S72141G	Fracture of neck of femur (hip)

226	S72141H	Fracture of neck of femur (hip)
226	S72141J	Fracture of neck of femur (hip)
226	S72141K	Fracture of neck of femur (hip)
226	S72141M	Fracture of neck of femur (hip)
226	S72141N	Fracture of neck of femur (hip)
226	S72141P	Fracture of neck of femur (hip)
226	S72141Q	Fracture of neck of femur (hip)
226	S72141R	Fracture of neck of femur (hip)
226	S72141S	Fracture of neck of femur (hip)
226	S72142A	Fracture of neck of femur (hip)
226	S72142B	Fracture of neck of femur (hip)
226	S72142C	Fracture of neck of femur (hip)
226	S72142D	Fracture of neck of femur (hip)
226	S72142E	Fracture of neck of femur (hip)
226	S72142F	Fracture of neck of femur (hip)
226	S72142G	Fracture of neck of femur (hip)
226	S72142H	Fracture of neck of femur (hip)
226	S72142J	Fracture of neck of femur (hip)
226	S72142K	Fracture of neck of femur (hip)
226	S72142M	Fracture of neck of femur (hip)
226	S72142N	Fracture of neck of femur (hip)
226	S72142P	Fracture of neck of femur (hip)
226	S72142Q	Fracture of neck of femur (hip)
226	S72142R	Fracture of neck of femur (hip)
226	S72142S	Fracture of neck of femur (hip)
226	S72143A	Fracture of neck of femur (hip)
226	S72143B	Fracture of neck of femur (hip)
226	S72143C	Fracture of neck of femur (hip)
226	S72143D	Fracture of neck of femur (hip)
226	S72143E	Fracture of neck of femur (hip)
226	S72143F	Fracture of neck of femur (hip)
226	S72143G	Fracture of neck of femur (hip)
226	S72143H	Fracture of neck of femur (hip)
226	S72143J	Fracture of neck of femur (hip)
226	S72143K	Fracture of neck of femur (hip)
226	S72143M	Fracture of neck of femur (hip)
226	S72143N	Fracture of neck of femur (hip)
226	S72143P	Fracture of neck of femur (hip)
226	S72143Q	Fracture of neck of femur (hip)
226	S72143R	Fracture of neck of femur (hip)
226	S72143S	Fracture of neck of femur (hip)
226	S72144A	Fracture of neck of femur (hip)
226	S72144B	Fracture of neck of femur (hip)
226	S72144C	Fracture of neck of femur (hip)
226	S72144D	Fracture of neck of femur (hip)
226	S72144E	Fracture of neck of femur (hip)
226	S72144F	Fracture of neck of femur (hip)
226	S72144G	Fracture of neck of femur (hip)
226	S72144H	Fracture of neck of femur (hip)
226	S72144J	Fracture of neck of femur (hip)
226	S72144K	Fracture of neck of femur (hip)

226	S72144M	Fracture of neck of femur (hip)
226	S72144N	Fracture of neck of femur (hip)
226	S72144P	Fracture of neck of femur (hip)
226	S72144Q	Fracture of neck of femur (hip)
226	S72144R	Fracture of neck of femur (hip)
226	S72144S	Fracture of neck of femur (hip)
226	S72145A	Fracture of neck of femur (hip)
226	S72145B	Fracture of neck of femur (hip)
226	S72145C	Fracture of neck of femur (hip)
226	S72145D	Fracture of neck of femur (hip)
226	S72145E	Fracture of neck of femur (hip)
226	S72145F	Fracture of neck of femur (hip)
226	S72145G	Fracture of neck of femur (hip)
226	S72145H	Fracture of neck of femur (hip)
226	S72145J	Fracture of neck of femur (hip)
226	S72145K	Fracture of neck of femur (hip)
226	S72145M	Fracture of neck of femur (hip)
226	S72145N	Fracture of neck of femur (hip)
226	S72145P	Fracture of neck of femur (hip)
226	S72145Q	Fracture of neck of femur (hip)
226	S72145R	Fracture of neck of femur (hip)
226	S72145S	Fracture of neck of femur (hip)
226	S72146A	Fracture of neck of femur (hip)
226	S72146B	Fracture of neck of femur (hip)
226	S72146C	Fracture of neck of femur (hip)
226	S72146D	Fracture of neck of femur (hip)
226	S72146E	Fracture of neck of femur (hip)
226	S72146F	Fracture of neck of femur (hip)
226	S72146G	Fracture of neck of femur (hip)
226	S72146H	Fracture of neck of femur (hip)
226	S72146J	Fracture of neck of femur (hip)
226	S72146K	Fracture of neck of femur (hip)
226	S72146M	Fracture of neck of femur (hip)
226	S72146N	Fracture of neck of femur (hip)
226	S72146P	Fracture of neck of femur (hip)
226	S72146Q	Fracture of neck of femur (hip)
226	S72146R	Fracture of neck of femur (hip)
226	S72146S	Fracture of neck of femur (hip)
226	S7221XA	Fracture of neck of femur (hip)
226	S7221XB	Fracture of neck of femur (hip)
226	S7221XC	Fracture of neck of femur (hip)
226	S7221XD	Fracture of neck of femur (hip)
226	S7221XE	Fracture of neck of femur (hip)
226	S7221XF	Fracture of neck of femur (hip)
226	S7221XG	Fracture of neck of femur (hip)
226	S7221XH	Fracture of neck of femur (hip)
226	S7221XJ	Fracture of neck of femur (hip)
226	S7221XK	Fracture of neck of femur (hip)
226	S7221XM	Fracture of neck of femur (hip)
226	S7221XN	Fracture of neck of femur (hip)
226	S7221XP	Fracture of neck of femur (hip)

226	S7221XQ	Fracture of neck of femur (hip)
226	S7221XR	Fracture of neck of femur (hip)
226	S7221XS	Fracture of neck of femur (hip)
226	S7222XA	Fracture of neck of femur (hip)
226	S7222XB	Fracture of neck of femur (hip)
226	S7222XC	Fracture of neck of femur (hip)
226	S7222XD	Fracture of neck of femur (hip)
226	S7222XE	Fracture of neck of femur (hip)
226	S7222XF	Fracture of neck of femur (hip)
226	S7222XG	Fracture of neck of femur (hip)
226	S7222XH	Fracture of neck of femur (hip)
226	S7222XJ	Fracture of neck of femur (hip)
226	S7222XK	Fracture of neck of femur (hip)
226	S7222XM	Fracture of neck of femur (hip)
226	S7222XN	Fracture of neck of femur (hip)
226	S7222XP	Fracture of neck of femur (hip)
226	S7222XQ	Fracture of neck of femur (hip)
226	S7222XR	Fracture of neck of femur (hip)
226	S7222XS	Fracture of neck of femur (hip)
226	S7223XA	Fracture of neck of femur (hip)
226	S7223XB	Fracture of neck of femur (hip)
226	S7223XC	Fracture of neck of femur (hip)
226	S7223XD	Fracture of neck of femur (hip)
226	S7223XE	Fracture of neck of femur (hip)
226	S7223XF	Fracture of neck of femur (hip)
226	S7223XG	Fracture of neck of femur (hip)
226	S7223XH	Fracture of neck of femur (hip)
226	S7223XJ	Fracture of neck of femur (hip)
226	S7223XK	Fracture of neck of femur (hip)
226	S7223XM	Fracture of neck of femur (hip)
226	S7223XN	Fracture of neck of femur (hip)
226	S7223XP	Fracture of neck of femur (hip)
226	S7223XQ	Fracture of neck of femur (hip)
226	S7223XR	Fracture of neck of femur (hip)
226	S7223XS	Fracture of neck of femur (hip)
226	S7224XA	Fracture of neck of femur (hip)
226	S7224XB	Fracture of neck of femur (hip)
226	S7224XC	Fracture of neck of femur (hip)
226	S7224XD	Fracture of neck of femur (hip)
226	S7224XE	Fracture of neck of femur (hip)
226	S7224XF	Fracture of neck of femur (hip)
226	S7224XG	Fracture of neck of femur (hip)
226	S7224XH	Fracture of neck of femur (hip)
226	S7224XJ	Fracture of neck of femur (hip)
226	S7224XK	Fracture of neck of femur (hip)
226	S7224XM	Fracture of neck of femur (hip)
226	S7224XN	Fracture of neck of femur (hip)
226	S7224XP	Fracture of neck of femur (hip)
226	S7224XQ	Fracture of neck of femur (hip)
226	S7224XR	Fracture of neck of femur (hip)
226	S7224XS	Fracture of neck of femur (hip)

226	S7225XA	Fracture of neck of femur (hip)
226	S7225XB	Fracture of neck of femur (hip)
226	S7225XC	Fracture of neck of femur (hip)
226	S7225XD	Fracture of neck of femur (hip)
226	S7225XE	Fracture of neck of femur (hip)
226	S7225XF	Fracture of neck of femur (hip)
226	S7225XG	Fracture of neck of femur (hip)
226	S7225XH	Fracture of neck of femur (hip)
226	S7225XJ	Fracture of neck of femur (hip)
226	S7225XK	Fracture of neck of femur (hip)
226	S7225XM	Fracture of neck of femur (hip)
226	S7225XN	Fracture of neck of femur (hip)
226	S7225XP	Fracture of neck of femur (hip)
226	S7225XQ	Fracture of neck of femur (hip)
226	S7225XR	Fracture of neck of femur (hip)
226	S7225XS	Fracture of neck of femur (hip)
226	S7226XA	Fracture of neck of femur (hip)
226	S7226XB	Fracture of neck of femur (hip)
226	S7226XC	Fracture of neck of femur (hip)
226	S7226XD	Fracture of neck of femur (hip)
226	S7226XE	Fracture of neck of femur (hip)
226	S7226XF	Fracture of neck of femur (hip)
226	S7226XG	Fracture of neck of femur (hip)
226	S7226XH	Fracture of neck of femur (hip)
226	S7226XJ	Fracture of neck of femur (hip)
226	S7226XK	Fracture of neck of femur (hip)
226	S7226XM	Fracture of neck of femur (hip)
226	S7226XN	Fracture of neck of femur (hip)
226	S7226XP	Fracture of neck of femur (hip)
226	S7226XQ	Fracture of neck of femur (hip)
226	S7226XR	Fracture of neck of femur (hip)
226	S7226XS	Fracture of neck of femur (hip)
227	G9611	Spinal cord injury
227	S140XXA	Spinal cord injury
227	S140XXD	Spinal cord injury
227	S140XXS	Spinal cord injury
227	S14101A	Spinal cord injury
227	S14101D	Spinal cord injury
227	S14101S	Spinal cord injury
227	S14102A	Spinal cord injury
227	S14102D	Spinal cord injury
227	S14102S	Spinal cord injury
227	S14103A	Spinal cord injury
227	S14103D	Spinal cord injury
227	S14103S	Spinal cord injury
227	S14104A	Spinal cord injury
227	S14104D	Spinal cord injury
227	S14104S	Spinal cord injury
227	S14105A	Spinal cord injury
227	S14105D	Spinal cord injury
227	S14105S	Spinal cord injury

227	S14106A	Spinal cord injury
227	S14106D	Spinal cord injury
227	S14106S	Spinal cord injury
227	S14107A	Spinal cord injury
227	S14107D	Spinal cord injury
227	S14107S	Spinal cord injury
227	S14108A	Spinal cord injury
227	S14108D	Spinal cord injury
227	S14108S	Spinal cord injury
227	S14109A	Spinal cord injury
227	S14109D	Spinal cord injury
227	S14109S	Spinal cord injury
227	S14111A	Spinal cord injury
227	S14111D	Spinal cord injury
227	S14111S	Spinal cord injury
227	S14112A	Spinal cord injury
227	S14112D	Spinal cord injury
227	S14112S	Spinal cord injury
227	S14113A	Spinal cord injury
227	S14113D	Spinal cord injury
227	S14113S	Spinal cord injury
227	S14114A	Spinal cord injury
227	S14114D	Spinal cord injury
227	S14114S	Spinal cord injury
227	S14115A	Spinal cord injury
227	S14115D	Spinal cord injury
227	S14115S	Spinal cord injury
227	S14116A	Spinal cord injury
227	S14116D	Spinal cord injury
227	S14116S	Spinal cord injury
227	S14117A	Spinal cord injury
227	S14117D	Spinal cord injury
227	S14117S	Spinal cord injury
227	S14118A	Spinal cord injury
227	S14118D	Spinal cord injury
227	S14118S	Spinal cord injury
227	S14119A	Spinal cord injury
227	S14119D	Spinal cord injury
227	S14119S	Spinal cord injury
227	S14121A	Spinal cord injury
227	S14121D	Spinal cord injury
227	S14121S	Spinal cord injury
227	S14122A	Spinal cord injury
227	S14122D	Spinal cord injury
227	S14122S	Spinal cord injury
227	S14123A	Spinal cord injury
227	S14123D	Spinal cord injury
227	S14123S	Spinal cord injury
227	S14124A	Spinal cord injury
227	S14124D	Spinal cord injury
227	S14124S	Spinal cord injury

227	S14125A	Spinal cord injury
227	S14125D	Spinal cord injury
227	S14125S	Spinal cord injury
227	S14126A	Spinal cord injury
227	S14126D	Spinal cord injury
227	S14126S	Spinal cord injury
227	S14127A	Spinal cord injury
227	S14127D	Spinal cord injury
227	S14127S	Spinal cord injury
227	S14128A	Spinal cord injury
227	S14128D	Spinal cord injury
227	S14128S	Spinal cord injury
227	S14129A	Spinal cord injury
227	S14129D	Spinal cord injury
227	S14129S	Spinal cord injury
227	S14131A	Spinal cord injury
227	S14131D	Spinal cord injury
227	S14131S	Spinal cord injury
227	S14132A	Spinal cord injury
227	S14132D	Spinal cord injury
227	S14132S	Spinal cord injury
227	S14133A	Spinal cord injury
227	S14133D	Spinal cord injury
227	S14133S	Spinal cord injury
227	S14134A	Spinal cord injury
227	S14134D	Spinal cord injury
227	S14134S	Spinal cord injury
227	S14135A	Spinal cord injury
227	S14135D	Spinal cord injury
227	S14135S	Spinal cord injury
227	S14136A	Spinal cord injury
227	S14136D	Spinal cord injury
227	S14136S	Spinal cord injury
227	S14137A	Spinal cord injury
227	S14137D	Spinal cord injury
227	S14137S	Spinal cord injury
227	S14138A	Spinal cord injury
227	S14138D	Spinal cord injury
227	S14138S	Spinal cord injury
227	S14139A	Spinal cord injury
227	S14139D	Spinal cord injury
227	S14139S	Spinal cord injury
227	S14141A	Spinal cord injury
227	S14141D	Spinal cord injury
227	S14141S	Spinal cord injury
227	S14142A	Spinal cord injury
227	S14142D	Spinal cord injury
227	S14142S	Spinal cord injury
227	S14143A	Spinal cord injury
227	S14143D	Spinal cord injury
227	S14143S	Spinal cord injury

227	S14144A	Spinal cord injury
227	S14144D	Spinal cord injury
227	S14144S	Spinal cord injury
227	S14145A	Spinal cord injury
227	S14145D	Spinal cord injury
227	S14145S	Spinal cord injury
227	S14146A	Spinal cord injury
227	S14146D	Spinal cord injury
227	S14146S	Spinal cord injury
227	S14147A	Spinal cord injury
227	S14147D	Spinal cord injury
227	S14147S	Spinal cord injury
227	S14148A	Spinal cord injury
227	S14148D	Spinal cord injury
227	S14148S	Spinal cord injury
227	S14149A	Spinal cord injury
227	S14149D	Spinal cord injury
227	S14149S	Spinal cord injury
227	S14151A	Spinal cord injury
227	S14151D	Spinal cord injury
227	S14151S	Spinal cord injury
227	S14152A	Spinal cord injury
227	S14152D	Spinal cord injury
227	S14152S	Spinal cord injury
227	S14153A	Spinal cord injury
227	S14153D	Spinal cord injury
227	S14153S	Spinal cord injury
227	S14154A	Spinal cord injury
227	S14154D	Spinal cord injury
227	S14154S	Spinal cord injury
227	S14155A	Spinal cord injury
227	S14155D	Spinal cord injury
227	S14155S	Spinal cord injury
227	S14156A	Spinal cord injury
227	S14156D	Spinal cord injury
227	S14156S	Spinal cord injury
227	S14157A	Spinal cord injury
227	S14157D	Spinal cord injury
227	S14157S	Spinal cord injury
227	S14158A	Spinal cord injury
227	S14158D	Spinal cord injury
227	S14158S	Spinal cord injury
227	S14159A	Spinal cord injury
227	S14159D	Spinal cord injury
227	S14159S	Spinal cord injury
227	S240XXA	Spinal cord injury
227	S240XXD	Spinal cord injury
227	S240XXS	Spinal cord injury
227	S24101A	Spinal cord injury
227	S24101D	Spinal cord injury
227	S24101S	Spinal cord injury

227	S24102A	Spinal cord injury
227	S24102D	Spinal cord injury
227	S24102S	Spinal cord injury
227	S24103A	Spinal cord injury
227	S24103D	Spinal cord injury
227	S24103S	Spinal cord injury
227	S24104A	Spinal cord injury
227	S24104D	Spinal cord injury
227	S24104S	Spinal cord injury
227	S24109A	Spinal cord injury
227	S24109D	Spinal cord injury
227	S24109S	Spinal cord injury
227	S24111A	Spinal cord injury
227	S24111D	Spinal cord injury
227	S24111S	Spinal cord injury
227	S24112A	Spinal cord injury
227	S24112D	Spinal cord injury
227	S24112S	Spinal cord injury
227	S24113A	Spinal cord injury
227	S24113D	Spinal cord injury
227	S24113S	Spinal cord injury
227	S24114A	Spinal cord injury
227	S24114D	Spinal cord injury
227	S24114S	Spinal cord injury
227	S24119A	Spinal cord injury
227	S24119D	Spinal cord injury
227	S24119S	Spinal cord injury
227	S24131A	Spinal cord injury
227	S24131D	Spinal cord injury
227	S24131S	Spinal cord injury
227	S24132A	Spinal cord injury
227	S24132D	Spinal cord injury
227	S24132S	Spinal cord injury
227	S24133A	Spinal cord injury
227	S24133D	Spinal cord injury
227	S24133S	Spinal cord injury
227	S24134A	Spinal cord injury
227	S24134D	Spinal cord injury
227	S24134S	Spinal cord injury
227	S24139A	Spinal cord injury
227	S24139D	Spinal cord injury
227	S24139S	Spinal cord injury
227	S24141A	Spinal cord injury
227	S24141D	Spinal cord injury
227	S24141S	Spinal cord injury
227	S24142A	Spinal cord injury
227	S24142D	Spinal cord injury
227	S24142S	Spinal cord injury
227	S24143A	Spinal cord injury
227	S24143D	Spinal cord injury
227	S24143S	Spinal cord injury

227	S24144A	Spinal cord injury
227	S24144D	Spinal cord injury
227	S24144S	Spinal cord injury
227	S24149A	Spinal cord injury
227	S24149D	Spinal cord injury
227	S24149S	Spinal cord injury
227	S24151A	Spinal cord injury
227	S24151D	Spinal cord injury
227	S24151S	Spinal cord injury
227	S24152A	Spinal cord injury
227	S24152D	Spinal cord injury
227	S24152S	Spinal cord injury
227	S24153A	Spinal cord injury
227	S24153D	Spinal cord injury
227	S24153S	Spinal cord injury
227	S24154A	Spinal cord injury
227	S24154D	Spinal cord injury
227	S24154S	Spinal cord injury
227	S24159A	Spinal cord injury
227	S24159D	Spinal cord injury
227	S24159S	Spinal cord injury
227	S3401XA	Spinal cord injury
227	S3401XD	Spinal cord injury
227	S3401XS	Spinal cord injury
227	S3402XA	Spinal cord injury
227	S3402XD	Spinal cord injury
227	S3402XS	Spinal cord injury
227	S34101A	Spinal cord injury
227	S34101D	Spinal cord injury
227	S34101S	Spinal cord injury
227	S34102A	Spinal cord injury
227	S34102D	Spinal cord injury
227	S34102S	Spinal cord injury
227	S34103A	Spinal cord injury
227	S34103D	Spinal cord injury
227	S34103S	Spinal cord injury
227	S34104A	Spinal cord injury
227	S34104D	Spinal cord injury
227	S34104S	Spinal cord injury
227	S34105A	Spinal cord injury
227	S34105D	Spinal cord injury
227	S34105S	Spinal cord injury
227	S34109A	Spinal cord injury
227	S34109D	Spinal cord injury
227	S34109S	Spinal cord injury
227	S34111A	Spinal cord injury
227	S34111D	Spinal cord injury
227	S34111S	Spinal cord injury
227	S34112A	Spinal cord injury
227	S34112D	Spinal cord injury
227	S34112S	Spinal cord injury

227	S34113A	Spinal cord injury
227	S34113D	Spinal cord injury
227	S34113S	Spinal cord injury
227	S34114A	Spinal cord injury
227	S34114D	Spinal cord injury
227	S34114S	Spinal cord injury
227	S34115A	Spinal cord injury
227	S34115D	Spinal cord injury
227	S34115S	Spinal cord injury
227	S34119A	Spinal cord injury
227	S34119D	Spinal cord injury
227	S34119S	Spinal cord injury
227	S34121A	Spinal cord injury
227	S34121D	Spinal cord injury
227	S34121S	Spinal cord injury
227	S34122A	Spinal cord injury
227	S34122D	Spinal cord injury
227	S34122S	Spinal cord injury
227	S34123A	Spinal cord injury
227	S34123D	Spinal cord injury
227	S34123S	Spinal cord injury
227	S34124A	Spinal cord injury
227	S34124D	Spinal cord injury
227	S34124S	Spinal cord injury
227	S34125A	Spinal cord injury
227	S34125D	Spinal cord injury
227	S34125S	Spinal cord injury
227	S34129A	Spinal cord injury
227	S34129D	Spinal cord injury
227	S34129S	Spinal cord injury
227	S34131A	Spinal cord injury
227	S34131D	Spinal cord injury
227	S34131S	Spinal cord injury
227	S34132A	Spinal cord injury
227	S34132D	Spinal cord injury
227	S34132S	Spinal cord injury
227	S34139A	Spinal cord injury
227	S34139D	Spinal cord injury
227	S34139S	Spinal cord injury
228	S020XXA	Skull and face fractures
228	S020XXB	Skull and face fractures
228	S020XXD	Skull and face fractures
228	S020XXG	Skull and face fractures
228	S020XXK	Skull and face fractures
228	S020XXS	Skull and face fractures
228	S0210XA	Skull and face fractures
228	S0210XB	Skull and face fractures
228	S0210XD	Skull and face fractures
228	S0210XG	Skull and face fractures
228	S0210XK	Skull and face fractures
228	S0210XS	Skull and face fractures

228	S02110A	Skull and face fractures
228	S02110B	Skull and face fractures
228	S02110D	Skull and face fractures
228	S02110G	Skull and face fractures
228	S02110K	Skull and face fractures
228	S02110S	Skull and face fractures
228	S02111A	Skull and face fractures
228	S02111B	Skull and face fractures
228	S02111D	Skull and face fractures
228	S02111G	Skull and face fractures
228	S02111K	Skull and face fractures
228	S02111S	Skull and face fractures
228	S02112A	Skull and face fractures
228	S02112B	Skull and face fractures
228	S02112D	Skull and face fractures
228	S02112G	Skull and face fractures
228	S02112K	Skull and face fractures
228	S02112S	Skull and face fractures
228	S02113A	Skull and face fractures
228	S02113B	Skull and face fractures
228	S02113D	Skull and face fractures
228	S02113G	Skull and face fractures
228	S02113K	Skull and face fractures
228	S02113S	Skull and face fractures
228	S02118A	Skull and face fractures
228	S02118B	Skull and face fractures
228	S02118D	Skull and face fractures
228	S02118G	Skull and face fractures
228	S02118K	Skull and face fractures
228	S02118S	Skull and face fractures
228	S02119A	Skull and face fractures
228	S02119B	Skull and face fractures
228	S02119D	Skull and face fractures
228	S02119G	Skull and face fractures
228	S02119K	Skull and face fractures
228	S02119S	Skull and face fractures
228	S0219XA	Skull and face fractures
228	S0219XB	Skull and face fractures
228	S0219XD	Skull and face fractures
228	S0219XG	Skull and face fractures
228	S0219XK	Skull and face fractures
228	S0219XS	Skull and face fractures
228	S022XXA	Skull and face fractures
228	S022XXB	Skull and face fractures
228	S022XXD	Skull and face fractures
228	S022XXG	Skull and face fractures
228	S022XXK	Skull and face fractures
228	S022XXS	Skull and face fractures
228	S023XXA	Skull and face fractures
228	S023XXB	Skull and face fractures
228	S023XXD	Skull and face fractures

228	S023XXG	Skull and face fractures
228	S023XXK	Skull and face fractures
228	S023XXS	Skull and face fractures
228	S02400A	Skull and face fractures
228	S02400B	Skull and face fractures
228	S02400D	Skull and face fractures
228	S02400G	Skull and face fractures
228	S02400K	Skull and face fractures
228	S02400S	Skull and face fractures
228	S02401A	Skull and face fractures
228	S02401B	Skull and face fractures
228	S02401D	Skull and face fractures
228	S02401G	Skull and face fractures
228	S02401K	Skull and face fractures
228	S02401S	Skull and face fractures
228	S02402A	Skull and face fractures
228	S02402B	Skull and face fractures
228	S02402D	Skull and face fractures
228	S02402G	Skull and face fractures
228	S02402K	Skull and face fractures
228	S02402S	Skull and face fractures
228	S02411A	Skull and face fractures
228	S02411B	Skull and face fractures
228	S02411D	Skull and face fractures
228	S02411G	Skull and face fractures
228	S02411K	Skull and face fractures
228	S02411S	Skull and face fractures
228	S02412A	Skull and face fractures
228	S02412B	Skull and face fractures
228	S02412D	Skull and face fractures
228	S02412G	Skull and face fractures
228	S02412K	Skull and face fractures
228	S02412S	Skull and face fractures
228	S02413A	Skull and face fractures
228	S02413B	Skull and face fractures
228	S02413D	Skull and face fractures
228	S02413G	Skull and face fractures
228	S02413K	Skull and face fractures
228	S02413S	Skull and face fractures
228	S0242XA	Skull and face fractures
228	S0242XB	Skull and face fractures
228	S0242XD	Skull and face fractures
228	S0242XG	Skull and face fractures
228	S0242XK	Skull and face fractures
228	S0242XS	Skull and face fractures
228	S025XXA	Skull and face fractures
228	S025XXB	Skull and face fractures
228	S025XXD	Skull and face fractures
228	S025XXG	Skull and face fractures
228	S025XXK	Skull and face fractures
228	S025XXS	Skull and face fractures

228	S02600A	Skull and face fractures
228	S02600B	Skull and face fractures
228	S02600D	Skull and face fractures
228	S02600G	Skull and face fractures
228	S02600K	Skull and face fractures
228	S02600S	Skull and face fractures
228	S02609A	Skull and face fractures
228	S02609B	Skull and face fractures
228	S02609D	Skull and face fractures
228	S02609G	Skull and face fractures
228	S02609K	Skull and face fractures
228	S02609S	Skull and face fractures
228	S0261XA	Skull and face fractures
228	S0261XB	Skull and face fractures
228	S0261XD	Skull and face fractures
228	S0261XG	Skull and face fractures
228	S0261XK	Skull and face fractures
228	S0261XS	Skull and face fractures
228	S0262XA	Skull and face fractures
228	S0262XB	Skull and face fractures
228	S0262XD	Skull and face fractures
228	S0262XG	Skull and face fractures
228	S0262XK	Skull and face fractures
228	S0262XS	Skull and face fractures
228	S0263XA	Skull and face fractures
228	S0263XB	Skull and face fractures
228	S0263XD	Skull and face fractures
228	S0263XG	Skull and face fractures
228	S0263XK	Skull and face fractures
228	S0263XS	Skull and face fractures
228	S0264XA	Skull and face fractures
228	S0264XB	Skull and face fractures
228	S0264XD	Skull and face fractures
228	S0264XG	Skull and face fractures
228	S0264XK	Skull and face fractures
228	S0264XS	Skull and face fractures
228	S0265XA	Skull and face fractures
228	S0265XB	Skull and face fractures
228	S0265XD	Skull and face fractures
228	S0265XG	Skull and face fractures
228	S0265XK	Skull and face fractures
228	S0265XS	Skull and face fractures
228	S0266XA	Skull and face fractures
228	S0266XB	Skull and face fractures
228	S0266XD	Skull and face fractures
228	S0266XG	Skull and face fractures
228	S0266XK	Skull and face fractures
228	S0266XS	Skull and face fractures
228	S0267XA	Skull and face fractures
228	S0267XB	Skull and face fractures
228	S0267XD	Skull and face fractures

228	S0267XG	Skull and face fractures
228	S0267XK	Skull and face fractures
228	S0267XS	Skull and face fractures
228	S0269XA	Skull and face fractures
228	S0269XB	Skull and face fractures
228	S0269XD	Skull and face fractures
228	S0269XG	Skull and face fractures
228	S0269XK	Skull and face fractures
228	S0269XS	Skull and face fractures
228	S028XXA	Skull and face fractures
228	S028XXB	Skull and face fractures
228	S028XXD	Skull and face fractures
228	S028XXG	Skull and face fractures
228	S028XXK	Skull and face fractures
228	S028XXS	Skull and face fractures
228	S0291XA	Skull and face fractures
228	S0291XB	Skull and face fractures
228	S0291XD	Skull and face fractures
228	S0291XG	Skull and face fractures
228	S0291XK	Skull and face fractures
228	S0291XS	Skull and face fractures
228	S0292XA	Skull and face fractures
228	S0292XB	Skull and face fractures
228	S0292XD	Skull and face fractures
228	S0292XG	Skull and face fractures
228	S0292XK	Skull and face fractures
228	S0292XS	Skull and face fractures
229	M84311A	Fracture of upper limb
229	M84311D	Fracture of upper limb
229	M84311G	Fracture of upper limb
229	M84311K	Fracture of upper limb
229	M84311P	Fracture of upper limb
229	M84311S	Fracture of upper limb
229	M84312A	Fracture of upper limb
229	M84312D	Fracture of upper limb
229	M84312G	Fracture of upper limb
229	M84312K	Fracture of upper limb
229	M84312P	Fracture of upper limb
229	M84312S	Fracture of upper limb
229	M84319A	Fracture of upper limb
229	M84319D	Fracture of upper limb
229	M84319G	Fracture of upper limb
229	M84319K	Fracture of upper limb
229	M84319P	Fracture of upper limb
229	M84319S	Fracture of upper limb
229	M84321A	Fracture of upper limb
229	M84321D	Fracture of upper limb
229	M84321G	Fracture of upper limb
229	M84321K	Fracture of upper limb
229	M84321P	Fracture of upper limb
229	M84321S	Fracture of upper limb

229	M84322A	Fracture of upper limb
229	M84322D	Fracture of upper limb
229	M84322G	Fracture of upper limb
229	M84322K	Fracture of upper limb
229	M84322P	Fracture of upper limb
229	M84322S	Fracture of upper limb
229	M84329A	Fracture of upper limb
229	M84329D	Fracture of upper limb
229	M84329G	Fracture of upper limb
229	M84329K	Fracture of upper limb
229	M84329P	Fracture of upper limb
229	M84329S	Fracture of upper limb
229	M84331A	Fracture of upper limb
229	M84331D	Fracture of upper limb
229	M84331G	Fracture of upper limb
229	M84331K	Fracture of upper limb
229	M84331P	Fracture of upper limb
229	M84331S	Fracture of upper limb
229	M84332A	Fracture of upper limb
229	M84332D	Fracture of upper limb
229	M84332G	Fracture of upper limb
229	M84332K	Fracture of upper limb
229	M84332P	Fracture of upper limb
229	M84332S	Fracture of upper limb
229	M84333A	Fracture of upper limb
229	M84333D	Fracture of upper limb
229	M84333G	Fracture of upper limb
229	M84333K	Fracture of upper limb
229	M84333P	Fracture of upper limb
229	M84333S	Fracture of upper limb
229	M84334A	Fracture of upper limb
229	M84334D	Fracture of upper limb
229	M84334G	Fracture of upper limb
229	M84334K	Fracture of upper limb
229	M84334P	Fracture of upper limb
229	M84334S	Fracture of upper limb
229	M84339A	Fracture of upper limb
229	M84339D	Fracture of upper limb
229	M84339G	Fracture of upper limb
229	M84339K	Fracture of upper limb
229	M84339P	Fracture of upper limb
229	M84339S	Fracture of upper limb
229	M84341A	Fracture of upper limb
229	M84341D	Fracture of upper limb
229	M84341G	Fracture of upper limb
229	M84341K	Fracture of upper limb
229	M84341P	Fracture of upper limb
229	M84341S	Fracture of upper limb
229	M84342A	Fracture of upper limb
229	M84342D	Fracture of upper limb
229	M84342G	Fracture of upper limb

229	M84342K	Fracture of upper limb
229	M84342P	Fracture of upper limb
229	M84342S	Fracture of upper limb
229	M84343A	Fracture of upper limb
229	M84343D	Fracture of upper limb
229	M84343G	Fracture of upper limb
229	M84343K	Fracture of upper limb
229	M84343P	Fracture of upper limb
229	M84343S	Fracture of upper limb
229	M84344A	Fracture of upper limb
229	M84344D	Fracture of upper limb
229	M84344G	Fracture of upper limb
229	M84344K	Fracture of upper limb
229	M84344P	Fracture of upper limb
229	M84344S	Fracture of upper limb
229	M84345A	Fracture of upper limb
229	M84345D	Fracture of upper limb
229	M84345G	Fracture of upper limb
229	M84345K	Fracture of upper limb
229	M84345P	Fracture of upper limb
229	M84345S	Fracture of upper limb
229	M84346A	Fracture of upper limb
229	M84346D	Fracture of upper limb
229	M84346G	Fracture of upper limb
229	M84346K	Fracture of upper limb
229	M84346P	Fracture of upper limb
229	M84346S	Fracture of upper limb
229	S42001A	Fracture of upper limb
229	S42001B	Fracture of upper limb
229	S42001S	Fracture of upper limb
229	S42002A	Fracture of upper limb
229	S42002B	Fracture of upper limb
229	S42002S	Fracture of upper limb
229	S42009A	Fracture of upper limb
229	S42009B	Fracture of upper limb
229	S42009S	Fracture of upper limb
229	S42011A	Fracture of upper limb
229	S42011B	Fracture of upper limb
229	S42011S	Fracture of upper limb
229	S42012A	Fracture of upper limb
229	S42012B	Fracture of upper limb
229	S42012S	Fracture of upper limb
229	S42013A	Fracture of upper limb
229	S42013B	Fracture of upper limb
229	S42013S	Fracture of upper limb
229	S42014A	Fracture of upper limb
229	S42014B	Fracture of upper limb
229	S42014S	Fracture of upper limb
229	S42015A	Fracture of upper limb
229	S42015B	Fracture of upper limb
229	S42015S	Fracture of upper limb

229	S42016A	Fracture of upper limb
229	S42016B	Fracture of upper limb
229	S42016S	Fracture of upper limb
229	S42017A	Fracture of upper limb
229	S42017B	Fracture of upper limb
229	S42017S	Fracture of upper limb
229	S42018A	Fracture of upper limb
229	S42018B	Fracture of upper limb
229	S42018S	Fracture of upper limb
229	S42019A	Fracture of upper limb
229	S42019B	Fracture of upper limb
229	S42019S	Fracture of upper limb
229	S42021A	Fracture of upper limb
229	S42021B	Fracture of upper limb
229	S42021S	Fracture of upper limb
229	S42022A	Fracture of upper limb
229	S42022B	Fracture of upper limb
229	S42022S	Fracture of upper limb
229	S42023A	Fracture of upper limb
229	S42023B	Fracture of upper limb
229	S42023S	Fracture of upper limb
229	S42024A	Fracture of upper limb
229	S42024B	Fracture of upper limb
229	S42024S	Fracture of upper limb
229	S42025A	Fracture of upper limb
229	S42025B	Fracture of upper limb
229	S42025S	Fracture of upper limb
229	S42026A	Fracture of upper limb
229	S42026B	Fracture of upper limb
229	S42026S	Fracture of upper limb
229	S42031A	Fracture of upper limb
229	S42031B	Fracture of upper limb
229	S42031S	Fracture of upper limb
229	S42032A	Fracture of upper limb
229	S42032B	Fracture of upper limb
229	S42032S	Fracture of upper limb
229	S42033A	Fracture of upper limb
229	S42033B	Fracture of upper limb
229	S42033S	Fracture of upper limb
229	S42034A	Fracture of upper limb
229	S42034B	Fracture of upper limb
229	S42034S	Fracture of upper limb
229	S42035A	Fracture of upper limb
229	S42035B	Fracture of upper limb
229	S42035S	Fracture of upper limb
229	S42036A	Fracture of upper limb
229	S42036B	Fracture of upper limb
229	S42036S	Fracture of upper limb
229	S42101A	Fracture of upper limb
229	S42101B	Fracture of upper limb
229	S42101D	Fracture of upper limb

229	S42101G	Fracture of upper limb
229	S42101K	Fracture of upper limb
229	S42101P	Fracture of upper limb
229	S42101S	Fracture of upper limb
229	S42102A	Fracture of upper limb
229	S42102B	Fracture of upper limb
229	S42102D	Fracture of upper limb
229	S42102G	Fracture of upper limb
229	S42102K	Fracture of upper limb
229	S42102P	Fracture of upper limb
229	S42102S	Fracture of upper limb
229	S42109A	Fracture of upper limb
229	S42109B	Fracture of upper limb
229	S42109D	Fracture of upper limb
229	S42109G	Fracture of upper limb
229	S42109K	Fracture of upper limb
229	S42109P	Fracture of upper limb
229	S42109S	Fracture of upper limb
229	S42111A	Fracture of upper limb
229	S42111B	Fracture of upper limb
229	S42111D	Fracture of upper limb
229	S42111G	Fracture of upper limb
229	S42111K	Fracture of upper limb
229	S42111P	Fracture of upper limb
229	S42111S	Fracture of upper limb
229	S42112A	Fracture of upper limb
229	S42112B	Fracture of upper limb
229	S42112D	Fracture of upper limb
229	S42112G	Fracture of upper limb
229	S42112K	Fracture of upper limb
229	S42112P	Fracture of upper limb
229	S42112S	Fracture of upper limb
229	S42113A	Fracture of upper limb
229	S42113B	Fracture of upper limb
229	S42113D	Fracture of upper limb
229	S42113G	Fracture of upper limb
229	S42113K	Fracture of upper limb
229	S42113P	Fracture of upper limb
229	S42113S	Fracture of upper limb
229	S42114A	Fracture of upper limb
229	S42114B	Fracture of upper limb
229	S42114D	Fracture of upper limb
229	S42114G	Fracture of upper limb
229	S42114K	Fracture of upper limb
229	S42114P	Fracture of upper limb
229	S42114S	Fracture of upper limb
229	S42115A	Fracture of upper limb
229	S42115B	Fracture of upper limb
229	S42115D	Fracture of upper limb
229	S42115G	Fracture of upper limb
229	S42115K	Fracture of upper limb

229	S42115P	Fracture of upper limb
229	S42115S	Fracture of upper limb
229	S42116A	Fracture of upper limb
229	S42116B	Fracture of upper limb
229	S42116D	Fracture of upper limb
229	S42116G	Fracture of upper limb
229	S42116K	Fracture of upper limb
229	S42116P	Fracture of upper limb
229	S42116S	Fracture of upper limb
229	S42121A	Fracture of upper limb
229	S42121B	Fracture of upper limb
229	S42121D	Fracture of upper limb
229	S42121G	Fracture of upper limb
229	S42121K	Fracture of upper limb
229	S42121P	Fracture of upper limb
229	S42121S	Fracture of upper limb
229	S42122A	Fracture of upper limb
229	S42122B	Fracture of upper limb
229	S42122D	Fracture of upper limb
229	S42122G	Fracture of upper limb
229	S42122K	Fracture of upper limb
229	S42122P	Fracture of upper limb
229	S42122S	Fracture of upper limb
229	S42123A	Fracture of upper limb
229	S42123B	Fracture of upper limb
229	S42123D	Fracture of upper limb
229	S42123G	Fracture of upper limb
229	S42123K	Fracture of upper limb
229	S42123P	Fracture of upper limb
229	S42123S	Fracture of upper limb
229	S42124A	Fracture of upper limb
229	S42124B	Fracture of upper limb
229	S42124D	Fracture of upper limb
229	S42124G	Fracture of upper limb
229	S42124K	Fracture of upper limb
229	S42124P	Fracture of upper limb
229	S42124S	Fracture of upper limb
229	S42125A	Fracture of upper limb
229	S42125B	Fracture of upper limb
229	S42125D	Fracture of upper limb
229	S42125G	Fracture of upper limb
229	S42125K	Fracture of upper limb
229	S42125P	Fracture of upper limb
229	S42125S	Fracture of upper limb
229	S42126A	Fracture of upper limb
229	S42126B	Fracture of upper limb
229	S42126D	Fracture of upper limb
229	S42126G	Fracture of upper limb
229	S42126K	Fracture of upper limb
229	S42126P	Fracture of upper limb
229	S42126S	Fracture of upper limb

229	S42131A	Fracture of upper limb
229	S42131B	Fracture of upper limb
229	S42131D	Fracture of upper limb
229	S42131G	Fracture of upper limb
229	S42131K	Fracture of upper limb
229	S42131P	Fracture of upper limb
229	S42131S	Fracture of upper limb
229	S42132A	Fracture of upper limb
229	S42132B	Fracture of upper limb
229	S42132D	Fracture of upper limb
229	S42132G	Fracture of upper limb
229	S42132K	Fracture of upper limb
229	S42132P	Fracture of upper limb
229	S42132S	Fracture of upper limb
229	S42133A	Fracture of upper limb
229	S42133B	Fracture of upper limb
229	S42133D	Fracture of upper limb
229	S42133G	Fracture of upper limb
229	S42133K	Fracture of upper limb
229	S42133P	Fracture of upper limb
229	S42133S	Fracture of upper limb
229	S42134A	Fracture of upper limb
229	S42134B	Fracture of upper limb
229	S42134D	Fracture of upper limb
229	S42134G	Fracture of upper limb
229	S42134K	Fracture of upper limb
229	S42134P	Fracture of upper limb
229	S42134S	Fracture of upper limb
229	S42135A	Fracture of upper limb
229	S42135B	Fracture of upper limb
229	S42135D	Fracture of upper limb
229	S42135G	Fracture of upper limb
229	S42135K	Fracture of upper limb
229	S42135P	Fracture of upper limb
229	S42135S	Fracture of upper limb
229	S42136A	Fracture of upper limb
229	S42136B	Fracture of upper limb
229	S42136D	Fracture of upper limb
229	S42136G	Fracture of upper limb
229	S42136K	Fracture of upper limb
229	S42136P	Fracture of upper limb
229	S42136S	Fracture of upper limb
229	S42141A	Fracture of upper limb
229	S42141B	Fracture of upper limb
229	S42141D	Fracture of upper limb
229	S42141G	Fracture of upper limb
229	S42141K	Fracture of upper limb
229	S42141P	Fracture of upper limb
229	S42141S	Fracture of upper limb
229	S42142A	Fracture of upper limb
229	S42142B	Fracture of upper limb

229	S42142D	Fracture of upper limb
229	S42142G	Fracture of upper limb
229	S42142K	Fracture of upper limb
229	S42142P	Fracture of upper limb
229	S42142S	Fracture of upper limb
229	S42143A	Fracture of upper limb
229	S42143B	Fracture of upper limb
229	S42143D	Fracture of upper limb
229	S42143G	Fracture of upper limb
229	S42143K	Fracture of upper limb
229	S42143P	Fracture of upper limb
229	S42143S	Fracture of upper limb
229	S42144A	Fracture of upper limb
229	S42144B	Fracture of upper limb
229	S42144D	Fracture of upper limb
229	S42144G	Fracture of upper limb
229	S42144K	Fracture of upper limb
229	S42144P	Fracture of upper limb
229	S42144S	Fracture of upper limb
229	S42145A	Fracture of upper limb
229	S42145B	Fracture of upper limb
229	S42145D	Fracture of upper limb
229	S42145G	Fracture of upper limb
229	S42145K	Fracture of upper limb
229	S42145P	Fracture of upper limb
229	S42145S	Fracture of upper limb
229	S42146A	Fracture of upper limb
229	S42146B	Fracture of upper limb
229	S42146D	Fracture of upper limb
229	S42146G	Fracture of upper limb
229	S42146K	Fracture of upper limb
229	S42146P	Fracture of upper limb
229	S42146S	Fracture of upper limb
229	S42151A	Fracture of upper limb
229	S42151B	Fracture of upper limb
229	S42151D	Fracture of upper limb
229	S42151G	Fracture of upper limb
229	S42151K	Fracture of upper limb
229	S42151P	Fracture of upper limb
229	S42151S	Fracture of upper limb
229	S42152A	Fracture of upper limb
229	S42152B	Fracture of upper limb
229	S42152D	Fracture of upper limb
229	S42152G	Fracture of upper limb
229	S42152K	Fracture of upper limb
229	S42152P	Fracture of upper limb
229	S42152S	Fracture of upper limb
229	S42153A	Fracture of upper limb
229	S42153B	Fracture of upper limb
229	S42153D	Fracture of upper limb
229	S42153G	Fracture of upper limb

229	S42153K	Fracture of upper limb
229	S42153P	Fracture of upper limb
229	S42153S	Fracture of upper limb
229	S42154A	Fracture of upper limb
229	S42154B	Fracture of upper limb
229	S42154D	Fracture of upper limb
229	S42154G	Fracture of upper limb
229	S42154K	Fracture of upper limb
229	S42154P	Fracture of upper limb
229	S42154S	Fracture of upper limb
229	S42155A	Fracture of upper limb
229	S42155B	Fracture of upper limb
229	S42155D	Fracture of upper limb
229	S42155G	Fracture of upper limb
229	S42155K	Fracture of upper limb
229	S42155P	Fracture of upper limb
229	S42155S	Fracture of upper limb
229	S42156A	Fracture of upper limb
229	S42156B	Fracture of upper limb
229	S42156D	Fracture of upper limb
229	S42156G	Fracture of upper limb
229	S42156K	Fracture of upper limb
229	S42156P	Fracture of upper limb
229	S42156S	Fracture of upper limb
229	S42191A	Fracture of upper limb
229	S42191B	Fracture of upper limb
229	S42191D	Fracture of upper limb
229	S42191G	Fracture of upper limb
229	S42191K	Fracture of upper limb
229	S42191P	Fracture of upper limb
229	S42191S	Fracture of upper limb
229	S42192A	Fracture of upper limb
229	S42192B	Fracture of upper limb
229	S42192D	Fracture of upper limb
229	S42192G	Fracture of upper limb
229	S42192K	Fracture of upper limb
229	S42192P	Fracture of upper limb
229	S42192S	Fracture of upper limb
229	S42199A	Fracture of upper limb
229	S42199B	Fracture of upper limb
229	S42199D	Fracture of upper limb
229	S42199G	Fracture of upper limb
229	S42199K	Fracture of upper limb
229	S42199P	Fracture of upper limb
229	S42199S	Fracture of upper limb
229	S42201A	Fracture of upper limb
229	S42201B	Fracture of upper limb
229	S42201D	Fracture of upper limb
229	S42201G	Fracture of upper limb
229	S42201K	Fracture of upper limb
229	S42201P	Fracture of upper limb

229	S42201S	Fracture of upper limb
229	S42202A	Fracture of upper limb
229	S42202B	Fracture of upper limb
229	S42202D	Fracture of upper limb
229	S42202G	Fracture of upper limb
229	S42202K	Fracture of upper limb
229	S42202P	Fracture of upper limb
229	S42202S	Fracture of upper limb
229	S42209A	Fracture of upper limb
229	S42209B	Fracture of upper limb
229	S42209D	Fracture of upper limb
229	S42209G	Fracture of upper limb
229	S42209K	Fracture of upper limb
229	S42209P	Fracture of upper limb
229	S42209S	Fracture of upper limb
229	S42211A	Fracture of upper limb
229	S42211B	Fracture of upper limb
229	S42211D	Fracture of upper limb
229	S42211G	Fracture of upper limb
229	S42211K	Fracture of upper limb
229	S42211P	Fracture of upper limb
229	S42211S	Fracture of upper limb
229	S42212A	Fracture of upper limb
229	S42212B	Fracture of upper limb
229	S42212D	Fracture of upper limb
229	S42212G	Fracture of upper limb
229	S42212K	Fracture of upper limb
229	S42212P	Fracture of upper limb
229	S42212S	Fracture of upper limb
229	S42213A	Fracture of upper limb
229	S42213B	Fracture of upper limb
229	S42213D	Fracture of upper limb
229	S42213G	Fracture of upper limb
229	S42213K	Fracture of upper limb
229	S42213P	Fracture of upper limb
229	S42213S	Fracture of upper limb
229	S42214A	Fracture of upper limb
229	S42214B	Fracture of upper limb
229	S42214D	Fracture of upper limb
229	S42214G	Fracture of upper limb
229	S42214K	Fracture of upper limb
229	S42214P	Fracture of upper limb
229	S42214S	Fracture of upper limb
229	S42215A	Fracture of upper limb
229	S42215B	Fracture of upper limb
229	S42215D	Fracture of upper limb
229	S42215G	Fracture of upper limb
229	S42215K	Fracture of upper limb
229	S42215P	Fracture of upper limb
229	S42215S	Fracture of upper limb
229	S42216A	Fracture of upper limb

229	S42216B	Fracture of upper limb
229	S42216D	Fracture of upper limb
229	S42216G	Fracture of upper limb
229	S42216K	Fracture of upper limb
229	S42216P	Fracture of upper limb
229	S42216S	Fracture of upper limb
229	S42221A	Fracture of upper limb
229	S42221B	Fracture of upper limb
229	S42221D	Fracture of upper limb
229	S42221G	Fracture of upper limb
229	S42221K	Fracture of upper limb
229	S42221P	Fracture of upper limb
229	S42221S	Fracture of upper limb
229	S42222A	Fracture of upper limb
229	S42222B	Fracture of upper limb
229	S42222D	Fracture of upper limb
229	S42222G	Fracture of upper limb
229	S42222K	Fracture of upper limb
229	S42222P	Fracture of upper limb
229	S42222S	Fracture of upper limb
229	S42223A	Fracture of upper limb
229	S42223B	Fracture of upper limb
229	S42223D	Fracture of upper limb
229	S42223G	Fracture of upper limb
229	S42223K	Fracture of upper limb
229	S42223P	Fracture of upper limb
229	S42223S	Fracture of upper limb
229	S42224A	Fracture of upper limb
229	S42224B	Fracture of upper limb
229	S42224D	Fracture of upper limb
229	S42224G	Fracture of upper limb
229	S42224K	Fracture of upper limb
229	S42224P	Fracture of upper limb
229	S42224S	Fracture of upper limb
229	S42225A	Fracture of upper limb
229	S42225B	Fracture of upper limb
229	S42225D	Fracture of upper limb
229	S42225G	Fracture of upper limb
229	S42225K	Fracture of upper limb
229	S42225P	Fracture of upper limb
229	S42225S	Fracture of upper limb
229	S42226A	Fracture of upper limb
229	S42226B	Fracture of upper limb
229	S42226D	Fracture of upper limb
229	S42226G	Fracture of upper limb
229	S42226K	Fracture of upper limb
229	S42226P	Fracture of upper limb
229	S42226S	Fracture of upper limb
229	S42231A	Fracture of upper limb
229	S42231B	Fracture of upper limb
229	S42231D	Fracture of upper limb

229	S42231G	Fracture of upper limb
229	S42231K	Fracture of upper limb
229	S42231P	Fracture of upper limb
229	S42231S	Fracture of upper limb
229	S42232A	Fracture of upper limb
229	S42232B	Fracture of upper limb
229	S42232D	Fracture of upper limb
229	S42232G	Fracture of upper limb
229	S42232K	Fracture of upper limb
229	S42232P	Fracture of upper limb
229	S42232S	Fracture of upper limb
229	S42239A	Fracture of upper limb
229	S42239B	Fracture of upper limb
229	S42239D	Fracture of upper limb
229	S42239G	Fracture of upper limb
229	S42239K	Fracture of upper limb
229	S42239P	Fracture of upper limb
229	S42239S	Fracture of upper limb
229	S42241A	Fracture of upper limb
229	S42241B	Fracture of upper limb
229	S42241D	Fracture of upper limb
229	S42241G	Fracture of upper limb
229	S42241K	Fracture of upper limb
229	S42241P	Fracture of upper limb
229	S42241S	Fracture of upper limb
229	S42242A	Fracture of upper limb
229	S42242B	Fracture of upper limb
229	S42242D	Fracture of upper limb
229	S42242G	Fracture of upper limb
229	S42242K	Fracture of upper limb
229	S42242P	Fracture of upper limb
229	S42242S	Fracture of upper limb
229	S42249A	Fracture of upper limb
229	S42249B	Fracture of upper limb
229	S42249D	Fracture of upper limb
229	S42249G	Fracture of upper limb
229	S42249K	Fracture of upper limb
229	S42249P	Fracture of upper limb
229	S42249S	Fracture of upper limb
229	S42251A	Fracture of upper limb
229	S42251B	Fracture of upper limb
229	S42251D	Fracture of upper limb
229	S42251G	Fracture of upper limb
229	S42251K	Fracture of upper limb
229	S42251P	Fracture of upper limb
229	S42251S	Fracture of upper limb
229	S42252A	Fracture of upper limb
229	S42252B	Fracture of upper limb
229	S42252D	Fracture of upper limb
229	S42252G	Fracture of upper limb
229	S42252K	Fracture of upper limb

229	S42252P	Fracture of upper limb
229	S42252S	Fracture of upper limb
229	S42253A	Fracture of upper limb
229	S42253B	Fracture of upper limb
229	S42253D	Fracture of upper limb
229	S42253G	Fracture of upper limb
229	S42253K	Fracture of upper limb
229	S42253P	Fracture of upper limb
229	S42253S	Fracture of upper limb
229	S42254A	Fracture of upper limb
229	S42254B	Fracture of upper limb
229	S42254D	Fracture of upper limb
229	S42254G	Fracture of upper limb
229	S42254K	Fracture of upper limb
229	S42254P	Fracture of upper limb
229	S42254S	Fracture of upper limb
229	S42255A	Fracture of upper limb
229	S42255B	Fracture of upper limb
229	S42255D	Fracture of upper limb
229	S42255G	Fracture of upper limb
229	S42255K	Fracture of upper limb
229	S42255P	Fracture of upper limb
229	S42255S	Fracture of upper limb
229	S42256A	Fracture of upper limb
229	S42256B	Fracture of upper limb
229	S42256D	Fracture of upper limb
229	S42256G	Fracture of upper limb
229	S42256K	Fracture of upper limb
229	S42256P	Fracture of upper limb
229	S42256S	Fracture of upper limb
229	S42261A	Fracture of upper limb
229	S42261B	Fracture of upper limb
229	S42261D	Fracture of upper limb
229	S42261G	Fracture of upper limb
229	S42261K	Fracture of upper limb
229	S42261P	Fracture of upper limb
229	S42261S	Fracture of upper limb
229	S42262A	Fracture of upper limb
229	S42262B	Fracture of upper limb
229	S42262D	Fracture of upper limb
229	S42262G	Fracture of upper limb
229	S42262K	Fracture of upper limb
229	S42262P	Fracture of upper limb
229	S42262S	Fracture of upper limb
229	S42263A	Fracture of upper limb
229	S42263B	Fracture of upper limb
229	S42263D	Fracture of upper limb
229	S42263G	Fracture of upper limb
229	S42263K	Fracture of upper limb
229	S42263P	Fracture of upper limb
229	S42263S	Fracture of upper limb

229	S42264A	Fracture of upper limb
229	S42264B	Fracture of upper limb
229	S42264D	Fracture of upper limb
229	S42264G	Fracture of upper limb
229	S42264K	Fracture of upper limb
229	S42264P	Fracture of upper limb
229	S42264S	Fracture of upper limb
229	S42265A	Fracture of upper limb
229	S42265B	Fracture of upper limb
229	S42265D	Fracture of upper limb
229	S42265G	Fracture of upper limb
229	S42265K	Fracture of upper limb
229	S42265P	Fracture of upper limb
229	S42265S	Fracture of upper limb
229	S42266A	Fracture of upper limb
229	S42266B	Fracture of upper limb
229	S42266D	Fracture of upper limb
229	S42266G	Fracture of upper limb
229	S42266K	Fracture of upper limb
229	S42266P	Fracture of upper limb
229	S42266S	Fracture of upper limb
229	S42271A	Fracture of upper limb
229	S42271D	Fracture of upper limb
229	S42271G	Fracture of upper limb
229	S42271K	Fracture of upper limb
229	S42271P	Fracture of upper limb
229	S42271S	Fracture of upper limb
229	S42272A	Fracture of upper limb
229	S42272D	Fracture of upper limb
229	S42272G	Fracture of upper limb
229	S42272K	Fracture of upper limb
229	S42272P	Fracture of upper limb
229	S42272S	Fracture of upper limb
229	S42279A	Fracture of upper limb
229	S42279D	Fracture of upper limb
229	S42279G	Fracture of upper limb
229	S42279K	Fracture of upper limb
229	S42279P	Fracture of upper limb
229	S42279S	Fracture of upper limb
229	S42291A	Fracture of upper limb
229	S42291B	Fracture of upper limb
229	S42291D	Fracture of upper limb
229	S42291G	Fracture of upper limb
229	S42291K	Fracture of upper limb
229	S42291P	Fracture of upper limb
229	S42291S	Fracture of upper limb
229	S42292A	Fracture of upper limb
229	S42292B	Fracture of upper limb
229	S42292D	Fracture of upper limb
229	S42292G	Fracture of upper limb
229	S42292K	Fracture of upper limb

229	S42292P	Fracture of upper limb
229	S42292S	Fracture of upper limb
229	S42293A	Fracture of upper limb
229	S42293B	Fracture of upper limb
229	S42293D	Fracture of upper limb
229	S42293G	Fracture of upper limb
229	S42293K	Fracture of upper limb
229	S42293P	Fracture of upper limb
229	S42293S	Fracture of upper limb
229	S42294A	Fracture of upper limb
229	S42294B	Fracture of upper limb
229	S42294D	Fracture of upper limb
229	S42294G	Fracture of upper limb
229	S42294K	Fracture of upper limb
229	S42294P	Fracture of upper limb
229	S42294S	Fracture of upper limb
229	S42295A	Fracture of upper limb
229	S42295B	Fracture of upper limb
229	S42295D	Fracture of upper limb
229	S42295G	Fracture of upper limb
229	S42295K	Fracture of upper limb
229	S42295P	Fracture of upper limb
229	S42295S	Fracture of upper limb
229	S42296A	Fracture of upper limb
229	S42296B	Fracture of upper limb
229	S42296D	Fracture of upper limb
229	S42296G	Fracture of upper limb
229	S42296K	Fracture of upper limb
229	S42296P	Fracture of upper limb
229	S42296S	Fracture of upper limb
229	S42301A	Fracture of upper limb
229	S42301B	Fracture of upper limb
229	S42301D	Fracture of upper limb
229	S42301G	Fracture of upper limb
229	S42301K	Fracture of upper limb
229	S42301P	Fracture of upper limb
229	S42301S	Fracture of upper limb
229	S42302A	Fracture of upper limb
229	S42302B	Fracture of upper limb
229	S42302D	Fracture of upper limb
229	S42302G	Fracture of upper limb
229	S42302K	Fracture of upper limb
229	S42302P	Fracture of upper limb
229	S42302S	Fracture of upper limb
229	S42309A	Fracture of upper limb
229	S42309B	Fracture of upper limb
229	S42309D	Fracture of upper limb
229	S42309G	Fracture of upper limb
229	S42309K	Fracture of upper limb
229	S42309P	Fracture of upper limb
229	S42309S	Fracture of upper limb

229	S42311A	Fracture of upper limb
229	S42311D	Fracture of upper limb
229	S42311G	Fracture of upper limb
229	S42311K	Fracture of upper limb
229	S42311P	Fracture of upper limb
229	S42311S	Fracture of upper limb
229	S42312A	Fracture of upper limb
229	S42312D	Fracture of upper limb
229	S42312G	Fracture of upper limb
229	S42312K	Fracture of upper limb
229	S42312P	Fracture of upper limb
229	S42312S	Fracture of upper limb
229	S42319A	Fracture of upper limb
229	S42319D	Fracture of upper limb
229	S42319G	Fracture of upper limb
229	S42319K	Fracture of upper limb
229	S42319P	Fracture of upper limb
229	S42319S	Fracture of upper limb
229	S42321A	Fracture of upper limb
229	S42321B	Fracture of upper limb
229	S42321D	Fracture of upper limb
229	S42321G	Fracture of upper limb
229	S42321K	Fracture of upper limb
229	S42321P	Fracture of upper limb
229	S42321S	Fracture of upper limb
229	S42322A	Fracture of upper limb
229	S42322B	Fracture of upper limb
229	S42322D	Fracture of upper limb
229	S42322G	Fracture of upper limb
229	S42322K	Fracture of upper limb
229	S42322P	Fracture of upper limb
229	S42322S	Fracture of upper limb
229	S42323A	Fracture of upper limb
229	S42323B	Fracture of upper limb
229	S42323D	Fracture of upper limb
229	S42323G	Fracture of upper limb
229	S42323K	Fracture of upper limb
229	S42323P	Fracture of upper limb
229	S42323S	Fracture of upper limb
229	S42324A	Fracture of upper limb
229	S42324B	Fracture of upper limb
229	S42324D	Fracture of upper limb
229	S42324G	Fracture of upper limb
229	S42324K	Fracture of upper limb
229	S42324P	Fracture of upper limb
229	S42324S	Fracture of upper limb
229	S42325A	Fracture of upper limb
229	S42325B	Fracture of upper limb
229	S42325D	Fracture of upper limb
229	S42325G	Fracture of upper limb
229	S42325K	Fracture of upper limb

229	S42325P	Fracture of upper limb
229	S42325S	Fracture of upper limb
229	S42326A	Fracture of upper limb
229	S42326B	Fracture of upper limb
229	S42326D	Fracture of upper limb
229	S42326G	Fracture of upper limb
229	S42326K	Fracture of upper limb
229	S42326P	Fracture of upper limb
229	S42326S	Fracture of upper limb
229	S42331A	Fracture of upper limb
229	S42331B	Fracture of upper limb
229	S42331D	Fracture of upper limb
229	S42331G	Fracture of upper limb
229	S42331K	Fracture of upper limb
229	S42331P	Fracture of upper limb
229	S42331S	Fracture of upper limb
229	S42332A	Fracture of upper limb
229	S42332B	Fracture of upper limb
229	S42332D	Fracture of upper limb
229	S42332G	Fracture of upper limb
229	S42332K	Fracture of upper limb
229	S42332P	Fracture of upper limb
229	S42332S	Fracture of upper limb
229	S42333A	Fracture of upper limb
229	S42333B	Fracture of upper limb
229	S42333D	Fracture of upper limb
229	S42333G	Fracture of upper limb
229	S42333K	Fracture of upper limb
229	S42333P	Fracture of upper limb
229	S42333S	Fracture of upper limb
229	S42334A	Fracture of upper limb
229	S42334B	Fracture of upper limb
229	S42334D	Fracture of upper limb
229	S42334G	Fracture of upper limb
229	S42334K	Fracture of upper limb
229	S42334P	Fracture of upper limb
229	S42334S	Fracture of upper limb
229	S42335A	Fracture of upper limb
229	S42335B	Fracture of upper limb
229	S42335D	Fracture of upper limb
229	S42335G	Fracture of upper limb
229	S42335K	Fracture of upper limb
229	S42335P	Fracture of upper limb
229	S42335S	Fracture of upper limb
229	S42336A	Fracture of upper limb
229	S42336B	Fracture of upper limb
229	S42336D	Fracture of upper limb
229	S42336G	Fracture of upper limb
229	S42336K	Fracture of upper limb
229	S42336P	Fracture of upper limb
229	S42336S	Fracture of upper limb

229	S42341A	Fracture of upper limb
229	S42341B	Fracture of upper limb
229	S42341D	Fracture of upper limb
229	S42341G	Fracture of upper limb
229	S42341K	Fracture of upper limb
229	S42341P	Fracture of upper limb
229	S42341S	Fracture of upper limb
229	S42342A	Fracture of upper limb
229	S42342B	Fracture of upper limb
229	S42342D	Fracture of upper limb
229	S42342G	Fracture of upper limb
229	S42342K	Fracture of upper limb
229	S42342P	Fracture of upper limb
229	S42342S	Fracture of upper limb
229	S42343A	Fracture of upper limb
229	S42343B	Fracture of upper limb
229	S42343D	Fracture of upper limb
229	S42343G	Fracture of upper limb
229	S42343K	Fracture of upper limb
229	S42343P	Fracture of upper limb
229	S42343S	Fracture of upper limb
229	S42344A	Fracture of upper limb
229	S42344B	Fracture of upper limb
229	S42344D	Fracture of upper limb
229	S42344G	Fracture of upper limb
229	S42344K	Fracture of upper limb
229	S42344P	Fracture of upper limb
229	S42344S	Fracture of upper limb
229	S42345A	Fracture of upper limb
229	S42345B	Fracture of upper limb
229	S42345D	Fracture of upper limb
229	S42345G	Fracture of upper limb
229	S42345K	Fracture of upper limb
229	S42345P	Fracture of upper limb
229	S42345S	Fracture of upper limb
229	S42346A	Fracture of upper limb
229	S42346B	Fracture of upper limb
229	S42346D	Fracture of upper limb
229	S42346G	Fracture of upper limb
229	S42346K	Fracture of upper limb
229	S42346P	Fracture of upper limb
229	S42346S	Fracture of upper limb
229	S42351A	Fracture of upper limb
229	S42351B	Fracture of upper limb
229	S42351D	Fracture of upper limb
229	S42351G	Fracture of upper limb
229	S42351K	Fracture of upper limb
229	S42351P	Fracture of upper limb
229	S42351S	Fracture of upper limb
229	S42352A	Fracture of upper limb
229	S42352B	Fracture of upper limb

229	S42352D	Fracture of upper limb
229	S42352G	Fracture of upper limb
229	S42352K	Fracture of upper limb
229	S42352P	Fracture of upper limb
229	S42352S	Fracture of upper limb
229	S42353A	Fracture of upper limb
229	S42353B	Fracture of upper limb
229	S42353D	Fracture of upper limb
229	S42353G	Fracture of upper limb
229	S42353K	Fracture of upper limb
229	S42353P	Fracture of upper limb
229	S42353S	Fracture of upper limb
229	S42354A	Fracture of upper limb
229	S42354B	Fracture of upper limb
229	S42354D	Fracture of upper limb
229	S42354G	Fracture of upper limb
229	S42354K	Fracture of upper limb
229	S42354P	Fracture of upper limb
229	S42354S	Fracture of upper limb
229	S42355A	Fracture of upper limb
229	S42355B	Fracture of upper limb
229	S42355D	Fracture of upper limb
229	S42355G	Fracture of upper limb
229	S42355K	Fracture of upper limb
229	S42355P	Fracture of upper limb
229	S42355S	Fracture of upper limb
229	S42356A	Fracture of upper limb
229	S42356B	Fracture of upper limb
229	S42356D	Fracture of upper limb
229	S42356G	Fracture of upper limb
229	S42356K	Fracture of upper limb
229	S42356P	Fracture of upper limb
229	S42356S	Fracture of upper limb
229	S42361A	Fracture of upper limb
229	S42361B	Fracture of upper limb
229	S42361D	Fracture of upper limb
229	S42361G	Fracture of upper limb
229	S42361K	Fracture of upper limb
229	S42361P	Fracture of upper limb
229	S42361S	Fracture of upper limb
229	S42362A	Fracture of upper limb
229	S42362B	Fracture of upper limb
229	S42362D	Fracture of upper limb
229	S42362G	Fracture of upper limb
229	S42362K	Fracture of upper limb
229	S42362P	Fracture of upper limb
229	S42362S	Fracture of upper limb
229	S42363A	Fracture of upper limb
229	S42363B	Fracture of upper limb
229	S42363D	Fracture of upper limb
229	S42363G	Fracture of upper limb

229	S42363K	Fracture of upper limb
229	S42363P	Fracture of upper limb
229	S42363S	Fracture of upper limb
229	S42364A	Fracture of upper limb
229	S42364B	Fracture of upper limb
229	S42364D	Fracture of upper limb
229	S42364G	Fracture of upper limb
229	S42364K	Fracture of upper limb
229	S42364P	Fracture of upper limb
229	S42364S	Fracture of upper limb
229	S42365A	Fracture of upper limb
229	S42365B	Fracture of upper limb
229	S42365D	Fracture of upper limb
229	S42365G	Fracture of upper limb
229	S42365K	Fracture of upper limb
229	S42365P	Fracture of upper limb
229	S42365S	Fracture of upper limb
229	S42366A	Fracture of upper limb
229	S42366B	Fracture of upper limb
229	S42366D	Fracture of upper limb
229	S42366G	Fracture of upper limb
229	S42366K	Fracture of upper limb
229	S42366P	Fracture of upper limb
229	S42366S	Fracture of upper limb
229	S42391A	Fracture of upper limb
229	S42391B	Fracture of upper limb
229	S42391D	Fracture of upper limb
229	S42391G	Fracture of upper limb
229	S42391K	Fracture of upper limb
229	S42391P	Fracture of upper limb
229	S42391S	Fracture of upper limb
229	S42392A	Fracture of upper limb
229	S42392B	Fracture of upper limb
229	S42392D	Fracture of upper limb
229	S42392G	Fracture of upper limb
229	S42392K	Fracture of upper limb
229	S42392P	Fracture of upper limb
229	S42392S	Fracture of upper limb
229	S42399A	Fracture of upper limb
229	S42399B	Fracture of upper limb
229	S42399D	Fracture of upper limb
229	S42399G	Fracture of upper limb
229	S42399K	Fracture of upper limb
229	S42399P	Fracture of upper limb
229	S42399S	Fracture of upper limb
229	S42401A	Fracture of upper limb
229	S42401B	Fracture of upper limb
229	S42401D	Fracture of upper limb
229	S42401G	Fracture of upper limb
229	S42401K	Fracture of upper limb
229	S42401P	Fracture of upper limb

229	S42401S	Fracture of upper limb
229	S42402A	Fracture of upper limb
229	S42402B	Fracture of upper limb
229	S42402D	Fracture of upper limb
229	S42402G	Fracture of upper limb
229	S42402K	Fracture of upper limb
229	S42402P	Fracture of upper limb
229	S42402S	Fracture of upper limb
229	S42409A	Fracture of upper limb
229	S42409B	Fracture of upper limb
229	S42409D	Fracture of upper limb
229	S42409G	Fracture of upper limb
229	S42409K	Fracture of upper limb
229	S42409P	Fracture of upper limb
229	S42409S	Fracture of upper limb
229	S42411A	Fracture of upper limb
229	S42411B	Fracture of upper limb
229	S42411D	Fracture of upper limb
229	S42411G	Fracture of upper limb
229	S42411K	Fracture of upper limb
229	S42411P	Fracture of upper limb
229	S42411S	Fracture of upper limb
229	S42412A	Fracture of upper limb
229	S42412B	Fracture of upper limb
229	S42412D	Fracture of upper limb
229	S42412G	Fracture of upper limb
229	S42412K	Fracture of upper limb
229	S42412P	Fracture of upper limb
229	S42412S	Fracture of upper limb
229	S42413A	Fracture of upper limb
229	S42413B	Fracture of upper limb
229	S42413D	Fracture of upper limb
229	S42413G	Fracture of upper limb
229	S42413K	Fracture of upper limb
229	S42413P	Fracture of upper limb
229	S42413S	Fracture of upper limb
229	S42414A	Fracture of upper limb
229	S42414B	Fracture of upper limb
229	S42414D	Fracture of upper limb
229	S42414G	Fracture of upper limb
229	S42414K	Fracture of upper limb
229	S42414P	Fracture of upper limb
229	S42414S	Fracture of upper limb
229	S42415A	Fracture of upper limb
229	S42415B	Fracture of upper limb
229	S42415D	Fracture of upper limb
229	S42415G	Fracture of upper limb
229	S42415K	Fracture of upper limb
229	S42415P	Fracture of upper limb
229	S42415S	Fracture of upper limb
229	S42416A	Fracture of upper limb

229	S42416B	Fracture of upper limb
229	S42416D	Fracture of upper limb
229	S42416G	Fracture of upper limb
229	S42416K	Fracture of upper limb
229	S42416P	Fracture of upper limb
229	S42416S	Fracture of upper limb
229	S42421A	Fracture of upper limb
229	S42421B	Fracture of upper limb
229	S42421D	Fracture of upper limb
229	S42421G	Fracture of upper limb
229	S42421K	Fracture of upper limb
229	S42421P	Fracture of upper limb
229	S42421S	Fracture of upper limb
229	S42422A	Fracture of upper limb
229	S42422B	Fracture of upper limb
229	S42422D	Fracture of upper limb
229	S42422G	Fracture of upper limb
229	S42422K	Fracture of upper limb
229	S42422P	Fracture of upper limb
229	S42422S	Fracture of upper limb
229	S42423A	Fracture of upper limb
229	S42423B	Fracture of upper limb
229	S42423D	Fracture of upper limb
229	S42423G	Fracture of upper limb
229	S42423K	Fracture of upper limb
229	S42423P	Fracture of upper limb
229	S42423S	Fracture of upper limb
229	S42424A	Fracture of upper limb
229	S42424B	Fracture of upper limb
229	S42424D	Fracture of upper limb
229	S42424G	Fracture of upper limb
229	S42424K	Fracture of upper limb
229	S42424P	Fracture of upper limb
229	S42424S	Fracture of upper limb
229	S42425A	Fracture of upper limb
229	S42425B	Fracture of upper limb
229	S42425D	Fracture of upper limb
229	S42425G	Fracture of upper limb
229	S42425K	Fracture of upper limb
229	S42425P	Fracture of upper limb
229	S42425S	Fracture of upper limb
229	S42426A	Fracture of upper limb
229	S42426B	Fracture of upper limb
229	S42426D	Fracture of upper limb
229	S42426G	Fracture of upper limb
229	S42426K	Fracture of upper limb
229	S42426P	Fracture of upper limb
229	S42426S	Fracture of upper limb
229	S42431A	Fracture of upper limb
229	S42431B	Fracture of upper limb
229	S42431D	Fracture of upper limb

229	S42431G	Fracture of upper limb
229	S42431K	Fracture of upper limb
229	S42431P	Fracture of upper limb
229	S42431S	Fracture of upper limb
229	S42432A	Fracture of upper limb
229	S42432B	Fracture of upper limb
229	S42432D	Fracture of upper limb
229	S42432G	Fracture of upper limb
229	S42432K	Fracture of upper limb
229	S42432P	Fracture of upper limb
229	S42432S	Fracture of upper limb
229	S42433A	Fracture of upper limb
229	S42433B	Fracture of upper limb
229	S42433D	Fracture of upper limb
229	S42433G	Fracture of upper limb
229	S42433K	Fracture of upper limb
229	S42433P	Fracture of upper limb
229	S42433S	Fracture of upper limb
229	S42434A	Fracture of upper limb
229	S42434B	Fracture of upper limb
229	S42434D	Fracture of upper limb
229	S42434G	Fracture of upper limb
229	S42434K	Fracture of upper limb
229	S42434P	Fracture of upper limb
229	S42434S	Fracture of upper limb
229	S42435A	Fracture of upper limb
229	S42435B	Fracture of upper limb
229	S42435D	Fracture of upper limb
229	S42435G	Fracture of upper limb
229	S42435K	Fracture of upper limb
229	S42435P	Fracture of upper limb
229	S42435S	Fracture of upper limb
229	S42436A	Fracture of upper limb
229	S42436B	Fracture of upper limb
229	S42436D	Fracture of upper limb
229	S42436G	Fracture of upper limb
229	S42436K	Fracture of upper limb
229	S42436P	Fracture of upper limb
229	S42436S	Fracture of upper limb
229	S42441A	Fracture of upper limb
229	S42441B	Fracture of upper limb
229	S42441D	Fracture of upper limb
229	S42441G	Fracture of upper limb
229	S42441K	Fracture of upper limb
229	S42441P	Fracture of upper limb
229	S42441S	Fracture of upper limb
229	S42442A	Fracture of upper limb
229	S42442B	Fracture of upper limb
229	S42442D	Fracture of upper limb
229	S42442G	Fracture of upper limb
229	S42442K	Fracture of upper limb

229	S42442P	Fracture of upper limb
229	S42442S	Fracture of upper limb
229	S42443A	Fracture of upper limb
229	S42443B	Fracture of upper limb
229	S42443D	Fracture of upper limb
229	S42443G	Fracture of upper limb
229	S42443K	Fracture of upper limb
229	S42443P	Fracture of upper limb
229	S42443S	Fracture of upper limb
229	S42444A	Fracture of upper limb
229	S42444B	Fracture of upper limb
229	S42444D	Fracture of upper limb
229	S42444G	Fracture of upper limb
229	S42444K	Fracture of upper limb
229	S42444P	Fracture of upper limb
229	S42444S	Fracture of upper limb
229	S42445A	Fracture of upper limb
229	S42445B	Fracture of upper limb
229	S42445D	Fracture of upper limb
229	S42445G	Fracture of upper limb
229	S42445K	Fracture of upper limb
229	S42445P	Fracture of upper limb
229	S42445S	Fracture of upper limb
229	S42446A	Fracture of upper limb
229	S42446B	Fracture of upper limb
229	S42446D	Fracture of upper limb
229	S42446G	Fracture of upper limb
229	S42446K	Fracture of upper limb
229	S42446P	Fracture of upper limb
229	S42446S	Fracture of upper limb
229	S42447A	Fracture of upper limb
229	S42447B	Fracture of upper limb
229	S42447D	Fracture of upper limb
229	S42447G	Fracture of upper limb
229	S42447K	Fracture of upper limb
229	S42447P	Fracture of upper limb
229	S42447S	Fracture of upper limb
229	S42448A	Fracture of upper limb
229	S42448B	Fracture of upper limb
229	S42448D	Fracture of upper limb
229	S42448G	Fracture of upper limb
229	S42448K	Fracture of upper limb
229	S42448P	Fracture of upper limb
229	S42448S	Fracture of upper limb
229	S42449A	Fracture of upper limb
229	S42449B	Fracture of upper limb
229	S42449D	Fracture of upper limb
229	S42449G	Fracture of upper limb
229	S42449K	Fracture of upper limb
229	S42449P	Fracture of upper limb
229	S42449S	Fracture of upper limb

229	S42451A	Fracture of upper limb
229	S42451B	Fracture of upper limb
229	S42451D	Fracture of upper limb
229	S42451G	Fracture of upper limb
229	S42451K	Fracture of upper limb
229	S42451P	Fracture of upper limb
229	S42451S	Fracture of upper limb
229	S42452A	Fracture of upper limb
229	S42452B	Fracture of upper limb
229	S42452D	Fracture of upper limb
229	S42452G	Fracture of upper limb
229	S42452K	Fracture of upper limb
229	S42452P	Fracture of upper limb
229	S42452S	Fracture of upper limb
229	S42453A	Fracture of upper limb
229	S42453B	Fracture of upper limb
229	S42453D	Fracture of upper limb
229	S42453G	Fracture of upper limb
229	S42453K	Fracture of upper limb
229	S42453P	Fracture of upper limb
229	S42453S	Fracture of upper limb
229	S42454A	Fracture of upper limb
229	S42454B	Fracture of upper limb
229	S42454D	Fracture of upper limb
229	S42454G	Fracture of upper limb
229	S42454K	Fracture of upper limb
229	S42454P	Fracture of upper limb
229	S42454S	Fracture of upper limb
229	S42455A	Fracture of upper limb
229	S42455B	Fracture of upper limb
229	S42455D	Fracture of upper limb
229	S42455G	Fracture of upper limb
229	S42455K	Fracture of upper limb
229	S42455P	Fracture of upper limb
229	S42455S	Fracture of upper limb
229	S42456A	Fracture of upper limb
229	S42456B	Fracture of upper limb
229	S42456D	Fracture of upper limb
229	S42456G	Fracture of upper limb
229	S42456K	Fracture of upper limb
229	S42456P	Fracture of upper limb
229	S42456S	Fracture of upper limb
229	S42461A	Fracture of upper limb
229	S42461B	Fracture of upper limb
229	S42461D	Fracture of upper limb
229	S42461G	Fracture of upper limb
229	S42461K	Fracture of upper limb
229	S42461P	Fracture of upper limb
229	S42461S	Fracture of upper limb
229	S42462A	Fracture of upper limb
229	S42462B	Fracture of upper limb

229	S42462D	Fracture of upper limb
229	S42462G	Fracture of upper limb
229	S42462K	Fracture of upper limb
229	S42462P	Fracture of upper limb
229	S42462S	Fracture of upper limb
229	S42463A	Fracture of upper limb
229	S42463B	Fracture of upper limb
229	S42463D	Fracture of upper limb
229	S42463G	Fracture of upper limb
229	S42463K	Fracture of upper limb
229	S42463P	Fracture of upper limb
229	S42463S	Fracture of upper limb
229	S42464A	Fracture of upper limb
229	S42464B	Fracture of upper limb
229	S42464D	Fracture of upper limb
229	S42464G	Fracture of upper limb
229	S42464K	Fracture of upper limb
229	S42464P	Fracture of upper limb
229	S42464S	Fracture of upper limb
229	S42465A	Fracture of upper limb
229	S42465B	Fracture of upper limb
229	S42465D	Fracture of upper limb
229	S42465G	Fracture of upper limb
229	S42465K	Fracture of upper limb
229	S42465P	Fracture of upper limb
229	S42465S	Fracture of upper limb
229	S42466A	Fracture of upper limb
229	S42466B	Fracture of upper limb
229	S42466D	Fracture of upper limb
229	S42466G	Fracture of upper limb
229	S42466K	Fracture of upper limb
229	S42466P	Fracture of upper limb
229	S42466S	Fracture of upper limb
229	S42471A	Fracture of upper limb
229	S42471B	Fracture of upper limb
229	S42471D	Fracture of upper limb
229	S42471G	Fracture of upper limb
229	S42471K	Fracture of upper limb
229	S42471P	Fracture of upper limb
229	S42471S	Fracture of upper limb
229	S42472A	Fracture of upper limb
229	S42472B	Fracture of upper limb
229	S42472D	Fracture of upper limb
229	S42472G	Fracture of upper limb
229	S42472K	Fracture of upper limb
229	S42472P	Fracture of upper limb
229	S42472S	Fracture of upper limb
229	S42473A	Fracture of upper limb
229	S42473B	Fracture of upper limb
229	S42473D	Fracture of upper limb
229	S42473G	Fracture of upper limb

229	S42473K	Fracture of upper limb
229	S42473P	Fracture of upper limb
229	S42473S	Fracture of upper limb
229	S42474A	Fracture of upper limb
229	S42474B	Fracture of upper limb
229	S42474D	Fracture of upper limb
229	S42474G	Fracture of upper limb
229	S42474K	Fracture of upper limb
229	S42474P	Fracture of upper limb
229	S42474S	Fracture of upper limb
229	S42475A	Fracture of upper limb
229	S42475B	Fracture of upper limb
229	S42475D	Fracture of upper limb
229	S42475G	Fracture of upper limb
229	S42475K	Fracture of upper limb
229	S42475P	Fracture of upper limb
229	S42475S	Fracture of upper limb
229	S42476A	Fracture of upper limb
229	S42476B	Fracture of upper limb
229	S42476D	Fracture of upper limb
229	S42476G	Fracture of upper limb
229	S42476K	Fracture of upper limb
229	S42476P	Fracture of upper limb
229	S42476S	Fracture of upper limb
229	S42481A	Fracture of upper limb
229	S42481D	Fracture of upper limb
229	S42481G	Fracture of upper limb
229	S42481K	Fracture of upper limb
229	S42481P	Fracture of upper limb
229	S42481S	Fracture of upper limb
229	S42482A	Fracture of upper limb
229	S42482D	Fracture of upper limb
229	S42482G	Fracture of upper limb
229	S42482K	Fracture of upper limb
229	S42482P	Fracture of upper limb
229	S42482S	Fracture of upper limb
229	S42489A	Fracture of upper limb
229	S42489D	Fracture of upper limb
229	S42489G	Fracture of upper limb
229	S42489K	Fracture of upper limb
229	S42489P	Fracture of upper limb
229	S42489S	Fracture of upper limb
229	S42491A	Fracture of upper limb
229	S42491B	Fracture of upper limb
229	S42491D	Fracture of upper limb
229	S42491G	Fracture of upper limb
229	S42491K	Fracture of upper limb
229	S42491P	Fracture of upper limb
229	S42491S	Fracture of upper limb
229	S42492A	Fracture of upper limb
229	S42492B	Fracture of upper limb

229	S42492D	Fracture of upper limb
229	S42492G	Fracture of upper limb
229	S42492K	Fracture of upper limb
229	S42492P	Fracture of upper limb
229	S42492S	Fracture of upper limb
229	S42493A	Fracture of upper limb
229	S42493B	Fracture of upper limb
229	S42493D	Fracture of upper limb
229	S42493G	Fracture of upper limb
229	S42493K	Fracture of upper limb
229	S42493P	Fracture of upper limb
229	S42493S	Fracture of upper limb
229	S42494A	Fracture of upper limb
229	S42494B	Fracture of upper limb
229	S42494D	Fracture of upper limb
229	S42494G	Fracture of upper limb
229	S42494K	Fracture of upper limb
229	S42494P	Fracture of upper limb
229	S42494S	Fracture of upper limb
229	S42495A	Fracture of upper limb
229	S42495B	Fracture of upper limb
229	S42495D	Fracture of upper limb
229	S42495G	Fracture of upper limb
229	S42495K	Fracture of upper limb
229	S42495P	Fracture of upper limb
229	S42495S	Fracture of upper limb
229	S42496A	Fracture of upper limb
229	S42496B	Fracture of upper limb
229	S42496D	Fracture of upper limb
229	S42496G	Fracture of upper limb
229	S42496K	Fracture of upper limb
229	S42496P	Fracture of upper limb
229	S42496S	Fracture of upper limb
229	S4290XA	Fracture of upper limb
229	S4290XB	Fracture of upper limb
229	S4290XD	Fracture of upper limb
229	S4290XG	Fracture of upper limb
229	S4290XK	Fracture of upper limb
229	S4290XP	Fracture of upper limb
229	S4290XS	Fracture of upper limb
229	S4291XA	Fracture of upper limb
229	S4291XB	Fracture of upper limb
229	S4291XD	Fracture of upper limb
229	S4291XG	Fracture of upper limb
229	S4291XK	Fracture of upper limb
229	S4291XP	Fracture of upper limb
229	S4291XS	Fracture of upper limb
229	S4292XA	Fracture of upper limb
229	S4292XB	Fracture of upper limb
229	S4292XD	Fracture of upper limb
229	S4292XG	Fracture of upper limb

229	S4292XK	Fracture of upper limb
229	S4292XP	Fracture of upper limb
229	S4292XS	Fracture of upper limb
229	S49001A	Fracture of upper limb
229	S49001D	Fracture of upper limb
229	S49001G	Fracture of upper limb
229	S49001K	Fracture of upper limb
229	S49001P	Fracture of upper limb
229	S49001S	Fracture of upper limb
229	S49002A	Fracture of upper limb
229	S49002D	Fracture of upper limb
229	S49002G	Fracture of upper limb
229	S49002K	Fracture of upper limb
229	S49002P	Fracture of upper limb
229	S49002S	Fracture of upper limb
229	S49009A	Fracture of upper limb
229	S49009D	Fracture of upper limb
229	S49009G	Fracture of upper limb
229	S49009K	Fracture of upper limb
229	S49009P	Fracture of upper limb
229	S49009S	Fracture of upper limb
229	S49011A	Fracture of upper limb
229	S49011D	Fracture of upper limb
229	S49011G	Fracture of upper limb
229	S49011K	Fracture of upper limb
229	S49011P	Fracture of upper limb
229	S49011S	Fracture of upper limb
229	S49012A	Fracture of upper limb
229	S49012D	Fracture of upper limb
229	S49012G	Fracture of upper limb
229	S49012K	Fracture of upper limb
229	S49012P	Fracture of upper limb
229	S49012S	Fracture of upper limb
229	S49019A	Fracture of upper limb
229	S49019D	Fracture of upper limb
229	S49019G	Fracture of upper limb
229	S49019K	Fracture of upper limb
229	S49019P	Fracture of upper limb
229	S49019S	Fracture of upper limb
229	S49021A	Fracture of upper limb
229	S49021D	Fracture of upper limb
229	S49021G	Fracture of upper limb
229	S49021K	Fracture of upper limb
229	S49021P	Fracture of upper limb
229	S49021S	Fracture of upper limb
229	S49022A	Fracture of upper limb
229	S49022D	Fracture of upper limb
229	S49022G	Fracture of upper limb
229	S49022K	Fracture of upper limb
229	S49022P	Fracture of upper limb
229	S49022S	Fracture of upper limb

229	S49029A	Fracture of upper limb
229	S49029D	Fracture of upper limb
229	S49029G	Fracture of upper limb
229	S49029K	Fracture of upper limb
229	S49029P	Fracture of upper limb
229	S49029S	Fracture of upper limb
229	S49031A	Fracture of upper limb
229	S49031D	Fracture of upper limb
229	S49031G	Fracture of upper limb
229	S49031K	Fracture of upper limb
229	S49031P	Fracture of upper limb
229	S49031S	Fracture of upper limb
229	S49032A	Fracture of upper limb
229	S49032D	Fracture of upper limb
229	S49032G	Fracture of upper limb
229	S49032K	Fracture of upper limb
229	S49032P	Fracture of upper limb
229	S49032S	Fracture of upper limb
229	S49039A	Fracture of upper limb
229	S49039D	Fracture of upper limb
229	S49039G	Fracture of upper limb
229	S49039K	Fracture of upper limb
229	S49039P	Fracture of upper limb
229	S49039S	Fracture of upper limb
229	S49041A	Fracture of upper limb
229	S49041D	Fracture of upper limb
229	S49041G	Fracture of upper limb
229	S49041K	Fracture of upper limb
229	S49041P	Fracture of upper limb
229	S49041S	Fracture of upper limb
229	S49042A	Fracture of upper limb
229	S49042D	Fracture of upper limb
229	S49042G	Fracture of upper limb
229	S49042K	Fracture of upper limb
229	S49042P	Fracture of upper limb
229	S49042S	Fracture of upper limb
229	S49049A	Fracture of upper limb
229	S49049D	Fracture of upper limb
229	S49049G	Fracture of upper limb
229	S49049K	Fracture of upper limb
229	S49049P	Fracture of upper limb
229	S49049S	Fracture of upper limb
229	S49091A	Fracture of upper limb
229	S49091D	Fracture of upper limb
229	S49091G	Fracture of upper limb
229	S49091K	Fracture of upper limb
229	S49091P	Fracture of upper limb
229	S49091S	Fracture of upper limb
229	S49092A	Fracture of upper limb
229	S49092D	Fracture of upper limb
229	S49092G	Fracture of upper limb

229	S49092K	Fracture of upper limb
229	S49092P	Fracture of upper limb
229	S49092S	Fracture of upper limb
229	S49099A	Fracture of upper limb
229	S49099D	Fracture of upper limb
229	S49099G	Fracture of upper limb
229	S49099K	Fracture of upper limb
229	S49099P	Fracture of upper limb
229	S49099S	Fracture of upper limb
229	S49101A	Fracture of upper limb
229	S49101D	Fracture of upper limb
229	S49101G	Fracture of upper limb
229	S49101K	Fracture of upper limb
229	S49101P	Fracture of upper limb
229	S49101S	Fracture of upper limb
229	S49102A	Fracture of upper limb
229	S49102D	Fracture of upper limb
229	S49102G	Fracture of upper limb
229	S49102K	Fracture of upper limb
229	S49102P	Fracture of upper limb
229	S49102S	Fracture of upper limb
229	S49109A	Fracture of upper limb
229	S49109D	Fracture of upper limb
229	S49109G	Fracture of upper limb
229	S49109K	Fracture of upper limb
229	S49109P	Fracture of upper limb
229	S49109S	Fracture of upper limb
229	S49111A	Fracture of upper limb
229	S49111D	Fracture of upper limb
229	S49111G	Fracture of upper limb
229	S49111K	Fracture of upper limb
229	S49111P	Fracture of upper limb
229	S49111S	Fracture of upper limb
229	S49112A	Fracture of upper limb
229	S49112D	Fracture of upper limb
229	S49112G	Fracture of upper limb
229	S49112K	Fracture of upper limb
229	S49112P	Fracture of upper limb
229	S49112S	Fracture of upper limb
229	S49119A	Fracture of upper limb
229	S49119D	Fracture of upper limb
229	S49119G	Fracture of upper limb
229	S49119K	Fracture of upper limb
229	S49119P	Fracture of upper limb
229	S49119S	Fracture of upper limb
229	S49121A	Fracture of upper limb
229	S49121D	Fracture of upper limb
229	S49121G	Fracture of upper limb
229	S49121K	Fracture of upper limb
229	S49121P	Fracture of upper limb
229	S49121S	Fracture of upper limb

229	S49122A	Fracture of upper limb
229	S49122D	Fracture of upper limb
229	S49122G	Fracture of upper limb
229	S49122K	Fracture of upper limb
229	S49122P	Fracture of upper limb
229	S49122S	Fracture of upper limb
229	S49129A	Fracture of upper limb
229	S49129D	Fracture of upper limb
229	S49129G	Fracture of upper limb
229	S49129K	Fracture of upper limb
229	S49129P	Fracture of upper limb
229	S49129S	Fracture of upper limb
229	S49131A	Fracture of upper limb
229	S49131D	Fracture of upper limb
229	S49131G	Fracture of upper limb
229	S49131K	Fracture of upper limb
229	S49131P	Fracture of upper limb
229	S49131S	Fracture of upper limb
229	S49132A	Fracture of upper limb
229	S49132D	Fracture of upper limb
229	S49132G	Fracture of upper limb
229	S49132K	Fracture of upper limb
229	S49132P	Fracture of upper limb
229	S49132S	Fracture of upper limb
229	S49139A	Fracture of upper limb
229	S49139D	Fracture of upper limb
229	S49139G	Fracture of upper limb
229	S49139K	Fracture of upper limb
229	S49139P	Fracture of upper limb
229	S49139S	Fracture of upper limb
229	S49141A	Fracture of upper limb
229	S49141D	Fracture of upper limb
229	S49141G	Fracture of upper limb
229	S49141K	Fracture of upper limb
229	S49141P	Fracture of upper limb
229	S49141S	Fracture of upper limb
229	S49142A	Fracture of upper limb
229	S49142D	Fracture of upper limb
229	S49142G	Fracture of upper limb
229	S49142K	Fracture of upper limb
229	S49142P	Fracture of upper limb
229	S49142S	Fracture of upper limb
229	S49149A	Fracture of upper limb
229	S49149D	Fracture of upper limb
229	S49149G	Fracture of upper limb
229	S49149K	Fracture of upper limb
229	S49149P	Fracture of upper limb
229	S49149S	Fracture of upper limb
229	S49191A	Fracture of upper limb
229	S49191D	Fracture of upper limb
229	S49191G	Fracture of upper limb

229	S49191K	Fracture of upper limb
229	S49191P	Fracture of upper limb
229	S49191S	Fracture of upper limb
229	S49192A	Fracture of upper limb
229	S49192D	Fracture of upper limb
229	S49192G	Fracture of upper limb
229	S49192K	Fracture of upper limb
229	S49192P	Fracture of upper limb
229	S49192S	Fracture of upper limb
229	S49199A	Fracture of upper limb
229	S49199D	Fracture of upper limb
229	S49199G	Fracture of upper limb
229	S49199K	Fracture of upper limb
229	S49199P	Fracture of upper limb
229	S49199S	Fracture of upper limb
229	S52001A	Fracture of upper limb
229	S52001B	Fracture of upper limb
229	S52001C	Fracture of upper limb
229	S52001D	Fracture of upper limb
229	S52001E	Fracture of upper limb
229	S52001F	Fracture of upper limb
229	S52001G	Fracture of upper limb
229	S52001H	Fracture of upper limb
229	S52001J	Fracture of upper limb
229	S52001K	Fracture of upper limb
229	S52001M	Fracture of upper limb
229	S52001N	Fracture of upper limb
229	S52001P	Fracture of upper limb
229	S52001Q	Fracture of upper limb
229	S52001R	Fracture of upper limb
229	S52001S	Fracture of upper limb
229	S52002A	Fracture of upper limb
229	S52002B	Fracture of upper limb
229	S52002C	Fracture of upper limb
229	S52002D	Fracture of upper limb
229	S52002E	Fracture of upper limb
229	S52002F	Fracture of upper limb
229	S52002G	Fracture of upper limb
229	S52002H	Fracture of upper limb
229	S52002J	Fracture of upper limb
229	S52002K	Fracture of upper limb
229	S52002M	Fracture of upper limb
229	S52002N	Fracture of upper limb
229	S52002P	Fracture of upper limb
229	S52002Q	Fracture of upper limb
229	S52002R	Fracture of upper limb
229	S52002S	Fracture of upper limb
229	S52009A	Fracture of upper limb
229	S52009B	Fracture of upper limb
229	S52009C	Fracture of upper limb
229	S52009D	Fracture of upper limb

229	S52009E	Fracture of upper limb
229	S52009F	Fracture of upper limb
229	S52009G	Fracture of upper limb
229	S52009H	Fracture of upper limb
229	S52009J	Fracture of upper limb
229	S52009K	Fracture of upper limb
229	S52009M	Fracture of upper limb
229	S52009N	Fracture of upper limb
229	S52009P	Fracture of upper limb
229	S52009Q	Fracture of upper limb
229	S52009R	Fracture of upper limb
229	S52009S	Fracture of upper limb
229	S52011A	Fracture of upper limb
229	S52011D	Fracture of upper limb
229	S52011G	Fracture of upper limb
229	S52011K	Fracture of upper limb
229	S52011P	Fracture of upper limb
229	S52011S	Fracture of upper limb
229	S52012A	Fracture of upper limb
229	S52012D	Fracture of upper limb
229	S52012G	Fracture of upper limb
229	S52012K	Fracture of upper limb
229	S52012P	Fracture of upper limb
229	S52012S	Fracture of upper limb
229	S52019A	Fracture of upper limb
229	S52019D	Fracture of upper limb
229	S52019G	Fracture of upper limb
229	S52019K	Fracture of upper limb
229	S52019P	Fracture of upper limb
229	S52019S	Fracture of upper limb
229	S52021A	Fracture of upper limb
229	S52021B	Fracture of upper limb
229	S52021C	Fracture of upper limb
229	S52021D	Fracture of upper limb
229	S52021E	Fracture of upper limb
229	S52021F	Fracture of upper limb
229	S52021G	Fracture of upper limb
229	S52021H	Fracture of upper limb
229	S52021J	Fracture of upper limb
229	S52021K	Fracture of upper limb
229	S52021M	Fracture of upper limb
229	S52021N	Fracture of upper limb
229	S52021P	Fracture of upper limb
229	S52021Q	Fracture of upper limb
229	S52021R	Fracture of upper limb
229	S52021S	Fracture of upper limb
229	S52022A	Fracture of upper limb
229	S52022B	Fracture of upper limb
229	S52022C	Fracture of upper limb
229	S52022D	Fracture of upper limb
229	S52022E	Fracture of upper limb

229	S52022F	Fracture of upper limb
229	S52022G	Fracture of upper limb
229	S52022H	Fracture of upper limb
229	S52022J	Fracture of upper limb
229	S52022K	Fracture of upper limb
229	S52022M	Fracture of upper limb
229	S52022N	Fracture of upper limb
229	S52022P	Fracture of upper limb
229	S52022Q	Fracture of upper limb
229	S52022R	Fracture of upper limb
229	S52022S	Fracture of upper limb
229	S52023A	Fracture of upper limb
229	S52023B	Fracture of upper limb
229	S52023C	Fracture of upper limb
229	S52023D	Fracture of upper limb
229	S52023E	Fracture of upper limb
229	S52023F	Fracture of upper limb
229	S52023G	Fracture of upper limb
229	S52023H	Fracture of upper limb
229	S52023J	Fracture of upper limb
229	S52023K	Fracture of upper limb
229	S52023M	Fracture of upper limb
229	S52023N	Fracture of upper limb
229	S52023P	Fracture of upper limb
229	S52023Q	Fracture of upper limb
229	S52023R	Fracture of upper limb
229	S52023S	Fracture of upper limb
229	S52024A	Fracture of upper limb
229	S52024B	Fracture of upper limb
229	S52024C	Fracture of upper limb
229	S52024D	Fracture of upper limb
229	S52024E	Fracture of upper limb
229	S52024F	Fracture of upper limb
229	S52024G	Fracture of upper limb
229	S52024H	Fracture of upper limb
229	S52024J	Fracture of upper limb
229	S52024K	Fracture of upper limb
229	S52024M	Fracture of upper limb
229	S52024N	Fracture of upper limb
229	S52024P	Fracture of upper limb
229	S52024Q	Fracture of upper limb
229	S52024R	Fracture of upper limb
229	S52024S	Fracture of upper limb
229	S52025A	Fracture of upper limb
229	S52025B	Fracture of upper limb
229	S52025C	Fracture of upper limb
229	S52025D	Fracture of upper limb
229	S52025E	Fracture of upper limb
229	S52025F	Fracture of upper limb
229	S52025G	Fracture of upper limb
229	S52025H	Fracture of upper limb

229	S52025J	Fracture of upper limb
229	S52025K	Fracture of upper limb
229	S52025M	Fracture of upper limb
229	S52025N	Fracture of upper limb
229	S52025P	Fracture of upper limb
229	S52025Q	Fracture of upper limb
229	S52025R	Fracture of upper limb
229	S52025S	Fracture of upper limb
229	S52026A	Fracture of upper limb
229	S52026B	Fracture of upper limb
229	S52026C	Fracture of upper limb
229	S52026D	Fracture of upper limb
229	S52026E	Fracture of upper limb
229	S52026F	Fracture of upper limb
229	S52026G	Fracture of upper limb
229	S52026H	Fracture of upper limb
229	S52026J	Fracture of upper limb
229	S52026K	Fracture of upper limb
229	S52026M	Fracture of upper limb
229	S52026N	Fracture of upper limb
229	S52026P	Fracture of upper limb
229	S52026Q	Fracture of upper limb
229	S52026R	Fracture of upper limb
229	S52026S	Fracture of upper limb
229	S52031A	Fracture of upper limb
229	S52031B	Fracture of upper limb
229	S52031C	Fracture of upper limb
229	S52031D	Fracture of upper limb
229	S52031E	Fracture of upper limb
229	S52031F	Fracture of upper limb
229	S52031G	Fracture of upper limb
229	S52031H	Fracture of upper limb
229	S52031J	Fracture of upper limb
229	S52031K	Fracture of upper limb
229	S52031M	Fracture of upper limb
229	S52031N	Fracture of upper limb
229	S52031P	Fracture of upper limb
229	S52031Q	Fracture of upper limb
229	S52031R	Fracture of upper limb
229	S52031S	Fracture of upper limb
229	S52032A	Fracture of upper limb
229	S52032B	Fracture of upper limb
229	S52032C	Fracture of upper limb
229	S52032D	Fracture of upper limb
229	S52032E	Fracture of upper limb
229	S52032F	Fracture of upper limb
229	S52032G	Fracture of upper limb
229	S52032H	Fracture of upper limb
229	S52032J	Fracture of upper limb
229	S52032K	Fracture of upper limb
229	S52032M	Fracture of upper limb

229	S52032N	Fracture of upper limb
229	S52032P	Fracture of upper limb
229	S52032Q	Fracture of upper limb
229	S52032R	Fracture of upper limb
229	S52032S	Fracture of upper limb
229	S52033A	Fracture of upper limb
229	S52033B	Fracture of upper limb
229	S52033C	Fracture of upper limb
229	S52033D	Fracture of upper limb
229	S52033E	Fracture of upper limb
229	S52033F	Fracture of upper limb
229	S52033G	Fracture of upper limb
229	S52033H	Fracture of upper limb
229	S52033J	Fracture of upper limb
229	S52033K	Fracture of upper limb
229	S52033M	Fracture of upper limb
229	S52033N	Fracture of upper limb
229	S52033P	Fracture of upper limb
229	S52033Q	Fracture of upper limb
229	S52033R	Fracture of upper limb
229	S52033S	Fracture of upper limb
229	S52034A	Fracture of upper limb
229	S52034B	Fracture of upper limb
229	S52034C	Fracture of upper limb
229	S52034D	Fracture of upper limb
229	S52034E	Fracture of upper limb
229	S52034F	Fracture of upper limb
229	S52034G	Fracture of upper limb
229	S52034H	Fracture of upper limb
229	S52034J	Fracture of upper limb
229	S52034K	Fracture of upper limb
229	S52034M	Fracture of upper limb
229	S52034N	Fracture of upper limb
229	S52034P	Fracture of upper limb
229	S52034Q	Fracture of upper limb
229	S52034R	Fracture of upper limb
229	S52034S	Fracture of upper limb
229	S52035A	Fracture of upper limb
229	S52035B	Fracture of upper limb
229	S52035C	Fracture of upper limb
229	S52035D	Fracture of upper limb
229	S52035E	Fracture of upper limb
229	S52035F	Fracture of upper limb
229	S52035G	Fracture of upper limb
229	S52035H	Fracture of upper limb
229	S52035J	Fracture of upper limb
229	S52035K	Fracture of upper limb
229	S52035M	Fracture of upper limb
229	S52035N	Fracture of upper limb
229	S52035P	Fracture of upper limb
229	S52035Q	Fracture of upper limb

229	S52035R	Fracture of upper limb
229	S52035S	Fracture of upper limb
229	S52036A	Fracture of upper limb
229	S52036B	Fracture of upper limb
229	S52036C	Fracture of upper limb
229	S52036D	Fracture of upper limb
229	S52036E	Fracture of upper limb
229	S52036F	Fracture of upper limb
229	S52036G	Fracture of upper limb
229	S52036H	Fracture of upper limb
229	S52036J	Fracture of upper limb
229	S52036K	Fracture of upper limb
229	S52036M	Fracture of upper limb
229	S52036N	Fracture of upper limb
229	S52036P	Fracture of upper limb
229	S52036Q	Fracture of upper limb
229	S52036R	Fracture of upper limb
229	S52036S	Fracture of upper limb
229	S52041A	Fracture of upper limb
229	S52041B	Fracture of upper limb
229	S52041C	Fracture of upper limb
229	S52041D	Fracture of upper limb
229	S52041E	Fracture of upper limb
229	S52041F	Fracture of upper limb
229	S52041G	Fracture of upper limb
229	S52041H	Fracture of upper limb
229	S52041J	Fracture of upper limb
229	S52041K	Fracture of upper limb
229	S52041M	Fracture of upper limb
229	S52041N	Fracture of upper limb
229	S52041P	Fracture of upper limb
229	S52041Q	Fracture of upper limb
229	S52041R	Fracture of upper limb
229	S52041S	Fracture of upper limb
229	S52042A	Fracture of upper limb
229	S52042B	Fracture of upper limb
229	S52042C	Fracture of upper limb
229	S52042D	Fracture of upper limb
229	S52042E	Fracture of upper limb
229	S52042F	Fracture of upper limb
229	S52042G	Fracture of upper limb
229	S52042H	Fracture of upper limb
229	S52042J	Fracture of upper limb
229	S52042K	Fracture of upper limb
229	S52042M	Fracture of upper limb
229	S52042N	Fracture of upper limb
229	S52042P	Fracture of upper limb
229	S52042Q	Fracture of upper limb
229	S52042R	Fracture of upper limb
229	S52042S	Fracture of upper limb
229	S52043A	Fracture of upper limb

229	S52043B	Fracture of upper limb
229	S52043C	Fracture of upper limb
229	S52043D	Fracture of upper limb
229	S52043E	Fracture of upper limb
229	S52043F	Fracture of upper limb
229	S52043G	Fracture of upper limb
229	S52043H	Fracture of upper limb
229	S52043J	Fracture of upper limb
229	S52043K	Fracture of upper limb
229	S52043M	Fracture of upper limb
229	S52043N	Fracture of upper limb
229	S52043P	Fracture of upper limb
229	S52043Q	Fracture of upper limb
229	S52043R	Fracture of upper limb
229	S52043S	Fracture of upper limb
229	S52044A	Fracture of upper limb
229	S52044B	Fracture of upper limb
229	S52044C	Fracture of upper limb
229	S52044D	Fracture of upper limb
229	S52044E	Fracture of upper limb
229	S52044F	Fracture of upper limb
229	S52044G	Fracture of upper limb
229	S52044H	Fracture of upper limb
229	S52044J	Fracture of upper limb
229	S52044K	Fracture of upper limb
229	S52044M	Fracture of upper limb
229	S52044N	Fracture of upper limb
229	S52044P	Fracture of upper limb
229	S52044Q	Fracture of upper limb
229	S52044R	Fracture of upper limb
229	S52044S	Fracture of upper limb
229	S52045A	Fracture of upper limb
229	S52045B	Fracture of upper limb
229	S52045C	Fracture of upper limb
229	S52045D	Fracture of upper limb
229	S52045E	Fracture of upper limb
229	S52045F	Fracture of upper limb
229	S52045G	Fracture of upper limb
229	S52045H	Fracture of upper limb
229	S52045J	Fracture of upper limb
229	S52045K	Fracture of upper limb
229	S52045M	Fracture of upper limb
229	S52045N	Fracture of upper limb
229	S52045P	Fracture of upper limb
229	S52045Q	Fracture of upper limb
229	S52045R	Fracture of upper limb
229	S52045S	Fracture of upper limb
229	S52046A	Fracture of upper limb
229	S52046B	Fracture of upper limb
229	S52046C	Fracture of upper limb
229	S52046D	Fracture of upper limb

229	S52046E	Fracture of upper limb
229	S52046F	Fracture of upper limb
229	S52046G	Fracture of upper limb
229	S52046H	Fracture of upper limb
229	S52046J	Fracture of upper limb
229	S52046K	Fracture of upper limb
229	S52046M	Fracture of upper limb
229	S52046N	Fracture of upper limb
229	S52046P	Fracture of upper limb
229	S52046Q	Fracture of upper limb
229	S52046R	Fracture of upper limb
229	S52046S	Fracture of upper limb
229	S52091A	Fracture of upper limb
229	S52091B	Fracture of upper limb
229	S52091C	Fracture of upper limb
229	S52091D	Fracture of upper limb
229	S52091E	Fracture of upper limb
229	S52091F	Fracture of upper limb
229	S52091G	Fracture of upper limb
229	S52091H	Fracture of upper limb
229	S52091J	Fracture of upper limb
229	S52091K	Fracture of upper limb
229	S52091M	Fracture of upper limb
229	S52091N	Fracture of upper limb
229	S52091P	Fracture of upper limb
229	S52091Q	Fracture of upper limb
229	S52091R	Fracture of upper limb
229	S52091S	Fracture of upper limb
229	S52092A	Fracture of upper limb
229	S52092B	Fracture of upper limb
229	S52092C	Fracture of upper limb
229	S52092D	Fracture of upper limb
229	S52092E	Fracture of upper limb
229	S52092F	Fracture of upper limb
229	S52092G	Fracture of upper limb
229	S52092H	Fracture of upper limb
229	S52092J	Fracture of upper limb
229	S52092K	Fracture of upper limb
229	S52092M	Fracture of upper limb
229	S52092N	Fracture of upper limb
229	S52092P	Fracture of upper limb
229	S52092Q	Fracture of upper limb
229	S52092R	Fracture of upper limb
229	S52092S	Fracture of upper limb
229	S52099A	Fracture of upper limb
229	S52099B	Fracture of upper limb
229	S52099C	Fracture of upper limb
229	S52099D	Fracture of upper limb
229	S52099E	Fracture of upper limb
229	S52099F	Fracture of upper limb
229	S52099G	Fracture of upper limb

229	S52099H	Fracture of upper limb
229	S52099J	Fracture of upper limb
229	S52099K	Fracture of upper limb
229	S52099M	Fracture of upper limb
229	S52099N	Fracture of upper limb
229	S52099P	Fracture of upper limb
229	S52099Q	Fracture of upper limb
229	S52099R	Fracture of upper limb
229	S52099S	Fracture of upper limb
229	S52101A	Fracture of upper limb
229	S52101B	Fracture of upper limb
229	S52101C	Fracture of upper limb
229	S52101D	Fracture of upper limb
229	S52101E	Fracture of upper limb
229	S52101F	Fracture of upper limb
229	S52101G	Fracture of upper limb
229	S52101H	Fracture of upper limb
229	S52101J	Fracture of upper limb
229	S52101K	Fracture of upper limb
229	S52101M	Fracture of upper limb
229	S52101N	Fracture of upper limb
229	S52101P	Fracture of upper limb
229	S52101Q	Fracture of upper limb
229	S52101R	Fracture of upper limb
229	S52101S	Fracture of upper limb
229	S52102A	Fracture of upper limb
229	S52102B	Fracture of upper limb
229	S52102C	Fracture of upper limb
229	S52102D	Fracture of upper limb
229	S52102E	Fracture of upper limb
229	S52102F	Fracture of upper limb
229	S52102G	Fracture of upper limb
229	S52102H	Fracture of upper limb
229	S52102J	Fracture of upper limb
229	S52102K	Fracture of upper limb
229	S52102M	Fracture of upper limb
229	S52102N	Fracture of upper limb
229	S52102P	Fracture of upper limb
229	S52102Q	Fracture of upper limb
229	S52102R	Fracture of upper limb
229	S52102S	Fracture of upper limb
229	S52109A	Fracture of upper limb
229	S52109B	Fracture of upper limb
229	S52109C	Fracture of upper limb
229	S52109D	Fracture of upper limb
229	S52109E	Fracture of upper limb
229	S52109F	Fracture of upper limb
229	S52109G	Fracture of upper limb
229	S52109H	Fracture of upper limb
229	S52109J	Fracture of upper limb
229	S52109K	Fracture of upper limb

229	S52109M	Fracture of upper limb
229	S52109N	Fracture of upper limb
229	S52109P	Fracture of upper limb
229	S52109Q	Fracture of upper limb
229	S52109R	Fracture of upper limb
229	S52109S	Fracture of upper limb
229	S52111A	Fracture of upper limb
229	S52111D	Fracture of upper limb
229	S52111G	Fracture of upper limb
229	S52111K	Fracture of upper limb
229	S52111P	Fracture of upper limb
229	S52111S	Fracture of upper limb
229	S52112A	Fracture of upper limb
229	S52112D	Fracture of upper limb
229	S52112G	Fracture of upper limb
229	S52112K	Fracture of upper limb
229	S52112P	Fracture of upper limb
229	S52112S	Fracture of upper limb
229	S52119A	Fracture of upper limb
229	S52119D	Fracture of upper limb
229	S52119G	Fracture of upper limb
229	S52119K	Fracture of upper limb
229	S52119P	Fracture of upper limb
229	S52119S	Fracture of upper limb
229	S52121A	Fracture of upper limb
229	S52121B	Fracture of upper limb
229	S52121C	Fracture of upper limb
229	S52121D	Fracture of upper limb
229	S52121E	Fracture of upper limb
229	S52121F	Fracture of upper limb
229	S52121G	Fracture of upper limb
229	S52121H	Fracture of upper limb
229	S52121J	Fracture of upper limb
229	S52121K	Fracture of upper limb
229	S52121M	Fracture of upper limb
229	S52121N	Fracture of upper limb
229	S52121P	Fracture of upper limb
229	S52121Q	Fracture of upper limb
229	S52121R	Fracture of upper limb
229	S52121S	Fracture of upper limb
229	S52122A	Fracture of upper limb
229	S52122B	Fracture of upper limb
229	S52122C	Fracture of upper limb
229	S52122D	Fracture of upper limb
229	S52122E	Fracture of upper limb
229	S52122F	Fracture of upper limb
229	S52122G	Fracture of upper limb
229	S52122H	Fracture of upper limb
229	S52122J	Fracture of upper limb
229	S52122K	Fracture of upper limb
229	S52122M	Fracture of upper limb

229	S52122N	Fracture of upper limb
229	S52122P	Fracture of upper limb
229	S52122Q	Fracture of upper limb
229	S52122R	Fracture of upper limb
229	S52122S	Fracture of upper limb
229	S52123A	Fracture of upper limb
229	S52123B	Fracture of upper limb
229	S52123C	Fracture of upper limb
229	S52123D	Fracture of upper limb
229	S52123E	Fracture of upper limb
229	S52123F	Fracture of upper limb
229	S52123G	Fracture of upper limb
229	S52123H	Fracture of upper limb
229	S52123J	Fracture of upper limb
229	S52123K	Fracture of upper limb
229	S52123M	Fracture of upper limb
229	S52123N	Fracture of upper limb
229	S52123P	Fracture of upper limb
229	S52123Q	Fracture of upper limb
229	S52123R	Fracture of upper limb
229	S52123S	Fracture of upper limb
229	S52124A	Fracture of upper limb
229	S52124B	Fracture of upper limb
229	S52124C	Fracture of upper limb
229	S52124D	Fracture of upper limb
229	S52124E	Fracture of upper limb
229	S52124F	Fracture of upper limb
229	S52124G	Fracture of upper limb
229	S52124H	Fracture of upper limb
229	S52124J	Fracture of upper limb
229	S52124K	Fracture of upper limb
229	S52124M	Fracture of upper limb
229	S52124N	Fracture of upper limb
229	S52124P	Fracture of upper limb
229	S52124Q	Fracture of upper limb
229	S52124R	Fracture of upper limb
229	S52124S	Fracture of upper limb
229	S52125A	Fracture of upper limb
229	S52125B	Fracture of upper limb
229	S52125C	Fracture of upper limb
229	S52125D	Fracture of upper limb
229	S52125E	Fracture of upper limb
229	S52125F	Fracture of upper limb
229	S52125G	Fracture of upper limb
229	S52125H	Fracture of upper limb
229	S52125J	Fracture of upper limb
229	S52125K	Fracture of upper limb
229	S52125M	Fracture of upper limb
229	S52125N	Fracture of upper limb
229	S52125P	Fracture of upper limb
229	S52125Q	Fracture of upper limb

229	S52125R	Fracture of upper limb
229	S52125S	Fracture of upper limb
229	S52126A	Fracture of upper limb
229	S52126B	Fracture of upper limb
229	S52126C	Fracture of upper limb
229	S52126D	Fracture of upper limb
229	S52126E	Fracture of upper limb
229	S52126F	Fracture of upper limb
229	S52126G	Fracture of upper limb
229	S52126H	Fracture of upper limb
229	S52126J	Fracture of upper limb
229	S52126K	Fracture of upper limb
229	S52126M	Fracture of upper limb
229	S52126N	Fracture of upper limb
229	S52126P	Fracture of upper limb
229	S52126Q	Fracture of upper limb
229	S52126R	Fracture of upper limb
229	S52126S	Fracture of upper limb
229	S52131A	Fracture of upper limb
229	S52131B	Fracture of upper limb
229	S52131C	Fracture of upper limb
229	S52131D	Fracture of upper limb
229	S52131E	Fracture of upper limb
229	S52131F	Fracture of upper limb
229	S52131G	Fracture of upper limb
229	S52131H	Fracture of upper limb
229	S52131J	Fracture of upper limb
229	S52131K	Fracture of upper limb
229	S52131M	Fracture of upper limb
229	S52131N	Fracture of upper limb
229	S52131P	Fracture of upper limb
229	S52131Q	Fracture of upper limb
229	S52131R	Fracture of upper limb
229	S52131S	Fracture of upper limb
229	S52132A	Fracture of upper limb
229	S52132B	Fracture of upper limb
229	S52132C	Fracture of upper limb
229	S52132D	Fracture of upper limb
229	S52132E	Fracture of upper limb
229	S52132F	Fracture of upper limb
229	S52132G	Fracture of upper limb
229	S52132H	Fracture of upper limb
229	S52132J	Fracture of upper limb
229	S52132K	Fracture of upper limb
229	S52132M	Fracture of upper limb
229	S52132N	Fracture of upper limb
229	S52132P	Fracture of upper limb
229	S52132Q	Fracture of upper limb
229	S52132R	Fracture of upper limb
229	S52132S	Fracture of upper limb
229	S52133A	Fracture of upper limb

229	S52133B	Fracture of upper limb
229	S52133C	Fracture of upper limb
229	S52133D	Fracture of upper limb
229	S52133E	Fracture of upper limb
229	S52133F	Fracture of upper limb
229	S52133G	Fracture of upper limb
229	S52133H	Fracture of upper limb
229	S52133J	Fracture of upper limb
229	S52133K	Fracture of upper limb
229	S52133M	Fracture of upper limb
229	S52133N	Fracture of upper limb
229	S52133P	Fracture of upper limb
229	S52133Q	Fracture of upper limb
229	S52133R	Fracture of upper limb
229	S52133S	Fracture of upper limb
229	S52134A	Fracture of upper limb
229	S52134B	Fracture of upper limb
229	S52134C	Fracture of upper limb
229	S52134D	Fracture of upper limb
229	S52134E	Fracture of upper limb
229	S52134F	Fracture of upper limb
229	S52134G	Fracture of upper limb
229	S52134H	Fracture of upper limb
229	S52134J	Fracture of upper limb
229	S52134K	Fracture of upper limb
229	S52134M	Fracture of upper limb
229	S52134N	Fracture of upper limb
229	S52134P	Fracture of upper limb
229	S52134Q	Fracture of upper limb
229	S52134R	Fracture of upper limb
229	S52134S	Fracture of upper limb
229	S52135A	Fracture of upper limb
229	S52135B	Fracture of upper limb
229	S52135C	Fracture of upper limb
229	S52135D	Fracture of upper limb
229	S52135E	Fracture of upper limb
229	S52135F	Fracture of upper limb
229	S52135G	Fracture of upper limb
229	S52135H	Fracture of upper limb
229	S52135J	Fracture of upper limb
229	S52135K	Fracture of upper limb
229	S52135M	Fracture of upper limb
229	S52135N	Fracture of upper limb
229	S52135P	Fracture of upper limb
229	S52135Q	Fracture of upper limb
229	S52135R	Fracture of upper limb
229	S52135S	Fracture of upper limb
229	S52136A	Fracture of upper limb
229	S52136B	Fracture of upper limb
229	S52136C	Fracture of upper limb
229	S52136D	Fracture of upper limb

229	S52136E	Fracture of upper limb
229	S52136F	Fracture of upper limb
229	S52136G	Fracture of upper limb
229	S52136H	Fracture of upper limb
229	S52136J	Fracture of upper limb
229	S52136K	Fracture of upper limb
229	S52136M	Fracture of upper limb
229	S52136N	Fracture of upper limb
229	S52136P	Fracture of upper limb
229	S52136Q	Fracture of upper limb
229	S52136R	Fracture of upper limb
229	S52136S	Fracture of upper limb
229	S52181A	Fracture of upper limb
229	S52181B	Fracture of upper limb
229	S52181C	Fracture of upper limb
229	S52181D	Fracture of upper limb
229	S52181E	Fracture of upper limb
229	S52181F	Fracture of upper limb
229	S52181G	Fracture of upper limb
229	S52181H	Fracture of upper limb
229	S52181J	Fracture of upper limb
229	S52181K	Fracture of upper limb
229	S52181M	Fracture of upper limb
229	S52181N	Fracture of upper limb
229	S52181P	Fracture of upper limb
229	S52181Q	Fracture of upper limb
229	S52181R	Fracture of upper limb
229	S52181S	Fracture of upper limb
229	S52182A	Fracture of upper limb
229	S52182B	Fracture of upper limb
229	S52182C	Fracture of upper limb
229	S52182D	Fracture of upper limb
229	S52182E	Fracture of upper limb
229	S52182F	Fracture of upper limb
229	S52182G	Fracture of upper limb
229	S52182H	Fracture of upper limb
229	S52182J	Fracture of upper limb
229	S52182K	Fracture of upper limb
229	S52182M	Fracture of upper limb
229	S52182N	Fracture of upper limb
229	S52182P	Fracture of upper limb
229	S52182Q	Fracture of upper limb
229	S52182R	Fracture of upper limb
229	S52182S	Fracture of upper limb
229	S52189A	Fracture of upper limb
229	S52189B	Fracture of upper limb
229	S52189C	Fracture of upper limb
229	S52189D	Fracture of upper limb
229	S52189E	Fracture of upper limb
229	S52189F	Fracture of upper limb
229	S52189G	Fracture of upper limb

229	S52189H	Fracture of upper limb
229	S52189J	Fracture of upper limb
229	S52189K	Fracture of upper limb
229	S52189M	Fracture of upper limb
229	S52189N	Fracture of upper limb
229	S52189P	Fracture of upper limb
229	S52189Q	Fracture of upper limb
229	S52189R	Fracture of upper limb
229	S52189S	Fracture of upper limb
229	S52201A	Fracture of upper limb
229	S52201B	Fracture of upper limb
229	S52201C	Fracture of upper limb
229	S52201D	Fracture of upper limb
229	S52201E	Fracture of upper limb
229	S52201F	Fracture of upper limb
229	S52201G	Fracture of upper limb
229	S52201H	Fracture of upper limb
229	S52201J	Fracture of upper limb
229	S52201K	Fracture of upper limb
229	S52201M	Fracture of upper limb
229	S52201N	Fracture of upper limb
229	S52201P	Fracture of upper limb
229	S52201Q	Fracture of upper limb
229	S52201R	Fracture of upper limb
229	S52201S	Fracture of upper limb
229	S52202A	Fracture of upper limb
229	S52202B	Fracture of upper limb
229	S52202C	Fracture of upper limb
229	S52202D	Fracture of upper limb
229	S52202E	Fracture of upper limb
229	S52202F	Fracture of upper limb
229	S52202G	Fracture of upper limb
229	S52202H	Fracture of upper limb
229	S52202J	Fracture of upper limb
229	S52202K	Fracture of upper limb
229	S52202M	Fracture of upper limb
229	S52202N	Fracture of upper limb
229	S52202P	Fracture of upper limb
229	S52202Q	Fracture of upper limb
229	S52202R	Fracture of upper limb
229	S52202S	Fracture of upper limb
229	S52209A	Fracture of upper limb
229	S52209B	Fracture of upper limb
229	S52209C	Fracture of upper limb
229	S52209D	Fracture of upper limb
229	S52209E	Fracture of upper limb
229	S52209F	Fracture of upper limb
229	S52209G	Fracture of upper limb
229	S52209H	Fracture of upper limb
229	S52209J	Fracture of upper limb
229	S52209K	Fracture of upper limb

229	S52209M	Fracture of upper limb
229	S52209N	Fracture of upper limb
229	S52209P	Fracture of upper limb
229	S52209Q	Fracture of upper limb
229	S52209R	Fracture of upper limb
229	S52209S	Fracture of upper limb
229	S52211A	Fracture of upper limb
229	S52211D	Fracture of upper limb
229	S52211G	Fracture of upper limb
229	S52211K	Fracture of upper limb
229	S52211P	Fracture of upper limb
229	S52211S	Fracture of upper limb
229	S52212A	Fracture of upper limb
229	S52212D	Fracture of upper limb
229	S52212G	Fracture of upper limb
229	S52212K	Fracture of upper limb
229	S52212P	Fracture of upper limb
229	S52212S	Fracture of upper limb
229	S52219A	Fracture of upper limb
229	S52219D	Fracture of upper limb
229	S52219G	Fracture of upper limb
229	S52219K	Fracture of upper limb
229	S52219P	Fracture of upper limb
229	S52219S	Fracture of upper limb
229	S52221A	Fracture of upper limb
229	S52221B	Fracture of upper limb
229	S52221C	Fracture of upper limb
229	S52221D	Fracture of upper limb
229	S52221E	Fracture of upper limb
229	S52221F	Fracture of upper limb
229	S52221G	Fracture of upper limb
229	S52221H	Fracture of upper limb
229	S52221J	Fracture of upper limb
229	S52221K	Fracture of upper limb
229	S52221M	Fracture of upper limb
229	S52221N	Fracture of upper limb
229	S52221P	Fracture of upper limb
229	S52221Q	Fracture of upper limb
229	S52221R	Fracture of upper limb
229	S52221S	Fracture of upper limb
229	S52222A	Fracture of upper limb
229	S52222B	Fracture of upper limb
229	S52222C	Fracture of upper limb
229	S52222D	Fracture of upper limb
229	S52222E	Fracture of upper limb
229	S52222F	Fracture of upper limb
229	S52222G	Fracture of upper limb
229	S52222H	Fracture of upper limb
229	S52222J	Fracture of upper limb
229	S52222K	Fracture of upper limb
229	S52222M	Fracture of upper limb

229	S52222N	Fracture of upper limb
229	S52222P	Fracture of upper limb
229	S52222Q	Fracture of upper limb
229	S52222R	Fracture of upper limb
229	S52222S	Fracture of upper limb
229	S52223A	Fracture of upper limb
229	S52223B	Fracture of upper limb
229	S52223C	Fracture of upper limb
229	S52223D	Fracture of upper limb
229	S52223E	Fracture of upper limb
229	S52223F	Fracture of upper limb
229	S52223G	Fracture of upper limb
229	S52223H	Fracture of upper limb
229	S52223J	Fracture of upper limb
229	S52223K	Fracture of upper limb
229	S52223M	Fracture of upper limb
229	S52223N	Fracture of upper limb
229	S52223P	Fracture of upper limb
229	S52223Q	Fracture of upper limb
229	S52223R	Fracture of upper limb
229	S52223S	Fracture of upper limb
229	S52224A	Fracture of upper limb
229	S52224B	Fracture of upper limb
229	S52224C	Fracture of upper limb
229	S52224D	Fracture of upper limb
229	S52224E	Fracture of upper limb
229	S52224F	Fracture of upper limb
229	S52224G	Fracture of upper limb
229	S52224H	Fracture of upper limb
229	S52224J	Fracture of upper limb
229	S52224K	Fracture of upper limb
229	S52224M	Fracture of upper limb
229	S52224N	Fracture of upper limb
229	S52224P	Fracture of upper limb
229	S52224Q	Fracture of upper limb
229	S52224R	Fracture of upper limb
229	S52224S	Fracture of upper limb
229	S52225A	Fracture of upper limb
229	S52225B	Fracture of upper limb
229	S52225C	Fracture of upper limb
229	S52225D	Fracture of upper limb
229	S52225E	Fracture of upper limb
229	S52225F	Fracture of upper limb
229	S52225G	Fracture of upper limb
229	S52225H	Fracture of upper limb
229	S52225J	Fracture of upper limb
229	S52225K	Fracture of upper limb
229	S52225M	Fracture of upper limb
229	S52225N	Fracture of upper limb
229	S52225P	Fracture of upper limb
229	S52225Q	Fracture of upper limb

229	S52225R	Fracture of upper limb
229	S52225S	Fracture of upper limb
229	S52226A	Fracture of upper limb
229	S52226B	Fracture of upper limb
229	S52226C	Fracture of upper limb
229	S52226D	Fracture of upper limb
229	S52226E	Fracture of upper limb
229	S52226F	Fracture of upper limb
229	S52226G	Fracture of upper limb
229	S52226H	Fracture of upper limb
229	S52226J	Fracture of upper limb
229	S52226K	Fracture of upper limb
229	S52226M	Fracture of upper limb
229	S52226N	Fracture of upper limb
229	S52226P	Fracture of upper limb
229	S52226Q	Fracture of upper limb
229	S52226R	Fracture of upper limb
229	S52226S	Fracture of upper limb
229	S52231A	Fracture of upper limb
229	S52231B	Fracture of upper limb
229	S52231C	Fracture of upper limb
229	S52231D	Fracture of upper limb
229	S52231E	Fracture of upper limb
229	S52231F	Fracture of upper limb
229	S52231G	Fracture of upper limb
229	S52231H	Fracture of upper limb
229	S52231J	Fracture of upper limb
229	S52231K	Fracture of upper limb
229	S52231M	Fracture of upper limb
229	S52231N	Fracture of upper limb
229	S52231P	Fracture of upper limb
229	S52231Q	Fracture of upper limb
229	S52231R	Fracture of upper limb
229	S52231S	Fracture of upper limb
229	S52232A	Fracture of upper limb
229	S52232B	Fracture of upper limb
229	S52232C	Fracture of upper limb
229	S52232D	Fracture of upper limb
229	S52232E	Fracture of upper limb
229	S52232F	Fracture of upper limb
229	S52232G	Fracture of upper limb
229	S52232H	Fracture of upper limb
229	S52232J	Fracture of upper limb
229	S52232K	Fracture of upper limb
229	S52232M	Fracture of upper limb
229	S52232N	Fracture of upper limb
229	S52232P	Fracture of upper limb
229	S52232Q	Fracture of upper limb
229	S52232R	Fracture of upper limb
229	S52232S	Fracture of upper limb
229	S52233A	Fracture of upper limb

229	S52233B	Fracture of upper limb
229	S52233C	Fracture of upper limb
229	S52233D	Fracture of upper limb
229	S52233E	Fracture of upper limb
229	S52233F	Fracture of upper limb
229	S52233G	Fracture of upper limb
229	S52233H	Fracture of upper limb
229	S52233J	Fracture of upper limb
229	S52233K	Fracture of upper limb
229	S52233M	Fracture of upper limb
229	S52233N	Fracture of upper limb
229	S52233P	Fracture of upper limb
229	S52233Q	Fracture of upper limb
229	S52233R	Fracture of upper limb
229	S52233S	Fracture of upper limb
229	S52234A	Fracture of upper limb
229	S52234B	Fracture of upper limb
229	S52234C	Fracture of upper limb
229	S52234D	Fracture of upper limb
229	S52234E	Fracture of upper limb
229	S52234F	Fracture of upper limb
229	S52234G	Fracture of upper limb
229	S52234H	Fracture of upper limb
229	S52234J	Fracture of upper limb
229	S52234K	Fracture of upper limb
229	S52234M	Fracture of upper limb
229	S52234N	Fracture of upper limb
229	S52234P	Fracture of upper limb
229	S52234Q	Fracture of upper limb
229	S52234R	Fracture of upper limb
229	S52234S	Fracture of upper limb
229	S52235A	Fracture of upper limb
229	S52235B	Fracture of upper limb
229	S52235C	Fracture of upper limb
229	S52235D	Fracture of upper limb
229	S52235E	Fracture of upper limb
229	S52235F	Fracture of upper limb
229	S52235G	Fracture of upper limb
229	S52235H	Fracture of upper limb
229	S52235J	Fracture of upper limb
229	S52235K	Fracture of upper limb
229	S52235M	Fracture of upper limb
229	S52235N	Fracture of upper limb
229	S52235P	Fracture of upper limb
229	S52235Q	Fracture of upper limb
229	S52235R	Fracture of upper limb
229	S52235S	Fracture of upper limb
229	S52236A	Fracture of upper limb
229	S52236B	Fracture of upper limb
229	S52236C	Fracture of upper limb
229	S52236D	Fracture of upper limb

229	S52236E	Fracture of upper limb
229	S52236F	Fracture of upper limb
229	S52236G	Fracture of upper limb
229	S52236H	Fracture of upper limb
229	S52236J	Fracture of upper limb
229	S52236K	Fracture of upper limb
229	S52236M	Fracture of upper limb
229	S52236N	Fracture of upper limb
229	S52236P	Fracture of upper limb
229	S52236Q	Fracture of upper limb
229	S52236R	Fracture of upper limb
229	S52236S	Fracture of upper limb
229	S52241A	Fracture of upper limb
229	S52241B	Fracture of upper limb
229	S52241C	Fracture of upper limb
229	S52241D	Fracture of upper limb
229	S52241E	Fracture of upper limb
229	S52241F	Fracture of upper limb
229	S52241G	Fracture of upper limb
229	S52241H	Fracture of upper limb
229	S52241J	Fracture of upper limb
229	S52241K	Fracture of upper limb
229	S52241M	Fracture of upper limb
229	S52241N	Fracture of upper limb
229	S52241P	Fracture of upper limb
229	S52241Q	Fracture of upper limb
229	S52241R	Fracture of upper limb
229	S52241S	Fracture of upper limb
229	S52242A	Fracture of upper limb
229	S52242B	Fracture of upper limb
229	S52242C	Fracture of upper limb
229	S52242D	Fracture of upper limb
229	S52242E	Fracture of upper limb
229	S52242F	Fracture of upper limb
229	S52242G	Fracture of upper limb
229	S52242H	Fracture of upper limb
229	S52242J	Fracture of upper limb
229	S52242K	Fracture of upper limb
229	S52242M	Fracture of upper limb
229	S52242N	Fracture of upper limb
229	S52242P	Fracture of upper limb
229	S52242Q	Fracture of upper limb
229	S52242R	Fracture of upper limb
229	S52242S	Fracture of upper limb
229	S52243A	Fracture of upper limb
229	S52243B	Fracture of upper limb
229	S52243C	Fracture of upper limb
229	S52243D	Fracture of upper limb
229	S52243E	Fracture of upper limb
229	S52243F	Fracture of upper limb
229	S52243G	Fracture of upper limb

229	S52243H	Fracture of upper limb
229	S52243J	Fracture of upper limb
229	S52243K	Fracture of upper limb
229	S52243M	Fracture of upper limb
229	S52243N	Fracture of upper limb
229	S52243P	Fracture of upper limb
229	S52243Q	Fracture of upper limb
229	S52243R	Fracture of upper limb
229	S52243S	Fracture of upper limb
229	S52244A	Fracture of upper limb
229	S52244B	Fracture of upper limb
229	S52244C	Fracture of upper limb
229	S52244D	Fracture of upper limb
229	S52244E	Fracture of upper limb
229	S52244F	Fracture of upper limb
229	S52244G	Fracture of upper limb
229	S52244H	Fracture of upper limb
229	S52244J	Fracture of upper limb
229	S52244K	Fracture of upper limb
229	S52244M	Fracture of upper limb
229	S52244N	Fracture of upper limb
229	S52244P	Fracture of upper limb
229	S52244Q	Fracture of upper limb
229	S52244R	Fracture of upper limb
229	S52244S	Fracture of upper limb
229	S52245A	Fracture of upper limb
229	S52245B	Fracture of upper limb
229	S52245C	Fracture of upper limb
229	S52245D	Fracture of upper limb
229	S52245E	Fracture of upper limb
229	S52245F	Fracture of upper limb
229	S52245G	Fracture of upper limb
229	S52245H	Fracture of upper limb
229	S52245J	Fracture of upper limb
229	S52245K	Fracture of upper limb
229	S52245M	Fracture of upper limb
229	S52245N	Fracture of upper limb
229	S52245P	Fracture of upper limb
229	S52245Q	Fracture of upper limb
229	S52245R	Fracture of upper limb
229	S52245S	Fracture of upper limb
229	S52246A	Fracture of upper limb
229	S52246B	Fracture of upper limb
229	S52246C	Fracture of upper limb
229	S52246D	Fracture of upper limb
229	S52246E	Fracture of upper limb
229	S52246F	Fracture of upper limb
229	S52246G	Fracture of upper limb
229	S52246H	Fracture of upper limb
229	S52246J	Fracture of upper limb
229	S52246K	Fracture of upper limb

229	S52246M	Fracture of upper limb
229	S52246N	Fracture of upper limb
229	S52246P	Fracture of upper limb
229	S52246Q	Fracture of upper limb
229	S52246R	Fracture of upper limb
229	S52246S	Fracture of upper limb
229	S52251A	Fracture of upper limb
229	S52251B	Fracture of upper limb
229	S52251C	Fracture of upper limb
229	S52251D	Fracture of upper limb
229	S52251E	Fracture of upper limb
229	S52251F	Fracture of upper limb
229	S52251G	Fracture of upper limb
229	S52251H	Fracture of upper limb
229	S52251J	Fracture of upper limb
229	S52251K	Fracture of upper limb
229	S52251M	Fracture of upper limb
229	S52251N	Fracture of upper limb
229	S52251P	Fracture of upper limb
229	S52251Q	Fracture of upper limb
229	S52251R	Fracture of upper limb
229	S52251S	Fracture of upper limb
229	S52252A	Fracture of upper limb
229	S52252B	Fracture of upper limb
229	S52252C	Fracture of upper limb
229	S52252D	Fracture of upper limb
229	S52252E	Fracture of upper limb
229	S52252F	Fracture of upper limb
229	S52252G	Fracture of upper limb
229	S52252H	Fracture of upper limb
229	S52252J	Fracture of upper limb
229	S52252K	Fracture of upper limb
229	S52252M	Fracture of upper limb
229	S52252N	Fracture of upper limb
229	S52252P	Fracture of upper limb
229	S52252Q	Fracture of upper limb
229	S52252R	Fracture of upper limb
229	S52252S	Fracture of upper limb
229	S52253A	Fracture of upper limb
229	S52253B	Fracture of upper limb
229	S52253C	Fracture of upper limb
229	S52253D	Fracture of upper limb
229	S52253E	Fracture of upper limb
229	S52253F	Fracture of upper limb
229	S52253G	Fracture of upper limb
229	S52253H	Fracture of upper limb
229	S52253J	Fracture of upper limb
229	S52253K	Fracture of upper limb
229	S52253M	Fracture of upper limb
229	S52253N	Fracture of upper limb
229	S52253P	Fracture of upper limb

229	S52253Q	Fracture of upper limb
229	S52253R	Fracture of upper limb
229	S52253S	Fracture of upper limb
229	S52254A	Fracture of upper limb
229	S52254B	Fracture of upper limb
229	S52254C	Fracture of upper limb
229	S52254D	Fracture of upper limb
229	S52254E	Fracture of upper limb
229	S52254F	Fracture of upper limb
229	S52254G	Fracture of upper limb
229	S52254H	Fracture of upper limb
229	S52254J	Fracture of upper limb
229	S52254K	Fracture of upper limb
229	S52254M	Fracture of upper limb
229	S52254N	Fracture of upper limb
229	S52254P	Fracture of upper limb
229	S52254Q	Fracture of upper limb
229	S52254R	Fracture of upper limb
229	S52254S	Fracture of upper limb
229	S52255A	Fracture of upper limb
229	S52255B	Fracture of upper limb
229	S52255C	Fracture of upper limb
229	S52255D	Fracture of upper limb
229	S52255E	Fracture of upper limb
229	S52255F	Fracture of upper limb
229	S52255G	Fracture of upper limb
229	S52255H	Fracture of upper limb
229	S52255J	Fracture of upper limb
229	S52255K	Fracture of upper limb
229	S52255M	Fracture of upper limb
229	S52255N	Fracture of upper limb
229	S52255P	Fracture of upper limb
229	S52255Q	Fracture of upper limb
229	S52255R	Fracture of upper limb
229	S52255S	Fracture of upper limb
229	S52256A	Fracture of upper limb
229	S52256B	Fracture of upper limb
229	S52256C	Fracture of upper limb
229	S52256D	Fracture of upper limb
229	S52256E	Fracture of upper limb
229	S52256F	Fracture of upper limb
229	S52256G	Fracture of upper limb
229	S52256H	Fracture of upper limb
229	S52256J	Fracture of upper limb
229	S52256K	Fracture of upper limb
229	S52256M	Fracture of upper limb
229	S52256N	Fracture of upper limb
229	S52256P	Fracture of upper limb
229	S52256Q	Fracture of upper limb
229	S52256R	Fracture of upper limb
229	S52256S	Fracture of upper limb

229	S52261A	Fracture of upper limb
229	S52261B	Fracture of upper limb
229	S52261C	Fracture of upper limb
229	S52261D	Fracture of upper limb
229	S52261E	Fracture of upper limb
229	S52261F	Fracture of upper limb
229	S52261G	Fracture of upper limb
229	S52261H	Fracture of upper limb
229	S52261J	Fracture of upper limb
229	S52261K	Fracture of upper limb
229	S52261M	Fracture of upper limb
229	S52261N	Fracture of upper limb
229	S52261P	Fracture of upper limb
229	S52261Q	Fracture of upper limb
229	S52261R	Fracture of upper limb
229	S52261S	Fracture of upper limb
229	S52262A	Fracture of upper limb
229	S52262B	Fracture of upper limb
229	S52262C	Fracture of upper limb
229	S52262D	Fracture of upper limb
229	S52262E	Fracture of upper limb
229	S52262F	Fracture of upper limb
229	S52262G	Fracture of upper limb
229	S52262H	Fracture of upper limb
229	S52262J	Fracture of upper limb
229	S52262K	Fracture of upper limb
229	S52262M	Fracture of upper limb
229	S52262N	Fracture of upper limb
229	S52262P	Fracture of upper limb
229	S52262Q	Fracture of upper limb
229	S52262R	Fracture of upper limb
229	S52262S	Fracture of upper limb
229	S52263A	Fracture of upper limb
229	S52263B	Fracture of upper limb
229	S52263C	Fracture of upper limb
229	S52263D	Fracture of upper limb
229	S52263E	Fracture of upper limb
229	S52263F	Fracture of upper limb
229	S52263G	Fracture of upper limb
229	S52263H	Fracture of upper limb
229	S52263J	Fracture of upper limb
229	S52263K	Fracture of upper limb
229	S52263M	Fracture of upper limb
229	S52263N	Fracture of upper limb
229	S52263P	Fracture of upper limb
229	S52263Q	Fracture of upper limb
229	S52263R	Fracture of upper limb
229	S52263S	Fracture of upper limb
229	S52264A	Fracture of upper limb
229	S52264B	Fracture of upper limb
229	S52264C	Fracture of upper limb

229	S52264D	Fracture of upper limb
229	S52264E	Fracture of upper limb
229	S52264F	Fracture of upper limb
229	S52264G	Fracture of upper limb
229	S52264H	Fracture of upper limb
229	S52264J	Fracture of upper limb
229	S52264K	Fracture of upper limb
229	S52264M	Fracture of upper limb
229	S52264N	Fracture of upper limb
229	S52264P	Fracture of upper limb
229	S52264Q	Fracture of upper limb
229	S52264R	Fracture of upper limb
229	S52264S	Fracture of upper limb
229	S52265A	Fracture of upper limb
229	S52265B	Fracture of upper limb
229	S52265C	Fracture of upper limb
229	S52265D	Fracture of upper limb
229	S52265E	Fracture of upper limb
229	S52265F	Fracture of upper limb
229	S52265G	Fracture of upper limb
229	S52265H	Fracture of upper limb
229	S52265J	Fracture of upper limb
229	S52265K	Fracture of upper limb
229	S52265M	Fracture of upper limb
229	S52265N	Fracture of upper limb
229	S52265P	Fracture of upper limb
229	S52265Q	Fracture of upper limb
229	S52265R	Fracture of upper limb
229	S52265S	Fracture of upper limb
229	S52266A	Fracture of upper limb
229	S52266B	Fracture of upper limb
229	S52266C	Fracture of upper limb
229	S52266D	Fracture of upper limb
229	S52266E	Fracture of upper limb
229	S52266F	Fracture of upper limb
229	S52266G	Fracture of upper limb
229	S52266H	Fracture of upper limb
229	S52266J	Fracture of upper limb
229	S52266K	Fracture of upper limb
229	S52266M	Fracture of upper limb
229	S52266N	Fracture of upper limb
229	S52266P	Fracture of upper limb
229	S52266Q	Fracture of upper limb
229	S52266R	Fracture of upper limb
229	S52266S	Fracture of upper limb
229	S52271A	Fracture of upper limb
229	S52271B	Fracture of upper limb
229	S52271C	Fracture of upper limb
229	S52271D	Fracture of upper limb
229	S52271E	Fracture of upper limb
229	S52271F	Fracture of upper limb

229	S52271G	Fracture of upper limb
229	S52271H	Fracture of upper limb
229	S52271J	Fracture of upper limb
229	S52271K	Fracture of upper limb
229	S52271M	Fracture of upper limb
229	S52271N	Fracture of upper limb
229	S52271P	Fracture of upper limb
229	S52271Q	Fracture of upper limb
229	S52271R	Fracture of upper limb
229	S52271S	Fracture of upper limb
229	S52272A	Fracture of upper limb
229	S52272B	Fracture of upper limb
229	S52272C	Fracture of upper limb
229	S52272D	Fracture of upper limb
229	S52272E	Fracture of upper limb
229	S52272F	Fracture of upper limb
229	S52272G	Fracture of upper limb
229	S52272H	Fracture of upper limb
229	S52272J	Fracture of upper limb
229	S52272K	Fracture of upper limb
229	S52272M	Fracture of upper limb
229	S52272N	Fracture of upper limb
229	S52272P	Fracture of upper limb
229	S52272Q	Fracture of upper limb
229	S52272R	Fracture of upper limb
229	S52272S	Fracture of upper limb
229	S52279A	Fracture of upper limb
229	S52279B	Fracture of upper limb
229	S52279C	Fracture of upper limb
229	S52279D	Fracture of upper limb
229	S52279E	Fracture of upper limb
229	S52279F	Fracture of upper limb
229	S52279G	Fracture of upper limb
229	S52279H	Fracture of upper limb
229	S52279J	Fracture of upper limb
229	S52279K	Fracture of upper limb
229	S52279M	Fracture of upper limb
229	S52279N	Fracture of upper limb
229	S52279P	Fracture of upper limb
229	S52279Q	Fracture of upper limb
229	S52279R	Fracture of upper limb
229	S52279S	Fracture of upper limb
229	S52281A	Fracture of upper limb
229	S52281B	Fracture of upper limb
229	S52281C	Fracture of upper limb
229	S52281D	Fracture of upper limb
229	S52281E	Fracture of upper limb
229	S52281F	Fracture of upper limb
229	S52281G	Fracture of upper limb
229	S52281H	Fracture of upper limb
229	S52281J	Fracture of upper limb

229	S52281K	Fracture of upper limb
229	S52281M	Fracture of upper limb
229	S52281N	Fracture of upper limb
229	S52281P	Fracture of upper limb
229	S52281Q	Fracture of upper limb
229	S52281R	Fracture of upper limb
229	S52281S	Fracture of upper limb
229	S52282A	Fracture of upper limb
229	S52282B	Fracture of upper limb
229	S52282C	Fracture of upper limb
229	S52282D	Fracture of upper limb
229	S52282E	Fracture of upper limb
229	S52282F	Fracture of upper limb
229	S52282G	Fracture of upper limb
229	S52282H	Fracture of upper limb
229	S52282J	Fracture of upper limb
229	S52282K	Fracture of upper limb
229	S52282M	Fracture of upper limb
229	S52282N	Fracture of upper limb
229	S52282P	Fracture of upper limb
229	S52282Q	Fracture of upper limb
229	S52282R	Fracture of upper limb
229	S52282S	Fracture of upper limb
229	S52283A	Fracture of upper limb
229	S52283B	Fracture of upper limb
229	S52283C	Fracture of upper limb
229	S52283D	Fracture of upper limb
229	S52283E	Fracture of upper limb
229	S52283F	Fracture of upper limb
229	S52283G	Fracture of upper limb
229	S52283H	Fracture of upper limb
229	S52283J	Fracture of upper limb
229	S52283K	Fracture of upper limb
229	S52283M	Fracture of upper limb
229	S52283N	Fracture of upper limb
229	S52283P	Fracture of upper limb
229	S52283Q	Fracture of upper limb
229	S52283R	Fracture of upper limb
229	S52283S	Fracture of upper limb
229	S52291A	Fracture of upper limb
229	S52291B	Fracture of upper limb
229	S52291C	Fracture of upper limb
229	S52291D	Fracture of upper limb
229	S52291E	Fracture of upper limb
229	S52291F	Fracture of upper limb
229	S52291G	Fracture of upper limb
229	S52291H	Fracture of upper limb
229	S52291J	Fracture of upper limb
229	S52291K	Fracture of upper limb
229	S52291M	Fracture of upper limb
229	S52291N	Fracture of upper limb

229	S52291P	Fracture of upper limb
229	S52291Q	Fracture of upper limb
229	S52291R	Fracture of upper limb
229	S52291S	Fracture of upper limb
229	S52292A	Fracture of upper limb
229	S52292B	Fracture of upper limb
229	S52292C	Fracture of upper limb
229	S52292D	Fracture of upper limb
229	S52292E	Fracture of upper limb
229	S52292F	Fracture of upper limb
229	S52292G	Fracture of upper limb
229	S52292H	Fracture of upper limb
229	S52292J	Fracture of upper limb
229	S52292K	Fracture of upper limb
229	S52292M	Fracture of upper limb
229	S52292N	Fracture of upper limb
229	S52292P	Fracture of upper limb
229	S52292Q	Fracture of upper limb
229	S52292R	Fracture of upper limb
229	S52292S	Fracture of upper limb
229	S52299A	Fracture of upper limb
229	S52299B	Fracture of upper limb
229	S52299C	Fracture of upper limb
229	S52299D	Fracture of upper limb
229	S52299E	Fracture of upper limb
229	S52299F	Fracture of upper limb
229	S52299G	Fracture of upper limb
229	S52299H	Fracture of upper limb
229	S52299J	Fracture of upper limb
229	S52299K	Fracture of upper limb
229	S52299M	Fracture of upper limb
229	S52299N	Fracture of upper limb
229	S52299P	Fracture of upper limb
229	S52299Q	Fracture of upper limb
229	S52299R	Fracture of upper limb
229	S52299S	Fracture of upper limb
229	S52301A	Fracture of upper limb
229	S52301B	Fracture of upper limb
229	S52301C	Fracture of upper limb
229	S52301D	Fracture of upper limb
229	S52301E	Fracture of upper limb
229	S52301F	Fracture of upper limb
229	S52301G	Fracture of upper limb
229	S52301H	Fracture of upper limb
229	S52301J	Fracture of upper limb
229	S52301K	Fracture of upper limb
229	S52301M	Fracture of upper limb
229	S52301N	Fracture of upper limb
229	S52301P	Fracture of upper limb
229	S52301Q	Fracture of upper limb
229	S52301R	Fracture of upper limb

229	S52301S	Fracture of upper limb
229	S52302A	Fracture of upper limb
229	S52302B	Fracture of upper limb
229	S52302C	Fracture of upper limb
229	S52302D	Fracture of upper limb
229	S52302E	Fracture of upper limb
229	S52302F	Fracture of upper limb
229	S52302G	Fracture of upper limb
229	S52302H	Fracture of upper limb
229	S52302J	Fracture of upper limb
229	S52302K	Fracture of upper limb
229	S52302M	Fracture of upper limb
229	S52302N	Fracture of upper limb
229	S52302P	Fracture of upper limb
229	S52302Q	Fracture of upper limb
229	S52302R	Fracture of upper limb
229	S52302S	Fracture of upper limb
229	S52309A	Fracture of upper limb
229	S52309B	Fracture of upper limb
229	S52309C	Fracture of upper limb
229	S52309D	Fracture of upper limb
229	S52309E	Fracture of upper limb
229	S52309F	Fracture of upper limb
229	S52309G	Fracture of upper limb
229	S52309H	Fracture of upper limb
229	S52309J	Fracture of upper limb
229	S52309K	Fracture of upper limb
229	S52309M	Fracture of upper limb
229	S52309N	Fracture of upper limb
229	S52309P	Fracture of upper limb
229	S52309Q	Fracture of upper limb
229	S52309R	Fracture of upper limb
229	S52309S	Fracture of upper limb
229	S52311A	Fracture of upper limb
229	S52311D	Fracture of upper limb
229	S52311G	Fracture of upper limb
229	S52311K	Fracture of upper limb
229	S52311P	Fracture of upper limb
229	S52311S	Fracture of upper limb
229	S52312A	Fracture of upper limb
229	S52312D	Fracture of upper limb
229	S52312G	Fracture of upper limb
229	S52312K	Fracture of upper limb
229	S52312P	Fracture of upper limb
229	S52312S	Fracture of upper limb
229	S52319A	Fracture of upper limb
229	S52319D	Fracture of upper limb
229	S52319G	Fracture of upper limb
229	S52319K	Fracture of upper limb
229	S52319P	Fracture of upper limb
229	S52319S	Fracture of upper limb

229	S52321A	Fracture of upper limb
229	S52321B	Fracture of upper limb
229	S52321C	Fracture of upper limb
229	S52321D	Fracture of upper limb
229	S52321E	Fracture of upper limb
229	S52321F	Fracture of upper limb
229	S52321G	Fracture of upper limb
229	S52321H	Fracture of upper limb
229	S52321J	Fracture of upper limb
229	S52321K	Fracture of upper limb
229	S52321M	Fracture of upper limb
229	S52321N	Fracture of upper limb
229	S52321P	Fracture of upper limb
229	S52321Q	Fracture of upper limb
229	S52321R	Fracture of upper limb
229	S52321S	Fracture of upper limb
229	S52322A	Fracture of upper limb
229	S52322B	Fracture of upper limb
229	S52322C	Fracture of upper limb
229	S52322D	Fracture of upper limb
229	S52322E	Fracture of upper limb
229	S52322F	Fracture of upper limb
229	S52322G	Fracture of upper limb
229	S52322H	Fracture of upper limb
229	S52322J	Fracture of upper limb
229	S52322K	Fracture of upper limb
229	S52322M	Fracture of upper limb
229	S52322N	Fracture of upper limb
229	S52322P	Fracture of upper limb
229	S52322Q	Fracture of upper limb
229	S52322R	Fracture of upper limb
229	S52322S	Fracture of upper limb
229	S52323A	Fracture of upper limb
229	S52323B	Fracture of upper limb
229	S52323C	Fracture of upper limb
229	S52323D	Fracture of upper limb
229	S52323E	Fracture of upper limb
229	S52323F	Fracture of upper limb
229	S52323G	Fracture of upper limb
229	S52323H	Fracture of upper limb
229	S52323J	Fracture of upper limb
229	S52323K	Fracture of upper limb
229	S52323M	Fracture of upper limb
229	S52323N	Fracture of upper limb
229	S52323P	Fracture of upper limb
229	S52323Q	Fracture of upper limb
229	S52323R	Fracture of upper limb
229	S52323S	Fracture of upper limb
229	S52324A	Fracture of upper limb
229	S52324B	Fracture of upper limb
229	S52324C	Fracture of upper limb

229	S52324D	Fracture of upper limb
229	S52324E	Fracture of upper limb
229	S52324F	Fracture of upper limb
229	S52324G	Fracture of upper limb
229	S52324H	Fracture of upper limb
229	S52324J	Fracture of upper limb
229	S52324K	Fracture of upper limb
229	S52324M	Fracture of upper limb
229	S52324N	Fracture of upper limb
229	S52324P	Fracture of upper limb
229	S52324Q	Fracture of upper limb
229	S52324R	Fracture of upper limb
229	S52324S	Fracture of upper limb
229	S52325A	Fracture of upper limb
229	S52325B	Fracture of upper limb
229	S52325C	Fracture of upper limb
229	S52325D	Fracture of upper limb
229	S52325E	Fracture of upper limb
229	S52325F	Fracture of upper limb
229	S52325G	Fracture of upper limb
229	S52325H	Fracture of upper limb
229	S52325J	Fracture of upper limb
229	S52325K	Fracture of upper limb
229	S52325M	Fracture of upper limb
229	S52325N	Fracture of upper limb
229	S52325P	Fracture of upper limb
229	S52325Q	Fracture of upper limb
229	S52325R	Fracture of upper limb
229	S52325S	Fracture of upper limb
229	S52326A	Fracture of upper limb
229	S52326B	Fracture of upper limb
229	S52326C	Fracture of upper limb
229	S52326D	Fracture of upper limb
229	S52326E	Fracture of upper limb
229	S52326F	Fracture of upper limb
229	S52326G	Fracture of upper limb
229	S52326H	Fracture of upper limb
229	S52326J	Fracture of upper limb
229	S52326K	Fracture of upper limb
229	S52326M	Fracture of upper limb
229	S52326N	Fracture of upper limb
229	S52326P	Fracture of upper limb
229	S52326Q	Fracture of upper limb
229	S52326R	Fracture of upper limb
229	S52326S	Fracture of upper limb
229	S52331A	Fracture of upper limb
229	S52331B	Fracture of upper limb
229	S52331C	Fracture of upper limb
229	S52331D	Fracture of upper limb
229	S52331E	Fracture of upper limb
229	S52331F	Fracture of upper limb

229	S52331G	Fracture of upper limb
229	S52331H	Fracture of upper limb
229	S52331J	Fracture of upper limb
229	S52331K	Fracture of upper limb
229	S52331M	Fracture of upper limb
229	S52331N	Fracture of upper limb
229	S52331P	Fracture of upper limb
229	S52331Q	Fracture of upper limb
229	S52331R	Fracture of upper limb
229	S52331S	Fracture of upper limb
229	S52332A	Fracture of upper limb
229	S52332B	Fracture of upper limb
229	S52332C	Fracture of upper limb
229	S52332D	Fracture of upper limb
229	S52332E	Fracture of upper limb
229	S52332F	Fracture of upper limb
229	S52332G	Fracture of upper limb
229	S52332H	Fracture of upper limb
229	S52332J	Fracture of upper limb
229	S52332K	Fracture of upper limb
229	S52332M	Fracture of upper limb
229	S52332N	Fracture of upper limb
229	S52332P	Fracture of upper limb
229	S52332Q	Fracture of upper limb
229	S52332R	Fracture of upper limb
229	S52332S	Fracture of upper limb
229	S52333A	Fracture of upper limb
229	S52333B	Fracture of upper limb
229	S52333C	Fracture of upper limb
229	S52333D	Fracture of upper limb
229	S52333E	Fracture of upper limb
229	S52333F	Fracture of upper limb
229	S52333G	Fracture of upper limb
229	S52333H	Fracture of upper limb
229	S52333J	Fracture of upper limb
229	S52333K	Fracture of upper limb
229	S52333M	Fracture of upper limb
229	S52333N	Fracture of upper limb
229	S52333P	Fracture of upper limb
229	S52333Q	Fracture of upper limb
229	S52333R	Fracture of upper limb
229	S52333S	Fracture of upper limb
229	S52334A	Fracture of upper limb
229	S52334B	Fracture of upper limb
229	S52334C	Fracture of upper limb
229	S52334D	Fracture of upper limb
229	S52334E	Fracture of upper limb
229	S52334F	Fracture of upper limb
229	S52334G	Fracture of upper limb
229	S52334H	Fracture of upper limb
229	S52334J	Fracture of upper limb

229	S52334K	Fracture of upper limb
229	S52334M	Fracture of upper limb
229	S52334N	Fracture of upper limb
229	S52334P	Fracture of upper limb
229	S52334Q	Fracture of upper limb
229	S52334R	Fracture of upper limb
229	S52334S	Fracture of upper limb
229	S52335A	Fracture of upper limb
229	S52335B	Fracture of upper limb
229	S52335C	Fracture of upper limb
229	S52335D	Fracture of upper limb
229	S52335E	Fracture of upper limb
229	S52335F	Fracture of upper limb
229	S52335G	Fracture of upper limb
229	S52335H	Fracture of upper limb
229	S52335J	Fracture of upper limb
229	S52335K	Fracture of upper limb
229	S52335M	Fracture of upper limb
229	S52335N	Fracture of upper limb
229	S52335P	Fracture of upper limb
229	S52335Q	Fracture of upper limb
229	S52335R	Fracture of upper limb
229	S52335S	Fracture of upper limb
229	S52336A	Fracture of upper limb
229	S52336B	Fracture of upper limb
229	S52336C	Fracture of upper limb
229	S52336D	Fracture of upper limb
229	S52336E	Fracture of upper limb
229	S52336F	Fracture of upper limb
229	S52336G	Fracture of upper limb
229	S52336H	Fracture of upper limb
229	S52336J	Fracture of upper limb
229	S52336K	Fracture of upper limb
229	S52336M	Fracture of upper limb
229	S52336N	Fracture of upper limb
229	S52336P	Fracture of upper limb
229	S52336Q	Fracture of upper limb
229	S52336R	Fracture of upper limb
229	S52336S	Fracture of upper limb
229	S52341A	Fracture of upper limb
229	S52341B	Fracture of upper limb
229	S52341C	Fracture of upper limb
229	S52341D	Fracture of upper limb
229	S52341E	Fracture of upper limb
229	S52341F	Fracture of upper limb
229	S52341G	Fracture of upper limb
229	S52341H	Fracture of upper limb
229	S52341J	Fracture of upper limb
229	S52341K	Fracture of upper limb
229	S52341M	Fracture of upper limb
229	S52341N	Fracture of upper limb

229	S52341P	Fracture of upper limb
229	S52341Q	Fracture of upper limb
229	S52341R	Fracture of upper limb
229	S52341S	Fracture of upper limb
229	S52342A	Fracture of upper limb
229	S52342B	Fracture of upper limb
229	S52342C	Fracture of upper limb
229	S52342D	Fracture of upper limb
229	S52342E	Fracture of upper limb
229	S52342F	Fracture of upper limb
229	S52342G	Fracture of upper limb
229	S52342H	Fracture of upper limb
229	S52342J	Fracture of upper limb
229	S52342K	Fracture of upper limb
229	S52342M	Fracture of upper limb
229	S52342N	Fracture of upper limb
229	S52342P	Fracture of upper limb
229	S52342Q	Fracture of upper limb
229	S52342R	Fracture of upper limb
229	S52342S	Fracture of upper limb
229	S52343A	Fracture of upper limb
229	S52343B	Fracture of upper limb
229	S52343C	Fracture of upper limb
229	S52343D	Fracture of upper limb
229	S52343E	Fracture of upper limb
229	S52343F	Fracture of upper limb
229	S52343G	Fracture of upper limb
229	S52343H	Fracture of upper limb
229	S52343J	Fracture of upper limb
229	S52343K	Fracture of upper limb
229	S52343M	Fracture of upper limb
229	S52343N	Fracture of upper limb
229	S52343P	Fracture of upper limb
229	S52343Q	Fracture of upper limb
229	S52343R	Fracture of upper limb
229	S52343S	Fracture of upper limb
229	S52344A	Fracture of upper limb
229	S52344B	Fracture of upper limb
229	S52344C	Fracture of upper limb
229	S52344D	Fracture of upper limb
229	S52344E	Fracture of upper limb
229	S52344F	Fracture of upper limb
229	S52344G	Fracture of upper limb
229	S52344H	Fracture of upper limb
229	S52344J	Fracture of upper limb
229	S52344K	Fracture of upper limb
229	S52344M	Fracture of upper limb
229	S52344N	Fracture of upper limb
229	S52344P	Fracture of upper limb
229	S52344Q	Fracture of upper limb
229	S52344R	Fracture of upper limb

229	S52344S	Fracture of upper limb
229	S52345A	Fracture of upper limb
229	S52345B	Fracture of upper limb
229	S52345C	Fracture of upper limb
229	S52345D	Fracture of upper limb
229	S52345E	Fracture of upper limb
229	S52345F	Fracture of upper limb
229	S52345G	Fracture of upper limb
229	S52345H	Fracture of upper limb
229	S52345J	Fracture of upper limb
229	S52345K	Fracture of upper limb
229	S52345M	Fracture of upper limb
229	S52345N	Fracture of upper limb
229	S52345P	Fracture of upper limb
229	S52345Q	Fracture of upper limb
229	S52345R	Fracture of upper limb
229	S52345S	Fracture of upper limb
229	S52346A	Fracture of upper limb
229	S52346B	Fracture of upper limb
229	S52346C	Fracture of upper limb
229	S52346D	Fracture of upper limb
229	S52346E	Fracture of upper limb
229	S52346F	Fracture of upper limb
229	S52346G	Fracture of upper limb
229	S52346H	Fracture of upper limb
229	S52346J	Fracture of upper limb
229	S52346K	Fracture of upper limb
229	S52346M	Fracture of upper limb
229	S52346N	Fracture of upper limb
229	S52346P	Fracture of upper limb
229	S52346Q	Fracture of upper limb
229	S52346R	Fracture of upper limb
229	S52346S	Fracture of upper limb
229	S52351A	Fracture of upper limb
229	S52351B	Fracture of upper limb
229	S52351C	Fracture of upper limb
229	S52351D	Fracture of upper limb
229	S52351E	Fracture of upper limb
229	S52351F	Fracture of upper limb
229	S52351G	Fracture of upper limb
229	S52351H	Fracture of upper limb
229	S52351J	Fracture of upper limb
229	S52351K	Fracture of upper limb
229	S52351M	Fracture of upper limb
229	S52351N	Fracture of upper limb
229	S52351P	Fracture of upper limb
229	S52351Q	Fracture of upper limb
229	S52351R	Fracture of upper limb
229	S52351S	Fracture of upper limb
229	S52352A	Fracture of upper limb
229	S52352B	Fracture of upper limb

229	S52352C	Fracture of upper limb
229	S52352D	Fracture of upper limb
229	S52352E	Fracture of upper limb
229	S52352F	Fracture of upper limb
229	S52352G	Fracture of upper limb
229	S52352H	Fracture of upper limb
229	S52352J	Fracture of upper limb
229	S52352K	Fracture of upper limb
229	S52352M	Fracture of upper limb
229	S52352N	Fracture of upper limb
229	S52352P	Fracture of upper limb
229	S52352Q	Fracture of upper limb
229	S52352R	Fracture of upper limb
229	S52352S	Fracture of upper limb
229	S52353A	Fracture of upper limb
229	S52353B	Fracture of upper limb
229	S52353C	Fracture of upper limb
229	S52353D	Fracture of upper limb
229	S52353E	Fracture of upper limb
229	S52353F	Fracture of upper limb
229	S52353G	Fracture of upper limb
229	S52353H	Fracture of upper limb
229	S52353J	Fracture of upper limb
229	S52353K	Fracture of upper limb
229	S52353M	Fracture of upper limb
229	S52353N	Fracture of upper limb
229	S52353P	Fracture of upper limb
229	S52353Q	Fracture of upper limb
229	S52353R	Fracture of upper limb
229	S52353S	Fracture of upper limb
229	S52354A	Fracture of upper limb
229	S52354B	Fracture of upper limb
229	S52354C	Fracture of upper limb
229	S52354D	Fracture of upper limb
229	S52354E	Fracture of upper limb
229	S52354F	Fracture of upper limb
229	S52354G	Fracture of upper limb
229	S52354H	Fracture of upper limb
229	S52354J	Fracture of upper limb
229	S52354K	Fracture of upper limb
229	S52354M	Fracture of upper limb
229	S52354N	Fracture of upper limb
229	S52354P	Fracture of upper limb
229	S52354Q	Fracture of upper limb
229	S52354R	Fracture of upper limb
229	S52354S	Fracture of upper limb
229	S52355A	Fracture of upper limb
229	S52355B	Fracture of upper limb
229	S52355C	Fracture of upper limb
229	S52355D	Fracture of upper limb
229	S52355E	Fracture of upper limb

229	S52355F	Fracture of upper limb
229	S52355G	Fracture of upper limb
229	S52355H	Fracture of upper limb
229	S52355J	Fracture of upper limb
229	S52355K	Fracture of upper limb
229	S52355M	Fracture of upper limb
229	S52355N	Fracture of upper limb
229	S52355P	Fracture of upper limb
229	S52355Q	Fracture of upper limb
229	S52355R	Fracture of upper limb
229	S52355S	Fracture of upper limb
229	S52356A	Fracture of upper limb
229	S52356B	Fracture of upper limb
229	S52356C	Fracture of upper limb
229	S52356D	Fracture of upper limb
229	S52356E	Fracture of upper limb
229	S52356F	Fracture of upper limb
229	S52356G	Fracture of upper limb
229	S52356H	Fracture of upper limb
229	S52356J	Fracture of upper limb
229	S52356K	Fracture of upper limb
229	S52356M	Fracture of upper limb
229	S52356N	Fracture of upper limb
229	S52356P	Fracture of upper limb
229	S52356Q	Fracture of upper limb
229	S52356R	Fracture of upper limb
229	S52356S	Fracture of upper limb
229	S52361A	Fracture of upper limb
229	S52361B	Fracture of upper limb
229	S52361C	Fracture of upper limb
229	S52361D	Fracture of upper limb
229	S52361E	Fracture of upper limb
229	S52361F	Fracture of upper limb
229	S52361G	Fracture of upper limb
229	S52361H	Fracture of upper limb
229	S52361J	Fracture of upper limb
229	S52361K	Fracture of upper limb
229	S52361M	Fracture of upper limb
229	S52361N	Fracture of upper limb
229	S52361P	Fracture of upper limb
229	S52361Q	Fracture of upper limb
229	S52361R	Fracture of upper limb
229	S52361S	Fracture of upper limb
229	S52362A	Fracture of upper limb
229	S52362B	Fracture of upper limb
229	S52362C	Fracture of upper limb
229	S52362D	Fracture of upper limb
229	S52362E	Fracture of upper limb
229	S52362F	Fracture of upper limb
229	S52362G	Fracture of upper limb
229	S52362H	Fracture of upper limb

229	S52362J	Fracture of upper limb
229	S52362K	Fracture of upper limb
229	S52362M	Fracture of upper limb
229	S52362N	Fracture of upper limb
229	S52362P	Fracture of upper limb
229	S52362Q	Fracture of upper limb
229	S52362R	Fracture of upper limb
229	S52362S	Fracture of upper limb
229	S52363A	Fracture of upper limb
229	S52363B	Fracture of upper limb
229	S52363C	Fracture of upper limb
229	S52363D	Fracture of upper limb
229	S52363E	Fracture of upper limb
229	S52363F	Fracture of upper limb
229	S52363G	Fracture of upper limb
229	S52363H	Fracture of upper limb
229	S52363J	Fracture of upper limb
229	S52363K	Fracture of upper limb
229	S52363M	Fracture of upper limb
229	S52363N	Fracture of upper limb
229	S52363P	Fracture of upper limb
229	S52363Q	Fracture of upper limb
229	S52363R	Fracture of upper limb
229	S52363S	Fracture of upper limb
229	S52364A	Fracture of upper limb
229	S52364B	Fracture of upper limb
229	S52364C	Fracture of upper limb
229	S52364D	Fracture of upper limb
229	S52364E	Fracture of upper limb
229	S52364F	Fracture of upper limb
229	S52364G	Fracture of upper limb
229	S52364H	Fracture of upper limb
229	S52364J	Fracture of upper limb
229	S52364K	Fracture of upper limb
229	S52364M	Fracture of upper limb
229	S52364N	Fracture of upper limb
229	S52364P	Fracture of upper limb
229	S52364Q	Fracture of upper limb
229	S52364R	Fracture of upper limb
229	S52364S	Fracture of upper limb
229	S52365A	Fracture of upper limb
229	S52365B	Fracture of upper limb
229	S52365C	Fracture of upper limb
229	S52365D	Fracture of upper limb
229	S52365E	Fracture of upper limb
229	S52365F	Fracture of upper limb
229	S52365G	Fracture of upper limb
229	S52365H	Fracture of upper limb
229	S52365J	Fracture of upper limb
229	S52365K	Fracture of upper limb
229	S52365M	Fracture of upper limb

229	S52365N	Fracture of upper limb
229	S52365P	Fracture of upper limb
229	S52365Q	Fracture of upper limb
229	S52365R	Fracture of upper limb
229	S52365S	Fracture of upper limb
229	S52366A	Fracture of upper limb
229	S52366B	Fracture of upper limb
229	S52366C	Fracture of upper limb
229	S52366D	Fracture of upper limb
229	S52366E	Fracture of upper limb
229	S52366F	Fracture of upper limb
229	S52366G	Fracture of upper limb
229	S52366H	Fracture of upper limb
229	S52366J	Fracture of upper limb
229	S52366K	Fracture of upper limb
229	S52366M	Fracture of upper limb
229	S52366N	Fracture of upper limb
229	S52366P	Fracture of upper limb
229	S52366Q	Fracture of upper limb
229	S52366R	Fracture of upper limb
229	S52366S	Fracture of upper limb
229	S52371A	Fracture of upper limb
229	S52371B	Fracture of upper limb
229	S52371C	Fracture of upper limb
229	S52371D	Fracture of upper limb
229	S52371E	Fracture of upper limb
229	S52371F	Fracture of upper limb
229	S52371G	Fracture of upper limb
229	S52371H	Fracture of upper limb
229	S52371J	Fracture of upper limb
229	S52371K	Fracture of upper limb
229	S52371M	Fracture of upper limb
229	S52371N	Fracture of upper limb
229	S52371P	Fracture of upper limb
229	S52371Q	Fracture of upper limb
229	S52371R	Fracture of upper limb
229	S52371S	Fracture of upper limb
229	S52372A	Fracture of upper limb
229	S52372B	Fracture of upper limb
229	S52372C	Fracture of upper limb
229	S52372D	Fracture of upper limb
229	S52372E	Fracture of upper limb
229	S52372F	Fracture of upper limb
229	S52372G	Fracture of upper limb
229	S52372H	Fracture of upper limb
229	S52372J	Fracture of upper limb
229	S52372K	Fracture of upper limb
229	S52372M	Fracture of upper limb
229	S52372N	Fracture of upper limb
229	S52372P	Fracture of upper limb
229	S52372Q	Fracture of upper limb

229	S52372R	Fracture of upper limb
229	S52372S	Fracture of upper limb
229	S52379A	Fracture of upper limb
229	S52379B	Fracture of upper limb
229	S52379C	Fracture of upper limb
229	S52379D	Fracture of upper limb
229	S52379E	Fracture of upper limb
229	S52379F	Fracture of upper limb
229	S52379G	Fracture of upper limb
229	S52379H	Fracture of upper limb
229	S52379J	Fracture of upper limb
229	S52379K	Fracture of upper limb
229	S52379M	Fracture of upper limb
229	S52379N	Fracture of upper limb
229	S52379P	Fracture of upper limb
229	S52379Q	Fracture of upper limb
229	S52379R	Fracture of upper limb
229	S52379S	Fracture of upper limb
229	S52381A	Fracture of upper limb
229	S52381B	Fracture of upper limb
229	S52381C	Fracture of upper limb
229	S52381D	Fracture of upper limb
229	S52381E	Fracture of upper limb
229	S52381F	Fracture of upper limb
229	S52381G	Fracture of upper limb
229	S52381H	Fracture of upper limb
229	S52381J	Fracture of upper limb
229	S52381K	Fracture of upper limb
229	S52381M	Fracture of upper limb
229	S52381N	Fracture of upper limb
229	S52381P	Fracture of upper limb
229	S52381Q	Fracture of upper limb
229	S52381R	Fracture of upper limb
229	S52381S	Fracture of upper limb
229	S52382A	Fracture of upper limb
229	S52382B	Fracture of upper limb
229	S52382C	Fracture of upper limb
229	S52382D	Fracture of upper limb
229	S52382E	Fracture of upper limb
229	S52382F	Fracture of upper limb
229	S52382G	Fracture of upper limb
229	S52382H	Fracture of upper limb
229	S52382J	Fracture of upper limb
229	S52382K	Fracture of upper limb
229	S52382M	Fracture of upper limb
229	S52382N	Fracture of upper limb
229	S52382P	Fracture of upper limb
229	S52382Q	Fracture of upper limb
229	S52382R	Fracture of upper limb
229	S52382S	Fracture of upper limb
229	S52389A	Fracture of upper limb

229	S52389B	Fracture of upper limb
229	S52389C	Fracture of upper limb
229	S52389D	Fracture of upper limb
229	S52389E	Fracture of upper limb
229	S52389F	Fracture of upper limb
229	S52389G	Fracture of upper limb
229	S52389H	Fracture of upper limb
229	S52389J	Fracture of upper limb
229	S52389K	Fracture of upper limb
229	S52389M	Fracture of upper limb
229	S52389N	Fracture of upper limb
229	S52389P	Fracture of upper limb
229	S52389Q	Fracture of upper limb
229	S52389R	Fracture of upper limb
229	S52389S	Fracture of upper limb
229	S52391A	Fracture of upper limb
229	S52391B	Fracture of upper limb
229	S52391C	Fracture of upper limb
229	S52391D	Fracture of upper limb
229	S52391E	Fracture of upper limb
229	S52391F	Fracture of upper limb
229	S52391G	Fracture of upper limb
229	S52391H	Fracture of upper limb
229	S52391J	Fracture of upper limb
229	S52391K	Fracture of upper limb
229	S52391M	Fracture of upper limb
229	S52391N	Fracture of upper limb
229	S52391P	Fracture of upper limb
229	S52391Q	Fracture of upper limb
229	S52391R	Fracture of upper limb
229	S52391S	Fracture of upper limb
229	S52392A	Fracture of upper limb
229	S52392B	Fracture of upper limb
229	S52392C	Fracture of upper limb
229	S52392D	Fracture of upper limb
229	S52392E	Fracture of upper limb
229	S52392F	Fracture of upper limb
229	S52392G	Fracture of upper limb
229	S52392H	Fracture of upper limb
229	S52392J	Fracture of upper limb
229	S52392K	Fracture of upper limb
229	S52392M	Fracture of upper limb
229	S52392N	Fracture of upper limb
229	S52392P	Fracture of upper limb
229	S52392Q	Fracture of upper limb
229	S52392R	Fracture of upper limb
229	S52392S	Fracture of upper limb
229	S52399A	Fracture of upper limb
229	S52399B	Fracture of upper limb
229	S52399C	Fracture of upper limb
229	S52399D	Fracture of upper limb

229	S52399E	Fracture of upper limb
229	S52399F	Fracture of upper limb
229	S52399G	Fracture of upper limb
229	S52399H	Fracture of upper limb
229	S52399J	Fracture of upper limb
229	S52399K	Fracture of upper limb
229	S52399M	Fracture of upper limb
229	S52399N	Fracture of upper limb
229	S52399P	Fracture of upper limb
229	S52399Q	Fracture of upper limb
229	S52399R	Fracture of upper limb
229	S52399S	Fracture of upper limb
229	S52501A	Fracture of upper limb
229	S52501B	Fracture of upper limb
229	S52501C	Fracture of upper limb
229	S52501D	Fracture of upper limb
229	S52501E	Fracture of upper limb
229	S52501F	Fracture of upper limb
229	S52501G	Fracture of upper limb
229	S52501H	Fracture of upper limb
229	S52501J	Fracture of upper limb
229	S52501K	Fracture of upper limb
229	S52501M	Fracture of upper limb
229	S52501N	Fracture of upper limb
229	S52501P	Fracture of upper limb
229	S52501Q	Fracture of upper limb
229	S52501R	Fracture of upper limb
229	S52501S	Fracture of upper limb
229	S52502A	Fracture of upper limb
229	S52502B	Fracture of upper limb
229	S52502C	Fracture of upper limb
229	S52502D	Fracture of upper limb
229	S52502E	Fracture of upper limb
229	S52502F	Fracture of upper limb
229	S52502G	Fracture of upper limb
229	S52502H	Fracture of upper limb
229	S52502J	Fracture of upper limb
229	S52502K	Fracture of upper limb
229	S52502M	Fracture of upper limb
229	S52502N	Fracture of upper limb
229	S52502P	Fracture of upper limb
229	S52502Q	Fracture of upper limb
229	S52502R	Fracture of upper limb
229	S52502S	Fracture of upper limb
229	S52509A	Fracture of upper limb
229	S52509B	Fracture of upper limb
229	S52509C	Fracture of upper limb
229	S52509D	Fracture of upper limb
229	S52509E	Fracture of upper limb
229	S52509F	Fracture of upper limb
229	S52509G	Fracture of upper limb

229	S52509H	Fracture of upper limb
229	S52509J	Fracture of upper limb
229	S52509K	Fracture of upper limb
229	S52509M	Fracture of upper limb
229	S52509N	Fracture of upper limb
229	S52509P	Fracture of upper limb
229	S52509Q	Fracture of upper limb
229	S52509R	Fracture of upper limb
229	S52509S	Fracture of upper limb
229	S52511A	Fracture of upper limb
229	S52511B	Fracture of upper limb
229	S52511C	Fracture of upper limb
229	S52511D	Fracture of upper limb
229	S52511E	Fracture of upper limb
229	S52511F	Fracture of upper limb
229	S52511G	Fracture of upper limb
229	S52511H	Fracture of upper limb
229	S52511J	Fracture of upper limb
229	S52511K	Fracture of upper limb
229	S52511M	Fracture of upper limb
229	S52511N	Fracture of upper limb
229	S52511P	Fracture of upper limb
229	S52511Q	Fracture of upper limb
229	S52511R	Fracture of upper limb
229	S52511S	Fracture of upper limb
229	S52512A	Fracture of upper limb
229	S52512B	Fracture of upper limb
229	S52512C	Fracture of upper limb
229	S52512D	Fracture of upper limb
229	S52512E	Fracture of upper limb
229	S52512F	Fracture of upper limb
229	S52512G	Fracture of upper limb
229	S52512H	Fracture of upper limb
229	S52512J	Fracture of upper limb
229	S52512K	Fracture of upper limb
229	S52512M	Fracture of upper limb
229	S52512N	Fracture of upper limb
229	S52512P	Fracture of upper limb
229	S52512Q	Fracture of upper limb
229	S52512R	Fracture of upper limb
229	S52512S	Fracture of upper limb
229	S52513A	Fracture of upper limb
229	S52513B	Fracture of upper limb
229	S52513C	Fracture of upper limb
229	S52513D	Fracture of upper limb
229	S52513E	Fracture of upper limb
229	S52513F	Fracture of upper limb
229	S52513G	Fracture of upper limb
229	S52513H	Fracture of upper limb
229	S52513J	Fracture of upper limb
229	S52513K	Fracture of upper limb

229	S52513M	Fracture of upper limb
229	S52513N	Fracture of upper limb
229	S52513P	Fracture of upper limb
229	S52513Q	Fracture of upper limb
229	S52513R	Fracture of upper limb
229	S52513S	Fracture of upper limb
229	S52514A	Fracture of upper limb
229	S52514B	Fracture of upper limb
229	S52514C	Fracture of upper limb
229	S52514D	Fracture of upper limb
229	S52514E	Fracture of upper limb
229	S52514F	Fracture of upper limb
229	S52514G	Fracture of upper limb
229	S52514H	Fracture of upper limb
229	S52514J	Fracture of upper limb
229	S52514K	Fracture of upper limb
229	S52514M	Fracture of upper limb
229	S52514N	Fracture of upper limb
229	S52514P	Fracture of upper limb
229	S52514Q	Fracture of upper limb
229	S52514R	Fracture of upper limb
229	S52514S	Fracture of upper limb
229	S52515A	Fracture of upper limb
229	S52515B	Fracture of upper limb
229	S52515C	Fracture of upper limb
229	S52515D	Fracture of upper limb
229	S52515E	Fracture of upper limb
229	S52515F	Fracture of upper limb
229	S52515G	Fracture of upper limb
229	S52515H	Fracture of upper limb
229	S52515J	Fracture of upper limb
229	S52515K	Fracture of upper limb
229	S52515M	Fracture of upper limb
229	S52515N	Fracture of upper limb
229	S52515P	Fracture of upper limb
229	S52515Q	Fracture of upper limb
229	S52515R	Fracture of upper limb
229	S52515S	Fracture of upper limb
229	S52516A	Fracture of upper limb
229	S52516B	Fracture of upper limb
229	S52516C	Fracture of upper limb
229	S52516D	Fracture of upper limb
229	S52516E	Fracture of upper limb
229	S52516F	Fracture of upper limb
229	S52516G	Fracture of upper limb
229	S52516H	Fracture of upper limb
229	S52516J	Fracture of upper limb
229	S52516K	Fracture of upper limb
229	S52516M	Fracture of upper limb
229	S52516N	Fracture of upper limb
229	S52516P	Fracture of upper limb

229	S52516Q	Fracture of upper limb
229	S52516R	Fracture of upper limb
229	S52516S	Fracture of upper limb
229	S52521A	Fracture of upper limb
229	S52521D	Fracture of upper limb
229	S52521G	Fracture of upper limb
229	S52521K	Fracture of upper limb
229	S52521P	Fracture of upper limb
229	S52521S	Fracture of upper limb
229	S52522A	Fracture of upper limb
229	S52522D	Fracture of upper limb
229	S52522G	Fracture of upper limb
229	S52522K	Fracture of upper limb
229	S52522P	Fracture of upper limb
229	S52522S	Fracture of upper limb
229	S52529A	Fracture of upper limb
229	S52529D	Fracture of upper limb
229	S52529G	Fracture of upper limb
229	S52529K	Fracture of upper limb
229	S52529P	Fracture of upper limb
229	S52529S	Fracture of upper limb
229	S52531A	Fracture of upper limb
229	S52531B	Fracture of upper limb
229	S52531C	Fracture of upper limb
229	S52531D	Fracture of upper limb
229	S52531E	Fracture of upper limb
229	S52531F	Fracture of upper limb
229	S52531G	Fracture of upper limb
229	S52531H	Fracture of upper limb
229	S52531J	Fracture of upper limb
229	S52531K	Fracture of upper limb
229	S52531M	Fracture of upper limb
229	S52531N	Fracture of upper limb
229	S52531P	Fracture of upper limb
229	S52531Q	Fracture of upper limb
229	S52531R	Fracture of upper limb
229	S52531S	Fracture of upper limb
229	S52532A	Fracture of upper limb
229	S52532B	Fracture of upper limb
229	S52532C	Fracture of upper limb
229	S52532D	Fracture of upper limb
229	S52532E	Fracture of upper limb
229	S52532F	Fracture of upper limb
229	S52532G	Fracture of upper limb
229	S52532H	Fracture of upper limb
229	S52532J	Fracture of upper limb
229	S52532K	Fracture of upper limb
229	S52532M	Fracture of upper limb
229	S52532N	Fracture of upper limb
229	S52532P	Fracture of upper limb
229	S52532Q	Fracture of upper limb

229	S52532R	Fracture of upper limb
229	S52532S	Fracture of upper limb
229	S52539A	Fracture of upper limb
229	S52539B	Fracture of upper limb
229	S52539C	Fracture of upper limb
229	S52539D	Fracture of upper limb
229	S52539E	Fracture of upper limb
229	S52539F	Fracture of upper limb
229	S52539G	Fracture of upper limb
229	S52539H	Fracture of upper limb
229	S52539J	Fracture of upper limb
229	S52539K	Fracture of upper limb
229	S52539M	Fracture of upper limb
229	S52539N	Fracture of upper limb
229	S52539P	Fracture of upper limb
229	S52539Q	Fracture of upper limb
229	S52539R	Fracture of upper limb
229	S52539S	Fracture of upper limb
229	S52541A	Fracture of upper limb
229	S52541B	Fracture of upper limb
229	S52541C	Fracture of upper limb
229	S52541D	Fracture of upper limb
229	S52541E	Fracture of upper limb
229	S52541F	Fracture of upper limb
229	S52541G	Fracture of upper limb
229	S52541H	Fracture of upper limb
229	S52541J	Fracture of upper limb
229	S52541K	Fracture of upper limb
229	S52541M	Fracture of upper limb
229	S52541N	Fracture of upper limb
229	S52541P	Fracture of upper limb
229	S52541Q	Fracture of upper limb
229	S52541R	Fracture of upper limb
229	S52541S	Fracture of upper limb
229	S52542A	Fracture of upper limb
229	S52542B	Fracture of upper limb
229	S52542C	Fracture of upper limb
229	S52542D	Fracture of upper limb
229	S52542E	Fracture of upper limb
229	S52542F	Fracture of upper limb
229	S52542G	Fracture of upper limb
229	S52542H	Fracture of upper limb
229	S52542J	Fracture of upper limb
229	S52542K	Fracture of upper limb
229	S52542M	Fracture of upper limb
229	S52542N	Fracture of upper limb
229	S52542P	Fracture of upper limb
229	S52542Q	Fracture of upper limb
229	S52542R	Fracture of upper limb
229	S52542S	Fracture of upper limb
229	S52549A	Fracture of upper limb

229	S52549B	Fracture of upper limb
229	S52549C	Fracture of upper limb
229	S52549D	Fracture of upper limb
229	S52549E	Fracture of upper limb
229	S52549F	Fracture of upper limb
229	S52549G	Fracture of upper limb
229	S52549H	Fracture of upper limb
229	S52549J	Fracture of upper limb
229	S52549K	Fracture of upper limb
229	S52549M	Fracture of upper limb
229	S52549N	Fracture of upper limb
229	S52549P	Fracture of upper limb
229	S52549Q	Fracture of upper limb
229	S52549R	Fracture of upper limb
229	S52549S	Fracture of upper limb
229	S52551A	Fracture of upper limb
229	S52551B	Fracture of upper limb
229	S52551C	Fracture of upper limb
229	S52551D	Fracture of upper limb
229	S52551E	Fracture of upper limb
229	S52551F	Fracture of upper limb
229	S52551G	Fracture of upper limb
229	S52551H	Fracture of upper limb
229	S52551J	Fracture of upper limb
229	S52551K	Fracture of upper limb
229	S52551M	Fracture of upper limb
229	S52551N	Fracture of upper limb
229	S52551P	Fracture of upper limb
229	S52551Q	Fracture of upper limb
229	S52551R	Fracture of upper limb
229	S52551S	Fracture of upper limb
229	S52552A	Fracture of upper limb
229	S52552B	Fracture of upper limb
229	S52552C	Fracture of upper limb
229	S52552D	Fracture of upper limb
229	S52552E	Fracture of upper limb
229	S52552F	Fracture of upper limb
229	S52552G	Fracture of upper limb
229	S52552H	Fracture of upper limb
229	S52552J	Fracture of upper limb
229	S52552K	Fracture of upper limb
229	S52552M	Fracture of upper limb
229	S52552N	Fracture of upper limb
229	S52552P	Fracture of upper limb
229	S52552Q	Fracture of upper limb
229	S52552R	Fracture of upper limb
229	S52552S	Fracture of upper limb
229	S52559A	Fracture of upper limb
229	S52559B	Fracture of upper limb
229	S52559C	Fracture of upper limb
229	S52559D	Fracture of upper limb

229	S52559E	Fracture of upper limb
229	S52559F	Fracture of upper limb
229	S52559G	Fracture of upper limb
229	S52559H	Fracture of upper limb
229	S52559J	Fracture of upper limb
229	S52559K	Fracture of upper limb
229	S52559M	Fracture of upper limb
229	S52559N	Fracture of upper limb
229	S52559P	Fracture of upper limb
229	S52559Q	Fracture of upper limb
229	S52559R	Fracture of upper limb
229	S52559S	Fracture of upper limb
229	S52561A	Fracture of upper limb
229	S52561B	Fracture of upper limb
229	S52561C	Fracture of upper limb
229	S52561D	Fracture of upper limb
229	S52561E	Fracture of upper limb
229	S52561F	Fracture of upper limb
229	S52561G	Fracture of upper limb
229	S52561H	Fracture of upper limb
229	S52561J	Fracture of upper limb
229	S52561K	Fracture of upper limb
229	S52561M	Fracture of upper limb
229	S52561N	Fracture of upper limb
229	S52561P	Fracture of upper limb
229	S52561Q	Fracture of upper limb
229	S52561R	Fracture of upper limb
229	S52561S	Fracture of upper limb
229	S52562A	Fracture of upper limb
229	S52562B	Fracture of upper limb
229	S52562C	Fracture of upper limb
229	S52562D	Fracture of upper limb
229	S52562E	Fracture of upper limb
229	S52562F	Fracture of upper limb
229	S52562G	Fracture of upper limb
229	S52562H	Fracture of upper limb
229	S52562J	Fracture of upper limb
229	S52562K	Fracture of upper limb
229	S52562M	Fracture of upper limb
229	S52562N	Fracture of upper limb
229	S52562P	Fracture of upper limb
229	S52562Q	Fracture of upper limb
229	S52562R	Fracture of upper limb
229	S52562S	Fracture of upper limb
229	S52569A	Fracture of upper limb
229	S52569B	Fracture of upper limb
229	S52569C	Fracture of upper limb
229	S52569D	Fracture of upper limb
229	S52569E	Fracture of upper limb
229	S52569F	Fracture of upper limb
229	S52569G	Fracture of upper limb

229	S52569H	Fracture of upper limb
229	S52569J	Fracture of upper limb
229	S52569K	Fracture of upper limb
229	S52569M	Fracture of upper limb
229	S52569N	Fracture of upper limb
229	S52569P	Fracture of upper limb
229	S52569Q	Fracture of upper limb
229	S52569R	Fracture of upper limb
229	S52569S	Fracture of upper limb
229	S52571A	Fracture of upper limb
229	S52571B	Fracture of upper limb
229	S52571C	Fracture of upper limb
229	S52571D	Fracture of upper limb
229	S52571E	Fracture of upper limb
229	S52571F	Fracture of upper limb
229	S52571G	Fracture of upper limb
229	S52571H	Fracture of upper limb
229	S52571J	Fracture of upper limb
229	S52571K	Fracture of upper limb
229	S52571M	Fracture of upper limb
229	S52571N	Fracture of upper limb
229	S52571P	Fracture of upper limb
229	S52571Q	Fracture of upper limb
229	S52571R	Fracture of upper limb
229	S52571S	Fracture of upper limb
229	S52572A	Fracture of upper limb
229	S52572B	Fracture of upper limb
229	S52572C	Fracture of upper limb
229	S52572D	Fracture of upper limb
229	S52572E	Fracture of upper limb
229	S52572F	Fracture of upper limb
229	S52572G	Fracture of upper limb
229	S52572H	Fracture of upper limb
229	S52572J	Fracture of upper limb
229	S52572K	Fracture of upper limb
229	S52572M	Fracture of upper limb
229	S52572N	Fracture of upper limb
229	S52572P	Fracture of upper limb
229	S52572Q	Fracture of upper limb
229	S52572R	Fracture of upper limb
229	S52572S	Fracture of upper limb
229	S52579A	Fracture of upper limb
229	S52579B	Fracture of upper limb
229	S52579C	Fracture of upper limb
229	S52579D	Fracture of upper limb
229	S52579E	Fracture of upper limb
229	S52579F	Fracture of upper limb
229	S52579G	Fracture of upper limb
229	S52579H	Fracture of upper limb
229	S52579J	Fracture of upper limb
229	S52579K	Fracture of upper limb

229	S52579M	Fracture of upper limb
229	S52579N	Fracture of upper limb
229	S52579P	Fracture of upper limb
229	S52579Q	Fracture of upper limb
229	S52579R	Fracture of upper limb
229	S52579S	Fracture of upper limb
229	S52591A	Fracture of upper limb
229	S52591B	Fracture of upper limb
229	S52591C	Fracture of upper limb
229	S52591D	Fracture of upper limb
229	S52591E	Fracture of upper limb
229	S52591F	Fracture of upper limb
229	S52591G	Fracture of upper limb
229	S52591H	Fracture of upper limb
229	S52591J	Fracture of upper limb
229	S52591K	Fracture of upper limb
229	S52591M	Fracture of upper limb
229	S52591N	Fracture of upper limb
229	S52591P	Fracture of upper limb
229	S52591Q	Fracture of upper limb
229	S52591R	Fracture of upper limb
229	S52591S	Fracture of upper limb
229	S52592A	Fracture of upper limb
229	S52592B	Fracture of upper limb
229	S52592C	Fracture of upper limb
229	S52592D	Fracture of upper limb
229	S52592E	Fracture of upper limb
229	S52592F	Fracture of upper limb
229	S52592G	Fracture of upper limb
229	S52592H	Fracture of upper limb
229	S52592J	Fracture of upper limb
229	S52592K	Fracture of upper limb
229	S52592M	Fracture of upper limb
229	S52592N	Fracture of upper limb
229	S52592P	Fracture of upper limb
229	S52592Q	Fracture of upper limb
229	S52592R	Fracture of upper limb
229	S52592S	Fracture of upper limb
229	S52599A	Fracture of upper limb
229	S52599B	Fracture of upper limb
229	S52599C	Fracture of upper limb
229	S52599D	Fracture of upper limb
229	S52599E	Fracture of upper limb
229	S52599F	Fracture of upper limb
229	S52599G	Fracture of upper limb
229	S52599H	Fracture of upper limb
229	S52599J	Fracture of upper limb
229	S52599K	Fracture of upper limb
229	S52599M	Fracture of upper limb
229	S52599N	Fracture of upper limb
229	S52599P	Fracture of upper limb

229	S52599Q	Fracture of upper limb
229	S52599R	Fracture of upper limb
229	S52599S	Fracture of upper limb
229	S52601A	Fracture of upper limb
229	S52601B	Fracture of upper limb
229	S52601C	Fracture of upper limb
229	S52601D	Fracture of upper limb
229	S52601E	Fracture of upper limb
229	S52601F	Fracture of upper limb
229	S52601G	Fracture of upper limb
229	S52601H	Fracture of upper limb
229	S52601J	Fracture of upper limb
229	S52601K	Fracture of upper limb
229	S52601M	Fracture of upper limb
229	S52601N	Fracture of upper limb
229	S52601P	Fracture of upper limb
229	S52601Q	Fracture of upper limb
229	S52601R	Fracture of upper limb
229	S52601S	Fracture of upper limb
229	S52602A	Fracture of upper limb
229	S52602B	Fracture of upper limb
229	S52602C	Fracture of upper limb
229	S52602D	Fracture of upper limb
229	S52602E	Fracture of upper limb
229	S52602F	Fracture of upper limb
229	S52602G	Fracture of upper limb
229	S52602H	Fracture of upper limb
229	S52602J	Fracture of upper limb
229	S52602K	Fracture of upper limb
229	S52602M	Fracture of upper limb
229	S52602N	Fracture of upper limb
229	S52602P	Fracture of upper limb
229	S52602Q	Fracture of upper limb
229	S52602R	Fracture of upper limb
229	S52602S	Fracture of upper limb
229	S52609A	Fracture of upper limb
229	S52609B	Fracture of upper limb
229	S52609C	Fracture of upper limb
229	S52609D	Fracture of upper limb
229	S52609E	Fracture of upper limb
229	S52609F	Fracture of upper limb
229	S52609G	Fracture of upper limb
229	S52609H	Fracture of upper limb
229	S52609J	Fracture of upper limb
229	S52609K	Fracture of upper limb
229	S52609M	Fracture of upper limb
229	S52609N	Fracture of upper limb
229	S52609P	Fracture of upper limb
229	S52609Q	Fracture of upper limb
229	S52609R	Fracture of upper limb
229	S52609S	Fracture of upper limb

229	S52611A	Fracture of upper limb
229	S52611B	Fracture of upper limb
229	S52611C	Fracture of upper limb
229	S52611D	Fracture of upper limb
229	S52611E	Fracture of upper limb
229	S52611F	Fracture of upper limb
229	S52611G	Fracture of upper limb
229	S52611H	Fracture of upper limb
229	S52611J	Fracture of upper limb
229	S52611K	Fracture of upper limb
229	S52611M	Fracture of upper limb
229	S52611N	Fracture of upper limb
229	S52611P	Fracture of upper limb
229	S52611Q	Fracture of upper limb
229	S52611R	Fracture of upper limb
229	S52611S	Fracture of upper limb
229	S52612A	Fracture of upper limb
229	S52612B	Fracture of upper limb
229	S52612C	Fracture of upper limb
229	S52612D	Fracture of upper limb
229	S52612E	Fracture of upper limb
229	S52612F	Fracture of upper limb
229	S52612G	Fracture of upper limb
229	S52612H	Fracture of upper limb
229	S52612J	Fracture of upper limb
229	S52612K	Fracture of upper limb
229	S52612M	Fracture of upper limb
229	S52612N	Fracture of upper limb
229	S52612P	Fracture of upper limb
229	S52612Q	Fracture of upper limb
229	S52612R	Fracture of upper limb
229	S52612S	Fracture of upper limb
229	S52613A	Fracture of upper limb
229	S52613B	Fracture of upper limb
229	S52613C	Fracture of upper limb
229	S52613D	Fracture of upper limb
229	S52613E	Fracture of upper limb
229	S52613F	Fracture of upper limb
229	S52613G	Fracture of upper limb
229	S52613H	Fracture of upper limb
229	S52613J	Fracture of upper limb
229	S52613K	Fracture of upper limb
229	S52613M	Fracture of upper limb
229	S52613N	Fracture of upper limb
229	S52613P	Fracture of upper limb
229	S52613Q	Fracture of upper limb
229	S52613R	Fracture of upper limb
229	S52613S	Fracture of upper limb
229	S52614A	Fracture of upper limb
229	S52614B	Fracture of upper limb
229	S52614C	Fracture of upper limb

229	S52614D	Fracture of upper limb
229	S52614E	Fracture of upper limb
229	S52614F	Fracture of upper limb
229	S52614G	Fracture of upper limb
229	S52614H	Fracture of upper limb
229	S52614J	Fracture of upper limb
229	S52614K	Fracture of upper limb
229	S52614M	Fracture of upper limb
229	S52614N	Fracture of upper limb
229	S52614P	Fracture of upper limb
229	S52614Q	Fracture of upper limb
229	S52614R	Fracture of upper limb
229	S52614S	Fracture of upper limb
229	S52615A	Fracture of upper limb
229	S52615B	Fracture of upper limb
229	S52615C	Fracture of upper limb
229	S52615D	Fracture of upper limb
229	S52615E	Fracture of upper limb
229	S52615F	Fracture of upper limb
229	S52615G	Fracture of upper limb
229	S52615H	Fracture of upper limb
229	S52615J	Fracture of upper limb
229	S52615K	Fracture of upper limb
229	S52615M	Fracture of upper limb
229	S52615N	Fracture of upper limb
229	S52615P	Fracture of upper limb
229	S52615Q	Fracture of upper limb
229	S52615R	Fracture of upper limb
229	S52615S	Fracture of upper limb
229	S52616A	Fracture of upper limb
229	S52616B	Fracture of upper limb
229	S52616C	Fracture of upper limb
229	S52616D	Fracture of upper limb
229	S52616E	Fracture of upper limb
229	S52616F	Fracture of upper limb
229	S52616G	Fracture of upper limb
229	S52616H	Fracture of upper limb
229	S52616J	Fracture of upper limb
229	S52616K	Fracture of upper limb
229	S52616M	Fracture of upper limb
229	S52616N	Fracture of upper limb
229	S52616P	Fracture of upper limb
229	S52616Q	Fracture of upper limb
229	S52616R	Fracture of upper limb
229	S52616S	Fracture of upper limb
229	S52621A	Fracture of upper limb
229	S52621D	Fracture of upper limb
229	S52621G	Fracture of upper limb
229	S52621K	Fracture of upper limb
229	S52621P	Fracture of upper limb
229	S52621S	Fracture of upper limb

229	S52622A	Fracture of upper limb
229	S52622D	Fracture of upper limb
229	S52622G	Fracture of upper limb
229	S52622K	Fracture of upper limb
229	S52622P	Fracture of upper limb
229	S52622S	Fracture of upper limb
229	S52629A	Fracture of upper limb
229	S52629D	Fracture of upper limb
229	S52629G	Fracture of upper limb
229	S52629K	Fracture of upper limb
229	S52629P	Fracture of upper limb
229	S52629S	Fracture of upper limb
229	S52691A	Fracture of upper limb
229	S52691B	Fracture of upper limb
229	S52691C	Fracture of upper limb
229	S52691D	Fracture of upper limb
229	S52691E	Fracture of upper limb
229	S52691F	Fracture of upper limb
229	S52691G	Fracture of upper limb
229	S52691H	Fracture of upper limb
229	S52691J	Fracture of upper limb
229	S52691K	Fracture of upper limb
229	S52691M	Fracture of upper limb
229	S52691N	Fracture of upper limb
229	S52691P	Fracture of upper limb
229	S52691Q	Fracture of upper limb
229	S52691R	Fracture of upper limb
229	S52691S	Fracture of upper limb
229	S52692A	Fracture of upper limb
229	S52692B	Fracture of upper limb
229	S52692C	Fracture of upper limb
229	S52692D	Fracture of upper limb
229	S52692E	Fracture of upper limb
229	S52692F	Fracture of upper limb
229	S52692G	Fracture of upper limb
229	S52692H	Fracture of upper limb
229	S52692J	Fracture of upper limb
229	S52692K	Fracture of upper limb
229	S52692M	Fracture of upper limb
229	S52692N	Fracture of upper limb
229	S52692P	Fracture of upper limb
229	S52692Q	Fracture of upper limb
229	S52692R	Fracture of upper limb
229	S52692S	Fracture of upper limb
229	S52699A	Fracture of upper limb
229	S52699B	Fracture of upper limb
229	S52699C	Fracture of upper limb
229	S52699D	Fracture of upper limb
229	S52699E	Fracture of upper limb
229	S52699F	Fracture of upper limb
229	S52699G	Fracture of upper limb

229	S52699H	Fracture of upper limb
229	S52699J	Fracture of upper limb
229	S52699K	Fracture of upper limb
229	S52699M	Fracture of upper limb
229	S52699N	Fracture of upper limb
229	S52699P	Fracture of upper limb
229	S52699Q	Fracture of upper limb
229	S52699R	Fracture of upper limb
229	S52699S	Fracture of upper limb
229	S5290XA	Fracture of upper limb
229	S5290XB	Fracture of upper limb
229	S5290XC	Fracture of upper limb
229	S5290XD	Fracture of upper limb
229	S5290XE	Fracture of upper limb
229	S5290XF	Fracture of upper limb
229	S5290XG	Fracture of upper limb
229	S5290XH	Fracture of upper limb
229	S5290XJ	Fracture of upper limb
229	S5290XK	Fracture of upper limb
229	S5290XM	Fracture of upper limb
229	S5290XN	Fracture of upper limb
229	S5290XP	Fracture of upper limb
229	S5290XQ	Fracture of upper limb
229	S5290XR	Fracture of upper limb
229	S5290XS	Fracture of upper limb
229	S5291XA	Fracture of upper limb
229	S5291XB	Fracture of upper limb
229	S5291XC	Fracture of upper limb
229	S5291XD	Fracture of upper limb
229	S5291XE	Fracture of upper limb
229	S5291XF	Fracture of upper limb
229	S5291XG	Fracture of upper limb
229	S5291XH	Fracture of upper limb
229	S5291XJ	Fracture of upper limb
229	S5291XK	Fracture of upper limb
229	S5291XM	Fracture of upper limb
229	S5291XN	Fracture of upper limb
229	S5291XP	Fracture of upper limb
229	S5291XQ	Fracture of upper limb
229	S5291XR	Fracture of upper limb
229	S5291XS	Fracture of upper limb
229	S5292XA	Fracture of upper limb
229	S5292XB	Fracture of upper limb
229	S5292XC	Fracture of upper limb
229	S5292XD	Fracture of upper limb
229	S5292XE	Fracture of upper limb
229	S5292XF	Fracture of upper limb
229	S5292XG	Fracture of upper limb
229	S5292XH	Fracture of upper limb
229	S5292XJ	Fracture of upper limb
229	S5292XK	Fracture of upper limb

229	S5292XM	Fracture of upper limb
229	S5292XN	Fracture of upper limb
229	S5292XP	Fracture of upper limb
229	S5292XQ	Fracture of upper limb
229	S5292XR	Fracture of upper limb
229	S5292XS	Fracture of upper limb
229	S59001A	Fracture of upper limb
229	S59001D	Fracture of upper limb
229	S59001G	Fracture of upper limb
229	S59001K	Fracture of upper limb
229	S59001P	Fracture of upper limb
229	S59001S	Fracture of upper limb
229	S59002A	Fracture of upper limb
229	S59002D	Fracture of upper limb
229	S59002G	Fracture of upper limb
229	S59002K	Fracture of upper limb
229	S59002P	Fracture of upper limb
229	S59002S	Fracture of upper limb
229	S59009A	Fracture of upper limb
229	S59009D	Fracture of upper limb
229	S59009G	Fracture of upper limb
229	S59009K	Fracture of upper limb
229	S59009P	Fracture of upper limb
229	S59009S	Fracture of upper limb
229	S59011A	Fracture of upper limb
229	S59011D	Fracture of upper limb
229	S59011G	Fracture of upper limb
229	S59011K	Fracture of upper limb
229	S59011P	Fracture of upper limb
229	S59011S	Fracture of upper limb
229	S59012A	Fracture of upper limb
229	S59012D	Fracture of upper limb
229	S59012G	Fracture of upper limb
229	S59012K	Fracture of upper limb
229	S59012P	Fracture of upper limb
229	S59012S	Fracture of upper limb
229	S59019A	Fracture of upper limb
229	S59019D	Fracture of upper limb
229	S59019G	Fracture of upper limb
229	S59019K	Fracture of upper limb
229	S59019P	Fracture of upper limb
229	S59019S	Fracture of upper limb
229	S59021A	Fracture of upper limb
229	S59021D	Fracture of upper limb
229	S59021G	Fracture of upper limb
229	S59021K	Fracture of upper limb
229	S59021P	Fracture of upper limb
229	S59021S	Fracture of upper limb
229	S59022A	Fracture of upper limb
229	S59022D	Fracture of upper limb
229	S59022G	Fracture of upper limb

229	S59022K	Fracture of upper limb
229	S59022P	Fracture of upper limb
229	S59022S	Fracture of upper limb
229	S59029A	Fracture of upper limb
229	S59029D	Fracture of upper limb
229	S59029G	Fracture of upper limb
229	S59029K	Fracture of upper limb
229	S59029P	Fracture of upper limb
229	S59029S	Fracture of upper limb
229	S59031A	Fracture of upper limb
229	S59031D	Fracture of upper limb
229	S59031G	Fracture of upper limb
229	S59031K	Fracture of upper limb
229	S59031P	Fracture of upper limb
229	S59031S	Fracture of upper limb
229	S59032A	Fracture of upper limb
229	S59032D	Fracture of upper limb
229	S59032G	Fracture of upper limb
229	S59032K	Fracture of upper limb
229	S59032P	Fracture of upper limb
229	S59032S	Fracture of upper limb
229	S59039A	Fracture of upper limb
229	S59039D	Fracture of upper limb
229	S59039G	Fracture of upper limb
229	S59039K	Fracture of upper limb
229	S59039P	Fracture of upper limb
229	S59039S	Fracture of upper limb
229	S59041A	Fracture of upper limb
229	S59041D	Fracture of upper limb
229	S59041G	Fracture of upper limb
229	S59041K	Fracture of upper limb
229	S59041P	Fracture of upper limb
229	S59041S	Fracture of upper limb
229	S59042A	Fracture of upper limb
229	S59042D	Fracture of upper limb
229	S59042G	Fracture of upper limb
229	S59042K	Fracture of upper limb
229	S59042P	Fracture of upper limb
229	S59042S	Fracture of upper limb
229	S59049A	Fracture of upper limb
229	S59049D	Fracture of upper limb
229	S59049G	Fracture of upper limb
229	S59049K	Fracture of upper limb
229	S59049P	Fracture of upper limb
229	S59049S	Fracture of upper limb
229	S59091A	Fracture of upper limb
229	S59091D	Fracture of upper limb
229	S59091G	Fracture of upper limb
229	S59091K	Fracture of upper limb
229	S59091P	Fracture of upper limb
229	S59091S	Fracture of upper limb

229	S59092A	Fracture of upper limb
229	S59092D	Fracture of upper limb
229	S59092G	Fracture of upper limb
229	S59092K	Fracture of upper limb
229	S59092P	Fracture of upper limb
229	S59092S	Fracture of upper limb
229	S59099A	Fracture of upper limb
229	S59099D	Fracture of upper limb
229	S59099G	Fracture of upper limb
229	S59099K	Fracture of upper limb
229	S59099P	Fracture of upper limb
229	S59099S	Fracture of upper limb
229	S59101A	Fracture of upper limb
229	S59101D	Fracture of upper limb
229	S59101G	Fracture of upper limb
229	S59101K	Fracture of upper limb
229	S59101P	Fracture of upper limb
229	S59101S	Fracture of upper limb
229	S59102A	Fracture of upper limb
229	S59102D	Fracture of upper limb
229	S59102G	Fracture of upper limb
229	S59102K	Fracture of upper limb
229	S59102P	Fracture of upper limb
229	S59102S	Fracture of upper limb
229	S59109A	Fracture of upper limb
229	S59109D	Fracture of upper limb
229	S59109G	Fracture of upper limb
229	S59109K	Fracture of upper limb
229	S59109P	Fracture of upper limb
229	S59109S	Fracture of upper limb
229	S59111A	Fracture of upper limb
229	S59111D	Fracture of upper limb
229	S59111G	Fracture of upper limb
229	S59111K	Fracture of upper limb
229	S59111P	Fracture of upper limb
229	S59111S	Fracture of upper limb
229	S59112A	Fracture of upper limb
229	S59112D	Fracture of upper limb
229	S59112G	Fracture of upper limb
229	S59112K	Fracture of upper limb
229	S59112P	Fracture of upper limb
229	S59112S	Fracture of upper limb
229	S59119A	Fracture of upper limb
229	S59119D	Fracture of upper limb
229	S59119G	Fracture of upper limb
229	S59119K	Fracture of upper limb
229	S59119P	Fracture of upper limb
229	S59119S	Fracture of upper limb
229	S59121A	Fracture of upper limb
229	S59121D	Fracture of upper limb
229	S59121G	Fracture of upper limb

229	S59121K	Fracture of upper limb
229	S59121P	Fracture of upper limb
229	S59121S	Fracture of upper limb
229	S59122A	Fracture of upper limb
229	S59122D	Fracture of upper limb
229	S59122G	Fracture of upper limb
229	S59122K	Fracture of upper limb
229	S59122P	Fracture of upper limb
229	S59122S	Fracture of upper limb
229	S59129A	Fracture of upper limb
229	S59129D	Fracture of upper limb
229	S59129G	Fracture of upper limb
229	S59129K	Fracture of upper limb
229	S59129P	Fracture of upper limb
229	S59129S	Fracture of upper limb
229	S59131A	Fracture of upper limb
229	S59131D	Fracture of upper limb
229	S59131G	Fracture of upper limb
229	S59131K	Fracture of upper limb
229	S59131P	Fracture of upper limb
229	S59131S	Fracture of upper limb
229	S59132A	Fracture of upper limb
229	S59132D	Fracture of upper limb
229	S59132G	Fracture of upper limb
229	S59132K	Fracture of upper limb
229	S59132P	Fracture of upper limb
229	S59132S	Fracture of upper limb
229	S59139A	Fracture of upper limb
229	S59139D	Fracture of upper limb
229	S59139G	Fracture of upper limb
229	S59139K	Fracture of upper limb
229	S59139P	Fracture of upper limb
229	S59139S	Fracture of upper limb
229	S59141A	Fracture of upper limb
229	S59141D	Fracture of upper limb
229	S59141G	Fracture of upper limb
229	S59141K	Fracture of upper limb
229	S59141P	Fracture of upper limb
229	S59141S	Fracture of upper limb
229	S59142A	Fracture of upper limb
229	S59142D	Fracture of upper limb
229	S59142G	Fracture of upper limb
229	S59142K	Fracture of upper limb
229	S59142P	Fracture of upper limb
229	S59142S	Fracture of upper limb
229	S59149A	Fracture of upper limb
229	S59149D	Fracture of upper limb
229	S59149G	Fracture of upper limb
229	S59149K	Fracture of upper limb
229	S59149P	Fracture of upper limb
229	S59149S	Fracture of upper limb

229	S59191A	Fracture of upper limb
229	S59191D	Fracture of upper limb
229	S59191G	Fracture of upper limb
229	S59191K	Fracture of upper limb
229	S59191P	Fracture of upper limb
229	S59191S	Fracture of upper limb
229	S59192A	Fracture of upper limb
229	S59192D	Fracture of upper limb
229	S59192G	Fracture of upper limb
229	S59192K	Fracture of upper limb
229	S59192P	Fracture of upper limb
229	S59192S	Fracture of upper limb
229	S59199A	Fracture of upper limb
229	S59199D	Fracture of upper limb
229	S59199G	Fracture of upper limb
229	S59199K	Fracture of upper limb
229	S59199P	Fracture of upper limb
229	S59199S	Fracture of upper limb
229	S59201A	Fracture of upper limb
229	S59201D	Fracture of upper limb
229	S59201G	Fracture of upper limb
229	S59201K	Fracture of upper limb
229	S59201P	Fracture of upper limb
229	S59201S	Fracture of upper limb
229	S59202A	Fracture of upper limb
229	S59202D	Fracture of upper limb
229	S59202G	Fracture of upper limb
229	S59202K	Fracture of upper limb
229	S59202P	Fracture of upper limb
229	S59202S	Fracture of upper limb
229	S59209A	Fracture of upper limb
229	S59209D	Fracture of upper limb
229	S59209G	Fracture of upper limb
229	S59209K	Fracture of upper limb
229	S59209P	Fracture of upper limb
229	S59209S	Fracture of upper limb
229	S59211A	Fracture of upper limb
229	S59211D	Fracture of upper limb
229	S59211G	Fracture of upper limb
229	S59211K	Fracture of upper limb
229	S59211P	Fracture of upper limb
229	S59211S	Fracture of upper limb
229	S59212A	Fracture of upper limb
229	S59212D	Fracture of upper limb
229	S59212G	Fracture of upper limb
229	S59212K	Fracture of upper limb
229	S59212P	Fracture of upper limb
229	S59212S	Fracture of upper limb
229	S59219A	Fracture of upper limb
229	S59219D	Fracture of upper limb
229	S59219G	Fracture of upper limb

229	S59219K	Fracture of upper limb
229	S59219P	Fracture of upper limb
229	S59219S	Fracture of upper limb
229	S59221A	Fracture of upper limb
229	S59221D	Fracture of upper limb
229	S59221G	Fracture of upper limb
229	S59221K	Fracture of upper limb
229	S59221P	Fracture of upper limb
229	S59221S	Fracture of upper limb
229	S59222A	Fracture of upper limb
229	S59222D	Fracture of upper limb
229	S59222G	Fracture of upper limb
229	S59222K	Fracture of upper limb
229	S59222P	Fracture of upper limb
229	S59222S	Fracture of upper limb
229	S59229A	Fracture of upper limb
229	S59229D	Fracture of upper limb
229	S59229G	Fracture of upper limb
229	S59229K	Fracture of upper limb
229	S59229P	Fracture of upper limb
229	S59229S	Fracture of upper limb
229	S59231A	Fracture of upper limb
229	S59231D	Fracture of upper limb
229	S59231G	Fracture of upper limb
229	S59231K	Fracture of upper limb
229	S59231P	Fracture of upper limb
229	S59231S	Fracture of upper limb
229	S59232A	Fracture of upper limb
229	S59232D	Fracture of upper limb
229	S59232G	Fracture of upper limb
229	S59232K	Fracture of upper limb
229	S59232P	Fracture of upper limb
229	S59232S	Fracture of upper limb
229	S59239A	Fracture of upper limb
229	S59239D	Fracture of upper limb
229	S59239G	Fracture of upper limb
229	S59239K	Fracture of upper limb
229	S59239P	Fracture of upper limb
229	S59239S	Fracture of upper limb
229	S59241A	Fracture of upper limb
229	S59241D	Fracture of upper limb
229	S59241G	Fracture of upper limb
229	S59241K	Fracture of upper limb
229	S59241P	Fracture of upper limb
229	S59241S	Fracture of upper limb
229	S59242A	Fracture of upper limb
229	S59242D	Fracture of upper limb
229	S59242G	Fracture of upper limb
229	S59242K	Fracture of upper limb
229	S59242P	Fracture of upper limb
229	S59242S	Fracture of upper limb

229	S59249A	Fracture of upper limb
229	S59249D	Fracture of upper limb
229	S59249G	Fracture of upper limb
229	S59249K	Fracture of upper limb
229	S59249P	Fracture of upper limb
229	S59249S	Fracture of upper limb
229	S59291A	Fracture of upper limb
229	S59291D	Fracture of upper limb
229	S59291G	Fracture of upper limb
229	S59291K	Fracture of upper limb
229	S59291P	Fracture of upper limb
229	S59291S	Fracture of upper limb
229	S59292A	Fracture of upper limb
229	S59292D	Fracture of upper limb
229	S59292G	Fracture of upper limb
229	S59292K	Fracture of upper limb
229	S59292P	Fracture of upper limb
229	S59292S	Fracture of upper limb
229	S59299A	Fracture of upper limb
229	S59299D	Fracture of upper limb
229	S59299G	Fracture of upper limb
229	S59299K	Fracture of upper limb
229	S59299P	Fracture of upper limb
229	S59299S	Fracture of upper limb
229	S62001A	Fracture of upper limb
229	S62001B	Fracture of upper limb
229	S62001D	Fracture of upper limb
229	S62001G	Fracture of upper limb
229	S62001K	Fracture of upper limb
229	S62001P	Fracture of upper limb
229	S62001S	Fracture of upper limb
229	S62002A	Fracture of upper limb
229	S62002B	Fracture of upper limb
229	S62002D	Fracture of upper limb
229	S62002G	Fracture of upper limb
229	S62002K	Fracture of upper limb
229	S62002P	Fracture of upper limb
229	S62002S	Fracture of upper limb
229	S62009A	Fracture of upper limb
229	S62009B	Fracture of upper limb
229	S62009D	Fracture of upper limb
229	S62009G	Fracture of upper limb
229	S62009K	Fracture of upper limb
229	S62009P	Fracture of upper limb
229	S62009S	Fracture of upper limb
229	S62011A	Fracture of upper limb
229	S62011B	Fracture of upper limb
229	S62011D	Fracture of upper limb
229	S62011G	Fracture of upper limb
229	S62011K	Fracture of upper limb
229	S62011P	Fracture of upper limb

229	S62011S	Fracture of upper limb
229	S62012A	Fracture of upper limb
229	S62012B	Fracture of upper limb
229	S62012D	Fracture of upper limb
229	S62012G	Fracture of upper limb
229	S62012K	Fracture of upper limb
229	S62012P	Fracture of upper limb
229	S62012S	Fracture of upper limb
229	S62013A	Fracture of upper limb
229	S62013B	Fracture of upper limb
229	S62013D	Fracture of upper limb
229	S62013G	Fracture of upper limb
229	S62013K	Fracture of upper limb
229	S62013P	Fracture of upper limb
229	S62013S	Fracture of upper limb
229	S62014A	Fracture of upper limb
229	S62014B	Fracture of upper limb
229	S62014D	Fracture of upper limb
229	S62014G	Fracture of upper limb
229	S62014K	Fracture of upper limb
229	S62014P	Fracture of upper limb
229	S62014S	Fracture of upper limb
229	S62015A	Fracture of upper limb
229	S62015B	Fracture of upper limb
229	S62015D	Fracture of upper limb
229	S62015G	Fracture of upper limb
229	S62015K	Fracture of upper limb
229	S62015P	Fracture of upper limb
229	S62015S	Fracture of upper limb
229	S62016A	Fracture of upper limb
229	S62016B	Fracture of upper limb
229	S62016D	Fracture of upper limb
229	S62016G	Fracture of upper limb
229	S62016K	Fracture of upper limb
229	S62016P	Fracture of upper limb
229	S62016S	Fracture of upper limb
229	S62021A	Fracture of upper limb
229	S62021B	Fracture of upper limb
229	S62021D	Fracture of upper limb
229	S62021G	Fracture of upper limb
229	S62021K	Fracture of upper limb
229	S62021P	Fracture of upper limb
229	S62021S	Fracture of upper limb
229	S62022A	Fracture of upper limb
229	S62022B	Fracture of upper limb
229	S62022D	Fracture of upper limb
229	S62022G	Fracture of upper limb
229	S62022K	Fracture of upper limb
229	S62022P	Fracture of upper limb
229	S62022S	Fracture of upper limb
229	S62023A	Fracture of upper limb

229	S62023B	Fracture of upper limb
229	S62023D	Fracture of upper limb
229	S62023G	Fracture of upper limb
229	S62023K	Fracture of upper limb
229	S62023P	Fracture of upper limb
229	S62023S	Fracture of upper limb
229	S62024A	Fracture of upper limb
229	S62024B	Fracture of upper limb
229	S62024D	Fracture of upper limb
229	S62024G	Fracture of upper limb
229	S62024K	Fracture of upper limb
229	S62024P	Fracture of upper limb
229	S62024S	Fracture of upper limb
229	S62025A	Fracture of upper limb
229	S62025B	Fracture of upper limb
229	S62025D	Fracture of upper limb
229	S62025G	Fracture of upper limb
229	S62025K	Fracture of upper limb
229	S62025P	Fracture of upper limb
229	S62025S	Fracture of upper limb
229	S62026A	Fracture of upper limb
229	S62026B	Fracture of upper limb
229	S62026D	Fracture of upper limb
229	S62026G	Fracture of upper limb
229	S62026K	Fracture of upper limb
229	S62026P	Fracture of upper limb
229	S62026S	Fracture of upper limb
229	S62031A	Fracture of upper limb
229	S62031B	Fracture of upper limb
229	S62031D	Fracture of upper limb
229	S62031G	Fracture of upper limb
229	S62031K	Fracture of upper limb
229	S62031P	Fracture of upper limb
229	S62031S	Fracture of upper limb
229	S62032A	Fracture of upper limb
229	S62032B	Fracture of upper limb
229	S62032D	Fracture of upper limb
229	S62032G	Fracture of upper limb
229	S62032K	Fracture of upper limb
229	S62032P	Fracture of upper limb
229	S62032S	Fracture of upper limb
229	S62033A	Fracture of upper limb
229	S62033B	Fracture of upper limb
229	S62033D	Fracture of upper limb
229	S62033G	Fracture of upper limb
229	S62033K	Fracture of upper limb
229	S62033P	Fracture of upper limb
229	S62033S	Fracture of upper limb
229	S62034A	Fracture of upper limb
229	S62034B	Fracture of upper limb
229	S62034D	Fracture of upper limb

229	S62034G	Fracture of upper limb
229	S62034K	Fracture of upper limb
229	S62034P	Fracture of upper limb
229	S62034S	Fracture of upper limb
229	S62035A	Fracture of upper limb
229	S62035B	Fracture of upper limb
229	S62035D	Fracture of upper limb
229	S62035G	Fracture of upper limb
229	S62035K	Fracture of upper limb
229	S62035P	Fracture of upper limb
229	S62035S	Fracture of upper limb
229	S62036A	Fracture of upper limb
229	S62036B	Fracture of upper limb
229	S62036D	Fracture of upper limb
229	S62036G	Fracture of upper limb
229	S62036K	Fracture of upper limb
229	S62036P	Fracture of upper limb
229	S62036S	Fracture of upper limb
229	S62101A	Fracture of upper limb
229	S62101B	Fracture of upper limb
229	S62101D	Fracture of upper limb
229	S62101G	Fracture of upper limb
229	S62101K	Fracture of upper limb
229	S62101P	Fracture of upper limb
229	S62101S	Fracture of upper limb
229	S62102A	Fracture of upper limb
229	S62102B	Fracture of upper limb
229	S62102D	Fracture of upper limb
229	S62102G	Fracture of upper limb
229	S62102K	Fracture of upper limb
229	S62102P	Fracture of upper limb
229	S62102S	Fracture of upper limb
229	S62109A	Fracture of upper limb
229	S62109B	Fracture of upper limb
229	S62109D	Fracture of upper limb
229	S62109G	Fracture of upper limb
229	S62109K	Fracture of upper limb
229	S62109P	Fracture of upper limb
229	S62109S	Fracture of upper limb
229	S62111A	Fracture of upper limb
229	S62111B	Fracture of upper limb
229	S62111D	Fracture of upper limb
229	S62111G	Fracture of upper limb
229	S62111K	Fracture of upper limb
229	S62111P	Fracture of upper limb
229	S62111S	Fracture of upper limb
229	S62112A	Fracture of upper limb
229	S62112B	Fracture of upper limb
229	S62112D	Fracture of upper limb
229	S62112G	Fracture of upper limb
229	S62112K	Fracture of upper limb

229	S62112P	Fracture of upper limb
229	S62112S	Fracture of upper limb
229	S62113A	Fracture of upper limb
229	S62113B	Fracture of upper limb
229	S62113D	Fracture of upper limb
229	S62113G	Fracture of upper limb
229	S62113K	Fracture of upper limb
229	S62113P	Fracture of upper limb
229	S62113S	Fracture of upper limb
229	S62114A	Fracture of upper limb
229	S62114B	Fracture of upper limb
229	S62114D	Fracture of upper limb
229	S62114G	Fracture of upper limb
229	S62114K	Fracture of upper limb
229	S62114P	Fracture of upper limb
229	S62114S	Fracture of upper limb
229	S62115A	Fracture of upper limb
229	S62115B	Fracture of upper limb
229	S62115D	Fracture of upper limb
229	S62115G	Fracture of upper limb
229	S62115K	Fracture of upper limb
229	S62115P	Fracture of upper limb
229	S62115S	Fracture of upper limb
229	S62116A	Fracture of upper limb
229	S62116B	Fracture of upper limb
229	S62116D	Fracture of upper limb
229	S62116G	Fracture of upper limb
229	S62116K	Fracture of upper limb
229	S62116P	Fracture of upper limb
229	S62116S	Fracture of upper limb
229	S62121A	Fracture of upper limb
229	S62121B	Fracture of upper limb
229	S62121D	Fracture of upper limb
229	S62121G	Fracture of upper limb
229	S62121K	Fracture of upper limb
229	S62121P	Fracture of upper limb
229	S62121S	Fracture of upper limb
229	S62122A	Fracture of upper limb
229	S62122B	Fracture of upper limb
229	S62122D	Fracture of upper limb
229	S62122G	Fracture of upper limb
229	S62122K	Fracture of upper limb
229	S62122P	Fracture of upper limb
229	S62122S	Fracture of upper limb
229	S62123A	Fracture of upper limb
229	S62123B	Fracture of upper limb
229	S62123D	Fracture of upper limb
229	S62123G	Fracture of upper limb
229	S62123K	Fracture of upper limb
229	S62123P	Fracture of upper limb
229	S62123S	Fracture of upper limb

229	S62124A	Fracture of upper limb
229	S62124B	Fracture of upper limb
229	S62124D	Fracture of upper limb
229	S62124G	Fracture of upper limb
229	S62124K	Fracture of upper limb
229	S62124P	Fracture of upper limb
229	S62124S	Fracture of upper limb
229	S62125A	Fracture of upper limb
229	S62125B	Fracture of upper limb
229	S62125D	Fracture of upper limb
229	S62125G	Fracture of upper limb
229	S62125K	Fracture of upper limb
229	S62125P	Fracture of upper limb
229	S62125S	Fracture of upper limb
229	S62126A	Fracture of upper limb
229	S62126B	Fracture of upper limb
229	S62126D	Fracture of upper limb
229	S62126G	Fracture of upper limb
229	S62126K	Fracture of upper limb
229	S62126P	Fracture of upper limb
229	S62126S	Fracture of upper limb
229	S62131A	Fracture of upper limb
229	S62131B	Fracture of upper limb
229	S62131D	Fracture of upper limb
229	S62131G	Fracture of upper limb
229	S62131K	Fracture of upper limb
229	S62131P	Fracture of upper limb
229	S62131S	Fracture of upper limb
229	S62132A	Fracture of upper limb
229	S62132B	Fracture of upper limb
229	S62132D	Fracture of upper limb
229	S62132G	Fracture of upper limb
229	S62132K	Fracture of upper limb
229	S62132P	Fracture of upper limb
229	S62132S	Fracture of upper limb
229	S62133A	Fracture of upper limb
229	S62133B	Fracture of upper limb
229	S62133D	Fracture of upper limb
229	S62133G	Fracture of upper limb
229	S62133K	Fracture of upper limb
229	S62133P	Fracture of upper limb
229	S62133S	Fracture of upper limb
229	S62134A	Fracture of upper limb
229	S62134B	Fracture of upper limb
229	S62134D	Fracture of upper limb
229	S62134G	Fracture of upper limb
229	S62134K	Fracture of upper limb
229	S62134P	Fracture of upper limb
229	S62134S	Fracture of upper limb
229	S62135A	Fracture of upper limb
229	S62135B	Fracture of upper limb

229	S62135D	Fracture of upper limb
229	S62135G	Fracture of upper limb
229	S62135K	Fracture of upper limb
229	S62135P	Fracture of upper limb
229	S62135S	Fracture of upper limb
229	S62136A	Fracture of upper limb
229	S62136B	Fracture of upper limb
229	S62136D	Fracture of upper limb
229	S62136G	Fracture of upper limb
229	S62136K	Fracture of upper limb
229	S62136P	Fracture of upper limb
229	S62136S	Fracture of upper limb
229	S62141A	Fracture of upper limb
229	S62141B	Fracture of upper limb
229	S62141D	Fracture of upper limb
229	S62141G	Fracture of upper limb
229	S62141K	Fracture of upper limb
229	S62141P	Fracture of upper limb
229	S62141S	Fracture of upper limb
229	S62142A	Fracture of upper limb
229	S62142B	Fracture of upper limb
229	S62142D	Fracture of upper limb
229	S62142G	Fracture of upper limb
229	S62142K	Fracture of upper limb
229	S62142P	Fracture of upper limb
229	S62142S	Fracture of upper limb
229	S62143A	Fracture of upper limb
229	S62143B	Fracture of upper limb
229	S62143D	Fracture of upper limb
229	S62143G	Fracture of upper limb
229	S62143K	Fracture of upper limb
229	S62143P	Fracture of upper limb
229	S62143S	Fracture of upper limb
229	S62144A	Fracture of upper limb
229	S62144B	Fracture of upper limb
229	S62144D	Fracture of upper limb
229	S62144G	Fracture of upper limb
229	S62144K	Fracture of upper limb
229	S62144P	Fracture of upper limb
229	S62144S	Fracture of upper limb
229	S62145A	Fracture of upper limb
229	S62145B	Fracture of upper limb
229	S62145D	Fracture of upper limb
229	S62145G	Fracture of upper limb
229	S62145K	Fracture of upper limb
229	S62145P	Fracture of upper limb
229	S62145S	Fracture of upper limb
229	S62146A	Fracture of upper limb
229	S62146B	Fracture of upper limb
229	S62146D	Fracture of upper limb
229	S62146G	Fracture of upper limb

229	S62146K	Fracture of upper limb
229	S62146P	Fracture of upper limb
229	S62146S	Fracture of upper limb
229	S62151A	Fracture of upper limb
229	S62151B	Fracture of upper limb
229	S62151D	Fracture of upper limb
229	S62151G	Fracture of upper limb
229	S62151K	Fracture of upper limb
229	S62151P	Fracture of upper limb
229	S62151S	Fracture of upper limb
229	S62152A	Fracture of upper limb
229	S62152B	Fracture of upper limb
229	S62152D	Fracture of upper limb
229	S62152G	Fracture of upper limb
229	S62152K	Fracture of upper limb
229	S62152P	Fracture of upper limb
229	S62152S	Fracture of upper limb
229	S62153A	Fracture of upper limb
229	S62153B	Fracture of upper limb
229	S62153D	Fracture of upper limb
229	S62153G	Fracture of upper limb
229	S62153K	Fracture of upper limb
229	S62153P	Fracture of upper limb
229	S62153S	Fracture of upper limb
229	S62154A	Fracture of upper limb
229	S62154B	Fracture of upper limb
229	S62154D	Fracture of upper limb
229	S62154G	Fracture of upper limb
229	S62154K	Fracture of upper limb
229	S62154P	Fracture of upper limb
229	S62154S	Fracture of upper limb
229	S62155A	Fracture of upper limb
229	S62155B	Fracture of upper limb
229	S62155D	Fracture of upper limb
229	S62155G	Fracture of upper limb
229	S62155K	Fracture of upper limb
229	S62155P	Fracture of upper limb
229	S62155S	Fracture of upper limb
229	S62156A	Fracture of upper limb
229	S62156B	Fracture of upper limb
229	S62156D	Fracture of upper limb
229	S62156G	Fracture of upper limb
229	S62156K	Fracture of upper limb
229	S62156P	Fracture of upper limb
229	S62156S	Fracture of upper limb
229	S62161A	Fracture of upper limb
229	S62161B	Fracture of upper limb
229	S62161D	Fracture of upper limb
229	S62161G	Fracture of upper limb
229	S62161K	Fracture of upper limb
229	S62161P	Fracture of upper limb

229	S62161S	Fracture of upper limb
229	S62162A	Fracture of upper limb
229	S62162B	Fracture of upper limb
229	S62162D	Fracture of upper limb
229	S62162G	Fracture of upper limb
229	S62162K	Fracture of upper limb
229	S62162P	Fracture of upper limb
229	S62162S	Fracture of upper limb
229	S62163A	Fracture of upper limb
229	S62163B	Fracture of upper limb
229	S62163D	Fracture of upper limb
229	S62163G	Fracture of upper limb
229	S62163K	Fracture of upper limb
229	S62163P	Fracture of upper limb
229	S62163S	Fracture of upper limb
229	S62164A	Fracture of upper limb
229	S62164B	Fracture of upper limb
229	S62164D	Fracture of upper limb
229	S62164G	Fracture of upper limb
229	S62164K	Fracture of upper limb
229	S62164P	Fracture of upper limb
229	S62164S	Fracture of upper limb
229	S62165A	Fracture of upper limb
229	S62165B	Fracture of upper limb
229	S62165D	Fracture of upper limb
229	S62165G	Fracture of upper limb
229	S62165K	Fracture of upper limb
229	S62165P	Fracture of upper limb
229	S62165S	Fracture of upper limb
229	S62166A	Fracture of upper limb
229	S62166B	Fracture of upper limb
229	S62166D	Fracture of upper limb
229	S62166G	Fracture of upper limb
229	S62166K	Fracture of upper limb
229	S62166P	Fracture of upper limb
229	S62166S	Fracture of upper limb
229	S62171A	Fracture of upper limb
229	S62171B	Fracture of upper limb
229	S62171D	Fracture of upper limb
229	S62171G	Fracture of upper limb
229	S62171K	Fracture of upper limb
229	S62171P	Fracture of upper limb
229	S62171S	Fracture of upper limb
229	S62172A	Fracture of upper limb
229	S62172B	Fracture of upper limb
229	S62172D	Fracture of upper limb
229	S62172G	Fracture of upper limb
229	S62172K	Fracture of upper limb
229	S62172P	Fracture of upper limb
229	S62172S	Fracture of upper limb
229	S62173A	Fracture of upper limb

229	S62173B	Fracture of upper limb
229	S62173D	Fracture of upper limb
229	S62173G	Fracture of upper limb
229	S62173K	Fracture of upper limb
229	S62173P	Fracture of upper limb
229	S62173S	Fracture of upper limb
229	S62174A	Fracture of upper limb
229	S62174B	Fracture of upper limb
229	S62174D	Fracture of upper limb
229	S62174G	Fracture of upper limb
229	S62174K	Fracture of upper limb
229	S62174P	Fracture of upper limb
229	S62174S	Fracture of upper limb
229	S62175A	Fracture of upper limb
229	S62175B	Fracture of upper limb
229	S62175D	Fracture of upper limb
229	S62175G	Fracture of upper limb
229	S62175K	Fracture of upper limb
229	S62175P	Fracture of upper limb
229	S62175S	Fracture of upper limb
229	S62176A	Fracture of upper limb
229	S62176B	Fracture of upper limb
229	S62176D	Fracture of upper limb
229	S62176G	Fracture of upper limb
229	S62176K	Fracture of upper limb
229	S62176P	Fracture of upper limb
229	S62176S	Fracture of upper limb
229	S62181A	Fracture of upper limb
229	S62181B	Fracture of upper limb
229	S62181D	Fracture of upper limb
229	S62181G	Fracture of upper limb
229	S62181K	Fracture of upper limb
229	S62181P	Fracture of upper limb
229	S62181S	Fracture of upper limb
229	S62182A	Fracture of upper limb
229	S62182B	Fracture of upper limb
229	S62182D	Fracture of upper limb
229	S62182G	Fracture of upper limb
229	S62182K	Fracture of upper limb
229	S62182P	Fracture of upper limb
229	S62182S	Fracture of upper limb
229	S62183A	Fracture of upper limb
229	S62183B	Fracture of upper limb
229	S62183D	Fracture of upper limb
229	S62183G	Fracture of upper limb
229	S62183K	Fracture of upper limb
229	S62183P	Fracture of upper limb
229	S62183S	Fracture of upper limb
229	S62184A	Fracture of upper limb
229	S62184B	Fracture of upper limb
229	S62184D	Fracture of upper limb

229	S62184G	Fracture of upper limb
229	S62184K	Fracture of upper limb
229	S62184P	Fracture of upper limb
229	S62184S	Fracture of upper limb
229	S62185A	Fracture of upper limb
229	S62185B	Fracture of upper limb
229	S62185D	Fracture of upper limb
229	S62185G	Fracture of upper limb
229	S62185K	Fracture of upper limb
229	S62185P	Fracture of upper limb
229	S62185S	Fracture of upper limb
229	S62186A	Fracture of upper limb
229	S62186B	Fracture of upper limb
229	S62186D	Fracture of upper limb
229	S62186G	Fracture of upper limb
229	S62186K	Fracture of upper limb
229	S62186P	Fracture of upper limb
229	S62186S	Fracture of upper limb
229	S62201A	Fracture of upper limb
229	S62201B	Fracture of upper limb
229	S62201D	Fracture of upper limb
229	S62201G	Fracture of upper limb
229	S62201K	Fracture of upper limb
229	S62201P	Fracture of upper limb
229	S62201S	Fracture of upper limb
229	S62202A	Fracture of upper limb
229	S62202B	Fracture of upper limb
229	S62202D	Fracture of upper limb
229	S62202G	Fracture of upper limb
229	S62202K	Fracture of upper limb
229	S62202P	Fracture of upper limb
229	S62202S	Fracture of upper limb
229	S62209A	Fracture of upper limb
229	S62209B	Fracture of upper limb
229	S62209D	Fracture of upper limb
229	S62209G	Fracture of upper limb
229	S62209K	Fracture of upper limb
229	S62209P	Fracture of upper limb
229	S62209S	Fracture of upper limb
229	S62211A	Fracture of upper limb
229	S62211B	Fracture of upper limb
229	S62211D	Fracture of upper limb
229	S62211G	Fracture of upper limb
229	S62211K	Fracture of upper limb
229	S62211P	Fracture of upper limb
229	S62211S	Fracture of upper limb
229	S62212A	Fracture of upper limb
229	S62212B	Fracture of upper limb
229	S62212D	Fracture of upper limb
229	S62212G	Fracture of upper limb
229	S62212K	Fracture of upper limb

229	S62212P	Fracture of upper limb
229	S62212S	Fracture of upper limb
229	S62213A	Fracture of upper limb
229	S62213B	Fracture of upper limb
229	S62213D	Fracture of upper limb
229	S62213G	Fracture of upper limb
229	S62213K	Fracture of upper limb
229	S62213P	Fracture of upper limb
229	S62213S	Fracture of upper limb
229	S62221A	Fracture of upper limb
229	S62221B	Fracture of upper limb
229	S62221D	Fracture of upper limb
229	S62221G	Fracture of upper limb
229	S62221K	Fracture of upper limb
229	S62221P	Fracture of upper limb
229	S62221S	Fracture of upper limb
229	S62222A	Fracture of upper limb
229	S62222B	Fracture of upper limb
229	S62222D	Fracture of upper limb
229	S62222G	Fracture of upper limb
229	S62222K	Fracture of upper limb
229	S62222P	Fracture of upper limb
229	S62222S	Fracture of upper limb
229	S62223A	Fracture of upper limb
229	S62223B	Fracture of upper limb
229	S62223D	Fracture of upper limb
229	S62223G	Fracture of upper limb
229	S62223K	Fracture of upper limb
229	S62223P	Fracture of upper limb
229	S62223S	Fracture of upper limb
229	S62224A	Fracture of upper limb
229	S62224B	Fracture of upper limb
229	S62224D	Fracture of upper limb
229	S62224G	Fracture of upper limb
229	S62224K	Fracture of upper limb
229	S62224P	Fracture of upper limb
229	S62224S	Fracture of upper limb
229	S62225A	Fracture of upper limb
229	S62225B	Fracture of upper limb
229	S62225D	Fracture of upper limb
229	S62225G	Fracture of upper limb
229	S62225K	Fracture of upper limb
229	S62225P	Fracture of upper limb
229	S62225S	Fracture of upper limb
229	S62226A	Fracture of upper limb
229	S62226B	Fracture of upper limb
229	S62226D	Fracture of upper limb
229	S62226G	Fracture of upper limb
229	S62226K	Fracture of upper limb
229	S62226P	Fracture of upper limb
229	S62226S	Fracture of upper limb

229	S62231A	Fracture of upper limb
229	S62231B	Fracture of upper limb
229	S62231D	Fracture of upper limb
229	S62231G	Fracture of upper limb
229	S62231K	Fracture of upper limb
229	S62231P	Fracture of upper limb
229	S62231S	Fracture of upper limb
229	S62232A	Fracture of upper limb
229	S62232B	Fracture of upper limb
229	S62232D	Fracture of upper limb
229	S62232G	Fracture of upper limb
229	S62232K	Fracture of upper limb
229	S62232P	Fracture of upper limb
229	S62232S	Fracture of upper limb
229	S62233A	Fracture of upper limb
229	S62233B	Fracture of upper limb
229	S62233D	Fracture of upper limb
229	S62233G	Fracture of upper limb
229	S62233K	Fracture of upper limb
229	S62233P	Fracture of upper limb
229	S62233S	Fracture of upper limb
229	S62234A	Fracture of upper limb
229	S62234B	Fracture of upper limb
229	S62234D	Fracture of upper limb
229	S62234G	Fracture of upper limb
229	S62234K	Fracture of upper limb
229	S62234P	Fracture of upper limb
229	S62234S	Fracture of upper limb
229	S62235A	Fracture of upper limb
229	S62235B	Fracture of upper limb
229	S62235D	Fracture of upper limb
229	S62235G	Fracture of upper limb
229	S62235K	Fracture of upper limb
229	S62235P	Fracture of upper limb
229	S62235S	Fracture of upper limb
229	S62236A	Fracture of upper limb
229	S62236B	Fracture of upper limb
229	S62236D	Fracture of upper limb
229	S62236G	Fracture of upper limb
229	S62236K	Fracture of upper limb
229	S62236P	Fracture of upper limb
229	S62236S	Fracture of upper limb
229	S62241A	Fracture of upper limb
229	S62241B	Fracture of upper limb
229	S62241D	Fracture of upper limb
229	S62241G	Fracture of upper limb
229	S62241K	Fracture of upper limb
229	S62241P	Fracture of upper limb
229	S62241S	Fracture of upper limb
229	S62242A	Fracture of upper limb
229	S62242B	Fracture of upper limb

229	S62242D	Fracture of upper limb
229	S62242G	Fracture of upper limb
229	S62242K	Fracture of upper limb
229	S62242P	Fracture of upper limb
229	S62242S	Fracture of upper limb
229	S62243A	Fracture of upper limb
229	S62243B	Fracture of upper limb
229	S62243D	Fracture of upper limb
229	S62243G	Fracture of upper limb
229	S62243K	Fracture of upper limb
229	S62243P	Fracture of upper limb
229	S62243S	Fracture of upper limb
229	S62244A	Fracture of upper limb
229	S62244B	Fracture of upper limb
229	S62244D	Fracture of upper limb
229	S62244G	Fracture of upper limb
229	S62244K	Fracture of upper limb
229	S62244P	Fracture of upper limb
229	S62244S	Fracture of upper limb
229	S62245A	Fracture of upper limb
229	S62245B	Fracture of upper limb
229	S62245D	Fracture of upper limb
229	S62245G	Fracture of upper limb
229	S62245K	Fracture of upper limb
229	S62245P	Fracture of upper limb
229	S62245S	Fracture of upper limb
229	S62246A	Fracture of upper limb
229	S62246B	Fracture of upper limb
229	S62246D	Fracture of upper limb
229	S62246G	Fracture of upper limb
229	S62246K	Fracture of upper limb
229	S62246P	Fracture of upper limb
229	S62246S	Fracture of upper limb
229	S62251A	Fracture of upper limb
229	S62251B	Fracture of upper limb
229	S62251D	Fracture of upper limb
229	S62251G	Fracture of upper limb
229	S62251K	Fracture of upper limb
229	S62251P	Fracture of upper limb
229	S62251S	Fracture of upper limb
229	S62252A	Fracture of upper limb
229	S62252B	Fracture of upper limb
229	S62252D	Fracture of upper limb
229	S62252G	Fracture of upper limb
229	S62252K	Fracture of upper limb
229	S62252P	Fracture of upper limb
229	S62252S	Fracture of upper limb
229	S62253A	Fracture of upper limb
229	S62253B	Fracture of upper limb
229	S62253D	Fracture of upper limb
229	S62253G	Fracture of upper limb

229	S62253K	Fracture of upper limb
229	S62253P	Fracture of upper limb
229	S62253S	Fracture of upper limb
229	S62254A	Fracture of upper limb
229	S62254B	Fracture of upper limb
229	S62254D	Fracture of upper limb
229	S62254G	Fracture of upper limb
229	S62254K	Fracture of upper limb
229	S62254P	Fracture of upper limb
229	S62254S	Fracture of upper limb
229	S62255A	Fracture of upper limb
229	S62255B	Fracture of upper limb
229	S62255D	Fracture of upper limb
229	S62255G	Fracture of upper limb
229	S62255K	Fracture of upper limb
229	S62255P	Fracture of upper limb
229	S62255S	Fracture of upper limb
229	S62256A	Fracture of upper limb
229	S62256B	Fracture of upper limb
229	S62256D	Fracture of upper limb
229	S62256G	Fracture of upper limb
229	S62256K	Fracture of upper limb
229	S62256P	Fracture of upper limb
229	S62256S	Fracture of upper limb
229	S62291A	Fracture of upper limb
229	S62291B	Fracture of upper limb
229	S62291D	Fracture of upper limb
229	S62291G	Fracture of upper limb
229	S62291K	Fracture of upper limb
229	S62291P	Fracture of upper limb
229	S62291S	Fracture of upper limb
229	S62292A	Fracture of upper limb
229	S62292B	Fracture of upper limb
229	S62292D	Fracture of upper limb
229	S62292G	Fracture of upper limb
229	S62292K	Fracture of upper limb
229	S62292P	Fracture of upper limb
229	S62292S	Fracture of upper limb
229	S62299A	Fracture of upper limb
229	S62299B	Fracture of upper limb
229	S62299D	Fracture of upper limb
229	S62299G	Fracture of upper limb
229	S62299K	Fracture of upper limb
229	S62299P	Fracture of upper limb
229	S62299S	Fracture of upper limb
229	S62300A	Fracture of upper limb
229	S62300B	Fracture of upper limb
229	S62300D	Fracture of upper limb
229	S62300G	Fracture of upper limb
229	S62300K	Fracture of upper limb
229	S62300P	Fracture of upper limb

229	S62300S	Fracture of upper limb
229	S62301A	Fracture of upper limb
229	S62301B	Fracture of upper limb
229	S62301D	Fracture of upper limb
229	S62301G	Fracture of upper limb
229	S62301K	Fracture of upper limb
229	S62301P	Fracture of upper limb
229	S62301S	Fracture of upper limb
229	S62302A	Fracture of upper limb
229	S62302B	Fracture of upper limb
229	S62302D	Fracture of upper limb
229	S62302G	Fracture of upper limb
229	S62302K	Fracture of upper limb
229	S62302P	Fracture of upper limb
229	S62302S	Fracture of upper limb
229	S62303A	Fracture of upper limb
229	S62303B	Fracture of upper limb
229	S62303D	Fracture of upper limb
229	S62303G	Fracture of upper limb
229	S62303K	Fracture of upper limb
229	S62303P	Fracture of upper limb
229	S62303S	Fracture of upper limb
229	S62304A	Fracture of upper limb
229	S62304B	Fracture of upper limb
229	S62304D	Fracture of upper limb
229	S62304G	Fracture of upper limb
229	S62304K	Fracture of upper limb
229	S62304P	Fracture of upper limb
229	S62304S	Fracture of upper limb
229	S62305A	Fracture of upper limb
229	S62305B	Fracture of upper limb
229	S62305D	Fracture of upper limb
229	S62305G	Fracture of upper limb
229	S62305K	Fracture of upper limb
229	S62305P	Fracture of upper limb
229	S62305S	Fracture of upper limb
229	S62306A	Fracture of upper limb
229	S62306B	Fracture of upper limb
229	S62306D	Fracture of upper limb
229	S62306G	Fracture of upper limb
229	S62306K	Fracture of upper limb
229	S62306P	Fracture of upper limb
229	S62306S	Fracture of upper limb
229	S62307A	Fracture of upper limb
229	S62307B	Fracture of upper limb
229	S62307D	Fracture of upper limb
229	S62307G	Fracture of upper limb
229	S62307K	Fracture of upper limb
229	S62307P	Fracture of upper limb
229	S62307S	Fracture of upper limb
229	S62308A	Fracture of upper limb

229	S62308B	Fracture of upper limb
229	S62308D	Fracture of upper limb
229	S62308G	Fracture of upper limb
229	S62308K	Fracture of upper limb
229	S62308P	Fracture of upper limb
229	S62308S	Fracture of upper limb
229	S62309A	Fracture of upper limb
229	S62309B	Fracture of upper limb
229	S62309D	Fracture of upper limb
229	S62309G	Fracture of upper limb
229	S62309K	Fracture of upper limb
229	S62309P	Fracture of upper limb
229	S62309S	Fracture of upper limb
229	S62310A	Fracture of upper limb
229	S62310B	Fracture of upper limb
229	S62310D	Fracture of upper limb
229	S62310G	Fracture of upper limb
229	S62310K	Fracture of upper limb
229	S62310P	Fracture of upper limb
229	S62310S	Fracture of upper limb
229	S62311A	Fracture of upper limb
229	S62311B	Fracture of upper limb
229	S62311D	Fracture of upper limb
229	S62311G	Fracture of upper limb
229	S62311K	Fracture of upper limb
229	S62311P	Fracture of upper limb
229	S62311S	Fracture of upper limb
229	S62312A	Fracture of upper limb
229	S62312B	Fracture of upper limb
229	S62312D	Fracture of upper limb
229	S62312G	Fracture of upper limb
229	S62312K	Fracture of upper limb
229	S62312P	Fracture of upper limb
229	S62312S	Fracture of upper limb
229	S62313A	Fracture of upper limb
229	S62313B	Fracture of upper limb
229	S62313D	Fracture of upper limb
229	S62313G	Fracture of upper limb
229	S62313K	Fracture of upper limb
229	S62313P	Fracture of upper limb
229	S62313S	Fracture of upper limb
229	S62314A	Fracture of upper limb
229	S62314B	Fracture of upper limb
229	S62314D	Fracture of upper limb
229	S62314G	Fracture of upper limb
229	S62314K	Fracture of upper limb
229	S62314P	Fracture of upper limb
229	S62314S	Fracture of upper limb
229	S62315A	Fracture of upper limb
229	S62315B	Fracture of upper limb
229	S62315D	Fracture of upper limb

229	S62315G	Fracture of upper limb
229	S62315K	Fracture of upper limb
229	S62315P	Fracture of upper limb
229	S62315S	Fracture of upper limb
229	S62316A	Fracture of upper limb
229	S62316B	Fracture of upper limb
229	S62316D	Fracture of upper limb
229	S62316G	Fracture of upper limb
229	S62316K	Fracture of upper limb
229	S62316P	Fracture of upper limb
229	S62316S	Fracture of upper limb
229	S62317A	Fracture of upper limb
229	S62317B	Fracture of upper limb
229	S62317D	Fracture of upper limb
229	S62317G	Fracture of upper limb
229	S62317K	Fracture of upper limb
229	S62317P	Fracture of upper limb
229	S62317S	Fracture of upper limb
229	S62318A	Fracture of upper limb
229	S62318B	Fracture of upper limb
229	S62318D	Fracture of upper limb
229	S62318G	Fracture of upper limb
229	S62318K	Fracture of upper limb
229	S62318P	Fracture of upper limb
229	S62318S	Fracture of upper limb
229	S62319A	Fracture of upper limb
229	S62319B	Fracture of upper limb
229	S62319D	Fracture of upper limb
229	S62319G	Fracture of upper limb
229	S62319K	Fracture of upper limb
229	S62319P	Fracture of upper limb
229	S62319S	Fracture of upper limb
229	S62320A	Fracture of upper limb
229	S62320B	Fracture of upper limb
229	S62320D	Fracture of upper limb
229	S62320G	Fracture of upper limb
229	S62320K	Fracture of upper limb
229	S62320P	Fracture of upper limb
229	S62320S	Fracture of upper limb
229	S62321A	Fracture of upper limb
229	S62321B	Fracture of upper limb
229	S62321D	Fracture of upper limb
229	S62321G	Fracture of upper limb
229	S62321K	Fracture of upper limb
229	S62321P	Fracture of upper limb
229	S62321S	Fracture of upper limb
229	S62322A	Fracture of upper limb
229	S62322B	Fracture of upper limb
229	S62322D	Fracture of upper limb
229	S62322G	Fracture of upper limb
229	S62322K	Fracture of upper limb

229	S62322P	Fracture of upper limb
229	S62322S	Fracture of upper limb
229	S62323A	Fracture of upper limb
229	S62323B	Fracture of upper limb
229	S62323D	Fracture of upper limb
229	S62323G	Fracture of upper limb
229	S62323K	Fracture of upper limb
229	S62323P	Fracture of upper limb
229	S62323S	Fracture of upper limb
229	S62324A	Fracture of upper limb
229	S62324B	Fracture of upper limb
229	S62324D	Fracture of upper limb
229	S62324G	Fracture of upper limb
229	S62324K	Fracture of upper limb
229	S62324P	Fracture of upper limb
229	S62324S	Fracture of upper limb
229	S62325A	Fracture of upper limb
229	S62325B	Fracture of upper limb
229	S62325D	Fracture of upper limb
229	S62325G	Fracture of upper limb
229	S62325K	Fracture of upper limb
229	S62325P	Fracture of upper limb
229	S62325S	Fracture of upper limb
229	S62326A	Fracture of upper limb
229	S62326B	Fracture of upper limb
229	S62326D	Fracture of upper limb
229	S62326G	Fracture of upper limb
229	S62326K	Fracture of upper limb
229	S62326P	Fracture of upper limb
229	S62326S	Fracture of upper limb
229	S62327A	Fracture of upper limb
229	S62327B	Fracture of upper limb
229	S62327D	Fracture of upper limb
229	S62327G	Fracture of upper limb
229	S62327K	Fracture of upper limb
229	S62327P	Fracture of upper limb
229	S62327S	Fracture of upper limb
229	S62328A	Fracture of upper limb
229	S62328B	Fracture of upper limb
229	S62328D	Fracture of upper limb
229	S62328G	Fracture of upper limb
229	S62328K	Fracture of upper limb
229	S62328P	Fracture of upper limb
229	S62328S	Fracture of upper limb
229	S62329A	Fracture of upper limb
229	S62329B	Fracture of upper limb
229	S62329D	Fracture of upper limb
229	S62329G	Fracture of upper limb
229	S62329K	Fracture of upper limb
229	S62329P	Fracture of upper limb
229	S62329S	Fracture of upper limb

229	S62330A	Fracture of upper limb
229	S62330B	Fracture of upper limb
229	S62330D	Fracture of upper limb
229	S62330G	Fracture of upper limb
229	S62330K	Fracture of upper limb
229	S62330P	Fracture of upper limb
229	S62330S	Fracture of upper limb
229	S62331A	Fracture of upper limb
229	S62331B	Fracture of upper limb
229	S62331D	Fracture of upper limb
229	S62331G	Fracture of upper limb
229	S62331K	Fracture of upper limb
229	S62331P	Fracture of upper limb
229	S62331S	Fracture of upper limb
229	S62332A	Fracture of upper limb
229	S62332B	Fracture of upper limb
229	S62332D	Fracture of upper limb
229	S62332G	Fracture of upper limb
229	S62332K	Fracture of upper limb
229	S62332P	Fracture of upper limb
229	S62332S	Fracture of upper limb
229	S62333A	Fracture of upper limb
229	S62333B	Fracture of upper limb
229	S62333D	Fracture of upper limb
229	S62333G	Fracture of upper limb
229	S62333K	Fracture of upper limb
229	S62333P	Fracture of upper limb
229	S62333S	Fracture of upper limb
229	S62334A	Fracture of upper limb
229	S62334B	Fracture of upper limb
229	S62334D	Fracture of upper limb
229	S62334G	Fracture of upper limb
229	S62334K	Fracture of upper limb
229	S62334P	Fracture of upper limb
229	S62334S	Fracture of upper limb
229	S62335A	Fracture of upper limb
229	S62335B	Fracture of upper limb
229	S62335D	Fracture of upper limb
229	S62335G	Fracture of upper limb
229	S62335K	Fracture of upper limb
229	S62335P	Fracture of upper limb
229	S62335S	Fracture of upper limb
229	S62336A	Fracture of upper limb
229	S62336B	Fracture of upper limb
229	S62336D	Fracture of upper limb
229	S62336G	Fracture of upper limb
229	S62336K	Fracture of upper limb
229	S62336P	Fracture of upper limb
229	S62336S	Fracture of upper limb
229	S62337A	Fracture of upper limb
229	S62337B	Fracture of upper limb

229	S62337D	Fracture of upper limb
229	S62337G	Fracture of upper limb
229	S62337K	Fracture of upper limb
229	S62337P	Fracture of upper limb
229	S62337S	Fracture of upper limb
229	S62338A	Fracture of upper limb
229	S62338B	Fracture of upper limb
229	S62338D	Fracture of upper limb
229	S62338G	Fracture of upper limb
229	S62338K	Fracture of upper limb
229	S62338P	Fracture of upper limb
229	S62338S	Fracture of upper limb
229	S62339A	Fracture of upper limb
229	S62339B	Fracture of upper limb
229	S62339D	Fracture of upper limb
229	S62339G	Fracture of upper limb
229	S62339K	Fracture of upper limb
229	S62339P	Fracture of upper limb
229	S62339S	Fracture of upper limb
229	S62340A	Fracture of upper limb
229	S62340B	Fracture of upper limb
229	S62340D	Fracture of upper limb
229	S62340G	Fracture of upper limb
229	S62340K	Fracture of upper limb
229	S62340P	Fracture of upper limb
229	S62340S	Fracture of upper limb
229	S62341A	Fracture of upper limb
229	S62341B	Fracture of upper limb
229	S62341D	Fracture of upper limb
229	S62341G	Fracture of upper limb
229	S62341K	Fracture of upper limb
229	S62341P	Fracture of upper limb
229	S62341S	Fracture of upper limb
229	S62342A	Fracture of upper limb
229	S62342B	Fracture of upper limb
229	S62342D	Fracture of upper limb
229	S62342G	Fracture of upper limb
229	S62342K	Fracture of upper limb
229	S62342P	Fracture of upper limb
229	S62342S	Fracture of upper limb
229	S62343A	Fracture of upper limb
229	S62343B	Fracture of upper limb
229	S62343D	Fracture of upper limb
229	S62343G	Fracture of upper limb
229	S62343K	Fracture of upper limb
229	S62343P	Fracture of upper limb
229	S62343S	Fracture of upper limb
229	S62344A	Fracture of upper limb
229	S62344B	Fracture of upper limb
229	S62344D	Fracture of upper limb
229	S62344G	Fracture of upper limb

229	S62344K	Fracture of upper limb
229	S62344P	Fracture of upper limb
229	S62344S	Fracture of upper limb
229	S62345A	Fracture of upper limb
229	S62345B	Fracture of upper limb
229	S62345D	Fracture of upper limb
229	S62345G	Fracture of upper limb
229	S62345K	Fracture of upper limb
229	S62345P	Fracture of upper limb
229	S62345S	Fracture of upper limb
229	S62346A	Fracture of upper limb
229	S62346B	Fracture of upper limb
229	S62346D	Fracture of upper limb
229	S62346G	Fracture of upper limb
229	S62346K	Fracture of upper limb
229	S62346P	Fracture of upper limb
229	S62346S	Fracture of upper limb
229	S62347A	Fracture of upper limb
229	S62347B	Fracture of upper limb
229	S62347D	Fracture of upper limb
229	S62347G	Fracture of upper limb
229	S62347K	Fracture of upper limb
229	S62347P	Fracture of upper limb
229	S62347S	Fracture of upper limb
229	S62348A	Fracture of upper limb
229	S62348B	Fracture of upper limb
229	S62348D	Fracture of upper limb
229	S62348G	Fracture of upper limb
229	S62348K	Fracture of upper limb
229	S62348P	Fracture of upper limb
229	S62348S	Fracture of upper limb
229	S62349A	Fracture of upper limb
229	S62349B	Fracture of upper limb
229	S62349D	Fracture of upper limb
229	S62349G	Fracture of upper limb
229	S62349K	Fracture of upper limb
229	S62349P	Fracture of upper limb
229	S62349S	Fracture of upper limb
229	S62350A	Fracture of upper limb
229	S62350B	Fracture of upper limb
229	S62350D	Fracture of upper limb
229	S62350G	Fracture of upper limb
229	S62350K	Fracture of upper limb
229	S62350P	Fracture of upper limb
229	S62350S	Fracture of upper limb
229	S62351A	Fracture of upper limb
229	S62351B	Fracture of upper limb
229	S62351D	Fracture of upper limb
229	S62351G	Fracture of upper limb
229	S62351K	Fracture of upper limb
229	S62351P	Fracture of upper limb

229	S62351S	Fracture of upper limb
229	S62352A	Fracture of upper limb
229	S62352B	Fracture of upper limb
229	S62352D	Fracture of upper limb
229	S62352G	Fracture of upper limb
229	S62352K	Fracture of upper limb
229	S62352P	Fracture of upper limb
229	S62352S	Fracture of upper limb
229	S62353A	Fracture of upper limb
229	S62353B	Fracture of upper limb
229	S62353D	Fracture of upper limb
229	S62353G	Fracture of upper limb
229	S62353K	Fracture of upper limb
229	S62353P	Fracture of upper limb
229	S62353S	Fracture of upper limb
229	S62354A	Fracture of upper limb
229	S62354B	Fracture of upper limb
229	S62354D	Fracture of upper limb
229	S62354G	Fracture of upper limb
229	S62354K	Fracture of upper limb
229	S62354P	Fracture of upper limb
229	S62354S	Fracture of upper limb
229	S62355A	Fracture of upper limb
229	S62355B	Fracture of upper limb
229	S62355D	Fracture of upper limb
229	S62355G	Fracture of upper limb
229	S62355K	Fracture of upper limb
229	S62355P	Fracture of upper limb
229	S62355S	Fracture of upper limb
229	S62356A	Fracture of upper limb
229	S62356B	Fracture of upper limb
229	S62356D	Fracture of upper limb
229	S62356G	Fracture of upper limb
229	S62356K	Fracture of upper limb
229	S62356P	Fracture of upper limb
229	S62356S	Fracture of upper limb
229	S62357A	Fracture of upper limb
229	S62357B	Fracture of upper limb
229	S62357D	Fracture of upper limb
229	S62357G	Fracture of upper limb
229	S62357K	Fracture of upper limb
229	S62357P	Fracture of upper limb
229	S62357S	Fracture of upper limb
229	S62358A	Fracture of upper limb
229	S62358B	Fracture of upper limb
229	S62358D	Fracture of upper limb
229	S62358G	Fracture of upper limb
229	S62358K	Fracture of upper limb
229	S62358P	Fracture of upper limb
229	S62358S	Fracture of upper limb
229	S62359A	Fracture of upper limb

229	S62359B	Fracture of upper limb
229	S62359D	Fracture of upper limb
229	S62359G	Fracture of upper limb
229	S62359K	Fracture of upper limb
229	S62359P	Fracture of upper limb
229	S62359S	Fracture of upper limb
229	S62360A	Fracture of upper limb
229	S62360B	Fracture of upper limb
229	S62360D	Fracture of upper limb
229	S62360G	Fracture of upper limb
229	S62360K	Fracture of upper limb
229	S62360P	Fracture of upper limb
229	S62360S	Fracture of upper limb
229	S62361A	Fracture of upper limb
229	S62361B	Fracture of upper limb
229	S62361D	Fracture of upper limb
229	S62361G	Fracture of upper limb
229	S62361K	Fracture of upper limb
229	S62361P	Fracture of upper limb
229	S62361S	Fracture of upper limb
229	S62362A	Fracture of upper limb
229	S62362B	Fracture of upper limb
229	S62362D	Fracture of upper limb
229	S62362G	Fracture of upper limb
229	S62362K	Fracture of upper limb
229	S62362P	Fracture of upper limb
229	S62362S	Fracture of upper limb
229	S62363A	Fracture of upper limb
229	S62363B	Fracture of upper limb
229	S62363D	Fracture of upper limb
229	S62363G	Fracture of upper limb
229	S62363K	Fracture of upper limb
229	S62363P	Fracture of upper limb
229	S62363S	Fracture of upper limb
229	S62364A	Fracture of upper limb
229	S62364B	Fracture of upper limb
229	S62364D	Fracture of upper limb
229	S62364G	Fracture of upper limb
229	S62364K	Fracture of upper limb
229	S62364P	Fracture of upper limb
229	S62364S	Fracture of upper limb
229	S62365A	Fracture of upper limb
229	S62365B	Fracture of upper limb
229	S62365D	Fracture of upper limb
229	S62365G	Fracture of upper limb
229	S62365K	Fracture of upper limb
229	S62365P	Fracture of upper limb
229	S62365S	Fracture of upper limb
229	S62366A	Fracture of upper limb
229	S62366B	Fracture of upper limb
229	S62366D	Fracture of upper limb

229	S62366G	Fracture of upper limb
229	S62366K	Fracture of upper limb
229	S62366P	Fracture of upper limb
229	S62366S	Fracture of upper limb
229	S62367A	Fracture of upper limb
229	S62367B	Fracture of upper limb
229	S62367D	Fracture of upper limb
229	S62367G	Fracture of upper limb
229	S62367K	Fracture of upper limb
229	S62367P	Fracture of upper limb
229	S62367S	Fracture of upper limb
229	S62368A	Fracture of upper limb
229	S62368B	Fracture of upper limb
229	S62368D	Fracture of upper limb
229	S62368G	Fracture of upper limb
229	S62368K	Fracture of upper limb
229	S62368P	Fracture of upper limb
229	S62368S	Fracture of upper limb
229	S62369A	Fracture of upper limb
229	S62369B	Fracture of upper limb
229	S62369D	Fracture of upper limb
229	S62369G	Fracture of upper limb
229	S62369K	Fracture of upper limb
229	S62369P	Fracture of upper limb
229	S62369S	Fracture of upper limb
229	S62390A	Fracture of upper limb
229	S62390B	Fracture of upper limb
229	S62390D	Fracture of upper limb
229	S62390G	Fracture of upper limb
229	S62390K	Fracture of upper limb
229	S62390P	Fracture of upper limb
229	S62390S	Fracture of upper limb
229	S62391A	Fracture of upper limb
229	S62391B	Fracture of upper limb
229	S62391D	Fracture of upper limb
229	S62391G	Fracture of upper limb
229	S62391K	Fracture of upper limb
229	S62391P	Fracture of upper limb
229	S62391S	Fracture of upper limb
229	S62392A	Fracture of upper limb
229	S62392B	Fracture of upper limb
229	S62392D	Fracture of upper limb
229	S62392G	Fracture of upper limb
229	S62392K	Fracture of upper limb
229	S62392P	Fracture of upper limb
229	S62392S	Fracture of upper limb
229	S62393A	Fracture of upper limb
229	S62393B	Fracture of upper limb
229	S62393D	Fracture of upper limb
229	S62393G	Fracture of upper limb
229	S62393K	Fracture of upper limb

229	S62393P	Fracture of upper limb
229	S62393S	Fracture of upper limb
229	S62394A	Fracture of upper limb
229	S62394B	Fracture of upper limb
229	S62394D	Fracture of upper limb
229	S62394G	Fracture of upper limb
229	S62394K	Fracture of upper limb
229	S62394P	Fracture of upper limb
229	S62394S	Fracture of upper limb
229	S62395A	Fracture of upper limb
229	S62395B	Fracture of upper limb
229	S62395D	Fracture of upper limb
229	S62395G	Fracture of upper limb
229	S62395K	Fracture of upper limb
229	S62395P	Fracture of upper limb
229	S62395S	Fracture of upper limb
229	S62396A	Fracture of upper limb
229	S62396B	Fracture of upper limb
229	S62396D	Fracture of upper limb
229	S62396G	Fracture of upper limb
229	S62396K	Fracture of upper limb
229	S62396P	Fracture of upper limb
229	S62396S	Fracture of upper limb
229	S62397A	Fracture of upper limb
229	S62397B	Fracture of upper limb
229	S62397D	Fracture of upper limb
229	S62397G	Fracture of upper limb
229	S62397K	Fracture of upper limb
229	S62397P	Fracture of upper limb
229	S62397S	Fracture of upper limb
229	S62398A	Fracture of upper limb
229	S62398B	Fracture of upper limb
229	S62398D	Fracture of upper limb
229	S62398G	Fracture of upper limb
229	S62398K	Fracture of upper limb
229	S62398P	Fracture of upper limb
229	S62398S	Fracture of upper limb
229	S62399A	Fracture of upper limb
229	S62399B	Fracture of upper limb
229	S62399D	Fracture of upper limb
229	S62399G	Fracture of upper limb
229	S62399K	Fracture of upper limb
229	S62399P	Fracture of upper limb
229	S62399S	Fracture of upper limb
229	S62501A	Fracture of upper limb
229	S62501B	Fracture of upper limb
229	S62501D	Fracture of upper limb
229	S62501G	Fracture of upper limb
229	S62501K	Fracture of upper limb
229	S62501P	Fracture of upper limb
229	S62501S	Fracture of upper limb

229	S62502A	Fracture of upper limb
229	S62502B	Fracture of upper limb
229	S62502D	Fracture of upper limb
229	S62502G	Fracture of upper limb
229	S62502K	Fracture of upper limb
229	S62502P	Fracture of upper limb
229	S62502S	Fracture of upper limb
229	S62509A	Fracture of upper limb
229	S62509B	Fracture of upper limb
229	S62509D	Fracture of upper limb
229	S62509G	Fracture of upper limb
229	S62509K	Fracture of upper limb
229	S62509P	Fracture of upper limb
229	S62509S	Fracture of upper limb
229	S62511A	Fracture of upper limb
229	S62511B	Fracture of upper limb
229	S62511D	Fracture of upper limb
229	S62511G	Fracture of upper limb
229	S62511K	Fracture of upper limb
229	S62511P	Fracture of upper limb
229	S62511S	Fracture of upper limb
229	S62512A	Fracture of upper limb
229	S62512B	Fracture of upper limb
229	S62512D	Fracture of upper limb
229	S62512G	Fracture of upper limb
229	S62512K	Fracture of upper limb
229	S62512P	Fracture of upper limb
229	S62512S	Fracture of upper limb
229	S62513A	Fracture of upper limb
229	S62513B	Fracture of upper limb
229	S62513D	Fracture of upper limb
229	S62513G	Fracture of upper limb
229	S62513K	Fracture of upper limb
229	S62513P	Fracture of upper limb
229	S62513S	Fracture of upper limb
229	S62514A	Fracture of upper limb
229	S62514B	Fracture of upper limb
229	S62514D	Fracture of upper limb
229	S62514G	Fracture of upper limb
229	S62514K	Fracture of upper limb
229	S62514P	Fracture of upper limb
229	S62514S	Fracture of upper limb
229	S62515A	Fracture of upper limb
229	S62515B	Fracture of upper limb
229	S62515D	Fracture of upper limb
229	S62515G	Fracture of upper limb
229	S62515K	Fracture of upper limb
229	S62515P	Fracture of upper limb
229	S62515S	Fracture of upper limb
229	S62516A	Fracture of upper limb
229	S62516B	Fracture of upper limb

229	S62516D	Fracture of upper limb
229	S62516G	Fracture of upper limb
229	S62516K	Fracture of upper limb
229	S62516P	Fracture of upper limb
229	S62516S	Fracture of upper limb
229	S62521A	Fracture of upper limb
229	S62521B	Fracture of upper limb
229	S62521D	Fracture of upper limb
229	S62521G	Fracture of upper limb
229	S62521K	Fracture of upper limb
229	S62521P	Fracture of upper limb
229	S62521S	Fracture of upper limb
229	S62522A	Fracture of upper limb
229	S62522B	Fracture of upper limb
229	S62522D	Fracture of upper limb
229	S62522G	Fracture of upper limb
229	S62522K	Fracture of upper limb
229	S62522P	Fracture of upper limb
229	S62522S	Fracture of upper limb
229	S62523A	Fracture of upper limb
229	S62523B	Fracture of upper limb
229	S62523D	Fracture of upper limb
229	S62523G	Fracture of upper limb
229	S62523K	Fracture of upper limb
229	S62523P	Fracture of upper limb
229	S62523S	Fracture of upper limb
229	S62524A	Fracture of upper limb
229	S62524B	Fracture of upper limb
229	S62524D	Fracture of upper limb
229	S62524G	Fracture of upper limb
229	S62524K	Fracture of upper limb
229	S62524P	Fracture of upper limb
229	S62524S	Fracture of upper limb
229	S62525A	Fracture of upper limb
229	S62525B	Fracture of upper limb
229	S62525D	Fracture of upper limb
229	S62525G	Fracture of upper limb
229	S62525K	Fracture of upper limb
229	S62525P	Fracture of upper limb
229	S62525S	Fracture of upper limb
229	S62526A	Fracture of upper limb
229	S62526B	Fracture of upper limb
229	S62526D	Fracture of upper limb
229	S62526G	Fracture of upper limb
229	S62526K	Fracture of upper limb
229	S62526P	Fracture of upper limb
229	S62526S	Fracture of upper limb
229	S62600A	Fracture of upper limb
229	S62600B	Fracture of upper limb
229	S62600D	Fracture of upper limb
229	S62600G	Fracture of upper limb

229	S62600K	Fracture of upper limb
229	S62600P	Fracture of upper limb
229	S62600S	Fracture of upper limb
229	S62601A	Fracture of upper limb
229	S62601B	Fracture of upper limb
229	S62601D	Fracture of upper limb
229	S62601G	Fracture of upper limb
229	S62601K	Fracture of upper limb
229	S62601P	Fracture of upper limb
229	S62601S	Fracture of upper limb
229	S62602A	Fracture of upper limb
229	S62602B	Fracture of upper limb
229	S62602D	Fracture of upper limb
229	S62602G	Fracture of upper limb
229	S62602K	Fracture of upper limb
229	S62602P	Fracture of upper limb
229	S62602S	Fracture of upper limb
229	S62603A	Fracture of upper limb
229	S62603B	Fracture of upper limb
229	S62603D	Fracture of upper limb
229	S62603G	Fracture of upper limb
229	S62603K	Fracture of upper limb
229	S62603P	Fracture of upper limb
229	S62603S	Fracture of upper limb
229	S62604A	Fracture of upper limb
229	S62604B	Fracture of upper limb
229	S62604D	Fracture of upper limb
229	S62604G	Fracture of upper limb
229	S62604K	Fracture of upper limb
229	S62604P	Fracture of upper limb
229	S62604S	Fracture of upper limb
229	S62605A	Fracture of upper limb
229	S62605B	Fracture of upper limb
229	S62605D	Fracture of upper limb
229	S62605G	Fracture of upper limb
229	S62605K	Fracture of upper limb
229	S62605P	Fracture of upper limb
229	S62605S	Fracture of upper limb
229	S62606A	Fracture of upper limb
229	S62606B	Fracture of upper limb
229	S62606D	Fracture of upper limb
229	S62606G	Fracture of upper limb
229	S62606K	Fracture of upper limb
229	S62606P	Fracture of upper limb
229	S62606S	Fracture of upper limb
229	S62607A	Fracture of upper limb
229	S62607B	Fracture of upper limb
229	S62607D	Fracture of upper limb
229	S62607G	Fracture of upper limb
229	S62607K	Fracture of upper limb
229	S62607P	Fracture of upper limb

229	S62607S	Fracture of upper limb
229	S62608A	Fracture of upper limb
229	S62608B	Fracture of upper limb
229	S62608D	Fracture of upper limb
229	S62608G	Fracture of upper limb
229	S62608K	Fracture of upper limb
229	S62608P	Fracture of upper limb
229	S62608S	Fracture of upper limb
229	S62609A	Fracture of upper limb
229	S62609B	Fracture of upper limb
229	S62609D	Fracture of upper limb
229	S62609G	Fracture of upper limb
229	S62609K	Fracture of upper limb
229	S62609P	Fracture of upper limb
229	S62609S	Fracture of upper limb
229	S62610A	Fracture of upper limb
229	S62610B	Fracture of upper limb
229	S62610D	Fracture of upper limb
229	S62610G	Fracture of upper limb
229	S62610K	Fracture of upper limb
229	S62610P	Fracture of upper limb
229	S62610S	Fracture of upper limb
229	S62611A	Fracture of upper limb
229	S62611B	Fracture of upper limb
229	S62611D	Fracture of upper limb
229	S62611G	Fracture of upper limb
229	S62611K	Fracture of upper limb
229	S62611P	Fracture of upper limb
229	S62611S	Fracture of upper limb
229	S62612A	Fracture of upper limb
229	S62612B	Fracture of upper limb
229	S62612D	Fracture of upper limb
229	S62612G	Fracture of upper limb
229	S62612K	Fracture of upper limb
229	S62612P	Fracture of upper limb
229	S62612S	Fracture of upper limb
229	S62613A	Fracture of upper limb
229	S62613B	Fracture of upper limb
229	S62613D	Fracture of upper limb
229	S62613G	Fracture of upper limb
229	S62613K	Fracture of upper limb
229	S62613P	Fracture of upper limb
229	S62613S	Fracture of upper limb
229	S62614A	Fracture of upper limb
229	S62614B	Fracture of upper limb
229	S62614D	Fracture of upper limb
229	S62614G	Fracture of upper limb
229	S62614K	Fracture of upper limb
229	S62614P	Fracture of upper limb
229	S62614S	Fracture of upper limb
229	S62615A	Fracture of upper limb

229	S62615B	Fracture of upper limb
229	S62615D	Fracture of upper limb
229	S62615G	Fracture of upper limb
229	S62615K	Fracture of upper limb
229	S62615P	Fracture of upper limb
229	S62615S	Fracture of upper limb
229	S62616A	Fracture of upper limb
229	S62616B	Fracture of upper limb
229	S62616D	Fracture of upper limb
229	S62616G	Fracture of upper limb
229	S62616K	Fracture of upper limb
229	S62616P	Fracture of upper limb
229	S62616S	Fracture of upper limb
229	S62617A	Fracture of upper limb
229	S62617B	Fracture of upper limb
229	S62617D	Fracture of upper limb
229	S62617G	Fracture of upper limb
229	S62617K	Fracture of upper limb
229	S62617P	Fracture of upper limb
229	S62617S	Fracture of upper limb
229	S62618A	Fracture of upper limb
229	S62618B	Fracture of upper limb
229	S62618D	Fracture of upper limb
229	S62618G	Fracture of upper limb
229	S62618K	Fracture of upper limb
229	S62618P	Fracture of upper limb
229	S62618S	Fracture of upper limb
229	S62619A	Fracture of upper limb
229	S62619B	Fracture of upper limb
229	S62619D	Fracture of upper limb
229	S62619G	Fracture of upper limb
229	S62619K	Fracture of upper limb
229	S62619P	Fracture of upper limb
229	S62619S	Fracture of upper limb
229	S62620A	Fracture of upper limb
229	S62620B	Fracture of upper limb
229	S62620D	Fracture of upper limb
229	S62620G	Fracture of upper limb
229	S62620K	Fracture of upper limb
229	S62620P	Fracture of upper limb
229	S62620S	Fracture of upper limb
229	S62621A	Fracture of upper limb
229	S62621B	Fracture of upper limb
229	S62621D	Fracture of upper limb
229	S62621G	Fracture of upper limb
229	S62621K	Fracture of upper limb
229	S62621P	Fracture of upper limb
229	S62621S	Fracture of upper limb
229	S62622A	Fracture of upper limb
229	S62622B	Fracture of upper limb
229	S62622D	Fracture of upper limb

229	S62622G	Fracture of upper limb
229	S62622K	Fracture of upper limb
229	S62622P	Fracture of upper limb
229	S62622S	Fracture of upper limb
229	S62623A	Fracture of upper limb
229	S62623B	Fracture of upper limb
229	S62623D	Fracture of upper limb
229	S62623G	Fracture of upper limb
229	S62623K	Fracture of upper limb
229	S62623P	Fracture of upper limb
229	S62623S	Fracture of upper limb
229	S62624A	Fracture of upper limb
229	S62624B	Fracture of upper limb
229	S62624D	Fracture of upper limb
229	S62624G	Fracture of upper limb
229	S62624K	Fracture of upper limb
229	S62624P	Fracture of upper limb
229	S62624S	Fracture of upper limb
229	S62625A	Fracture of upper limb
229	S62625B	Fracture of upper limb
229	S62625D	Fracture of upper limb
229	S62625G	Fracture of upper limb
229	S62625K	Fracture of upper limb
229	S62625P	Fracture of upper limb
229	S62625S	Fracture of upper limb
229	S62626A	Fracture of upper limb
229	S62626B	Fracture of upper limb
229	S62626D	Fracture of upper limb
229	S62626G	Fracture of upper limb
229	S62626K	Fracture of upper limb
229	S62626P	Fracture of upper limb
229	S62626S	Fracture of upper limb
229	S62627A	Fracture of upper limb
229	S62627B	Fracture of upper limb
229	S62627D	Fracture of upper limb
229	S62627G	Fracture of upper limb
229	S62627K	Fracture of upper limb
229	S62627P	Fracture of upper limb
229	S62627S	Fracture of upper limb
229	S62628A	Fracture of upper limb
229	S62628B	Fracture of upper limb
229	S62628D	Fracture of upper limb
229	S62628G	Fracture of upper limb
229	S62628K	Fracture of upper limb
229	S62628P	Fracture of upper limb
229	S62628S	Fracture of upper limb
229	S62629A	Fracture of upper limb
229	S62629B	Fracture of upper limb
229	S62629D	Fracture of upper limb
229	S62629G	Fracture of upper limb
229	S62629K	Fracture of upper limb

229	S62629P	Fracture of upper limb
229	S62629S	Fracture of upper limb
229	S62630A	Fracture of upper limb
229	S62630B	Fracture of upper limb
229	S62630D	Fracture of upper limb
229	S62630G	Fracture of upper limb
229	S62630K	Fracture of upper limb
229	S62630P	Fracture of upper limb
229	S62630S	Fracture of upper limb
229	S62631A	Fracture of upper limb
229	S62631B	Fracture of upper limb
229	S62631D	Fracture of upper limb
229	S62631G	Fracture of upper limb
229	S62631K	Fracture of upper limb
229	S62631P	Fracture of upper limb
229	S62631S	Fracture of upper limb
229	S62632A	Fracture of upper limb
229	S62632B	Fracture of upper limb
229	S62632D	Fracture of upper limb
229	S62632G	Fracture of upper limb
229	S62632K	Fracture of upper limb
229	S62632P	Fracture of upper limb
229	S62632S	Fracture of upper limb
229	S62633A	Fracture of upper limb
229	S62633B	Fracture of upper limb
229	S62633D	Fracture of upper limb
229	S62633G	Fracture of upper limb
229	S62633K	Fracture of upper limb
229	S62633P	Fracture of upper limb
229	S62633S	Fracture of upper limb
229	S62634A	Fracture of upper limb
229	S62634B	Fracture of upper limb
229	S62634D	Fracture of upper limb
229	S62634G	Fracture of upper limb
229	S62634K	Fracture of upper limb
229	S62634P	Fracture of upper limb
229	S62634S	Fracture of upper limb
229	S62635A	Fracture of upper limb
229	S62635B	Fracture of upper limb
229	S62635D	Fracture of upper limb
229	S62635G	Fracture of upper limb
229	S62635K	Fracture of upper limb
229	S62635P	Fracture of upper limb
229	S62635S	Fracture of upper limb
229	S62636A	Fracture of upper limb
229	S62636B	Fracture of upper limb
229	S62636D	Fracture of upper limb
229	S62636G	Fracture of upper limb
229	S62636K	Fracture of upper limb
229	S62636P	Fracture of upper limb
229	S62636S	Fracture of upper limb

229	S62637A	Fracture of upper limb
229	S62637B	Fracture of upper limb
229	S62637D	Fracture of upper limb
229	S62637G	Fracture of upper limb
229	S62637K	Fracture of upper limb
229	S62637P	Fracture of upper limb
229	S62637S	Fracture of upper limb
229	S62638A	Fracture of upper limb
229	S62638B	Fracture of upper limb
229	S62638D	Fracture of upper limb
229	S62638G	Fracture of upper limb
229	S62638K	Fracture of upper limb
229	S62638P	Fracture of upper limb
229	S62638S	Fracture of upper limb
229	S62639A	Fracture of upper limb
229	S62639B	Fracture of upper limb
229	S62639D	Fracture of upper limb
229	S62639G	Fracture of upper limb
229	S62639K	Fracture of upper limb
229	S62639P	Fracture of upper limb
229	S62639S	Fracture of upper limb
229	S62640A	Fracture of upper limb
229	S62640B	Fracture of upper limb
229	S62640D	Fracture of upper limb
229	S62640G	Fracture of upper limb
229	S62640K	Fracture of upper limb
229	S62640P	Fracture of upper limb
229	S62640S	Fracture of upper limb
229	S62641A	Fracture of upper limb
229	S62641B	Fracture of upper limb
229	S62641D	Fracture of upper limb
229	S62641G	Fracture of upper limb
229	S62641K	Fracture of upper limb
229	S62641P	Fracture of upper limb
229	S62641S	Fracture of upper limb
229	S62642A	Fracture of upper limb
229	S62642B	Fracture of upper limb
229	S62642D	Fracture of upper limb
229	S62642G	Fracture of upper limb
229	S62642K	Fracture of upper limb
229	S62642P	Fracture of upper limb
229	S62642S	Fracture of upper limb
229	S62643A	Fracture of upper limb
229	S62643B	Fracture of upper limb
229	S62643D	Fracture of upper limb
229	S62643G	Fracture of upper limb
229	S62643K	Fracture of upper limb
229	S62643P	Fracture of upper limb
229	S62643S	Fracture of upper limb
229	S62644A	Fracture of upper limb
229	S62644B	Fracture of upper limb

229	S62644D	Fracture of upper limb
229	S62644G	Fracture of upper limb
229	S62644K	Fracture of upper limb
229	S62644P	Fracture of upper limb
229	S62644S	Fracture of upper limb
229	S62645A	Fracture of upper limb
229	S62645B	Fracture of upper limb
229	S62645D	Fracture of upper limb
229	S62645G	Fracture of upper limb
229	S62645K	Fracture of upper limb
229	S62645P	Fracture of upper limb
229	S62645S	Fracture of upper limb
229	S62646A	Fracture of upper limb
229	S62646B	Fracture of upper limb
229	S62646D	Fracture of upper limb
229	S62646G	Fracture of upper limb
229	S62646K	Fracture of upper limb
229	S62646P	Fracture of upper limb
229	S62646S	Fracture of upper limb
229	S62647A	Fracture of upper limb
229	S62647B	Fracture of upper limb
229	S62647D	Fracture of upper limb
229	S62647G	Fracture of upper limb
229	S62647K	Fracture of upper limb
229	S62647P	Fracture of upper limb
229	S62647S	Fracture of upper limb
229	S62648A	Fracture of upper limb
229	S62648B	Fracture of upper limb
229	S62648D	Fracture of upper limb
229	S62648G	Fracture of upper limb
229	S62648K	Fracture of upper limb
229	S62648P	Fracture of upper limb
229	S62648S	Fracture of upper limb
229	S62649A	Fracture of upper limb
229	S62649B	Fracture of upper limb
229	S62649D	Fracture of upper limb
229	S62649G	Fracture of upper limb
229	S62649K	Fracture of upper limb
229	S62649P	Fracture of upper limb
229	S62649S	Fracture of upper limb
229	S62650A	Fracture of upper limb
229	S62650B	Fracture of upper limb
229	S62650D	Fracture of upper limb
229	S62650G	Fracture of upper limb
229	S62650K	Fracture of upper limb
229	S62650P	Fracture of upper limb
229	S62650S	Fracture of upper limb
229	S62651A	Fracture of upper limb
229	S62651B	Fracture of upper limb
229	S62651D	Fracture of upper limb
229	S62651G	Fracture of upper limb

229	S62651K	Fracture of upper limb
229	S62651P	Fracture of upper limb
229	S62651S	Fracture of upper limb
229	S62652A	Fracture of upper limb
229	S62652B	Fracture of upper limb
229	S62652D	Fracture of upper limb
229	S62652G	Fracture of upper limb
229	S62652K	Fracture of upper limb
229	S62652P	Fracture of upper limb
229	S62652S	Fracture of upper limb
229	S62653A	Fracture of upper limb
229	S62653B	Fracture of upper limb
229	S62653D	Fracture of upper limb
229	S62653G	Fracture of upper limb
229	S62653K	Fracture of upper limb
229	S62653P	Fracture of upper limb
229	S62653S	Fracture of upper limb
229	S62654A	Fracture of upper limb
229	S62654B	Fracture of upper limb
229	S62654D	Fracture of upper limb
229	S62654G	Fracture of upper limb
229	S62654K	Fracture of upper limb
229	S62654P	Fracture of upper limb
229	S62654S	Fracture of upper limb
229	S62655A	Fracture of upper limb
229	S62655B	Fracture of upper limb
229	S62655D	Fracture of upper limb
229	S62655G	Fracture of upper limb
229	S62655K	Fracture of upper limb
229	S62655P	Fracture of upper limb
229	S62655S	Fracture of upper limb
229	S62656A	Fracture of upper limb
229	S62656B	Fracture of upper limb
229	S62656D	Fracture of upper limb
229	S62656G	Fracture of upper limb
229	S62656K	Fracture of upper limb
229	S62656P	Fracture of upper limb
229	S62656S	Fracture of upper limb
229	S62657A	Fracture of upper limb
229	S62657B	Fracture of upper limb
229	S62657D	Fracture of upper limb
229	S62657G	Fracture of upper limb
229	S62657K	Fracture of upper limb
229	S62657P	Fracture of upper limb
229	S62657S	Fracture of upper limb
229	S62658A	Fracture of upper limb
229	S62658B	Fracture of upper limb
229	S62658D	Fracture of upper limb
229	S62658G	Fracture of upper limb
229	S62658K	Fracture of upper limb
229	S62658P	Fracture of upper limb

229	S62658S	Fracture of upper limb
229	S62659A	Fracture of upper limb
229	S62659B	Fracture of upper limb
229	S62659D	Fracture of upper limb
229	S62659G	Fracture of upper limb
229	S62659K	Fracture of upper limb
229	S62659P	Fracture of upper limb
229	S62659S	Fracture of upper limb
229	S62660A	Fracture of upper limb
229	S62660B	Fracture of upper limb
229	S62660D	Fracture of upper limb
229	S62660G	Fracture of upper limb
229	S62660K	Fracture of upper limb
229	S62660P	Fracture of upper limb
229	S62660S	Fracture of upper limb
229	S62661A	Fracture of upper limb
229	S62661B	Fracture of upper limb
229	S62661D	Fracture of upper limb
229	S62661G	Fracture of upper limb
229	S62661K	Fracture of upper limb
229	S62661P	Fracture of upper limb
229	S62661S	Fracture of upper limb
229	S62662A	Fracture of upper limb
229	S62662B	Fracture of upper limb
229	S62662D	Fracture of upper limb
229	S62662G	Fracture of upper limb
229	S62662K	Fracture of upper limb
229	S62662P	Fracture of upper limb
229	S62662S	Fracture of upper limb
229	S62663A	Fracture of upper limb
229	S62663B	Fracture of upper limb
229	S62663D	Fracture of upper limb
229	S62663G	Fracture of upper limb
229	S62663K	Fracture of upper limb
229	S62663P	Fracture of upper limb
229	S62663S	Fracture of upper limb
229	S62664A	Fracture of upper limb
229	S62664B	Fracture of upper limb
229	S62664D	Fracture of upper limb
229	S62664G	Fracture of upper limb
229	S62664K	Fracture of upper limb
229	S62664P	Fracture of upper limb
229	S62664S	Fracture of upper limb
229	S62665A	Fracture of upper limb
229	S62665B	Fracture of upper limb
229	S62665D	Fracture of upper limb
229	S62665G	Fracture of upper limb
229	S62665K	Fracture of upper limb
229	S62665P	Fracture of upper limb
229	S62665S	Fracture of upper limb
229	S62666A	Fracture of upper limb

229	S62666B	Fracture of upper limb
229	S62666D	Fracture of upper limb
229	S62666G	Fracture of upper limb
229	S62666K	Fracture of upper limb
229	S62666P	Fracture of upper limb
229	S62666S	Fracture of upper limb
229	S62667A	Fracture of upper limb
229	S62667B	Fracture of upper limb
229	S62667D	Fracture of upper limb
229	S62667G	Fracture of upper limb
229	S62667K	Fracture of upper limb
229	S62667P	Fracture of upper limb
229	S62667S	Fracture of upper limb
229	S62668A	Fracture of upper limb
229	S62668B	Fracture of upper limb
229	S62668D	Fracture of upper limb
229	S62668G	Fracture of upper limb
229	S62668K	Fracture of upper limb
229	S62668P	Fracture of upper limb
229	S62668S	Fracture of upper limb
229	S62669A	Fracture of upper limb
229	S62669B	Fracture of upper limb
229	S62669D	Fracture of upper limb
229	S62669G	Fracture of upper limb
229	S62669K	Fracture of upper limb
229	S62669P	Fracture of upper limb
229	S62669S	Fracture of upper limb
229	S6290XA	Fracture of upper limb
229	S6290XB	Fracture of upper limb
229	S6290XD	Fracture of upper limb
229	S6290XG	Fracture of upper limb
229	S6290XK	Fracture of upper limb
229	S6290XP	Fracture of upper limb
229	S6290XS	Fracture of upper limb
229	S6291XA	Fracture of upper limb
229	S6291XB	Fracture of upper limb
229	S6291XD	Fracture of upper limb
229	S6291XG	Fracture of upper limb
229	S6291XK	Fracture of upper limb
229	S6291XP	Fracture of upper limb
229	S6291XS	Fracture of upper limb
229	S6292XA	Fracture of upper limb
229	S6292XB	Fracture of upper limb
229	S6292XD	Fracture of upper limb
229	S6292XG	Fracture of upper limb
229	S6292XK	Fracture of upper limb
229	S6292XP	Fracture of upper limb
229	S6292XS	Fracture of upper limb
230	M84361A	Fracture of lower limb
230	M84361D	Fracture of lower limb
230	M84361G	Fracture of lower limb

230	M84361K	Fracture of lower limb
230	M84361P	Fracture of lower limb
230	M84361S	Fracture of lower limb
230	M84362A	Fracture of lower limb
230	M84362D	Fracture of lower limb
230	M84362G	Fracture of lower limb
230	M84362K	Fracture of lower limb
230	M84362P	Fracture of lower limb
230	M84362S	Fracture of lower limb
230	M84363A	Fracture of lower limb
230	M84363D	Fracture of lower limb
230	M84363G	Fracture of lower limb
230	M84363K	Fracture of lower limb
230	M84363P	Fracture of lower limb
230	M84363S	Fracture of lower limb
230	M84364A	Fracture of lower limb
230	M84364D	Fracture of lower limb
230	M84364G	Fracture of lower limb
230	M84364K	Fracture of lower limb
230	M84364P	Fracture of lower limb
230	M84364S	Fracture of lower limb
230	M84369A	Fracture of lower limb
230	M84369D	Fracture of lower limb
230	M84369G	Fracture of lower limb
230	M84369K	Fracture of lower limb
230	M84369P	Fracture of lower limb
230	M84369S	Fracture of lower limb
230	M84371A	Fracture of lower limb
230	M84371D	Fracture of lower limb
230	M84371G	Fracture of lower limb
230	M84371K	Fracture of lower limb
230	M84371P	Fracture of lower limb
230	M84371S	Fracture of lower limb
230	M84372A	Fracture of lower limb
230	M84372D	Fracture of lower limb
230	M84372G	Fracture of lower limb
230	M84372K	Fracture of lower limb
230	M84372P	Fracture of lower limb
230	M84372S	Fracture of lower limb
230	M84373A	Fracture of lower limb
230	M84373D	Fracture of lower limb
230	M84373G	Fracture of lower limb
230	M84373K	Fracture of lower limb
230	M84373P	Fracture of lower limb
230	M84373S	Fracture of lower limb
230	M84374A	Fracture of lower limb
230	M84374D	Fracture of lower limb
230	M84374G	Fracture of lower limb
230	M84374K	Fracture of lower limb
230	M84374P	Fracture of lower limb
230	M84374S	Fracture of lower limb

230	M84375A	Fracture of lower limb
230	M84375D	Fracture of lower limb
230	M84375G	Fracture of lower limb
230	M84375K	Fracture of lower limb
230	M84375P	Fracture of lower limb
230	M84375S	Fracture of lower limb
230	M84376A	Fracture of lower limb
230	M84376D	Fracture of lower limb
230	M84376G	Fracture of lower limb
230	M84376K	Fracture of lower limb
230	M84376P	Fracture of lower limb
230	M84376S	Fracture of lower limb
230	M84377A	Fracture of lower limb
230	M84377D	Fracture of lower limb
230	M84377G	Fracture of lower limb
230	M84377K	Fracture of lower limb
230	M84377P	Fracture of lower limb
230	M84377S	Fracture of lower limb
230	M84378A	Fracture of lower limb
230	M84378D	Fracture of lower limb
230	M84378G	Fracture of lower limb
230	M84378K	Fracture of lower limb
230	M84378P	Fracture of lower limb
230	M84378S	Fracture of lower limb
230	M84379A	Fracture of lower limb
230	M84379D	Fracture of lower limb
230	M84379G	Fracture of lower limb
230	M84379K	Fracture of lower limb
230	M84379P	Fracture of lower limb
230	M84379S	Fracture of lower limb
230	S72301A	Fracture of lower limb
230	S72301B	Fracture of lower limb
230	S72301C	Fracture of lower limb
230	S72301D	Fracture of lower limb
230	S72301E	Fracture of lower limb
230	S72301F	Fracture of lower limb
230	S72301G	Fracture of lower limb
230	S72301H	Fracture of lower limb
230	S72301J	Fracture of lower limb
230	S72301K	Fracture of lower limb
230	S72301M	Fracture of lower limb
230	S72301N	Fracture of lower limb
230	S72301P	Fracture of lower limb
230	S72301Q	Fracture of lower limb
230	S72301R	Fracture of lower limb
230	S72301S	Fracture of lower limb
230	S72302A	Fracture of lower limb
230	S72302B	Fracture of lower limb
230	S72302C	Fracture of lower limb
230	S72302D	Fracture of lower limb
230	S72302E	Fracture of lower limb

230	S72302F	Fracture of lower limb
230	S72302G	Fracture of lower limb
230	S72302H	Fracture of lower limb
230	S72302J	Fracture of lower limb
230	S72302K	Fracture of lower limb
230	S72302M	Fracture of lower limb
230	S72302N	Fracture of lower limb
230	S72302P	Fracture of lower limb
230	S72302Q	Fracture of lower limb
230	S72302R	Fracture of lower limb
230	S72302S	Fracture of lower limb
230	S72309A	Fracture of lower limb
230	S72309B	Fracture of lower limb
230	S72309C	Fracture of lower limb
230	S72309D	Fracture of lower limb
230	S72309E	Fracture of lower limb
230	S72309F	Fracture of lower limb
230	S72309G	Fracture of lower limb
230	S72309H	Fracture of lower limb
230	S72309J	Fracture of lower limb
230	S72309K	Fracture of lower limb
230	S72309M	Fracture of lower limb
230	S72309N	Fracture of lower limb
230	S72309P	Fracture of lower limb
230	S72309Q	Fracture of lower limb
230	S72309R	Fracture of lower limb
230	S72309S	Fracture of lower limb
230	S72321A	Fracture of lower limb
230	S72321B	Fracture of lower limb
230	S72321C	Fracture of lower limb
230	S72321D	Fracture of lower limb
230	S72321E	Fracture of lower limb
230	S72321F	Fracture of lower limb
230	S72321G	Fracture of lower limb
230	S72321H	Fracture of lower limb
230	S72321J	Fracture of lower limb
230	S72321K	Fracture of lower limb
230	S72321M	Fracture of lower limb
230	S72321N	Fracture of lower limb
230	S72321P	Fracture of lower limb
230	S72321Q	Fracture of lower limb
230	S72321R	Fracture of lower limb
230	S72321S	Fracture of lower limb
230	S72322A	Fracture of lower limb
230	S72322B	Fracture of lower limb
230	S72322C	Fracture of lower limb
230	S72322D	Fracture of lower limb
230	S72322E	Fracture of lower limb
230	S72322F	Fracture of lower limb
230	S72322G	Fracture of lower limb
230	S72322H	Fracture of lower limb

230	S72322J	Fracture of lower limb
230	S72322K	Fracture of lower limb
230	S72322M	Fracture of lower limb
230	S72322N	Fracture of lower limb
230	S72322P	Fracture of lower limb
230	S72322Q	Fracture of lower limb
230	S72322R	Fracture of lower limb
230	S72322S	Fracture of lower limb
230	S72323A	Fracture of lower limb
230	S72323B	Fracture of lower limb
230	S72323C	Fracture of lower limb
230	S72323D	Fracture of lower limb
230	S72323E	Fracture of lower limb
230	S72323F	Fracture of lower limb
230	S72323G	Fracture of lower limb
230	S72323H	Fracture of lower limb
230	S72323J	Fracture of lower limb
230	S72323K	Fracture of lower limb
230	S72323M	Fracture of lower limb
230	S72323N	Fracture of lower limb
230	S72323P	Fracture of lower limb
230	S72323Q	Fracture of lower limb
230	S72323R	Fracture of lower limb
230	S72323S	Fracture of lower limb
230	S72324A	Fracture of lower limb
230	S72324B	Fracture of lower limb
230	S72324C	Fracture of lower limb
230	S72324D	Fracture of lower limb
230	S72324E	Fracture of lower limb
230	S72324F	Fracture of lower limb
230	S72324G	Fracture of lower limb
230	S72324H	Fracture of lower limb
230	S72324J	Fracture of lower limb
230	S72324K	Fracture of lower limb
230	S72324M	Fracture of lower limb
230	S72324N	Fracture of lower limb
230	S72324P	Fracture of lower limb
230	S72324Q	Fracture of lower limb
230	S72324R	Fracture of lower limb
230	S72324S	Fracture of lower limb
230	S72325A	Fracture of lower limb
230	S72325B	Fracture of lower limb
230	S72325C	Fracture of lower limb
230	S72325D	Fracture of lower limb
230	S72325E	Fracture of lower limb
230	S72325F	Fracture of lower limb
230	S72325G	Fracture of lower limb
230	S72325H	Fracture of lower limb
230	S72325J	Fracture of lower limb
230	S72325K	Fracture of lower limb
230	S72325M	Fracture of lower limb

230	S72325N	Fracture of lower limb
230	S72325P	Fracture of lower limb
230	S72325Q	Fracture of lower limb
230	S72325R	Fracture of lower limb
230	S72325S	Fracture of lower limb
230	S72326A	Fracture of lower limb
230	S72326B	Fracture of lower limb
230	S72326C	Fracture of lower limb
230	S72326D	Fracture of lower limb
230	S72326E	Fracture of lower limb
230	S72326F	Fracture of lower limb
230	S72326G	Fracture of lower limb
230	S72326H	Fracture of lower limb
230	S72326J	Fracture of lower limb
230	S72326K	Fracture of lower limb
230	S72326M	Fracture of lower limb
230	S72326N	Fracture of lower limb
230	S72326P	Fracture of lower limb
230	S72326Q	Fracture of lower limb
230	S72326R	Fracture of lower limb
230	S72326S	Fracture of lower limb
230	S72331A	Fracture of lower limb
230	S72331B	Fracture of lower limb
230	S72331C	Fracture of lower limb
230	S72331D	Fracture of lower limb
230	S72331E	Fracture of lower limb
230	S72331F	Fracture of lower limb
230	S72331G	Fracture of lower limb
230	S72331H	Fracture of lower limb
230	S72331J	Fracture of lower limb
230	S72331K	Fracture of lower limb
230	S72331M	Fracture of lower limb
230	S72331N	Fracture of lower limb
230	S72331P	Fracture of lower limb
230	S72331Q	Fracture of lower limb
230	S72331R	Fracture of lower limb
230	S72331S	Fracture of lower limb
230	S72332A	Fracture of lower limb
230	S72332B	Fracture of lower limb
230	S72332C	Fracture of lower limb
230	S72332D	Fracture of lower limb
230	S72332E	Fracture of lower limb
230	S72332F	Fracture of lower limb
230	S72332G	Fracture of lower limb
230	S72332H	Fracture of lower limb
230	S72332J	Fracture of lower limb
230	S72332K	Fracture of lower limb
230	S72332M	Fracture of lower limb
230	S72332N	Fracture of lower limb
230	S72332P	Fracture of lower limb
230	S72332Q	Fracture of lower limb

230	S72332R	Fracture of lower limb
230	S72332S	Fracture of lower limb
230	S72333A	Fracture of lower limb
230	S72333B	Fracture of lower limb
230	S72333C	Fracture of lower limb
230	S72333D	Fracture of lower limb
230	S72333E	Fracture of lower limb
230	S72333F	Fracture of lower limb
230	S72333G	Fracture of lower limb
230	S72333H	Fracture of lower limb
230	S72333J	Fracture of lower limb
230	S72333K	Fracture of lower limb
230	S72333M	Fracture of lower limb
230	S72333N	Fracture of lower limb
230	S72333P	Fracture of lower limb
230	S72333Q	Fracture of lower limb
230	S72333R	Fracture of lower limb
230	S72333S	Fracture of lower limb
230	S72334A	Fracture of lower limb
230	S72334B	Fracture of lower limb
230	S72334C	Fracture of lower limb
230	S72334D	Fracture of lower limb
230	S72334E	Fracture of lower limb
230	S72334F	Fracture of lower limb
230	S72334G	Fracture of lower limb
230	S72334H	Fracture of lower limb
230	S72334J	Fracture of lower limb
230	S72334K	Fracture of lower limb
230	S72334M	Fracture of lower limb
230	S72334N	Fracture of lower limb
230	S72334P	Fracture of lower limb
230	S72334Q	Fracture of lower limb
230	S72334R	Fracture of lower limb
230	S72334S	Fracture of lower limb
230	S72335A	Fracture of lower limb
230	S72335B	Fracture of lower limb
230	S72335C	Fracture of lower limb
230	S72335D	Fracture of lower limb
230	S72335E	Fracture of lower limb
230	S72335F	Fracture of lower limb
230	S72335G	Fracture of lower limb
230	S72335H	Fracture of lower limb
230	S72335J	Fracture of lower limb
230	S72335K	Fracture of lower limb
230	S72335M	Fracture of lower limb
230	S72335N	Fracture of lower limb
230	S72335P	Fracture of lower limb
230	S72335Q	Fracture of lower limb
230	S72335R	Fracture of lower limb
230	S72335S	Fracture of lower limb
230	S72336A	Fracture of lower limb

230	S72336B	Fracture of lower limb
230	S72336C	Fracture of lower limb
230	S72336D	Fracture of lower limb
230	S72336E	Fracture of lower limb
230	S72336F	Fracture of lower limb
230	S72336G	Fracture of lower limb
230	S72336H	Fracture of lower limb
230	S72336J	Fracture of lower limb
230	S72336K	Fracture of lower limb
230	S72336M	Fracture of lower limb
230	S72336N	Fracture of lower limb
230	S72336P	Fracture of lower limb
230	S72336Q	Fracture of lower limb
230	S72336R	Fracture of lower limb
230	S72336S	Fracture of lower limb
230	S72341A	Fracture of lower limb
230	S72341B	Fracture of lower limb
230	S72341C	Fracture of lower limb
230	S72341D	Fracture of lower limb
230	S72341E	Fracture of lower limb
230	S72341F	Fracture of lower limb
230	S72341G	Fracture of lower limb
230	S72341H	Fracture of lower limb
230	S72341J	Fracture of lower limb
230	S72341K	Fracture of lower limb
230	S72341M	Fracture of lower limb
230	S72341N	Fracture of lower limb
230	S72341P	Fracture of lower limb
230	S72341Q	Fracture of lower limb
230	S72341R	Fracture of lower limb
230	S72341S	Fracture of lower limb
230	S72342A	Fracture of lower limb
230	S72342B	Fracture of lower limb
230	S72342C	Fracture of lower limb
230	S72342D	Fracture of lower limb
230	S72342E	Fracture of lower limb
230	S72342F	Fracture of lower limb
230	S72342G	Fracture of lower limb
230	S72342H	Fracture of lower limb
230	S72342J	Fracture of lower limb
230	S72342K	Fracture of lower limb
230	S72342M	Fracture of lower limb
230	S72342N	Fracture of lower limb
230	S72342P	Fracture of lower limb
230	S72342Q	Fracture of lower limb
230	S72342R	Fracture of lower limb
230	S72342S	Fracture of lower limb
230	S72343A	Fracture of lower limb
230	S72343B	Fracture of lower limb
230	S72343C	Fracture of lower limb
230	S72343D	Fracture of lower limb

230	S72343E	Fracture of lower limb
230	S72343F	Fracture of lower limb
230	S72343G	Fracture of lower limb
230	S72343H	Fracture of lower limb
230	S72343J	Fracture of lower limb
230	S72343K	Fracture of lower limb
230	S72343M	Fracture of lower limb
230	S72343N	Fracture of lower limb
230	S72343P	Fracture of lower limb
230	S72343Q	Fracture of lower limb
230	S72343R	Fracture of lower limb
230	S72343S	Fracture of lower limb
230	S72344A	Fracture of lower limb
230	S72344B	Fracture of lower limb
230	S72344C	Fracture of lower limb
230	S72344D	Fracture of lower limb
230	S72344E	Fracture of lower limb
230	S72344F	Fracture of lower limb
230	S72344G	Fracture of lower limb
230	S72344H	Fracture of lower limb
230	S72344J	Fracture of lower limb
230	S72344K	Fracture of lower limb
230	S72344M	Fracture of lower limb
230	S72344N	Fracture of lower limb
230	S72344P	Fracture of lower limb
230	S72344Q	Fracture of lower limb
230	S72344R	Fracture of lower limb
230	S72344S	Fracture of lower limb
230	S72345A	Fracture of lower limb
230	S72345B	Fracture of lower limb
230	S72345C	Fracture of lower limb
230	S72345D	Fracture of lower limb
230	S72345E	Fracture of lower limb
230	S72345F	Fracture of lower limb
230	S72345G	Fracture of lower limb
230	S72345H	Fracture of lower limb
230	S72345J	Fracture of lower limb
230	S72345K	Fracture of lower limb
230	S72345M	Fracture of lower limb
230	S72345N	Fracture of lower limb
230	S72345P	Fracture of lower limb
230	S72345Q	Fracture of lower limb
230	S72345R	Fracture of lower limb
230	S72345S	Fracture of lower limb
230	S72346A	Fracture of lower limb
230	S72346B	Fracture of lower limb
230	S72346C	Fracture of lower limb
230	S72346D	Fracture of lower limb
230	S72346E	Fracture of lower limb
230	S72346F	Fracture of lower limb
230	S72346G	Fracture of lower limb

230	S72346H	Fracture of lower limb
230	S72346J	Fracture of lower limb
230	S72346K	Fracture of lower limb
230	S72346M	Fracture of lower limb
230	S72346N	Fracture of lower limb
230	S72346P	Fracture of lower limb
230	S72346Q	Fracture of lower limb
230	S72346R	Fracture of lower limb
230	S72346S	Fracture of lower limb
230	S72351A	Fracture of lower limb
230	S72351B	Fracture of lower limb
230	S72351C	Fracture of lower limb
230	S72351D	Fracture of lower limb
230	S72351E	Fracture of lower limb
230	S72351F	Fracture of lower limb
230	S72351G	Fracture of lower limb
230	S72351H	Fracture of lower limb
230	S72351J	Fracture of lower limb
230	S72351K	Fracture of lower limb
230	S72351M	Fracture of lower limb
230	S72351N	Fracture of lower limb
230	S72351P	Fracture of lower limb
230	S72351Q	Fracture of lower limb
230	S72351R	Fracture of lower limb
230	S72351S	Fracture of lower limb
230	S72352A	Fracture of lower limb
230	S72352B	Fracture of lower limb
230	S72352C	Fracture of lower limb
230	S72352D	Fracture of lower limb
230	S72352E	Fracture of lower limb
230	S72352F	Fracture of lower limb
230	S72352G	Fracture of lower limb
230	S72352H	Fracture of lower limb
230	S72352J	Fracture of lower limb
230	S72352K	Fracture of lower limb
230	S72352M	Fracture of lower limb
230	S72352N	Fracture of lower limb
230	S72352P	Fracture of lower limb
230	S72352Q	Fracture of lower limb
230	S72352R	Fracture of lower limb
230	S72352S	Fracture of lower limb
230	S72353A	Fracture of lower limb
230	S72353B	Fracture of lower limb
230	S72353C	Fracture of lower limb
230	S72353D	Fracture of lower limb
230	S72353E	Fracture of lower limb
230	S72353F	Fracture of lower limb
230	S72353G	Fracture of lower limb
230	S72353H	Fracture of lower limb
230	S72353J	Fracture of lower limb
230	S72353K	Fracture of lower limb

230	S72353M	Fracture of lower limb
230	S72353N	Fracture of lower limb
230	S72353P	Fracture of lower limb
230	S72353Q	Fracture of lower limb
230	S72353R	Fracture of lower limb
230	S72353S	Fracture of lower limb
230	S72354A	Fracture of lower limb
230	S72354B	Fracture of lower limb
230	S72354C	Fracture of lower limb
230	S72354D	Fracture of lower limb
230	S72354E	Fracture of lower limb
230	S72354F	Fracture of lower limb
230	S72354G	Fracture of lower limb
230	S72354H	Fracture of lower limb
230	S72354J	Fracture of lower limb
230	S72354K	Fracture of lower limb
230	S72354M	Fracture of lower limb
230	S72354N	Fracture of lower limb
230	S72354P	Fracture of lower limb
230	S72354Q	Fracture of lower limb
230	S72354R	Fracture of lower limb
230	S72354S	Fracture of lower limb
230	S72355A	Fracture of lower limb
230	S72355B	Fracture of lower limb
230	S72355C	Fracture of lower limb
230	S72355D	Fracture of lower limb
230	S72355E	Fracture of lower limb
230	S72355F	Fracture of lower limb
230	S72355G	Fracture of lower limb
230	S72355H	Fracture of lower limb
230	S72355J	Fracture of lower limb
230	S72355K	Fracture of lower limb
230	S72355M	Fracture of lower limb
230	S72355N	Fracture of lower limb
230	S72355P	Fracture of lower limb
230	S72355Q	Fracture of lower limb
230	S72355R	Fracture of lower limb
230	S72355S	Fracture of lower limb
230	S72356A	Fracture of lower limb
230	S72356B	Fracture of lower limb
230	S72356C	Fracture of lower limb
230	S72356D	Fracture of lower limb
230	S72356E	Fracture of lower limb
230	S72356F	Fracture of lower limb
230	S72356G	Fracture of lower limb
230	S72356H	Fracture of lower limb
230	S72356J	Fracture of lower limb
230	S72356K	Fracture of lower limb
230	S72356M	Fracture of lower limb
230	S72356N	Fracture of lower limb
230	S72356P	Fracture of lower limb

230	S72356Q	Fracture of lower limb
230	S72356R	Fracture of lower limb
230	S72356S	Fracture of lower limb
230	S72361A	Fracture of lower limb
230	S72361B	Fracture of lower limb
230	S72361C	Fracture of lower limb
230	S72361D	Fracture of lower limb
230	S72361E	Fracture of lower limb
230	S72361F	Fracture of lower limb
230	S72361G	Fracture of lower limb
230	S72361H	Fracture of lower limb
230	S72361J	Fracture of lower limb
230	S72361K	Fracture of lower limb
230	S72361M	Fracture of lower limb
230	S72361N	Fracture of lower limb
230	S72361P	Fracture of lower limb
230	S72361Q	Fracture of lower limb
230	S72361R	Fracture of lower limb
230	S72361S	Fracture of lower limb
230	S72362A	Fracture of lower limb
230	S72362B	Fracture of lower limb
230	S72362C	Fracture of lower limb
230	S72362D	Fracture of lower limb
230	S72362E	Fracture of lower limb
230	S72362F	Fracture of lower limb
230	S72362G	Fracture of lower limb
230	S72362H	Fracture of lower limb
230	S72362J	Fracture of lower limb
230	S72362K	Fracture of lower limb
230	S72362M	Fracture of lower limb
230	S72362N	Fracture of lower limb
230	S72362P	Fracture of lower limb
230	S72362Q	Fracture of lower limb
230	S72362R	Fracture of lower limb
230	S72362S	Fracture of lower limb
230	S72363A	Fracture of lower limb
230	S72363B	Fracture of lower limb
230	S72363C	Fracture of lower limb
230	S72363D	Fracture of lower limb
230	S72363E	Fracture of lower limb
230	S72363F	Fracture of lower limb
230	S72363G	Fracture of lower limb
230	S72363H	Fracture of lower limb
230	S72363J	Fracture of lower limb
230	S72363K	Fracture of lower limb
230	S72363M	Fracture of lower limb
230	S72363N	Fracture of lower limb
230	S72363P	Fracture of lower limb
230	S72363Q	Fracture of lower limb
230	S72363R	Fracture of lower limb
230	S72363S	Fracture of lower limb

230	S72364A	Fracture of lower limb
230	S72364B	Fracture of lower limb
230	S72364C	Fracture of lower limb
230	S72364D	Fracture of lower limb
230	S72364E	Fracture of lower limb
230	S72364F	Fracture of lower limb
230	S72364G	Fracture of lower limb
230	S72364H	Fracture of lower limb
230	S72364J	Fracture of lower limb
230	S72364K	Fracture of lower limb
230	S72364M	Fracture of lower limb
230	S72364N	Fracture of lower limb
230	S72364P	Fracture of lower limb
230	S72364Q	Fracture of lower limb
230	S72364R	Fracture of lower limb
230	S72364S	Fracture of lower limb
230	S72365A	Fracture of lower limb
230	S72365B	Fracture of lower limb
230	S72365C	Fracture of lower limb
230	S72365D	Fracture of lower limb
230	S72365E	Fracture of lower limb
230	S72365F	Fracture of lower limb
230	S72365G	Fracture of lower limb
230	S72365H	Fracture of lower limb
230	S72365J	Fracture of lower limb
230	S72365K	Fracture of lower limb
230	S72365M	Fracture of lower limb
230	S72365N	Fracture of lower limb
230	S72365P	Fracture of lower limb
230	S72365Q	Fracture of lower limb
230	S72365R	Fracture of lower limb
230	S72365S	Fracture of lower limb
230	S72366A	Fracture of lower limb
230	S72366B	Fracture of lower limb
230	S72366C	Fracture of lower limb
230	S72366D	Fracture of lower limb
230	S72366E	Fracture of lower limb
230	S72366F	Fracture of lower limb
230	S72366G	Fracture of lower limb
230	S72366H	Fracture of lower limb
230	S72366J	Fracture of lower limb
230	S72366K	Fracture of lower limb
230	S72366M	Fracture of lower limb
230	S72366N	Fracture of lower limb
230	S72366P	Fracture of lower limb
230	S72366Q	Fracture of lower limb
230	S72366R	Fracture of lower limb
230	S72366S	Fracture of lower limb
230	S72391A	Fracture of lower limb
230	S72391B	Fracture of lower limb
230	S72391C	Fracture of lower limb

230	S72391D	Fracture of lower limb
230	S72391E	Fracture of lower limb
230	S72391F	Fracture of lower limb
230	S72391G	Fracture of lower limb
230	S72391H	Fracture of lower limb
230	S72391J	Fracture of lower limb
230	S72391K	Fracture of lower limb
230	S72391M	Fracture of lower limb
230	S72391N	Fracture of lower limb
230	S72391P	Fracture of lower limb
230	S72391Q	Fracture of lower limb
230	S72391R	Fracture of lower limb
230	S72391S	Fracture of lower limb
230	S72392A	Fracture of lower limb
230	S72392B	Fracture of lower limb
230	S72392C	Fracture of lower limb
230	S72392D	Fracture of lower limb
230	S72392E	Fracture of lower limb
230	S72392F	Fracture of lower limb
230	S72392G	Fracture of lower limb
230	S72392H	Fracture of lower limb
230	S72392J	Fracture of lower limb
230	S72392K	Fracture of lower limb
230	S72392M	Fracture of lower limb
230	S72392N	Fracture of lower limb
230	S72392P	Fracture of lower limb
230	S72392Q	Fracture of lower limb
230	S72392R	Fracture of lower limb
230	S72392S	Fracture of lower limb
230	S72399A	Fracture of lower limb
230	S72399B	Fracture of lower limb
230	S72399C	Fracture of lower limb
230	S72399D	Fracture of lower limb
230	S72399E	Fracture of lower limb
230	S72399F	Fracture of lower limb
230	S72399G	Fracture of lower limb
230	S72399H	Fracture of lower limb
230	S72399J	Fracture of lower limb
230	S72399K	Fracture of lower limb
230	S72399M	Fracture of lower limb
230	S72399N	Fracture of lower limb
230	S72399P	Fracture of lower limb
230	S72399Q	Fracture of lower limb
230	S72399R	Fracture of lower limb
230	S72399S	Fracture of lower limb
230	S72401A	Fracture of lower limb
230	S72401B	Fracture of lower limb
230	S72401C	Fracture of lower limb
230	S72401D	Fracture of lower limb
230	S72401E	Fracture of lower limb
230	S72401F	Fracture of lower limb

230	S72401G	Fracture of lower limb
230	S72401H	Fracture of lower limb
230	S72401J	Fracture of lower limb
230	S72401K	Fracture of lower limb
230	S72401M	Fracture of lower limb
230	S72401N	Fracture of lower limb
230	S72401P	Fracture of lower limb
230	S72401Q	Fracture of lower limb
230	S72401R	Fracture of lower limb
230	S72401S	Fracture of lower limb
230	S72402A	Fracture of lower limb
230	S72402B	Fracture of lower limb
230	S72402C	Fracture of lower limb
230	S72402D	Fracture of lower limb
230	S72402E	Fracture of lower limb
230	S72402F	Fracture of lower limb
230	S72402G	Fracture of lower limb
230	S72402H	Fracture of lower limb
230	S72402J	Fracture of lower limb
230	S72402K	Fracture of lower limb
230	S72402M	Fracture of lower limb
230	S72402N	Fracture of lower limb
230	S72402P	Fracture of lower limb
230	S72402Q	Fracture of lower limb
230	S72402R	Fracture of lower limb
230	S72402S	Fracture of lower limb
230	S72409A	Fracture of lower limb
230	S72409B	Fracture of lower limb
230	S72409C	Fracture of lower limb
230	S72409D	Fracture of lower limb
230	S72409E	Fracture of lower limb
230	S72409F	Fracture of lower limb
230	S72409G	Fracture of lower limb
230	S72409H	Fracture of lower limb
230	S72409J	Fracture of lower limb
230	S72409K	Fracture of lower limb
230	S72409M	Fracture of lower limb
230	S72409N	Fracture of lower limb
230	S72409P	Fracture of lower limb
230	S72409Q	Fracture of lower limb
230	S72409R	Fracture of lower limb
230	S72409S	Fracture of lower limb
230	S72411A	Fracture of lower limb
230	S72411B	Fracture of lower limb
230	S72411C	Fracture of lower limb
230	S72411D	Fracture of lower limb
230	S72411E	Fracture of lower limb
230	S72411F	Fracture of lower limb
230	S72411G	Fracture of lower limb
230	S72411H	Fracture of lower limb
230	S72411J	Fracture of lower limb

230	S72411K	Fracture of lower limb
230	S72411M	Fracture of lower limb
230	S72411N	Fracture of lower limb
230	S72411P	Fracture of lower limb
230	S72411Q	Fracture of lower limb
230	S72411R	Fracture of lower limb
230	S72411S	Fracture of lower limb
230	S72412A	Fracture of lower limb
230	S72412B	Fracture of lower limb
230	S72412C	Fracture of lower limb
230	S72412D	Fracture of lower limb
230	S72412E	Fracture of lower limb
230	S72412F	Fracture of lower limb
230	S72412G	Fracture of lower limb
230	S72412H	Fracture of lower limb
230	S72412J	Fracture of lower limb
230	S72412K	Fracture of lower limb
230	S72412M	Fracture of lower limb
230	S72412N	Fracture of lower limb
230	S72412P	Fracture of lower limb
230	S72412Q	Fracture of lower limb
230	S72412R	Fracture of lower limb
230	S72412S	Fracture of lower limb
230	S72413A	Fracture of lower limb
230	S72413B	Fracture of lower limb
230	S72413C	Fracture of lower limb
230	S72413D	Fracture of lower limb
230	S72413E	Fracture of lower limb
230	S72413F	Fracture of lower limb
230	S72413G	Fracture of lower limb
230	S72413H	Fracture of lower limb
230	S72413J	Fracture of lower limb
230	S72413K	Fracture of lower limb
230	S72413M	Fracture of lower limb
230	S72413N	Fracture of lower limb
230	S72413P	Fracture of lower limb
230	S72413Q	Fracture of lower limb
230	S72413R	Fracture of lower limb
230	S72413S	Fracture of lower limb
230	S72414A	Fracture of lower limb
230	S72414B	Fracture of lower limb
230	S72414C	Fracture of lower limb
230	S72414D	Fracture of lower limb
230	S72414E	Fracture of lower limb
230	S72414F	Fracture of lower limb
230	S72414G	Fracture of lower limb
230	S72414H	Fracture of lower limb
230	S72414J	Fracture of lower limb
230	S72414K	Fracture of lower limb
230	S72414M	Fracture of lower limb
230	S72414N	Fracture of lower limb

230	S72414P	Fracture of lower limb
230	S72414Q	Fracture of lower limb
230	S72414R	Fracture of lower limb
230	S72414S	Fracture of lower limb
230	S72415A	Fracture of lower limb
230	S72415B	Fracture of lower limb
230	S72415C	Fracture of lower limb
230	S72415D	Fracture of lower limb
230	S72415E	Fracture of lower limb
230	S72415F	Fracture of lower limb
230	S72415G	Fracture of lower limb
230	S72415H	Fracture of lower limb
230	S72415J	Fracture of lower limb
230	S72415K	Fracture of lower limb
230	S72415M	Fracture of lower limb
230	S72415N	Fracture of lower limb
230	S72415P	Fracture of lower limb
230	S72415Q	Fracture of lower limb
230	S72415R	Fracture of lower limb
230	S72415S	Fracture of lower limb
230	S72416A	Fracture of lower limb
230	S72416B	Fracture of lower limb
230	S72416C	Fracture of lower limb
230	S72416D	Fracture of lower limb
230	S72416E	Fracture of lower limb
230	S72416F	Fracture of lower limb
230	S72416G	Fracture of lower limb
230	S72416H	Fracture of lower limb
230	S72416J	Fracture of lower limb
230	S72416K	Fracture of lower limb
230	S72416M	Fracture of lower limb
230	S72416N	Fracture of lower limb
230	S72416P	Fracture of lower limb
230	S72416Q	Fracture of lower limb
230	S72416R	Fracture of lower limb
230	S72416S	Fracture of lower limb
230	S72421A	Fracture of lower limb
230	S72421B	Fracture of lower limb
230	S72421C	Fracture of lower limb
230	S72421D	Fracture of lower limb
230	S72421E	Fracture of lower limb
230	S72421F	Fracture of lower limb
230	S72421G	Fracture of lower limb
230	S72421H	Fracture of lower limb
230	S72421J	Fracture of lower limb
230	S72421K	Fracture of lower limb
230	S72421M	Fracture of lower limb
230	S72421N	Fracture of lower limb
230	S72421P	Fracture of lower limb
230	S72421Q	Fracture of lower limb
230	S72421R	Fracture of lower limb

230	S72421S	Fracture of lower limb
230	S72422A	Fracture of lower limb
230	S72422B	Fracture of lower limb
230	S72422C	Fracture of lower limb
230	S72422D	Fracture of lower limb
230	S72422E	Fracture of lower limb
230	S72422F	Fracture of lower limb
230	S72422G	Fracture of lower limb
230	S72422H	Fracture of lower limb
230	S72422J	Fracture of lower limb
230	S72422K	Fracture of lower limb
230	S72422M	Fracture of lower limb
230	S72422N	Fracture of lower limb
230	S72422P	Fracture of lower limb
230	S72422Q	Fracture of lower limb
230	S72422R	Fracture of lower limb
230	S72422S	Fracture of lower limb
230	S72423A	Fracture of lower limb
230	S72423B	Fracture of lower limb
230	S72423C	Fracture of lower limb
230	S72423D	Fracture of lower limb
230	S72423E	Fracture of lower limb
230	S72423F	Fracture of lower limb
230	S72423G	Fracture of lower limb
230	S72423H	Fracture of lower limb
230	S72423J	Fracture of lower limb
230	S72423K	Fracture of lower limb
230	S72423M	Fracture of lower limb
230	S72423N	Fracture of lower limb
230	S72423P	Fracture of lower limb
230	S72423Q	Fracture of lower limb
230	S72423R	Fracture of lower limb
230	S72423S	Fracture of lower limb
230	S72424A	Fracture of lower limb
230	S72424B	Fracture of lower limb
230	S72424C	Fracture of lower limb
230	S72424D	Fracture of lower limb
230	S72424E	Fracture of lower limb
230	S72424F	Fracture of lower limb
230	S72424G	Fracture of lower limb
230	S72424H	Fracture of lower limb
230	S72424J	Fracture of lower limb
230	S72424K	Fracture of lower limb
230	S72424M	Fracture of lower limb
230	S72424N	Fracture of lower limb
230	S72424P	Fracture of lower limb
230	S72424Q	Fracture of lower limb
230	S72424R	Fracture of lower limb
230	S72424S	Fracture of lower limb
230	S72425A	Fracture of lower limb
230	S72425B	Fracture of lower limb

230	S72425C	Fracture of lower limb
230	S72425D	Fracture of lower limb
230	S72425E	Fracture of lower limb
230	S72425F	Fracture of lower limb
230	S72425G	Fracture of lower limb
230	S72425H	Fracture of lower limb
230	S72425J	Fracture of lower limb
230	S72425K	Fracture of lower limb
230	S72425M	Fracture of lower limb
230	S72425N	Fracture of lower limb
230	S72425P	Fracture of lower limb
230	S72425Q	Fracture of lower limb
230	S72425R	Fracture of lower limb
230	S72425S	Fracture of lower limb
230	S72426A	Fracture of lower limb
230	S72426B	Fracture of lower limb
230	S72426C	Fracture of lower limb
230	S72426D	Fracture of lower limb
230	S72426E	Fracture of lower limb
230	S72426F	Fracture of lower limb
230	S72426G	Fracture of lower limb
230	S72426H	Fracture of lower limb
230	S72426J	Fracture of lower limb
230	S72426K	Fracture of lower limb
230	S72426M	Fracture of lower limb
230	S72426N	Fracture of lower limb
230	S72426P	Fracture of lower limb
230	S72426Q	Fracture of lower limb
230	S72426R	Fracture of lower limb
230	S72426S	Fracture of lower limb
230	S72431A	Fracture of lower limb
230	S72431B	Fracture of lower limb
230	S72431C	Fracture of lower limb
230	S72431D	Fracture of lower limb
230	S72431E	Fracture of lower limb
230	S72431F	Fracture of lower limb
230	S72431G	Fracture of lower limb
230	S72431H	Fracture of lower limb
230	S72431J	Fracture of lower limb
230	S72431K	Fracture of lower limb
230	S72431M	Fracture of lower limb
230	S72431N	Fracture of lower limb
230	S72431P	Fracture of lower limb
230	S72431Q	Fracture of lower limb
230	S72431R	Fracture of lower limb
230	S72431S	Fracture of lower limb
230	S72432A	Fracture of lower limb
230	S72432B	Fracture of lower limb
230	S72432C	Fracture of lower limb
230	S72432D	Fracture of lower limb
230	S72432E	Fracture of lower limb

230	S72432F	Fracture of lower limb
230	S72432G	Fracture of lower limb
230	S72432H	Fracture of lower limb
230	S72432J	Fracture of lower limb
230	S72432K	Fracture of lower limb
230	S72432M	Fracture of lower limb
230	S72432N	Fracture of lower limb
230	S72432P	Fracture of lower limb
230	S72432Q	Fracture of lower limb
230	S72432R	Fracture of lower limb
230	S72432S	Fracture of lower limb
230	S72433A	Fracture of lower limb
230	S72433B	Fracture of lower limb
230	S72433C	Fracture of lower limb
230	S72433D	Fracture of lower limb
230	S72433E	Fracture of lower limb
230	S72433F	Fracture of lower limb
230	S72433G	Fracture of lower limb
230	S72433H	Fracture of lower limb
230	S72433J	Fracture of lower limb
230	S72433K	Fracture of lower limb
230	S72433M	Fracture of lower limb
230	S72433N	Fracture of lower limb
230	S72433P	Fracture of lower limb
230	S72433Q	Fracture of lower limb
230	S72433R	Fracture of lower limb
230	S72433S	Fracture of lower limb
230	S72434A	Fracture of lower limb
230	S72434B	Fracture of lower limb
230	S72434C	Fracture of lower limb
230	S72434D	Fracture of lower limb
230	S72434E	Fracture of lower limb
230	S72434F	Fracture of lower limb
230	S72434G	Fracture of lower limb
230	S72434H	Fracture of lower limb
230	S72434J	Fracture of lower limb
230	S72434K	Fracture of lower limb
230	S72434M	Fracture of lower limb
230	S72434N	Fracture of lower limb
230	S72434P	Fracture of lower limb
230	S72434Q	Fracture of lower limb
230	S72434R	Fracture of lower limb
230	S72434S	Fracture of lower limb
230	S72435A	Fracture of lower limb
230	S72435B	Fracture of lower limb
230	S72435C	Fracture of lower limb
230	S72435D	Fracture of lower limb
230	S72435E	Fracture of lower limb
230	S72435F	Fracture of lower limb
230	S72435G	Fracture of lower limb
230	S72435H	Fracture of lower limb

230	S72435J	Fracture of lower limb
230	S72435K	Fracture of lower limb
230	S72435M	Fracture of lower limb
230	S72435N	Fracture of lower limb
230	S72435P	Fracture of lower limb
230	S72435Q	Fracture of lower limb
230	S72435R	Fracture of lower limb
230	S72435S	Fracture of lower limb
230	S72436A	Fracture of lower limb
230	S72436B	Fracture of lower limb
230	S72436C	Fracture of lower limb
230	S72436D	Fracture of lower limb
230	S72436E	Fracture of lower limb
230	S72436F	Fracture of lower limb
230	S72436G	Fracture of lower limb
230	S72436H	Fracture of lower limb
230	S72436J	Fracture of lower limb
230	S72436K	Fracture of lower limb
230	S72436M	Fracture of lower limb
230	S72436N	Fracture of lower limb
230	S72436P	Fracture of lower limb
230	S72436Q	Fracture of lower limb
230	S72436R	Fracture of lower limb
230	S72436S	Fracture of lower limb
230	S72441A	Fracture of lower limb
230	S72441B	Fracture of lower limb
230	S72441C	Fracture of lower limb
230	S72441D	Fracture of lower limb
230	S72441E	Fracture of lower limb
230	S72441F	Fracture of lower limb
230	S72441G	Fracture of lower limb
230	S72441H	Fracture of lower limb
230	S72441J	Fracture of lower limb
230	S72441K	Fracture of lower limb
230	S72441M	Fracture of lower limb
230	S72441N	Fracture of lower limb
230	S72441P	Fracture of lower limb
230	S72441Q	Fracture of lower limb
230	S72441R	Fracture of lower limb
230	S72441S	Fracture of lower limb
230	S72442A	Fracture of lower limb
230	S72442B	Fracture of lower limb
230	S72442C	Fracture of lower limb
230	S72442D	Fracture of lower limb
230	S72442E	Fracture of lower limb
230	S72442F	Fracture of lower limb
230	S72442G	Fracture of lower limb
230	S72442H	Fracture of lower limb
230	S72442J	Fracture of lower limb
230	S72442K	Fracture of lower limb
230	S72442M	Fracture of lower limb

230	S72442N	Fracture of lower limb
230	S72442P	Fracture of lower limb
230	S72442Q	Fracture of lower limb
230	S72442R	Fracture of lower limb
230	S72442S	Fracture of lower limb
230	S72443A	Fracture of lower limb
230	S72443B	Fracture of lower limb
230	S72443C	Fracture of lower limb
230	S72443D	Fracture of lower limb
230	S72443E	Fracture of lower limb
230	S72443F	Fracture of lower limb
230	S72443G	Fracture of lower limb
230	S72443H	Fracture of lower limb
230	S72443J	Fracture of lower limb
230	S72443K	Fracture of lower limb
230	S72443M	Fracture of lower limb
230	S72443N	Fracture of lower limb
230	S72443P	Fracture of lower limb
230	S72443Q	Fracture of lower limb
230	S72443R	Fracture of lower limb
230	S72443S	Fracture of lower limb
230	S72444A	Fracture of lower limb
230	S72444B	Fracture of lower limb
230	S72444C	Fracture of lower limb
230	S72444D	Fracture of lower limb
230	S72444E	Fracture of lower limb
230	S72444F	Fracture of lower limb
230	S72444G	Fracture of lower limb
230	S72444H	Fracture of lower limb
230	S72444J	Fracture of lower limb
230	S72444K	Fracture of lower limb
230	S72444M	Fracture of lower limb
230	S72444N	Fracture of lower limb
230	S72444P	Fracture of lower limb
230	S72444Q	Fracture of lower limb
230	S72444R	Fracture of lower limb
230	S72444S	Fracture of lower limb
230	S72445A	Fracture of lower limb
230	S72445B	Fracture of lower limb
230	S72445C	Fracture of lower limb
230	S72445D	Fracture of lower limb
230	S72445E	Fracture of lower limb
230	S72445F	Fracture of lower limb
230	S72445G	Fracture of lower limb
230	S72445H	Fracture of lower limb
230	S72445J	Fracture of lower limb
230	S72445K	Fracture of lower limb
230	S72445M	Fracture of lower limb
230	S72445N	Fracture of lower limb
230	S72445P	Fracture of lower limb
230	S72445Q	Fracture of lower limb

230	S72445R	Fracture of lower limb
230	S72445S	Fracture of lower limb
230	S72446A	Fracture of lower limb
230	S72446B	Fracture of lower limb
230	S72446C	Fracture of lower limb
230	S72446D	Fracture of lower limb
230	S72446E	Fracture of lower limb
230	S72446F	Fracture of lower limb
230	S72446G	Fracture of lower limb
230	S72446H	Fracture of lower limb
230	S72446J	Fracture of lower limb
230	S72446K	Fracture of lower limb
230	S72446M	Fracture of lower limb
230	S72446N	Fracture of lower limb
230	S72446P	Fracture of lower limb
230	S72446Q	Fracture of lower limb
230	S72446R	Fracture of lower limb
230	S72446S	Fracture of lower limb
230	S72451A	Fracture of lower limb
230	S72451B	Fracture of lower limb
230	S72451C	Fracture of lower limb
230	S72451D	Fracture of lower limb
230	S72451E	Fracture of lower limb
230	S72451F	Fracture of lower limb
230	S72451G	Fracture of lower limb
230	S72451H	Fracture of lower limb
230	S72451J	Fracture of lower limb
230	S72451K	Fracture of lower limb
230	S72451M	Fracture of lower limb
230	S72451N	Fracture of lower limb
230	S72451P	Fracture of lower limb
230	S72451Q	Fracture of lower limb
230	S72451R	Fracture of lower limb
230	S72451S	Fracture of lower limb
230	S72452A	Fracture of lower limb
230	S72452B	Fracture of lower limb
230	S72452C	Fracture of lower limb
230	S72452D	Fracture of lower limb
230	S72452E	Fracture of lower limb
230	S72452F	Fracture of lower limb
230	S72452G	Fracture of lower limb
230	S72452H	Fracture of lower limb
230	S72452J	Fracture of lower limb
230	S72452K	Fracture of lower limb
230	S72452M	Fracture of lower limb
230	S72452N	Fracture of lower limb
230	S72452P	Fracture of lower limb
230	S72452Q	Fracture of lower limb
230	S72452R	Fracture of lower limb
230	S72452S	Fracture of lower limb
230	S72453A	Fracture of lower limb

230	S72453B	Fracture of lower limb
230	S72453C	Fracture of lower limb
230	S72453D	Fracture of lower limb
230	S72453E	Fracture of lower limb
230	S72453F	Fracture of lower limb
230	S72453G	Fracture of lower limb
230	S72453H	Fracture of lower limb
230	S72453J	Fracture of lower limb
230	S72453K	Fracture of lower limb
230	S72453M	Fracture of lower limb
230	S72453N	Fracture of lower limb
230	S72453P	Fracture of lower limb
230	S72453Q	Fracture of lower limb
230	S72453R	Fracture of lower limb
230	S72453S	Fracture of lower limb
230	S72454A	Fracture of lower limb
230	S72454B	Fracture of lower limb
230	S72454C	Fracture of lower limb
230	S72454D	Fracture of lower limb
230	S72454E	Fracture of lower limb
230	S72454F	Fracture of lower limb
230	S72454G	Fracture of lower limb
230	S72454H	Fracture of lower limb
230	S72454J	Fracture of lower limb
230	S72454K	Fracture of lower limb
230	S72454M	Fracture of lower limb
230	S72454N	Fracture of lower limb
230	S72454P	Fracture of lower limb
230	S72454Q	Fracture of lower limb
230	S72454R	Fracture of lower limb
230	S72454S	Fracture of lower limb
230	S72455A	Fracture of lower limb
230	S72455B	Fracture of lower limb
230	S72455C	Fracture of lower limb
230	S72455D	Fracture of lower limb
230	S72455E	Fracture of lower limb
230	S72455F	Fracture of lower limb
230	S72455G	Fracture of lower limb
230	S72455H	Fracture of lower limb
230	S72455J	Fracture of lower limb
230	S72455K	Fracture of lower limb
230	S72455M	Fracture of lower limb
230	S72455N	Fracture of lower limb
230	S72455P	Fracture of lower limb
230	S72455Q	Fracture of lower limb
230	S72455R	Fracture of lower limb
230	S72455S	Fracture of lower limb
230	S72456A	Fracture of lower limb
230	S72456B	Fracture of lower limb
230	S72456C	Fracture of lower limb
230	S72456D	Fracture of lower limb

230	S72456E	Fracture of lower limb
230	S72456F	Fracture of lower limb
230	S72456G	Fracture of lower limb
230	S72456H	Fracture of lower limb
230	S72456J	Fracture of lower limb
230	S72456K	Fracture of lower limb
230	S72456M	Fracture of lower limb
230	S72456N	Fracture of lower limb
230	S72456P	Fracture of lower limb
230	S72456Q	Fracture of lower limb
230	S72456R	Fracture of lower limb
230	S72456S	Fracture of lower limb
230	S72461A	Fracture of lower limb
230	S72461B	Fracture of lower limb
230	S72461C	Fracture of lower limb
230	S72461D	Fracture of lower limb
230	S72461E	Fracture of lower limb
230	S72461F	Fracture of lower limb
230	S72461G	Fracture of lower limb
230	S72461H	Fracture of lower limb
230	S72461J	Fracture of lower limb
230	S72461K	Fracture of lower limb
230	S72461M	Fracture of lower limb
230	S72461N	Fracture of lower limb
230	S72461P	Fracture of lower limb
230	S72461Q	Fracture of lower limb
230	S72461R	Fracture of lower limb
230	S72461S	Fracture of lower limb
230	S72462A	Fracture of lower limb
230	S72462B	Fracture of lower limb
230	S72462C	Fracture of lower limb
230	S72462D	Fracture of lower limb
230	S72462E	Fracture of lower limb
230	S72462F	Fracture of lower limb
230	S72462G	Fracture of lower limb
230	S72462H	Fracture of lower limb
230	S72462J	Fracture of lower limb
230	S72462K	Fracture of lower limb
230	S72462M	Fracture of lower limb
230	S72462N	Fracture of lower limb
230	S72462P	Fracture of lower limb
230	S72462Q	Fracture of lower limb
230	S72462R	Fracture of lower limb
230	S72462S	Fracture of lower limb
230	S72463A	Fracture of lower limb
230	S72463B	Fracture of lower limb
230	S72463C	Fracture of lower limb
230	S72463D	Fracture of lower limb
230	S72463E	Fracture of lower limb
230	S72463F	Fracture of lower limb
230	S72463G	Fracture of lower limb

230	S72463H	Fracture of lower limb
230	S72463J	Fracture of lower limb
230	S72463K	Fracture of lower limb
230	S72463M	Fracture of lower limb
230	S72463N	Fracture of lower limb
230	S72463P	Fracture of lower limb
230	S72463Q	Fracture of lower limb
230	S72463R	Fracture of lower limb
230	S72463S	Fracture of lower limb
230	S72464A	Fracture of lower limb
230	S72464B	Fracture of lower limb
230	S72464C	Fracture of lower limb
230	S72464D	Fracture of lower limb
230	S72464E	Fracture of lower limb
230	S72464F	Fracture of lower limb
230	S72464G	Fracture of lower limb
230	S72464H	Fracture of lower limb
230	S72464J	Fracture of lower limb
230	S72464K	Fracture of lower limb
230	S72464M	Fracture of lower limb
230	S72464N	Fracture of lower limb
230	S72464P	Fracture of lower limb
230	S72464Q	Fracture of lower limb
230	S72464R	Fracture of lower limb
230	S72464S	Fracture of lower limb
230	S72465A	Fracture of lower limb
230	S72465B	Fracture of lower limb
230	S72465C	Fracture of lower limb
230	S72465D	Fracture of lower limb
230	S72465E	Fracture of lower limb
230	S72465F	Fracture of lower limb
230	S72465G	Fracture of lower limb
230	S72465H	Fracture of lower limb
230	S72465J	Fracture of lower limb
230	S72465K	Fracture of lower limb
230	S72465M	Fracture of lower limb
230	S72465N	Fracture of lower limb
230	S72465P	Fracture of lower limb
230	S72465Q	Fracture of lower limb
230	S72465R	Fracture of lower limb
230	S72465S	Fracture of lower limb
230	S72466A	Fracture of lower limb
230	S72466B	Fracture of lower limb
230	S72466C	Fracture of lower limb
230	S72466D	Fracture of lower limb
230	S72466E	Fracture of lower limb
230	S72466F	Fracture of lower limb
230	S72466G	Fracture of lower limb
230	S72466H	Fracture of lower limb
230	S72466J	Fracture of lower limb
230	S72466K	Fracture of lower limb

230	S72466M	Fracture of lower limb
230	S72466N	Fracture of lower limb
230	S72466P	Fracture of lower limb
230	S72466Q	Fracture of lower limb
230	S72466R	Fracture of lower limb
230	S72466S	Fracture of lower limb
230	S72471A	Fracture of lower limb
230	S72471D	Fracture of lower limb
230	S72471G	Fracture of lower limb
230	S72471K	Fracture of lower limb
230	S72471P	Fracture of lower limb
230	S72471S	Fracture of lower limb
230	S72472A	Fracture of lower limb
230	S72472D	Fracture of lower limb
230	S72472G	Fracture of lower limb
230	S72472K	Fracture of lower limb
230	S72472P	Fracture of lower limb
230	S72472S	Fracture of lower limb
230	S72479A	Fracture of lower limb
230	S72479D	Fracture of lower limb
230	S72479G	Fracture of lower limb
230	S72479K	Fracture of lower limb
230	S72479P	Fracture of lower limb
230	S72479S	Fracture of lower limb
230	S72491A	Fracture of lower limb
230	S72491B	Fracture of lower limb
230	S72491C	Fracture of lower limb
230	S72491D	Fracture of lower limb
230	S72491E	Fracture of lower limb
230	S72491F	Fracture of lower limb
230	S72491G	Fracture of lower limb
230	S72491H	Fracture of lower limb
230	S72491J	Fracture of lower limb
230	S72491K	Fracture of lower limb
230	S72491M	Fracture of lower limb
230	S72491N	Fracture of lower limb
230	S72491P	Fracture of lower limb
230	S72491Q	Fracture of lower limb
230	S72491R	Fracture of lower limb
230	S72491S	Fracture of lower limb
230	S72492A	Fracture of lower limb
230	S72492B	Fracture of lower limb
230	S72492C	Fracture of lower limb
230	S72492D	Fracture of lower limb
230	S72492E	Fracture of lower limb
230	S72492F	Fracture of lower limb
230	S72492G	Fracture of lower limb
230	S72492H	Fracture of lower limb
230	S72492J	Fracture of lower limb
230	S72492K	Fracture of lower limb
230	S72492M	Fracture of lower limb

230	S72492N	Fracture of lower limb
230	S72492P	Fracture of lower limb
230	S72492Q	Fracture of lower limb
230	S72492R	Fracture of lower limb
230	S72492S	Fracture of lower limb
230	S72499A	Fracture of lower limb
230	S72499B	Fracture of lower limb
230	S72499C	Fracture of lower limb
230	S72499D	Fracture of lower limb
230	S72499E	Fracture of lower limb
230	S72499F	Fracture of lower limb
230	S72499G	Fracture of lower limb
230	S72499H	Fracture of lower limb
230	S72499J	Fracture of lower limb
230	S72499K	Fracture of lower limb
230	S72499M	Fracture of lower limb
230	S72499N	Fracture of lower limb
230	S72499P	Fracture of lower limb
230	S72499Q	Fracture of lower limb
230	S72499R	Fracture of lower limb
230	S72499S	Fracture of lower limb
230	S728X1A	Fracture of lower limb
230	S728X1B	Fracture of lower limb
230	S728X1C	Fracture of lower limb
230	S728X1D	Fracture of lower limb
230	S728X1E	Fracture of lower limb
230	S728X1F	Fracture of lower limb
230	S728X1G	Fracture of lower limb
230	S728X1H	Fracture of lower limb
230	S728X1J	Fracture of lower limb
230	S728X1K	Fracture of lower limb
230	S728X1M	Fracture of lower limb
230	S728X1N	Fracture of lower limb
230	S728X1P	Fracture of lower limb
230	S728X1Q	Fracture of lower limb
230	S728X1R	Fracture of lower limb
230	S728X1S	Fracture of lower limb
230	S728X2A	Fracture of lower limb
230	S728X2B	Fracture of lower limb
230	S728X2C	Fracture of lower limb
230	S728X2D	Fracture of lower limb
230	S728X2E	Fracture of lower limb
230	S728X2F	Fracture of lower limb
230	S728X2G	Fracture of lower limb
230	S728X2H	Fracture of lower limb
230	S728X2J	Fracture of lower limb
230	S728X2K	Fracture of lower limb
230	S728X2M	Fracture of lower limb
230	S728X2N	Fracture of lower limb
230	S728X2P	Fracture of lower limb
230	S728X2Q	Fracture of lower limb

230	S728X2R	Fracture of lower limb
230	S728X2S	Fracture of lower limb
230	S728X9A	Fracture of lower limb
230	S728X9B	Fracture of lower limb
230	S728X9C	Fracture of lower limb
230	S728X9D	Fracture of lower limb
230	S728X9E	Fracture of lower limb
230	S728X9F	Fracture of lower limb
230	S728X9G	Fracture of lower limb
230	S728X9H	Fracture of lower limb
230	S728X9J	Fracture of lower limb
230	S728X9K	Fracture of lower limb
230	S728X9M	Fracture of lower limb
230	S728X9N	Fracture of lower limb
230	S728X9P	Fracture of lower limb
230	S728X9Q	Fracture of lower limb
230	S728X9R	Fracture of lower limb
230	S728X9S	Fracture of lower limb
230	S7290XA	Fracture of lower limb
230	S7290XB	Fracture of lower limb
230	S7290XC	Fracture of lower limb
230	S7290XD	Fracture of lower limb
230	S7290XE	Fracture of lower limb
230	S7290XF	Fracture of lower limb
230	S7290XG	Fracture of lower limb
230	S7290XH	Fracture of lower limb
230	S7290XJ	Fracture of lower limb
230	S7290XK	Fracture of lower limb
230	S7290XM	Fracture of lower limb
230	S7290XN	Fracture of lower limb
230	S7290XP	Fracture of lower limb
230	S7290XQ	Fracture of lower limb
230	S7290XR	Fracture of lower limb
230	S7290XS	Fracture of lower limb
230	S7291XA	Fracture of lower limb
230	S7291XB	Fracture of lower limb
230	S7291XC	Fracture of lower limb
230	S7291XD	Fracture of lower limb
230	S7291XE	Fracture of lower limb
230	S7291XF	Fracture of lower limb
230	S7291XG	Fracture of lower limb
230	S7291XH	Fracture of lower limb
230	S7291XJ	Fracture of lower limb
230	S7291XK	Fracture of lower limb
230	S7291XM	Fracture of lower limb
230	S7291XN	Fracture of lower limb
230	S7291XP	Fracture of lower limb
230	S7291XQ	Fracture of lower limb
230	S7291XR	Fracture of lower limb
230	S7291XS	Fracture of lower limb
230	S7292XA	Fracture of lower limb

230	S7292XB	Fracture of lower limb
230	S7292XC	Fracture of lower limb
230	S7292XD	Fracture of lower limb
230	S7292XE	Fracture of lower limb
230	S7292XF	Fracture of lower limb
230	S7292XG	Fracture of lower limb
230	S7292XH	Fracture of lower limb
230	S7292XJ	Fracture of lower limb
230	S7292XK	Fracture of lower limb
230	S7292XM	Fracture of lower limb
230	S7292XN	Fracture of lower limb
230	S7292XP	Fracture of lower limb
230	S7292XQ	Fracture of lower limb
230	S7292XR	Fracture of lower limb
230	S7292XS	Fracture of lower limb
230	S79001A	Fracture of lower limb
230	S79001D	Fracture of lower limb
230	S79001G	Fracture of lower limb
230	S79001K	Fracture of lower limb
230	S79001P	Fracture of lower limb
230	S79001S	Fracture of lower limb
230	S79002A	Fracture of lower limb
230	S79002D	Fracture of lower limb
230	S79002G	Fracture of lower limb
230	S79002K	Fracture of lower limb
230	S79002P	Fracture of lower limb
230	S79002S	Fracture of lower limb
230	S79009A	Fracture of lower limb
230	S79009D	Fracture of lower limb
230	S79009G	Fracture of lower limb
230	S79009K	Fracture of lower limb
230	S79009P	Fracture of lower limb
230	S79009S	Fracture of lower limb
230	S79011A	Fracture of lower limb
230	S79011D	Fracture of lower limb
230	S79011G	Fracture of lower limb
230	S79011K	Fracture of lower limb
230	S79011P	Fracture of lower limb
230	S79011S	Fracture of lower limb
230	S79012A	Fracture of lower limb
230	S79012D	Fracture of lower limb
230	S79012G	Fracture of lower limb
230	S79012K	Fracture of lower limb
230	S79012P	Fracture of lower limb
230	S79012S	Fracture of lower limb
230	S79019A	Fracture of lower limb
230	S79019D	Fracture of lower limb
230	S79019G	Fracture of lower limb
230	S79019K	Fracture of lower limb
230	S79019P	Fracture of lower limb
230	S79019S	Fracture of lower limb

230	S79091A	Fracture of lower limb
230	S79091D	Fracture of lower limb
230	S79091G	Fracture of lower limb
230	S79091K	Fracture of lower limb
230	S79091P	Fracture of lower limb
230	S79091S	Fracture of lower limb
230	S79092A	Fracture of lower limb
230	S79092D	Fracture of lower limb
230	S79092G	Fracture of lower limb
230	S79092K	Fracture of lower limb
230	S79092P	Fracture of lower limb
230	S79092S	Fracture of lower limb
230	S79099A	Fracture of lower limb
230	S79099D	Fracture of lower limb
230	S79099G	Fracture of lower limb
230	S79099K	Fracture of lower limb
230	S79099P	Fracture of lower limb
230	S79099S	Fracture of lower limb
230	S79101A	Fracture of lower limb
230	S79101D	Fracture of lower limb
230	S79101G	Fracture of lower limb
230	S79101K	Fracture of lower limb
230	S79101P	Fracture of lower limb
230	S79101S	Fracture of lower limb
230	S79102A	Fracture of lower limb
230	S79102D	Fracture of lower limb
230	S79102G	Fracture of lower limb
230	S79102K	Fracture of lower limb
230	S79102P	Fracture of lower limb
230	S79102S	Fracture of lower limb
230	S79109A	Fracture of lower limb
230	S79109D	Fracture of lower limb
230	S79109G	Fracture of lower limb
230	S79109K	Fracture of lower limb
230	S79109P	Fracture of lower limb
230	S79109S	Fracture of lower limb
230	S79111A	Fracture of lower limb
230	S79111D	Fracture of lower limb
230	S79111G	Fracture of lower limb
230	S79111K	Fracture of lower limb
230	S79111P	Fracture of lower limb
230	S79111S	Fracture of lower limb
230	S79112A	Fracture of lower limb
230	S79112D	Fracture of lower limb
230	S79112G	Fracture of lower limb
230	S79112K	Fracture of lower limb
230	S79112P	Fracture of lower limb
230	S79112S	Fracture of lower limb
230	S79119A	Fracture of lower limb
230	S79119D	Fracture of lower limb
230	S79119G	Fracture of lower limb

230	S79119K	Fracture of lower limb
230	S79119P	Fracture of lower limb
230	S79119S	Fracture of lower limb
230	S79121A	Fracture of lower limb
230	S79121D	Fracture of lower limb
230	S79121G	Fracture of lower limb
230	S79121K	Fracture of lower limb
230	S79121P	Fracture of lower limb
230	S79121S	Fracture of lower limb
230	S79122A	Fracture of lower limb
230	S79122D	Fracture of lower limb
230	S79122G	Fracture of lower limb
230	S79122K	Fracture of lower limb
230	S79122P	Fracture of lower limb
230	S79122S	Fracture of lower limb
230	S79129A	Fracture of lower limb
230	S79129D	Fracture of lower limb
230	S79129G	Fracture of lower limb
230	S79129K	Fracture of lower limb
230	S79129P	Fracture of lower limb
230	S79129S	Fracture of lower limb
230	S79131A	Fracture of lower limb
230	S79131D	Fracture of lower limb
230	S79131G	Fracture of lower limb
230	S79131K	Fracture of lower limb
230	S79131P	Fracture of lower limb
230	S79131S	Fracture of lower limb
230	S79132A	Fracture of lower limb
230	S79132D	Fracture of lower limb
230	S79132G	Fracture of lower limb
230	S79132K	Fracture of lower limb
230	S79132P	Fracture of lower limb
230	S79132S	Fracture of lower limb
230	S79139A	Fracture of lower limb
230	S79139D	Fracture of lower limb
230	S79139G	Fracture of lower limb
230	S79139K	Fracture of lower limb
230	S79139P	Fracture of lower limb
230	S79139S	Fracture of lower limb
230	S79141A	Fracture of lower limb
230	S79141D	Fracture of lower limb
230	S79141G	Fracture of lower limb
230	S79141K	Fracture of lower limb
230	S79141P	Fracture of lower limb
230	S79141S	Fracture of lower limb
230	S79142A	Fracture of lower limb
230	S79142D	Fracture of lower limb
230	S79142G	Fracture of lower limb
230	S79142K	Fracture of lower limb
230	S79142P	Fracture of lower limb
230	S79142S	Fracture of lower limb

230	S79149A	Fracture of lower limb
230	S79149D	Fracture of lower limb
230	S79149G	Fracture of lower limb
230	S79149K	Fracture of lower limb
230	S79149P	Fracture of lower limb
230	S79149S	Fracture of lower limb
230	S79191A	Fracture of lower limb
230	S79191D	Fracture of lower limb
230	S79191G	Fracture of lower limb
230	S79191K	Fracture of lower limb
230	S79191P	Fracture of lower limb
230	S79191S	Fracture of lower limb
230	S79192A	Fracture of lower limb
230	S79192D	Fracture of lower limb
230	S79192G	Fracture of lower limb
230	S79192K	Fracture of lower limb
230	S79192P	Fracture of lower limb
230	S79192S	Fracture of lower limb
230	S79199A	Fracture of lower limb
230	S79199D	Fracture of lower limb
230	S79199G	Fracture of lower limb
230	S79199K	Fracture of lower limb
230	S79199P	Fracture of lower limb
230	S79199S	Fracture of lower limb
230	S82001A	Fracture of lower limb
230	S82001B	Fracture of lower limb
230	S82001C	Fracture of lower limb
230	S82001D	Fracture of lower limb
230	S82001E	Fracture of lower limb
230	S82001F	Fracture of lower limb
230	S82001G	Fracture of lower limb
230	S82001H	Fracture of lower limb
230	S82001J	Fracture of lower limb
230	S82001K	Fracture of lower limb
230	S82001M	Fracture of lower limb
230	S82001N	Fracture of lower limb
230	S82001P	Fracture of lower limb
230	S82001Q	Fracture of lower limb
230	S82001R	Fracture of lower limb
230	S82001S	Fracture of lower limb
230	S82002A	Fracture of lower limb
230	S82002B	Fracture of lower limb
230	S82002C	Fracture of lower limb
230	S82002D	Fracture of lower limb
230	S82002E	Fracture of lower limb
230	S82002F	Fracture of lower limb
230	S82002G	Fracture of lower limb
230	S82002H	Fracture of lower limb
230	S82002J	Fracture of lower limb
230	S82002K	Fracture of lower limb
230	S82002M	Fracture of lower limb

230	S82002N	Fracture of lower limb
230	S82002P	Fracture of lower limb
230	S82002Q	Fracture of lower limb
230	S82002R	Fracture of lower limb
230	S82002S	Fracture of lower limb
230	S82009A	Fracture of lower limb
230	S82009B	Fracture of lower limb
230	S82009C	Fracture of lower limb
230	S82009D	Fracture of lower limb
230	S82009E	Fracture of lower limb
230	S82009F	Fracture of lower limb
230	S82009G	Fracture of lower limb
230	S82009H	Fracture of lower limb
230	S82009J	Fracture of lower limb
230	S82009K	Fracture of lower limb
230	S82009M	Fracture of lower limb
230	S82009N	Fracture of lower limb
230	S82009P	Fracture of lower limb
230	S82009Q	Fracture of lower limb
230	S82009R	Fracture of lower limb
230	S82009S	Fracture of lower limb
230	S82011A	Fracture of lower limb
230	S82011B	Fracture of lower limb
230	S82011C	Fracture of lower limb
230	S82011D	Fracture of lower limb
230	S82011E	Fracture of lower limb
230	S82011F	Fracture of lower limb
230	S82011G	Fracture of lower limb
230	S82011H	Fracture of lower limb
230	S82011J	Fracture of lower limb
230	S82011K	Fracture of lower limb
230	S82011M	Fracture of lower limb
230	S82011N	Fracture of lower limb
230	S82011P	Fracture of lower limb
230	S82011Q	Fracture of lower limb
230	S82011R	Fracture of lower limb
230	S82011S	Fracture of lower limb
230	S82012A	Fracture of lower limb
230	S82012B	Fracture of lower limb
230	S82012C	Fracture of lower limb
230	S82012D	Fracture of lower limb
230	S82012E	Fracture of lower limb
230	S82012F	Fracture of lower limb
230	S82012G	Fracture of lower limb
230	S82012H	Fracture of lower limb
230	S82012J	Fracture of lower limb
230	S82012K	Fracture of lower limb
230	S82012M	Fracture of lower limb
230	S82012N	Fracture of lower limb
230	S82012P	Fracture of lower limb
230	S82012Q	Fracture of lower limb

230	S82012R	Fracture of lower limb
230	S82012S	Fracture of lower limb
230	S82013A	Fracture of lower limb
230	S82013B	Fracture of lower limb
230	S82013C	Fracture of lower limb
230	S82013D	Fracture of lower limb
230	S82013E	Fracture of lower limb
230	S82013F	Fracture of lower limb
230	S82013G	Fracture of lower limb
230	S82013H	Fracture of lower limb
230	S82013J	Fracture of lower limb
230	S82013K	Fracture of lower limb
230	S82013M	Fracture of lower limb
230	S82013N	Fracture of lower limb
230	S82013P	Fracture of lower limb
230	S82013Q	Fracture of lower limb
230	S82013R	Fracture of lower limb
230	S82013S	Fracture of lower limb
230	S82014A	Fracture of lower limb
230	S82014B	Fracture of lower limb
230	S82014C	Fracture of lower limb
230	S82014D	Fracture of lower limb
230	S82014E	Fracture of lower limb
230	S82014F	Fracture of lower limb
230	S82014G	Fracture of lower limb
230	S82014H	Fracture of lower limb
230	S82014J	Fracture of lower limb
230	S82014K	Fracture of lower limb
230	S82014M	Fracture of lower limb
230	S82014N	Fracture of lower limb
230	S82014P	Fracture of lower limb
230	S82014Q	Fracture of lower limb
230	S82014R	Fracture of lower limb
230	S82014S	Fracture of lower limb
230	S82015A	Fracture of lower limb
230	S82015B	Fracture of lower limb
230	S82015C	Fracture of lower limb
230	S82015D	Fracture of lower limb
230	S82015E	Fracture of lower limb
230	S82015F	Fracture of lower limb
230	S82015G	Fracture of lower limb
230	S82015H	Fracture of lower limb
230	S82015J	Fracture of lower limb
230	S82015K	Fracture of lower limb
230	S82015M	Fracture of lower limb
230	S82015N	Fracture of lower limb
230	S82015P	Fracture of lower limb
230	S82015Q	Fracture of lower limb
230	S82015R	Fracture of lower limb
230	S82015S	Fracture of lower limb
230	S82016A	Fracture of lower limb

230	S82016B	Fracture of lower limb
230	S82016C	Fracture of lower limb
230	S82016D	Fracture of lower limb
230	S82016E	Fracture of lower limb
230	S82016F	Fracture of lower limb
230	S82016G	Fracture of lower limb
230	S82016H	Fracture of lower limb
230	S82016J	Fracture of lower limb
230	S82016K	Fracture of lower limb
230	S82016M	Fracture of lower limb
230	S82016N	Fracture of lower limb
230	S82016P	Fracture of lower limb
230	S82016Q	Fracture of lower limb
230	S82016R	Fracture of lower limb
230	S82016S	Fracture of lower limb
230	S82021A	Fracture of lower limb
230	S82021B	Fracture of lower limb
230	S82021C	Fracture of lower limb
230	S82021D	Fracture of lower limb
230	S82021E	Fracture of lower limb
230	S82021F	Fracture of lower limb
230	S82021G	Fracture of lower limb
230	S82021H	Fracture of lower limb
230	S82021J	Fracture of lower limb
230	S82021K	Fracture of lower limb
230	S82021M	Fracture of lower limb
230	S82021N	Fracture of lower limb
230	S82021P	Fracture of lower limb
230	S82021Q	Fracture of lower limb
230	S82021R	Fracture of lower limb
230	S82021S	Fracture of lower limb
230	S82022A	Fracture of lower limb
230	S82022B	Fracture of lower limb
230	S82022C	Fracture of lower limb
230	S82022D	Fracture of lower limb
230	S82022E	Fracture of lower limb
230	S82022F	Fracture of lower limb
230	S82022G	Fracture of lower limb
230	S82022H	Fracture of lower limb
230	S82022J	Fracture of lower limb
230	S82022K	Fracture of lower limb
230	S82022M	Fracture of lower limb
230	S82022N	Fracture of lower limb
230	S82022P	Fracture of lower limb
230	S82022Q	Fracture of lower limb
230	S82022R	Fracture of lower limb
230	S82022S	Fracture of lower limb
230	S82023A	Fracture of lower limb
230	S82023B	Fracture of lower limb
230	S82023C	Fracture of lower limb
230	S82023D	Fracture of lower limb

230	S82023E	Fracture of lower limb
230	S82023F	Fracture of lower limb
230	S82023G	Fracture of lower limb
230	S82023H	Fracture of lower limb
230	S82023J	Fracture of lower limb
230	S82023K	Fracture of lower limb
230	S82023M	Fracture of lower limb
230	S82023N	Fracture of lower limb
230	S82023P	Fracture of lower limb
230	S82023Q	Fracture of lower limb
230	S82023R	Fracture of lower limb
230	S82023S	Fracture of lower limb
230	S82024A	Fracture of lower limb
230	S82024B	Fracture of lower limb
230	S82024C	Fracture of lower limb
230	S82024D	Fracture of lower limb
230	S82024E	Fracture of lower limb
230	S82024F	Fracture of lower limb
230	S82024G	Fracture of lower limb
230	S82024H	Fracture of lower limb
230	S82024J	Fracture of lower limb
230	S82024K	Fracture of lower limb
230	S82024M	Fracture of lower limb
230	S82024N	Fracture of lower limb
230	S82024P	Fracture of lower limb
230	S82024Q	Fracture of lower limb
230	S82024R	Fracture of lower limb
230	S82024S	Fracture of lower limb
230	S82025A	Fracture of lower limb
230	S82025B	Fracture of lower limb
230	S82025C	Fracture of lower limb
230	S82025D	Fracture of lower limb
230	S82025E	Fracture of lower limb
230	S82025F	Fracture of lower limb
230	S82025G	Fracture of lower limb
230	S82025H	Fracture of lower limb
230	S82025J	Fracture of lower limb
230	S82025K	Fracture of lower limb
230	S82025M	Fracture of lower limb
230	S82025N	Fracture of lower limb
230	S82025P	Fracture of lower limb
230	S82025Q	Fracture of lower limb
230	S82025R	Fracture of lower limb
230	S82025S	Fracture of lower limb
230	S82026A	Fracture of lower limb
230	S82026B	Fracture of lower limb
230	S82026C	Fracture of lower limb
230	S82026D	Fracture of lower limb
230	S82026E	Fracture of lower limb
230	S82026F	Fracture of lower limb
230	S82026G	Fracture of lower limb

230	S82026H	Fracture of lower limb
230	S82026J	Fracture of lower limb
230	S82026K	Fracture of lower limb
230	S82026M	Fracture of lower limb
230	S82026N	Fracture of lower limb
230	S82026P	Fracture of lower limb
230	S82026Q	Fracture of lower limb
230	S82026R	Fracture of lower limb
230	S82026S	Fracture of lower limb
230	S82031A	Fracture of lower limb
230	S82031B	Fracture of lower limb
230	S82031C	Fracture of lower limb
230	S82031D	Fracture of lower limb
230	S82031E	Fracture of lower limb
230	S82031F	Fracture of lower limb
230	S82031G	Fracture of lower limb
230	S82031H	Fracture of lower limb
230	S82031J	Fracture of lower limb
230	S82031K	Fracture of lower limb
230	S82031M	Fracture of lower limb
230	S82031N	Fracture of lower limb
230	S82031P	Fracture of lower limb
230	S82031Q	Fracture of lower limb
230	S82031R	Fracture of lower limb
230	S82031S	Fracture of lower limb
230	S82032A	Fracture of lower limb
230	S82032B	Fracture of lower limb
230	S82032C	Fracture of lower limb
230	S82032D	Fracture of lower limb
230	S82032E	Fracture of lower limb
230	S82032F	Fracture of lower limb
230	S82032G	Fracture of lower limb
230	S82032H	Fracture of lower limb
230	S82032J	Fracture of lower limb
230	S82032K	Fracture of lower limb
230	S82032M	Fracture of lower limb
230	S82032N	Fracture of lower limb
230	S82032P	Fracture of lower limb
230	S82032Q	Fracture of lower limb
230	S82032R	Fracture of lower limb
230	S82032S	Fracture of lower limb
230	S82033A	Fracture of lower limb
230	S82033B	Fracture of lower limb
230	S82033C	Fracture of lower limb
230	S82033D	Fracture of lower limb
230	S82033E	Fracture of lower limb
230	S82033F	Fracture of lower limb
230	S82033G	Fracture of lower limb
230	S82033H	Fracture of lower limb
230	S82033J	Fracture of lower limb
230	S82033K	Fracture of lower limb

230	S82033M	Fracture of lower limb
230	S82033N	Fracture of lower limb
230	S82033P	Fracture of lower limb
230	S82033Q	Fracture of lower limb
230	S82033R	Fracture of lower limb
230	S82033S	Fracture of lower limb
230	S82034A	Fracture of lower limb
230	S82034B	Fracture of lower limb
230	S82034C	Fracture of lower limb
230	S82034D	Fracture of lower limb
230	S82034E	Fracture of lower limb
230	S82034F	Fracture of lower limb
230	S82034G	Fracture of lower limb
230	S82034H	Fracture of lower limb
230	S82034J	Fracture of lower limb
230	S82034K	Fracture of lower limb
230	S82034M	Fracture of lower limb
230	S82034N	Fracture of lower limb
230	S82034P	Fracture of lower limb
230	S82034Q	Fracture of lower limb
230	S82034R	Fracture of lower limb
230	S82034S	Fracture of lower limb
230	S82035A	Fracture of lower limb
230	S82035B	Fracture of lower limb
230	S82035C	Fracture of lower limb
230	S82035D	Fracture of lower limb
230	S82035E	Fracture of lower limb
230	S82035F	Fracture of lower limb
230	S82035G	Fracture of lower limb
230	S82035H	Fracture of lower limb
230	S82035J	Fracture of lower limb
230	S82035K	Fracture of lower limb
230	S82035M	Fracture of lower limb
230	S82035N	Fracture of lower limb
230	S82035P	Fracture of lower limb
230	S82035Q	Fracture of lower limb
230	S82035R	Fracture of lower limb
230	S82035S	Fracture of lower limb
230	S82036A	Fracture of lower limb
230	S82036B	Fracture of lower limb
230	S82036C	Fracture of lower limb
230	S82036D	Fracture of lower limb
230	S82036E	Fracture of lower limb
230	S82036F	Fracture of lower limb
230	S82036G	Fracture of lower limb
230	S82036H	Fracture of lower limb
230	S82036J	Fracture of lower limb
230	S82036K	Fracture of lower limb
230	S82036M	Fracture of lower limb
230	S82036N	Fracture of lower limb
230	S82036P	Fracture of lower limb

230	S82036Q	Fracture of lower limb
230	S82036R	Fracture of lower limb
230	S82036S	Fracture of lower limb
230	S82041A	Fracture of lower limb
230	S82041B	Fracture of lower limb
230	S82041C	Fracture of lower limb
230	S82041D	Fracture of lower limb
230	S82041E	Fracture of lower limb
230	S82041F	Fracture of lower limb
230	S82041G	Fracture of lower limb
230	S82041H	Fracture of lower limb
230	S82041J	Fracture of lower limb
230	S82041K	Fracture of lower limb
230	S82041M	Fracture of lower limb
230	S82041N	Fracture of lower limb
230	S82041P	Fracture of lower limb
230	S82041Q	Fracture of lower limb
230	S82041R	Fracture of lower limb
230	S82041S	Fracture of lower limb
230	S82042A	Fracture of lower limb
230	S82042B	Fracture of lower limb
230	S82042C	Fracture of lower limb
230	S82042D	Fracture of lower limb
230	S82042E	Fracture of lower limb
230	S82042F	Fracture of lower limb
230	S82042G	Fracture of lower limb
230	S82042H	Fracture of lower limb
230	S82042J	Fracture of lower limb
230	S82042K	Fracture of lower limb
230	S82042M	Fracture of lower limb
230	S82042N	Fracture of lower limb
230	S82042P	Fracture of lower limb
230	S82042Q	Fracture of lower limb
230	S82042R	Fracture of lower limb
230	S82042S	Fracture of lower limb
230	S82043A	Fracture of lower limb
230	S82043B	Fracture of lower limb
230	S82043C	Fracture of lower limb
230	S82043D	Fracture of lower limb
230	S82043E	Fracture of lower limb
230	S82043F	Fracture of lower limb
230	S82043G	Fracture of lower limb
230	S82043H	Fracture of lower limb
230	S82043J	Fracture of lower limb
230	S82043K	Fracture of lower limb
230	S82043M	Fracture of lower limb
230	S82043N	Fracture of lower limb
230	S82043P	Fracture of lower limb
230	S82043Q	Fracture of lower limb
230	S82043R	Fracture of lower limb
230	S82043S	Fracture of lower limb

230	S82044A	Fracture of lower limb
230	S82044B	Fracture of lower limb
230	S82044C	Fracture of lower limb
230	S82044D	Fracture of lower limb
230	S82044E	Fracture of lower limb
230	S82044F	Fracture of lower limb
230	S82044G	Fracture of lower limb
230	S82044H	Fracture of lower limb
230	S82044J	Fracture of lower limb
230	S82044K	Fracture of lower limb
230	S82044M	Fracture of lower limb
230	S82044N	Fracture of lower limb
230	S82044P	Fracture of lower limb
230	S82044Q	Fracture of lower limb
230	S82044R	Fracture of lower limb
230	S82044S	Fracture of lower limb
230	S82045A	Fracture of lower limb
230	S82045B	Fracture of lower limb
230	S82045C	Fracture of lower limb
230	S82045D	Fracture of lower limb
230	S82045E	Fracture of lower limb
230	S82045F	Fracture of lower limb
230	S82045G	Fracture of lower limb
230	S82045H	Fracture of lower limb
230	S82045J	Fracture of lower limb
230	S82045K	Fracture of lower limb
230	S82045M	Fracture of lower limb
230	S82045N	Fracture of lower limb
230	S82045P	Fracture of lower limb
230	S82045Q	Fracture of lower limb
230	S82045R	Fracture of lower limb
230	S82045S	Fracture of lower limb
230	S82046A	Fracture of lower limb
230	S82046B	Fracture of lower limb
230	S82046C	Fracture of lower limb
230	S82046D	Fracture of lower limb
230	S82046E	Fracture of lower limb
230	S82046F	Fracture of lower limb
230	S82046G	Fracture of lower limb
230	S82046H	Fracture of lower limb
230	S82046J	Fracture of lower limb
230	S82046K	Fracture of lower limb
230	S82046M	Fracture of lower limb
230	S82046N	Fracture of lower limb
230	S82046P	Fracture of lower limb
230	S82046Q	Fracture of lower limb
230	S82046R	Fracture of lower limb
230	S82046S	Fracture of lower limb
230	S82091A	Fracture of lower limb
230	S82091B	Fracture of lower limb
230	S82091C	Fracture of lower limb

230	S82091D	Fracture of lower limb
230	S82091E	Fracture of lower limb
230	S82091F	Fracture of lower limb
230	S82091G	Fracture of lower limb
230	S82091H	Fracture of lower limb
230	S82091J	Fracture of lower limb
230	S82091K	Fracture of lower limb
230	S82091M	Fracture of lower limb
230	S82091N	Fracture of lower limb
230	S82091P	Fracture of lower limb
230	S82091Q	Fracture of lower limb
230	S82091R	Fracture of lower limb
230	S82091S	Fracture of lower limb
230	S82092A	Fracture of lower limb
230	S82092B	Fracture of lower limb
230	S82092C	Fracture of lower limb
230	S82092D	Fracture of lower limb
230	S82092E	Fracture of lower limb
230	S82092F	Fracture of lower limb
230	S82092G	Fracture of lower limb
230	S82092H	Fracture of lower limb
230	S82092J	Fracture of lower limb
230	S82092K	Fracture of lower limb
230	S82092M	Fracture of lower limb
230	S82092N	Fracture of lower limb
230	S82092P	Fracture of lower limb
230	S82092Q	Fracture of lower limb
230	S82092R	Fracture of lower limb
230	S82092S	Fracture of lower limb
230	S82099A	Fracture of lower limb
230	S82099B	Fracture of lower limb
230	S82099C	Fracture of lower limb
230	S82099D	Fracture of lower limb
230	S82099E	Fracture of lower limb
230	S82099F	Fracture of lower limb
230	S82099G	Fracture of lower limb
230	S82099H	Fracture of lower limb
230	S82099J	Fracture of lower limb
230	S82099K	Fracture of lower limb
230	S82099M	Fracture of lower limb
230	S82099N	Fracture of lower limb
230	S82099P	Fracture of lower limb
230	S82099Q	Fracture of lower limb
230	S82099R	Fracture of lower limb
230	S82099S	Fracture of lower limb
230	S82101A	Fracture of lower limb
230	S82101B	Fracture of lower limb
230	S82101C	Fracture of lower limb
230	S82101D	Fracture of lower limb
230	S82101E	Fracture of lower limb
230	S82101F	Fracture of lower limb

230	S82101G	Fracture of lower limb
230	S82101H	Fracture of lower limb
230	S82101J	Fracture of lower limb
230	S82101K	Fracture of lower limb
230	S82101M	Fracture of lower limb
230	S82101N	Fracture of lower limb
230	S82101P	Fracture of lower limb
230	S82101Q	Fracture of lower limb
230	S82101R	Fracture of lower limb
230	S82101S	Fracture of lower limb
230	S82102A	Fracture of lower limb
230	S82102B	Fracture of lower limb
230	S82102C	Fracture of lower limb
230	S82102D	Fracture of lower limb
230	S82102E	Fracture of lower limb
230	S82102F	Fracture of lower limb
230	S82102G	Fracture of lower limb
230	S82102H	Fracture of lower limb
230	S82102J	Fracture of lower limb
230	S82102K	Fracture of lower limb
230	S82102M	Fracture of lower limb
230	S82102N	Fracture of lower limb
230	S82102P	Fracture of lower limb
230	S82102Q	Fracture of lower limb
230	S82102R	Fracture of lower limb
230	S82102S	Fracture of lower limb
230	S82109A	Fracture of lower limb
230	S82109B	Fracture of lower limb
230	S82109C	Fracture of lower limb
230	S82109D	Fracture of lower limb
230	S82109E	Fracture of lower limb
230	S82109F	Fracture of lower limb
230	S82109G	Fracture of lower limb
230	S82109H	Fracture of lower limb
230	S82109J	Fracture of lower limb
230	S82109K	Fracture of lower limb
230	S82109M	Fracture of lower limb
230	S82109N	Fracture of lower limb
230	S82109P	Fracture of lower limb
230	S82109Q	Fracture of lower limb
230	S82109R	Fracture of lower limb
230	S82109S	Fracture of lower limb
230	S82111A	Fracture of lower limb
230	S82111B	Fracture of lower limb
230	S82111C	Fracture of lower limb
230	S82111D	Fracture of lower limb
230	S82111E	Fracture of lower limb
230	S82111F	Fracture of lower limb
230	S82111G	Fracture of lower limb
230	S82111H	Fracture of lower limb
230	S82111J	Fracture of lower limb

230	S82111K	Fracture of lower limb
230	S82111M	Fracture of lower limb
230	S82111N	Fracture of lower limb
230	S82111P	Fracture of lower limb
230	S82111Q	Fracture of lower limb
230	S82111R	Fracture of lower limb
230	S82111S	Fracture of lower limb
230	S82112A	Fracture of lower limb
230	S82112B	Fracture of lower limb
230	S82112C	Fracture of lower limb
230	S82112D	Fracture of lower limb
230	S82112E	Fracture of lower limb
230	S82112F	Fracture of lower limb
230	S82112G	Fracture of lower limb
230	S82112H	Fracture of lower limb
230	S82112J	Fracture of lower limb
230	S82112K	Fracture of lower limb
230	S82112M	Fracture of lower limb
230	S82112N	Fracture of lower limb
230	S82112P	Fracture of lower limb
230	S82112Q	Fracture of lower limb
230	S82112R	Fracture of lower limb
230	S82112S	Fracture of lower limb
230	S82113A	Fracture of lower limb
230	S82113B	Fracture of lower limb
230	S82113C	Fracture of lower limb
230	S82113D	Fracture of lower limb
230	S82113E	Fracture of lower limb
230	S82113F	Fracture of lower limb
230	S82113G	Fracture of lower limb
230	S82113H	Fracture of lower limb
230	S82113J	Fracture of lower limb
230	S82113K	Fracture of lower limb
230	S82113M	Fracture of lower limb
230	S82113N	Fracture of lower limb
230	S82113P	Fracture of lower limb
230	S82113Q	Fracture of lower limb
230	S82113R	Fracture of lower limb
230	S82113S	Fracture of lower limb
230	S82114A	Fracture of lower limb
230	S82114B	Fracture of lower limb
230	S82114C	Fracture of lower limb
230	S82114D	Fracture of lower limb
230	S82114E	Fracture of lower limb
230	S82114F	Fracture of lower limb
230	S82114G	Fracture of lower limb
230	S82114H	Fracture of lower limb
230	S82114J	Fracture of lower limb
230	S82114K	Fracture of lower limb
230	S82114M	Fracture of lower limb
230	S82114N	Fracture of lower limb

230	S82114P	Fracture of lower limb
230	S82114Q	Fracture of lower limb
230	S82114R	Fracture of lower limb
230	S82114S	Fracture of lower limb
230	S82115A	Fracture of lower limb
230	S82115B	Fracture of lower limb
230	S82115C	Fracture of lower limb
230	S82115D	Fracture of lower limb
230	S82115E	Fracture of lower limb
230	S82115F	Fracture of lower limb
230	S82115G	Fracture of lower limb
230	S82115H	Fracture of lower limb
230	S82115J	Fracture of lower limb
230	S82115K	Fracture of lower limb
230	S82115M	Fracture of lower limb
230	S82115N	Fracture of lower limb
230	S82115P	Fracture of lower limb
230	S82115Q	Fracture of lower limb
230	S82115R	Fracture of lower limb
230	S82115S	Fracture of lower limb
230	S82116A	Fracture of lower limb
230	S82116B	Fracture of lower limb
230	S82116C	Fracture of lower limb
230	S82116D	Fracture of lower limb
230	S82116E	Fracture of lower limb
230	S82116F	Fracture of lower limb
230	S82116G	Fracture of lower limb
230	S82116H	Fracture of lower limb
230	S82116J	Fracture of lower limb
230	S82116K	Fracture of lower limb
230	S82116M	Fracture of lower limb
230	S82116N	Fracture of lower limb
230	S82116P	Fracture of lower limb
230	S82116Q	Fracture of lower limb
230	S82116R	Fracture of lower limb
230	S82116S	Fracture of lower limb
230	S82121A	Fracture of lower limb
230	S82121B	Fracture of lower limb
230	S82121C	Fracture of lower limb
230	S82121D	Fracture of lower limb
230	S82121E	Fracture of lower limb
230	S82121F	Fracture of lower limb
230	S82121G	Fracture of lower limb
230	S82121H	Fracture of lower limb
230	S82121J	Fracture of lower limb
230	S82121K	Fracture of lower limb
230	S82121M	Fracture of lower limb
230	S82121N	Fracture of lower limb
230	S82121P	Fracture of lower limb
230	S82121Q	Fracture of lower limb
230	S82121R	Fracture of lower limb

230	S82121S	Fracture of lower limb
230	S82122A	Fracture of lower limb
230	S82122B	Fracture of lower limb
230	S82122C	Fracture of lower limb
230	S82122D	Fracture of lower limb
230	S82122E	Fracture of lower limb
230	S82122F	Fracture of lower limb
230	S82122G	Fracture of lower limb
230	S82122H	Fracture of lower limb
230	S82122J	Fracture of lower limb
230	S82122K	Fracture of lower limb
230	S82122M	Fracture of lower limb
230	S82122N	Fracture of lower limb
230	S82122P	Fracture of lower limb
230	S82122Q	Fracture of lower limb
230	S82122R	Fracture of lower limb
230	S82122S	Fracture of lower limb
230	S82123A	Fracture of lower limb
230	S82123B	Fracture of lower limb
230	S82123C	Fracture of lower limb
230	S82123D	Fracture of lower limb
230	S82123E	Fracture of lower limb
230	S82123F	Fracture of lower limb
230	S82123G	Fracture of lower limb
230	S82123H	Fracture of lower limb
230	S82123J	Fracture of lower limb
230	S82123K	Fracture of lower limb
230	S82123M	Fracture of lower limb
230	S82123N	Fracture of lower limb
230	S82123P	Fracture of lower limb
230	S82123Q	Fracture of lower limb
230	S82123R	Fracture of lower limb
230	S82123S	Fracture of lower limb
230	S82124A	Fracture of lower limb
230	S82124B	Fracture of lower limb
230	S82124C	Fracture of lower limb
230	S82124D	Fracture of lower limb
230	S82124E	Fracture of lower limb
230	S82124F	Fracture of lower limb
230	S82124G	Fracture of lower limb
230	S82124H	Fracture of lower limb
230	S82124J	Fracture of lower limb
230	S82124K	Fracture of lower limb
230	S82124M	Fracture of lower limb
230	S82124N	Fracture of lower limb
230	S82124P	Fracture of lower limb
230	S82124Q	Fracture of lower limb
230	S82124R	Fracture of lower limb
230	S82124S	Fracture of lower limb
230	S82125A	Fracture of lower limb
230	S82125B	Fracture of lower limb

230	S82125C	Fracture of lower limb
230	S82125D	Fracture of lower limb
230	S82125E	Fracture of lower limb
230	S82125F	Fracture of lower limb
230	S82125G	Fracture of lower limb
230	S82125H	Fracture of lower limb
230	S82125J	Fracture of lower limb
230	S82125K	Fracture of lower limb
230	S82125M	Fracture of lower limb
230	S82125N	Fracture of lower limb
230	S82125P	Fracture of lower limb
230	S82125Q	Fracture of lower limb
230	S82125R	Fracture of lower limb
230	S82125S	Fracture of lower limb
230	S82126A	Fracture of lower limb
230	S82126B	Fracture of lower limb
230	S82126C	Fracture of lower limb
230	S82126D	Fracture of lower limb
230	S82126E	Fracture of lower limb
230	S82126F	Fracture of lower limb
230	S82126G	Fracture of lower limb
230	S82126H	Fracture of lower limb
230	S82126J	Fracture of lower limb
230	S82126K	Fracture of lower limb
230	S82126M	Fracture of lower limb
230	S82126N	Fracture of lower limb
230	S82126P	Fracture of lower limb
230	S82126Q	Fracture of lower limb
230	S82126R	Fracture of lower limb
230	S82126S	Fracture of lower limb
230	S82131A	Fracture of lower limb
230	S82131B	Fracture of lower limb
230	S82131C	Fracture of lower limb
230	S82131D	Fracture of lower limb
230	S82131E	Fracture of lower limb
230	S82131F	Fracture of lower limb
230	S82131G	Fracture of lower limb
230	S82131H	Fracture of lower limb
230	S82131J	Fracture of lower limb
230	S82131K	Fracture of lower limb
230	S82131M	Fracture of lower limb
230	S82131N	Fracture of lower limb
230	S82131P	Fracture of lower limb
230	S82131Q	Fracture of lower limb
230	S82131R	Fracture of lower limb
230	S82131S	Fracture of lower limb
230	S82132A	Fracture of lower limb
230	S82132B	Fracture of lower limb
230	S82132C	Fracture of lower limb
230	S82132D	Fracture of lower limb
230	S82132E	Fracture of lower limb

230	S82132F	Fracture of lower limb
230	S82132G	Fracture of lower limb
230	S82132H	Fracture of lower limb
230	S82132J	Fracture of lower limb
230	S82132K	Fracture of lower limb
230	S82132M	Fracture of lower limb
230	S82132N	Fracture of lower limb
230	S82132P	Fracture of lower limb
230	S82132Q	Fracture of lower limb
230	S82132R	Fracture of lower limb
230	S82132S	Fracture of lower limb
230	S82133A	Fracture of lower limb
230	S82133B	Fracture of lower limb
230	S82133C	Fracture of lower limb
230	S82133D	Fracture of lower limb
230	S82133E	Fracture of lower limb
230	S82133F	Fracture of lower limb
230	S82133G	Fracture of lower limb
230	S82133H	Fracture of lower limb
230	S82133J	Fracture of lower limb
230	S82133K	Fracture of lower limb
230	S82133M	Fracture of lower limb
230	S82133N	Fracture of lower limb
230	S82133P	Fracture of lower limb
230	S82133Q	Fracture of lower limb
230	S82133R	Fracture of lower limb
230	S82133S	Fracture of lower limb
230	S82134A	Fracture of lower limb
230	S82134B	Fracture of lower limb
230	S82134C	Fracture of lower limb
230	S82134D	Fracture of lower limb
230	S82134E	Fracture of lower limb
230	S82134F	Fracture of lower limb
230	S82134G	Fracture of lower limb
230	S82134H	Fracture of lower limb
230	S82134J	Fracture of lower limb
230	S82134K	Fracture of lower limb
230	S82134M	Fracture of lower limb
230	S82134N	Fracture of lower limb
230	S82134P	Fracture of lower limb
230	S82134Q	Fracture of lower limb
230	S82134R	Fracture of lower limb
230	S82134S	Fracture of lower limb
230	S82135A	Fracture of lower limb
230	S82135B	Fracture of lower limb
230	S82135C	Fracture of lower limb
230	S82135D	Fracture of lower limb
230	S82135E	Fracture of lower limb
230	S82135F	Fracture of lower limb
230	S82135G	Fracture of lower limb
230	S82135H	Fracture of lower limb

230	S82135J	Fracture of lower limb
230	S82135K	Fracture of lower limb
230	S82135M	Fracture of lower limb
230	S82135N	Fracture of lower limb
230	S82135P	Fracture of lower limb
230	S82135Q	Fracture of lower limb
230	S82135R	Fracture of lower limb
230	S82135S	Fracture of lower limb
230	S82136A	Fracture of lower limb
230	S82136B	Fracture of lower limb
230	S82136C	Fracture of lower limb
230	S82136D	Fracture of lower limb
230	S82136E	Fracture of lower limb
230	S82136F	Fracture of lower limb
230	S82136G	Fracture of lower limb
230	S82136H	Fracture of lower limb
230	S82136J	Fracture of lower limb
230	S82136K	Fracture of lower limb
230	S82136M	Fracture of lower limb
230	S82136N	Fracture of lower limb
230	S82136P	Fracture of lower limb
230	S82136Q	Fracture of lower limb
230	S82136R	Fracture of lower limb
230	S82136S	Fracture of lower limb
230	S82141A	Fracture of lower limb
230	S82141B	Fracture of lower limb
230	S82141C	Fracture of lower limb
230	S82141D	Fracture of lower limb
230	S82141E	Fracture of lower limb
230	S82141F	Fracture of lower limb
230	S82141G	Fracture of lower limb
230	S82141H	Fracture of lower limb
230	S82141J	Fracture of lower limb
230	S82141K	Fracture of lower limb
230	S82141M	Fracture of lower limb
230	S82141N	Fracture of lower limb
230	S82141P	Fracture of lower limb
230	S82141Q	Fracture of lower limb
230	S82141R	Fracture of lower limb
230	S82141S	Fracture of lower limb
230	S82142A	Fracture of lower limb
230	S82142B	Fracture of lower limb
230	S82142C	Fracture of lower limb
230	S82142D	Fracture of lower limb
230	S82142E	Fracture of lower limb
230	S82142F	Fracture of lower limb
230	S82142G	Fracture of lower limb
230	S82142H	Fracture of lower limb
230	S82142J	Fracture of lower limb
230	S82142K	Fracture of lower limb
230	S82142M	Fracture of lower limb

230	S82142N	Fracture of lower limb
230	S82142P	Fracture of lower limb
230	S82142Q	Fracture of lower limb
230	S82142R	Fracture of lower limb
230	S82142S	Fracture of lower limb
230	S82143A	Fracture of lower limb
230	S82143B	Fracture of lower limb
230	S82143C	Fracture of lower limb
230	S82143D	Fracture of lower limb
230	S82143E	Fracture of lower limb
230	S82143F	Fracture of lower limb
230	S82143G	Fracture of lower limb
230	S82143H	Fracture of lower limb
230	S82143J	Fracture of lower limb
230	S82143K	Fracture of lower limb
230	S82143M	Fracture of lower limb
230	S82143N	Fracture of lower limb
230	S82143P	Fracture of lower limb
230	S82143Q	Fracture of lower limb
230	S82143R	Fracture of lower limb
230	S82143S	Fracture of lower limb
230	S82144A	Fracture of lower limb
230	S82144B	Fracture of lower limb
230	S82144C	Fracture of lower limb
230	S82144D	Fracture of lower limb
230	S82144E	Fracture of lower limb
230	S82144F	Fracture of lower limb
230	S82144G	Fracture of lower limb
230	S82144H	Fracture of lower limb
230	S82144J	Fracture of lower limb
230	S82144K	Fracture of lower limb
230	S82144M	Fracture of lower limb
230	S82144N	Fracture of lower limb
230	S82144P	Fracture of lower limb
230	S82144Q	Fracture of lower limb
230	S82144R	Fracture of lower limb
230	S82144S	Fracture of lower limb
230	S82145A	Fracture of lower limb
230	S82145B	Fracture of lower limb
230	S82145C	Fracture of lower limb
230	S82145D	Fracture of lower limb
230	S82145E	Fracture of lower limb
230	S82145F	Fracture of lower limb
230	S82145G	Fracture of lower limb
230	S82145H	Fracture of lower limb
230	S82145J	Fracture of lower limb
230	S82145K	Fracture of lower limb
230	S82145M	Fracture of lower limb
230	S82145N	Fracture of lower limb
230	S82145P	Fracture of lower limb
230	S82145Q	Fracture of lower limb

230	S82145R	Fracture of lower limb
230	S82145S	Fracture of lower limb
230	S82146A	Fracture of lower limb
230	S82146B	Fracture of lower limb
230	S82146C	Fracture of lower limb
230	S82146D	Fracture of lower limb
230	S82146E	Fracture of lower limb
230	S82146F	Fracture of lower limb
230	S82146G	Fracture of lower limb
230	S82146H	Fracture of lower limb
230	S82146J	Fracture of lower limb
230	S82146K	Fracture of lower limb
230	S82146M	Fracture of lower limb
230	S82146N	Fracture of lower limb
230	S82146P	Fracture of lower limb
230	S82146Q	Fracture of lower limb
230	S82146R	Fracture of lower limb
230	S82146S	Fracture of lower limb
230	S82151A	Fracture of lower limb
230	S82151B	Fracture of lower limb
230	S82151C	Fracture of lower limb
230	S82151D	Fracture of lower limb
230	S82151E	Fracture of lower limb
230	S82151F	Fracture of lower limb
230	S82151G	Fracture of lower limb
230	S82151H	Fracture of lower limb
230	S82151J	Fracture of lower limb
230	S82151K	Fracture of lower limb
230	S82151M	Fracture of lower limb
230	S82151N	Fracture of lower limb
230	S82151P	Fracture of lower limb
230	S82151Q	Fracture of lower limb
230	S82151R	Fracture of lower limb
230	S82151S	Fracture of lower limb
230	S82152A	Fracture of lower limb
230	S82152B	Fracture of lower limb
230	S82152C	Fracture of lower limb
230	S82152D	Fracture of lower limb
230	S82152E	Fracture of lower limb
230	S82152F	Fracture of lower limb
230	S82152G	Fracture of lower limb
230	S82152H	Fracture of lower limb
230	S82152J	Fracture of lower limb
230	S82152K	Fracture of lower limb
230	S82152M	Fracture of lower limb
230	S82152N	Fracture of lower limb
230	S82152P	Fracture of lower limb
230	S82152Q	Fracture of lower limb
230	S82152R	Fracture of lower limb
230	S82152S	Fracture of lower limb
230	S82153A	Fracture of lower limb

230	S82153B	Fracture of lower limb
230	S82153C	Fracture of lower limb
230	S82153D	Fracture of lower limb
230	S82153E	Fracture of lower limb
230	S82153F	Fracture of lower limb
230	S82153G	Fracture of lower limb
230	S82153H	Fracture of lower limb
230	S82153J	Fracture of lower limb
230	S82153K	Fracture of lower limb
230	S82153M	Fracture of lower limb
230	S82153N	Fracture of lower limb
230	S82153P	Fracture of lower limb
230	S82153Q	Fracture of lower limb
230	S82153R	Fracture of lower limb
230	S82153S	Fracture of lower limb
230	S82154A	Fracture of lower limb
230	S82154B	Fracture of lower limb
230	S82154C	Fracture of lower limb
230	S82154D	Fracture of lower limb
230	S82154E	Fracture of lower limb
230	S82154F	Fracture of lower limb
230	S82154G	Fracture of lower limb
230	S82154H	Fracture of lower limb
230	S82154J	Fracture of lower limb
230	S82154K	Fracture of lower limb
230	S82154M	Fracture of lower limb
230	S82154N	Fracture of lower limb
230	S82154P	Fracture of lower limb
230	S82154Q	Fracture of lower limb
230	S82154R	Fracture of lower limb
230	S82154S	Fracture of lower limb
230	S82155A	Fracture of lower limb
230	S82155B	Fracture of lower limb
230	S82155C	Fracture of lower limb
230	S82155D	Fracture of lower limb
230	S82155E	Fracture of lower limb
230	S82155F	Fracture of lower limb
230	S82155G	Fracture of lower limb
230	S82155H	Fracture of lower limb
230	S82155J	Fracture of lower limb
230	S82155K	Fracture of lower limb
230	S82155M	Fracture of lower limb
230	S82155N	Fracture of lower limb
230	S82155P	Fracture of lower limb
230	S82155Q	Fracture of lower limb
230	S82155R	Fracture of lower limb
230	S82155S	Fracture of lower limb
230	S82156A	Fracture of lower limb
230	S82156B	Fracture of lower limb
230	S82156C	Fracture of lower limb
230	S82156D	Fracture of lower limb

230	S82156E	Fracture of lower limb
230	S82156F	Fracture of lower limb
230	S82156G	Fracture of lower limb
230	S82156H	Fracture of lower limb
230	S82156J	Fracture of lower limb
230	S82156K	Fracture of lower limb
230	S82156M	Fracture of lower limb
230	S82156N	Fracture of lower limb
230	S82156P	Fracture of lower limb
230	S82156Q	Fracture of lower limb
230	S82156R	Fracture of lower limb
230	S82156S	Fracture of lower limb
230	S82161A	Fracture of lower limb
230	S82161D	Fracture of lower limb
230	S82161G	Fracture of lower limb
230	S82161K	Fracture of lower limb
230	S82161P	Fracture of lower limb
230	S82161S	Fracture of lower limb
230	S82162A	Fracture of lower limb
230	S82162D	Fracture of lower limb
230	S82162G	Fracture of lower limb
230	S82162K	Fracture of lower limb
230	S82162P	Fracture of lower limb
230	S82162S	Fracture of lower limb
230	S82169A	Fracture of lower limb
230	S82169D	Fracture of lower limb
230	S82169G	Fracture of lower limb
230	S82169K	Fracture of lower limb
230	S82169P	Fracture of lower limb
230	S82169S	Fracture of lower limb
230	S82191A	Fracture of lower limb
230	S82191B	Fracture of lower limb
230	S82191C	Fracture of lower limb
230	S82191D	Fracture of lower limb
230	S82191E	Fracture of lower limb
230	S82191F	Fracture of lower limb
230	S82191G	Fracture of lower limb
230	S82191H	Fracture of lower limb
230	S82191J	Fracture of lower limb
230	S82191K	Fracture of lower limb
230	S82191M	Fracture of lower limb
230	S82191N	Fracture of lower limb
230	S82191P	Fracture of lower limb
230	S82191Q	Fracture of lower limb
230	S82191R	Fracture of lower limb
230	S82191S	Fracture of lower limb
230	S82192A	Fracture of lower limb
230	S82192B	Fracture of lower limb
230	S82192C	Fracture of lower limb
230	S82192D	Fracture of lower limb
230	S82192E	Fracture of lower limb

230	S82192F	Fracture of lower limb
230	S82192G	Fracture of lower limb
230	S82192H	Fracture of lower limb
230	S82192J	Fracture of lower limb
230	S82192K	Fracture of lower limb
230	S82192M	Fracture of lower limb
230	S82192N	Fracture of lower limb
230	S82192P	Fracture of lower limb
230	S82192Q	Fracture of lower limb
230	S82192R	Fracture of lower limb
230	S82192S	Fracture of lower limb
230	S82199A	Fracture of lower limb
230	S82199B	Fracture of lower limb
230	S82199C	Fracture of lower limb
230	S82199D	Fracture of lower limb
230	S82199E	Fracture of lower limb
230	S82199F	Fracture of lower limb
230	S82199G	Fracture of lower limb
230	S82199H	Fracture of lower limb
230	S82199J	Fracture of lower limb
230	S82199K	Fracture of lower limb
230	S82199M	Fracture of lower limb
230	S82199N	Fracture of lower limb
230	S82199P	Fracture of lower limb
230	S82199Q	Fracture of lower limb
230	S82199R	Fracture of lower limb
230	S82199S	Fracture of lower limb
230	S82201A	Fracture of lower limb
230	S82201B	Fracture of lower limb
230	S82201C	Fracture of lower limb
230	S82201D	Fracture of lower limb
230	S82201E	Fracture of lower limb
230	S82201F	Fracture of lower limb
230	S82201G	Fracture of lower limb
230	S82201H	Fracture of lower limb
230	S82201J	Fracture of lower limb
230	S82201K	Fracture of lower limb
230	S82201M	Fracture of lower limb
230	S82201N	Fracture of lower limb
230	S82201P	Fracture of lower limb
230	S82201Q	Fracture of lower limb
230	S82201R	Fracture of lower limb
230	S82201S	Fracture of lower limb
230	S82202A	Fracture of lower limb
230	S82202B	Fracture of lower limb
230	S82202C	Fracture of lower limb
230	S82202D	Fracture of lower limb
230	S82202E	Fracture of lower limb
230	S82202F	Fracture of lower limb
230	S82202G	Fracture of lower limb
230	S82202H	Fracture of lower limb

230	S82202J	Fracture of lower limb
230	S82202K	Fracture of lower limb
230	S82202M	Fracture of lower limb
230	S82202N	Fracture of lower limb
230	S82202P	Fracture of lower limb
230	S82202Q	Fracture of lower limb
230	S82202R	Fracture of lower limb
230	S82202S	Fracture of lower limb
230	S82209A	Fracture of lower limb
230	S82209B	Fracture of lower limb
230	S82209C	Fracture of lower limb
230	S82209D	Fracture of lower limb
230	S82209E	Fracture of lower limb
230	S82209F	Fracture of lower limb
230	S82209G	Fracture of lower limb
230	S82209H	Fracture of lower limb
230	S82209J	Fracture of lower limb
230	S82209K	Fracture of lower limb
230	S82209M	Fracture of lower limb
230	S82209N	Fracture of lower limb
230	S82209P	Fracture of lower limb
230	S82209Q	Fracture of lower limb
230	S82209R	Fracture of lower limb
230	S82209S	Fracture of lower limb
230	S82221A	Fracture of lower limb
230	S82221B	Fracture of lower limb
230	S82221C	Fracture of lower limb
230	S82221D	Fracture of lower limb
230	S82221E	Fracture of lower limb
230	S82221F	Fracture of lower limb
230	S82221G	Fracture of lower limb
230	S82221H	Fracture of lower limb
230	S82221J	Fracture of lower limb
230	S82221K	Fracture of lower limb
230	S82221M	Fracture of lower limb
230	S82221N	Fracture of lower limb
230	S82221P	Fracture of lower limb
230	S82221Q	Fracture of lower limb
230	S82221R	Fracture of lower limb
230	S82221S	Fracture of lower limb
230	S82222A	Fracture of lower limb
230	S82222B	Fracture of lower limb
230	S82222C	Fracture of lower limb
230	S82222D	Fracture of lower limb
230	S82222E	Fracture of lower limb
230	S82222F	Fracture of lower limb
230	S82222G	Fracture of lower limb
230	S82222H	Fracture of lower limb
230	S82222J	Fracture of lower limb
230	S82222K	Fracture of lower limb
230	S82222M	Fracture of lower limb

230	S82222N	Fracture of lower limb
230	S82222P	Fracture of lower limb
230	S82222Q	Fracture of lower limb
230	S82222R	Fracture of lower limb
230	S82222S	Fracture of lower limb
230	S82223A	Fracture of lower limb
230	S82223B	Fracture of lower limb
230	S82223C	Fracture of lower limb
230	S82223D	Fracture of lower limb
230	S82223E	Fracture of lower limb
230	S82223F	Fracture of lower limb
230	S82223G	Fracture of lower limb
230	S82223H	Fracture of lower limb
230	S82223J	Fracture of lower limb
230	S82223K	Fracture of lower limb
230	S82223M	Fracture of lower limb
230	S82223N	Fracture of lower limb
230	S82223P	Fracture of lower limb
230	S82223Q	Fracture of lower limb
230	S82223R	Fracture of lower limb
230	S82223S	Fracture of lower limb
230	S82224A	Fracture of lower limb
230	S82224B	Fracture of lower limb
230	S82224C	Fracture of lower limb
230	S82224D	Fracture of lower limb
230	S82224E	Fracture of lower limb
230	S82224F	Fracture of lower limb
230	S82224G	Fracture of lower limb
230	S82224H	Fracture of lower limb
230	S82224J	Fracture of lower limb
230	S82224K	Fracture of lower limb
230	S82224M	Fracture of lower limb
230	S82224N	Fracture of lower limb
230	S82224P	Fracture of lower limb
230	S82224Q	Fracture of lower limb
230	S82224R	Fracture of lower limb
230	S82224S	Fracture of lower limb
230	S82225A	Fracture of lower limb
230	S82225B	Fracture of lower limb
230	S82225C	Fracture of lower limb
230	S82225D	Fracture of lower limb
230	S82225E	Fracture of lower limb
230	S82225F	Fracture of lower limb
230	S82225G	Fracture of lower limb
230	S82225H	Fracture of lower limb
230	S82225J	Fracture of lower limb
230	S82225K	Fracture of lower limb
230	S82225M	Fracture of lower limb
230	S82225N	Fracture of lower limb
230	S82225P	Fracture of lower limb
230	S82225Q	Fracture of lower limb

230	S82225R	Fracture of lower limb
230	S82225S	Fracture of lower limb
230	S82226A	Fracture of lower limb
230	S82226B	Fracture of lower limb
230	S82226C	Fracture of lower limb
230	S82226D	Fracture of lower limb
230	S82226E	Fracture of lower limb
230	S82226F	Fracture of lower limb
230	S82226G	Fracture of lower limb
230	S82226H	Fracture of lower limb
230	S82226J	Fracture of lower limb
230	S82226K	Fracture of lower limb
230	S82226M	Fracture of lower limb
230	S82226N	Fracture of lower limb
230	S82226P	Fracture of lower limb
230	S82226Q	Fracture of lower limb
230	S82226R	Fracture of lower limb
230	S82226S	Fracture of lower limb
230	S82231A	Fracture of lower limb
230	S82231B	Fracture of lower limb
230	S82231C	Fracture of lower limb
230	S82231D	Fracture of lower limb
230	S82231E	Fracture of lower limb
230	S82231F	Fracture of lower limb
230	S82231G	Fracture of lower limb
230	S82231H	Fracture of lower limb
230	S82231J	Fracture of lower limb
230	S82231K	Fracture of lower limb
230	S82231M	Fracture of lower limb
230	S82231N	Fracture of lower limb
230	S82231P	Fracture of lower limb
230	S82231Q	Fracture of lower limb
230	S82231R	Fracture of lower limb
230	S82231S	Fracture of lower limb
230	S82232A	Fracture of lower limb
230	S82232B	Fracture of lower limb
230	S82232C	Fracture of lower limb
230	S82232D	Fracture of lower limb
230	S82232E	Fracture of lower limb
230	S82232F	Fracture of lower limb
230	S82232G	Fracture of lower limb
230	S82232H	Fracture of lower limb
230	S82232J	Fracture of lower limb
230	S82232K	Fracture of lower limb
230	S82232M	Fracture of lower limb
230	S82232N	Fracture of lower limb
230	S82232P	Fracture of lower limb
230	S82232Q	Fracture of lower limb
230	S82232R	Fracture of lower limb
230	S82232S	Fracture of lower limb
230	S82233A	Fracture of lower limb

230	S82233B	Fracture of lower limb
230	S82233C	Fracture of lower limb
230	S82233D	Fracture of lower limb
230	S82233E	Fracture of lower limb
230	S82233F	Fracture of lower limb
230	S82233G	Fracture of lower limb
230	S82233H	Fracture of lower limb
230	S82233J	Fracture of lower limb
230	S82233K	Fracture of lower limb
230	S82233M	Fracture of lower limb
230	S82233N	Fracture of lower limb
230	S82233P	Fracture of lower limb
230	S82233Q	Fracture of lower limb
230	S82233R	Fracture of lower limb
230	S82233S	Fracture of lower limb
230	S82234A	Fracture of lower limb
230	S82234B	Fracture of lower limb
230	S82234C	Fracture of lower limb
230	S82234D	Fracture of lower limb
230	S82234E	Fracture of lower limb
230	S82234F	Fracture of lower limb
230	S82234G	Fracture of lower limb
230	S82234H	Fracture of lower limb
230	S82234J	Fracture of lower limb
230	S82234K	Fracture of lower limb
230	S82234M	Fracture of lower limb
230	S82234N	Fracture of lower limb
230	S82234P	Fracture of lower limb
230	S82234Q	Fracture of lower limb
230	S82234R	Fracture of lower limb
230	S82234S	Fracture of lower limb
230	S82235A	Fracture of lower limb
230	S82235B	Fracture of lower limb
230	S82235C	Fracture of lower limb
230	S82235D	Fracture of lower limb
230	S82235E	Fracture of lower limb
230	S82235F	Fracture of lower limb
230	S82235G	Fracture of lower limb
230	S82235H	Fracture of lower limb
230	S82235J	Fracture of lower limb
230	S82235K	Fracture of lower limb
230	S82235M	Fracture of lower limb
230	S82235N	Fracture of lower limb
230	S82235P	Fracture of lower limb
230	S82235Q	Fracture of lower limb
230	S82235R	Fracture of lower limb
230	S82235S	Fracture of lower limb
230	S82236A	Fracture of lower limb
230	S82236B	Fracture of lower limb
230	S82236C	Fracture of lower limb
230	S82236D	Fracture of lower limb

230	S82236E	Fracture of lower limb
230	S82236F	Fracture of lower limb
230	S82236G	Fracture of lower limb
230	S82236H	Fracture of lower limb
230	S82236J	Fracture of lower limb
230	S82236K	Fracture of lower limb
230	S82236M	Fracture of lower limb
230	S82236N	Fracture of lower limb
230	S82236P	Fracture of lower limb
230	S82236Q	Fracture of lower limb
230	S82236R	Fracture of lower limb
230	S82236S	Fracture of lower limb
230	S82241A	Fracture of lower limb
230	S82241B	Fracture of lower limb
230	S82241C	Fracture of lower limb
230	S82241D	Fracture of lower limb
230	S82241E	Fracture of lower limb
230	S82241F	Fracture of lower limb
230	S82241G	Fracture of lower limb
230	S82241H	Fracture of lower limb
230	S82241J	Fracture of lower limb
230	S82241K	Fracture of lower limb
230	S82241M	Fracture of lower limb
230	S82241N	Fracture of lower limb
230	S82241P	Fracture of lower limb
230	S82241Q	Fracture of lower limb
230	S82241R	Fracture of lower limb
230	S82241S	Fracture of lower limb
230	S82242A	Fracture of lower limb
230	S82242B	Fracture of lower limb
230	S82242C	Fracture of lower limb
230	S82242D	Fracture of lower limb
230	S82242E	Fracture of lower limb
230	S82242F	Fracture of lower limb
230	S82242G	Fracture of lower limb
230	S82242H	Fracture of lower limb
230	S82242J	Fracture of lower limb
230	S82242K	Fracture of lower limb
230	S82242M	Fracture of lower limb
230	S82242N	Fracture of lower limb
230	S82242P	Fracture of lower limb
230	S82242Q	Fracture of lower limb
230	S82242R	Fracture of lower limb
230	S82242S	Fracture of lower limb
230	S82243A	Fracture of lower limb
230	S82243B	Fracture of lower limb
230	S82243C	Fracture of lower limb
230	S82243D	Fracture of lower limb
230	S82243E	Fracture of lower limb
230	S82243F	Fracture of lower limb
230	S82243G	Fracture of lower limb

230	S82243H	Fracture of lower limb
230	S82243J	Fracture of lower limb
230	S82243K	Fracture of lower limb
230	S82243M	Fracture of lower limb
230	S82243N	Fracture of lower limb
230	S82243P	Fracture of lower limb
230	S82243Q	Fracture of lower limb
230	S82243R	Fracture of lower limb
230	S82243S	Fracture of lower limb
230	S82244A	Fracture of lower limb
230	S82244B	Fracture of lower limb
230	S82244C	Fracture of lower limb
230	S82244D	Fracture of lower limb
230	S82244E	Fracture of lower limb
230	S82244F	Fracture of lower limb
230	S82244G	Fracture of lower limb
230	S82244H	Fracture of lower limb
230	S82244J	Fracture of lower limb
230	S82244K	Fracture of lower limb
230	S82244M	Fracture of lower limb
230	S82244N	Fracture of lower limb
230	S82244P	Fracture of lower limb
230	S82244Q	Fracture of lower limb
230	S82244R	Fracture of lower limb
230	S82244S	Fracture of lower limb
230	S82245A	Fracture of lower limb
230	S82245B	Fracture of lower limb
230	S82245C	Fracture of lower limb
230	S82245D	Fracture of lower limb
230	S82245E	Fracture of lower limb
230	S82245F	Fracture of lower limb
230	S82245G	Fracture of lower limb
230	S82245H	Fracture of lower limb
230	S82245J	Fracture of lower limb
230	S82245K	Fracture of lower limb
230	S82245M	Fracture of lower limb
230	S82245N	Fracture of lower limb
230	S82245P	Fracture of lower limb
230	S82245Q	Fracture of lower limb
230	S82245R	Fracture of lower limb
230	S82245S	Fracture of lower limb
230	S82246A	Fracture of lower limb
230	S82246B	Fracture of lower limb
230	S82246C	Fracture of lower limb
230	S82246D	Fracture of lower limb
230	S82246E	Fracture of lower limb
230	S82246F	Fracture of lower limb
230	S82246G	Fracture of lower limb
230	S82246H	Fracture of lower limb
230	S82246J	Fracture of lower limb
230	S82246K	Fracture of lower limb

230	S82246M	Fracture of lower limb
230	S82246N	Fracture of lower limb
230	S82246P	Fracture of lower limb
230	S82246Q	Fracture of lower limb
230	S82246R	Fracture of lower limb
230	S82246S	Fracture of lower limb
230	S82251A	Fracture of lower limb
230	S82251B	Fracture of lower limb
230	S82251C	Fracture of lower limb
230	S82251D	Fracture of lower limb
230	S82251E	Fracture of lower limb
230	S82251F	Fracture of lower limb
230	S82251G	Fracture of lower limb
230	S82251H	Fracture of lower limb
230	S82251J	Fracture of lower limb
230	S82251K	Fracture of lower limb
230	S82251M	Fracture of lower limb
230	S82251N	Fracture of lower limb
230	S82251P	Fracture of lower limb
230	S82251Q	Fracture of lower limb
230	S82251R	Fracture of lower limb
230	S82251S	Fracture of lower limb
230	S82252A	Fracture of lower limb
230	S82252B	Fracture of lower limb
230	S82252C	Fracture of lower limb
230	S82252D	Fracture of lower limb
230	S82252E	Fracture of lower limb
230	S82252F	Fracture of lower limb
230	S82252G	Fracture of lower limb
230	S82252H	Fracture of lower limb
230	S82252J	Fracture of lower limb
230	S82252K	Fracture of lower limb
230	S82252M	Fracture of lower limb
230	S82252N	Fracture of lower limb
230	S82252P	Fracture of lower limb
230	S82252Q	Fracture of lower limb
230	S82252R	Fracture of lower limb
230	S82252S	Fracture of lower limb
230	S82253A	Fracture of lower limb
230	S82253B	Fracture of lower limb
230	S82253C	Fracture of lower limb
230	S82253D	Fracture of lower limb
230	S82253E	Fracture of lower limb
230	S82253F	Fracture of lower limb
230	S82253G	Fracture of lower limb
230	S82253H	Fracture of lower limb
230	S82253J	Fracture of lower limb
230	S82253K	Fracture of lower limb
230	S82253M	Fracture of lower limb
230	S82253N	Fracture of lower limb
230	S82253P	Fracture of lower limb

230	S82253Q	Fracture of lower limb
230	S82253R	Fracture of lower limb
230	S82253S	Fracture of lower limb
230	S82254A	Fracture of lower limb
230	S82254B	Fracture of lower limb
230	S82254C	Fracture of lower limb
230	S82254D	Fracture of lower limb
230	S82254E	Fracture of lower limb
230	S82254F	Fracture of lower limb
230	S82254G	Fracture of lower limb
230	S82254H	Fracture of lower limb
230	S82254J	Fracture of lower limb
230	S82254K	Fracture of lower limb
230	S82254M	Fracture of lower limb
230	S82254N	Fracture of lower limb
230	S82254P	Fracture of lower limb
230	S82254Q	Fracture of lower limb
230	S82254R	Fracture of lower limb
230	S82254S	Fracture of lower limb
230	S82255A	Fracture of lower limb
230	S82255B	Fracture of lower limb
230	S82255C	Fracture of lower limb
230	S82255D	Fracture of lower limb
230	S82255E	Fracture of lower limb
230	S82255F	Fracture of lower limb
230	S82255G	Fracture of lower limb
230	S82255H	Fracture of lower limb
230	S82255J	Fracture of lower limb
230	S82255K	Fracture of lower limb
230	S82255M	Fracture of lower limb
230	S82255N	Fracture of lower limb
230	S82255P	Fracture of lower limb
230	S82255Q	Fracture of lower limb
230	S82255R	Fracture of lower limb
230	S82255S	Fracture of lower limb
230	S82256A	Fracture of lower limb
230	S82256B	Fracture of lower limb
230	S82256C	Fracture of lower limb
230	S82256D	Fracture of lower limb
230	S82256E	Fracture of lower limb
230	S82256F	Fracture of lower limb
230	S82256G	Fracture of lower limb
230	S82256H	Fracture of lower limb
230	S82256J	Fracture of lower limb
230	S82256K	Fracture of lower limb
230	S82256M	Fracture of lower limb
230	S82256N	Fracture of lower limb
230	S82256P	Fracture of lower limb
230	S82256Q	Fracture of lower limb
230	S82256R	Fracture of lower limb
230	S82256S	Fracture of lower limb

230	S82261A	Fracture of lower limb
230	S82261B	Fracture of lower limb
230	S82261C	Fracture of lower limb
230	S82261D	Fracture of lower limb
230	S82261E	Fracture of lower limb
230	S82261F	Fracture of lower limb
230	S82261G	Fracture of lower limb
230	S82261H	Fracture of lower limb
230	S82261J	Fracture of lower limb
230	S82261K	Fracture of lower limb
230	S82261M	Fracture of lower limb
230	S82261N	Fracture of lower limb
230	S82261P	Fracture of lower limb
230	S82261Q	Fracture of lower limb
230	S82261R	Fracture of lower limb
230	S82261S	Fracture of lower limb
230	S82262A	Fracture of lower limb
230	S82262B	Fracture of lower limb
230	S82262C	Fracture of lower limb
230	S82262D	Fracture of lower limb
230	S82262E	Fracture of lower limb
230	S82262F	Fracture of lower limb
230	S82262G	Fracture of lower limb
230	S82262H	Fracture of lower limb
230	S82262J	Fracture of lower limb
230	S82262K	Fracture of lower limb
230	S82262M	Fracture of lower limb
230	S82262N	Fracture of lower limb
230	S82262P	Fracture of lower limb
230	S82262Q	Fracture of lower limb
230	S82262R	Fracture of lower limb
230	S82262S	Fracture of lower limb
230	S82263A	Fracture of lower limb
230	S82263B	Fracture of lower limb
230	S82263C	Fracture of lower limb
230	S82263D	Fracture of lower limb
230	S82263E	Fracture of lower limb
230	S82263F	Fracture of lower limb
230	S82263G	Fracture of lower limb
230	S82263H	Fracture of lower limb
230	S82263J	Fracture of lower limb
230	S82263K	Fracture of lower limb
230	S82263M	Fracture of lower limb
230	S82263N	Fracture of lower limb
230	S82263P	Fracture of lower limb
230	S82263Q	Fracture of lower limb
230	S82263R	Fracture of lower limb
230	S82263S	Fracture of lower limb
230	S82264A	Fracture of lower limb
230	S82264B	Fracture of lower limb
230	S82264C	Fracture of lower limb

230	S82264D	Fracture of lower limb
230	S82264E	Fracture of lower limb
230	S82264F	Fracture of lower limb
230	S82264G	Fracture of lower limb
230	S82264H	Fracture of lower limb
230	S82264J	Fracture of lower limb
230	S82264K	Fracture of lower limb
230	S82264M	Fracture of lower limb
230	S82264N	Fracture of lower limb
230	S82264P	Fracture of lower limb
230	S82264Q	Fracture of lower limb
230	S82264R	Fracture of lower limb
230	S82264S	Fracture of lower limb
230	S82265A	Fracture of lower limb
230	S82265B	Fracture of lower limb
230	S82265C	Fracture of lower limb
230	S82265D	Fracture of lower limb
230	S82265E	Fracture of lower limb
230	S82265F	Fracture of lower limb
230	S82265G	Fracture of lower limb
230	S82265H	Fracture of lower limb
230	S82265J	Fracture of lower limb
230	S82265K	Fracture of lower limb
230	S82265M	Fracture of lower limb
230	S82265N	Fracture of lower limb
230	S82265P	Fracture of lower limb
230	S82265Q	Fracture of lower limb
230	S82265R	Fracture of lower limb
230	S82265S	Fracture of lower limb
230	S82266A	Fracture of lower limb
230	S82266B	Fracture of lower limb
230	S82266C	Fracture of lower limb
230	S82266D	Fracture of lower limb
230	S82266E	Fracture of lower limb
230	S82266F	Fracture of lower limb
230	S82266G	Fracture of lower limb
230	S82266H	Fracture of lower limb
230	S82266J	Fracture of lower limb
230	S82266K	Fracture of lower limb
230	S82266M	Fracture of lower limb
230	S82266N	Fracture of lower limb
230	S82266P	Fracture of lower limb
230	S82266Q	Fracture of lower limb
230	S82266R	Fracture of lower limb
230	S82266S	Fracture of lower limb
230	S82291A	Fracture of lower limb
230	S82291B	Fracture of lower limb
230	S82291C	Fracture of lower limb
230	S82291D	Fracture of lower limb
230	S82291E	Fracture of lower limb
230	S82291F	Fracture of lower limb

230	S82291G	Fracture of lower limb
230	S82291H	Fracture of lower limb
230	S82291J	Fracture of lower limb
230	S82291K	Fracture of lower limb
230	S82291M	Fracture of lower limb
230	S82291N	Fracture of lower limb
230	S82291P	Fracture of lower limb
230	S82291Q	Fracture of lower limb
230	S82291R	Fracture of lower limb
230	S82291S	Fracture of lower limb
230	S82292A	Fracture of lower limb
230	S82292B	Fracture of lower limb
230	S82292C	Fracture of lower limb
230	S82292D	Fracture of lower limb
230	S82292E	Fracture of lower limb
230	S82292F	Fracture of lower limb
230	S82292G	Fracture of lower limb
230	S82292H	Fracture of lower limb
230	S82292J	Fracture of lower limb
230	S82292K	Fracture of lower limb
230	S82292M	Fracture of lower limb
230	S82292N	Fracture of lower limb
230	S82292P	Fracture of lower limb
230	S82292Q	Fracture of lower limb
230	S82292R	Fracture of lower limb
230	S82292S	Fracture of lower limb
230	S82299A	Fracture of lower limb
230	S82299B	Fracture of lower limb
230	S82299C	Fracture of lower limb
230	S82299D	Fracture of lower limb
230	S82299E	Fracture of lower limb
230	S82299F	Fracture of lower limb
230	S82299G	Fracture of lower limb
230	S82299H	Fracture of lower limb
230	S82299J	Fracture of lower limb
230	S82299K	Fracture of lower limb
230	S82299M	Fracture of lower limb
230	S82299N	Fracture of lower limb
230	S82299P	Fracture of lower limb
230	S82299Q	Fracture of lower limb
230	S82299R	Fracture of lower limb
230	S82299S	Fracture of lower limb
230	S82301A	Fracture of lower limb
230	S82301B	Fracture of lower limb
230	S82301C	Fracture of lower limb
230	S82301D	Fracture of lower limb
230	S82301E	Fracture of lower limb
230	S82301F	Fracture of lower limb
230	S82301G	Fracture of lower limb
230	S82301H	Fracture of lower limb
230	S82301J	Fracture of lower limb

230	S82301K	Fracture of lower limb
230	S82301M	Fracture of lower limb
230	S82301N	Fracture of lower limb
230	S82301P	Fracture of lower limb
230	S82301Q	Fracture of lower limb
230	S82301R	Fracture of lower limb
230	S82301S	Fracture of lower limb
230	S82302A	Fracture of lower limb
230	S82302B	Fracture of lower limb
230	S82302C	Fracture of lower limb
230	S82302D	Fracture of lower limb
230	S82302E	Fracture of lower limb
230	S82302F	Fracture of lower limb
230	S82302G	Fracture of lower limb
230	S82302H	Fracture of lower limb
230	S82302J	Fracture of lower limb
230	S82302K	Fracture of lower limb
230	S82302M	Fracture of lower limb
230	S82302N	Fracture of lower limb
230	S82302P	Fracture of lower limb
230	S82302Q	Fracture of lower limb
230	S82302R	Fracture of lower limb
230	S82302S	Fracture of lower limb
230	S82309A	Fracture of lower limb
230	S82309B	Fracture of lower limb
230	S82309C	Fracture of lower limb
230	S82309D	Fracture of lower limb
230	S82309E	Fracture of lower limb
230	S82309F	Fracture of lower limb
230	S82309G	Fracture of lower limb
230	S82309H	Fracture of lower limb
230	S82309J	Fracture of lower limb
230	S82309K	Fracture of lower limb
230	S82309M	Fracture of lower limb
230	S82309N	Fracture of lower limb
230	S82309P	Fracture of lower limb
230	S82309Q	Fracture of lower limb
230	S82309R	Fracture of lower limb
230	S82309S	Fracture of lower limb
230	S82311A	Fracture of lower limb
230	S82311D	Fracture of lower limb
230	S82311G	Fracture of lower limb
230	S82311K	Fracture of lower limb
230	S82311P	Fracture of lower limb
230	S82311S	Fracture of lower limb
230	S82312A	Fracture of lower limb
230	S82312D	Fracture of lower limb
230	S82312G	Fracture of lower limb
230	S82312K	Fracture of lower limb
230	S82312P	Fracture of lower limb
230	S82312S	Fracture of lower limb

230	S82319A	Fracture of lower limb
230	S82319D	Fracture of lower limb
230	S82319G	Fracture of lower limb
230	S82319K	Fracture of lower limb
230	S82319P	Fracture of lower limb
230	S82319S	Fracture of lower limb
230	S82391A	Fracture of lower limb
230	S82391B	Fracture of lower limb
230	S82391C	Fracture of lower limb
230	S82391D	Fracture of lower limb
230	S82391E	Fracture of lower limb
230	S82391F	Fracture of lower limb
230	S82391G	Fracture of lower limb
230	S82391H	Fracture of lower limb
230	S82391J	Fracture of lower limb
230	S82391K	Fracture of lower limb
230	S82391M	Fracture of lower limb
230	S82391N	Fracture of lower limb
230	S82391P	Fracture of lower limb
230	S82391Q	Fracture of lower limb
230	S82391R	Fracture of lower limb
230	S82391S	Fracture of lower limb
230	S82392A	Fracture of lower limb
230	S82392B	Fracture of lower limb
230	S82392C	Fracture of lower limb
230	S82392D	Fracture of lower limb
230	S82392E	Fracture of lower limb
230	S82392F	Fracture of lower limb
230	S82392G	Fracture of lower limb
230	S82392H	Fracture of lower limb
230	S82392J	Fracture of lower limb
230	S82392K	Fracture of lower limb
230	S82392M	Fracture of lower limb
230	S82392N	Fracture of lower limb
230	S82392P	Fracture of lower limb
230	S82392Q	Fracture of lower limb
230	S82392R	Fracture of lower limb
230	S82392S	Fracture of lower limb
230	S82399A	Fracture of lower limb
230	S82399B	Fracture of lower limb
230	S82399C	Fracture of lower limb
230	S82399D	Fracture of lower limb
230	S82399E	Fracture of lower limb
230	S82399F	Fracture of lower limb
230	S82399G	Fracture of lower limb
230	S82399H	Fracture of lower limb
230	S82399J	Fracture of lower limb
230	S82399K	Fracture of lower limb
230	S82399M	Fracture of lower limb
230	S82399N	Fracture of lower limb
230	S82399P	Fracture of lower limb

230	S82399Q	Fracture of lower limb
230	S82399R	Fracture of lower limb
230	S82399S	Fracture of lower limb
230	S82401A	Fracture of lower limb
230	S82401B	Fracture of lower limb
230	S82401C	Fracture of lower limb
230	S82401D	Fracture of lower limb
230	S82401E	Fracture of lower limb
230	S82401F	Fracture of lower limb
230	S82401G	Fracture of lower limb
230	S82401H	Fracture of lower limb
230	S82401J	Fracture of lower limb
230	S82401K	Fracture of lower limb
230	S82401M	Fracture of lower limb
230	S82401N	Fracture of lower limb
230	S82401P	Fracture of lower limb
230	S82401Q	Fracture of lower limb
230	S82401R	Fracture of lower limb
230	S82401S	Fracture of lower limb
230	S82402A	Fracture of lower limb
230	S82402B	Fracture of lower limb
230	S82402C	Fracture of lower limb
230	S82402D	Fracture of lower limb
230	S82402E	Fracture of lower limb
230	S82402F	Fracture of lower limb
230	S82402G	Fracture of lower limb
230	S82402H	Fracture of lower limb
230	S82402J	Fracture of lower limb
230	S82402K	Fracture of lower limb
230	S82402M	Fracture of lower limb
230	S82402N	Fracture of lower limb
230	S82402P	Fracture of lower limb
230	S82402Q	Fracture of lower limb
230	S82402R	Fracture of lower limb
230	S82402S	Fracture of lower limb
230	S82409A	Fracture of lower limb
230	S82409B	Fracture of lower limb
230	S82409C	Fracture of lower limb
230	S82409D	Fracture of lower limb
230	S82409E	Fracture of lower limb
230	S82409F	Fracture of lower limb
230	S82409G	Fracture of lower limb
230	S82409H	Fracture of lower limb
230	S82409J	Fracture of lower limb
230	S82409K	Fracture of lower limb
230	S82409M	Fracture of lower limb
230	S82409N	Fracture of lower limb
230	S82409P	Fracture of lower limb
230	S82409Q	Fracture of lower limb
230	S82409R	Fracture of lower limb
230	S82409S	Fracture of lower limb

230	S82421A	Fracture of lower limb
230	S82421B	Fracture of lower limb
230	S82421C	Fracture of lower limb
230	S82421D	Fracture of lower limb
230	S82421E	Fracture of lower limb
230	S82421F	Fracture of lower limb
230	S82421G	Fracture of lower limb
230	S82421H	Fracture of lower limb
230	S82421J	Fracture of lower limb
230	S82421K	Fracture of lower limb
230	S82421M	Fracture of lower limb
230	S82421N	Fracture of lower limb
230	S82421P	Fracture of lower limb
230	S82421Q	Fracture of lower limb
230	S82421R	Fracture of lower limb
230	S82421S	Fracture of lower limb
230	S82422A	Fracture of lower limb
230	S82422B	Fracture of lower limb
230	S82422C	Fracture of lower limb
230	S82422D	Fracture of lower limb
230	S82422E	Fracture of lower limb
230	S82422F	Fracture of lower limb
230	S82422G	Fracture of lower limb
230	S82422H	Fracture of lower limb
230	S82422J	Fracture of lower limb
230	S82422K	Fracture of lower limb
230	S82422M	Fracture of lower limb
230	S82422N	Fracture of lower limb
230	S82422P	Fracture of lower limb
230	S82422Q	Fracture of lower limb
230	S82422R	Fracture of lower limb
230	S82422S	Fracture of lower limb
230	S82423A	Fracture of lower limb
230	S82423B	Fracture of lower limb
230	S82423C	Fracture of lower limb
230	S82423D	Fracture of lower limb
230	S82423E	Fracture of lower limb
230	S82423F	Fracture of lower limb
230	S82423G	Fracture of lower limb
230	S82423H	Fracture of lower limb
230	S82423J	Fracture of lower limb
230	S82423K	Fracture of lower limb
230	S82423M	Fracture of lower limb
230	S82423N	Fracture of lower limb
230	S82423P	Fracture of lower limb
230	S82423Q	Fracture of lower limb
230	S82423R	Fracture of lower limb
230	S82423S	Fracture of lower limb
230	S82424A	Fracture of lower limb
230	S82424B	Fracture of lower limb
230	S82424C	Fracture of lower limb

230	S82424D	Fracture of lower limb
230	S82424E	Fracture of lower limb
230	S82424F	Fracture of lower limb
230	S82424G	Fracture of lower limb
230	S82424H	Fracture of lower limb
230	S82424J	Fracture of lower limb
230	S82424K	Fracture of lower limb
230	S82424M	Fracture of lower limb
230	S82424N	Fracture of lower limb
230	S82424P	Fracture of lower limb
230	S82424Q	Fracture of lower limb
230	S82424R	Fracture of lower limb
230	S82424S	Fracture of lower limb
230	S82425A	Fracture of lower limb
230	S82425B	Fracture of lower limb
230	S82425C	Fracture of lower limb
230	S82425D	Fracture of lower limb
230	S82425E	Fracture of lower limb
230	S82425F	Fracture of lower limb
230	S82425G	Fracture of lower limb
230	S82425H	Fracture of lower limb
230	S82425J	Fracture of lower limb
230	S82425K	Fracture of lower limb
230	S82425M	Fracture of lower limb
230	S82425N	Fracture of lower limb
230	S82425P	Fracture of lower limb
230	S82425Q	Fracture of lower limb
230	S82425R	Fracture of lower limb
230	S82425S	Fracture of lower limb
230	S82426A	Fracture of lower limb
230	S82426B	Fracture of lower limb
230	S82426C	Fracture of lower limb
230	S82426D	Fracture of lower limb
230	S82426E	Fracture of lower limb
230	S82426F	Fracture of lower limb
230	S82426G	Fracture of lower limb
230	S82426H	Fracture of lower limb
230	S82426J	Fracture of lower limb
230	S82426K	Fracture of lower limb
230	S82426M	Fracture of lower limb
230	S82426N	Fracture of lower limb
230	S82426P	Fracture of lower limb
230	S82426Q	Fracture of lower limb
230	S82426R	Fracture of lower limb
230	S82426S	Fracture of lower limb
230	S82431A	Fracture of lower limb
230	S82431B	Fracture of lower limb
230	S82431C	Fracture of lower limb
230	S82431D	Fracture of lower limb
230	S82431E	Fracture of lower limb
230	S82431F	Fracture of lower limb

230	S82431G	Fracture of lower limb
230	S82431H	Fracture of lower limb
230	S82431J	Fracture of lower limb
230	S82431K	Fracture of lower limb
230	S82431M	Fracture of lower limb
230	S82431N	Fracture of lower limb
230	S82431P	Fracture of lower limb
230	S82431Q	Fracture of lower limb
230	S82431R	Fracture of lower limb
230	S82431S	Fracture of lower limb
230	S82432A	Fracture of lower limb
230	S82432B	Fracture of lower limb
230	S82432C	Fracture of lower limb
230	S82432D	Fracture of lower limb
230	S82432E	Fracture of lower limb
230	S82432F	Fracture of lower limb
230	S82432G	Fracture of lower limb
230	S82432H	Fracture of lower limb
230	S82432J	Fracture of lower limb
230	S82432K	Fracture of lower limb
230	S82432M	Fracture of lower limb
230	S82432N	Fracture of lower limb
230	S82432P	Fracture of lower limb
230	S82432Q	Fracture of lower limb
230	S82432R	Fracture of lower limb
230	S82432S	Fracture of lower limb
230	S82433A	Fracture of lower limb
230	S82433B	Fracture of lower limb
230	S82433C	Fracture of lower limb
230	S82433D	Fracture of lower limb
230	S82433E	Fracture of lower limb
230	S82433F	Fracture of lower limb
230	S82433G	Fracture of lower limb
230	S82433H	Fracture of lower limb
230	S82433J	Fracture of lower limb
230	S82433K	Fracture of lower limb
230	S82433M	Fracture of lower limb
230	S82433N	Fracture of lower limb
230	S82433P	Fracture of lower limb
230	S82433Q	Fracture of lower limb
230	S82433R	Fracture of lower limb
230	S82433S	Fracture of lower limb
230	S82434A	Fracture of lower limb
230	S82434B	Fracture of lower limb
230	S82434C	Fracture of lower limb
230	S82434D	Fracture of lower limb
230	S82434E	Fracture of lower limb
230	S82434F	Fracture of lower limb
230	S82434G	Fracture of lower limb
230	S82434H	Fracture of lower limb
230	S82434J	Fracture of lower limb

230	S82434K	Fracture of lower limb
230	S82434M	Fracture of lower limb
230	S82434N	Fracture of lower limb
230	S82434P	Fracture of lower limb
230	S82434Q	Fracture of lower limb
230	S82434R	Fracture of lower limb
230	S82434S	Fracture of lower limb
230	S82435A	Fracture of lower limb
230	S82435B	Fracture of lower limb
230	S82435C	Fracture of lower limb
230	S82435D	Fracture of lower limb
230	S82435E	Fracture of lower limb
230	S82435F	Fracture of lower limb
230	S82435G	Fracture of lower limb
230	S82435H	Fracture of lower limb
230	S82435J	Fracture of lower limb
230	S82435K	Fracture of lower limb
230	S82435M	Fracture of lower limb
230	S82435N	Fracture of lower limb
230	S82435P	Fracture of lower limb
230	S82435Q	Fracture of lower limb
230	S82435R	Fracture of lower limb
230	S82435S	Fracture of lower limb
230	S82436A	Fracture of lower limb
230	S82436B	Fracture of lower limb
230	S82436C	Fracture of lower limb
230	S82436D	Fracture of lower limb
230	S82436E	Fracture of lower limb
230	S82436F	Fracture of lower limb
230	S82436G	Fracture of lower limb
230	S82436H	Fracture of lower limb
230	S82436J	Fracture of lower limb
230	S82436K	Fracture of lower limb
230	S82436M	Fracture of lower limb
230	S82436N	Fracture of lower limb
230	S82436P	Fracture of lower limb
230	S82436Q	Fracture of lower limb
230	S82436R	Fracture of lower limb
230	S82436S	Fracture of lower limb
230	S82441A	Fracture of lower limb
230	S82441B	Fracture of lower limb
230	S82441C	Fracture of lower limb
230	S82441D	Fracture of lower limb
230	S82441E	Fracture of lower limb
230	S82441F	Fracture of lower limb
230	S82441G	Fracture of lower limb
230	S82441H	Fracture of lower limb
230	S82441J	Fracture of lower limb
230	S82441K	Fracture of lower limb
230	S82441M	Fracture of lower limb
230	S82441N	Fracture of lower limb

230	S82441P	Fracture of lower limb
230	S82441Q	Fracture of lower limb
230	S82441R	Fracture of lower limb
230	S82441S	Fracture of lower limb
230	S82442A	Fracture of lower limb
230	S82442B	Fracture of lower limb
230	S82442C	Fracture of lower limb
230	S82442D	Fracture of lower limb
230	S82442E	Fracture of lower limb
230	S82442F	Fracture of lower limb
230	S82442G	Fracture of lower limb
230	S82442H	Fracture of lower limb
230	S82442J	Fracture of lower limb
230	S82442K	Fracture of lower limb
230	S82442M	Fracture of lower limb
230	S82442N	Fracture of lower limb
230	S82442P	Fracture of lower limb
230	S82442Q	Fracture of lower limb
230	S82442R	Fracture of lower limb
230	S82442S	Fracture of lower limb
230	S82443A	Fracture of lower limb
230	S82443B	Fracture of lower limb
230	S82443C	Fracture of lower limb
230	S82443D	Fracture of lower limb
230	S82443E	Fracture of lower limb
230	S82443F	Fracture of lower limb
230	S82443G	Fracture of lower limb
230	S82443H	Fracture of lower limb
230	S82443J	Fracture of lower limb
230	S82443K	Fracture of lower limb
230	S82443M	Fracture of lower limb
230	S82443N	Fracture of lower limb
230	S82443P	Fracture of lower limb
230	S82443Q	Fracture of lower limb
230	S82443R	Fracture of lower limb
230	S82443S	Fracture of lower limb
230	S82444A	Fracture of lower limb
230	S82444B	Fracture of lower limb
230	S82444C	Fracture of lower limb
230	S82444D	Fracture of lower limb
230	S82444E	Fracture of lower limb
230	S82444F	Fracture of lower limb
230	S82444G	Fracture of lower limb
230	S82444H	Fracture of lower limb
230	S82444J	Fracture of lower limb
230	S82444K	Fracture of lower limb
230	S82444M	Fracture of lower limb
230	S82444N	Fracture of lower limb
230	S82444P	Fracture of lower limb
230	S82444Q	Fracture of lower limb
230	S82444R	Fracture of lower limb

230	S82444S	Fracture of lower limb
230	S82445A	Fracture of lower limb
230	S82445B	Fracture of lower limb
230	S82445C	Fracture of lower limb
230	S82445D	Fracture of lower limb
230	S82445E	Fracture of lower limb
230	S82445F	Fracture of lower limb
230	S82445G	Fracture of lower limb
230	S82445H	Fracture of lower limb
230	S82445J	Fracture of lower limb
230	S82445K	Fracture of lower limb
230	S82445M	Fracture of lower limb
230	S82445N	Fracture of lower limb
230	S82445P	Fracture of lower limb
230	S82445Q	Fracture of lower limb
230	S82445R	Fracture of lower limb
230	S82445S	Fracture of lower limb
230	S82446A	Fracture of lower limb
230	S82446B	Fracture of lower limb
230	S82446C	Fracture of lower limb
230	S82446D	Fracture of lower limb
230	S82446E	Fracture of lower limb
230	S82446F	Fracture of lower limb
230	S82446G	Fracture of lower limb
230	S82446H	Fracture of lower limb
230	S82446J	Fracture of lower limb
230	S82446K	Fracture of lower limb
230	S82446M	Fracture of lower limb
230	S82446N	Fracture of lower limb
230	S82446P	Fracture of lower limb
230	S82446Q	Fracture of lower limb
230	S82446R	Fracture of lower limb
230	S82446S	Fracture of lower limb
230	S82451A	Fracture of lower limb
230	S82451B	Fracture of lower limb
230	S82451C	Fracture of lower limb
230	S82451D	Fracture of lower limb
230	S82451E	Fracture of lower limb
230	S82451F	Fracture of lower limb
230	S82451G	Fracture of lower limb
230	S82451H	Fracture of lower limb
230	S82451J	Fracture of lower limb
230	S82451K	Fracture of lower limb
230	S82451M	Fracture of lower limb
230	S82451N	Fracture of lower limb
230	S82451P	Fracture of lower limb
230	S82451Q	Fracture of lower limb
230	S82451R	Fracture of lower limb
230	S82451S	Fracture of lower limb
230	S82452A	Fracture of lower limb
230	S82452B	Fracture of lower limb

230	S82452C	Fracture of lower limb
230	S82452D	Fracture of lower limb
230	S82452E	Fracture of lower limb
230	S82452F	Fracture of lower limb
230	S82452G	Fracture of lower limb
230	S82452H	Fracture of lower limb
230	S82452J	Fracture of lower limb
230	S82452K	Fracture of lower limb
230	S82452M	Fracture of lower limb
230	S82452N	Fracture of lower limb
230	S82452P	Fracture of lower limb
230	S82452Q	Fracture of lower limb
230	S82452R	Fracture of lower limb
230	S82452S	Fracture of lower limb
230	S82453A	Fracture of lower limb
230	S82453B	Fracture of lower limb
230	S82453C	Fracture of lower limb
230	S82453D	Fracture of lower limb
230	S82453E	Fracture of lower limb
230	S82453F	Fracture of lower limb
230	S82453G	Fracture of lower limb
230	S82453H	Fracture of lower limb
230	S82453J	Fracture of lower limb
230	S82453K	Fracture of lower limb
230	S82453M	Fracture of lower limb
230	S82453N	Fracture of lower limb
230	S82453P	Fracture of lower limb
230	S82453Q	Fracture of lower limb
230	S82453R	Fracture of lower limb
230	S82453S	Fracture of lower limb
230	S82454A	Fracture of lower limb
230	S82454B	Fracture of lower limb
230	S82454C	Fracture of lower limb
230	S82454D	Fracture of lower limb
230	S82454E	Fracture of lower limb
230	S82454F	Fracture of lower limb
230	S82454G	Fracture of lower limb
230	S82454H	Fracture of lower limb
230	S82454J	Fracture of lower limb
230	S82454K	Fracture of lower limb
230	S82454M	Fracture of lower limb
230	S82454N	Fracture of lower limb
230	S82454P	Fracture of lower limb
230	S82454Q	Fracture of lower limb
230	S82454R	Fracture of lower limb
230	S82454S	Fracture of lower limb
230	S82455A	Fracture of lower limb
230	S82455B	Fracture of lower limb
230	S82455C	Fracture of lower limb
230	S82455D	Fracture of lower limb
230	S82455E	Fracture of lower limb

230	S82455F	Fracture of lower limb
230	S82455G	Fracture of lower limb
230	S82455H	Fracture of lower limb
230	S82455J	Fracture of lower limb
230	S82455K	Fracture of lower limb
230	S82455M	Fracture of lower limb
230	S82455N	Fracture of lower limb
230	S82455P	Fracture of lower limb
230	S82455Q	Fracture of lower limb
230	S82455R	Fracture of lower limb
230	S82455S	Fracture of lower limb
230	S82456A	Fracture of lower limb
230	S82456B	Fracture of lower limb
230	S82456C	Fracture of lower limb
230	S82456D	Fracture of lower limb
230	S82456E	Fracture of lower limb
230	S82456F	Fracture of lower limb
230	S82456G	Fracture of lower limb
230	S82456H	Fracture of lower limb
230	S82456J	Fracture of lower limb
230	S82456K	Fracture of lower limb
230	S82456M	Fracture of lower limb
230	S82456N	Fracture of lower limb
230	S82456P	Fracture of lower limb
230	S82456Q	Fracture of lower limb
230	S82456R	Fracture of lower limb
230	S82456S	Fracture of lower limb
230	S82461A	Fracture of lower limb
230	S82461B	Fracture of lower limb
230	S82461C	Fracture of lower limb
230	S82461D	Fracture of lower limb
230	S82461E	Fracture of lower limb
230	S82461F	Fracture of lower limb
230	S82461G	Fracture of lower limb
230	S82461H	Fracture of lower limb
230	S82461J	Fracture of lower limb
230	S82461K	Fracture of lower limb
230	S82461M	Fracture of lower limb
230	S82461N	Fracture of lower limb
230	S82461P	Fracture of lower limb
230	S82461Q	Fracture of lower limb
230	S82461R	Fracture of lower limb
230	S82461S	Fracture of lower limb
230	S82462A	Fracture of lower limb
230	S82462B	Fracture of lower limb
230	S82462C	Fracture of lower limb
230	S82462D	Fracture of lower limb
230	S82462E	Fracture of lower limb
230	S82462F	Fracture of lower limb
230	S82462G	Fracture of lower limb
230	S82462H	Fracture of lower limb

230	S82462J	Fracture of lower limb
230	S82462K	Fracture of lower limb
230	S82462M	Fracture of lower limb
230	S82462N	Fracture of lower limb
230	S82462P	Fracture of lower limb
230	S82462Q	Fracture of lower limb
230	S82462R	Fracture of lower limb
230	S82462S	Fracture of lower limb
230	S82463A	Fracture of lower limb
230	S82463B	Fracture of lower limb
230	S82463C	Fracture of lower limb
230	S82463D	Fracture of lower limb
230	S82463E	Fracture of lower limb
230	S82463F	Fracture of lower limb
230	S82463G	Fracture of lower limb
230	S82463H	Fracture of lower limb
230	S82463J	Fracture of lower limb
230	S82463K	Fracture of lower limb
230	S82463M	Fracture of lower limb
230	S82463N	Fracture of lower limb
230	S82463P	Fracture of lower limb
230	S82463Q	Fracture of lower limb
230	S82463R	Fracture of lower limb
230	S82463S	Fracture of lower limb
230	S82464A	Fracture of lower limb
230	S82464B	Fracture of lower limb
230	S82464C	Fracture of lower limb
230	S82464D	Fracture of lower limb
230	S82464E	Fracture of lower limb
230	S82464F	Fracture of lower limb
230	S82464G	Fracture of lower limb
230	S82464H	Fracture of lower limb
230	S82464J	Fracture of lower limb
230	S82464K	Fracture of lower limb
230	S82464M	Fracture of lower limb
230	S82464N	Fracture of lower limb
230	S82464P	Fracture of lower limb
230	S82464Q	Fracture of lower limb
230	S82464R	Fracture of lower limb
230	S82464S	Fracture of lower limb
230	S82465A	Fracture of lower limb
230	S82465B	Fracture of lower limb
230	S82465C	Fracture of lower limb
230	S82465D	Fracture of lower limb
230	S82465E	Fracture of lower limb
230	S82465F	Fracture of lower limb
230	S82465G	Fracture of lower limb
230	S82465H	Fracture of lower limb
230	S82465J	Fracture of lower limb
230	S82465K	Fracture of lower limb
230	S82465M	Fracture of lower limb

230	S82465N	Fracture of lower limb
230	S82465P	Fracture of lower limb
230	S82465Q	Fracture of lower limb
230	S82465R	Fracture of lower limb
230	S82465S	Fracture of lower limb
230	S82466A	Fracture of lower limb
230	S82466B	Fracture of lower limb
230	S82466C	Fracture of lower limb
230	S82466D	Fracture of lower limb
230	S82466E	Fracture of lower limb
230	S82466F	Fracture of lower limb
230	S82466G	Fracture of lower limb
230	S82466H	Fracture of lower limb
230	S82466J	Fracture of lower limb
230	S82466K	Fracture of lower limb
230	S82466M	Fracture of lower limb
230	S82466N	Fracture of lower limb
230	S82466P	Fracture of lower limb
230	S82466Q	Fracture of lower limb
230	S82466R	Fracture of lower limb
230	S82466S	Fracture of lower limb
230	S82491A	Fracture of lower limb
230	S82491B	Fracture of lower limb
230	S82491C	Fracture of lower limb
230	S82491D	Fracture of lower limb
230	S82491E	Fracture of lower limb
230	S82491F	Fracture of lower limb
230	S82491G	Fracture of lower limb
230	S82491H	Fracture of lower limb
230	S82491J	Fracture of lower limb
230	S82491K	Fracture of lower limb
230	S82491M	Fracture of lower limb
230	S82491N	Fracture of lower limb
230	S82491P	Fracture of lower limb
230	S82491Q	Fracture of lower limb
230	S82491R	Fracture of lower limb
230	S82491S	Fracture of lower limb
230	S82492A	Fracture of lower limb
230	S82492B	Fracture of lower limb
230	S82492C	Fracture of lower limb
230	S82492D	Fracture of lower limb
230	S82492E	Fracture of lower limb
230	S82492F	Fracture of lower limb
230	S82492G	Fracture of lower limb
230	S82492H	Fracture of lower limb
230	S82492J	Fracture of lower limb
230	S82492K	Fracture of lower limb
230	S82492M	Fracture of lower limb
230	S82492N	Fracture of lower limb
230	S82492P	Fracture of lower limb
230	S82492Q	Fracture of lower limb

230	S82492R	Fracture of lower limb
230	S82492S	Fracture of lower limb
230	S82499A	Fracture of lower limb
230	S82499B	Fracture of lower limb
230	S82499C	Fracture of lower limb
230	S82499D	Fracture of lower limb
230	S82499E	Fracture of lower limb
230	S82499F	Fracture of lower limb
230	S82499G	Fracture of lower limb
230	S82499H	Fracture of lower limb
230	S82499J	Fracture of lower limb
230	S82499K	Fracture of lower limb
230	S82499M	Fracture of lower limb
230	S82499N	Fracture of lower limb
230	S82499P	Fracture of lower limb
230	S82499Q	Fracture of lower limb
230	S82499R	Fracture of lower limb
230	S82499S	Fracture of lower limb
230	S8251XA	Fracture of lower limb
230	S8251XB	Fracture of lower limb
230	S8251XC	Fracture of lower limb
230	S8251XD	Fracture of lower limb
230	S8251XE	Fracture of lower limb
230	S8251XF	Fracture of lower limb
230	S8251XG	Fracture of lower limb
230	S8251XH	Fracture of lower limb
230	S8251XJ	Fracture of lower limb
230	S8251XK	Fracture of lower limb
230	S8251XM	Fracture of lower limb
230	S8251XN	Fracture of lower limb
230	S8251XP	Fracture of lower limb
230	S8251XQ	Fracture of lower limb
230	S8251XR	Fracture of lower limb
230	S8251XS	Fracture of lower limb
230	S8252XA	Fracture of lower limb
230	S8252XB	Fracture of lower limb
230	S8252XC	Fracture of lower limb
230	S8252XD	Fracture of lower limb
230	S8252XE	Fracture of lower limb
230	S8252XF	Fracture of lower limb
230	S8252XG	Fracture of lower limb
230	S8252XH	Fracture of lower limb
230	S8252XJ	Fracture of lower limb
230	S8252XK	Fracture of lower limb
230	S8252XM	Fracture of lower limb
230	S8252XN	Fracture of lower limb
230	S8252XP	Fracture of lower limb
230	S8252XQ	Fracture of lower limb
230	S8252XR	Fracture of lower limb
230	S8252XS	Fracture of lower limb
230	S8253XA	Fracture of lower limb

230	S8253XB	Fracture of lower limb
230	S8253XC	Fracture of lower limb
230	S8253XD	Fracture of lower limb
230	S8253XE	Fracture of lower limb
230	S8253XF	Fracture of lower limb
230	S8253XG	Fracture of lower limb
230	S8253XH	Fracture of lower limb
230	S8253XJ	Fracture of lower limb
230	S8253XK	Fracture of lower limb
230	S8253XM	Fracture of lower limb
230	S8253XN	Fracture of lower limb
230	S8253XP	Fracture of lower limb
230	S8253XQ	Fracture of lower limb
230	S8253XR	Fracture of lower limb
230	S8253XS	Fracture of lower limb
230	S8254XA	Fracture of lower limb
230	S8254XB	Fracture of lower limb
230	S8254XC	Fracture of lower limb
230	S8254XD	Fracture of lower limb
230	S8254XE	Fracture of lower limb
230	S8254XF	Fracture of lower limb
230	S8254XG	Fracture of lower limb
230	S8254XH	Fracture of lower limb
230	S8254XJ	Fracture of lower limb
230	S8254XK	Fracture of lower limb
230	S8254XM	Fracture of lower limb
230	S8254XN	Fracture of lower limb
230	S8254XP	Fracture of lower limb
230	S8254XQ	Fracture of lower limb
230	S8254XR	Fracture of lower limb
230	S8254XS	Fracture of lower limb
230	S8255XA	Fracture of lower limb
230	S8255XB	Fracture of lower limb
230	S8255XC	Fracture of lower limb
230	S8255XD	Fracture of lower limb
230	S8255XE	Fracture of lower limb
230	S8255XF	Fracture of lower limb
230	S8255XG	Fracture of lower limb
230	S8255XH	Fracture of lower limb
230	S8255XJ	Fracture of lower limb
230	S8255XK	Fracture of lower limb
230	S8255XM	Fracture of lower limb
230	S8255XN	Fracture of lower limb
230	S8255XP	Fracture of lower limb
230	S8255XQ	Fracture of lower limb
230	S8255XR	Fracture of lower limb
230	S8255XS	Fracture of lower limb
230	S8256XA	Fracture of lower limb
230	S8256XB	Fracture of lower limb
230	S8256XC	Fracture of lower limb
230	S8256XD	Fracture of lower limb

230	S8256XE	Fracture of lower limb
230	S8256XF	Fracture of lower limb
230	S8256XG	Fracture of lower limb
230	S8256XH	Fracture of lower limb
230	S8256XJ	Fracture of lower limb
230	S8256XK	Fracture of lower limb
230	S8256XM	Fracture of lower limb
230	S8256XN	Fracture of lower limb
230	S8256XP	Fracture of lower limb
230	S8256XQ	Fracture of lower limb
230	S8256XR	Fracture of lower limb
230	S8256XS	Fracture of lower limb
230	S8261XA	Fracture of lower limb
230	S8261XB	Fracture of lower limb
230	S8261XC	Fracture of lower limb
230	S8261XD	Fracture of lower limb
230	S8261XE	Fracture of lower limb
230	S8261XF	Fracture of lower limb
230	S8261XG	Fracture of lower limb
230	S8261XH	Fracture of lower limb
230	S8261XJ	Fracture of lower limb
230	S8261XK	Fracture of lower limb
230	S8261XM	Fracture of lower limb
230	S8261XN	Fracture of lower limb
230	S8261XP	Fracture of lower limb
230	S8261XQ	Fracture of lower limb
230	S8261XR	Fracture of lower limb
230	S8261XS	Fracture of lower limb
230	S8262XA	Fracture of lower limb
230	S8262XB	Fracture of lower limb
230	S8262XC	Fracture of lower limb
230	S8262XD	Fracture of lower limb
230	S8262XE	Fracture of lower limb
230	S8262XF	Fracture of lower limb
230	S8262XG	Fracture of lower limb
230	S8262XH	Fracture of lower limb
230	S8262XJ	Fracture of lower limb
230	S8262XK	Fracture of lower limb
230	S8262XM	Fracture of lower limb
230	S8262XN	Fracture of lower limb
230	S8262XP	Fracture of lower limb
230	S8262XQ	Fracture of lower limb
230	S8262XR	Fracture of lower limb
230	S8262XS	Fracture of lower limb
230	S8263XA	Fracture of lower limb
230	S8263XB	Fracture of lower limb
230	S8263XC	Fracture of lower limb
230	S8263XD	Fracture of lower limb
230	S8263XE	Fracture of lower limb
230	S8263XF	Fracture of lower limb
230	S8263XG	Fracture of lower limb

230	S8263XH	Fracture of lower limb
230	S8263XJ	Fracture of lower limb
230	S8263XK	Fracture of lower limb
230	S8263XM	Fracture of lower limb
230	S8263XN	Fracture of lower limb
230	S8263XP	Fracture of lower limb
230	S8263XQ	Fracture of lower limb
230	S8263XR	Fracture of lower limb
230	S8263XS	Fracture of lower limb
230	S8264XA	Fracture of lower limb
230	S8264XB	Fracture of lower limb
230	S8264XC	Fracture of lower limb
230	S8264XD	Fracture of lower limb
230	S8264XE	Fracture of lower limb
230	S8264XF	Fracture of lower limb
230	S8264XG	Fracture of lower limb
230	S8264XH	Fracture of lower limb
230	S8264XJ	Fracture of lower limb
230	S8264XK	Fracture of lower limb
230	S8264XM	Fracture of lower limb
230	S8264XN	Fracture of lower limb
230	S8264XP	Fracture of lower limb
230	S8264XQ	Fracture of lower limb
230	S8264XR	Fracture of lower limb
230	S8264XS	Fracture of lower limb
230	S8265XA	Fracture of lower limb
230	S8265XB	Fracture of lower limb
230	S8265XC	Fracture of lower limb
230	S8265XD	Fracture of lower limb
230	S8265XE	Fracture of lower limb
230	S8265XF	Fracture of lower limb
230	S8265XG	Fracture of lower limb
230	S8265XH	Fracture of lower limb
230	S8265XJ	Fracture of lower limb
230	S8265XK	Fracture of lower limb
230	S8265XM	Fracture of lower limb
230	S8265XN	Fracture of lower limb
230	S8265XP	Fracture of lower limb
230	S8265XQ	Fracture of lower limb
230	S8265XR	Fracture of lower limb
230	S8265XS	Fracture of lower limb
230	S8266XA	Fracture of lower limb
230	S8266XB	Fracture of lower limb
230	S8266XC	Fracture of lower limb
230	S8266XD	Fracture of lower limb
230	S8266XE	Fracture of lower limb
230	S8266XF	Fracture of lower limb
230	S8266XG	Fracture of lower limb
230	S8266XH	Fracture of lower limb
230	S8266XJ	Fracture of lower limb
230	S8266XK	Fracture of lower limb

230	S8266XM	Fracture of lower limb
230	S8266XN	Fracture of lower limb
230	S8266XP	Fracture of lower limb
230	S8266XQ	Fracture of lower limb
230	S8266XR	Fracture of lower limb
230	S8266XS	Fracture of lower limb
230	S82811A	Fracture of lower limb
230	S82811D	Fracture of lower limb
230	S82811G	Fracture of lower limb
230	S82811K	Fracture of lower limb
230	S82811P	Fracture of lower limb
230	S82811S	Fracture of lower limb
230	S82812A	Fracture of lower limb
230	S82812D	Fracture of lower limb
230	S82812G	Fracture of lower limb
230	S82812K	Fracture of lower limb
230	S82812P	Fracture of lower limb
230	S82812S	Fracture of lower limb
230	S82819A	Fracture of lower limb
230	S82819D	Fracture of lower limb
230	S82819G	Fracture of lower limb
230	S82819K	Fracture of lower limb
230	S82819P	Fracture of lower limb
230	S82819S	Fracture of lower limb
230	S82821A	Fracture of lower limb
230	S82821D	Fracture of lower limb
230	S82821G	Fracture of lower limb
230	S82821K	Fracture of lower limb
230	S82821P	Fracture of lower limb
230	S82821S	Fracture of lower limb
230	S82822A	Fracture of lower limb
230	S82822D	Fracture of lower limb
230	S82822G	Fracture of lower limb
230	S82822K	Fracture of lower limb
230	S82822P	Fracture of lower limb
230	S82822S	Fracture of lower limb
230	S82829A	Fracture of lower limb
230	S82829D	Fracture of lower limb
230	S82829G	Fracture of lower limb
230	S82829K	Fracture of lower limb
230	S82829P	Fracture of lower limb
230	S82829S	Fracture of lower limb
230	S82831A	Fracture of lower limb
230	S82831B	Fracture of lower limb
230	S82831C	Fracture of lower limb
230	S82831D	Fracture of lower limb
230	S82831E	Fracture of lower limb
230	S82831F	Fracture of lower limb
230	S82831G	Fracture of lower limb
230	S82831H	Fracture of lower limb
230	S82831J	Fracture of lower limb

230	S82831K	Fracture of lower limb
230	S82831M	Fracture of lower limb
230	S82831N	Fracture of lower limb
230	S82831P	Fracture of lower limb
230	S82831Q	Fracture of lower limb
230	S82831R	Fracture of lower limb
230	S82831S	Fracture of lower limb
230	S82832A	Fracture of lower limb
230	S82832B	Fracture of lower limb
230	S82832C	Fracture of lower limb
230	S82832D	Fracture of lower limb
230	S82832E	Fracture of lower limb
230	S82832F	Fracture of lower limb
230	S82832G	Fracture of lower limb
230	S82832H	Fracture of lower limb
230	S82832J	Fracture of lower limb
230	S82832K	Fracture of lower limb
230	S82832M	Fracture of lower limb
230	S82832N	Fracture of lower limb
230	S82832P	Fracture of lower limb
230	S82832Q	Fracture of lower limb
230	S82832R	Fracture of lower limb
230	S82832S	Fracture of lower limb
230	S82839A	Fracture of lower limb
230	S82839B	Fracture of lower limb
230	S82839C	Fracture of lower limb
230	S82839D	Fracture of lower limb
230	S82839E	Fracture of lower limb
230	S82839F	Fracture of lower limb
230	S82839G	Fracture of lower limb
230	S82839H	Fracture of lower limb
230	S82839J	Fracture of lower limb
230	S82839K	Fracture of lower limb
230	S82839M	Fracture of lower limb
230	S82839N	Fracture of lower limb
230	S82839P	Fracture of lower limb
230	S82839Q	Fracture of lower limb
230	S82839R	Fracture of lower limb
230	S82839S	Fracture of lower limb
230	S82841A	Fracture of lower limb
230	S82841B	Fracture of lower limb
230	S82841C	Fracture of lower limb
230	S82841D	Fracture of lower limb
230	S82841E	Fracture of lower limb
230	S82841F	Fracture of lower limb
230	S82841G	Fracture of lower limb
230	S82841H	Fracture of lower limb
230	S82841J	Fracture of lower limb
230	S82841K	Fracture of lower limb
230	S82841M	Fracture of lower limb
230	S82841N	Fracture of lower limb

230	S82841P	Fracture of lower limb
230	S82841Q	Fracture of lower limb
230	S82841R	Fracture of lower limb
230	S82841S	Fracture of lower limb
230	S82842A	Fracture of lower limb
230	S82842B	Fracture of lower limb
230	S82842C	Fracture of lower limb
230	S82842D	Fracture of lower limb
230	S82842E	Fracture of lower limb
230	S82842F	Fracture of lower limb
230	S82842G	Fracture of lower limb
230	S82842H	Fracture of lower limb
230	S82842J	Fracture of lower limb
230	S82842K	Fracture of lower limb
230	S82842M	Fracture of lower limb
230	S82842N	Fracture of lower limb
230	S82842P	Fracture of lower limb
230	S82842Q	Fracture of lower limb
230	S82842R	Fracture of lower limb
230	S82842S	Fracture of lower limb
230	S82843A	Fracture of lower limb
230	S82843B	Fracture of lower limb
230	S82843C	Fracture of lower limb
230	S82843D	Fracture of lower limb
230	S82843E	Fracture of lower limb
230	S82843F	Fracture of lower limb
230	S82843G	Fracture of lower limb
230	S82843H	Fracture of lower limb
230	S82843J	Fracture of lower limb
230	S82843K	Fracture of lower limb
230	S82843M	Fracture of lower limb
230	S82843N	Fracture of lower limb
230	S82843P	Fracture of lower limb
230	S82843Q	Fracture of lower limb
230	S82843R	Fracture of lower limb
230	S82843S	Fracture of lower limb
230	S82844A	Fracture of lower limb
230	S82844B	Fracture of lower limb
230	S82844C	Fracture of lower limb
230	S82844D	Fracture of lower limb
230	S82844E	Fracture of lower limb
230	S82844F	Fracture of lower limb
230	S82844G	Fracture of lower limb
230	S82844H	Fracture of lower limb
230	S82844J	Fracture of lower limb
230	S82844K	Fracture of lower limb
230	S82844M	Fracture of lower limb
230	S82844N	Fracture of lower limb
230	S82844P	Fracture of lower limb
230	S82844Q	Fracture of lower limb
230	S82844R	Fracture of lower limb

230	S82844S	Fracture of lower limb
230	S82845A	Fracture of lower limb
230	S82845B	Fracture of lower limb
230	S82845C	Fracture of lower limb
230	S82845D	Fracture of lower limb
230	S82845E	Fracture of lower limb
230	S82845F	Fracture of lower limb
230	S82845G	Fracture of lower limb
230	S82845H	Fracture of lower limb
230	S82845J	Fracture of lower limb
230	S82845K	Fracture of lower limb
230	S82845M	Fracture of lower limb
230	S82845N	Fracture of lower limb
230	S82845P	Fracture of lower limb
230	S82845Q	Fracture of lower limb
230	S82845R	Fracture of lower limb
230	S82845S	Fracture of lower limb
230	S82846A	Fracture of lower limb
230	S82846B	Fracture of lower limb
230	S82846C	Fracture of lower limb
230	S82846D	Fracture of lower limb
230	S82846E	Fracture of lower limb
230	S82846F	Fracture of lower limb
230	S82846G	Fracture of lower limb
230	S82846H	Fracture of lower limb
230	S82846J	Fracture of lower limb
230	S82846K	Fracture of lower limb
230	S82846M	Fracture of lower limb
230	S82846N	Fracture of lower limb
230	S82846P	Fracture of lower limb
230	S82846Q	Fracture of lower limb
230	S82846R	Fracture of lower limb
230	S82846S	Fracture of lower limb
230	S82851A	Fracture of lower limb
230	S82851B	Fracture of lower limb
230	S82851C	Fracture of lower limb
230	S82851D	Fracture of lower limb
230	S82851E	Fracture of lower limb
230	S82851F	Fracture of lower limb
230	S82851G	Fracture of lower limb
230	S82851H	Fracture of lower limb
230	S82851J	Fracture of lower limb
230	S82851K	Fracture of lower limb
230	S82851M	Fracture of lower limb
230	S82851N	Fracture of lower limb
230	S82851P	Fracture of lower limb
230	S82851Q	Fracture of lower limb
230	S82851R	Fracture of lower limb
230	S82851S	Fracture of lower limb
230	S82852A	Fracture of lower limb
230	S82852B	Fracture of lower limb

230	S82852C	Fracture of lower limb
230	S82852D	Fracture of lower limb
230	S82852E	Fracture of lower limb
230	S82852F	Fracture of lower limb
230	S82852G	Fracture of lower limb
230	S82852H	Fracture of lower limb
230	S82852J	Fracture of lower limb
230	S82852K	Fracture of lower limb
230	S82852M	Fracture of lower limb
230	S82852N	Fracture of lower limb
230	S82852P	Fracture of lower limb
230	S82852Q	Fracture of lower limb
230	S82852R	Fracture of lower limb
230	S82852S	Fracture of lower limb
230	S82853A	Fracture of lower limb
230	S82853B	Fracture of lower limb
230	S82853C	Fracture of lower limb
230	S82853D	Fracture of lower limb
230	S82853E	Fracture of lower limb
230	S82853F	Fracture of lower limb
230	S82853G	Fracture of lower limb
230	S82853H	Fracture of lower limb
230	S82853J	Fracture of lower limb
230	S82853K	Fracture of lower limb
230	S82853M	Fracture of lower limb
230	S82853N	Fracture of lower limb
230	S82853P	Fracture of lower limb
230	S82853Q	Fracture of lower limb
230	S82853R	Fracture of lower limb
230	S82853S	Fracture of lower limb
230	S82854A	Fracture of lower limb
230	S82854B	Fracture of lower limb
230	S82854C	Fracture of lower limb
230	S82854D	Fracture of lower limb
230	S82854E	Fracture of lower limb
230	S82854F	Fracture of lower limb
230	S82854G	Fracture of lower limb
230	S82854H	Fracture of lower limb
230	S82854J	Fracture of lower limb
230	S82854K	Fracture of lower limb
230	S82854M	Fracture of lower limb
230	S82854N	Fracture of lower limb
230	S82854P	Fracture of lower limb
230	S82854Q	Fracture of lower limb
230	S82854R	Fracture of lower limb
230	S82854S	Fracture of lower limb
230	S82855A	Fracture of lower limb
230	S82855B	Fracture of lower limb
230	S82855C	Fracture of lower limb
230	S82855D	Fracture of lower limb
230	S82855E	Fracture of lower limb

230	S82855F	Fracture of lower limb
230	S82855G	Fracture of lower limb
230	S82855H	Fracture of lower limb
230	S82855J	Fracture of lower limb
230	S82855K	Fracture of lower limb
230	S82855M	Fracture of lower limb
230	S82855N	Fracture of lower limb
230	S82855P	Fracture of lower limb
230	S82855Q	Fracture of lower limb
230	S82855R	Fracture of lower limb
230	S82855S	Fracture of lower limb
230	S82856A	Fracture of lower limb
230	S82856B	Fracture of lower limb
230	S82856C	Fracture of lower limb
230	S82856D	Fracture of lower limb
230	S82856E	Fracture of lower limb
230	S82856F	Fracture of lower limb
230	S82856G	Fracture of lower limb
230	S82856H	Fracture of lower limb
230	S82856J	Fracture of lower limb
230	S82856K	Fracture of lower limb
230	S82856M	Fracture of lower limb
230	S82856N	Fracture of lower limb
230	S82856P	Fracture of lower limb
230	S82856Q	Fracture of lower limb
230	S82856R	Fracture of lower limb
230	S82856S	Fracture of lower limb
230	S82861A	Fracture of lower limb
230	S82861B	Fracture of lower limb
230	S82861C	Fracture of lower limb
230	S82861D	Fracture of lower limb
230	S82861E	Fracture of lower limb
230	S82861F	Fracture of lower limb
230	S82861G	Fracture of lower limb
230	S82861H	Fracture of lower limb
230	S82861J	Fracture of lower limb
230	S82861K	Fracture of lower limb
230	S82861M	Fracture of lower limb
230	S82861N	Fracture of lower limb
230	S82861P	Fracture of lower limb
230	S82861Q	Fracture of lower limb
230	S82861R	Fracture of lower limb
230	S82861S	Fracture of lower limb
230	S82862A	Fracture of lower limb
230	S82862B	Fracture of lower limb
230	S82862C	Fracture of lower limb
230	S82862D	Fracture of lower limb
230	S82862E	Fracture of lower limb
230	S82862F	Fracture of lower limb
230	S82862G	Fracture of lower limb
230	S82862H	Fracture of lower limb

230	S82862J	Fracture of lower limb
230	S82862K	Fracture of lower limb
230	S82862M	Fracture of lower limb
230	S82862N	Fracture of lower limb
230	S82862P	Fracture of lower limb
230	S82862Q	Fracture of lower limb
230	S82862R	Fracture of lower limb
230	S82862S	Fracture of lower limb
230	S82863A	Fracture of lower limb
230	S82863B	Fracture of lower limb
230	S82863C	Fracture of lower limb
230	S82863D	Fracture of lower limb
230	S82863E	Fracture of lower limb
230	S82863F	Fracture of lower limb
230	S82863G	Fracture of lower limb
230	S82863H	Fracture of lower limb
230	S82863J	Fracture of lower limb
230	S82863K	Fracture of lower limb
230	S82863M	Fracture of lower limb
230	S82863N	Fracture of lower limb
230	S82863P	Fracture of lower limb
230	S82863Q	Fracture of lower limb
230	S82863R	Fracture of lower limb
230	S82863S	Fracture of lower limb
230	S82864A	Fracture of lower limb
230	S82864B	Fracture of lower limb
230	S82864C	Fracture of lower limb
230	S82864D	Fracture of lower limb
230	S82864E	Fracture of lower limb
230	S82864F	Fracture of lower limb
230	S82864G	Fracture of lower limb
230	S82864H	Fracture of lower limb
230	S82864J	Fracture of lower limb
230	S82864K	Fracture of lower limb
230	S82864M	Fracture of lower limb
230	S82864N	Fracture of lower limb
230	S82864P	Fracture of lower limb
230	S82864Q	Fracture of lower limb
230	S82864R	Fracture of lower limb
230	S82864S	Fracture of lower limb
230	S82865A	Fracture of lower limb
230	S82865B	Fracture of lower limb
230	S82865C	Fracture of lower limb
230	S82865D	Fracture of lower limb
230	S82865E	Fracture of lower limb
230	S82865F	Fracture of lower limb
230	S82865G	Fracture of lower limb
230	S82865H	Fracture of lower limb
230	S82865J	Fracture of lower limb
230	S82865K	Fracture of lower limb
230	S82865M	Fracture of lower limb

230	S82865N	Fracture of lower limb
230	S82865P	Fracture of lower limb
230	S82865Q	Fracture of lower limb
230	S82865R	Fracture of lower limb
230	S82865S	Fracture of lower limb
230	S82866A	Fracture of lower limb
230	S82866B	Fracture of lower limb
230	S82866C	Fracture of lower limb
230	S82866D	Fracture of lower limb
230	S82866E	Fracture of lower limb
230	S82866F	Fracture of lower limb
230	S82866G	Fracture of lower limb
230	S82866H	Fracture of lower limb
230	S82866J	Fracture of lower limb
230	S82866K	Fracture of lower limb
230	S82866M	Fracture of lower limb
230	S82866N	Fracture of lower limb
230	S82866P	Fracture of lower limb
230	S82866Q	Fracture of lower limb
230	S82866R	Fracture of lower limb
230	S82866S	Fracture of lower limb
230	S82871A	Fracture of lower limb
230	S82871B	Fracture of lower limb
230	S82871C	Fracture of lower limb
230	S82871D	Fracture of lower limb
230	S82871E	Fracture of lower limb
230	S82871F	Fracture of lower limb
230	S82871G	Fracture of lower limb
230	S82871H	Fracture of lower limb
230	S82871J	Fracture of lower limb
230	S82871K	Fracture of lower limb
230	S82871M	Fracture of lower limb
230	S82871N	Fracture of lower limb
230	S82871P	Fracture of lower limb
230	S82871Q	Fracture of lower limb
230	S82871R	Fracture of lower limb
230	S82871S	Fracture of lower limb
230	S82872A	Fracture of lower limb
230	S82872B	Fracture of lower limb
230	S82872C	Fracture of lower limb
230	S82872D	Fracture of lower limb
230	S82872E	Fracture of lower limb
230	S82872F	Fracture of lower limb
230	S82872G	Fracture of lower limb
230	S82872H	Fracture of lower limb
230	S82872J	Fracture of lower limb
230	S82872K	Fracture of lower limb
230	S82872M	Fracture of lower limb
230	S82872N	Fracture of lower limb
230	S82872P	Fracture of lower limb
230	S82872Q	Fracture of lower limb

230	S82872R	Fracture of lower limb
230	S82872S	Fracture of lower limb
230	S82873A	Fracture of lower limb
230	S82873B	Fracture of lower limb
230	S82873C	Fracture of lower limb
230	S82873D	Fracture of lower limb
230	S82873E	Fracture of lower limb
230	S82873F	Fracture of lower limb
230	S82873G	Fracture of lower limb
230	S82873H	Fracture of lower limb
230	S82873J	Fracture of lower limb
230	S82873K	Fracture of lower limb
230	S82873M	Fracture of lower limb
230	S82873N	Fracture of lower limb
230	S82873P	Fracture of lower limb
230	S82873Q	Fracture of lower limb
230	S82873R	Fracture of lower limb
230	S82873S	Fracture of lower limb
230	S82874A	Fracture of lower limb
230	S82874B	Fracture of lower limb
230	S82874C	Fracture of lower limb
230	S82874D	Fracture of lower limb
230	S82874E	Fracture of lower limb
230	S82874F	Fracture of lower limb
230	S82874G	Fracture of lower limb
230	S82874H	Fracture of lower limb
230	S82874J	Fracture of lower limb
230	S82874K	Fracture of lower limb
230	S82874M	Fracture of lower limb
230	S82874N	Fracture of lower limb
230	S82874P	Fracture of lower limb
230	S82874Q	Fracture of lower limb
230	S82874R	Fracture of lower limb
230	S82874S	Fracture of lower limb
230	S82875A	Fracture of lower limb
230	S82875B	Fracture of lower limb
230	S82875C	Fracture of lower limb
230	S82875D	Fracture of lower limb
230	S82875E	Fracture of lower limb
230	S82875F	Fracture of lower limb
230	S82875G	Fracture of lower limb
230	S82875H	Fracture of lower limb
230	S82875J	Fracture of lower limb
230	S82875K	Fracture of lower limb
230	S82875M	Fracture of lower limb
230	S82875N	Fracture of lower limb
230	S82875P	Fracture of lower limb
230	S82875Q	Fracture of lower limb
230	S82875R	Fracture of lower limb
230	S82875S	Fracture of lower limb
230	S82876A	Fracture of lower limb

230	S82876B	Fracture of lower limb
230	S82876C	Fracture of lower limb
230	S82876D	Fracture of lower limb
230	S82876E	Fracture of lower limb
230	S82876F	Fracture of lower limb
230	S82876G	Fracture of lower limb
230	S82876H	Fracture of lower limb
230	S82876J	Fracture of lower limb
230	S82876K	Fracture of lower limb
230	S82876M	Fracture of lower limb
230	S82876N	Fracture of lower limb
230	S82876P	Fracture of lower limb
230	S82876Q	Fracture of lower limb
230	S82876R	Fracture of lower limb
230	S82876S	Fracture of lower limb
230	S82891A	Fracture of lower limb
230	S82891B	Fracture of lower limb
230	S82891C	Fracture of lower limb
230	S82891D	Fracture of lower limb
230	S82891E	Fracture of lower limb
230	S82891F	Fracture of lower limb
230	S82891G	Fracture of lower limb
230	S82891H	Fracture of lower limb
230	S82891J	Fracture of lower limb
230	S82891K	Fracture of lower limb
230	S82891M	Fracture of lower limb
230	S82891N	Fracture of lower limb
230	S82891P	Fracture of lower limb
230	S82891Q	Fracture of lower limb
230	S82891R	Fracture of lower limb
230	S82891S	Fracture of lower limb
230	S82892A	Fracture of lower limb
230	S82892B	Fracture of lower limb
230	S82892C	Fracture of lower limb
230	S82892D	Fracture of lower limb
230	S82892E	Fracture of lower limb
230	S82892F	Fracture of lower limb
230	S82892G	Fracture of lower limb
230	S82892H	Fracture of lower limb
230	S82892J	Fracture of lower limb
230	S82892K	Fracture of lower limb
230	S82892M	Fracture of lower limb
230	S82892N	Fracture of lower limb
230	S82892P	Fracture of lower limb
230	S82892Q	Fracture of lower limb
230	S82892R	Fracture of lower limb
230	S82892S	Fracture of lower limb
230	S82899A	Fracture of lower limb
230	S82899B	Fracture of lower limb
230	S82899C	Fracture of lower limb
230	S82899D	Fracture of lower limb

230	S82899E	Fracture of lower limb
230	S82899F	Fracture of lower limb
230	S82899G	Fracture of lower limb
230	S82899H	Fracture of lower limb
230	S82899J	Fracture of lower limb
230	S82899K	Fracture of lower limb
230	S82899M	Fracture of lower limb
230	S82899N	Fracture of lower limb
230	S82899P	Fracture of lower limb
230	S82899Q	Fracture of lower limb
230	S82899R	Fracture of lower limb
230	S82899S	Fracture of lower limb
230	S8290XA	Fracture of lower limb
230	S8290XB	Fracture of lower limb
230	S8290XC	Fracture of lower limb
230	S8290XD	Fracture of lower limb
230	S8290XE	Fracture of lower limb
230	S8290XF	Fracture of lower limb
230	S8290XG	Fracture of lower limb
230	S8290XH	Fracture of lower limb
230	S8290XJ	Fracture of lower limb
230	S8290XK	Fracture of lower limb
230	S8290XM	Fracture of lower limb
230	S8290XN	Fracture of lower limb
230	S8290XP	Fracture of lower limb
230	S8290XQ	Fracture of lower limb
230	S8290XR	Fracture of lower limb
230	S8290XS	Fracture of lower limb
230	S8291XA	Fracture of lower limb
230	S8291XB	Fracture of lower limb
230	S8291XC	Fracture of lower limb
230	S8291XD	Fracture of lower limb
230	S8291XE	Fracture of lower limb
230	S8291XF	Fracture of lower limb
230	S8291XG	Fracture of lower limb
230	S8291XH	Fracture of lower limb
230	S8291XJ	Fracture of lower limb
230	S8291XK	Fracture of lower limb
230	S8291XM	Fracture of lower limb
230	S8291XN	Fracture of lower limb
230	S8291XP	Fracture of lower limb
230	S8291XQ	Fracture of lower limb
230	S8291XR	Fracture of lower limb
230	S8291XS	Fracture of lower limb
230	S8292XA	Fracture of lower limb
230	S8292XB	Fracture of lower limb
230	S8292XC	Fracture of lower limb
230	S8292XD	Fracture of lower limb
230	S8292XE	Fracture of lower limb
230	S8292XF	Fracture of lower limb
230	S8292XG	Fracture of lower limb

230	S8292XH	Fracture of lower limb
230	S8292XJ	Fracture of lower limb
230	S8292XK	Fracture of lower limb
230	S8292XM	Fracture of lower limb
230	S8292XN	Fracture of lower limb
230	S8292XP	Fracture of lower limb
230	S8292XQ	Fracture of lower limb
230	S8292XR	Fracture of lower limb
230	S8292XS	Fracture of lower limb
230	S89001A	Fracture of lower limb
230	S89001D	Fracture of lower limb
230	S89001G	Fracture of lower limb
230	S89001K	Fracture of lower limb
230	S89001P	Fracture of lower limb
230	S89001S	Fracture of lower limb
230	S89002A	Fracture of lower limb
230	S89002D	Fracture of lower limb
230	S89002G	Fracture of lower limb
230	S89002K	Fracture of lower limb
230	S89002P	Fracture of lower limb
230	S89002S	Fracture of lower limb
230	S89009A	Fracture of lower limb
230	S89009D	Fracture of lower limb
230	S89009G	Fracture of lower limb
230	S89009K	Fracture of lower limb
230	S89009P	Fracture of lower limb
230	S89009S	Fracture of lower limb
230	S89011A	Fracture of lower limb
230	S89011D	Fracture of lower limb
230	S89011G	Fracture of lower limb
230	S89011K	Fracture of lower limb
230	S89011P	Fracture of lower limb
230	S89011S	Fracture of lower limb
230	S89012A	Fracture of lower limb
230	S89012D	Fracture of lower limb
230	S89012G	Fracture of lower limb
230	S89012K	Fracture of lower limb
230	S89012P	Fracture of lower limb
230	S89012S	Fracture of lower limb
230	S89019A	Fracture of lower limb
230	S89019D	Fracture of lower limb
230	S89019G	Fracture of lower limb
230	S89019K	Fracture of lower limb
230	S89019P	Fracture of lower limb
230	S89019S	Fracture of lower limb
230	S89021A	Fracture of lower limb
230	S89021D	Fracture of lower limb
230	S89021G	Fracture of lower limb
230	S89021K	Fracture of lower limb
230	S89021P	Fracture of lower limb
230	S89021S	Fracture of lower limb

230	S89022A	Fracture of lower limb
230	S89022D	Fracture of lower limb
230	S89022G	Fracture of lower limb
230	S89022K	Fracture of lower limb
230	S89022P	Fracture of lower limb
230	S89022S	Fracture of lower limb
230	S89029A	Fracture of lower limb
230	S89029D	Fracture of lower limb
230	S89029G	Fracture of lower limb
230	S89029K	Fracture of lower limb
230	S89029P	Fracture of lower limb
230	S89029S	Fracture of lower limb
230	S89031A	Fracture of lower limb
230	S89031D	Fracture of lower limb
230	S89031G	Fracture of lower limb
230	S89031K	Fracture of lower limb
230	S89031P	Fracture of lower limb
230	S89031S	Fracture of lower limb
230	S89032A	Fracture of lower limb
230	S89032D	Fracture of lower limb
230	S89032G	Fracture of lower limb
230	S89032K	Fracture of lower limb
230	S89032P	Fracture of lower limb
230	S89032S	Fracture of lower limb
230	S89039A	Fracture of lower limb
230	S89039D	Fracture of lower limb
230	S89039G	Fracture of lower limb
230	S89039K	Fracture of lower limb
230	S89039P	Fracture of lower limb
230	S89039S	Fracture of lower limb
230	S89041A	Fracture of lower limb
230	S89041D	Fracture of lower limb
230	S89041G	Fracture of lower limb
230	S89041K	Fracture of lower limb
230	S89041P	Fracture of lower limb
230	S89041S	Fracture of lower limb
230	S89042A	Fracture of lower limb
230	S89042D	Fracture of lower limb
230	S89042G	Fracture of lower limb
230	S89042K	Fracture of lower limb
230	S89042P	Fracture of lower limb
230	S89042S	Fracture of lower limb
230	S89049A	Fracture of lower limb
230	S89049D	Fracture of lower limb
230	S89049G	Fracture of lower limb
230	S89049K	Fracture of lower limb
230	S89049P	Fracture of lower limb
230	S89049S	Fracture of lower limb
230	S89091A	Fracture of lower limb
230	S89091D	Fracture of lower limb
230	S89091G	Fracture of lower limb

230	S89091K	Fracture of lower limb
230	S89091P	Fracture of lower limb
230	S89091S	Fracture of lower limb
230	S89092A	Fracture of lower limb
230	S89092D	Fracture of lower limb
230	S89092G	Fracture of lower limb
230	S89092K	Fracture of lower limb
230	S89092P	Fracture of lower limb
230	S89092S	Fracture of lower limb
230	S89099A	Fracture of lower limb
230	S89099D	Fracture of lower limb
230	S89099G	Fracture of lower limb
230	S89099K	Fracture of lower limb
230	S89099P	Fracture of lower limb
230	S89099S	Fracture of lower limb
230	S89101A	Fracture of lower limb
230	S89101D	Fracture of lower limb
230	S89101G	Fracture of lower limb
230	S89101K	Fracture of lower limb
230	S89101P	Fracture of lower limb
230	S89101S	Fracture of lower limb
230	S89102A	Fracture of lower limb
230	S89102D	Fracture of lower limb
230	S89102G	Fracture of lower limb
230	S89102K	Fracture of lower limb
230	S89102P	Fracture of lower limb
230	S89102S	Fracture of lower limb
230	S89109A	Fracture of lower limb
230	S89109D	Fracture of lower limb
230	S89109G	Fracture of lower limb
230	S89109K	Fracture of lower limb
230	S89109P	Fracture of lower limb
230	S89109S	Fracture of lower limb
230	S89111A	Fracture of lower limb
230	S89111D	Fracture of lower limb
230	S89111G	Fracture of lower limb
230	S89111K	Fracture of lower limb
230	S89111P	Fracture of lower limb
230	S89111S	Fracture of lower limb
230	S89112A	Fracture of lower limb
230	S89112D	Fracture of lower limb
230	S89112G	Fracture of lower limb
230	S89112K	Fracture of lower limb
230	S89112P	Fracture of lower limb
230	S89112S	Fracture of lower limb
230	S89119A	Fracture of lower limb
230	S89119D	Fracture of lower limb
230	S89119G	Fracture of lower limb
230	S89119K	Fracture of lower limb
230	S89119P	Fracture of lower limb
230	S89119S	Fracture of lower limb

230	S89121A	Fracture of lower limb
230	S89121D	Fracture of lower limb
230	S89121G	Fracture of lower limb
230	S89121K	Fracture of lower limb
230	S89121P	Fracture of lower limb
230	S89121S	Fracture of lower limb
230	S89122A	Fracture of lower limb
230	S89122D	Fracture of lower limb
230	S89122G	Fracture of lower limb
230	S89122K	Fracture of lower limb
230	S89122P	Fracture of lower limb
230	S89122S	Fracture of lower limb
230	S89129A	Fracture of lower limb
230	S89129D	Fracture of lower limb
230	S89129G	Fracture of lower limb
230	S89129K	Fracture of lower limb
230	S89129P	Fracture of lower limb
230	S89129S	Fracture of lower limb
230	S89131A	Fracture of lower limb
230	S89131D	Fracture of lower limb
230	S89131G	Fracture of lower limb
230	S89131K	Fracture of lower limb
230	S89131P	Fracture of lower limb
230	S89131S	Fracture of lower limb
230	S89132A	Fracture of lower limb
230	S89132D	Fracture of lower limb
230	S89132G	Fracture of lower limb
230	S89132K	Fracture of lower limb
230	S89132P	Fracture of lower limb
230	S89132S	Fracture of lower limb
230	S89139A	Fracture of lower limb
230	S89139D	Fracture of lower limb
230	S89139G	Fracture of lower limb
230	S89139K	Fracture of lower limb
230	S89139P	Fracture of lower limb
230	S89139S	Fracture of lower limb
230	S89141A	Fracture of lower limb
230	S89141D	Fracture of lower limb
230	S89141G	Fracture of lower limb
230	S89141K	Fracture of lower limb
230	S89141P	Fracture of lower limb
230	S89141S	Fracture of lower limb
230	S89142A	Fracture of lower limb
230	S89142D	Fracture of lower limb
230	S89142G	Fracture of lower limb
230	S89142K	Fracture of lower limb
230	S89142P	Fracture of lower limb
230	S89142S	Fracture of lower limb
230	S89149A	Fracture of lower limb
230	S89149D	Fracture of lower limb
230	S89149G	Fracture of lower limb

230	S89149K	Fracture of lower limb
230	S89149P	Fracture of lower limb
230	S89149S	Fracture of lower limb
230	S89191A	Fracture of lower limb
230	S89191D	Fracture of lower limb
230	S89191G	Fracture of lower limb
230	S89191K	Fracture of lower limb
230	S89191P	Fracture of lower limb
230	S89191S	Fracture of lower limb
230	S89192A	Fracture of lower limb
230	S89192D	Fracture of lower limb
230	S89192G	Fracture of lower limb
230	S89192K	Fracture of lower limb
230	S89192P	Fracture of lower limb
230	S89192S	Fracture of lower limb
230	S89199A	Fracture of lower limb
230	S89199D	Fracture of lower limb
230	S89199G	Fracture of lower limb
230	S89199K	Fracture of lower limb
230	S89199P	Fracture of lower limb
230	S89199S	Fracture of lower limb
230	S89201A	Fracture of lower limb
230	S89201D	Fracture of lower limb
230	S89201G	Fracture of lower limb
230	S89201K	Fracture of lower limb
230	S89201P	Fracture of lower limb
230	S89201S	Fracture of lower limb
230	S89202A	Fracture of lower limb
230	S89202D	Fracture of lower limb
230	S89202G	Fracture of lower limb
230	S89202K	Fracture of lower limb
230	S89202P	Fracture of lower limb
230	S89202S	Fracture of lower limb
230	S89209A	Fracture of lower limb
230	S89209D	Fracture of lower limb
230	S89209G	Fracture of lower limb
230	S89209K	Fracture of lower limb
230	S89209P	Fracture of lower limb
230	S89209S	Fracture of lower limb
230	S89211A	Fracture of lower limb
230	S89211D	Fracture of lower limb
230	S89211G	Fracture of lower limb
230	S89211K	Fracture of lower limb
230	S89211P	Fracture of lower limb
230	S89211S	Fracture of lower limb
230	S89212A	Fracture of lower limb
230	S89212D	Fracture of lower limb
230	S89212G	Fracture of lower limb
230	S89212K	Fracture of lower limb
230	S89212P	Fracture of lower limb
230	S89212S	Fracture of lower limb

230	S89219A	Fracture of lower limb
230	S89219D	Fracture of lower limb
230	S89219G	Fracture of lower limb
230	S89219K	Fracture of lower limb
230	S89219P	Fracture of lower limb
230	S89219S	Fracture of lower limb
230	S89221A	Fracture of lower limb
230	S89221D	Fracture of lower limb
230	S89221G	Fracture of lower limb
230	S89221K	Fracture of lower limb
230	S89221P	Fracture of lower limb
230	S89221S	Fracture of lower limb
230	S89222A	Fracture of lower limb
230	S89222D	Fracture of lower limb
230	S89222G	Fracture of lower limb
230	S89222K	Fracture of lower limb
230	S89222P	Fracture of lower limb
230	S89222S	Fracture of lower limb
230	S89229A	Fracture of lower limb
230	S89229D	Fracture of lower limb
230	S89229G	Fracture of lower limb
230	S89229K	Fracture of lower limb
230	S89229P	Fracture of lower limb
230	S89229S	Fracture of lower limb
230	S89291A	Fracture of lower limb
230	S89291D	Fracture of lower limb
230	S89291G	Fracture of lower limb
230	S89291K	Fracture of lower limb
230	S89291P	Fracture of lower limb
230	S89291S	Fracture of lower limb
230	S89292A	Fracture of lower limb
230	S89292D	Fracture of lower limb
230	S89292G	Fracture of lower limb
230	S89292K	Fracture of lower limb
230	S89292P	Fracture of lower limb
230	S89292S	Fracture of lower limb
230	S89299A	Fracture of lower limb
230	S89299D	Fracture of lower limb
230	S89299G	Fracture of lower limb
230	S89299K	Fracture of lower limb
230	S89299P	Fracture of lower limb
230	S89299S	Fracture of lower limb
230	S89301A	Fracture of lower limb
230	S89301D	Fracture of lower limb
230	S89301G	Fracture of lower limb
230	S89301K	Fracture of lower limb
230	S89301P	Fracture of lower limb
230	S89301S	Fracture of lower limb
230	S89302A	Fracture of lower limb
230	S89302D	Fracture of lower limb
230	S89302G	Fracture of lower limb

230	S89302K	Fracture of lower limb
230	S89302P	Fracture of lower limb
230	S89302S	Fracture of lower limb
230	S89309A	Fracture of lower limb
230	S89309D	Fracture of lower limb
230	S89309G	Fracture of lower limb
230	S89309K	Fracture of lower limb
230	S89309P	Fracture of lower limb
230	S89309S	Fracture of lower limb
230	S89311A	Fracture of lower limb
230	S89311D	Fracture of lower limb
230	S89311G	Fracture of lower limb
230	S89311K	Fracture of lower limb
230	S89311P	Fracture of lower limb
230	S89311S	Fracture of lower limb
230	S89312A	Fracture of lower limb
230	S89312D	Fracture of lower limb
230	S89312G	Fracture of lower limb
230	S89312K	Fracture of lower limb
230	S89312P	Fracture of lower limb
230	S89312S	Fracture of lower limb
230	S89319A	Fracture of lower limb
230	S89319D	Fracture of lower limb
230	S89319G	Fracture of lower limb
230	S89319K	Fracture of lower limb
230	S89319P	Fracture of lower limb
230	S89319S	Fracture of lower limb
230	S89321A	Fracture of lower limb
230	S89321D	Fracture of lower limb
230	S89321G	Fracture of lower limb
230	S89321K	Fracture of lower limb
230	S89321P	Fracture of lower limb
230	S89321S	Fracture of lower limb
230	S89322A	Fracture of lower limb
230	S89322D	Fracture of lower limb
230	S89322G	Fracture of lower limb
230	S89322K	Fracture of lower limb
230	S89322P	Fracture of lower limb
230	S89322S	Fracture of lower limb
230	S89329A	Fracture of lower limb
230	S89329D	Fracture of lower limb
230	S89329G	Fracture of lower limb
230	S89329K	Fracture of lower limb
230	S89329P	Fracture of lower limb
230	S89329S	Fracture of lower limb
230	S89391A	Fracture of lower limb
230	S89391D	Fracture of lower limb
230	S89391G	Fracture of lower limb
230	S89391K	Fracture of lower limb
230	S89391P	Fracture of lower limb
230	S89391S	Fracture of lower limb

230	S89392A	Fracture of lower limb
230	S89392D	Fracture of lower limb
230	S89392G	Fracture of lower limb
230	S89392K	Fracture of lower limb
230	S89392P	Fracture of lower limb
230	S89392S	Fracture of lower limb
230	S89399A	Fracture of lower limb
230	S89399D	Fracture of lower limb
230	S89399G	Fracture of lower limb
230	S89399K	Fracture of lower limb
230	S89399P	Fracture of lower limb
230	S89399S	Fracture of lower limb
230	S92001A	Fracture of lower limb
230	S92001B	Fracture of lower limb
230	S92001D	Fracture of lower limb
230	S92001G	Fracture of lower limb
230	S92001K	Fracture of lower limb
230	S92001P	Fracture of lower limb
230	S92001S	Fracture of lower limb
230	S92002A	Fracture of lower limb
230	S92002B	Fracture of lower limb
230	S92002D	Fracture of lower limb
230	S92002G	Fracture of lower limb
230	S92002K	Fracture of lower limb
230	S92002P	Fracture of lower limb
230	S92002S	Fracture of lower limb
230	S92009A	Fracture of lower limb
230	S92009B	Fracture of lower limb
230	S92009D	Fracture of lower limb
230	S92009G	Fracture of lower limb
230	S92009K	Fracture of lower limb
230	S92009P	Fracture of lower limb
230	S92009S	Fracture of lower limb
230	S92011A	Fracture of lower limb
230	S92011B	Fracture of lower limb
230	S92011D	Fracture of lower limb
230	S92011G	Fracture of lower limb
230	S92011K	Fracture of lower limb
230	S92011P	Fracture of lower limb
230	S92011S	Fracture of lower limb
230	S92012A	Fracture of lower limb
230	S92012B	Fracture of lower limb
230	S92012D	Fracture of lower limb
230	S92012G	Fracture of lower limb
230	S92012K	Fracture of lower limb
230	S92012P	Fracture of lower limb
230	S92012S	Fracture of lower limb
230	S92013A	Fracture of lower limb
230	S92013B	Fracture of lower limb
230	S92013D	Fracture of lower limb
230	S92013G	Fracture of lower limb

230	S92013K	Fracture of lower limb
230	S92013P	Fracture of lower limb
230	S92013S	Fracture of lower limb
230	S92014A	Fracture of lower limb
230	S92014B	Fracture of lower limb
230	S92014D	Fracture of lower limb
230	S92014G	Fracture of lower limb
230	S92014K	Fracture of lower limb
230	S92014P	Fracture of lower limb
230	S92014S	Fracture of lower limb
230	S92015A	Fracture of lower limb
230	S92015B	Fracture of lower limb
230	S92015D	Fracture of lower limb
230	S92015G	Fracture of lower limb
230	S92015K	Fracture of lower limb
230	S92015P	Fracture of lower limb
230	S92015S	Fracture of lower limb
230	S92016A	Fracture of lower limb
230	S92016B	Fracture of lower limb
230	S92016D	Fracture of lower limb
230	S92016G	Fracture of lower limb
230	S92016K	Fracture of lower limb
230	S92016P	Fracture of lower limb
230	S92016S	Fracture of lower limb
230	S92021A	Fracture of lower limb
230	S92021B	Fracture of lower limb
230	S92021D	Fracture of lower limb
230	S92021G	Fracture of lower limb
230	S92021K	Fracture of lower limb
230	S92021P	Fracture of lower limb
230	S92021S	Fracture of lower limb
230	S92022A	Fracture of lower limb
230	S92022B	Fracture of lower limb
230	S92022D	Fracture of lower limb
230	S92022G	Fracture of lower limb
230	S92022K	Fracture of lower limb
230	S92022P	Fracture of lower limb
230	S92022S	Fracture of lower limb
230	S92023A	Fracture of lower limb
230	S92023B	Fracture of lower limb
230	S92023D	Fracture of lower limb
230	S92023G	Fracture of lower limb
230	S92023K	Fracture of lower limb
230	S92023P	Fracture of lower limb
230	S92023S	Fracture of lower limb
230	S92024A	Fracture of lower limb
230	S92024B	Fracture of lower limb
230	S92024D	Fracture of lower limb
230	S92024G	Fracture of lower limb
230	S92024K	Fracture of lower limb
230	S92024P	Fracture of lower limb

230	S92024S	Fracture of lower limb
230	S92025A	Fracture of lower limb
230	S92025B	Fracture of lower limb
230	S92025D	Fracture of lower limb
230	S92025G	Fracture of lower limb
230	S92025K	Fracture of lower limb
230	S92025P	Fracture of lower limb
230	S92025S	Fracture of lower limb
230	S92026A	Fracture of lower limb
230	S92026B	Fracture of lower limb
230	S92026D	Fracture of lower limb
230	S92026G	Fracture of lower limb
230	S92026K	Fracture of lower limb
230	S92026P	Fracture of lower limb
230	S92026S	Fracture of lower limb
230	S92031A	Fracture of lower limb
230	S92031B	Fracture of lower limb
230	S92031D	Fracture of lower limb
230	S92031G	Fracture of lower limb
230	S92031K	Fracture of lower limb
230	S92031P	Fracture of lower limb
230	S92031S	Fracture of lower limb
230	S92032A	Fracture of lower limb
230	S92032B	Fracture of lower limb
230	S92032D	Fracture of lower limb
230	S92032G	Fracture of lower limb
230	S92032K	Fracture of lower limb
230	S92032P	Fracture of lower limb
230	S92032S	Fracture of lower limb
230	S92033A	Fracture of lower limb
230	S92033B	Fracture of lower limb
230	S92033D	Fracture of lower limb
230	S92033G	Fracture of lower limb
230	S92033K	Fracture of lower limb
230	S92033P	Fracture of lower limb
230	S92033S	Fracture of lower limb
230	S92034A	Fracture of lower limb
230	S92034B	Fracture of lower limb
230	S92034D	Fracture of lower limb
230	S92034G	Fracture of lower limb
230	S92034K	Fracture of lower limb
230	S92034P	Fracture of lower limb
230	S92034S	Fracture of lower limb
230	S92035A	Fracture of lower limb
230	S92035B	Fracture of lower limb
230	S92035D	Fracture of lower limb
230	S92035G	Fracture of lower limb
230	S92035K	Fracture of lower limb
230	S92035P	Fracture of lower limb
230	S92035S	Fracture of lower limb
230	S92036A	Fracture of lower limb

230	S92036B	Fracture of lower limb
230	S92036D	Fracture of lower limb
230	S92036G	Fracture of lower limb
230	S92036K	Fracture of lower limb
230	S92036P	Fracture of lower limb
230	S92036S	Fracture of lower limb
230	S92041A	Fracture of lower limb
230	S92041B	Fracture of lower limb
230	S92041D	Fracture of lower limb
230	S92041G	Fracture of lower limb
230	S92041K	Fracture of lower limb
230	S92041P	Fracture of lower limb
230	S92041S	Fracture of lower limb
230	S92042A	Fracture of lower limb
230	S92042B	Fracture of lower limb
230	S92042D	Fracture of lower limb
230	S92042G	Fracture of lower limb
230	S92042K	Fracture of lower limb
230	S92042P	Fracture of lower limb
230	S92042S	Fracture of lower limb
230	S92043A	Fracture of lower limb
230	S92043B	Fracture of lower limb
230	S92043D	Fracture of lower limb
230	S92043G	Fracture of lower limb
230	S92043K	Fracture of lower limb
230	S92043P	Fracture of lower limb
230	S92043S	Fracture of lower limb
230	S92044A	Fracture of lower limb
230	S92044B	Fracture of lower limb
230	S92044D	Fracture of lower limb
230	S92044G	Fracture of lower limb
230	S92044K	Fracture of lower limb
230	S92044P	Fracture of lower limb
230	S92044S	Fracture of lower limb
230	S92045A	Fracture of lower limb
230	S92045B	Fracture of lower limb
230	S92045D	Fracture of lower limb
230	S92045G	Fracture of lower limb
230	S92045K	Fracture of lower limb
230	S92045P	Fracture of lower limb
230	S92045S	Fracture of lower limb
230	S92046A	Fracture of lower limb
230	S92046B	Fracture of lower limb
230	S92046D	Fracture of lower limb
230	S92046G	Fracture of lower limb
230	S92046K	Fracture of lower limb
230	S92046P	Fracture of lower limb
230	S92046S	Fracture of lower limb
230	S92051A	Fracture of lower limb
230	S92051B	Fracture of lower limb
230	S92051D	Fracture of lower limb

230	S92051G	Fracture of lower limb
230	S92051K	Fracture of lower limb
230	S92051P	Fracture of lower limb
230	S92051S	Fracture of lower limb
230	S92052A	Fracture of lower limb
230	S92052B	Fracture of lower limb
230	S92052D	Fracture of lower limb
230	S92052G	Fracture of lower limb
230	S92052K	Fracture of lower limb
230	S92052P	Fracture of lower limb
230	S92052S	Fracture of lower limb
230	S92053A	Fracture of lower limb
230	S92053B	Fracture of lower limb
230	S92053D	Fracture of lower limb
230	S92053G	Fracture of lower limb
230	S92053K	Fracture of lower limb
230	S92053P	Fracture of lower limb
230	S92053S	Fracture of lower limb
230	S92054A	Fracture of lower limb
230	S92054B	Fracture of lower limb
230	S92054D	Fracture of lower limb
230	S92054G	Fracture of lower limb
230	S92054K	Fracture of lower limb
230	S92054P	Fracture of lower limb
230	S92054S	Fracture of lower limb
230	S92055A	Fracture of lower limb
230	S92055B	Fracture of lower limb
230	S92055D	Fracture of lower limb
230	S92055G	Fracture of lower limb
230	S92055K	Fracture of lower limb
230	S92055P	Fracture of lower limb
230	S92055S	Fracture of lower limb
230	S92056A	Fracture of lower limb
230	S92056B	Fracture of lower limb
230	S92056D	Fracture of lower limb
230	S92056G	Fracture of lower limb
230	S92056K	Fracture of lower limb
230	S92056P	Fracture of lower limb
230	S92056S	Fracture of lower limb
230	S92061A	Fracture of lower limb
230	S92061B	Fracture of lower limb
230	S92061D	Fracture of lower limb
230	S92061G	Fracture of lower limb
230	S92061K	Fracture of lower limb
230	S92061P	Fracture of lower limb
230	S92061S	Fracture of lower limb
230	S92062A	Fracture of lower limb
230	S92062B	Fracture of lower limb
230	S92062D	Fracture of lower limb
230	S92062G	Fracture of lower limb
230	S92062K	Fracture of lower limb

230	S92062P	Fracture of lower limb
230	S92062S	Fracture of lower limb
230	S92063A	Fracture of lower limb
230	S92063B	Fracture of lower limb
230	S92063D	Fracture of lower limb
230	S92063G	Fracture of lower limb
230	S92063K	Fracture of lower limb
230	S92063P	Fracture of lower limb
230	S92063S	Fracture of lower limb
230	S92064A	Fracture of lower limb
230	S92064B	Fracture of lower limb
230	S92064D	Fracture of lower limb
230	S92064G	Fracture of lower limb
230	S92064K	Fracture of lower limb
230	S92064P	Fracture of lower limb
230	S92064S	Fracture of lower limb
230	S92065A	Fracture of lower limb
230	S92065B	Fracture of lower limb
230	S92065D	Fracture of lower limb
230	S92065G	Fracture of lower limb
230	S92065K	Fracture of lower limb
230	S92065P	Fracture of lower limb
230	S92065S	Fracture of lower limb
230	S92066A	Fracture of lower limb
230	S92066B	Fracture of lower limb
230	S92066D	Fracture of lower limb
230	S92066G	Fracture of lower limb
230	S92066K	Fracture of lower limb
230	S92066P	Fracture of lower limb
230	S92066S	Fracture of lower limb
230	S92101A	Fracture of lower limb
230	S92101B	Fracture of lower limb
230	S92101D	Fracture of lower limb
230	S92101G	Fracture of lower limb
230	S92101K	Fracture of lower limb
230	S92101P	Fracture of lower limb
230	S92101S	Fracture of lower limb
230	S92102A	Fracture of lower limb
230	S92102B	Fracture of lower limb
230	S92102D	Fracture of lower limb
230	S92102G	Fracture of lower limb
230	S92102K	Fracture of lower limb
230	S92102P	Fracture of lower limb
230	S92102S	Fracture of lower limb
230	S92109A	Fracture of lower limb
230	S92109B	Fracture of lower limb
230	S92109D	Fracture of lower limb
230	S92109G	Fracture of lower limb
230	S92109K	Fracture of lower limb
230	S92109P	Fracture of lower limb
230	S92109S	Fracture of lower limb

230	S92111A	Fracture of lower limb
230	S92111B	Fracture of lower limb
230	S92111D	Fracture of lower limb
230	S92111G	Fracture of lower limb
230	S92111K	Fracture of lower limb
230	S92111P	Fracture of lower limb
230	S92111S	Fracture of lower limb
230	S92112A	Fracture of lower limb
230	S92112B	Fracture of lower limb
230	S92112D	Fracture of lower limb
230	S92112G	Fracture of lower limb
230	S92112K	Fracture of lower limb
230	S92112P	Fracture of lower limb
230	S92112S	Fracture of lower limb
230	S92113A	Fracture of lower limb
230	S92113B	Fracture of lower limb
230	S92113D	Fracture of lower limb
230	S92113G	Fracture of lower limb
230	S92113K	Fracture of lower limb
230	S92113P	Fracture of lower limb
230	S92113S	Fracture of lower limb
230	S92114A	Fracture of lower limb
230	S92114B	Fracture of lower limb
230	S92114D	Fracture of lower limb
230	S92114G	Fracture of lower limb
230	S92114K	Fracture of lower limb
230	S92114P	Fracture of lower limb
230	S92114S	Fracture of lower limb
230	S92115A	Fracture of lower limb
230	S92115B	Fracture of lower limb
230	S92115D	Fracture of lower limb
230	S92115G	Fracture of lower limb
230	S92115K	Fracture of lower limb
230	S92115P	Fracture of lower limb
230	S92115S	Fracture of lower limb
230	S92116A	Fracture of lower limb
230	S92116B	Fracture of lower limb
230	S92116D	Fracture of lower limb
230	S92116G	Fracture of lower limb
230	S92116K	Fracture of lower limb
230	S92116P	Fracture of lower limb
230	S92116S	Fracture of lower limb
230	S92121A	Fracture of lower limb
230	S92121B	Fracture of lower limb
230	S92121D	Fracture of lower limb
230	S92121G	Fracture of lower limb
230	S92121K	Fracture of lower limb
230	S92121P	Fracture of lower limb
230	S92121S	Fracture of lower limb
230	S92122A	Fracture of lower limb
230	S92122B	Fracture of lower limb

230	S92122D	Fracture of lower limb
230	S92122G	Fracture of lower limb
230	S92122K	Fracture of lower limb
230	S92122P	Fracture of lower limb
230	S92122S	Fracture of lower limb
230	S92123A	Fracture of lower limb
230	S92123B	Fracture of lower limb
230	S92123D	Fracture of lower limb
230	S92123G	Fracture of lower limb
230	S92123K	Fracture of lower limb
230	S92123P	Fracture of lower limb
230	S92123S	Fracture of lower limb
230	S92124A	Fracture of lower limb
230	S92124B	Fracture of lower limb
230	S92124D	Fracture of lower limb
230	S92124G	Fracture of lower limb
230	S92124K	Fracture of lower limb
230	S92124P	Fracture of lower limb
230	S92124S	Fracture of lower limb
230	S92125A	Fracture of lower limb
230	S92125B	Fracture of lower limb
230	S92125D	Fracture of lower limb
230	S92125G	Fracture of lower limb
230	S92125K	Fracture of lower limb
230	S92125P	Fracture of lower limb
230	S92125S	Fracture of lower limb
230	S92126A	Fracture of lower limb
230	S92126B	Fracture of lower limb
230	S92126D	Fracture of lower limb
230	S92126G	Fracture of lower limb
230	S92126K	Fracture of lower limb
230	S92126P	Fracture of lower limb
230	S92126S	Fracture of lower limb
230	S92131A	Fracture of lower limb
230	S92131B	Fracture of lower limb
230	S92131D	Fracture of lower limb
230	S92131G	Fracture of lower limb
230	S92131K	Fracture of lower limb
230	S92131P	Fracture of lower limb
230	S92131S	Fracture of lower limb
230	S92132A	Fracture of lower limb
230	S92132B	Fracture of lower limb
230	S92132D	Fracture of lower limb
230	S92132G	Fracture of lower limb
230	S92132K	Fracture of lower limb
230	S92132P	Fracture of lower limb
230	S92132S	Fracture of lower limb
230	S92133A	Fracture of lower limb
230	S92133B	Fracture of lower limb
230	S92133D	Fracture of lower limb
230	S92133G	Fracture of lower limb

230	S92133K	Fracture of lower limb
230	S92133P	Fracture of lower limb
230	S92133S	Fracture of lower limb
230	S92134A	Fracture of lower limb
230	S92134B	Fracture of lower limb
230	S92134D	Fracture of lower limb
230	S92134G	Fracture of lower limb
230	S92134K	Fracture of lower limb
230	S92134P	Fracture of lower limb
230	S92134S	Fracture of lower limb
230	S92135A	Fracture of lower limb
230	S92135B	Fracture of lower limb
230	S92135D	Fracture of lower limb
230	S92135G	Fracture of lower limb
230	S92135K	Fracture of lower limb
230	S92135P	Fracture of lower limb
230	S92135S	Fracture of lower limb
230	S92136A	Fracture of lower limb
230	S92136B	Fracture of lower limb
230	S92136D	Fracture of lower limb
230	S92136G	Fracture of lower limb
230	S92136K	Fracture of lower limb
230	S92136P	Fracture of lower limb
230	S92136S	Fracture of lower limb
230	S92141A	Fracture of lower limb
230	S92141B	Fracture of lower limb
230	S92141D	Fracture of lower limb
230	S92141G	Fracture of lower limb
230	S92141K	Fracture of lower limb
230	S92141P	Fracture of lower limb
230	S92141S	Fracture of lower limb
230	S92142A	Fracture of lower limb
230	S92142B	Fracture of lower limb
230	S92142D	Fracture of lower limb
230	S92142G	Fracture of lower limb
230	S92142K	Fracture of lower limb
230	S92142P	Fracture of lower limb
230	S92142S	Fracture of lower limb
230	S92143A	Fracture of lower limb
230	S92143B	Fracture of lower limb
230	S92143D	Fracture of lower limb
230	S92143G	Fracture of lower limb
230	S92143K	Fracture of lower limb
230	S92143P	Fracture of lower limb
230	S92143S	Fracture of lower limb
230	S92144A	Fracture of lower limb
230	S92144B	Fracture of lower limb
230	S92144D	Fracture of lower limb
230	S92144G	Fracture of lower limb
230	S92144K	Fracture of lower limb
230	S92144P	Fracture of lower limb

230	S92144S	Fracture of lower limb
230	S92145A	Fracture of lower limb
230	S92145B	Fracture of lower limb
230	S92145D	Fracture of lower limb
230	S92145G	Fracture of lower limb
230	S92145K	Fracture of lower limb
230	S92145P	Fracture of lower limb
230	S92145S	Fracture of lower limb
230	S92146A	Fracture of lower limb
230	S92146B	Fracture of lower limb
230	S92146D	Fracture of lower limb
230	S92146G	Fracture of lower limb
230	S92146K	Fracture of lower limb
230	S92146P	Fracture of lower limb
230	S92146S	Fracture of lower limb
230	S92151A	Fracture of lower limb
230	S92151B	Fracture of lower limb
230	S92151D	Fracture of lower limb
230	S92151G	Fracture of lower limb
230	S92151K	Fracture of lower limb
230	S92151P	Fracture of lower limb
230	S92151S	Fracture of lower limb
230	S92152A	Fracture of lower limb
230	S92152B	Fracture of lower limb
230	S92152D	Fracture of lower limb
230	S92152G	Fracture of lower limb
230	S92152K	Fracture of lower limb
230	S92152P	Fracture of lower limb
230	S92152S	Fracture of lower limb
230	S92153A	Fracture of lower limb
230	S92153B	Fracture of lower limb
230	S92153D	Fracture of lower limb
230	S92153G	Fracture of lower limb
230	S92153K	Fracture of lower limb
230	S92153P	Fracture of lower limb
230	S92153S	Fracture of lower limb
230	S92154A	Fracture of lower limb
230	S92154B	Fracture of lower limb
230	S92154D	Fracture of lower limb
230	S92154G	Fracture of lower limb
230	S92154K	Fracture of lower limb
230	S92154P	Fracture of lower limb
230	S92154S	Fracture of lower limb
230	S92155A	Fracture of lower limb
230	S92155B	Fracture of lower limb
230	S92155D	Fracture of lower limb
230	S92155G	Fracture of lower limb
230	S92155K	Fracture of lower limb
230	S92155P	Fracture of lower limb
230	S92155S	Fracture of lower limb
230	S92156A	Fracture of lower limb

230	S92156B	Fracture of lower limb
230	S92156D	Fracture of lower limb
230	S92156G	Fracture of lower limb
230	S92156K	Fracture of lower limb
230	S92156P	Fracture of lower limb
230	S92156S	Fracture of lower limb
230	S92191A	Fracture of lower limb
230	S92191B	Fracture of lower limb
230	S92191D	Fracture of lower limb
230	S92191G	Fracture of lower limb
230	S92191K	Fracture of lower limb
230	S92191P	Fracture of lower limb
230	S92191S	Fracture of lower limb
230	S92192A	Fracture of lower limb
230	S92192B	Fracture of lower limb
230	S92192D	Fracture of lower limb
230	S92192G	Fracture of lower limb
230	S92192K	Fracture of lower limb
230	S92192P	Fracture of lower limb
230	S92192S	Fracture of lower limb
230	S92199A	Fracture of lower limb
230	S92199B	Fracture of lower limb
230	S92199D	Fracture of lower limb
230	S92199G	Fracture of lower limb
230	S92199K	Fracture of lower limb
230	S92199P	Fracture of lower limb
230	S92199S	Fracture of lower limb
230	S92201A	Fracture of lower limb
230	S92201B	Fracture of lower limb
230	S92201D	Fracture of lower limb
230	S92201G	Fracture of lower limb
230	S92201K	Fracture of lower limb
230	S92201P	Fracture of lower limb
230	S92201S	Fracture of lower limb
230	S92202A	Fracture of lower limb
230	S92202B	Fracture of lower limb
230	S92202D	Fracture of lower limb
230	S92202G	Fracture of lower limb
230	S92202K	Fracture of lower limb
230	S92202P	Fracture of lower limb
230	S92202S	Fracture of lower limb
230	S92209A	Fracture of lower limb
230	S92209B	Fracture of lower limb
230	S92209D	Fracture of lower limb
230	S92209G	Fracture of lower limb
230	S92209K	Fracture of lower limb
230	S92209P	Fracture of lower limb
230	S92209S	Fracture of lower limb
230	S92211A	Fracture of lower limb
230	S92211B	Fracture of lower limb
230	S92211D	Fracture of lower limb

230	S92211G	Fracture of lower limb
230	S92211K	Fracture of lower limb
230	S92211P	Fracture of lower limb
230	S92211S	Fracture of lower limb
230	S92212A	Fracture of lower limb
230	S92212B	Fracture of lower limb
230	S92212D	Fracture of lower limb
230	S92212G	Fracture of lower limb
230	S92212K	Fracture of lower limb
230	S92212P	Fracture of lower limb
230	S92212S	Fracture of lower limb
230	S92213A	Fracture of lower limb
230	S92213B	Fracture of lower limb
230	S92213D	Fracture of lower limb
230	S92213G	Fracture of lower limb
230	S92213K	Fracture of lower limb
230	S92213P	Fracture of lower limb
230	S92213S	Fracture of lower limb
230	S92214A	Fracture of lower limb
230	S92214B	Fracture of lower limb
230	S92214D	Fracture of lower limb
230	S92214G	Fracture of lower limb
230	S92214K	Fracture of lower limb
230	S92214P	Fracture of lower limb
230	S92214S	Fracture of lower limb
230	S92215A	Fracture of lower limb
230	S92215B	Fracture of lower limb
230	S92215D	Fracture of lower limb
230	S92215G	Fracture of lower limb
230	S92215K	Fracture of lower limb
230	S92215P	Fracture of lower limb
230	S92215S	Fracture of lower limb
230	S92216A	Fracture of lower limb
230	S92216B	Fracture of lower limb
230	S92216D	Fracture of lower limb
230	S92216G	Fracture of lower limb
230	S92216K	Fracture of lower limb
230	S92216P	Fracture of lower limb
230	S92216S	Fracture of lower limb
230	S92221A	Fracture of lower limb
230	S92221B	Fracture of lower limb
230	S92221D	Fracture of lower limb
230	S92221G	Fracture of lower limb
230	S92221K	Fracture of lower limb
230	S92221P	Fracture of lower limb
230	S92221S	Fracture of lower limb
230	S92222A	Fracture of lower limb
230	S92222B	Fracture of lower limb
230	S92222D	Fracture of lower limb
230	S92222G	Fracture of lower limb
230	S92222K	Fracture of lower limb

230	S92222P	Fracture of lower limb
230	S92222S	Fracture of lower limb
230	S92223A	Fracture of lower limb
230	S92223B	Fracture of lower limb
230	S92223D	Fracture of lower limb
230	S92223G	Fracture of lower limb
230	S92223K	Fracture of lower limb
230	S92223P	Fracture of lower limb
230	S92223S	Fracture of lower limb
230	S92224A	Fracture of lower limb
230	S92224B	Fracture of lower limb
230	S92224D	Fracture of lower limb
230	S92224G	Fracture of lower limb
230	S92224K	Fracture of lower limb
230	S92224P	Fracture of lower limb
230	S92224S	Fracture of lower limb
230	S92225A	Fracture of lower limb
230	S92225B	Fracture of lower limb
230	S92225D	Fracture of lower limb
230	S92225G	Fracture of lower limb
230	S92225K	Fracture of lower limb
230	S92225P	Fracture of lower limb
230	S92225S	Fracture of lower limb
230	S92226A	Fracture of lower limb
230	S92226B	Fracture of lower limb
230	S92226D	Fracture of lower limb
230	S92226G	Fracture of lower limb
230	S92226K	Fracture of lower limb
230	S92226P	Fracture of lower limb
230	S92226S	Fracture of lower limb
230	S92231A	Fracture of lower limb
230	S92231B	Fracture of lower limb
230	S92231D	Fracture of lower limb
230	S92231G	Fracture of lower limb
230	S92231K	Fracture of lower limb
230	S92231P	Fracture of lower limb
230	S92231S	Fracture of lower limb
230	S92232A	Fracture of lower limb
230	S92232B	Fracture of lower limb
230	S92232D	Fracture of lower limb
230	S92232G	Fracture of lower limb
230	S92232K	Fracture of lower limb
230	S92232P	Fracture of lower limb
230	S92232S	Fracture of lower limb
230	S92233A	Fracture of lower limb
230	S92233B	Fracture of lower limb
230	S92233D	Fracture of lower limb
230	S92233G	Fracture of lower limb
230	S92233K	Fracture of lower limb
230	S92233P	Fracture of lower limb
230	S92233S	Fracture of lower limb

230	S92234A	Fracture of lower limb
230	S92234B	Fracture of lower limb
230	S92234D	Fracture of lower limb
230	S92234G	Fracture of lower limb
230	S92234K	Fracture of lower limb
230	S92234P	Fracture of lower limb
230	S92234S	Fracture of lower limb
230	S92235A	Fracture of lower limb
230	S92235B	Fracture of lower limb
230	S92235D	Fracture of lower limb
230	S92235G	Fracture of lower limb
230	S92235K	Fracture of lower limb
230	S92235P	Fracture of lower limb
230	S92235S	Fracture of lower limb
230	S92236A	Fracture of lower limb
230	S92236B	Fracture of lower limb
230	S92236D	Fracture of lower limb
230	S92236G	Fracture of lower limb
230	S92236K	Fracture of lower limb
230	S92236P	Fracture of lower limb
230	S92236S	Fracture of lower limb
230	S92241A	Fracture of lower limb
230	S92241B	Fracture of lower limb
230	S92241D	Fracture of lower limb
230	S92241G	Fracture of lower limb
230	S92241K	Fracture of lower limb
230	S92241P	Fracture of lower limb
230	S92241S	Fracture of lower limb
230	S92242A	Fracture of lower limb
230	S92242B	Fracture of lower limb
230	S92242D	Fracture of lower limb
230	S92242G	Fracture of lower limb
230	S92242K	Fracture of lower limb
230	S92242P	Fracture of lower limb
230	S92242S	Fracture of lower limb
230	S92243A	Fracture of lower limb
230	S92243B	Fracture of lower limb
230	S92243D	Fracture of lower limb
230	S92243G	Fracture of lower limb
230	S92243K	Fracture of lower limb
230	S92243P	Fracture of lower limb
230	S92243S	Fracture of lower limb
230	S92244A	Fracture of lower limb
230	S92244B	Fracture of lower limb
230	S92244D	Fracture of lower limb
230	S92244G	Fracture of lower limb
230	S92244K	Fracture of lower limb
230	S92244P	Fracture of lower limb
230	S92244S	Fracture of lower limb
230	S92245A	Fracture of lower limb
230	S92245B	Fracture of lower limb

230	S92245D	Fracture of lower limb
230	S92245G	Fracture of lower limb
230	S92245K	Fracture of lower limb
230	S92245P	Fracture of lower limb
230	S92245S	Fracture of lower limb
230	S92246A	Fracture of lower limb
230	S92246B	Fracture of lower limb
230	S92246D	Fracture of lower limb
230	S92246G	Fracture of lower limb
230	S92246K	Fracture of lower limb
230	S92246P	Fracture of lower limb
230	S92246S	Fracture of lower limb
230	S92251A	Fracture of lower limb
230	S92251B	Fracture of lower limb
230	S92251D	Fracture of lower limb
230	S92251G	Fracture of lower limb
230	S92251K	Fracture of lower limb
230	S92251P	Fracture of lower limb
230	S92251S	Fracture of lower limb
230	S92252A	Fracture of lower limb
230	S92252B	Fracture of lower limb
230	S92252D	Fracture of lower limb
230	S92252G	Fracture of lower limb
230	S92252K	Fracture of lower limb
230	S92252P	Fracture of lower limb
230	S92252S	Fracture of lower limb
230	S92253A	Fracture of lower limb
230	S92253B	Fracture of lower limb
230	S92253D	Fracture of lower limb
230	S92253G	Fracture of lower limb
230	S92253K	Fracture of lower limb
230	S92253P	Fracture of lower limb
230	S92253S	Fracture of lower limb
230	S92254A	Fracture of lower limb
230	S92254B	Fracture of lower limb
230	S92254D	Fracture of lower limb
230	S92254G	Fracture of lower limb
230	S92254K	Fracture of lower limb
230	S92254P	Fracture of lower limb
230	S92254S	Fracture of lower limb
230	S92255A	Fracture of lower limb
230	S92255B	Fracture of lower limb
230	S92255D	Fracture of lower limb
230	S92255G	Fracture of lower limb
230	S92255K	Fracture of lower limb
230	S92255P	Fracture of lower limb
230	S92255S	Fracture of lower limb
230	S92256A	Fracture of lower limb
230	S92256B	Fracture of lower limb
230	S92256D	Fracture of lower limb
230	S92256G	Fracture of lower limb

230	S92256K	Fracture of lower limb
230	S92256P	Fracture of lower limb
230	S92256S	Fracture of lower limb
230	S92301A	Fracture of lower limb
230	S92301B	Fracture of lower limb
230	S92301D	Fracture of lower limb
230	S92301G	Fracture of lower limb
230	S92301K	Fracture of lower limb
230	S92301P	Fracture of lower limb
230	S92301S	Fracture of lower limb
230	S92302A	Fracture of lower limb
230	S92302B	Fracture of lower limb
230	S92302D	Fracture of lower limb
230	S92302G	Fracture of lower limb
230	S92302K	Fracture of lower limb
230	S92302P	Fracture of lower limb
230	S92302S	Fracture of lower limb
230	S92309A	Fracture of lower limb
230	S92309B	Fracture of lower limb
230	S92309D	Fracture of lower limb
230	S92309G	Fracture of lower limb
230	S92309K	Fracture of lower limb
230	S92309P	Fracture of lower limb
230	S92309S	Fracture of lower limb
230	S92311A	Fracture of lower limb
230	S92311B	Fracture of lower limb
230	S92311D	Fracture of lower limb
230	S92311G	Fracture of lower limb
230	S92311K	Fracture of lower limb
230	S92311P	Fracture of lower limb
230	S92311S	Fracture of lower limb
230	S92312A	Fracture of lower limb
230	S92312B	Fracture of lower limb
230	S92312D	Fracture of lower limb
230	S92312G	Fracture of lower limb
230	S92312K	Fracture of lower limb
230	S92312P	Fracture of lower limb
230	S92312S	Fracture of lower limb
230	S92313A	Fracture of lower limb
230	S92313B	Fracture of lower limb
230	S92313D	Fracture of lower limb
230	S92313G	Fracture of lower limb
230	S92313K	Fracture of lower limb
230	S92313P	Fracture of lower limb
230	S92313S	Fracture of lower limb
230	S92314A	Fracture of lower limb
230	S92314B	Fracture of lower limb
230	S92314D	Fracture of lower limb
230	S92314G	Fracture of lower limb
230	S92314K	Fracture of lower limb
230	S92314P	Fracture of lower limb

230	S92314S	Fracture of lower limb
230	S92315A	Fracture of lower limb
230	S92315B	Fracture of lower limb
230	S92315D	Fracture of lower limb
230	S92315G	Fracture of lower limb
230	S92315K	Fracture of lower limb
230	S92315P	Fracture of lower limb
230	S92315S	Fracture of lower limb
230	S92316A	Fracture of lower limb
230	S92316B	Fracture of lower limb
230	S92316D	Fracture of lower limb
230	S92316G	Fracture of lower limb
230	S92316K	Fracture of lower limb
230	S92316P	Fracture of lower limb
230	S92316S	Fracture of lower limb
230	S92321A	Fracture of lower limb
230	S92321B	Fracture of lower limb
230	S92321D	Fracture of lower limb
230	S92321G	Fracture of lower limb
230	S92321K	Fracture of lower limb
230	S92321P	Fracture of lower limb
230	S92321S	Fracture of lower limb
230	S92322A	Fracture of lower limb
230	S92322B	Fracture of lower limb
230	S92322D	Fracture of lower limb
230	S92322G	Fracture of lower limb
230	S92322K	Fracture of lower limb
230	S92322P	Fracture of lower limb
230	S92322S	Fracture of lower limb
230	S92323A	Fracture of lower limb
230	S92323B	Fracture of lower limb
230	S92323D	Fracture of lower limb
230	S92323G	Fracture of lower limb
230	S92323K	Fracture of lower limb
230	S92323P	Fracture of lower limb
230	S92323S	Fracture of lower limb
230	S92324A	Fracture of lower limb
230	S92324B	Fracture of lower limb
230	S92324D	Fracture of lower limb
230	S92324G	Fracture of lower limb
230	S92324K	Fracture of lower limb
230	S92324P	Fracture of lower limb
230	S92324S	Fracture of lower limb
230	S92325A	Fracture of lower limb
230	S92325B	Fracture of lower limb
230	S92325D	Fracture of lower limb
230	S92325G	Fracture of lower limb
230	S92325K	Fracture of lower limb
230	S92325P	Fracture of lower limb
230	S92325S	Fracture of lower limb
230	S92326A	Fracture of lower limb

230	S92326B	Fracture of lower limb
230	S92326D	Fracture of lower limb
230	S92326G	Fracture of lower limb
230	S92326K	Fracture of lower limb
230	S92326P	Fracture of lower limb
230	S92326S	Fracture of lower limb
230	S92331A	Fracture of lower limb
230	S92331B	Fracture of lower limb
230	S92331D	Fracture of lower limb
230	S92331G	Fracture of lower limb
230	S92331K	Fracture of lower limb
230	S92331P	Fracture of lower limb
230	S92331S	Fracture of lower limb
230	S92332A	Fracture of lower limb
230	S92332B	Fracture of lower limb
230	S92332D	Fracture of lower limb
230	S92332G	Fracture of lower limb
230	S92332K	Fracture of lower limb
230	S92332P	Fracture of lower limb
230	S92332S	Fracture of lower limb
230	S92333A	Fracture of lower limb
230	S92333B	Fracture of lower limb
230	S92333D	Fracture of lower limb
230	S92333G	Fracture of lower limb
230	S92333K	Fracture of lower limb
230	S92333P	Fracture of lower limb
230	S92333S	Fracture of lower limb
230	S92334A	Fracture of lower limb
230	S92334B	Fracture of lower limb
230	S92334D	Fracture of lower limb
230	S92334G	Fracture of lower limb
230	S92334K	Fracture of lower limb
230	S92334P	Fracture of lower limb
230	S92334S	Fracture of lower limb
230	S92335A	Fracture of lower limb
230	S92335B	Fracture of lower limb
230	S92335D	Fracture of lower limb
230	S92335G	Fracture of lower limb
230	S92335K	Fracture of lower limb
230	S92335P	Fracture of lower limb
230	S92335S	Fracture of lower limb
230	S92336A	Fracture of lower limb
230	S92336B	Fracture of lower limb
230	S92336D	Fracture of lower limb
230	S92336G	Fracture of lower limb
230	S92336K	Fracture of lower limb
230	S92336P	Fracture of lower limb
230	S92336S	Fracture of lower limb
230	S92341A	Fracture of lower limb
230	S92341B	Fracture of lower limb
230	S92341D	Fracture of lower limb

230	S92341G	Fracture of lower limb
230	S92341K	Fracture of lower limb
230	S92341P	Fracture of lower limb
230	S92341S	Fracture of lower limb
230	S92342A	Fracture of lower limb
230	S92342B	Fracture of lower limb
230	S92342D	Fracture of lower limb
230	S92342G	Fracture of lower limb
230	S92342K	Fracture of lower limb
230	S92342P	Fracture of lower limb
230	S92342S	Fracture of lower limb
230	S92343A	Fracture of lower limb
230	S92343B	Fracture of lower limb
230	S92343D	Fracture of lower limb
230	S92343G	Fracture of lower limb
230	S92343K	Fracture of lower limb
230	S92343P	Fracture of lower limb
230	S92343S	Fracture of lower limb
230	S92344A	Fracture of lower limb
230	S92344B	Fracture of lower limb
230	S92344D	Fracture of lower limb
230	S92344G	Fracture of lower limb
230	S92344K	Fracture of lower limb
230	S92344P	Fracture of lower limb
230	S92344S	Fracture of lower limb
230	S92345A	Fracture of lower limb
230	S92345B	Fracture of lower limb
230	S92345D	Fracture of lower limb
230	S92345G	Fracture of lower limb
230	S92345K	Fracture of lower limb
230	S92345P	Fracture of lower limb
230	S92345S	Fracture of lower limb
230	S92346A	Fracture of lower limb
230	S92346B	Fracture of lower limb
230	S92346D	Fracture of lower limb
230	S92346G	Fracture of lower limb
230	S92346K	Fracture of lower limb
230	S92346P	Fracture of lower limb
230	S92346S	Fracture of lower limb
230	S92351A	Fracture of lower limb
230	S92351B	Fracture of lower limb
230	S92351D	Fracture of lower limb
230	S92351G	Fracture of lower limb
230	S92351K	Fracture of lower limb
230	S92351P	Fracture of lower limb
230	S92351S	Fracture of lower limb
230	S92352A	Fracture of lower limb
230	S92352B	Fracture of lower limb
230	S92352D	Fracture of lower limb
230	S92352G	Fracture of lower limb
230	S92352K	Fracture of lower limb

230	S92352P	Fracture of lower limb
230	S92352S	Fracture of lower limb
230	S92353A	Fracture of lower limb
230	S92353B	Fracture of lower limb
230	S92353D	Fracture of lower limb
230	S92353G	Fracture of lower limb
230	S92353K	Fracture of lower limb
230	S92353P	Fracture of lower limb
230	S92353S	Fracture of lower limb
230	S92354A	Fracture of lower limb
230	S92354B	Fracture of lower limb
230	S92354D	Fracture of lower limb
230	S92354G	Fracture of lower limb
230	S92354K	Fracture of lower limb
230	S92354P	Fracture of lower limb
230	S92354S	Fracture of lower limb
230	S92355A	Fracture of lower limb
230	S92355B	Fracture of lower limb
230	S92355D	Fracture of lower limb
230	S92355G	Fracture of lower limb
230	S92355K	Fracture of lower limb
230	S92355P	Fracture of lower limb
230	S92355S	Fracture of lower limb
230	S92356A	Fracture of lower limb
230	S92356B	Fracture of lower limb
230	S92356D	Fracture of lower limb
230	S92356G	Fracture of lower limb
230	S92356K	Fracture of lower limb
230	S92356P	Fracture of lower limb
230	S92356S	Fracture of lower limb
230	S92401A	Fracture of lower limb
230	S92401B	Fracture of lower limb
230	S92401D	Fracture of lower limb
230	S92401G	Fracture of lower limb
230	S92401K	Fracture of lower limb
230	S92401P	Fracture of lower limb
230	S92401S	Fracture of lower limb
230	S92402A	Fracture of lower limb
230	S92402B	Fracture of lower limb
230	S92402D	Fracture of lower limb
230	S92402G	Fracture of lower limb
230	S92402K	Fracture of lower limb
230	S92402P	Fracture of lower limb
230	S92402S	Fracture of lower limb
230	S92403A	Fracture of lower limb
230	S92403B	Fracture of lower limb
230	S92403D	Fracture of lower limb
230	S92403G	Fracture of lower limb
230	S92403K	Fracture of lower limb
230	S92403P	Fracture of lower limb
230	S92403S	Fracture of lower limb

230	S92404A	Fracture of lower limb
230	S92404B	Fracture of lower limb
230	S92404D	Fracture of lower limb
230	S92404G	Fracture of lower limb
230	S92404K	Fracture of lower limb
230	S92404P	Fracture of lower limb
230	S92404S	Fracture of lower limb
230	S92405A	Fracture of lower limb
230	S92405B	Fracture of lower limb
230	S92405D	Fracture of lower limb
230	S92405G	Fracture of lower limb
230	S92405K	Fracture of lower limb
230	S92405P	Fracture of lower limb
230	S92405S	Fracture of lower limb
230	S92406A	Fracture of lower limb
230	S92406B	Fracture of lower limb
230	S92406D	Fracture of lower limb
230	S92406G	Fracture of lower limb
230	S92406K	Fracture of lower limb
230	S92406P	Fracture of lower limb
230	S92406S	Fracture of lower limb
230	S92411A	Fracture of lower limb
230	S92411B	Fracture of lower limb
230	S92411D	Fracture of lower limb
230	S92411G	Fracture of lower limb
230	S92411K	Fracture of lower limb
230	S92411P	Fracture of lower limb
230	S92411S	Fracture of lower limb
230	S92412A	Fracture of lower limb
230	S92412B	Fracture of lower limb
230	S92412D	Fracture of lower limb
230	S92412G	Fracture of lower limb
230	S92412K	Fracture of lower limb
230	S92412P	Fracture of lower limb
230	S92412S	Fracture of lower limb
230	S92413A	Fracture of lower limb
230	S92413B	Fracture of lower limb
230	S92413D	Fracture of lower limb
230	S92413G	Fracture of lower limb
230	S92413K	Fracture of lower limb
230	S92413P	Fracture of lower limb
230	S92413S	Fracture of lower limb
230	S92414A	Fracture of lower limb
230	S92414B	Fracture of lower limb
230	S92414D	Fracture of lower limb
230	S92414G	Fracture of lower limb
230	S92414K	Fracture of lower limb
230	S92414P	Fracture of lower limb
230	S92414S	Fracture of lower limb
230	S92415A	Fracture of lower limb
230	S92415B	Fracture of lower limb

230	S92415D	Fracture of lower limb
230	S92415G	Fracture of lower limb
230	S92415K	Fracture of lower limb
230	S92415P	Fracture of lower limb
230	S92415S	Fracture of lower limb
230	S92416A	Fracture of lower limb
230	S92416B	Fracture of lower limb
230	S92416D	Fracture of lower limb
230	S92416G	Fracture of lower limb
230	S92416K	Fracture of lower limb
230	S92416P	Fracture of lower limb
230	S92416S	Fracture of lower limb
230	S92421A	Fracture of lower limb
230	S92421B	Fracture of lower limb
230	S92421D	Fracture of lower limb
230	S92421G	Fracture of lower limb
230	S92421K	Fracture of lower limb
230	S92421P	Fracture of lower limb
230	S92421S	Fracture of lower limb
230	S92422A	Fracture of lower limb
230	S92422B	Fracture of lower limb
230	S92422D	Fracture of lower limb
230	S92422G	Fracture of lower limb
230	S92422K	Fracture of lower limb
230	S92422P	Fracture of lower limb
230	S92422S	Fracture of lower limb
230	S92423A	Fracture of lower limb
230	S92423B	Fracture of lower limb
230	S92423D	Fracture of lower limb
230	S92423G	Fracture of lower limb
230	S92423K	Fracture of lower limb
230	S92423P	Fracture of lower limb
230	S92423S	Fracture of lower limb
230	S92424A	Fracture of lower limb
230	S92424B	Fracture of lower limb
230	S92424D	Fracture of lower limb
230	S92424G	Fracture of lower limb
230	S92424K	Fracture of lower limb
230	S92424P	Fracture of lower limb
230	S92424S	Fracture of lower limb
230	S92425A	Fracture of lower limb
230	S92425B	Fracture of lower limb
230	S92425D	Fracture of lower limb
230	S92425G	Fracture of lower limb
230	S92425K	Fracture of lower limb
230	S92425P	Fracture of lower limb
230	S92425S	Fracture of lower limb
230	S92426A	Fracture of lower limb
230	S92426B	Fracture of lower limb
230	S92426D	Fracture of lower limb
230	S92426G	Fracture of lower limb

230	S92426K	Fracture of lower limb
230	S92426P	Fracture of lower limb
230	S92426S	Fracture of lower limb
230	S92491A	Fracture of lower limb
230	S92491B	Fracture of lower limb
230	S92491D	Fracture of lower limb
230	S92491G	Fracture of lower limb
230	S92491K	Fracture of lower limb
230	S92491P	Fracture of lower limb
230	S92491S	Fracture of lower limb
230	S92492A	Fracture of lower limb
230	S92492B	Fracture of lower limb
230	S92492D	Fracture of lower limb
230	S92492G	Fracture of lower limb
230	S92492K	Fracture of lower limb
230	S92492P	Fracture of lower limb
230	S92492S	Fracture of lower limb
230	S92499A	Fracture of lower limb
230	S92499B	Fracture of lower limb
230	S92499D	Fracture of lower limb
230	S92499G	Fracture of lower limb
230	S92499K	Fracture of lower limb
230	S92499P	Fracture of lower limb
230	S92499S	Fracture of lower limb
230	S92501A	Fracture of lower limb
230	S92501B	Fracture of lower limb
230	S92501D	Fracture of lower limb
230	S92501G	Fracture of lower limb
230	S92501K	Fracture of lower limb
230	S92501P	Fracture of lower limb
230	S92501S	Fracture of lower limb
230	S92502A	Fracture of lower limb
230	S92502B	Fracture of lower limb
230	S92502D	Fracture of lower limb
230	S92502G	Fracture of lower limb
230	S92502K	Fracture of lower limb
230	S92502P	Fracture of lower limb
230	S92502S	Fracture of lower limb
230	S92503A	Fracture of lower limb
230	S92503B	Fracture of lower limb
230	S92503D	Fracture of lower limb
230	S92503G	Fracture of lower limb
230	S92503K	Fracture of lower limb
230	S92503P	Fracture of lower limb
230	S92503S	Fracture of lower limb
230	S92504A	Fracture of lower limb
230	S92504B	Fracture of lower limb
230	S92504D	Fracture of lower limb
230	S92504G	Fracture of lower limb
230	S92504K	Fracture of lower limb
230	S92504P	Fracture of lower limb

230	S92504S	Fracture of lower limb
230	S92505A	Fracture of lower limb
230	S92505B	Fracture of lower limb
230	S92505D	Fracture of lower limb
230	S92505G	Fracture of lower limb
230	S92505K	Fracture of lower limb
230	S92505P	Fracture of lower limb
230	S92505S	Fracture of lower limb
230	S92506A	Fracture of lower limb
230	S92506B	Fracture of lower limb
230	S92506D	Fracture of lower limb
230	S92506G	Fracture of lower limb
230	S92506K	Fracture of lower limb
230	S92506P	Fracture of lower limb
230	S92506S	Fracture of lower limb
230	S92511A	Fracture of lower limb
230	S92511B	Fracture of lower limb
230	S92511D	Fracture of lower limb
230	S92511G	Fracture of lower limb
230	S92511K	Fracture of lower limb
230	S92511P	Fracture of lower limb
230	S92511S	Fracture of lower limb
230	S92512A	Fracture of lower limb
230	S92512B	Fracture of lower limb
230	S92512D	Fracture of lower limb
230	S92512G	Fracture of lower limb
230	S92512K	Fracture of lower limb
230	S92512P	Fracture of lower limb
230	S92512S	Fracture of lower limb
230	S92513A	Fracture of lower limb
230	S92513B	Fracture of lower limb
230	S92513D	Fracture of lower limb
230	S92513G	Fracture of lower limb
230	S92513K	Fracture of lower limb
230	S92513P	Fracture of lower limb
230	S92513S	Fracture of lower limb
230	S92514A	Fracture of lower limb
230	S92514B	Fracture of lower limb
230	S92514D	Fracture of lower limb
230	S92514G	Fracture of lower limb
230	S92514K	Fracture of lower limb
230	S92514P	Fracture of lower limb
230	S92514S	Fracture of lower limb
230	S92515A	Fracture of lower limb
230	S92515B	Fracture of lower limb
230	S92515D	Fracture of lower limb
230	S92515G	Fracture of lower limb
230	S92515K	Fracture of lower limb
230	S92515P	Fracture of lower limb
230	S92515S	Fracture of lower limb
230	S92516A	Fracture of lower limb

230	S92516B	Fracture of lower limb
230	S92516D	Fracture of lower limb
230	S92516G	Fracture of lower limb
230	S92516K	Fracture of lower limb
230	S92516P	Fracture of lower limb
230	S92516S	Fracture of lower limb
230	S92521A	Fracture of lower limb
230	S92521B	Fracture of lower limb
230	S92521D	Fracture of lower limb
230	S92521G	Fracture of lower limb
230	S92521K	Fracture of lower limb
230	S92521P	Fracture of lower limb
230	S92521S	Fracture of lower limb
230	S92522A	Fracture of lower limb
230	S92522B	Fracture of lower limb
230	S92522D	Fracture of lower limb
230	S92522G	Fracture of lower limb
230	S92522K	Fracture of lower limb
230	S92522P	Fracture of lower limb
230	S92522S	Fracture of lower limb
230	S92523A	Fracture of lower limb
230	S92523B	Fracture of lower limb
230	S92523D	Fracture of lower limb
230	S92523G	Fracture of lower limb
230	S92523K	Fracture of lower limb
230	S92523P	Fracture of lower limb
230	S92523S	Fracture of lower limb
230	S92524A	Fracture of lower limb
230	S92524B	Fracture of lower limb
230	S92524D	Fracture of lower limb
230	S92524G	Fracture of lower limb
230	S92524K	Fracture of lower limb
230	S92524P	Fracture of lower limb
230	S92524S	Fracture of lower limb
230	S92525A	Fracture of lower limb
230	S92525B	Fracture of lower limb
230	S92525D	Fracture of lower limb
230	S92525G	Fracture of lower limb
230	S92525K	Fracture of lower limb
230	S92525P	Fracture of lower limb
230	S92525S	Fracture of lower limb
230	S92526A	Fracture of lower limb
230	S92526B	Fracture of lower limb
230	S92526D	Fracture of lower limb
230	S92526G	Fracture of lower limb
230	S92526K	Fracture of lower limb
230	S92526P	Fracture of lower limb
230	S92526S	Fracture of lower limb
230	S92531A	Fracture of lower limb
230	S92531B	Fracture of lower limb
230	S92531D	Fracture of lower limb

230	S92531G	Fracture of lower limb
230	S92531K	Fracture of lower limb
230	S92531P	Fracture of lower limb
230	S92531S	Fracture of lower limb
230	S92532A	Fracture of lower limb
230	S92532B	Fracture of lower limb
230	S92532D	Fracture of lower limb
230	S92532G	Fracture of lower limb
230	S92532K	Fracture of lower limb
230	S92532P	Fracture of lower limb
230	S92532S	Fracture of lower limb
230	S92533A	Fracture of lower limb
230	S92533B	Fracture of lower limb
230	S92533D	Fracture of lower limb
230	S92533G	Fracture of lower limb
230	S92533K	Fracture of lower limb
230	S92533P	Fracture of lower limb
230	S92533S	Fracture of lower limb
230	S92534A	Fracture of lower limb
230	S92534B	Fracture of lower limb
230	S92534D	Fracture of lower limb
230	S92534G	Fracture of lower limb
230	S92534K	Fracture of lower limb
230	S92534P	Fracture of lower limb
230	S92534S	Fracture of lower limb
230	S92535A	Fracture of lower limb
230	S92535B	Fracture of lower limb
230	S92535D	Fracture of lower limb
230	S92535G	Fracture of lower limb
230	S92535K	Fracture of lower limb
230	S92535P	Fracture of lower limb
230	S92535S	Fracture of lower limb
230	S92536A	Fracture of lower limb
230	S92536B	Fracture of lower limb
230	S92536D	Fracture of lower limb
230	S92536G	Fracture of lower limb
230	S92536K	Fracture of lower limb
230	S92536P	Fracture of lower limb
230	S92536S	Fracture of lower limb
230	S92591A	Fracture of lower limb
230	S92591B	Fracture of lower limb
230	S92591D	Fracture of lower limb
230	S92591G	Fracture of lower limb
230	S92591K	Fracture of lower limb
230	S92591P	Fracture of lower limb
230	S92591S	Fracture of lower limb
230	S92592A	Fracture of lower limb
230	S92592B	Fracture of lower limb
230	S92592D	Fracture of lower limb
230	S92592G	Fracture of lower limb
230	S92592K	Fracture of lower limb

230	S92592P	Fracture of lower limb
230	S92592S	Fracture of lower limb
230	S92599A	Fracture of lower limb
230	S92599B	Fracture of lower limb
230	S92599D	Fracture of lower limb
230	S92599G	Fracture of lower limb
230	S92599K	Fracture of lower limb
230	S92599P	Fracture of lower limb
230	S92599S	Fracture of lower limb
230	S92901A	Fracture of lower limb
230	S92901B	Fracture of lower limb
230	S92901D	Fracture of lower limb
230	S92901G	Fracture of lower limb
230	S92901K	Fracture of lower limb
230	S92901P	Fracture of lower limb
230	S92901S	Fracture of lower limb
230	S92902A	Fracture of lower limb
230	S92902B	Fracture of lower limb
230	S92902D	Fracture of lower limb
230	S92902G	Fracture of lower limb
230	S92902K	Fracture of lower limb
230	S92902P	Fracture of lower limb
230	S92902S	Fracture of lower limb
230	S92909A	Fracture of lower limb
230	S92909B	Fracture of lower limb
230	S92909D	Fracture of lower limb
230	S92909G	Fracture of lower limb
230	S92909K	Fracture of lower limb
230	S92909P	Fracture of lower limb
230	S92909S	Fracture of lower limb
230	S92911A	Fracture of lower limb
230	S92911B	Fracture of lower limb
230	S92911D	Fracture of lower limb
230	S92911G	Fracture of lower limb
230	S92911K	Fracture of lower limb
230	S92911P	Fracture of lower limb
230	S92911S	Fracture of lower limb
230	S92912A	Fracture of lower limb
230	S92912B	Fracture of lower limb
230	S92912D	Fracture of lower limb
230	S92912G	Fracture of lower limb
230	S92912K	Fracture of lower limb
230	S92912P	Fracture of lower limb
230	S92912S	Fracture of lower limb
230	S92919A	Fracture of lower limb
230	S92919B	Fracture of lower limb
230	S92919D	Fracture of lower limb
230	S92919G	Fracture of lower limb
230	S92919K	Fracture of lower limb
230	S92919P	Fracture of lower limb
230	S92919S	Fracture of lower limb

231	M4840XA	Other fractures
231	M4840XD	Other fractures
231	M4840XG	Other fractures
231	M4840XS	Other fractures
231	M4841XA	Other fractures
231	M4841XD	Other fractures
231	M4841XG	Other fractures
231	M4841XS	Other fractures
231	M4842XA	Other fractures
231	M4842XD	Other fractures
231	M4842XG	Other fractures
231	M4842XS	Other fractures
231	M4843XA	Other fractures
231	M4843XD	Other fractures
231	M4843XG	Other fractures
231	M4843XS	Other fractures
231	M4844XA	Other fractures
231	M4844XD	Other fractures
231	M4844XG	Other fractures
231	M4844XS	Other fractures
231	M4845XA	Other fractures
231	M4845XD	Other fractures
231	M4845XG	Other fractures
231	M4845XS	Other fractures
231	M4846XA	Other fractures
231	M4846XD	Other fractures
231	M4846XG	Other fractures
231	M4846XS	Other fractures
231	M4847XA	Other fractures
231	M4847XD	Other fractures
231	M4847XG	Other fractures
231	M4847XS	Other fractures
231	M4848XA	Other fractures
231	M4848XD	Other fractures
231	M4848XG	Other fractures
231	M4848XS	Other fractures
231	M4850XA	Other fractures
231	M4850XD	Other fractures
231	M4850XG	Other fractures
231	M4850XS	Other fractures
231	M4851XA	Other fractures
231	M4851XD	Other fractures
231	M4851XG	Other fractures
231	M4851XS	Other fractures
231	M4852XA	Other fractures
231	M4852XD	Other fractures
231	M4852XG	Other fractures
231	M4852XS	Other fractures
231	M4853XA	Other fractures
231	M4853XD	Other fractures
231	M4853XG	Other fractures

231	M4853XS	Other fractures
231	M4854XA	Other fractures
231	M4854XD	Other fractures
231	M4854XG	Other fractures
231	M4854XS	Other fractures
231	M4855XA	Other fractures
231	M4855XD	Other fractures
231	M4855XG	Other fractures
231	M4855XS	Other fractures
231	M4856XA	Other fractures
231	M4856XD	Other fractures
231	M4856XG	Other fractures
231	M4856XS	Other fractures
231	M4857XA	Other fractures
231	M4857XD	Other fractures
231	M4857XG	Other fractures
231	M4857XS	Other fractures
231	M4858XA	Other fractures
231	M4858XD	Other fractures
231	M4858XG	Other fractures
231	M4858XS	Other fractures
231	M8430XA	Other fractures
231	M8430XD	Other fractures
231	M8430XG	Other fractures
231	M8430XK	Other fractures
231	M8430XP	Other fractures
231	M8430XS	Other fractures
231	M84350A	Other fractures
231	M84350D	Other fractures
231	M84350G	Other fractures
231	M84350S	Other fractures
231	M8438XA	Other fractures
231	M8438XD	Other fractures
231	M8438XG	Other fractures
231	M8438XK	Other fractures
231	M8438XP	Other fractures
231	M8438XS	Other fractures
231	S12000A	Other fractures
231	S12000B	Other fractures
231	S12000D	Other fractures
231	S12000G	Other fractures
231	S12000K	Other fractures
231	S12000S	Other fractures
231	S12001A	Other fractures
231	S12001B	Other fractures
231	S12001D	Other fractures
231	S12001G	Other fractures
231	S12001K	Other fractures
231	S12001S	Other fractures
231	S1201XA	Other fractures
231	S1201XB	Other fractures

231	S1201XD	Other fractures
231	S1201XG	Other fractures
231	S1201XK	Other fractures
231	S1201XS	Other fractures
231	S1202XA	Other fractures
231	S1202XB	Other fractures
231	S1202XD	Other fractures
231	S1202XG	Other fractures
231	S1202XK	Other fractures
231	S1202XS	Other fractures
231	S12030A	Other fractures
231	S12030B	Other fractures
231	S12030D	Other fractures
231	S12030G	Other fractures
231	S12030K	Other fractures
231	S12030S	Other fractures
231	S12031A	Other fractures
231	S12031B	Other fractures
231	S12031D	Other fractures
231	S12031G	Other fractures
231	S12031K	Other fractures
231	S12031S	Other fractures
231	S12040A	Other fractures
231	S12040B	Other fractures
231	S12040D	Other fractures
231	S12040G	Other fractures
231	S12040K	Other fractures
231	S12040S	Other fractures
231	S12041A	Other fractures
231	S12041B	Other fractures
231	S12041D	Other fractures
231	S12041G	Other fractures
231	S12041K	Other fractures
231	S12041S	Other fractures
231	S12090A	Other fractures
231	S12090B	Other fractures
231	S12090D	Other fractures
231	S12090G	Other fractures
231	S12090K	Other fractures
231	S12090S	Other fractures
231	S12091A	Other fractures
231	S12091B	Other fractures
231	S12091D	Other fractures
231	S12091G	Other fractures
231	S12091K	Other fractures
231	S12091S	Other fractures
231	S12100A	Other fractures
231	S12100B	Other fractures
231	S12100D	Other fractures
231	S12100G	Other fractures
231	S12100K	Other fractures

231	S12100S	Other fractures
231	S12101A	Other fractures
231	S12101B	Other fractures
231	S12101D	Other fractures
231	S12101G	Other fractures
231	S12101K	Other fractures
231	S12101S	Other fractures
231	S12110A	Other fractures
231	S12110B	Other fractures
231	S12110D	Other fractures
231	S12110G	Other fractures
231	S12110K	Other fractures
231	S12110S	Other fractures
231	S12111A	Other fractures
231	S12111B	Other fractures
231	S12111D	Other fractures
231	S12111G	Other fractures
231	S12111K	Other fractures
231	S12111S	Other fractures
231	S12112A	Other fractures
231	S12112B	Other fractures
231	S12112D	Other fractures
231	S12112G	Other fractures
231	S12112K	Other fractures
231	S12112S	Other fractures
231	S12120A	Other fractures
231	S12120B	Other fractures
231	S12120D	Other fractures
231	S12120G	Other fractures
231	S12120K	Other fractures
231	S12120S	Other fractures
231	S12121A	Other fractures
231	S12121B	Other fractures
231	S12121D	Other fractures
231	S12121G	Other fractures
231	S12121K	Other fractures
231	S12121S	Other fractures
231	S12130A	Other fractures
231	S12130B	Other fractures
231	S12130D	Other fractures
231	S12130G	Other fractures
231	S12130K	Other fractures
231	S12130S	Other fractures
231	S12131A	Other fractures
231	S12131B	Other fractures
231	S12131D	Other fractures
231	S12131G	Other fractures
231	S12131K	Other fractures
231	S12131S	Other fractures
231	S1214XA	Other fractures
231	S1214XB	Other fractures

231	S1214XD	Other fractures
231	S1214XG	Other fractures
231	S1214XK	Other fractures
231	S1214XS	Other fractures
231	S12150A	Other fractures
231	S12150B	Other fractures
231	S12150D	Other fractures
231	S12150G	Other fractures
231	S12150K	Other fractures
231	S12150S	Other fractures
231	S12151A	Other fractures
231	S12151B	Other fractures
231	S12151D	Other fractures
231	S12151G	Other fractures
231	S12151K	Other fractures
231	S12151S	Other fractures
231	S12190A	Other fractures
231	S12190B	Other fractures
231	S12190D	Other fractures
231	S12190G	Other fractures
231	S12190K	Other fractures
231	S12190S	Other fractures
231	S12191A	Other fractures
231	S12191B	Other fractures
231	S12191D	Other fractures
231	S12191G	Other fractures
231	S12191K	Other fractures
231	S12191S	Other fractures
231	S12200A	Other fractures
231	S12200B	Other fractures
231	S12200D	Other fractures
231	S12200G	Other fractures
231	S12200K	Other fractures
231	S12200S	Other fractures
231	S12201A	Other fractures
231	S12201B	Other fractures
231	S12201D	Other fractures
231	S12201G	Other fractures
231	S12201K	Other fractures
231	S12201S	Other fractures
231	S12230A	Other fractures
231	S12230B	Other fractures
231	S12230D	Other fractures
231	S12230G	Other fractures
231	S12230K	Other fractures
231	S12230S	Other fractures
231	S12231A	Other fractures
231	S12231B	Other fractures
231	S12231D	Other fractures
231	S12231G	Other fractures
231	S12231K	Other fractures

231	S12231S	Other fractures
231	S1224XA	Other fractures
231	S1224XB	Other fractures
231	S1224XD	Other fractures
231	S1224XG	Other fractures
231	S1224XK	Other fractures
231	S1224XS	Other fractures
231	S12250A	Other fractures
231	S12250B	Other fractures
231	S12250D	Other fractures
231	S12250G	Other fractures
231	S12250K	Other fractures
231	S12250S	Other fractures
231	S12251A	Other fractures
231	S12251B	Other fractures
231	S12251D	Other fractures
231	S12251G	Other fractures
231	S12251K	Other fractures
231	S12251S	Other fractures
231	S12290A	Other fractures
231	S12290B	Other fractures
231	S12290D	Other fractures
231	S12290G	Other fractures
231	S12290K	Other fractures
231	S12290S	Other fractures
231	S12291A	Other fractures
231	S12291B	Other fractures
231	S12291D	Other fractures
231	S12291G	Other fractures
231	S12291K	Other fractures
231	S12291S	Other fractures
231	S12300A	Other fractures
231	S12300B	Other fractures
231	S12300D	Other fractures
231	S12300G	Other fractures
231	S12300K	Other fractures
231	S12300S	Other fractures
231	S12301A	Other fractures
231	S12301B	Other fractures
231	S12301D	Other fractures
231	S12301G	Other fractures
231	S12301K	Other fractures
231	S12301S	Other fractures
231	S12330A	Other fractures
231	S12330B	Other fractures
231	S12330D	Other fractures
231	S12330G	Other fractures
231	S12330K	Other fractures
231	S12330S	Other fractures
231	S12331A	Other fractures
231	S12331B	Other fractures

231	S12331D	Other fractures
231	S12331G	Other fractures
231	S12331K	Other fractures
231	S12331S	Other fractures
231	S1234XA	Other fractures
231	S1234XB	Other fractures
231	S1234XD	Other fractures
231	S1234XG	Other fractures
231	S1234XK	Other fractures
231	S1234XS	Other fractures
231	S12350A	Other fractures
231	S12350B	Other fractures
231	S12350D	Other fractures
231	S12350G	Other fractures
231	S12350K	Other fractures
231	S12350S	Other fractures
231	S12351A	Other fractures
231	S12351B	Other fractures
231	S12351D	Other fractures
231	S12351G	Other fractures
231	S12351K	Other fractures
231	S12351S	Other fractures
231	S12390A	Other fractures
231	S12390B	Other fractures
231	S12390D	Other fractures
231	S12390G	Other fractures
231	S12390K	Other fractures
231	S12390S	Other fractures
231	S12391A	Other fractures
231	S12391B	Other fractures
231	S12391D	Other fractures
231	S12391G	Other fractures
231	S12391K	Other fractures
231	S12391S	Other fractures
231	S12400A	Other fractures
231	S12400B	Other fractures
231	S12400D	Other fractures
231	S12400G	Other fractures
231	S12400K	Other fractures
231	S12400S	Other fractures
231	S12401A	Other fractures
231	S12401B	Other fractures
231	S12401D	Other fractures
231	S12401G	Other fractures
231	S12401K	Other fractures
231	S12401S	Other fractures
231	S12430A	Other fractures
231	S12430B	Other fractures
231	S12430D	Other fractures
231	S12430G	Other fractures
231	S12430K	Other fractures

231	S12430S	Other fractures
231	S12431A	Other fractures
231	S12431B	Other fractures
231	S12431D	Other fractures
231	S12431G	Other fractures
231	S12431K	Other fractures
231	S12431S	Other fractures
231	S1244XA	Other fractures
231	S1244XB	Other fractures
231	S1244XD	Other fractures
231	S1244XG	Other fractures
231	S1244XK	Other fractures
231	S1244XS	Other fractures
231	S12450A	Other fractures
231	S12450B	Other fractures
231	S12450D	Other fractures
231	S12450G	Other fractures
231	S12450K	Other fractures
231	S12450S	Other fractures
231	S12451A	Other fractures
231	S12451B	Other fractures
231	S12451D	Other fractures
231	S12451G	Other fractures
231	S12451K	Other fractures
231	S12451S	Other fractures
231	S12490A	Other fractures
231	S12490B	Other fractures
231	S12490D	Other fractures
231	S12490G	Other fractures
231	S12490K	Other fractures
231	S12490S	Other fractures
231	S12491A	Other fractures
231	S12491B	Other fractures
231	S12491D	Other fractures
231	S12491G	Other fractures
231	S12491K	Other fractures
231	S12491S	Other fractures
231	S12500A	Other fractures
231	S12500B	Other fractures
231	S12500D	Other fractures
231	S12500G	Other fractures
231	S12500K	Other fractures
231	S12500S	Other fractures
231	S12501A	Other fractures
231	S12501B	Other fractures
231	S12501D	Other fractures
231	S12501G	Other fractures
231	S12501K	Other fractures
231	S12501S	Other fractures
231	S12530A	Other fractures
231	S12530B	Other fractures

231	S12530D	Other fractures
231	S12530G	Other fractures
231	S12530K	Other fractures
231	S12530S	Other fractures
231	S12531A	Other fractures
231	S12531B	Other fractures
231	S12531D	Other fractures
231	S12531G	Other fractures
231	S12531K	Other fractures
231	S12531S	Other fractures
231	S1254XA	Other fractures
231	S1254XB	Other fractures
231	S1254XD	Other fractures
231	S1254XG	Other fractures
231	S1254XK	Other fractures
231	S1254XS	Other fractures
231	S12550A	Other fractures
231	S12550B	Other fractures
231	S12550D	Other fractures
231	S12550G	Other fractures
231	S12550K	Other fractures
231	S12550S	Other fractures
231	S12551A	Other fractures
231	S12551B	Other fractures
231	S12551D	Other fractures
231	S12551G	Other fractures
231	S12551K	Other fractures
231	S12551S	Other fractures
231	S12590A	Other fractures
231	S12590B	Other fractures
231	S12590D	Other fractures
231	S12590G	Other fractures
231	S12590K	Other fractures
231	S12590S	Other fractures
231	S12591A	Other fractures
231	S12591B	Other fractures
231	S12591D	Other fractures
231	S12591G	Other fractures
231	S12591K	Other fractures
231	S12591S	Other fractures
231	S12600A	Other fractures
231	S12600B	Other fractures
231	S12600D	Other fractures
231	S12600G	Other fractures
231	S12600K	Other fractures
231	S12600S	Other fractures
231	S12601A	Other fractures
231	S12601B	Other fractures
231	S12601D	Other fractures
231	S12601G	Other fractures
231	S12601K	Other fractures

231	S12601S	Other fractures
231	S12630A	Other fractures
231	S12630B	Other fractures
231	S12630D	Other fractures
231	S12630G	Other fractures
231	S12630K	Other fractures
231	S12630S	Other fractures
231	S12631A	Other fractures
231	S12631B	Other fractures
231	S12631D	Other fractures
231	S12631G	Other fractures
231	S12631K	Other fractures
231	S12631S	Other fractures
231	S1264XA	Other fractures
231	S1264XB	Other fractures
231	S1264XD	Other fractures
231	S1264XG	Other fractures
231	S1264XK	Other fractures
231	S1264XS	Other fractures
231	S12650A	Other fractures
231	S12650B	Other fractures
231	S12650D	Other fractures
231	S12650G	Other fractures
231	S12650K	Other fractures
231	S12650S	Other fractures
231	S12651A	Other fractures
231	S12651B	Other fractures
231	S12651D	Other fractures
231	S12651G	Other fractures
231	S12651K	Other fractures
231	S12651S	Other fractures
231	S12690A	Other fractures
231	S12690B	Other fractures
231	S12690D	Other fractures
231	S12690G	Other fractures
231	S12690K	Other fractures
231	S12690S	Other fractures
231	S12691A	Other fractures
231	S12691B	Other fractures
231	S12691D	Other fractures
231	S12691G	Other fractures
231	S12691K	Other fractures
231	S12691S	Other fractures
231	S128XXA	Other fractures
231	S128XXD	Other fractures
231	S128XXS	Other fractures
231	S129XXA	Other fractures
231	S129XXD	Other fractures
231	S129XXS	Other fractures
231	S22000A	Other fractures
231	S22000B	Other fractures

231	S22000D	Other fractures
231	S22000G	Other fractures
231	S22000K	Other fractures
231	S22000S	Other fractures
231	S22001A	Other fractures
231	S22001B	Other fractures
231	S22001D	Other fractures
231	S22001G	Other fractures
231	S22001K	Other fractures
231	S22001S	Other fractures
231	S22002A	Other fractures
231	S22002B	Other fractures
231	S22002D	Other fractures
231	S22002G	Other fractures
231	S22002K	Other fractures
231	S22002S	Other fractures
231	S22008A	Other fractures
231	S22008B	Other fractures
231	S22008D	Other fractures
231	S22008G	Other fractures
231	S22008K	Other fractures
231	S22008S	Other fractures
231	S22009A	Other fractures
231	S22009B	Other fractures
231	S22009D	Other fractures
231	S22009G	Other fractures
231	S22009K	Other fractures
231	S22009S	Other fractures
231	S22010A	Other fractures
231	S22010B	Other fractures
231	S22010D	Other fractures
231	S22010G	Other fractures
231	S22010K	Other fractures
231	S22010S	Other fractures
231	S22011A	Other fractures
231	S22011B	Other fractures
231	S22011D	Other fractures
231	S22011G	Other fractures
231	S22011K	Other fractures
231	S22011S	Other fractures
231	S22012A	Other fractures
231	S22012B	Other fractures
231	S22012D	Other fractures
231	S22012G	Other fractures
231	S22012K	Other fractures
231	S22012S	Other fractures
231	S22018A	Other fractures
231	S22018B	Other fractures
231	S22018D	Other fractures
231	S22018G	Other fractures
231	S22018K	Other fractures

231	S22018S	Other fractures
231	S22019A	Other fractures
231	S22019B	Other fractures
231	S22019D	Other fractures
231	S22019G	Other fractures
231	S22019K	Other fractures
231	S22019S	Other fractures
231	S22020A	Other fractures
231	S22020B	Other fractures
231	S22020D	Other fractures
231	S22020G	Other fractures
231	S22020K	Other fractures
231	S22020S	Other fractures
231	S22021A	Other fractures
231	S22021B	Other fractures
231	S22021D	Other fractures
231	S22021G	Other fractures
231	S22021K	Other fractures
231	S22021S	Other fractures
231	S22022A	Other fractures
231	S22022B	Other fractures
231	S22022D	Other fractures
231	S22022G	Other fractures
231	S22022K	Other fractures
231	S22022S	Other fractures
231	S22028A	Other fractures
231	S22028B	Other fractures
231	S22028D	Other fractures
231	S22028G	Other fractures
231	S22028K	Other fractures
231	S22028S	Other fractures
231	S22029A	Other fractures
231	S22029B	Other fractures
231	S22029D	Other fractures
231	S22029G	Other fractures
231	S22029K	Other fractures
231	S22029S	Other fractures
231	S22030A	Other fractures
231	S22030B	Other fractures
231	S22030D	Other fractures
231	S22030G	Other fractures
231	S22030K	Other fractures
231	S22030S	Other fractures
231	S22031A	Other fractures
231	S22031B	Other fractures
231	S22031D	Other fractures
231	S22031G	Other fractures
231	S22031K	Other fractures
231	S22031S	Other fractures
231	S22032A	Other fractures
231	S22032B	Other fractures

231	S22032D	Other fractures
231	S22032G	Other fractures
231	S22032K	Other fractures
231	S22032S	Other fractures
231	S22038A	Other fractures
231	S22038B	Other fractures
231	S22038D	Other fractures
231	S22038G	Other fractures
231	S22038K	Other fractures
231	S22038S	Other fractures
231	S22039A	Other fractures
231	S22039B	Other fractures
231	S22039D	Other fractures
231	S22039G	Other fractures
231	S22039K	Other fractures
231	S22039S	Other fractures
231	S22040A	Other fractures
231	S22040B	Other fractures
231	S22040D	Other fractures
231	S22040G	Other fractures
231	S22040K	Other fractures
231	S22040S	Other fractures
231	S22041A	Other fractures
231	S22041B	Other fractures
231	S22041D	Other fractures
231	S22041G	Other fractures
231	S22041K	Other fractures
231	S22041S	Other fractures
231	S22042A	Other fractures
231	S22042B	Other fractures
231	S22042D	Other fractures
231	S22042G	Other fractures
231	S22042K	Other fractures
231	S22042S	Other fractures
231	S22048A	Other fractures
231	S22048B	Other fractures
231	S22048D	Other fractures
231	S22048G	Other fractures
231	S22048K	Other fractures
231	S22048S	Other fractures
231	S22049A	Other fractures
231	S22049B	Other fractures
231	S22049D	Other fractures
231	S22049G	Other fractures
231	S22049K	Other fractures
231	S22049S	Other fractures
231	S22050A	Other fractures
231	S22050B	Other fractures
231	S22050D	Other fractures
231	S22050G	Other fractures
231	S22050K	Other fractures

231	S22050S	Other fractures
231	S22051A	Other fractures
231	S22051B	Other fractures
231	S22051D	Other fractures
231	S22051G	Other fractures
231	S22051K	Other fractures
231	S22051S	Other fractures
231	S22052A	Other fractures
231	S22052B	Other fractures
231	S22052D	Other fractures
231	S22052G	Other fractures
231	S22052K	Other fractures
231	S22052S	Other fractures
231	S22058A	Other fractures
231	S22058B	Other fractures
231	S22058D	Other fractures
231	S22058G	Other fractures
231	S22058K	Other fractures
231	S22058S	Other fractures
231	S22059A	Other fractures
231	S22059B	Other fractures
231	S22059D	Other fractures
231	S22059G	Other fractures
231	S22059K	Other fractures
231	S22059S	Other fractures
231	S22060A	Other fractures
231	S22060B	Other fractures
231	S22060D	Other fractures
231	S22060G	Other fractures
231	S22060K	Other fractures
231	S22060S	Other fractures
231	S22061A	Other fractures
231	S22061B	Other fractures
231	S22061D	Other fractures
231	S22061G	Other fractures
231	S22061K	Other fractures
231	S22061S	Other fractures
231	S22062A	Other fractures
231	S22062B	Other fractures
231	S22062D	Other fractures
231	S22062G	Other fractures
231	S22062K	Other fractures
231	S22062S	Other fractures
231	S22068A	Other fractures
231	S22068B	Other fractures
231	S22068D	Other fractures
231	S22068G	Other fractures
231	S22068K	Other fractures
231	S22068S	Other fractures
231	S22069A	Other fractures
231	S22069B	Other fractures

231	S22069D	Other fractures
231	S22069G	Other fractures
231	S22069K	Other fractures
231	S22069S	Other fractures
231	S22070A	Other fractures
231	S22070B	Other fractures
231	S22070D	Other fractures
231	S22070G	Other fractures
231	S22070K	Other fractures
231	S22070S	Other fractures
231	S22071A	Other fractures
231	S22071B	Other fractures
231	S22071D	Other fractures
231	S22071G	Other fractures
231	S22071K	Other fractures
231	S22071S	Other fractures
231	S22072A	Other fractures
231	S22072B	Other fractures
231	S22072D	Other fractures
231	S22072G	Other fractures
231	S22072K	Other fractures
231	S22072S	Other fractures
231	S22078A	Other fractures
231	S22078B	Other fractures
231	S22078D	Other fractures
231	S22078G	Other fractures
231	S22078K	Other fractures
231	S22078S	Other fractures
231	S22079A	Other fractures
231	S22079B	Other fractures
231	S22079D	Other fractures
231	S22079G	Other fractures
231	S22079K	Other fractures
231	S22079S	Other fractures
231	S22080A	Other fractures
231	S22080B	Other fractures
231	S22080D	Other fractures
231	S22080G	Other fractures
231	S22080K	Other fractures
231	S22080S	Other fractures
231	S22081A	Other fractures
231	S22081B	Other fractures
231	S22081D	Other fractures
231	S22081G	Other fractures
231	S22081K	Other fractures
231	S22081S	Other fractures
231	S22082A	Other fractures
231	S22082B	Other fractures
231	S22082D	Other fractures
231	S22082G	Other fractures
231	S22082K	Other fractures

231	S22082S	Other fractures
231	S22088A	Other fractures
231	S22088B	Other fractures
231	S22088D	Other fractures
231	S22088G	Other fractures
231	S22088K	Other fractures
231	S22088S	Other fractures
231	S22089A	Other fractures
231	S22089B	Other fractures
231	S22089D	Other fractures
231	S22089G	Other fractures
231	S22089K	Other fractures
231	S22089S	Other fractures
231	S2220XA	Other fractures
231	S2220XB	Other fractures
231	S2220XD	Other fractures
231	S2220XG	Other fractures
231	S2220XK	Other fractures
231	S2220XS	Other fractures
231	S2221XA	Other fractures
231	S2221XB	Other fractures
231	S2221XD	Other fractures
231	S2221XG	Other fractures
231	S2221XK	Other fractures
231	S2221XS	Other fractures
231	S2222XA	Other fractures
231	S2222XB	Other fractures
231	S2222XD	Other fractures
231	S2222XG	Other fractures
231	S2222XK	Other fractures
231	S2222XS	Other fractures
231	S2223XA	Other fractures
231	S2223XB	Other fractures
231	S2223XD	Other fractures
231	S2223XG	Other fractures
231	S2223XK	Other fractures
231	S2223XS	Other fractures
231	S2224XA	Other fractures
231	S2224XB	Other fractures
231	S2224XD	Other fractures
231	S2224XG	Other fractures
231	S2224XK	Other fractures
231	S2224XS	Other fractures
231	S2231XA	Other fractures
231	S2231XB	Other fractures
231	S2231XD	Other fractures
231	S2231XG	Other fractures
231	S2231XK	Other fractures
231	S2231XS	Other fractures
231	S2232XA	Other fractures
231	S2232XB	Other fractures

231	S2232XD	Other fractures
231	S2232XG	Other fractures
231	S2232XK	Other fractures
231	S2232XS	Other fractures
231	S2239XA	Other fractures
231	S2239XB	Other fractures
231	S2239XD	Other fractures
231	S2239XG	Other fractures
231	S2239XK	Other fractures
231	S2239XS	Other fractures
231	S2241XA	Other fractures
231	S2241XB	Other fractures
231	S2241XD	Other fractures
231	S2241XG	Other fractures
231	S2241XK	Other fractures
231	S2241XS	Other fractures
231	S2242XA	Other fractures
231	S2242XB	Other fractures
231	S2242XD	Other fractures
231	S2242XG	Other fractures
231	S2242XK	Other fractures
231	S2242XS	Other fractures
231	S2243XA	Other fractures
231	S2243XB	Other fractures
231	S2243XD	Other fractures
231	S2243XG	Other fractures
231	S2243XK	Other fractures
231	S2243XS	Other fractures
231	S2249XA	Other fractures
231	S2249XB	Other fractures
231	S2249XD	Other fractures
231	S2249XG	Other fractures
231	S2249XK	Other fractures
231	S2249XS	Other fractures
231	S225XXA	Other fractures
231	S225XXB	Other fractures
231	S225XXD	Other fractures
231	S225XXG	Other fractures
231	S225XXK	Other fractures
231	S225XXS	Other fractures
231	S229XXA	Other fractures
231	S229XXB	Other fractures
231	S229XXD	Other fractures
231	S229XXG	Other fractures
231	S229XXK	Other fractures
231	S229XXS	Other fractures
231	S32000A	Other fractures
231	S32000B	Other fractures
231	S32000D	Other fractures
231	S32000G	Other fractures
231	S32000K	Other fractures

231	S32000S	Other fractures
231	S32001A	Other fractures
231	S32001B	Other fractures
231	S32001D	Other fractures
231	S32001G	Other fractures
231	S32001K	Other fractures
231	S32001S	Other fractures
231	S32002A	Other fractures
231	S32002B	Other fractures
231	S32002D	Other fractures
231	S32002G	Other fractures
231	S32002K	Other fractures
231	S32002S	Other fractures
231	S32008A	Other fractures
231	S32008B	Other fractures
231	S32008D	Other fractures
231	S32008G	Other fractures
231	S32008K	Other fractures
231	S32008S	Other fractures
231	S32009A	Other fractures
231	S32009B	Other fractures
231	S32009D	Other fractures
231	S32009G	Other fractures
231	S32009K	Other fractures
231	S32009S	Other fractures
231	S32010A	Other fractures
231	S32010B	Other fractures
231	S32010D	Other fractures
231	S32010G	Other fractures
231	S32010K	Other fractures
231	S32010S	Other fractures
231	S32011A	Other fractures
231	S32011B	Other fractures
231	S32011D	Other fractures
231	S32011G	Other fractures
231	S32011K	Other fractures
231	S32011S	Other fractures
231	S32012A	Other fractures
231	S32012B	Other fractures
231	S32012D	Other fractures
231	S32012G	Other fractures
231	S32012K	Other fractures
231	S32012S	Other fractures
231	S32018A	Other fractures
231	S32018B	Other fractures
231	S32018D	Other fractures
231	S32018G	Other fractures
231	S32018K	Other fractures
231	S32018S	Other fractures
231	S32019A	Other fractures
231	S32019B	Other fractures

231	S32019D	Other fractures
231	S32019G	Other fractures
231	S32019K	Other fractures
231	S32019S	Other fractures
231	S32020A	Other fractures
231	S32020B	Other fractures
231	S32020D	Other fractures
231	S32020G	Other fractures
231	S32020K	Other fractures
231	S32020S	Other fractures
231	S32021A	Other fractures
231	S32021B	Other fractures
231	S32021D	Other fractures
231	S32021G	Other fractures
231	S32021K	Other fractures
231	S32021S	Other fractures
231	S32022A	Other fractures
231	S32022B	Other fractures
231	S32022D	Other fractures
231	S32022G	Other fractures
231	S32022K	Other fractures
231	S32022S	Other fractures
231	S32028A	Other fractures
231	S32028B	Other fractures
231	S32028D	Other fractures
231	S32028G	Other fractures
231	S32028K	Other fractures
231	S32028S	Other fractures
231	S32029A	Other fractures
231	S32029B	Other fractures
231	S32029D	Other fractures
231	S32029G	Other fractures
231	S32029K	Other fractures
231	S32029S	Other fractures
231	S32030A	Other fractures
231	S32030B	Other fractures
231	S32030D	Other fractures
231	S32030G	Other fractures
231	S32030K	Other fractures
231	S32030S	Other fractures
231	S32031A	Other fractures
231	S32031B	Other fractures
231	S32031D	Other fractures
231	S32031G	Other fractures
231	S32031K	Other fractures
231	S32031S	Other fractures
231	S32032A	Other fractures
231	S32032B	Other fractures
231	S32032D	Other fractures
231	S32032G	Other fractures
231	S32032K	Other fractures

231	S32032S	Other fractures
231	S32038A	Other fractures
231	S32038B	Other fractures
231	S32038D	Other fractures
231	S32038G	Other fractures
231	S32038K	Other fractures
231	S32038S	Other fractures
231	S32039A	Other fractures
231	S32039B	Other fractures
231	S32039D	Other fractures
231	S32039G	Other fractures
231	S32039K	Other fractures
231	S32039S	Other fractures
231	S32040A	Other fractures
231	S32040B	Other fractures
231	S32040D	Other fractures
231	S32040G	Other fractures
231	S32040K	Other fractures
231	S32040S	Other fractures
231	S32041A	Other fractures
231	S32041B	Other fractures
231	S32041D	Other fractures
231	S32041G	Other fractures
231	S32041K	Other fractures
231	S32041S	Other fractures
231	S32042A	Other fractures
231	S32042B	Other fractures
231	S32042D	Other fractures
231	S32042G	Other fractures
231	S32042K	Other fractures
231	S32042S	Other fractures
231	S32048A	Other fractures
231	S32048B	Other fractures
231	S32048D	Other fractures
231	S32048G	Other fractures
231	S32048K	Other fractures
231	S32048S	Other fractures
231	S32049A	Other fractures
231	S32049B	Other fractures
231	S32049D	Other fractures
231	S32049G	Other fractures
231	S32049K	Other fractures
231	S32049S	Other fractures
231	S32050A	Other fractures
231	S32050B	Other fractures
231	S32050D	Other fractures
231	S32050G	Other fractures
231	S32050K	Other fractures
231	S32050S	Other fractures
231	S32051A	Other fractures
231	S32051B	Other fractures

231	S32051D	Other fractures
231	S32051G	Other fractures
231	S32051K	Other fractures
231	S32051S	Other fractures
231	S32052A	Other fractures
231	S32052B	Other fractures
231	S32052D	Other fractures
231	S32052G	Other fractures
231	S32052K	Other fractures
231	S32052S	Other fractures
231	S32058A	Other fractures
231	S32058B	Other fractures
231	S32058D	Other fractures
231	S32058G	Other fractures
231	S32058K	Other fractures
231	S32058S	Other fractures
231	S32059A	Other fractures
231	S32059B	Other fractures
231	S32059D	Other fractures
231	S32059G	Other fractures
231	S32059K	Other fractures
231	S32059S	Other fractures
231	S3210XA	Other fractures
231	S3210XB	Other fractures
231	S3210XD	Other fractures
231	S3210XG	Other fractures
231	S3210XK	Other fractures
231	S3210XS	Other fractures
231	S32110A	Other fractures
231	S32110B	Other fractures
231	S32110D	Other fractures
231	S32110G	Other fractures
231	S32110K	Other fractures
231	S32110S	Other fractures
231	S32111A	Other fractures
231	S32111B	Other fractures
231	S32111D	Other fractures
231	S32111G	Other fractures
231	S32111K	Other fractures
231	S32111S	Other fractures
231	S32112A	Other fractures
231	S32112B	Other fractures
231	S32112D	Other fractures
231	S32112G	Other fractures
231	S32112K	Other fractures
231	S32112S	Other fractures
231	S32119A	Other fractures
231	S32119B	Other fractures
231	S32119D	Other fractures
231	S32119G	Other fractures
231	S32119K	Other fractures

231	S32119S	Other fractures
231	S32120A	Other fractures
231	S32120B	Other fractures
231	S32120D	Other fractures
231	S32120G	Other fractures
231	S32120K	Other fractures
231	S32120S	Other fractures
231	S32121A	Other fractures
231	S32121B	Other fractures
231	S32121D	Other fractures
231	S32121G	Other fractures
231	S32121K	Other fractures
231	S32121S	Other fractures
231	S32122A	Other fractures
231	S32122B	Other fractures
231	S32122D	Other fractures
231	S32122G	Other fractures
231	S32122K	Other fractures
231	S32122S	Other fractures
231	S32129A	Other fractures
231	S32129B	Other fractures
231	S32129D	Other fractures
231	S32129G	Other fractures
231	S32129K	Other fractures
231	S32129S	Other fractures
231	S32130A	Other fractures
231	S32130B	Other fractures
231	S32130D	Other fractures
231	S32130G	Other fractures
231	S32130K	Other fractures
231	S32130S	Other fractures
231	S32131A	Other fractures
231	S32131B	Other fractures
231	S32131D	Other fractures
231	S32131G	Other fractures
231	S32131K	Other fractures
231	S32131S	Other fractures
231	S32132A	Other fractures
231	S32132B	Other fractures
231	S32132D	Other fractures
231	S32132G	Other fractures
231	S32132K	Other fractures
231	S32132S	Other fractures
231	S32139A	Other fractures
231	S32139B	Other fractures
231	S32139D	Other fractures
231	S32139G	Other fractures
231	S32139K	Other fractures
231	S32139S	Other fractures
231	S3214XA	Other fractures
231	S3214XB	Other fractures

231	S3214XD	Other fractures
231	S3214XG	Other fractures
231	S3214XK	Other fractures
231	S3214XS	Other fractures
231	S3215XA	Other fractures
231	S3215XB	Other fractures
231	S3215XD	Other fractures
231	S3215XG	Other fractures
231	S3215XK	Other fractures
231	S3215XS	Other fractures
231	S3216XA	Other fractures
231	S3216XB	Other fractures
231	S3216XD	Other fractures
231	S3216XG	Other fractures
231	S3216XK	Other fractures
231	S3216XS	Other fractures
231	S3217XA	Other fractures
231	S3217XB	Other fractures
231	S3217XD	Other fractures
231	S3217XG	Other fractures
231	S3217XK	Other fractures
231	S3217XS	Other fractures
231	S3219XA	Other fractures
231	S3219XB	Other fractures
231	S3219XD	Other fractures
231	S3219XG	Other fractures
231	S3219XK	Other fractures
231	S3219XS	Other fractures
231	S322XXA	Other fractures
231	S322XXB	Other fractures
231	S322XXD	Other fractures
231	S322XXG	Other fractures
231	S322XXK	Other fractures
231	S322XXS	Other fractures
231	S32301A	Other fractures
231	S32301B	Other fractures
231	S32301D	Other fractures
231	S32301G	Other fractures
231	S32301K	Other fractures
231	S32301S	Other fractures
231	S32302A	Other fractures
231	S32302B	Other fractures
231	S32302D	Other fractures
231	S32302G	Other fractures
231	S32302K	Other fractures
231	S32302S	Other fractures
231	S32309A	Other fractures
231	S32309B	Other fractures
231	S32309D	Other fractures
231	S32309G	Other fractures
231	S32309K	Other fractures

231	S32309S	Other fractures
231	S32311A	Other fractures
231	S32311B	Other fractures
231	S32311D	Other fractures
231	S32311G	Other fractures
231	S32311K	Other fractures
231	S32311S	Other fractures
231	S32312A	Other fractures
231	S32312B	Other fractures
231	S32312D	Other fractures
231	S32312G	Other fractures
231	S32312K	Other fractures
231	S32312S	Other fractures
231	S32313A	Other fractures
231	S32313B	Other fractures
231	S32313D	Other fractures
231	S32313G	Other fractures
231	S32313K	Other fractures
231	S32313S	Other fractures
231	S32314A	Other fractures
231	S32314B	Other fractures
231	S32314D	Other fractures
231	S32314G	Other fractures
231	S32314K	Other fractures
231	S32314S	Other fractures
231	S32315A	Other fractures
231	S32315B	Other fractures
231	S32315D	Other fractures
231	S32315G	Other fractures
231	S32315K	Other fractures
231	S32315S	Other fractures
231	S32316A	Other fractures
231	S32316B	Other fractures
231	S32316D	Other fractures
231	S32316G	Other fractures
231	S32316K	Other fractures
231	S32316S	Other fractures
231	S32391A	Other fractures
231	S32391B	Other fractures
231	S32391D	Other fractures
231	S32391G	Other fractures
231	S32391K	Other fractures
231	S32391S	Other fractures
231	S32392A	Other fractures
231	S32392B	Other fractures
231	S32392D	Other fractures
231	S32392G	Other fractures
231	S32392K	Other fractures
231	S32392S	Other fractures
231	S32399A	Other fractures
231	S32399B	Other fractures

231	S32399D	Other fractures
231	S32399G	Other fractures
231	S32399K	Other fractures
231	S32399S	Other fractures
231	S32401A	Other fractures
231	S32401B	Other fractures
231	S32401D	Other fractures
231	S32401G	Other fractures
231	S32401K	Other fractures
231	S32401S	Other fractures
231	S32402A	Other fractures
231	S32402B	Other fractures
231	S32402D	Other fractures
231	S32402G	Other fractures
231	S32402K	Other fractures
231	S32402S	Other fractures
231	S32409A	Other fractures
231	S32409B	Other fractures
231	S32409D	Other fractures
231	S32409G	Other fractures
231	S32409K	Other fractures
231	S32409S	Other fractures
231	S32411A	Other fractures
231	S32411B	Other fractures
231	S32411D	Other fractures
231	S32411G	Other fractures
231	S32411K	Other fractures
231	S32411S	Other fractures
231	S32412A	Other fractures
231	S32412B	Other fractures
231	S32412D	Other fractures
231	S32412G	Other fractures
231	S32412K	Other fractures
231	S32412S	Other fractures
231	S32413A	Other fractures
231	S32413B	Other fractures
231	S32413D	Other fractures
231	S32413G	Other fractures
231	S32413K	Other fractures
231	S32413S	Other fractures
231	S32414A	Other fractures
231	S32414B	Other fractures
231	S32414D	Other fractures
231	S32414G	Other fractures
231	S32414K	Other fractures
231	S32414S	Other fractures
231	S32415A	Other fractures
231	S32415B	Other fractures
231	S32415D	Other fractures
231	S32415G	Other fractures
231	S32415K	Other fractures

231	S32415S	Other fractures
231	S32416A	Other fractures
231	S32416B	Other fractures
231	S32416D	Other fractures
231	S32416G	Other fractures
231	S32416K	Other fractures
231	S32416S	Other fractures
231	S32421A	Other fractures
231	S32421B	Other fractures
231	S32421D	Other fractures
231	S32421G	Other fractures
231	S32421K	Other fractures
231	S32421S	Other fractures
231	S32422A	Other fractures
231	S32422B	Other fractures
231	S32422D	Other fractures
231	S32422G	Other fractures
231	S32422K	Other fractures
231	S32422S	Other fractures
231	S32423A	Other fractures
231	S32423B	Other fractures
231	S32423D	Other fractures
231	S32423G	Other fractures
231	S32423K	Other fractures
231	S32423S	Other fractures
231	S32424A	Other fractures
231	S32424B	Other fractures
231	S32424D	Other fractures
231	S32424G	Other fractures
231	S32424K	Other fractures
231	S32424S	Other fractures
231	S32425A	Other fractures
231	S32425B	Other fractures
231	S32425D	Other fractures
231	S32425G	Other fractures
231	S32425K	Other fractures
231	S32425S	Other fractures
231	S32426A	Other fractures
231	S32426B	Other fractures
231	S32426D	Other fractures
231	S32426G	Other fractures
231	S32426K	Other fractures
231	S32426S	Other fractures
231	S32431A	Other fractures
231	S32431B	Other fractures
231	S32431D	Other fractures
231	S32431G	Other fractures
231	S32431K	Other fractures
231	S32431S	Other fractures
231	S32432A	Other fractures
231	S32432B	Other fractures

231	S32432D	Other fractures
231	S32432G	Other fractures
231	S32432K	Other fractures
231	S32432S	Other fractures
231	S32433A	Other fractures
231	S32433B	Other fractures
231	S32433D	Other fractures
231	S32433G	Other fractures
231	S32433K	Other fractures
231	S32433S	Other fractures
231	S32434A	Other fractures
231	S32434B	Other fractures
231	S32434D	Other fractures
231	S32434G	Other fractures
231	S32434K	Other fractures
231	S32434S	Other fractures
231	S32435A	Other fractures
231	S32435B	Other fractures
231	S32435D	Other fractures
231	S32435G	Other fractures
231	S32435K	Other fractures
231	S32435S	Other fractures
231	S32436A	Other fractures
231	S32436B	Other fractures
231	S32436D	Other fractures
231	S32436G	Other fractures
231	S32436K	Other fractures
231	S32436S	Other fractures
231	S32441A	Other fractures
231	S32441B	Other fractures
231	S32441D	Other fractures
231	S32441G	Other fractures
231	S32441K	Other fractures
231	S32441S	Other fractures
231	S32442A	Other fractures
231	S32442B	Other fractures
231	S32442D	Other fractures
231	S32442G	Other fractures
231	S32442K	Other fractures
231	S32442S	Other fractures
231	S32443A	Other fractures
231	S32443B	Other fractures
231	S32443D	Other fractures
231	S32443G	Other fractures
231	S32443K	Other fractures
231	S32443S	Other fractures
231	S32444A	Other fractures
231	S32444B	Other fractures
231	S32444D	Other fractures
231	S32444G	Other fractures
231	S32444K	Other fractures

231	S32444S	Other fractures
231	S32445A	Other fractures
231	S32445B	Other fractures
231	S32445D	Other fractures
231	S32445G	Other fractures
231	S32445K	Other fractures
231	S32445S	Other fractures
231	S32446A	Other fractures
231	S32446B	Other fractures
231	S32446D	Other fractures
231	S32446G	Other fractures
231	S32446K	Other fractures
231	S32446S	Other fractures
231	S32451A	Other fractures
231	S32451B	Other fractures
231	S32451D	Other fractures
231	S32451G	Other fractures
231	S32451K	Other fractures
231	S32451S	Other fractures
231	S32452A	Other fractures
231	S32452B	Other fractures
231	S32452D	Other fractures
231	S32452G	Other fractures
231	S32452K	Other fractures
231	S32452S	Other fractures
231	S32453A	Other fractures
231	S32453B	Other fractures
231	S32453D	Other fractures
231	S32453G	Other fractures
231	S32453K	Other fractures
231	S32453S	Other fractures
231	S32454A	Other fractures
231	S32454B	Other fractures
231	S32454D	Other fractures
231	S32454G	Other fractures
231	S32454K	Other fractures
231	S32454S	Other fractures
231	S32455A	Other fractures
231	S32455B	Other fractures
231	S32455D	Other fractures
231	S32455G	Other fractures
231	S32455K	Other fractures
231	S32455S	Other fractures
231	S32456A	Other fractures
231	S32456B	Other fractures
231	S32456D	Other fractures
231	S32456G	Other fractures
231	S32456K	Other fractures
231	S32456S	Other fractures
231	S32461A	Other fractures
231	S32461B	Other fractures

231	S32461D	Other fractures
231	S32461G	Other fractures
231	S32461K	Other fractures
231	S32461S	Other fractures
231	S32462A	Other fractures
231	S32462B	Other fractures
231	S32462D	Other fractures
231	S32462G	Other fractures
231	S32462K	Other fractures
231	S32462S	Other fractures
231	S32463A	Other fractures
231	S32463B	Other fractures
231	S32463D	Other fractures
231	S32463G	Other fractures
231	S32463K	Other fractures
231	S32463S	Other fractures
231	S32464A	Other fractures
231	S32464B	Other fractures
231	S32464D	Other fractures
231	S32464G	Other fractures
231	S32464K	Other fractures
231	S32464S	Other fractures
231	S32465A	Other fractures
231	S32465B	Other fractures
231	S32465D	Other fractures
231	S32465G	Other fractures
231	S32465K	Other fractures
231	S32465S	Other fractures
231	S32466A	Other fractures
231	S32466B	Other fractures
231	S32466D	Other fractures
231	S32466G	Other fractures
231	S32466K	Other fractures
231	S32466S	Other fractures
231	S32471A	Other fractures
231	S32471B	Other fractures
231	S32471D	Other fractures
231	S32471G	Other fractures
231	S32471K	Other fractures
231	S32471S	Other fractures
231	S32472A	Other fractures
231	S32472B	Other fractures
231	S32472D	Other fractures
231	S32472G	Other fractures
231	S32472K	Other fractures
231	S32472S	Other fractures
231	S32473A	Other fractures
231	S32473B	Other fractures
231	S32473D	Other fractures
231	S32473G	Other fractures
231	S32473K	Other fractures

231	S32473S	Other fractures
231	S32474A	Other fractures
231	S32474B	Other fractures
231	S32474D	Other fractures
231	S32474G	Other fractures
231	S32474K	Other fractures
231	S32474S	Other fractures
231	S32475A	Other fractures
231	S32475B	Other fractures
231	S32475D	Other fractures
231	S32475G	Other fractures
231	S32475K	Other fractures
231	S32475S	Other fractures
231	S32476A	Other fractures
231	S32476B	Other fractures
231	S32476D	Other fractures
231	S32476G	Other fractures
231	S32476K	Other fractures
231	S32476S	Other fractures
231	S32481A	Other fractures
231	S32481B	Other fractures
231	S32481D	Other fractures
231	S32481G	Other fractures
231	S32481K	Other fractures
231	S32481S	Other fractures
231	S32482A	Other fractures
231	S32482B	Other fractures
231	S32482D	Other fractures
231	S32482G	Other fractures
231	S32482K	Other fractures
231	S32482S	Other fractures
231	S32483A	Other fractures
231	S32483B	Other fractures
231	S32483D	Other fractures
231	S32483G	Other fractures
231	S32483K	Other fractures
231	S32483S	Other fractures
231	S32484A	Other fractures
231	S32484B	Other fractures
231	S32484D	Other fractures
231	S32484G	Other fractures
231	S32484K	Other fractures
231	S32484S	Other fractures
231	S32485A	Other fractures
231	S32485B	Other fractures
231	S32485D	Other fractures
231	S32485G	Other fractures
231	S32485K	Other fractures
231	S32485S	Other fractures
231	S32486A	Other fractures
231	S32486B	Other fractures

231	S32486D	Other fractures
231	S32486G	Other fractures
231	S32486K	Other fractures
231	S32486S	Other fractures
231	S32491A	Other fractures
231	S32491B	Other fractures
231	S32491D	Other fractures
231	S32491G	Other fractures
231	S32491K	Other fractures
231	S32491S	Other fractures
231	S32492A	Other fractures
231	S32492B	Other fractures
231	S32492D	Other fractures
231	S32492G	Other fractures
231	S32492K	Other fractures
231	S32492S	Other fractures
231	S32499A	Other fractures
231	S32499B	Other fractures
231	S32499D	Other fractures
231	S32499G	Other fractures
231	S32499K	Other fractures
231	S32499S	Other fractures
231	S32501A	Other fractures
231	S32501B	Other fractures
231	S32501D	Other fractures
231	S32501G	Other fractures
231	S32501K	Other fractures
231	S32501S	Other fractures
231	S32502A	Other fractures
231	S32502B	Other fractures
231	S32502D	Other fractures
[truncated: 1,834,919 more chars]
